# SeagrassDB: An open-source transcriptomics landscape for phylogenetically profiled seagrasses and aquatic plants

**DOI:** 10.1038/s41598-017-18782-0

**Published:** 2018-02-09

**Authors:** Gaurav Sablok, Regan J. Hayward, Peter A. Davey, Rosiane P. Santos, Martin Schliep, Anthony Larkum, Mathieu Pernice, Rudy Dolferus, Peter J. Ralph

**Affiliations:** 1 Climate Change Cluster (C3), University of Technology Sydney, PO Box 123 Broadway, NSW 2007 Australia; 2 Laboratório de Recursos Genéticos, Universidade Federal de São João Del-Rei, Campus CTAN, São João Del Rei, Minas Gerais 36307-352 Brazil; 3 CSIRO Agriculture and Food, GPO Box 1700, Canberra, ACT 2601 Australia

## Abstract

Seagrasses and aquatic plants are important clades of higher plants, significant for carbon sequestration and marine ecological restoration. They are valuable in the sense that they allow us to understand how plants have developed traits to adapt to high salinity and photosynthetically challenged environments. Here, we present a large-scale phylogenetically profiled transcriptomics repository covering seagrasses and aquatic plants. SeagrassDB encompasses a total of 1,052,262 unigenes with a minimum and maximum contig length of 8,831 bp and 16,705 bp respectively. SeagrassDB provides access to 34,455 transcription factors, 470,568 PFAM domains, 382,528 prosite models and 482,121 InterPro domains across 9 species. SeagrassDB allows for the comparative gene mining using BLAST-based approaches and subsequent unigenes sequence retrieval with associated features such as expression (FPKM values), gene ontologies, functional assignments, family level classification, Interpro domains, KEGG orthology (KO), transcription factors and prosite information. SeagrassDB is available to the scientific community for exploring the functional genic landscape of seagrass and aquatic plants at: http://115.146.91.129/index.php.

## Introduction

Transcriptomics-assisted gene mining approaches have been widely used for understanding the physiological implications of how an organism responds to biotic and abiotic stress conditions. Next generation sequencing (NGS) based transcriptomics has not only accelerated but has also played a key role in the identification of new functional genes across diverse species, which has been leveraged to understand the genetic basis of ecological adaptation to their surrounding environment. The origin and evolution of aquatic plants has been previously re-visited^1^ and with the availability of increasing transcriptomics and genomic resources, it will be more apparent to hypothesize the origin and diversification of aquatic plants^1^. Phylogenetically, the origin of aquatic plants dates back to the Cretaceous era (145 MYA ago)^2^ and shows signatures of early divergence of aquatic and terrestrial plants^1^. Seagrasses belong to the order of Alismatales^3,4^, which represent a large order of monocotyledons comprising of 13 families and 165 genera widely represented by seagrasses and freshwater aquatic species^5^. Seagrasses have been described as paraphyletic hydrophilus angiosperms with genera belonging to the families *Cymodoceaceae*, *Zosteraceae* and *Hydrocharitaceae*^1,3,4^. The early evolutionary divergence of seagrasses from land plants highlights their suitability as models for identifying and capturing the genes and associated pathways, which can shed evidence on the functional divergence of these species, particularly within the angiosperm lineage^1^. Ancestrally acquired traits of evolutionary specialization includes aerenchyma, a dynamic carbonic–carbonate system and efficient photosynthetic systems allowing them to survive in light-limited environments^6^. In addition, they have also exhibit morphological and physiological specific changes such as leaf structure, carbon concentrating mechanisms (CCMs), adaptation to light limitation, submergence, tolerance to high salinity and resisting wave action and tidal currents thus making them an attractive model system to study in regards to their adaptation to marine environments^4,6^.

Recently, whole genome sequences of *Zostera marina*^7^ and *Zostera muelleri*^8^ have provided insight into the partial loss of the ethylene pathway. Additionally, salt tolerance and reproductive mechanisms have been reviewed and subsequently revisited in recent genome^7,8^ and previously described transcriptional reports using a plethora of next generation sequencing technologies^9–12^. Nonetheless, RNA Sequencing (RNA-Seq) has been widely used as the method of choice to understand the functional and phenotypic plasticity of non-model plants including seagrasses^9,13^, paving the way for dissecting species adaptation to the marine environment. Transcriptomics repositories such as PhytometaSync (http://www.phytometasyn.ca) and the 1KP project (onekp.com) have been buil and made publicly available for land plants, which enabled functional gene mining, exploration of phenotypic plasticity, metabolism genes and phylogenetic inferences in land plants. However, the only available transcriptome portal in-case of marine plants is Dr. Zompo database (http://drzompo.uni-muenster.de)^14^, providing transcriptomic resources for two seagrasses namely *Zostera marina* and *Posidonia oceanica* respectively^14^. It is worth to mention that these species only exist in the Northern hemisphere^14^, as such, this database is limited concerning the species coverage. The lack of such resources for other seagrasses and aquatic plants, specifically phylogenetically and ecologically relevant species prompted us to develop SeagrassDB, an open access portal to disseminate the expressed gene repertoire to the marine scientific community. To the best of our knowledge, this is the first resource portal which provides large scale access to 1,052,262 unigenes representing 34,455 transcription factors, 470,568 PFAM domains, 382,528 prosite models and 482,121 InterPro domains across 8 seagrass species and 1 freshwater aquatic plant species for functional gene mining and phylogenomic exploration. SeagrassDB will serve as a resource for mining functional genes, understanding and cataloguing stress-related functional changes, as well as performing comparative transcriptomics across aquatic and land plant species.

## Material and Methods

### Illumina sequencing, and assembly of seagrass and aquatic plant transcriptomes

Leaf samples from 6 seagrass species *Cymodocea serrulata, Halodule uninervis, Halophila ovalis, Phyllospadix iwatensis, Syringodium isoetifolium, Zostera muelleri* and one aquatic species *Lemna minor* were collected from all around Australia. RNA was extracted from leaf samples following manufacturer’s instructions and subsequent contamination of genomic DNA was removed using the column purification step as implemented in PureLink™ DNase (Life Technologies). Quality controls of the RNA samples was done using the RNA 6000 Nano Kit Agilent (Agilent 2100 Bioanalyzer, Australia). RNA quantification was further confirmed at AGRF sequencing facility, Melbourne, Australia and only high-quality RNA with RIN number greater than 7 were subsequently sequenced using Illumina Hiseq 2000 at AGRF, Melbourne, Australia. All the sequencing data from in this study has been deposited to EBI and can be accessed under the project code: PRJEB22311 (ERP103988). Quality checking of the raw reads was done using FASTQC (https://www.bioinformatics.babraham.ac.uk/projects/fastqc/). Based on the FASTQC reports, quality cleaning of the raw reads was done using Trimmomatic version 3.2^15^ using 2:30:10 SLIDINGWINDOW:4:5 LEADING:5 TRAILING:5 MINLEN:50 in PE mode. Quality cleaned reads were assembled using Trinity version 2^16,17^ with Kmer = 25 and default K-min-cov = 1. Assembled transcripts were further clustered using CD-HIT-EST^18^ using a word size of 8 and an identity overlap of 0.95 and non-redundant transcripts were re-assembled using the overlap-layout consensus algorithm implemented in Contig Assembly Program (CAP3)^19^ to obtain unigenes with the following settings; identity cut-off threshold of 95%, overlap length cut-off of 50, specific clipping range of N > 50, specific gap penalty factor of 3 and a max number of word occurrences of 1000^20^. In case of *Zostera marina*, raw sequencing reads were retrieved from a previously published study with the following NCBI SRA accession number SRP035489^11^ and were subsequently assembled using the parameters defined above using Trinity version 2^16,17^. For *Posidonia oceanica*, assembled contigs were obtained from the recent transcriptome^10^ and are available from NCBI under the entry GEMD01000000. In-silico expression profiling of the assembled unigenes were done using RSEM^21^ . FPKM has been used as a measure of the unigene expression estimates^21^.

### Transcriptome completeness, Domain completeness, single copy orthologs and functional annotations

Assembled non-redundant unigenes were functionally assessed for transcriptome completeness using two independent approaches: (1) BUSCO^22^, which uses the entire embryophyte dataset, which represents the evolutionary informed near-universal single copy orthologs from OrthoDB v9 in trans mode and (2) DOGMA^23^, which uses a set of PFAM modeled evolutionary conserved set of protein domains. Additionally, completeness of assembled transcriptomes was assessed using 3790 single copy conserved orthologs from *Arabidopsis thaliana* (available from: http://compgenomics.ucdavis.edu/compositae_reference.php) using reciprocal best blast (RBH) orthology approaches. Assembled unigenes were functionally annotated by performing BLASTx searches against NCBI (www.ncbi.nlm.nih.gov), UniProt/TrEMBL (www.uniprot.org) with an E-value cutoff of 1E-5, min-identity = 50% and functional annotations were retrieved from UniProt/TrEMBL flat files available from (www.uniprot.org). Coding regions were predicted using GeneMarkST^24^, which employs unsupervised learning models for identifying the coding regions^24^. For the identification of transcription factors, curated BLASTx searches against the plant transcription factor databases available from http://planttfdb.cbi.pku.edu.cn and http://plntfdb.bio.uni-potsdam.de/v3.0/ were performed. In addition, transcription factors were also identified using plant TFcat^25^. KEGG based representation of unigenes was done using KEGG Mapper and KEGG (www.genome.jp/kegg/).

### Development of SeagrassDB

SeagrassDB has been developed using MySQL version: 14.04.01 Distribution 5.5.54 (http://www.mysql.com/), APACHE version: 2.4.7 (http://www.apache.org/) and PHP version: 5.5.9 (http://www.php.net/) with several of the built-in functionalities coded in PHP5 for fast interaction with the user-defined queries. The present version of the SeagrassDB supports a three-tiered architecture, where the middle tier representing MySQL is effectively interacting with query-based search patterns from the client-based PHP tier. The database is hosted on National eResearch Collaboration Tools and Resources (NeCTAR) on a 64-bit virtual machine running Ubuntu version 14.04.05 with 12GB of RAM. Linux architecture is supported by a LAMP server. The portal works well with CSS3 enabled browsers including Google Chrome, Safari and Mozilla Firefox.

## Results and Discussion

Climate change associated with rapid increase in global CO_2_ emissions is a key challenge, which needs to be evaluated for conservation of seagrass meadows and associated rates of carbon sequestration^26–29^. In addition, light acclimation and adaptation of seagrass species to variations in light intensities have been widely studied, which has allowed biologists to understand light adaptation in marine plants^30–33^. Leveraging the recent advances in the high throughput sequencing approaches, attempts have been made to address the ecological and reproductive adaption of aquatic plants using genomics and transcriptomics approaches^7,8,34,35^. With the recently available genome sequences of *Zostera marina*^7^ and *Zostera mulleri*^8^, attempts have been made to identify key genes linked to aquatic adaptation and their possible applications to improve crop domestication, which will subsequently allow us to develop sustainable approaches for feeding the global population of ca. 9.5 billion people by 2050^36^.

As compared to genome sequencing approaches, transcriptomics-assisted gene discovery and candidate gene validation approaches have been widely used to unravel the species specific genetic adaptation. Rapid development in comparative genomics and transcriptomics has enabled the identification of early onset markers for physiological stress and senescence^29,33–35^. Several research groups have addressed this issue by developing open-access transcriptomics portals for land plants; however, these attempts have been limited in marine and aquatic plants, which presents a bottleneck to develop forward genetic approaches to understand the ecological speciation and genetics of marine and aquatic plants. Another potential bottleneck is the availability of the transcriptomics data under a unified browsing portal with systematic annotations, which can enable the high throughput mining of genes for a diverse number of marine and aquatic species. Taking these considerations into account, we developed SeagrassDB, which represents a unified transcriptomics portal for seagrasses and aquatic plants and provides a comprehensive resource to explore the functional gene space in seagrasses and aquatic plants as well as to explore the phylogenomics perspective and evolutionary of ancestral characters in aquatic plants and seagrasses.

### Transcriptome assessment in SeagrassDB

**Figure 1:**
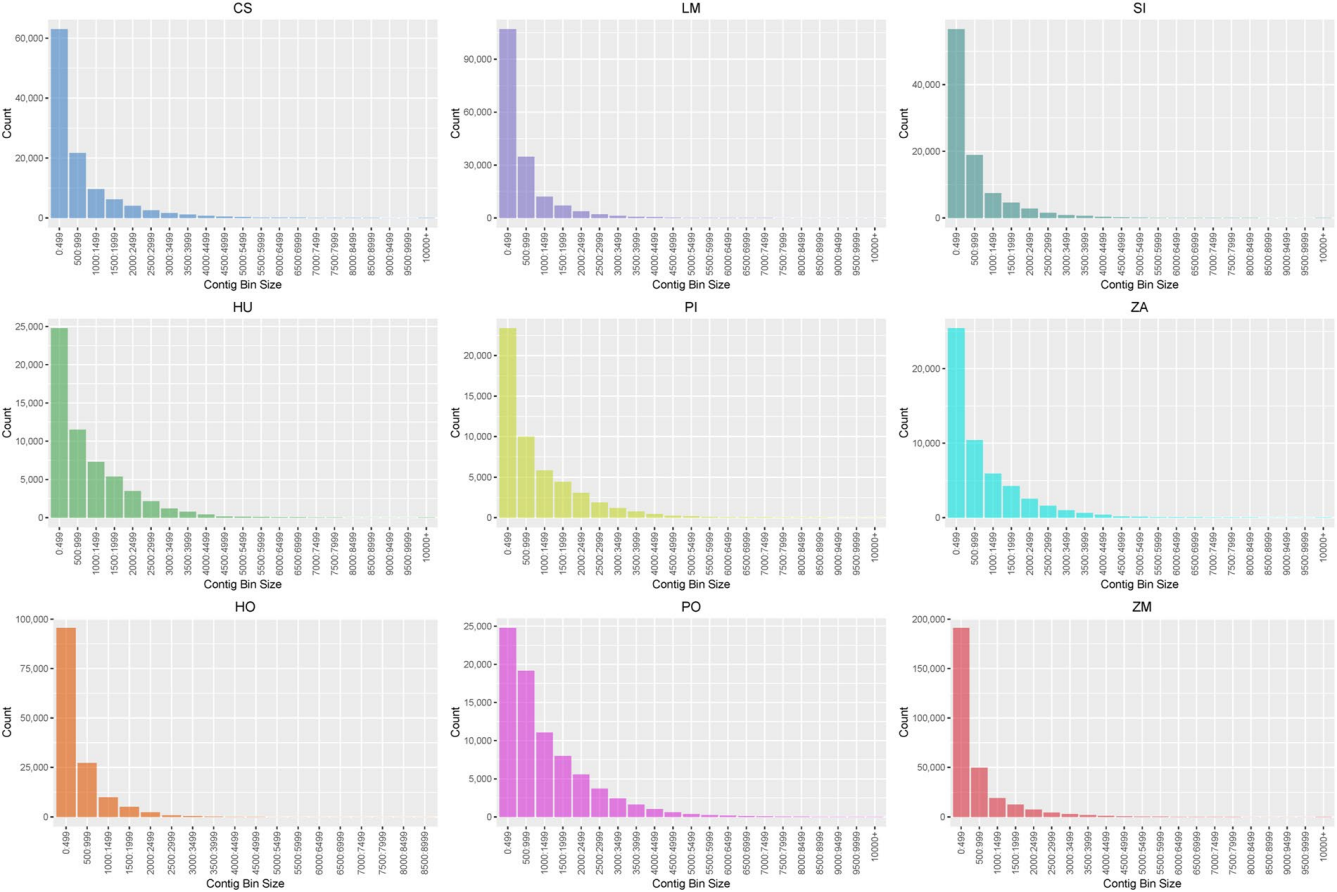
Contig binning across the assembled species in SeagrassDB.

Transcriptomics has been widely applied to study several factors affecting the seagrass distribution, which involves phylogeographic differentiation^37^, tissue specific transcriptomics to address reproductive biology^38^ and to understand abiotic response to environmental conditions^39^. Table 1 and Fig. 1 presents the summary statistics of the transcriptome assembly present in SeagrassDB. The number of assembled unigenes varied from 51,707 in *Phyllospadix iwatensis* to 293,045 in *Zostera muelleri*. The assembled unigenes showed an N50 value of 1,836 bp in *Phyllospadix iwatensis* and an N50 value of 724 bp in *Halophila ovalis*. Overall the observed N50 is in line with previous reports for higher plants^13^ and previously reported N50 values in *Posidonia oceanica*^10^ and *Zostera marina*^11^, thus providing a good representation of the assembled transcriptomes. Functional annotation using BLASTx (E-value 1E-5) based searches revealed a cumulative percentage of transcriptome annotations for *Cymodocea serrulata* (49.46%)*, Halodule uninervis* (62.93%)*, Halophila ovalis* (55.85%)*, Lemna minor* (38.01%)*, Phyllospadix iwatensis* (62.93%)*, Syringodium isoetifolium* (42.14%)*, Zostera muelleri* (41.44%)*, Zostera marina* (59.63%) and *Posidonia oceanica* (49.03%) respectively, thus providing further evidence of the high coverage of the assembled transcriptomes.

**Table 1:** Summary statistics of transcriptomics in SeagrassDB.

Summary Statistics	SI	HU	LM	HO	CS	PI	PO	ZA	ZM
Total number of reads (PE)	30800346	39950720	37793836	42671860	41836870	43133914	70453120	55525824	60812923
Total number of Unigenes	94218	57490	169790	141858	112178	51707	79235	52741	293045
Median length (bp)	408	624	388	360	429	577	853	528	366
Maximum contig length (bp)	15898	14423	12316	8831	12258	12507	16705	15776	26925
N50 (bp)	1157	1741	938	724	1528	1836	2041	1672	1171
Number of contigs (>1 kb)	18721	21223	28134	19068	27509	18336	35285	16905	52326
Number of predicted ORFs	53254	33310	79652	66706	57517	27819	34245	24824	130627
Unigenes with BLASTx against UniprotKB	39965	36181	64552	79240	55494	32540	38849	31450	121446
Unigenes with PFAM	37192	32745	61879	75022	51916	29777	37467	30146	114424
Unigenes with GO	37036	32734	61343	75039	51523	29572	38389	30860	113401
Unigenes with InterPro	38232	34127	63042	76553	53439	30932	38062	30643	117091
Unigenes with Prosite	28570	22320	51819	65065	39200	21111	33130	26831	94482
Unigenes with TF	3045	3161	4444	3500	3652	2722	3033	2528	8370

Species name corresponds to *Cymodocea serrulata* (CS), *Halodule uninervis* (HU), *Halophila ovalis* (HO), *Lemna minor* (LM), *Phyllospadix iwatensis* (PI), *Syringodium isoetifolium* (SI), *Zostera muelleri* (ZM), *Zostera marina* (ZA) and *Posidonia oceanica* (PO).

**Figure 2:**
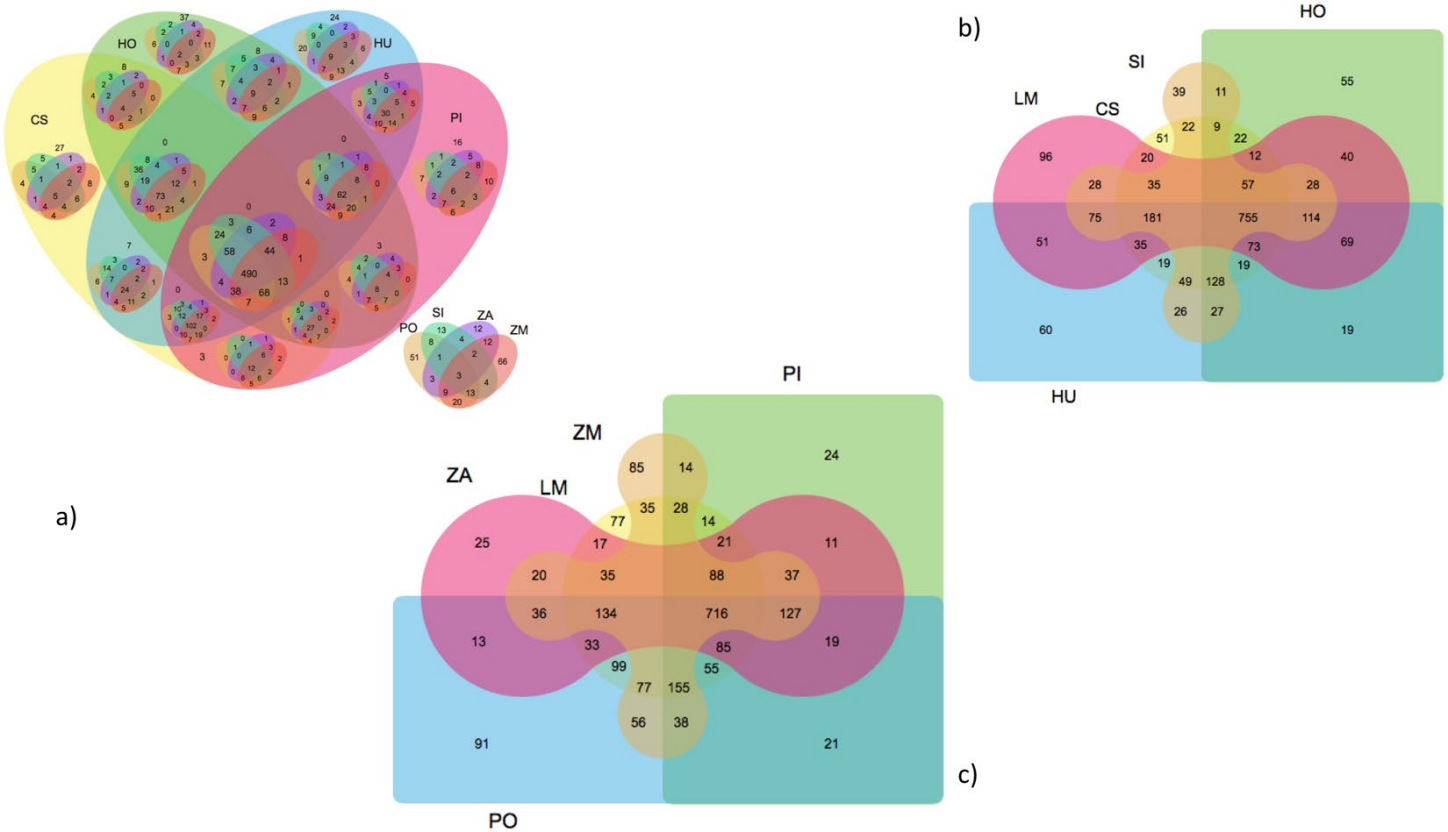
(**a**) Venn diagram using VennPainter available from https://github.com/linguoliang/VennPainter shows the shared single copy orthologs across aquatic plant species; (**b**) showing the shared single copy orthologs across the Cymodoceaceae, Araceae and Hydrocharitaceae; and (**c**) showing the shared single copy orthologs across the Zosteracea, Posidoniceae and Araceae.

Transcriptome completeness has been evaluated using three independent measures, including 1) BUSCO^22^, which uses embryophyta specific lineage conserved single copy orthologs derived from OrthoDB v9, 2) DOGMA^23^, which is used to access the protein domain completeness based on the presence and absence of evolutionary conserved functional domains and 3) *Arabidopsis thaliana* single copy orthologs. Tables 2 and 3 represent the summary statistics of BUSCO and DOGMA based transcriptome completeness. It is worth highlighting that all the species sequenced in the present study showed a high degree of completeness using single-copy BUSCO (Table 2), which is analogous to the high representation of the identified completed proteins domains as revealed by DOGMA (Table 3). Orthology reassignments indicated a set of 2402 single copy conserved orthologs across seagrass and aquatic plant species present in SeagrassDB. Figure 2 represents nested and Edwards Venn diagram based representation of the shared single copy conserved orthologs across the phylogenetically profiled aquatic plant species.

**Table 2:** BUSCO assessment of transcriptome completeness in SeagrassDB.

	SI	HU	LM	HO	CS	PI	PO	ZA	ZM
Complete BUSCOs	878	935	1056	742	800	887	1107	862	1113
Complete and single-copy BUSCOs	740	781	859	628	628	757	917	729	759
Complete and duplicated BUSCOs	138	154	197	114	172	130	190	133	334
Fragmented BUSCOs	161	148	107	179	153	111	112	139	95
Missing BUSCOs	401	357	277	519	487	442	221	439	232
Total BUSCO groups searched	1440	1440	1440	1440	1440	1440	1440	1440	1440

In the case of BUSCO, entire embryophyta datasets were used as a lineage for the assessment of proteome completeness in trans mode of BUSCO (Simão *et al*.^22^). BUSCO uses a set of the evolutionary informed near-universal single copy orthologs from OrthoDB v9. *Cymodocea serrulata (CS), *Halodule uninervis* (HU), *Halophila ovalis* (HO), *Lemna minor* (LM), *Phyllospadix iwatensis* (PI), *Syringodium isoetifolium* (SI), *Zostera muelleri* (ZM), *Zostera marina* (ZA) and *Posidonia oceanica* (PO).

**Table 3:** DOGMA based assessment of transcriptome completeness in SeagrassDB.

CDA size	SI	HU	LM	HO	CS	PI	PO	ZA	ZM
Found	1804	1811	1918	1473	1775	1707	1876	1676	1963
Expected	2017	2017	2017	2017	2017	2017	2017	2017	2017
Completeness	89.44	89.79	95.09	73.03	88	84.63	93.01	83.09	97.32

Domain completeness of the assembled transcriptome was assessed using DOGMA version 2.00 (Dohmen *et al*.^23^) based on 965 single-domain CDAs (Conserved Domain Arrangements) and 1,052 multiple-domain CDAs across eukaryotes. DOGMA uses a set of the PFAM modeled evolutionary conserved set of the conserved protein domains. CDA Size: The size of the CDAs that were found to be conserved in the core species; Found: The number of these CDAs that were found; Expected: The number of expected CDAs (=all CDAs that were found to be conserved among the core species); %Completeness: Number of CDAs found (in percent). *Cymodocea serrulata (CS), *Halodule uninervis* (HU), *Halophila ovalis* (HO), *Lemna minor* (LM), *Phyllospadix iwatensis* (PI), *Syringodium isoetifolium* (SI), *Zostera muelleri* (ZM), *Zostera marina* (ZA) and *Posidonia oceanica* (PO).

### SeagrassDB: a unified platform for browsing 9 aquatic plant species

Systematic approaches for storing and visualization of transcriptomics resources for marine and aquatic plant species has been previously addressed through the development of Dr. Zompo^14^, which provides information for only two species, *Zostera marina*^11^ and *Posidonia oceanica*^10^. Although Dr. Zompo^14^ represents the data in a unified framework, it does not include other seagrass species, which have evolved over time. Additional limitations of Dr. Zompo^14^ include the absence of expression estimates for the assembled unigenes. Transcriptomics assisted gene discovery with the available expression estimates helps identify candidate genes accurately, where multiple in-paralogs have been predicted using homology based approaches. Previously, it has been widely shown that FPKM values of 1 represent the abundance and expression of one transcript per cell^40^. SeagrassDB bridges all the gaps and provides a unified platform for accessing all the associated information within the transcript assemblies present in SeagrassDB.

**Figure 3:**
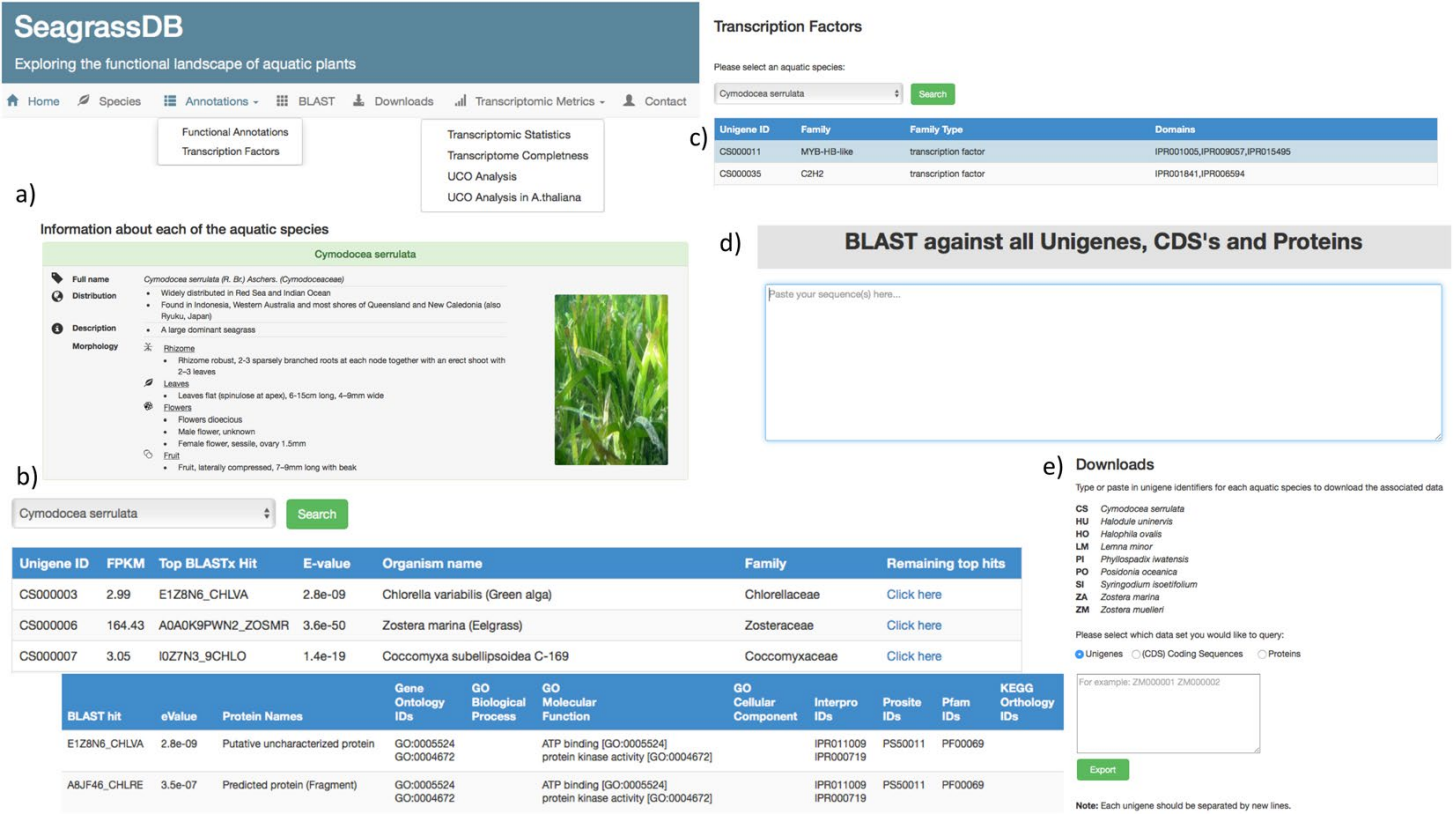
Browsing SeagrassDB.

SeagrassDB searching and browsing patterns are given in Fig. 3, which displays the hierarchal information stored in SeagrassDB. In addition to the hierarchically stored information in SeagrassDB, we also provide a species information page (Fig. 3a) that allows the user to browse through the morphological and physiological traits of these aquatic plants. The functional annotations page allows for species selection as a first curated step, which presents the unigenes associated with the selected species and respective information such as FPKM, BLASTx hit, E-value, family, GO annotations, Interpro, Prosite and associated PFAM domains for each unigene (Fig. 3b and c). SeagrassDB provides transcriptome completeness assessments as well as the binning of unigenes according to length and the orthology searches against the single copy conserved orthologous genes in *Arabidopsis thaliana* (Fig. 3). While the transcriptome assemblies report in-paralogs in addition to the orthologs, the display table only shows single copy orthologs across all the species. In addition to this, BLAST enabled searches and downloads of user enabled curated queries are present (Fig. 3d and e) for down-stream analysis.

### Transcription factors and KEGG representation in SeagrassDB

Transcription factors play an important role in regulating the gene expression of plants. Apart from regulating the gene expression, their roles and diversification have been widely addressed^41^. Recent studies in land plants have focussed on the development of activation domains by the fusion of the designed transcription factor with proteins of interest^42^. Given the importance of transcription factors, identifying transcription factors is crucial to the understanding of the regulatory roles of the transcripts as identified through high-throughput sequencing approaches. Although the regulatory roles of transcription factors have been widely studied across the land plants, limited information on the role of transcription factors and their subsequent role as gene regulators is present across the aquatic plants^29,33–35^.

Transcription factor classification revealed a total of *Cymodocea serrulata* (3652)*; Halodule uninervis* (3161)*; Halophila ovalis* (3500)*; Lemna minor* (4444)*; Phyllospadix iwatensis* (2722)*; Syringodium isoetifolium* (3045)*; Zostera muelleri* (8370)*; Zostera marina* (2524) and *Posidonia oceanica* (3033) transcription factors respectively. Interestingly, among the identified transcription factors, WD-40 like and C2H2 were the most abundant transcription factors across all the species (Supplementary Table 1). It is worth highlighting that the WD-40 family represents a 40 amino acid motif ending in Trp-Asp, which has been shown to play key roles in light signalling and cell development^43^. Furthermore, the C2H2 family of transcription factors have been previously shown to play important roles in cell development and photomorphogenesis^44,45^. Abundance of these transcription factor families may indicate towards their role as regulatory genes in controlling abiotic stress mediated development. Nonetheless, the availability of these annotated transcription factors will allow for a deeper understanding the functional gene space and encourage mining for seagrasses and aquatic plants.

Evolution of genes and biochemical pathways has been a prime focus to understand the metabolic divergence of species in response to environmental constraints^46^. Recently, it has been speculated that the evolution of specialized metabolic pathways is related to lifestyle adaptations^47^. We performed KEGG based mapping of unigenes to classify them to the respective pathways, revealing a total of *Cymodocea serrulata* (7232)*; Halodule uninervis* (7465)*; Halophila ovalis* (6309)*; Lemna minor* (6474)*; Phyllospadix iwatensis* (6702)*; Syringodium isoetifolium* (6703)*; Zostera muelleri* (9508)*; Zostera marina* (6307) and *Posidonia oceanica* (7345) KEGG orthology (KO) terms respectively. Using transcriptomics and proteomic approaches, arrays of genes and proteins have been shown to be differentially expressed across light, temperature and ocean acidification conditions^29–35,47^. It is also worthwhile mentioning that previous estimates of accelerated evolution of seagrass genes such as those involved in photosynthetic and metabolic pathways but also in translation pathways^1^ are all examples of convergent evolution in seagrasses.

### Applications of SeagrassDB: Case example of a salt sensitive gene from sequence, structure and phylogenetic conservation

Proton pumps play an important role in adaptation of plants to salt tolerance. Physiological significance of protons pumps has been widely elucidated across land plants including the model plant *Arabidopsis thaliana*. However, physiological evidence of the proton pumps has been only established in *Zostera marina*^48^. In model land plants, proton pumps play an important role in the Na+ and K+ homeostasis and also maintain the cyclic transport of ions across the plasma membrane^49^. The lack of resources for seagrass species till now has limited the understanding of these proton pumps in such species except for a few previous studies in *Zostera marina*^48,49^.

**Figure 4:**
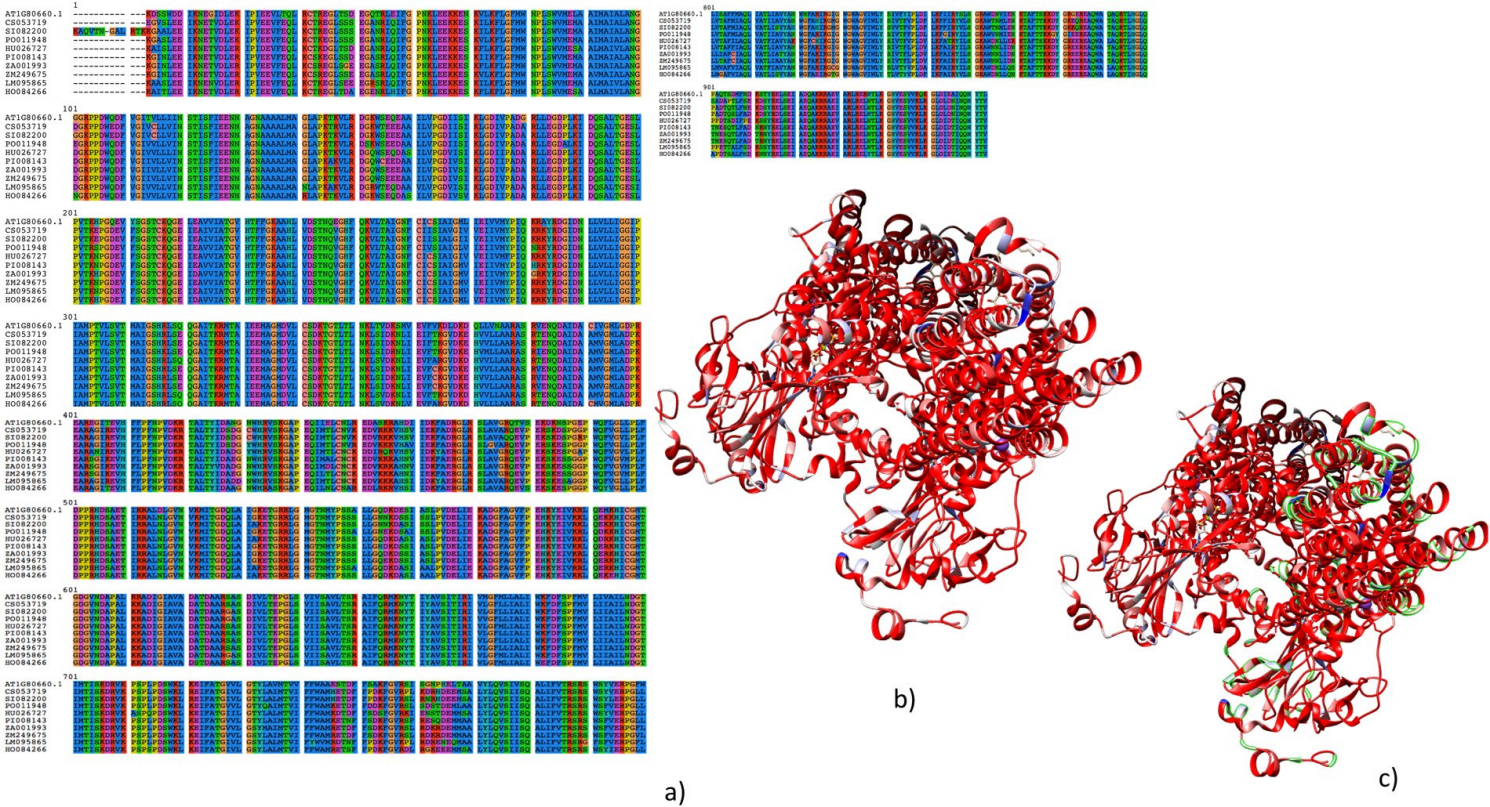
(**a**) Shows the protein alignment of H^+^-ATPase; (**b**) and (**c**) shows the structural conservation of H^+^-ATPases across the land and aquatic plants.

To demonstrate the possible applications of SeagrassDB, we performed a case study by performing a BLASTx search of the H^+^-ATPase, which is a proton-pump and maintains the proton-motive force across the cell membrane^49^. Previously, H^+^-ATPase has been shown to be a decisive factor for hyperosmotic stress and has been demonstrated to confer the salt tolerant ATPase activity in *Zostera marina*^49^. To compare, we used the model plant *Arabidopsis thaliana* H^+^-ATPase as a query to perform BLASTx searches against diverse species present in SeagrassDB with an E-value cutoff of 1E-5 revealing the presence of H^+^-ATPase across all the species. Subsequently, protein alignments were done using MSAProbs^50^, revealing a high degree of conservation across the domains present in H^+^-ATPase (Fig. 4a). To understand whether the sequence based modifications are supported by the structural models, we downloaded the structure model of H^+^-ATPase from PDB (5KSD)^51^, and mapped the conservation scores to the H^+^-ATPase model, which revealed overall high conservation of the H^+^-ATPase gene (Fig. 4b). The backbone of this model supported high conservation of residues across the structural model as revealed by Chimera available from https://www.cgl.ucsf.edu/chimera/ (Fig. 4c).

**Figure 5:**
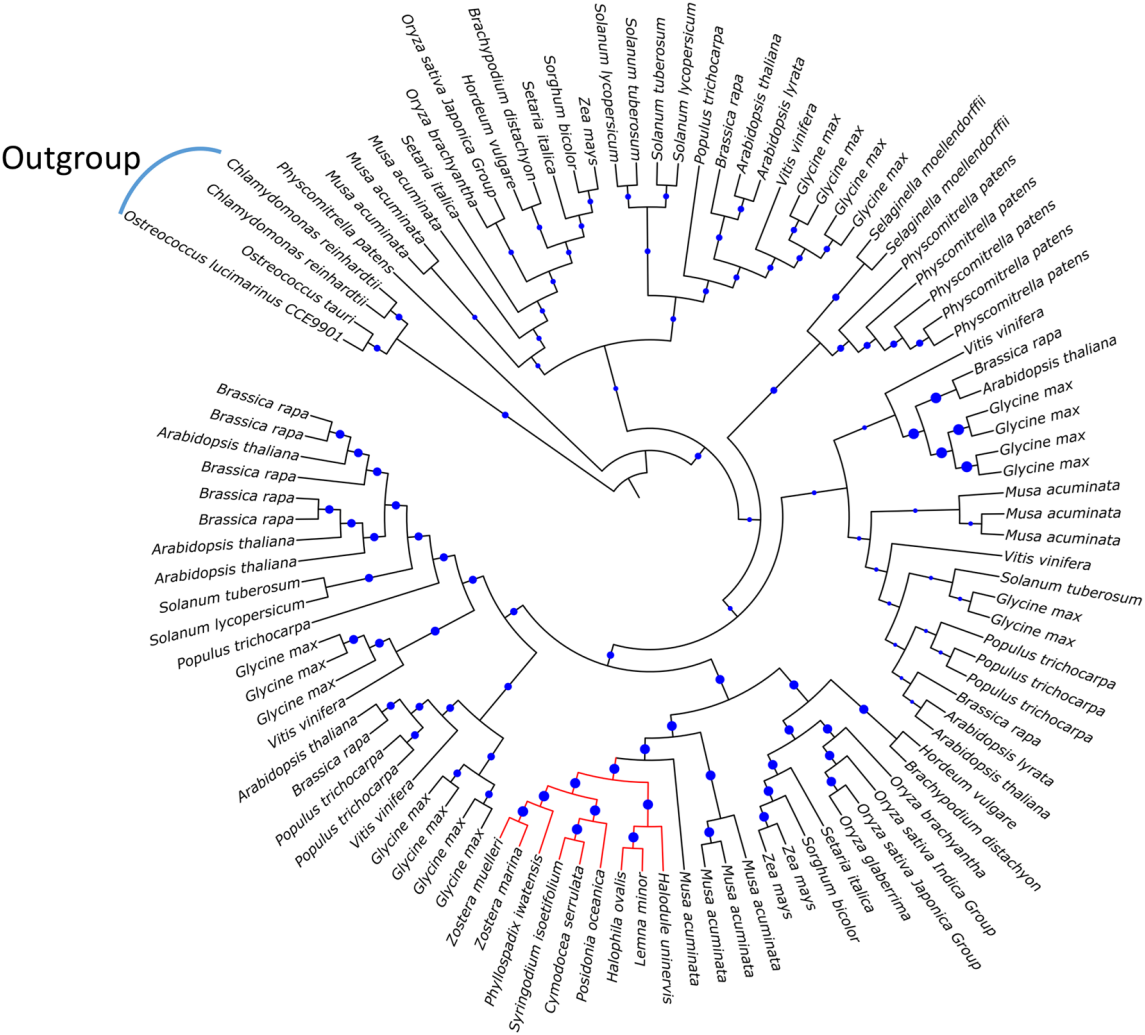
Phylogenetic resolution of H^+^ ATPase across the evolutionary time scale.

To demonstrate the importance of SeagrassDB as a source of phylogenomics in seagrass and aquatic species, we further assessed the phylogenetic ancestral tree reconstruction using RAxML version 8^52^. Figure 5 represents the evolutionary classification of the H^+^ ATPase gene across the land plants and aquatic plants using *Chlamydomonas reinhardtii* and *Osterococcus tauri* as the outgroup species. For phylogenetic characters based leaf sorting, all branches showing bootstrapped data of more than 50% were retained. Interestingly, the observed protein conservation across the proton pumps revealed reliable phylogenetic placement, with all the seagrass species representing a distinct clade (Fig. 5). This observation supports the exemplified usage of transcripts and proteins models present in SeagrassDB for construction of ancestral states and also to study the protein model evolution. Transcriptomics assisted phylogenetic profiling has recently gained importance due to the unavailability of complete genomes in several of the non-model species. Illustrative examples of application of SeagrassDB from sequence-based methods to phylogenetic placement will broaden the understanding of the evolution and phylogenetic placement of marine plants.

## Conclusion

Although functional genomics is the forefront focus in land plant research, limited studies have been performed in seagrasses due to the lack of sequence resources. Identification of regulatory genes and pathways in marine plants will not only advance the understanding of marine physiological adaptations but will also play a key role in identifying the evolutionary forces that contribute to regulate these genes and pathways, in turn addressing the rapid radiation of aquatic plants during and after the Cretaceous era. SeagrassDB has been developed with the goal to accelerate functional genomics approaches in seagrasses and aquatic plants and to obtain further information through comparative transcriptomics to understand the genes, which could be functionally transferred for crop domestication.

## Electronic supplementary material

Phylogenetic ancestral state reconstruction of H+-ATPase using RAXML

X-ray crystallography structure of ATPase

Distribution and abundance of the transcription factors across the seagrasses and aquatic plants

Alignment visualization of H+-ATPase across the land, aquatic and seagrasses
