# Supplementary material for: SeagrassDB: An open-source transcriptomics landscape for phylogenetically profiled seagrasses and aquatic plants
Source: Sci Rep. 2018 Feb 9;8:2749. doi: 10.1038/s41598-017-18782-0 (PMC5807536; doi:10.1038/s41598-017-18782-0)
Supplement: Supplementary file 1 — Phylogenetic ancestral state reconstruction of H+-ATPase using RAXML [file 41598_2017_18782_MOESM1_ESM.pdf]

SeagrassDB: An open-source transcriptomics landscape for phylogenetically profiled seagrasses and aquatic plants

Gaurav Sablok1*$, Regan J. Hayward1*, Peter A. Davey1, Rosiane Santos2, Martin Schliep1, Anthony Larkum1, Mathieu Pernice1, Rudy Dolferus3, Peter J Ralph1

1Climate Change Cluster (C3), University of Technology Sydney, PO Box 123 Broadway NSW 2007, Australia; 2Laboratório de Recursos Genéticos, Universidade Federal de São João Del-Rei, Campus CTAN, São João Del Rei- Minas Gerais, CEP 36307-352, Brazil 3CSIRO Agriculture and Food, GPO Box 1700, Canberra ACT 2601, Australia.

(3702AT1G806601,(3711Bra0351631P,
(((3847GLYMA08G231501,3847GLYMA07G029401)203,
(3847GLYMA13G446501,3847GLYMA15G006701)204)202,
(29760VIT09s0002g02260t01,
(((((((4641GSMUAAchr4P22300001,4641GSMUAAchr4P22290001)122,
((((4533OB12G268301,39947LOCOs12g441501)126,
((4513MLOC98443,15368BRADI4G005171)128,(4555Si021111m,
(4558Sb08g0230701,4577GRMZM2G035520P01)130)129)127)125,4555Si034107m
)124,4641GSMUAAchr9P10970001)123)121,
(((4081Solyc03g11340021,4113PGSC0003DMT400065168)133,
(4113PGSC0003DMT400083041,4081Solyc06g07110021)134)132,
(3694POPTR0012s073501,((29760VIT17s0000g05540t01,
((3847GLYMA06G202001,3847GLYMA04G343701)139,
(3847GLYMA05G014601,3847GLYMA17G104201)140)138)137,(3711Bra0292491P,
(3702AT5G626701,59689scaffold8029421)142)141)136)135)131)120,
(((70448Q017J6,436017A4RYL8)145,
(3055EDP06215,3055EDP06054)146)144,3218PP1S30218V61)143)119,
((3218PP1S32130V61,(3218PP1S137291V61,(3218PP1S32133V61,
(3218PP1S611V61,3218PP1S13322V61)151)150)149)148,
(88036EFJ37569,88036EFJ34065)152)147)118,
(((((3847GLYMA17G293701,3847GLYMA14G173601)157,
(3847GLYMA06G079901,3847GLYMA04G079501)158)156,
(29760VIT04s0008g02460t01,
((3694POPTR0018s037001,3694POPTR0006s289901)161,(3711Bra0078451P,
3702AT2G245201)162)160)159)155,
((((4081Solyc07g01778021,4113PGSC0003DMT400010497)166,
((3702AT2G189601,((3711Bra0244521P,
3711Bra0388351P)170,59689fgenesh2kg33537AT2G189601)169)168,
(3711Bra0111721P,((3711Bra0102991P,
3711Bra0102981P)173,3702AT4G301902)172)171)167)165,3694POPTR0018s097
501)164,
(((3847GLYMA09G062503,3847GLYMA15G175301)176,3847GLYMA17G069301)175,
29760VIT11s0052g00620t01)174)163)154,
(((4513MLOC758464,15368BRADI5G246901)179,((4533OB04G349701,
((39947LOCOs04g561601,4538ORGLA04G02424001)183,39946BGIOSGA017272PA)
182)181,(4555Si021113m,(4558Sb06g0312401,
(4577GRMZM2G019404P01,4577GRMZM2G006894P01)186)185)184)180)178,
((4641GSMUAAchr3P27910001,
(4641GSMUAAchr1P10350001,4641GSMUAAchr2P04890001)189)188,
(4641GSMUAAchr10P30810001,(((LM095865,HO084266)193,HU026727)192,
((PO011948,(CS053719,SI082200)196)195,(PI008143,

(ZA001993,ZM249675)198)197)194)191)190)187)177)153)117,
(((4641GSMUAAchr5P04450001,4641GSMUAAchr3P04540001)115,4641GSMUAAchr
9P13200001)114,(29760VIT11s0149g00210t01,
(((3847GLYMA17G111901,3847GLYMA13G223701)111,4113PGSC0003DMT40003091
3)110,
(((3694POPTR0018s120401,3694POPTR0006s202901)108,3694POPTR0006s00770
1)107,
((59689fgenesh1pmCscaffold5000607,3702AT3G426401)105,3711Bra0082881P
)104)106)109)112)113)116)199)200)201);
