# Supplementary material for: SeagrassDB: An open-source transcriptomics landscape for phylogenetically profiled seagrasses and aquatic plants
Source: Sci Rep. 2018 Feb 9;8:2749. doi: 10.1038/s41598-017-18782-0 (PMC5807536; doi:10.1038/s41598-017-18782-0)
Supplement: Supplementary file 4 — Alignment visualization of H+-ATPase across the land, aquatic and seagrasses [file 41598_2017_18782_MOESM4_ESM.pdf]

Alignment: /Users/dr.gauravsablok/Desktop/H_ATPase_Aligned_Cleaned.fasta
Seaview [blocks=10 fontsize=10 A4-landscape] on Sat May 20 03:46:55 2017

SeagrassDB: An open-source transcriptomics landscape for phylogenetically profiled seagrasses and aquatic plants: Gaurav Sablok1*$, Regan J. Hayward1*, Peter A. Davey1, Rosiane Santos2, Martin Schliep1, Anthony Larkum1, Mathieu Pernice1, Rudy Dolferus3, Peter J Ralph1 :
1Climate Change Cluster (C3), University of Technology Sydney, PO Box 123 Broadway NSW 2007, Australia; 2Laboratório de Recursos Genéticos, Universidade Federal de São João Del-Rei, Campus CTAN, São João Del Rei- Minas Gerais, CEP 36307-352, Brazil 3CSIRO Agricult
Food, GPO Box 1700, Canberra ACT 2601, Australia.

```
             1
AT1G80660.1  ---------- ---KDSSWDD IKNEGIDLEK IPIEEVLTQL RCTREGLTSD EGQTRLEIFG PNKLEEKKEN KVLKFLGFMW NPLSWVMELA AIMAIALANG
CS053719     ---------- ---EGVSLEE IKNETVDLEK IPVEEVFEQL KCTREGLSSS EGANRIQIFG PNKLEEKKES KILKFLGFMW NPLSWVMEMA AIMAIALANG
SI082200     KAQVTN-GAL RTKKGAALEE IKNETVDLEK IPVEEVFEQL KCTREGLSSS EGANRIQIFG PNKLEEKKES KILKFLGFMW NPLSWVMEMA AIMAIALANG
PO011948     ---------- ---KGASLEE IKNETVDLEK IPVEEVFEQL KCTKEGLSSS EGANRLQIFG PNKLEEKKES KVLKFLGFMW NPLSWVMEMA AIMAIALANG
HU026727     ---------- ---KAISLEE IKNETVDLER IPIDEVFEQL KCTKDGLTSD EGANRLQIFG PNKLEEKKES KFLKFLGFMW NPLSWVMESA ALMAIVLANG
PI008143     ---------- ---KGINLEE IKNETVDLER IPVEEVFEQL KCSKEGLSSD EGASRLQIFG PNKLEEKKES KIIKFLGFMW NPLSWVMEMA AIMAIALANG
ZA001993     ---------- ---KGINLEE IKNETVDLER IPVEEVFEQL KCSREGLSGE EGASRLQIFG PNKLEEKKES KFLKFLGFMW NPLSWVMEMA AIMAIALANG
ZM249675     ---------- ---KGINLEE IKNETVDLER IPVEEVFEQL KCSREGLSSE EGASRLQIFG PNKLEEKKES KVLKFLGFMW NPLSWVMEMA AVMAIALANG
LM095865     ---------- ---KAASLEE IKNENVDLER IPIEEVFEQL KCTKEGLSEE EGRSRLQIFG PNKLEEKKES KILKFLGFMW NPLSWVMEMA ALMAIVLANG
HO084266     ---------- ---KAITLEE IKNETVDLER IPIEEVFEQL KCTREGLTDA EGENRLHIFG PNKLEEKKES KFLKFLGFMW NPLSWVMESA ALMAIVLANG

             101
AT1G80660.1  GGRPPDWQDF VGITVLLIIN STISFIEENN AGNAAAALMA GLAPKTKVLR DGKWSEQEAA ILVPGDIISI KLGDIVPADG RLLDGDPLKI DQSALTGESL
CS053719     DGKPPDWEDF VGIVCLLVIN STISFIEENN AGNAAAALMA GLAPKTRVLR DGKWTEEEAA ILVPGDIISI KLGDIVPADA RLLEGDPLKI DQSALTGESL
SI082200     GGKPPDWEDF VGIVCLLVIN STISFIEENN AGNAAAALMA GLAPKTKVLR DGKWSEEEAA ILVPGDIISI KLGDIVPADA RLLEGDPLKI DQSALTGESL
PO011948     EGRPPDWQDF VGIVVLLVIN STISFIEENN AGNAAAALMA GLAPKTKVLR DSKWSEEDAA LLVPGDIISI KLGDIVPADA RLLEGDALKI DQSALTGESL
HU026727     DGKPPDWQDF VGIVVLLLIN STISFIEENN AGNAAAALMA GLAPKTKVLR DGQWSEQDAS ILVPGDVISI KLGDIIPADA RLLEGDPLKI DQSALTGESL
PI008143     DGRPPDWQDF VGIIVLLVIN STISFIEENN AGNAAAALMA GLAPKAKVLR DGQWCEEDAA ILVPGDIVSI KLGDIIPADA RLLEGDPLKI DQSALTGESL
ZA001993     DGRPPDWQDF VGIIVLLVIN STISFIEENN AGNAAAALMA GLAPKTKVLR DGQWSEEEAA ILVPGDIVSI KLGDIVPADA RLLEGDPLKI DQSALTGESL
ZM249675     DGKPPDWQDF VGIIVLLVIN STISFIEENN AGNAAAALMA GLAPKTKVLR DGQWSEEEAA ILVPGDIVSI KLGDIVPADA RLLEGDPLKI DQSALTGESL
LM095865     DGRPPDWQDF VGIVVLLVIN STISFIEENN AGNAAAALMA NLAPKAKText DGRWTEQDAA VLVPGDVISI KLGDIVPADA RLLEGDPLKI DQSALTGESI
HO084266     NGKPPDWQDF VGIVVLLVIN STISFIEENN AGNAAAALMA RLAPKTKVLR DGKWSEQDAS ILVPGDIVSV KLGDIIPADA RLLEGDPLKI DQSALTGESL

             201
AT1G80660.1  PVTKHPGQEV YSGSTCKQGE LEAVVIATGV HTFFGKAAHL VDSTNQEGHF QKVLTAIGNF CICSIAIGML IEIVVMYPIQ KRAYRDGIDN LLVLLIGGIP
CS053719     PVTKEPGDEV FSGSTCKQGE IEAIVIATGV HTFFGKAAHL VDSTNQVGHF QKVLTAIGNF CIISIAVGIV VEIIVMYPIQ KRKYRDGIDN LLVLLIGGIP
SI082200     PVTKEPGDEV FSGSTCKQGE IEAIVIATGV HTFFGKAAHL VDSTNQVGHF QKVLTAIGNF CIISIALGII VEIIVMYPIQ KRKYRDGIDN LLVLLIGGIP
PO011948     PVTRSPGDEV FSGSTCKQGE IEAVVIATGV HTFFGKAAHL VDSTNQVGHF QKVLTAIGNF CIVSIAIGLV IEIIVMYPIQ NRKYRDGIDN LLVLLIGGIP
HU026727     PVTKNPGDEI FSGSTCKQGE IEAVVIATGV HTFFGKAAHL VDSTNQIGHF QKVLTAIGNF CICSIAIGIV VEIIVMFPIQ KRKYRDGIDN LLVLLIGGIP
PI008143     PVTKNPGDEV FSGSTCKQGE IEAVVIATGV HTFFGKAAHL VDSTNQVGHF QKVLTAIGNF CICSIAIGMV IEIIVMYPIQ HRKYRDGIDN LLVLLIGGIP
ZA001993     PVTKHPGDEV FSGSTCKQGE IDAVVIATGV HTFFGKAAHL VDSTNQVGHF QKVLTAIGNF CICSIAIGMV IEIIVMYPIQ GRKYRDGIDN LLVLLIGGIP
ZM249675     PVTKNPGDEV FSGSTCKQGE IEAVVIATGV HTFFGKAAHL VDSTNQVGHF QKVLTAIGNF CICSIAIGMV IEIIVMYPIQ RRKYRDGIDN LLVLLIGGIP
LM095865     PVTKNPGDEV FSGSTCKQGE IEAIVIATGV HTFFGKAAHL VDSTNQVGHF QKVLTAIGNF CICSIAIGMV VEIIVMYPIQ KRKYRDGIDN LLVLLIGGIP
HO084266     PVTKHPGDEI FSGSTCKQGE IDAVVIATGV HTFFGKAAHL VDSTNQVGHF QKVLTAIGNF CICSIAIGML VEIIVMYPIQ KRKYRDGIDN LLVLLIGGIP

             301
AT1G80660.1  IAMPTVLSVT MAIGSHRLSQ QGAITKRMTA IEEMAGMDVL CSDKTGTLTL NKLTVDKSMV EVFVKDLDKD QLLVNAARAS RVENQDAIDA CIVGMLGDPR
CS053719     IAMPTVLSVT MAIGSHRLSE QGAITKRMTA IEEMAGMDVL CSDKTGTLTL NKLSIDKNLI EIFTKGVDKE HVVLLAARAS RTENQDAIDA AMVGMLADPK
SI082200     IAMPTVLSVT MAIGSHRLSE QGAITKRMTA IEEMAGMDVL CSDKTGTLTL NKLSIDKNLI EIFTKGVDKE HVVLLAARAS RTENQDAIDA AMVGMLADPK
PO011948     IAMPTVLSVT MAIGSHKLSE QGAITKRMTA IEEMAGMDVL CSDKTGTLTL NKLSIDRNLI EVFTKGVDKE HVMLLAARAS RIENQDAIDA AMVGMLADPK
HU026727     IAMPTVLSVT MAIGSHRLSQ QGAITKRMTA IEEMAGMDVL CSDKTGTLTL NKLSVDKNLI EVFARGVDKD HVVLLAARAS RTENQDAIDA AMVGMLADPK
PI008143     IAMPTVLSVT MAIGSHRLSE QGAITKRMTA IEEMAGMDVL CSDKTGTLTL NKLSIDKNLI EVFCKGVDKE HVVLLAARAS RVENQDAIDA AMVGMLADPK
ZA001993     IAMPTVLSVT MAIGSHRLSE QGAITKRMTA IEEMAGMDVL CSDKTGTLTL NKLSIDKNLI EVFCKGVDKE HVVLLAARAS RVENQDAIDA AMVGMLADPK
ZM249675     IAMPTVLSVT MAIGSHKLSE QGAITKRMTA IEEMAGMDVL CSDKTGTLTL NKLSIDKNLI EVFCKGVDKE HVVLLAARAS RVENQDAIDA AMVGMLADPK
LM095865     IAMPTVLSVT MAIGSHRLSQ QGAITKRMTA IEEMAGMDVL CSDKTGTLTL NKLSVDKNLI EVFTKGVDKD HVVLLAARAS RTENQDAIDA AMVGMLADPK
HO084266     IAMPTVLSVT MAIGSHRLSQ QGAITKRMTA IEEMAGMDVL CSDKTGTLTL NKLSVDKNLV EVFAKGVDKD HVLLLAARAS RTENQDAIDA CMVGMLADPK
```

```
            401
AT1G80660.1  EAREGITEVH FFPFNPVDKR TAITYIDANG NWHRVSKGAP EQIIELCNLR EDASKRAHDI IDKFADRGLR SLAVGRQTVS EKDKNSPGEP WQFLGLLPLF
CS053719     EARAGIREVH FFPFNPVDKR TALTYIDSDG CWHRVSKGAP EQIMTLCNVK EDVRKKVHSV IEKFADRGLR SLAVARQEVP ERSKDSPGGP WQFVGLLPLF
SI082200     EARAGIREVH FFPFNPVDKR TALTYIDSDG CWHRVSKGAP EQIMTLCNVK EDVRKKVHSV IEKFADRGLR SLAVAQQEVP EKSKDSPGRP WQFVGVLPLF
PO011948     EARAGIREVH FLPFNPVDKR TALTYIDSDG YWHRVSKGAP EQIMTLCNVR EDVKKKVHAV IEKFADRGLR SLGVARQEVP ERSKESPGGP WQFVGLLPLF
HU026727     EARANIREVH FFPFNPVDKR TALTYIDADG YWHRVSKGAP EQIMTLCNCK DDIRQKVHSV IDKYAERGLR SLAVGRQEVP EKSKESPGAP WQFVGVLPLF
PI008143     EARSGIKEVH FLPFNPVDKR TALTYIDAEG NWHRVSKGAP EQIMALCNCK EDVKKKAHNV IEKFAERGLR SLAVGRQEVP EKSKESSGGP WQFVGVMPLF
ZA001993     EARSGIKEVH FLPFNPVDKR TALTYVDADG NWHRVSKGAP EQIMDLCNCK EDVKRKAHNV IDKFAERGLR SLAVGRQEVP EKSKESSGGP WQFVGVMPLF
ZM249675     EARSGIKEVH FLPFNPVDKR TALTYIDAEG NWHRVSKGAP EQILTLCNCK EDVKKKAHNI IEKFAERGLR SLAVGRQEVP EKSKESSGGP WQFVGVMPLF
LM095865     EARAGIREVH FFPFNPVDKR TALTYIDADD NWHRVSKGAP EQIMTLCNCK EDVKKKAHSI IDKYAERGLR SLAVARQEVP EKSKESAGGP WQFVGLLPLF
HO084266     EARAGITEVH FLPFNPVDKR TALTYIDAAG NWHRASKGAP EQILNLCNAR EDLRKRVHSI IDKFAERGLR SLAVARQEVS EKSKESPGGP WQYVGLLPLF

            501
AT1G80660.1  DPPRHDSAET IRRALDLGVN VKMITGDQLA IGKETGRRLG MGTNMYPSSA LLGQDKDESI ASLPVDELIE KADGFAGVFP EHKYEIVKRL QEMKHICGMT
CS053719     DPPRHDSAET IRRALNLGVN VKMITGDQLA IGKETGRRLG MGTNMYPSSS LLGNNKDSSI SSLPVDELIE KADGFAGVFP EHKYEIVKKL QEKKHICGMT
SI082200     DPPRHDSAET IRRALNLGVN VKMITGDQLA IAKETGRRLG MGTNMYPSSS LLGNNKDASI SSLPVDELIE KADGFAGVFP EHKYEIVRKL QEKKHICGMT
PO011948     DPPRHDSAET IRRALNLGVN VKMITGDQLA IGKETGRRLG MGTNMYPSSA LLGNEKDSAI SSLPVDELIE KADGFAGVFP EHKYEIVKRL QDKKHICGMT
HU026727     DPPRHDSAET IRRALNLGVN VKMITGDQLA IAKETGRRLG MGTNMYPSSS LLGQDKDASI ASLPVDELIE KADGFAGVFP EHKYEIVRKL QDRKHICGMT
PI008143     DPPRHDSAET IRRALNLGVN VKMITGDQLA IGKETGRRLG MGTNMYPSSS LLGHDKDSSI ASLPVDELIE KADGFAGVFP EHKYEIVKKL QERKHICGMT
ZA001993     DPPRHDSAET IRRALNLGVN VKMITGDQLA IGKETGRRLG MGTNMYPSSS LLGQDKDSSI ASLPVDELIE KADGFAGVFP EHKYEIVKRL QERKHICGMT
ZM249675     DPPRHDSAET IRRALNLGVN VKMITGDQLA IGKETGRRLG MGTNMYPSSS LLGQEKDSSI ASLPVDELIE KADGFAGVFP EHKYEIVKRL QERKHICGMT
LM095865     DPPRHDSAET IRRALNLGVN VKMITGDQLA IAKETGRRLG MGTNMYPSSA LLGQDKDASI AALPVDELIE KADGFAGVFP EHKYEIVRRL QERKHICGMT
HO084266     DPPRHDSAET IRKALNLGVN VKMITGDQLA IAKETGRRLG MGTNMYPSSS LLGQDKDASI AALPVDELIE KADGFAGVFP EHKYEIVKKL QEKEHICGMT

            601
AT1G80660.1  GDGVNDAPAL KRADIGIAVA DATDAARSAS DIVLTEPGLS VIVSAVLTSR AIFQRMKNYT IYAVSITIRI VMGFMLLALI WKFDFSPFMV LIVAILNDGT
CS053719     GDGVNDAPAL KKADIGIAVA DATDAARSAS DIVLTEPGLS VIISAVLTSR AIFQRMKNYT IYAVSITIRI VLGFLLIALI WKFDFSPFMV LIIAILNDGT
SI082200     GDGVNDAPAL KKADIGIAVA DATDAARGAS DIVLTEPGLS VIISAVLTSR AIFQRMKNYT IYAVSITIRI VLGFLLIALI WKFDFSPFMV LIIAILNDGT
PO011948     GDGVNDAPAL KRADIGIAVA DATDAARGAS DIVLTEPGLS VIISAVLTSR AIFQRMKNYT IYAVSITIRI VVGFLLIALI WKFDFSPFMV LIIAILNDGT
HU026727     GDGVNDAPAL KKADIGIAVA DATDAARSAS DIVLTEPGLS VIISAVLTSR AIFQRMKNYT IYAVSITIRI VLGFMLIALI WKFDFSPFMV LIIAILNDGT
PI008143     GDGVNDAPAL KKADIGIAVA DATDAARSAS DIVLTEPGLS VIISAVLTSR AIFQRMKNYT IYAVSITIRI VLGFLLIALI WKFDFSPFMV LIIAILNDGT
ZA001993     GDGVNDAPAL KKADIGIAVA DATDAARSAS DIVLTEPGLS VIISAVLTSR AIFQRMKNYT IYAVSITIRI VLGFLLIALI WKFDFSPFMV LIIAILNDGT
ZM249675     GDGVNDAPAL KKADIGIAVA DATDAARSAS DIVLTEPGLS VIISAVLTSR AIFQRMKNYT IYAVSITIRI VLGFLLIALI WKFDFSPFMV LIIAILNDGT
LM095865     GDGVNDAPAL KKADIGIAVA DATDAARGAS DIVLTEPGLS VIISAVLTSR AIFQRMKNYT IYAVSITIRI VLGFMLIALI WKFDFSPFMV LIIAILNDGT
HO084266     GDGVNDAPAL KKADIGIAVA DSTDAARSAS DIVLTEPGLS VIISAVLTSR AIFQRMKNYT IYAVSITIRI VLGFMLIALI WEFDFSPFMV LIIAILNDGT

            701
AT1G80660.1  IMTISKDRVK PSPLPDSWKL KEIFATGVVL GTYLAVMTVV FFWAAESTDF FSAKFGVRSI SGNPHELTAA VYLQVSIVSQ ALIFVTRSRS WSYVERPGFW
CS053719     IMTISKDRVK PSPLPDSWKL REIFATGIVL GTYLAIMTVI FFWAMHETDF FPDKFGVRPL KDRHDEEMSA LYLQVSIISQ ALIFVTRSRS WSYVERPGLL
SI082200     IMTISKDRVK PSPLPDSWKL REIFATGIVL GTYLALMTVI FFWAMHETDF FPDKFGVRSL RNRHDEEMSA LYLQVSIISQ ALIFVTRSRS WSYLERPGLL
PO011948     IMTISKDRVK PSPLPDSWKL KEIFATGVVL GTYQAIMTVI FFWAMKETDF FDDKFGVRSL SDSTDEMLAA LYLQVSIISQ ALIFVTRSRS WSFVERPGLL
HU026727     IMTISKDRVK ASPQPDSWKL KEIFATGIIL GTYLALMTVI FFWAMKDTDF FSDSFGVRKI ENSTDEMMSA LYLQVSIISQ ALIFVTRSRS WSYVERPGLL
PI008143     IMTISKDRVK PSPLPDSWKL KEIFATGVVL GTYLAIMTVI FFWAMKETNF FSDKFGVRSF RESQDEMMAA LYLQVSIISQ ALIFVTRSRS WSFVERPGLL
ZA001993     IMTISKDRVK PSPLPDSWKL KEIFATGVVL GSYMAIMTVI FFWAMRETDF FSDKFGVRSL RDKRDEMMAA LYLQVSIISQ ALIFVTRSRS WSFVERPGFL
ZM249675     IMTISKDRVK PSPLPDSWKL KEIFATGVVL GSYLAIMTVI FFWAMRETDF FSDKFGVRSL RDKRDEMMAA LYLQVSIISQ ALIFVTRSRS WSFVERPGVL
LM095865     IMTISKDRVK PSPLPDSWKL KEIFATGIVL GSYLALMTVI FYWVMRDTNF FPDKFGVKPL RDRENEQMAA LYLQVSIISQ ALIFVTRSRG FSFVERPGFL
HO084266     IMTISKDRVK PSPSPDSWKL KEIFATGIVL GSYLALMTVI FFWAMRETSF FPDKFGVRDL RGKEEEMMSA LYLQVSIISQ ALIFVTRSRS WSYIERPGLL
```

```
            801
AT1G80660.1 LISAFFMAQL IATLIAVYAN WNFARIRGIG WGWAGVIWLY SIVFYIPLDI LKFIIRYSLS GRAWDNVIEN KTAFTSKKDY GKGEREAQWA QAQRTLHGLQ
CS053719    LVTAFMIAQL VATIIAVYAN WGFAHIKGMG WGWAGVIWLY SIVTFVPLDF LKFFIRYVLS GKAWTNMLEN KTAFTTKKDY GKEQREAQWA TAQRTLHGLQ
SI082200    LVTAFMIAQL VATLISVYAH WGFAHIKGMG WGWAGVIWLY SVVTFVPLDF LKFFIRYVLS GKAWTNMLEN KTAFTTKKDY GKKQREAQWA TAQRTLHGLQ
PO011948    LVTAFMLAQL VATIIAVYAN WGFARIKGIG WGWAGVIWLY SIVTFFPLDL LKFGIRYILS GKAWVNMIEN KTAFTTKKGY GIEEREAQWA TAQRTLHGLQ
HU026727    LVTAFILAQL VATFIAVYAK WGFCHIKGIG WGWAGVIWLY SIVTFVPLDV LKFATRYILS GKAWDNLLER KTAFTSKKDY GREEREAQWA LAQRTLHGLQ
PI008143    LVTAFFIAQL VATLIAVYAN WGFAKIKGIG WGWAGVIWLY SIVTFLPLDV LKFAIRYILS GKAWNNLIDN RTAFTTKKDY GREEREAQWA TAQRTLHGLQ
ZA001993    LLIAFCIAQL VATLIATYAN WGFAKIKGIG WGWAGVIWLY SIVTFLPLDV LKFAIRYILS GKAWNNLIDN KTAFTTKKDY GREEREAQWA TAQRTLHGLQ
ZM249675    LLTAFCIAQL VATLIAAYAN WGFAKIKGIG WGWAGVIWLY SIVTFLPLDV IKFAIRYILS GKAWNNLIDN KTAFTTKKDY GREEREAQWA TAQRTLHGLQ
LM095865    LMVAFVIAQL VATFIAVYAN WGFARIKGCG WGWAGVIWLY SLVTYVPLDA IKFAIRYILS GKAWDNLLQS KTAFTTKKDY GREEREAQWA LAQRTLHGLQ
HO084266    LMGAFVIAQL VATLISVYAN WGFAEIRGTG WGWAGVIWLY TLVTYVPLDF IKFAIRYVLS GKAWDNLLQN KTAFTTKKDY GREEREAQWA LAQRTIHGLQ

            901
AT1G80660.1 PAQTSDMFND KSTYRELSEI ADQAKRRAEV ARLRERHTLK GHVESVVKQK GLDIEAIQQH YTL
CS053719    SADAPTLFSE KDSYRELSEI AEQAKRRAEI ARLRELNTLK GHVESVVKLK GLDLDNINQH YTV
SI082200    PADTQTLFNE KDSYRELSEI AEQAKRRAEI ARLRELNTLK GHVESVVKLK GLDLDNINQH YTV
PO011948    PADTQSLFAD KDSYRDLSEI AEQAKKRAEV ARLRELHTLK GHVESVVKLK GLDLDNINQH YTV
HU026727    PPDTSDIFPE KSSYRELSEI AEQAKRRAEV ARLRELNTLK GHVESVVKLK GLDIDTIQQH YTV
PI008143    TNESQTLFAD NRNYRELSEI AEQAKRRAEV ARLRELNTLK GHVESVVKLK GLDIDTIQQH YTV
ZA001993    TNESQTLFAD TRNYRELSEI AEQAKRRAEV ARLRELNTLK GHVESVVKLK GLDIDTIQQH YTV
ZM249675    TNESQTLFAD TRNYRELSEI AEQAKRRAEV ARLRELNTLK GHVESVVKLK GLDIDTIQQH YTV
LM095865    PPETTALFSD KSSYRELSEI AEQAKRRAEI ARLRELNTLK GHVESVVKLK GLDIDTIQQH YTV
HO084266    APDTSALFHE KNNYRELSEI AEQAKKRAEI ARLRELYTLK GHVESVVKLK GLDIETIQQH YTV
```
