# Supplementary material for: SeagrassDB: An open-source transcriptomics landscape for phylogenetically profiled seagrasses and aquatic plants
Source: Sci Rep. 2018 Feb 9;8:2749. doi: 10.1038/s41598-017-18782-0 (PMC5807536; doi:10.1038/s41598-017-18782-0)
Supplement: Supplementary file 2 — X-ray crystallography structure of ATPase [file 41598_2017_18782_MOESM2_ESM.pdf]

SeagrassDB: An open-source transcriptomics landscape for phylogenetically profiled seagrasses and aquatic plants

Gaurav Sablok1*$, Regan J. Hayward1*, Peter A. Davey1, Rosiane Santos2, Martin Schliep1, Anthony Larkum1, Mathieu Pernice1, Rudy Dolferus3, Peter J Ralph1
1Climate Change Cluster (C3), University of Technology Sydney, PO Box 123 Broadway NSW 2007, Australia; 2Laboratório de Recursos Genéticos, Universidade Federal de São João Del-Rei, Campus CTAN, São João Del Rei- Minas Gerais, CEP 36307-352, Brazil 3CSIRO Agriculture and Food, GPO Box 1700, Canberra ACT 2601, Australia.

```
HEADER    TRANSPORT PROTEIN                       08-JUL-16   5KSD
TITLE     CRYSTAL STRUCTURE OF A PLASMA MEMBRANE PROTON PUMP
COMPND    MOL_ID: 1;
COMPND   2 MOLECULE: ATPASE 2, PLASMA MEMBRANE-TYPE;
COMPND   3 CHAIN: A, B;
COMPND   4 SYNONYM: PROTON PUMP 2;
COMPND   5 EC: 3.6.3.6;
COMPND   6 ENGINEERED: YES
SOURCE    MOL_ID: 1;
SOURCE   2 ORGANISM_SCIENTIFIC: ARABIDOPSIS THALIANA;
SOURCE   3 ORGANISM_COMMON: MOUSE-EAR CRESS;
SOURCE   4 ORGANISM_TAXID: 3702;
SOURCE   5 GENE: AHA2, AT4G30190, F9N11.40;
SOURCE   6 EXPRESSION_SYSTEM: SACCHAROMYCES CEREVISIAE;
SOURCE   7 EXPRESSION_SYSTEM_TAXID: 4932;
SOURCE   8 EXPRESSION_SYSTEM_STRAIN: RS-72;
SOURCE   9 EXPRESSION_SYSTEM_VECTOR_TYPE: PLASMID;
SOURCE  10 EXPRESSION_SYSTEM_PLASMID: PMP-652
KEYWDS    P-TYPE ATPASE PROTON TRANSPORT, TRANSPORT PROTEIN
EXPDTA    X-RAY DIFFRACTION
AUTHOR    T.CROLL,B.P.PEDERSEN,P.NISSEN
REVDAT   3   17-MAY-17 5KSD    1       JRNL
REVDAT   2   10-MAY-17 5KSD    1       JRNL
REVDAT   1   10-AUG-16 5KSD    0
SPRSDE     10-AUG-16 5KSD      3B8C
JRNL        AUTH   D.FOCHT,T.I.CROLL,B.P.PEDERSEN,P.NISSEN
JRNL        TITL   IMPROVED MODEL OF PROTON PUMP CRYSTAL STRUCTURE
OBTAINED BY
JRNL        TITL 2 INTERACTIVE MOLECULAR DYNAMICS FLEXIBLE FITTING
EXPANDS THE
JRNL        TITL 3 MECHANISTIC MODEL FOR PROTON TRANSLOCATION IN P-
TYPE
JRNL        TITL 4 ATPASES.
JRNL        REF    FRONT PHYSIOL                   V.   8   202 2017
JRNL        REFN                   ESSN 1664-042X
JRNL        PMID   28443028
JRNL        DOI    10.3389/FPHYS.2017.00202
REMARK   1
REMARK   1 REFERENCE 1
REMARK   1  AUTH   B.P.PEDERSEN,M.J.BUCH-
PEDERSEN,J.P.MORTH,M.G.PALMGREN,
REMARK   1  AUTH 2 P.NISSEN
REMARK   1  TITL   CRYSTAL STRUCTURE OF THE PLASMA MEMBRANE PROTON
```

```
PUMP.
REMARK   1  REF    NATURE                         V. 450  1111 2007
REMARK   1  REFN                  ESSN 1476-4687
REMARK   1  PMID   18075595
REMARK   1  DOI    10.1038/NATURE06417
REMARK   2
REMARK   2 RESOLUTION.    3.50 ANGSTROMS.
REMARK   3
REMARK   3 REFINEMENT.
REMARK   3   PROGRAM     : PHENIX DEV_2376
REMARK   3   AUTHORS     : PAUL ADAMS,PAVEL AFONINE,VINCENT CHEN,IAN
REMARK   3               : DAVIS,KRESHNA GOPAL,RALF GROSSE-
KUNSTLEVE,
REMARK   3               : LI-WEI HUNG,ROBERT IMMORMINO,TOM IOERGER,
REMARK   3               : AIRLIE MCCOY,ERIK MCKEE,NIGEL MORIARTY,
REMARK   3               : REETAL PAI,RANDY READ,JANE RICHARDSON,
REMARK   3               : DAVID RICHARDSON,TOD ROMO,JIM
SACCHETTINI,
REMARK   3               : NICHOLAS SAUTER,JACOB SMITH,LAURENT
REMARK   3               : STORONI,TOM TERWILLIGER,PETER ZWART
REMARK   3
REMARK   3    REFINEMENT TARGET : NULL
REMARK   3
REMARK   3  DATA USED IN REFINEMENT.
REMARK   3   RESOLUTION RANGE HIGH (ANGSTROMS) : 3.50
REMARK   3   RESOLUTION RANGE LOW  (ANGSTROMS) : 52.02
REMARK   3   MIN(FOBS/SIGMA_FOBS)              : 1.380
REMARK   3   COMPLETENESS FOR RANGE        (%) : 99.7
REMARK   3   NUMBER OF REFLECTIONS             : 49405
REMARK   3
REMARK   3  FIT TO DATA USED IN REFINEMENT.
REMARK   3   R VALUE     (WORKING + TEST SET) : 0.289
REMARK   3   R VALUE            (WORKING SET) : 0.287
REMARK   3   FREE R VALUE                     : 0.324
REMARK   3   FREE R VALUE TEST SET SIZE   (%) : 4.040
REMARK   3   FREE R VALUE TEST SET COUNT      : 1998
REMARK   3
REMARK   3  FIT TO DATA USED IN REFINEMENT (IN BINS).
REMARK   3   BIN  RESOLUTION RANGE  COMPL.    NWORK NFREE   RWORK
RFREE
REMARK   3     1 52.0241 -  8.4246    0.98     3559   149  0.2580
0.3287
REMARK   3     2  8.4246 -  6.6913    1.00     3482   147  0.2584
0.2695
REMARK   3     3  6.6913 -  5.8468    1.00     3435   145  0.2761
0.2916
REMARK   3     4  5.8468 -  5.3128    1.00     3386   143  0.2769
0.3395
REMARK   3     5  5.3128 -  4.9323    1.00     3394   143  0.2571
0.3213
REMARK   3     6  4.9323 -  4.6417    1.00     3390   143  0.2601
0.2647
REMARK   3     7  4.6417 -  4.4094    1.00     3364   142  0.2563
0.2902
```

```
REMARK   3     8  4.4094 -  4.2175    1.00     3354   141  0.2817
0.3160
REMARK   3     9  4.2175 -  4.0552    1.00     3343   141  0.3094
0.2966
REMARK   3    10  4.0552 -  3.9153    1.00     3359   142  0.3300
0.3628
REMARK   3    11  3.9153 -  3.7929    1.00     3385   143  0.3522
0.4128
REMARK   3    12  3.7929 -  3.6846    1.00     3332   140  0.3761
0.3970
REMARK   3    13  3.6846 -  3.5876    1.00     3339   140  0.4056
0.4177
REMARK   3    14  3.5876 -  3.5001    0.98     3285   139  0.4084
0.3910
REMARK   3
REMARK   3  BULK SOLVENT MODELLING.
REMARK   3   METHOD USED        : NULL
REMARK   3   SOLVENT RADIUS     : 1.11
REMARK   3   SHRINKAGE RADIUS   : 0.90
REMARK   3   K_SOL              : NULL
REMARK   3   B_SOL              : NULL
REMARK   3
REMARK   3  ERROR ESTIMATES.
REMARK   3   COORDINATE ERROR (MAXIMUM-LIKELIHOOD BASED)     : 0.680
REMARK   3   PHASE ERROR (DEGREES, MAXIMUM-LIKELIHOOD BASED) :
37.360
REMARK   3
REMARK   3  B VALUES.
REMARK   3   FROM WILSON PLOT           (A**2) : 109.6
REMARK   3   MEAN B VALUE      (OVERALL, A**2) : 139.6
REMARK   3   OVERALL ANISOTROPIC B VALUE.
REMARK   3    B11 (A**2) : NULL
REMARK   3    B22 (A**2) : NULL
REMARK   3    B33 (A**2) : NULL
REMARK   3    B12 (A**2) : NULL
REMARK   3    B13 (A**2) : NULL
REMARK   3    B23 (A**2) : NULL
REMARK   3
REMARK   3  TWINNING INFORMATION.
REMARK   3   FRACTION: NULL
REMARK   3   OPERATOR: NULL
REMARK   3
REMARK   3  DEVIATIONS FROM IDEAL VALUES.
REMARK   3                RMSD          COUNT
REMARK   3   BOND      :  0.003         13222
REMARK   3   ANGLE     :  0.873         17928
REMARK   3   CHIRALITY :  0.048          2092
REMARK   3   PLANARITY :  0.010          2248
REMARK   3   DIHEDRAL  : 17.191          7892
REMARK   3
REMARK   3  TLS DETAILS
REMARK   3   NUMBER OF TLS GROUPS  : 6
REMARK   3   TLS GROUP : 1
REMARK   3    SELECTION: (CHAIN A AND (RESID 12:479 OR RESID 1001 OR
```

```
RESID
REMARK   3                1002 OR RESID 1003 OR RESID 1006))
REMARK   3    ORIGIN FOR THE GROUP (A): -16.2064 -16.5817  35.0501
REMARK   3    T TENSOR
REMARK   3      T11:   1.0067 T22:   1.6661
REMARK   3      T33:   0.9703 T12:  -0.0422
REMARK   3      T13:   0.1583 T23:   0.0331
REMARK   3    L TENSOR
REMARK   3      L11:   1.6521 L22:   1.7824
REMARK   3      L33:   2.9441 L12:   1.5329
REMARK   3      L13:  -1.1685 L23:  -1.5796
REMARK   3    S TENSOR
REMARK   3      S11:   0.0740 S12:   0.1314 S13:   0.0962
REMARK   3      S21:  -0.3202 S22:   0.6792 S23:   0.6111
REMARK   3      S31:   0.3851 S32:  -1.6194 S33:  -0.5954
REMARK   3   TLS GROUP : 2
REMARK   3    SELECTION: (CHAIN A AND (RESID 480:703 OR RESID 1005))
REMARK   3    ORIGIN FOR THE GROUP (A):  -1.4642  -2.5672  33.6865
REMARK   3    T TENSOR
REMARK   3      T11:   0.7953 T22:   1.3193
REMARK   3      T33:   0.6883 T12:  -0.0494
REMARK   3      T13:   0.0107 T23:  -0.3058
REMARK   3    L TENSOR
REMARK   3      L11:   0.5072 L22:   2.8500
REMARK   3      L33:   4.0622 L12:  -1.0419
REMARK   3      L13:   0.0608 L23:  -1.4186
REMARK   3    S TENSOR
REMARK   3      S11:  -0.0345 S12:  -0.5002 S13:   0.2022
REMARK   3      S21:   0.2476 S22:   0.1873 S23:   0.3703
REMARK   3      S31:  -0.8664 S32:  -0.5776 S33:  -0.1109
REMARK   3   TLS GROUP : 3
REMARK   3    SELECTION: (CHAIN A AND (RESID 704:844 OR RESID 1007))
REMARK   3    ORIGIN FOR THE GROUP (A):  14.7174   1.7972  -0.4317
REMARK   3    T TENSOR
REMARK   3      T11:   1.0701 T22:   0.8192
REMARK   3      T33:   0.7566 T12:  -0.2166
REMARK   3      T13:   0.0569 T23:   0.2255
REMARK   3    L TENSOR
REMARK   3      L11:   2.8300 L22:   3.6640
REMARK   3      L33:   1.7845 L12:   0.1318
REMARK   3      L13:   0.5559 L23:   1.7385
REMARK   3    S TENSOR
REMARK   3      S11:  -0.1006 S12:   0.8057 S13:  -0.2103
REMARK   3      S21:   0.4945 S22:   0.2419 S23:  -0.4036
REMARK   3      S31:  -0.7286 S32:   0.5032 S33:  -0.0559
REMARK   3   TLS GROUP : 4
REMARK   3    SELECTION: (CHAIN B AND (RESID 12:479 OR RESID 1001 OR
RESID
REMARK   3                1002 OR RESID 1003 OR RESID 1006))
REMARK   3    ORIGIN FOR THE GROUP (A):  16.7653 -40.1589  73.9063
REMARK   3    T TENSOR
REMARK   3      T11:   1.8489 T22:   1.6781
REMARK   3      T33:   1.1023 T12:  -0.0693
REMARK   3      T13:  -0.2539 T23:  -0.0333
```

```
REMARK   3    L TENSOR
REMARK   3      L11:  -0.0247 L22:   1.0867
REMARK   3      L33:   3.9840 L12:   0.0162
REMARK   3      L13:   1.1906 L23:   1.6723
REMARK   3    S TENSOR
REMARK   3      S11:   0.5394 S12:   0.1291 S13:  -0.7395
REMARK   3      S21:   0.8180 S22:   0.1107 S23:  -0.0977
REMARK   3      S31:   2.1709 S32:   0.1719 S33:  -0.6028
REMARK   3   TLS GROUP : 5
REMARK   3    SELECTION: (CHAIN B AND (RESID 480:703 OR RESID 1005))
REMARK   3    ORIGIN FOR THE GROUP (A):  24.9544 -21.6986  75.5427
REMARK   3    T TENSOR
REMARK   3      T11:   0.8864 T22:   0.8661
REMARK   3      T33:   0.5696 T12:  -0.1325
REMARK   3      T13:  -0.1075 T23:  -0.0804
REMARK   3    L TENSOR
REMARK   3      L11:   3.5475 L22:   0.7274
REMARK   3      L33:   4.8799 L12:   0.4908
REMARK   3      L13:   1.6638 L23:   0.7820
REMARK   3    S TENSOR
REMARK   3      S11:   0.4434 S12:  -0.2278 S13:  -0.1369
REMARK   3      S21:   0.0581 S22:  -0.1759 S23:  -0.2318
REMARK   3      S31:   0.1864 S32:   0.3055 S33:  -0.4473
REMARK   3   TLS GROUP : 6
REMARK   3    SELECTION: (CHAIN B AND (RESID 704:844 OR RESID 1007))
REMARK   3    ORIGIN FOR THE GROUP (A):  24.1436  -5.7696 110.1553
REMARK   3    T TENSOR
REMARK   3      T11:   0.8981 T22:   0.8658
REMARK   3      T33:   0.9135 T12:  -0.9531
REMARK   3      T13:  -0.5711 T23:  -0.0745
REMARK   3    L TENSOR
REMARK   3      L11:   2.6903 L22:   1.8544
REMARK   3      L33:   0.6825 L12:   0.7000
REMARK   3      L13:   1.0256 L23:  -0.1894
REMARK   3    S TENSOR
REMARK   3      S11:  -0.1232 S12:  -0.9612 S13:   0.0070
REMARK   3      S21:   0.8915 S22:   0.2188 S23:   0.2359
REMARK   3      S31:  -0.5339 S32:  -0.5188 S33:   0.1913
REMARK   3
REMARK   3  NCS DETAILS
REMARK   3   NUMBER OF NCS GROUPS : NULL
REMARK   3
REMARK   3  OTHER REFINEMENT REMARKS: NULL
REMARK   4
REMARK   4 5KSD COMPLIES WITH FORMAT V. 3.30, 13-JUL-11
REMARK 100
REMARK 100 THIS ENTRY HAS BEEN PROCESSED BY PDBE ON 13-JUL-16.
REMARK 100 THE DEPOSITION ID IS D_1000220780.
REMARK 200
REMARK 200 EXPERIMENTAL DETAILS
REMARK 200  EXPERIMENT TYPE                : X-RAY DIFFRACTION
REMARK 200  DATE OF DATA COLLECTION        : 03-DEC-06
REMARK 200  TEMPERATURE           (KELVIN) : 100
REMARK 200  PH                             : 6.0
```

```
REMARK 200  NUMBER OF CRYSTALS USED        : 1
REMARK 200
REMARK 200  SYNCHROTRON              (Y/N) : Y
REMARK 200  RADIATION SOURCE               : SLS
REMARK 200  BEAMLINE                       : X06SA
REMARK 200  X-RAY GENERATOR MODEL          : NULL
REMARK 200  MONOCHROMATIC OR LAUE    (M/L) : M
REMARK 200  WAVELENGTH OR RANGE        (A) : 1.007829
REMARK 200  MONOCHROMATOR                  : SI(111) MONOCHROMATOR
REMARK 200  OPTICS                         : NULL
REMARK 200
REMARK 200  DETECTOR TYPE                  : CCD
REMARK 200  DETECTOR MANUFACTURER          : MARMOSAIC 225 MM CCD
REMARK 200  INTENSITY-INTEGRATION SOFTWARE : XDS
REMARK 200  DATA SCALING SOFTWARE          : XSCALE
REMARK 200
REMARK 200  NUMBER OF UNIQUE REFLECTIONS   : 49499
REMARK 200  RESOLUTION RANGE HIGH      (A) : 3.500
REMARK 200  RESOLUTION RANGE LOW       (A) : 100.000
REMARK 200  REJECTION CRITERIA  (SIGMA(I)) : -3.000
REMARK 200
REMARK 200 OVERALL.
REMARK 200  COMPLETENESS FOR RANGE     (%) : 99.8
REMARK 200  DATA REDUNDANCY                : 7.700
REMARK 200  R MERGE                    (I) : 0.15400
REMARK 200  R SYM                      (I) : NULL
REMARK 200  <I/SIGMA(I)> FOR THE DATA SET  : 8.6700
REMARK 200
REMARK 200 IN THE HIGHEST RESOLUTION SHELL.
REMARK 200  HIGHEST RESOLUTION SHELL, RANGE HIGH (A) : 3.50
REMARK 200  HIGHEST RESOLUTION SHELL, RANGE LOW  (A) : 3.60
REMARK 200  COMPLETENESS FOR SHELL     (%) : 99.9
REMARK 200  DATA REDUNDANCY IN SHELL       : NULL
REMARK 200  R MERGE FOR SHELL          (I) : 0.84900
REMARK 200  R SYM FOR SHELL            (I) : NULL
REMARK 200  <I/SIGMA(I)> FOR SHELL         : 1.830
REMARK 200
REMARK 200 DIFFRACTION PROTOCOL: SINGLE WAVELENGTH
REMARK 200 METHOD USED TO DETERMINE THE STRUCTURE: SIRAS
REMARK 200 SOFTWARE USED: AUTOSHARP
REMARK 200 STARTING MODEL: NULL
REMARK 200
REMARK 200 REMARK: LARGE CRYSTALS, OFTEN WITH A GROWTH DEFECT IN THE
CENTER.
REMARK 200  SEE PEDERSEN BP ET AL NATURE 450 (2007) SUPPLEMENTARY
REMARK 200  INFORMATION FOR DETAILS AND PICTURES.
REMARK 280
REMARK 280 CRYSTAL
REMARK 280 SOLVENT CONTENT, VS   (%): 75.04
REMARK 280 MATTHEWS COEFFICIENT, VM (ANGSTROMS**3/DA): 4.93
REMARK 280
REMARK 280 CRYSTALLIZATION CONDITIONS: PEG 400, SUCROSE, KCL, C12E8,
CYMAL-5,
REMARK 280  DDM, MES PH 6.0, VAPOR DIFFUSION, HANGING DROP,
```

```
TEMPERATURE 277K
REMARK 290
REMARK 290 CRYSTALLOGRAPHIC SYMMETRY
REMARK 290 SYMMETRY OPERATORS FOR SPACE GROUP: P 21 21 21
REMARK 290
REMARK 290      SYMOP   SYMMETRY
REMARK 290     NNNMMM   OPERATOR
REMARK 290       1555   X,Y,Z
REMARK 290       2555   -X+1/2,-Y,Z+1/2
REMARK 290       3555   -X,Y+1/2,-Z+1/2
REMARK 290       4555   X+1/2,-Y+1/2,-Z
REMARK 290
REMARK 290     WHERE NNN -> OPERATOR NUMBER
REMARK 290           MMM -> TRANSLATION VECTOR
REMARK 290
REMARK 290 CRYSTALLOGRAPHIC SYMMETRY TRANSFORMATIONS
REMARK 290 THE FOLLOWING TRANSFORMATIONS OPERATE ON THE ATOM/HETATM
REMARK 290 RECORDS IN THIS ENTRY TO PRODUCE CRYSTALLOGRAPHICALLY
REMARK 290 RELATED MOLECULES.
REMARK 290   SMTRY1   1  1.000000  0.000000  0.000000        0.00000
REMARK 290   SMTRY2   1  0.000000  1.000000  0.000000        0.00000
REMARK 290   SMTRY3   1  0.000000  0.000000  1.000000        0.00000
REMARK 290   SMTRY1   2 -1.000000  0.000000  0.000000       42.64500
REMARK 290   SMTRY2   2  0.000000 -1.000000  0.000000        0.00000
REMARK 290   SMTRY3   2  0.000000  0.000000  1.000000      156.05500
REMARK 290   SMTRY1   3 -1.000000  0.000000  0.000000        0.00000
REMARK 290   SMTRY2   3  0.000000  1.000000  0.000000       72.21000
REMARK 290   SMTRY3   3  0.000000  0.000000 -1.000000      156.05500
REMARK 290   SMTRY1   4  1.000000  0.000000  0.000000       42.64500
REMARK 290   SMTRY2   4  0.000000 -1.000000  0.000000       72.21000
REMARK 290   SMTRY3   4  0.000000  0.000000 -1.000000        0.00000
REMARK 290
REMARK 290 REMARK: NULL
REMARK 300
REMARK 300 BIOMOLECULE: 1, 2
REMARK 300 SEE REMARK 350 FOR THE AUTHOR PROVIDED AND/OR PROGRAM
REMARK 300 GENERATED ASSEMBLY INFORMATION FOR THE STRUCTURE IN
REMARK 300 THIS ENTRY. THE REMARK MAY ALSO PROVIDE INFORMATION ON
REMARK 300 BURIED SURFACE AREA.
REMARK 350
REMARK 350 COORDINATES FOR A COMPLETE MULTIMER REPRESENTING THE
KNOWN
REMARK 350 BIOLOGICALLY SIGNIFICANT OLIGOMERIZATION STATE OF THE
REMARK 350 MOLECULE CAN BE GENERATED BY APPLYING BIOMT
TRANSFORMATIONS
REMARK 350 GIVEN BELOW.  BOTH NON-CRYSTALLOGRAPHIC AND
REMARK 350 CRYSTALLOGRAPHIC OPERATIONS ARE GIVEN.
REMARK 350
REMARK 350 BIOMOLECULE: 1
REMARK 350 AUTHOR DETERMINED BIOLOGICAL UNIT: MONOMERIC
REMARK 350 APPLY THE FOLLOWING TO CHAINS: A
REMARK 350   BIOMT1   1  1.000000  0.000000  0.000000        0.00000
REMARK 350   BIOMT2   1  0.000000  1.000000  0.000000        0.00000
REMARK 350   BIOMT3   1  0.000000  0.000000  1.000000        0.00000
```

```
REMARK 350
REMARK 350 BIOMOLECULE: 2
REMARK 350 AUTHOR DETERMINED BIOLOGICAL UNIT: MONOMERIC
REMARK 350 APPLY THE FOLLOWING TO CHAINS: B
REMARK 350   BIOMT1   1  1.000000  0.000000  0.000000        0.00000
REMARK 350   BIOMT2   1  0.000000  1.000000  0.000000        0.00000
REMARK 350   BIOMT3   1  0.000000  0.000000  1.000000        0.00000
REMARK 500
REMARK 500 GEOMETRY AND STEREOCHEMISTRY
REMARK 500 SUBTOPIC: COVALENT BOND LENGTHS
REMARK 500
REMARK 500 THE STEREOCHEMICAL PARAMETERS OF THE FOLLOWING RESIDUES
REMARK 500 HAVE VALUES WHICH DEVIATE FROM EXPECTED VALUES BY MORE
REMARK 500 THAN 6*RMSD (M=MODEL NUMBER; RES=RESIDUE NAME; C=CHAIN
REMARK 500 IDENTIFIER; SSEQ=SEQUENCE NUMBER; I=INSERTION CODE).
REMARK 500
REMARK 500 STANDARD TABLE:
REMARK 500 FORMAT: (10X,I3,1X,2(A3,1X,A1,I4,A1,1X,A4,3X),1X,F6.3)
REMARK 500
REMARK 500 EXPECTED VALUES PROTEIN: ENGH AND HUBER, 1999
REMARK 500 EXPECTED VALUES NUCLEIC ACID: CLOWNEY ET AL 1996
REMARK 500
REMARK 500  M RES CSSEQI ATM1   RES CSSEQI ATM2   DEVIATION
REMARK 500    ILE A 285   C     PRO A 286   N       0.157
REMARK 500
REMARK 500 REMARK: NULL
REMARK 500
REMARK 500 GEOMETRY AND STEREOCHEMISTRY
REMARK 500 SUBTOPIC: COVALENT BOND ANGLES
REMARK 500
REMARK 500 THE STEREOCHEMICAL PARAMETERS OF THE FOLLOWING RESIDUES
REMARK 500 HAVE VALUES WHICH DEVIATE FROM EXPECTED VALUES BY MORE
REMARK 500 THAN 6*RMSD (M=MODEL NUMBER; RES=RESIDUE NAME; C=CHAIN
REMARK 500 IDENTIFIER; SSEQ=SEQUENCE NUMBER; I=INSERTION CODE).
REMARK 500
REMARK 500 STANDARD TABLE:
REMARK 500 FORMAT: (10X,I3,1X,A3,1X,A1,I4,A1,3(1X,A4,2X),12X,F5.1)
REMARK 500
REMARK 500 EXPECTED VALUES PROTEIN: ENGH AND HUBER, 1999
REMARK 500 EXPECTED VALUES NUCLEIC ACID: CLOWNEY ET AL 1996
REMARK 500
REMARK 500  M RES CSSEQI ATM1   ATM2   ATM3
REMARK 500    ARG B 773   CD  -  NE  -  CZ  ANGL. DEV. =  13.3
DEGREES
REMARK 500
REMARK 500 REMARK: NULL
REMARK 500
REMARK 500 GEOMETRY AND STEREOCHEMISTRY
REMARK 500 SUBTOPIC: TORSION ANGLES
REMARK 500
REMARK 500 TORSION ANGLES OUTSIDE THE EXPECTED RAMACHANDRAN REGIONS:
REMARK 500 (M=MODEL NUMBER; RES=RESIDUE NAME; C=CHAIN IDENTIFIER;
REMARK 500 SSEQ=SEQUENCE NUMBER; I=INSERTION CODE).
REMARK 500
```

```
REMARK 500 STANDARD TABLE:
REMARK 500 FORMAT:(10X,I3,1X,A3,1X,A1,I4,A1,4X,F7.2,3X,F7.2)
REMARK 500
REMARK 500 EXPECTED VALUES: GJ KLEYWEGT AND TA JONES (1996). PHI/
PSI-
REMARK 500 CHOLOGY: RAMACHANDRAN REVISITED. STRUCTURE 4, 1395 - 1400
REMARK 500
REMARK 500  M RES CSSEQI        PSI       PHI
REMARK 500    LEU A  14      -87.60   -134.34
REMARK 500    GLU A  15     -135.40     44.58
REMARK 500    GLU A  20     -138.74     56.89
REMARK 500    LYS A  27      -69.02    -98.75
REMARK 500    THR A  34      117.83    -25.62
REMARK 500    TRP A  66       36.38   -161.85
REMARK 500    PRO A 150      131.97    -39.13
REMARK 500    LEU A 169      -67.24    -92.80
REMARK 500    ALA A 180      -52.09     77.73
REMARK 500    PRO A 192      133.26    -37.82
REMARK 500    ILE A 212      -62.27   -102.66
REMARK 500    HIS A 225      -92.42    -96.30
REMARK 500    ILE A 257      -89.35    -84.50
REMARK 500    TYR A 270      -74.74   -100.99
REMARK 500    GLU A 369      -72.94   -108.16
REMARK 500    ALA A 383      -42.08     82.49
REMARK 500    LYS A 434      107.83    -59.90
REMARK 500    PRO A 476      123.63    -37.98
REMARK 500    ASP A 543     -121.25     52.61
REMARK 500    ARG A 579       31.14    -75.13
REMARK 500    LYS A 580      -18.66     67.98
REMARK 500    ASP A 588      -27.95   -141.43
REMARK 500    HIS A 732      -82.07   -136.26
REMARK 500    ARG A 747      -66.16   -134.71
REMARK 500    SER A 776     -152.74    -97.17
REMARK 500    LEU B  14      -90.13    -91.18
REMARK 500    GLU B  15     -120.05     60.32
REMARK 500    LEU B  26      -54.04   -125.51
REMARK 500    THR B  34      125.13    -27.01
REMARK 500    GLN B  43       34.39    -81.29
REMARK 500    ASN B  48       -5.71     70.15
REMARK 500    LEU B  50       64.05     60.82
REMARK 500    GLU B  52      -45.75     81.08
REMARK 500    LEU B  58      164.32    -49.16
REMARK 500    PRO B 150      140.00    -39.07
REMARK 500    LEU B 169      -75.33    -98.15
REMARK 500    ALA B 180      -48.78     77.85
REMARK 500    THR B 182      -23.07   -143.74
REMARK 500    PRO B 192      129.72    -38.86
REMARK 500    ILE B 212      -20.30   -141.87
REMARK 500    ILE B 257      -90.26    -86.05
REMARK 500    TYR B 270      -81.36   -121.86
REMARK 500    MET B 323      124.26    -35.30
REMARK 500    CYS B 350      -46.46     80.65
REMARK 500    GLU B 369      -59.54   -132.79
REMARK 500    ASP B 384       96.69   -167.78
```

```
REMARK 500    ASN B 437      -49.11     79.03
REMARK 500    PRO B 476      127.36    -37.39
REMARK 500    THR B 540      -65.96   -145.12
REMARK 500    ASP B 543     -175.85     63.29
REMARK 500
REMARK 500 THIS ENTRY HAS      59 RAMACHANDRAN OUTLIERS.
REMARK 500
REMARK 500 REMARK: NULL
REMARK 500
REMARK 500 GEOMETRY AND STEREOCHEMISTRY
REMARK 500 SUBTOPIC: NON-CIS, NON-TRANS
REMARK 500
REMARK 500 THE FOLLOWING PEPTIDE BONDS DEVIATE SIGNIFICANTLY FROM
BOTH
REMARK 500 CIS AND TRANS CONFORMATION.  CIS BONDS, IF ANY, ARE
LISTED
REMARK 500 ON CISPEP RECORDS.  TRANS IS DEFINED AS 180 +/- 30 AND
REMARK 500 CIS IS DEFINED AS 0 +/- 30 DEGREES.
REMARK 500                                 MODEL     OMEGA
REMARK 500 ASP A  487     PRO A  488                 -70.96
REMARK 500 SER B  472     PRO B  473                 149.99
REMARK 500 ASP B  487     PRO B  488                 -42.20
REMARK 500
REMARK 500 REMARK: NULL
REMARK 500
REMARK 500 GEOMETRY AND STEREOCHEMISTRY
REMARK 500 SUBTOPIC: PLANAR GROUPS
REMARK 500
REMARK 500 PLANAR GROUPS IN THE FOLLOWING RESIDUES HAVE A TOTAL
REMARK 500 RMS DISTANCE OF ALL ATOMS FROM THE BEST-FIT PLANE
REMARK 500 BY MORE THAN AN EXPECTED VALUE OF 6*RMSD, WITH AN
REMARK 500 RMSD 0.02 ANGSTROMS, OR AT LEAST ONE ATOM HAS
REMARK 500 AN RMSD GREATER THAN THIS VALUE
REMARK 500 (M=MODEL NUMBER; RES=RESIDUE NAME; C=CHAIN IDENTIFIER;
REMARK 500 SSEQ=SEQUENCE NUMBER; I=INSERTION CODE).
REMARK 500
REMARK 500  M RES CSSEQI        RMS     TYPE
REMARK 500    ARG B 456         0.10    SIDE CHAIN
REMARK 500    ARG B 773         0.14    SIDE CHAIN
REMARK 500
REMARK 500 REMARK: NULL
REMARK 620
REMARK 620 METAL COORDINATION
REMARK 620 (M=MODEL NUMBER; RES=RESIDUE NAME; C=CHAIN IDENTIFIER;
REMARK 620 SSEQ=SEQUENCE NUMBER; I=INSERTION CODE):
REMARK 620
REMARK 620 COORDINATION ANGLES FOR:  M RES CSSEQI METAL
REMARK 620                              MG A 902  MG
REMARK 620 N RES CSSEQI ATOM
REMARK 620 1 ASP A 372   OD1
REMARK 620 2 ASP A 372   OD2  63.3
REMARK 620 N                    1
REMARK 620
REMARK 620 COORDINATION ANGLES FOR:  M RES CSSEQI METAL
```

```
REMARK 620                              MG A 901  MG
REMARK 620 N RES CSSEQI ATOM
REMARK 620 1 ASP A 588   OD2
REMARK 620 2 ACP A 904   O1B 151.1
REMARK 620 3 ACP A 904   O1G 130.4  73.7
REMARK 620 N                    1     2
REMARK 620
REMARK 620 COORDINATION ANGLES FOR:  M RES CSSEQI METAL
REMARK 620                               K A 903   K
REMARK 620 N RES CSSEQI ATOM
REMARK 620 1 LEU A 596   O
REMARK 620 2 LYS A 597   O    72.2
REMARK 620 3 ALA A 599   O    79.5  89.5
REMARK 620 4 ASP A 617   OD1  74.8 144.9  95.8
REMARK 620 5 ASP A 617   OD2 120.7 158.2 109.4  46.5
REMARK 620 N                    1     2     3     4
REMARK 620
REMARK 620 COORDINATION ANGLES FOR:  M RES CSSEQI METAL
REMARK 620                              MG B 901  MG
REMARK 620 N RES CSSEQI ATOM
REMARK 620 1 ASP B 588   OD2
REMARK 620 2 ACP B 904   O2G 125.6
REMARK 620 3 ACP B 904   O2B 159.1  75.3
REMARK 620 N                    1     2
REMARK 620
REMARK 620 COORDINATION ANGLES FOR:  M RES CSSEQI METAL
REMARK 620                               K B 903   K
REMARK 620 N RES CSSEQI ATOM
REMARK 620 1 LEU B 596   O
REMARK 620 2 LYS B 597   O    68.8
REMARK 620 3 ALA B 599   O    74.1  78.8
REMARK 620 4 ASP B 617   OD1  74.9 140.7 105.5
REMARK 620 5 ASP B 617   OD2 120.6 158.7 121.3  46.1
REMARK 620 N                    1     2     3     4
REMARK 800
REMARK 800 SITE
REMARK 800 SITE_IDENTIFIER: AC1
REMARK 800 EVIDENCE_CODE: SOFTWARE
REMARK 800 SITE_DESCRIPTION: binding site for residue MG A 901
REMARK 800
REMARK 800 SITE_IDENTIFIER: AC2
REMARK 800 EVIDENCE_CODE: SOFTWARE
REMARK 800 SITE_DESCRIPTION: binding site for residue MG A 902
REMARK 800
REMARK 800 SITE_IDENTIFIER: AC3
REMARK 800 EVIDENCE_CODE: SOFTWARE
REMARK 800 SITE_DESCRIPTION: binding site for residue K A 903
REMARK 800
REMARK 800 SITE_IDENTIFIER: AC4
REMARK 800 EVIDENCE_CODE: SOFTWARE
REMARK 800 SITE_DESCRIPTION: binding site for residue ACP A 904
REMARK 800
REMARK 800 SITE_IDENTIFIER: AC5
REMARK 800 EVIDENCE_CODE: SOFTWARE
```

```
REMARK 800 SITE_DESCRIPTION: binding site for residue LMT A 905
REMARK 800
REMARK 800 SITE_IDENTIFIER: AC6
REMARK 800 EVIDENCE_CODE: SOFTWARE
REMARK 800 SITE_DESCRIPTION: binding site for residue MG B 901
REMARK 800
REMARK 800 SITE_IDENTIFIER: AC7
REMARK 800 EVIDENCE_CODE: SOFTWARE
REMARK 800 SITE_DESCRIPTION: binding site for residue MG B 902
REMARK 800
REMARK 800 SITE_IDENTIFIER: AC8
REMARK 800 EVIDENCE_CODE: SOFTWARE
REMARK 800 SITE_DESCRIPTION: binding site for residue K B 903
REMARK 800
REMARK 800 SITE_IDENTIFIER: AC9
REMARK 800 EVIDENCE_CODE: SOFTWARE
REMARK 800 SITE_DESCRIPTION: binding site for residue ACP B 904
REMARK 800
REMARK 800 SITE_IDENTIFIER: AD1
REMARK 800 EVIDENCE_CODE: SOFTWARE
REMARK 800 SITE_DESCRIPTION: binding site for residue LMT B 905
DBREF  5KSD A   12   844  UNP    P19456   PMA2_ARATH      12    844
DBREF  5KSD B   12   844  UNP    P19456   PMA2_ARATH      12    844
SEQRES   1 A  833  VAL ASP LEU GLU LYS ILE PRO ILE GLU GLU VAL PHE
GLN
SEQRES   2 A  833  GLN LEU LYS CYS SER ARG GLU GLY LEU THR THR GLN
GLU
SEQRES   3 A  833  GLY GLU ASP ARG ILE GLN ILE PHE GLY PRO ASN LYS
LEU
SEQRES   4 A  833  GLU GLU LYS LYS GLU SER LYS LEU LEU LYS PHE LEU
GLY
SEQRES   5 A  833  PHE MET TRP ASN PRO LEU SER TRP VAL MET GLU MET
ALA
SEQRES   6 A  833  ALA ILE MET ALA ILE ALA LEU ALA ASN GLY ASP GLY
ARG
SEQRES   7 A  833  PRO PRO ASP TRP GLN ASP PHE VAL GLY ILE ILE CYS
LEU
SEQRES   8 A  833  LEU VAL ILE ASN SER THR ILE SER PHE ILE GLU GLU
ASN
SEQRES   9 A  833  ASN ALA GLY ASN ALA ALA ALA ALA LEU MET ALA GLY
LEU
SEQRES  10 A  833  ALA PRO LYS THR LYS VAL LEU ARG ASP GLY LYS TRP
SER
SEQRES  11 A  833  GLU GLN GLU ALA ALA ILE LEU VAL PRO GLY ASP ILE
VAL
SEQRES  12 A  833  SER ILE LYS LEU GLY ASP ILE ILE PRO ALA ASP ALA
ARG
SEQRES  13 A  833  LEU LEU GLU GLY ASP PRO LEU LYS VAL ASP GLN SER
ALA
SEQRES  14 A  833  LEU THR GLY GLU SER LEU PRO VAL THR LYS HIS PRO
GLY
SEQRES  15 A  833  GLN GLU VAL PHE SER GLY SER THR CYS LYS GLN GLY
GLU
SEQRES  16 A  833  ILE GLU ALA VAL VAL ILE ALA THR GLY VAL HIS THR
```

PHE
SEQRES 17 A 833 PHE GLY LYS ALA ALA HIS LEU VAL ASP SER THR ASN
GLN
SEQRES 18 A 833 VAL GLY HIS PHE GLN LYS VAL LEU THR ALA ILE GLY
ASN
SEQRES 19 A 833 PHE CYS ILE CYS SER ILE ALA ILE GLY MET VAL ILE
GLU
SEQRES 20 A 833 ILE ILE VAL MET TYR PRO ILE GLN ARG ARG LYS TYR
ARG
SEQRES 21 A 833 ASP GLY ILE ASP ASN LEU LEU VAL LEU LEU ILE GLY
GLY
SEQRES 22 A 833 ILE PRO ILE ALA MET PRO THR VAL LEU SER VAL THR
MET
SEQRES 23 A 833 ALA ILE GLY SER HIS ARG LEU SER GLN GLN GLY ALA
ILE
SEQRES 24 A 833 THR LYS ARG MET THR ALA ILE GLU GLU MET ALA GLY
MET
SEQRES 25 A 833 ASP VAL LEU CYS SER ASP LYS THR GLY THR LEU THR
LEU
SEQRES 26 A 833 ASN LYS LEU SER VAL ASP LYS ASN LEU VAL GLU VAL
PHE
SEQRES 27 A 833 CYS LYS GLY VAL GLU LYS ASP GLN VAL LEU LEU PHE
ALA
SEQRES 28 A 833 ALA MET ALA SER ARG VAL GLU ASN GLN ASP ALA ILE
ASP
SEQRES 29 A 833 ALA ALA MET VAL GLY MET LEU ALA ASP PRO LYS GLU
ALA
SEQRES 30 A 833 ARG ALA GLY ILE ARG GLU VAL HIS PHE LEU PRO PHE
ASN
SEQRES 31 A 833 PRO VAL ASP LYS ARG THR ALA LEU THR TYR ILE ASP
GLY
SEQRES 32 A 833 SER GLY ASN TRP HIS ARG VAL SER LYS GLY ALA PRO
GLU
SEQRES 33 A 833 GLN ILE LEU GLU LEU ALA LYS ALA SER ASN ASP LEU
SER
SEQRES 34 A 833 LYS LYS VAL LEU SER ILE ILE ASP LYS TYR ALA GLU
ARG
SEQRES 35 A 833 GLY LEU ARG SER LEU ALA VAL ALA ARG GLN VAL VAL
PRO
SEQRES 36 A 833 GLU LYS THR LYS GLU SER PRO GLY ALA PRO TRP GLU
PHE
SEQRES 37 A 833 VAL GLY LEU LEU PRO LEU PHE ASP PRO PRO ARG HIS
ASP
SEQRES 38 A 833 SER ALA GLU THR ILE ARG ARG ALA LEU ASN LEU GLY
VAL
SEQRES 39 A 833 ASN VAL LYS MET ILE THR GLY ASP GLN LEU ALA ILE
GLY
SEQRES 40 A 833 LYS GLU THR GLY ARG ARG LEU GLY MET GLY THR ASN
MET
SEQRES 41 A 833 TYR PRO SER SER ALA LEU LEU GLY THR HIS LYS ASP
ALA
SEQRES 42 A 833 ASN LEU ALA SER ILE PRO VAL GLU GLU LEU ILE GLU
LYS
SEQRES 43 A 833 ALA ASP GLY PHE ALA GLY VAL PHE PRO GLU HIS LYS

TYR
SEQRES 44 A 833 GLU ILE VAL LYS LYS LEU GLN GLU ARG LYS HIS ILE
VAL
SEQRES 45 A 833 GLY MET THR GLY ASP GLY VAL ASN ASP ALA PRO ALA
LEU
SEQRES 46 A 833 LYS LYS ALA ASP ILE GLY ILE ALA VAL ALA ASP ALA
THR
SEQRES 47 A 833 ASP ALA ALA ARG GLY ALA SER ASP ILE VAL LEU THR
GLU
SEQRES 48 A 833 PRO GLY LEU SER VAL ILE ILE SER ALA VAL LEU THR
SER
SEQRES 49 A 833 ARG ALA ILE PHE GLN ARG MET LYS ASN TYR THR ILE
TYR
SEQRES 50 A 833 ALA VAL SER ILE THR ILE ARG ILE VAL PHE GLY PHE
MET
SEQRES 51 A 833 LEU ILE ALA LEU ILE TRP GLU PHE ASP PHE SER ALA
PHE
SEQRES 52 A 833 MET VAL LEU ILE ILE ALA ILE LEU ASN ASP GLY THR
ILE
SEQRES 53 A 833 MET THR ILE SER LYS ASP ARG VAL LYS PRO SER PRO
THR
SEQRES 54 A 833 PRO ASP SER TRP LYS LEU LYS GLU ILE PHE ALA THR
GLY
SEQRES 55 A 833 VAL VAL LEU GLY GLY TYR GLN ALA ILE MET THR VAL
ILE
SEQRES 56 A 833 PHE PHE TRP ALA ALA HIS LYS THR ASP PHE PHE SER
ASP
SEQRES 57 A 833 THR PHE GLY VAL ARG SER ILE ARG ASP ASN ASN HIS
GLU
SEQRES 58 A 833 LEU MET GLY ALA VAL TYR LEU GLN VAL SER ILE ILE
SER
SEQRES 59 A 833 GLN ALA LEU ILE PHE VAL THR ARG SER ARG SER TRP
SER
SEQRES 60 A 833 PHE VAL GLU ARG PRO GLY ALA LEU LEU MET ILE ALA
PHE
SEQRES 61 A 833 LEU ILE ALA GLN LEU ILE ALA THR LEU ILE ALA VAL
TYR
SEQRES 62 A 833 ALA ASN TRP GLU PHE ALA LYS ILE ARG GLY ILE GLY
TRP
SEQRES 63 A 833 GLY TRP ALA GLY VAL ILE TRP LEU TYR SER ILE VAL
THR
SEQRES 64 A 833 TYR PHE PRO LEU ASP VAL PHE LYS PHE ALA ILE ARG
TYR
SEQRES 65 A 833 ILE
SEQRES   1 B 833 VAL ASP LEU GLU LYS ILE PRO ILE GLU GLU VAL PHE
GLN
SEQRES   2 B 833 GLN LEU LYS CYS SER ARG GLU GLY LEU THR THR GLN
GLU
SEQRES   3 B 833 GLY GLU ASP ARG ILE GLN ILE PHE GLY PRO ASN LYS
LEU
SEQRES   4 B 833 GLU GLU LYS LYS GLU SER LYS LEU LEU LYS PHE LEU
GLY
SEQRES   5 B 833 PHE MET TRP ASN PRO LEU SER TRP VAL MET GLU MET
ALA

SEQRES   6 B 833 ALA ILE MET ALA ILE ALA LEU ALA ASN GLY ASP GLY
ARG
SEQRES   7 B 833 PRO PRO ASP TRP GLN ASP PHE VAL GLY ILE ILE CYS
LEU
SEQRES   8 B 833 LEU VAL ILE ASN SER THR ILE SER PHE ILE GLU GLU
ASN
SEQRES   9 B 833 ASN ALA GLY ASN ALA ALA ALA ALA LEU MET ALA GLY
LEU
SEQRES 10 B 833 ALA PRO LYS THR LYS VAL LEU ARG ASP GLY LYS TRP
SER
SEQRES 11 B 833 GLU GLN GLU ALA ALA ILE LEU VAL PRO GLY ASP ILE
VAL
SEQRES 12 B 833 SER ILE LYS LEU GLY ASP ILE ILE PRO ALA ASP ALA
ARG
SEQRES 13 B 833 LEU LEU GLU GLY ASP PRO LEU LYS VAL ASP GLN SER
ALA
SEQRES 14 B 833 LEU THR GLY GLU SER LEU PRO VAL THR LYS HIS PRO
GLY
SEQRES 15 B 833 GLN GLU VAL PHE SER GLY SER THR CYS LYS GLN GLY
GLU
SEQRES 16 B 833 ILE GLU ALA VAL VAL ILE ALA THR GLY VAL HIS THR
PHE
SEQRES 17 B 833 PHE GLY LYS ALA ALA HIS LEU VAL ASP SER THR ASN
GLN
SEQRES 18 B 833 VAL GLY HIS PHE GLN LYS VAL LEU THR ALA ILE GLY
ASN
SEQRES 19 B 833 PHE CYS ILE CYS SER ILE ALA ILE GLY MET VAL ILE
GLU
SEQRES 20 B 833 ILE ILE VAL MET TYR PRO ILE GLN ARG ARG LYS TYR
ARG
SEQRES 21 B 833 ASP GLY ILE ASP ASN LEU LEU VAL LEU LEU ILE GLY
GLY
SEQRES 22 B 833 ILE PRO ILE ALA MET PRO THR VAL LEU SER VAL THR
MET
SEQRES 23 B 833 ALA ILE GLY SER HIS ARG LEU SER GLN GLN GLY ALA
ILE
SEQRES 24 B 833 THR LYS ARG MET THR ALA ILE GLU GLU MET ALA GLY
MET
SEQRES 25 B 833 ASP VAL LEU CYS SER ASP LYS THR GLY THR LEU THR
LEU
SEQRES 26 B 833 ASN LYS LEU SER VAL ASP LYS ASN LEU VAL GLU VAL
PHE
SEQRES 27 B 833 CYS LYS GLY VAL GLU LYS ASP GLN VAL LEU LEU PHE
ALA
SEQRES 28 B 833 ALA MET ALA SER ARG VAL GLU ASN GLN ASP ALA ILE
ASP
SEQRES 29 B 833 ALA ALA MET VAL GLY MET LEU ALA ASP PRO LYS GLU
ALA
SEQRES 30 B 833 ARG ALA GLY ILE ARG GLU VAL HIS PHE LEU PRO PHE
ASN
SEQRES 31 B 833 PRO VAL ASP LYS ARG THR ALA LEU THR TYR ILE ASP
GLY
SEQRES 32 B 833 SER GLY ASN TRP HIS ARG VAL SER LYS GLY ALA PRO
GLU

SEQRES 33 B 833 GLN ILE LEU GLU LEU ALA LYS ALA SER ASN ASP LEU
SER
SEQRES 34 B 833 LYS LYS VAL LEU SER ILE ILE ASP LYS TYR ALA GLU
ARG
SEQRES 35 B 833 GLY LEU ARG SER LEU ALA VAL ALA ARG GLN VAL VAL
PRO
SEQRES 36 B 833 GLU LYS THR LYS GLU SER PRO GLY ALA PRO TRP GLU
PHE
SEQRES 37 B 833 VAL GLY LEU LEU PRO LEU PHE ASP PRO PRO ARG HIS
ASP
SEQRES 38 B 833 SER ALA GLU THR ILE ARG ARG ALA LEU ASN LEU GLY
VAL
SEQRES 39 B 833 ASN VAL LYS MET ILE THR GLY ASP GLN LEU ALA ILE
GLY
SEQRES 40 B 833 LYS GLU THR GLY ARG ARG LEU GLY MET GLY THR ASN
MET
SEQRES 41 B 833 TYR PRO SER SER ALA LEU LEU GLY THR HIS LYS ASP
ALA
SEQRES 42 B 833 ASN LEU ALA SER ILE PRO VAL GLU GLU LEU ILE GLU
LYS
SEQRES 43 B 833 ALA ASP GLY PHE ALA GLY VAL PHE PRO GLU HIS LYS
TYR
SEQRES 44 B 833 GLU ILE VAL LYS LYS LEU GLN GLU ARG LYS HIS ILE
VAL
SEQRES 45 B 833 GLY MET THR GLY ASP GLY VAL ASN ASP ALA PRO ALA
LEU
SEQRES 46 B 833 LYS LYS ALA ASP ILE GLY ILE ALA VAL ALA ASP ALA
THR
SEQRES 47 B 833 ASP ALA ALA ARG GLY ALA SER ASP ILE VAL LEU THR
GLU
SEQRES 48 B 833 PRO GLY LEU SER VAL ILE ILE SER ALA VAL LEU THR
SER
SEQRES 49 B 833 ARG ALA ILE PHE GLN ARG MET LYS ASN TYR THR ILE
TYR
SEQRES 50 B 833 ALA VAL SER ILE THR ILE ARG ILE VAL PHE GLY PHE
MET
SEQRES 51 B 833 LEU ILE ALA LEU ILE TRP GLU PHE ASP PHE SER ALA
PHE
SEQRES 52 B 833 MET VAL LEU ILE ILE ALA ILE LEU ASN ASP GLY THR
ILE
SEQRES 53 B 833 MET THR ILE SER LYS ASP ARG VAL LYS PRO SER PRO
THR
SEQRES 54 B 833 PRO ASP SER TRP LYS LEU LYS GLU ILE PHE ALA THR
GLY
SEQRES 55 B 833 VAL VAL LEU GLY GLY TYR GLN ALA ILE MET THR VAL
ILE
SEQRES 56 B 833 PHE PHE TRP ALA ALA HIS LYS THR ASP PHE PHE SER
ASP
SEQRES 57 B 833 THR PHE GLY VAL ARG SER ILE ARG ASP ASN ASN HIS
GLU
SEQRES 58 B 833 LEU MET GLY ALA VAL TYR LEU GLN VAL SER ILE ILE
SER
SEQRES 59 B 833 GLN ALA LEU ILE PHE VAL THR ARG SER ARG SER TRP
SER

```
SEQRES  60 B  833  PHE VAL GLU ARG PRO GLY ALA LEU LEU MET ILE ALA
PHE
SEQRES  61 B  833  LEU ILE ALA GLN LEU ILE ALA THR LEU ILE ALA VAL
TYR
SEQRES  62 B  833  ALA ASN TRP GLU PHE ALA LYS ILE ARG GLY ILE GLY
TRP
SEQRES  63 B  833  GLY TRP ALA GLY VAL ILE TRP LEU TYR SER ILE VAL
THR
SEQRES  64 B  833  TYR PHE PRO LEU ASP VAL PHE LYS PHE ALA ILE ARG
TYR
SEQRES  65 B  833  ILE
HET     MG  A 901       1
HET     MG  A 902       1
HET      K  A 903       1
HET    ACP  A 904      31
HET    LMT  A 905      35
HET     MG  B 901       1
HET     MG  B 902       1
HET      K  B 903       1
HET    ACP  B 904      31
HET    LMT  B 905      35
HETNAM      MG MAGNESIUM ION
HETNAM       K POTASSIUM ION
HETNAM     ACP PHOSPHOMETHYLPHOSPHONIC ACID ADENYLATE ESTER
HETNAM     LMT DODECYL-BETA-D-MALTOSIDE
HETSYN     ACP ADENOSINE-5'-[BETA, GAMMA-METHYLENE]TRIPHOSPHATE
FORMUL   3   MG    4(MG 2+)
FORMUL   5    K    2(K 1+)
FORMUL   6  ACP    2(C11 H18 N5 O12 P3)
FORMUL   7  LMT    2(C24 H46 O11)
HELIX    1 AA1 ILE A   19  LYS A   27  1
9
HELIX    2 AA2 THR A   35  GLY A   46  1
12
HELIX    3 AA3 SER A   56  LYS A   60  5
5
HELIX    4 AA4 ASN A   67  ALA A   84  1
18
HELIX    5 AA5 ARG A   89  ALA A  121  1
33
HELIX    6 AA6 ALA A  121  ALA A  129  1
9
HELIX    7 AA7 THR A  218  ALA A  224  1
7
HELIX    8 AA8 GLY A  234  MET A  255  1
22
HELIX    9 AA9 ILE A  257  GLN A  266  1
10
HELIX   10 AB1 TYR A  270  ASP A  272  5
3
HELIX   11 AB2 GLY A  273  ILE A  285  1
13
HELIX   12 AB3 ILE A  287  GLY A  308  1
22
```

```
HELIX   13 AB4 THR A  315  MET A  323  1
9
HELIX   14 AB5 ASP A  342  VAL A  346  5
5
HELIX   15 AB6 GLN A  357  ALA A  365  1
9
HELIX   16 AB7 ASP A  372  LEU A  382  1
11
HELIX   17 AB8 ASP A  384  ALA A  390  1
7
HELIX   18 AB9 ALA A  425  LEU A  430  1
6
HELIX   19 AC1 SER A  436  GLY A  454  1
19
HELIX   20 AC2 ASP A  492  LEU A  503  1
12
HELIX   21 AC3 GLN A  514  GLY A  526  1
13
HELIX   22 AC4 SER A  534  LEU A  538  1
5
HELIX   23 AC5 PRO A  550  ALA A  558  1
9
HELIX   24 AC6 PHE A  565  ARG A  579  1
15
HELIX   25 AC7 GLY A  589  ASN A  591  5
3
HELIX   26 AC8 ASP A  592  ALA A  599  1
8
HELIX   27 AC9 THR A  609  SER A  616  1
8
HELIX   28 AD1 GLY A  624  GLU A  668  1
45
HELIX   29 AD2 SER A  672  ILE A  687  1
16
HELIX   30 AD3 MET A  688  ASP A  693  5
6
HELIX   31 AD4 LYS A  705  ALA A  731  1
27
HELIX   32 AD5 ASP A  735  THR A  740  1
6
HELIX   33 AD6 ASN A  749  SER A  774  1
26
HELIX   34 AD7 GLY A  784  ALA A  805  1
22
HELIX   35 AD8 GLY A  816  ILE A  844  1
29
HELIX   36 AD9 ILE B   19  LEU B   26  1
8
HELIX   37 AE1 THR B   35  ILE B   42  1
8
HELIX   38 AE2 GLN B   43  GLY B   46  5
4
HELIX   39 AE3 LEU B   58  MET B   65  1
8
```

```
HELIX   40 AE4 ASN B   67  ALA B   84  1
18
HELIX   41 AE5 GLY B   88  ALA B  129  1
42
HELIX   42 AE6 THR B  214  HIS B  217  5
4
HELIX   43 AE7 THR B  218  ALA B  223  1
6
HELIX   44 AE8 GLY B  234  VAL B  256  1
23
HELIX   45 AE9 ILE B  257  GLN B  266  1
10
HELIX   46 AF1 GLY B  273  ILE B  285  1
13
HELIX   47 AF2 ALA B  288  GLY B  308  1
21
HELIX   48 AF3 THR B  315  MET B  323  1
9
HELIX   49 AF4 ASP B  342  VAL B  346  5
5
HELIX   50 AF5 GLN B  357  ALA B  365  1
9
HELIX   51 AF6 ASP B  372  LEU B  382  1
11
HELIX   52 AF7 ASP B  384  ARG B  389  1
6
HELIX   53 AF8 ALA B  425  GLU B  431  1
7
HELIX   54 AF9 ASN B  437  GLY B  454  1
18
HELIX   55 AG1 ARG B  490  LEU B  503  1
14
HELIX   56 AG2 GLN B  514  GLY B  526  1
13
HELIX   57 AG3 SER B  534  LEU B  538  1
5
HELIX   58 AG4 PRO B  550  ALA B  558  1
9
HELIX   59 AG5 PHE B  565  ARG B  579  1
15
HELIX   60 AG6 ASP B  592  ALA B  599  1
8
HELIX   61 AG7 THR B  609  SER B  616  1
8
HELIX   62 AG8 GLY B  624  GLU B  668  1
45
HELIX   63 AG9 SER B  672  ILE B  687  1
16
HELIX   64 AH1 LYS B  705  HIS B  732  1
28
HELIX   65 AH2 ASP B  735  GLY B  742  1
8
HELIX   66 AH3 ASN B  749  SER B  774  1
26
```

```
HELIX   67 AH4 TRP B  777  ARG B  782  1
6
HELIX   68 AH5 GLY B  784  ALA B  805  1
22
HELIX   69 AH6 TRP B  807  LYS B  811  5
5
HELIX   70 AH7 GLY B  816  TYR B  843  1
28
SHEET    1 AA1 2 LYS A 131  ARG A 136  0
SHEET    2 AA1 2 LYS A 139  GLU A 144 -1  O  SER A 141   N  VAL A
134
SHEET    1 AA2 4 ASP A 152  ILE A 156  0
SHEET    2 AA2 4 ILE A 207  VAL A 211 -1  O  ALA A 209   N  VAL A
154
SHEET    3 AA2 4 ALA A 166  GLU A 170 -1  N  ARG A 167   O  VAL A
210
SHEET    4 AA2 4 GLU A 195  VAL A 196 -1  O  VAL A 196   N  ALA A
166
SHEET    1 AA3 3 VAL A 188  LYS A 190  0
SHEET    2 AA3 3 LEU A 174  ASP A 177 -1  N  LEU A 174   O  LYS A
190
SHEET    3 AA3 3 THR A 201  CYS A 202 -1  O  THR A 201   N  ASP A
177
SHEET    1 AA4 8 ALA A 309  ILE A 310  0
SHEET    2 AA4 8 ILE A 618  LEU A 620 -1  O  VAL A 619   N  ILE A
310
SHEET    3 AA4 8 ILE A 601  ALA A 604  1  N  GLY A 602   O  ILE A
618
SHEET    4 AA4 8 VAL A 583  GLY A 587  1  N  MET A 585   O  ILE A
603
SHEET    5 AA4 8 VAL A 325  ASP A 329  1  N  CYS A 327   O  GLY A
584
SHEET    6 AA4 8 ASN A 506  ILE A 510  1  O  ASN A 506   N  LEU A
326
SHEET    7 AA4 8 GLY A 560  ALA A 562  1  O  PHE A 561   N  MET A
509
SHEET    8 AA4 8 TYR A 532  PRO A 533  1  N  TYR A 532   O  ALA A
562
SHEET    1 AA5 6 GLU A 347  VAL A 348  0
SHEET    2 AA5 6 GLU A 478  LEU A 485 -1  O  LEU A 482   N  GLU A
347
SHEET    3 AA5 6 ARG A 456  VAL A 464 -1  N  ARG A 456   O  LEU A
485
SHEET    4 AA5 6 ASN A 417  GLY A 424 -1  N  ARG A 420   O  GLN A
463
SHEET    5 AA5 6 ARG A 406  ASP A 413 -1  N  THR A 407   O  LYS A
423
SHEET    6 AA5 6 GLU A 394  LEU A 398 -1  N  VAL A 395   O  THR A
410
SHEET    1 AA6 2 LYS B 131  ARG B 136  0
SHEET    2 AA6 2 LYS B 139  GLU B 144 -1  O  GLN B 143   N  THR B
132
SHEET    1 AA7 4 ILE B 153  ILE B 156  0
SHEET    2 AA7 4 ILE B 207  ALA B 213 -1  O  ALA B 209   N  VAL B
```

```
154
SHEET    3 AA7 4 ASP B 165  LEU B 168 -1  N  ASP B 165   O  ILE B
212
SHEET    4 AA7 4 GLU B 195  VAL B 196 -1  O  VAL B 196   N  ALA B
166
SHEET    1 AA8 3 VAL B 188  LYS B 190  0
SHEET    2 AA8 3 LEU B 174  ASP B 177 -1  N  LEU B 174   O  LYS B
190
SHEET    3 AA8 3 THR B 201  LYS B 203 -1  O  LYS B 203   N  LYS B
175
SHEET    1 AA9 8 ALA B 309  THR B 311  0
SHEET    2 AA9 8 ILE B 618  LEU B 620 -1  O  VAL B 619   N  ILE B
310
SHEET    3 AA9 8 ILE B 601  ALA B 604  1  N  ALA B 604   O  ILE B
618
SHEET    4 AA9 8 VAL B 583  GLY B 587  1  N  MET B 585   O  ILE B
601
SHEET    5 AA9 8 VAL B 325  ASP B 329  1  N  CYS B 327   O  GLY B
584
SHEET    6 AA9 8 ASN B 506  ILE B 510  1  O  ASN B 506   N  LEU B
326
SHEET    7 AA9 8 GLY B 560  ALA B 562  1  O  PHE B 561   N  MET B
509
SHEET    8 AA9 8 TYR B 532  PRO B 533  1  N  TYR B 532   O  ALA B
562
SHEET    1 AB1 6 GLU B 347  VAL B 348  0
SHEET    2 AB1 6 GLU B 478  LEU B 485 -1  O  LEU B 482   N  GLU B
347
SHEET    3 AB1 6 ARG B 456  VAL B 464 -1  N  ARG B 462   O  GLU B
478
SHEET    4 AB1 6 ASN B 417  GLY B 424 -1  N  ARG B 420   O  GLN B
463
SHEET    5 AB1 6 ARG B 406  ASP B 413 -1  N  TYR B 411   O  HIS B
419
SHEET    6 AB1 6 GLU B 394  LEU B 398 -1  N  LEU B 398   O  ALA B
408
LINK         OD1 ASP A 372                MG    MG A 902     1555
1555  2.12
LINK         OD2 ASP A 372                MG    MG A 902     1555
1555  2.06
LINK         OD2 ASP A 588                MG    MG A 901     1555
1555  2.20
LINK         O   LEU A 596                 K     K A 903     1555
1555  3.14
LINK         O   LYS A 597                 K     K A 903     1555
1555  2.45
LINK         O   ALA A 599                 K     K A 903     1555
1555  2.69
LINK         OD1 ASP A 617                 K     K A 903     1555
1555  2.72
LINK         OD2 ASP A 617                 K     K A 903     1555
1555  2.84
LINK         OD2 ASP B 588                MG    MG B 901     1555
1555  2.22
```

```
LINK         O   LEU B 596                 K     K B 903     1555
1555  3.15
LINK         O   LYS B 597                 K     K B 903     1555
1555  2.70
LINK         O   ALA B 599                 K     K B 903     1555
1555  2.65
LINK         OD1 ASP B 617                 K     K B 903     1555
1555  2.77
LINK         OD2 ASP B 617                 K     K B 903     1555
1555  2.83
LINK        MG    MG A 901                 O1B ACP A 904     1555
1555  2.46
LINK        MG    MG A 901                 O1G ACP A 904     1555
1555  2.52
LINK        MG    MG B 901                 O2G ACP B 904     1555
1555  2.73
LINK        MG    MG B 901                 O2B ACP B 904     1555
1555  2.29
LINK        MG    MG B 902                 O2A ACP B 904     1555
1555  2.27
SITE     1 AC1  2 ASP A 588  ACP A 904
SITE     1 AC2  1 ASP A 372
SITE     1 AC3  4 LEU A 596  LYS A 597  ALA A 599  ASP A 617
SITE     1 AC4 12 THR A 331  ASP A 372  ILE A 374  ASP A 375
SITE     2 AC4 12 PHE A 400  THR A 407  ALA A 425  SER A 457
SITE     3 AC4 12 LEU A 458  THR A 511  GLY A 512   MG A 901
SITE     1 AC5  3 ARG A 775  SER A 776  ARG A 842
SITE     1 AC6  2 ASP B 588  ACP B 904
SITE     1 AC7  1 ACP B 904
SITE     1 AC8  4 LEU B 596  LYS B 597  ALA B 599  ASP B 617
SITE     1 AC9 14 LYS B 330  THR B 331  ASP B 372  ILE B 374
SITE     2 AC9 14 ASP B 375  PHE B 400  THR B 407  ALA B 425
SITE     3 AC9 14 ARG B 456  LEU B 458  THR B 511  GLY B 512
SITE     4 AC9 14  MG B 901   MG B 902
SITE     1 AD1  3 ARG B 775  SER B 776  ARG B 842
CRYST1   85.290  144.420  312.110  90.00  90.00  90.00 P 21 21 21
8
ORIGX1      1.000000  0.000000  0.000000        0.00000
ORIGX2      0.000000  1.000000  0.000000        0.00000
ORIGX3      0.000000  0.000000  1.000000        0.00000
SCALE1      0.011725  0.000000  0.000000        0.00000
SCALE2      0.000000  0.006924  0.000000        0.00000
SCALE3      0.000000  0.000000  0.003204        0.00000
ATOM      1  N   VAL A  12     -47.911 -24.871  34.202  1.00230.89
N
ANISOU    1  N   VAL A  12    17786  46528  23413  -6753  -2502
11439       N
ATOM      2  CA  VAL A  12     -46.463 -24.954  34.042  1.00224.66
C
ANISOU    2  CA  VAL A  12    17414  45217  22731  -6780  -2439
10838       C
ATOM      3  C   VAL A  12     -45.963 -26.348  34.405  1.00223.21
C
ANISOU    3  C   VAL A  12    17703  44789  22319  -7383  -2607
```

```
10419       C
ATOM      4  O   VAL A  12      -46.443 -26.966  35.356  1.00212.28
O
ANISOU    4  O   VAL A  12    16289  43483  20885  -7466  -2590
10438       O
ATOM      5  CB  VAL A  12      -45.744 -23.880  34.878  1.00189.08
C
ANISOU    5  CB  VAL A  12    12790  40363  18691  -5971  -1994
10568       C
ATOM      6  CG1 VAL A  12      -45.939 -22.505  34.255  1.00190.57
C
ANISOU    6  CG1 VAL A  12    12646  40664  19097  -5424  -1836
10885       C
ATOM      7  CG2 VAL A  12      -46.253 -23.893  36.305  1.00189.57
C
ANISOU    7  CG2 VAL A  12    12659  40457  18913  -5628  -1750
10599       C
ATOM      8  N   ASP A  13      -44.999 -26.840  33.623  1.00227.02
N
ANISOU    8  N   ASP A  13    18636  44959  22661  -7807  -2765
10043       N
ATOM      9  CA  ASP A  13      -44.447 -28.169  33.867  1.00234.45
C
ANISOU    9  CA  ASP A  13    20089  45600  23389  -8392  -2914
9628       C
ATOM     10  C   ASP A  13      -43.752 -28.239  35.223  1.00219.81
C
ANISOU   10  C   ASP A  13    18313  43383  21821  -8036  -2623
9227       C
ATOM     11  O   ASP A  13      -43.917 -29.215  35.965  1.00182.08
O
ANISOU   11  O   ASP A  13    13714  38552  16917  -8338  -2681
9113       O
ATOM     12  CB  ASP A  13      -43.482 -28.538  32.739  1.00253.27
C
ANISOU   12  CB  ASP A  13    22954  47666  25610  -8843  -3089
9287       C
ATOM     13  CG  ASP A  13      -43.155 -30.017  32.706  1.00278.67
C
ANISOU   13  CG  ASP A  13    26746  50621  28517  -9570  -3297
8961       C
ATOM     14  OD1 ASP A  13      -43.900 -30.806  33.323  1.00290.69
O
ANISOU   14  OD1 ASP A  13    28262  52321  29866  -9827  -3381
9110       O
ATOM     15  OD2 ASP A  13      -42.154 -30.389  32.058  1.00283.84
O
ANISOU   15  OD2 ASP A  13    27875  50872  29099  -9878  -3367
8555       O
ATOM     16  N   LEU A  14      -42.973 -27.213  35.563  1.00212.59
N
ANISOU   16  N   LEU A  14    17282  42214  21279  -7403  -2297
9016       N
ATOM     17  CA  LEU A  14      -42.494 -27.013  36.925  1.00206.07
```

```
C
ANISOU   17  CA  LEU A  14    16434  41121  20743  -6944  -1952
8737       C
ATOM     18  C   LEU A  14     -42.682 -25.548  37.300  1.00227.05
C
ANISOU   18  C   LEU A  14    18663  43855  23749  -6120  -1595
8922       C
ATOM     19  O   LEU A  14     -43.715 -25.167  37.862  1.00242.37
O
ANISOU   19  O   LEU A  14    20230  46107  25752  -5822  -1498
9291       O
ATOM     20  CB  LEU A  14     -41.025 -27.441  37.045  1.00171.51
C
ANISOU   20  CB  LEU A  14    12557  36173  16437  -7069  -1868
8135       C
ATOM     21  CG  LEU A  14     -40.356 -27.665  38.413  1.00165.62
C
ANISOU   21  CG  LEU A  14    12088  34970  15871  -6752  -1591
7720       C
ATOM     22  CD1 LEU A  14     -40.048 -26.377  39.169  1.00163.38
C
ANISOU   22  CD1 LEU A  14    11591  34536  15951  -5915  -1161
7646       C
ATOM     23  CD2 LEU A  14     -41.204 -28.588  39.277  1.00177.13
C
ANISOU   23  CD2 LEU A  14    13363  36759  17181  -7123  -1692
7928       C
ATOM     24  N   GLU A  15     -41.681 -24.729  36.978  1.00211.56
N
ANISOU   24  N   GLU A  15    16784  41588  22011  -5756  -1389
8662       N
ATOM     25  CA  GLU A  15     -41.630 -23.302  37.282  1.00219.56
C
ANISOU   25  CA  GLU A  15    17503  42553  23367  -4958  -1009
8746       C
ATOM     26  C   GLU A  15     -42.062 -22.958  38.708  1.00260.28
C
ANISOU   26  C   GLU A  15    22465  47702  28727  -4467   -675
8777       C
ATOM     27  O   GLU A  15     -41.651 -23.620  39.667  1.00269.51
O
ANISOU   27  O   GLU A  15    23851  48660  29890  -4580   -591
8468       O
ATOM     28  CB  GLU A  15     -42.479 -22.533  36.262  1.00214.83
C
ANISOU   28  CB  GLU A  15    16558  42326  22742  -4830  -1130
9252       C
ATOM     29  CG  GLU A  15     -42.024 -21.097  36.005  1.00213.77
C
ANISOU   29  CG  GLU A  15    16278  42023  22922  -4143   -814
9245       C
ATOM     30  CD  GLU A  15     -42.818 -20.423  34.904  1.00247.92
C
ANISOU   30  CD  GLU A  15    20292  46707  27199  -4083   -968
```

```
9759       C
ATOM     31  OE1 GLU A  15      -43.534 -19.443  35.199  1.00262.86
O
ANISOU   31  OE1 GLU A  15    21835  48742  29299  -3521   -732
10101       O
ATOM     32  OE2 GLU A  15      -42.726 -20.872  33.742  1.00257.88
O
ANISOU   32  OE2 GLU A  15    21681  48092  28209  -4608  -1317
9822       O
ATOM     33  N   LYS A  16      -42.886 -21.914  38.848  1.00267.02
N
ANISOU   33  N   LYS A  16    22928  48768  29759  -3927   -477
9150       N
ATOM     34  CA  LYS A  16      -43.343 -21.460  40.160  1.00242.64
C
ANISOU   34  CA  LYS A  16    19662  45654  26876  -3428   -128
9187       C
ATOM     35  C   LYS A  16      -44.126 -22.529  40.920  1.00240.09
C
ANISOU   35  C   LYS A  16    19298  45570  26357  -3821   -296
9305       C
ATOM     36  O   LYS A  16      -43.938 -22.693  42.132  1.00242.02
O
ANISOU   36  O   LYS A  16    19630  45631  26697  -3657    -67
9068       O
ATOM     37  CB  LYS A  16      -44.186 -20.196  39.990  1.00216.78
C
ANISOU   37  CB  LYS A  16    15988  42574  23805  -2846     79
9619       C
ATOM     38  CG  LYS A  16      -44.787 -19.644  41.270  1.00199.12
C
ANISOU   38  CG  LYS A  16    13559  40317  21780  -2328    452
9698       C
ATOM     39  CD  LYS A  16      -45.491 -18.324  40.998  1.00198.46
C
ANISOU   39  CD  LYS A  16    13137  40347  21922  -1737    686
10096       C
ATOM     40  CE  LYS A  16      -46.104 -17.746  42.260  1.00213.55
C
ANISOU   40  CE  LYS A  16    14884  42206  24049  -1229   1081
10165       C
ATOM     41  NZ  LYS A  16      -46.825 -16.471  41.987  1.00219.23
N
ANISOU   41  NZ  LYS A  16    15291  43011  24994   -663   1323
10570       N
ATOM     42  N   ILE A  17      -45.010 -23.253  40.241  1.00239.71
N
ANISOU   42  N   ILE A  17    19131  45930  26019  -4347   -687
9671       N
ATOM     43  CA  ILE A  17      -45.804 -24.277  40.938  1.00229.18
C
ANISOU   43  CA  ILE A  17    17761  44838  24478  -4732   -854
9804       C
ATOM     44  C   ILE A  17      -44.891 -25.372  41.481  1.00204.45
```

```
C
ANISOU   44  C   ILE A  17     15069  41373  21238  -5143   -923
9310       C
ATOM     45  O   ILE A  17      -44.011 -25.869  40.750  1.00199.56
O
ANISOU   45  O   ILE A  17     14793  40527  20502  -5525  -1105
9029       O
ATOM     46  CB  ILE A  17      -46.876 -24.864  40.004  1.00243.25
C
ANISOU   46  CB  ILE A  17     19383  47112  25928  -5261  -1270
10286       C
ATOM     47  CG1 ILE A  17      -47.788 -23.750  39.484  1.00251.01
C
ANISOU   47  CG1 ILE A  17     19914  48431  27028  -4837  -1187
10817       C
ATOM     48  CG2 ILE A  17      -47.694 -25.924  40.727  1.00246.28
C
ANISOU   48  CG2 ILE A  17     19746  47745  26086  -5661  -1436
10422       C
ATOM     49  CD1 ILE A  17      -48.520 -23.000  40.577  1.00250.18
C
ANISOU   49  CD1 ILE A  17     19461  48416  27179  -4228   -831
11024       C
ATOM     50  N   PRO A  18      -45.054 -25.787  42.744  1.00199.83
N
ANISOU   50  N   PRO A  18     14503  40737  20687  -5092   -783
9194       N
ATOM     51  CA  PRO A  18      -44.139 -26.775  43.330  1.00213.60
C
ANISOU   51  CA  PRO A  18     16669  42134  22354  -5440   -817
8732       C
ATOM     52  C   PRO A  18      -44.582 -28.212  43.087  1.00204.23
C
ANISOU   52  C   PRO A  18     15668  41122  20808  -6206  -1228
8812       C
ATOM     53  O   PRO A  18      -45.289 -28.796  43.914  1.00196.88
O
ANISOU   53  O   PRO A  18     14654  40371  19779  -6344  -1266
8938       O
ATOM     54  CB  PRO A  18      -44.169 -26.431  44.826  1.00208.77
C
ANISOU   54  CB  PRO A  18     15973  41401  21951  -4985   -449
8604       C
ATOM     55  CG  PRO A  18      -45.527 -25.844  45.032  1.00194.30
C
ANISOU   55  CG  PRO A  18     13675  39988  20164  -4688   -380
9093       C
ATOM     56  CD  PRO A  18      -45.817 -25.064  43.776  1.00211.34
C
ANISOU   56  CD  PRO A  18     15623  42330  22347  -4547   -466
9398       C
ATOM     57  N   ILE A  19      -44.169 -28.794  41.958  1.00176.66
N
ANISOU   57  N   ILE A  19     12457  37558  17107  -6717  -1528
```

```
8728       N
ATOM     58  CA  ILE A  19     -44.338 -30.229  41.757  1.00177.98
C
ANISOU   58  CA  ILE A  19    12945  37748  16932  -7468  -1878
8686       C
ATOM     59  C   ILE A  19     -43.358 -31.014  42.621  1.00193.39
C
ANISOU   59  C   ILE A  19    15304  39250  18925  -7626  -1789
8211       C
ATOM     60  O   ILE A  19     -43.626 -32.170  42.973  1.00175.20
O
ANISOU   60  O   ILE A  19    13218  36952  16399  -8113  -1982
8184       O
ATOM     61  CB  ILE A  19     -44.180 -30.578  40.265  1.00178.98
C
ANISOU   61  CB  ILE A  19    13300  37893  16812  -7968  -2198
8721       C
ATOM     62  CG1 ILE A  19     -45.052 -29.655  39.411  1.00190.66
C
ANISOU   62  CG1 ILE A  19    14358  39803  18280  -7748  -2253
9202       C
ATOM     63  CG2 ILE A  19     -44.569 -32.024  40.007  1.00181.47
C
ANISOU   63  CG2 ILE A  19    13947  38269  16733  -8747  -2548
8741       C
ATOM     64  CD1 ILE A  19     -46.523 -29.684  39.779  1.00210.45
C
ANISOU   64  CD1 ILE A  19    16462  42833  20668  -7734  -2325
9720       C
ATOM     65  N   GLU A  20     -42.247 -30.392  42.994  1.00208.91
N
ANISOU   65  N   GLU A  20    17378  40830  21166  -7213  -1488
7857       N
ATOM     66  CA  GLU A  20     -41.391 -30.775  44.130  1.00165.69
C
ANISOU   66  CA  GLU A  20    12166  34975  15816  -7137  -1281
7470       C
ATOM     67  C   GLU A  20     -40.916 -32.227  43.983  1.00194.11
C
ANISOU   67  C   GLU A  20    16262  38311  19181  -7833  -1548
7229       C
ATOM     68  O   GLU A  20     -40.542 -32.645  42.877  1.00188.70
O
ANISOU   68  O   GLU A  20    15912  37445  18342  -8198  -1776
7116       O
ATOM     69  CB  GLU A  20     -42.171 -30.479  45.402  1.00167.36
C
ANISOU   69  CB  GLU A  20    12057  35404  16128  -6773  -1075
7646       C
ATOM     70  CG  GLU A  20     -41.397 -29.734  46.479  1.00179.82
C
ANISOU   70  CG  GLU A  20    13644  36686  17993  -6204   -651
7360       C
ATOM     71  CD  GLU A  20     -40.331 -30.597  47.130  1.00203.61
```

```
C
ANISOU   71  CD  GLU A  20    17140  39238  20985  -6437   -636
6926       C
ATOM     72  OE1 GLU A  20     -40.699 -31.557  47.839  1.00210.62
O
ANISOU   72  OE1 GLU A  20    18080  40214  21732  -6795   -750
6965       O
ATOM     73  OE2 GLU A  20     -39.130 -30.321  46.934  1.00198.55
O
ANISOU   73  OE2 GLU A  20    16946  38030  20466  -6182   -541
6504       O
ATOM     74  N   GLU A  21     -40.904 -32.991  45.080  1.00209.70
N
ANISOU   74  N   GLU A  21    18376  40174  21124  -7967  -1515
7114       N
ATOM     75  CA  GLU A  21     -40.417 -34.371  45.103  1.00193.82
C
ANISOU   75  CA  GLU A  21    16901  37818  18924  -8542  -1725
6858       C
ATOM     76  C   GLU A  21     -41.035 -35.278  44.041  1.00200.49
C
ANISOU   76  C   GLU A  21    17901  38849  19429  -9221  -2112
7041       C
ATOM     77  O   GLU A  21     -40.356 -36.172  43.521  1.00214.16
O
ANISOU   77  O   GLU A  21    20137  40209  21027  -9690  -2266
6779       O
ATOM     78  CB  GLU A  21     -40.662 -34.950  46.502  1.00183.77
C
ANISOU   78  CB  GLU A  21    15594  36573  17656  -8573  -1639
6851       C
ATOM     79  CG  GLU A  21     -40.499 -36.452  46.629  1.00164.58
C
ANISOU   79  CG  GLU A  21    13625  33906  15003  -9207  -1875
6708       C
ATOM     80  CD  GLU A  21     -40.410 -36.901  48.074  1.00163.67
C
ANISOU   80  CD  GLU A  21    13551  33684  14951  -9131  -1739
6611       C
ATOM     81  OE1 GLU A  21     -41.378 -36.669  48.829  1.00166.06
O
ANISOU   81  OE1 GLU A  21    13490  34362  15244  -8940  -1688
6869       O
ATOM     82  OE2 GLU A  21     -39.371 -37.480  48.456  1.00160.71
O
ANISOU   82  OE2 GLU A  21    13677  32749  14636  -9158  -1695
6236       O
ATOM     83  N   VAL A  22     -42.304 -35.063  43.692  1.00183.38
N
ANISOU   83  N   VAL A  22    15373  37198  17105  -9254  -2263
7473       N
ATOM     84  CA  VAL A  22     -42.911 -35.790  42.576  1.00194.33
C
ANISOU   84  CA  VAL A  22    16934  38767  18135  -9854  -2619
```

```
7662        C
ATOM     85  C   VAL A  22      -42.179 -35.533  41.260  1.00199.40
C
ANISOU   85  C   VAL A  22    17818  39190  18754  -9991  -2698
7499        C
ATOM     86  O   VAL A  22      -41.970 -36.456  40.464  1.00205.06
O
ANISOU   86  O   VAL A  22    18993  39714  19207 -10577  -2931
7371        O
ATOM     87  CB  VAL A  22      -44.406 -35.434  42.466  1.00207.00
C
ANISOU   87  CB  VAL A  22    18046  41005  19597  -9792  -2730
8203        C
ATOM     88  CG1 VAL A  22      -45.047 -36.163  41.289  1.00206.50
C
ANISOU   88  CG1 VAL A  22    18174  41159  19128 -10432  -3087
8423        C
ATOM     89  CG2 VAL A  22      -45.125 -35.768  43.765  1.00220.30
C
ANISOU   89  CG2 VAL A  22    19526  42891  21287  -9701  -2659
8345        C
ATOM     90  N   PHE A  23      -41.782 -34.283  41.011  1.00200.86
N
ANISOU   90  N   PHE A  23    17727  39384  19205  -9456  -2493
7493        N
ATOM     91  CA  PHE A  23      -41.247 -33.905  39.702  1.00203.38
C
ANISOU   91  CA  PHE A  23    18197  39585  19494  -9556  -2580
7407        C
ATOM     92  C   PHE A  23      -40.002 -34.701  39.307  1.00227.14
C
ANISOU   92  C   PHE A  23    21907  41945  22451  -9882  -2625
6898        C
ATOM     93  O   PHE A  23      -39.914 -35.208  38.183  1.00254.98
O
ANISOU   93  O   PHE A  23    25746  45402  25731 -10389  -2856
6858        O
ATOM     94  CB  PHE A  23      -40.943 -32.405  39.690  1.00181.10
C
ANISOU   94  CB  PHE A  23    14981  36825  17005  -8849  -2301
7452        C
ATOM     95  CG  PHE A  23      -40.524 -31.880  38.346  1.00196.06
C
ANISOU   95  CG  PHE A  23    17005  38620  18869  -8854  -2390
7405        C
ATOM     96  CD1 PHE A  23      -39.185 -31.675  38.053  1.00202.20
C
ANISOU   96  CD1 PHE A  23    18328  38714  19785  -8574  -2254
6891        C
ATOM     97  CD2 PHE A  23      -41.468 -31.589  37.376  1.00191.48
C
ANISOU   97  CD2 PHE A  23    16130  38524  18100  -9036  -2606
7840        C
ATOM     98  CE1 PHE A  23      -38.797 -31.190  36.819  1.00197.81
```

```
C
ANISOU   98  CE1 PHE A  23    17912  38055  19192  -8572  -2332
6838       C
ATOM     99  CE2 PHE A  23     -41.086 -31.103  36.140  1.00172.55
C
ANISOU   99  CE2 PHE A  23    13804  36089  15667  -9095  -2697
7824       C
ATOM    100  CZ  PHE A  23     -39.749 -30.904  35.861  1.00168.04
C
ANISOU  100  CZ  PHE A  23    13736  34872  15240  -8860  -2556
7310       C
ATOM    101  N   GLN A  24     -39.027 -34.822  40.212  1.00225.03
N
ANISOU  101  N   GLN A  24    21988  41114  22397  -9521  -2394
6479       N
ATOM    102  CA  GLN A  24     -37.840 -35.625  39.912  1.00225.63
C
ANISOU  102  CA  GLN A  24    22814  40476  22439  -9714  -2409
5981       C
ATOM    103  C   GLN A  24     -38.161 -37.105  39.707  1.00231.60
C
ANISOU  103  C   GLN A  24    23923  41205  22869 -10516  -2670
5990       C
ATOM    104  O   GLN A  24     -37.626 -37.739  38.791  1.00238.45
O
ANISOU  104  O   GLN A  24    25320  41710  23572 -10909  -2791
5757       O
ATOM    105  CB  GLN A  24     -36.790 -35.454  41.013  1.00215.05
C
ANISOU  105  CB  GLN A  24    21712  38599  21397  -9168  -2116
5593       C
ATOM    106  CG  GLN A  24     -37.257 -35.784  42.420  1.00208.60
C
ANISOU  106  CG  GLN A  24    20662  37963  20633  -9114  -2030
5711       C
ATOM    107  CD  GLN A  24     -36.132 -35.692  43.432  1.00222.80
C
ANISOU  107  CD  GLN A  24    22756  39207  22692  -8646  -1769
5314       C
ATOM    108  OE1 GLN A  24     -36.117 -34.804  44.283  1.00246.07
O
ANISOU  108  OE1 GLN A  24    25388  42250  25856  -8103  -1527
5340       O
ATOM    109  NE2 GLN A  24     -35.177 -36.613  43.340  1.00208.35
N
ANISOU  109  NE2 GLN A  24    21539  36791  20835  -8864  -1806
4955       N
ATOM    110  N   GLN A  25     -39.029 -37.671  40.546  1.00216.59
N
ANISOU  110  N   GLN A  25    21757  39672  20867 -10777  -2744
6251       N
ATOM    111  CA  GLN A  25     -39.452 -39.060  40.373  1.00193.56
C
ANISOU  111  CA  GLN A  25    19150  36775  17620 -11564  -2995
```

```
6299       C
ATOM    112  C   GLN A  25     -40.220 -39.289  39.073  1.00189.05
C
ANISOU  112  C   GLN A  25    18660  36486  16686 -12010  -3272
6533       C
ATOM    113  O   GLN A  25     -40.003 -40.296  38.389  1.00190.58
O
ANISOU  113  O   GLN A  25    19418  36381  16613 -12568  -3426
6360       O
ATOM    114  CB  GLN A  25     -40.285 -39.500  41.576  1.00186.88
C
ANISOU  114  CB  GLN A  25    18075  36197  16732 -11540  -2996
6512       C
ATOM    115  CG  GLN A  25     -39.471 -39.622  42.856  1.00180.68
C
ANISOU  115  CG  GLN A  25    17378  35048  16223 -11250  -2754
6241       C
ATOM    116  CD  GLN A  25     -40.324 -39.920  44.071  1.00169.68
C
ANISOU  116  CD  GLN A  25    15705  33964  14801 -11187  -2741
6466       C
ATOM    117  OE1 GLN A  25     -40.345 -41.045  44.571  1.00170.61
O
ANISOU  117  OE1 GLN A  25    16154  33888  14782 -11550  -2829
6381       O
ATOM    118  NE2 GLN A  25     -41.036 -38.909  44.553  1.00170.35
N
ANISOU  118  NE2 GLN A  25    15199  34510  15017 -10707  -2619
6750       N
ATOM    119  N   LEU A  26     -41.123 -38.373  38.718  1.00194.79
N
ANISOU  119  N   LEU A  26    18850  37773  17390 -11770  -3319
6935       N
ATOM    120  CA  LEU A  26     -41.843 -38.482  37.450  1.00195.75
C
ANISOU  120  CA  LEU A  26    19015  38195  17165 -12174  -3569
7200       C
ATOM    121  C   LEU A  26     -40.917 -38.422  36.237  1.00179.20
C
ANISOU  121  C   LEU A  26    17343  35720  15025 -12373  -3604
6903       C
ATOM    122  O   LEU A  26     -41.058 -39.222  35.305  1.00181.72
O
ANISOU  122  O   LEU A  26    18108  35949  14989 -12956  -3796
6875       O
ATOM    123  CB  LEU A  26     -42.910 -37.389  37.362  1.00195.31
C
ANISOU  123  CB  LEU A  26    18268  38789  17154 -11795  -3574
7707       C
ATOM    124  CG  LEU A  26     -43.744 -37.346  36.079  1.00188.15
C
ANISOU  124  CG  LEU A  26    17313  38276  15901 -12163  -3817
8068       C
ATOM    125  CD1 LEU A  26     -44.454 -38.673  35.856  1.00192.13
```

```
C
ANISOU  125  CD1 LEU A  26    18175  38873  15953 -12875  -4055
8185       C
ATOM    126  CD2 LEU A  26     -44.750 -36.211  36.141  1.00192.21
C
ANISOU  126  CD2 LEU A  26    17113  39395  16521 -11703  -3780
8585       C
ATOM    127  N   LYS A  27     -39.964 -37.492  36.224  1.00185.97
N
ANISOU  127  N   LYS A  27    18093  36346  16223 -11907  -3408
6675       N
ATOM    128  CA  LYS A  27     -39.115 -37.314  35.049  1.00185.08
C
ANISOU  128  CA  LYS A  27    18335  35908  16077 -12057  -3440
6412       C
ATOM    129  C   LYS A  27     -37.753 -37.992  35.154  1.00184.02
C
ANISOU  129  C   LYS A  27    18961  34921  16038 -12028  -3287
5817       C
ATOM    130  O   LYS A  27     -37.480 -38.955  34.430  1.00188.94
O
ANISOU  130  O   LYS A  27    20145  35246  16398 -12624  -3417
5622       O
ATOM    131  CB  LYS A  27     -38.928 -35.821  34.760  1.00171.21
C
ANISOU  131  CB  LYS A  27    16162  34298  14590 -11390  -3291
6506       C
ATOM    132  CG  LYS A  27     -40.156 -35.161  34.146  1.00184.88
C
ANISOU  132  CG  LYS A  27    17329  36745  16171 -11424  -3457
7068       C
ATOM    133  CD  LYS A  27     -41.101 -34.617  35.207  1.00176.72
C
ANISOU  133  CD  LYS A  27    15674  36175  15297 -10969  -3345
7451       C
ATOM    134  CE  LYS A  27     -42.354 -34.024  34.582  1.00181.45
C
ANISOU  134  CE  LYS A  27    15810  37399  15734 -10935  -3487
8018       C
ATOM    135  NZ  LYS A  27     -42.033 -32.990  33.565  1.00217.66
N
ANISOU  135  NZ  LYS A  27    20248  42038  20416 -10702  -3472
8076       N
ATOM    136  N   CYS A  28     -36.888 -37.479  36.032  1.00186.05
N
ANISOU  136  N   CYS A  28    19257  34769  16664 -11324  -2995
5534       N
ATOM    137  CA  CYS A  28     -35.527 -38.000  36.150  1.00187.76
C
ANISOU  137  CA  CYS A  28    20154  34171  17013 -11188  -2824
4994       C
ATOM    138  C   CYS A  28     -35.506 -39.492  36.470  1.00191.40
C
ANISOU  138  C   CYS A  28    21087  34355  17280 -11771  -2914
```

```
4861       C
ATOM    139  O   CYS A  28      -34.701 -40.240  35.901  1.00202.23
O
ANISOU  139  O   CYS A  28     23108  35162  18568 -12041  -2897
4514       O
ATOM    140  CB  CYS A  28      -34.758 -37.217  37.215  1.00172.68
C
ANISOU  140  CB  CYS A  28     18120  31980  15512 -10375  -2519
4790       C
ATOM    141  SG  CYS A  28      -33.053 -37.773  37.472  1.00152.46
S
ANISOU  141  SG  CYS A  28     16313  28460  13155 -10137  -2297
4179       S
ATOM    142  N   SER A  29      -36.361 -39.938  37.391  1.00187.18
N
ANISOU  142  N   SER A  29     20249  34189  16681 -11956  -2988
5128       N
ATOM    143  CA  SER A  29      -36.666 -41.347  37.642  1.00192.86
C
ANISOU  143  CA  SER A  29     21316  34808  17154 -12614  -3126
5121       C
ATOM    144  C   SER A  29      -35.516 -42.062  38.339  1.00206.08
C
ANISOU  144  C   SER A  29     23554  35727  19019 -12433  -2927
4687       C
ATOM    145  O   SER A  29      -35.543 -43.292  38.476  1.00221.36
O
ANISOU  145  O   SER A  29     25905  37424  20777 -12951  -3001
4607       O
ATOM    146  CB  SER A  29      -37.042 -42.099  36.357  1.00194.59
C
ANISOU  146  CB  SER A  29     21881  35090  16963 -13422  -3374
5170       C
ATOM    147  OG  SER A  29      -38.096 -41.449  35.667  1.00202.72
O
ANISOU  147  OG  SER A  29     22453  36775  17796 -13487  -3552
5582       O
ATOM    148  N   ARG A  30      -34.523 -41.309  38.807  1.00202.09
N
ANISOU  148  N   ARG A  30     23059  34855  18871 -11711  -2673
4428       N
ATOM    149  CA  ARG A  30      -33.291 -41.797  39.415  1.00187.97
C
ANISOU  149  CA  ARG A  30     21761  32348  17309 -11437  -2465
4027       C
ATOM    150  C   ARG A  30      -32.766 -40.658  40.277  1.00175.04
C
ANISOU  150  C   ARG A  30     19781  30689  16036 -10626  -2233
3960       C
ATOM    151  O   ARG A  30      -33.242 -39.524  40.187  1.00184.67
O
ANISOU  151  O   ARG A  30     20490  32351  17325 -10296  -2215
4167       O
ATOM    152  CB  ARG A  30      -32.245 -42.244  38.379  1.00186.47
```

```
C
ANISOU  152  CB  ARG A  30    22246  31515  17088 -11583  -2408
3650       C
ATOM    153  CG  ARG A  30     -32.668 -43.384  37.451  1.00180.10
C
ANISOU  153  CG  ARG A  30    21875  30654  15901 -12414  -2602
3664       C
ATOM    154  CD  ARG A  30     -31.533 -43.838  36.543  1.00173.18
C
ANISOU  154  CD  ARG A  30    21704  29069  15028 -12500  -2481
3254       C
ATOM    155  NE  ARG A  30     -31.936 -44.951  35.685  1.00176.52
N
ANISOU  155  NE  ARG A  30    22592  29410  15069 -13321  -2636
3248       N
ATOM    156  CZ  ARG A  30     -32.586 -44.819  34.533  1.00187.91
C
ANISOU  156  CZ  ARG A  30    24004  31203  16191 -13800  -2829
3398       C
ATOM    157  NH1 ARG A  30     -32.879 -45.891  33.810  1.00170.68
N
ANISOU  157  NH1 ARG A  30    22310  28894  13647 -14576  -2948
3360       N
ATOM    158  NH2 ARG A  30     -32.920 -43.615  34.088  1.00191.97
N
ANISOU  158  NH2 ARG A  30    24020  32180  16739 -13514  -2895
3586       N
ATOM    159  N   GLU A  31     -31.780 -40.962  41.117  1.00158.35
N
ANISOU  159  N   GLU A  31    17956  28057  14152 -10314  -2049
3682       N
ATOM    160  CA  GLU A  31     -31.283 -39.963  42.055  1.00146.21
C
ANISOU  160  CA  GLU A  31    16123  26499  12930  -9603  -1829
3616       C
ATOM    161  C   GLU A  31     -29.765 -39.990  42.127  1.00142.50
C
ANISOU  161  C   GLU A  31    16117  25321  12704  -9230  -1628
3205       C
ATOM    162  O   GLU A  31     -29.165 -41.053  42.312  1.00142.55
O
ANISOU  162  O   GLU A  31    16594  24857  12713  -9447  -1612
3022       O
ATOM    163  CB  GLU A  31     -31.878 -40.193  43.451  1.00146.79
C
ANISOU  163  CB  GLU A  31    15878  26857  13040  -9569  -1813
3814       C
ATOM    164  CG  GLU A  31     -33.389 -40.013  43.521  1.00165.41
C
ANISOU  164  CG  GLU A  31    17683  29969  15198  -9843  -1976
4254       C
ATOM    165  CD  GLU A  31     -33.953 -40.315  44.896  1.00188.93
C
ANISOU  165  CD  GLU A  31    20382  33206  18199  -9840  -1949
```

```
4434       C
ATOM    166  OE1 GLU A  31     -33.898 -41.489  45.318  1.00183.07
O
ANISOU  166  OE1 GLU A  31    19954  32246  17359 -10225  -2026
4386       O
ATOM    167  OE2 GLU A  31     -34.455 -39.380  45.554  1.00206.82
O
ANISOU  167  OE2 GLU A  31    22122  35881  20578  -9454  -1837
4625       O
ATOM    168  N   GLY A  32     -29.153 -38.816  41.983  1.00156.42
N
ANISOU  168  N   GLY A  32    17740  27016  14676  -8669  -1469
3080       N
ATOM    169  CA  GLY A  32     -27.765 -38.625  42.352  1.00178.14
C
ANISOU  169  CA  GLY A  32    20788  29201  17696  -8212  -1256
2744       C
ATOM    170  C   GLY A  32     -26.768 -39.033  41.288  1.00205.20
C
ANISOU  170  C   GLY A  32    24769  32070  21128  -8311  -1226
2442       C
ATOM    171  O   GLY A  32     -26.210 -38.185  40.585  1.00208.08
O
ANISOU  171  O   GLY A  32    25154  32321  21587  -8004  -1141
2299       O
ATOM    172  N   LEU A  33     -26.542 -40.338  41.164  1.00219.48
N
ANISOU  172  N   LEU A  33    27041  33517  22835  -8740  -1280
2343       N
ATOM    173  CA  LEU A  33     -25.360 -40.883  40.511  1.00209.38
C
ANISOU  173  CA  LEU A  33    26348  31574  21633  -8750  -1171
2016       C
ATOM    174  C   LEU A  33     -25.567 -41.155  39.023  1.00201.20
C
ANISOU  174  C   LEU A  33    25603  30486  20357  -9187  -1275
1961       C
ATOM    175  O   LEU A  33     -24.736 -41.839  38.417  1.00202.62
O
ANISOU  175  O   LEU A  33    26328  30111  20546  -9322  -1188
1705       O
ATOM    176  CB  LEU A  33     -24.899 -42.150  41.244  1.00192.39
C
ANISOU  176  CB  LEU A  33    24572  28991  19537  -8928  -1123
1932       C
ATOM    177  CG  LEU A  33     -25.908 -43.210  41.712  1.00160.66
C
ANISOU  177  CG  LEU A  33    20547  25200  15298  -9478  -1289
2158       C
ATOM    178  CD1 LEU A  33     -26.732 -43.811  40.576  1.00145.92
C
ANISOU  178  CD1 LEU A  33    18872  23486  13086 -10139  -1473
2243       C
ATOM    179  CD2 LEU A  33     -25.199 -44.304  42.507  1.00139.68
```

```
C
ANISOU  179  CD2 LEU A  33     18267  22033  12772  -9510  -1191
2047       C
ATOM    180  N   THR A  34      -26.667 -40.655  38.445  1.00191.78
N
ANISOU  180  N   THR A  34     24057  29866  18946  -9420  -1452
2213       N
ATOM    181  CA  THR A  34      -27.079 -40.838  37.051  1.00198.65
C
ANISOU  181  CA  THR A  34     25119  30825  19532  -9903  -1596
2232       C
ATOM    182  C   THR A  34      -25.927 -41.121  36.090  1.00198.86
C
ANISOU  182  C   THR A  34     25740  30219  19599  -9905  -1460
1868       C
ATOM    183  O   THR A  34      -25.026 -40.292  35.925  1.00176.35
O
ANISOU  183  O   THR A  34     22890  27140  16974  -9391  -1299
1676       O
ATOM    184  CB  THR A  34      -27.851 -39.607  36.569  1.00197.66
C
ANISOU  184  CB  THR A  34     24453  31305  19344  -9758  -1700
2480       C
ATOM    185  OG1 THR A  34      -28.662 -39.101  37.636  1.00176.58
O
ANISOU  185  OG1 THR A  34     21197  29148  16748  -9535  -1730
2776       O
ATOM    186  CG2 THR A  34      -28.744 -39.970  35.391  1.00198.46
C
ANISOU  186  CG2 THR A  34     24612  31722  19070 -10420  -1936
2656       C
ATOM    187  N   THR A  35      -25.952 -42.294  35.463  1.00213.70
N
ANISOU  187  N   THR A  35     28137  31807  21253 -10491  -1510
1770       N
ATOM    188  CA  THR A  35      -24.829 -42.765  34.663  1.00204.43
C
ANISOU  188  CA  THR A  35     27591  29959  20124 -10520  -1337
1411       C
ATOM    189  C   THR A  35      -24.604 -41.909  33.416  1.00200.01
C
ANISOU  189  C   THR A  35     27046  29453  19495 -10450  -1338
1315       C
ATOM    190  O   THR A  35      -25.526 -41.305  32.861  1.00197.19
O
ANISOU  190  O   THR A  35     26338  29652  18932 -10646  -1531
1551       O
ATOM    191  CB  THR A  35      -25.036 -44.231  34.270  1.00185.21
C
ANISOU  191  CB  THR A  35     25719  27221  17431 -11215  -1375
1347       C
ATOM    192  OG1 THR A  35      -23.782 -44.807  33.881  1.00206.81
O
ANISOU  192  OG1 THR A  35     29075  29196  20309 -11116  -1127
```

```
987       O
ATOM    193  CG2 THR A  35      -26.035 -44.361  33.126  1.00160.65
C
ANISOU  193  CG2 THR A  35    22644  24498  13898 -11884  -1602
1495       C
ATOM    194  N   GLN A  36      -23.333 -41.848  33.010  1.00196.59
N
ANISOU  194  N   GLN A  36    27007  28435  19254 -10145  -1113
979       N
ATOM    195  CA  GLN A  36      -22.889 -41.022  31.887  1.00189.43
C
ANISOU  195  CA  GLN A  36    26160  27484  18330 -10001  -1070
837       C
ATOM    196  C   GLN A  36      -23.611 -41.367  30.585  1.00187.71
C
ANISOU  196  C   GLN A  36    26159  27458  17704 -10688  -1244
897       C
ATOM    197  O   GLN A  36      -23.935 -40.476  29.792  1.00189.78
O
ANISOU  197  O   GLN A  36    26180  28070  17857 -10678  -1350
991       O
ATOM    198  CB  GLN A  36      -21.376 -41.165  31.716  1.00168.75
C
ANISOU  198  CB  GLN A  36    24003  24145  15970  -9636   -782
460       C
ATOM    199  CG  GLN A  36      -20.579 -40.683  32.921  1.00171.62
C
ANISOU  199  CG  GLN A  36    24131  24350  16728  -8946   -618
409       C
ATOM    200  CD  GLN A  36      -19.083 -40.858  32.746  1.00193.75
C
ANISOU  200  CD  GLN A  36    27364  26465  19785  -8602   -341
73       C
ATOM    201  OE1 GLN A  36      -18.435 -40.087  32.037  1.00201.07
O
ANISOU  201  OE1 GLN A  36    28330  27278  20789  -8330   -247
-88       O
ATOM    202  NE2 GLN A  36      -18.525 -41.873  33.396  1.00193.49
N
ANISOU  202  NE2 GLN A  36    27647  25979  19891  -8607   -205
-17       N
ATOM    203  N   GLU A  37      -23.861 -42.657  30.347  1.00195.26
N
ANISOU  203  N   GLU A  37    27583  28184  18424 -11306  -1274
848       N
ATOM    204  CA  GLU A  37      -24.615 -43.094  29.172  1.00212.90
C
ANISOU  204  CA  GLU A  37    30053  30619  20220 -12054  -1453
917       C
ATOM    205  C   GLU A  37      -26.012 -42.480  29.111  1.00201.51
C
ANISOU  205  C   GLU A  37    27994  30025  18546 -12298  -1770
1345       C
ATOM    206  O   GLU A  37      -26.496 -42.137  28.026  1.00211.02
```

```
O
ANISOU  206  O   GLU A  37    29166  31540  19473 -12653  -1925
1443       O
ATOM    207  CB  GLU A  37     -24.709 -44.620  29.156  1.00239.85
C
ANISOU  207  CB  GLU A  37    34055  33649  23429 -12670  -1418
814       C
ATOM    208  CG  GLU A  37     -23.376 -45.326  28.964  1.00264.08
C
ANISOU  208  CG  GLU A  37    37805  35851  26680 -12524  -1087
403       C
ATOM    209  CD  GLU A  37     -23.528 -46.827  28.813  1.00280.94
C
ANISOU  209  CD  GLU A  37    40568  37595  28583 -13180  -1034
306       C
ATOM    210  OE1 GLU A  37     -24.136 -47.267  27.814  1.00294.48
O
ANISOU  210  OE1 GLU A  37    42579  39424  29885 -13885  -1156
318       O
ATOM    211  OE2 GLU A  37     -23.039 -47.567  29.692  1.00286.77
O
ANISOU  211  OE2 GLU A  37    41509  37907  29543 -13004   -868
225       O
ATOM    212  N   GLY A  38     -26.675 -42.339  30.256  1.00172.39
N
ANISOU  212  N   GLY A  38    23805  26731  14964 -12118  -1864
1618       N
ATOM    213  CA  GLY A  38     -27.983 -41.700  30.274  1.00177.39
C
ANISOU  213  CA  GLY A  38    23798  28180  15422 -12274  -2134
2053       C
ATOM    214  C   GLY A  38     -27.965 -40.236  29.868  1.00172.60
C
ANISOU  214  C   GLY A  38    22726  27912  14943 -11794  -2147
2158       C
ATOM    215  O   GLY A  38     -28.876 -39.762  29.184  1.00169.01
O
ANISOU  215  O   GLY A  38    21942  28024  14250 -12081  -2366
2455       O
ATOM    216  N   GLU A  39     -26.936 -39.498  30.288  1.00168.43
N
ANISOU  216  N   GLU A  39    22157  27050  14789 -11068  -1915
1936       N
ATOM    217  CA  GLU A  39     -26.766 -38.113  29.845  1.00181.47
C
ANISOU  217  CA  GLU A  39    23452  28923  16574 -10596  -1890
1984       C
ATOM    218  C   GLU A  39     -26.598 -37.983  28.332  1.00187.96
C
ANISOU  218  C   GLU A  39    24575  29680  17160 -10931  -1951
1881       C
ATOM    219  O   GLU A  39     -27.194 -37.094  27.713  1.00178.70
O
ANISOU  219  O   GLU A  39    23017  28995  15885 -10929  -2097
```

```
2128        O
ATOM    220  CB  GLU A  39      -25.557 -37.492  30.546  1.00199.71
C
ANISOU  220  CB  GLU A  39    25778  30799  19305  -9822  -1612
1712        C
ATOM    221  CG  GLU A  39      -25.675 -37.370  32.053  1.00224.38
C
ANISOU  221  CG  GLU A  39    28548  34030  22676  -9416  -1539
1821        C
ATOM    222  CD  GLU A  39      -24.501 -36.625  32.658  1.00204.18
C
ANISOU  222  CD  GLU A  39    25980  31103  20498  -8681  -1283
1576        C
ATOM    223  OE1 GLU A  39      -23.356 -37.103  32.514  1.00201.48
O
ANISOU  223  OE1 GLU A  39    26130  30147  20275  -8588  -1112
1237        O
ATOM    224  OE2 GLU A  39      -24.722 -35.560  33.271  1.00190.88
O
ANISOU  224  OE2 GLU A  39    23798  29740  18989  -8203  -1242
1729        O
ATOM    225  N   ASP A  40      -25.775 -38.836  27.721  1.00192.23
N
ANISOU  225  N   ASP A  40    25801  29617  17619 -11207  -1827
1525        N
ATOM    226  CA  ASP A  40      -25.469 -38.700  26.296  1.00186.84
C
ANISOU  226  CA  ASP A  40    25460  28806  16726 -11495  -1842
1374        C
ATOM    227  C   ASP A  40      -26.701 -38.773  25.392  1.00195.36
C
ANISOU  227  C   ASP A  40    26382  30482  17365 -12194  -2154
1703        C
ATOM    228  O   ASP A  40      -26.820 -37.985  24.446  1.00211.80
O
ANISOU  228  O   ASP A  40    28323  32815  19335 -12216  -2245
1788        O
ATOM    229  CB  ASP A  40      -24.452 -39.764  25.881  1.00187.79
C
ANISOU  229  CB  ASP A  40    26374  28159  16817 -11724  -1627
945       C
ATOM    230  CG  ASP A  40      -23.052 -39.449  26.365  1.00182.34
C
ANISOU  230  CG  ASP A  40    25851  26882  16549 -11011  -1314
610       C
ATOM    231  OD1 ASP A  40      -22.810 -38.293  26.773  1.00174.59
O
ANISOU  231  OD1 ASP A  40    24421  26080  15835 -10384  -1273
673       O
ATOM    232  OD2 ASP A  40      -22.192 -40.354  26.331  1.00177.01
O
ANISOU  232  OD2 ASP A  40    25756  25563  15935 -11084  -1102
295       O
ATOM    233  N   ARG A  41      -27.622 -39.705  25.651  1.00198.44
```

```
N
ANISOU  233  N   ARG A  41     26788  31118  17491 -12787  -2330
1907       N
ATOM    234  CA  ARG A  41      -28.799 -39.839  24.787  1.00198.50
C
ANISOU  234  CA  ARG A  41     26654  31716  17051 -13515  -2644
2241       C
ATOM    235  C   ARG A  41      -29.657 -38.576  24.737  1.00182.65
C
ANISOU  235  C   ARG A  41     23859  30467  15072 -13256  -2839
2686       C
ATOM    236  O   ARG A  41      -30.106 -38.170  23.658  1.00165.52
O
ANISOU  236  O   ARG A  41     21596  28657  12638 -13595  -3022
2865       O
ATOM    237  CB  ARG A  41      -29.645 -41.028  25.242  1.00182.48
C
ANISOU  237  CB  ARG A  41     24730  29842  14761 -14153  -2793
2403       C
ATOM    238  CG  ARG A  41      -28.935 -42.368  25.154  1.00185.49
C
ANISOU  238  CG  ARG A  41     25930  29493  15054 -14526  -2611
2003       C
ATOM    239  CD  ARG A  41      -28.353 -42.585  23.766  1.00197.01
C
ANISOU  239  CD  ARG A  41     28000  30582  16273 -14911  -2540
1710       C
ATOM    240  NE  ARG A  41      -29.387 -42.567  22.734  1.00194.02
N
ANISOU  240  NE  ARG A  41     27534  30754  15432 -15517  -2800
2021       N
ATOM    241  CZ  ARG A  41      -29.167 -42.836  21.451  1.00186.67
C
ANISOU  241  CZ  ARG A  41     27117  29601  14209 -15834  -2723
1880       C
ATOM    242  NH1 ARG A  41      -27.948 -43.155  21.038  1.00196.55
N
ANISOU  242  NH1 ARG A  41     29000  30109  15570 -15721  -2434
1399       N
ATOM    243  NH2 ARG A  41      -30.168 -42.795  20.582  1.00185.02
N
ANISOU  243  NH2 ARG A  41     26786  29915  13599 -16262  -2918
2237       N
ATOM    244  N   ILE A  42      -29.904 -37.944  25.882  1.00179.14
N
ANISOU  244  N   ILE A  42     22848  30275  14941 -12667  -2795
2881       N
ATOM    245  CA  ILE A  42      -30.607 -36.663  25.881  1.00178.41
C
ANISOU  245  CA  ILE A  42     22020  30829  14940 -12311  -2912
3283       C
ATOM    246  C   ILE A  42      -29.719 -35.500  25.430  1.00167.49
C
ANISOU  246  C   ILE A  42     20603  29224  13810 -11691  -2741
```

```
3097       C
ATOM    247  O   ILE A  42      -30.210 -34.550  24.807  1.00157.79
O
ANISOU  247  O   ILE A  42    18966  28458  12530 -11615  -2867
3383       O
ATOM    248  CB  ILE A  42      -31.234 -36.414  27.265  1.00181.34
C
ANISOU  248  CB  ILE A  42    21817  31547  15537 -11928  -2898
3559       C
ATOM    249  CG1 ILE A  42      -31.921 -35.044  27.323  1.00192.85
C
ANISOU  249  CG1 ILE A  42    22515  33627  17131 -11485  -2960
3971       C
ATOM    250  CG2 ILE A  42      -30.204 -36.595  28.358  1.00155.15
C
ANISOU  250  CG2 ILE A  42    18732  27633  12583 -11375  -2608
3193       C
ATOM    251  CD1 ILE A  42      -33.080 -34.891  26.348  1.00184.85
C
ANISOU  251  CD1 ILE A  42    21174  33301  15759 -12048  -3278
4430       C
ATOM    252  N   GLN A  43      -28.413 -35.552  25.706  1.00156.74
N
ANISOU  252  N   GLN A  43    19662  27171  12722 -11255  -2459
2638       N
ATOM    253  CA  GLN A  43      -27.503 -34.539  25.171  1.00153.88
C
ANISOU  253  CA  GLN A  43    19344  26561  12562 -10743  -2300
2430       C
ATOM    254  C   GLN A  43      -27.504 -34.520  23.646  1.00187.54
C
ANISOU  254  C   GLN A  43    23889  30862  16506 -11228  -2428
2400       C
ATOM    255  O   GLN A  43      -27.525 -33.445  23.035  1.00202.33
O
ANISOU  255  O   GLN A  43    25482  32976  18418 -10979  -2464
2528       O
ATOM    256  CB  GLN A  43      -26.089 -34.772  25.702  1.00145.57
C
ANISOU  256  CB  GLN A  43    18738  24752  11820 -10285  -1987
1947       C
ATOM    257  CG  GLN A  43      -25.063 -33.771  25.193  1.00151.78
C
ANISOU  257  CG  GLN A  43    19601  25247  12820  -9758  -1808
1707       C
ATOM    258  CD  GLN A  43      -23.648 -34.121  25.611  1.00155.50
C
ANISOU  258  CD  GLN A  43    20546  24976  13562  -9390  -1513
1244       C
ATOM    259  OE1 GLN A  43      -22.791 -34.401  24.772  1.00144.20
O
ANISOU  259  OE1 GLN A  43    19625  23087  12078  -9509  -1400
926       O
ATOM    260  NE2 GLN A  43      -23.396 -34.108  26.915  1.00151.17
```

```
N
ANISOU  260  NE2 GLN A  43     19822  24315  13302  -8946  -1381
1219       N
ATOM    261  N   ILE A  44      -27.478 -35.692  23.011  1.00209.19
N
ANISOU  261  N   ILE A  44     27199  33361  18924 -11933  -2489
2233       N
ATOM    262  CA  ILE A  44      -27.526 -35.743  21.552  1.00211.14
C
ANISOU  262  CA  ILE A  44     27747  33658  18817 -12476  -2614
2205       C
ATOM    263  C   ILE A  44      -28.935 -35.519  21.015  1.00204.41
C
ANISOU  263  C   ILE A  44     26427  33619  17619 -12999  -2971
2733       C
ATOM    264  O   ILE A  44      -29.094 -35.217  19.825  1.00202.69
O
ANISOU  264  O   ILE A  44     26279  33600  17135 -13354  -3111
2814       O
ATOM    265  CB  ILE A  44      -26.969 -37.073  21.015  1.00188.82
C
ANISOU  265  CB  ILE A  44     25748  30249  15748 -13067  -2517
1818       C
ATOM    266  CG1 ILE A  44      -27.882 -38.240  21.398  1.00163.88
C
ANISOU  266  CG1 ILE A  44     22671  27286  12309 -13727  -2684
1995       C
ATOM    267  CG2 ILE A  44      -25.548 -37.295  21.520  1.00164.27
C
ANISOU  267  CG2 ILE A  44     23076  26339  13000 -12529  -2154
1329       C
ATOM    268  CD1 ILE A  44      -27.378 -39.587  20.935  1.00166.13
C
ANISOU  268  CD1 ILE A  44     23793  26971  12358 -14316  -2561
1621       C
ATOM    269  N   PHE A  45      -29.962 -35.666  21.856  1.00171.42
N
ANISOU  269  N   PHE A  45     21768  29934  13432 -13070  -3124
3110       N
ATOM    270  CA  PHE A  45      -31.319 -35.319  21.443  1.00181.27
C
ANISOU  270  CA  PHE A  45     22457  32015  14403 -13472  -3457
3676       C
ATOM    271  C   PHE A  45      -31.448 -33.851  21.049  1.00184.70
C
ANISOU  271  C   PHE A  45     22343  32832  15003 -12969  -3490
3941       C
ATOM    272  O   PHE A  45      -32.089 -33.532  20.041  1.00193.16
O
ANISOU  272  O   PHE A  45     23230  34380  15783 -13384  -3732
4254       O
ATOM    273  CB  PHE A  45      -32.303 -35.656  22.566  1.00169.69
C
ANISOU  273  CB  PHE A  45     20530  30974  12970 -13509  -3563
```

```
4021       C
ATOM    274  CG  PHE A  45     -33.710 -35.199  22.299  1.00202.29
C
ANISOU  274  CG  PHE A  45    23977  36002  16883 -13807  -3880
4656       C
ATOM    275  CD1 PHE A  45     -34.231 -34.102  22.968  1.00172.62
C
ANISOU  275  CD1 PHE A  45    19458  32706  13422 -13176  -3863
5029       C
ATOM    276  CD2 PHE A  45     -34.508 -35.856  21.375  1.00210.55
C
ANISOU  276  CD2 PHE A  45    25215  37302  17481 -14534  -4056
4901       C
ATOM    277  CE1 PHE A  45     -35.523 -33.672  22.724  1.00183.04
C
ANISOU  277  CE1 PHE A  45    20119  34857  14569 -13418  -4136
5647       C
ATOM    278  CE2 PHE A  45     -35.803 -35.431  21.126  1.00197.53
C
ANISOU  278  CE2 PHE A  45    22991  36371  15688 -14633  -4227
5524       C
ATOM    279  CZ  PHE A  45     -36.310 -34.338  21.802  1.00194.41
C
ANISOU  279  CZ  PHE A  45    21789  36489  15587 -14064  -4265
5900       C
ATOM    280  N   GLY A  46     -30.852 -32.941  21.817  1.00170.63
N
ANISOU  280  N   GLY A  46    20302  30854  13675 -12097  -3250
3834       N
ATOM    281  CA  GLY A  46     -30.940 -31.539  21.475  1.00163.86
C
ANISOU  281  CA  GLY A  46    18956  30311  12991 -11595  -3249
4072       C
ATOM    282  C   GLY A  46     -30.532 -30.530  22.537  1.00160.01
C
ANISOU  282  C   GLY A  46    18091  29713  12994 -10655  -2993
4038       C
ATOM    283  O   GLY A  46     -29.969 -29.477  22.219  1.00154.62
O
ANISOU  283  O   GLY A  46    17315  28916  12517 -10146  -2864
3963       O
ATOM    284  N   PRO A  47     -30.822 -30.811  23.824  1.00156.11
N
ANISOU  284  N   PRO A  47    17374  29259  12683 -10423  -2910
4097       N
ATOM    285  CA  PRO A  47     -30.388 -29.891  24.885  1.00151.99
C
ANISOU  285  CA  PRO A  47    16547  28597  12605  -9561  -2644
4030       C
ATOM    286  C   PRO A  47     -28.905 -29.546  24.910  1.00147.19
C
ANISOU  286  C   PRO A  47    16376  27287  12264  -9054  -2359
3508       C
ATOM    287  O   PRO A  47     -28.036 -30.415  25.040  1.00145.58
```

```
O
ANISOU  287  O   PRO A  47    16741  26513  12060  -9182  -2245
3086       O
ATOM    288  CB  PRO A  47     -30.792 -30.646  26.155  1.00152.09
C
ANISOU  288  CB  PRO A  47    16462  28646  12680  -9595  -2614
4074       C
ATOM    289  CG  PRO A  47     -32.082 -31.266  25.770  1.00157.32
C
ANISOU  289  CG  PRO A  47    16894  29906  12976 -10306  -2931
4504       C
ATOM    290  CD  PRO A  47     -31.884 -31.709  24.323  1.00159.63
C
ANISOU  290  CD  PRO A  47    17641  30099  12914 -10935  -3103
4391       C
ATOM    291  N   ASN A  48     -28.629 -28.247  24.790  1.00162.54
N
ANISOU  291  N   ASN A  48    18034  29283  14439  -8468  -2235
3561       N
ATOM    292  CA  ASN A  48     -27.333 -27.652  25.088  1.00163.17
C
ANISOU  292  CA  ASN A  48    18361  28799  14837  -7845  -1943
3146       C
ATOM    293  C   ASN A  48     -27.585 -26.280  25.700  1.00177.07
C
ANISOU  293  C   ASN A  48    19553  30827  16898  -7148  -1800
3381       C
ATOM    294  O   ASN A  48     -28.703 -25.759  25.658  1.00204.16
O
ANISOU  294  O   ASN A  48    22434  34856  20282  -7160  -1929
3860       O
ATOM    295  CB  ASN A  48     -26.438 -27.564  23.846  1.00169.52
C
ANISOU  295  CB  ASN A  48    19636  29239  15536  -7980  -1928
2845       C
ATOM    296  CG  ASN A  48     -26.035 -28.928  23.324  1.00182.53
C
ANISOU  296  CG  ASN A  48    21921  30511  16921  -8611  -1990
2544       C
ATOM    297  OD1 ASN A  48     -26.333 -29.285  22.184  1.00217.29
O
ANISOU  297  OD1 ASN A  48    26521  35046  20994  -9187  -2178
2609       O
ATOM    298  ND2 ASN A  48     -25.354 -29.703  24.162  1.00141.85
N
ANISOU  298  ND2 ASN A  48    17105  24883  11909  -8513  -1822
2221       N
ATOM    299  N   LYS A  49     -26.536 -25.701  26.292  1.00170.59
N
ANISOU  299  N   LYS A  49    18871  29557  16387  -6541  -1520
3050       N
ATOM    300  CA  LYS A  49     -26.721 -24.513  27.124  1.00172.27
C
ANISOU  300  CA  LYS A  49    18609  29949  16897  -5873  -1331
```

```
3219       C
ATOM    301  C   LYS A  49      -27.317 -23.336  26.347  1.00193.66
C
ANISOU  301  C   LYS A  49    20895  33079  19607  -5698  -1390
3583       C
ATOM    302  O   LYS A  49      -28.252 -22.685  26.829  1.00205.20
O
ANISOU  302  O   LYS A  49    21799  35002  21165  -5455  -1372
3987       O
ATOM    303  CB  LYS A  49      -25.397 -24.122  27.783  1.00189.96
C
ANISOU  303  CB  LYS A  49    21138  31610  19430  -5323  -1032
2773       C
ATOM    304  CG  LYS A  49      -25.483 -22.867  28.643  1.00193.90
C
ANISOU  304  CG  LYS A  49    21222  32230  20222  -4642   -800
2895       C
ATOM    305  CD  LYS A  49      -24.211 -22.662  29.456  1.00177.91
C
ANISOU  305  CD  LYS A  49    19498  29653  18446  -4188   -525
2460       C
ATOM    306  CE  LYS A  49      -24.367 -21.528  30.460  1.00154.61
C
ANISOU  306  CE  LYS A  49    16166  26824  15755  -3574   -281
2572       C
ATOM    307  NZ  LYS A  49      -24.542 -20.203  29.807  1.00171.18
N
ANISOU  307  NZ  LYS A  49    18005  29101  17937  -3236   -212
2755       N
ATOM    308  N   LEU A  50      -26.814 -23.048  25.140  1.00189.83
N
ANISOU  308  N   LEU A  50    20657  32451  19018  -5817  -1453
3467       N
ATOM    309  CA  LEU A  50      -27.323 -21.885  24.415  1.00181.28
C
ANISOU  309  CA  LEU A  50    19175  31749  17955  -5618  -1500
3820       C
ATOM    310  C   LEU A  50      -27.162 -22.049  22.904  1.00151.60
C
ANISOU  310  C   LEU A  50    15686  27996  13918  -6089  -1708
3800       C
ATOM    311  O   LEU A  50      -26.264 -22.749  22.427  1.00140.04
O
ANISOU  311  O   LEU A  50    14797  26073  12338  -6363  -1704
3383       O
ATOM    312  CB  LEU A  50      -26.619 -20.604  24.890  1.00194.02
C
ANISOU  312  CB  LEU A  50    20693  33109  19919  -4843  -1190
3673       C
ATOM    313  CG  LEU A  50      -27.442 -19.332  25.151  1.00205.59
C
ANISOU  313  CG  LEU A  50    21533  35012  21571  -4367  -1096
4123       C
ATOM    314  CD1 LEU A  50      -28.038 -18.743  23.882  1.00200.84
```

```
C
ANISOU  314  CD1 LEU A  50    20679  34808  20822  -4541  -1292
4505       C
ATOM    315  CD2 LEU A  50     -28.540 -19.589  26.183  1.00225.75
C
ANISOU  315  CD2 LEU A  50    23631  37982  24162  -4358  -1101
4471       C
ATOM    316  N   GLU A  51     -28.060 -21.389  22.164  1.00145.57
N
ANISOU  316  N   GLU A  51    14491  27768  13050  -6180  -1880
4275       N
ATOM    317  CA  GLU A  51     -28.042 -21.357  20.701  1.00170.87
C
ANISOU  317  CA  GLU A  51    17862  31079  15983  -6612  -2091
4342       C
ATOM    318  C   GLU A  51     -26.800 -20.677  20.121  1.00177.56
C
ANISOU  318  C   GLU A  51    19077  31430  16956  -6277  -1915
3952       C
ATOM    319  O   GLU A  51     -26.281 -19.705  20.676  1.00174.60
O
ANISOU  319  O   GLU A  51    18586  30848  16905  -5603  -1657
3846       O
ATOM    320  CB  GLU A  51     -29.308 -20.665  20.195  1.00184.14
C
ANISOU  320  CB  GLU A  51    18906  33483  17576  -6688  -2294
5001       C
ATOM    321  CG  GLU A  51     -30.583 -21.422  20.548  1.00184.22
C
ANISOU  321  CG  GLU A  51    18560  34044  17392  -7147  -2521
5425       C
ATOM    322  CD  GLU A  51     -31.819 -20.836  19.897  1.00202.98
C
ANISOU  322  CD  GLU A  51    20318  37163  19641  -7310  -2758
6108       C
ATOM    323  OE1 GLU A  51     -32.176 -19.686  20.226  1.00215.88
O
ANISOU  323  OE1 GLU A  51    21442  39026  21555  -6712  -2613
6426       O
ATOM    324  OE2 GLU A  51     -32.434 -21.528  19.058  1.00209.32
O
ANISOU  324  OE2 GLU A  51    21147  38323  20062  -8044  -3082
6338       O
ATOM    325  N   GLU A  52     -26.332 -21.209  18.988  1.00187.80
N
ANISOU  325  N   GLU A  52    20836  32540  17978  -6779  -2049
3738       N
ATOM    326  CA  GLU A  52     -25.283 -20.612  18.157  1.00198.82
C
ANISOU  326  CA  GLU A  52    22571  33557  19415  -6596  -1940
3431       C
ATOM    327  C   GLU A  52     -25.701 -19.296  17.488  1.00190.29
C
ANISOU  327  C   GLU A  52    21061  32849  18391  -6325  -1993
```

```
3818       C
ATOM    328  O   GLU A  52      -26.871 -19.077  17.161  1.00188.66
O
ANISOU  328  O   GLU A  52    20379  33253  18049  -6543  -2214
4359       O
ATOM    329  CB  GLU A  52      -24.831 -21.613  17.089  1.00201.17
C
ANISOU  329  CB  GLU A  52    23459  33614  19361  -7283  -2079
3146       C
ATOM    330  CG  GLU A  52      -23.712 -21.127  16.162  1.00195.47
C
ANISOU  330  CG  GLU A  52    23141  32484  18647  -7165  -1966
2800       C
ATOM    331  CD  GLU A  52      -22.412 -20.814  16.890  1.00192.64
C
ANISOU  331  CD  GLU A  52    23053  31505  18635  -6545  -1627
2321       C
ATOM    332  OE1 GLU A  52      -21.432 -21.564  16.699  1.00197.78
O
ANISOU  332  OE1 GLU A  52    24275  31635  19237  -6712  -1516
1858       O
ATOM    333  OE2 GLU A  52      -22.355 -19.806  17.627  1.00189.63
O
ANISOU  333  OE2 GLU A  52    22322  31158  18572  -5897  -1462
2415       O
ATOM    334  N   LYS A  53      -24.711 -18.415  17.297  1.00188.51
N
ANISOU  334  N   LYS A  53    21006  32246  18374  -5840  -1781
3547       N
ATOM    335  CA  LYS A  53      -24.895 -17.127  16.627  1.00191.75
C
ANISOU  335  CA  LYS A  53    21096  32898  18861  -5539  -1790
3844       C
ATOM    336  C   LYS A  53      -25.318 -17.266  15.161  1.00221.26
C
ANISOU  336  C   LYS A  53    24877  36961  22231  -6152  -2095
4082       C
ATOM    337  O   LYS A  53      -24.864 -18.159  14.439  1.00229.35
O
ANISOU  337  O   LYS A  53    26406  37764  22972  -6707  -2200
3787       O
ATOM    338  CB  LYS A  53      -23.606 -16.306  16.711  1.00170.85
C
ANISOU  338  CB  LYS A  53    18729  29706  16479  -4965  -1497
3425       C
ATOM    339  CG  LYS A  53      -23.181 -15.934  18.124  1.00164.03
C
ANISOU  339  CG  LYS A  53    17785  28558  15981  -4321  -1187
3230       C
ATOM    340  CD  LYS A  53      -24.192 -15.008  18.786  1.00198.43
C
ANISOU  340  CD  LYS A  53    21493  33357  20544  -3888  -1126
3723       C
ATOM    341  CE  LYS A  53      -23.756 -14.622  20.194  1.00178.87
```

```
C
ANISOU  341  CE  LYS A  53    18972  30586  18402  -3278   -800
3510       C
ATOM    342  NZ  LYS A  53     -22.505 -13.812  20.199  1.00124.03
N
ANISOU  342  NZ  LYS A  53    12328  23137  11660  -2808   -541
3115       N
ATOM    343  N   LYS A  54     -26.196 -16.355  14.737  1.00227.29
N
ANISOU  343  N   LYS A  54    25102  38255  23004  -6041  -2221
4634       N
ATOM    344  CA  LYS A  54     -26.685 -16.228  13.363  1.00223.16
C
ANISOU  344  CA  LYS A  54    24502  38127  22160  -6546  -2516
4965       C
ATOM    345  C   LYS A  54     -25.589 -15.890  12.347  1.00246.08
C
ANISOU  345  C   LYS A  54    27883  40626  24989  -6572  -2460
4594       C
ATOM    346  O   LYS A  54     -24.561 -15.292  12.677  1.00260.41
O
ANISOU  346  O   LYS A  54    29913  41950  27081  -6019  -2175
4217       O
ATOM    347  CB  LYS A  54     -27.773 -15.154  13.289  1.00196.91
C
ANISOU  347  CB  LYS A  54    20443  35419  18955  -6258  -2603
5655       C
ATOM    348  CG  LYS A  54     -27.289 -13.756  13.641  1.00165.74
C
ANISOU  348  CG  LYS A  54    16308  31255  15412  -5415  -2303
5641       C
ATOM    349  CD  LYS A  54     -28.439 -12.764  13.695  1.00156.74
C
ANISOU  349  CD  LYS A  54    14421  30710  14421  -5098  -2349
6352       C
ATOM    350  CE  LYS A  54     -29.147 -12.660  12.353  1.00179.83
C
ANISOU  350  CE  LYS A  54    17126  34173  17027  -5627  -2706
6843       C
ATOM    351  NZ  LYS A  54     -30.221 -11.628  12.372  1.00175.70
N
ANISOU  351  NZ  LYS A  54    15854  34222  16684  -5267  -2732
7571       N
ATOM    352  N   GLU A  55     -25.822 -16.321  11.095  1.00256.48
N
ANISOU  352  N   GLU A  55    29380  42167  25904  -7268  -2739
4704       N
ATOM    353  CA  GLU A  55     -25.035 -16.004   9.888  1.00253.69
C
ANISOU  353  CA  GLU A  55    29415  41584  25391  -7437  -2759
4477       C
ATOM    354  C   GLU A  55     -23.738 -16.789   9.717  1.00254.05
C
ANISOU  354  C   GLU A  55    30225  40941  25360  -7610  -2588
```

```
3767       C
ATOM    355  O   GLU A  55     -22.856 -16.353   8.968  1.00250.21
O
ANISOU  355  O   GLU A  55    30063  40146  24859  -7535  -2499
3494       O
ATOM    356  CB  GLU A  55     -24.723 -14.505   9.778  1.00231.96
C
ANISOU  356  CB  GLU A  55    26387  38804  22944  -6749  -2601
4613       C
ATOM    357  CG  GLU A  55     -25.952 -13.625   9.594  1.00207.00
C
ANISOU  357  CG  GLU A  55    22500  36329  19823  -6623  -2778
5359       C
ATOM    358  CD  GLU A  55     -25.598 -12.174   9.332  1.00184.07
C
ANISOU  358  CD  GLU A  55    19393  33359  17188  -6000  -2621
5482       C
ATOM    359  OE1 GLU A  55     -25.052 -11.518  10.243  1.00167.37
O
ANISOU  359  OE1 GLU A  55    17248  30887  15460  -5286  -2297
5268       O
ATOM    360  OE2 GLU A  55     -25.869 -11.695   8.208  1.00156.76
O
ANISOU  360  OE2 GLU A  55    15816  30206  13539  -6247  -2822
5798       O
ATOM    361  N   SER A  56     -23.589 -17.935  10.376  1.00247.37
N
ANISOU  361  N   SER A  56    29676  39842  24471  -7834  -2531
3469       N
ATOM    362  CA  SER A  56     -22.430 -18.793  10.150  1.00223.85
C
ANISOU  362  CA  SER A  56    27425  36230  21400  -8055  -2373
2838       C
ATOM    363  C   SER A  56     -22.525 -19.603   8.858  1.00236.29
C
ANISOU  363  C   SER A  56    29420  37874  22485  -8920  -2588
2780       C
ATOM    364  O   SER A  56     -21.487 -19.989   8.309  1.00238.69
O
ANISOU  364  O   SER A  56    30320  37670  22700  -9068  -2442
2293       O
ATOM    365  CB  SER A  56     -22.235 -19.740  11.335  1.00201.38
C
ANISOU  365  CB  SER A  56    24748  33073  18695  -7976  -2222
2562       C
ATOM    366  OG  SER A  56     -21.951 -19.012  12.516  1.00203.50
O
ANISOU  366  OG  SER A  56    24726  33191  19402  -7189  -1985
2528       O
ATOM    367  N   LYS A  57     -23.735 -19.860   8.361  1.00249.86
N
ANISOU  367  N   LYS A  57    30844  40212  23878  -9499  -2920
3269       N
ATOM    368  CA  LYS A  57     -23.922 -20.596   7.114  1.00239.54
```

```
C
ANISOU  368  CA  LYS A  57    29922  39031  22061 -10384  -3145
3255       C
ATOM    369  C   LYS A  57     -23.333 -19.867   5.907  1.00224.25
C
ANISOU  369  C   LYS A  57    28179  37019  20008 -10424  -3153
3176       C
ATOM    370  O   LYS A  57     -23.308 -18.635   5.837  1.00221.53
O
ANISOU  370  O   LYS A  57    27446  36829  19897  -9893  -3127
3408       O
ATOM    371  CB  LYS A  57     -25.407 -20.863   6.862  1.00223.36
C
ANISOU  371  CB  LYS A  57    27422  37748  19695 -10969  -3524
3877       C
ATOM    372  CG  LYS A  57     -26.263 -19.616   6.722  1.00216.31
C
ANISOU  372  CG  LYS A  57    25776  37487  18925 -10645  -3698
4527       C
ATOM    373  CD  LYS A  57     -27.727 -19.984   6.534  1.00203.11
C
ANISOU  373  CD  LYS A  57    23646  36585  16942 -11244  -4071
5157       C
ATOM    374  CE  LYS A  57     -28.620 -18.753   6.525  1.00184.98
C
ANISOU  374  CE  LYS A  57    20540  34921  14824 -10848  -4212
5851       C
ATOM    375  NZ  LYS A  57     -28.392 -17.911   5.316  1.00174.16
N
ANISOU  375  NZ  LYS A  57    19170  33678  13326 -10915  -4315
5996       N
ATOM    376  N   LEU A  58     -22.832 -20.667   4.960  1.00210.79
N
ANISOU  376  N   LEU A  58    27114  35042  17935 -11068  -3166
2826       N
ATOM    377  CA  LEU A  58     -22.425 -20.283   3.607  1.00205.70
C
ANISOU  377  CA  LEU A  58    26744  34388  17024 -11385  -3233
2760       C
ATOM    378  C   LEU A  58     -21.117 -19.504   3.554  1.00185.14
C
ANISOU  378  C   LEU A  58    24372  31235  14736 -10738  -2921
2345       C
ATOM    379  O   LEU A  58     -20.757 -18.999   2.481  1.00178.37
O
ANISOU  379  O   LEU A  58    23679  30384  13709 -10896  -2963
2316       O
ATOM    380  CB  LEU A  58     -23.517 -19.486   2.881  1.00198.81
C
ANISOU  380  CB  LEU A  58    25300  34285  15953 -11619  -3589
3442       C
ATOM    381  CG  LEU A  58     -24.907 -20.124   2.807  1.00188.10
C
ANISOU  381  CG  LEU A  58    23627  33590  14250 -12301  -3947
```

```
3956       C
ATOM    382  CD1 LEU A  58      -25.908 -19.156   2.198  1.00171.92
C
ANISOU  382  CD1 LEU A  58    20912  32245  12165 -12333  -4227
4665       C
ATOM    383  CD2 LEU A  58      -24.860 -21.421   2.013  1.00171.55
C
ANISOU  383  CD2 LEU A  58    22143  31193  11845 -13133  -3910
3668       C
ATOM    384  N   LEU A  59      -20.394 -19.396   4.670  1.00171.75
N
ANISOU  384  N   LEU A  59    22698  29082  13479 -10039  -2617
2030       N
ATOM    385  CA  LEU A  59      -19.177 -18.582   4.752  1.00149.51
C
ANISOU  385  CA  LEU A  59    20036  25780  10993  -9376  -2323
1673       C
ATOM    386  C   LEU A  59      -19.423 -17.147   4.291  1.00151.97
C
ANISOU  386  C   LEU A  59    19886  26447  11410  -9019  -2422
2057       C
ATOM    387  O   LEU A  59      -18.602 -16.547   3.595  1.00150.88
O
ANISOU  387  O   LEU A  59    19979  26064  11286  -8874  -2314
1844       O
ATOM    388  CB  LEU A  59      -18.035 -19.223   3.959  1.00158.69
C
ANISOU  388  CB  LEU A  59    21953  26376  11968  -9698  -2140
1101       C
ATOM    389  CG  LEU A  59      -17.719 -20.683   4.288  1.00158.36
C
ANISOU  389  CG  LEU A  59    22440  25922  11808 -10080  -2008
703       C
ATOM    390  CD1 LEU A  59      -16.622 -21.221   3.380  1.00173.94
C
ANISOU  390  CD1 LEU A  59    25145  27354  13590 -10393  -1806
176       C
ATOM    391  CD2 LEU A  59      -17.334 -20.833   5.750  1.00140.30
C
ANISOU  391  CD2 LEU A  59    20038  23312   9957  -9448  -1775
524       C
ATOM    392  N   LYS A  60      -20.581 -16.601   4.674  1.00184.19
N
ANISOU  392  N   LYS A  60    23306  31114  15564  -8880  -2623
2643       N
ATOM    393  CA  LYS A  60      -21.022 -15.314   4.144  1.00203.67
C
ANISOU  393  CA  LYS A  60    25298  33999  18087  -8634  -2755
3109       C
ATOM    394  C   LYS A  60      -20.018 -14.212   4.466  1.00216.35
C
ANISOU  394  C   LYS A  60    26908  35196  20097  -7828  -2458
2863       C
ATOM    395  O   LYS A  60      -19.670 -13.400   3.601  1.00229.88
```

```
O
ANISOU  395  O   LYS A  60    28653  36923  21769  -7764  -2475
2907       O
ATOM    396  CB  LYS A  60     -22.406 -14.966   4.696  1.00199.49
C
ANISOU  396  CB  LYS A  60    24038  34121  17637  -8531  -2961
3774       C
ATOM    397  CG  LYS A  60     -22.947 -13.618   4.232  1.00188.75
C
ANISOU  397  CG  LYS A  60    22129  33210  16378  -8222  -3079
4321       C
ATOM    398  CD  LYS A  60     -23.082 -13.543   2.717  1.00178.54
C
ANISOU  398  CD  LYS A  60    20980  32192  14664  -8854  -3343
4497       C
ATOM    399  CE  LYS A  60     -24.025 -14.612   2.185  1.00173.97
C
ANISOU  399  CE  LYS A  60    20423  32073  13604  -9773  -3684
4757       C
ATOM    400  NZ  LYS A  60     -24.251 -14.478   0.719  1.00165.70
N
ANISOU  400  NZ  LYS A  60    19468  31365  12125 -10418  -3964
4992       N
ATOM    401  N   PHE A  61     -19.540 -14.170   5.711  1.00222.60
N
ANISOU  401  N   PHE A  61    27677  35629  21270  -7229  -2186
2607       N
ATOM    402  CA  PHE A  61     -18.485 -13.247   6.113  1.00217.41
C
ANISOU  402  CA  PHE A  61    27094  34530  20980  -6504  -1881
2309       C
ATOM    403  C   PHE A  61     -17.223 -13.964   6.578  1.00227.05
C
ANISOU  403  C   PHE A  61    28882  35076  22312  -6393  -1600
1648       C
ATOM    404  O   PHE A  61     -16.316 -13.316   7.115  1.00244.56
O
ANISOU  404  O   PHE A  61    31162  36911  24850  -5788  -1334
1377       O
ATOM    405  CB  PHE A  61     -18.987 -12.313   7.219  1.00203.23
C
ANISOU  405  CB  PHE A  61    24721  32920  19576  -5802  -1777
2630       C
ATOM    406  CG  PHE A  61     -20.136 -11.442   6.801  1.00169.21
C
ANISOU  406  CG  PHE A  61    19820  29237  15234  -5779  -1997
3301       C
ATOM    407  CD1 PHE A  61     -21.271 -11.351   7.588  1.00164.82
C
ANISOU  407  CD1 PHE A  61    18708  29116  14801  -5626  -2075
3769       C
ATOM    408  CD2 PHE A  61     -20.082 -10.716   5.623  1.00145.27
C
ANISOU  408  CD2 PHE A  61    16777  26367  12054  -5907  -2118
```

```
3483       C
ATOM    409  CE1 PHE A  61     -22.331 -10.551   7.209  1.00171.49
C
ANISOU  409  CE1 PHE A  61    18979  30544  15637  -5580  -2260
4421       C
ATOM    410  CE2 PHE A  61     -21.140  -9.914   5.238  1.00142.97
C
ANISOU  410  CE2 PHE A  61    15917  26658  11746  -5873  -2320
4139       C
ATOM    411  CZ  PHE A  61     -22.265  -9.832   6.033  1.00144.95
C
ANISOU  411  CZ  PHE A  61    15601  27338  12136  -5700  -2386
4617       C
ATOM    412  N   LEU A  62     -17.139 -15.279   6.386  1.00215.52
N
ANISOU  412  N   LEU A  62    27835  33459  20593  -6966  -1646
1397       N
ATOM    413  CA  LEU A  62     -15.988 -16.075   6.790  1.00209.87
C
ANISOU  413  CA  LEU A  62    27658  32110  19974  -6898  -1379
806       C
ATOM    414  C   LEU A  62     -15.119 -16.445   5.596  1.00173.15
C
ANISOU  414  C   LEU A  62    23596  27129  15065  -7305  -1323
433       C
ATOM    415  O   LEU A  62     -14.076 -17.087   5.763  1.00176.70
O
ANISOU  415  O   LEU A  62    24526  27029  15584  -7263  -1080
-62       O
ATOM    416  CB  LEU A  62     -16.461 -17.338   7.524  1.00241.96
C
ANISOU  416  CB  LEU A  62    31799  36166  23969  -7208  -1416
767       C
ATOM    417  CG  LEU A  62     -15.445 -18.253   8.215  1.00243.42
C
ANISOU  417  CG  LEU A  62    32448  35736  24304  -7090  -1141
239       C
ATOM    418  CD1 LEU A  62     -14.515 -17.451   9.114  1.00232.23
C
ANISOU  418  CD1 LEU A  62    30938  33973  23326  -6290   -861
25       C
ATOM    419  CD2 LEU A  62     -16.158 -19.334   9.007  1.00234.14
C
ANISOU  419  CD2 LEU A  62    31222  34667  23074  -7362  -1220
327       C
ATOM    420  N   GLY A  63     -15.516 -16.021   4.401  1.00158.80
N
ANISOU  420  N   GLY A  63    21738  25640  12958  -7681  -1532
674       N
ATOM    421  CA  GLY A  63     -14.714 -16.124   3.201  1.00178.74
C
ANISOU  421  CA  GLY A  63    24769  27900  15243  -8018  -1477
363       C
ATOM    422  C   GLY A  63     -14.631 -14.791   2.487  1.00146.54
```

```
C
ANISOU  422  C   GLY A  63    20457  24024  11197  -7772  -1539
581       C
ATOM    423  O   GLY A  63     -13.966 -14.667   1.454  1.00133.79
O
ANISOU  423  O   GLY A  63    19210  22228   9398  -7996  -1498
360       O
ATOM    424  N   PHE A  64     -15.306 -13.783   3.038  1.00149.54
N
ANISOU  424  N   PHE A  64    20231  24771  11816  -7302  -1623
1019       N
ATOM    425  CA  PHE A  64     -15.476 -12.501   2.368  1.00150.00
C
ANISOU  425  CA  PHE A  64    19989  25110  11896  -7096  -1721
1343       C
ATOM    426  C   PHE A  64     -15.435 -11.362   3.376  1.00130.78
C
ANISOU  426  C   PHE A  64    17109  22661   9920  -6255  -1560
1496       C
ATOM    427  O   PHE A  64     -16.083 -11.422   4.425  1.00130.50
O
ANISOU  427  O   PHE A  64    16710  22793  10081  -5995  -1557
1708       O
ATOM    428  CB  PHE A  64     -16.806 -12.486   1.599  1.00184.10
C
ANISOU  428  CB  PHE A  64    23961  30111  15877  -7650  -2104
1930       C
ATOM    429  CG  PHE A  64     -17.143 -11.161   0.974  1.00205.03
C
ANISOU  429  CG  PHE A  64    26220  33115  18567  -7425  -2228
2363       C
ATOM    430  CD1 PHE A  64     -16.356 -10.624  -0.029  1.00221.38
C
ANISOU  430  CD1 PHE A  64    28586  34996  20533  -7473  -2180
2174       C
ATOM    431  CD2 PHE A  64     -18.266 -10.460   1.386  1.00217.13
C
ANISOU  431  CD2 PHE A  64    27081  35173  20246  -7165  -2383
2978       C
ATOM    432  CE1 PHE A  64     -16.676  -9.404  -0.602  1.00228.34
C
ANISOU  432  CE1 PHE A  64    29105  36198  21454  -7265  -2296
2591       C
ATOM    433  CE2 PHE A  64     -18.592  -9.243   0.818  1.00225.82
C
ANISOU  433  CE2 PHE A  64    27812  36587  21401  -6938  -2482
3406       C
ATOM    434  CZ  PHE A  64     -17.797  -8.714  -0.178  1.00228.07
C
ANISOU  434  CZ  PHE A  64    28403  36673  21579  -6992  -2445
3214       C
ATOM    435  N   MET A  65     -14.665 -10.330   3.040  1.00140.57
N
ANISOU  435  N   MET A  65    18402  23694  11313  -5854  -1416
```

```
1381       N
ATOM    436  CA  MET A  65     -14.409  -9.160   3.866  1.00154.82
C
ANISOU  436  CA  MET A  65    19896  25394  13534  -5064  -1215
1447       C
ATOM    437  C   MET A  65     -15.305  -8.013   3.416  1.00180.25
C
ANISOU  437  C   MET A  65    22604  29113  16771  -4921  -1390
2041       C
ATOM    438  O   MET A  65     -15.880  -8.044   2.324  1.00181.32
O
ANISOU  438  O   MET A  65    22684  29619  16589  -5423  -1657
2342       O
ATOM    439  CB  MET A  65     -12.939  -8.741   3.782  1.00153.73
C
ANISOU  439  CB  MET A  65    20165  24694  13554  -4720   -932
936       C
ATOM    440  CG  MET A  65     -12.587  -7.981   2.511  1.00169.08
C
ANISOU  440  CG  MET A  65    22229  26678  15335  -4845   -997
980       C
ATOM    441  SD  MET A  65     -12.896  -8.911   0.996  1.00161.14
S
ANISOU  441  SD  MET A  65    21557  25889  13778  -5799  -1277
1002       S
ATOM    442  CE  MET A  65     -13.238  -7.577  -0.149  1.00129.62
C
ANISOU  442  CE  MET A  65    17298  22271   9682  -5789  -1458
1441       C
ATOM    443  N   TRP A  66     -15.413  -6.986   4.261  1.00168.97
N
ANISOU  443  N   TRP A  66    20805  27685  15710  -4234  -1227
2219       N
ATOM    444  CA  TRP A  66     -16.083  -5.774   3.804  1.00162.36
C
ANISOU  444  CA  TRP A  66    19524  27230  14935  -4009  -1329
2750       C
ATOM    445  C   TRP A  66     -15.760  -4.559   4.668  1.00154.63
C
ANISOU  445  C   TRP A  66    18330  26043  14381  -3196  -1040
2762       C
ATOM    446  O   TRP A  66     -16.630  -3.713   4.901  1.00143.37
O
ANISOU  446  O   TRP A  66    16401  24957  13118  -2874  -1063
3271       O
ATOM    447  CB  TRP A  66     -17.595  -6.013   3.787  1.00171.87
C
ANISOU  447  CB  TRP A  66    20217  29073  16012  -4295  -1613
3372       C
ATOM    448  CG  TRP A  66     -18.280  -5.504   2.569  1.00172.06
C
ANISOU  448  CG  TRP A  66    20000  29576  15799  -4625  -1896
3877       C
ATOM    449  CD1 TRP A  66     -17.745  -4.718   1.593  1.00176.18
```

```
C
ANISOU  449  CD1 TRP A  66    20666  30021  16256  -4604  -1902
3864       C
ATOM    450  CD2 TRP A  66     -19.620  -5.799   2.160  1.00176.86
C
ANISOU  450  CD2 TRP A  66    20187  30837  16175  -5077  -2233
4480       C
ATOM    451  NE1 TRP A  66     -18.679  -4.480   0.614  1.00193.17
N
ANISOU  451  NE1 TRP A  66    22500  32737  18157  -5000  -2224
4436       N
ATOM    452  CE2 TRP A  66     -19.838  -5.136   0.938  1.00180.87
C
ANISOU  452  CE2 TRP A  66    20587  31645  16492  -5301  -2435
4829       C
ATOM    453  CE3 TRP A  66     -20.659  -6.550   2.716  1.00181.65
C
ANISOU  453  CE3 TRP A  66    20484  31819  16715  -5321  -2389
4776       C
ATOM    454  CZ2 TRP A  66     -21.055  -5.198   0.263  1.00190.68
C
ANISOU  454  CZ2 TRP A  66    21409  33562  17478  -5763  -2793
5478       C
ATOM    455  CZ3 TRP A  66     -21.865  -6.612   2.044  1.00187.64
C
ANISOU  455  CZ3 TRP A  66    20829  33247  17220  -5779  -2739
5412       C
ATOM    456  CH2 TRP A  66     -22.053  -5.940   0.831  1.00201.23
C
ANISOU  456  CH2 TRP A  66    22438  35267  18752  -5998  -2941
5764       C
ATOM    457  N   ASN A  67     -14.522  -4.462   5.154  1.00164.46
N
ANISOU  457  N   ASN A  67    19951  26733  15803  -2865   -755
2219       N
ATOM    458  CA  ASN A  67     -14.119  -3.307   5.942  1.00183.48
C
ANISOU  458  CA  ASN A  67    22222  28911  18580  -2141   -468
2186       C
ATOM    459  C   ASN A  67     -14.100  -2.036   5.084  1.00161.02
C
ANISOU  459  C   ASN A  67    19254  26168  15759  -1947   -482
2452       C
ATOM    460  O   ASN A  67     -14.013  -2.105   3.856  1.00194.42
O
ANISOU  460  O   ASN A  67    23637  30517  19718  -2369   -675
2502       O
ATOM    461  CB  ASN A  67     -12.769  -3.569   6.622  1.00213.24
C
ANISOU  461  CB  ASN A  67    26433  32090  22497  -1903   -187
1551       C
ATOM    462  CG  ASN A  67     -11.617  -3.754   5.643  1.00228.26
C
ANISOU  462  CG  ASN A  67    28842  33667  24219  -2168   -171
```

```
1132        C
ATOM    463  OD1 ASN A  67      -11.698  -3.394   4.474  1.00236.65
O
ANISOU  463  OD1 ASN A  67    29938  34893  25086  -2421   -323
1292        O
ATOM    464  ND2 ASN A  67      -10.526  -4.330   6.135  1.00232.17
N
ANISOU  464  ND2 ASN A  67    29728  33704  24783  -2108     23
601        N
ATOM    465  N   PRO A  68      -14.220  -0.855   5.720  1.00152.62
N
ANISOU  465  N   PRO A  68    17911  25064  15013  -1316   -271
2645        N
ATOM    466  CA  PRO A  68      -14.089   0.426   5.001  1.00149.43
C
ANISOU  466  CA  PRO A  68    17423  24680  14672  -1061   -233
2865        C
ATOM    467  C   PRO A  68      -12.950   0.544   3.990  1.00197.88
C
ANISOU  467  C   PRO A  68    24029  30518  20640  -1268   -234
2489        C
ATOM    468  O   PRO A  68      -13.143   1.089   2.900  1.00219.81
O
ANISOU  468  O   PRO A  68    26743  33502  23273  -1433   -390
2762        O
ATOM    469  CB  PRO A  68      -13.906   1.430   6.146  1.00154.00
C
ANISOU  469  CB  PRO A  68    17859  25013  15639   -329    115
2842        C
ATOM    470  CG  PRO A  68      -14.732   0.856   7.237  1.00169.63
C
ANISOU  470  CG  PRO A  68    19544  27177  17729   -251    133
2998        C
ATOM    471  CD  PRO A  68      -14.501  -0.638   7.151  1.00177.48
C
ANISOU  471  CD  PRO A  68    20824  28138  18473   -814    -40
2672        C
ATOM    472  N   LEU A  69      -11.760   0.057   4.350  1.00174.95
N
ANISOU  472  N   LEU A  69    21580  27138  17757  -1253    -55
1882        N
ATOM    473  CA  LEU A  69      -10.627   0.051   3.423  1.00142.65
C
ANISOU  473  CA  LEU A  69    17950  22749  13500  -1468    -37
1492        C
ATOM    474  C   LEU A  69      -10.910  -0.753   2.156  1.00156.73
C
ANISOU  474  C   LEU A  69    19879  24785  14888  -2179   -341
1566        C
ATOM    475  O   LEU A  69      -10.563  -0.324   1.050  1.00166.18
O
ANISOU  475  O   LEU A  69    21233  25992  15916  -2369   -420
1576        O
ATOM    476  CB  LEU A  69       -9.385  -0.495   4.129  1.00139.46
```

```
C
ANISOU  476  CB  LEU A  69    17964  21829  13197  -1346    205
866       C
ATOM    477  CG  LEU A  69      -8.962   0.193   5.428  1.00145.75
C
ANISOU  477  CG  LEU A  69    18689  22344  14347   -709    512
721       C
ATOM    478  CD1 LEU A  69      -7.657  -0.397   5.937  1.00151.75
C
ANISOU  478  CD1 LEU A  69    19874  22625  15158   -672    710
122       C
ATOM    479  CD2 LEU A  69      -8.834   1.694   5.227  1.00137.09
C
ANISOU  479  CD2 LEU A  69    17470  21207  13409   -274    642
906       C
ATOM    480  N   SER A  70     -11.538  -1.919   2.298  1.00171.33
N
ANISOU  480  N   SER A  70    21694  26837  16568  -2601   -508
1614       N
ATOM    481  CA  SER A  70     -12.011  -2.681   1.143  1.00190.81
C
ANISOU  481  CA  SER A  70    24260  29609  18632  -3322   -813
1755       C
ATOM    482  C   SER A  70     -13.042  -1.922   0.310  1.00198.59
C
ANISOU  482  C   SER A  70    24834  31123  19499  -3445  -1068
2392       C
ATOM    483  O   SER A  70     -13.044  -2.024  -0.922  1.00211.87
O
ANISOU  483  O   SER A  70    26671  32969  20862  -3938  -1270
2467       O
ATOM    484  CB  SER A  70     -12.591  -4.020   1.601  1.00199.27
C
ANISOU  484  CB  SER A  70    25335  30810  19566  -3716   -931
1728       C
ATOM    485  OG  SER A  70     -11.615  -4.797   2.273  1.00198.55
O
ANISOU  485  OG  SER A  70    25644  30230  19564  -3647   -707
1154       O
ATOM    486  N   TRP A  71     -13.933  -1.168   0.956  1.00198.98
N
ANISOU  486  N   TRP A  71    24356  31450  19798  -3012  -1057
2872       N
ATOM    487  CA  TRP A  71     -14.846  -0.298   0.214  1.00176.86
C
ANISOU  487  CA  TRP A  71    21128  29124  16945  -3026  -1259
3513       C
ATOM    488  C   TRP A  71     -14.116   0.726  -0.653  1.00166.62
C
ANISOU  488  C   TRP A  71    20006  27654  15646  -2879  -1197
3458       C
ATOM    489  O   TRP A  71     -14.473   0.928  -1.819  1.00201.50
O
ANISOU  489  O   TRP A  71    24360  32395  19807  -3265  -1446
```

```
3776       O
ATOM    490  CB  TRP A  71      -15.754   0.442   1.200  1.00169.73
C
ANISOU  490  CB  TRP A  71     19660  28440  16390  -2450  -1157
3979       C
ATOM    491  CG  TRP A  71      -16.809  -0.381   1.864  1.00156.50
C
ANISOU  491  CG  TRP A  71     17658  27113  14691  -2626  -1289
4246       C
ATOM    492  CD1 TRP A  71      -16.777  -0.880   3.134  1.00131.95
C
ANISOU  492  CD1 TRP A  71     14546  23809  11781  -2373  -1104
4010       C
ATOM    493  CD2 TRP A  71      -18.080  -0.755   1.319  1.00177.80
C
ANISOU  493  CD2 TRP A  71     19961  30440  17156  -3088  -1636
4833       C
ATOM    494  NE1 TRP A  71      -17.935  -1.565   3.403  1.00135.01
N
ANISOU  494  NE1 TRP A  71     14575  24650  12073  -2652  -1309
4393       N
ATOM    495  CE2 TRP A  71      -18.753  -1.501   2.306  1.00152.54
C
ANISOU  495  CE2 TRP A  71     16545  27387  14028  -3095  -1638
4906       C
ATOM    496  CE3 TRP A  71      -18.708  -0.541   0.089  1.00191.31
C
ANISOU  496  CE3 TRP A  71     21477  32621  18591  -3522  -1954
5315       C
ATOM    497  CZ2 TRP A  71      -20.024  -2.033   2.103  1.00165.75
C
ANISOU  497  CZ2 TRP A  71     17811  29657  15507  -3516  -1944
5440       C
ATOM    498  CZ3 TRP A  71      -19.969  -1.073  -0.113  1.00184.16
C
ANISOU  498  CZ3 TRP A  71     20163  32321  17488  -3951  -2266
5856       C
ATOM    499  CH2 TRP A  71      -20.613  -1.810   0.888  1.00178.64
C
ANISOU  499  CH2 TRP A  71     19252  31754  16867  -3945  -2258
5913       C
ATOM    500  N   VAL A  72      -13.094   1.387  -0.107  1.00140.94
N
ANISOU  500  N   VAL A  72     16976  23910  12666  -2346   -874
3071       N
ATOM    501  CA  VAL A  72      -12.270   2.285  -0.918  1.00126.21
C
ANISOU  501  CA  VAL A  72     15340  21830  10782  -2234   -799
2948       C
ATOM    502  C   VAL A  72      -11.509   1.538  -2.013  1.00126.03
C
ANISOU  502  C   VAL A  72     15817  21681  10390  -2849   -923
2565       C
ATOM    503  O   VAL A  72      -11.409   2.013  -3.151  1.00156.56
```

O
ANISOU 503 O VAL A 72 19751 25674 14062 -3083 -1059
2712 O
ATOM 504 CB VAL A 72 -11.317 3.092 -0.018 1.00121.98
C
ANISOU 504 CB VAL A 72 14954 20792 10603 -1561 -419
2593 C
ATOM 505 CG1 VAL A 72 -10.526 4.090 -0.850 1.00121.42
C
ANISOU 505 CG1 VAL A 72 15092 20524 10518 -1438 -346
2508 C
ATOM 506 CG2 VAL A 72 -12.100 3.804 1.072 1.00133.79
C
ANISOU 506 CG2 VAL A 72 15987 22400 12447 -978 -268
2960 C
ATOM 507 N MET A 73 -10.968 0.361 -1.693 1.00123.95
N
ANISOU 507 N MET A 73 15913 21159 10023 -3120 -864
2077 N
ATOM 508 CA MET A 73 -10.376 -0.506 -2.714 1.00128.69
C
ANISOU 508 CA MET A 73 16989 21656 10253 -3755 -968
1733 C
ATOM 509 C MET A 73 -11.362 -0.916 -3.806 1.00160.58
C
ANISOU 509 C MET A 73 20893 26223 13899 -4431 -1340
2159 C
ATOM 510 O MET A 73 -11.001 -0.955 -4.988 1.00158.56
O
ANISOU 510 O MET A 73 20919 25987 13339 -4869 -1452
2077 O
ATOM 511 CB MET A 73 -9.778 -1.746 -2.052 1.00129.86
C
ANISOU 511 CB MET A 73 17493 21452 10395 -3892 -823
1203 C
ATOM 512 CG MET A 73 -8.441 -1.496 -1.373 1.00137.32
C
ANISOU 512 CG MET A 73 18743 21823 11609 -3412 -473
665 C
ATOM 513 SD MET A 73 -7.083 -1.287 -2.541 1.00115.81
S
ANISOU 513 SD MET A 73 16553 18748 8700 -3610 -370
228 S
ATOM 514 CE MET A 73 -6.747 -2.997 -2.959 1.00116.48
C
ANISOU 514 CE MET A 73 17122 18674 8459 -4292 -401
-191 C
ATOM 515 N GLU A 74 -12.602 -1.238 -3.438 1.00172.83
N
ANISOU 515 N GLU A 74 22021 28216 15432 -4552 -1537
2618 N
ATOM 516 CA GLU A 74 -13.638 -1.465 -4.445 1.00173.69
C
ANISOU 516 CA GLU A 74 21915 28901 15179 -5167 -1913

3123 C
ATOM 517 C GLU A 74 -13.854 -0.252 -5.345 1.00167.92
C
ANISOU 517 C GLU A 74 20949 28429 14423 -5074 -2036
3562 C
ATOM 518 O GLU A 74 -13.976 -0.394 -6.567 1.00196.78
O
ANISOU 518 O GLU A 74 24735 32331 17701 -5659 -2276
3696 O
ATOM 519 CB GLU A 74 -14.951 -1.859 -3.769 1.00188.85
C
ANISOU 519 CB GLU A 74 23340 31271 17145 -5210 -2083
3597 C
ATOM 520 CG GLU A 74 -16.131 -1.940 -4.727 1.00195.15
C
ANISOU 520 CG GLU A 74 23807 32739 17601 -5791 -2487
4229 C
ATOM 521 CD GLU A 74 -17.411 -2.384 -4.048 1.00205.45
C
ANISOU 521 CD GLU A 74 24618 34501 18943 -5855 -2652
4695 C
ATOM 522 OE1 GLU A 74 -17.449 -3.523 -3.537 1.00214.77
O
ANISOU 522 OE1 GLU A 74 25991 35585 20028 -6135 -2645
4414 O
ATOM 523 OE2 GLU A 74 -18.379 -1.596 -4.026 1.00196.48
O
ANISOU 523 OE2 GLU A 74 22897 33820 17938 -5622 -2782
5356 O
ATOM 524 N MET A 75 -13.908 0.948 -4.765 1.00161.27
N
ANISOU 524 N MET A 75 19776 27530 13969 -4357 -1866
3794 N
ATOM 525 CA MET A 75 -13.953 2.166 -5.572 1.00157.53
C
ANISOU 525 CA MET A 75 19131 27210 13515 -4195 -1928
4159 C
ATOM 526 C MET A 75 -12.735 2.322 -6.478 1.00174.74
C
ANISOU 526 C MET A 75 21847 29031 15515 -4386 -1850
3696 C
ATOM 527 O MET A 75 -12.870 2.727 -7.638 1.00207.28
O
ANISOU 527 O MET A 75 25963 33405 19390 -4717 -2054
3964 O
ATOM 528 CB MET A 75 -14.088 3.390 -4.666 1.00154.75
C
ANISOU 528 CB MET A 75 18411 26747 13641 -3346 -1682
4396 C
ATOM 529 CG MET A 75 -14.172 4.703 -5.430 1.00161.01
C
ANISOU 529 CG MET A 75 19009 27676 14493 -3122 -1719
4808 C
ATOM 530 SD MET A 75 -14.408 6.142 -4.372 1.00163.12

```
S
ANISOU  530  SD  MET A  75    18866  27797  15316  -2136  -1397
5112       S
ATOM    531  CE  MET A  75     -12.731   6.422  -3.808  1.00182.87
C
ANISOU  531  CE  MET A  75    21953  29518  18010  -1727   -988
4284       C
ATOM    532  N   ALA A  76     -11.540   2.006  -5.976  1.00134.09
N
ANISOU  532  N   ALA A  76    17155  23311  10482  -4190  -1557
3019       N
ATOM    533  CA  ALA A  76     -10.346   2.032  -6.821  1.00132.44
C
ANISOU  533  CA  ALA A  76    17473  22756  10093  -4399  -1466
2549       C
ATOM    534  C   ALA A  76     -10.443   1.089  -8.018  1.00143.73
C
ANISOU  534  C   ALA A  76    19201  24393  11017  -5260  -1721
2487       C
ATOM    535  O   ALA A  76     -10.084   1.469  -9.139  1.00180.72
O
ANISOU  535  O   ALA A  76    24081  29117  15468  -5544  -1809
2494       O
ATOM    536  CB  ALA A  76      -9.111   1.695  -5.984  1.00128.39
C
ANISOU  536  CB  ALA A  76    17361  21630   9792  -4068  -1114
1861       C
ATOM    537  N   ALA A  77     -10.915  -0.140  -7.808  1.00136.64
N
ANISOU  537  N   ALA A  77    18366  23620   9930  -5704  -1834
2417       N
ATOM    538  CA  ALA A  77     -11.151  -1.044  -8.934  1.00140.19
C
ANISOU  538  CA  ALA A  77    19089  24306   9870  -6571  -2085
2407       C
ATOM    539  C   ALA A  77     -12.200  -0.498  -9.899  1.00145.37
C
ANISOU  539  C   ALA A  77    19365  25591  10279  -6927  -2452
3098       C
ATOM    540  O   ALA A  77     -12.000  -0.504 -11.120  1.00147.76
O
ANISOU  540  O   ALA A  77    19918  26008  10215  -7455  -2602
3097       O
ATOM    541  CB  ALA A  77     -11.564  -2.421  -8.414  1.00140.70
C
ANISOU  541  CB  ALA A  77    19257  24398   9804  -6948  -2130
2250       C
ATOM    542  N   ILE A  78     -13.330  -0.029  -9.368  1.00165.02
N
ANISOU  542  N   ILE A  78    21237  28503  12962  -6649  -2597
3711       N
ATOM    543  CA  ILE A  78     -14.359   0.604 -10.192  1.00152.45
C
ANISOU  543  CA  ILE A  78    19196  27531  11198  -6892  -2936
```

```
4449       C
ATOM    544  C   ILE A  78      -13.836   1.861 -10.878  1.00152.26
C
ANISOU  544  C   ILE A  78    19176  27422  11254  -6604  -2884
4559       C
ATOM    545  O   ILE A  78      -14.134   2.106 -12.053  1.00200.96
O
ANISOU  545  O   ILE A  78    25314  33947  17096  -7081  -3150
4890       O
ATOM    546  CB  ILE A  78      -15.614   0.895  -9.349  1.00154.41
C
ANISOU  546  CB  ILE A  78    18758  28203  11709  -6541  -3039
5076       C
ATOM    547  CG1 ILE A  78      -16.283  -0.417  -8.933  1.00155.79
C
ANISOU  547  CG1 ILE A  78    18917  28584  11692  -7011  -3180
5058       C
ATOM    548  CG2 ILE A  78      -16.592   1.765 -10.122  1.00159.47
C
ANISOU  548  CG2 ILE A  78    18875  29449  12266  -6632  -3340
5885       C
ATOM    549  CD1 ILE A  78      -16.761  -1.255 -10.101  1.00160.45
C
ANISOU  549  CD1 ILE A  78    19672  29589  11703  -7997  -3541
5211       C
ATOM    550  N   MET A  79      -13.043   2.670 -10.170  1.00147.98
N
ANISOU  550  N   MET A  79    18685  26412  11129  -5852  -2546
4285       N
ATOM    551  CA  MET A  79      -12.516   3.896 -10.767  1.00147.69
C
ANISOU  551  CA  MET A  79    18667  26262  11185  -5552  -2475
4376       C
ATOM    552  C   MET A  79      -11.712   3.625 -12.034  1.00148.22
C
ANISOU  552  C   MET A  79    19259  26215  10843  -6143  -2547
4035       C
ATOM    553  O   MET A  79      -11.803   4.387 -13.005  1.00150.81
O
ANISOU  553  O   MET A  79    19507  26768  11027  -6281  -2704
4366       O
ATOM    554  CB  MET A  79      -11.650   4.648  -9.755  1.00142.69
C
ANISOU  554  CB  MET A  79    18112  25079  11025  -4721  -2070
4020       C
ATOM    555  CG  MET A  79      -11.159   6.000 -10.253  1.00168.47
C
ANISOU  555  CG  MET A  79    21367  28216  14426  -4349  -1975
4145       C
ATOM    556  SD  MET A  79      -10.138   6.890  -9.063  1.00165.56
S
ANISOU  556  SD  MET A  79    21121  27208  14575  -3430  -1500
3724       S
ATOM    557  CE  MET A  79       -8.566   6.064  -9.299  1.00132.66
```

```
C
ANISOU  557  CE  MET A  79    17696  22489  10219  -3723  -1307
2822       C
ATOM    558  N   ALA A  80     -10.907   2.561 -12.047  1.00145.94
N
ANISOU  558  N   ALA A  80    19514  25567  10368  -6487  -2419
3382       N
ATOM    559  CA  ALA A  80     -10.220   2.181 -13.278  1.00183.93
C
ANISOU  559  CA  ALA A  80    24840  30291  14755  -7115  -2479
3061       C
ATOM    560  C   ALA A  80     -11.209   1.885 -14.404  1.00160.80
C
ANISOU  560  C   ALA A  80    21764  27981  11351  -7898  -2899
3569       C
ATOM    561  O   ALA A  80     -11.063   2.391 -15.523  1.00155.22
O
ANISOU  561  O   ALA A  80    21153  27434  10390  -8206  -3041
3724       O
ATOM    562  CB  ALA A  80      -9.318   0.973 -13.019  1.00169.39
C
ANISOU  562  CB  ALA A  80    23575  27976  12809  -7352  -2252
2317       C
ATOM    563  N   ILE A  81     -12.226   1.068 -14.122  1.00155.54
N
ANISOU  563  N   ILE A  81    20863  27686  10548  -8249  -3110
3846       N
ATOM    564  CA  ILE A  81     -13.299   0.821 -15.085  1.00161.54
C
ANISOU  564  CA  ILE A  81    21401  29108  10868  -8987  -3537
4416       C
ATOM    565  C   ILE A  81     -14.081   2.098 -15.386  1.00164.53
C
ANISOU  565  C   ILE A  81    21171  29955  11390  -8692  -3747
5196       C
ATOM    566  O   ILE A  81     -14.394   2.392 -16.546  1.00168.66
O
ANISOU  566  O   ILE A  81    21648  30868  11566  -9192  -4027
5563       O
ATOM    567  CB  ILE A  81     -14.220  -0.305 -14.580  1.00163.53
C
ANISOU  567  CB  ILE A  81    21504  29647  10982  -9372  -3699
4550       C
ATOM    568  CG1 ILE A  81     -13.403  -1.562 -14.274  1.00160.64
C
ANISOU  568  CG1 ILE A  81    21767  28772  10498  -9634  -3461
3774       C
ATOM    569  CG2 ILE A  81     -15.298  -0.612 -15.607  1.00169.99
C
ANISOU  569  CG2 ILE A  81    22077  31052  11461 -10136  -4092
5074       C
ATOM    570  CD1 ILE A  81     -12.575  -2.051 -15.441  1.00161.51
C
ANISOU  570  CD1 ILE A  81    22503  28606  10258 -10240  -3402
```

```
3296       C
ATOM    571  N   ALA A  82     -14.414   2.869 -14.346  1.00162.75
N
ANISOU  571  N   ALA A  82    20471  29694  11673  -7882  -3604
5471       N
ATOM    572  CA  ALA A  82     -15.186   4.097 -14.526  1.00165.73
C
ANISOU  572  CA  ALA A  82    20245  30480  12244  -7518  -3754
6236       C
ATOM    573  C   ALA A  82     -14.472   5.101 -15.422  1.00165.63
C
ANISOU  573  C   ALA A  82    20413  30323  12196  -7424  -3715
6222       C
ATOM    574  O   ALA A  82     -15.104   5.741 -16.271  1.00170.06
O
ANISOU  574  O   ALA A  82    20650  31358  12605  -7629  -3993
6849       O
ATOM    575  CB  ALA A  82     -15.496   4.728 -13.168  1.00163.33
C
ANISOU  575  CB  ALA A  82    19503  30036  12517  -6614  -3512
6412       C
ATOM    576  N   LEU A  83     -13.162   5.252 -15.258  1.00160.82
N
ANISOU  576  N   LEU A  83    20304  29080  11719  -7127  -3380
5536       N
ATOM    577  CA  LEU A  83     -12.406   6.236 -16.023  1.00189.50
C
ANISOU  577  CA  LEU A  83    24124  32530  15347  -6988  -3309
5481       C
ATOM    578  C   LEU A  83     -11.861   5.681 -17.334  1.00190.89
C
ANISOU  578  C   LEU A  83    24815  32731  14982  -7805  -3458
5188       C
ATOM    579  O   LEU A  83     -11.226   6.426 -18.089  1.00174.79
O
ANISOU  579  O   LEU A  83    22964  30570  12880  -7785  -3423
5135       O
ATOM    580  CB  LEU A  83     -11.249   6.789 -15.179  1.00156.44
C
ANISOU  580  CB  LEU A  83    20193  27661  11585  -6232  -2866
4922       C
ATOM    581  CG  LEU A  83     -11.523   7.923 -14.181  1.00153.02
C
ANISOU  581  CG  LEU A  83    19313  27127  11700  -5324  -2664
5241       C
ATOM    582  CD1 LEU A  83     -11.850   9.219 -14.913  1.00156.14
C
ANISOU  582  CD1 LEU A  83    19404  27776  12147  -5143  -2786
5835       C
ATOM    583  CD2 LEU A  83     -12.618   7.579 -13.179  1.00154.10
C
ANISOU  583  CD2 LEU A  83    18963  27537  12050  -5105  -2713
5607       C
ATOM    584  N   ALA A  84     -12.094   4.404 -17.625  1.00201.31
```

```
N
ANISOU  584  N   ALA A  84    26383  34199  15906  -8531  -3609
4995       N
ATOM    585  CA  ALA A  84     -11.756   3.816 -18.914  1.00199.00
C
ANISOU  585  CA  ALA A  84    26562  34000  15048  -9396  -3771
4783       C
ATOM    586  C   ALA A  84     -12.939   3.770 -19.871  1.00220.36
C
ANISOU  586  C   ALA A  84    28841  37238  17647  -9948  -4135
5385       C
ATOM    587  O   ALA A  84     -12.796   3.251 -20.983  1.00229.33
O
ANISOU  587  O   ALA A  84    30257  38324  18555 -10614  -4200
5176       O
ATOM    588  CB  ALA A  84     -11.198   2.403 -18.722  1.00191.25
C
ANISOU  588  CB  ALA A  84    26115  32606  13945  -9778  -3580
4052       C
ATOM    589  N   ASN A  85     -14.101   4.285 -19.458  1.00236.37
N
ANISOU  589  N   ASN A  85    30192  39762  19855  -9684  -4347
6127       N
ATOM    590  CA  ASN A  85     -15.341   4.206 -20.236  1.00245.20
C
ANISOU  590  CA  ASN A  85    30824  41425  20914 -10184  -4687
6742       C
ATOM    591  C   ASN A  85     -15.745   2.755 -20.491  1.00254.90
C
ANISOU  591  C   ASN A  85    32245  42688  21918 -10950  -4769
6481       C
ATOM    592  O   ASN A  85     -16.121   2.382 -21.604  1.00245.79
O
ANISOU  592  O   ASN A  85    31114  41727  20549 -11660  -4948
6585       O
ATOM    593  CB  ASN A  85     -15.232   4.979 -21.555  1.00222.91
C
ANISOU  593  CB  ASN A  85    27979  38731  17986 -10445  -4826
6974       C
ATOM    594  CG  ASN A  85     -14.946   6.453 -21.347  1.00225.66
C
ANISOU  594  CG  ASN A  85    28099  39070  18572  -9700  -4755
7314       C
ATOM    595  OD1 ASN A  85     -15.268   7.017 -20.302  1.00226.04
O
ANISOU  595  OD1 ASN A  85    27782  39196  18909  -9014  -4679
7644       O
ATOM    596  ND2 ASN A  85     -14.341   7.086 -22.346  1.00184.39
N
ANISOU  596  ND2 ASN A  85    23091  33736  13235  -9834  -4760
7245       N
ATOM    597  N   GLY A  86     -15.663   1.927 -19.450  1.00263.54
N
ANISOU  597  N   GLY A  86    33489  43584  23061 -10816  -4622
```

```
6147        N
ATOM    598  CA   GLY A  86      -15.944   0.510 -19.583  1.00258.45
C
ANISOU  598  CA   GLY A  86    33091  42888  22220 -11493  -4645
5847        C
ATOM    599  C    GLY A  86      -14.859  -0.255 -20.312  1.00254.19
C
ANISOU  599  C    GLY A  86    33305  41824  21452 -11969  -4433
5099        C
ATOM    600  O    GLY A  86      -13.805  -0.556 -19.744  1.00246.24
O
ANISOU  600  O    GLY A  86    32775  40290  20496 -11674  -4127
4471        O
ATOM    601  N    ASP A  87      -15.111  -0.570 -21.582  1.00259.96
N
ANISOU  601  N    ASP A  87    34136  42690  21947 -12703  -4572
5177        N
ATOM    602  CA   ASP A  87      -14.181  -1.317 -22.417  1.00255.40
C
ANISOU  602  CA   ASP A  87    34244  41637  21158 -13213  -4358
4540        C
ATOM    603  C    ASP A  87      -13.342  -0.412 -23.312  1.00251.12
C
ANISOU  603  C    ASP A  87    33907  40930  20577 -13128  -4295
4429        C
ATOM    604  O    ASP A  87      -12.616  -0.911 -24.178  1.00245.63
O
ANISOU  604  O    ASP A  87    33732  39884  19712 -13573  -4127
3980        O
ATOM    605  CB   ASP A  87      -14.939  -2.353 -23.255  1.00238.89
C
ANISOU  605  CB   ASP A  87    32209  39754  18806 -14133  -4501
4659        C
ATOM    606  CG   ASP A  87      -15.892  -1.721 -24.259  1.00219.47
C
ANISOU  606  CG   ASP A  87    29269  37888  16231 -14539  -4853
5350        C
ATOM    607  OD1 ASP A  87      -16.473  -2.472 -25.072  1.00205.00
O
ANISOU  607  OD1 ASP A  87    27495  36242  14155 -15340  -4968
5469        O
ATOM    608  OD2 ASP A  87      -16.084  -0.487 -24.227  1.00214.24
O
ANISOU  608  OD2 ASP A  87    28166  37507  15728 -14063  -5002
5796        O
ATOM    609  N    GLY A  88      -13.435   0.905 -23.123  1.00242.50
N
ANISOU  609  N    GLY A  88    32416  40074  19649 -12558  -4408
4848        N
ATOM    610  CA   GLY A  88      -12.686   1.829 -23.960  1.00247.76
C
ANISOU  610  CA   GLY A  88    33248  40610  20280 -12466  -4363
4788        C
ATOM    611  C    GLY A  88      -11.183   1.684 -23.814  1.00248.59
```

```
C
ANISOU  611  C   GLY A  88    34001  40041  20411 -12212  -3976
4004       C
ATOM    612  O   GLY A  88     -10.442   1.801 -24.793  1.00239.14
O
ANISOU  612  O   GLY A  88    33165  38605  19091 -12480  -3873
3723       O
ATOM    613  N   ARG A  89     -10.711   1.429 -22.595  1.00254.49
N
ANISOU  613  N   ARG A  89    34887  40475  21332 -11692  -3745
3651       N
ATOM    614  CA  ARG A  89      -9.284   1.285 -22.327  1.00248.55
C
ANISOU  614  CA  ARG A  89    34701  39076  20659 -11388  -3353
2914       C
ATOM    615  C   ARG A  89      -9.011  -0.067 -21.680  1.00236.53
C
ANISOU  615  C   ARG A  89    33524  37176  19172 -11514  -3120
2387       C
ATOM    616  O   ARG A  89      -9.530  -0.345 -20.584  1.00206.14
O
ANISOU  616  O   ARG A  89    29442  33449  15433 -11244  -3155
2505       O
ATOM    617  CB  ARG A  89      -8.805   2.434 -21.436  1.00222.82
C
ANISOU  617  CB  ARG A  89    31299  35749  17612 -10553  -3247
2975       C
ATOM    618  CG  ARG A  89      -7.308   2.668 -21.424  1.00213.58
C
ANISOU  618  CG  ARG A  89    30642  33983  16527 -10235  -2880
2323       C
ATOM    619  CD  ARG A  89      -7.000   4.009 -20.777  1.00210.99
C
ANISOU  619  CD  ARG A  89    30118  33695  16353  -9504  -2830
2535       C
ATOM    620  NE  ARG A  89      -7.387   4.052 -19.372  1.00217.14
N
ANISOU  620  NE  ARG A  89    30567  34457  17479  -8901  -2747
2650       N
ATOM    621  CZ  ARG A  89      -7.773   5.154 -18.737  1.00228.83
C
ANISOU  621  CZ  ARG A  89    31544  36022  19380  -8202  -2741
3079       C
ATOM    622  NH1 ARG A  89      -7.663   6.336 -19.326  1.00221.89
N
ANISOU  622  NH1 ARG A  89    30521  35239  18547  -8005  -2799
3391       N
ATOM    623  NH2 ARG A  89      -8.127   5.090 -17.461  1.00253.01
N
ANISOU  623  NH2 ARG A  89    34302  38992  22838  -7654  -2618
3127       N
ATOM    624  N   PRO A  90      -8.219  -0.925 -22.316  1.00251.28
N
ANISOU  624  N   PRO A  90    35930  38578  20967 -11899  -2865
```

```
1834        N
ATOM    625  CA  PRO A  90      -8.048  -2.307 -21.837  1.00237.50
C
ANISOU  625  CA  PRO A  90    34507  36476  19256 -12086  -2640
1398        C
ATOM    626  C   PRO A  90      -7.259  -2.430 -20.540  1.00209.26
C
ANISOU  626  C   PRO A  90    31085  32473  15953 -11418  -2342
954        C
ATOM    627  O   PRO A  90      -7.624  -3.262 -19.696  1.00208.81
O
ANISOU  627  O   PRO A  90    31006  32377  15956 -11403  -2306
875        O
ATOM    628  CB  PRO A  90      -7.302  -2.983 -22.999  1.00228.22
C
ANISOU  628  CB  PRO A  90    33853  34901  17960 -12600  -2404
994        C
ATOM    629  CG  PRO A  90      -7.761  -2.240 -24.203  1.00229.52
C
ANISOU  629  CG  PRO A  90    33826  35472  17911 -13000  -2681
1428        C
ATOM    630  CD  PRO A  90      -7.870  -0.800 -23.743  1.00229.57
C
ANISOU  630  CD  PRO A  90    33414  35773  18039 -12389  -2864
1787        C
ATOM    631  N   PRO A  91      -6.179  -1.660 -20.316  1.00198.76
N
ANISOU  631  N   PRO A  91    29912  30812  14798 -10868  -2116
654        N
ATOM    632  CA  PRO A  91      -5.320  -1.997 -19.163  1.00219.13
C
ANISOU  632  CA  PRO A  91    32704  32905  17652 -10318  -1778
152        C
ATOM    633  C   PRO A  91      -5.956  -1.737 -17.806  1.00224.77
C
ANISOU  633  C   PRO A  91    33053  33886  18462  -9870  -1894
401        C
ATOM    634  O   PRO A  91      -5.559  -2.377 -16.824  1.00179.75
O
ANISOU  634  O   PRO A  91    27499  27850  12946  -9590  -1664
34       O
ATOM    635  CB  PRO A  91      -4.079  -1.112 -19.370  1.00195.59
C
ANISOU  635  CB  PRO A  91    29927  29566  14822  -9882  -1537
-156        C
ATOM    636  CG  PRO A  91      -4.538  -0.014 -20.249  1.00194.15
C
ANISOU  636  CG  PRO A  91    29499  29823  14445 -10033  -1827
326        C
ATOM    637  CD  PRO A  91      -5.522  -0.652 -21.169  1.00196.89
C
ANISOU  637  CD  PRO A  91    29755  30519  14533 -10780  -2090
652        C
ATOM    638  N   ASP A  92      -6.916  -0.816 -17.712  1.00232.34
```

```
N
ANISOU  638  N   ASP A  92    33522  35429  19326  -9767  -2225
1041       N
ATOM    639  CA  ASP A  92      -7.637  -0.619 -16.457  1.00179.86
C
ANISOU  639  CA  ASP A  92    26462  29054  12822  -9339  -2321
1363       C
ATOM    640  C   ASP A  92      -8.346  -1.892 -16.005  1.00181.46
C
ANISOU  640  C   ASP A  92    26655  29350  12942  -9706  -2395
1344       C
ATOM    641  O   ASP A  92      -8.364  -2.212 -14.811  1.00227.33
O
ANISOU  641  O   ASP A  92    32358  34989  19028  -9287  -2252
1212       O
ATOM    642  CB  ASP A  92      -8.631   0.534 -16.589  1.00182.00
C
ANISOU  642  CB  ASP A  92    26098  29857  13197  -9115  -2614
2133       C
ATOM    643  CG  ASP A  92      -7.965   1.832 -16.996  1.00198.17
C
ANISOU  643  CG  ASP A  92    28121  31765  15409  -8696  -2518
2174       C
ATOM    644  OD1 ASP A  92      -7.669   2.652 -16.100  1.00155.92
O
ANISOU  644  OD1 ASP A  92    22535  26175  10534  -7901  -2314
2175       O
ATOM    645  OD2 ASP A  92      -7.740   2.032 -18.208  1.00231.15
O
ANISOU  645  OD2 ASP A  92    32529  36070  19227  -9177  -2642
2203       O
ATOM    646  N   TRP A  93      -8.933  -2.630 -16.949  1.00188.39
N
ANISOU  646  N   TRP A  93    27572  30380  13629 -10405  -2559
1467       N
ATOM    647  CA  TRP A  93      -9.503  -3.940 -16.641  1.00192.43
C
ANISOU  647  CA  TRP A  93    28143  30879  14091 -10803  -2575
1385       C
ATOM    648  C   TRP A  93      -8.448  -4.930 -16.153  1.00203.95
C
ANISOU  648  C   TRP A  93    30128  31658  15705 -10688  -2169
663       C
ATOM    649  O   TRP A  93      -8.708  -5.709 -15.229  1.00247.62
O
ANISOU  649  O   TRP A  93    35652  37115  21316 -10621  -2113
553       O
ATOM    650  CB  TRP A  93     -10.234  -4.485 -17.868  1.00193.37
C
ANISOU  650  CB  TRP A  93    28260  31257  13955 -11612  -2786
1640       C
ATOM    651  CG  TRP A  93     -10.956  -5.778 -17.630  1.00186.04
C
ANISOU  651  CG  TRP A  93    27366  30381  12938 -12082  -2830
```

```
1644       C
ATOM    652  CD1 TRP A  93     -12.257  -5.927 -17.244  1.00189.39
C
ANISOU  652  CD1 TRP A  93    27312  31342  13305 -12254  -3137
2176       C
ATOM    653  CD2 TRP A  93     -10.430  -7.102 -17.785  1.00178.96
C
ANISOU  653  CD2 TRP A  93    27003  28981  12013 -12436  -2541
1123       C
ATOM    654  NE1 TRP A  93     -12.570  -7.261 -17.137  1.00196.92
N
ANISOU  654  NE1 TRP A  93    28490  32160  14171 -12721  -3069
1993       N
ATOM    655  CE2 TRP A  93     -11.466  -8.003 -17.465  1.00195.23
C
ANISOU  655  CE2 TRP A  93    28906  31302  13968 -12836  -2700
1356       C
ATOM    656  CE3 TRP A  93      -9.184  -7.613 -18.157  1.00167.89
C
ANISOU  656  CE3 TRP A  93    26177  26927  10685 -12426  -2145
516       C
ATOM    657  CZ2 TRP A  93     -11.293  -9.385 -17.507  1.00198.90
C
ANISOU  657  CZ2 TRP A  93    29807  31391  14375 -13235  -2473
996       C
ATOM    658  CZ3 TRP A  93      -9.014  -8.986 -18.199  1.00177.80
C
ANISOU  658  CZ3 TRP A  93    27834  27813  11910 -12786  -1910
188       C
ATOM    659  CH2 TRP A  93     -10.062  -9.856 -17.875  1.00199.75
C
ANISOU  659  CH2 TRP A  93    30481  30856  14558 -13192  -2073
423       C
ATOM    660  N   GLN A  94      -7.262  -4.933 -16.765  1.00179.34
N
ANISOU  660  N   GLN A  94    27445  28037  12658 -10650  -1876
193       N
ATOM    661  CA  GLN A  94      -6.174  -5.753 -16.235  1.00187.16
C
ANISOU  661  CA  GLN A  94    28862  28363  13887 -10404  -1458
-439       C
ATOM    662  C   GLN A  94      -5.803  -5.350 -14.808  1.00159.98
C
ANISOU  662  C   GLN A  94    25293  24791  10702  -9683  -1329
-591       C
ATOM    663  O   GLN A  94      -5.557  -6.210 -13.955  1.00141.48
O
ANISOU  663  O   GLN A  94    23091  22134   8529  -9532  -1129
-895       O
ATOM    664  CB  GLN A  94      -4.946  -5.651 -17.142  1.00191.10
C
ANISOU  664  CB  GLN A  94    29757  28387  14466 -10398  -1166
-830       C
ATOM    665  CG  GLN A  94      -5.176  -5.939 -18.621  1.00181.97
```

```
C
ANISOU  665  CG  GLN A  94    28765  27332  13043 -11093  -1252
-702       C
ATOM    666  CD  GLN A  94      -5.735  -7.323 -18.883  1.00189.60
C
ANISOU  666  CD  GLN A  94    29918  28265  13858 -11696  -1232
-717       C
ATOM    667  OE1 GLN A  94      -6.631  -7.494 -19.709  1.00192.74
O
ANISOU  667  OE1 GLN A  94    30222  29044  13965 -12320  -1484
-364       O
ATOM    668  NE2 GLN A  94      -5.208  -8.319 -18.180  1.00211.80
N
ANISOU  668  NE2 GLN A  94    32991  30616  16868 -11516   -925
-1107       N
ATOM    669  N   ASP A  95      -5.715  -4.047 -14.540  1.00146.17
N
ANISOU  669  N   ASP A  95    23297  23257   8985  -9230  -1412
-383       N
ATOM    670  CA  ASP A  95      -5.545  -3.575 -13.167  1.00137.03
C
ANISOU  670  CA  ASP A  95    21916  22009   8142  -8516  -1288
-417       C
ATOM    671  C   ASP A  95      -6.708  -3.980 -12.265  1.00137.97
C
ANISOU  671  C   ASP A  95    21606  22441   8375  -8462  -1468
-50       C
ATOM    672  O   ASP A  95      -6.498  -4.424 -11.131  1.00134.96
O
ANISOU  672  O   ASP A  95    21199  21781   8297  -8072  -1274
-276       O
ATOM    673  CB  ASP A  95      -5.369  -2.056 -13.147  1.00135.43
C
ANISOU  673  CB  ASP A  95    21372  21874   8212  -7932  -1294
-147       C
ATOM    674  CG  ASP A  95      -4.035  -1.616 -13.712  1.00137.97
C
ANISOU  674  CG  ASP A  95    22099  21785   8538  -7807  -1041
-584       C
ATOM    675  OD1 ASP A  95      -3.936  -0.454 -14.159  1.00164.54
O
ANISOU  675  OD1 ASP A  95    25292  25271  11956  -7588  -1105
-346       O
ATOM    676  OD2 ASP A  95      -3.086  -2.428 -13.701  1.00131.60
O
ANISOU  676  OD2 ASP A  95    21777  20535   7691  -7918   -769
-1153       O
ATOM    677  N   PHE A  96      -7.940  -3.823 -12.752  1.00142.27
N
ANISOU  677  N   PHE A  96    21795  23584   8677  -8853  -1842
538       N
ATOM    678  CA  PHE A  96      -9.125  -4.194 -11.979  1.00143.72
C
ANISOU  678  CA  PHE A  96    21540  24128   8937  -8850  -2037
```

```
941       C
ATOM    679  C   PHE A  96      -9.116  -5.656 -11.534  1.00158.36
C
ANISOU  679  C   PHE A  96    23723  25774  10671  -9205  -1943
577       C
ATOM    680  O   PHE A  96      -9.396  -5.952 -10.366  1.00162.12
O
ANISOU  680  O   PHE A  96    23972  26181  11444  -8831  -1866
586       O
ATOM    681  CB  PHE A  96     -10.371  -3.858 -12.812  1.00149.07
C
ANISOU  681  CB  PHE A  96    21838  25506   9295  -9340  -2467
1628       C
ATOM    682  CG  PHE A  96     -11.621  -4.602 -12.416  1.00152.11
C
ANISOU  682  CG  PHE A  96    21913  26328   9554  -9684  -2721
2008       C
ATOM    683  CD1 PHE A  96     -12.567  -3.997 -11.605  1.00152.35
C
ANISOU  683  CD1 PHE A  96    21281  26711   9894  -9216  -2849
2556       C
ATOM    684  CD2 PHE A  96     -11.899  -5.862 -12.932  1.00155.23
C
ANISOU  684  CD2 PHE A  96    22633  26747   9600 -10455  -2806
1847       C
ATOM    685  CE1 PHE A  96     -13.730  -4.658 -11.258  1.00155.33
C
ANISOU  685  CE1 PHE A  96    21348  27519  10152  -9538  -3088
2931       C
ATOM    686  CE2 PHE A  96     -13.061  -6.528 -12.589  1.00158.22
C
ANISOU  686  CE2 PHE A  96    22699  27502   9917 -10764  -3026
2205       C
ATOM    687  CZ  PHE A  96     -13.981  -5.923 -11.755  1.00158.25
C
ANISOU  687  CZ  PHE A  96    22081  27981  10067 -10369  -3219
2769       C
ATOM    688  N   VAL A  97      -8.805  -6.589 -12.436  1.00150.04
N
ANISOU  688  N   VAL A  97    23166  24543   9299  -9864  -1903
263       N
ATOM    689  CA  VAL A  97      -8.641  -7.980 -12.009  1.00175.10
C
ANISOU  689  CA  VAL A  97    26648  27337  12547 -10064  -1706
-120       C
ATOM    690  C   VAL A  97      -7.447  -8.139 -11.067  1.00158.10
C
ANISOU  690  C   VAL A  97    24774  24589  10707  -9483  -1315
-682       C
ATOM    691  O   VAL A  97      -7.496  -8.926 -10.115  1.00172.75
O
ANISOU  691  O   VAL A  97    26677  26274  12686  -9372  -1205
-858       O
ATOM    692  CB  VAL A  97      -8.560  -8.930 -13.221  1.00149.85
```

```
C
ANISOU  692  CB  VAL A  97    23819  23923   9193 -10744  -1636
-274       C
ATOM    693  CG1 VAL A  97      -9.835  -8.837 -14.048  1.00155.38
C
ANISOU  693  CG1 VAL A  97    24206  25238   9592 -11348  -2030
306       C
ATOM    694  CG2 VAL A  97      -7.342  -8.651 -14.074  1.00148.80
C
ANISOU  694  CG2 VAL A  97    24063  23334   9140 -10663  -1361
-647       C
ATOM    695  N   GLY A  98      -6.365  -7.398 -11.309  1.00137.73
N
ANISOU  695  N   GLY A  98    22364  21689   8278  -9106  -1099
-953       N
ATOM    696  CA  GLY A  98      -5.252  -7.381 -10.368  1.00142.90
C
ANISOU  696  CA  GLY A  98    23196  21827   9272  -8496   -742
-1418       C
ATOM    697  C   GLY A  98      -5.613  -6.903  -8.972  1.00162.26
C
ANISOU  697  C   GLY A  98    25174  24358  12118  -7832   -740
-1214       C
ATOM    698  O   GLY A  98      -5.194  -7.496  -7.975  1.00126.94
O
ANISOU  698  O   GLY A  98    20787  19544   7902  -7528   -519
-1509       O
ATOM    699  N   ILE A  99      -6.381  -5.816  -8.881  1.00153.25
N
ANISOU  699  N   ILE A  99    23502  23633  11094  -7559   -960
-688       N
ATOM    700  CA  ILE A  99      -6.861  -5.324  -7.590  1.00128.08
C
ANISOU  700  CA  ILE A  99    19811  20534   8320  -6923   -950
-430       C
ATOM    701  C   ILE A  99      -7.713  -6.365  -6.867  1.00129.28
C
ANISOU  701  C   ILE A  99    19836  20841   8444  -7136  -1050
-323       C
ATOM    702  O   ILE A  99      -7.547  -6.589  -5.662  1.00126.31
O
ANISOU  702  O   ILE A  99    19357  20241   8396  -6687   -879
-467       O
ATOM    703  CB  ILE A  99      -7.623  -3.999  -7.778  1.00129.48
C
ANISOU  703  CB  ILE A  99    19458  21151   8586  -6659  -1165
158       C
ATOM    704  CG1 ILE A  99      -6.673  -2.905  -8.271  1.00127.57
C
ANISOU  704  CG1 ILE A  99    19338  20682   8449  -6327  -1016
15       C
ATOM    705  CG2 ILE A  99      -8.300  -3.575  -6.490  1.00128.03
C
ANISOU  705  CG2 ILE A  99    18751  21107   8787  -6078  -1160
```

```
470       C
ATOM    706  CD1 ILE A  99      -7.374  -1.689  -8.827  1.00129.95
C
ANISOU  706  CD1 ILE A  99    19226  21414   8734  -6232  -1243
587       C
ATOM    707  N   ILE A 100      -8.634  -7.013  -7.580  1.00133.75
N
ANISOU  707  N   ILE A 100    20411  21801   8608  -7842  -1334
-63       N
ATOM    708  CA  ILE A 100      -9.400  -8.116  -6.995  1.00135.23
C
ANISOU  708  CA  ILE A 100    20547  22120   8713  -8140  -1429
3       C
ATOM    709  C   ILE A 100      -8.498  -9.261  -6.540  1.00132.96
C
ANISOU  709  C   ILE A 100    20776  21270   8473  -8184  -1127
-609       C
ATOM    710  O   ILE A 100      -8.700  -9.828  -5.459  1.00131.46
O
ANISOU  710  O   ILE A 100    20476  20978   8494  -7967  -1051
-663       O
ATOM    711  CB  ILE A 100     -10.466  -8.609  -7.991  1.00145.94
C
ANISOU  711  CB  ILE A 100    21882  24000   9568  -8979  -1790
370       C
ATOM    712  CG1 ILE A 100     -11.535  -7.541  -8.205  1.00149.43
C
ANISOU  712  CG1 ILE A 100    21693  25057  10025  -8873  -2105
1076       C
ATOM    713  CG2 ILE A 100     -11.101  -9.909  -7.509  1.00142.52
C
ANISOU  713  CG2 ILE A 100    21530  23632   8989  -9395  -1859
341       C
ATOM    714  CD1 ILE A 100     -12.443  -7.838  -9.368  1.00149.00
C
ANISOU  714  CD1 ILE A 100    21620  25540   9451  -9710  -2474
1462       C
ATOM    715  N   CYS A 101      -7.494  -9.619  -7.342  1.00132.84
N
ANISOU  715  N   CYS A 101    21324  20880   8271  -8454   -938
-1065       N
ATOM    716  CA  CYS A 101      -6.529 -10.634  -6.916  1.00144.88
C
ANISOU  716  CA  CYS A 101    23335  21827   9887  -8420   -605
-1642       C
ATOM    717  C   CYS A 101      -5.792 -10.262  -5.630  1.00176.88
C
ANISOU  717  C   CYS A 101    27226  25518  14462  -7597   -336
-1841       C
ATOM    718  O   CYS A 101      -5.593 -11.119  -4.762  1.00206.37
O
ANISOU  718  O   CYS A 101    31076  28980  18356  -7483   -175
-2071       O
ATOM    719  CB  CYS A 101      -5.525 -10.913  -8.035  1.00131.51
```

```
C
ANISOU  719  CB  CYS A 101    22091  19692   8183  -8623   -374
-1986       C
ATOM    720  SG  CYS A 101      -6.240 -11.699  -9.489  1.00151.69
S
ANISOU  720  SG  CYS A 101    24815  22423  10395  -9495   -542
-1779       S
ATOM    721  N   LEU A 102      -5.373  -9.003  -5.484  1.00160.03
N
ANISOU  721  N   LEU A 102    24841  23374  12589  -7035   -281
-1755       N
ATOM    722  CA  LEU A 102      -4.817  -8.560  -4.204  1.00139.38
C
ANISOU  722  CA  LEU A 102    22020  20490  10449  -6278    -62
-1877       C
ATOM    723  C   LEU A 102      -5.813  -8.704  -3.058  1.00118.45
C
ANISOU  723  C   LEU A 102    18922  18095   7988  -6063   -188
-1557       C
ATOM    724  O   LEU A 102      -5.465  -9.194  -1.978  1.00115.89
O
ANISOU  724  O   LEU A 102    18617  17494   7923  -5749     -5
-1771       O
ATOM    725  CB  LEU A 102      -4.352  -7.106  -4.307  1.00157.23
C
ANISOU  725  CB  LEU A 102    24073  22754  12913  -5772    -13
-1780       C
ATOM    726  CG  LEU A 102      -4.002  -6.423  -2.978  1.00112.51
C
ANISOU  726  CG  LEU A 102    18114  16919   7714  -5003    163
-1796       C
ATOM    727  CD1 LEU A 102      -2.879  -7.155  -2.260  1.00109.39
C
ANISOU  727  CD1 LEU A 102    18043  16000   7518  -4785    479
-2303       C
ATOM    728  CD2 LEU A 102      -3.649  -4.956  -3.174  1.00111.09
C
ANISOU  728  CD2 LEU A 102    17748  16769   7691  -4570    196
-1666       C
ATOM    729  N   LEU A 103      -7.057  -8.275  -3.272  1.00121.29
N
ANISOU  729  N   LEU A 103    18865  18995   8226  -6226   -496
-1025       N
ATOM    730  CA  LEU A 103      -8.107  -8.480  -2.277  1.00121.77
C
ANISOU  730  CA  LEU A 103    18496  19346   8424  -6094   -628
-689       C
ATOM    731  C   LEU A 103      -8.355  -9.956  -1.971  1.00122.96
C
ANISOU  731  C   LEU A 103    18892  19405   8424  -6527   -631
-870       C
ATOM    732  O   LEU A 103      -8.518 -10.334  -0.806  1.00121.22
O
ANISOU  732  O   LEU A 103    18512  19100   8445  -6237   -551
```

```
-892       O
ATOM    733  CB  LEU A 103      -9.397  -7.803  -2.742  1.00125.32
C
ANISOU  733  CB  LEU A 103    18467  20420   8730  -6261   -965
-57       C
ATOM    734  CG  LEU A 103      -9.340  -6.272  -2.747  1.00124.14
C
ANISOU  734  CG  LEU A 103    17969  20381   8818  -5709   -949
210       C
ATOM    735  CD1 LEU A 103     -10.542  -5.677  -3.461  1.00128.37
C
ANISOU  735  CD1 LEU A 103    18086  21528   9160  -5962  -1284
841       C
ATOM    736  CD2 LEU A 103      -9.225  -5.724  -1.338  1.00120.63
C
ANISOU  736  CD2 LEU A 103    17222  19779   8832  -4961   -751
213       C
ATOM    737  N   VAL A 104      -8.395 -10.804  -2.999  1.00126.10
N
ANISOU  737  N   VAL A 104    19690  19811   8412  -7235   -715
-999       N
ATOM    738  CA  VAL A 104      -8.507 -12.249  -2.785  1.00127.39
C
ANISOU  738  CA  VAL A 104    20179  19809   8414  -7674   -674
-1228       C
ATOM    739  C   VAL A 104      -7.325 -12.803  -1.991  1.00123.62
C
ANISOU  739  C   VAL A 104    20033  18713   8225  -7296   -304
-1752       C
ATOM    740  O   VAL A 104      -7.506 -13.572  -1.039  1.00122.83
O
ANISOU  740  O   VAL A 104    19906  18500   8263  -7217   -241
-1818       O
ATOM    741  CB  VAL A 104      -8.657 -12.973  -4.135  1.00131.65
C
ANISOU  741  CB  VAL A 104    21157  20427   8438  -8521   -792
-1308       C
ATOM    742  CG1 VAL A 104      -8.440 -14.472  -3.968  1.00132.61
C
ANISOU  742  CG1 VAL A 104    21761  20209   8417  -8929   -642
-1676       C
ATOM    743  CG2 VAL A 104     -10.026 -12.690  -4.739  1.00140.36
C
ANISOU  743  CG2 VAL A 104    21889  22209   9232  -8983  -1203
-728       C
ATOM    744  N   ILE A 105      -6.102 -12.428  -2.367  1.00121.42
N
ANISOU  744  N   ILE A 105    20054  18039   8040  -7062    -57
-2113       N
ATOM    745  CA  ILE A 105      -4.922 -12.852  -1.610  1.00129.39
C
ANISOU  745  CA  ILE A 105    21329  18484   9349  -6657    297
-2570       C
ATOM    746  C   ILE A 105      -4.945 -12.317  -0.180  1.00128.59
```

```
C
ANISOU  746  C   ILE A 105    20795  18378   9683  -5964    357
-2452       C
ATOM    747  O   ILE A 105      -4.679 -13.054   0.778  1.00112.73
O
ANISOU  747  O   ILE A 105    18852  16110   7870  -5788    511
-2636       O
ATOM    748  CB  ILE A 105      -3.637 -12.435  -2.348  1.00119.74
C
ANISOU  748  CB  ILE A 105    20462  16897   8135  -6540    534
-2928       C
ATOM    749  CG1 ILE A 105      -3.479 -13.249  -3.633  1.00137.22
C
ANISOU  749  CG1 ILE A 105    23082  18956  10102  -7132    567
-3053       C
ATOM    750  CG2 ILE A 105      -2.419 -12.617  -1.458  1.00150.81
C
ANISOU  750  CG2 ILE A 105    24545  20317  12442  -6007    882
-3314       C
ATOM    751  CD1 ILE A 105      -3.229 -14.727  -3.394  1.00143.66
C
ANISOU  751  CD1 ILE A 105    24155  19367  11062  -7278    768
-3243       C
ATOM    752  N   ASN A 106      -5.264 -11.034  -0.012  1.00120.17
N
ANISOU  752  N   ASN A 106    19297  17591   8771  -5568    248
-2141       N
ATOM    753  CA  ASN A 106      -5.368 -10.461   1.329  1.00110.29
C
ANISOU  753  CA  ASN A 106    17640  16359   7907  -4937    311
-2011       C
ATOM    754  C   ASN A 106      -6.427 -11.158   2.177  1.00111.48
C
ANISOU  754  C   ASN A 106    17530  16752   8074  -5044    169
-1770       C
ATOM    755  O   ASN A 106      -6.175 -11.510   3.334  1.00109.13
O
ANISOU  755  O   ASN A 106    17177  16248   8037  -4714    316
-1901       O
ATOM    756  CB  ASN A 106      -5.664  -8.964   1.235  1.00110.06
C
ANISOU  756  CB  ASN A 106    17214  16608   7997  -4559    218
-1681       C
ATOM    757  CG  ASN A 106      -5.927  -8.334   2.591  1.00107.32
C
ANISOU  757  CG  ASN A 106    16444  16317   8015  -3950    282
-1509       C
ATOM    758  OD1 ASN A 106      -7.077  -8.137   2.984  1.00108.91
O
ANISOU  758  OD1 ASN A 106    16235  16912   8234  -3929    101
-1097       O
ATOM    759  ND2 ASN A 106      -4.858  -8.011   3.311  1.00103.63
N
ANISOU  759  ND2 ASN A 106    16076  15466   7834  -3464    548
```

```
-1816       N
ATOM    760  N   SER A 107      -7.617 -11.376   1.616  1.00115.27
N
ANISOU  760  N   SER A 107    17845  17685   8268  -5524   -123
-1405       N
ATOM    761  CA  SER A 107      -8.663 -12.110   2.326  1.00116.85
C
ANISOU  761  CA  SER A 107    17812  18143   8442  -5702   -274
-1165       C
ATOM    762  C   SER A 107      -8.258 -13.539   2.673  1.00116.72
C
ANISOU  762  C   SER A 107    18206  17766   8377  -5970   -133
-1531       C
ATOM    763  O   SER A 107      -8.505 -14.003   3.792  1.00155.03
O
ANISOU  763  O   SER A 107    22905  22579  13419  -5772    -89
-1514       O
ATOM    764  CB  SER A 107      -9.948 -12.113   1.499  1.00133.49
C
ANISOU  764  CB  SER A 107    19698  20817  10204  -6245   -624
-705       C
ATOM    765  OG  SER A 107     -10.388 -10.791   1.245  1.00174.10
O
ANISOU  765  OG  SER A 107    24418  26307  15424  -5960   -750
-314       O
ATOM    766  N   THR A 108      -7.635 -14.254   1.736  1.00118.08
N
ANISOU  766  N   THR A 108    18909  17659   8296  -6418    -43
-1860       N
ATOM    767  CA  THR A 108      -7.220 -15.629   2.009  1.00118.32
C
ANISOU  767  CA  THR A 108    19367  17304   8284  -6672    126
-2209       C
ATOM    768  C   THR A 108      -6.234 -15.716   3.171  1.00133.77
C
ANISOU  768  C   THR A 108    21358  18815  10653  -6069    424
-2505       C
ATOM    769  O   THR A 108      -6.424 -16.514   4.096  1.00133.38
O
ANISOU  769  O   THR A 108    21291  18666  10723  -6030    469
-2539       O
ATOM    770  CB  THR A 108      -6.611 -16.249   0.751  1.00120.53
C
ANISOU  770  CB  THR A 108    20237  17317   8241  -7204    228
-2532       C
ATOM    771  OG1 THR A 108      -7.580 -16.236  -0.305  1.00143.89
O
ANISOU  771  OG1 THR A 108    23170  20721  10780  -7831    -71
-2239       O
ATOM    772  CG2 THR A 108      -6.181 -17.686   1.020  1.00121.14
C
ANISOU  772  CG2 THR A 108    20782  16959   8286  -7455    440
-2888       C
ATOM    773  N   ILE A 109      -5.179 -14.904   3.148  1.00138.77
```

```
N
ANISOU  773  N   ILE A 109    22034  19190  11500  -5611    620
-2706       N
ATOM    774  CA  ILE A 109      -4.228 -14.894   4.259  1.00142.50
C
ANISOU  774  CA  ILE A 109    22503  19284  12358  -5041    885
-2950       C
ATOM    775  C   ILE A 109      -4.859 -14.346   5.540  1.00131.51
C
ANISOU  775  C   ILE A 109    20592  18143  11234  -4592    798
-2661       C
ATOM    776  O   ILE A 109      -4.581 -14.841   6.638  1.00149.30
O
ANISOU  776  O   ILE A 109    22818  20191  13718  -4335    930
-2774       O
ATOM    777  CB  ILE A 109      -2.947 -14.129   3.866  1.00145.12
C
ANISOU  777  CB  ILE A 109    23006  19309  12824  -4702   1105
-3220       C
ATOM    778  CG1 ILE A 109      -1.802 -14.476   4.824  1.00120.41
C
ANISOU  778  CG1 ILE A 109    20007  15721  10024  -4268   1405
-3537       C
ATOM    779  CG2 ILE A 109      -3.178 -12.619   3.801  1.00139.77
C
ANISOU  779  CG2 ILE A 109    21939  18929  12237  -4350    990
-2956       C
ATOM    780  CD1 ILE A 109      -0.431 -14.093   4.302  1.00119.40
C
ANISOU  780  CD1 ILE A 109    20161  15230   9977  -4066   1654
-3862       C
ATOM    781  N   SER A 110      -5.703 -13.317   5.428  1.00106.41
N
ANISOU  781  N   SER A 110    16997  15401   8033  -4485    593
-2280       N
ATOM    782  CA  SER A 110      -6.383 -12.767   6.601  1.00110.29
C
ANISOU  782  CA  SER A 110    16996  16144   8764  -4073    531
-1987       C
ATOM    783  C   SER A 110      -7.270 -13.787   7.309  1.00116.51
C
ANISOU  783  C   SER A 110    17674  17086   9510  -4310    419
-1845       C
ATOM    784  O   SER A 110      -7.156 -13.984   8.524  1.00124.94
O
ANISOU  784  O   SER A 110    18608  18037  10827  -3977    527
-1890       O
ATOM    785  CB  SER A 110      -7.215 -11.550   6.193  1.00106.18
C
ANISOU  785  CB  SER A 110    16070  16074   8201  -3972    338
-1570       C
ATOM    786  OG  SER A 110      -7.859 -10.966   7.311  1.00107.06
O
ANISOU  786  OG  SER A 110    15717  16407   8554  -3548    318
```

```
-1290       O
ATOM    787  N   PHE A 111      -8.157 -14.448   6.570  1.00110.15
N
ANISOU  787  N   PHE A 111    16926  16552   8375  -4908    198
-1668       N
ATOM    788  CA  PHE A 111      -9.096 -15.376   7.194  1.00119.87
C
ANISOU  788  CA  PHE A 111    18030  17977   9538  -5173     66
-1494       C
ATOM    789  C   PHE A 111      -8.484 -16.711   7.610  1.00120.68
C
ANISOU  789  C   PHE A 111    18548  17644   9659  -5338    241
-1859       C
ATOM    790  O   PHE A 111      -8.936 -17.300   8.598  1.00179.35
O
ANISOU  790  O   PHE A 111    25833  25118  17193  -5293    224
-1780       O
ATOM    791  CB  PHE A 111     -10.299 -15.591   6.274  1.00123.94
C
ANISOU  791  CB  PHE A 111    18442  18973   9677  -5784   -248
-1143       C
ATOM    792  CG  PHE A 111     -11.128 -14.351   6.081  1.00125.99
C
ANISOU  792  CG  PHE A 111    18186  19729   9957  -5592   -440
-679       C
ATOM    793  CD1 PHE A 111     -11.672 -13.699   7.175  1.00132.13
C
ANISOU  793  CD1 PHE A 111    18466  20722  11015  -5098   -442
-402       C
ATOM    794  CD2 PHE A 111     -11.358 -13.831   4.819  1.00173.41
C
ANISOU  794  CD2 PHE A 111    24208  25975  15704  -5898   -603
-511       C
ATOM    795  CE1 PHE A 111     -12.428 -12.554   7.016  1.00138.74
C
ANISOU  795  CE1 PHE A 111    18831  21987  11896  -4885   -582
38       C
ATOM    796  CE2 PHE A 111     -12.116 -12.684   4.654  1.00170.48
C
ANISOU  796  CE2 PHE A 111    23348  26053  15375  -5693   -768
-52       C
ATOM    797  CZ  PHE A 111     -12.652 -12.046   5.755  1.00132.63
C
ANISOU  797  CZ  PHE A 111    18061  21451  10883  -5173   -747
224       C
ATOM    798  N   ILE A 112      -7.483 -17.218   6.891  1.00111.39
N
ANISOU  798  N   ILE A 112    17884  16049   8391  -5524    421
-2243       N
ATOM    799  CA  ILE A 112      -6.779 -18.411   7.362  1.00131.45
C
ANISOU  799  CA  ILE A 112    20809  18121  11013  -5579    642
-2588       C
ATOM    800  C   ILE A 112      -6.026 -18.153   8.668  1.00144.64
```

```
C
ANISOU  800  C   ILE A 112    22323  19533  13100  -4930    849
-2714       C
ATOM    801  O   ILE A 112      -6.056 -18.984   9.585  1.00163.51
O
ANISOU  801  O   ILE A 112    24736  21777  15612  -4891    915
-2766       O
ATOM    802  CB  ILE A 112      -5.853 -18.969   6.260  1.00143.30
C
ANISOU  802  CB  ILE A 112    22896  19217  12334  -5901    825
-2963       C
ATOM    803  CG1 ILE A 112      -5.465 -20.416   6.575  1.00138.99
C
ANISOU  803  CG1 ILE A 112    22769  18252  11787  -6125   1014
-3240       C
ATOM    804  CG2 ILE A 112      -4.600 -18.116   6.069  1.00108.92
C
ANISOU  804  CG2 ILE A 112    18624  14573   8188  -5438   1047
-3202       C
ATOM    805  CD1 ILE A 112      -6.641 -21.365   6.648  1.00133.79
C
ANISOU  805  CD1 ILE A 112    22115  17841  10879  -6664    809
-3042       C
ATOM    806  N   GLU A 113      -5.337 -17.014   8.781  1.00144.18
N
ANISOU  806  N   GLU A 113    22114  19417  13252  -4435    953
-2760       N
ATOM    807  CA  GLU A 113      -4.602 -16.718  10.009  1.00146.94
C
ANISOU  807  CA  GLU A 113    22319  19542  13967  -3853   1143
-2873       C
ATOM    808  C   GLU A 113      -5.512 -16.495  11.216  1.00147.43
C
ANISOU  808  C   GLU A 113    21922  19918  14176  -3623   1021
-2573       C
ATOM    809  O   GLU A 113      -5.222 -16.990  12.311  1.00157.80
O
ANISOU  809  O   GLU A 113    23212  21051  15695  -3406   1135
-2659       O
ATOM    810  CB  GLU A 113      -3.709 -15.495   9.791  1.00139.90
C
ANISOU  810  CB  GLU A 113    21382  18547  13228  -3427   1269
-2977       C
ATOM    811  CG  GLU A 113      -2.653 -15.278  10.868  1.00129.39
C
ANISOU  811  CG  GLU A 113    20020  16905  12236  -2897   1503
-3172       C
ATOM    812  CD  GLU A 113      -1.509 -16.275  10.783  1.00138.21
C
ANISOU  812  CD  GLU A 113    21569  17532  13414  -2958   1741
-3534       C
ATOM    813  OE1 GLU A 113      -1.726 -17.468  11.085  1.00145.61
O
ANISOU  813  OE1 GLU A 113    22663  18344  14316  -3203   1756
```

```
-3580       O
ATOM    814  OE2 GLU A 113      -0.389 -15.864  10.409  1.00140.75
O
ANISOU  814  OE2 GLU A 113    22070  17589  13822  -2757   1924
-3761       O
ATOM    815  N   GLU A 114      -6.611 -15.757  11.046  1.00101.14
N
ANISOU  815  N   GLU A 114    15686  14527   8217  -3662    801
-2207       N
ATOM    816  CA  GLU A 114      -7.615 -15.662  12.106  1.00101.27
C
ANISOU  816  CA  GLU A 114    15273  14868   8336  -3513    686
-1898       C
ATOM    817  C   GLU A 114      -8.234 -17.013  12.458  1.00104.51
C
ANISOU  817  C   GLU A 114    15776  15306   8625  -3915    598
-1865       C
ATOM    818  O   GLU A 114      -8.462 -17.305  13.638  1.00177.45
O
ANISOU  818  O   GLU A 114    24829  24559  18036  -3715    629
-1806       O
ATOM    819  CB  GLU A 114      -8.690 -14.640  11.735  1.00102.91
C
ANISOU  819  CB  GLU A 114    15064  15579   8457  -3495    480
-1483       C
ATOM    820  CG  GLU A 114      -8.187 -13.202  11.833  1.00100.56
C
ANISOU  820  CG  GLU A 114    14585  15260   8363  -2965    598
-1468       C
ATOM    821  CD  GLU A 114      -9.306 -12.179  11.879  1.00121.34
C
ANISOU  821  CD  GLU A 114    16728  18370  11007  -2808    448
-1016       C
ATOM    822  OE1 GLU A 114     -10.487 -12.579  11.806  1.00122.93
O
ANISOU  822  OE1 GLU A 114    16700  18952  11054  -3118    236
-693      O
ATOM    823  OE2 GLU A 114      -8.998 -10.974  12.006  1.00100.37
O
ANISOU  823  OE2 GLU A 114    13913  15702   8520  -2369    554
-975      O
ATOM    824  N   ASN A 115      -8.524 -17.846  11.457  1.00106.30
N
ANISOU  824  N   ASN A 115    16300  15544   8544  -4501    489
-1900       N
ATOM    825  CA  ASN A 115      -8.974 -19.208  11.740  1.00115.26
C
ANISOU  825  CA  ASN A 115    17611  16630   9553  -4914    439
-1922       C
ATOM    826  C   ASN A 115      -7.928 -20.003  12.515  1.00123.57
C
ANISOU  826  C   ASN A 115    18958  17165  10826  -4706    699
-2267       C
ATOM    827  O   ASN A 115      -8.261 -20.703  13.479  1.00134.28
```

```
O
ANISOU  827  O   ASN A 115    20234  18515  12272  -4702    695
-2215       O
ATOM    828  CB  ASN A 115      -9.327 -19.920  10.433  1.00135.05
C
ANISOU  828  CB  ASN A 115    20463  19185  11666  -5605    314
-1947       C
ATOM    829  CG  ASN A 115      -9.999 -21.260  10.660  1.00140.10
C
ANISOU  829  CG  ASN A 115    21260  19846  12126  -6098    226
-1911       C
ATOM    830  OD1 ASN A 115     -10.362 -21.604  11.784  1.00135.19
O
ANISOU  830  OD1 ASN A 115    20423  19278  11665  -5932    218
-1809       O
ATOM    831  ND2 ASN A 115     -10.164 -22.029   9.588  1.00159.87
N
ANISOU  831  ND2 ASN A 115    24159  22302  14280  -6730    166
-2000       N
ATOM    832  N   ASN A 116      -6.661 -19.913  12.109  1.00129.69
N
ANISOU  832  N   ASN A 116    20063  17516  11695  -4536    927
-2602       N
ATOM    833  CA  ASN A 116      -5.582 -20.553  12.859  1.00149.23
C
ANISOU  833  CA  ASN A 116    22771  19512  14417  -4274   1188
-2895       C
ATOM    834  C   ASN A 116      -5.484 -20.017  14.286  1.00163.27
C
ANISOU  834  C   ASN A 116    24168  21354  16514  -3731   1233
-2794       C
ATOM    835  O   ASN A 116      -5.351 -20.789  15.243  1.00178.61
O
ANISOU  835  O   ASN A 116    26134  23131  18599  -3658   1309
-2841       O
ATOM    836  CB  ASN A 116      -4.256 -20.365  12.119  1.00165.86
C
ANISOU  836  CB  ASN A 116    25234  21211  16574  -4150   1421
-3226       C
ATOM    837  CG  ASN A 116      -3.091 -21.022  12.833  1.00167.38
C
ANISOU  837  CG  ASN A 116    25650  20916  17031  -3874   1699
-3499       C
ATOM    838  OD1 ASN A 116      -3.257 -22.036  13.512  1.00165.76
O
ANISOU  838  OD1 ASN A 116    25523  20582  16877  -3977   1731
-3505       O
ATOM    839  ND2 ASN A 116      -1.904 -20.444  12.688  1.00164.60
N
ANISOU  839  ND2 ASN A 116    25390  20301  16849  -3522   1899
-3705       N
ATOM    840  N   ALA A 117      -5.548 -18.694  14.445  1.00162.34
N
ANISOU  840  N   ALA A 117    23712  21468  16502  -3358   1196
```

```
-2654       N
ATOM    841  CA  ALA A 117      -5.571 -18.086  15.775  1.00143.18
C
ANISOU  841  CA  ALA A 117    20922  19140  14341  -2876   1238
-2541       C
ATOM    842  C   ALA A 117      -6.784 -18.516  16.597  1.00145.77
C
ANISOU  842  C   ALA A 117    20955  19793  14639  -3002   1074
-2259       C
ATOM    843  O   ALA A 117      -6.663 -18.781  17.799  1.00141.58
O
ANISOU  843  O   ALA A 117    20305  19190  14298  -2769   1148
-2262       O
ATOM    844  CB  ALA A 117      -5.527 -16.564  15.644  1.00136.06
C
ANISOU  844  CB  ALA A 117    19749  18432  13518  -2511   1236
-2430       C
ATOM    845  N   GLY A 118      -7.960 -18.587  15.972  1.00155.23
N
ANISOU  845  N   GLY A 118    22020  21368  15592  -3380    847
-1997       N
ATOM    846  CA  GLY A 118      -9.133 -19.105  16.666  1.00148.55
C
ANISOU  846  CA  GLY A 118    20911  20841  14692  -3559    685
-1723       C
ATOM    847  C   GLY A 118      -9.006 -20.554  17.100  1.00140.95
C
ANISOU  847  C   GLY A 118    20227  19623  13704  -3837    726
-1870       C
ATOM    848  O   GLY A 118      -9.449 -20.925  18.190  1.00125.68
O
ANISOU  848  O   GLY A 118    18094  17785  11874  -3753    705
-1751       O
ATOM    849  N   ASN A 119      -8.404 -21.393  16.255  1.00155.69
N
ANISOU  849  N   ASN A 119    22570  21152  15434  -4172    802
-2128       N
ATOM    850  CA  ASN A 119      -8.095 -22.766  16.652  1.00164.84
C
ANISOU  850  CA  ASN A 119    24053  21974  16606  -4385    901
-2304       C
ATOM    851  C   ASN A 119      -7.146 -22.825  17.846  1.00162.94
C
ANISOU  851  C   ASN A 119    23791  21417  16703  -3902   1112
-2461       C
ATOM    852  O   ASN A 119      -7.331 -23.649  18.750  1.00163.30
O
ANISOU  852  O   ASN A 119    23826  21394  16826  -3936   1123
-2429       O
ATOM    853  CB  ASN A 119      -7.505 -23.535  15.471  1.00173.33
C
ANISOU  853  CB  ASN A 119    25672  22693  17490  -4782   1001
-2576       C
ATOM    854  CG  ASN A 119      -7.189 -24.976  15.819  1.00175.90
```

```
C
ANISOU  854  CG  ASN A 119    26369  22633  17832  -5008   1135
-2755       C
ATOM    855  OD1 ASN A 119      -6.025 -25.371  15.883  1.00178.59
O
ANISOU  855  OD1 ASN A 119    27019  22494  18343  -4820   1391
-3036       O
ATOM    856  ND2 ASN A 119      -8.228 -25.768  16.057  1.00175.30
N
ANISOU  856  ND2 ASN A 119    26255  22766  17584  -5407    968
-2574       N
ATOM    857  N   ALA A 120      -6.128 -21.962  17.875  1.00150.45
N
ANISOU  857  N   ALA A 120    22198  19653  15314  -3466   1273
-2615       N
ATOM    858  CA  ALA A 120      -5.199 -21.953  19.001  1.00120.11
C
ANISOU  858  CA  ALA A 120    18315  15545  11775  -3025   1460
-2740       C
ATOM    859  C   ALA A 120      -5.812 -21.388  20.277  1.00116.82
C
ANISOU  859  C   ALA A 120    17444  15442  11499  -2727   1383
-2507       C
ATOM    860  O   ALA A 120      -5.271 -21.627  21.362  1.00131.27
O
ANISOU  860  O   ALA A 120    19232  17104  13540  -2464   1497
-2564       O
ATOM    861  CB  ALA A 120      -3.940 -21.161  18.643  1.00132.52
C
ANISOU  861  CB  ALA A 120    20000  16862  13491  -2677   1646
-2959       C
ATOM    862  N   ALA A 121      -6.918 -20.655  20.179  1.00132.79
N
ANISOU  862  N   ALA A 121    19128  17915  13410  -2764   1202
-2235       N
ATOM    863  CA  ALA A 121      -7.602 -20.119  21.349  1.00101.04
C
ANISOU  863  CA  ALA A 121    14680  14204   9506  -2499   1149
-2001       C
ATOM    864  C   ALA A 121      -8.733 -21.019  21.840  1.00106.55
C
ANISOU  864  C   ALA A 121    15250  15141  10094  -2820    991
-1787       C
ATOM    865  O   ALA A 121      -9.467 -20.625  22.752  1.00107.18
O
ANISOU  865  O   ALA A 121    14960  15523  10242  -2651    936
-1562       O
ATOM    866  CB  ALA A 121      -8.146 -18.718  21.048  1.00 93.65
C
ANISOU  866  CB  ALA A 121    13415  13616   8552  -2287   1084
-1802       C
ATOM    867  N   ALA A 122      -8.880 -22.219  21.270  1.00111.75
N
ANISOU  867  N   ALA A 122    16219  15661  10579  -3285    932
```

```
-1856       N
ATOM    868  CA  ALA A 122      -9.893 -23.155  21.752  1.00106.53
C
ANISOU  868  CA  ALA A 122    15473  15199   9804  -3621    786
-1667       C
ATOM    869  C   ALA A 122      -9.669 -23.527  23.215  1.00136.63
C
ANISOU  869  C   ALA A 122    19170  18908  13837  -3363    878
-1672       C
ATOM    870  O   ALA A 122     -10.619 -23.555  24.007  1.00145.42
O
ANISOU  870  O   ALA A 122    19963  20349  14941  -3381    766
-1427       O
ATOM    871  CB  ALA A 122      -9.904 -24.409  20.878  1.00103.38
C
ANISOU  871  CB  ALA A 122    15513  14583   9184  -4168    752
-1795       C
ATOM    872  N   ALA A 123      -8.422 -23.821  23.589  1.00139.91
N
ANISOU  872  N   ALA A 123    19831  18884  14447  -3129   1080
-1931       N
ATOM    873  CA  ALA A 123      -8.104 -24.074  24.992  1.00101.42
C
ANISOU  873  CA  ALA A 123    14833  13917   9786  -2861   1168
-1926       C
ATOM    874  C   ALA A 123      -8.392 -22.854  25.859  1.00 93.57
C
ANISOU  874  C   ALA A 123    13406  13230   8916  -2455   1169
-1769       C
ATOM    875  O   ALA A 123      -8.905 -22.984  26.977  1.00 93.46
O
ANISOU  875  O   ALA A 123    13152  13393   8966  -2379   1138
-1618       O
ATOM    876  CB  ALA A 123      -6.641 -24.497  25.132  1.00 93.87
C
ANISOU  876  CB  ALA A 123    14194  12452   9019  -2667   1386
-2204       C
ATOM    877  N   LEU A 124      -8.059 -21.660  25.362  1.00 92.99
N
ANISOU  877  N   LEU A 124    13250  13209   8874  -2195   1221
-1809       N
ATOM    878  CA  LEU A 124      -8.374 -20.425  26.076  1.00 90.77
C
ANISOU  878  CA  LEU A 124    12591  13203   8695  -1820   1249
-1664       C
ATOM    879  C   LEU A 124      -9.876 -20.264  26.290  1.00 92.82
C
ANISOU  879  C   LEU A 124    12492  13938   8836  -1960   1083
-1334       C
ATOM    880  O   LEU A 124     -10.327 -19.957  27.400  1.00112.86
O
ANISOU  880  O   LEU A 124    14744  16673  11466  -1759   1110
-1191       O
ATOM    881  CB  LEU A 124      -7.819 -19.233  25.297  1.00 89.47
```

```
C
ANISOU  881  CB  LEU A 124    12445  12998   8553  -1581   1326
-1757       C
ATOM    882  CG  LEU A 124      -7.861 -17.855  25.952  1.00 87.82
C
ANISOU  882  CG  LEU A 124    11933  12965   8469  -1149   1419
-1672       C
ATOM    883  CD1 LEU A 124      -6.513 -17.520  26.570  1.00 85.06
C
ANISOU  883  CD1 LEU A 124    11719  12293   8306   -818   1616
-1911       C
ATOM    884  CD2 LEU A 124      -8.282 -16.802  24.939  1.00 88.51
C
ANISOU  884  CD2 LEU A 124    11907  13256   8466  -1104   1370
-1562       C
ATOM    885  N   MET A 125     -10.667 -20.462  25.233  1.00125.58
N
ANISOU  885  N   MET A 125    16650  18292  12774  -2314    916
-1200       N
ATOM    886  CA  MET A 125     -12.123 -20.479  25.368  1.00 97.76
C
ANISOU  886  CA  MET A 125    12784  15239   9120  -2511    738
-855       C
ATOM    887  C   MET A 125     -12.606 -21.591  26.296  1.00116.71
C
ANISOU  887  C   MET A 125    15158  17680  11508  -2721    680
-778       C
ATOM    888  O   MET A 125     -13.522 -21.382  27.100  1.00137.33
O
ANISOU  888  O   MET A 125    17410  20631  14137  -2651    630
-528       O
ATOM    889  CB  MET A 125     -12.773 -20.605  23.989  1.00110.85
C
ANISOU  889  CB  MET A 125    14498  17095  10526  -2920    553
-729       C
ATOM    890  CG  MET A 125     -13.042 -19.271  23.299  1.00151.70
C
ANISOU  890  CG  MET A 125    19441  22511  15688  -2713    535
-579       C
ATOM    891  SD  MET A 125     -11.556 -18.352  22.859  1.00208.96
S
ANISOU  891  SD  MET A 125    26941  29360  23092  -2322    747
-907       S
ATOM    892  CE  MET A 125     -12.259 -16.764  22.424  1.00165.40
C
ANISOU  892  CE  MET A 125    21027  24233  17583  -2048    716
-615       C
ATOM    893  N   ALA A 126     -12.008 -22.781  26.194  1.00124.24
N
ANISOU  893  N   ALA A 126    16490  18284  12433  -2976    699
-985       N
ATOM    894  CA  ALA A 126     -12.323 -23.869  27.119  1.00135.73
C
ANISOU  894  CA  ALA A 126    17960  19715  13896  -3156    665
```

```
-935       C
ATOM    895  C   ALA A 126     -12.024 -23.507  28.572  1.00154.73
C
ANISOU  895  C   ALA A 126    20154  22111  16527  -2748    793
-930       C
ATOM    896  O   ALA A 126     -12.803 -23.843  29.472  1.00132.98
O
ANISOU  896  O   ALA A 126    17170  19590  13765  -2811    729
-742       O
ATOM    897  CB  ALA A 126     -11.558 -25.130  26.715  1.00129.31
C
ANISOU  897  CB  ALA A 126    17633  18451  13048  -3441    714
-1181       C
ATOM    898  N   GLY A 127     -10.902 -22.830  28.823  1.00145.86
N
ANISOU  898  N   GLY A 127    19104  20727  15587  -2352    973
-1131       N
ATOM    899  CA  GLY A 127     -10.566 -22.448  30.189  1.00118.73
C
ANISOU  899  CA  GLY A 127    15490  17285  12338  -1997   1097
-1133       C
ATOM    900  C   GLY A 127     -11.569 -21.506  30.832  1.00120.17
C
ANISOU  900  C   GLY A 127    15237  17899  12523  -1805   1081
-882       C
ATOM    901  O   GLY A 127     -11.882 -21.637  32.018  1.00134.45
O
ANISOU  901  O   GLY A 127    16863  19827  14396  -1719   1111
-786       O
ATOM    902  N   LEU A 128     -12.088 -20.552  30.066  1.00121.06
N
ANISOU  902  N   LEU A 128    15179  18248  12570  -1735   1047
-762       N
ATOM    903  CA  LEU A 128     -13.011 -19.543  30.575  1.00114.24
C
ANISOU  903  CA  LEU A 128    13902  17773  11733  -1504   1073
-511       C
ATOM    904  C   LEU A 128     -14.481 -19.949  30.486  1.00148.10
C
ANISOU  904  C   LEU A 128    17912  22491  15870  -1802    890
-180       C
ATOM    905  O   LEU A 128     -15.343 -19.180  30.925  1.00171.49
O
ANISOU  905  O   LEU A 128    20502  25798  18860  -1614    921
66       O
ATOM    906  CB  LEU A 128     -12.784 -18.209  29.846  1.00104.24
C
ANISOU  906  CB  LEU A 128    12572  16533  10500  -1222   1157
-524       C
ATOM    907  CG  LEU A 128     -12.938 -18.129  28.322  1.00151.89
C
ANISOU  907  CG  LEU A 128    18713  22608  16390  -1447   1026
-494       C
ATOM    908  CD1 LEU A 128     -14.386 -17.914  27.889  1.00174.21
```

```
C
ANISOU  908  CD1 LEU A 128    21193  25916  19081  -1621    862
-119       C
ATOM    909  CD2 LEU A 128     -12.041 -17.037  27.761  1.00173.46
C
ANISOU  909  CD2 LEU A 128    21560  25142  19207  -1137   1163
-667       C
ATOM    910  N   ALA A 129     -14.792 -21.119  29.929  1.00123.37
N
ANISOU  910  N   ALA A 129    14952  19347  12576  -2263    713
-159       N
ATOM    911  CA  ALA A 129     -16.176 -21.565  29.847  1.00108.68
C
ANISOU  911  CA  ALA A 129    12832  17912  10550  -2594    522
166       C
ATOM    912  C   ALA A 129     -16.803 -21.725  31.235  1.00145.75
C
ANISOU  912  C   ALA A 129    17242  22819  15317  -2495    559
329       C
ATOM    913  O   ALA A 129     -16.122 -22.076  32.204  1.00172.25
O
ANISOU  913  O   ALA A 129    20728  25922  18796  -2358    675
153       O
ATOM    914  CB  ALA A 129     -16.263 -22.886  29.086  1.00105.55
C
ANISOU  914  CB  ALA A 129    12747  17402   9955  -3142    347
112       C
ATOM    915  N   PRO A 130     -18.106 -21.453  31.351  1.00139.00
N
ANISOU  915  N   PRO A 130    15984  22444  14386  -2561    465
683       N
ATOM    916  CA  PRO A 130     -18.890 -21.936  32.496  1.00148.18
C
ANISOU  916  CA  PRO A 130    16903  23848  15552  -2626    448
869       C
ATOM    917  C   PRO A 130     -18.719 -23.429  32.744  1.00129.49
C
ANISOU  917  C   PRO A 130    14820  21286  13093  -3033    335
758       C
ATOM    918  O   PRO A 130     -18.718 -24.239  31.814  1.00108.21
O
ANISOU  918  O   PRO A 130    12382  18499  10235  -3445    180
713       O
ATOM    919  CB  PRO A 130     -20.330 -21.594  32.098  1.00144.39
C
ANISOU  919  CB  PRO A 130    15994  23919  14949  -2764    307
1287       C
ATOM    920  CG  PRO A 130     -20.179 -20.392  31.222  1.00112.88
C
ANISOU  920  CG  PRO A 130    11919  19968  11002  -2499    367
1321       C
ATOM    921  CD  PRO A 130     -18.939 -20.684  30.411  1.00108.31
C
ANISOU  921  CD  PRO A 130    11825  18921  10407  -2585    370
```

```
957        C
ATOM    922  N   LYS A 131      -18.579 -23.791  34.020  1.00115.53
N
ANISOU  922  N   LYS A 131    13020  19449  11426  -2924    424
716        N
ATOM    923  CA  LYS A 131      -18.322 -25.167  34.415  1.00133.56
C
ANISOU  923  CA  LYS A 131    15577  21507  13662  -3248    351
609        C
ATOM    924  C   LYS A 131      -19.194 -25.530  35.609  1.00160.81
C
ANISOU  924  C   LYS A 131    18740  25256  17104  -3308    326
828        C
ATOM    925  O   LYS A 131      -19.379 -24.725  36.526  1.00171.40
O
ANISOU  925  O   LYS A 131    19798  26765  18564  -2957    469
908        O
ATOM    926  CB  LYS A 131      -16.843 -25.378  34.764  1.00140.64
C
ANISOU  926  CB  LYS A 131    16837  21877  14721  -3041    507
263        C
ATOM    927  CG  LYS A 131      -15.900 -25.178  33.589  1.00145.38
C
ANISOU  927  CG  LYS A 131    17764  22146  15329  -3014    540
26        C
ATOM    928  CD  LYS A 131      -14.445 -25.335  33.997  1.00101.32
C
ANISOU  928  CD  LYS A 131    12490  16081   9927  -2779    706
-278        C
ATOM    929  CE  LYS A 131      -13.519 -25.073  32.818  1.00137.79
C
ANISOU  929  CE  LYS A 131    17410  20388  14556  -2734    757
-506        C
ATOM    930  NZ  LYS A 131      -12.086 -25.248  33.182  1.00147.58
N
ANISOU  930  NZ  LYS A 131    18927  21170  15975  -2508    920
-775        N
ATOM    931  N   THR A 132      -19.726 -26.750  35.580  1.00171.45
N
ANISOU  931  N   THR A 132    20185  26659  18300  -3770    156
919        N
ATOM    932  CA  THR A 132      -20.702 -27.247  36.539  1.00174.57
C
ANISOU  932  CA  THR A 132    20312  27378  18637  -3933     87
1161        C
ATOM    933  C   THR A 132      -20.122 -28.426  37.308  1.00160.08
C
ANISOU  933  C   THR A 132    18775  25210  16836  -4096     95
1002        C
ATOM    934  O   THR A 132      -19.256 -29.149  36.805  1.00163.66
O
ANISOU  934  O   THR A 132    19654  25239  17292  -4247     89
770        O
ATOM    935  CB  THR A 132      -22.014 -27.668  35.853  1.00172.44
```

C
ANISOU 935 CB THR A 132 19848 27540 18131 -4387 -145
1471 C
ATOM 936 OG1 THR A 132 -22.973 -28.055 36.845 1.00157.10
O
ANISOU 936 OG1 THR A 132 17605 25941 16145 -4509 -195
1720 O
ATOM 937 CG2 THR A 132 -21.779 -28.834 34.906 1.00195.35
C
ANISOU 937 CG2 THR A 132 23180 30189 20854 -4897 -303
1347 C
ATOM 938 N LYS A 133 -20.593 -28.614 38.539 1.00138.34
N
ANISOU 938 N LYS A 133 15798 22649 14118 -4056 122
1137 N
ATOM 939 CA LYS A 133 -20.029 -29.613 39.438 1.00144.72
C
ANISOU 939 CA LYS A 133 16840 23164 14984 -4151 145
1016 C
ATOM 940 C LYS A 133 -21.093 -30.660 39.727 1.00140.68
C
ANISOU 940 C LYS A 133 16241 22912 14299 -4610 -32
1246 C
ATOM 941 O LYS A 133 -22.180 -30.335 40.217 1.00121.79
O
ANISOU 941 O LYS A 133 13445 20985 11844 -4624 -71
1515 O
ATOM 942 CB LYS A 133 -19.539 -28.993 40.751 1.00165.34
C
ANISOU 942 CB LYS A 133 19305 25741 17776 -3731 335
957 C
ATOM 943 CG LYS A 133 -18.443 -27.948 40.624 1.00166.63
C
ANISOU 943 CG LYS A 133 19556 25649 18105 -3283 521
730 C
ATOM 944 CD LYS A 133 -19.004 -26.551 40.423 1.00183.09
C
ANISOU 944 CD LYS A 133 21298 28063 20206 -2982 612
849 C
ATOM 945 CE LYS A 133 -17.891 -25.547 40.176 1.00178.73
C
ANISOU 945 CE LYS A 133 20878 27232 19799 -2580 787
611 C
ATOM 946 NZ LYS A 133 -18.419 -24.172 39.965 1.00182.04
N
ANISOU 946 NZ LYS A 133 20989 27937 20243 -2275 897
728 N
ATOM 947 N VAL A 134 -20.770 -31.915 39.414 1.00154.11
N
ANISOU 947 N VAL A 134 18328 24302 15925 -4984 -124
1142 N
ATOM 948 CA VAL A 134 -21.739 -32.993 39.280 1.00138.28
C
ANISOU 948 CA VAL A 134 16344 22492 13705 -5523 -319

1331 C
ATOM 949 C VAL A 134 -21.151 -34.239 39.925 1.00139.60
C
ANISOU 949 C VAL A 134 16859 22266 13918 -5701 -304
1208 C
ATOM 950 O VAL A 134 -19.946 -34.330 40.172 1.00155.03
O
ANISOU 950 O VAL A 134 19081 23770 16055 -5450 -160
972 O
ATOM 951 CB VAL A 134 -22.113 -33.277 37.807 1.00119.99
C
ANISOU 951 CB VAL A 134 14204 20212 11177 -5924 -472
1351 C
ATOM 952 CG1 VAL A 134 -22.899 -32.114 37.219 1.00120.67
C
ANISOU 952 CG1 VAL A 134 13880 20769 11199 -5797 -522
1559 C
ATOM 953 CG2 VAL A 134 -20.862 -33.540 36.988 1.00118.51
C
ANISOU 953 CG2 VAL A 134 14516 19456 11057 -5888 -379
1020 C
ATOM 954 N LEU A 135 -22.017 -35.206 40.204 1.00134.82
N
ANISOU 954 N LEU A 135 16236 21843 13146 -6140 -452
1390 N
ATOM 955 CA LEU A 135 -21.594 -36.518 40.680 1.00148.32
C
ANISOU 955 CA LEU A 135 18304 23183 14869 -6390 -457
1307 C
ATOM 956 C LEU A 135 -22.124 -37.559 39.704 1.00139.35
C
ANISOU 956 C LEU A 135 17467 21997 13483 -6991 -617
1340 C
ATOM 957 O LEU A 135 -23.340 -37.759 39.603 1.00153.75
O
ANISOU 957 O LEU A 135 19064 24266 15088 -7358 -792
1593 O
ATOM 958 CB LEU A 135 -22.086 -36.780 42.104 1.00151.32
C
ANISOU 958 CB LEU A 135 18421 23795 15280 -6371 -468
1486 C
ATOM 959 CG LEU A 135 -21.647 -35.763 43.162 1.00118.73
C
ANISOU 959 CG LEU A 135 14001 19749 11360 -5829 -304
1463 C
ATOM 960 CD1 LEU A 135 -22.720 -34.706 43.393 1.00131.58
C
ANISOU 960 CD1 LEU A 135 15102 21966 12927 -5685 -317
1695 C
ATOM 961 CD2 LEU A 135 -21.276 -36.458 44.466 1.00118.31
C
ANISOU 961 CD2 LEU A 135 14013 19534 11407 -5808 -259
1468 C
ATOM 962 N ARG A 136 -21.213 -38.222 38.993 1.00140.12

```
N
ANISOU  962  N   ARG A 136    18079  21554  13607  -7103   -546
1092       N
ATOM    963  CA  ARG A 136     -21.557 -39.057  37.849  1.00168.52
C
ANISOU  963  CA  ARG A 136    22035  25039  16957  -7657   -655
1062       C
ATOM    964  C   ARG A 136     -20.928 -40.421  38.072  1.00175.03
C
ANISOU  964  C   ARG A 136    23368  25317  17821  -7885   -578
916       C
ATOM    965  O   ARG A 136     -19.712 -40.516  38.268  1.00192.57
O
ANISOU  965  O   ARG A 136    25843  27044  20280  -7554   -388
700       O
ATOM    966  CB  ARG A 136     -21.040 -38.450  36.540  1.00175.74
C
ANISOU  966  CB  ARG A 136    23129  25796  17848  -7578   -604
879       C
ATOM    967  CG  ARG A 136     -21.478 -37.017  36.264  1.00176.25
C
ANISOU  967  CG  ARG A 136    22722  26326  17917  -7277   -645
1005       C
ATOM    968  CD  ARG A 136     -22.926 -36.905  35.823  1.00178.59
C
ANISOU  968  CD  ARG A 136    22699  27225  17933  -7682   -876
1318       C
ATOM    969  NE  ARG A 136     -23.849 -36.969  36.952  1.00183.32
N
ANISOU  969  NE  ARG A 136    22885  28249  18519  -7703   -957
1596       N
ATOM    970  CZ  ARG A 136     -25.165 -36.826  36.844  1.00164.38
C
ANISOU  970  CZ  ARG A 136    20105  26439  15914  -7991  -1145
1924       C
ATOM    971  NH1 ARG A 136     -25.696 -36.488  35.679  1.00168.51
N
ANISOU  971  NH1 ARG A 136    20566  27222  16237  -8242  -1278
2032       N
ATOM    972  NH2 ARG A 136     -25.935 -36.910  37.921  1.00142.07
N
ANISOU  972  NH2 ARG A 136    16918  23971  13093  -7987  -1190
2163       N
ATOM    973  N   ASP A 137     -21.756 -41.468  38.068  1.00163.60
N
ANISOU  973  N   ASP A 137    22055  23962  16145  -8443   -718
1053       N
ATOM    974  CA  ASP A 137     -21.345 -42.812  38.473  1.00160.44
C
ANISOU  974  CA  ASP A 137    22091  23091  15777  -8679   -649
974       C
ATOM    975  C   ASP A 137     -20.917 -42.845  39.935  1.00160.09
C
ANISOU  975  C   ASP A 137    21857  22980  15988  -8299   -565
```

1029 C
ATOM 976 O ASP A 137 -20.161 -43.731 40.347 1.00173.97
O
ANISOU 976 O ASP A 137 23965 24248 17889 -8282 -443
927 O
ATOM 977 CB ASP A 137 -20.228 -43.363 37.577 1.00152.14
C
ANISOU 977 CB ASP A 137 21644 21367 14797 -8700 -464
667 C
ATOM 978 CG ASP A 137 -20.653 -43.495 36.129 1.00144.80
C
ANISOU 978 CG ASP A 137 20977 20465 13574 -9165 -543
600 C
ATOM 979 OD1 ASP A 137 -19.779 -43.394 35.242 1.00162.67
O
ANISOU 979 OD1 ASP A 137 23588 22322 15898 -9057 -389
349 O
ATOM 980 OD2 ASP A 137 -21.859 -43.695 35.876 1.00142.88
O
ANISOU 980 OD2 ASP A 137 20591 20666 13031 -9653 -759
807 O
ATOM 981 N GLY A 138 -21.393 -41.886 40.727 1.00150.15
N
ANISOU 981 N GLY A 138 20052 22210 14786 -7997 -618
1199 N
ATOM 982 CA GLY A 138 -20.973 -41.750 42.104 1.00141.56
C
ANISOU 982 CA GLY A 138 18761 21104 13923 -7625 -533
1246 C
ATOM 983 C GLY A 138 -19.657 -41.031 42.294 1.00142.83
C
ANISOU 983 C GLY A 138 18955 20942 14373 -7050 -336
1043 C
ATOM 984 O GLY A 138 -19.113 -41.052 43.404 1.00137.97
O
ANISOU 984 O GLY A 138 18253 20224 13945 -6770 -255
1059 O
ATOM 985 N LYS A 139 -19.126 -40.395 41.250 1.00165.17
N
ANISOU 985 N LYS A 139 21903 23623 17232 -6884 -264
862 N
ATOM 986 CA LYS A 139 -17.820 -39.744 41.295 1.00170.02
C
ANISOU 986 CA LYS A 139 22586 23907 18107 -6371 -76
659 C
ATOM 987 C LYS A 139 -18.023 -38.234 41.239 1.00164.51
C
ANISOU 987 C LYS A 139 21468 23606 17433 -6010 -69
682 C
ATOM 988 O LYS A 139 -18.498 -37.700 40.231 1.00184.64
O
ANISOU 988 O LYS A 139 23957 26355 19842 -6110 -133
681 O
ATOM 989 CB LYS A 139 -16.929 -40.218 40.149 1.00169.44

```
C
ANISOU  989  CB  LYS A 139    23016  23295  18069  -6440     38
415       C
ATOM    990  CG  LYS A 139     -15.518 -39.647  40.193  1.00166.52
C
ANISOU  990  CG  LYS A 139    22730  22564  17975  -5927    237
216       C
ATOM    991  CD  LYS A 139     -14.633 -40.240  39.109  1.00173.02
C
ANISOU  991  CD  LYS A 139    24070  22828  18843  -6006    377
-16       C
ATOM    992  CE  LYS A 139     -13.223 -39.672  39.183  1.00155.59
C
ANISOU  992  CE  LYS A 139    21913  20287  16915  -5493    574
-188       C
ATOM    993  NZ  LYS A 139     -12.331 -40.239  38.135  1.00139.36
N
ANISOU  993  NZ  LYS A 139    20353  17679  14919  -5542    741
-411       N
ATOM    994  N   TRP A 140     -17.662 -37.557  42.327  1.00155.38
N
ANISOU  994  N   TRP A 140    20034  22557  16447  -5606     14
712       N
ATOM    995  CA  TRP A 140     -17.731 -36.105  42.435  1.00158.01
C
ANISOU  995  CA  TRP A 140    19999  23206  16832  -5216     69
718       C
ATOM    996  C   TRP A 140     -16.829 -35.452  41.391  1.00179.25
C
ANISOU  996  C   TRP A 140    22877  25622  19609  -4975    174
491       C
ATOM    997  O   TRP A 140     -15.602 -35.574  41.465  1.00195.73
O
ANISOU  997  O   TRP A 140    25208  27282  21880  -4746    309
310       O
ATOM    998  CB  TRP A 140     -17.347 -35.697  43.861  1.00163.41
C
ANISOU  998  CB  TRP A 140    20456  23960  17674  -4881    163
759       C
ATOM    999  CG  TRP A 140     -17.566 -34.263  44.229  1.00176.19
C
ANISOU  999  CG  TRP A 140    21686  25930  19328  -4512    241
793       C
ATOM   1000  CD1 TRP A 140     -18.231 -33.313  43.508  1.00176.26
C
ANISOU 1000  CD1 TRP A 140    21469  26253  19249  -4445    222
843       C
ATOM   1001  CD2 TRP A 140     -17.165 -33.632  45.451  1.00167.59
C
ANISOU 1001  CD2 TRP A 140    20395  24919  18362  -4179    360
797       C
ATOM   1002  NE1 TRP A 140     -18.239 -32.120  44.193  1.00173.90
N
ANISOU 1002  NE1 TRP A 140    20858  26188  19030  -4063    347
```

```
865       N
ATOM   1003  CE2 TRP A 140     -17.594 -32.292  45.391  1.00180.35
C
ANISOU 1003  CE2 TRP A 140    21696  26867  19963  -3910    434
826       C
ATOM   1004  CE3 TRP A 140     -16.473 -34.069  46.585  1.00115.92
C
ANISOU 1004  CE3 TRP A 140    13917  18196  11933  -4094    415
787       C
ATOM   1005  CZ2 TRP A 140     -17.354 -31.385  46.422  1.00171.08
C
ANISOU 1005  CZ2 TRP A 140    20302  25827  18872  -3576    578
819       C
ATOM   1006  CZ3 TRP A 140     -16.235 -33.168  47.607  1.00110.93
C
ANISOU 1006  CZ3 TRP A 140    13050  17730  11370  -3783    533
792       C
ATOM   1007  CH2 TRP A 140     -16.675 -31.841  47.519  1.00138.29
C
ANISOU 1007  CH2 TRP A 140    16234  21505  14806  -3534    622
795       C
ATOM   1008  N   SER A 141     -17.421 -34.759  40.417  1.00161.44
N
ANISOU 1008  N   SER A 141    20497  23619  17223  -5028    113
516       N
ATOM   1009  CA  SER A 141     -16.700 -34.349  39.219  1.00130.08
C
ANISOU 1009  CA  SER A 141    16762  19384  13280  -4923    182
310       C
ATOM   1010  C   SER A 141     -17.087 -32.928  38.829  1.00131.34
C
ANISOU 1010  C   SER A 141    16583  19903  13419  -4662    189
361       C
ATOM   1011  O   SER A 141     -18.146 -32.421  39.206  1.00129.51
O
ANISOU 1011  O   SER A 141    15960  20148  13099  -4673    113
585       O
ATOM   1012  CB  SER A 141     -16.974 -35.303  38.046  1.00118.63
C
ANISOU 1012  CB  SER A 141    15671  17762  11642  -5408     86
266       C
ATOM   1013  OG  SER A 141     -16.613 -36.634  38.372  1.00114.68
O
ANISOU 1013  OG  SER A 141    15519  16889  11168  -5647    108
217       O
ATOM   1014  N   GLU A 142     -16.203 -32.289  38.064  1.00156.72
N
ANISOU 1014  N   GLU A 142    19953  22872  16723  -4416    295
158       N
ATOM   1015  CA  GLU A 142     -16.428 -30.956  37.521  1.00177.68
C
ANISOU 1015  CA  GLU A 142    22355  25788  19366  -4166    317
180       C
ATOM   1016  C   GLU A 142     -16.430 -31.023  36.000  1.00178.58
```

```
C
ANISOU 1016  C   GLU A 142    22703  25805  19343  -4401    251
96       C
ATOM   1017  O   GLU A 142     -15.492 -31.561  35.401  1.00173.28
O
ANISOU 1017  O   GLU A 142    22439  24684  18715  -4457    322
-129       O
ATOM   1018  CB  GLU A 142     -15.346 -29.986  38.007  1.00161.68
C
ANISOU 1018  CB  GLU A 142    20289  23581  17559  -3649    508
15       C
ATOM   1019  CG  GLU A 142     -15.475 -28.571  37.473  1.00178.52
C
ANISOU 1019  CG  GLU A 142    22197  25931  19700  -3359    560
21       C
ATOM   1020  CD  GLU A 142     -14.368 -27.662  37.973  1.00185.93
C
ANISOU 1020  CD  GLU A 142    23137  26671  20837  -2889    753
-155       C
ATOM   1021  OE1 GLU A 142     -14.656 -26.763  38.791  1.00187.93
O
ANISOU 1021  OE1 GLU A 142    23087  27177  21143  -2616    835
-67       O
ATOM   1022  OE2 GLU A 142     -13.208 -27.849  37.550  1.00190.37
O
ANISOU 1022  OE2 GLU A 142    24011  26824  21496  -2803    833
-379       O
ATOM   1023  N   GLN A 143     -17.475 -30.477  35.377  1.00147.36
N
ANISOU 1023  N   GLN A 143    18488  22277  15225  -4540    125
290       N
ATOM   1024  CA  GLN A 143     -17.648 -30.591  33.935  1.00111.69
C
ANISOU 1024  CA  GLN A 143    14169  17741  10528  -4846     27
256       C
ATOM   1025  C   GLN A 143     -18.083 -29.245  33.368  1.00110.73
C
ANISOU 1025  C   GLN A 143    13718  17970  10385  -4622     10
378       C
ATOM   1026  O   GLN A 143     -18.602 -28.385  34.082  1.00110.15
O
ANISOU 1026  O   GLN A 143    13229  18235  10389  -4331     43
556       O
ATOM   1027  CB  GLN A 143     -18.677 -31.676  33.575  1.00123.93
C
ANISOU 1027  CB  GLN A 143    15792  19479  11817  -5451   -175
429       C
ATOM   1028  CG  GLN A 143     -18.345 -33.079  34.092  1.00115.65
C
ANISOU 1028  CG  GLN A 143    15092  18077  10773  -5716   -158
330       C
ATOM   1029  CD  GLN A 143     -17.018 -33.626  33.590  1.00114.59
C
ANISOU 1029  CD  GLN A 143    15480  17315  10743  -5676     -1
```

2 C
ATOM 1030 OE1 GLN A 143 -16.422 -33.100 32.649 1.00143.96
O
ANISOU 1030 OE1 GLN A 143 19357 20867 14476 -5561 67
-163 O
ATOM 1031 NE2 GLN A 143 -16.546 -34.690 34.229 1.00114.88
N
ANISOU 1031 NE2 GLN A 143 15783 17000 10864 -5762 68
-81 N
ATOM 1032 N GLU A 144 -17.860 -29.071 32.067 1.00111.26
N
ANISOU 1032 N GLU A 144 13988 17940 10346 -4761 -27
283 N
ATOM 1033 CA GLU A 144 -18.364 -27.901 31.358 1.00112.70
C
ANISOU 1033 CA GLU A 144 13877 18467 10476 -4626 -75
435 C
ATOM 1034 C GLU A 144 -19.891 -27.874 31.322 1.00114.69
C
ANISOU 1034 C GLU A 144 13728 19307 10540 -4909 -275
825 C
ATOM 1035 O GLU A 144 -20.558 -28.912 31.284 1.00117.32
O
ANISOU 1035 O GLU A 144 14135 19755 10687 -5389 -431
942 O
ATOM 1036 CB GLU A 144 -17.794 -27.838 29.940 1.00159.57
C
ANISOU 1036 CB GLU A 144 20147 24169 16313 -4775 -86
252 C
ATOM 1037 CG GLU A 144 -16.309 -27.492 29.904 1.00161.65
C
ANISOU 1037 CG GLU A 144 20700 23932 16787 -4392 131
-91 C
ATOM 1038 CD GLU A 144 -15.709 -27.595 28.516 1.00170.30
C
ANISOU 1038 CD GLU A 144 22174 24757 17774 -4582 136
-293 C
ATOM 1039 OE1 GLU A 144 -16.173 -26.869 27.611 1.00177.41
O
ANISOU 1039 OE1 GLU A 144 22932 25925 18551 -4639 44
-173 O
ATOM 1040 OE2 GLU A 144 -14.762 -28.390 28.335 1.00173.84
O
ANISOU 1040 OE2 GLU A 144 23059 24726 18267 -4665 244
-562 O
ATOM 1041 N ALA A 145 -20.439 -26.654 31.334 1.00114.58
N
ANISOU 1041 N ALA A 145 13284 19669 10581 -4601 -258
1040 N
ATOM 1042 CA ALA A 145 -21.882 -26.447 31.418 1.00117.54
C
ANISOU 1042 CA ALA A 145 13191 20641 10827 -4765 -413
1456 C
ATOM 1043 C ALA A 145 -22.623 -26.822 30.139 1.00120.94

```
C
ANISOU 1043  C   ALA A 145    13651  21332  10969  -5299   -657
1641       C
ATOM   1044  O   ALA A 145     -23.849 -26.976  30.176  1.00155.86
O
ANISOU 1044  O   ALA A 145    17731  26250  15240  -5571   -827
2002       O
ATOM   1045  CB  ALA A 145     -22.181 -24.989  31.773  1.00116.61
C
ANISOU 1045  CB  ALA A 145    12623  20805  10879  -4238   -282
1632       C
ATOM   1046  N   ALA A 146     -21.923 -26.981  29.012  1.00129.63
N
ANISOU 1046  N   ALA A 146    15148  22128  11976  -5476   -677
1414       N
ATOM   1047  CA  ALA A 146     -22.581 -27.491  27.813  1.00136.57
C
ANISOU 1047  CA  ALA A 146    16126  23224  12539  -6069   -913
1562       C
ATOM   1048  C   ALA A 146     -22.855 -28.985  27.908  1.00141.76
C
ANISOU 1048  C   ALA A 146    17087  23783  12994  -6650  -1034
1524       C
ATOM   1049  O   ALA A 146     -23.680 -29.505  27.149  1.00149.10
O
ANISOU 1049  O   ALA A 146    18025  25002  13624  -7216  -1257
1723       O
ATOM   1050  CB  ALA A 146     -21.734 -27.190  26.576  1.00157.09
C
ANISOU 1050  CB  ALA A 146    19081  25520  15088  -6093   -877
1316       C
ATOM   1051  N   ILE A 147     -22.179 -29.679  28.825  1.00142.97
N
ANISOU 1051  N   ILE A 147    17490  23536  13297  -6534   -892
1285       N
ATOM   1052  CA  ILE A 147     -22.407 -31.103  29.040  1.00152.04
C
ANISOU 1052  CA  ILE A 147    18935  24551  14284  -7044   -978
1248       C
ATOM   1053  C   ILE A 147     -23.666 -31.323  29.869  1.00170.69
C
ANISOU 1053  C   ILE A 147    20859  27426  16570  -7206  -1123
1617       C
ATOM   1054  O   ILE A 147     -24.244 -32.418  29.841  1.00170.47
O
ANISOU 1054  O   ILE A 147    20980  27469  16322  -7752  -1270
1703       O
ATOM   1055  CB  ILE A 147     -21.173 -31.736  29.706  1.00145.35
C
ANISOU 1055  CB  ILE A 147    18503  23075  13648  -6827   -763
882       C
ATOM   1056  CG1 ILE A 147     -19.914 -31.416  28.897  1.00189.49
C
ANISOU 1056  CG1 ILE A 147    24477  28186  19335  -6618   -601
```

```
538       C
ATOM   1057  CG2 ILE A 147      -21.322 -33.247  29.822  1.00129.95
C
ANISOU 1057  CG2 ILE A 147    16927  20913  11536  -7359   -826
824       C
ATOM   1058  CD1 ILE A 147      -19.984 -31.857  27.446  1.00173.74
C
ANISOU 1058  CD1 ILE A 147    22841  26116  17056  -7135   -705
465       C
ATOM   1059  N   LEU A 148      -24.112 -30.290  30.583  1.00169.50
N
ANISOU 1059  N   LEU A 148    20179  27634  16590  -6752  -1072
1840       N
ATOM   1060  CA  LEU A 148      -25.218 -30.402  31.526  1.00138.65
C
ANISOU 1060  CA  LEU A 148    15828  24193  12660  -6807  -1156
2177       C
ATOM   1061  C   LEU A 148      -26.493 -30.898  30.854  1.00134.07
C
ANISOU 1061  C   LEU A 148    15075  24116  11750  -7427  -1439
2529       C
ATOM   1062  O   LEU A 148      -26.809 -30.530  29.719  1.00144.48
O
ANISOU 1062  O   LEU A 148    16348  25648  12900  -7635  -1570
2656       O
ATOM   1063  CB  LEU A 148      -25.474 -29.046  32.182  1.00127.95
C
ANISOU 1063  CB  LEU A 148    13948  23135  11534  -6200  -1020
2356       C
ATOM   1064  CG  LEU A 148      -26.488 -29.011  33.325  1.00129.29
C
ANISOU 1064  CG  LEU A 148    13641  23744  11740  -6129  -1031
2674       C
ATOM   1065  CD1 LEU A 148      -26.037 -29.899  34.477  1.00127.90
C
ANISOU 1065  CD1 LEU A 148    13690  23256  11651  -6137   -943
2480       C
ATOM   1066  CD2 LEU A 148      -26.713 -27.583  33.792  1.00128.11
C
ANISOU 1066  CD2 LEU A 148    13016  23853  11807  -5527   -859
2838       C
ATOM   1067  N   VAL A 149      -27.222 -31.740  31.576  1.00137.90
N
ANISOU 1067  N   VAL A 149    15460  24803  12134  -7739  -1539
2699       N
ATOM   1068  CA  VAL A 149      -28.429 -32.398  31.096  1.00154.53
C
ANISOU 1068  CA  VAL A 149    17429  27378  13908  -8393  -1815
3032       C
ATOM   1069  C   VAL A 149      -29.611 -31.910  31.926  1.00168.95
C
ANISOU 1069  C   VAL A 149    18587  29830  15777  -8245  -1868
3471       C
ATOM   1070  O   VAL A 149      -29.646 -32.129  33.140  1.00180.87
```

```
O
ANISOU 1070  O   VAL A 149    19984  31306  17434  -8041  -1762
3462       O
ATOM   1071  CB  VAL A 149     -28.310 -33.929  31.168  1.00170.84
C
ANISOU 1071  CB  VAL A 149    19993  29137  15783  -8967  -1891
2862       C
ATOM   1072  CG1 VAL A 149     -29.680 -34.567  31.083  1.00163.74
C
ANISOU 1072  CG1 VAL A 149    18848  28794  14571  -9587  -2161
3255       C
ATOM   1073  CG2 VAL A 149     -27.415 -34.434  30.047  1.00165.85
C
ANISOU 1073  CG2 VAL A 149    19995  27994  15028  -9248  -1870
2512       C
ATOM   1074  N   PRO A 150     -30.579 -31.207  31.323  1.00159.78
N
ANISOU 1074  N   PRO A 150    16957  29250  14503  -8317  -2015
3873       N
ATOM   1075  CA  PRO A 150     -31.907 -31.044  31.939  1.00182.64
C
ANISOU 1075  CA  PRO A 150    19237  32803  17354  -8370  -2117
4356       C
ATOM   1076  C   PRO A 150     -32.450 -32.280  32.647  1.00186.33
C
ANISOU 1076  C   PRO A 150    19779  33356  17661  -8840  -2233
4427       C
ATOM   1077  O   PRO A 150     -32.441 -33.379  32.085  1.00188.25
O
ANISOU 1077  O   PRO A 150    20441  33452  17634  -9469  -2402
4326       O
ATOM   1078  CB  PRO A 150     -32.783 -30.664  30.740  1.00164.84
C
ANISOU 1078  CB  PRO A 150    16690  31081  14862  -8711  -2358
4744       C
ATOM   1079  CG  PRO A 150     -31.847 -29.876  29.880  1.00164.64
C
ANISOU 1079  CG  PRO A 150    16896  30716  14942  -8399  -2251
4492       C
ATOM   1080  CD  PRO A 150     -30.549 -30.653  29.958  1.00145.96
C
ANISOU 1080  CD  PRO A 150    15236  27600  12622  -8438  -2121
3931       C
ATOM   1081  N   GLY A 151     -32.943 -32.105  33.875  1.00202.06
N
ANISOU 1081  N   GLY A 151    21379  35585  19810  -8554  -2132
4602       N
ATOM   1082  CA  GLY A 151     -33.461 -33.214  34.658  1.00210.53
C
ANISOU 1082  CA  GLY A 151    22490  36750  20753  -8954  -2226
4680       C
ATOM   1083  C   GLY A 151     -32.451 -34.138  35.311  1.00205.41
C
ANISOU 1083  C   GLY A 151    22404  35455  20186  -8969  -2105
```

```
4243       C
ATOM   1084  O   GLY A 151     -32.493 -35.354  35.103  1.00219.13
O
ANISOU 1084  O   GLY A 151    24529  37032  21699  -9551  -2248
4160       O
ATOM   1085  N   ASP A 152     -31.539 -33.578  36.102  1.00186.78
N
ANISOU 1085  N   ASP A 152    20103  32723  18144  -8346  -1837
3975       N
ATOM   1086  CA  ASP A 152     -30.515 -34.370  36.772  1.00177.72
C
ANISOU 1086  CA  ASP A 152    19448  30969  17109  -8298  -1708
3589       C
ATOM   1087  C   ASP A 152     -30.051 -33.608  38.007  1.00187.30
C
ANISOU 1087  C   ASP A 152    20445  32080  18640  -7631  -1448
3497       C
ATOM   1088  O   ASP A 152     -30.389 -32.439  38.199  1.00183.64
O
ANISOU 1088  O   ASP A 152    19534  31929  18313  -7195  -1341
3670       O
ATOM   1089  CB  ASP A 152     -29.347 -34.683  35.834  1.00160.32
C
ANISOU 1089  CB  ASP A 152    17852  28166  14898  -8374  -1669
3195       C
ATOM   1090  CG  ASP A 152     -28.642 -35.973  36.197  1.00166.53
C
ANISOU 1090  CG  ASP A 152    19193  28413  15668  -8638  -1637
2907       C
ATOM   1091  OD1 ASP A 152     -28.015 -36.578  35.302  1.00165.83
O
ANISOU 1091  OD1 ASP A 152    19622  27912  15473  -8926  -1657
2662       O
ATOM   1092  OD2 ASP A 152     -28.716 -36.386  37.374  1.00187.70
O
ANISOU 1092  OD2 ASP A 152    21799  31075  18442  -8558  -1580
2932       O
ATOM   1093  N   ILE A 153     -29.287 -34.292  38.858  1.00190.90
N
ANISOU 1093  N   ILE A 153    21228  32097  19207  -7570  -1341
3236       N
ATOM   1094  CA  ILE A 153     -28.971 -33.812  40.201  1.00179.18
C
ANISOU 1094  CA  ILE A 153    19553  30560  17966  -7069  -1127
3181       C
ATOM   1095  C   ILE A 153     -27.529 -33.322  40.231  1.00161.07
C
ANISOU 1095  C   ILE A 153    17580  27708  15910  -6601   -915
2795       C
ATOM   1096  O   ILE A 153     -26.612 -34.027  39.792  1.00165.50
O
ANISOU 1096  O   ILE A 153    18645  27766  16469  -6752   -920
2513       O
ATOM   1097  CB  ILE A 153     -29.201 -34.903  41.262  1.00165.81
```

```
C
ANISOU 1097  CB  ILE A 153    17941  28837  16224  -7339  -1169
3216       C
ATOM   1098  CG1 ILE A 153     -30.575 -35.554  41.068  1.00175.05
C
ANISOU 1098  CG1 ILE A 153    18870  30525  17118  -7905  -1411
3581       C
ATOM   1099  CG2 ILE A 153     -29.057 -34.320  42.668  1.00169.22
C
ANISOU 1099  CG2 ILE A 153    18106  29321  16869  -6858   -960
3215       C
ATOM   1100  CD1 ILE A 153     -31.174 -36.139  42.332  1.00161.64
C
ANISOU 1100  CD1 ILE A 153    16980  29038  15397  -8028  -1420
3746       C
ATOM   1101  N   VAL A 154     -27.336 -32.112  40.757  1.00140.28
N
ANISOU 1101  N   VAL A 154    14655  25165  13480  -6037   -714
2791       N
ATOM   1102  CA  VAL A 154     -26.076 -31.384  40.691  1.00124.40
C
ANISOU 1102  CA  VAL A 154    12863  22721  11682  -5566   -514
2473       C
ATOM   1103  C   VAL A 154     -25.733 -30.868  42.084  1.00122.27
C
ANISOU 1103  C   VAL A 154    12436  22412  11607  -5128   -295
2412       C
ATOM   1104  O   VAL A 154     -26.569 -30.845  42.987  1.00144.39
O
ANISOU 1104  O   VAL A 154    14900  25575  14387  -5132   -278
2637       O
ATOM   1105  CB  VAL A 154     -26.119 -30.218  39.682  1.00124.09
C
ANISOU 1105  CB  VAL A 154    12661  22817  11670  -5319   -479
2514       C
ATOM   1106  CG1 VAL A 154     -26.538 -30.715  38.307  1.00126.58
C
ANISOU 1106  CG1 VAL A 154    13112  23226  11757  -5800   -711
2604       C
ATOM   1107  CG2 VAL A 154     -27.057 -29.126  40.177  1.00125.10
C
ANISOU 1107  CG2 VAL A 154    12218  23454  11860  -5002   -380
2816       C
ATOM   1108  N   SER A 155     -24.478 -30.453  42.247  1.00118.99
N
ANISOU 1108  N   SER A 155    12276  21560  11373  -4767   -124
2105       N
ATOM   1109  CA  SER A 155     -23.977 -29.924  43.509  1.00125.49
C
ANISOU 1109  CA  SER A 155    13014  22299  12368  -4367     90
2008       C
ATOM   1110  C   SER A 155     -23.561 -28.477  43.294  1.00114.87
C
ANISOU 1110  C   SER A 155    11542  20938  11164  -3868    288
```

```
1917       C
ATOM   1111  O   SER A 155     -22.788 -28.177  42.377  1.00113.36
O
ANISOU 1111  O   SER A 155    11589  20463  11020  -3768    300
1726       O
ATOM   1112  CB  SER A 155     -22.795 -30.750  44.028  1.00116.11
C
ANISOU 1112  CB  SER A 155    12242  20608  11268  -4397    120
1743       C
ATOM   1113  OG  SER A 155     -22.360 -30.280  45.293  1.00113.34
O
ANISOU 1113  OG  SER A 155    11796  20219  11050  -4068    304
1677       O
ATOM   1114  N   ILE A 156     -24.068 -27.587  44.143  1.00115.03
N
ANISOU 1114  N   ILE A 156    11205  21252  11251  -3562    461
2050       N
ATOM   1115  CA  ILE A 156     -23.918 -26.148  43.968  1.00113.89
C
ANISOU 1115  CA  ILE A 156    10890  21158  11225  -3100    668
2021       C
ATOM   1116  C   ILE A 156     -23.228 -25.567  45.193  1.00115.46
C
ANISOU 1116  C   ILE A 156    11119  21191  11557  -2741    921
1849       C
ATOM   1117  O   ILE A 156     -23.723 -25.711  46.318  1.00179.15
O
ANISOU 1117  O   ILE A 156    19003  29458  19607  -2752    993
1961       O
ATOM   1118  CB  ILE A 156     -25.275 -25.464  43.737  1.00116.72
C
ANISOU 1118  CB  ILE A 156    10767  22048  11534  -3045    681
2378       C
ATOM   1119  CG1 ILE A 156     -25.890 -25.924  42.414  1.00118.92
C
ANISOU 1119  CG1 ILE A 156    11018  22506  11662  -3412    420
2556       C
ATOM   1120  CG2 ILE A 156     -25.114 -23.957  43.752  1.00115.70
C
ANISOU 1120  CG2 ILE A 156    10470  21940  11550  -2527    948
2351       C
ATOM   1121  CD1 ILE A 156     -27.368 -25.630  42.290  1.00122.49
C
ANISOU 1121  CD1 ILE A 156    10965  23547  12029  -3493    364
2984       C
ATOM   1122  N   LYS A 157     -22.091 -24.913  44.975  1.00111.93
N
ANISOU 1122  N   LYS A 157    10908  20392  11228  -2448   1052
1580       N
ATOM   1123  CA  LYS A 157     -21.265 -24.357  46.035  1.00125.76
C
ANISOU 1123  CA  LYS A 157    12749  21947  13087  -2144   1279
1386       C
ATOM   1124  C   LYS A 157     -21.346 -22.834  46.013  1.00116.87
```

```
C
ANISOU 1124  C   LYS A 157    11441  20919  12045  -1711   1533
1383       C
ATOM   1125  O   LYS A 157     -21.807 -22.229  45.042  1.00135.31
O
ANISOU 1125  O   LYS A 157    13634  23394  14384  -1619   1522
1495       O
ATOM   1126  CB  LYS A 157     -19.812 -24.835  45.911  1.00149.54
C
ANISOU 1126  CB  LYS A 157    16185  24467  16165  -2160   1242
1086       C
ATOM   1127  CG  LYS A 157     -19.663 -26.336  46.136  1.00149.05
C
ANISOU 1127  CG  LYS A 157    16323  24263  16046  -2547   1044
1088       C
ATOM   1128  CD  LYS A 157     -18.213 -26.792  46.141  1.00133.81
C
ANISOU 1128  CD  LYS A 157    14780  21851  14211  -2519   1045
824       C
ATOM   1129  CE  LYS A 157     -17.564 -26.614  44.781  1.00125.39
C
ANISOU 1129  CE  LYS A 157    13942  20518  13183  -2474   1006
667       C
ATOM   1130  NZ  LYS A 157     -16.168 -27.131  44.780  1.00122.77
N
ANISOU 1130  NZ  LYS A 157    13971  19721  12954  -2448   1017
436       N
ATOM   1131  N   LEU A 158     -20.903 -22.221  47.111  1.00105.39
N
ANISOU 1131  N   LEU A 158    10000  19392  10651  -1459   1769
1263       N
ATOM   1132  CA  LEU A 158     -20.932 -20.767  47.236  1.00105.16
C
ANISOU 1132  CA  LEU A 158     9843  19414  10698  -1047   2057
1237       C
ATOM   1133  C   LEU A 158     -20.117 -20.101  46.132  1.00111.63
C
ANISOU 1133  C   LEU A 158    10859  19965  11592   -858   2070
1060       C
ATOM   1134  O   LEU A 158     -18.952 -20.441  45.908  1.00159.75
O
ANISOU 1134  O   LEU A 158    17281  25702  17714   -903   1999
817       O
ATOM   1135  CB  LEU A 158     -20.408 -20.348  48.613  1.00119.34
C
ANISOU 1135  CB  LEU A 158    11714  21115  12515   -881   2297
1087       C
ATOM   1136  CG  LEU A 158     -20.651 -18.910  49.090  1.00139.53
C
ANISOU 1136  CG  LEU A 158    14126  23766  15122   -492   2652
1084       C
ATOM   1137  CD1 LEU A 158     -20.664 -18.865  50.611  1.00144.16
C
ANISOU 1137  CD1 LEU A 158    14699  24414  15661   -485   2845
```

```
1046       C
ATOM   1138  CD2 LEU A 158     -19.612 -17.929  48.547  1.00102.52
C
ANISOU 1138  CD2 LEU A 158     9673  18764  10517   -211   2787
841       C
ATOM   1139  N   GLY A 159     -20.743 -19.147  45.443  1.00117.90
N
ANISOU 1139  N   GLY A 159    11438  20940  12420   -641   2167
1203       N
ATOM   1140  CA  GLY A 159     -20.138 -18.435  44.336  1.00106.16
C
ANISOU 1140  CA  GLY A 159    10092  19251  10994   -460   2182
1080       C
ATOM   1141  C   GLY A 159     -20.143 -19.157  43.005  1.00114.78
C
ANISOU 1141  C   GLY A 159    11279  20305  12029   -737   1889
1118       C
ATOM   1142  O   GLY A 159     -19.567 -18.638  42.040  1.00125.23
O
ANISOU 1142  O   GLY A 159    12748  21442  13391   -617   1886
999       O
ATOM   1143  N   ASP A 160     -20.766 -20.330  42.918  1.00126.47
N
ANISOU 1143  N   ASP A 160    12699  21950  13404  -1121   1651
1273       N
ATOM   1144  CA  ASP A 160     -21.048 -20.932  41.622  1.00140.46
C
ANISOU 1144  CA  ASP A 160    14516  23767  15085  -1418   1391
1364       C
ATOM   1145  C   ASP A 160     -21.999 -20.046  40.825  1.00182.34
C
ANISOU 1145  C   ASP A 160    19490  29407  20384  -1284   1410
1637       C
ATOM   1146  O   ASP A 160     -22.865 -19.367  41.383  1.00208.12
O
ANISOU 1146  O   ASP A 160    22402  32985  23688  -1075   1570
1864       O
ATOM   1147  CB  ASP A 160     -21.648 -22.326  41.801  1.00135.83
C
ANISOU 1147  CB  ASP A 160    13917  23323  14369  -1876   1155
1501       C
ATOM   1148  CG  ASP A 160     -20.615 -23.355  42.215  1.00143.53
C
ANISOU 1148  CG  ASP A 160    15276  23908  15350  -2058   1088
1241       C
ATOM   1149  OD1 ASP A 160     -21.012 -24.485  42.570  1.00149.19
O
ANISOU 1149  OD1 ASP A 160    16012  24695  15976  -2403    934
1331       O
ATOM   1150  OD2 ASP A 160     -19.408 -23.032  42.187  1.00117.78
O
ANISOU 1150  OD2 ASP A 160    12288  20274  12187  -1855   1192
964       O
ATOM   1151  N   ILE A 161     -21.836 -20.050  39.505  1.00178.65
```

```
N
ANISOU 1151  N   ILE A 161    19133  28875  19869  -1402   1256
1628       N
ATOM   1152  CA  ILE A 161     -22.844 -19.494  38.610  1.00172.73
C
ANISOU 1152  CA  ILE A 161    18060  28494  19077  -1397   1189
1949       C
ATOM   1153  C   ILE A 161     -23.837 -20.595  38.260  1.00171.55
C
ANISOU 1153  C   ILE A 161    17764  28667  18748  -1889    903
2214       C
ATOM   1154  O   ILE A 161     -23.459 -21.632  37.704  1.00162.94
O
ANISOU 1154  O   ILE A 161    16974  27396  17540  -2277    688
2084       O
ATOM   1155  CB  ILE A 161     -22.204 -18.901  37.346  1.00183.31
C
ANISOU 1155  CB  ILE A 161    19583  29633  20433  -1302   1160
1825       C
ATOM   1156  CG1 ILE A 161     -21.109 -17.902  37.723  1.00186.64
C
ANISOU 1156  CG1 ILE A 161    20199  29700  21016   -858   1434
1531       C
ATOM   1157  CG2 ILE A 161     -23.260 -18.232  36.482  1.00199.41
C
ANISOU 1157  CG2 ILE A 161    21252  32078  22438  -1268   1101
2195       C
ATOM   1158  CD1 ILE A 161     -20.293 -17.421  36.543  1.00214.66
C
ANISOU 1158  CD1 ILE A 161    23990  32991  24580   -783   1406
1354       C
ATOM   1159  N   ILE A 162     -25.103 -20.369  38.593  1.00178.85
N
ANISOU 1159  N   ILE A 162    18237  30066  19651  -1877    915
2590       N
ATOM   1160  CA  ILE A 162     -26.153 -21.356  38.320  1.00181.63
C
ANISOU 1160  CA  ILE A 162    18402  30789  19820  -2358    643
2887       C
ATOM   1161  C   ILE A 162     -26.306 -21.538  36.814  1.00185.31
C
ANISOU 1161  C   ILE A 162    18931  31335  20144  -2662    394
2986       C
ATOM   1162  O   ILE A 162     -26.403 -20.538  36.076  1.00201.65
O
ANISOU 1162  O   ILE A 162    20849  33499  22270  -2419    451
3100       O
ATOM   1163  CB  ILE A 162     -27.482 -20.916  38.956  1.00194.48
C
ANISOU 1163  CB  ILE A 162    19483  32940  21473  -2229    736
3302       C
ATOM   1164  CG1 ILE A 162     -27.317 -20.723  40.464  1.00185.98
C
ANISOU 1164  CG1 ILE A 162    18372  31775  20517  -1947   1004
```

```
3185       C
ATOM   1165  CG2 ILE A 162     -28.564 -21.944  38.688  1.00224.80
C
ANISOU 1165  CG2 ILE A 162    23116  37189  25108  -2754    443
3623       C
ATOM   1166  CD1 ILE A 162     -26.834 -21.958  41.188  1.00190.67
C
ANISOU 1166  CD1 ILE A 162    19268  32143  21033  -2264    896
2963       C
ATOM   1167  N   PRO A 163     -26.335 -22.766  36.300  1.00176.78
N
ANISOU 1167  N   PRO A 163    18084  30216  18870  -3199    125
2951       N
ATOM   1168  CA  PRO A 163     -26.094 -22.958  34.864  1.00167.07
C
ANISOU 1168  CA  PRO A 163    17076  28904  17500  -3487    -73
2908       C
ATOM   1169  C   PRO A 163     -27.396 -23.198  34.117  1.00143.57
C
ANISOU 1169  C   PRO A 163    13758  26472  14318  -3883   -325
3349       C
ATOM   1170  O   PRO A 163     -27.444 -23.146  32.884  1.00127.87
O
ANISOU 1170  O   PRO A 163    11843  24545  12197  -4112   -491
3413       O
ATOM   1171  CB  PRO A 163     -25.201 -24.204  34.829  1.00181.39
C
ANISOU 1171  CB  PRO A 163    19425  30267  19229  -3834   -169
2555       C
ATOM   1172  CG  PRO A 163     -25.678 -24.986  36.014  1.00173.00
C
ANISOU 1172  CG  PRO A 163    18258  29324  18150  -3977   -176
2636       C
ATOM   1173  CD  PRO A 163     -25.935 -23.951  37.077  1.00178.13
C
ANISOU 1173  CD  PRO A 163    18543  30143  18995  -3459     78
2743       C
ATOM   1174  N   ALA A 164     -28.458 -23.465  34.874  1.00150.66
N
ANISOU 1174  N   ALA A 164    14278  27785  15181  -3980   -357
3665       N
ATOM   1175  CA  ALA A 164     -29.739 -23.893  34.329  1.00168.15
C
ANISOU 1175  CA  ALA A 164    16158  30553  17179  -4429   -620
4106       C
ATOM   1176  C   ALA A 164     -30.811 -23.587  35.363  1.00140.09
C
ANISOU 1176  C   ALA A 164    12066  27456  13706  -4238   -514
4468       C
ATOM   1177  O   ALA A 164     -30.512 -23.226  36.504  1.00142.37
O
ANISOU 1177  O   ALA A 164    12320  27587  14186  -3835   -253
4329       O
ATOM   1178  CB  ALA A 164     -29.729 -25.380  33.962  1.00189.10
```

```
C
ANISOU 1178  CB  ALA A 164    19170  33111  19570  -5110   -891
4005       C
ATOM   1179  N   ASP A 165     -32.070 -23.731  34.955  1.00148.01
N
ANISOU 1179  N   ASP A 165    12644  29040  14552  -4545   -714
4945       N
ATOM   1180  CA  ASP A 165     -33.138 -23.823  35.939  1.00142.30
C
ANISOU 1180  CA  ASP A 165    11456  28762  13851  -4520   -665
5288       C
ATOM   1181  C   ASP A 165     -32.909 -25.043  36.821  1.00141.32
C
ANISOU 1181  C   ASP A 165    11624  28431  13640  -4835   -717
5059       C
ATOM   1182  O   ASP A 165     -32.494 -26.101  36.343  1.00140.98
O
ANISOU 1182  O   ASP A 165    12008  28149  13409  -5324   -930
4851       O
ATOM   1183  CB  ASP A 165     -34.498 -23.917  35.246  1.00161.31
C
ANISOU 1183  CB  ASP A 165    13377  31844  16070  -4885   -918
5855       C
ATOM   1184  CG  ASP A 165     -34.879 -22.637  34.528  1.00183.26
C
ANISOU 1184  CG  ASP A 165    15763  34900  18968  -4517   -842
6174       C
ATOM   1185  OD1 ASP A 165     -34.267 -21.588  34.817  1.00193.41
O
ANISOU 1185  OD1 ASP A 165    17072  35906  20510  -3911   -537
5997       O
ATOM   1186  OD2 ASP A 165     -35.790 -22.680  33.674  1.00179.52
O
ANISOU 1186  OD2 ASP A 165    14958  34928  18323  -4846  -1088
6614       O
ATOM   1187  N   ALA A 166     -33.181 -24.900  38.117  1.00161.61
N
ANISOU 1187  N   ALA A 166    13976  31084  16346  -4559   -509
5102       N
ATOM   1188  CA  ALA A 166     -32.967 -26.012  39.029  1.00160.29
C
ANISOU 1188  CA  ALA A 166    14064  30729  16111  -4830   -547
4904       C
ATOM   1189  C   ALA A 166     -33.913 -25.921  40.216  1.00149.71
C
ANISOU 1189  C   ALA A 166    12269  29789  14825  -4701   -417
5192       C
ATOM   1190  O   ALA A 166     -34.317 -24.830  40.628  1.00148.57
O
ANISOU 1190  O   ALA A 166    11730  29863  14859  -4216   -168
5382       O
ATOM   1191  CB  ALA A 166     -31.516 -26.057  39.526  1.00188.26
C
ANISOU 1191  CB  ALA A 166    18125  33604  19800  -4570   -367
```

```
4371       C
ATOM   1192  N   ARG A 167     -34.253 -27.084  40.765  1.00143.29
N
ANISOU 1192  N   ARG A 167    11532  29054  13857  -5142   -572
5219       N
ATOM   1193  CA  ARG A 167     -35.007 -27.194  42.008  1.00144.80
C
ANISOU 1193  CA  ARG A 167    11381  29555  14083  -5073   -451
5422       C
ATOM   1194  C   ARG A 167     -34.059 -27.656  43.106  1.00160.06
C
ANISOU 1194  C   ARG A 167    13710  31005  16102  -4967   -304
5012       C
ATOM   1195  O   ARG A 167     -33.414 -28.702  42.970  1.00145.62
O
ANISOU 1195  O   ARG A 167    12342  28822  14163  -5333   -472
4751       O
ATOM   1196  CB  ARG A 167     -36.179 -28.168  41.881  1.00152.94
C
ANISOU 1196  CB  ARG A 167    12163  31085  14861  -5672   -741
5801       C
ATOM   1197  CG  ARG A 167     -36.542 -28.830  43.210  1.00203.34
C
ANISOU 1197  CG  ARG A 167    18468  37566  21225  -5779   -681
5828       C
ATOM   1198  CD  ARG A 167     -37.814 -29.659  43.131  1.00247.53
C
ANISOU 1198  CD  ARG A 167    23743  43728  26581  -6339   -947
6254       C
ATOM   1199  NE  ARG A 167     -38.002 -30.462  44.338  1.00246.01
N
ANISOU 1199  NE  ARG A 167    23585  43544  26345  -6512   -921
6218       N
ATOM   1200  CZ  ARG A 167     -37.377 -31.609  44.587  1.00248.92
C
ANISOU 1200  CZ  ARG A 167    24451  43521  26604  -6880  -1052
5921       C
ATOM   1201  NH1 ARG A 167     -37.587 -32.241  45.733  1.00246.06
N
ANISOU 1201  NH1 ARG A 167    24078  43195  26219  -6997  -1012
5921       N
ATOM   1202  NH2 ARG A 167     -36.519 -32.110  43.708  1.00266.69
N
ANISOU 1202  NH2 ARG A 167    27220  45329  28781  -7115  -1203
5626       N
ATOM   1203  N   LEU A 168     -33.960 -26.872  44.176  1.00167.50
N
ANISOU 1203  N   LEU A 168    14482  31924  17237  -4473     18
4962       N
ATOM   1204  CA  LEU A 168     -33.125 -27.261  45.304  1.00137.42
C
ANISOU 1204  CA  LEU A 168    10999  27718  13497  -4381    157
4619       C
ATOM   1205  C   LEU A 168     -33.631 -28.553  45.934  1.00139.13
```

```
C
ANISOU 1205  C   LEU A 168    11237  28083  13543  -4874    -30
4718       C
ATOM   1206  O   LEU A 168     -34.838 -28.772  46.065  1.00142.68
O
ANISOU 1206  O   LEU A 168    11277  29053  13883  -5094   -120
5104       O
ATOM   1207  CB  LEU A 168     -33.102 -26.149  46.349  1.00136.59
C
ANISOU 1207  CB  LEU A 168    10667  27640  13590  -3810    543
4597       C
ATOM   1208  CG  LEU A 168     -32.729 -24.762  45.836  1.00135.37
C
ANISOU 1208  CG  LEU A 168    10446  27374  13613  -3288    773
4537       C
ATOM   1209  CD1 LEU A 168     -32.686 -23.796  46.995  1.00134.77
C
ANISOU 1209  CD1 LEU A 168    10217  27284  13706  -2785   1175
4483       C
ATOM   1210  CD2 LEU A 168     -31.390 -24.796  45.111  1.00153.92
C
ANISOU 1210  CD2 LEU A 168    13310  29176  15997  -3263    706
4138       C
ATOM   1211  N   LEU A 169     -32.692 -29.414  46.321  1.00136.73
N
ANISOU 1211  N   LEU A 169    11409  27320  13221  -5047    -87
4384       N
ATOM   1212  CA  LEU A 169     -33.016 -30.793  46.704  1.00138.23
C
ANISOU 1212  CA  LEU A 169    11732  27554  13234  -5581   -305
4440       C
ATOM   1213  C   LEU A 169     -33.239 -30.875  48.212  1.00138.27
C
ANISOU 1213  C   LEU A 169    11582  27666  13289  -5460   -134
4470       C
ATOM   1214  O   LEU A 169     -34.357 -31.124  48.673  1.00158.90
O
ANISOU 1214  O   LEU A 169    13823  30750  15801  -5651   -177
4794       O
ATOM   1215  CB  LEU A 169     -31.908 -31.746  46.250  1.00145.65
C
ANISOU 1215  CB  LEU A 169    13279  27929  14132  -5850   -454
4093       C
ATOM   1216  CG  LEU A 169     -32.101 -33.221  46.609  1.00159.50
C
ANISOU 1216  CG  LEU A 169    15255  29632  15716  -6399   -662
4113       C
ATOM   1217  CD1 LEU A 169     -33.420 -33.747  46.056  1.00141.78
C
ANISOU 1217  CD1 LEU A 169    12729  27907  13234  -6899   -909
4496       C
ATOM   1218  CD2 LEU A 169     -30.929 -34.060  46.114  1.00160.87
C
ANISOU 1218  CD2 LEU A 169    16042  29194  15887  -6590   -752
```

```
3764       C
ATOM   1219  N   GLU A 170     -32.176 -30.666  48.983  1.00135.03
N
ANISOU 1219  N   GLU A 170    11450  26832  13022  -5158     59
4142       N
ATOM   1220  CA  GLU A 170     -32.240 -30.682  50.438  1.00134.82
C
ANISOU 1220  CA  GLU A 170    11326  26862  13038  -5033    239
4130       C
ATOM   1221  C   GLU A 170     -31.003 -29.973  50.973  1.00164.59
C
ANISOU 1221  C   GLU A 170    15357  30194  16987  -4587    490
3777       C
ATOM   1222  O   GLU A 170     -30.042 -29.723  50.243  1.00155.76
O
ANISOU 1222  O   GLU A 170    14541  28692  15949  -4447    482
3530       O
ATOM   1223  CB  GLU A 170     -32.367 -32.110  50.982  1.00135.83
C
ANISOU 1223  CB  GLU A 170    11636  26955  13020  -5530     31
4154       C
ATOM   1224  CG  GLU A 170     -31.154 -32.995  50.765  1.00143.95
C
ANISOU 1224  CG  GLU A 170    13231  27404  14058  -5710    -96
3831       C
ATOM   1225  CD  GLU A 170     -31.423 -34.437  51.154  1.00156.36
C
ANISOU 1225  CD  GLU A 170    14969  28963  15476  -6235   -312
3905       C
ATOM   1226  OE1 GLU A 170     -31.737 -34.688  52.336  1.00135.73
O
ANISOU 1226  OE1 GLU A 170    12222  26505  12844  -6268   -245
3989       O
ATOM   1227  OE2 GLU A 170     -31.329 -35.319  50.275  1.00197.04
O
ANISOU 1227  OE2 GLU A 170    20401  33945  20518  -6626   -539
3879       O
ATOM   1228  N   GLY A 171     -31.038 -29.658  52.262  1.00160.35
N
ANISOU 1228  N   GLY A 171    14702  29727  16497  -4389    713
3759       N
ATOM   1229  CA  GLY A 171     -30.019 -28.849  52.897  1.00138.19
C
ANISOU 1229  CA  GLY A 171    12078  26594  13834  -3973    980
3469       C
ATOM   1230  C   GLY A 171     -30.492 -27.429  53.162  1.00140.98
C
ANISOU 1230  C   GLY A 171    12091  27190  14285  -3512   1312
3560       C
ATOM   1231  O   GLY A 171     -31.520 -26.968  52.660  1.00175.24
O
ANISOU 1231  O   GLY A 171    16050  31917  18616  -3452   1335
3849       O
ATOM   1232  N   ASP A 172     -29.708 -26.734  53.981  1.00137.52
```

```
N
ANISOU 1232  N   ASP A 172    11803  26510  13938  -3188   1580
3319       N
ATOM   1233  CA  ASP A 172     -30.117 -25.438  54.508  1.00152.95
C
ANISOU 1233  CA  ASP A 172    13489  28651  15974  -2761   1954
3375       C
ATOM   1234  C   ASP A 172     -30.366 -24.416  53.398  1.00129.18
C
ANISOU 1234  C   ASP A 172    10308  25709  13066  -2455   2042
3463       C
ATOM   1235  O   ASP A 172     -29.678 -24.426  52.370  1.00126.19
O
ANISOU 1235  O   ASP A 172    10162  25061  12725  -2457   1888
3319       O
ATOM   1236  CB  ASP A 172     -29.054 -24.911  55.473  1.00191.23
C
ANISOU 1236  CB  ASP A 172    18619  33163  20876  -2521   2204
3053       C
ATOM   1237  CG  ASP A 172     -28.978 -25.721  56.752  1.00220.58
C
ANISOU 1237  CG  ASP A 172    22422  36899  24491  -2777   2179
3023       C
ATOM   1238  OD1 ASP A 172     -28.155 -25.379  57.628  1.00230.33
O
ANISOU 1238  OD1 ASP A 172    23875  37903  25738  -2644   2361
2790       O
ATOM   1239  OD2 ASP A 172     -29.742 -26.701  56.882  1.00230.89
O
ANISOU 1239  OD2 ASP A 172    23577  38458  25691  -3131   1973
3242       O
ATOM   1240  N   PRO A 173     -31.363 -23.533  53.574  1.00130.74
N
ANISOU 1240  N   PRO A 173    10091  26270  13313  -2186   2297
3717       N
ATOM   1241  CA  PRO A 173     -31.512 -22.329  52.737  1.00131.15
C
ANISOU 1241  CA  PRO A 173     9982  26352  13495  -1792   2474
3790       C
ATOM   1242  C   PRO A 173     -30.230 -21.582  52.394  1.00145.15
C
ANISOU 1242  C   PRO A 173    12131  27646  15373  -1500   2595
3428       C
ATOM   1243  O   PRO A 173     -29.312 -21.490  53.216  1.00138.23
O
ANISOU 1243  O   PRO A 173    11561  26457  14503  -1426   2732
3124       O
ATOM   1244  CB  PRO A 173     -32.448 -21.450  53.577  1.00134.04
C
ANISOU 1244  CB  PRO A 173     9962  27046  13921  -1475   2866
4005       C
ATOM   1245  CG  PRO A 173     -33.281 -22.437  54.327  1.00173.82
C
ANISOU 1245  CG  PRO A 173    14793  32424  18826  -1834   2741
```

```
4220       C
ATOM   1246  CD  PRO A 173     -32.328 -23.547  54.686  1.00142.09
C
ANISOU 1246  CD  PRO A 173    11208  28078  14703  -2197   2484
3933       C
ATOM   1247  N   LEU A 174     -30.163 -21.047  51.175  1.00171.65
N
ANISOU 1247  N   LEU A 174    15458  30960  18802  -1352   2535
3474       N
ATOM   1248  CA  LEU A 174     -29.003 -20.307  50.696  1.00124.47
C
ANISOU 1248  CA  LEU A 174     9815  24555  12925  -1082   2634
3159       C
ATOM   1249  C   LEU A 174     -29.512 -19.093  49.928  1.00126.02
C
ANISOU 1249  C   LEU A 174     9743  24895  13241   -702   2826
3347       C
ATOM   1250  O   LEU A 174     -30.685 -19.025  49.550  1.00187.40
O
ANISOU 1250  O   LEU A 174    17096  33091  21015   -716   2794
3732       O
ATOM   1251  CB  LEU A 174     -28.084 -21.187  49.830  1.00121.91
C
ANISOU 1251  CB  LEU A 174     9858  23915  12547  -1384   2279
2953       C
ATOM   1252  CG  LEU A 174     -28.405 -21.575  48.378  1.00122.71
C
ANISOU 1252  CG  LEU A 174     9899  24119  12605  -1602   1970
3117       C
ATOM   1253  CD1 LEU A 174     -29.826 -22.097  48.201  1.00126.48
C
ANISOU 1253  CD1 LEU A 174     9955  25118  12985  -1868   1809
3542       C
ATOM   1254  CD2 LEU A 174     -28.066 -20.481  47.372  1.00121.90
C
ANISOU 1254  CD2 LEU A 174     9815  23887  12613  -1269   2073
3071       C
ATOM   1255  N   LYS A 175     -28.626 -18.128  49.694  1.00123.87
N
ANISOU 1255  N   LYS A 175     9709  24280  13074   -364   3026
3093       N
ATOM   1256  CA  LYS A 175     -28.991 -16.877  49.038  1.00125.21
C
ANISOU 1256  CA  LYS A 175     9670  24526  13376     41   3252
3244       C
ATOM   1257  C   LYS A 175     -28.402 -16.793  47.636  1.00123.51
C
ANISOU 1257  C   LYS A 175     9626  24118  13184     14   3022
3167       C
ATOM   1258  O   LYS A 175     -27.199 -17.002  47.448  1.00137.53
O
ANISOU 1258  O   LYS A 175    11821  25490  14945    -55   2925
2816       O
ATOM   1259  CB  LYS A 175     -28.519 -15.674  49.861  1.00124.55
```

```
C
ANISOU 1259  CB  LYS A 175     9720  24210  13395    477   3716
3031       C
ATOM   1260  CG  LYS A 175     -29.071 -15.614  51.276  1.00126.42
C
ANISOU 1260  CG  LYS A 175     9815  24615  13603    532   4005
3084       C
ATOM   1261  CD  LYS A 175     -28.881 -14.227  51.877  1.00126.89
C
ANISOU 1261  CD  LYS A 175     9941  24504  13768   1000   4516
2955       C
ATOM   1262  CE  LYS A 175     -29.353 -14.171  53.323  1.00133.81
C
ANISOU 1262  CE  LYS A 175    10731  25516  14594   1030   4829
2967       C
ATOM   1263  NZ  LYS A 175     -29.299 -12.787  53.871  1.00129.90
N
ANISOU 1263  NZ  LYS A 175    10339  24807  14210   1471   5332
2846       N
ATOM   1264  N   VAL A 176     -29.255 -16.485  46.658  1.00125.98
N
ANISOU 1264  N   VAL A 176     9602  24734  13529     62   2940
3511       N
ATOM   1265  CA  VAL A 176     -28.873 -16.320  45.261  1.00125.03
C
ANISOU 1265  CA  VAL A 176     9586  24498  13420     35   2733
3499       C
ATOM   1266  C   VAL A 176     -29.129 -14.871  44.867  1.00126.33
C
ANISOU 1266  C   VAL A 176     9554  24698  13748    529   3039
3641       C
ATOM   1267  O   VAL A 176     -30.053 -14.227  45.372  1.00157.30
O
ANISOU 1267  O   VAL A 176    13107  28901  17758    800   3317
3922       O
ATOM   1268  CB  VAL A 176     -29.622 -17.281  44.310  1.00137.35
C
ANISOU 1268  CB  VAL A 176    10947  26394  14847   -407   2321
3799       C
ATOM   1269  CG1 VAL A 176     -29.401 -18.721  44.714  1.00139.85
C
ANISOU 1269  CG1 VAL A 176    11478  26654  15005   -894   2044
3664       C
ATOM   1270  CG2 VAL A 176     -31.111 -16.956  44.274  1.00195.36
C
ANISOU 1270  CG2 VAL A 176    17710  34298  22219   -328   2384
4318       C
ATOM   1271  N   ASP A 177     -28.301 -14.357  43.960  1.00124.25
N
ANISOU 1271  N   ASP A 177     9541  24136  13531    654   3004
3453       N
ATOM   1272  CA  ASP A 177     -28.401 -12.975  43.497  1.00126.01
C
ANISOU 1272  CA  ASP A 177     9638  24328  13912   1119   3283
```

```
3558       C
ATOM   1273  C   ASP A 177     -29.026 -12.974  42.103  1.00129.33
C
ANISOU 1273  C   ASP A 177     9792  25031  14316   1009   3019
3905       C
ATOM   1274  O   ASP A 177     -28.426 -13.468  41.144  1.00125.44
O
ANISOU 1274  O   ASP A 177     9541  24387  13732    739   2710
3766       O
ATOM   1275  CB  ASP A 177     -27.026 -12.308  43.493  1.00144.39
C
ANISOU 1275  CB  ASP A 177    12432  26133  16299   1344   3448
3112       C
ATOM   1276  CG  ASP A 177     -27.088 -10.832  43.136  1.00152.54
C
ANISOU 1276  CG  ASP A 177    13374  27090  17496   1840   3781
3195       C
ATOM   1277  OD1 ASP A 177     -28.200 -10.311  42.913  1.00176.62
O
ANISOU 1277  OD1 ASP A 177    15986  30488  20632   2039   3901
3613       O
ATOM   1278  OD2 ASP A 177     -26.017 -10.193  43.070  1.00144.64
O
ANISOU 1278  OD2 ASP A 177    12736  25682  16540   2030   3926
2856       O
ATOM   1279  N   GLN A 178     -30.233 -12.415  41.996  1.00157.50
N
ANISOU 1279  N   GLN A 178    12858  29016  17968   1212   3149
4371       N
ATOM   1280  CA  GLN A 178     -30.945 -12.296  40.731  1.00133.80
C
ANISOU 1280  CA  GLN A 178     9533  26349  14956   1131   2922
4775       C
ATOM   1281  C   GLN A 178     -30.761 -10.934  40.067  1.00134.21
C
ANISOU 1281  C   GLN A 178     9545  26268  15179   1596   3158
4843       C
ATOM   1282  O   GLN A 178     -31.443 -10.645  39.079  1.00136.89
O
ANISOU 1282  O   GLN A 178     9556  26919  15539   1604   3025
5241       O
ATOM   1283  CB  GLN A 178     -32.441 -12.550  40.946  1.00138.41
C
ANISOU 1283  CB  GLN A 178     9532  27528  15529   1050   2891
5318       C
ATOM   1284  CG  GLN A 178     -32.820 -13.954  41.398  1.00138.75
C
ANISOU 1284  CG  GLN A 178     9548  27791  15380    526   2597
5345       C
ATOM   1285  CD  GLN A 178     -32.253 -15.043  40.512  1.00158.22
C
ANISOU 1285  CD  GLN A 178    12336  30139  17641    -15   2134
5162       C
ATOM   1286  OE1 GLN A 178     -31.700 -16.027  41.001  1.00155.13
```

O
ANISOU 1286 OE1 GLN A 178   12272 29539 17131   -334   1991
4856   O
ATOM   1287 NE2 GLN A 178   -32.392 -14.877 39.202 1.00207.64
N
ANISOU 1287 NE2 GLN A 178   18511 36527 23858   -124   1909
5356   N
ATOM   1288 N   SER A 179   -29.869 -10.094 40.587 1.00131.81
N
ANISOU 1288 N   SER A 179   9569 25523 14992   1967   3502
4482   N
ATOM   1289 CA SER A 179   -29.605 -8.796 39.977 1.00145.48
C
ANISOU 1289 CA SER A 179   11318 27075 16881   2402   3740
4511   C
ATOM   1290 C   SER A 179   -29.143 -8.936 38.529 1.00143.41
C
ANISOU 1290 C   SER A 179   11193 26752 16543   2190   3388
4496   C
ATOM   1291 O   SER A 179   -28.502 -9.921 38.152 1.00161.80
O
ANISOU 1291 O   SER A 179   13821 28949 18706   1762   3044
4242   O
ATOM   1292 CB SER A 179   -28.555 -8.029 40.780 1.00153.32
C
ANISOU 1292 CB SER A 179   12735 27560 17960   2731   4121
4051   C
ATOM   1293 OG SER A 179   -27.351 -8.768 40.886 1.00150.89
O
ANISOU 1293 OG SER A 179   12916 26896 17519   2427   3913
3573   O
ATOM   1294 N   ALA A 180   -29.524 -7.941 37.717 1.00133.00
N
ANISOU 1294 N   ALA A 180   9640 25543 15351   2495   3491
4795   N
ATOM   1295 CA ALA A 180   -29.351 -7.888 36.264 1.00155.52
C
ANISOU 1295 CA ALA A 180   12510 28438 18144   2338   3188
4907   C
ATOM   1296 C   ALA A 180   -30.374 -8.741 35.523 1.00171.70
C
ANISOU 1296 C   ALA A 180   14169 31022 20046   1906   2781
5356   C
ATOM   1297 O   ALA A 180   -31.041 -8.254 34.604 1.00193.41
O
ANISOU 1297 O   ALA A 180   16574 34078 22835   1976   2697
5787   O
ATOM   1298 CB ALA A 180   -27.929 -8.285 35.846 1.00128.33
C
ANISOU 1298 CB ALA A 180   9650 24523 14586   2110   3006
4369   C
ATOM   1299 N   LEU A 181   -30.498 -10.013 35.906 1.00159.38
N
ANISOU 1299 N   LEU A 181   12667 29582 18309   1442   2522

```
5272       N
ATOM   1300  CA  LEU A 181      -31.517 -10.873  35.309  1.00186.35
C
ANISOU 1300  CA  LEU A 181    15723  33523  21560    987   2145
5698       C
ATOM   1301  C   LEU A 181      -32.923 -10.370  35.617  1.00173.58
C
ANISOU 1301  C   LEU A 181    13456  32430  20065   1233   2311
6295       C
ATOM   1302  O   LEU A 181      -33.772 -10.279  34.722  1.00147.09
O
ANISOU 1302  O   LEU A 181     9696  29517  16674   1116   2110
6789       O
ATOM   1303  CB  LEU A 181      -31.333 -12.312  35.792  1.00137.07
C
ANISOU 1303  CB  LEU A 181     9710  27255  15114    466   1885
5461       C
ATOM   1304  CG  LEU A 181      -29.927 -12.883  35.592  1.00132.41
C
ANISOU 1304  CG  LEU A 181     9756  26132  14423    241   1751
4879       C
ATOM   1305  CD1 LEU A 181      -29.849 -14.311  36.105  1.00131.44
C
ANISOU 1305  CD1 LEU A 181     9822  25999  14119   -252   1518
4702       C
ATOM   1306  CD2 LEU A 181      -29.512 -12.808  34.129  1.00131.97
C
ANISOU 1306  CD2 LEU A 181     9864  26007  14273     53   1496
4865       C
ATOM   1307  N   THR A 182      -33.189 -10.033  36.879  1.00144.06
N
ANISOU 1307  N   THR A 182     9603  28662  16470   1570   2687
6275       N
ATOM   1308  CA  THR A 182      -34.465  -9.445  37.259  1.00156.99
C
ANISOU 1308  CA  THR A 182    10637  30749  18263   1885   2930
6823       C
ATOM   1309  C   THR A 182      -34.347  -8.014  37.765  1.00161.65
C
ANISOU 1309  C   THR A 182    11236  31055  19129   2574   3463
6789       C
ATOM   1310  O   THR A 182      -35.378  -7.363  37.968  1.00213.45
O
ANISOU 1310  O   THR A 182    17553  37712  25834   2866   3597
7153       O
ATOM   1311  CB  THR A 182      -35.153 -10.309  38.329  1.00182.75
C
ANISOU 1311  CB  THR A 182    13701  34287  21448   1665   2925
6913       C
ATOM   1312  OG1 THR A 182      -36.574 -10.134  38.251  1.00221.00
O
ANISOU 1312  OG1 THR A 182    18042  39583  26343   1728   2880
7498       O
ATOM   1313  CG2 THR A 182      -34.671  -9.934  39.725  1.00169.28
```

```
C
ANISOU 1313  CG2 THR A 182    12242  32215  19863   2014   3372
6539       C
ATOM   1314  N   GLY A 183     -33.133  -7.505  37.958  1.00158.63
N
ANISOU 1314  N   GLY A 183    11337  30134  18801   2795   3681
6285       N
ATOM   1315  CA  GLY A 183     -32.962  -6.113  38.330  1.00157.08
C
ANISOU 1315  CA  GLY A 183    11320  29525  18840   3381   4118
6183       C
ATOM   1316  C   GLY A 183     -33.314  -5.765  39.760  1.00186.22
C
ANISOU 1316  C   GLY A 183    15140  32972  22642   3634   4462
6047       C
ATOM   1317  O   GLY A 183     -33.692  -4.620  40.030  1.00203.50
O
ANISOU 1317  O   GLY A 183    17371  34918  25033   4068   4772
6133       O
ATOM   1318  N   GLU A 184     -33.204  -6.712  40.691  1.00188.31
N
ANISOU 1318  N   GLU A 184    15478  33294  22777   3356   4421
5838       N
ATOM   1319  CA  GLU A 184     -33.445  -6.436  42.103  1.00146.66
C
ANISOU 1319  CA  GLU A 184    10356  27784  17584   3551   4747
5665       C
ATOM   1320  C   GLU A 184     -32.153  -6.676  42.872  1.00151.60
C
ANISOU 1320  C   GLU A 184    11474  28004  18123   3471   4878
5071       C
ATOM   1321  O   GLU A 184     -31.598  -7.780  42.839  1.00138.99
O
ANISOU 1321  O   GLU A 184     9929  26548  16333   3078   4637
4907       O
ATOM   1322  CB  GLU A 184     -34.589  -7.295  42.646  1.00149.69
C
ANISOU 1322  CB  GLU A 184    10369  28612  17893   3305   4601
5989       C
ATOM   1323  CG  GLU A 184     -35.951  -6.903  42.084  1.00155.08
C
ANISOU 1323  CG  GLU A 184    10582  29661  18679   3443   4532
6591       C
ATOM   1324  CD  GLU A 184     -37.108  -7.526  42.841  1.00190.84
C
ANISOU 1324  CD  GLU A 184    14797  34547  23165   3293   4482
6880       C
ATOM   1325  OE1 GLU A 184     -37.870  -6.777  43.488  1.00194.52
O
ANISOU 1325  OE1 GLU A 184    15178  34935  23798   3641   4785
7038       O
ATOM   1326  OE2 GLU A 184     -37.258  -8.765  42.786  1.00193.23
O
ANISOU 1326  OE2 GLU A 184    14949  35204  23265   2815   4144
```

```
6947       O
ATOM   1327  N   SER A 185     -31.686  -5.632  43.561  1.00173.25
N
ANISOU 1327  N   SER A 185    14584  30245  20998   3821   5255
4761       N
ATOM   1328  CA  SER A 185     -30.373  -5.650  44.203  1.00178.77
C
ANISOU 1328  CA  SER A 185    15791  30512  21620   3773   5387
4198       C
ATOM   1329  C   SER A 185     -30.248  -6.720  45.284  1.00160.08
C
ANISOU 1329  C   SER A 185    13481  28247  19093   3455   5335
4013       C
ATOM   1330  O   SER A 185     -29.217  -7.399  45.373  1.00141.27
O
ANISOU 1330  O   SER A 185    11351  25769  16555   3211   5215
3690       O
ATOM   1331  CB  SER A 185     -30.064  -4.270  44.786  1.00183.80
C
ANISOU 1331  CB  SER A 185    16791  30620  22424   4158   5792
3944       C
ATOM   1332  OG  SER A 185     -30.034  -3.282  43.770  1.00159.40
O
ANISOU 1332  OG  SER A 185    13699  27394  19470   4436   5835
4081       O
ATOM   1333  N   LEU A 186     -31.274  -6.892  46.112  1.00139.02
N
ANISOU 1333  N   LEU A 186    10586  25779  16458   3452   5424
4222       N
ATOM   1334  CA  LEU A 186     -31.137  -7.804  47.248  1.00137.81
C
ANISOU 1334  CA  LEU A 186    10521  25681  16158   3171   5413
4028       C
ATOM   1335  C   LEU A 186     -31.212  -9.265  46.816  1.00136.51
C
ANISOU 1335  C   LEU A 186    10112  25960  15796   2704   5000
4182       C
ATOM   1336  O   LEU A 186     -32.181  -9.667  46.163  1.00139.15
O
ANISOU 1336  O   LEU A 186    10025  26722  16124   2577   4768
4612       O
ATOM   1337  CB  LEU A 186     -32.212  -7.520  48.293  1.00141.79
C
ANISOU 1337  CB  LEU A 186    10868  26254  16752   3306   5653
4191       C
ATOM   1338  CG  LEU A 186     -31.982  -8.171  49.660  1.00149.08
C
ANISOU 1338  CG  LEU A 186    11969  27128  17548   3086   5741
3926       C
ATOM   1339  CD1 LEU A 186     -30.883  -7.445  50.427  1.00138.34
C
ANISOU 1339  CD1 LEU A 186    11139  25230  16195   3217   6042
3423       C
ATOM   1340  CD2 LEU A 186     -33.270  -8.213  50.472  1.00154.28
```

```
C
ANISOU 1340  CD2 LEU A 186    12336  28022  18261   3130   5874
4202       C
ATOM   1341  N   PRO A 187     -30.208 -10.075  47.158  1.00132.72
N
ANISOU 1341  N   PRO A 187     9897  25387  15144   2417   4893
3847       N
ATOM   1342  CA  PRO A 187     -30.311 -11.525  46.969  1.00131.83
C
ANISOU 1342  CA  PRO A 187     9739  25492  14860   1889   4425
3896       C
ATOM   1343  C   PRO A 187     -31.483 -12.116  47.739  1.00135.04
C
ANISOU 1343  C   PRO A 187     9750  26341  15220   1745   4444
4215       C
ATOM   1344  O   PRO A 187     -31.915 -11.582  48.763  1.00136.95
O
ANISOU 1344  O   PRO A 187     9976  26515  15543   1980   4771
4207       O
ATOM   1345  CB  PRO A 187     -28.968 -12.041  47.495  1.00127.55
C
ANISOU 1345  CB  PRO A 187     9738  24516  14210   1678   4332
3381       C
ATOM   1346  CG  PRO A 187     -28.627 -11.078  48.576  1.00127.49
C
ANISOU 1346  CG  PRO A 187     9900  24273  14269   2030   4823
3162       C
ATOM   1347  CD  PRO A 187     -29.082  -9.728  48.042  1.00129.82
C
ANISOU 1347  CD  PRO A 187    10019  24558  14750   2504   5133
3358       C
ATOM   1348  N   VAL A 188     -31.999 -13.236  47.237  1.00135.79
N
ANISOU 1348  N   VAL A 188     9657  26746  15191   1311   4013
4435       N
ATOM   1349  CA  VAL A 188     -33.215 -13.848  47.759  1.00139.21
C
ANISOU 1349  CA  VAL A 188     9660  27664  15569   1130   3969
4806       C
ATOM   1350  C   VAL A 188     -32.876 -15.215  48.343  1.00137.31
C
ANISOU 1350  C   VAL A 188     9651  27381  15139    629   3671
4598       C
ATOM   1351  O   VAL A 188     -32.164 -16.010  47.717  1.00134.69
O
ANISOU 1351  O   VAL A 188     9621  26856  14699    285   3302
4398       O
ATOM   1352  CB  VAL A 188     -34.305 -13.961  46.677  1.00184.21
C
ANISOU 1352  CB  VAL A 188    14871  33845  21275   1029   3728
5330       C
ATOM   1353  CG1 VAL A 188     -34.857 -12.585  46.341  1.00178.90
C
ANISOU 1353  CG1 VAL A 188    14051  33094  20830   1555   3990
```

```
5541       C
ATOM   1354  CG2 VAL A 188     -33.751 -14.620  45.422  1.00161.98
C
ANISOU 1354  CG2 VAL A 188    12277  30927  18342    658   3255
5239       C
ATOM   1355  N   THR A 189     -33.371 -15.470  49.553  1.00138.77
N
ANISOU 1355  N   THR A 189     9708  27730  15289    600   3855
4646       N
ATOM   1356  CA  THR A 189     -33.247 -16.778  50.188  1.00153.73
C
ANISOU 1356  CA  THR A 189    11749  29652  17009    129   3588
4528       C
ATOM   1357  C   THR A 189     -33.988 -17.849  49.393  1.00157.48
C
ANISOU 1357  C   THR A 189    11979  30498  17357   -331   3134
4848       C
ATOM   1358  O   THR A 189     -35.124 -17.645  48.951  1.00143.10
O
ANISOU 1358  O   THR A 189     9676  29129  15567   -290   3121
5299       O
ATOM   1359  CB  THR A 189     -33.753 -16.721  51.633  1.00206.31
C
ANISOU 1359  CB  THR A 189    18270  36460  23658    214   3909
4562       C
ATOM   1360  OG1 THR A 189     -33.566 -17.996  52.261  1.00229.39
O
ANISOU 1360  OG1 THR A 189    21360  39386  26411   -249   3642
4440       O
ATOM   1361  CG2 THR A 189     -35.225 -16.326  51.698  1.00217.11
C
ANISOU 1361  CG2 THR A 189    19195  38172  25126    391   4002
4994       C
ATOM   1362  N   LYS A 190     -33.326 -18.986  49.185  1.00136.96
N
ANISOU 1362  N   LYS A 190     9723  27704  14614   -776   2765
4623       N
ATOM   1363  CA  LYS A 190     -33.887 -20.101  48.432  1.00144.89
C
ANISOU 1363  CA  LYS A 190    10601  28989  15462  -1279   2324
4861       C
ATOM   1364  C   LYS A 190     -33.806 -21.383  49.250  1.00137.80
C
ANISOU 1364  C   LYS A 190     9874  28074  14408  -1716   2135
4748       C
ATOM   1365  O   LYS A 190     -32.732 -21.748  49.737  1.00134.54
O
ANISOU 1365  O   LYS A 190     9903  27238  13978  -1776   2128
4356       O
ATOM   1366  CB  LYS A 190     -33.157 -20.282  47.098  1.00136.31
C
ANISOU 1366  CB  LYS A 190     9803  27647  14341  -1433   2035
4712       C
ATOM   1367  CG  LYS A 190     -33.325 -19.120  46.139  1.00140.08
```

```
C
ANISOU 1367  CG  LYS A 190    10087  28188  14951  -1068   2156
4877       C
ATOM   1368  CD  LYS A 190     -34.696 -19.139  45.487  1.00172.57
C
ANISOU 1368  CD  LYS A 190    13653  32890  19025  -1194   2013
5429       C
ATOM   1369  CE  LYS A 190     -34.834 -18.025  44.464  1.00178.36
C
ANISOU 1369  CE  LYS A 190    14194  33685  19888   -850   2105
5620       C
ATOM   1370  NZ  LYS A 190     -36.084 -18.163  43.668  1.00167.97
N
ANISOU 1370  NZ  LYS A 190    12363  32952  18507  -1050   1888
6177       N
ATOM   1371  N   HIS A 191     -34.942 -22.059  49.392  1.00141.12
N
ANISOU 1371  N   HIS A 191     9933  28967  14718  -2022   1980
5113       N
ATOM   1372  CA  HIS A 191     -35.137 -23.198  50.276  1.00141.47
C
ANISOU 1372  CA  HIS A 191    10039  29095  14619  -2416   1842
5096       C
ATOM   1373  C   HIS A 191     -35.322 -24.473  49.459  1.00142.09
C
ANISOU 1373  C   HIS A 191    10212  29269  14508  -3007   1373
5194       C
ATOM   1374  O   HIS A 191     -35.671 -24.412  48.276  1.00143.41
O
ANISOU 1374  O   HIS A 191    10247  29606  14638  -3123   1175
5395       O
ATOM   1375  CB  HIS A 191     -36.369 -22.991  51.172  1.00145.22
C
ANISOU 1375  CB  HIS A 191    10015  30062  15099  -2326   2058
5452       C
ATOM   1376  CG  HIS A 191     -36.292 -21.783  52.054  1.00145.22
C
ANISOU 1376  CG  HIS A 191     9930  29980  15267  -1775   2559
5367       C
ATOM   1377  ND1 HIS A 191     -36.424 -21.856  53.424  1.00145.67
N
ANISOU 1377  ND1 HIS A 191     9975  30069  15305  -1731   2802
5296       N
ATOM   1378  CD2 HIS A 191     -36.116 -20.473  51.764  1.00145.08
C
ANISOU 1378  CD2 HIS A 191     9851  29843  15428  -1261   2876
5344       C
ATOM   1379  CE1 HIS A 191     -36.322 -20.644  53.940  1.00145.85
C
ANISOU 1379  CE1 HIS A 191    10007  29922  15488  -1213   3227
5190       C
ATOM   1380  NE2 HIS A 191     -36.135 -19.786  52.954  1.00145.50
N
ANISOU 1380  NE2 HIS A 191     9925  29789  15569   -917   3290
```

```
5223       N
ATOM   1381  N   PRO A 192      -35.035 -25.655  50.046  1.00141.19
N
ANISOU 1381  N   PRO A 192    10363  29020  14262  -3407   1189
5042       N
ATOM   1382  CA  PRO A 192      -35.386 -26.928  49.392  1.00159.44
C
ANISOU 1382  CA  PRO A 192    12745  31462  16372  -4008    774
5174       C
ATOM   1383  C   PRO A 192      -36.716 -26.928  48.652  1.00155.84
C
ANISOU 1383  C   PRO A 192    11795  31596  15820  -4211    612
5667       C
ATOM   1384  O   PRO A 192      -37.732 -26.470  49.184  1.00160.96
O
ANISOU 1384  O   PRO A 192    11954  32697  16505  -4050    784
6003       O
ATOM   1385  CB  PRO A 192      -35.407 -27.914  50.566  1.00142.56
C
ANISOU 1385  CB  PRO A 192    10726  29302  14137  -4292    736
5104       C
ATOM   1386  CG  PRO A 192      -34.361 -27.387  51.467  1.00139.27
C
ANISOU 1386  CG  PRO A 192    10591  28463  13861  -3914   1022
4735       C
ATOM   1387  CD  PRO A 192      -34.506 -25.882  51.403  1.00139.48
C
ANISOU 1387  CD  PRO A 192    10356  28577  14062  -3346   1366
4792       C
ATOM   1388  N   GLY A 193      -36.719 -27.446  47.423  1.00147.35
N
ANISOU 1388  N   GLY A 193    10843  30529  14614  -4578    286
5725       N
ATOM   1389  CA  GLY A 193      -37.892 -27.456  46.581  1.00151.42
C
ANISOU 1389  CA  GLY A 193    10918  31600  15013  -4821     90
6196       C
ATOM   1390  C   GLY A 193      -38.103 -26.199  45.761  1.00152.23
C
ANISOU 1390  C   GLY A 193    10726  31858  15257  -4409    222
6386       C
ATOM   1391  O   GLY A 193      -38.748 -26.257  44.709  1.00207.31
O
ANISOU 1391  O   GLY A 193    17473  39175  22119  -4662    -14
6705       O
ATOM   1392  N   GLN A 194      -37.580 -25.066  46.216  1.00150.18
N
ANISOU 1392  N   GLN A 194    10473  31359  15230  -3799    592
6207       N
ATOM   1393  CA  GLN A 194      -37.746 -23.814  45.494  1.00169.03
C
ANISOU 1393  CA  GLN A 194    12596  33856  17773  -3365    755
6382       C
ATOM   1394  C   GLN A 194      -36.905 -23.803  44.218  1.00169.56
```

```
C
ANISOU 1394  C   GLN A 194    13033  33589  17803  -3470    550
6163       C
ATOM   1395  O   GLN A 194     -35.850 -24.437  44.133  1.00207.17
O
ANISOU 1395  O   GLN A 194    18337  37869  22509  -3659    433
5743       O
ATOM   1396  CB  GLN A 194     -37.374 -22.640  46.398  1.00171.56
C
ANISOU 1396  CB  GLN A 194    12882  33965  18338  -2709   1233
6217       C
ATOM   1397  CG  GLN A 194     -37.975 -21.306  46.004  1.00183.14
C
ANISOU 1397  CG  GLN A 194    13905  35694  19987  -2214   1491
6542       C
ATOM   1398  CD  GLN A 194     -37.794 -20.261  47.088  1.00187.31
C
ANISOU 1398  CD  GLN A 194    14385  36057  20728  -1622   2000
6410       C
ATOM   1399  OE1 GLN A 194     -37.105 -20.495  48.081  1.00148.54
O
ANISOU 1399  OE1 GLN A 194     9808  30812  15817  -1590   2142
6042       O
ATOM   1400  NE2 GLN A 194     -38.416 -19.102  46.906  1.00192.53
N
ANISOU 1400  NE2 GLN A 194    14747  36836  21570  -1139   2240
6670       N
ATOM   1401  N   GLU A 195     -37.390 -23.071  43.218  1.00184.63
N
ANISOU 1401  N   GLU A 195    14635  35768  19748  -3340    514
6470       N
ATOM   1402  CA  GLU A 195     -36.739 -22.998  41.915  1.00193.70
C
ANISOU 1402  CA  GLU A 195    16074  36678  20844  -3457    314
6325       C
ATOM   1403  C   GLU A 195     -35.705 -21.878  41.874  1.00204.58
C
ANISOU 1403  C   GLU A 195    17695  37589  22448  -2883    614
5992       C
ATOM   1404  O   GLU A 195     -35.911 -20.800  42.438  1.00211.65
O
ANISOU 1404  O   GLU A 195    18327  38543  23549  -2347    970
6086       O
ATOM   1405  CB  GLU A 195     -37.780 -22.798  40.811  1.00212.48
C
ANISOU 1405  CB  GLU A 195    18000  39608  23125  -3652     94
6847       C
ATOM   1406  CG  GLU A 195     -37.196 -22.522  39.433  1.00205.74
C
ANISOU 1406  CG  GLU A 195    17387  38559  22228  -3721    -76
6746       C
ATOM   1407  CD  GLU A 195     -38.261 -22.349  38.368  1.00212.70
C
ANISOU 1407  CD  GLU A 195    17800  40027  22989  -3956   -314
```

```
7299       C
ATOM   1408  OE1 GLU A 195      -38.249 -23.121  37.386  1.00218.51
O
ANISOU 1408  OE1 GLU A 195    18734  40816  23476  -4521   -677
7314       O
ATOM   1409  OE2 GLU A 195      -39.110 -21.445  38.514  1.00216.95
O
ANISOU 1409  OE2 GLU A 195    17776  40976  23680  -3583   -129
7729       O
ATOM   1410  N   VAL A 196      -34.585 -22.152  41.200  1.00192.47
N
ANISOU 1410  N   VAL A 196    16680  35580  20869  -3010    480
5600       N
ATOM   1411  CA  VAL A 196      -33.566 -21.163  40.872  1.00156.14
C
ANISOU 1411  CA  VAL A 196    12336  30549  16440  -2559    689
5297       C
ATOM   1412  C   VAL A 196      -33.309 -21.218  39.370  1.00162.42
C
ANISOU 1412  C   VAL A 196    13285  31298  17128  -2797    422
5309       C
ATOM   1413  O   VAL A 196      -33.677 -22.177  38.688  1.00184.62
O
ANISOU 1413  O   VAL A 196    16140  34292  19715  -3353     78
5436       O
ATOM   1414  CB  VAL A 196      -32.257 -21.384  41.658  1.00138.08
C
ANISOU 1414  CB  VAL A 196    10574  27668  14223  -2431    837
4752       C
ATOM   1415  CG1 VAL A 196      -32.495 -21.178  43.146  1.00135.32
C
ANISOU 1415  CG1 VAL A 196    10070  27370  13974  -2171   1130
4743       C
ATOM   1416  CG2 VAL A 196      -31.705 -22.774  41.388  1.00164.16
C
ANISOU 1416  CG2 VAL A 196    14311  30724  17338  -2980    524
4510       C
ATOM   1417  N   PHE A 197      -32.671 -20.168  38.854  1.00147.37
N
ANISOU 1417  N   PHE A 197    11478  29143  15372  -2387    589
5171       N
ATOM   1418  CA  PHE A 197      -32.614 -19.913  37.421  1.00144.41
C
ANISOU 1418  CA  PHE A 197    11129  28817  14924  -2516    389
5275       C
ATOM   1419  C   PHE A 197      -31.165 -19.738  36.984  1.00163.67
C
ANISOU 1419  C   PHE A 197    14126  30647  17412  -2395    435
4770       C
ATOM   1420  O   PHE A 197      -30.291 -19.393  37.783  1.00194.79
O
ANISOU 1420  O   PHE A 197    18322  34185  21505  -2051    693
4414       O
ATOM   1421  CB  PHE A 197      -33.413 -18.658  37.040  1.00164.20
```

```
C
ANISOU 1421  CB  PHE A 197    13117  31700  17573  -2114    544
5710       C
ATOM   1422  CG  PHE A 197     -34.818 -18.643  37.573  1.00185.87
C
ANISOU 1422  CG  PHE A 197    15258  35044  20322  -2118    576
6231       C
ATOM   1423  CD1 PHE A 197     -35.837 -19.295  36.901  1.00163.44
C
ANISOU 1423  CD1 PHE A 197    12101  32727  17272  -2613    236
6666       C
ATOM   1424  CD2 PHE A 197     -35.119 -17.965  38.744  1.00188.65
C
ANISOU 1424  CD2 PHE A 197    15361  35440  20877  -1638    957
6293       C
ATOM   1425  CE1 PHE A 197     -37.129 -19.276  37.391  1.00155.82
C
ANISOU 1425  CE1 PHE A 197    10554  32337  16315  -2615    266
7169       C
ATOM   1426  CE2 PHE A 197     -36.410 -17.943  39.238  1.00150.33
C
ANISOU 1426  CE2 PHE A 197     9942  31139  16039  -1623   1011
6780       C
ATOM   1427  CZ  PHE A 197     -37.415 -18.600  38.561  1.00154.14
C
ANISOU 1427  CZ  PHE A 197    10084  32158  16326  -2105    661
7228       C
ATOM   1428  N   SER A 198     -30.922 -19.982  35.695  1.00144.11
N
ANISOU 1428  N   SER A 198    11835  28125  14794  -2701    180
4752       N
ATOM   1429  CA  SER A 198     -29.586 -19.829  35.132  1.00149.05
C
ANISOU 1429  CA  SER A 198    12972  28204  15454  -2614    209
4303       C
ATOM   1430  C   SER A 198     -29.085 -18.396  35.281  1.00155.79
C
ANISOU 1430  C   SER A 198    13776  28862  16554  -1970    540
4208       C
ATOM   1431  O   SER A 198     -29.853 -17.432  35.234  1.00145.76
O
ANISOU 1431  O   SER A 198    12067  27920  15394  -1651    677
4564       O
ATOM   1432  CB  SER A 198     -29.572 -20.231  33.656  1.00176.51
C
ANISOU 1432  CB  SER A 198    16604  31736  18725  -3053   -104
4359       C
ATOM   1433  OG  SER A 198     -30.189 -19.247  32.848  1.00228.79
O
ANISOU 1433  OG  SER A 198    22852  38697  25380  -2873   -111
4725       O
ATOM   1434  N   GLY A 199     -27.769 -18.272  35.461  1.00169.70
N
ANISOU 1434  N   GLY A 199    16000  30076  18403  -1784    676
```

```
3731       N
ATOM   1435  CA  GLY A 199     -27.092 -16.996  35.530  1.00184.16
C
ANISOU 1435  CA  GLY A 199    17893  31642  20437  -1235    973
3566       C
ATOM   1436  C   GLY A 199     -27.035 -16.406  36.920  1.00178.64
C
ANISOU 1436  C   GLY A 199    17099  30863  19915   -805   1324
3485       C
ATOM   1437  O   GLY A 199     -26.272 -15.457  37.148  1.00175.22
O
ANISOU 1437  O   GLY A 199    16823  30119  19634   -385   1592
3255       O
ATOM   1438  N   SER A 200     -27.822 -16.940  37.847  1.00162.32
N
ANISOU 1438  N   SER A 200    14789  29069  17816   -922   1333
3668       N
ATOM   1439  CA  SER A 200     -27.780 -16.549  39.247  1.00159.10
C
ANISOU 1439  CA  SER A 200    14325  28589  17539   -593   1655
3573       C
ATOM   1440  C   SER A 200     -26.445 -16.925  39.882  1.00185.53
C
ANISOU 1440  C   SER A 200    18173  31420  20901   -593   1721
3071       C
ATOM   1441  O   SER A 200     -25.776 -17.875  39.464  1.00148.60
O
ANISOU 1441  O   SER A 200    13837  26510  16115   -942   1482
2848       O
ATOM   1442  CB  SER A 200     -28.926 -17.200  40.016  1.00143.17
C
ANISOU 1442  CB  SER A 200    11950  26996  15450   -798   1605
3884       C
ATOM   1443  OG  SER A 200     -29.052 -16.621  41.300  1.00136.00
O
ANISOU 1443  OG  SER A 200    10922  26080  14673   -436   1959
3855       O
ATOM   1444  N   THR A 201     -26.057 -16.165  40.903  1.00191.82
N
ANISOU 1444  N   THR A 201    19014  32039  21831   -202   2062
2905       N
ATOM   1445  CA  THR A 201     -24.825 -16.404  41.642  1.00120.27
C
ANISOU 1445  CA  THR A 201    10376  22533  12788   -174   2152
2471       C
ATOM   1446  C   THR A 201     -25.174 -16.545  43.117  1.00130.21
C
ANISOU 1446  C   THR A 201    11518  23895  14059   -116   2344
2487       C
ATOM   1447  O   THR A 201     -25.845 -15.679  43.686  1.00154.62
O
ANISOU 1447  O   THR A 201    14325  27186  17237    201   2629
2666       O
ATOM   1448  CB  THR A 201     -23.823 -15.263  41.436  1.00118.24
```

```
C
ANISOU 1448  CB  THR A 201    10358  21916  12654    217   2387
2204       C
ATOM   1449  OG1 THR A 201     -24.449 -14.011  41.747  1.00114.10
O
ANISOU 1449  OG1 THR A 201     9544  21558  12251    643   2714
2396       O
ATOM   1450  CG2 THR A 201     -23.339 -15.237  39.994  1.00148.65
C
ANISOU 1450  CG2 THR A 201    14378  25625  16476    119   2181
2143       C
ATOM   1451  N   CYS A 202     -24.720 -17.640  43.727  1.00125.85
N
ANISOU 1451  N   CYS A 202    11193  23202  13423   -421   2199
2307       N
ATOM   1452  CA  CYS A 202     -24.913 -17.869  45.158  1.00114.18
C
ANISOU 1452  CA  CYS A 202     9658  21789  11936   -411   2358
2288       C
ATOM   1453  C   CYS A 202     -24.109 -16.874  45.983  1.00112.34
C
ANISOU 1453  C   CYS A 202     9606  21276  11800    -32   2709
2021       C
ATOM   1454  O   CYS A 202     -22.874 -16.895  45.963  1.00198.82
O
ANISOU 1454  O   CYS A 202    20935  31840  22768    -18   2699
1691       O
ATOM   1455  CB  CYS A 202     -24.511 -19.297  45.516  1.00113.09
C
ANISOU 1455  CB  CYS A 202     9752  21529  11688   -836   2101
2158       C
ATOM   1456  SG  CYS A 202     -25.397 -20.572  44.609  1.00151.24
S
ANISOU 1456  SG  CYS A 202    14448  26651  16367  -1355   1692
2434       S
ATOM   1457  N   LYS A 203     -24.803 -15.997  46.711  1.00114.29
N
ANISOU 1457  N   LYS A 203     9595  21722  12110    266   3033
2169       N
ATOM   1458  CA  LYS A 203     -24.114 -15.165  47.690  1.00112.98
C
ANISOU 1458  CA  LYS A 203     9624  21308  11995    554   3381
1912       C
ATOM   1459  C   LYS A 203     -23.698 -15.976  48.910  1.00111.94
C
ANISOU 1459  C   LYS A 203     9666  21094  11774    323   3357
1744       C
ATOM   1460  O   LYS A 203     -22.664 -15.687  49.524  1.00145.14
O
ANISOU 1460  O   LYS A 203    14180  24991  15977    403   3494
1442       O
ATOM   1461  CB  LYS A 203     -25.006 -13.997  48.113  1.00115.76
C
ANISOU 1461  CB  LYS A 203     9674  21874  12437    937   3773
```

```
2118       C
ATOM   1462  CG  LYS A 203     -25.128 -12.882  47.090  1.00116.44
C
ANISOU 1462  CG  LYS A 203     9665  21933  12643   1273   3902
2219       C
ATOM   1463  CD  LYS A 203     -23.811 -12.146  46.934  1.00113.45
C
ANISOU 1463  CD  LYS A 203     9696  21103  12305   1466   4035
1845       C
ATOM   1464  CE  LYS A 203     -23.988 -10.856  46.152  1.00129.65
C
ANISOU 1464  CE  LYS A 203    11654  23121  14488   1860   4256
1948       C
ATOM   1465  NZ  LYS A 203     -22.683 -10.192  45.876  1.00164.13
N
ANISOU 1465  NZ  LYS A 203    16428  27054  18881   2007   4347
1588       N
ATOM   1466  N   GLN A 204     -24.484 -16.990  49.274  1.00113.60
N
ANISOU 1466  N   GLN A 204     9679  21585  11900     21   3176
1949       N
ATOM   1467  CA  GLN A 204     -24.307 -17.703  50.533  1.00113.29
C
ANISOU 1467  CA  GLN A 204     9736  21534  11774   -183   3181
1855       C
ATOM   1468  C   GLN A 204     -24.665 -19.174  50.353  1.00113.81
C
ANISOU 1468  C   GLN A 204     9758  21742  11741   -632   2805
1991       C
ATOM   1469  O   GLN A 204     -25.178 -19.593  49.313  1.00114.80
O
ANISOU 1469  O   GLN A 204     9753  22016  11849   -793   2565
2175       O
ATOM   1470  CB  GLN A 204     -25.161 -17.088  51.652  1.00124.18
C
ANISOU 1470  CB  GLN A 204    10859  23171  13153      5   3528
1997       C
ATOM   1471  CG  GLN A 204     -24.978 -15.588  51.857  1.00129.83
C
ANISOU 1471  CG  GLN A 204    11607  23760  13961    454   3953
1893       C
ATOM   1472  CD  GLN A 204     -25.596 -15.088  53.147  1.00179.65
C
ANISOU 1472  CD  GLN A 204    17773  30238  20248    599   4329
1953       C
ATOM   1473  OE1 GLN A 204     -26.050 -15.875  53.978  1.00193.62
O
ANISOU 1473  OE1 GLN A 204    19438  32205  21923    354   4264
2044       O
ATOM   1474  NE2 GLN A 204     -25.621 -13.771  53.319  1.00191.70
N
ANISOU 1474  NE2 GLN A 204    19306  31676  21856    993   4741
1901       N
ATOM   1475  N   GLY A 205     -24.380 -19.952  51.397  1.00113.29
```

N
ANISOU 1475 N   GLY A 205   9818 21627 11599   -853   2760
1899   N
ATOM   1476 CA GLY A 205   -24.758 -21.350 51.528 1.00114.10
C
ANISOU 1476 CA GLY A 205   9891 21861 11601 -1279   2458
2027   C
ATOM   1477 C   GLY A 205   -24.355 -22.344 50.456 1.00113.05
C
ANISOU 1477 C   GLY A 205   9955 21559 11440 -1580   2099
1990   C
ATOM   1478 O   GLY A 205   -23.735 -21.990 49.448 1.00136.59
O
ANISOU 1478 O   GLY A 205   13102 24315 14481 -1470   2051
1861   O
ATOM   1479 N   GLU A 206   -24.707 -23.611 50.686 1.00114.04
N
ANISOU 1479 N   GLU A 206   10080 21784 11467 -1975   1858
2100   N
ATOM   1480 CA GLU A 206   -24.353 -24.720 49.807 1.00113.45
C
ANISOU 1480 CA GLU A 206   10235 21527 11345 -2316   1536
2059   C
ATOM   1481 C   GLU A 206   -25.428 -25.795 49.879 1.00116.18
C
ANISOU 1481 C   GLU A 206   10391 22192 11560 -2724   1317
2332   C
ATOM   1482 O   GLU A 206   -25.853 -26.163 50.978 1.00152.80
O
ANISOU 1482 O   GLU A 206   14908 27003 16145 -2827   1364
2425   O
ATOM   1483 CB GLU A 206   -22.997 -25.309 50.207 1.00110.78
C
ANISOU 1483 CB GLU A 206   10306 20744 11042 -2382   1488
1770   C
ATOM   1484 CG GLU A 206   -22.588 -26.528 49.411 1.00110.43
C
ANISOU 1484 CG GLU A 206   10534 20463 10960 -2728   1200
1718   C
ATOM   1485 CD GLU A 206   -21.286 -27.120 49.904 1.00131.33
C
ANISOU 1485 CD GLU A 206   13544 22689 13665 -2763   1179
1479   C
ATOM   1486 OE1 GLU A 206   -21.286 -28.306 50.296 1.00145.77
O
ANISOU 1486 OE1 GLU A 206   15487 24456 15442 -3077   1022
1526   O
ATOM   1487 OE2 GLU A 206   -20.265 -26.401 49.903 1.00162.74
O
ANISOU 1487 OE2 GLU A 206   17687 26404 17743 -2479   1321
1261   O
ATOM   1488 N   ILE A 207   -25.863 -26.299 48.719 1.00117.40
N
ANISOU 1488 N   ILE A 207   10531 22432 11645 -2979   1080

```
2459       N
ATOM   1489  CA  ILE A 207     -26.961 -27.260  48.644  1.00132.19
C
ANISOU 1489  CA  ILE A 207    12213  24645  13370  -3399    860
2742       C
ATOM   1490  C   ILE A 207     -26.799 -28.123  47.400  1.00120.63
C
ANISOU 1490  C   ILE A 207    10992  23027  11816  -3763    573
2718       C
ATOM   1491  O   ILE A 207     -26.099 -27.763  46.452  1.00134.69
O
ANISOU 1491  O   ILE A 207    12980  24543  13650  -3645    562
2548       O
ATOM   1492  CB  ILE A 207     -28.366 -26.600  48.653  1.00123.48
C
ANISOU 1492  CB  ILE A 207    10589  24093  12234  -3309    941
3104       C
ATOM   1493  CG1 ILE A 207     -28.367 -25.210  48.003  1.00123.14
C
ANISOU 1493  CG1 ILE A 207    10385  24093  12308  -2878   1133
3121       C
ATOM   1494  CG2 ILE A 207     -28.929 -26.549  50.069  1.00124.70
C
ANISOU 1494  CG2 ILE A 207    10506  24493  12380  -3246   1117
3219       C
ATOM   1495  CD1 ILE A 207     -27.890 -25.143  46.569  1.00122.18
C
ANISOU 1495  CD1 ILE A 207    10462  23774  12187  -2928    973
3031       C
ATOM   1496  N   GLU A 208     -27.447 -29.284  47.416  1.00122.82
N
ANISOU 1496  N   GLU A 208    11261  23464  11942  -4228    349
2883       N
ATOM   1497  CA  GLU A 208     -27.672 -30.051  46.201  1.00124.23
C
ANISOU 1497  CA  GLU A 208    11591  23628  11984  -4637     83
2943       C
ATOM   1498  C   GLU A 208     -28.987 -29.616  45.558  1.00127.40
C
ANISOU 1498  C   GLU A 208    11552  24571  12281  -4731      1
3306       C
ATOM   1499  O   GLU A 208     -29.882 -29.083  46.219  1.00129.08
O
ANISOU 1499  O   GLU A 208    11337  25200  12509  -4578    116
3556       O
ATOM   1500  CB  GLU A 208     -27.694 -31.550  46.503  1.00125.29
C
ANISOU 1500  CB  GLU A 208    11973  23639  11992  -5126   -114
2939       C
ATOM   1501  CG  GLU A 208     -26.386 -32.088  47.062  1.00122.56
C
ANISOU 1501  CG  GLU A 208    12059  22752  11756  -5058    -53
2622       C
ATOM   1502  CD  GLU A 208     -26.451 -33.571  47.376  1.00142.05
```

```
C
ANISOU 1502  CD  GLU A 208    14769  25091  14113  -5527   -230
2645       C
ATOM   1503  OE1 GLU A 208     -26.528 -34.380  46.428  1.00133.26
O
ANISOU 1503  OE1 GLU A 208    13897  23859  12879  -5910   -414
2641       O
ATOM   1504  OE2 GLU A 208     -26.426 -33.929  48.572  1.00193.04
O
ANISOU 1504  OE2 GLU A 208    21191  31560  20596  -5527   -177
2668       O
ATOM   1505  N   ALA A 209     -29.097 -29.849  44.250  1.00157.40
N
ANISOU 1505  N   ALA A 209    15460  28372  15973  -4990   -194
3346       N
ATOM   1506  CA  ALA A 209     -30.270 -29.407  43.507  1.00153.39
C
ANISOU 1506  CA  ALA A 209    14538  28382  15362  -5092   -295
3711       C
ATOM   1507  C   ALA A 209     -30.479 -30.276  42.275  1.00133.40
C
ANISOU 1507  C   ALA A 209    12216  25856  12615  -5637   -596
3765       C
ATOM   1508  O   ALA A 209     -29.578 -30.993  41.831  1.00139.55
O
ANISOU 1508  O   ALA A 209    13491  26171  13361  -5836   -673
3472       O
ATOM   1509  CB  ALA A 209     -30.152 -27.934  43.099  1.00138.71
C
ANISOU 1509  CB  ALA A 209    12459  26584  13661  -4574    -97
3730       C
ATOM   1510  N   VAL A 210     -31.693 -30.204  41.734  1.00137.00
N
ANISOU 1510  N   VAL A 210    12284  26850  12918  -5885   -754
4156       N
ATOM   1511  CA  VAL A 210     -32.087 -30.910  40.519  1.00149.96
C
ANISOU 1511  CA  VAL A 210    14058  28607  14314  -6445  -1048
4273       C
ATOM   1512  C   VAL A 210     -32.278 -29.858  39.433  1.00140.11
C
ANISOU 1512  C   VAL A 210    12589  27560  13086  -6248  -1053
4418       C
ATOM   1513  O   VAL A 210     -33.213 -29.050  39.502  1.00154.58
O
ANISOU 1513  O   VAL A 210    13883  29894  14955  -6051  -1007
4785       O
ATOM   1514  CB  VAL A 210     -33.364 -31.737  40.725  1.00172.81
C
ANISOU 1514  CB  VAL A 210    16675  32007  16979  -6966  -1265
4646       C
ATOM   1515  CG1 VAL A 210     -33.666 -32.568  39.486  1.00151.51
C
ANISOU 1515  CG1 VAL A 210    14202  29374  13991  -7615  -1571
```

```
4723       C
ATOM   1516  CG2 VAL A 210     -33.232 -32.626  41.958  1.00143.04
C
ANISOU 1516  CG2 VAL A 210    13055  28074  13218  -7083  -1225
4535       C
ATOM   1517  N   VAL A 211     -31.410 -29.878  38.418  1.00138.51
N
ANISOU 1517  N   VAL A 211    12797  26969  12860  -6306  -1101
4149       N
ATOM   1518  CA  VAL A 211     -31.559 -28.991  37.267  1.00149.09
C
ANISOU 1518  CA  VAL A 211    13979  28482  14186  -6196  -1142
4283       C
ATOM   1519  C   VAL A 211     -32.813 -29.340  36.477  1.00159.29
C
ANISOU 1519  C   VAL A 211    14969  30349  15206  -6724  -1431
4720       C
ATOM   1520  O   VAL A 211     -33.172 -30.515  36.320  1.00178.51
O
ANISOU 1520  O   VAL A 211    17588  32840  17397  -7327  -1653
4764       O
ATOM   1521  CB  VAL A 211     -30.308 -29.031  36.371  1.00151.50
C
ANISOU 1521  CB  VAL A 211    14829  28226  14508  -6183  -1130
3876       C
ATOM   1522  CG1 VAL A 211     -29.111 -28.455  37.110  1.00156.62
C
ANISOU 1522  CG1 VAL A 211    15684  28386  15440  -5606   -838
3505       C
ATOM   1523  CG2 VAL A 211     -30.027 -30.443  35.885  1.00148.33
C
ANISOU 1523  CG2 VAL A 211    14931  27548  13880  -6805  -1330
3696       C
ATOM   1524  N   ILE A 212     -33.497 -28.304  35.988  1.00180.24
N
ANISOU 1524  N   ILE A 212    17146  33440  17897  -6502  -1425
5066       N
ATOM   1525  CA  ILE A 212     -34.801 -28.431  35.343  1.00199.37
C
ANISOU 1525  CA  ILE A 212    19146  36514  20091  -6927  -1682
5574       C
ATOM   1526  C   ILE A 212     -34.671 -28.338  33.827  1.00196.62
C
ANISOU 1526  C   ILE A 212    18966  36177  19563  -7228  -1884
5599       C
ATOM   1527  O   ILE A 212     -34.994 -29.294  33.111  1.00219.19
O
ANISOU 1527  O   ILE A 212    22028  39142  22112  -7904  -2165
5663       O
ATOM   1528  CB  ILE A 212     -35.785 -27.369  35.867  1.00194.97
C
ANISOU 1528  CB  ILE A 212    17876  36506  19700  -6489  -1537
6027       C
ATOM   1529  CG1 ILE A 212     -36.018 -27.550  37.368  1.00195.98
```

```
C
ANISOU 1529  CG1 ILE A 212    17838  36666  19960  -6271  -1347
6022       C
ATOM   1530  CG2 ILE A 212     -37.103 -27.442  35.107  1.00190.32
C
ANISOU 1530  CG2 ILE A 212    16812  36619  18882  -6917  -1815
6596       C
ATOM   1531  CD1 ILE A 212     -36.602 -26.330  38.042  1.00186.11
C
ANISOU 1531  CD1 ILE A 212    16013  35744  18956  -5659  -1068
6312       C
ATOM   1532  N   ALA A 213     -34.201 -27.197  33.321  1.00186.26
N
ANISOU 1532  N   ALA A 213    17591  34753  18427  -6756  -1741
5546       N
ATOM   1533  CA  ALA A 213     -34.117 -27.000  31.880  1.00186.84
C
ANISOU 1533  CA  ALA A 213    17781  34876  18333  -7016  -1925
5601       C
ATOM   1534  C   ALA A 213     -32.970 -26.054  31.546  1.00189.77
C
ANISOU 1534  C   ALA A 213    18399  34774  18932  -6478  -1704
5258       C
ATOM   1535  O   ALA A 213     -32.669 -25.121  32.295  1.00191.97
O
ANISOU 1535  O   ALA A 213    18497  34937  19507  -5831  -1417
5194       O
ATOM   1536  CB  ALA A 213     -35.434 -26.455  31.315  1.00176.83
C
ANISOU 1536  CB  ALA A 213    15874  34356  16956  -7144  -2099
6227       C
ATOM   1537  N   THR A 214     -32.337 -26.319  30.401  1.00168.11
N
ANISOU 1537  N   THR A 214    16087  31757  16029  -6781  -1834
5034       N
ATOM   1538  CA  THR A 214     -31.247 -25.515  29.855  1.00143.64
C
ANISOU 1538  CA  THR A 214    13260  28225  13090  -6379  -1671
4715       C
ATOM   1539  C   THR A 214     -31.554 -24.029  29.697  1.00157.03
C
ANISOU 1539  C   THR A 214    14477  30197  14992  -5819  -1533
4996       C
ATOM   1540  O   THR A 214     -32.717 -23.615  29.662  1.00174.29
O
ANISOU 1540  O   THR A 214    16095  32980  17148  -5828  -1618
5511       O
ATOM   1541  CB  THR A 214     -30.823 -26.085  28.499  1.00144.45
C
ANISOU 1541  CB  THR A 214    13822  28140  12922  -6910  -1880
4544       C
ATOM   1542  OG1 THR A 214     -31.953 -26.689  27.857  1.00149.27
O
ANISOU 1542  OG1 THR A 214    14229  29285  13200  -7569  -2201
```

```
4946       O
ATOM   1543  CG2 THR A 214     -29.732 -27.128  28.679  1.00149.78
C
ANISOU 1543  CG2 THR A 214    15172  28169  13567  -7107  -1819
4014       C
ATOM   1544  N   GLY A 215     -30.482 -23.232  29.613  1.00154.20
N
ANISOU 1544  N   GLY A 215    14353  29389  14848  -5327  -1306
4662       N
ATOM   1545  CA  GLY A 215     -30.595 -21.782  29.528  1.00153.15
C
ANISOU 1545  CA  GLY A 215    13847  29403  14939  -4740  -1122
4858       C
ATOM   1546  C   GLY A 215     -31.473 -21.265  28.404  1.00146.90
C
ANISOU 1546  C   GLY A 215    12675  29130  14011  -4920  -1326
5336       C
ATOM   1547  O   GLY A 215     -32.102 -20.212  28.540  1.00170.72
O
ANISOU 1547  O   GLY A 215    15183  32486  17197  -4501  -1206
5706       O
ATOM   1548  N   VAL A 216     -31.524 -21.983  27.278  1.00145.63
N
ANISOU 1548  N   VAL A 216    12758  29035  13541  -5545  -1626
5346       N
ATOM   1549  CA  VAL A 216     -32.466 -21.635  26.214  1.00149.93
C
ANISOU 1549  CA  VAL A 216    12919  30147  13902  -5828  -1873
5854       C
ATOM   1550  C   VAL A 216     -33.905 -21.689  26.716  1.00168.70
C
ANISOU 1550  C   VAL A 216    14652  33203  16242  -5931  -1976
6439       C
ATOM   1551  O   VAL A 216     -34.737 -20.852  26.345  1.00157.20
O
ANISOU 1551  O   VAL A 216    12649  32247  14833  -5765  -2015
6949       O
ATOM   1552  CB  VAL A 216     -32.244 -22.541  24.984  1.00151.36
C
ANISOU 1552  CB  VAL A 216    13542  30257  13710  -6567  -2177
5722       C
ATOM   1553  CG1 VAL A 216     -32.557 -24.001  25.300  1.00152.83
C
ANISOU 1553  CG1 VAL A 216    13963  30464  13641  -7204  -2357
5647       C
ATOM   1554  CG2 VAL A 216     -33.078 -22.053  23.801  1.00155.61
C
ANISOU 1554  CG2 VAL A 216    13710  31358  14056  -6841  -2430
6230       C
ATOM   1555  N   HIS A 217     -34.223 -22.659  27.571  1.00180.81
N
ANISOU 1555  N   HIS A 217    16224  34773  17701  -6193  -2013
6394       N
ATOM   1556  CA  HIS A 217     -35.571 -22.738  28.119  1.00189.05
```

```
C
ANISOU 1556  CA  HIS A 217    16655  36460  18716  -6287  -2095
6939       C
ATOM   1557  C   HIS A 217     -35.752 -21.836  29.333  1.00178.08
C
ANISOU 1557  C   HIS A 217    14869  35100  17693  -5542  -1739
7036       C
ATOM   1558  O   HIS A 217     -36.881 -21.437  29.639  1.00203.64
O
ANISOU 1558  O   HIS A 217    17480  38906  20986  -5424  -1734
7569       O
ATOM   1559  CB  HIS A 217     -35.903 -24.188  28.475  1.00195.81
C
ANISOU 1559  CB  HIS A 217    17718  37375  19307  -6936  -2301
6878       C
ATOM   1560  CG  HIS A 217     -35.733 -25.140  27.332  1.00187.41
C
ANISOU 1560  CG  HIS A 217    17098  36247  17862  -7705  -2620
6757       C
ATOM   1561  ND1 HIS A 217     -34.707 -26.057  27.272  1.00170.05
N
ANISOU 1561  ND1 HIS A 217    15612  33434  15564  -7974  -2609
6194       N
ATOM   1562  CD2 HIS A 217     -36.455 -25.309  26.199  1.00168.85
C
ANISOU 1562  CD2 HIS A 217    14589  34368  15197  -8272  -2944
7134       C
ATOM   1563  CE1 HIS A 217     -34.808 -26.755  26.154  1.00175.26
C
ANISOU 1563  CE1 HIS A 217    16560  34166  15865  -8672  -2892
6206       C
ATOM   1564  NE2 HIS A 217     -35.860 -26.320  25.485  1.00165.35
N
ANISOU 1564  NE2 HIS A 217    14792  33576  14459  -8885  -3110
6768       N
ATOM   1565  N   THR A 218     -34.667 -21.524  30.040  1.00170.14
N
ANISOU 1565  N   THR A 218    14217  33499  16928  -5057  -1435
6539       N
ATOM   1566  CA  THR A 218     -34.713 -20.548  31.121  1.00174.92
C
ANISOU 1566  CA  THR A 218    14517  34072  17874  -4337  -1062
6581       C
ATOM   1567  C   THR A 218     -35.279 -19.211  30.640  1.00183.52
C
ANISOU 1567  C   THR A 218    15095  35505  19127  -3899   -952
7019       C
ATOM   1568  O   THR A 218     -35.153 -18.836  29.471  1.00197.47
O
ANISOU 1568  O   THR A 218    16896  37323  20812  -4003  -1099
7115       O
ATOM   1569  CB  THR A 218     -33.305 -20.363  31.704  1.00172.68
C
ANISOU 1569  CB  THR A 218    14761  33067  17781  -3945   -787
```

```
5955       C
ATOM   1570  OG1 THR A 218     -32.874 -21.591  32.302  1.00177.00
O
ANISOU 1570  OG1 THR A 218    15711  33329  18211  -4302   -861
5613       O
ATOM   1571  CG2 THR A 218     -33.277 -19.278  32.774  1.00175.07
C
ANISOU 1571  CG2 THR A 218    14805  33300  18412  -3217   -380
5964       C
ATOM   1572  N   PHE A 219     -35.915 -18.496  31.574  1.00185.86
N
ANISOU 1572  N   PHE A 219    14920  36038  19663  -3403   -675
7294       N
ATOM   1573  CA  PHE A 219     -36.599 -17.238  31.278  1.00199.95
C
ANISOU 1573  CA  PHE A 219    16152  38181  21637  -2942   -522
7778       C
ATOM   1574  C   PHE A 219     -35.682 -16.214  30.609  1.00208.97
C
ANISOU 1574  C   PHE A 219    17542  38926  22933  -2534   -368
7546       C
ATOM   1575  O   PHE A 219     -36.130 -15.440  29.756  1.00224.00
O
ANISOU 1575  O   PHE A 219    19116  41123  24871  -2410   -417
7941       O
ATOM   1576  CB  PHE A 219     -37.216 -16.676  32.563  1.00197.82
C
ANISOU 1576  CB  PHE A 219    15459  38078  21626  -2422   -162
7983       C
ATOM   1577  CG  PHE A 219     -36.261 -15.871  33.403  1.00188.49
C
ANISOU 1577  CG  PHE A 219    14563  36329  20725  -1782    275
7535       C
ATOM   1578  CD1 PHE A 219     -36.306 -14.486  33.399  1.00187.60
C
ANISOU 1578  CD1 PHE A 219    14206  36187  20886  -1137    609
7698       C
ATOM   1579  CD2 PHE A 219     -35.326 -16.503  34.207  1.00193.33
C
ANISOU 1579  CD2 PHE A 219    15692  36442  21321  -1841    356
6967       C
ATOM   1580  CE1 PHE A 219     -35.430 -13.746  34.176  1.00185.61
C
ANISOU 1580  CE1 PHE A 219    14245  35414  20864   -591   1013
7280       C
ATOM   1581  CE2 PHE A 219     -34.448 -15.770  34.985  1.00202.82
C
ANISOU 1581  CE2 PHE A 219    17155  37153  22755  -1298    741
6571       C
ATOM   1582  CZ  PHE A 219     -34.501 -14.389  34.969  1.00193.91
C
ANISOU 1582  CZ  PHE A 219    15803  35998  21876   -687   1070
6717       C
ATOM   1583  N   PHE A 220     -34.402 -16.183  30.994  1.00197.07
```

```
N
ANISOU 1583  N   PHE A 220    16595  36763  21518  -2321   -181
6929       N
ATOM   1584  CA  PHE A 220     -33.425 -15.345  30.297  1.00194.70
C
ANISOU 1584  CA  PHE A 220    16599  36055  21322  -2019    -71
6658       C
ATOM   1585  C   PHE A 220     -33.342 -15.671  28.808  1.00206.35
C
ANISOU 1585  C   PHE A 220    18220  37638  22547  -2512   -438
6737       C
ATOM   1586  O   PHE A 220     -33.303 -14.762  27.970  1.00203.03
O
ANISOU 1586  O   PHE A 220    17678  37272  22192  -2296   -420
6913       O
ATOM   1587  CB  PHE A 220     -32.053 -15.498  30.960  1.00171.90
C
ANISOU 1587  CB  PHE A 220    14312  32479  18525  -1833    132
5981       C
ATOM   1588  CG  PHE A 220     -30.943 -14.783  30.240  1.00166.90
C
ANISOU 1588  CG  PHE A 220    14051  31397  17966  -1593    220
5651       C
ATOM   1589  CD1 PHE A 220     -30.015 -15.492  29.492  1.00155.04
C
ANISOU 1589  CD1 PHE A 220    13077  29549  16283  -1986      8
5261       C
ATOM   1590  CD2 PHE A 220     -30.822 -13.405  30.316  1.00162.28
C
ANISOU 1590  CD2 PHE A 220    13300  30724  17635   -974    534
5729       C
ATOM   1591  CE1 PHE A 220     -28.993 -14.838  28.829  1.00154.92
C
ANISOU 1591  CE1 PHE A 220    13394  29135  16334  -1770     92
4964       C
ATOM   1592  CE2 PHE A 220     -29.801 -12.745  29.655  1.00157.91
C
ANISOU 1592  CE2 PHE A 220    13091  29765  17142   -769    610
5430       C
ATOM   1593  CZ  PHE A 220     -28.886 -13.464  28.910  1.00155.50
C
ANISOU 1593  CZ  PHE A 220    13288  29143  16651  -1169    383
5050       C
ATOM   1594  N   GLY A 221     -33.314 -16.957  28.457  1.00199.93
N
ANISOU 1594  N   GLY A 221    17681  36846  21436  -3186   -761
6610       N
ATOM   1595  CA  GLY A 221     -33.321 -17.329  27.049  1.00171.08
C
ANISOU 1595  CA  GLY A 221    14172  33326  17503  -3722  -1109
6700       C
ATOM   1596  C   GLY A 221     -34.643 -17.046  26.360  1.00156.07
C
ANISOU 1596  C   GLY A 221    11654  32153  15490  -3924  -1332
```

```
7408       C
ATOM   1597  O   GLY A 221     -34.673 -16.627  25.199  1.00157.63
O
ANISOU 1597  O   GLY A 221    11808  32496  15588  -4054  -1495
7597       O
ATOM   1598  N   LYS A 222     -35.751 -17.270  27.067  1.00159.22
N
ANISOU 1598  N   LYS A 222    11558  33036  15903  -3962  -1344
7827       N
ATOM   1599  CA  LYS A 222     -37.075 -16.899  26.572  1.00182.04
C
ANISOU 1599  CA  LYS A 222    13765  36664  18738  -4066  -1513
8568       C
ATOM   1600  C   LYS A 222     -37.208 -15.395  26.342  1.00200.46
C
ANISOU 1600  C   LYS A 222    15715  39081  21368  -3391  -1264
8872       C
ATOM   1601  O   LYS A 222     -37.767 -14.962  25.328  1.00203.18
O
ANISOU 1601  O   LYS A 222    15730  39840  21629  -3521  -1459
9341       O
ATOM   1602  CB  LYS A 222     -38.133 -17.387  27.565  1.00167.53
C
ANISOU 1602  CB  LYS A 222    11483  35264  16906  -4145  -1502
8907       C
ATOM   1603  CG  LYS A 222     -39.579 -17.116  27.184  1.00173.66
C
ANISOU 1603  CG  LYS A 222    11500  36856  17625  -4275  -1677
9716       C
ATOM   1604  CD  LYS A 222     -39.952 -17.804  25.884  1.00184.67
C
ANISOU 1604  CD  LYS A 222    12924  38625  18617  -5071  -2161
9963       C
ATOM   1605  CE  LYS A 222     -41.464 -17.876  25.721  1.00187.06
C
ANISOU 1605  CE  LYS A 222    12743  39565  18766  -5261  -2336
10671       C
ATOM   1606  NZ  LYS A 222     -42.096 -16.531  25.624  1.00187.96
N
ANISOU 1606  NZ  LYS A 222    12405  39864  19147  -4590  -2107
11162       N
ATOM   1607  N   ALA A 223     -36.705 -14.584  27.276  1.00193.82
N
ANISOU 1607  N   ALA A 223    14923  37852  20868  -2682   -829
8622       N
ATOM   1608  CA  ALA A 223     -36.754 -13.128  27.137  1.00194.50
C
ANISOU 1608  CA  ALA A 223    14709  37936  21256  -2003   -537
8861       C
ATOM   1609  C   ALA A 223     -36.005 -12.615  25.908  1.00212.49
C
ANISOU 1609  C   ALA A 223    17281  39976  23480  -2025   -644
8718       C
ATOM   1610  O   ALA A 223     -36.485 -11.705  25.223  1.00223.00
```

```
O
ANISOU 1610  O   ALA A 223    18222  41603  24905  -1796   -639
9180       O
ATOM   1611  CB  ALA A 223     -36.203 -12.467  28.402  1.00183.34
C
ANISOU 1611  CB  ALA A 223    13425  36064  20172  -1316    -43
8512       C
ATOM   1612  N   ALA A 224     -34.835 -13.178  25.606  1.00203.80
N
ANISOU 1612  N   ALA A 224    16853  38346  22235  -2289   -732
8103       N
ATOM   1613  CA  ALA A 224     -34.066 -12.757  24.435  1.00195.63
C
ANISOU 1613  CA  ALA A 224    16136  37065  21130  -2343   -833
7929       C
ATOM   1614  C   ALA A 224     -34.753 -12.985  23.090  1.00201.09
C
ANISOU 1614  C   ALA A 224    16597  38275  21534  -2899  -1248
8396       C
ATOM   1615  O   ALA A 224     -34.907 -14.126  22.642  1.00194.83
O
ANISOU 1615  O   ALA A 224    15990  37634  20403  -3610  -1590
8354       O
ATOM   1616  CB  ALA A 224     -32.708 -13.463  24.430  1.00151.58
C
ANISOU 1616  CB  ALA A 224    11318  30836  15439  -2562   -845
7185       C
ATOM   1617  N   HIS A 225     -35.165 -11.890  22.442  1.00199.63
N
ANISOU 1617  N   HIS A 225    16015  38359  21476  -2586  -1211
8850       N
ATOM   1618  CA  HIS A 225     -35.842 -11.929  21.148  1.00209.23
C
ANISOU 1618  CA  HIS A 225    16951  40107  22439  -3061  -1593
9361       C
ATOM   1619  C   HIS A 225     -34.874 -11.708  19.988  1.00225.86
C
ANISOU 1619  C   HIS A 225    19534  41873  24411  -3225  -1710
9046       C
ATOM   1620  O   HIS A 225     -34.277 -12.665  19.485  1.00246.16
O
ANISOU 1620  O   HIS A 225    22628  44221  26682  -3806  -1938
8643       O
ATOM   1621  CB  HIS A 225     -36.971 -10.896  21.098  1.00207.55
C
ANISOU 1621  CB  HIS A 225    15958  40459  22442  -2646  -1504
10134       C
ATOM   1622  CG  HIS A 225     -38.204 -11.314  21.836  1.00213.70
C
ANISOU 1622  CG  HIS A 225    16170  41796  23229  -2734  -1542
10622       C
ATOM   1623  ND1 HIS A 225     -38.230 -11.504  23.200  1.00210.98
N
ANISOU 1623  ND1 HIS A 225    15821  41266  23077  -2413  -1246
```

```
10406       N
ATOM   1624  CD2 HIS A 225     -39.456 -11.582  21.396  1.00236.34
C
ANISOU 1624  CD2 HIS A 225    18449  45424  25926  -3126  -1844
11328       C
ATOM   1625  CE1 HIS A 225     -39.445 -11.870  23.569  1.00221.48
C
ANISOU 1625  CE1 HIS A 225    16586  43205  24361  -2589  -1355
10948       C
ATOM   1626  NE2 HIS A 225     -40.209 -11.924  22.492  1.00238.68
N
ANISOU 1626  NE2 HIS A 225    18552  45839  26297  -2997  -1691
11461       N
ATOM   1627  N   LEU A 226     -34.712 -10.458  19.553  1.00225.02
N
ANISOU 1627  N   LEU A 226    19262  41714  24523  -2719  -1541
9226       N
ATOM   1628  CA  LEU A 226     -33.844 -10.127  18.428  1.00214.10
C
ANISOU 1628  CA  LEU A 226    18280  40042  23026  -2836  -1640
8979       C
ATOM   1629  C   LEU A 226     -32.497  -9.606  18.918  1.00209.94
C
ANISOU 1629  C   LEU A 226    18279  38750  22740  -2309  -1273
8310       C
ATOM   1630  O   LEU A 226     -32.437  -8.674  19.727  1.00211.46
O
ANISOU 1630  O   LEU A 226    18296  38763  23285  -1607   -892
8323       O
ATOM   1631  CB  LEU A 226     -34.502  -9.090  17.515  1.00187.17
C
ANISOU 1631  CB  LEU A 226    14364  37077  19674  -2669  -1724
9629       C
ATOM   1632  CG  LEU A 226     -35.858  -9.454  16.907  1.00176.40
C
ANISOU 1632  CG  LEU A 226    12413  36537  18073  -3180  -2106
10384       C
ATOM   1633  CD1 LEU A 226     -36.407  -8.293  16.093  1.00179.35
C
ANISOU 1633  CD1 LEU A 226    12280  37297  18569  -2899  -2133
11028       C
ATOM   1634  CD2 LEU A 226     -35.739 -10.704  16.050  1.00175.66
C
ANISOU 1634  CD2 LEU A 226    12699  36557  17488  -4124  -2547
10208       C
ATOM   1635  N   VAL A 227     -31.422 -10.220  18.416  1.00177.21
N
ANISOU 1635  N   VAL A 227    14781  34159  18391  -2668  -1381
7733       N
ATOM   1636  CA  VAL A 227     -30.063  -9.809  18.753  1.00182.83
C
ANISOU 1636  CA  VAL A 227    16021  34159  19288  -2253  -1078
7092       C
ATOM   1637  C   VAL A 227     -29.782  -8.380  18.298  1.00196.61
```

```
C
ANISOU 1637  C   VAL A 227    17649  35795  21261  -1702   -879
7231       C
ATOM   1638  O   VAL A 227     -30.226  -7.941  17.228  1.00177.85
O
ANISOU 1638  O   VAL A 227    15031  33764  18780  -1855  -1080
7652       O
ATOM   1639  CB  VAL A 227     -29.051 -10.800  18.150  1.00193.97
C
ANISOU 1639  CB  VAL A 227    18108  35179  20414  -2800  -1261
6522       C
ATOM   1640  CG1 VAL A 227     -29.061 -10.729  16.628  1.00200.00
C
ANISOU 1640  CG1 VAL A 227    18943  36140  20906  -3243  -1560
6700       C
ATOM   1641  CG2 VAL A 227     -27.650 -10.540  18.698  1.00141.70
C
ANISOU 1641  CG2 VAL A 227    12014  27835  13991  -2388   -943
5852       C
ATOM   1642  N   ASP A 228     -29.062  -7.634  19.137  1.00216.89
N
ANISOU 1642  N   ASP A 228    20383  37888  24138  -1066   -478
6894       N
ATOM   1643  CA  ASP A 228     -28.668  -6.265  18.837  1.00209.07
C
ANISOU 1643  CA  ASP A 228    19359  36697  23382   -507   -233
6946       C
ATOM   1644  C   ASP A 228     -27.244  -6.053  19.341  1.00166.54
C
ANISOU 1644  C   ASP A 228    14556  30597  18123   -203     51
6238       C
ATOM   1645  O   ASP A 228     -26.729  -6.842  20.138  1.00140.46
O
ANISOU 1645  O   ASP A 228    11568  27006  14795   -308    112
5797       O
ATOM   1646  CB  ASP A 228     -29.627  -5.239  19.459  1.00204.17
C
ANISOU 1646  CB  ASP A 228    18135  36369  23073     88     37
7484       C
ATOM   1647  CG  ASP A 228     -29.497  -3.862  18.828  1.00200.34
C
ANISOU 1647  CG  ASP A 228    17524  35821  22777    551    202
7715       C
ATOM   1648  OD1 ASP A 228     -30.200  -2.931  19.274  1.00194.69
O
ANISOU 1648  OD1 ASP A 228    16353  35283  22339   1085    468
8144       O
ATOM   1649  OD2 ASP A 228     -28.693  -3.713  17.884  1.00190.01
O
ANISOU 1649  OD2 ASP A 228    16577  34276  21342    383     80
7471       O
ATOM   1650  N   SER A 229     -26.610  -4.989  18.840  1.00164.41
N
ANISOU 1650  N   SER A 229    14424  30060  17983    155    212
```

```
6154       N
ATOM   1651  CA  SER A 229     -25.210  -4.665  19.117  1.00173.17
C
ANISOU 1651  CA  SER A 229    16083  30520  19194    421    458
5520       C
ATOM   1652  C   SER A 229     -24.828  -4.880  20.578  1.00206.70
C
ANISOU 1652  C   SER A 229    20485  34445  23608    707    752
5150       C
ATOM   1653  O   SER A 229     -25.474  -4.355  21.488  1.00235.65
O
ANISOU 1653  O   SER A 229    23807  38236  27492   1122   1005
5394       O
ATOM   1654  CB  SER A 229     -24.929  -3.215  18.718  1.00155.15
C
ANISOU 1654  CB  SER A 229    13748  28083  17117    937    688
5638       C
ATOM   1655  OG  SER A 229     -25.173  -3.012  17.338  1.00148.45
O
ANISOU 1655  OG  SER A 229    12797  27505  16104    658    409
5952       O
ATOM   1656  N   THR A 230     -23.769  -5.661  20.790  1.00178.42
N
ANISOU 1656  N   THR A 230    17423  30449  19920    477    726
4568       N
ATOM   1657  CA  THR A 230     -23.252  -5.974  22.118  1.00173.25
C
ANISOU 1657  CA  THR A 230    16975  29464  19388    676    967
4174       C
ATOM   1658  C   THR A 230     -21.792  -5.548  22.210  1.00190.45
C
ANISOU 1658  C   THR A 230    19665  31050  21646    907   1174
3614       C
ATOM   1659  O   THR A 230     -20.958  -6.020  21.430  1.00196.14
O
ANISOU 1659  O   THR A 230    20772  31547  22204    590   1006
3303       O
ATOM   1660  CB  THR A 230     -23.396  -7.465  22.431  1.00156.43
C
ANISOU 1660  CB  THR A 230    14958  27418  17058    156    733
4034       C
ATOM   1661  OG1 THR A 230     -24.783  -7.825  22.414  1.00177.74
O
ANISOU 1661  OG1 THR A 230    17159  30691  19681    -57    550
4573       O
ATOM   1662  CG2 THR A 230     -22.812  -7.781  23.803  1.00121.58
C
ANISOU 1662  CG2 THR A 230    10763  22661  12771    359    975
3635       C
ATOM   1663  N   ASN A 231     -21.491  -4.659  23.161  1.00204.88
N
ANISOU 1663  N   ASN A 231    21500  32631  23715   1444   1547
3491       N
ATOM   1664  CA  ASN A 231     -20.164  -4.048  23.305  1.00201.77
```

```
C
ANISOU 1664  CA  ASN A 231    21542  31709  23414   1715   1777
3015       C
ATOM   1665  C   ASN A 231     -19.741  -3.343  22.012  1.00220.09
C
ANISOU 1665  C   ASN A 231    23980  33954  25690   1724   1700
3048       C
ATOM   1666  O   ASN A 231     -18.662  -3.574  21.462  1.00220.92
O
ANISOU 1666  O   ASN A 231    24504  33741  25694   1547   1625
2659       O
ATOM   1667  CB  ASN A 231     -19.129  -5.089  23.752  1.00165.56
C
ANISOU 1667  CB  ASN A 231    17403  26775  18727   1445   1721
2483       C
ATOM   1668  CG  ASN A 231     -17.760  -4.483  24.016  1.00138.18
C
ANISOU 1668  CG  ASN A 231    14353  22791  15356   1721   1961
2012       C
ATOM   1669  OD1 ASN A 231     -16.746  -4.965  23.511  1.00139.60
O
ANISOU 1669  OD1 ASN A 231    14919  22693  15429   1488   1850
1653       O
ATOM   1670  ND2 ASN A 231     -17.727  -3.423  24.816  1.00132.69
N
ANISOU 1670  ND2 ASN A 231    13588  21969  14861   2209   2306
2017       N
ATOM   1671  N   GLN A 232     -20.636  -2.485  21.518  1.00210.45
N
ANISOU 1671  N   GLN A 232    22364  33048  24550   1931   1719
3545       N
ATOM   1672  CA  GLN A 232     -20.420  -1.771  20.261  1.00199.70
C
ANISOU 1672  CA  GLN A 232    21041  31690  23148   1937   1629
3673       C
ATOM   1673  C   GLN A 232     -19.196  -0.858  20.319  1.00192.31
C
ANISOU 1673  C   GLN A 232    20496  30243  22331   2286   1901
3268       C
ATOM   1674  O   GLN A 232     -18.476  -0.708  19.325  1.00166.99
O
ANISOU 1674  O   GLN A 232    17555  26874  19017   2137   1782
3103       O
ATOM   1675  CB  GLN A 232     -21.671  -0.968  19.903  1.00183.37
C
ANISOU 1675  CB  GLN A 232    18424  30063  21186   2161   1641
4334       C
ATOM   1676  CG  GLN A 232     -21.657  -0.393  18.497  1.00181.94
C
ANISOU 1676  CG  GLN A 232    18208  29997  20922   2071   1467
4570       C
ATOM   1677  CD  GLN A 232     -23.009   0.146  18.075  1.00168.92
C
ANISOU 1677  CD  GLN A 232    15962  28877  19343   2186   1398
```

```
5297       C
ATOM   1678  OE1 GLN A 232     -23.773  -0.530  17.386  1.00173.30
O
ANISOU 1678  OE1 GLN A 232    16261  29885  19700   1739   1046
5646       O
ATOM   1679  NE2 GLN A 232     -23.314   1.369  18.490  1.00174.38
N
ANISOU 1679  NE2 GLN A 232    16428  29514  20315   2780   1745
5541       N
ATOM   1680  N   VAL A 233     -18.943  -0.248  21.475  1.00217.72
N
ANISOU 1680  N   VAL A 233    23765  33206  25753   2728   2269
3102       N
ATOM   1681  CA  VAL A 233     -17.975   0.837  21.627  1.00207.86
C
ANISOU 1681  CA  VAL A 233    22817  31522  24639   3118   2575
2810       C
ATOM   1682  C   VAL A 233     -16.572   0.356  21.984  1.00209.48
C
ANISOU 1682  C   VAL A 233    23540  31281  24772   2984   2606
2189       C
ATOM   1683  O   VAL A 233     -15.724   1.161  22.387  1.00214.79
O
ANISOU 1683  O   VAL A 233    24478  31584  25551   3291   2885
1903       O
ATOM   1684  CB  VAL A 233     -18.470   1.847  22.681  1.00185.88
C
ANISOU 1684  CB  VAL A 233    19827  28695  22103   3665   2991
2978       C
ATOM   1685  CG1 VAL A 233     -19.780   2.480  22.235  1.00174.01
C
ANISOU 1685  CG1 VAL A 233    17804  27604  20708   3865   3001
3624       C
ATOM   1686  CG2 VAL A 233     -18.630   1.169  24.039  1.00141.87
C
ANISOU 1686  CG2 VAL A 233    14236  23111  16558   3656   3107
2819       C
ATOM   1687  N   GLY A 234     -16.321  -0.949  21.868  1.00186.67
N
ANISOU 1687  N   GLY A 234    20797  28422  21706   2528   2336
1990       N
ATOM   1688  CA  GLY A 234     -15.016  -1.513  22.180  1.00148.71
C
ANISOU 1688  CA  GLY A 234    16449  23215  16838   2388   2352
1443       C
ATOM   1689  C   GLY A 234     -13.817  -0.780  21.600  1.00120.87
C
ANISOU 1689  C   GLY A 234    13283  19321  13320   2512   2453
1135       C
ATOM   1690  O   GLY A 234     -13.704  -0.588  20.386  1.00101.72
O
ANISOU 1690  O   GLY A 234    10906  16942  10800   2366   2290
1215       O
ATOM   1691  N   HIS A 235     -12.920  -0.366  22.501  1.00133.29
```

```
N
ANISOU 1691  N   HIS A 235    15109  20539  14996   2766   2723
786       N
ATOM   1692  CA  HIS A 235     -11.889   0.629  22.199  1.00158.22
C
ANISOU 1692  CA  HIS A 235    18558  23357  18202   2992   2904
541       C
ATOM   1693  C   HIS A 235     -10.919   0.186  21.108  1.00161.17
C
ANISOU 1693  C   HIS A 235    19247  23564  18425   2684   2695
279       C
ATOM   1694  O   HIS A 235     -10.498   1.000  20.278  1.00162.46
O
ANISOU 1694  O   HIS A 235    19526  23616  18585   2782   2728
268       O
ATOM   1695  CB  HIS A 235     -11.118   0.962  23.476  1.00132.93
C
ANISOU 1695  CB  HIS A 235    15572  19838  15099   3236   3204
209       C
ATOM   1696  CG  HIS A 235     -10.138   2.082  23.319  1.00 95.32
C
ANISOU 1696  CG  HIS A 235    11090  14741  10387   3486   3425
-18       C
ATOM   1697  ND1 HIS A 235      -8.776   1.882  23.342  1.00 89.38
N
ANISOU 1697  ND1 HIS A 235    10718  13666   9575   3377   3432
-448       N
ATOM   1698  CD2 HIS A 235     -10.319   3.410  23.130  1.00108.78
C
ANISOU 1698  CD2 HIS A 235    12751  16383  12196   3832   3652
137       C
ATOM   1699  CE1 HIS A 235      -8.159   3.040  23.186  1.00 89.72
C
ANISOU 1699  CE1 HIS A 235    10942  13476   9670   3626   3643
-556       C
ATOM   1700  NE2 HIS A 235      -9.073   3.983  23.050  1.00 96.83
N
ANISOU 1700  NE2 HIS A 235    11607  14514  10670   3905   3783
-214       N
ATOM   1701  N   PHE A 236     -10.534  -1.091  21.107  1.00135.40
N
ANISOU 1701  N   PHE A 236    16139  20265  15043   2319   2502
62       N
ATOM   1702  CA  PHE A 236      -9.566  -1.591  20.132  1.00141.32
C
ANISOU 1702  CA  PHE A 236    17215  20826  15655   2027   2342
-214       C
ATOM   1703  C   PHE A 236     -10.017  -1.348  18.694  1.00 93.60
C
ANISOU 1703  C   PHE A 236    11086  14985   9493   1852   2143
38       C
ATOM   1704  O   PHE A 236      -9.221  -0.932  17.844  1.00 92.63
O
ANISOU 1704  O   PHE A 236    11202  14677   9319   1828   2139
```

```
-128       O
ATOM   1705  CB  PHE A 236      -9.318  -3.080  20.382  1.00149.68
C
ANISOU 1705  CB  PHE A 236    18408  21848  16613   1660   2178
-413       C
ATOM   1706  CG  PHE A 236      -8.480  -3.748  19.330  1.00124.30
C
ANISOU 1706  CG  PHE A 236    15513  18465  13249   1324   2018
-660       C
ATOM   1707  CD1 PHE A 236      -8.891  -4.936  18.749  1.00139.18
C
ANISOU 1707  CD1 PHE A 236    17414  20500  14969    884   1769
-604       C
ATOM   1708  CD2 PHE A 236      -7.269  -3.200  18.937  1.00115.40
C
ANISOU 1708  CD2 PHE A 236    14685  17022  12140   1435   2133
-955       C
ATOM   1709  CE1 PHE A 236      -8.116  -5.558  17.784  1.00167.67
C
ANISOU 1709  CE1 PHE A 236    21345  23926  18436    572   1659
-846       C
ATOM   1710  CE2 PHE A 236      -6.491  -3.815  17.974  1.00123.73
C
ANISOU 1710  CE2 PHE A 236    16035  17914  13062   1136   2014
-1183       C
ATOM   1711  CZ  PHE A 236      -6.915  -4.996  17.397  1.00152.22
C
ANISOU 1711  CZ  PHE A 236    19672  21655  16511    709   1788
-1134       C
ATOM   1712  N   GLN A 237     -11.280  -1.635  18.393  1.00102.40
N
ANISOU 1712  N   GLN A 237    11861  16497  10550   1700   1964
446       N
ATOM   1713  CA  GLN A 237     -11.810  -1.384  17.054  1.00132.26
C
ANISOU 1713  CA  GLN A 237    15519  20527  14205   1511   1757
745       C
ATOM   1714  C   GLN A 237     -11.788   0.100  16.686  1.00135.33
C
ANISOU 1714  C   GLN A 237    15830  20875  14714   1895   1926
912       C
ATOM   1715  O   GLN A 237     -11.462   0.458  15.547  1.00161.88
O
ANISOU 1715  O   GLN A 237    19313  24217  17978   1780   1824
924       O
ATOM   1716  CB  GLN A 237     -13.231  -1.944  16.954  1.00165.42
C
ANISOU 1716  CB  GLN A 237    19321  25202  18328   1286   1541
1196       C
ATOM   1717  CG  GLN A 237     -13.675  -2.364  15.551  1.00164.86
C
ANISOU 1717  CG  GLN A 237    19209  25409  18022    833   1218
1419       C
ATOM   1718  CD  GLN A 237     -13.731  -1.214  14.564  1.00173.72
```

```
C
ANISOU 1718  CD  GLN A 237    20252  26601  19152    988   1218
1650       C
ATOM   1719  OE1 GLN A 237     -14.491  -0.263  14.745  1.00164.72
O
ANISOU 1719  OE1 GLN A 237    18764  25659  18163   1332   1325
2037       O
ATOM   1720  NE2 GLN A 237     -12.925  -1.298  13.512  1.00180.41
N
ANISOU 1720  NE2 GLN A 237    21425  27281  19842    739   1113
1423       N
ATOM   1721  N   LYS A 238     -12.121   0.978  17.635  1.00120.53
N
ANISOU 1721  N   LYS A 238    13776  18974  13048   2348   2200
1037       N
ATOM   1722  CA  LYS A 238     -12.081   2.421  17.388  1.00105.90
C
ANISOU 1722  CA  LYS A 238    11878  17032  11328   2746   2410
1184       C
ATOM   1723  C   LYS A 238     -10.678   2.956  17.092  1.00101.84
C
ANISOU 1723  C   LYS A 238    11793  16097  10806   2827   2538
763       C
ATOM   1724  O   LYS A 238     -10.499   3.741  16.154  1.00139.97
O
ANISOU 1724  O   LYS A 238    16670  20898  15616   2888   2526
860       O
ATOM   1725  CB  LYS A 238     -12.685   3.158  18.584  1.00117.40
C
ANISOU 1725  CB  LYS A 238    13103  18499  13005   3201   2723
1357       C
ATOM   1726  CG  LYS A 238     -14.088   2.698  18.951  1.00105.42
C
ANISOU 1726  CG  LYS A 238    11131  17407  11515   3162   2631
1793       C
ATOM   1727  CD  LYS A 238     -15.008   2.685  17.740  1.00153.43
C
ANISOU 1727  CD  LYS A 238    16902  23895  17499   2956   2351
2270       C
ATOM   1728  CE  LYS A 238     -16.413   2.240  18.119  1.00182.84
C
ANISOU 1728  CE  LYS A 238    20147  28073  21250   2909   2256
2733       C
ATOM   1729  NZ  LYS A 238     -17.317   2.180  16.937  1.00183.96
N
ANISOU 1729  NZ  LYS A 238    19968  28655  21274   2661   1956
3227       N
ATOM   1730  N   VAL A 239      -9.671   2.564  17.876  1.00102.53
N
ANISOU 1730  N   VAL A 239    12183  15868  10906   2826   2658
315       N
ATOM   1731  CA  VAL A 239      -8.297   2.950  17.537  1.00130.70
C
ANISOU 1731  CA  VAL A 239    16148  19067  14444   2847   2747
```

```
-78       C
ATOM   1732  C   VAL A 239      -7.848   2.329  16.218  1.00130.25
C
ANISOU 1732  C   VAL A 239    16271  19026  14194   2447   2476
-178       C
ATOM   1733  O   VAL A 239      -7.136   2.964  15.430  1.00128.91
O
ANISOU 1733  O   VAL A 239    16305  18689  13986   2474   2502
-295       O
ATOM   1734  CB  VAL A 239      -7.326   2.624  18.688  1.00163.59
C
ANISOU 1734  CB  VAL A 239    20567  22927  18661   2913   2921
-499       C
ATOM   1735  CG1 VAL A 239      -7.674   3.452  19.913  1.00201.33
C
ANISOU 1735  CG1 VAL A 239    25229  27656  23612   3313   3230
-424       C
ATOM   1736  CG2 VAL A 239      -7.329   1.145  19.018  1.00159.54
C
ANISOU 1736  CG2 VAL A 239    20073  22484  18063   2577   2736
-628       C
ATOM   1737  N   LEU A 240      -8.240   1.081  15.961  1.00120.74
N
ANISOU 1737  N   LEU A 240    15013  18009  12853   2058   2227
-144       N
ATOM   1738  CA  LEU A 240      -7.948   0.456  14.674  1.00 98.30
C
ANISOU 1738  CA  LEU A 240    12343  15203   9803   1641   1980
-212       C
ATOM   1739  C   LEU A 240      -8.545   1.237  13.505  1.00115.38
C
ANISOU 1739  C   LEU A 240    14344  17597  11900   1623   1863
144       C
ATOM   1740  O   LEU A 240      -7.883   1.434  12.479  1.00164.48
O
ANISOU 1740  O   LEU A 240    20792  23700  18002   1473   1797
11       O
ATOM   1741  CB  LEU A 240      -8.479  -0.978  14.687  1.00 93.86
C
ANISOU 1741  CB  LEU A 240    11726  14832   9105   1228   1753
-179       C
ATOM   1742  CG  LEU A 240      -8.036  -1.981  13.626  1.00113.04
C
ANISOU 1742  CG  LEU A 240    14425  17220  11304    734   1538
-364       C
ATOM   1743  CD1 LEU A 240      -6.559  -2.249  13.735  1.00120.98
C
ANISOU 1743  CD1 LEU A 240    15845  17795  12327    741   1674
-861       C
ATOM   1744  CD2 LEU A 240      -8.823  -3.273  13.775  1.00126.27
C
ANISOU 1744  CD2 LEU A 240    15992  19127  12860    358   1337
-248       C
ATOM   1745  N   THR A 241      -9.794   1.685  13.640  1.00 98.71
```

```
N
ANISOU 1745  N   THR A 241    11825  15820   9860   1772   1839
614       N
ATOM   1746  CA  THR A 241     -10.395   2.580  12.652  1.00101.57
C
ANISOU 1746  CA  THR A 241    11984  16407  10201   1831   1759
1012       C
ATOM   1747  C   THR A 241      -9.662   3.916  12.535  1.00103.01
C
ANISOU 1747  C   THR A 241    12328  16301  10511   2215   2002
906       C
ATOM   1748  O   THR A 241      -9.476   4.431  11.425  1.00133.68
O
ANISOU 1748  O   THR A 241    16280  20207  14305   2132   1910
997       O
ATOM   1749  CB  THR A 241     -11.868   2.813  12.989  1.00132.80
C
ANISOU 1749  CB  THR A 241    15436  20770  14251   1977   1729
1559       C
ATOM   1750  OG1 THR A 241     -12.542   1.551  13.085  1.00164.12
O
ANISOU 1750  OG1 THR A 241    19262  25015  18080   1586   1491
1656       O
ATOM   1751  CG2 THR A 241     -12.539   3.653  11.910  1.00125.92
C
ANISOU 1751  CG2 THR A 241    14319  20173  13354   2009   1616
2028       C
ATOM   1752  N   ALA A 242      -9.242   4.496  13.663  1.00117.30
N
ANISOU 1752  N   ALA A 242    14213  17841  12513   2614   2313
717       N
ATOM   1753  CA  ALA A 242      -8.495   5.754  13.625  1.00129.61
C
ANISOU 1753  CA  ALA A 242    15968  19099  14181   2957   2563
585       C
ATOM   1754  C   ALA A 242      -7.188   5.649  12.842  1.00111.43
C
ANISOU 1754  C   ALA A 242    14076  16524  11739   2742   2505
186       C
ATOM   1755  O   ALA A 242      -6.838   6.568  12.092  1.00 95.65
O
ANISOU 1755  O   ALA A 242    12177  14430   9734   2846   2551
226       O
ATOM   1756  CB  ALA A 242      -8.221   6.236  15.051  1.00130.33
C
ANISOU 1756  CB  ALA A 242    16115  18943  14461   3343   2904
403       C
ATOM   1757  N   ILE A 243      -6.444   4.553  13.004  1.00122.06
N
ANISOU 1757  N   ILE A 243    15663  17734  12981   2453   2419
-191        N
ATOM   1758  CA  ILE A 243      -5.283   4.323  12.143  1.00 98.22
C
ANISOU 1758  CA  ILE A 243    13005  14494   9818   2212   2348
```

```
-533       C
ATOM   1759  C   ILE A 243      -5.703   4.233  10.679  1.00 97.01
C
ANISOU 1759  C   ILE A 243    12801  14577   9479   1896   2087
-298       C
ATOM   1760  O   ILE A 243      -5.051   4.796   9.791  1.00130.70
O
ANISOU 1760  O   ILE A 243    17266  18718  13674   1862   2087
-392       O
ATOM   1761  CB  ILE A 243      -4.523   3.063  12.597  1.00109.59
C
ANISOU 1761  CB  ILE A 243    14676  15767  11197   1964   2312
-928       C
ATOM   1762  CG1 ILE A 243      -4.066   3.210  14.049  1.00107.96
C
ANISOU 1762  CG1 ILE A 243    14517  15343  11161   2262   2561
-1142       C
ATOM   1763  CG2 ILE A 243      -3.328   2.801  11.692  1.00 86.77
C
ANISOU 1763  CG2 ILE A 243    12150  12647   8170   1727   2266
-1269       C
ATOM   1764  CD1 ILE A 243      -3.762   1.889  14.722  1.00111.34
C
ANISOU 1764  CD1 ILE A 243    15030  15710  11564   2051   2506
-1380       C
ATOM   1765  N   GLY A 244      -6.802   3.528  10.410  1.00 95.74
N
ANISOU 1765  N   GLY A 244    12376  14777   9225   1639   1858
19       N
ATOM   1766  CA  GLY A 244      -7.388   3.529   9.077  1.00 98.61
C
ANISOU 1766  CA  GLY A 244    12633  15432   9402   1332   1600
326       C
ATOM   1767  C   GLY A 244      -7.810   4.904   8.591  1.00120.50
C
ANISOU 1767  C   GLY A 244    15214  18310  12262   1626   1659
690       C
ATOM   1768  O   GLY A 244      -7.612   5.250   7.425  1.00159.28
O
ANISOU 1768  O   GLY A 244    20218  23267  17035   1451   1536
761       O
ATOM   1769  N   ASN A 245      -8.427   5.692   9.468  1.00101.63
N
ANISOU 1769  N   ASN A 245    12554  15960  10102   2070   1857
939       N
ATOM   1770  CA  ASN A 245      -8.850   7.043   9.110  1.00103.89
C
ANISOU 1770  CA  ASN A 245    12655  16309  10510   2409   1964
1304       C
ATOM   1771  C   ASN A 245      -7.687   7.939   8.682  1.00102.00
C
ANISOU 1771  C   ASN A 245    12766  15708  10281   2558   2123
1017       C
ATOM   1772  O   ASN A 245      -7.781   8.646   7.672  1.00115.31
```

```
O
ANISOU 1772  O   ASN A 245    14422  17476  11912   2545   2046
1247       O
ATOM   1773  CB  ASN A 245      -9.595   7.663  10.295  1.00137.54
C
ANISOU 1773  CB  ASN A 245    16627  20599  15035   2885   2220
1546       C
ATOM   1774  CG  ASN A 245     -10.060   9.079  10.022  1.00153.58
C
ANISOU 1774  CG  ASN A 245    18468  22658  17227   3287   2384
1937       C
ATOM   1775  OD1 ASN A 245      -9.663  10.017  10.714  1.00146.18
O
ANISOU 1775  OD1 ASN A 245    17650  21417  16476   3709   2717
1814       O
ATOM   1776  ND2 ASN A 245     -10.914   9.240   9.019  1.00154.37
N
ANISOU 1776  ND2 ASN A 245    18276  23124  17253   3148   2156
2425       N
ATOM   1777  N   PHE A 246      -6.581   7.924   9.431  1.00100.09
N
ANISOU 1777  N   PHE A 246    12849  15079  10101   2686   2337
531       N
ATOM   1778  CA  PHE A 246      -5.442   8.783   9.094  1.00100.76
C
ANISOU 1778  CA  PHE A 246    13267  14824  10192   2822   2498
252       C
ATOM   1779  C   PHE A 246      -4.802   8.471   7.739  1.00134.34
C
ANISOU 1779  C   PHE A 246    17752  19076  14214   2430   2280
111       C
ATOM   1780  O   PHE A 246      -4.505   9.392   6.968  1.00181.43
O
ANISOU 1780  O   PHE A 246    23806  24971  20159   2509   2309
186       O
ATOM   1781  CB  PHE A 246      -4.385   8.683  10.194  1.00117.40
C
ANISOU 1781  CB  PHE A 246    15658  16563  12385   2974   2739
-230       C
ATOM   1782  CG  PHE A 246      -3.190   9.562   9.964  1.00137.93
C
ANISOU 1782  CG  PHE A 246    18596  18821  14991   3108   2916
-522       C
ATOM   1783  CD1 PHE A 246      -1.910   9.038  10.034  1.00130.46
C
ANISOU 1783  CD1 PHE A 246    17990  17618  13961   2933   2936
-1000       C
ATOM   1784  CD2 PHE A 246      -3.345  10.906   9.669  1.00143.27
C
ANISOU 1784  CD2 PHE A 246    19245  19433  15758   3406   3067
-303       C
ATOM   1785  CE1 PHE A 246      -0.804   9.836   9.816  1.00112.90
C
ANISOU 1785  CE1 PHE A 246    16063  15103  11731   3035   3091
```

```
-1257       C
ATOM   1786  CE2 PHE A 246      -2.242  11.711   9.451  1.00134.84
C
ANISOU 1786  CE2 PHE A 246    18500  18051  14680   3504   3225
-573       C
ATOM   1787  CZ  PHE A 246      -0.970  11.175   9.525  1.00116.25
C
ANISOU 1787  CZ  PHE A 246    16473  15467  12229   3309   3231
-1052       C
ATOM   1788  N   CYS A 247      -4.575   7.193   7.425  1.00122.91
N
ANISOU 1788  N   CYS A 247    16422  17690  12588   2002   2079
-96       N
ATOM   1789  CA  CYS A 247      -4.055   6.833   6.102  1.00112.47
C
ANISOU 1789  CA  CYS A 247    15325  16383  11027   1596   1883
-217       C
ATOM   1790  C   CYS A 247      -5.006   7.195   4.961  1.00107.93
C
ANISOU 1790  C   CYS A 247    14511  16169  10328   1425   1653
264       C
ATOM   1791  O   CYS A 247      -4.568   7.706   3.923  1.00 99.98
O
ANISOU 1791  O   CYS A 247    13660  15125   9204   1312   1597
260       O
ATOM   1792  CB  CYS A 247      -3.687   5.347   6.049  1.00121.31
C
ANISOU 1792  CB  CYS A 247    16629  17477  11986   1176   1748
-527       C
ATOM   1793  SG  CYS A 247      -4.912   4.191   6.659  1.00156.90
S
ANISOU 1793  SG  CYS A 247    20830  22308  16476    984   1580
-290       S
ATOM   1794  N   ILE A 248      -6.305   6.940   5.130  1.00128.04
N
ANISOU 1794  N   ILE A 248    16670  19083  12894   1393   1512
699       N
ATOM   1795  CA  ILE A 248      -7.300   7.388   4.153  1.00106.64
C
ANISOU 1795  CA  ILE A 248    13664  16758  10094   1276   1300
1237       C
ATOM   1796  C   ILE A 248      -7.307   8.907   3.992  1.00107.78
C
ANISOU 1796  C   ILE A 248    13724  16818  10408   1709   1473
1479       C
ATOM   1797  O   ILE A 248      -7.402   9.419   2.869  1.00110.08
O
ANISOU 1797  O   ILE A 248    13998  17247  10580   1573   1333
1713       O
ATOM   1798  CB  ILE A 248      -8.694   6.860   4.543  1.00115.16
C
ANISOU 1798  CB  ILE A 248    14306  18254  11197   1209   1147
1676       C
ATOM   1799  CG1 ILE A 248      -8.725   5.331   4.480  1.00129.44
```

```
C
ANISOU 1799  CG1 ILE A 248    16227  20166  12788    696    939
1465       C
ATOM   1800  CG2 ILE A 248      -9.764   7.451   3.643  1.00115.82
C
ANISOU 1800  CG2 ILE A 248    14022  18759  11226   1152    948
2301       C
ATOM   1801  CD1 ILE A 248      -8.498   4.769   3.098  1.00136.18
C
ANISOU 1801  CD1 ILE A 248    17290  21142  13311    126    671
1413       C
ATOM   1802  N   CYS A 249      -7.206   9.652   5.093  1.00114.40
N
ANISOU 1802  N   CYS A 249    14525  17425  11517   2220   1787
1431       N
ATOM   1803  CA  CYS A 249      -7.053  11.103   4.987  1.00129.03
C
ANISOU 1803  CA  CYS A 249    16382  19112  13531   2636   2002
1587       C
ATOM   1804  C   CYS A 249      -5.810  11.511   4.198  1.00127.63
C
ANISOU 1804  C   CYS A 249    16616  18647  13232   2523   2028
1239       C
ATOM   1805  O   CYS A 249      -5.864  12.443   3.387  1.00153.92
O
ANISOU 1805  O   CYS A 249    19924  22006  16554   2606   2016
1485       O
ATOM   1806  CB  CYS A 249      -7.018  11.730   6.381  1.00120.18
C
ANISOU 1806  CB  CYS A 249    15239  17734  12690   3155   2368
1500       C
ATOM   1807  SG  CYS A 249      -8.578  11.632   7.284  1.00171.00
S
ANISOU 1807  SG  CYS A 249    21149  24501  19321   3410   2412
2004       S
ATOM   1808  N   SER A 250      -4.682  10.834   4.419  1.00102.51
N
ANISOU 1808  N   SER A 250    13799  15192   9959   2339   2069
685       N
ATOM   1809  CA  SER A 250      -3.472  11.126   3.651  1.00 99.66
C
ANISOU 1809  CA  SER A 250    13820  14576   9469   2201   2090
346       C
ATOM   1810  C   SER A 250      -3.657  10.918   2.149  1.00111.71
C
ANISOU 1810  C   SER A 250    15353  16350  10741   1777   1794
532       C
ATOM   1811  O   SER A 250      -3.219  11.748   1.343  1.00102.37
O
ANISOU 1811  O   SER A 250    14306  15083   9506   1798   1809
563       O
ATOM   1812  CB  SER A 250      -2.317  10.264   4.161  1.00 95.86
C
ANISOU 1812  CB  SER A 250    13677  13807   8937   2054   2169
```

```
-236       C
ATOM   1813  OG  SER A 250      -2.097  10.471   5.545  1.00107.29
O
ANISOU 1813  OG  SER A 250    15132  15037  10596   2412   2432
-408       O
ATOM   1814  N   ILE A 251      -4.304   9.822   1.752  1.00112.67
N
ANISOU 1814  N   ILE A 251    15343  16781  10685   1364   1525
657       N
ATOM   1815  CA  ILE A 251      -4.653   9.623   0.345  1.00106.00
C
ANISOU 1815  CA  ILE A 251    14472  16229   9575    923   1229
896       C
ATOM   1816  C   ILE A 251      -5.581  10.721  -0.167  1.00109.69
C
ANISOU 1816  C   ILE A 251    14602  16964  10112   1120   1162
1495       C
ATOM   1817  O   ILE A 251      -5.388  11.247  -1.270  1.00123.04
O
ANISOU 1817  O   ILE A 251    16376  18712  11661    963   1054
1618       O
ATOM   1818  CB  ILE A 251      -5.262   8.223   0.149  1.00107.16
C
ANISOU 1818  CB  ILE A 251    14537  16664   9516    435    969
933       C
ATOM   1819  CG1 ILE A 251      -4.216   7.151   0.457  1.00103.77
C
ANISOU 1819  CG1 ILE A 251    14494  15932   9003    216   1044
332       C
ATOM   1820  CG2 ILE A 251      -5.782   8.055  -1.269  1.00110.60
C
ANISOU 1820  CG2 ILE A 251    14912  17455   9656    -50    651
1244       C
ATOM   1821  CD1 ILE A 251      -4.794   5.772   0.625  1.00104.52
C
ANISOU 1821  CD1 ILE A 251    14523  16227   8962   -165    868
325       C
ATOM   1822  N   ALA A 252      -6.601  11.084   0.615  1.00130.51
N
ANISOU 1822  N   ALA A 252    16848  19768  12972   1468   1235
1891       N
ATOM   1823  CA  ALA A 252      -7.476  12.190   0.227  1.00115.66
C
ANISOU 1823  CA  ALA A 252    14624  18112  11208   1733   1221
2488       C
ATOM   1824  C   ALA A 252      -6.713  13.505   0.086  1.00137.46
C
ANISOU 1824  C   ALA A 252    17598  20531  14101   2098   1466
2396       C
ATOM   1825  O   ALA A 252      -6.928  14.255  -0.873  1.00150.70
O
ANISOU 1825  O   ALA A 252    19194  22340  15724   2078   1368
2736       O
ATOM   1826  CB  ALA A 252      -8.612  12.338   1.239  1.00117.65
```

```
C
ANISOU 1826  CB  ALA A 252    14442  18548  11712   2099   1325
2881       C
ATOM   1827  N   ILE A 253      -5.827  13.804   1.037  1.00141.10
N
ANISOU 1827  N   ILE A 253    18329  20558  14725   2418   1782
1953       N
ATOM   1828  CA  ILE A 253      -4.934  14.956   0.912  1.00130.93
C
ANISOU 1828  CA  ILE A 253    17318  18909  13522   2698   2017
1777       C
ATOM   1829  C   ILE A 253      -4.039  14.838  -0.318  1.00136.85
C
ANISOU 1829  C   ILE A 253    18388  19605  14005   2297   1849
1538       C
ATOM   1830  O   ILE A 253      -3.856  15.808  -1.066  1.00145.40
O
ANISOU 1830  O   ILE A 253    19529  20640  15075   2380   1867
1707       O
ATOM   1831  CB  ILE A 253      -4.105  15.125   2.199  1.00123.61
C
ANISOU 1831  CB  ILE A 253    16641  17554  12771   3023   2360
1309       C
ATOM   1832  CG1 ILE A 253      -5.009  15.536   3.362  1.00128.06
C
ANISOU 1832  CG1 ILE A 253    16905  18141  13612   3477   2582
1589       C
ATOM   1833  CG2 ILE A 253      -3.003  16.153   1.997  1.00124.94
C
ANISOU 1833  CG2 ILE A 253    17165  17343  12964   3200   2574
1044       C
ATOM   1834  CD1 ILE A 253      -5.679  16.881   3.172  1.00140.43
C
ANISOU 1834  CD1 ILE A 253    18263  19728  15366   3884   2735
2083       C
ATOM   1835  N   GLY A 254      -3.478  13.652  -0.554  1.00140.11
N
ANISOU 1835  N   GLY A 254    19015  20017  14201   1860   1698
1151       N
ATOM   1836  CA  GLY A 254      -2.641  13.455  -1.730  1.00180.63
C
ANISOU 1836  CA  GLY A 254    24461  25103  19069   1457   1556
911       C
ATOM   1837  C   GLY A 254      -3.390  13.525  -3.049  1.00132.16
C
ANISOU 1837  C   GLY A 254    18140  19356  12720   1118   1248
1366       C
ATOM   1838  O   GLY A 254      -2.992  14.254  -3.962  1.00111.04
O
ANISOU 1838  O   GLY A 254    15599  16639   9951   1063   1219
1426       O
ATOM   1839  N   MET A 255      -4.486  12.779  -3.168  1.00112.66
N
ANISOU 1839  N   MET A 255    15358  17286  10163    870   1006
```

```
1708       N
ATOM   1840  CA  MET A 255      -5.138  12.546  -4.455  1.00116.34
C
ANISOU 1840  CA  MET A 255    15685  18166  10354    403    663
2086       C
ATOM   1841  C   MET A 255      -6.334  13.470  -4.678  1.00130.97
C
ANISOU 1841  C   MET A 255    17073  20354  12337    645    578
2809       C
ATOM   1842  O   MET A 255      -6.380  14.192  -5.678  1.00122.84
O
ANISOU 1842  O   MET A 255    16023  19442  11210    564    468
3091       O
ATOM   1843  CB  MET A 255      -5.509  11.053  -4.595  1.00127.33
C
ANISOU 1843  CB  MET A 255    17074  19801  11505   -130    425
1988       C
ATOM   1844  CG  MET A 255      -6.885  10.620  -4.112  1.00132.35
C
ANISOU 1844  CG  MET A 255    17247  20835  12204   -131    276
2454       C
ATOM   1845  SD  MET A 255      -7.252   8.880  -4.366  1.00132.30
S
ANISOU 1845  SD  MET A 255    17300  21091  11876   -815     -3
2315       S
ATOM   1846  CE  MET A 255      -8.977   8.846  -3.888  1.00123.97
C
ANISOU 1846  CE  MET A 255    15606  20559  10939   -711   -172
3026       C
ATOM   1847  N   VAL A 256      -7.307  13.456  -3.765  1.00166.19
N
ANISOU 1847  N   VAL A 256    21150  24975  17019    946    634
3131       N
ATOM   1848  CA  VAL A 256      -8.556  14.191  -3.956  1.00193.68
C
ANISOU 1848  CA  VAL A 256    24133  28824  20631   1162    547
3870       C
ATOM   1849  C   VAL A 256      -8.299  15.690  -4.045  1.00209.16
C
ANISOU 1849  C   VAL A 256    26109  30551  22812   1654    781
4049       C
ATOM   1850  O   VAL A 256      -8.948  16.399  -4.824  1.00238.06
O
ANISOU 1850  O   VAL A 256    29503  34481  26468   1678    648
4611       O
ATOM   1851  CB  VAL A 256      -9.551  13.852  -2.828  1.00165.76
C
ANISOU 1851  CB  VAL A 256    20210  25457  17314   1427    617
4118       C
ATOM   1852  CG1 VAL A 256     -10.870  14.587  -3.037  1.00147.78
C
ANISOU 1852  CG1 VAL A 256    17382  23583  15185   1662    537
4921       C
ATOM   1853  CG2 VAL A 256      -9.781  12.350  -2.752  1.00154.03
```

```
C
ANISOU 1853  CG2 VAL A 256    18737  24190  15599    916    384
3930       C
ATOM   1854  N   ILE A 257      -7.354  16.196  -3.259  1.00175.45
N
ANISOU 1854  N   ILE A 257    22154  25780  18730   2037   1130
3593       N
ATOM   1855  CA  ILE A 257      -6.852  17.554  -3.450  1.00162.76
C
ANISOU 1855  CA  ILE A 257    20691  23879  17271   2414   1358
3639       C
ATOM   1856  C   ILE A 257      -5.744  17.682  -4.496  1.00135.23
C
ANISOU 1856  C   ILE A 257    17619  20226  13535   2085   1270
3303       C
ATOM   1857  O   ILE A 257      -6.022  17.899  -5.681  1.00160.23
O
ANISOU 1857  O   ILE A 257    20702  23661  16518   1805   1023
3640       O
ATOM   1858  CB  ILE A 257      -6.375  18.129  -2.103  1.00181.83
C
ANISOU 1858  CB  ILE A 257    23259  25837  19990   2967   1790
3328       C
ATOM   1859  CG1 ILE A 257      -7.449  17.933  -1.032  1.00208.60
C
ANISOU 1859  CG1 ILE A 257    26259  29387  23614   3274   1894
3624       C
ATOM   1860  CG2 ILE A 257      -6.059  19.613  -2.236  1.00172.92
C
ANISOU 1860  CG2 ILE A 257    22241  24424  19038   3390   2048
3464       C
ATOM   1861  CD1 ILE A 257      -8.790  18.543  -1.388  1.00208.43
C
ANISOU 1861  CD1 ILE A 257    25726  29742  23727   3476   1812
4411       C
ATOM   1862  N   GLU A 258      -4.488  17.535  -4.065  1.00117.87
N
ANISOU 1862  N   GLU A 258    15859  17603  11322   2103   1469
2654       N
ATOM   1863  CA  GLU A 258      -3.349  18.063  -4.819  1.00156.43
C
ANISOU 1863  CA  GLU A 258    21139  22225  16073   1989   1514
2348       C
ATOM   1864  C   GLU A 258      -3.218  17.497  -6.235  1.00186.03
C
ANISOU 1864  C   GLU A 258    24984  26240  19458   1382   1172
2372       C
ATOM   1865  O   GLU A 258      -2.984  18.254  -7.185  1.00189.83
O
ANISOU 1865  O   GLU A 258    25551  26730  19844   1316   1112
2532       O
ATOM   1866  CB  GLU A 258      -2.061  17.822  -4.033  1.00122.97
C
ANISOU 1866  CB  GLU A 258    17312  17537  11872   2074   1767
```

```
1656       C
ATOM   1867  CG  GLU A 258      -1.963  18.618  -2.743  1.00157.85
C
ANISOU 1867  CG  GLU A 258    21734  21629  16612   2652   2139
1585       C
ATOM   1868  CD  GLU A 258      -0.555  18.647  -2.185  1.00174.30
C
ANISOU 1868  CD  GLU A 258    24254  23273  18700   2708   2376
948       C
ATOM   1869  OE1 GLU A 258       0.337  19.206  -2.856  1.00183.67
O
ANISOU 1869  OE1 GLU A 258    25730  24273  19783   2628   2412
761       O
ATOM   1870  OE2 GLU A 258      -0.339  18.111  -1.078  1.00193.85
O
ANISOU 1870  OE2 GLU A 258    26771  25605  21278   2822   2521
650       O
ATOM   1871  N   ILE A 259      -3.346  16.180  -6.410  1.00183.57
N
ANISOU 1871  N   ILE A 259    24687  26134  18927    918    956
2205       N
ATOM   1872  CA  ILE A 259      -3.321  15.625  -7.768  1.00136.70
C
ANISOU 1872  CA  ILE A 259    18848  20469  12622    305    638
2253       C
ATOM   1873  C   ILE A 259      -4.479  16.158  -8.606  1.00144.37
C
ANISOU 1873  C   ILE A 259    19428  21891  13533    217    383
2981       C
ATOM   1874  O   ILE A 259      -4.303  16.533  -9.772  1.00164.05
O
ANISOU 1874  O   ILE A 259    22014  24502  15814    -62    222
3125       O
ATOM   1875  CB  ILE A 259      -3.304  14.087  -7.739  1.00132.09
C
ANISOU 1875  CB  ILE A 259    18369  20002  11817   -177    482
1946       C
ATOM   1876  CG1 ILE A 259      -2.049  13.580  -7.032  1.00118.27
C
ANISOU 1876  CG1 ILE A 259    17019  17801  10119   -105    732
1240       C
ATOM   1877  CG2 ILE A 259      -3.339  13.536  -9.153  1.00149.99
C
ANISOU 1877  CG2 ILE A 259    20757  22550  13681   -837    172
2008       C
ATOM   1878  CD1 ILE A 259      -2.031  12.085  -6.853  1.00112.13
C
ANISOU 1878  CD1 ILE A 259    16343  17083   9177   -502    631
940       C
ATOM   1879  N   ILE A 260      -5.684  16.176  -8.042  1.00128.02
N
ANISOU 1879  N   ILE A 260    16900  20099  11641    437    336
3468       N
ATOM   1880  CA  ILE A 260      -6.803  16.821  -8.723  1.00151.87
```

```
C
ANISOU 1880  CA  ILE A 260    19493  23544  14666    448    130
4225       C
ATOM   1881  C   ILE A 260      -6.529  18.306  -8.941  1.00161.15
C
ANISOU 1881  C   ILE A 260    20692  24502  16036    890    322
4435       C
ATOM   1882  O   ILE A 260      -6.843  18.861 -10.000  1.00163.27
O
ANISOU 1882  O   ILE A 260    20841  25015  16179    727    128
4866       O
ATOM   1883  CB  ILE A 260      -8.108  16.592  -7.938  1.00163.09
C
ANISOU 1883  CB  ILE A 260    20404  25277  16288    671     96
4700       C
ATOM   1884  CG1 ILE A 260      -8.452  15.102  -7.903  1.00164.80
C
ANISOU 1884  CG1 ILE A 260    20595  25763  16260    146   -145
4549       C
ATOM   1885  CG2 ILE A 260      -9.252  17.383  -8.552  1.00169.34
C
ANISOU 1885  CG2 ILE A 260    20710  26491  17142    775    -74
5533       C
ATOM   1886  CD1 ILE A 260      -8.710  14.503  -9.267  1.00165.27
C
ANISOU 1886  CD1 ILE A 260    20668  26233  15894   -572   -547
4737       C
ATOM   1887  N   VAL A 261      -5.945  18.971  -7.942  1.00162.88
N
ANISOU 1887  N   VAL A 261    21075  24258  16554   1436    706
4144       N
ATOM   1888  CA  VAL A 261      -5.559  20.378  -8.069  1.00158.36
C
ANISOU 1888  CA  VAL A 261    20604  23402  16165   1856    935
4262       C
ATOM   1889  C   VAL A 261      -4.571  20.616  -9.212  1.00142.11
C
ANISOU 1889  C   VAL A 261    18926  21232  13838   1507    834
4010       C
ATOM   1890  O   VAL A 261      -4.704  21.590  -9.962  1.00151.54
O
ANISOU 1890  O   VAL A 261    20058  22480  15041   1594    798
4390       O
ATOM   1891  CB  VAL A 261      -4.998  20.889  -6.727  1.00150.95
C
ANISOU 1891  CB  VAL A 261    19849  21964  15543   2418   1373
3891       C
ATOM   1892  CG1 VAL A 261      -4.222  22.184  -6.924  1.00136.11
C
ANISOU 1892  CG1 VAL A 261    18247  19701  13769   2726   1620
3806       C
ATOM   1893  CG2 VAL A 261      -6.128  21.093  -5.725  1.00160.99
C
ANISOU 1893  CG2 VAL A 261    20694  23348  17128   2874   1520
```

```
4309       C
ATOM   1894  N   MET A 262      -3.583  19.733  -9.388  1.00125.99
N
ANISOU 1894  N   MET A 262    17274  19041  11554   1108    791
3392       N
ATOM   1895  CA  MET A 262      -2.608  19.962 -10.457  1.00125.32
C
ANISOU 1895  CA  MET A 262    17561  18839  11217    784    723
3134       C
ATOM   1896  C   MET A 262      -3.140  19.697 -11.864  1.00129.09
C
ANISOU 1896  C   MET A 262    17911  19774  11361    234    332
3522       C
ATOM   1897  O   MET A 262      -2.528  20.172 -12.827  1.00129.56
O
ANISOU 1897  O   MET A 262    18207  19780  11242     31    276
3467       O
ATOM   1898  CB  MET A 262      -1.334  19.143 -10.220  1.00151.69
C
ANISOU 1898  CB  MET A 262    21359  21860  14418    555    834
2361       C
ATOM   1899  CG  MET A 262      -1.505  17.643 -10.153  1.00186.21
C
ANISOU 1899  CG  MET A 262    25736  26421  18593    116    658
2131       C
ATOM   1900  SD  MET A 262       0.057  16.809  -9.800  1.00208.73
S
ANISOU 1900  SD  MET A 262    29117  28844  21348    -51    852
1255       S
ATOM   1901  CE  MET A 262       0.136  16.964  -8.018  1.00177.03
C
ANISOU 1901  CE  MET A 262    25024  24511  17730    560   1189
1056       C
ATOM   1902  N   TYR A 263      -4.238  18.955 -12.023  1.00146.72
N
ANISOU 1902  N   TYR A 263    19794  22465  13489    -42     58
3908       N
ATOM   1903  CA  TYR A 263      -4.768  18.731 -13.370  1.00165.62
C
ANISOU 1903  CA  TYR A 263    22066  25322  15540   -606   -325
4305       C
ATOM   1904  C   TYR A 263      -5.139  20.016 -14.108  1.00152.92
C
ANISOU 1904  C   TYR A 263    20262  23847  13995   -421   -385
4890       C
ATOM   1905  O   TYR A 263      -4.769  20.140 -15.288  1.00186.33
O
ANISOU 1905  O   TYR A 263    24677  28189  17929   -840   -571
4908       O
ATOM   1906  CB  TYR A 263      -5.940  17.741 -13.337  1.00153.89
C
ANISOU 1906  CB  TYR A 263    20213  24326  13933   -933   -608
4655       C
ATOM   1907  CG  TYR A 263      -5.542  16.311 -13.053  1.00163.73
```

```
C
ANISOU 1907  CG  TYR A 263    21721  25512  14979  -1340   -642
4093       C
ATOM   1908  CD1 TYR A 263      -6.505  15.322 -12.907  1.00178.92
C
ANISOU 1908  CD1 TYR A 263    23370  27823  16788  -1648   -868
4321       C
ATOM   1909  CD2 TYR A 263      -4.205  15.945 -12.945  1.00142.22
C
ANISOU 1909  CD2 TYR A 263    19514  22345  12179  -1420   -443
3353       C
ATOM   1910  CE1 TYR A 263      -6.151  14.008 -12.673  1.00176.21
C
ANISOU 1910  CE1 TYR A 263    23286  27407  16260  -2029   -889
3818       C
ATOM   1911  CE2 TYR A 263      -3.840  14.635 -12.692  1.00145.11
C
ANISOU 1911  CE2 TYR A 263    20117  22638  12381  -1770   -453
2862       C
ATOM   1912  CZ  TYR A 263      -4.818  13.669 -12.562  1.00158.27
C
ANISOU 1912  CZ  TYR A 263    21529  24671  13935  -2074   -673
3092       C
ATOM   1913  OH  TYR A 263      -4.462  12.367 -12.294  1.00166.18
O
ANISOU 1913  OH  TYR A 263    22785  25574  14783  -2413   -664
2608       O
ATOM   1914  N   PRO A 264      -5.851  21.002 -13.516  1.00145.59
N
ANISOU 1914  N   PRO A 264    18974  22909  13435    180   -229
5388       N
ATOM   1915  CA  PRO A 264      -6.285  22.140 -14.339  1.00173.18
C
ANISOU 1915  CA  PRO A 264    22256  26576  16968    302   -320
6011       C
ATOM   1916  C   PRO A 264      -5.414  23.373 -14.148  1.00193.92
C
ANISOU 1916  C   PRO A 264    25169  28696  19815    782     19
5814       C
ATOM   1917  O   PRO A 264      -5.396  24.256 -15.011  1.00233.51
O
ANISOU 1917  O   PRO A 264    30173  33766  24785    779    -52
6158       O
ATOM   1918  CB  PRO A 264      -7.708  22.416 -13.837  1.00175.17
C
ANISOU 1918  CB  PRO A 264    21900  27160  17494    661   -353
6744       C
ATOM   1919  CG  PRO A 264      -7.645  22.016 -12.416  1.00161.52
C
ANISOU 1919  CG  PRO A 264    20193  25149  16030   1042    -64
6372       C
ATOM   1920  CD  PRO A 264      -6.775  20.798 -12.386  1.00148.61
C
ANISOU 1920  CD  PRO A 264    18970  23384  14110    562   -126
```

```
5635       C
ATOM   1921  N   ILE A 265      -4.701  23.454 -13.022  1.00192.17
N
ANISOU 1921  N   ILE A 265    25204  27988  19823   1179    384
5281       N
ATOM   1922  CA  ILE A 265      -3.758  24.552 -12.820  1.00188.31
C
ANISOU 1922  CA  ILE A 265    25052  26997  19501   1568    712
5016       C
ATOM   1923  C   ILE A 265      -2.671  24.518 -13.887  1.00189.99
C
ANISOU 1923  C   ILE A 265    25691  27117  19380   1110    595
4642       C
ATOM   1924  O   ILE A 265      -2.289  25.557 -14.440  1.00198.93
O
ANISOU 1924  O   ILE A 265    26958  28095  20531   1237    667
4773       O
ATOM   1925  CB  ILE A 265      -3.167  24.505 -11.399  1.00179.59
C
ANISOU 1925  CB  ILE A 265    24158  25427  18652   1989   1098
4481       C
ATOM   1926  CG1 ILE A 265      -4.275  24.687 -10.361  1.00194.50
C
ANISOU 1926  CG1 ILE A 265    25625  27392  20885   2480   1249
4891       C
ATOM   1927  CG2 ILE A 265      -2.098  25.572 -11.229  1.00169.36
C
ANISOU 1927  CG2 ILE A 265    23257  23617  17472   2304   1421
4158       C
ATOM   1928  CD1 ILE A 265      -4.985  26.023 -10.454  1.00201.49
C
ANISOU 1928  CD1 ILE A 265    26241  28270  22045   2969   1389
5543       C
ATOM   1929  N   GLN A 266      -2.165  23.327 -14.198  1.00189.34
N
ANISOU 1929  N   GLN A 266    25832  27121  18988    572    427
4179       N
ATOM   1930  CA  GLN A 266      -1.156  23.149 -15.232  1.00216.99
C
ANISOU 1930  CA  GLN A 266    29737  30560  22149     92    320
3806       C
ATOM   1931  C   GLN A 266      -1.763  22.619 -16.529  1.00224.84
C
ANISOU 1931  C   GLN A 266    30585  32073  22772   -538   -102
4171       C
ATOM   1932  O   GLN A 266      -1.042  22.438 -17.516  1.00222.90
O
ANISOU 1932  O   GLN A 266    30655  31834  22204   -999   -220
3918       O
ATOM   1933  CB  GLN A 266      -0.063  22.197 -14.718  1.00195.23
C
ANISOU 1933  CB  GLN A 266    27378  27501  19298    -78    467
3007       C
ATOM   1934  CG  GLN A 266       1.214  22.093 -15.554  1.00194.82
```

```
C
ANISOU 1934  CG  GLN A 266    27800  27260  18962   -457    473
2508       C
ATOM   1935  CD  GLN A 266       1.786  23.446 -15.944  1.00185.42
C
ANISOU 1935  CD  GLN A 266    26774  25831  17848   -207    617
2590       C
ATOM   1936  OE1 GLN A 266       2.623  24.001 -15.232  1.00173.99
O
ANISOU 1936  OE1 GLN A 266    25559  23954  16594    163    929
2233       O
ATOM   1937  NE2 GLN A 266       1.358  23.969 -17.089  1.00154.29
N
ANISOU 1937  NE2 GLN A 266    22720  22168  13734   -436    384
3059       N
ATOM   1938  N   ARG A 267      -3.080  22.412 -16.553  1.00216.86
N
ANISOU 1938  N   ARG A 267    29096  31506  21794   -572   -326
4781       N
ATOM   1939  CA  ARG A 267      -3.819  21.883 -17.702  1.00186.44
C
ANISOU 1939  CA  ARG A 267    25046  28210  17585  -1190   -750
5205       C
ATOM   1940  C   ARG A 267      -3.193  20.584 -18.220  1.00171.41
C
ANISOU 1940  C   ARG A 267    23507  26353  15270  -1874   -896
4645       C
ATOM   1941  O   ARG A 267      -2.713  20.485 -19.351  1.00169.01
O
ANISOU 1941  O   ARG A 267    23461  26141  14613  -2380  -1060
4538       O
ATOM   1942  CB  ARG A 267      -3.961  22.939 -18.804  1.00175.91
C
ANISOU 1942  CB  ARG A 267    23633  27043  16161  -1251   -897
5711       C
ATOM   1943  CG  ARG A 267      -4.899  22.514 -19.930  1.00196.79
C
ANISOU 1943  CG  ARG A 267    25989  30323  18461  -1856  -1352
6280       C
ATOM   1944  CD  ARG A 267      -5.406  23.698 -20.733  1.00213.56
C
ANISOU 1944  CD  ARG A 267    27848  32670  20626  -1739  -1486
6999       C
ATOM   1945  NE  ARG A 267      -6.235  24.579 -19.912  1.00198.39
N
ANISOU 1945  NE  ARG A 267    25484  30721  19174  -1021  -1311
7549       N
ATOM   1946  CZ  ARG A 267      -7.502  24.329 -19.592  1.00169.47
C
ANISOU 1946  CZ  ARG A 267    21287  27471  15633   -935  -1464
8137       C
ATOM   1947  NH1 ARG A 267      -8.103  23.237 -20.044  1.00165.02
N
ANISOU 1947  NH1 ARG A 267    20563  27397  14738  -1553  -1820
```

```
8267       N
ATOM   1948  NH2 ARG A 267      -8.179  25.184 -18.837  1.00171.52
N
ANISOU 1948  NH2 ARG A 267    21175  27655  16339   -243  -1250
8610       N
ATOM   1949  N   ARG A 268      -3.184  19.586 -17.341  1.00163.21
N
ANISOU 1949  N   ARG A 268    22502  25223  14288  -1871   -809
4274       N
ATOM   1950  CA  ARG A 268      -2.580  18.294 -17.621  1.00144.61
C
ANISOU 1950  CA  ARG A 268    20504  22837  11604  -2437   -876
3704       C
ATOM   1951  C   ARG A 268      -3.496  17.439 -18.495  1.00146.82
C
ANISOU 1951  C   ARG A 268    20603  23674  11507  -3115  -1277
4061       C
ATOM   1952  O   ARG A 268      -4.639  17.795 -18.796  1.00146.34
O
ANISOU 1952  O   ARG A 268    20095  24053  11454  -3135  -1519
4767       O
ATOM   1953  CB  ARG A 268      -2.270  17.547 -16.324  1.00152.64
C
ANISOU 1953  CB  ARG A 268    21608  23551  12835  -2169   -632
3215       C
ATOM   1954  CG  ARG A 268      -1.217  18.179 -15.432  1.00189.68
C
ANISOU 1954  CG  ARG A 268    26551  27685  17836  -1607   -236
2753       C
ATOM   1955  CD  ARG A 268       0.112  18.294 -16.159  1.00185.31
C
ANISOU 1955  CD  ARG A 268    26489  26849  17073  -1848   -142
2253       C
ATOM   1956  NE  ARG A 268       1.195  18.663 -15.253  1.00181.83
N
ANISOU 1956  NE  ARG A 268    26311  25892  16885  -1397    224
1742       N
ATOM   1957  CZ  ARG A 268       2.451  18.873 -15.634  1.00173.66
C
ANISOU 1957  CZ  ARG A 268    25686  24550  15746  -1482    374
1279       C
ATOM   1958  NH1 ARG A 268       3.380  19.151 -14.730  1.00158.13
N
ANISOU 1958  NH1 ARG A 268    23924  22152  14007  -1089    693
844      N
ATOM   1959  NH2 ARG A 268       2.787  18.762 -16.912  1.00141.36
N
ANISOU 1959  NH2 ARG A 268    21805  20598  11308  -1986    205
1246       N
ATOM   1960  N   LYS A 269      -2.960  16.295 -18.913  1.00156.90
N
ANISOU 1960  N   LYS A 269    22246  24924  12443  -3688  -1337
3564       N
ATOM   1961  CA  LYS A 269      -3.780  15.202 -19.419  1.00159.17
```

C
ANISOU 1961 CA LYS A 269   22424 25666 12388 −4333 −1656
3757   C
ATOM   1962 C   LYS A 269   −4.819 14.780 −18.386 1.00172.42
C
ANISOU 1962 C   LYS A 269   23677 27533 14303 −4080 −1691
4046   C
ATOM   1963 O   LYS A 269   −4.567 14.801 −17.178 1.00185.17
O
ANISOU 1963 O   LYS A 269   25273 28811 16274 −3540 −1411
3780   O
ATOM   1964 CB LYS A 269   −2.889 14.016 −19.790 1.00170.69
C
ANISOU 1964 CB LYS A 269   24414 26929 13513 −4882 −1602
3062   C
ATOM   1965 CG LYS A 269   −2.172 13.392 −18.599 1.00177.83
C
ANISOU 1965 CG LYS A 269   25532 27369 14667 −4539 −1274
2438   C
ATOM   1966 CD LYS A 269   −0.917 12.646 −19.018 1.00192.30
C
ANISOU 1966 CD LYS A 269   27947 28861 16258 −4900 −1109
1714   C
ATOM   1967 CE LYS A 269   −1.238 11.480 −19.936 1.00185.24
C
ANISOU 1967 CE LYS A 269   27242 28260 14882 −5725 −1348
1661   C
ATOM   1968 NZ LYS A 269   −0.036 10.640 −20.193 1.00165.92
N
ANISOU 1968 NZ LYS A 269   25363 25436 12244 −6023 −1127
928   N
ATOM   1969 N   TYR A 270   −6.005 14.410 −18.870 1.00182.55
N
ANISOU 1969 N   TYR A 270   24607 29379 15376 −4488 −2042
4614   N
ATOM   1970 CA TYR A 270   −7.117 14.055 −17.994 1.00206.34
C
ANISOU 1970 CA TYR A 270   27158 32651 18591 −4286 −2113
4986   C
ATOM   1971 C   TYR A 270   −7.275 12.543 −17.887 1.00211.10
C
ANISOU 1971 C   TYR A 270   27925 33364 18920 −4836 −2223
4664   C
ATOM   1972 O   TYR A 270   −6.936 11.947 −16.858 1.00210.61
O
ANISOU 1972 O   TYR A 270   27987 32983 19053 −4585 −1994
4217   O
ATOM   1973 CB TYR A 270   −8.430 14.678 −18.473 1.00198.60
C
ANISOU 1973 CB TYR A 270   25599 32252 17606 −4316 −2422
5905   C
ATOM   1974 CG TYR A 270   −9.585 14.370 −17.543 1.00179.45
C
ANISOU 1974 CG TYR A 270   22667 30103 15414 −4068 −2474

```
6316       C
ATOM   1975  CD1 TYR A 270      -9.630  14.910 -16.264  1.00169.52
C
ANISOU 1975  CD1 TYR A 270    21214  28540  14656  -3276  -2156
6299       C
ATOM   1976  CD2 TYR A 270     -10.612  13.520 -17.932  1.00172.90
C
ANISOU 1976  CD2 TYR A 270    21571  29836  14288  -4649  -2832
6706       C
ATOM   1977  CE1 TYR A 270     -10.674  14.627 -15.405  1.00177.11
C
ANISOU 1977  CE1 TYR A 270    21716  29749  15829  -3046  -2184
6665       C
ATOM   1978  CE2 TYR A 270     -11.661  13.229 -17.077  1.00168.60
C
ANISOU 1978  CE2 TYR A 270    20553  29555  13953  -4429  -2877
7085       C
ATOM   1979  CZ  TYR A 270     -11.686  13.786 -15.816  1.00173.28
C
ANISOU 1979  CZ  TYR A 270    20950  29833  15056  -3615  -2547
7061       C
ATOM   1980  OH  TYR A 270     -12.725  13.500 -14.960  1.00161.06
O
ANISOU 1980  OH  TYR A 270    18933  28546  13716  -3393  -2575
7434       O
ATOM   1981  N   ARG A 271      -7.809  11.928 -18.947  1.00221.23
N
ANISOU 1981  N   ARG A 271    29210  35104  19742  -5603  -2576
4909       N
ATOM   1982  CA  ARG A 271      -8.193  10.519 -18.905  1.00231.37
C
ANISOU 1982  CA  ARG A 271    30595  36575  20740  -6179  -2722
4731       C
ATOM   1983  C   ARG A 271      -7.031   9.626 -18.486  1.00232.71
C
ANISOU 1983  C   ARG A 271    31335  36201  20883  -6235  -2427
3854       C
ATOM   1984  O   ARG A 271      -7.217   8.685 -17.704  1.00242.76
O
ANISOU 1984  O   ARG A 271    32624  37403  22213  -6254  -2363
3623       O
ATOM   1985  CB  ARG A 271      -8.759  10.088 -20.260  1.00246.43
C
ANISOU 1985  CB  ARG A 271    32521  39013  22100  -7059  -3125
5070       C
ATOM   1986  CG  ARG A 271      -7.993  10.598 -21.470  1.00247.21
C
ANISOU 1986  CG  ARG A 271    32970  39041  21917  -7374  -3159
4943       C
ATOM   1987  CD  ARG A 271      -8.347   9.804 -22.721  1.00247.80
C
ANISOU 1987  CD  ARG A 271    33232  39546  21375  -8371  -3502
5052       C
ATOM   1988  NE  ARG A 271      -7.639   8.532 -22.808  1.00247.53
```

```
N
ANISOU 1988  NE  ARG A 271    33777  39219  21053  -8863  -3367
4322       N
ATOM   1989  CZ  ARG A 271      -7.506   7.817 -23.919  1.00235.04
C
ANISOU 1989  CZ  ARG A 271    32478  37626  19201  -9585  -3453
4074       C
ATOM   1990  NH1 ARG A 271      -8.289   8.054 -24.960  1.00171.59
N
ANISOU 1990  NH1 ARG A 271    24147  30004  11046 -10029  -3761
4578       N
ATOM   1991  NH2 ARG A 271      -6.801   6.695 -23.889  1.00228.26
N
ANISOU 1991  NH2 ARG A 271    32106  36348  18274  -9868  -3226
3367       N
ATOM   1992  N   ASP A 272      -5.827   9.895 -18.987  1.00224.87
N
ANISOU 1992  N   ASP A 272    30805  34826  19810  -6257  -2240
3372       N
ATOM   1993  CA  ASP A 272      -4.691   9.021 -18.737  1.00212.12
C
ANISOU 1993  CA  ASP A 272    29737  32723  18137  -6364  -1966
2569       C
ATOM   1994  C   ASP A 272      -3.846   9.497 -17.562  1.00168.19
C
ANISOU 1994  C   ASP A 272    24232  26624  13050  -5580  -1578
2179       C
ATOM   1995  O   ASP A 272      -2.790   8.917 -17.290  1.00148.83
O
ANISOU 1995  O   ASP A 272    22206  23735  10607  -5572  -1319
1527       O
ATOM   1996  CB  ASP A 272      -3.826   8.910 -19.994  1.00256.24
C
ANISOU 1996  CB  ASP A 272    35821  38212  23327  -6904  -1973
2239       C
ATOM   1997  CG  ASP A 272      -4.536   8.197 -21.127  1.00271.04
C
ANISOU 1997  CG  ASP A 272    37750  40567  24664  -7788  -2325
2495       C
ATOM   1998  OD1 ASP A 272      -5.679   7.739 -20.917  1.00278.48
O
ANISOU 1998  OD1 ASP A 272    38346  41921  25542  -7994  -2570
2918       O
ATOM   1999  OD2 ASP A 272      -3.954   8.098 -22.228  1.00275.12
O
ANISOU 1999  OD2 ASP A 272    38660  41061  24812  -8296  -2355
2279       O
ATOM   2000  N   GLY A 273      -4.291  10.546 -16.864  1.00153.90
N
ANISOU 2000  N   GLY A 273    22004  24841  11630  -4931  -1525
2578       N
ATOM   2001  CA  GLY A 273      -3.655  10.992 -15.636  1.00167.92
C
ANISOU 2001  CA  GLY A 273    23794  26155  13853  -4203  -1171
```

```
2266       C
ATOM   2002  C   GLY A 273      -4.111  10.247 -14.399  1.00156.85
C
ANISOU 2002  C   GLY A 273    22213  24713  12671  -3977  -1088
2186       C
ATOM   2003  O   GLY A 273      -3.419  10.275 -13.376  1.00127.82
O
ANISOU 2003  O   GLY A 273    18658  20617   9291  -3508   -785
1781       O
ATOM   2004  N   ILE A 274      -5.272   9.590 -14.478  1.00172.15
N
ANISOU 2004  N   ILE A 274    23856  27098  14457  -4322  -1361
2581       N
ATOM   2005  CA  ILE A 274      -5.913   8.930 -13.344  1.00134.34
C
ANISOU 2005  CA  ILE A 274    18821  22356   9866  -4130  -1327
2618       C
ATOM   2006  C   ILE A 274      -5.036   7.847 -12.721  1.00143.84
C
ANISOU 2006  C   ILE A 274    20436  23144  11074  -4194  -1101
1911       C
ATOM   2007  O   ILE A 274      -5.206   7.514 -11.543  1.00159.85
O
ANISOU 2007  O   ILE A 274    22325  25042  13370  -3845   -961
1812       O
ATOM   2008  CB  ILE A 274      -7.268   8.353 -13.814  1.00139.20
C
ANISOU 2008  CB  ILE A 274    19094  23577  10219  -4645  -1706
3172       C
ATOM   2009  CG1 ILE A 274      -8.156   9.469 -14.369  1.00150.10
C
ANISOU 2009  CG1 ILE A 274    20012  25384  11636  -4526  -1922
3933       C
ATOM   2010  CG2 ILE A 274      -7.997   7.644 -12.684  1.00138.65
C
ANISOU 2010  CG2 ILE A 274    18755  23595  10331  -4486  -1692
3242       C
ATOM   2011  CD1 ILE A 274      -8.534  10.523 -13.360  1.00142.39
C
ANISOU 2011  CD1 ILE A 274    18623  24325  11155  -3709  -1734
4260       C
ATOM   2012  N   ASP A 275      -4.081   7.304 -13.481  1.00130.20
N
ANISOU 2012  N   ASP A 275    19210  21197   9065  -4619  -1047
1425       N
ATOM   2013  CA  ASP A 275      -3.111   6.364 -12.923  1.00141.05
C
ANISOU 2013  CA  ASP A 275    20989  22127  10475  -4624   -790
751       C
ATOM   2014  C   ASP A 275      -2.392   6.930 -11.702  1.00128.54
C
ANISOU 2014  C   ASP A 275    19386  20117   9338  -3885   -458
480       C
ATOM   2015  O   ASP A 275      -2.095   6.195 -10.753  1.00147.65
```

```
O
ANISOU 2015  O   ASP A 275    21893  22293  11913  -3729   -287
141       O
ATOM   2016  CB  ASP A 275      -2.094   5.971 -13.997  1.00153.87
C
ANISOU 2016  CB  ASP A 275    23145  23548  11769  -5099   -731
304       C
ATOM   2017  CG  ASP A 275      -2.722   5.205 -15.144  1.00182.74
C
ANISOU 2017  CG  ASP A 275    26910  27582  14940  -5910  -1027
475       C
ATOM   2018  OD1 ASP A 275      -3.924   4.877 -15.056  1.00190.79
O
ANISOU 2018  OD1 ASP A 275    27594  29021  15877  -6122  -1285
919       O
ATOM   2019  OD2 ASP A 275      -2.013   4.932 -16.136  1.00190.39
O
ANISOU 2019  OD2 ASP A 275    28304  28436  15599  -6351   -996
168       O
ATOM   2020  N   ASN A 276      -2.095   8.231 -11.710  1.00120.68
N
ANISOU 2020  N   ASN A 276    18289  19024   8539  -3445   -361
627       N
ATOM   2021  CA  ASN A 276      -1.537   8.877 -10.523  1.00116.92
C
ANISOU 2021  CA  ASN A 276    17765  18185   8475  -2755    -60
436       C
ATOM   2022  C   ASN A 276      -2.478   8.800  -9.321  1.00116.83
C
ANISOU 2022  C   ASN A 276    17346  18301   8741  -2390    -52
716       C
ATOM   2023  O   ASN A 276      -2.025   8.619  -8.185  1.00137.68
O
ANISOU 2023  O   ASN A 276    20035  20638  11639  -2018    185
406       O
ATOM   2024  CB  ASN A 276      -1.190  10.332 -10.839  1.00119.91
C
ANISOU 2024  CB  ASN A 276    18105  18475   8982  -2399     22
609       C
ATOM   2025  CG  ASN A 276      -0.135  10.455 -11.920  1.00116.56
C
ANISOU 2025  CG  ASN A 276    18099  17879   8309  -2707     55
284       C
ATOM   2026  OD1 ASN A 276       1.051  10.228 -11.678  1.00113.29
O
ANISOU 2026  OD1 ASN A 276    18034  17071   7939  -2626    295
-259       O
ATOM   2027  ND2 ASN A 276      -0.563  10.816 -13.125  1.00131.38
N
ANISOU 2027  ND2 ASN A 276    19930  20068   9921  -3071   -187
633       N
ATOM   2028  N   LEU A 277      -3.781   8.987  -9.532  1.00120.56
N
ANISOU 2028  N   LEU A 277    17401  19229   9176  -2476   -303
```

```
1322       N
ATOM   2029  CA  LEU A 277      -4.742   8.717  -8.463  1.00120.88
C
ANISOU 2029  CA  LEU A 277    17059  19435   9436  -2220   -314
1586       C
ATOM   2030  C   LEU A 277      -4.750   7.249  -8.043  1.00134.76
C
ANISOU 2030  C   LEU A 277    18966  21163  11074  -2557   -339
1260       C
ATOM   2031  O   LEU A 277      -4.763   6.939  -6.846  1.00177.43
O
ANISOU 2031  O   LEU A 277    24289  26402  16726  -2226   -174
1106       O
ATOM   2032  CB  LEU A 277      -6.149   9.143  -8.888  1.00125.60
C
ANISOU 2032  CB  LEU A 277    17162  20571   9988  -2301   -595
2335       C
ATOM   2033  CG  LEU A 277      -6.386  10.578  -9.356  1.00137.16
C
ANISOU 2033  CG  LEU A 277    18402  22139  11573  -1984   -605
2793       C
ATOM   2034  CD1 LEU A 277      -7.812  10.730  -9.847  1.00169.96
C
ANISOU 2034  CD1 LEU A 277    22060  26881  15638  -2171   -921
3547       C
ATOM   2035  CD2 LEU A 277      -6.123  11.544  -8.217  1.00125.10
C
ANISOU 2035  CD2 LEU A 277    16773  20276  10485  -1219   -270
2749       C
ATOM   2036  N   LEU A 278      -4.748   6.334  -9.014  1.00121.72
N
ANISOU 2036  N   LEU A 278    17549  19663   9035  -3227   -536
1153       N
ATOM   2037  CA  LEU A 278      -4.804   4.904  -8.715  1.00121.28
C
ANISOU 2037  CA  LEU A 278    17664  19579   8837  -3598   -562
864       C
ATOM   2038  C   LEU A 278      -3.603   4.392  -7.919  1.00116.73
C
ANISOU 2038  C   LEU A 278    17450  18472   8430  -3364   -241
213       C
ATOM   2039  O   LEU A 278      -3.772   3.583  -7.000  1.00115.41
O
ANISOU 2039  O   LEU A 278    17246  18227   8375  -3300   -175
68      O
ATOM   2040  CB  LEU A 278      -4.951   4.113 -10.015  1.00124.49
C
ANISOU 2040  CB  LEU A 278    18317  20215   8768  -4388   -804
854       C
ATOM   2041  CG  LEU A 278      -5.253   2.622  -9.850  1.00148.37
C
ANISOU 2041  CG  LEU A 278    21496  23286  11591  -4865   -876
658       C
ATOM   2042  CD1 LEU A 278      -6.499   2.430  -9.001  1.00157.62
```

```
C
ANISOU 2042  CD1 LEU A 278    22186  24794  12911  -4727  -1018
1094       C
ATOM   2043  CD2 LEU A 278      -5.418   1.953 -11.206  1.00166.01
C
ANISOU 2043  CD2 LEU A 278    24000  25751  13325  -5675  -1105
669       C
ATOM   2044  N   VAL A 279      -2.384   4.829  -8.255  1.00114.45
N
ANISOU 2044  N   VAL A 279    17502  17827   8157  -3246    -44
-169       N
ATOM   2045  CA  VAL A 279      -1.218   4.443  -7.453  1.00110.27
C
ANISOU 2045  CA  VAL A 279    17267  16812   7817  -2977    266
-738       C
ATOM   2046  C   VAL A 279      -1.350   4.889  -5.998  1.00107.74
C
ANISOU 2046  C   VAL A 279    16673  16367   7898  -2348    437
-687       C
ATOM   2047  O   VAL A 279      -0.991   4.144  -5.078  1.00138.37
O
ANISOU 2047  O   VAL A 279    20642  20019  11912  -2229    589
-998       O
ATOM   2048  CB  VAL A 279       0.089   4.961  -8.088  1.00116.77
C
ANISOU 2048  CB  VAL A 279    18460  17315   8594  -2942    443
-1102       C
ATOM   2049  CG1 VAL A 279       0.354   4.259  -9.410  1.00169.75
C
ANISOU 2049  CG1 VAL A 279    25520  24079  14896  -3599    332
-1268       C
ATOM   2050  CG2 VAL A 279       0.069   6.462  -8.270  1.00123.72
C
ANISOU 2050  CG2 VAL A 279    19152  18245   9610  -2571    452
-811       C
ATOM   2051  N   LEU A 280      -1.854   6.102  -5.761  1.00108.40
N
ANISOU 2051  N   LEU A 280    16431  16583   8174  -1940    430
-295       N
ATOM   2052  CA  LEU A 280      -2.115   6.538  -4.390  1.00107.46
C
ANISOU 2052  CA  LEU A 280    16045  16371   8414  -1371    597
-210       C
ATOM   2053  C   LEU A 280      -3.120   5.645  -3.665  1.00136.04
C
ANISOU 2053  C   LEU A 280    19403  20221  12067  -1456    486
-27       C
ATOM   2054  O   LEU A 280      -2.912   5.294  -2.497  1.00166.39
O
ANISOU 2054  O   LEU A 280    23233  23863  16122  -1177    659
-242       O
ATOM   2055  CB  LEU A 280      -2.587   7.992  -4.372  1.00107.87
C
ANISOU 2055  CB  LEU A 280    15804  16536   8647   -952    618
```

```
221        C
ATOM   2056  CG  LEU A 280      -1.472   8.999  -4.077  1.00105.02
C
ANISOU 2056  CG  LEU A 280    15653  15777   8474   -526    899
-61        C
ATOM   2057  CD1 LEU A 280      -0.423   9.011  -5.184  1.00104.50
C
ANISOU 2057  CD1 LEU A 280    15981  15548   8175   -838    897
-375        C
ATOM   2058  CD2 LEU A 280      -2.023  10.387  -3.807  1.00106.32
C
ANISOU 2058  CD2 LEU A 280    15524  16008   8867    -49    973
363        C
ATOM   2059  N   LEU A 281      -4.216   5.272  -4.328  1.00111.32
N
ANISOU 2059  N   LEU A 281    16054  17524   8719  -1854    193
381        N
ATOM   2060  CA  LEU A 281      -5.134   4.293  -3.746  1.00112.47
C
ANISOU 2060  CA  LEU A 281    15987  17901   8845  -2027     68
530        C
ATOM   2061  C   LEU A 281      -4.485   2.926  -3.558  1.00110.62
C
ANISOU 2061  C   LEU A 281    16121  17421   8487  -2351    132
21        C
ATOM   2062  O   LEU A 281      -4.488   2.376  -2.450  1.00130.96
O
ANISOU 2062  O   LEU A 281    18652  19863  11245  -2151    252
-138        O
ATOM   2063  CB  LEU A 281      -6.390   4.169  -4.607  1.00117.21
C
ANISOU 2063  CB  LEU A 281    16294  19042   9197  -2454   -278
1081        C
ATOM   2064  CG  LEU A 281      -7.320   5.380  -4.588  1.00119.69
C
ANISOU 2064  CG  LEU A 281    16127  19667   9682  -2098   -352
1700        C
ATOM   2065  CD1 LEU A 281      -8.223   5.394  -5.807  1.00130.60
C
ANISOU 2065  CD1 LEU A 281    17313  21552  10756  -2592   -702
2203        C
ATOM   2066  CD2 LEU A 281      -8.142   5.381  -3.306  1.00130.75
C
ANISOU 2066  CD2 LEU A 281    17134  21172  11373  -1695   -275
1940        C
ATOM   2067  N   ILE A 282      -3.930   2.356  -4.629  1.00111.38
N
ANISOU 2067  N   ILE A 282    16592  17452   8276  -2853     65
-233        N
ATOM   2068  CA  ILE A 282      -3.547   0.946  -4.601  1.00130.56
C
ANISOU 2068  CA  ILE A 282    19354  19708  10543  -3247     98
-631        C
ATOM   2069  C   ILE A 282      -2.418   0.693  -3.602  1.00140.04
```

C
ANISOU 2069 C   ILE A 282   20780 20423 12005 -2867   417
-1132   C
ATOM   2070 O   ILE A 282   -2.409 -0.334 -2.911 1.00144.60
O
ANISOU 2070 O   ILE A 282   21433 20885 12625 -2936   476
-1332   O
ATOM   2071 CB ILE A 282   -3.193   0.461 -6.023 1.00152.59
C
ANISOU 2071 CB ILE A 282   22521 22519 12936 -3872   -10
-789   C
ATOM   2072 CG1 ILE A 282   -3.205 -1.068 -6.090 1.00161.88
C
ANISOU 2072 CG1 ILE A 282   23985 23625 13896 -4375   -28
-1063   C
ATOM   2073 CG2 ILE A 282   -1.861   1.019 -6.506 1.00144.56
C
ANISOU 2073 CG2 ILE A 282   21848 21138 11940 -3729   203
-1165   C
ATOM   2074 CD1 ILE A 282   -4.492 -1.686 -5.601 1.00200.35
C
ANISOU 2074 CD1 ILE A 282   28538 28855 18731 -4553   -239
-711   C
ATOM   2075 N   GLY A 283   -1.457   1.608 -3.504 1.00164.44
N
ANISOU 2075 N   GLY A 283   23979 23234 15267 -2476   621
-1326   N
ATOM   2076 CA GLY A 283   -0.381   1.469 -2.540 1.00136.42
C
ANISOU 2076 CA GLY A 283   20606 19261 11965 -2109   911
-1755   C
ATOM   2077 C   GLY A 283   -0.617   2.090 -1.179 1.00147.29
C
ANISOU 2077 C   GLY A 283   21675 20601 13686 -1537   1034
-1629   C
ATOM   2078 O   GLY A 283   -0.058   1.633 -0.177 1.00166.42
O
ANISOU 2078 O   GLY A 283   24176 22765 16291 -1321   1216
-1916   O
ATOM   2079 N   GLY A 284   -1.447   3.132 -1.129 1.00144.45
N
ANISOU 2079 N   GLY A 284   20970 20497 13419 -1293   948
-1191   N
ATOM   2080 CA GLY A 284   -1.657   3.842   0.123 1.00134.94
C
ANISOU 2080 CA GLY A 284   19501 19235 12535   -738   1103
-1074   C
ATOM   2081 C   GLY A 284   -2.502   3.085   1.131 1.00113.48
C
ANISOU 2081 C   GLY A 284   16537 16662   9917   -703   1060
-944   C
ATOM   2082 O   GLY A 284   -2.213   3.105   2.331 1.00107.01
O
ANISOU 2082 O   GLY A 284   15679 15648   9332   -347   1250

```
-1101       O
ATOM   2083  N   ILE A 285      -3.550   2.409   0.670  1.00117.86
N
ANISOU 2083  N   ILE A 285    16927  17573  10283  -1092    807
-651       N
ATOM   2084  CA  ILE A 285      -4.473   1.728   1.576  1.00123.28
C
ANISOU 2084  CA  ILE A 285    17345  18448  11050  -1081    742
-472       C
ATOM   2085  C   ILE A 285      -3.865   0.456   2.156  1.00120.90
C
ANISOU 2085  C   ILE A 285    17310  17887  10739  -1241    833
-902       C
ATOM   2086  O   ILE A 285      -3.576  -0.489   1.409  1.00131.92
O
ANISOU 2086  O   ILE A 285    18993  19238  11892  -1714    747
-1108       O
ATOM   2087  CB  ILE A 285      -5.783   1.381   0.853  1.00131.48
C
ANISOU 2087  CB  ILE A 285    18120  19973  11862  -1495    427
-6       C
ATOM   2088  CG1 ILE A 285      -6.430   2.637   0.276  1.00137.28
C
ANISOU 2088  CG1 ILE A 285    18551  20987  12621  -1323    331
479       C
ATOM   2089  CG2 ILE A 285      -6.739   0.671   1.800  1.00126.37
C
ANISOU 2089  CG2 ILE A 285    17187  19533  11294  -1492    361
185       C
ATOM   2090  CD1 ILE A 285      -7.481   2.337  -0.763  1.00146.65
C
ANISOU 2090  CD1 ILE A 285    19549  22653  13519  -1825     -5
914       C
ATOM   2091  N   PRO A 286      -3.629   0.375   3.630  1.00142.08
N
ANISOU 2091  N   PRO A 286    19911  20367  13705   -827   1038
-1059       N
ATOM   2092  CA  PRO A 286      -3.043  -0.829   4.248  1.00116.72
C
ANISOU 2092  CA  PRO A 286    16932  16908  10510   -950   1129
-1437       C
ATOM   2093  C   PRO A 286      -4.121  -1.808   4.720  1.00103.61
C
ANISOU 2093  C   PRO A 286    15065  15504   8799  -1175    972
-1232       C
ATOM   2094  O   PRO A 286      -4.497  -1.875   5.892  1.00104.52
O
ANISOU 2094  O   PRO A 286    14962  15641   9108   -903   1044
-1159       O
ATOM   2095  CB  PRO A 286      -2.234  -0.242   5.405  1.00 91.82
C
ANISOU 2095  CB  PRO A 286    13791  13451   7646   -430   1400
-1659       C
ATOM   2096  CG  PRO A 286      -3.084   0.913   5.865  1.00 92.78
```

```
C
ANISOU 2096  CG  PRO A 286    13536  13790   7926    -54   1410
-1266       C
ATOM   2097  CD  PRO A 286      -3.800   1.453   4.622  1.00103.51
C
ANISOU 2097  CD  PRO A 286    14766  15456   9105   -265   1203
-903       C
ATOM   2098  N   ILE A 287      -4.646  -2.602   3.783  1.00 99.64
N
ANISOU 2098  N   ILE A 287    14640  15209   8010  -1716    752
-1134       N
ATOM   2099  CA  ILE A 287      -5.599  -3.644   4.153  1.00101.42
C
ANISOU 2099  CA  ILE A 287    14722  15665   8150  -2004    597
-971       C
ATOM   2100  C   ILE A 287      -4.949  -4.756   4.965  1.00128.60
C
ANISOU 2100  C   ILE A 287    18409  18782  11672  -2025    746
-1362       C
ATOM   2101  O   ILE A 287      -5.651  -5.530   5.626  1.00156.22
O
ANISOU 2101  O   ILE A 287    21762  22415  15179  -2134    672
-1253       O
ATOM   2102  CB  ILE A 287      -6.271  -4.223   2.895  1.00105.17
C
ANISOU 2102  CB  ILE A 287    15262  16437   8260  -2635    325
-783       C
ATOM   2103  CG1 ILE A 287      -5.212  -4.617   1.863  1.00104.82
C
ANISOU 2103  CG1 ILE A 287    15713  16101   8011  -2960    389
-1178       C
ATOM   2104  CG2 ILE A 287      -7.255  -3.219   2.309  1.00108.01
C
ANISOU 2104  CG2 ILE A 287    15244  17233   8562  -2612    129
-258       C
ATOM   2105  CD1 ILE A 287      -5.770  -5.349   0.666  1.00149.13
C
ANISOU 2105  CD1 ILE A 287    21478  21955  13230  -3648    151
-1068       C
ATOM   2106  N   ALA A 288      -3.619  -4.854   4.931  1.00121.17
N
ANISOU 2106  N   ALA A 288    17824  17422  10793  -1921    956
-1797       N
ATOM   2107  CA  ALA A 288      -2.892  -5.665   5.903  1.00131.39
C
ANISOU 2107  CA  ALA A 288    19289  18386  12247  -1791   1141
-2133       C
ATOM   2108  C   ALA A 288      -3.104  -5.174   7.331  1.00135.25
C
ANISOU 2108  C   ALA A 288    19471  18890  13026  -1300   1245
-2028       C
ATOM   2109  O   ALA A 288      -3.197  -5.981   8.263  1.00129.09
O
ANISOU 2109  O   ALA A 288    18666  18043  12340  -1281   1286
```

```
-2103       O
ATOM   2110  CB  ALA A 288      -1.403  -5.680   5.558  1.00109.14
C
ANISOU 2110  CB  ALA A 288    16859  15148   9460  -1724   1355
-2565       C
ATOM   2111  N   MET A 289      -3.177  -3.855   7.521  1.00124.27
N
ANISOU 2111  N   MET A 289    17865  17578  11775   -910   1303
-1857       N
ATOM   2112  CA  MET A 289      -3.106  -3.288   8.867  1.00111.47
C
ANISOU 2112  CA  MET A 289    16042  15882  10428   -423   1470
-1844       C
ATOM   2113  C   MET A 289      -4.167  -3.810   9.833  1.00100.71
C
ANISOU 2113  C   MET A 289    14382  14748   9133   -408   1396
-1609       C
ATOM   2114  O   MET A 289      -3.806  -4.106  10.986  1.00 95.55
O
ANISOU 2114  O   MET A 289    13730  13924   8651   -191   1539
-1772       O
ATOM   2115  CB  MET A 289      -3.121  -1.752   8.754  1.00107.70
C
ANISOU 2115  CB  MET A 289    15408  15458  10056    -54   1547
-1670       C
ATOM   2116  CG  MET A 289      -3.225  -0.952  10.056  1.00 89.08
C
ANISOU 2116  CG  MET A 289    12830  13062   7956    443   1730
-1597       C
ATOM   2117  SD  MET A 289      -4.885  -0.822  10.760  1.00 94.87
S
ANISOU 2117  SD  MET A 289    13078  14205   8765    558   1632
-1106       S
ATOM   2118  CE  MET A 289      -5.713   0.181   9.528  1.00 93.62
C
ANISOU 2118  CE  MET A 289    12709  14362   8500    518   1478
-673       C
ATOM   2119  N   PRO A 290      -5.448  -3.950   9.471  1.00 93.51
N
ANISOU 2119  N   PRO A 290    13205  14228   8098   -630   1180
-1225       N
ATOM   2120  CA  PRO A 290      -6.395  -4.470  10.471  1.00 94.28
C
ANISOU 2120  CA  PRO A 290    13020  14532   8271   -602   1128
-1019       C
ATOM   2121  C   PRO A 290      -6.185  -5.941  10.784  1.00 93.88
C
ANISOU 2121  C   PRO A 290    13179  14345   8145   -921   1096
-1255       C
ATOM   2122  O   PRO A 290      -6.339  -6.354  11.940  1.00153.95
O
ANISOU 2122  O   PRO A 290    20678  21920  15894   -769   1168
-1279       O
ATOM   2123  CB  PRO A 290      -7.759  -4.227   9.812  1.00 97.99
```

```
C
ANISOU 2123  CB  PRO A 290    13157  15475   8600   -803    890
-528       C
ATOM   2124  CG  PRO A 290      -7.542  -3.047   8.957  1.00116.91
C
ANISOU 2124  CG  PRO A 290    15545  17890  10984   -656    905
-421       C
ATOM   2125  CD  PRO A 290      -6.161  -3.262   8.381  1.00114.66
C
ANISOU 2125  CD  PRO A 290    15722  17213  10629   -766   1007
-881       C
ATOM   2126  N   THR A 291      -5.833  -6.744   9.778  1.00 94.83
N
ANISOU 2126  N   THR A 291    13615  14373   8042  -1365   1004
-1431       N
ATOM   2127  CA  THR A 291      -5.571  -8.166   9.988  1.00106.56
C
ANISOU 2127  CA  THR A 291    15354  15678   9454  -1678   1003
-1672       C
ATOM   2128  C   THR A 291      -4.382  -8.411  10.914  1.00110.09
C
ANISOU 2128  C   THR A 291    16001  15709  10119  -1379   1251
-2044       C
ATOM   2129  O   THR A 291      -4.470  -9.208  11.856  1.00177.75
O
ANISOU 2129  O   THR A 291    24547  24212  18777  -1368   1286
-2100       O
ATOM   2130  CB  THR A 291      -5.342  -8.856   8.644  1.00127.43
C
ANISOU 2130  CB  THR A 291    18344  18263  11809  -2199    903
-1808       C
ATOM   2131  OG1 THR A 291      -6.516  -8.727   7.833  1.00153.18
O
ANISOU 2131  OG1 THR A 291    21403  21956  14841  -2537    643
-1429       O
ATOM   2132  CG2 THR A 291      -5.044 -10.329   8.858  1.00132.52
C
ANISOU 2132  CG2 THR A 291    19288  18676  12388  -2510    942
-2066       C
ATOM   2133  N   VAL A 292      -3.259  -7.738  10.656  1.00 97.15
N
ANISOU 2133  N   VAL A 292    14551  13801   8563  -1146   1417
-2284       N
ATOM   2134  CA  VAL A 292      -2.039  -7.954  11.438  1.00127.39
C
ANISOU 2134  CA  VAL A 292    18570  17250  12583   -890   1644
-2623       C
ATOM   2135  C   VAL A 292      -2.248  -7.612  12.910  1.00127.08
C
ANISOU 2135  C   VAL A 292    18260  17253  12773   -505   1732
-2533       C
ATOM   2136  O   VAL A 292      -1.858  -8.374  13.803  1.00109.73
O
ANISOU 2136  O   VAL A 292    16125  14883  10685   -458   1818
```

```
-2686       O
ATOM   2137  CB  VAL A 292      -0.873  -7.149  10.835  1.00120.76
C
ANISOU 2137  CB  VAL A 292    17939  16171  11773   -719   1790
-2847       C
ATOM   2138  CG1 VAL A 292       0.370  -7.279  11.700  1.00 99.84
C
ANISOU 2138  CG1 VAL A 292    15432  13174   9327   -438   2016
-3149       C
ATOM   2139  CG2 VAL A 292      -0.584  -7.614   9.414  1.00 86.48
C
ANISOU 2139  CG2 VAL A 292    13907  11754   7196  -1125   1730
-2976       C
ATOM   2140  N   LEU A 293      -2.860  -6.462  13.188  1.00110.23
N
ANISOU 2140  N   LEU A 293    15830  15340  10713   -225   1724
-2279       N
ATOM   2141  CA  LEU A 293      -3.170  -6.109  14.571  1.00111.16
C
ANISOU 2141  CA  LEU A 293    15695  15515  11024    117   1821
-2180       C
ATOM   2142  C   LEU A 293      -4.163  -7.080  15.205  1.00131.59
C
ANISOU 2142  C   LEU A 293    18103  18307  13589    -67   1699
-2005       C
ATOM   2143  O   LEU A 293      -4.004  -7.464  16.369  1.00155.74
O
ANISOU 2143  O   LEU A 293    21118  21276  16782     78   1792
-2084       O
ATOM   2144  CB  LEU A 293      -3.689  -4.672  14.652  1.00 98.01
C
ANISOU 2144  CB  LEU A 293    13770  14033   9436    445   1866
-1929       C
ATOM   2145  CG  LEU A 293      -2.625  -3.571  14.792  1.00 82.17
C
ANISOU 2145  CG  LEU A 293    11895  11777   7549    790   2076
-2126       C
ATOM   2146  CD1 LEU A 293      -1.592  -3.613  13.678  1.00 86.13
C
ANISOU 2146  CD1 LEU A 293    12721  12059   7945    625   2081
-2377       C
ATOM   2147  CD2 LEU A 293      -3.242  -2.185  14.900  1.00 83.03
C
ANISOU 2147  CD2 LEU A 293    11758  12051   7737   1110   2141
-1857       C
ATOM   2148  N   SER A 294      -5.199  -7.475  14.463  1.00131.34
N
ANISOU 2148  N   SER A 294    17961  18566  13377   -403   1485
-1755       N
ATOM   2149  CA  SER A 294      -6.165  -8.446  14.975  1.00133.35
C
ANISOU 2149  CA  SER A 294    18056  19032  13581   -631   1352
-1581       C
ATOM   2150  C   SER A 294      -5.520  -9.777  15.361  1.00139.47
```

```
C
ANISOU 2150  C   SER A 294    19111  19528  14353   -833   1395
-1872       C
ATOM   2151  O   SER A 294      -5.804 -10.323  16.433  1.00170.63
O
ANISOU 2151  O   SER A 294    22942  23495  18395   -776   1416
-1842       O
ATOM   2152  CB  SER A 294      -7.268  -8.675  13.938  1.00161.99
C
ANISOU 2152  CB  SER A 294    21561  23016  16974  -1029   1100
-1276       C
ATOM   2153  OG  SER A 294      -8.227  -9.612  14.400  1.00142.83
O
ANISOU 2153  OG  SER A 294    18976  20814  14478  -1282    961
-1094       O
ATOM   2154  N   VAL A 295      -4.655 -10.322  14.505  1.00125.13
N
ANISOU 2154  N   VAL A 295    17666  17444  12434  -1066   1421
-2145       N
ATOM   2155  CA  VAL A 295      -4.010 -11.596  14.830  1.00107.55
C
ANISOU 2155  CA  VAL A 295    15720  14921  10224  -1238   1492
-2409       C
ATOM   2156  C   VAL A 295      -2.889 -11.460  15.863  1.00103.45
C
ANISOU 2156  C   VAL A 295    15266  14092   9947   -857   1714
-2647       C
ATOM   2157  O   VAL A 295      -2.584 -12.425  16.572  1.00167.64
O
ANISOU 2157  O   VAL A 295    23493  22049  18153   -901   1771
-2767       O
ATOM   2158  CB  VAL A 295      -3.493 -12.270  13.546  1.00123.43
C
ANISOU 2158  CB  VAL A 295    18122  16737  12041  -1626   1477
-2615       C
ATOM   2159  CG1 VAL A 295      -4.633 -12.476  12.555  1.00155.04
C
ANISOU 2159  CG1 VAL A 295    22067  21073  15769  -2067   1237
-2366       C
ATOM   2160  CG2 VAL A 295      -2.366 -11.459  12.923  1.00127.75
C
ANISOU 2160  CG2 VAL A 295    18854  17054  12633  -1426   1622
-2830       C
ATOM   2161  N   THR A 296      -2.257 -10.289  15.966  1.00 83.03
N
ANISOU 2161  N   THR A 296    12634  11437   7478   -501   1839
-2706       N
ATOM   2162  CA  THR A 296      -1.204 -10.085  16.962  1.00139.30
C
ANISOU 2162  CA  THR A 296    19805  18310  14812   -166   2036
-2907       C
ATOM   2163  C   THR A 296      -1.704 -10.249  18.398  1.00123.85
C
ANISOU 2163  C   THR A 296    17605  16467  12986      3   2052
```

```
-2782       C
ATOM   2164  O   THR A 296      -1.044 -10.890  19.224  1.00 78.58
O
ANISOU 2164  O   THR A 296    11957  10533   7368     66   2147
-2931       O
ATOM   2165  CB  THR A 296      -0.579  -8.700  16.781  1.00138.71
C
ANISOU 2165  CB  THR A 296    19715  18185  14805    150   2152
-2960       C
ATOM   2166  OG1 THR A 296       0.045  -8.620  15.494  1.00 94.71
O
ANISOU 2166  OG1 THR A 296    14397  12471   9117     -8   2157
-3111       O
ATOM   2167  CG2 THR A 296       0.463  -8.436  17.858  1.00143.89
C
ANISOU 2167  CG2 THR A 296    20400  18621  15650    464   2342
-3141       C
ATOM   2168  N   MET A 297      -2.860  -9.673  18.717  1.00104.34
N
ANISOU 2168  N   MET A 297    14822  14322  10502     81   1967
-2494       N
ATOM   2169  CA  MET A 297      -3.492  -9.932  20.009  1.00 80.91
C
ANISOU 2169  CA  MET A 297    11619  11493   7628    185   1972
-2355       C
ATOM   2170  C   MET A 297      -3.865 -11.399  20.203  1.00102.53
C
ANISOU 2170  C   MET A 297    14422  14231  10304   -143   1861
-2350       C
ATOM   2171  O   MET A 297      -3.670 -11.954  21.291  1.00161.69
O
ANISOU 2171  O   MET A 297    21890  21640  17904    -72   1921
-2401       O
ATOM   2172  CB  MET A 297      -4.727  -9.045  20.158  1.00 82.37
C
ANISOU 2172  CB  MET A 297    11455  12039   7804    317   1915
-2023       C
ATOM   2173  CG  MET A 297      -4.407  -7.577  20.384  1.00 81.20
C
ANISOU 2173  CG  MET A 297    11223  11866   7763    713   2079
-2018       C
ATOM   2174  SD  MET A 297      -5.883  -6.575  20.638  1.00118.18
S
ANISOU 2174  SD  MET A 297    15487  16945  12472    911   2062
-1602       S
ATOM   2175  CE  MET A 297      -5.159  -5.045  21.225  1.00128.18
C
ANISOU 2175  CE  MET A 297    16774  18036  13892   1388   2337
-1711       C
ATOM   2176  N   ALA A 298      -4.406 -12.042  19.169  1.00120.90
N
ANISOU 2176  N   ALA A 298    16840  16651  12446   -523   1701
-2281       N
ATOM   2177  CA  ALA A 298      -4.785 -13.450  19.277  1.00111.58
```

```
C
ANISOU 2177  CA   ALA A 298    15758  15454  11182   -875   1602
-2280       C
ATOM   2178  C    ALA A 298      -3.604 -14.375  19.572  1.00 84.47
C
ANISOU 2178  C    ALA A 298    12641  11614   7841   -894   1739
-2583       C
ATOM   2179  O    ALA A 298      -3.717 -15.277  20.410  1.00 84.40
O
ANISOU 2179  O    ALA A 298    12626  11552   7889   -959   1739
-2581       O
ATOM   2180  CB   ALA A 298      -5.495 -13.890  17.999  1.00142.81
C
ANISOU 2180  CB   ALA A 298    19802  19570  14889  -1316   1416
-2171       C
ATOM   2181  N    ILE A 299      -2.467 -14.182  18.899  1.00 83.20
N
ANISOU 2181  N    ILE A 299    12747  11164   7702   -834   1861
-2828       N
ATOM   2182  CA   ILE A 299      -1.265 -14.935  19.263  1.00113.47
C
ANISOU 2182  CA   ILE A 299    16835  14613  11665   -777   2023
-3086       C
ATOM   2183  C    ILE A 299      -0.732 -14.544  20.639  1.00 85.56
C
ANISOU 2183  C    ILE A 299    13135  11029   8346   -403   2142
-3103       C
ATOM   2184  O    ILE A 299      -0.186 -15.389  21.360  1.00 79.18
O
ANISOU 2184  O    ILE A 299    12413  10020   7651   -387   2218
-3192       O
ATOM   2185  CB   ILE A 299      -0.184 -14.809  18.167  1.00106.68
C
ANISOU 2185  CB   ILE A 299    16288  13474  10774   -805   2137
-3329       C
ATOM   2186  CG1 ILE A 299       0.259 -13.358  17.954  1.00116.82
C
ANISOU 2186  CG1 ILE A 299    17470  14812  12103   -500   2198
-3346       C
ATOM   2187  CG2 ILE A 299      -0.674 -15.429  16.862  1.00 84.14
C
ANISOU 2187  CG2 ILE A 299    13654  10635   7681  -1245   2032
-3337       C
ATOM   2188  CD1 ILE A 299       1.483 -12.943  18.757  1.00 77.41
C
ANISOU 2188  CD1 ILE A 299    12491   9603   7319   -144   2385
-3505       C
ATOM   2189  N    GLY A 300      -0.869 -13.276  21.028  1.00 78.49
N
ANISOU 2189  N    GLY A 300    12012  10305   7504   -111   2168
-3012       N
ATOM   2190  CA   GLY A 300      -0.488 -12.876  22.376  1.00106.28
C
ANISOU 2190  CA   GLY A 300    15378  13813  11191    197   2276
```

```
-3013       C
ATOM   2191  C   GLY A 300      -1.292 -13.552  23.472  1.00 84.53
C
ANISOU 2191  C   GLY A 300    12437  11213   8467    139   2208
-2854       C
ATOM   2192  O   GLY A 300      -0.738 -13.973  24.490  1.00 85.15
O
ANISOU 2192  O   GLY A 300    12519  11167   8669    241   2286
-2915       O
ATOM   2193  N   SER A 301      -2.606 -13.665  23.281  1.00 79.23
N
ANISOU 2193  N   SER A 301    11591  10832   7680    -37   2057
-2630       N
ATOM   2194  CA  SER A 301      -3.443 -14.403  24.225  1.00 80.16
C
ANISOU 2194  CA  SER A 301    11539  11113   7805   -139   1980
-2470       C
ATOM   2195  C   SER A 301      -3.038 -15.870  24.351  1.00 80.64
C
ANISOU 2195  C   SER A 301    11823  10939   7878   -379   1967
-2584       C
ATOM   2196  O   SER A 301      -3.034 -16.423  25.457  1.00141.55
O
ANISOU 2196  O   SER A 301    19464  18636  15682   -340   1988
-2552       O
ATOM   2197  CB  SER A 301      -4.909 -14.293  23.809  1.00128.62
C
ANISOU 2197  CB  SER A 301    17452  17618  13799   -322   1810
-2194       C
ATOM   2198  OG  SER A 301      -5.758 -14.860  24.790  1.00134.22
O
ANISOU 2198  OG  SER A 301    17958  18519  14519   -391   1745
-2020       O
ATOM   2199  N   HIS A 302      -2.705 -16.523  23.236  1.00 81.64
N
ANISOU 2199  N   HIS A 302    12234  10876   7910   -635   1945
-2712       N
ATOM   2200  CA  HIS A 302      -2.133 -17.867  23.313  1.00 82.16
C
ANISOU 2200  CA  HIS A 302    12562  10640   8013   -819   1993
-2850       C
ATOM   2201  C   HIS A 302      -0.808 -17.897  24.070  1.00 80.11
C
ANISOU 2201  C   HIS A 302    12383  10093   7963   -538   2172
-3014       C
ATOM   2202  O   HIS A 302      -0.559 -18.821  24.853  1.00 90.85
O
ANISOU 2202  O   HIS A 302    13784  11314   9420   -565   2206
-3018       O
ATOM   2203  CB  HIS A 302      -1.949 -18.448  21.912  1.00107.85
C
ANISOU 2203  CB  HIS A 302    16143  13715  11121  -1132   1981
-2981       C
ATOM   2204  CG  HIS A 302      -1.560 -19.893  21.910  1.00110.48
```

```
C
ANISOU 2204  CG  HIS A 302    16767  13738  11472  -1361   2043
-3099       C
ATOM   2205  ND1 HIS A 302      -2.482 -20.913  21.992  1.00130.03
N
ANISOU 2205  ND1 HIS A 302    19275  16301  13830  -1705   1922
-2984       N
ATOM   2206  CD2 HIS A 302      -0.346 -20.489  21.846  1.00 84.51
C
ANISOU 2206  CD2 HIS A 302    13751  10047   8313  -1289   2228
-3309       C
ATOM   2207  CE1 HIS A 302      -1.853 -22.075  21.973  1.00107.16
C
ANISOU 2207  CE1 HIS A 302    16683  13045  10989  -1835   2039
-3130       C
ATOM   2208  NE2 HIS A 302      -0.556 -21.846  21.885  1.00 96.26
N
ANISOU 2208  NE2 HIS A 302    15444  11361   9768  -1574   2231
-3321       N
ATOM   2209  N   ARG A 303       0.063 -16.914  23.839  1.00 78.40
N
ANISOU 2209  N   ARG A 303    12187   9789   7813   -280   2283
-3133       N
ATOM   2210  CA  ARG A 303       1.259 -16.783  24.670  1.00 76.54
C
ANISOU 2210  CA  ARG A 303    11963   9355   7763     -8   2434
-3243       C
ATOM   2211  C   ARG A 303       0.931 -16.605  26.150  1.00 79.85
C
ANISOU 2211  C   ARG A 303    12120   9951   8268    150   2419
-3103       C
ATOM   2212  O   ARG A 303       1.574 -17.219  27.010  1.00122.18
O
ANISOU 2212  O   ARG A 303    17500  15166  13758    213   2484
-3126       O
ATOM   2213  CB  ARG A 303       2.107 -15.609  24.183  1.00 78.72
C
ANISOU 2213  CB  ARG A 303    12275   9569   8065    220   2537
-3368       C
ATOM   2214  CG  ARG A 303       2.951 -15.900  22.958  1.00160.75
C
ANISOU 2214  CG  ARG A 303    22964  19683  18431    125   2621
-3561       C
ATOM   2215  CD  ARG A 303       3.928 -17.028  23.247  1.00148.06
C
ANISOU 2215  CD  ARG A 303    21541  17752  16965    113   2744
-3668       C
ATOM   2216  NE  ARG A 303       4.992 -17.107  22.252  1.00133.37
N
ANISOU 2216  NE  ARG A 303    19908  15623  15143    125   2850
-3827       N
ATOM   2217  CZ  ARG A 303       6.014 -17.953  22.319  1.00143.52
C
ANISOU 2217  CZ  ARG A 303    21257  16657  16616    173   2891
```

```
-3813       C
ATOM   2218  NH1 ARG A 303       6.033 -18.900  23.245  1.00168.44
N
ANISOU 2218  NH1 ARG A 303    24446  19696  19859    157   2976
-3806       N
ATOM   2219  NH2 ARG A 303       6.948 -17.940  21.376  1.00126.63
N
ANISOU 2219  NH2 ARG A 303    19175  14381  14559    207   2869
-3812       N
ATOM   2220  N   LEU A 304      -0.059 -15.770  26.471  1.00 75.79
N
ANISOU 2220  N   LEU A 304    11358   9752   7686    217   2347
-2947       N
ATOM   2221  CA  LEU A 304      -0.541 -15.689  27.848  1.00 75.48
C
ANISOU 2221  CA  LEU A 304    11082   9898   7700    321   2338
-2808       C
ATOM   2222  C   LEU A 304      -1.130 -17.005  28.345  1.00 76.89
C
ANISOU 2222  C   LEU A 304    11249  10094   7871     87   2243
-2705       C
ATOM   2223  O   LEU A 304      -0.958 -17.360  29.517  1.00102.29
O
ANISOU 2223  O   LEU A 304    14379  13310  11177    153   2270
-2661       O
ATOM   2224  CB  LEU A 304      -1.562 -14.563  27.985  1.00 78.92
C
ANISOU 2224  CB  LEU A 304    11266  10655   8066    437   2307
-2650       C
ATOM   2225  CG  LEU A 304      -1.035 -13.149  27.750  1.00111.62
C
ANISOU 2225  CG  LEU A 304    15397  14786  12227    703   2424
-2730       C
ATOM   2226  CD1 LEU A 304      -2.154 -12.150  27.965  1.00 97.89
C
ANISOU 2226  CD1 LEU A 304    13404  13351  10438    825   2417
-2540       C
ATOM   2227  CD2 LEU A 304       0.156 -12.847  28.651  1.00 72.55
C
ANISOU 2227  CD2 LEU A 304    10501   9670   7394    907   2567
-2865       C
ATOM   2228  N   SER A 305      -1.841 -17.737  27.484  1.00 78.74
N
ANISOU 2228  N   SER A 305    11578  10356   7985   -209   2128
-2659       N
ATOM   2229  CA  SER A 305      -2.292 -19.071  27.874  1.00 99.36
C
ANISOU 2229  CA  SER A 305    14235  12934  10582   -461   2050
-2586       C
ATOM   2230  C   SER A 305      -1.121 -19.981  28.228  1.00 79.77
C
ANISOU 2230  C   SER A 305    11969  10092   8247   -434   2163
-2724       C
ATOM   2231  O   SER A 305      -1.188 -20.736  29.205  1.00 80.08
```

```
O
ANISOU 2231  O   SER A 305    11956  10112   8357   -468   2151
-2646       O
ATOM   2232  CB  SER A 305      -3.130 -19.689  26.754  1.00135.71
C
ANISOU 2232  CB  SER A 305    18960  17597  15006   -824   1920
-2539       C
ATOM   2233  OG  SER A 305      -3.722 -20.911  27.165  1.00152.82
O
ANISOU 2233  OG  SER A 305    21160  19771  17135  -1093   1836
-2446       O
ATOM   2234  N   GLN A 306      -0.043 -19.929  27.445  1.00 87.17
N
ANISOU 2234  N   GLN A 306    13139  10744   9236   -369   2279
-2912       N
ATOM   2235  CA  GLN A 306       1.205 -20.563  27.860  1.00 78.63
C
ANISOU 2235  CA  GLN A 306    12207   9339   8331   -255   2418
-3016       C
ATOM   2236  C   GLN A 306       1.741 -19.950  29.149  1.00 86.44
C
ANISOU 2236  C   GLN A 306    12979  10414   9450     28   2470
-2964       C
ATOM   2237  O   GLN A 306       2.191 -20.670  30.049  1.00 76.93
O
ANISOU 2237  O   GLN A 306    11761   9100   8369     59   2504
-2915       O
ATOM   2238  CB  GLN A 306       2.245 -20.457  26.745  1.00 78.41
C
ANISOU 2238  CB  GLN A 306    12438   9024   8332   -212   2549
-3216       C
ATOM   2239  CG  GLN A 306       1.860 -21.167  25.461  1.00107.40
C
ANISOU 2239  CG  GLN A 306    16383  12562  11864   -526   2528
-3295       C
ATOM   2240  CD  GLN A 306       2.879 -20.961  24.359  1.00124.55
C
ANISOU 2240  CD  GLN A 306    18807  14465  14053   -475   2674
-3500       C
ATOM   2241  OE1 GLN A 306       3.865 -20.243  24.536  1.00108.96
O
ANISOU 2241  OE1 GLN A 306    16782  12421  12197   -197   2782
-3574       O
ATOM   2242  NE2 GLN A 306       2.651 -21.594  23.215  1.00102.08
N
ANISOU 2242  NE2 GLN A 306    16241  11470  11073   -765   2684
-3593       N
ATOM   2243  N   GLN A 307       1.705 -18.618  29.248  1.00 90.20
N
ANISOU 2243  N   GLN A 307    13297  11083   9892    222   2481
-2967       N
ATOM   2244  CA  GLN A 307       2.229 -17.933  30.427  1.00113.11
C
ANISOU 2244  CA  GLN A 307    16027  14070  12881    458   2544
```

```
-2937       C
ATOM   2245  C   GLN A 307       1.483 -18.335  31.694  1.00105.77
C
ANISOU 2245  C   GLN A 307    14901  13334  11953    407   2471
-2766       C
ATOM   2246  O   GLN A 307       2.101 -18.557  32.742  1.00 79.56
O
ANISOU 2246  O   GLN A 307    11522   9974   8733    493   2514
-2731       O
ATOM   2247  CB  GLN A 307       2.144 -16.419  30.222  1.00140.28
C
ANISOU 2247  CB  GLN A 307    19365  17677  16256    641   2580
-2972       C
ATOM   2248  CG  GLN A 307       3.116 -15.608  31.060  1.00135.77
C
ANISOU 2248  CG  GLN A 307    18731  17093  15762    872   2692
-3021       C
ATOM   2249  CD  GLN A 307       4.546 -15.762  30.587  1.00133.83
C
ANISOU 2249  CD  GLN A 307    18665  16568  15617    946   2792
-3166       C
ATOM   2250  OE1 GLN A 307       4.926 -15.230  29.544  1.00111.14
O
ANISOU 2250  OE1 GLN A 307    15915  13602  12712    988   2838
-3288       O
ATOM   2251  NE2 GLN A 307       5.348 -16.498  31.349  1.00144.39
N
ANISOU 2251  NE2 GLN A 307    20005  17778  17078    964   2828
-3136       N
ATOM   2252  N   GLY A 308       0.161 -18.439  31.619  1.00124.92
N
ANISOU 2252  N   GLY A 308    17214  15986  14263    256   2357
-2643       N
ATOM   2253  CA  GLY A 308      -0.615 -18.778  32.793  1.00 76.10
C
ANISOU 2253  CA  GLY A 308    10836  10009   8070    202   2293
-2477       C
ATOM   2254  C   GLY A 308      -1.776 -17.830  33.010  1.00 76.32
C
ANISOU 2254  C   GLY A 308    10627  10379   7993    255   2256
-2355       C
ATOM   2255  O   GLY A 308      -2.420 -17.861  34.062  1.00 76.69
O
ANISOU 2255  O   GLY A 308    10484  10629   8027    254   2234
-2221       O
ATOM   2256  N   ALA A 309      -2.039 -16.962  32.035  1.00 86.57
N
ANISOU 2256  N   ALA A 309    11928  11744   9220    312   2262
-2389       N
ATOM   2257  CA  ALA A 309      -3.083 -15.951  32.151  1.00106.58
C
ANISOU 2257  CA  ALA A 309    14231  14585  11678    408   2258
-2256       C
ATOM   2258  C   ALA A 309      -4.040 -16.067  30.974  1.00116.92
```

```
C
ANISOU 2258  C   ALA A 309    15525  16028  12872    223   2133
-2162       C
ATOM   2259  O   ALA A 309      -3.622 -15.973  29.816  1.00 78.18
O
ANISOU 2259  O   ALA A 309    10794  10980   7932    175   2123
-2270       O
ATOM   2260  CB  ALA A 309      -2.476 -14.546  32.217  1.00 75.15
C
ANISOU 2260  CB  ALA A 309    10238  10587   7729    697   2407
-2353       C
ATOM   2261  N   ILE A 310      -5.316 -16.280  31.273  1.00126.45
N
ANISOU 2261  N   ILE A 310    16517  17522  14006    102   2035
-1949       N
ATOM   2262  CA  ILE A 310      -6.354 -16.473  30.268  1.00 81.85
C
ANISOU 2262  CA  ILE A 310    10809  12064   8225   -122   1888
-1804       C
ATOM   2263  C   ILE A 310      -7.043 -15.128  30.068  1.00 82.18
C
ANISOU 2263  C   ILE A 310    10615  12365   8242     89   1931
-1663       C
ATOM   2264  O   ILE A 310      -7.773 -14.658  30.945  1.00124.03
O
ANISOU 2264  O   ILE A 310    15658  17901  13565    226   1981
-1502       O
ATOM   2265  CB  ILE A 310      -7.342 -17.573  30.676  1.00 83.75
C
ANISOU 2265  CB  ILE A 310    10946  12479   8396   -408   1746
-1628       C
ATOM   2266  CG1 ILE A 310      -6.602 -18.903  30.849  1.00 83.59
C
ANISOU 2266  CG1 ILE A 310    11189  12156   8416   -599   1728
-1767       C
ATOM   2267  CG2 ILE A 310      -8.463 -17.697  29.656  1.00 86.06
C
ANISOU 2267  CG2 ILE A 310    11150  13021   8529   -665   1581
-1446       C
ATOM   2268  CD1 ILE A 310      -7.497 -20.062  31.228  1.00 90.98
C
ANISOU 2268  CD1 ILE A 310    12070  13222   9276   -907   1592
-1612       C
ATOM   2269  N   THR A 311      -6.810 -14.503  28.916  1.00 82.09
N
ANISOU 2269  N   THR A 311    10696  12305   8190    121   1929
-1717       N
ATOM   2270  CA  THR A 311      -7.426 -13.218  28.584  1.00 82.64
C
ANISOU 2270  CA  THR A 311    10560  12595   8246    326   1975
-1568       C
ATOM   2271  C   THR A 311      -8.887 -13.445  28.218  1.00 85.33
C
ANISOU 2271  C   THR A 311    10647  13299   8476    128   1813
```

```
-1262       C
ATOM   2272  O   THR A 311      -9.239 -13.663  27.058  1.00127.83
O
ANISOU 2272  O   THR A 311    16074  18757  13739   -102   1668
-1191       O
ATOM   2273  CB  THR A 311      -6.674 -12.539  27.447  1.00 87.58
C
ANISOU 2273  CB  THR A 311    11374  13047   8857    401   2012
-1716       C
ATOM   2274  OG1 THR A 311      -5.777 -13.477  26.838  1.00143.54
O
ANISOU 2274  OG1 THR A 311    18774  19847  15919    187   1963
-1931       O
ATOM   2275  CG2 THR A 311      -5.883 -11.355  27.967  1.00 91.29
C
ANISOU 2275  CG2 THR A 311    11865  13386   9437    767   2221
-1845       C
ATOM   2276  N   LYS A 312      -9.746 -13.404  29.241  1.00 86.32
N
ANISOU 2276  N   LYS A 312    10499  13666   8631    202   1838
-1068       N
ATOM   2277  CA  LYS A 312     -11.189 -13.448  29.024  1.00106.52
C
ANISOU 2277  CA  LYS A 312    12748  16620  11103     67   1709
-731       C
ATOM   2278  C   LYS A 312     -11.658 -12.300  28.134  1.00125.11
C
ANISOU 2278  C   LYS A 312    14945  19151  13441    222   1722
-560       C
ATOM   2279  O   LYS A 312     -12.450 -12.504  27.207  1.00169.55
O
ANISOU 2279  O   LYS A 312    20461  25013  18946    -14   1542
-346       O
ATOM   2280  CB  LYS A 312     -11.910 -13.416  30.374  1.00111.58
C
ANISOU 2280  CB  LYS A 312    13120  17469  11806    193   1792
-571       C
ATOM   2281  CG  LYS A 312     -13.386 -13.053  30.307  1.00121.10
C
ANISOU 2281  CG  LYS A 312    13932  19110  12969    194   1735
-189       C
ATOM   2282  CD  LYS A 312     -14.198 -14.073  29.531  1.00149.98
C
ANISOU 2282  CD  LYS A 312    17534  22988  16464   -246   1463
-2      C
ATOM   2283  CE  LYS A 312     -15.686 -13.848  29.742  1.00168.09
C
ANISOU 2283  CE  LYS A 312    19389  25751  18726   -254   1408
409       C
ATOM   2284  NZ  LYS A 312     -16.125 -12.529  29.203  1.00171.25
N
ANISOU 2284  NZ  LYS A 312    19559  26330  19180     36   1498
618       N
ATOM   2285  N   ARG A 313     -11.180 -11.087  28.398  1.00108.97
```

N
ANISOU 2285 N   ARG A 313   12894 17000 11510   603   1934
-637   N
ATOM   2286 CA ARG A 313   -11.169 -10.025 27.399 1.00103.51
C
ANISOU 2286 CA ARG A 313   12182 16333 10815   756   1965
-573   C
ATOM   2287 C   ARG A 313   -9.888 -10.083 26.575 1.00110.24
C
ANISOU 2287 C   ARG A 313   13403 16840 11644   695   1957
-884   C
ATOM   2288 O   ARG A 313   -8.783 -10.047 27.128 1.00 84.82
O
ANISOU 2288 O   ARG A 313   10402 13322   8503   827   2093
-1164   O
ATOM   2289 CB ARG A 313   -11.318 -8.647 28.040 1.00104.49
C
ANISOU 2289 CB ARG A 313   12138 16495 11070   1195   2218
-495   C
ATOM   2290 CG ARG A 313   -12.741 -8.298 28.444 1.00123.76
C
ANISOU 2290 CG ARG A 313   14168 19320 13535   1298   2243
-110   C
ATOM   2291 CD ARG A 313   -12.787 -6.939 29.120 1.00154.30
C
ANISOU 2291 CD ARG A 313   17928 23157 17541   1748   2550
-71   C
ATOM   2292 NE ARG A 313   -12.238 -5.906 28.244 1.00173.61
N
ANISOU 2292 NE ARG A 313   20502 25447 20014   1936   2630
-138   N
ATOM   2293 CZ ARG A 313   -11.265 -5.065 28.575 1.00185.96
C
ANISOU 2293 CZ ARG A 313   22289 26714 21652   2195   2856
-391   C
ATOM   2294 NH1 ARG A 313   -10.894 -4.939 29.840 1.00196.62
N
ANISOU 2294 NH1 ARG A 313   23709 27935 23064   2343   3058
-551   N
ATOM   2295 NH2 ARG A 313   -10.822 -4.191 27.680 1.00189.67
N
ANISOU 2295 NH2 ARG A 313   22873 27061 22133   2328   2902
-431   N
ATOM   2296 N   MET A 314   -10.044 -10.167 25.252 1.00141.14
N
ANISOU 2296 N   MET A 314   17375 20811 15439   482   1800
-822   N
ATOM   2297 CA MET A 314   -8.900 -10.208 24.352 1.00126.64
C
ANISOU 2297 CA MET A 314   15883 18668 13566   407   1797
-1101   C
ATOM   2298 C   MET A 314   -8.201 -8.859 24.258 1.00 85.94
C
ANISOU 2298 C   MET A 314   10784 13361   8510   773   1989

```
-1208       C
ATOM   2299  O   MET A 314      -7.021  -8.810  23.894  1.00 83.42
O
ANISOU 2299  O   MET A 314    10755  12737   8203    794   2052
-1490       O
ATOM   2300  CB  MET A 314      -9.344 -10.661  22.960  1.00143.37
C
ANISOU 2300  CB  MET A 314    18054  20916  15503     43   1578
-991        C
ATOM   2301  CG  MET A 314      -9.947 -12.055  22.916  1.00153.48
C
ANISOU 2301  CG  MET A 314    19350  22312  16652   -384   1385
-917        C
ATOM   2302  SD  MET A 314      -8.751 -13.342  23.321  1.00135.28
S
ANISOU 2302  SD  MET A 314    17444  19580  14377   -547   1435
-1297       S
ATOM   2303  CE  MET A 314      -7.769 -13.356  21.822  1.00 88.10
C
ANISOU 2303  CE  MET A 314    11847  13326   8299   -712   1423
-1543       C
ATOM   2304  N   THR A 315      -8.900  -7.770  24.574  1.00 86.28
N
ANISOU 2304  N   THR A 315    10560  13601   8623   1060   2098
-983       N
ATOM   2305  CA  THR A 315      -8.383  -6.417  24.417  1.00 85.37
C
ANISOU 2305  CA  THR A 315    10492  13355   8588   1398   2286
-1046       C
ATOM   2306  C   THR A 315      -7.699  -5.892  25.676  1.00127.39
C
ANISOU 2306  C   THR A 315    15888  18478  14039   1696   2537
-1237       C
ATOM   2307  O   THR A 315      -7.317  -4.718  25.715  1.00126.77
O
ANISOU 2307  O   THR A 315    15853  18288  14026   1984   2724
-1288       O
ATOM   2308  CB  THR A 315      -9.515  -5.469  24.014  1.00 93.17
C
ANISOU 2308  CB  THR A 315    11166  14644   9590   1559   2295
-679        C
ATOM   2309  OG1 THR A 315     -10.602  -5.604  24.938  1.00136.81
O
ANISOU 2309  OG1 THR A 315    16377  20437  15167   1628   2325
-422        O
ATOM   2310  CG2 THR A 315     -10.013  -5.806  22.618  1.00126.20
C
ANISOU 2310  CG2 THR A 315    15310  19015  13624   1254   2044
-500        C
ATOM   2311  N   ALA A 316      -7.538  -6.731  26.702  1.00123.68
N
ANISOU 2311  N   ALA A 316    15441  17961  13592   1613   2543
-1336       N
ATOM   2312  CA  ALA A 316      -6.884  -6.334  27.944  1.00115.54
```

```
C
ANISOU 2312  CA  ALA A 316    14484  16764  12652   1836   2760
-1510       C
ATOM   2313  C   ALA A 316      -5.382  -6.119  27.790  1.00 99.56
C
ANISOU 2313  C   ALA A 316    12779  14402  10645   1883   2841
-1837       C
ATOM   2314  O   ALA A 316      -4.760  -5.545  28.690  1.00 77.27
O
ANISOU 2314  O   ALA A 316    10034  11446   7880   2073   3031
-1977       O
ATOM   2315  CB  ALA A 316      -7.149  -7.382  29.026  1.00121.16
C
ANISOU 2315  CB  ALA A 316    15122  17543  13369   1694   2713
-1499       C
ATOM   2316  N   ILE A 317      -4.793  -6.570  26.680  1.00 77.96
N
ANISOU 2316  N   ILE A 317    10232  11537   7852   1697   2708
-1957       N
ATOM   2317  CA  ILE A 317      -3.374  -6.343  26.409  1.00 75.88
C
ANISOU 2317  CA  ILE A 317    10254  10970   7607   1743   2787
-2245       C
ATOM   2318  C   ILE A 317      -3.027  -4.859  26.450  1.00 83.41
C
ANISOU 2318  C   ILE A 317    11239  11851   8600   2042   2986
-2287       C
ATOM   2319  O   ILE A 317      -2.005  -4.461  27.023  1.00 74.89
O
ANISOU 2319  O   ILE A 317    10319  10576   7561   2161   3130
-2492       O
ATOM   2320  CB  ILE A 317      -2.997  -6.962  25.049  1.00 91.75
C
ANISOU 2320  CB  ILE A 317    12441  12883   9538   1500   2630
-2328       C
ATOM   2321  CG1 ILE A 317      -3.228  -8.472  25.061  1.00 90.42
C
ANISOU 2321  CG1 ILE A 317    12302  12729   9326   1190   2467
-2322       C
ATOM   2322  CG2 ILE A 317      -1.550  -6.644  24.688  1.00 96.84
C
ANISOU 2322  CG2 ILE A 317    13359  13229  10206   1567   2726
-2607       C
ATOM   2323  CD1 ILE A 317      -2.371  -9.197  26.058  1.00 75.08
C
ANISOU 2323  CD1 ILE A 317    10471  10594   7463   1187   2532
-2497       C
ATOM   2324  N   GLU A 318      -3.867  -4.019  25.847  1.00100.46
N
ANISOU 2324  N   GLU A 318    13251  14172  10747   2158   2998
-2079       N
ATOM   2325  CA  GLU A 318      -3.661  -2.573  25.894  1.00128.60
C
ANISOU 2325  CA  GLU A 318    16845  17662  14355   2455   3208
```

```
-2090       C
ATOM   2326  C   GLU A 318      -3.676  -2.024  27.319  1.00137.02
C
ANISOU 2326  C   GLU A 318    17870  18705  15488   2670   3438
-2118       C
ATOM   2327  O   GLU A 318      -2.793  -1.248  27.705  1.00129.53
O
ANISOU 2327  O   GLU A 318    17094  17554  14566   2798   3604
-2303       O
ATOM   2328  CB  GLU A 318      -4.733  -1.890  25.041  1.00160.08
C
ANISOU 2328  CB  GLU A 318    20632  21859  18333   2541   3172
-1795       C
ATOM   2329  CG  GLU A 318      -4.933  -0.409  25.312  1.00169.36
C
ANISOU 2329  CG  GLU A 318    21762  23006  19581   2889   3424
-1711       C
ATOM   2330  CD  GLU A 318      -5.886   0.237  24.320  1.00117.03
C
ANISOU 2330  CD  GLU A 318    14938  16573  12955   2973   3374
-1397       C
ATOM   2331  OE1 GLU A 318      -5.492   1.226  23.667  1.00 85.36
O
ANISOU 2331  OE1 GLU A 318    11041  12439   8952   3121   3465
-1424       O
ATOM   2332  OE2 GLU A 318      -7.032  -0.247  24.195  1.00 84.29
O
ANISOU 2332  OE2 GLU A 318    10514  12715   8798   2881   3238
-1107       O
ATOM   2333  N   GLU A 319      -4.672  -2.411  28.116  1.00130.04
N
ANISOU 2333  N   GLU A 319    16756  18027  14627   2670   3438
-1932       N
ATOM   2334  CA  GLU A 319      -4.712  -2.012  29.523  1.00 80.79
C
ANISOU 2334  CA  GLU A 319    10491  11773   8432   2833   3658
-1967       C
ATOM   2335  C   GLU A 319      -3.569  -2.607  30.344  1.00 75.78
C
ANISOU 2335  C   GLU A 319    10056  10957   7781   2713   3666
-2236       C
ATOM   2336  O   GLU A 319      -2.950  -1.904  31.150  1.00 75.81
O
ANISOU 2336  O   GLU A 319    10109  10798   7897   2659   3698
-2343       O
ATOM   2337  CB  GLU A 319      -6.061  -2.397  30.129  1.00 79.92
C
ANISOU 2337  CB  GLU A 319    10077  11944   8346   2834   3642
-1696       C
ATOM   2338  CG  GLU A 319      -7.232  -1.619  29.556  1.00 82.44
C
ANISOU 2338  CG  GLU A 319    10153  12466   8704   3015   3694
-1384       C
ATOM   2339  CD  GLU A 319      -8.418  -2.506  29.244  1.00 97.93
```

```
C
ANISOU 2339  CD  GLU A 319    11823  14742  10643   2835   3473
-1094       C
ATOM   2340  OE1 GLU A 319      -9.519  -2.229  29.766  1.00132.24
O
ANISOU 2340  OE1 GLU A 319    15895  19310  15039   2975   3575
-833       O
ATOM   2341  OE2 GLU A 319      -8.251  -3.478  28.478  1.00 83.83
O
ANISOU 2341  OE2 GLU A 319    10089  12981   8783   2544   3210
-1124       O
ATOM   2342  N   MET A 320      -3.274  -3.894  30.155  1.00 75.00
N
ANISOU 2342  N   MET A 320     9988  10848   7660   2459   3453
-2291       N
ATOM   2343  CA  MET A 320      -2.134  -4.512  30.830  1.00109.79
C
ANISOU 2343  CA  MET A 320    14567  15081  12067   2350   3448
-2512       C
ATOM   2344  C   MET A 320      -0.809  -3.838  30.480  1.00119.69
C
ANISOU 2344  C   MET A 320    15965  16099  13413   2277   3383
-2698       C
ATOM   2345  O   MET A 320       0.029  -3.616  31.363  1.00 77.89
O
ANISOU 2345  O   MET A 320    10656  10738   8200   2186   3310
-2788       O
ATOM   2346  CB  MET A 320      -2.082  -5.999  30.483  1.00120.42
C
ANISOU 2346  CB  MET A 320    15917  16430  13406   2081   3217
-2511       C
ATOM   2347  CG  MET A 320      -3.135  -6.836  31.193  1.00 86.56
C
ANISOU 2347  CG  MET A 320    11417  12355   9116   1968   3133
-2326       C
ATOM   2348  SD  MET A 320      -2.779  -7.174  32.925  1.00 73.64
S
ANISOU 2348  SD  MET A 320     9769  10708   7503   1970   3234
-2389       S
ATOM   2349  CE  MET A 320      -1.620  -8.531  32.767  1.00 90.77
C
ANISOU 2349  CE  MET A 320    12123  12668   9697   1741   3078
-2550       C
ATOM   2350  N   ALA A 321      -0.596  -3.509  29.204  1.00121.32
N
ANISOU 2350  N   ALA A 321    16256  16236  13603   2286   3334
-2723       N
ATOM   2351  CA  ALA A 321       0.589  -2.744  28.818  1.00 80.10
C
ANISOU 2351  CA  ALA A 321    11084  10861   8490   2198   3199
-2846       C
ATOM   2352  C   ALA A 321       0.625  -1.366  29.471  1.00 73.52
C
ANISOU 2352  C   ALA A 321    10165  10021   7746   2260   3246
```

```
-2813       C
ATOM   2353  O   ALA A 321       1.696  -0.887  29.862  1.00 87.44
O
ANISOU 2353  O   ALA A 321    11936  11719   9567   2181   3126
-2909       O
ATOM   2354  CB  ALA A 321       0.659  -2.615  27.298  1.00 70.29
C
ANISOU 2354  CB  ALA A 321     9948   9563   7194   2202   3165
-2861       C
ATOM   2355  N   GLY A 322      -0.527  -0.711  29.599  1.00 72.12
N
ANISOU 2355  N   GLY A 322     9911   9931   7562   2427   3417
-2658       N
ATOM   2356  CA  GLY A 322      -0.584   0.619  30.177  1.00 93.23
C
ANISOU 2356  CA  GLY A 322    12540  12570  10313   2494   3485
-2627       C
ATOM   2357  C   GLY A 322      -0.671   0.679  31.686  1.00103.80
C
ANISOU 2357  C   GLY A 322    13816  13958  11664   2465   3536
-2639       C
ATOM   2358  O   GLY A 322      -0.603   1.772  32.256  1.00 73.71
O
ANISOU 2358  O   GLY A 322    10007  10108   7891   2497   3587
-2642       O
ATOM   2359  N   MET A 323      -0.838  -0.472  32.338  1.00 78.40
N
ANISOU 2359  N   MET A 323    10565  10826   8398   2406   3533
-2645       N
ATOM   2360  CA  MET A 323      -1.044  -0.537  33.781  1.00 74.52
C
ANISOU 2360  CA  MET A 323    10015  10409   7891   2381   3600
-2642       C
ATOM   2361  C   MET A 323       0.019   0.236  34.554  1.00 72.53
C
ANISOU 2361  C   MET A 323     9823  10092   7642   2291   3510
-2760       C
ATOM   2362  O   MET A 323       1.212   0.167  34.248  1.00117.00
O
ANISOU 2362  O   MET A 323    15527  15657  13271   2199   3339
-2861       O
ATOM   2363  CB  MET A 323      -1.064  -1.989  34.260  1.00 92.99
C
ANISOU 2363  CB  MET A 323    12340  12827  10165   2301   3573
-2654       C
ATOM   2364  CG  MET A 323      -1.540  -2.121  35.700  1.00 73.61
C
ANISOU 2364  CG  MET A 323     9810  10482   7675   2295   3679
-2613       C
ATOM   2365  SD  MET A 323      -1.137  -3.697  36.467  1.00 85.77
S
ANISOU 2365  SD  MET A 323    11373  12083   9134   2167   3617
-2657       S
ATOM   2366  CE  MET A 323      -2.165  -4.807  35.513  1.00 97.63
```

```
C
ANISOU 2366  CE  MET A 323    12838  13685  10573   2254   3680
-2529       C
ATOM   2367  N   ASP A 324      -0.439   1.003  35.544  1.00 73.89
N
ANISOU 2367  N   ASP A 324     9970  10299   7805   2338   3637
-2725       N
ATOM   2368  CA  ASP A 324       0.392   1.909  36.323  1.00 98.55
C
ANISOU 2368  CA  ASP A 324    13176  13377  10889   2282   3595
-2805       C
ATOM   2369  C   ASP A 324       0.453   1.543  37.800  1.00148.44
C
ANISOU 2369  C   ASP A 324    19498  19789  17114   2217   3633
-2821       C
ATOM   2370  O   ASP A 324       1.469   1.813  38.446  1.00159.18
O
ANISOU 2370  O   ASP A 324    20952  21132  18398   2134   3549
-2885       O
ATOM   2371  CB  ASP A 324      -0.123   3.352  36.190  1.00117.73
C
ANISOU 2371  CB  ASP A 324    15634  15732  13367   2397   3734
-2755       C
ATOM   2372  CG  ASP A 324       0.924   4.383  36.552  1.00171.52
C
ANISOU 2372  CG  ASP A 324    22574  22460  20135   2332   3670
-2842       C
ATOM   2373  OD1 ASP A 324       0.585   5.585  36.570  1.00182.16
O
ANISOU 2373  OD1 ASP A 324    23978  23726  21509   2416   3796
-2810       O
ATOM   2374  OD2 ASP A 324       2.084   3.997  36.811  1.00194.52
O
ANISOU 2374  OD2 ASP A 324    25540  25384  22984   2211   3508
-2922       O
ATOM   2375  N   VAL A 325      -0.594   0.925  38.346  1.00160.26
N
ANISOU 2375  N   VAL A 325    20899  21392  18600   2254   3768
-2744       N
ATOM   2376  CA  VAL A 325      -0.658   0.549  39.756  1.00104.24
C
ANISOU 2376  CA  VAL A 325    13803  14399  11404   2191   3827
-2746       C
ATOM   2377  C   VAL A 325      -1.407  -0.774  39.869  1.00109.23
C
ANISOU 2377  C   VAL A 325    14324  15150  12028   2186   3877
-2675       C
ATOM   2378  O   VAL A 325      -2.437  -0.970  39.214  1.00 76.69
O
ANISOU 2378  O   VAL A 325    10101  11064   7974   2288   3976
-2570       O
ATOM   2379  CB  VAL A 325      -1.328   1.645  40.613  1.00 95.59
C
ANISOU 2379  CB  VAL A 325    12719  13310  10290   2254   4014
```

```
-2715       C
ATOM   2380  CG1 VAL A 325      -2.726   1.956  40.105  1.00 98.26
C
ANISOU 2380  CG1 VAL A 325    12937  13664  10734   2421   4195
-2584       C
ATOM   2381  CG2 VAL A 325      -1.373   1.232  42.077  1.00125.54
C
ANISOU 2381  CG2 VAL A 325    16527  17214  13958   2171   4081
-2719       C
ATOM   2382  N   LEU A 326      -0.876  -1.697  40.669  1.00107.51
N
ANISOU 2382  N   LEU A 326    14138  14996  11717   2080   3815
-2702       N
ATOM   2383  CA  LEU A 326      -1.489  -3.007  40.867  1.00 75.75
C
ANISOU 2383  CA  LEU A 326    10031  11082   7668   2056   3866
-2634       C
ATOM   2384  C   LEU A 326      -1.870  -3.192  42.333  1.00107.71
C
ANISOU 2384  C   LEU A 326    14052  15261  11613   2004   3985
-2596       C
ATOM   2385  O   LEU A 326      -0.995  -3.292  43.200  1.00141.35
O
ANISOU 2385  O   LEU A 326    18421  19512  15774   1908   3928
-2635       O
ATOM   2386  CB  LEU A 326      -0.538  -4.107  40.402  1.00 73.34
C
ANISOU 2386  CB  LEU A 326     9805  10712   7350   1980   3704
-2679       C
ATOM   2387  CG  LEU A 326      -0.977  -5.555  40.602  1.00 73.38
C
ANISOU 2387  CG  LEU A 326     9772  10792   7318   1931   3754
-2619       C
ATOM   2388  CD1 LEU A 326      -2.360  -5.793  40.022  1.00 74.61
C
ANISOU 2388  CD1 LEU A 326     9797  11052   7498   2032   3887
-2502       C
ATOM   2389  CD2 LEU A 326       0.036  -6.466  39.953  1.00 71.83
C
ANISOU 2389  CD2 LEU A 326     9691  10455   7147   1859   3610
-2678       C
ATOM   2390  N   CYS A 327      -3.172  -3.244  42.606  1.00 95.93
N
ANISOU 2390  N   CYS A 327    12421  13888  10141   2068   4163
-2491       N
ATOM   2391  CA  CYS A 327      -3.699  -3.446  43.955  1.00 91.60
C
ANISOU 2391  CA  CYS A 327    11824  13484   9498   2016   4297
-2443       C
ATOM   2392  C   CYS A 327      -3.869  -4.942  44.204  1.00 78.88
C
ANISOU 2392  C   CYS A 327    10157  11986   7828   1933   4296
-2378       C
ATOM   2393  O   CYS A 327      -4.753  -5.576  43.617  1.00 79.33
```

```
O
ANISOU 2393  O   CYS A 327    10087  12133   7923   2013   4352
-2250       O
ATOM   2394  CB  CYS A 327      -5.019  -2.701  44.139  1.00 84.89
C
ANISOU 2394  CB  CYS A 327    10844  12703   8709   2143   4503
-2337       C
ATOM   2395  SG  CYS A 327      -4.848  -0.903  44.166  1.00 87.33
S
ANISOU 2395  SG  CYS A 327    11259  12863   9062   2231   4559
-2409       S
ATOM   2396  N   SER A 328      -3.034  -5.510  45.074  1.00 79.41
N
ANISOU 2396  N   SER A 328    10331  12052   7788   1790   4239
-2420       N
ATOM   2397  CA  SER A 328      -2.862  -6.957  45.174  1.00 78.92
C
ANISOU 2397  CA  SER A 328    10256  12021   7709   1638   4217
-2408       C
ATOM   2398  C   SER A 328      -3.329  -7.448  46.539  1.00 87.13
C
ANISOU 2398  C   SER A 328    11207  13237   8663   1482   4265
-2330       C
ATOM   2399  O   SER A 328      -2.808  -7.013  47.572  1.00 79.03
O
ANISOU 2399  O   SER A 328    10302  12203   7522   1398   4347
-2386       O
ATOM   2400  CB  SER A 328      -1.402  -7.353  44.940  1.00 75.64
C
ANISOU 2400  CB  SER A 328     9992  11441   7307   1517   4045
-2514       C
ATOM   2401  OG  SER A 328      -1.237  -8.756  45.040  1.00 75.20
O
ANISOU 2401  OG  SER A 328     9845  11420   7307   1337   3823
-2434       O
ATOM   2402  N   ASP A 329      -4.314  -8.346  46.535  1.00121.65
N
ANISOU 2402  N   ASP A 329    15364  17769  13090   1430   4132
-2153       N
ATOM   2403  CA  ASP A 329      -4.758  -9.004  47.758  1.00120.01
C
ANISOU 2403  CA  ASP A 329    15052  17735  12811   1257   4119
-2054       C
ATOM   2404  C   ASP A 329      -3.613  -9.791  48.385  1.00118.20
C
ANISOU 2404  C   ASP A 329    14918  17447  12543   1025   3954
-2099       C
ATOM   2405  O   ASP A 329      -3.088 -10.730  47.780  1.00122.38
O
ANISOU 2405  O   ASP A 329    15440  17888  13170    945   3713
-2069       O
ATOM   2406  CB  ASP A 329      -5.947  -9.920  47.468  1.00125.02
C
ANISOU 2406  CB  ASP A 329    15440  18544  13520   1222   3967
```

```
-1848       C
ATOM   2407  CG  ASP A 329      -7.201  -9.150  47.096  1.00131.47
C
ANISOU 2407  CG  ASP A 329    16104  19485  14363   1438   4148
-1744       C
ATOM   2408  OD1 ASP A 329      -7.271  -7.939  47.399  1.00155.77
O
ANISOU 2408  OD1 ASP A 329    19265  22528  17391   1610   4441
-1825       O
ATOM   2409  OD2 ASP A 329      -8.122  -9.757  46.511  1.00116.10
O
ANISOU 2409  OD2 ASP A 329    13955  17675  12485   1429   4005
-1569       O
ATOM   2410  N   LYS A 330      -3.224  -9.390  49.599  1.00117.60
N
ANISOU 2410  N   LYS A 330    14938  17420  12323    913   4097
-2160       N
ATOM   2411  CA  LYS A 330      -2.119 -10.035  50.303  1.00131.23
C
ANISOU 2411  CA  LYS A 330    16739  19126  13998    684   3951
-2170       C
ATOM   2412  C   LYS A 330      -2.359 -11.527  50.509  1.00119.41
C
ANISOU 2412  C   LYS A 330    15081  17713  12578    519   3693
-1995       C
ATOM   2413  O   LYS A 330      -1.477 -12.353  50.246  1.00129.71
O
ANISOU 2413  O   LYS A 330    16407  18913  13964    426   3487
-1966       O
ATOM   2414  CB  LYS A 330      -1.921  -9.357  51.659  1.00114.58
C
ANISOU 2414  CB  LYS A 330    14740  17111  11685    552   4158
-2235       C
ATOM   2415  CG  LYS A 330      -0.894 -10.027  52.549  1.00 84.95
C
ANISOU 2415  CG  LYS A 330    11026  13398   7853    281   4003
-2196       C
ATOM   2416  CD  LYS A 330      -0.768  -9.295  53.868  1.00 82.21
C
ANISOU 2416  CD  LYS A 330    10806  13162   7268    115   4216
-2265       C
ATOM   2417  CE  LYS A 330      -2.063  -9.404  54.661  1.00101.47
C
ANISOU 2417  CE  LYS A 330    13130  15786   9640     87   4348
-2182       C
ATOM   2418  NZ  LYS A 330      -2.276 -10.775  55.211  1.00 84.27
N
ANISOU 2418  NZ  LYS A 330    10772  13750   7498   -106   4103
-1989       N
ATOM   2419  N   THR A 331      -3.550 -11.888  50.984  1.00119.32
N
ANISOU 2419  N   THR A 331    14908  17883  12544    484   3715
-1869       N
ATOM   2420  CA  THR A 331      -3.890 -13.292  51.206  1.00115.86
```

```
C
ANISOU 2420  CA   THR A 331    14326  17529  12167    314   3481
-1697       C
ATOM   2421  C    THR A 331      -3.856 -14.115  49.922  1.00143.32
C
ANISOU 2421  C    THR A 331    17769  20874  15813    356   3265
-1651       C
ATOM   2422  O    THR A 331      -3.276 -15.205  49.890  1.00168.02
O
ANISOU 2422  O    THR A 331    20907  23919  19016    220   3062
-1585       O
ATOM   2423  CB   THR A 331      -5.254 -13.390  51.882  1.00 84.80
C
ANISOU 2423  CB   THR A 331    10222  13830   8169    280   3570
-1574       C
ATOM   2424  OG1 THR A 331      -5.235 -12.589  53.069  1.00 84.22
O
ANISOU 2424  OG1 THR A 331    10217  13856   7925    231   3803
-1639       O
ATOM   2425  CG2 THR A 331      -5.565 -14.833  52.251  1.00100.60
C
ANISOU 2425  CG2 THR A 331    12092  15923  10207     68   3333
-1396       C
ATOM   2426  N    GLY A 332      -4.446 -13.604  48.843  1.00155.61
N
ANISOU 2426  N    GLY A 332    19298  22398  17428    538   3316
-1681       N
ATOM   2427  CA   GLY A 332      -4.470 -14.375  47.608  1.00152.55
C
ANISOU 2427  CA   GLY A 332    18898  21894  17169    541   3119
-1644       C
ATOM   2428  C    GLY A 332      -3.137 -14.412  46.883  1.00144.70
C
ANISOU 2428  C    GLY A 332    18081  20646  16253    576   3050
-1772       C
ATOM   2429  O    GLY A 332      -2.618 -15.489  46.575  1.00 90.14
O
ANISOU 2429  O    GLY A 332    11206  13611   9432    466   2871
-1731       O
ATOM   2430  N    THR A 333      -2.564 -13.245  46.603  1.00180.89
N
ANISOU 2430  N    THR A 333    22782  25142  20806    731   3205
-1922       N
ATOM   2431  CA   THR A 333      -1.453 -13.138  45.665  1.00130.19
C
ANISOU 2431  CA   THR A 333    16511  18492  14462    799   3155
-2043       C
ATOM   2432  C    THR A 333      -0.095 -12.999  46.340  1.00 74.88
C
ANISOU 2432  C    THR A 333     9623  11403   7425    734   3174
-2115       C
ATOM   2433  O    THR A 333       0.879 -13.610  45.889  1.00 74.06
O
ANISOU 2433  O    THR A 333     9586  11136   7417    702   3055
```

```
-2128       O
ATOM   2434  CB  THR A 333      -1.673 -11.952  44.722  1.00118.94
C
ANISOU 2434  CB  THR A 333    15143  17016  13033   1009   3294
-2151       C
ATOM   2435  OG1 THR A 333      -2.527 -10.987  45.349  1.00114.00
O
ANISOU 2435  OG1 THR A 333    14454  16550  12311   1102   3501
-2138       O
ATOM   2436  CG2 THR A 333      -2.317 -12.426  43.424  1.00120.11
C
ANISOU 2436  CG2 THR A 333    15237  17131  13270   1042   3168
-2096       C
ATOM   2437  N   LEU A 334      -0.005 -12.212  47.412  1.00 94.29
N
ANISOU 2437  N   LEU A 334    12108  13974   9743    703   3330
-2153       N
ATOM   2438  CA  LEU A 334       1.272 -11.992  48.084  1.00 75.10
C
ANISOU 2438  CA  LEU A 334     9783  11502   7247    605   3344
-2205       C
ATOM   2439  C   LEU A 334       1.702 -13.159  48.969  1.00 76.76
C
ANISOU 2439  C   LEU A 334     9916  11774   7475    396   3179
-2052       C
ATOM   2440  O   LEU A 334       2.904 -13.334  49.196  1.00 75.24
O
ANISOU 2440  O   LEU A 334     9775  11519   7295    319   3115
-2042       O
ATOM   2441  CB  LEU A 334       1.218 -10.707  48.911  1.00 75.82
C
ANISOU 2441  CB  LEU A 334     9967  11688   7153    607   3583
-2309       C
ATOM   2442  CG  LEU A 334       0.942  -9.420  48.132  1.00 75.53
C
ANISOU 2442  CG  LEU A 334    10036  11566   7097    823   3782
-2458       C
ATOM   2443  CD1 LEU A 334       0.838  -8.224  49.066  1.00 76.67
C
ANISOU 2443  CD1 LEU A 334    10300  11781   7050    805   4051
-2557       C
ATOM   2444  CD2 LEU A 334       2.025  -9.190  47.093  1.00 74.04
C
ANISOU 2444  CD2 LEU A 334     9964  11179   6987    906   3724
-2563       C
ATOM   2445  N   THR A 335       0.767 -13.959  49.481  1.00 76.59
N
ANISOU 2445  N   THR A 335     9761  11883   7457    300   3107
-1914       N
ATOM   2446  CA  THR A 335       1.092 -14.939  50.509  1.00 77.37
C
ANISOU 2446  CA  THR A 335     9787  12066   7544     90   2976
-1756       C
ATOM   2447  C   THR A 335       0.642 -16.335  50.102  1.00 77.60
```

C
ANISOU 2447 C   THR A 335   9721 12041   7723   42   2787
-1612   C
ATOM   2448 O   THR A 335   -0.286 -16.506 49.306 1.00 77.59
O
ANISOU 2448 O   THR A 335   9680 12022   7778   120   2772
-1617   O
ATOM   2449 CB THR A 335   0.443 -14.577 51.853 1.00 78.78
C
ANISOU 2449 CB THR A 335   9916 12480   7535   -45   3088
-1716   C
ATOM   2450 OG1 THR A 335   -0.978 -14.515 51.692 1.00 80.25
O
ANISOU 2450 OG1 THR A 335   10006 12771   7715   26   3156
-1694   O
ATOM   2451 CG2 THR A 335   0.943 -13.229 52.346 1.00 78.90
C
ANISOU 2451 CG2 THR A 335   10069 12531   7377   -41   3294
-1866   C
ATOM   2452 N   LEU A 336   1.331 -17.333 50.665 1.00 78.03
N
ANISOU 2452 N   LEU A 336   9742 12069   7836   -102   2644
-1469   N
ATOM   2453 CA LEU A 336   1.187 -18.716 50.214 1.00 78.33
C
ANISOU 2453 CA LEU A 336   9740 11984   8036   -149   2476
-1339   C
ATOM   2454 C   LEU A 336   -0.216 -19.255 50.460 1.00 88.60
C
ANISOU 2454 C   LEU A 336   10935 13431   9300   -239   2436
-1247   C
ATOM   2455 O   LEU A 336   -0.782 -19.934 49.595 1.00141.67
O
ANISOU 2455 O   LEU A 336   17656 20054 16116   -226   2356
-1225   O
ATOM   2456 CB LEU A 336   2.227 -19.604 50.896 1.00 78.92
C
ANISOU 2456 CB LEU A 336   9791 12009   8185   -271   2358
-1175   C
ATOM   2457 CG LEU A 336   3.633 -19.553 50.297 1.00100.06
C
ANISOU 2457 CG LEU A 336   12550 14482 10986   -169   2351
-1209   C
ATOM   2458 CD1 LEU A 336   4.646 -20.197 51.231 1.00 85.02
C
ANISOU 2458 CD1 LEU A 336   10577 12607   9121   -295   2257
-1007   C
ATOM   2459 CD2 LEU A 336   3.652 -20.223 48.931 1.00151.62
C
ANISOU 2459 CD2 LEU A 336   19161 20745 17705   -48   2310
-1254   C
ATOM   2460 N   ASN A 337   -0.780 -18.987 51.644 1.00 80.35
N
ANISOU 2460 N   ASN A 337   9801 12625   8102   -355   2491

```
-1186       N
ATOM   2461  CA  ASN A 337      -2.016 -19.626  52.113  1.00 96.06
C
ANISOU 2461  CA  ASN A 337    11665  14784  10049   -478   2440
-1058       C
ATOM   2462  C   ASN A 337      -1.841 -21.132  52.334  1.00104.35
C
ANISOU 2462  C   ASN A 337    12685  15756  11208   -640   2246
-874       C
ATOM   2463  O   ASN A 337      -2.662 -21.943  51.902  1.00 82.98
O
ANISOU 2463  O   ASN A 337     9937  13034   8556   -698   2154
-800       O
ATOM   2464  CB  ASN A 337      -3.200 -19.339  51.188  1.00 81.63
C
ANISOU 2464  CB  ASN A 337     9791  12993   8231   -368   2486
-1109       C
ATOM   2465  CG  ASN A 337      -3.502 -17.864  51.081  1.00 81.30
C
ANISOU 2465  CG  ASN A 337     9763  13038   8090   -198   2701
-1255       C
ATOM   2466  OD1 ASN A 337      -3.201 -17.092  51.993  1.00 97.81
O
ANISOU 2466  OD1 ASN A 337    11882  15225  10056   -213   2840
-1307       O
ATOM   2467  ND2 ASN A 337      -4.097 -17.459  49.968  1.00112.60
N
ANISOU 2467  ND2 ASN A 337    13718  16963  12101    -47   2737
-1314       N
ATOM   2468  N   LYS A 338      -0.751 -21.498  53.006  1.00115.24
N
ANISOU 2468  N   LYS A 338    14085  17087  12615   -721   2185
-787       N
ATOM   2469  CA  LYS A 338      -0.711 -22.717  53.816  1.00132.35
C
ANISOU 2469  CA  LYS A 338    16183  19284  14820   -913   2040
-568       C
ATOM   2470  C   LYS A 338      -0.354 -22.284  55.239  1.00 99.57
C
ANISOU 2470  C   LYS A 338    11974  15357  10502  -1058   2075
-497       C
ATOM   2471  O   LYS A 338       0.773 -22.435  55.716  1.00140.78
O
ANISOU 2471  O   LYS A 338    17201  20543  15746  -1113   2022
-407       O
ATOM   2472  CB  LYS A 338       0.261 -23.800  53.248  1.00133.37
C
ANISOU 2472  CB  LYS A 338    16381  19126  15169   -888   1920
-469       C
ATOM   2473  CG  LYS A 338       1.688 -23.363  52.977  1.00133.98
C
ANISOU 2473  CG  LYS A 338    16524  19058  15324   -765   1955
-517       C
ATOM   2474  CD  LYS A 338       2.520 -24.564  52.551  1.00105.30
```

```
C
ANISOU 2474  CD  LYS A 338    12935  15153  11922   -740   1858
-375       C
ATOM   2475  CE  LYS A 338       1.949 -25.215  51.301  1.00105.75
C
ANISOU 2475  CE  LYS A 338    13100  14975  12106   -673   1847
-451       C
ATOM   2476  NZ  LYS A 338       1.823 -24.244  50.177  1.00134.04
N
ANISOU 2476  NZ  LYS A 338    16771  18496  15662   -507   1950
-694       N
ATOM   2477  N   LEU A 339      -1.360 -21.729  55.908  1.00 85.25
N
ANISOU 2477  N   LEU A 339    10097  13786   8510  -1130   2174
-530       N
ATOM   2478  CA  LEU A 339      -1.237 -21.145  57.236  1.00 93.61
C
ANISOU 2478  CA  LEU A 339    11130  15076   9360  -1285   2254
-507       C
ATOM   2479  C   LEU A 339      -0.708 -22.156  58.254  1.00 87.45
C
ANISOU 2479  C   LEU A 339    10283  14362   8580  -1514   2089
-268       C
ATOM   2480  O   LEU A 339      -0.964 -23.360  58.160  1.00 88.05
O
ANISOU 2480  O   LEU A 339    10302  14367   8784  -1577   1938
-105       O
ATOM   2481  CB  LEU A 339      -2.593 -20.604  57.688  1.00116.90
C
ANISOU 2481  CB  LEU A 339    14016  18249  12150  -1313   2401
-561       C
ATOM   2482  CG  LEU A 339      -3.332 -19.637  56.756  1.00 86.24
C
ANISOU 2482  CG  LEU A 339    10158  14338   8272  -1083   2573
-746       C
ATOM   2483  CD1 LEU A 339      -4.725 -19.342  57.295  1.00 87.58
C
ANISOU 2483  CD1 LEU A 339    10219  14744   8314  -1117   2705
-728       C
ATOM   2484  CD2 LEU A 339      -2.560 -18.351  56.531  1.00 90.13
C
ANISOU 2484  CD2 LEU A 339    10788  14757   8701   -949   2740
-940       C
ATOM   2485  N   SER A 340       0.046 -21.654  59.235  1.00 88.05
N
ANISOU 2485  N   SER A 340    10375  14578   8502  -1655   2120
-238       N
ATOM   2486  CA  SER A 340       0.740 -22.497  60.200  1.00 89.37
C
ANISOU 2486  CA  SER A 340    10470  14824   8664  -1874   1957
14       C
ATOM   2487  C   SER A 340       0.718 -21.830  61.569  1.00118.05
C
ANISOU 2487  C   SER A 340    14100  18746  12006  -2118   2039
```

```
24       C
ATOM   2488  O   SER A 340       0.856 -20.608  61.674  1.00117.16
O
ANISOU 2488  O   SER A 340    14088  18700  11726  -2099   2217
-168       O
ATOM   2489  CB  SER A 340       2.191 -22.760  59.779  1.00110.15
C
ANISOU 2489  CB  SER A 340    13121  17274  11458  -1801   1856
101        C
ATOM   2490  OG  SER A 340       2.245 -23.449  58.543  1.00168.16
O
ANISOU 2490  OG  SER A 340    20493  24331  19067  -1594   1799
91       O
ATOM   2491  N   VAL A 341       0.542 -22.641  62.613  1.00143.02
N
ANISOU 2491  N   VAL A 341    17166  22073  15101  -2361   1917
249        N
ATOM   2492  CA  VAL A 341       0.409 -22.174  63.990  1.00114.77
C
ANISOU 2492  CA  VAL A 341    13589  18789  11229  -2644   1983
281        C
ATOM   2493  C   VAL A 341       1.522 -22.786  64.826  1.00119.25
C
ANISOU 2493  C   VAL A 341    14089  19447  11775  -2875   1792
555        C
ATOM   2494  O   VAL A 341       1.681 -24.012  64.850  1.00138.29
O
ANISOU 2494  O   VAL A 341    16392  21787  14365  -2903   1597
804        O
ATOM   2495  CB  VAL A 341      -0.955 -22.547  64.598  1.00 95.15
C
ANISOU 2495  CB  VAL A 341    11032  16475   8644  -2764   2021
319        C
ATOM   2496  CG1 VAL A 341      -1.061 -22.025  66.023  1.00 97.00
C
ANISOU 2496  CG1 VAL A 341    11292  17006   8557  -3068   2115
335        C
ATOM   2497  CG2 VAL A 341      -2.079 -22.018  63.755  1.00 94.15
C
ANISOU 2497  CG2 VAL A 341    10929  16285   8560  -2531   2194
105        C
ATOM   2498  N   ASP A 342       2.284 -21.940  65.514  1.00125.61
N
ANISOU 2498  N   ASP A 342    14959  20409  12357  -3050   1851
523        N
ATOM   2499  CA  ASP A 342       3.136 -22.401  66.602  1.00124.43
C
ANISOU 2499  CA  ASP A 342    14727  20452  12097  -3356   1681
806        C
ATOM   2500  C   ASP A 342       2.325 -22.420  67.895  1.00121.41
C
ANISOU 2500  C   ASP A 342    14336  20358  11438  -3670   1721
857        C
ATOM   2501  O   ASP A 342       1.856 -21.374  68.357  1.00125.23
```

```
O
ANISOU 2501  O   ASP A 342    14949  20981  11652  -3774   1940
636       O
ATOM   2502  CB  ASP A 342       4.374 -21.512  66.740  1.00141.87
C
ANISOU 2502  CB  ASP A 342    17010  22724  14172  -3446   1706
771       C
ATOM   2503  CG  ASP A 342       4.029 -20.043  66.915  1.00145.79
C
ANISOU 2503  CG  ASP A 342    17704  23307  14382  -3497   1977
443       C
ATOM   2504  OD1 ASP A 342       4.696 -19.368  67.728  1.00158.10
O
ANISOU 2504  OD1 ASP A 342    19341  25062  15666  -3779   2009
450       O
ATOM   2505  OD2 ASP A 342       3.092 -19.564  66.243  1.00 96.57
O
ANISOU 2505  OD2 ASP A 342    11552  16946   8194  -3265   2164
190       O
ATOM   2506  N   LYS A 343       2.149 -23.612  68.469  1.00123.72
N
ANISOU 2506  N   LYS A 343    14486  20724  11798  -3818   1528
1147       N
ATOM   2507  CA  LYS A 343       1.172 -23.798  69.537  1.00110.26
C
ANISOU 2507  CA  LYS A 343    12758  19262   9873  -4077   1564
1192       C
ATOM   2508  C   LYS A 343       1.624 -23.179  70.852  1.00129.11
C
ANISOU 2508  C   LYS A 343    15202  21958  11894  -4468   1598
1238       C
ATOM   2509  O   LYS A 343       0.810 -23.057  71.774  1.00170.55
O
ANISOU 2509  O   LYS A 343    20477  27424  16902  -4699   1692
1212       O
ATOM   2510  CB  LYS A 343       0.847 -25.281  69.740  1.00111.59
C
ANISOU 2510  CB  LYS A 343    12770  19408  10221  -4131   1343
1494       C
ATOM   2511  CG  LYS A 343       1.976 -26.137  70.274  1.00111.64
C
ANISOU 2511  CG  LYS A 343    12654  19459  10304  -4299   1089
1868       C
ATOM   2512  CD  LYS A 343       1.562 -27.600  70.259  1.00127.54
C
ANISOU 2512  CD  LYS A 343    14545  21371  12542  -4285    907
2134       C
ATOM   2513  CE  LYS A 343       2.611 -28.495  70.891  1.00118.35
C
ANISOU 2513  CE  LYS A 343    13243  20263  11462  -4452    666
2551       C
ATOM   2514  NZ  LYS A 343       2.230 -29.931  70.773  1.00137.29
N
ANISOU 2514  NZ  LYS A 343    15552  22505  14108  -4406    513
```

```
2802       N
ATOM   2515  N   ASN A 344       2.903 -22.805  70.954  1.00105.17
N
ANISOU 2515  N   ASN A 344    12190  18962   8809  -4563   1524
1316       N
ATOM   2516  CA  ASN A 344       3.404 -22.091  72.124  1.00117.53
C
ANISOU 2516  CA  ASN A 344    13840  20824   9992  -4965   1565
1337       C
ATOM   2517  C   ASN A 344       2.610 -20.819  72.401  1.00147.95
C
ANISOU 2517  C   ASN A 344    17917  24753  13546  -5027   1900
968       C
ATOM   2518  O   ASN A 344       2.316 -20.508  73.561  1.00188.37
O
ANISOU 2518  O   ASN A 344    23114  30131  18328  -5386   1985
965       O
ATOM   2519  CB  ASN A 344       4.888 -21.767  71.934  1.00108.36
C
ANISOU 2519  CB  ASN A 344    12668  19661   8842  -5008   1452
1445       C
ATOM   2520  CG  ASN A 344       5.531 -21.210  73.190  1.00109.84
C
ANISOU 2520  CG  ASN A 344    12920  20187   8627  -5491   1433
1541       C
ATOM   2521  OD1 ASN A 344       5.032 -21.406  74.298  1.00117.62
O
ANISOU 2521  OD1 ASN A 344    13909  21416   9363  -5823   1426
1632       O
ATOM   2522  ND2 ASN A 344       6.649 -20.511  73.021  1.00122.49
N
ANISOU 2522  ND2 ASN A 344    14579  21815  10146  -5557   1422
1525       N
ATOM   2523  N   LEU A 345       2.251 -20.068  71.361  1.00112.56
N
ANISOU 2523  N   LEU A 345    13546  20043   9180  -4685   2107
661       N
ATOM   2524  CA  LEU A 345       1.532 -18.815  71.551  1.00105.63
C
ANISOU 2524  CA  LEU A 345    12885  19199   8051  -4697   2458
320       C
ATOM   2525  C   LEU A 345       0.017 -18.973  71.470  1.00113.75
C
ANISOU 2525  C   LEU A 345    13881  20210   9131  -4539   2622
210       C
ATOM   2526  O   LEU A 345      -0.700 -17.968  71.507  1.00113.40
O
ANISOU 2526  O   LEU A 345    13994  20160   8933  -4474   2943
-62       O
ATOM   2527  CB  LEU A 345       1.997 -17.778  70.526  1.00103.81
C
ANISOU 2527  CB  LEU A 345    12803  18749   7893  -4427   2617
58       C
ATOM   2528  CG  LEU A 345       3.456 -17.338  70.640  1.00104.18
```

```
C
ANISOU 2528  CG  LEU A 345    12917  18840   7827  -4607   2518
116       C
ATOM   2529  CD1 LEU A 345       3.777 -16.256  69.620  1.00121.25
C
ANISOU 2529  CD1 LEU A 345    15245  20780  10046  -4338   2705
-171       C
ATOM   2530  CD2 LEU A 345       3.763 -16.856  72.052  1.00131.79
C
ANISOU 2530  CD2 LEU A 345    16544  22641  10890  -5112   2574
150       C
ATOM   2531  N   VAL A 346      -0.486 -20.202  71.367  1.00105.30
N
ANISOU 2531  N   VAL A 346    12611  19130   8270  -4480   2420
427       N
ATOM   2532  CA  VAL A 346      -1.918 -20.439  71.514  1.00105.76
C
ANISOU 2532  CA  VAL A 346    12612  19245   8327  -4421   2547
377       C
ATOM   2533  C   VAL A 346      -2.323 -20.208  72.962  1.00108.46
C
ANISOU 2533  C   VAL A 346    13019  19886   8306  -4812   2669
396       C
ATOM   2534  O   VAL A 346      -1.733 -20.781  73.887  1.00110.05
O
ANISOU 2534  O   VAL A 346    13172  20272   8371  -5156   2470
634       O
ATOM   2535  CB  VAL A 346      -2.284 -21.857  71.055  1.00105.08
C
ANISOU 2535  CB  VAL A 346    12315  19073   8537  -4304   2284
615       C
ATOM   2536  CG1 VAL A 346      -3.763 -22.119  71.295  1.00115.11
C
ANISOU 2536  CG1 VAL A 346    13511  20449   9776  -4294   2400
590       C
ATOM   2537  CG2 VAL A 346      -1.951 -22.035  69.587  1.00102.61
C
ANISOU 2537  CG2 VAL A 346    11973  18452   8561  -3928   2204
563       C
ATOM   2538  N   GLU A 347      -3.334 -19.367  73.169  1.00117.93
N
ANISOU 2538  N   GLU A 347    14326  21135   9345  -4764   3007
159       N
ATOM   2539  CA  GLU A 347      -3.832 -19.049  74.501  1.00111.94
C
ANISOU 2539  CA  GLU A 347    13660  20640   8231  -5116   3190
133       C
ATOM   2540  C   GLU A 347      -5.261 -19.560  74.613  1.00112.54
C
ANISOU 2540  C   GLU A 347    13592  20796   8371  -5029   3272
169       C
ATOM   2541  O   GLU A 347      -6.076 -19.338  73.712  1.00111.28
O
ANISOU 2541  O   GLU A 347    13377  20495   8409  -4671   3413
```

```
44       O
ATOM   2542  CB  GLU A 347      -3.768 -17.543  74.777  1.00112.92
C
ANISOU 2542  CB  GLU A 347    14069  20758   8077  -5165   3577
-181       C
ATOM   2543  CG  GLU A 347      -2.352 -16.986  74.900  1.00112.96
C
ANISOU 2543  CG  GLU A 347    14238  20730   7951  -5347   3493
-210       C
ATOM   2544  CD  GLU A 347      -1.674 -17.364  76.208  1.00165.34
C
ANISOU 2544  CD  GLU A 347    20887  27568  14365  -5817   3268
-10        C
ATOM   2545  OE1 GLU A 347      -1.314 -18.548  76.380  1.00173.68
O
ANISOU 2545  OE1 GLU A 347    21732  28807  15451  -5997   2973
323        O
ATOM   2546  OE2 GLU A 347      -1.499 -16.474  77.066  1.00157.26
O
ANISOU 2546  OE2 GLU A 347    20088  26498  13166  -5997   3373
-174       O
ATOM   2547  N   VAL A 348      -5.559 -20.235  75.718  1.00129.97
N
ANISOU 2547  N   VAL A 348    15731  23247  10404  -5371   3179
356        N
ATOM   2548  CA  VAL A 348      -6.843 -20.890  75.914  1.00120.37
C
ANISOU 2548  CA  VAL A 348    14351  22141   9241  -5344   3204
443        C
ATOM   2549  C   VAL A 348      -7.636 -20.178  77.001  1.00119.93
C
ANISOU 2549  C   VAL A 348    14419  22294   8853  -5558   3549
301        C
ATOM   2550  O   VAL A 348      -7.092 -19.476  77.859  1.00157.51
O
ANISOU 2550  O   VAL A 348    19392  27018  13439  -5747   3597
188        O
ATOM   2551  CB  VAL A 348      -6.680 -22.386  76.254  1.00115.70
C
ANISOU 2551  CB  VAL A 348    13563  21641   8758  -5539   2811
794        C
ATOM   2552  CG1 VAL A 348      -5.894 -23.093  75.161  1.00130.69
C
ANISOU 2552  CG1 VAL A 348    15363  23288  11004  -5303   2507
926        C
ATOM   2553  CG2 VAL A 348      -6.006 -22.556  77.610  1.00118.05
C
ANISOU 2553  CG2 VAL A 348    13924  22185   8743  -6024   2706
953        C
ATOM   2554  N   PHE A 349      -8.953 -20.365  76.947  1.00118.66
N
ANISOU 2554  N   PHE A 349    14131  22193   8762  -5414   3693
293        N
ATOM   2555  CA  PHE A 349      -9.867 -19.890  77.973  1.00139.14
```

```
C
ANISOU 2555  CA  PHE A 349    16806  24829  11230  -5480   3910
185       C
ATOM   2556  C   PHE A 349     -10.855 -21.014  78.248  1.00143.17
C
ANISOU 2556  C   PHE A 349    17068  25560  11769  -5584   3793
413       C
ATOM   2557  O   PHE A 349     -10.913 -22.001  77.509  1.00178.40
O
ANISOU 2557  O   PHE A 349    21318  30075  16390  -5536   3574
614       O
ATOM   2558  CB  PHE A 349     -10.598 -18.613  77.541  1.00144.81
C
ANISOU 2558  CB  PHE A 349    17647  25343  12030  -5100   4308
-101       C
ATOM   2559  CG  PHE A 349      -9.680 -17.489  77.153  1.00166.54
C
ANISOU 2559  CG  PHE A 349    20649  27846  14783  -4967   4425
-324       C
ATOM   2560  CD1 PHE A 349      -9.327 -17.295  75.827  1.00118.11
C
ANISOU 2560  CD1 PHE A 349    14473  21570   8831  -4669   4421
-382       C
ATOM   2561  CD2 PHE A 349      -9.170 -16.627  78.110  1.00199.34
C
ANISOU 2561  CD2 PHE A 349    25081  31908  18749  -5154   4534
-474       C
ATOM   2562  CE1 PHE A 349      -8.484 -16.262  75.462  1.00117.36
C
ANISOU 2562  CE1 PHE A 349    14610  21242   8741  -4549   4519
-582       C
ATOM   2563  CE2 PHE A 349      -8.325 -15.592  77.751  1.00185.86
C
ANISOU 2563  CE2 PHE A 349    23606  29969  17042  -5049   4629
-668       C
ATOM   2564  CZ  PHE A 349      -7.982 -15.409  76.425  1.00119.48
C
ANISOU 2564  CZ  PHE A 349    15153  21418   8826  -4740   4620
-719       C
ATOM   2565  N   CYS A 350     -11.623 -20.872  79.330  1.00125.01
N
ANISOU 2565  N   CYS A 350    14808  23354   9335  -5724   3925
383       N
ATOM   2566  CA  CYS A 350     -12.540 -21.926  79.772  1.00148.98
C
ANISOU 2566  CA  CYS A 350    17625  26613  12369  -5873   3804
602       C
ATOM   2567  C   CYS A 350     -11.738 -23.181  80.117  1.00132.03
C
ANISOU 2567  C   CYS A 350    15380  24608  10176  -6224   3390
905       C
ATOM   2568  O   CYS A 350     -12.012 -24.280  79.630  1.00124.82
O
ANISOU 2568  O   CYS A 350    14248  23791   9389  -6240   3181
```

```
1145        O
ATOM   2569  CB  CYS A 350     -13.622 -22.221  78.728  1.00144.84
C
ANISOU 2569  CB  CYS A 350    16862  26116  12053  -5561   3889
648       C
ATOM   2570  SG  CYS A 350     -14.668 -20.809  78.305  1.00181.26
S
ANISOU 2570  SG  CYS A 350    21538  30574  16757  -5114   4374
365       S
ATOM   2571  N   LYS A 351     -10.719 -22.989  80.956  1.00128.89
N
ANISOU 2571  N   LYS A 351    15153  24214   9607  -6506   3272
908       N
ATOM   2572  CA  LYS A 351      -9.732 -24.024  81.239  1.00128.48
C
ANISOU 2572  CA  LYS A 351    15022  24265   9528  -6808   2882
1212       C
ATOM   2573  C   LYS A 351     -10.374 -25.335  81.680  1.00127.72
C
ANISOU 2573  C   LYS A 351    14716  24356   9454  -7004   2661
1507       C
ATOM   2574  O   LYS A 351     -11.404 -25.366  82.360  1.00129.67
O
ANISOU 2574  O   LYS A 351    14936  24714   9619  -7067   2779
1466       O
ATOM   2575  CB  LYS A 351      -8.728 -23.525  82.281  1.00155.27
C
ANISOU 2575  CB  LYS A 351    18616  27678  12700  -7115   2810
1168       C
ATOM   2576  CG  LYS A 351      -7.789 -22.466  81.708  1.00141.92
C
ANISOU 2576  CG  LYS A 351    17111  25798  11015  -6965   2926
956       C
ATOM   2577  CD  LYS A 351      -6.900 -21.825  82.756  1.00147.11
C
ANISOU 2577  CD  LYS A 351    17982  26476  11436  -7282   2887
879       C
ATOM   2578  CE  LYS A 351      -5.975 -20.802  82.111  1.00132.47
C
ANISOU 2578  CE  LYS A 351    16305  24427   9601  -7131   2991
679       C
ATOM   2579  NZ  LYS A 351      -4.881 -21.453  81.339  1.00125.78
N
ANISOU 2579  NZ  LYS A 351    15322  23589   8879  -7118   2705
920       N
ATOM   2580  N   GLY A 352      -9.726 -26.422  81.273  1.00126.50
N
ANISOU 2580  N   GLY A 352    14421  24219   9424  -7093   2343
1817       N
ATOM   2581  CA  GLY A 352     -10.234 -27.778  81.251  1.00126.72
C
ANISOU 2581  CA  GLY A 352    14247  24295   9605  -7158   2083
2106       C
ATOM   2582  C   GLY A 352     -10.020 -28.263  79.833  1.00152.72
```

```
C
ANISOU 2582  C   GLY A 352    17456  27263  13306  -6765   1906
2132       C
ATOM   2583  O   GLY A 352      -9.781 -29.448  79.578  1.00150.84
O
ANISOU 2583  O   GLY A 352    17108  26924  13282  -6777   1593
2394       O
ATOM   2584  N   VAL A 353     -10.112 -27.314  78.900  1.00173.34
N
ANISOU 2584  N   VAL A 353    20139  29703  16021  -6416   2128
1852       N
ATOM   2585  CA  VAL A 353      -9.479 -27.423  77.593  1.00163.03
C
ANISOU 2585  CA  VAL A 353    18829  28075  15039  -6073   1993
1821       C
ATOM   2586  C   VAL A 353      -8.010 -27.032  77.721  1.00145.08
C
ANISOU 2586  C   VAL A 353    16678  25727  12719  -6147   1904
1831       C
ATOM   2587  O   VAL A 353      -7.597 -26.386  78.689  1.00137.82
O
ANISOU 2587  O   VAL A 353    15874  24997  11494  -6425   2014
1785       O
ATOM   2588  CB  VAL A 353     -10.203 -26.560  76.545  1.00150.80
C
ANISOU 2588  CB  VAL A 353    17295  26393  13611  -5683   2261
1538       C
ATOM   2589  CG1 VAL A 353     -11.668 -26.965  76.447  1.00118.71
C
ANISOU 2589  CG1 VAL A 353    13074  22446   9584  -5632   2337
1570       C
ATOM   2590  CG2 VAL A 353     -10.069 -25.084  76.888  1.00167.06
C
ANISOU 2590  CG2 VAL A 353    19529  28514  15431  -5668   2614
1255       C
ATOM   2591  N   GLU A 354      -7.200 -27.430  76.743  1.00117.08
N
ANISOU 2591  N   GLU A 354    13109  21909   9466  -5916   1706
1898       N
ATOM   2592  CA  GLU A 354      -5.768 -27.201  76.859  1.00135.93
C
ANISOU 2592  CA  GLU A 354    15570  24243  11834  -5993   1585
1967       C
ATOM   2593  C   GLU A 354      -5.180 -26.881  75.489  1.00162.36
C
ANISOU 2593  C   GLU A 354    18956  27270  15462  -5601   1584
1826       C
ATOM   2594  O   GLU A 354      -5.761 -27.195  74.446  1.00170.81
O
ANISOU 2594  O   GLU A 354    19975  28140  16785  -5303   1584
1760       O
ATOM   2595  CB  GLU A 354      -5.075 -28.423  77.494  1.00118.77
C
ANISOU 2595  CB  GLU A 354    13286  22131   9709  -6251   1244
```

```
2364       C
ATOM   2596  CG  GLU A 354      -3.580 -28.277  77.757  1.00125.19
C
ANISOU 2596  CG  GLU A 354    14127  22950  10488  -6377   1093
2513       C
ATOM   2597  CD  GLU A 354      -3.049 -29.332  78.707  1.00178.20
C
ANISOU 2597  CD  GLU A 354    20720  29824  17163  -6707    802
2929       C
ATOM   2598  OE1 GLU A 354      -3.170 -30.534  78.387  1.00183.92
O
ANISOU 2598  OE1 GLU A 354    21323  30402  18155  -6616    597
3170       O
ATOM   2599  OE2 GLU A 354      -2.512 -28.960  79.772  1.00198.36
O
ANISOU 2599  OE2 GLU A 354    23310  32647  19411  -7071    783
3024       O
ATOM   2600  N   LYS A 355      -4.012 -26.230  75.524  1.00177.35
N
ANISOU 2600  N   LYS A 355    20951  29142  17292  -5633   1583
1785       N
ATOM   2601  CA  LYS A 355      -3.375 -25.680  74.329  1.00151.51
C
ANISOU 2601  CA  LYS A 355    17743  25598  14225  -5292   1628
1614       C
ATOM   2602  C   LYS A 355      -3.163 -26.739  73.252  1.00144.54
C
ANISOU 2602  C   LYS A 355    16753  24429  13736  -5024   1408
1765       C
ATOM   2603  O   LYS A 355      -3.287 -26.454  72.055  1.00152.03
O
ANISOU 2603  O   LYS A 355    17737  25138  14890  -4685   1486
1581       O
ATOM   2604  CB  LYS A 355      -2.045 -25.033  74.718  1.00134.82
C
ANISOU 2604  CB  LYS A 355    15720  23546  11958  -5452   1600
1631       C
ATOM   2605  CG  LYS A 355      -2.194 -23.844  75.663  1.00141.76
C
ANISOU 2605  CG  LYS A 355    16769  24665  12427  -5720   1860
1428       C
ATOM   2606  CD  LYS A 355      -0.896 -23.540  76.401  1.00144.97
C
ANISOU 2606  CD  LYS A 355    17234  25228  12619  -6043   1749
1561       C
ATOM   2607  CE  LYS A 355       0.174 -22.968  75.485  1.00121.19
C
ANISOU 2607  CE  LYS A 355    14281  22014   9751  -5819   1736
1466       C
ATOM   2608  NZ  LYS A 355       0.005 -21.501  75.293  1.00112.70
N
ANISOU 2608  NZ  LYS A 355    13438  20905   8476  -5753   2075
1080       N
ATOM   2609  N   ASP A 356      -2.841 -27.967  73.659  1.00146.11
```

```
N
ANISOU 2609  N   ASP A 356    16835  24639  14040  -5177   1144
2103       N
ATOM   2610  CA  ASP A 356      -2.646 -29.064  72.718  1.00141.16
C
ANISOU 2610  CA  ASP A 356    16136  23719  13780  -4951    957
2260       C
ATOM   2611  C   ASP A 356      -3.949 -29.604  72.139  1.00118.62
C
ANISOU 2611  C   ASP A 356    13250  20774  11047  -4821    992
2189       C
ATOM   2612  O   ASP A 356      -3.913 -30.292  71.113  1.00114.72
O
ANISOU 2612  O   ASP A 356    12749  19995  10842  -4596    902
2223       O
ATOM   2613  CB  ASP A 356      -1.875 -30.197  73.400  1.00168.28
C
ANISOU 2613  CB  ASP A 356    19464  27187  17289  -5159    686
2668       C
ATOM   2614  CG  ASP A 356      -1.280 -31.176  72.408  1.00166.00
C
ANISOU 2614  CG  ASP A 356    19138  26545  17389  -4903    528
2832       C
ATOM   2615  OD1 ASP A 356      -1.267 -32.391  72.700  1.00169.56
O
ANISOU 2615  OD1 ASP A 356    19507  26942  17974  -4998    344
3137       O
ATOM   2616  OD2 ASP A 356      -0.819 -30.729  71.337  1.00158.66
O
ANISOU 2616  OD2 ASP A 356    18273  25380  16628  -4609    602
2655       O
ATOM   2617  N   GLN A 357      -5.093 -29.312  72.756  1.00112.55
N
ANISOU 2617  N   GLN A 357    12463  20241  10059  -4968   1129
2097       N
ATOM   2618  CA  GLN A 357      -6.357 -29.896  72.321  1.00113.56
C
ANISOU 2618  CA  GLN A 357    12529  20340  10280  -4897   1139
2082       C
ATOM   2619  C   GLN A 357      -7.250 -28.928  71.555  1.00109.25
C
ANISOU 2619  C   GLN A 357    12014  19780   9716  -4653   1390
1770       C
ATOM   2620  O   GLN A 357      -8.122 -29.381  70.805  1.00108.77
O
ANISOU 2620  O   GLN A 357    11900  19632   9797  -4523   1376
1751       O
ATOM   2621  CB  GLN A 357      -7.125 -30.435  73.538  1.00139.42
C
ANISOU 2621  CB  GLN A 357    15720  23904  13349  -5234   1097
2256       C
ATOM   2622  CG  GLN A 357      -8.428 -31.155  73.214  1.00144.62
C
ANISOU 2622  CG  GLN A 357    16294  24573  14084  -5220   1077
```

```
2291       C
ATOM   2623  CD  GLN A 357      -9.060 -31.802  74.430  1.00165.79
C
ANISOU 2623  CD  GLN A 357    18889  27529  16575  -5570   1007
2497       C
ATOM   2624  OE1 GLN A 357      -8.779 -31.423  75.567  1.00171.45
O
ANISOU 2624  OE1 GLN A 357    19617  28485  17040  -5822   1050
2542       O
ATOM   2625  NE2 GLN A 357      -9.922 -32.785  74.196  1.00174.98
N
ANISOU 2625  NE2 GLN A 357    19976  28666  17841  -5612    898
2624       N
ATOM   2626  N   VAL A 358      -7.047 -27.617  71.696  1.00108.82
N
ANISOU 2626  N   VAL A 358    12049  19805   9494  -4594   1620
1538       N
ATOM   2627  CA  VAL A 358      -7.894 -26.670  70.980  1.00107.82
C
ANISOU 2627  CA  VAL A 358    11944  19659   9365  -4342   1875
1268       C
ATOM   2628  C   VAL A 358      -7.643 -26.686  69.475  1.00105.56
C
ANISOU 2628  C   VAL A 358    11683  19064   9360  -4004   1830
1168       C
ATOM   2629  O   VAL A 358      -8.523 -26.291  68.702  1.00109.24
O
ANISOU 2629  O   VAL A 358    12118  19501   9887  -3795   1965
1023       O
ATOM   2630  CB  VAL A 358      -7.684 -25.251  71.541  1.00117.08
C
ANISOU 2630  CB  VAL A 358    13239  20962  10283  -4370   2157
1045       C
ATOM   2631  CG1 VAL A 358      -8.119 -25.190  72.993  1.00161.43
C
ANISOU 2631  CG1 VAL A 358    18848  26892  15595  -4715   2247
1113       C
ATOM   2632  CG2 VAL A 358      -6.229 -24.831  71.400  1.00108.00
C
ANISOU 2632  CG2 VAL A 358    12211  19677   9149  -4353   2096
1011       C
ATOM   2633  N   LEU A 359      -6.460 -27.129  69.039  1.00135.05
N
ANISOU 2633  N   LEU A 359    15468  22578  13267  -3948   1649
1255       N
ATOM   2634  CA  LEU A 359      -6.235 -27.425  67.625  1.00106.48
C
ANISOU 2634  CA  LEU A 359    11876  18650   9929  -3666   1575
1200       C
ATOM   2635  C   LEU A 359      -7.163 -28.523  67.110  1.00117.16
C
ANISOU 2635  C   LEU A 359    13151  19935  11430  -3666   1450
1316       C
ATOM   2636  O   LEU A 359      -7.641 -28.457  65.971  1.00149.79
```

```
O
ANISOU 2636  O   LEU A 359    17294  23921  15700  -3456   1485
1197       O
ATOM   2637  CB  LEU A 359      -4.773 -27.817  67.403  1.00107.08
C
ANISOU 2637  CB  LEU A 359    12007  18516  10161  -3633   1413
1314       C
ATOM   2638  CG  LEU A 359      -3.711 -26.823  67.879  1.00117.25
C
ANISOU 2638  CG  LEU A 359    13370  19875  11303  -3671   1497
1235       C
ATOM   2639  CD1 LEU A 359      -2.315 -27.340  67.570  1.00127.16
C
ANISOU 2639  CD1 LEU A 359    14636  20930  12749  -3620   1320
1396       C
ATOM   2640  CD2 LEU A 359      -3.927 -25.455  67.255  1.00116.89
C
ANISOU 2640  CD2 LEU A 359    13420  19802  11191  -3454   1747
915       C
ATOM   2641  N   LEU A 360      -7.423 -29.543  67.932  1.00108.66
N
ANISOU 2641  N   LEU A 360    12002  18969  10314  -3922   1298
1555       N
ATOM   2642  CA  LEU A 360      -8.316 -30.631  67.532  1.00105.43
C
ANISOU 2642  CA  LEU A 360    11535  18506  10018  -3972   1174
1678       C
ATOM   2643  C   LEU A 360      -9.728 -30.142  67.227  1.00118.72
C
ANISOU 2643  C   LEU A 360    13135  20364  11610  -3917   1328
1544       C
ATOM   2644  O   LEU A 360     -10.335 -30.566  66.237  1.00121.17
O
ANISOU 2644  O   LEU A 360    13433  20553  12053  -3819   1279
1526       O
ATOM   2645  CB  LEU A 360      -8.349 -31.707  68.618  1.00142.83
C
ANISOU 2645  CB  LEU A 360    16211  23361  14695  -4281   1002
1963       C
ATOM   2646  CG  LEU A 360      -7.956 -33.122  68.188  1.00109.53
C
ANISOU 2646  CG  LEU A 360    12034  18867  10715  -4310    778
2181       C
ATOM   2647  CD1 LEU A 360      -7.874 -34.053  69.386  1.00137.77
C
ANISOU 2647  CD1 LEU A 360    15551  22576  14220  -4614    621
2478       C
ATOM   2648  CD2 LEU A 360      -8.946 -33.659  67.170  1.00107.70
C
ANISOU 2648  CD2 LEU A 360    11814  18511  10596  -4243    757
2129       C
ATOM   2649  N   PHE A 361     -10.271 -29.252  68.062  1.00133.54
N
ANISOU 2649  N   PHE A 361    14954  22528  13255  -3985   1523
```

```
1459        N
ATOM   2650  CA  PHE A 361     -11.578 -28.668  67.769  1.00144.49
C
ANISOU 2650  CA  PHE A 361    16239  24089  14572  -3889   1707
1347        C
ATOM   2651  C   PHE A 361     -11.580 -27.911  66.446  1.00146.01
C
ANISOU 2651  C   PHE A 361    16473  24104  14899  -3562   1815
1145        C
ATOM   2652  O   PHE A 361     -12.542 -28.000  65.673  1.00156.94
O
ANISOU 2652  O   PHE A 361    17766  25515  16349  -3467   1829
1136        O
ATOM   2653  CB  PHE A 361     -12.003 -27.739  68.907  1.00141.60
C
ANISOU 2653  CB  PHE A 361    15837  24022  13942  -3989   1951
1273        C
ATOM   2654  CG  PHE A 361     -12.034 -28.399  70.257  1.00138.13
C
ANISOU 2654  CG  PHE A 361    15360  23789  13336  -4334   1860
1464        C
ATOM   2655  CD1 PHE A 361     -11.572 -27.729  71.378  1.00117.82
C
ANISOU 2655  CD1 PHE A 361    12857  21376  10534  -4491   1994
1418        C
ATOM   2656  CD2 PHE A 361     -12.538 -29.681  70.408  1.00141.41
C
ANISOU 2656  CD2 PHE A 361    15684  24239  13805  -4525   1643
1692        C
ATOM   2657  CE1 PHE A 361     -11.600 -28.330  72.622  1.00131.71
C
ANISOU 2657  CE1 PHE A 361    14581  23340  12122  -4832   1903
1603        C
ATOM   2658  CE2 PHE A 361     -12.570 -30.288  71.650  1.00136.72
C
ANISOU 2658  CE2 PHE A 361    15054  23839  13055  -4848   1555
1880        C
ATOM   2659  CZ  PHE A 361     -12.100 -29.612  72.758  1.00138.97
C
ANISOU 2659  CZ  PHE A 361    15395  24295  13112  -5001   1680
1839        C
ATOM   2660  N   ALA A 362     -10.517 -27.155  66.169  1.00140.19
N
ANISOU 2660  N   ALA A 362    15866  23203  14196  -3404   1884
996        N
ATOM   2661  CA  ALA A 362     -10.376 -26.517  64.864  1.00132.39
C
ANISOU 2661  CA  ALA A 362    14934  22016  13352  -3102   1958
817        C
ATOM   2662  C   ALA A 362     -10.323 -27.534  63.726  1.00109.92
C
ANISOU 2662  C   ALA A 362    12108  18926  10730  -3049   1744
891        C
ATOM   2663  O   ALA A 362     -10.961 -27.344  62.684  1.00100.25
```

```
O
ANISOU 2663  O   ALA A 362    10851  17653   9585  -2890   1778
815       O
ATOM   2664  CB  ALA A 362      -9.128 -25.636  64.854  1.00148.73
C
ANISOU 2664  CB  ALA A 362    17151  23951  15409  -2984   2045
665       C
ATOM   2665  N   ALA A 363      -9.565 -28.617  63.907  1.00136.16
N
ANISOU 2665  N   ALA A 363    15493  22089  14153  -3186   1533
1049       N
ATOM   2666  CA  ALA A 363      -9.565 -29.718  62.946  1.00134.28
C
ANISOU 2666  CA  ALA A 363    15305  21602  14112  -3180   1350
1133       C
ATOM   2667  C   ALA A 363     -10.939 -30.366  62.801  1.00132.71
C
ANISOU 2667  C   ALA A 363    14997  21551  13877  -3319   1293
1231       C
ATOM   2668  O   ALA A 363     -11.354 -30.710  61.687  1.00145.85
O
ANISOU 2668  O   ALA A 363    16690  23079  15647  -3256   1237
1201       O
ATOM   2669  CB  ALA A 363      -8.528 -30.762  63.352  1.00153.33
C
ANISOU 2669  CB  ALA A 363    17797  23826  16635  -3305   1170
1316       C
ATOM   2670  N   MET A 364     -11.654 -30.551  63.912  1.00116.37
N
ANISOU 2670  N   MET A 364    12803  19769  11643  -3532   1304
1356       N
ATOM   2671  CA  MET A 364     -13.037 -31.022  63.847  1.00105.29
C
ANISOU 2671  CA  MET A 364    11263  18564  10176  -3669   1272
1454       C
ATOM   2672  C   MET A 364     -13.928 -30.100  63.021  1.00104.86
C
ANISOU 2672  C   MET A 364    11109  18630  10102  -3473   1430
1317       C
ATOM   2673  O   MET A 364     -14.715 -30.573  62.194  1.00105.92
O
ANISOU 2673  O   MET A 364    11192  18766  10285  -3505   1346
1367       O
ATOM   2674  CB  MET A 364     -13.606 -31.192  65.256  1.00126.47
C
ANISOU 2674  CB  MET A 364    13821  21562  12669  -3913   1296
1594       C
ATOM   2675  CG  MET A 364     -12.988 -32.339  66.040  1.00158.92
C
ANISOU 2675  CG  MET A 364    17995  25588  16799  -4157   1100
1798       C
ATOM   2676  SD  MET A 364     -13.643 -32.460  67.715  1.00159.11
S
ANISOU 2676  SD  MET A 364    17878  26003  16573  -4461   1135
```

1955 S
ATOM 2677 CE MET A 364 -12.277 -33.253 68.557 1.00149.59
C
ANISOU 2677 CE MET A 364 16786 24640 15410 -4630 951
2137 C
ATOM 2678 N ALA A 365 -13.812 -28.788 63.207 1.00104.16
N
ANISOU 2678 N ALA A 365 10997 18640 9939 -3278 1658
1155 N
ATOM 2679 CA ALA A 365 -14.661 -27.857 62.473 1.00104.02
C
ANISOU 2679 CA ALA A 365 10868 18740 9913 -3067 1829
1053 C
ATOM 2680 C ALA A 365 -14.123 -27.510 61.083 1.00102.12
C
ANISOU 2680 C ALA A 365 10742 18228 9832 -2829 1810
909 C
ATOM 2681 O ALA A 365 -14.489 -26.467 60.526 1.00101.67
O
ANISOU 2681 O ALA A 365 10628 18229 9774 -2602 1985
793 O
ATOM 2682 CB ALA A 365 -14.859 -26.578 63.291 1.00118.89
C
ANISOU 2682 CB ALA A 365 12692 20833 11647 -2955 2123
945 C
ATOM 2683 N SER A 366 -13.269 -28.355 60.514 1.00119.00
N
ANISOU 2683 N SER A 366 13042 20065 12108 -2873 1618
921 N
ATOM 2684 CA SER A 366 -12.750 -28.176 59.166 1.00155.52
C
ANISOU 2684 CA SER A 366 17792 24418 16882 -2681 1588
792 C
ATOM 2685 C SER A 366 -13.231 -29.319 58.282 1.00146.78
C
ANISOU 2685 C SER A 366 16717 23198 15856 -2827 1391
894 C
ATOM 2686 O SER A 366 -13.278 -30.473 58.720 1.00171.62
O
ANISOU 2686 O SER A 366 19895 26308 19007 -3065 1237
1047 O
ATOM 2687 CB SER A 366 -11.220 -28.130 59.168 1.00191.81
C
ANISOU 2687 CB SER A 366 22579 28723 21577 -2590 1565
702 C
ATOM 2688 OG SER A 366 -10.738 -27.010 59.889 1.00195.29
O
ANISOU 2688 OG SER A 366 23017 29262 21921 -2479 1747
592 O
ATOM 2689 N ARG A 367 -13.594 -29.001 57.040 1.00109.10
N
ANISOU 2689 N ARG A 367 11949 18369 11135 -2705 1400
815 N
ATOM 2690 CA ARG A 367 -13.851 -30.048 56.060 1.00100.31

```
C
ANISOU 2690  CA  ARG A 367    10932  17092  10090  -2858   1220
875       C
ATOM   2691  C   ARG A 367     -12.565 -30.810  55.753  1.00107.90
C
ANISOU 2691  C   ARG A 367    12151  17643  11201  -2863   1126
829       C
ATOM   2692  O   ARG A 367     -11.464 -30.252  55.773  1.00115.98
O
ANISOU 2692  O   ARG A 367    13272  18494  12303  -2666   1206
707       O
ATOM   2693  CB  ARG A 367     -14.467 -29.468  54.786  1.00100.07
C
ANISOU 2693  CB  ARG A 367    10853  17107  10064  -2740   1250
804       C
ATOM   2694  CG  ARG A 367     -15.871 -28.927  55.018  1.00127.53
C
ANISOU 2694  CG  ARG A 367    14043  21000  13413  -2759   1325
916       C
ATOM   2695  CD  ARG A 367     -16.826 -29.212  53.864  1.00128.63
C
ANISOU 2695  CD  ARG A 367    14112  21235  13528  -2867   1216
996       C
ATOM   2696  NE  ARG A 367     -16.613 -28.354  52.702  1.00169.13
N
ANISOU 2696  NE  ARG A 367    19278  26267  18715  -2635   1283
862       N
ATOM   2697  CZ  ARG A 367     -17.568 -28.027  51.837  1.00134.86
C
ANISOU 2697  CZ  ARG A 367    14781  22129  14329  -2637   1264
940       C
ATOM   2698  NH1 ARG A 367     -18.762 -28.597  51.922  1.00163.39
N
ANISOU 2698  NH1 ARG A 367    18206  26036  17840  -2883   1159
1152       N
ATOM   2699  NH2 ARG A 367     -17.297 -27.229  50.812  1.00101.75
N
ANISOU 2699  NH2 ARG A 367    10632  17838  10192  -2423   1324
823       N
ATOM   2700  N   VAL A 368     -12.715 -32.104  55.472  1.00126.30
N
ANISOU 2700  N   VAL A 368    14597  19818  13573  -3093    967
939       N
ATOM   2701  CA  VAL A 368     -11.580 -33.020  55.411  1.00137.79
C
ANISOU 2701  CA  VAL A 368    16284  20890  15180  -3122    894
953       C
ATOM   2702  C   VAL A 368     -11.297 -33.551  54.008  1.00173.00
C
ANISOU 2702  C   VAL A 368    20973  25010  19750  -3119    848
862       C
ATOM   2703  O   VAL A 368     -10.207 -34.106  53.786  1.00195.91
O
ANISOU 2703  O   VAL A 368    24079  27550  22808  -3061    843
```

```
839       O
ATOM   2704  CB  VAL A 368     -11.767 -34.196  56.397  1.00121.00
C
ANISOU 2704  CB  VAL A 368    14156  18786  13032  -3391    776
1166       C
ATOM   2705  CG1 VAL A 368     -12.860 -35.142  55.911  1.00129.75
C
ANISOU 2705  CG1 VAL A 368    15291  19935  14075  -3672    654
1268       C
ATOM   2706  CG2 VAL A 368     -10.449 -34.927  56.673  1.00102.21
C
ANISOU 2706  CG2 VAL A 368    11958  16054  10822  -3359    741
1221       C
ATOM   2707  N   GLU A 369     -12.203 -33.374  53.045  1.00134.14
N
ANISOU 2707  N   GLU A 369    16024  20191  14752  -3178    827
816       N
ATOM   2708  CA  GLU A 369     -12.006 -33.919  51.704  1.00131.07
C
ANISOU 2708  CA  GLU A 369    15877  19493  14432  -3231    784
727       C
ATOM   2709  C   GLU A 369     -11.701 -32.822  50.691  1.00120.91
C
ANISOU 2709  C   GLU A 369    14605  18165  13171  -2978    881
535       C
ATOM   2710  O   GLU A 369     -10.557 -32.701  50.246  1.00130.72
O
ANISOU 2710  O   GLU A 369    16025  19093  14551  -2796    946
405       O
ATOM   2711  CB  GLU A 369     -13.222 -34.739  51.269  1.00145.58
C
ANISOU 2711  CB  GLU A 369    17719  21452  16144  -3567    655
840       C
ATOM   2712  CG  GLU A 369     -13.049 -35.380  49.902  1.00175.80
C
ANISOU 2712  CG  GLU A 369    21837  24951  20008  -3683    615
744       C
ATOM   2713  CD  GLU A 369     -13.801 -36.686  49.763  1.00190.18
C
ANISOU 2713  CD  GLU A 369    23788  26730  21742  -4083    483
875       C
ATOM   2714  OE1 GLU A 369     -13.143 -37.737  49.610  1.00188.06
O
ANISOU 2714  OE1 GLU A 369    23814  26060  21578  -4184    478
869       O
ATOM   2715  OE2 GLU A 369     -15.048 -36.664  49.807  1.00192.88
O
ANISOU 2715  OE2 GLU A 369    23940  27435  21911  -4297    394
993       O
ATOM   2716  N   ASN A 370     -12.686 -32.014  50.308  1.00122.00
N
ANISOU 2716  N   ASN A 370    14551  18617  13188  -2955    898
529       N
ATOM   2717  CA  ASN A 370     -12.438 -30.834  49.487  1.00120.77
```

```
C
ANISOU 2717  CA  ASN A 370    14373  18463  13052  -2693   1001
368       C
ATOM   2718  C   ASN A 370     -12.361 -29.649  50.438  1.00101.12
C
ANISOU 2718  C   ASN A 370    11671  16213  10539  -2455   1142
350       C
ATOM   2719  O   ASN A 370     -13.368 -29.252  51.033  1.00 97.47
O
ANISOU 2719  O   ASN A 370    10964  16107   9963  -2492   1170
460       O
ATOM   2720  CB  ASN A 370     -13.529 -30.645  48.437  1.00149.08
C
ANISOU 2720  CB  ASN A 370    17879  22237  16528  -2804    942
397       C
ATOM   2721  CG  ASN A 370     -13.168 -29.591  47.405  1.00166.72
C
ANISOU 2721  CG  ASN A 370    20144  24404  18798  -2556   1031
235       C
ATOM   2722  OD1 ASN A 370     -13.839 -28.566  47.285  1.00189.92
O
ANISOU 2722  OD1 ASN A 370    22858  27629  21672  -2419   1096
260       O
ATOM   2723  ND2 ASN A 370     -12.105 -29.844  46.649  1.00138.75
N
ANISOU 2723  ND2 ASN A 370    16878  20475  15365  -2491   1046
77       N
ATOM   2724  N   GLN A 371     -11.167 -29.082  50.572  1.00 99.81
N
ANISOU 2724  N   GLN A 371    11604  15847  10471  -2222   1244
212       N
ATOM   2725  CA  GLN A 371     -10.800 -28.326  51.757  1.00 93.96
C
ANISOU 2725  CA  GLN A 371    10745  15251   9704  -2083   1362
205       C
ATOM   2726  C   GLN A 371     -10.528 -26.869  51.420  1.00 92.41
C
ANISOU 2726  C   GLN A 371    10506  15103   9503  -1794   1528
45       C
ATOM   2727  O   GLN A 371      -9.866 -26.562  50.424  1.00 91.17
O
ANISOU 2727  O   GLN A 371    10493  14719   9429  -1651   1550
-95       O
ATOM   2728  CB  GLN A 371      -9.559 -28.934  52.414  1.00 97.66
C
ANISOU 2728  CB  GLN A 371    11359  15473  10276  -2096   1333
217       C
ATOM   2729  CG  GLN A 371      -9.718 -30.391  52.818  1.00131.56
C
ANISOU 2729  CG  GLN A 371    15714  19679  14594  -2364   1186
388       C
ATOM   2730  CD  GLN A 371      -8.445 -30.977  53.402  1.00129.15
C
ANISOU 2730  CD  GLN A 371    15534  19120  14416  -2346   1164
```

```
434       C
ATOM   2731  OE1 GLN A 371      -8.393 -31.332  54.579  1.00 95.72
O
ANISOU 2731  OE1 GLN A 371    11220  15005  10147  -2459   1129
577       O
ATOM   2732  NE2 GLN A 371      -7.410 -31.085  52.575  1.00128.80
N
ANISOU 2732  NE2 GLN A 371    15679  18735  14523  -2206   1189
330       N
ATOM   2733  N   ASP A 372     -11.049 -25.977  52.259  1.00118.93
N
ANISOU 2733  N   ASP A 372    13681  18747  12759  -1713   1661
66       N
ATOM   2734  CA  ASP A 372     -10.521 -24.625  52.318  1.00106.50
C
ANISOU 2734  CA  ASP A 372    12112  17176  11179  -1449   1850
-89       C
ATOM   2735  C   ASP A 372      -9.076 -24.673  52.800  1.00114.17
C
ANISOU 2735  C   ASP A 372    13246  17919  12215  -1406   1860
-172       C
ATOM   2736  O   ASP A 372      -8.670 -25.591  53.518  1.00128.29
O
ANISOU 2736  O   ASP A 372    15072  19650  14023  -1572   1757
-68       O
ATOM   2737  CB  ASP A 372     -11.371 -23.762  53.254  1.00115.47
C
ANISOU 2737  CB  ASP A 372    13045  18641  12186  -1398   2021
-43       C
ATOM   2738  CG  ASP A 372     -11.154 -22.273  53.046  1.00126.32
C
ANISOU 2738  CG  ASP A 372    14425  20026  13545  -1117   2245
-200       C
ATOM   2739  OD1 ASP A 372     -10.478 -21.891  52.068  1.00134.39
O
ANISOU 2739  OD1 ASP A 372    15578  20832  14651   -967   2248
-333       O
ATOM   2740  OD2 ASP A 372     -11.668 -21.480  53.863  1.00122.06
O
ANISOU 2740  OD2 ASP A 372    13769  19702  12907  -1050   2435
-191       O
ATOM   2741  N   ALA A 373      -8.288 -23.683  52.377  1.00127.05
N
ANISOU 2741  N   ALA A 373    14969  19425  13880  -1186   1979
-341       N
ATOM   2742  CA  ALA A 373      -6.852 -23.715  52.643  1.00138.33
C
ANISOU 2742  CA  ALA A 373    16544  20636  15378  -1143   1975
-408       C
ATOM   2743  C   ALA A 373      -6.557 -23.759  54.140  1.00130.26
C
ANISOU 2743  C   ALA A 373    15469  19760  14264  -1265   1999
-323       C
ATOM   2744  O   ALA A 373      -5.701 -24.530  54.591  1.00114.54
```

```
O
ANISOU 2744  O   ALA A 373    13540  17643  12336  -1367   1894
-238        O
ATOM   2745  CB  ALA A 373      -6.174 -22.506  51.997  1.00129.37
C
ANISOU 2745  CB  ALA A 373    15499  19393  14264   -901   2115
-603        C
ATOM   2746  N   ILE A 374      -7.258 -22.939  54.923  1.00 88.73
N
ANISOU 2746  N   ILE A 374    10094  14766   8853  -1258   2147
-332        N
ATOM   2747  CA  ILE A 374      -7.156 -23.002  56.378  1.00118.41
C
ANISOU 2747  CA  ILE A 374    13803  18700  12487  -1420   2176
-246        C
ATOM   2748  C   ILE A 374      -7.684 -24.328  56.928  1.00107.76
C
ANISOU 2748  C   ILE A 374    12373  17431  11142  -1664   2002
-37        C
ATOM   2749  O   ILE A 374      -7.096 -24.905  57.851  1.00 91.74
O
ANISOU 2749  O   ILE A 374    10358  15408   9091  -1825   1920
74        O
ATOM   2750  CB  ILE A 374      -7.872 -21.783  56.998  1.00 90.39
C
ANISOU 2750  CB  ILE A 374    10175  15395   8772  -1348   2418
-323        C
ATOM   2751  CG1 ILE A 374      -7.561 -21.664  58.490  1.00 91.45
C
ANISOU 2751  CG1 ILE A 374    10311  15690   8747  -1524   2480
-279        C
ATOM   2752  CG2 ILE A 374      -9.375 -21.818  56.737  1.00 91.60
C
ANISOU 2752  CG2 ILE A 374    10156  15748   8899  -1336   2457
-246        C
ATOM   2753  CD1 ILE A 374      -7.922 -20.316  59.061  1.00 91.95
C
ANISOU 2753  CD1 ILE A 374    10381  15912   8645  -1437   2768
-409        C
ATOM   2754  N   ASP A 375      -8.796 -24.829  56.384  1.00 95.94
N
ANISOU 2754  N   ASP A 375    10785  16008   9662  -1713   1938
36        N
ATOM   2755  CA  ASP A 375      -9.313 -26.137  56.789  1.00102.27
C
ANISOU 2755  CA  ASP A 375    11530  16861  10467  -1961   1766
231        C
ATOM   2756  C   ASP A 375      -8.313 -27.260  56.525  1.00 93.15
C
ANISOU 2756  C   ASP A 375    10525  15403   9463  -2038   1594
297        C
ATOM   2757  O   ASP A 375      -8.089 -28.123  57.383  1.00 94.17
O
ANISOU 2757  O   ASP A 375    10647  15542   9590  -2223   1490
```

```
456       O
ATOM   2758  CB  ASP A 375     -10.634 -26.418  56.070  1.00102.78
C
ANISOU 2758  CB  ASP A 375    11484  17050  10519  -2006   1723
291       C
ATOM   2759  CG  ASP A 375     -11.794 -25.625  56.646  1.00138.51
C
ANISOU 2759  CG  ASP A 375    15804  21925  14898  -1983   1882
321       C
ATOM   2760  OD1 ASP A 375     -11.549 -24.548  57.230  1.00149.64
O
ANISOU 2760  OD1 ASP A 375    17204  23422  16231  -1850   2078
219       O
ATOM   2761  OD2 ASP A 375     -12.949 -26.084  56.520  1.00125.28
O
ANISOU 2761  OD2 ASP A 375    13984  20437  13180  -2106   1822
452       O
ATOM   2762  N   ALA A 376      -7.707 -27.267  55.338  1.00110.85
N
ANISOU 2762  N   ALA A 376    12905  17369  11843  -1893   1575
187       N
ATOM   2763  CA  ALA A 376      -6.688 -28.261  55.009  1.00 97.50
C
ANISOU 2763  CA  ALA A 376    11370  15356  10318  -1922   1458
240       C
ATOM   2764  C   ALA A 376      -5.490 -28.217  55.954  1.00 98.51
C
ANISOU 2764  C   ALA A 376    11515  15443  10470  -1916   1462
300       C
ATOM   2765  O   ALA A 376      -4.987 -29.267  56.372  1.00128.95
O
ANISOU 2765  O   ALA A 376    15408  19171  14417  -2036   1349
467       O
ATOM   2766  CB  ALA A 376      -6.226 -28.070  53.565  1.00100.12
C
ANISOU 2766  CB  ALA A 376    11851  15415  10775  -1747   1482
82       C
ATOM   2767  N   ALA A 377      -5.016 -27.019  56.299  1.00105.47
N
ANISOU 2767  N   ALA A 377    12373  16432  11268  -1791   1591
182       N
ATOM   2768  CA  ALA A 377      -3.921 -26.898  57.262  1.00152.51
C
ANISOU 2768  CA  ALA A 377    18331  22408  17207  -1830   1587
254       C
ATOM   2769  C   ALA A 377      -4.279 -27.467  58.632  1.00130.12
C
ANISOU 2769  C   ALA A 377    15388  19795  14256  -2078   1514
455       C
ATOM   2770  O   ALA A 377      -3.500 -28.229  59.217  1.00 93.18
O
ANISOU 2770  O   ALA A 377    10713  15046   9646  -2183   1403
635       O
ATOM   2771  CB  ALA A 377      -3.500 -25.433  57.385  1.00138.76
```

C
ANISOU 2771 CB ALA A 377   16604 20768 15352 -1693   1754
73   C
ATOM   2772 N   MET A 378   -5.448 -27.112 59.167 1.00 93.14
N
ANISOU 2772 N   MET A 378   10598 15388   9402 -2172   1582
444   N
ATOM   2773 CA MET A 378   -5.781 -27.541 60.524 1.00 94.80
C
ANISOU 2773 CA MET A 378   10710 15833   9476 -2417   1532
620   C
ATOM   2774 C   MET A 378   -6.150 -29.019 60.597 1.00 96.13
C
ANISOU 2774 C   MET A 378   10859 15928   9739 -2594   1348
833   C
ATOM   2775 O   MET A 378   -5.986 -29.639 61.654 1.00 97.48
O
ANISOU 2775 O   MET A 378   10980 16200   9859 -2795   1258
1025   O
ATOM   2776 CB MET A 378   -6.890 -26.660 61.094 1.00 95.39
C
ANISOU 2776 CB MET A 378   10681 16222   9340 -2452   1697
539   C
ATOM   2777 CG MET A 378   -6.392 -25.253 61.368 1.00 94.61
C
ANISOU 2777 CG MET A 378   10633 16197   9117 -2334   1896
357   C
ATOM   2778 SD MET A 378   -7.611 -24.109 62.021 1.00 95.60
S
ANISOU 2778 SD MET A 378   10671 16642   9010 -2332   2160
246   S
ATOM   2779 CE MET A 378   -6.634 -22.618 62.116 1.00 94.58
C
ANISOU 2779 CE MET A 378   10688 16464   8782 -2193   2372
23   C
ATOM   2780 N   VAL A 379   -6.650 -29.599 59.505 1.00 95.97
N
ANISOU 2780 N   VAL A 379   10889 15733   9841 -2544   1293
808   N
ATOM   2781 CA VAL A 379   -6.798 -31.052 59.442 1.00 97.29
C
ANISOU 2781 CA VAL A 379   11100 15748 10119 -2708   1128
996   C
ATOM   2782 C   VAL A 379   -5.446 -31.734 59.631 1.00 97.48
C
ANISOU 2782 C   VAL A 379   11214 15516 10309 -2691   1045
1127   C
ATOM   2783 O   VAL A 379   -5.340 -32.762 60.312 1.00 99.05
O
ANISOU 2783 O   VAL A 379   11400 15687 10545 -2865   928
1352   O
ATOM   2784 CB VAL A 379   -7.471 -31.468 58.120 1.00105.36
C
ANISOU 2784 CB VAL A 379   12202 16603 11225 -2671   1100

```
917       C
ATOM   2785  CG1 VAL A 379      -7.303 -32.963  57.876  1.00105.11
C
ANISOU 2785  CG1 VAL A 379    12296  16302  11338  -2814    958
1077       C
ATOM   2786  CG2 VAL A 379      -8.946 -31.098  58.140  1.00138.36
C
ANISOU 2786  CG2 VAL A 379    16239  21090  15241  -2756   1138
893       C
ATOM   2787  N   GLY A 380      -4.391 -31.165  59.048  1.00140.27
N
ANISOU 2787  N   GLY A 380    16713  20752  15832  -2477   1110
1010       N
ATOM   2788  CA  GLY A 380      -3.052 -31.712  59.138  1.00125.02
C
ANISOU 2788  CA  GLY A 380    14841  18584  14075  -2422   1054
1145       C
ATOM   2789  C   GLY A 380      -2.231 -31.232  60.312  1.00116.21
C
ANISOU 2789  C   GLY A 380    13625  17663  12867  -2480   1048
1256       C
ATOM   2790  O   GLY A 380      -1.171 -31.802  60.590  1.00125.04
O
ANISOU 2790  O   GLY A 380    14744  18642  14123  -2473    976
1445       O
ATOM   2791  N   MET A 381      -2.694 -30.194  61.012  1.00119.44
N
ANISOU 2791  N   MET A 381    13950  18393  13040  -2547   1132
1154       N
ATOM   2792  CA  MET A 381      -1.888 -29.536  62.035  1.00113.75
C
ANISOU 2792  CA  MET A 381    13169  17865  12186  -2622   1151
1211       C
ATOM   2793  C   MET A 381      -1.721 -30.400  63.279  1.00 99.54
C
ANISOU 2793  C   MET A 381    11277  16203  10341  -2877   1010
1513       C
ATOM   2794  O   MET A 381      -0.783 -30.180  64.052  1.00 99.20
O
ANISOU 2794  O   MET A 381    11186  16266  10240  -2960    973
1641       O
ATOM   2795  CB  MET A 381      -2.516 -28.192  62.407  1.00127.73
C
ANISOU 2795  CB  MET A 381    14916  19911  13704  -2634   1318
997       C
ATOM   2796  CG  MET A 381      -1.631 -27.295  63.257  1.00101.51
C
ANISOU 2796  CG  MET A 381    11591  16759  10219  -2708   1376
986       C
ATOM   2797  SD  MET A 381      -2.339 -25.654  63.470  1.00114.89
S
ANISOU 2797  SD  MET A 381    13324  18685  11645  -2674   1634
689       S
ATOM   2798  CE  MET A 381      -2.250 -25.040  61.789  1.00173.82
```

```
C
ANISOU 2798  CE  MET A 381    20886  25871  19286  -2320   1735
437       C
ATOM   2799  N   LEU A 382      -2.597 -31.380  63.476  1.00 99.82
N
ANISOU 2799  N   LEU A 382    11285  16249  10395  -3018    923
1643       N
ATOM   2800  CA  LEU A 382      -2.504 -32.327  64.578  1.00129.83
C
ANISOU 2800  CA  LEU A 382    15005  20152  14173  -3261    778
1949       C
ATOM   2801  C   LEU A 382      -1.745 -33.603  64.216  1.00112.87
C
ANISOU 2801  C   LEU A 382    12903  17676  12307  -3207    652
2185       C
ATOM   2802  O   LEU A 382      -1.840 -34.592  64.952  1.00117.34
O
ANISOU 2802  O   LEU A 382    13419  18269  12895  -3393    528
2454       O
ATOM   2803  CB  LEU A 382      -3.910 -32.655  65.092  1.00168.21
C
ANISOU 2803  CB  LEU A 382    19808  25228  18875  -3465    764
1969       C
ATOM   2804  CG  LEU A 382      -5.029 -32.919  64.075  1.00111.85
C
ANISOU 2804  CG  LEU A 382    12722  17979  11796  -3391    798
1827       C
ATOM   2805  CD1 LEU A 382      -4.755 -34.092  63.165  1.00102.74
C
ANISOU 2805  CD1 LEU A 382    11685  16447  10903  -3321    703
1920       C
ATOM   2806  CD2 LEU A 382      -6.336 -33.134  64.809  1.00103.73
C
ANISOU 2806  CD2 LEU A 382    11594  17241  10578  -3623    786
1885       C
ATOM   2807  N   ALA A 383      -1.060 -33.611  63.068  1.00105.44
N
ANISOU 2807  N   ALA A 383    12066  16413  11584  -2953    701
2088       N
ATOM   2808  CA  ALA A 383      -0.093 -34.626  62.648  1.00150.42
C
ANISOU 2808  CA  ALA A 383    17819  21762  17572  -2840    640
2292       C
ATOM   2809  C   ALA A 383      -0.740 -35.859  62.027  1.00163.15
C
ANISOU 2809  C   ALA A 383    19555  23091  19342  -2862    606
2347       C
ATOM   2810  O   ALA A 383      -0.241 -36.373  61.020  1.00190.24
O
ANISOU 2810  O   ALA A 383    23124  26148  23010  -2680    652
2323       O
ATOM   2811  CB  ALA A 383       0.813 -35.043  63.813  1.00129.53
C
ANISOU 2811  CB  ALA A 383    15040  19235  14943  -2968    525
```

```
2640       C
ATOM   2812  N   ASP A 384      -1.834 -36.349  62.597  1.00104.79
N
ANISOU 2812  N   ASP A 384    12132  15866  11818  -3095    536
2416       N
ATOM   2813  CA  ASP A 384      -2.501 -37.530  62.047  1.00108.45
C
ANISOU 2813  CA  ASP A 384    12729  16076  12399  -3166    498
2470       C
ATOM   2814  C   ASP A 384      -3.913 -37.163  61.621  1.00127.32
C
ANISOU 2814  C   ASP A 384    15137  18631  14608  -3259    531
2244       C
ATOM   2815  O   ASP A 384      -4.798 -37.019  62.486  1.00105.80
O
ANISOU 2815  O   ASP A 384    12287  16240  11672  -3467    488
2289       O
ATOM   2816  CB  ASP A 384      -2.531 -38.662  63.075  1.00108.51
C
ANISOU 2816  CB  ASP A 384    12687  16106  12436  -3384    367
2813       C
ATOM   2817  CG  ASP A 384      -3.178 -39.933  62.537  1.00117.25
C
ANISOU 2817  CG  ASP A 384    13964  16924  13662  -3481    335
2878       C
ATOM   2818  OD1 ASP A 384      -3.365 -40.881  63.330  1.00112.19
O
ANISOU 2818  OD1 ASP A 384    13300  16297  13031  -3677    232
3146       O
ATOM   2819  OD2 ASP A 384      -3.482 -39.999  61.326  1.00122.36
O
ANISOU 2819  OD2 ASP A 384    14781  17328  14382  -3380    413
2669       O
ATOM   2820  N   PRO A 385      -4.188 -37.058  60.319  1.00141.64
N
ANISOU 2820  N   PRO A 385    17093  20229  16494  -3126    606
2025       N
ATOM   2821  CA  PRO A 385      -5.500 -36.555  59.883  1.00103.45
C
ANISOU 2821  CA  PRO A 385    12232  15597  11478  -3200    636
1829       C
ATOM   2822  C   PRO A 385      -6.664 -37.426  60.319  1.00105.30
C
ANISOU 2822  C   PRO A 385    12442  15957  11609  -3486    537
1965       C
ATOM   2823  O   PRO A 385      -7.798 -36.933  60.376  1.00127.89
O
ANISOU 2823  O   PRO A 385    15198  19113  14282  -3584    551
1874       O
ATOM   2824  CB  PRO A 385      -5.364 -36.530  58.357  1.00124.84
C
ANISOU 2824  CB  PRO A 385    15125  17988  14319  -3027    709
1627       C
ATOM   2825  CG  PRO A 385      -3.921 -36.213  58.142  1.00120.61
```

```
C
ANISOU 2825  CG  PRO A 385    14635  17235  13956  -2784    774
1612       C
ATOM   2826  CD  PRO A 385      -3.194 -36.988  59.232  1.00115.45
C
ANISOU 2826  CD  PRO A 385    13922  16552  13391  -2867    691
1916       C
ATOM   2827  N   LYS A 386      -6.422 -38.706  60.615  1.00107.25
N
ANISOU 2827  N   LYS A 386    12781  15989  11980  -3618    447
2193       N
ATOM   2828  CA  LYS A 386      -7.475 -39.569  61.142  1.00109.19
C
ANISOU 2828  CA  LYS A 386    13007  16363  12119  -3916    345
2346       C
ATOM   2829  C   LYS A 386      -8.032 -39.033  62.457  1.00145.95
C
ANISOU 2829  C   LYS A 386    17425  21484  16545  -4072    310
2430       C
ATOM   2830  O   LYS A 386      -9.243 -39.098  62.699  1.00157.01
O
ANISOU 2830  O   LYS A 386    18742  23142  17773  -4271    277
2433       O
ATOM   2831  CB  LYS A 386      -6.933 -40.986  61.330  1.00111.37
C
ANISOU 2831  CB  LYS A 386    13432  16298  12586  -4005    272
2596       C
ATOM   2832  CG  LYS A 386      -6.467 -41.645  60.044  1.00111.66
C
ANISOU 2832  CG  LYS A 386    13742  15839  12843  -3880    339
2516       C
ATOM   2833  CD  LYS A 386      -7.601 -41.785  59.046  1.00112.61
C
ANISOU 2833  CD  LYS A 386    13988  15940  12857  -4017    344
2335       C
ATOM   2834  CE  LYS A 386      -7.134 -42.478  57.778  1.00138.74
C
ANISOU 2834  CE  LYS A 386    17610  18743  16362  -3933    425
2244       C
ATOM   2835  NZ  LYS A 386      -6.722 -43.885  58.034  1.00140.11
N
ANISOU 2835  NZ  LYS A 386    17972  18552  16711  -4033    409
2478       N
ATOM   2836  N   GLU A 387      -7.155 -38.514  63.323  1.00151.40
N
ANISOU 2836  N   GLU A 387    18009  22295  17223  -4002    321
2507       N
ATOM   2837  CA  GLU A 387      -7.562 -38.027  64.641  1.00154.56
C
ANISOU 2837  CA  GLU A 387    18216  23116  17392  -4175    303
2588       C
ATOM   2838  C   GLU A 387      -8.643 -36.955  64.556  1.00162.26
C
ANISOU 2838  C   GLU A 387    19079  24422  18151  -4172    412
```

```
2368       C
ATOM   2839  O   GLU A 387      -9.515 -36.882  65.429  1.00152.34
O
ANISOU 2839  O   GLU A 387    17690  23496  16698  -4375    404
2434       O
ATOM   2840  CB  GLU A 387      -6.345 -37.476  65.386  1.00156.26
C
ANISOU 2840  CB  GLU A 387    18363  23394  17613  -4093    315
2665       C
ATOM   2841  CG  GLU A 387      -5.312 -38.519  65.778  1.00167.27
C
ANISOU 2841  CG  GLU A 387    19798  24554  19202  -4121    200
2965       C
ATOM   2842  CD  GLU A 387      -4.208 -37.942  66.645  1.00161.79
C
ANISOU 2842  CD  GLU A 387    18993  24012  18467  -4100    188
3082       C
ATOM   2843  OE1 GLU A 387      -4.298 -36.749  67.006  1.00139.31
O
ANISOU 2843  OE1 GLU A 387    16065  21453  15414  -4098    273
2912       O
ATOM   2844  OE2 GLU A 387      -3.249 -38.679  66.959  1.00157.70
O
ANISOU 2844  OE2 GLU A 387    18473  23326  18120  -4092     99
3355       O
ATOM   2845  N   ALA A 388      -8.594 -36.116  63.520  1.00152.95
N
ANISOU 2845  N   ALA A 388    17945  23157  17011  -3938    525
2120       N
ATOM   2846  CA  ALA A 388      -9.606 -35.083  63.321  1.00133.91
C
ANISOU 2846  CA  ALA A 388    15424  21029  14428  -3894    647
1929       C
ATOM   2847  C   ALA A 388     -11.015 -35.659  63.218  1.00122.32
C
ANISOU 2847  C   ALA A 388    13891  19718  12866  -4097    592
1992       C
ATOM   2848  O   ALA A 388     -11.975 -35.049  63.700  1.00119.30
O
ANISOU 2848  O   ALA A 388    13346  19685  12299  -4163    671
1961       O
ATOM   2849  CB  ALA A 388      -9.275 -34.263  62.073  1.00131.13
C
ANISOU 2849  CB  ALA A 388    15153  20501  14168  -3610    755
1685       C
ATOM   2850  N   ARG A 389     -11.165 -36.823  62.586  1.00126.77
N
ANISOU 2850  N   ARG A 389    14587  20030  13551  -4205    473
2082       N
ATOM   2851  CA  ARG A 389     -12.479 -37.405  62.338  1.00138.96
C
ANISOU 2851  CA  ARG A 389    16090  21709  15002  -4422    407
2141       C
ATOM   2852  C   ARG A 389     -12.748 -38.678  63.133  1.00136.08
```

C
ANISOU 2852 C   ARG A 389   15745 21343 14617 -4724   263
2394   C
ATOM   2853 O   ARG A 389   -13.860 -39.212 63.058 1.00154.77
O
ANISOU 2853 O   ARG A 389   18069 23853 16884 -4948   197
2467   O
ATOM   2854 CB ARG A 389   -12.646 -37.708 60.841 1.00163.68
C
ANISOU 2854 CB ARG A 389   19382 24568 18239 -4363   391
2023   C
ATOM   2855 CG ARG A 389   -12.131 -36.635 59.873 1.00197.58
C
ANISOU 2855 CG ARG A 389   23715 28754 22603 -4053   515
1783   C
ATOM   2856 CD ARG A 389   -12.542 -35.212 60.238 1.00213.19
C
ANISOU 2856 CD ARG A 389   25480 31088 24436 -3905   657
1665   C
ATOM   2857 NE ARG A 389   -13.989 -35.037 60.300 1.00183.96
N
ANISOU 2857 NE ARG A 389   21592 27735 20568 -4042   665
1705   N
ATOM   2858 CZ ARG A 389   -14.588 -33.958 60.792 1.00161.89
C
ANISOU 2858 CZ ARG A 389   18591 25285 17635 -3955   806
1654   C
ATOM   2859 NH1 ARG A 389   -13.870 -32.903 61.151 1.00154.05
N
ANISOU 2859 NH1 ARG A 389   17586 24308 16637 -3737   953
1525   N
ATOM   2860 NH2 ARG A 389   -15.911 -33.892 60.820 1.00164.57
N
ANISOU 2860 NH2 ARG A 389   18744 25939 17845 -4076   816
1726   N
ATOM   2861 N   ALA A 390   -11.767 -39.179 63.882 1.00112.45
N
ANISOU 2861 N   ALA A 390   12808 18203 11714 -4743   207
2547   N
ATOM   2862 CA ALA A 390   -11.807 -40.519 64.460 1.00114.68
C
ANISOU 2862 CA ALA A 390   13160 18375 12036 -4996   67
2803   C
ATOM   2863 C   ALA A 390   -12.966 -40.773 65.422 1.00147.18
C
ANISOU 2863 C   ALA A 390   17112 22875 15935 -5296   6
2943   C
ATOM   2864 O   ALA A 390   -12.943 -40.321 66.572 1.00143.75
O
ANISOU 2864 O   ALA A 390   16519 22735 15364 -5359   27
3014   O
ATOM   2865 CB ALA A 390   -10.487 -40.807 65.179 1.00114.91
C
ANISOU 2865 CB ALA A 390   13226 18237 12198 -4929   31

```
2968       C
ATOM   2866  N   GLY A 391     -13.982 -41.499  64.951  1.00164.84
N
ANISOU 2866  N   GLY A 391    19392  25114  18126  -5502    -64
2982       N
ATOM   2867  CA  GLY A 391     -15.120 -41.903  65.761  1.00145.13
C
ANISOU 2867  CA  GLY A 391    16750  22958  15436  -5811   -134
3136       C
ATOM   2868  C   GLY A 391     -16.049 -40.807  66.231  1.00137.62
C
ANISOU 2868  C   GLY A 391    15532  22491  14266  -5805    -26
3050       C
ATOM   2869  O   GLY A 391     -16.812 -41.023  67.177  1.00126.56
O
ANISOU 2869  O   GLY A 391    13984  21406  12699  -6041    -58
3192       O
ATOM   2870  N   ILE A 392     -16.004 -39.632  65.608  1.00156.74
N
ANISOU 2870  N   ILE A 392    17892  24974  16689  -5536    117
2829       N
ATOM   2871  CA  ILE A 392     -16.745 -38.489  66.131  1.00160.34
C
ANISOU 2871  CA  ILE A 392    18106  25856  16960  -5479    269
2749       C
ATOM   2872  C   ILE A 392     -18.214 -38.526  65.697  1.00181.20
C
ANISOU 2872  C   ILE A 392    20593  28771  19484  -5613    268
2775       C
ATOM   2873  O   ILE A 392     -19.089 -38.028  66.419  1.00178.86
O
ANISOU 2873  O   ILE A 392    20073  28870  19016  -5683    361
2816       O
ATOM   2874  CB  ILE A 392     -16.026 -37.189  65.724  1.00148.31
C
ANISOU 2874  CB  ILE A 392    16588  24272  15490  -5131    438
2518       C
ATOM   2875  CG1 ILE A 392     -14.819 -36.968  66.638  1.00128.57
C
ANISOU 2875  CG1 ILE A 392    14144  21698  13008  -5078    457
2541       C
ATOM   2876  CG2 ILE A 392     -16.947 -35.987  65.787  1.00157.09
C
ANISOU 2876  CG2 ILE A 392    17486  25747  16453  -5017    629
2402       C
ATOM   2877  CD1 ILE A 392     -13.789 -36.023  66.083  1.00112.51
C
ANISOU 2877  CD1 ILE A 392    12195  19475  11079  -4765    569
2341       C
ATOM   2878  N   ARG A 393     -18.513 -39.157  64.558  1.00179.81
N
ANISOU 2878  N   ARG A 393    20533  28398  19387  -5675    165
2769       N
ATOM   2879  CA  ARG A 393     -19.878 -39.378  64.065  1.00170.96
```

```
C
ANISOU 2879  CA  ARG A 393    19278  27529  18152  -5861    119
2837       C
ATOM   2880  C   ARG A 393     -20.632 -38.048  63.930  1.00170.82
C
ANISOU 2880  C   ARG A 393    18993  27874  18038  -5661    299
2739       C
ATOM   2881  O   ARG A 393     -21.593 -37.745  64.642  1.00181.45
O
ANISOU 2881  O   ARG A 393    20090  29622  19230  -5755    370
2836       O
ATOM   2882  CB  ARG A 393     -20.632 -40.369  64.964  1.00177.75
C
ANISOU 2882  CB  ARG A 393    20068  28589  18881  -6235     -4
3072       C
ATOM   2883  CG  ARG A 393     -22.003 -40.785  64.433  1.00179.04
C
ANISOU 2883  CG  ARG A 393    20108  28998  18922  -6487    -87
3177       C
ATOM   2884  CD  ARG A 393     -22.717 -41.743  65.379  1.00180.64
C
ANISOU 2884  CD  ARG A 393    20240  29406  18986  -6865   -204
3411       C
ATOM   2885  NE  ARG A 393     -24.027 -42.139  64.866  1.00174.66
N
ANISOU 2885  NE  ARG A 393    19354  28909  18099  -7130   -292
3528       N
ATOM   2886  CZ  ARG A 393     -24.879 -42.930  65.510  1.00183.56
C
ANISOU 2886  CZ  ARG A 393    20389  30274  19081  -7488   -398
3737       C
ATOM   2887  NH1 ARG A 393     -26.051 -43.230  64.964  1.00177.90
N
ANISOU 2887  NH1 ARG A 393    19543  29811  18240  -7732   -482
3848       N
ATOM   2888  NH2 ARG A 393     -24.566 -43.420  66.702  1.00198.64
N
ANISOU 2888  NH2 ARG A 393    22328  32186  20961  -7619   -428
3851       N
ATOM   2889  N   GLU A 394     -20.129 -37.248  62.990  1.00176.89
N
ANISOU 2889  N   GLU A 394    19824  28473  18912  -5365    390
2548       N
ATOM   2890  CA  GLU A 394     -20.743 -35.973  62.640  1.00172.10
C
ANISOU 2890  CA  GLU A 394    18999  28136  18255  -5130    570
2451       C
ATOM   2891  C   GLU A 394     -22.159 -36.161  62.106  1.00163.81
C
ANISOU 2891  C   GLU A 394    17743  27394  17103  -5300    514
2585       C
ATOM   2892  O   GLU A 394     -22.454 -37.130  61.400  1.00167.37
O
ANISOU 2892  O   GLU A 394    18304  27730  17558  -5540    329
```

```
2666       O
ATOM   2893  CB  GLU A 394     -19.889 -35.238  61.604  1.00168.71
C
ANISOU 2893  CB  GLU A 394    18711  27421  17968  -4812    642
2235       C
ATOM   2894  CG  GLU A 394     -19.853 -35.904  60.238  1.00154.18
C
ANISOU 2894  CG  GLU A 394    17049  25315  16216  -4887    492
2210       C
ATOM   2895  CD  GLU A 394     -18.701 -35.421  59.383  1.00149.74
C
ANISOU 2895  CD  GLU A 394    16695  24387  15813  -4606    546
2000       C
ATOM   2896  OE1 GLU A 394     -18.959 -34.695  58.400  1.00164.06
O
ANISOU 2896  OE1 GLU A 394    18459  26234  17643  -4438    607
1899       O
ATOM   2897  OE2 GLU A 394     -17.541 -35.763  59.693  1.00142.87
O
ANISOU 2897  OE2 GLU A 394    16025  23209  15052  -4554    526
1953       O
ATOM   2898  N   VAL A 395     -23.047 -35.239  62.474  1.00161.86
N
ANISOU 2898  N   VAL A 395    17197  27545  16756  -5190    687
2622       N
ATOM   2899  CA  VAL A 395     -24.421 -35.264  61.993  1.00173.99
C
ANISOU 2899  CA  VAL A 395    18475  29432  18199  -5317    656
2782       C
ATOM   2900  C   VAL A 395     -24.750 -34.070  61.096  1.00165.60
C
ANISOU 2900  C   VAL A 395    17253  28484  17182  -5003    810
2701       C
ATOM   2901  O   VAL A 395     -25.647 -34.187  60.245  1.00181.43
O
ANISOU 2901  O   VAL A 395    19109  30676  19152  -5102    725
2823       O
ATOM   2902  CB  VAL A 395     -25.418 -35.345  63.170  1.00202.09
C
ANISOU 2902  CB  VAL A 395    21764  33419  21601  -5492    723
2970       C
ATOM   2903  CG1 VAL A 395     -25.375 -34.069  64.002  1.00210.19
C
ANISOU 2903  CG1 VAL A 395    22633  34628  22600  -5195   1028
2878       C
ATOM   2904  CG2 VAL A 395     -26.835 -35.640  62.677  1.00227.55
C
ANISOU 2904  CG2 VAL A 395    24721  37012  24726  -5698    638
3188       C
ATOM   2905  N   HIS A 396     -24.046 -32.947  61.231  1.00142.91
N
ANISOU 2905  N   HIS A 396    14413  25503  14382  -4647   1025
2513       N
ATOM   2906  CA  HIS A 396     -24.228 -31.805  60.342  1.00123.55
```

```
C
ANISOU 2906  CA  HIS A 396    11847  23102  11993  -4327   1176
2428       C
ATOM   2907  C   HIS A 396     -22.976 -30.939  60.364  1.00178.52
C
ANISOU 2907  C   HIS A 396    19013  29757  19059  -4006   1332
2172       C
ATOM   2908  O   HIS A 396     -22.256 -30.893  61.365  1.00207.50
O
ANISOU 2908  O   HIS A 396    22799  33330  22711  -3997   1404
2093       O
ATOM   2909  CB  HIS A 396     -25.446 -30.960  60.727  1.00120.74
C
ANISOU 2909  CB  HIS A 396    11113  23203  11557  -4209   1391
2568       C
ATOM   2910  CG  HIS A 396     -25.653 -29.773  59.839  1.00120.25
C
ANISOU 2910  CG  HIS A 396    10922  23192  11575  -3863   1559
2512       C
ATOM   2911  ND1 HIS A 396     -25.515 -28.476  60.284  1.00119.76
N
ANISOU 2911  ND1 HIS A 396    10786  23177  11541  -3511   1882
2398       N
ATOM   2912  CD2 HIS A 396     -25.970 -29.688  58.525  1.00120.32
C
ANISOU 2912  CD2 HIS A 396    10879  23203  11636  -3825   1453
2558       C
ATOM   2913  CE1 HIS A 396     -25.751 -27.643  59.286  1.00119.55
C
ANISOU 2913  CE1 HIS A 396    10654  23173  11595  -3252   1970
2388       C
ATOM   2914  NE2 HIS A 396     -26.029 -28.353  58.207  1.00119.86
N
ANISOU 2914  NE2 HIS A 396    10695  23199  11648  -3439   1704
2490       N
ATOM   2915  N   PHE A 397     -22.725 -30.256  59.245  1.00151.00
N
ANISOU 2915  N   PHE A 397    15568  26136  15669  -3765   1374
2056       N
ATOM   2916  CA  PHE A 397     -21.627 -29.304  59.141  1.00120.62
C
ANISOU 2916  CA  PHE A 397    11889  22026  11914  -3446   1535
1818       C
ATOM   2917  C   PHE A 397     -22.129 -27.987  58.566  1.00112.39
C
ANISOU 2917  C   PHE A 397    10663  21139  10901  -3122   1756
1783       C
ATOM   2918  O   PHE A 397     -22.795 -27.970  57.526  1.00113.13
O
ANISOU 2918  O   PHE A 397    10631  21334  11017  -3117   1678
1878       O
ATOM   2919  CB  PHE A 397     -20.479 -29.829  58.274  1.00110.30
C
ANISOU 2919  CB  PHE A 397    10896  20286  10726  -3453   1364
```

```
1677       C
ATOM   2920  CG  PHE A 397     -19.338 -28.861  58.164  1.00117.22
C
ANISOU 2920  CG  PHE A 397    11936  20913  11692  -3142   1520
1445       C
ATOM   2921  CD1 PHE A 397     -18.500 -28.641  59.246  1.00111.94
C
ANISOU 2921  CD1 PHE A 397    11371  20158  11002  -3105   1618
1357       C
ATOM   2922  CD2 PHE A 397     -19.135 -28.129  57.008  1.00111.01
C
ANISOU 2922  CD2 PHE A 397    11186  20002  10989  -2905   1572
1326       C
ATOM   2923  CE1 PHE A 397     -17.460 -27.734  59.166  1.00105.15
C
ANISOU 2923  CE1 PHE A 397    10658  19092  10203  -2849   1759
1154       C
ATOM   2924  CE2 PHE A 397     -18.098 -27.216  56.924  1.00104.72
C
ANISOU 2924  CE2 PHE A 397    10539  18986  10264  -2631   1720
1116       C
ATOM   2925  CZ  PHE A 397     -17.258 -27.021  58.005  1.00103.87
C
ANISOU 2925  CZ  PHE A 397    10540  18795  10132  -2608   1813
1028       C
ATOM   2926  N   LEU A 398     -21.795 -26.889  59.245  1.00135.13
N
ANISOU 2926  N   LEU A 398    13537  24031  13773  -2867   2034
1654       N
ATOM   2927  CA  LEU A 398     -22.156 -25.542  58.827  1.00125.29
C
ANISOU 2927  CA  LEU A 398    12150  22886  12567  -2521   2296
1606       C
ATOM   2928  C   LEU A 398     -20.968 -24.903  58.121  1.00160.59
C
ANISOU 2928  C   LEU A 398    16873  26997  17144  -2286   2332
1364       C
ATOM   2929  O   LEU A 398     -19.937 -24.663  58.767  1.00179.99
O
ANISOU 2929  O   LEU A 398    19542  29252  19595  -2240   2411
1191       O
ATOM   2930  CB  LEU A 398     -22.579 -24.704  60.036  1.00113.31
C
ANISOU 2930  CB  LEU A 398    10497  21592  10962  -2397   2622
1607       C
ATOM   2931  CG  LEU A 398     -23.115 -23.279  59.846  1.00121.77
C
ANISOU 2931  CG  LEU A 398    11403  22796  12069  -2030   2967
1589       C
ATOM   2932  CD1 LEU A 398     -21.999 -22.239  59.783  1.00134.30
C
ANISOU 2932  CD1 LEU A 398    13244  24080  13705  -1760   3162
1314       C
ATOM   2933  CD2 LEU A 398     -23.991 -23.200  58.606  1.00129.67
```

C
ANISOU 2933 CD2 LEU A 398   12168 23951 13149 -1943   2881
1766   C
ATOM   2934 N   PRO A 399   -21.049 -24.617 56.823 1.00123.11
N
ANISOU 2934 N   PRO A 399   12113 22176 12486 -2151   2273
1354   N
ATOM   2935 CA PRO A 399   -19.868 -24.167 56.081 1.00127.84
C
ANISOU 2935 CA PRO A 399   12971 22417 13186 -1966   2271
1128   C
ATOM   2936 C   PRO A 399   -19.690 -22.653 56.105 1.00105.60
C
ANISOU 2936 C   PRO A 399   10140 19585 10399 -1600   2589
993   C
ATOM   2937 O   PRO A 399   -20.591 -21.892 56.466 1.00107.39
O
ANISOU 2937 O   PRO A 399   10139 20072 10592 -1449   2823
1089   O
ATOM   2938 CB PRO A 399   -20.165 -24.644 54.651 1.00146.32
C
ANISOU 2938 CB PRO A 399   15300 24713 15583 -2040   2050
1201   C
ATOM   2939 CG PRO A 399   -21.655 -24.574 54.571 1.00108.84
C
ANISOU 2939 CG PRO A 399   10203 20375 10776 -2088   2074
1458   C
ATOM   2940 CD PRO A 399   -22.131 -25.044 55.920 1.00110.36
C
ANISOU 2940 CD PRO A 399   10282 20782 10869 -2267   2117
1569   C
ATOM   2941 N   PHE A 400   -18.483 -22.237 55.717 1.00103.26
N
ANISOU 2941 N   PHE A 400   10101 18964 10171 -1457   2606
771   N
ATOM   2942 CA PHE A 400   -18.092 -20.832 55.788 1.00102.56
C
ANISOU 2942 CA PHE A 400   10072 18796 10101 -1137   2903
606   C
ATOM   2943 C   PHE A 400   -19.010 -19.947 54.953 1.00103.72
C
ANISOU 2943 C   PHE A 400   10005 19102 10303   -886   3049
701   C
ATOM   2944 O   PHE A 400   -19.437 -20.316 53.857 1.00103.94
O
ANISOU 2944 O   PHE A 400   9939 19169 10384   -925   2863
814   O
ATOM   2945 CB PHE A 400   -16.643 -20.652 55.328 1.00 99.90
C
ANISOU 2945 CB PHE A 400   10037 18089   9830 -1059   2847
375   C
ATOM   2946 CG PHE A 400   -16.101 -19.270 55.578 1.00 99.20
C
ANISOU 2946 CG PHE A 400   10059 17899   9733   -785   3148

187 C
ATOM 2947 CD1 PHE A 400 -15.484 -18.961 56.781 1.00107.34
C
ANISOU 2947 CD1 PHE A 400 11222 18896 10664 -831 3300
73 C
ATOM 2948 CD2 PHE A 400 -16.221 -18.275 54.619 1.00 98.86
C
ANISOU 2948 CD2 PHE A 400 9996 17800 9767 -503 3282
132 C
ATOM 2949 CE1 PHE A 400 -14.990 -17.691 57.022 1.00 98.69
C
ANISOU 2949 CE1 PHE A 400 10261 17702 9536 -617 3587
-108 C
ATOM 2950 CE2 PHE A 400 -15.732 -17.002 54.855 1.00 98.40
C
ANISOU 2950 CE2 PHE A 400 10064 17630 9695 -262 3573
-42 C
ATOM 2951 CZ PHE A 400 -15.114 -16.710 56.059 1.00 98.34
C
ANISOU 2951 CZ PHE A 400 10210 17578 9577 -326 3730
-171 C
ATOM 2952 N ASN A 401 -19.306 -18.762 55.490 1.00104.71
N
ANISOU 2952 N ASN A 401 10061 19316 10407 -635 3396
663 N
ATOM 2953 CA ASN A 401 -20.194 -17.796 54.874 1.00134.81
C
ANISOU 2953 CA ASN A 401 13652 23286 14284 -351 3599
778 C
ATOM 2954 C ASN A 401 -19.630 -16.418 55.208 1.00119.10
C
ANISOU 2954 C ASN A 401 11825 21125 12303 -48 3959
572 C
ATOM 2955 O ASN A 401 -19.342 -16.144 56.387 1.00172.50
O
ANISOU 2955 O ASN A 401 18701 27872 18969 -80 4161
459 O
ATOM 2956 CB ASN A 401 -21.622 -17.951 55.405 1.00170.92
C
ANISOU 2956 CB ASN A 401 17875 28253 18813 -394 3693
1052 C
ATOM 2957 CG ASN A 401 -22.616 -17.041 54.712 1.00156.80
C
ANISOU 2957 CG ASN A 401 15805 26659 17112 -98 3885
1237 C
ATOM 2958 OD1 ASN A 401 -22.351 -16.511 53.634 1.00155.82
O
ANISOU 2958 OD1 ASN A 401 15723 26401 17081 84 3861
1200 O
ATOM 2959 ND2 ASN A 401 -23.778 -16.862 55.330 1.00130.85
N
ANISOU 2959 ND2 ASN A 401 12216 23701 13800 -48 4080
1457 N
ATOM 2960 N PRO A 402 -19.452 -15.537 54.217 1.00105.10

```
N
ANISOU 2960  N   PRO A 402    10084  19220  10628    223   4049
516       N
ATOM   2961  CA  PRO A 402     -18.695 -14.304  54.480  1.00104.31
C
ANISOU 2961  CA  PRO A 402    10214  18891  10526    470   4360
281       C
ATOM   2962  C   PRO A 402     -19.380 -13.362  55.455  1.00106.81
C
ANISOU 2962  C   PRO A 402    10442  19346  10794    656   4792
307       C
ATOM   2963  O   PRO A 402     -18.692 -12.578  56.120  1.00115.62
O
ANISOU 2963  O   PRO A 402    11807  20283  11842    736   5053
86       O
ATOM   2964  CB  PRO A 402     -18.578 -13.676  53.087  1.00103.48
C
ANISOU 2964  CB  PRO A 402    10110  18660  10547    707   4334
276       C
ATOM   2965  CG  PRO A 402     -18.483 -14.854  52.191  1.00108.04
C
ANISOU 2965  CG  PRO A 402    10656  19239  11157    470   3914
360       C
ATOM   2966  CD  PRO A 402     -19.450 -15.867  52.781  1.00104.17
C
ANISOU 2966  CD  PRO A 402     9926  19051  10604    220   3781
587       C
ATOM   2967  N   VAL A 403     -20.710 -13.403  55.562  1.00120.85
N
ANISOU 2967  N   VAL A 403    11881  21439  12597    717   4889
574       N
ATOM   2968  CA  VAL A 403     -21.384 -12.564  56.548  1.00124.45
C
ANISOU 2968  CA  VAL A 403    12255  22022  13010    893   5332
605       C
ATOM   2969  C   VAL A 403     -21.281 -13.156  57.946  1.00122.35
C
ANISOU 2969  C   VAL A 403    12071  21839  12580    612   5359
542       C
ATOM   2970  O   VAL A 403     -21.393 -12.420  58.933  1.00136.57
O
ANISOU 2970  O   VAL A 403    13957  23637  14294    701   5744
449       O
ATOM   2971  CB  VAL A 403     -22.861 -12.336  56.182  1.00135.64
C
ANISOU 2971  CB  VAL A 403    13250  23762  14526   1089   5461
942       C
ATOM   2972  CG1 VAL A 403     -22.970 -11.566  54.874  1.00141.57
C
ANISOU 2972  CG1 VAL A 403    13920  24435  15434   1396   5489
1018       C
ATOM   2973  CG2 VAL A 403     -23.601 -13.661  56.096  1.00162.60
C
ANISOU 2973  CG2 VAL A 403    16388  27480  17913    785   5103
```

```
1197        C
ATOM   2974  N   ASP A 404     -21.062 -14.467  58.055  1.00116.21
N
ANISOU 2974  N   ASP A 404    11285  21121  11748    265   4970
591        N
ATOM   2975  CA  ASP A 404     -20.997 -15.161  59.335  1.00126.93
C
ANISOU 2975  CA  ASP A 404    12695  22581  12952    -33   4944
571        C
ATOM   2976  C   ASP A 404     -19.568 -15.389  59.799  1.00117.34
C
ANISOU 2976  C   ASP A 404    11842  21097  11646   -225   4821
315        C
ATOM   2977  O   ASP A 404     -19.293 -15.343  61.002  1.00110.29
O
ANISOU 2977  O   ASP A 404    11083  20223  10601   -377   4967
219        O
ATOM   2978  CB  ASP A 404     -21.716 -16.509  59.239  1.00145.66
C
ANISOU 2978  CB  ASP A 404    14818  25205  15321   -310   4600
820        C
ATOM   2979  CG  ASP A 404     -23.202 -16.361  58.989  1.00145.87
C
ANISOU 2979  CG  ASP A 404    14447  25570  15406   -176   4719
1115        C
ATOM   2980  OD1 ASP A 404     -23.813 -15.427  59.550  1.00156.62
O
ANISOU 2980  OD1 ASP A 404    15708  27042  16759     53   5129
1145        O
ATOM   2981  OD2 ASP A 404     -23.758 -17.180  58.226  1.00157.64
O
ANISOU 2981  OD2 ASP A 404    15728  27220  16948   -307   4412
1327        O
ATOM   2982  N   LYS A 405     -18.657 -15.641  58.860  1.00145.41
N
ANISOU 2982  N   LYS A 405    15550  24415  15286   -231   4558
217        N
ATOM   2983  CA  LYS A 405     -17.244 -15.858  59.156  1.00138.74
C
ANISOU 2983  CA  LYS A 405    15020  23314  14381   -389   4424
5       C
ATOM   2984  C   LYS A 405     -17.030 -17.021  60.122  1.00116.40
C
ANISOU 2984  C   LYS A 405    12209  20576  11443   -753   4208
67       C
ATOM   2985  O   LYS A 405     -16.236 -16.929  61.057  1.00104.44
O
ANISOU 2985  O   LYS A 405    10896  18986   9801   -897   4268
-65        O
ATOM   2986  CB  LYS A 405     -16.596 -14.578  59.691  1.00127.22
C
ANISOU 2986  CB  LYS A 405    13807  21695  12837   -237   4780
-229        C
ATOM   2987  CG  LYS A 405     -16.757 -13.382  58.766  1.00142.62
```

```
C
ANISOU 2987  CG  LYS A 405    15763  23526  14899    133   5014
-292       C
ATOM   2988  CD  LYS A 405     -16.022 -12.161  59.291  1.00145.18
C
ANISOU 2988  CD  LYS A 405    16382  23656  15125    247   5359
-542       C
ATOM   2989  CE  LYS A 405     -15.941 -11.073  58.230  1.00133.62
C
ANISOU 2989  CE  LYS A 405    14971  22013  13788    595   5530
-617       C
ATOM   2990  NZ  LYS A 405     -17.289 -10.647  57.759  1.00148.37
N
ANISOU 2990  NZ  LYS A 405    16541  24059  15774    870   5721
-408       N
ATOM   2991  N   ARG A 406     -17.753 -18.121  59.912  1.00114.65
N
ANISOU 2991  N   ARG A 406    11778  20527  11258   -921   3954
281       N
ATOM   2992  CA  ARG A 406     -17.815 -19.168  60.921  1.00106.14
C
ANISOU 2992  CA  ARG A 406    10676  19582  10071  -1252   3797
379       C
ATOM   2993  C   ARG A 406     -18.063 -20.514  60.251  1.00105.71
C
ANISOU 2993  C   ARG A 406    10515  19551  10099  -1449   3411
552       C
ATOM   2994  O   ARG A 406     -18.740 -20.598  59.222  1.00120.73
O
ANISOU 2994  O   ARG A 406    12257  21515  12099  -1353   3328
663       O
ATOM   2995  CB  ARG A 406     -18.923 -18.881  61.948  1.00109.02
C
ANISOU 2995  CB  ARG A 406    10853  20253  10317  -1276   4065
489       C
ATOM   2996  CG  ARG A 406     -19.174 -20.011  62.941  1.00110.08
C
ANISOU 2996  CG  ARG A 406    10922  20567  10336  -1629   3893
627       C
ATOM   2997  CD  ARG A 406     -20.156 -19.624  64.043  1.00141.63
C
ANISOU 2997  CD  ARG A 406    14766  24851  14195  -1653   4201
703       C
ATOM   2998  NE  ARG A 406     -21.520 -19.453  63.544  1.00127.04
N
ANISOU 2998  NE  ARG A 406    12595  23251  12425  -1489   4307
901       N
ATOM   2999  CZ  ARG A 406     -22.134 -18.282  63.406  1.00116.49
C
ANISOU 2999  CZ  ARG A 406    11157  21979  11125  -1170   4680
889       C
ATOM   3000  NH1 ARG A 406     -21.462 -17.152  63.567  1.00135.88
N
ANISOU 3000  NH1 ARG A 406    13846  24225  13557   -969   4974
```

```
657       N
ATOM   3001  NH2 ARG A 406     -23.382 -18.243  62.960  1.00118.54
N
ANISOU 3001  NH2 ARG A 406    11082  22496  11463  -1041   4743
1122       N
ATOM   3002  N   THR A 407     -17.499 -21.564  60.849  1.00105.23
N
ANISOU 3002  N   THR A 407    10556  19438   9988  -1739   3182
583       N
ATOM   3003  CA  THR A 407     -17.813 -22.952  60.539  1.00105.45
C
ANISOU 3003  CA  THR A 407    10506  19504  10058  -1988   2852
760       C
ATOM   3004  C   THR A 407     -18.218 -23.647  61.833  1.00107.17
C
ANISOU 3004  C   THR A 407    10647  19935  10139  -2269   2826
892       C
ATOM   3005  O   THR A 407     -17.827 -23.224  62.922  1.00160.49
O
ANISOU 3005  O   THR A 407    17487  26720  16772  -2312   2993
811       O
ATOM   3006  CB  THR A 407     -16.627 -23.681  59.883  1.00123.05
C
ANISOU 3006  CB  THR A 407    12961  21401  12393  -2062   2582
685       C
ATOM   3007  OG1 THR A 407     -15.489 -23.637  60.752  1.00102.35
O
ANISOU 3007  OG1 THR A 407    10534  18640   9717  -2135   2601
577       O
ATOM   3008  CG2 THR A 407     -16.269 -23.031  58.558  1.00115.82
C
ANISOU 3008  CG2 THR A 407    12118  20286  11603  -1803   2602
558       C
ATOM   3009  N   ALA A 408     -19.006 -24.718  61.724  1.00108.93
N
ANISOU 3009  N   ALA A 408    10719  20310  10361  -2487   2615
1096       N
ATOM   3010  CA  ALA A 408     -19.314 -25.503  62.913  1.00129.50
C
ANISOU 3010  CA  ALA A 408    13268  23096  12840  -2781   2553
1230       C
ATOM   3011  C   ALA A 408     -19.613 -26.951  62.549  1.00137.35
C
ANISOU 3011  C   ALA A 408    14230  24084  13872  -3059   2219
1411       C
ATOM   3012  O   ALA A 408     -20.176 -27.238  61.490  1.00143.52
O
ANISOU 3012  O   ALA A 408    14922  24879  14729  -3043   2098
1488       O
ATOM   3013  CB  ALA A 408     -20.501 -24.906  63.680  1.00138.46
C
ANISOU 3013  CB  ALA A 408    14164  24590  13855  -2747   2825
1320       C
ATOM   3014  N   LEU A 409     -19.225 -27.860  63.444  1.00149.23
```

```
N
ANISOU 3014  N   LEU A 409    15820  25568  15314  -3331   2074
1484       N
ATOM   3015  CA  LEU A 409     -19.476 -29.291  63.304  1.00147.72
C
ANISOU 3015  CA  LEU A 409    15631  25355  15143  -3627   1779
1662       C
ATOM   3016  C   LEU A 409     -20.344 -29.774  64.459  1.00185.63
C
ANISOU 3016  C   LEU A 409    20263  30478  19789  -3884   1790
1841       C
ATOM   3017  O   LEU A 409     -20.036 -29.502  65.624  1.00208.97
O
ANISOU 3017  O   LEU A 409    23248  33516  22635  -3941   1912
1814       O
ATOM   3018  CB  LEU A 409     -18.167 -30.082  63.269  1.00144.97
C
ANISOU 3018  CB  LEU A 409    15547  24650  14886  -3722   1577
1621       C
ATOM   3019  CG  LEU A 409     -18.326 -31.573  62.971  1.00139.51
C
ANISOU 3019  CG  LEU A 409    14913  23852  14242  -4003   1293
1787       C
ATOM   3020  CD1 LEU A 409     -18.557 -31.797  61.485  1.00128.77
C
ANISOU 3020  CD1 LEU A 409    13596  22340  12993  -3941   1186
1759       C
ATOM   3021  CD2 LEU A 409     -17.122 -32.362  63.469  1.00137.15
C
ANISOU 3021  CD2 LEU A 409    14829  23276  14006  -4121   1149
1809       C
ATOM   3022  N   THR A 410     -21.424 -30.485  64.140  1.00172.82
N
ANISOU 3022  N   THR A 410    18472  29050  18144  -4061   1662
2027       N
ATOM   3023  CA  THR A 410     -22.317 -31.069  65.136  1.00157.90
C
ANISOU 3023  CA  THR A 410    16412  27474  16110  -4334   1643
2221       C
ATOM   3024  C   THR A 410     -22.175 -32.588  65.138  1.00156.07
C
ANISOU 3024  C   THR A 410    16295  27113  15891  -4674   1327
2367       C
ATOM   3025  O   THR A 410     -22.189 -33.213  64.073  1.00156.22
O
ANISOU 3025  O   THR A 410    16393  26961  16004  -4731   1138
2394       O
ATOM   3026  CB  THR A 410     -23.770 -30.676  64.863  1.00172.93
C
ANISOU 3026  CB  THR A 410    17995  29752  17960  -4290   1762
2355       C
ATOM   3027  OG1 THR A 410     -23.846 -29.265  64.625  1.00176.22
O
ANISOU 3027  OG1 THR A 410    18328  30219  18408  -3927   2061
```

```
2222       O
ATOM   3028  CG2 THR A 410     -24.651 -31.031  66.051  1.00205.45
C
ANISOU 3028  CG2 THR A 410    21922  34223  21916  -4526   1818
2531       C
ATOM   3029  N   TYR A 411     -22.037 -33.179  66.327  1.00152.39
N
ANISOU 3029  N   TYR A 411    15857  26720  15325  -4911   1278
2463       N
ATOM   3030  CA  TYR A 411     -21.670 -34.588  66.422  1.00140.01
C
ANISOU 3030  CA  TYR A 411    14446  24961  13789  -5207    998
2593       C
ATOM   3031  C   TYR A 411     -22.262 -35.219  67.679  1.00148.09
C
ANISOU 3031  C   TYR A 411    15359  26257  14652  -5518    961
2782       C
ATOM   3032  O   TYR A 411     -22.572 -34.538  68.660  1.00151.49
O
ANISOU 3032  O   TYR A 411    15655  26957  14947  -5497   1161
2772       O
ATOM   3033  CB  TYR A 411     -20.149 -34.770  66.389  1.00134.66
C
ANISOU 3033  CB  TYR A 411    14048  23879  13238  -5123    921
2484       C
ATOM   3034  CG  TYR A 411     -19.406 -34.132  67.541  1.00134.50
C
ANISOU 3034  CG  TYR A 411    14061  23904  13138  -5068   1064
2416       C
ATOM   3035  CD1 TYR A 411     -18.998 -34.884  68.635  1.00160.14
C
ANISOU 3035  CD1 TYR A 411    17369  27160  16317  -5321    952
2556       C
ATOM   3036  CD2 TYR A 411     -19.092 -32.778  67.523  1.00125.53
C
ANISOU 3036  CD2 TYR A 411    12912  22798  11985  -4783   1309
2218       C
ATOM   3037  CE1 TYR A 411     -18.308 -34.302  69.686  1.00178.99
C
ANISOU 3037  CE1 TYR A 411    19792  29610  18604  -5314   1070
2506       C
ATOM   3038  CE2 TYR A 411     -18.405 -32.188  68.568  1.00147.26
C
ANISOU 3038  CE2 TYR A 411    15723  25593  14634  -4777   1442
2149       C
ATOM   3039  CZ  TYR A 411     -18.012 -32.955  69.645  1.00169.49
C
ANISOU 3039  CZ  TYR A 411    18591  28441  17368  -5053   1315
2295       C
ATOM   3040  OH  TYR A 411     -17.332 -32.370  70.690  1.00164.53
O
ANISOU 3040  OH  TYR A 411    18024  27882  16610  -5090   1435
2239       O
ATOM   3041  N   ILE A 412     -22.414 -36.544  67.624  1.00144.29
```

```
N
ANISOU 3041  N   ILE A 412    14954  25689  14182  -5818    713
2950       N
ATOM   3042  CA  ILE A 412     -23.101 -37.342  68.641  1.00145.73
C
ANISOU 3042  CA  ILE A 412    15032  26122  14216  -6160    630
3162       C
ATOM   3043  C   ILE A 412     -22.111 -38.360  69.196  1.00142.89
C
ANISOU 3043  C   ILE A 412    14906  25478  13907  -6348    443
3245       C
ATOM   3044  O   ILE A 412     -21.661 -39.253  68.465  1.00125.19
O
ANISOU 3044  O   ILE A 412    12864  22903  11802  -6416    256
3278       O
ATOM   3045  CB  ILE A 412     -24.340 -38.048  68.076  1.00139.83
C
ANISOU 3045  CB  ILE A 412    14146  25567  13418  -6390    500
3328       C
ATOM   3046  CG1 ILE A 412     -25.281 -37.042  67.412  1.00149.21
C
ANISOU 3046  CG1 ILE A 412    15078  27034  14581  -6182    674
3283       C
ATOM   3047  CG2 ILE A 412     -25.063 -38.817  69.177  1.00136.39
C
ANISOU 3047  CG2 ILE A 412    13593  25412  12818  -6746    428
3546       C
ATOM   3048  CD1 ILE A 412     -26.408 -37.696  66.643  1.00153.68
C
ANISOU 3048  CD1 ILE A 412    15509  27777  15104  -6405    520
3453       C
ATOM   3049  N   ASP A 413     -21.768 -38.241  70.479  1.00145.44
N
ANISOU 3049  N   ASP A 413    15212  25927  14122  -6437    501
3291       N
ATOM   3050  CA  ASP A 413     -20.646 -38.985  71.036  1.00145.49
C
ANISOU 3050  CA  ASP A 413    15421  25667  14193  -6552    350
3375       C
ATOM   3051  C   ASP A 413     -21.013 -39.624  72.374  1.00139.60
C
ANISOU 3051  C   ASP A 413    14600  25165  13277  -6889    284
3585       C
ATOM   3052  O   ASP A 413     -22.024 -39.293  73.001  1.00129.63
O
ANISOU 3052  O   ASP A 413    13132  24292  11830  -7003    401
3626       O
ATOM   3053  CB  ASP A 413     -19.412 -38.082  71.194  1.00159.96
C
ANISOU 3053  CB  ASP A 413    17358  27337  16084  -6291    470
3206       C
ATOM   3054  CG  ASP A 413     -19.514 -37.147  72.387  1.00164.61
C
ANISOU 3054  CG  ASP A 413    17827  28249  16467  -6305    675
```

```
3157       C
ATOM   3055  OD1 ASP A 413     -18.468 -36.852  73.002  1.00182.33
O
ANISOU 3055  OD1 ASP A 413    20175  30405  18696  -6279    694
3126       O
ATOM   3056  OD2 ASP A 413     -20.634 -36.690  72.700  1.00193.05
O
ANISOU 3056  OD2 ASP A 413    21235  32196  19919  -6346    829
3153       O
ATOM   3057  N   GLY A 414     -20.154 -40.547  72.798  1.00135.91
N
ANISOU 3057  N   GLY A 414    14300  24460  12881  -7038    103
3729       N
ATOM   3058  CA  GLY A 414     -20.296 -41.248  74.067  1.00130.56
C
ANISOU 3058  CA  GLY A 414    13585  23961  12061  -7367      8
3952       C
ATOM   3059  C   GLY A 414     -21.570 -42.069  74.156  1.00132.83
C
ANISOU 3059  C   GLY A 414    13762  24463  12243  -7665    -86
4119       C
ATOM   3060  O   GLY A 414     -21.771 -43.034  73.408  1.00133.36
O
ANISOU 3060  O   GLY A 414    13937  24313  12420  -7772   -252
4206       O
ATOM   3061  N   SER A 415     -22.446 -41.693  75.088  1.00134.39
N
ANISOU 3061  N   SER A 415    13754  25092  12215  -7819     32
4165       N
ATOM   3062  CA  SER A 415     -23.713 -42.376  75.319  1.00167.21
C
ANISOU 3062  CA  SER A 415    17764  29527  16240  -8120    -37
4337       C
ATOM   3063  C   SER A 415     -24.860 -41.714  74.572  1.00179.89
C
ANISOU 3063  C   SER A 415    19160  31383  17805  -7995    107
4242       C
ATOM   3064  O   SER A 415     -26.026 -42.049  74.809  1.00185.79
O
ANISOU 3064  O   SER A 415    19724  32451  18417  -8221     95
4381       O
ATOM   3065  CB  SER A 415     -24.023 -42.431  76.818  1.00153.35
C
ANISOU 3065  CB  SER A 415    15895  28114  14257  -8385      7
4475       C
ATOM   3066  OG  SER A 415     -23.065 -43.210  77.515  1.00139.18
O
ANISOU 3066  OG  SER A 415    14271  26116  12493  -8555   -169
4629       O
ATOM   3067  N   GLY A 416     -24.549 -40.792  73.677  1.00156.90
N
ANISOU 3067  N   GLY A 416    16261  28343  15010  -7644    239
4030       N
ATOM   3068  CA  GLY A 416     -25.519 -40.107  72.864  1.00148.08
```

```
C
ANISOU 3068  CA  GLY A 416    14945  27433  13886  -7483    374
3954       C
ATOM   3069  C   GLY A 416     -25.632 -38.635  73.183  1.00157.61
C
ANISOU 3069  C   GLY A 416    15998  28860  15027  -7191    696
3782       C
ATOM   3070  O   GLY A 416     -26.680 -38.037  72.901  1.00135.20
O
ANISOU 3070  O   GLY A 416    12915  26319  12133  -7098    855
3785       O
ATOM   3071  N   ASN A 417     -24.594 -38.038  73.762  1.00163.24
N
ANISOU 3071  N   ASN A 417    16846  29438  15740  -7053    803
3647       N
ATOM   3072  CA  ASN A 417     -24.519 -36.592  73.903  1.00158.36
C
ANISOU 3072  CA  ASN A 417    16158  28933  15080  -6751   1122
3443       C
ATOM   3073  C   ASN A 417     -24.425 -35.950  72.528  1.00152.78
C
ANISOU 3073  C   ASN A 417    15460  28042  14547  -6410   1178
3285       C
ATOM   3074  O   ASN A 417     -23.776 -36.480  71.623  1.00129.29
O
ANISOU 3074  O   ASN A 417    12657  24730  11740  -6365    978
3262       O
ATOM   3075  CB  ASN A 417     -23.307 -36.204  74.750  1.00131.04
C
ANISOU 3075  CB  ASN A 417    12884  25334  11572  -6731   1181
3340       C
ATOM   3076  CG  ASN A 417     -23.384 -36.753  76.159  1.00143.40
C
ANISOU 3076  CG  ASN A 417    14437  27107  12941  -7081   1136
3500       C
ATOM   3077  OD1 ASN A 417     -24.437 -36.723  76.795  1.00168.37
O
ANISOU 3077  OD1 ASN A 417    17411  30624  15938  -7236   1254
3588       O
ATOM   3078  ND2 ASN A 417     -22.261 -37.264  76.655  1.00132.55
N
ANISOU 3078  ND2 ASN A 417    13253  25524  11585  -7210    966
3556       N
ATOM   3079  N   TRP A 418     -25.073 -34.803  72.370  1.00184.29
N
ANISOU 3079  N   TRP A 418    19270  32249  18501  -6165   1464
3183       N
ATOM   3080  CA  TRP A 418     -24.980 -34.024  71.142  1.00175.04
C
ANISOU 3080  CA  TRP A 418    18094  30930  17482  -5819   1551
3033       C
ATOM   3081  C   TRP A 418     -24.115 -32.799  71.417  1.00152.78
C
ANISOU 3081  C   TRP A 418    15399  27985  14664  -5538   1806
```

```
2795       C
ATOM   3082  O   TRP A 418     -24.537 -31.882  72.129  1.00175.39
O
ANISOU 3082  O   TRP A 418    18156  31083  17403  -5447   2112
2733       O
ATOM   3083  CB  TRP A 418     -26.370 -33.607  70.670  1.00169.36
C
ANISOU 3083  CB  TRP A 418    17064  30544  16740  -5730   1690
3126       C
ATOM   3084  CG  TRP A 418     -27.202 -34.680  70.044  1.00195.95
C
ANISOU 3084  CG  TRP A 418    20315  34014  20122  -5977   1429
3341       C
ATOM   3085  CD1 TRP A 418     -27.499 -35.905  70.569  1.00210.08
C
ANISOU 3085  CD1 TRP A 418    22116  35880  21825  -6365   1203
3532       C
ATOM   3086  CD2 TRP A 418     -27.899 -34.600  68.795  1.00197.49
C
ANISOU 3086  CD2 TRP A 418    20361  34272  20402  -5879   1373
3400       C
ATOM   3087  NE1 TRP A 418     -28.314 -36.603  69.710  1.00232.94
N
ANISOU 3087  NE1 TRP A 418    24901  38865  24740  -6527   1012
3692       N
ATOM   3088  CE2 TRP A 418     -28.577 -35.822  68.614  1.00212.83
C
ANISOU 3088  CE2 TRP A 418    22248  36325  22292  -6244   1106
3619       C
ATOM   3089  CE3 TRP A 418     -28.006 -33.617  67.806  1.00174.64
C
ANISOU 3089  CE3 TRP A 418    17384  31355  17618  -5536   1517
3298       C
ATOM   3090  CZ2 TRP A 418     -29.350 -36.086  67.485  1.00179.29
C
ANISOU 3090  CZ2 TRP A 418    17866  32178  18077  -6300    974
3737       C
ATOM   3091  CZ3 TRP A 418     -28.772 -33.882  66.686  1.00176.96
C
ANISOU 3091  CZ3 TRP A 418    17527  31755  17955  -5576   1380
3428       C
ATOM   3092  CH2 TRP A 418     -29.434 -35.106  66.534  1.00175.45
C
ANISOU 3092  CH2 TRP A 418    17285  31687  17691  -5967   1108
3644       C
ATOM   3093  N   HIS A 419     -22.918 -32.780  70.840  1.00129.96
N
ANISOU 3093  N   HIS A 419    12744  24725  11912  -5407   1695
2662       N
ATOM   3094  CA  HIS A 419     -21.952 -31.713  71.048  1.00145.14
C
ANISOU 3094  CA  HIS A 419    14817  26495  13833  -5179   1894
2441       C
ATOM   3095  C   HIS A 419     -21.735 -30.957  69.744  1.00179.22
```

```
C
ANISOU 3095  C   HIS A 419    19164  30614  18317  -4827   1965
2279       C
ATOM   3096  O   HIS A 419     -22.014 -31.465  68.655  1.00188.60
O
ANISOU 3096  O   HIS A 419    20320  31702  19637  -4798   1791
2337       O
ATOM   3097  CB  HIS A 419     -20.622 -32.264  71.570  1.00144.44
C
ANISOU 3097  CB  HIS A 419    14968  26160  13755  -5328   1711
2438       C
ATOM   3098  CG  HIS A 419     -20.631 -32.593  73.030  1.00185.52
C
ANISOU 3098  CG  HIS A 419    20165  31570  18756  -5630   1720
2550       C
ATOM   3099  ND1 HIS A 419     -20.592 -33.886  73.503  1.00185.62
N
ANISOU 3099  ND1 HIS A 419    20196  31580  18751  -5951   1451
2768       N
ATOM   3100  CD2 HIS A 419     -20.670 -31.794  74.123  1.00193.15
C
ANISOU 3100  CD2 HIS A 419    21124  32747  19518  -5677   1974
2473       C
ATOM   3101  CE1 HIS A 419     -20.608 -33.870  74.824  1.00181.29
C
ANISOU 3101  CE1 HIS A 419    19634  31250  17997  -6182   1521
2832       C
ATOM   3102  NE2 HIS A 419     -20.656 -32.613  75.226  1.00183.88
N
ANISOU 3102  NE2 HIS A 419    19953  31712  18200  -6032   1840
2649       N
ATOM   3103  N   ARG A 420     -21.231 -29.731  69.861  1.00187.07
N
ANISOU 3103  N   ARG A 420    20236  31552  19291  -4579   2227
2074       N
ATOM   3104  CA  ARG A 420     -20.938 -28.938  68.677  1.00165.70
C
ANISOU 3104  CA  ARG A 420    17574  28646  16736  -4240   2307
1913       C
ATOM   3105  C   ARG A 420     -19.698 -28.088  68.918  1.00180.60
C
ANISOU 3105  C   ARG A 420    19689  30317  18615  -4091   2440
1696       C
ATOM   3106  O   ARG A 420     -19.539 -27.498  69.990  1.00196.44
O
ANISOU 3106  O   ARG A 420    21742  32442  20454  -4149   2648
1627       O
ATOM   3107  CB  ARG A 420     -22.143 -28.065  68.304  1.00146.65
C
ANISOU 3107  CB  ARG A 420    14921  26482  14317  -4019   2571
1920       C
ATOM   3108  CG  ARG A 420     -21.940 -27.158  67.105  1.00151.01
C
ANISOU 3108  CG  ARG A 420    15497  26861  15018  -3661   2676
```

1774 C
ATOM 3109 CD ARG A 420 -23.181 -26.315 66.858 1.00164.12
C
ANISOU 3109 CD ARG A 420 16887 28797 16675 -3445 2951
1832 C
ATOM 3110 NE ARG A 420 -24.333 -27.137 66.503 1.00171.43
N
ANISOU 3110 NE ARG A 420 17555 29972 17610 -3606 2782
2082 N
ATOM 3111 CZ ARG A 420 -25.599 -26.792 66.704 1.00173.52
C
ANISOU 3111 CZ ARG A 420 17518 30587 17823 -3553 2975
2235 C
ATOM 3112 NH1 ARG A 420 -25.896 -25.775 67.499 1.00165.65
N
ANISOU 3112 NH1 ARG A 420 16461 29733 16746 -3385 3362
2165 N
ATOM 3113 NH2 ARG A 420 -26.566 -27.607 66.305 1.00188.78
N
ANISOU 3113 NH2 ARG A 420 19222 32746 19760 -3734 2778
2475 N
ATOM 3114 N VAL A 421 -18.825 -28.031 67.911 1.00139.15
N
ANISOU 3114 N VAL A 421 14587 24752 13530 -3921 2322
1589 N
ATOM 3115 CA VAL A 421 -17.594 -27.251 67.956 1.00122.56
C
ANISOU 3115 CA VAL A 421 12698 22431 11439 -3774 2418
1391 C
ATOM 3116 C VAL A 421 -17.557 -26.360 66.725 1.00111.16
C
ANISOU 3116 C VAL A 421 11268 20835 10134 -3425 2538
1233 C
ATOM 3117 O VAL A 421 -18.168 -26.669 65.697 1.00110.99
O
ANISOU 3117 O VAL A 421 11138 20800 10234 -3341 2436
1298 O
ATOM 3118 CB VAL A 421 -16.336 -28.145 68.021 1.00114.83
C
ANISOU 3118 CB VAL A 421 11901 21195 10534 -3928 2135
1438 C
ATOM 3119 CG1 VAL A 421 -16.216 -28.792 69.393 1.00142.41
C
ANISOU 3119 CG1 VAL A 421 15397 24846 13866 -4260 2057
1585 C
ATOM 3120 CG2 VAL A 421 -16.380 -29.200 66.924 1.00110.67
C
ANISOU 3120 CG2 VAL A 421 11377 20473 10200 -3938 1860
1540 C
ATOM 3121 N SER A 422 -16.837 -25.244 66.827 1.00110.21
N
ANISOU 3121 N SER A 422 11289 20605 9979 -3244 2751
1030 N
ATOM 3122 CA SER A 422 -16.915 -24.230 65.787 1.00116.59

```
C
ANISOU 3122  CA  SER A 422    12101  21305  10893  -2903   2921
881       C
ATOM   3123  C   SER A 422     -15.595 -23.483  65.648  1.00107.23
C
ANISOU 3123  C   SER A 422    11156  19863   9725  -2771   2987
673       C
ATOM   3124  O   SER A 422     -14.861 -23.300  66.622  1.00107.45
O
ANISOU 3124  O   SER A 422    11320  19890   9615  -2918   3039
617       O
ATOM   3125  CB  SER A 422     -18.045 -23.234  66.079  1.00127.15
C
ANISOU 3125  CB  SER A 422    13276  22892  12144  -2744   3274
862       C
ATOM   3126  OG  SER A 422     -18.087 -22.203  65.108  1.00139.29
O
ANISOU 3126  OG  SER A 422    14820  24316  13790  -2402   3452
733       O
ATOM   3127  N   LYS A 423     -15.309 -23.053  64.418  1.00105.53
N
ANISOU 3127  N   LYS A 423    10987  19445   9667  -2514   2978
569       N
ATOM   3128  CA  LYS A 423     -14.240 -22.109  64.124  1.00103.89
C
ANISOU 3128  CA  LYS A 423    10980  19014   9477  -2333   3097
357       C
ATOM   3129  C   LYS A 423     -14.846 -20.853  63.518  1.00104.08
C
ANISOU 3129  C   LYS A 423    10956  19060   9529  -2016   3394
237       C
ATOM   3130  O   LYS A 423     -15.896 -20.906  62.871  1.00115.83
O
ANISOU 3130  O   LYS A 423    12249  20665  11097  -1900   3408
338       O
ATOM   3131  CB  LYS A 423     -13.223 -22.654  63.117  1.00144.05
C
ANISOU 3131  CB  LYS A 423    16189  23802  14741  -2285   2835
330       C
ATOM   3132  CG  LYS A 423     -12.374 -23.817  63.553  1.00140.55
C
ANISOU 3132  CG  LYS A 423    15823  23259  14319  -2535   2557
445       C
ATOM   3133  CD  LYS A 423     -11.345 -24.090  62.464  1.00 99.20
C
ANISOU 3133  CD  LYS A 423    10720  17701   9270  -2412   2387
385       C
ATOM   3134  CE  LYS A 423     -12.020 -24.357  61.129  1.00 98.69
C
ANISOU 3134  CE  LYS A 423    10584  17558   9355  -2271   2309
398       C
ATOM   3135  NZ  LYS A 423     -11.042 -24.658  60.048  1.00 96.84
N
ANISOU 3135  NZ  LYS A 423    10496  17001   9296  -2163   2162
```

```
330        N
ATOM   3136  N   GLY A 424      -14.180 -19.724  63.713  1.00103.59
N
ANISOU 3136  N   GLY A 424    11071  18888   9400  -1884   3630
38        N
ATOM   3137  CA  GLY A 424      -14.516 -18.563  62.913  1.00103.39
C
ANISOU 3137  CA  GLY A 424    11041  18798   9446  -1550   3880
-82        C
ATOM   3138  C   GLY A 424      -13.642 -17.379  63.252  1.00102.96
C
ANISOU 3138  C   GLY A 424    11233  18597   9290  -1459   4134
-312       C
ATOM   3139  O   GLY A 424      -12.714 -17.466  64.063  1.00121.93
O
ANISOU 3139  O   GLY A 424    13809  20958  11563  -1671   4093
-378       O
ATOM   3140  N   ALA A 425      -13.955 -16.265  62.600  1.00106.94
N
ANISOU 3140  N   ALA A 425    11749  19029   9853  -1151   4394
-420       N
ATOM   3141  CA  ALA A 425      -13.324 -14.999  62.933  1.00122.37
C
ANISOU 3141  CA  ALA A 425    13946  20851  11696  -1051   4704
-645       C
ATOM   3142  C   ALA A 425      -13.656 -14.633  64.378  1.00135.79
C
ANISOU 3142  C   ALA A 425    15710  22722  13161  -1226   4986
-679       C
ATOM   3143  O   ALA A 425      -14.801 -14.803  64.812  1.00162.62
O
ANISOU 3143  O   ALA A 425    18914  26340  16533  -1229   5113
-550       O
ATOM   3144  CB  ALA A 425      -13.792 -13.894  61.988  1.00129.08
C
ANISOU 3144  CB  ALA A 425    14774  21605  12664   -672   4959
-719       C
ATOM   3145  N   PRO A 426      -12.689 -14.133  65.150  1.00132.51
N
ANISOU 3145  N   PRO A 426    15563  22224  12560  -1393   5093
-845       N
ATOM   3146  CA  PRO A 426      -12.890 -14.094  66.605  1.00141.35
C
ANISOU 3146  CA  PRO A 426    16756  23526  13426  -1669   5274
-854       C
ATOM   3147  C   PRO A 426      -13.951 -13.097  67.034  1.00151.02
C
ANISOU 3147  C   PRO A 426    17987  24790  14604  -1477   5722
-904       C
ATOM   3148  O   PRO A 426      -14.687 -13.361  67.992  1.00213.13
O
ANISOU 3148  O   PRO A 426    25784  32817  22379  -1613   5807
-807       O
ATOM   3149  CB  PRO A 426      -11.504 -13.694  67.127  1.00139.42
```

```
C
ANISOU 3149  CB  PRO A 426    16818  23153  13004  -1883   5262
-1021       C
ATOM   3150  CG  PRO A 426     -10.565 -14.274  66.138  1.00140.50
C
ANISOU 3150  CG  PRO A 426    16941  23116  13328  -1835   4887
-984       C
ATOM   3151  CD  PRO A 426     -11.259 -14.109  64.797  1.00154.02
C
ANISOU 3151  CD  PRO A 426    18492  24735  15295  -1460   4897
-958       C
ATOM   3152  N   GLU A 427     -14.048 -11.954  66.351  1.00146.25
N
ANISOU 3152  N   GLU A 427    17498  23930  14141  -1111   5945
-1008       N
ATOM   3153  CA  GLU A 427     -15.099 -10.986  66.657  1.00145.02
C
ANISOU 3153  CA  GLU A 427    17365  23685  14051   -825   6316
-983       C
ATOM   3154  C   GLU A 427     -16.493 -11.577  66.471  1.00150.94
C
ANISOU 3154  C   GLU A 427    17734  24718  14897   -741   6357
-768       C
ATOM   3155  O   GLU A 427     -17.385 -11.354  67.299  1.00188.75
O
ANISOU 3155  O   GLU A 427    22492  29572  19652   -716   6594
-692       O
ATOM   3156  CB  GLU A 427     -14.936  -9.744  65.779  1.00151.73
C
ANISOU 3156  CB  GLU A 427    18366  24231  15051   -425   6505
-1081       C
ATOM   3157  CG  GLU A 427     -13.668  -8.948  66.039  1.00164.63
C
ANISOU 3157  CG  GLU A 427    20377  25594  16583   -476   6484
-1277       C
ATOM   3158  CD  GLU A 427     -13.671  -7.605  65.335  1.00175.12
C
ANISOU 3158  CD  GLU A 427    21834  26677  18028    -65   6670
-1347       C
ATOM   3159  OE1 GLU A 427     -12.800  -7.385  64.466  1.00193.00
O
ANISOU 3159  OE1 GLU A 427    24192  28779  20361      2   6518
-1443       O
ATOM   3160  OE2 GLU A 427     -14.547  -6.771  65.648  1.00185.44
O
ANISOU 3160  OE2 GLU A 427    23118  27997  19346    187   6942
-1303       O
ATOM   3161  N   GLN A 428     -16.701 -12.330  65.389  1.00132.58
N
ANISOU 3161  N   GLN A 428    15110  22580  12686   -696   6120
-652       N
ATOM   3162  CA  GLN A 428     -17.957 -13.054  65.204  1.00139.98
C
ANISOU 3162  CA  GLN A 428    15652  23846  13690   -671   6066
```

```
-401       C
ATOM   3163  C   GLN A 428     -18.137 -14.178  66.223  1.00141.80
C
ANISOU 3163  C   GLN A 428    15797  24306  13777  -1052   5867
-279       C
ATOM   3164  O   GLN A 428     -19.235 -14.361  66.762  1.00182.88
O
ANISOU 3164  O   GLN A 428    20794  29737  18953  -1067   6007
-127       O
ATOM   3165  CB  GLN A 428     -18.030 -13.605  63.779  1.00142.96
C
ANISOU 3165  CB  GLN A 428    15836  24181  14302   -526   5717
-267       C
ATOM   3166  CG  GLN A 428     -17.846 -12.549  62.691  1.00137.46
C
ANISOU 3166  CG  GLN A 428    15207  23271  13751   -159   5879
-367       C
ATOM   3167  CD  GLN A 428     -18.479 -11.216  63.044  1.00135.24
C
ANISOU 3167  CD  GLN A 428    15004  22888  13495    123   6376
-424       C
ATOM   3168  OE1 GLN A 428     -17.780 -10.220  63.235  1.00113.95
O
ANISOU 3168  OE1 GLN A 428    12660  19848  10790    218   6577
-628       O
ATOM   3169  NE2 GLN A 428     -19.805 -11.187  63.124  1.00160.46
N
ANISOU 3169  NE2 GLN A 428    17927  26252  16790    260   6525
-217       N
ATOM   3170  N   ILE A 429     -17.079 -14.947  66.493  1.00137.73
N
ANISOU 3170  N   ILE A 429    15432  23711  13188  -1352   5530
-320       N
ATOM   3171  CA  ILE A 429     -17.135 -15.978  67.531  1.00132.19
C
ANISOU 3171  CA  ILE A 429    14682  23205  12340  -1728   5342
-202       C
ATOM   3172  C   ILE A 429     -17.431 -15.369  68.897  1.00116.80
C
ANISOU 3172  C   ILE A 429    12860  21365  10152  -1861   5716
-288       C
ATOM   3173  O   ILE A 429     -18.177 -15.944  69.701  1.00122.69
O
ANISOU 3173  O   ILE A 429    13468  22327  10820  -2039   5710
-146       O
ATOM   3174  CB  ILE A 429     -15.825 -16.788  67.546  1.00136.82
C
ANISOU 3174  CB  ILE A 429    15425  23652  12910  -1988   4943
-222       C
ATOM   3175  CG1 ILE A 429     -15.665 -17.572  66.243  1.00141.37
C
ANISOU 3175  CG1 ILE A 429    15874  24109  13732  -1880   4568
-113       C
ATOM   3176  CG2 ILE A 429     -15.788 -17.733  68.741  1.00119.48
```

```
C
ANISOU 3176  CG2 ILE A 429    13210  21646  10540  -2386   4784
-102       C
ATOM   3177  CD1 ILE A 429     -16.740 -18.613  66.016  1.00110.12
C
ANISOU 3177  CD1 ILE A 429    11620  20356   9864  -1945   4369
135       C
ATOM   3178  N   LEU A 430     -16.871 -14.191  69.175  1.00117.24
N
ANISOU 3178  N   LEU A 430    13246  21113  10186  -1727   5956
-499       N
ATOM   3179  CA  LEU A 430     -17.125 -13.506  70.438  1.00142.37
C
ANISOU 3179  CA  LEU A 430    16650  24203  13243  -1777   6238
-571       C
ATOM   3180  C   LEU A 430     -18.586 -13.095  70.599  1.00156.24
C
ANISOU 3180  C   LEU A 430    18234  26029  15100  -1521   6560
-456       C
ATOM   3181  O   LEU A 430     -19.020 -12.813  71.722  1.00175.38
O
ANISOU 3181  O   LEU A 430    20766  28460  17409  -1599   6776
-461       O
ATOM   3182  CB  LEU A 430     -16.207 -12.285  70.548  1.00131.10
C
ANISOU 3182  CB  LEU A 430    15610  22425  11776  -1661   6408
-797       C
ATOM   3183  CG  LEU A 430     -16.196 -11.460  71.834  1.00128.91
C
ANISOU 3183  CG  LEU A 430    15625  22022  11331  -1733   6669
-900       C
ATOM   3184  CD1 LEU A 430     -15.777 -12.325  73.011  1.00189.88
C
ANISOU 3184  CD1 LEU A 430    23393  29928  18824  -2219   6458
-870       C
ATOM   3185  CD2 LEU A 430     -15.260 -10.274  71.682  1.00126.14
C
ANISOU 3185  CD2 LEU A 430    15616  21352  10961  -1597   6771
-1101       C
ATOM   3186  N   GLU A 431     -19.357 -13.077  69.515  1.00172.38
N
ANISOU 3186  N   GLU A 431    20001  28143  17354  -1227   6592
-336       N
ATOM   3187  CA  GLU A 431     -20.775 -12.747  69.562  1.00160.64
C
ANISOU 3187  CA  GLU A 431    18297  26746  15993   -981   6881
-184       C
ATOM   3188  C   GLU A 431     -21.675 -13.981  69.516  1.00126.09
C
ANISOU 3188  C   GLU A 431    13512  22768  11629  -1150   6641
72       C
ATOM   3189  O   GLU A 431     -22.900 -13.842  69.599  1.00128.71
O
ANISOU 3189  O   GLU A 431    13618  23222  12062   -988   6840
```

```
229       O
ATOM   3190  CB  GLU A 431     -21.110 -11.792  68.406  1.00149.85
C
ANISOU 3190  CB  GLU A 431    16882  25187  14866   -536   7091
-190       C
ATOM   3191  CG  GLU A 431     -22.485 -11.139  68.448  1.00145.64
C
ANISOU 3191  CG  GLU A 431    16187  24648  14503   -245   7477
-66       C
ATOM   3192  CD  GLU A 431     -22.621 -10.024  67.430  1.00142.89
C
ANISOU 3192  CD  GLU A 431    15878  24024  14389    132   7712
-138       C
ATOM   3193  OE1 GLU A 431     -23.453 -10.156  66.508  1.00145.68
O
ANISOU 3193  OE1 GLU A 431    15868  24550  14935    308   7627
30       O
ATOM   3194  OE2 GLU A 431     -21.895  -9.015  67.550  1.00131.71
O
ANISOU 3194  OE2 GLU A 431    14851  22237  12956    258   7949
-331       O
ATOM   3195  N   LEU A 432     -21.102 -15.185  69.412  1.00123.82
N
ANISOU 3195  N   LEU A 432    13129  22677  11242  -1478   6224
135       N
ATOM   3196  CA  LEU A 432     -21.906 -16.402  69.503  1.00124.45
C
ANISOU 3196  CA  LEU A 432    12865  23119  11303  -1686   5984
393       C
ATOM   3197  C   LEU A 432     -22.593 -16.506  70.858  1.00127.55
C
ANISOU 3197  C   LEU A 432    13271  23621  11573  -1856   6152
445       C
ATOM   3198  O   LEU A 432     -23.790 -16.806  70.942  1.00203.03
O
ANISOU 3198  O   LEU A 432    22544  33402  21196  -1808   6205
648       O
ATOM   3199  CB  LEU A 432     -21.032 -17.633  69.264  1.00121.69
C
ANISOU 3199  CB  LEU A 432    12507  22847  10885  -2018   5519
443       C
ATOM   3200  CG  LEU A 432     -20.683 -18.015  67.827  1.00157.28
C
ANISOU 3200  CG  LEU A 432    16950  27184  15624  -1866   5176
492       C
ATOM   3201  CD1 LEU A 432     -19.777 -19.234  67.824  1.00156.26
C
ANISOU 3201  CD1 LEU A 432    16918  26962  15492  -2178   4706
531       C
ATOM   3202  CD2 LEU A 432     -21.945 -18.278  67.019  1.00148.55
C
ANISOU 3202  CD2 LEU A 432    15483  26279  14681  -1694   5134
730       C
ATOM   3203  N   ALA A 433     -21.846 -16.265  71.932  1.00127.97
```

```
N
ANISOU 3203  N   ALA A 433    13651  23529  11444  -2066   6226
272       N
ATOM   3204  CA  ALA A 433     -22.407 -15.868  73.219  1.00170.93
C
ANISOU 3204  CA  ALA A 433    19212  28960  16772  -2128   6520
245       C
ATOM   3205  C   ALA A 433     -21.670 -14.610  73.650  1.00161.16
C
ANISOU 3205  C   ALA A 433    18392  27369  15472  -2003   6819
-10       C
ATOM   3206  O   ALA A 433     -20.476 -14.668  73.962  1.00130.29
O
ANISOU 3206  O   ALA A 433    14738  23346  11418  -2235   6645
-153       O
ATOM   3207  CB  ALA A 433     -22.264 -16.976  74.262  1.00133.99
C
ANISOU 3207  CB  ALA A 433    14516  24504  11889  -2589   6260
331       C
ATOM   3208  N   LYS A 434     -22.372 -13.475  73.649  1.00173.60
N
ANISOU 3208  N   LYS A 434    20032  28770  17158  -1636   7260
-42       N
ATOM   3209  CA  LYS A 434     -21.716 -12.198  73.900  1.00163.12
C
ANISOU 3209  CA  LYS A 434    19096  27100  15783  -1457   7541
-252       C
ATOM   3210  C   LYS A 434     -21.070 -12.224  75.277  1.00163.77
C
ANISOU 3210  C   LYS A 434    19474  27162  15588  -1816   7515
-377       C
ATOM   3211  O   LYS A 434     -21.759 -12.181  76.302  1.00165.42
O
ANISOU 3211  O   LYS A 434    19696  27461  15695  -1892   7720
-332       O
ATOM   3212  CB  LYS A 434     -22.718 -11.047  73.783  1.00171.09
C
ANISOU 3212  CB  LYS A 434    20100  27963  16946   -990   8039
-202       C
ATOM   3213  CG  LYS A 434     -22.132  -9.669  74.045  1.00172.28
C
ANISOU 3213  CG  LYS A 434    20612  27835  17011   -752   8298
-374       C
ATOM   3214  CD  LYS A 434     -23.151  -8.577  73.753  1.00173.34
C
ANISOU 3214  CD  LYS A 434    20638  27943  17281   -218   8722
-256       C
ATOM   3215  CE  LYS A 434     -22.575  -7.196  74.014  1.00163.41
C
ANISOU 3215  CE  LYS A 434    19636  26592  15862    -46   8838
-479       C
ATOM   3216  NZ  LYS A 434     -23.568  -6.119  73.744  1.00169.52
N
ANISOU 3216  NZ  LYS A 434    20148  27591  16672    347   9095
```

```
-478       N
ATOM   3217  N   ALA A 435     -19.743 -12.295  75.302  1.00133.95
N
ANISOU 3217  N   ALA A 435    15926  23275  11692  -2050   7260
-527       N
ATOM   3218  CA  ALA A 435     -19.035 -12.649  76.517  1.00146.26
C
ANISOU 3218  CA  ALA A 435    17695  24885  12991  -2496   7109
-603       C
ATOM   3219  C   ALA A 435     -18.906 -11.446  77.448  1.00161.76
C
ANISOU 3219  C   ALA A 435    20009  26641  14812  -2450   7442
-773       C
ATOM   3220  O   ALA A 435     -19.250 -10.311  77.105  1.00159.18
O
ANISOU 3220  O   ALA A 435    19775  26122  14583  -2056   7767
-836       O
ATOM   3221  CB  ALA A 435     -17.656 -13.214  76.183  1.00131.30
C
ANISOU 3221  CB  ALA A 435    15883  22974  11031  -2775   6704
-662       C
ATOM   3222  N   SER A 436     -18.415 -11.722  78.653  1.00157.11
N
ANISOU 3222  N   SER A 436    19600  26116  13977  -2872   7340
-834       N
ATOM   3223  CA  SER A 436     -18.019 -10.664  79.571  1.00149.14
C
ANISOU 3223  CA  SER A 436    18956  24920  12790  -2943   7570
-1024       C
ATOM   3224  C   SER A 436     -16.976  -9.769  78.914  1.00141.19
C
ANISOU 3224  C   SER A 436    18188  23638  11822  -2811   7546
-1198       C
ATOM   3225  O   SER A 436     -16.125 -10.236  78.153  1.00174.22
O
ANISOU 3225  O   SER A 436    22322  27808  16067  -2886   7238
-1192       O
ATOM   3226  CB  SER A 436     -17.470 -11.264  80.865  1.00159.34
C
ANISOU 3226  CB  SER A 436    20384  26350  13809  -3493   7371
-1050       C
ATOM   3227  OG  SER A 436     -17.043 -10.250  81.758  1.00182.97
O
ANISOU 3227  OG  SER A 436    23738  29167  16615  -3614   7575
-1245       O
ATOM   3228  N   ASN A 437     -17.044  -8.470  79.210  1.00145.89
N
ANISOU 3228  N   ASN A 437    19036  24019  12375  -2619   7870
-1350       N
ATOM   3229  CA  ASN A 437     -16.118  -7.535  78.583  1.00152.54
C
ANISOU 3229  CA  ASN A 437    20103  24596  13259  -2485   7858
-1516       C
ATOM   3230  C   ASN A 437     -14.705  -7.678  79.132  1.00180.93
```

```
C
ANISOU 3230  C   ASN A 437    23952  28139  16653  -2961   7579
-1643       C
ATOM   3231  O   ASN A 437     -13.754  -7.230  78.486  1.00194.09
O
ANISOU 3231  O   ASN A 437    25751  29631  18364  -2922   7458
-1744       O
ATOM   3232  CB  ASN A 437     -16.612  -6.101  78.772  1.00148.77
C
ANISOU 3232  CB  ASN A 437    19813  23926  12788  -2175   8265
-1642       C
ATOM   3233  CG  ASN A 437     -17.856  -5.804  77.960  1.00153.95
C
ANISOU 3233  CG  ASN A 437    20184  24636  13673  -1641   8512
-1503       C
ATOM   3234  OD1 ASN A 437     -18.411  -6.689  77.310  1.00143.40
O
ANISOU 3234  OD1 ASN A 437    18520  23477  12488  -1519   8399
-1306       O
ATOM   3235  ND2 ASN A 437     -18.302  -4.554  77.994  1.00163.69
N
ANISOU 3235  ND2 ASN A 437    21529  25734  14932  -1346   8840
-1603       N
ATOM   3236  N   ASP A 438     -14.552  -8.290  80.308  1.00204.98
N
ANISOU 3236  N   ASP A 438    27054  31352  19478  -3414   7469
-1625       N
ATOM   3237  CA  ASP A 438     -13.242  -8.757  80.753  1.00218.38
C
ANISOU 3237  CA  ASP A 438    28881  33098  20994  -3899   7120
-1663       C
ATOM   3238  C   ASP A 438     -12.660  -9.791  79.792  1.00220.90
C
ANISOU 3238  C   ASP A 438    28958  33535  21437  -3926   6738
-1526       C
ATOM   3239  O   ASP A 438     -11.483  -9.719  79.421  1.00256.54
O
ANISOU 3239  O   ASP A 438    33575  37968  25930  -4056   6517
-1581       O
ATOM   3240  CB  ASP A 438     -13.355  -9.334  82.166  1.00228.97
C
ANISOU 3240  CB  ASP A 438    30265  34646  22087  -4364   7059
-1624       C
ATOM   3241  CG  ASP A 438     -12.010  -9.698  82.759  1.00238.30
C
ANISOU 3241  CG  ASP A 438    31588  35899  23058  -4887   6717
-1646       C
ATOM   3242  OD1 ASP A 438     -10.994  -9.091  82.358  1.00231.44
O
ANISOU 3242  OD1 ASP A 438    30896  34856  22187  -4907   6642
-1759       O
ATOM   3243  OD2 ASP A 438     -11.969 -10.593  83.630  1.00253.59
O
ANISOU 3243  OD2 ASP A 438    33443  38077  24831  -5282   6515
```

```
-1533       O
ATOM   3244  N   LEU A 439     -13.476 -10.768  79.385  1.00199.76
N
ANISOU 3244  N   LEU A 439    25953  31060  18888  -3815   6657
-1342       N
ATOM   3245  CA  LEU A 439     -13.067 -11.726  78.358  1.00187.26
C
ANISOU 3245  CA  LEU A 439    24123  29582  17444  -3794   6329
-1212       C
ATOM   3246  C   LEU A 439     -12.795 -11.052  77.016  1.00151.79
C
ANISOU 3246  C   LEU A 439    19648  24862  13164  -3391   6393
-1295       C
ATOM   3247  O   LEU A 439     -11.803 -11.362  76.347  1.00126.15
O
ANISOU 3247  O   LEU A 439    16390  21588   9955  -3460   6129
-1295       O
ATOM   3248  CB  LEU A 439     -14.145 -12.804  78.203  1.00163.28
C
ANISOU 3248  CB  LEU A 439    20735  26803  14502  -3752   6265
-1005       C
ATOM   3249  CG  LEU A 439     -13.806 -14.104  77.468  1.00127.93
C
ANISOU 3249  CG  LEU A 439    15978  22522  10108  -3868   5874
-826       C
ATOM   3250  CD1 LEU A 439     -14.599 -15.264  78.049  1.00148.51
C
ANISOU 3250  CD1 LEU A 439    18331  25434  12663  -4087   5739
-618       C
ATOM   3251  CD2 LEU A 439     -14.080 -13.976  75.978  1.00125.41
C
ANISOU 3251  CD2 LEU A 439    15492  22111  10047  -3455   5908
-818       C
ATOM   3252  N   SER A 440     -13.669 -10.133  76.607  1.00130.23
N
ANISOU 3252  N   SER A 440    16932  21976  10574  -2962   6740
-1349       N
ATOM   3253  CA  SER A 440     -13.476  -9.384  75.367  1.00128.34
C
ANISOU 3253  CA  SER A 440    16716  21514  10534  -2561   6816
-1421       C
ATOM   3254  C   SER A 440     -12.153  -8.620  75.333  1.00164.04
C
ANISOU 3254  C   SER A 440    21543  25813  14972  -2673   6735
-1599       C
ATOM   3255  O   SER A 440     -11.422  -8.671  74.338  1.00180.13
O
ANISOU 3255  O   SER A 440    23557  27761  17122  -2577   6557
-1619       O
ATOM   3256  CB  SER A 440     -14.648  -8.423  75.165  1.00130.70
C
ANISOU 3256  CB  SER A 440    16991  21709  10958  -2108   7218
-1425       C
ATOM   3257  OG  SER A 440     -14.516  -7.699  73.955  1.00129.04
```

```
O
ANISOU 3257  OG  SER A 440    16780  21308  10942  -1714   7276
-1472       O
ATOM   3258  N   LYS A 441     -11.829  -7.899  76.404  1.00169.20
N
ANISOU 3258  N   LYS A 441    22483  26378  15426  -2891   6865
-1728       N
ATOM   3259  CA  LYS A 441     -10.525  -7.241  76.495  1.00173.82
C
ANISOU 3259  CA  LYS A 441    23352  26786  15905  -3085   6761
-1882       C
ATOM   3260  C   LYS A 441      -9.343  -8.213  76.465  1.00175.16
C
ANISOU 3260  C   LYS A 441    23465  27098  15988  -3466   6353
-1813       C
ATOM   3261  O   LYS A 441      -8.341  -7.949  75.791  1.00191.03
O
ANISOU 3261  O   LYS A 441    25554  28984  18043  -3450   6206
-1873       O
ATOM   3262  CB  LYS A 441     -10.469  -6.390  77.764  1.00187.08
C
ANISOU 3262  CB  LYS A 441    25338  28380  17363  -3321   6971
-2024       C
ATOM   3263  CG  LYS A 441     -11.373  -5.167  77.732  1.00201.23
C
ANISOU 3263  CG  LYS A 441    27245  29980  19233  -2938   7384
-2124       C
ATOM   3264  CD  LYS A 441     -11.345  -4.415  79.054  1.00210.44
C
ANISOU 3264  CD  LYS A 441    28720  31073  20165  -3219   7598
-2270       C
ATOM   3265  CE  LYS A 441      -9.953  -3.904  79.381  1.00207.68
C
ANISOU 3265  CE  LYS A 441    28672  30584  19653  -3586   7448
-2421       C
ATOM   3266  NZ  LYS A 441      -9.468  -2.924  78.372  1.00206.37
N
ANISOU 3266  NZ  LYS A 441    28620  30153  19637  -3292   7496
-2528       N
ATOM   3267  N   LYS A 442      -9.429  -9.330  77.189  1.00171.56
N
ANISOU 3267  N   LYS A 442    22861  26914  15408  -3807   6160
-1672       N
ATOM   3268  CA  LYS A 442      -8.376 -10.347  77.124  1.00155.15
C
ANISOU 3268  CA  LYS A 442    20676  25012  13260  -4143   5759
-1553       C
ATOM   3269  C   LYS A 442      -8.174 -10.951  75.733  1.00175.58
C
ANISOU 3269  C   LYS A 442    23036  27609  16067  -3897   5579
-1466       C
ATOM   3270  O   LYS A 442      -7.038 -11.038  75.253  1.00199.57
O
ANISOU 3270  O   LYS A 442    26111  30611  19107  -3989   5364
```

```
-1470       O
ATOM   3271  CB  LYS A 442      -8.655 -11.452  78.147  1.00150.45
C
ANISOU 3271  CB  LYS A 442    19936  24720  12507  -4522   5589
-1384       C
ATOM   3272  CG  LYS A 442      -8.294 -11.060  79.569  1.00164.13
C
ANISOU 3272  CG  LYS A 442    21911  26485  13968  -4933   5624
-1455       C
ATOM   3273  CD  LYS A 442      -6.779 -10.968  79.703  1.00158.09
C
ANISOU 3273  CD  LYS A 442    21282  25713  13070  -5260   5364
-1469       C
ATOM   3274  CE  LYS A 442      -6.339 -10.720  81.134  1.00155.11
C
ANISOU 3274  CE  LYS A 442    21114  25417  12405  -5744   5338
-1510       C
ATOM   3275  NZ  LYS A 442      -4.852 -10.670  81.239  1.00151.99
N
ANISOU 3275  NZ  LYS A 442    20812  25053  11883  -6078   5064
-1488       N
ATOM   3276  N   VAL A 443      -9.249 -11.378  75.068  1.00156.36
N
ANISOU 3276  N   VAL A 443    20360  25236  13814  -3598   5661
-1381       N
ATOM   3277  CA  VAL A 443      -9.102 -11.930  73.717  1.00142.10
C
ANISOU 3277  CA  VAL A 443    18343  23438  12211  -3376   5500
-1313       C
ATOM   3278  C   VAL A 443      -8.577 -10.899  72.716  1.00116.13
C
ANISOU 3278  C   VAL A 443    15209  19856   9059  -3059   5601
-1474       C
ATOM   3279  O   VAL A 443      -7.679 -11.198  71.922  1.00130.17
O
ANISOU 3279  O   VAL A 443    16952  21606  10900  -3064   5390
-1467       O
ATOM   3280  CB  VAL A 443     -10.421 -12.576  73.247  1.00144.15
C
ANISOU 3280  CB  VAL A 443    18299  23846  12624  -3157   5566
-1181       C
ATOM   3281  CG1 VAL A 443     -10.828 -13.683  74.208  1.00123.39
C
ANISOU 3281  CG1 VAL A 443    15509  21516   9857  -3503   5414
-1003       C
ATOM   3282  CG2 VAL A 443     -11.538 -11.552  73.111  1.00150.20
C
ANISOU 3282  CG2 VAL A 443    19110  24457  13503  -2768   5951
-1263       C
ATOM   3283  N   LEU A 444      -9.110  -9.679  72.735  1.00117.85
N
ANISOU 3283  N   LEU A 444    15597  19856   9325  -2778   5920
-1607       N
ATOM   3284  CA  LEU A 444      -8.604  -8.630  71.848  1.00116.71
```

```
C
ANISOU 3284  CA  LEU A 444    15612  19431   9301  -2487   6005
-1747       C
ATOM   3285  C   LEU A 444      -7.132  -8.294  72.083  1.00146.35
C
ANISOU 3285  C   LEU A 444    19597  23090  12919  -2765   5835
-1844       C
ATOM   3286  O   LEU A 444      -6.392  -8.040  71.125  1.00186.15
O
ANISOU 3286  O   LEU A 444    24669  27991  18069  -2624   5735
-1897       O
ATOM   3287  CB  LEU A 444      -9.462  -7.372  71.977  1.00119.26
C
ANISOU 3287  CB  LEU A 444    16070  19568   9675  -2161   6373
-1840       C
ATOM   3288  CG  LEU A 444     -10.893  -7.497  71.453  1.00131.73
C
ANISOU 3288  CG  LEU A 444    17398  21219  11435  -1787   6566
-1727       C
ATOM   3289  CD1 LEU A 444     -11.711  -6.276  71.836  1.00160.26
C
ANISOU 3289  CD1 LEU A 444    21137  24703  15051  -1517   6932
-1792       C
ATOM   3290  CD2 LEU A 444     -10.896  -7.699  69.948  1.00131.15
C
ANISOU 3290  CD2 LEU A 444    17140  21096  11597  -1476   6463
-1684       C
ATOM   3291  N   SER A 445      -6.686  -8.278  73.340  1.00145.65
N
ANISOU 3291  N   SER A 445    19665  23084  12591  -3171   5797
-1861       N
ATOM   3292  CA  SER A 445      -5.268  -8.055  73.625  1.00145.11
C
ANISOU 3292  CA  SER A 445    19779  22980  12375  -3492   5609
-1916       C
ATOM   3293  C   SER A 445      -4.346  -9.098  72.991  1.00141.92
C
ANISOU 3293  C   SER A 445    19192  22726  12006  -3628   5265
-1786       C
ATOM   3294  O   SER A 445      -3.338  -8.743  72.369  1.00174.87
O
ANISOU 3294  O   SER A 445    23449  26779  16213  -3606   5161
-1842       O
ATOM   3295  CB  SER A 445      -5.048  -8.013  75.138  1.00147.24
C
ANISOU 3295  CB  SER A 445    20206  23367  12371  -3948   5606
-1923       C
ATOM   3296  OG  SER A 445      -3.697  -7.718  75.450  1.00148.45
O
ANISOU 3296  OG  SER A 445    20530  23502  12371  -4279   5433
-1966       O
ATOM   3297  N   ILE A 446      -4.662 -10.387  73.132  1.00126.25
N
ANISOU 3297  N   ILE A 446    16952  21006  10011  -3770   5086
```

```
-1598       N
ATOM   3298  CA  ILE A 446      -3.879 -11.412  72.437  1.00111.75
C
ANISOU 3298  CA  ILE A 446    14919  19311   8228  -3852   4781
-1450       C
ATOM   3299  C   ILE A 446      -4.058 -11.357  70.916  1.00116.53
C
ANISOU 3299  C   ILE A 446    15416  19770   9091  -3428   4817
-1501       C
ATOM   3300  O   ILE A 446      -3.097 -11.576  70.168  1.00119.80
O
ANISOU 3300  O   ILE A 446    15801  20158   9558  -3421   4644
-1483       O
ATOM   3301  CB  ILE A 446      -4.167 -12.809  73.024  1.00112.07
C
ANISOU 3301  CB  ILE A 446    14718  19676   8188  -4129   4571
-1208       C
ATOM   3302  CG1 ILE A 446      -5.656 -13.139  73.017  1.00122.51
C
ANISOU 3302  CG1 ILE A 446    15878  21066   9606  -3935   4731
-1174       C
ATOM   3303  CG2 ILE A 446      -3.603 -12.923  74.435  1.00114.60
C
ANISOU 3303  CG2 ILE A 446    15146  20152   8245  -4603   4450
-1132       C
ATOM   3304  CD1 ILE A 446      -5.955 -14.568  73.414  1.00123.09
C
ANISOU 3304  CD1 ILE A 446    15690  21446   9631  -4180   4501
-918       C
ATOM   3305  N   ILE A 447      -5.271 -11.082  70.428  1.00125.71
N
ANISOU 3305  N   ILE A 447    16503  20847  10415  -3075   5035
-1550       N
ATOM   3306  CA  ILE A 447      -5.465 -10.865  68.988  1.00109.51
C
ANISOU 3306  CA  ILE A 447    14368  18636   8604  -2669   5082
-1609       C
ATOM   3307  C   ILE A 447      -4.564  -9.752  68.458  1.00105.86
C
ANISOU 3307  C   ILE A 447    14144  17895   8184  -2527   5128
-1773       C
ATOM   3308  O   ILE A 447      -3.859  -9.927  67.457  1.00119.28
O
ANISOU 3308  O   ILE A 447    15799  19529   9992  -2426   4990
-1787       O
ATOM   3309  CB  ILE A 447      -6.944 -10.578  68.669  1.00107.45
C
ANISOU 3309  CB  ILE A 447    13996  18339   8493  -2324   5330
-1612       C
ATOM   3310  CG1 ILE A 447      -7.812 -11.814  68.902  1.00112.37
C
ANISOU 3310  CG1 ILE A 447    14325  19259   9110  -2443   5240
-1430       C
ATOM   3311  CG2 ILE A 447      -7.099 -10.085  67.242  1.00105.52
```

```
C
ANISOU 3311  CG2 ILE A 447    13710  17899   8485  -1904   5397
-1682       C
ATOM   3312  CD1 ILE A 447      -9.254 -11.487  69.217  1.00143.73
C
ANISOU 3312  CD1 ILE A 447    18221  23259  13133  -2246   5507
-1404       C
ATOM   3313  N   ASP A 448      -4.569  -8.595  69.120  1.00108.07
N
ANISOU 3313  N   ASP A 448    14678  18010   8375  -2528   5322
-1895       N
ATOM   3314  CA  ASP A 448      -3.644  -7.522  68.756  1.00111.92
C
ANISOU 3314  CA  ASP A 448    15401  18255   8871  -2459   5343
-2037       C
ATOM   3315  C   ASP A 448      -2.184  -7.897  68.976  1.00129.23
C
ANISOU 3315  C   ASP A 448    17647  20534  10922  -2822   5082
-2006       C
ATOM   3316  O   ASP A 448      -1.317  -7.462  68.211  1.00155.24
O
ANISOU 3316  O   ASP A 448    21019  23680  14285  -2729   5011
-2074       O
ATOM   3317  CB  ASP A 448      -3.989  -6.241  69.516  1.00117.86
C
ANISOU 3317  CB  ASP A 448    16408  18839   9534  -2427   5605
-2163       C
ATOM   3318  CG  ASP A 448      -5.265  -5.595  69.009  1.00127.92
C
ANISOU 3318  CG  ASP A 448    17635  19984  10983  -1975   5875
-2189       C
ATOM   3319  OD1 ASP A 448      -5.736  -4.624  69.637  1.00161.30
O
ANISOU 3319  OD1 ASP A 448    22033  24104  15148  -1919   6117
-2271       O
ATOM   3320  OD2 ASP A 448      -5.795  -6.055  67.977  1.00141.25
O
ANISOU 3320  OD2 ASP A 448    19105  21694  12868  -1685   5846
-2118       O
ATOM   3321  N   LYS A 449      -1.884  -8.682  70.010  1.00126.02
N
ANISOU 3321  N   LYS A 449    17189  20378  10316  -3236   4931
-1886       N
ATOM   3322  CA  LYS A 449      -0.523  -9.190  70.163  1.00131.68
C
ANISOU 3322  CA  LYS A 449    17892  21236  10906  -3574   4657
-1791       C
ATOM   3323  C   LYS A 449      -0.122 -10.108  69.013  1.00139.00
C
ANISOU 3323  C   LYS A 449    18594  22234  11986  -3436   4468
-1687       C
ATOM   3324  O   LYS A 449       1.005 -10.020  68.512  1.00152.19
O
ANISOU 3324  O   LYS A 449    20298  23869  13659  -3483   4333
```

```
-1685       O
ATOM   3325  CB  LYS A 449      -0.380  -9.914  71.501  1.00168.79
C
ANISOU 3325  CB  LYS A 449    22544  26218  15369  -4036   4515
-1634       C
ATOM   3326  CG  LYS A 449       1.042 -10.346  71.807  1.00170.54
C
ANISOU 3326  CG  LYS A 449    22746  26613  15438  -4413   4229
-1491       C
ATOM   3327  CD  LYS A 449       1.979  -9.152  71.889  1.00147.75
C
ANISOU 3327  CD  LYS A 449    20121  23548  12468  -4503   4276
-1645       C
ATOM   3328  CE  LYS A 449       3.403  -9.593  72.188  1.00148.85
C
ANISOU 3328  CE  LYS A 449    20207  23896  12452  -4887   3983
-1468       C
ATOM   3329  NZ  LYS A 449       4.324  -8.435  72.341  1.00156.70
N
ANISOU 3329  NZ  LYS A 449    21449  24746  13344  -5029   4018
-1609       N
ATOM   3330  N   TYR A 450      -1.018 -10.997  68.579  1.00128.48
N
ANISOU 3330  N   TYR A 450    17029  21011  10777  -3279   4460
-1595       N
ATOM   3331  CA  TYR A 450      -0.734 -11.787  67.382  1.00118.01
C
ANISOU 3331  CA  TYR A 450    15505  19721   9613  -3115   4319
-1526       C
ATOM   3332  C   TYR A 450      -0.529 -10.907  66.154  1.00143.05
C
ANISOU 3332  C   TYR A 450    18781  22595  12976  -2742   4423
-1710       C
ATOM   3333  O   TYR A 450       0.349 -11.179  65.328  1.00107.42
O
ANISOU 3333  O   TYR A 450    14216  17999   8598  -2661   4234
-1675       O
ATOM   3334  CB  TYR A 450      -1.860 -12.789  67.112  1.00111.87
C
ANISOU 3334  CB  TYR A 450    14462  18982   9060  -2933   4212
-1375       C
ATOM   3335  CG  TYR A 450      -2.092 -13.839  68.177  1.00112.33
C
ANISOU 3335  CG  TYR A 450    14376  19281   9023  -3243   4028
-1144       C
ATOM   3336  CD1 TYR A 450      -1.267 -13.941  69.288  1.00115.75
C
ANISOU 3336  CD1 TYR A 450    14891  19896   9192  -3665   3931
-1052       C
ATOM   3337  CD2 TYR A 450      -3.140 -14.743  68.056  1.00109.78
C
ANISOU 3337  CD2 TYR A 450    13830  19014   8867  -3131   3938
-1004       C
ATOM   3338  CE1 TYR A 450      -1.479 -14.915  70.249  1.00112.83
```

```
C
ANISOU 3338  CE1 TYR A 450    14383  19749   8737  -3952   3752
-822       C
ATOM   3339  CE2 TYR A 450      -3.361 -15.714  69.011  1.00108.73
C
ANISOU 3339  CE2 TYR A 450    13569  19094   8650  -3415   3767
-788       C
ATOM   3340  CZ  TYR A 450      -2.529 -15.797  70.106  1.00104.81
C
ANISOU 3340  CZ  TYR A 450    13156  18767   7900  -3818   3676
-697       C
ATOM   3341  OH  TYR A 450      -2.754 -16.758  71.067  1.00144.13
O
ANISOU 3341  OH  TYR A 450    18007  23964  12792  -4108   3503
-468       O
ATOM   3342  N   ALA A 451      -1.334  -9.851  66.013  1.00174.87
N
ANISOU 3342  N   ALA A 451    22943  26402  17098  -2461   4662
-1858       N
ATOM   3343  CA  ALA A 451      -1.153  -8.916  64.906  1.00148.40
C
ANISOU 3343  CA  ALA A 451    19700  22760  13924  -2109   4749
-2002       C
ATOM   3344  C   ALA A 451       0.196  -8.206  64.957  1.00117.36
C
ANISOU 3344  C   ALA A 451    15976  18710   9905  -2251   4673
-2077       C
ATOM   3345  O   ALA A 451       0.863  -8.055  63.927  1.00113.74
O
ANISOU 3345  O   ALA A 451    15523  18122   9571  -2089   4599
-2127       O
ATOM   3346  CB  ALA A 451      -2.290  -7.894  64.897  1.00139.15
C
ANISOU 3346  CB  ALA A 451    18619  21409  12843  -1800   5018
-2088       C
ATOM   3347  N   GLU A 452       0.617  -7.758  66.143  1.00133.44
N
ANISOU 3347  N   GLU A 452    18182  20796  11725  -2569   4688
-2085       N
ATOM   3348  CA  GLU A 452       1.980  -7.258  66.300  1.00146.06
C
ANISOU 3348  CA  GLU A 452    19938  22355  13202  -2795   4578
-2118       C
ATOM   3349  C   GLU A 452       3.025  -8.322  65.982  1.00144.36
C
ANISOU 3349  C   GLU A 452    19553  22346  12952  -2992   4318
-1971       C
ATOM   3350  O   GLU A 452       4.044  -8.026  65.347  1.00141.44
O
ANISOU 3350  O   GLU A 452    19233  21895  12614  -2970   4233
-2003       O
ATOM   3351  CB  GLU A 452       2.179  -6.725  67.718  1.00173.28
C
ANISOU 3351  CB  GLU A 452    23573  25864  16402  -3162   4627
```

```
-2134       C
ATOM   3352  CG  GLU A 452       3.572  -6.181  67.989  1.00188.40
C
ANISOU 3352  CG  GLU A 452    25647  27771  18166  -3455   4509
-2156       C
ATOM   3353  CD  GLU A 452       3.697  -5.571  69.371  1.00200.47
C
ANISOU 3353  CD  GLU A 452    27379  29342  19451  -3829   4574
-2194       C
ATOM   3354  OE1 GLU A 452       3.683  -6.331  70.363  1.00177.96
O
ANISOU 3354  OE1 GLU A 452    24442  26747  16425  -4171   4465
-2060       O
ATOM   3355  OE2 GLU A 452       3.807  -4.331  69.466  1.00201.42
O
ANISOU 3355  OE2 GLU A 452    27742  29237  19550  -3792   4731
-2354       O
ATOM   3356  N   ARG A 453       2.796  -9.564  66.417  1.00155.06
N
ANISOU 3356  N   ARG A 453    20696  23979  14239  -3182   4190
-1787       N
ATOM   3357  CA  ARG A 453       3.639 -10.670  65.969  1.00171.59
C
ANISOU 3357  CA  ARG A 453    22578  26234  16385  -3270   3926
-1592       C
ATOM   3358  C   ARG A 453       3.581 -10.838  64.457  1.00131.04
C
ANISOU 3358  C   ARG A 453    17334  20841  11615  -2821   3855
-1632       C
ATOM   3359  O   ARG A 453       4.599 -11.124  63.815  1.00 94.31
O
ANISOU 3359  O   ARG A 453    12613  16111   7112  -2763   3646
-1545       O
ATOM   3360  CB  ARG A 453       3.233 -11.968  66.671  1.00183.84
C
ANISOU 3360  CB  ARG A 453    23891  27973  17987  -3421   3697
-1319       C
ATOM   3361  CG  ARG A 453       3.493 -12.008  68.182  1.00179.64
C
ANISOU 3361  CG  ARG A 453    23428  27730  17099  -3919   3683
-1214       C
ATOM   3362  CD  ARG A 453       4.979 -12.096  68.550  1.00181.85
C
ANISOU 3362  CD  ARG A 453    23705  28139  17250  -4234   3451
-1046       C
ATOM   3363  NE  ARG A 453       5.707 -10.848  68.322  1.00203.17
N
ANISOU 3363  NE  ARG A 453    26659  30733  19805  -4275   3606
-1261       N
ATOM   3364  CZ  ARG A 453       6.949 -10.773  67.856  1.00197.71
C
ANISOU 3364  CZ  ARG A 453    25945  30045  19129  -4324   3453
-1187       C
ATOM   3365  NH1 ARG A 453       7.699 -11.861  67.779  1.00188.55
```

```
N
ANISOU 3365  NH1 ARG A 453    24513  28968  18159  -4352   3102
-863       N
ATOM   3366  NH2 ARG A 453       7.508  -9.584  67.659  1.00173.60
N
ANISOU 3366  NH2 ARG A 453    23121  26815  16024  -4313   3556
-1384       N
ATOM   3367  N   GLY A 454       2.400 -10.666  63.871  1.00131.93
N
ANISOU 3367  N   GLY A 454    17424  20833  11869  -2508   4031
-1749       N
ATOM   3368  CA  GLY A 454       2.265 -10.662  62.430  1.00144.89
C
ANISOU 3368  CA  GLY A 454    18998  22239  13812  -2107   4000
-1813       C
ATOM   3369  C   GLY A 454       1.405 -11.809  61.952  1.00108.01
C
ANISOU 3369  C   GLY A 454    14075  17555   9408  -1917   3830
-1655       C
ATOM   3370  O   GLY A 454       1.509 -12.243  60.801  1.00101.02
O
ANISOU 3370  O   GLY A 454    13090  16504   8791  -1663   3688
-1627       O
ATOM   3371  N   LEU A 455       0.552 -12.309  62.840  1.00 96.07
N
ANISOU 3371  N   LEU A 455    12474  16222   7808  -2064   3847
-1554       N
ATOM   3372  CA  LEU A 455      -0.128 -13.581  62.660  1.00 93.57
C
ANISOU 3372  CA  LEU A 455    11916  15942   7694  -2000   3637
-1356       C
ATOM   3373  C   LEU A 455      -1.630 -13.350  62.730  1.00 95.56
C
ANISOU 3373  C   LEU A 455    12121  16253   7936  -1865   3855
-1416       C
ATOM   3374  O   LEU A 455      -2.111 -12.597  63.583  1.00 94.48
O
ANISOU 3374  O   LEU A 455    12102  16229   7567  -1979   4123
-1519       O
ATOM   3375  CB  LEU A 455       0.303 -14.582  63.737  1.00 93.08
C
ANISOU 3375  CB  LEU A 455    11751  16073   7543  -2339   3406
-1116       C
ATOM   3376  CG  LEU A 455       1.813 -14.700  63.967  1.00 93.04
C
ANISOU 3376  CG  LEU A 455    11780  16081   7490  -2534   3221
-1016       C
ATOM   3377  CD1 LEU A 455       2.120 -15.606  65.148  1.00 94.74
C
ANISOU 3377  CD1 LEU A 455    11884  16522   7591  -2884   3018
-754       C
ATOM   3378  CD2 LEU A 455       2.508 -15.195  62.716  1.00147.30
C
ANISOU 3378  CD2 LEU A 455    18570  22731  14666  -2284   3030
```

```
-963       C
ATOM   3379  N   ARG A 456      -2.372 -13.990  61.833  1.00 99.06
N
ANISOU 3379  N   ARG A 456    12394  16622   8623  -1632   3751
-1344       N
ATOM   3380  CA  ARG A 456      -3.814 -13.843  61.894  1.00107.60
C
ANISOU 3380  CA  ARG A 456    13384  17795   9705  -1511   3935
-1354       C
ATOM   3381  C   ARG A 456      -4.399 -14.755  62.967  1.00 94.24
C
ANISOU 3381  C   ARG A 456    11556  16328   7921  -1763   3841
-1171       C
ATOM   3382  O   ARG A 456      -3.778 -15.727  63.406  1.00 94.21
O
ANISOU 3382  O   ARG A 456    11491  16378   7927  -1981   3577
-1004       O
ATOM   3383  CB  ARG A 456      -4.449 -14.131  60.533  1.00145.00
C
ANISOU 3383  CB  ARG A 456    17986  22402  14705  -1202   3860
-1333       C
ATOM   3384  CG  ARG A 456      -4.436 -15.589  60.119  1.00137.04
C
ANISOU 3384  CG  ARG A 456    16805  21376  13887  -1250   3520
-1132       C
ATOM   3385  CD  ARG A 456      -5.220 -15.843  58.829  1.00 98.96
C
ANISOU 3385  CD  ARG A 456    11865  16459   9278   -996   3467
-1115       C
ATOM   3386  NE  ARG A 456      -4.621 -15.213  57.666  1.00 87.75
N
ANISOU 3386  NE  ARG A 456    10550  14821   7971   -769   3493
-1257       N
ATOM   3387  CZ  ARG A 456      -4.873 -13.982  57.235  1.00109.72
C
ANISOU 3387  CZ  ARG A 456    13419  17548  10722   -559   3745
-1419       C
ATOM   3388  NH1 ARG A 456      -4.052 -13.399  56.384  1.00122.78
N
ANISOU 3388  NH1 ARG A 456    15203  19006  12442   -413   3757
-1551       N
ATOM   3389  NH2 ARG A 456      -5.839 -13.271  57.801  1.00 89.35
N
ANISOU 3389  NH2 ARG A 456    10816  15108   8024   -514   4012
-1450       N
ATOM   3390  N   SER A 457      -5.617 -14.425  63.387  1.00 95.81
N
ANISOU 3390  N   SER A 457    11703  16661   8039  -1723   4072
-1191       N
ATOM   3391  CA  SER A 457      -6.256 -15.045  64.539  1.00 97.72
C
ANISOU 3391  CA  SER A 457    11846  17139   8142  -1975   4060
-1053       C
ATOM   3392  C   SER A 457      -7.512 -15.789  64.111  1.00 97.90
```

```
C
ANISOU 3392  C   SER A 457    11628  17243   8327  -1847   3988
-910       C
ATOM   3393  O   SER A 457      -8.361 -15.229  63.411  1.00 97.92
O
ANISOU 3393  O   SER A 457    11571  17212   8422  -1575   4169
-972       O
ATOM   3394  CB  SER A 457      -6.606 -13.994  65.594  1.00130.60
C
ANISOU 3394  CB  SER A 457    16173  21424  12026  -2090   4433
-1196       C
ATOM   3395  OG  SER A 457      -7.180 -12.847  64.991  1.00122.02
O
ANISOU 3395  OG  SER A 457    15161  20225  10975  -1784   4763
-1372       O
ATOM   3396  N   LEU A 458      -7.624 -17.045  64.532  1.00107.01
N
ANISOU 3396  N   LEU A 458    12640  18509   9511  -2054   3723
-702       N
ATOM   3397  CA  LEU A 458      -8.859 -17.807  64.427  1.00114.05
C
ANISOU 3397  CA  LEU A 458    13311  19534  10490  -2031   3659
-547       C
ATOM   3398  C   LEU A 458      -9.241 -18.298  65.816  1.00136.87
C
ANISOU 3398  C   LEU A 458    16150  22670  13184  -2345   3664
-424       C
ATOM   3399  O   LEU A 458      -8.384 -18.753  66.579  1.00144.15
O
ANISOU 3399  O   LEU A 458    17142  23627  14003  -2612   3510
-353       O
ATOM   3400  CB  LEU A 458      -8.724 -18.986  63.446  1.00107.78
C
ANISOU 3400  CB  LEU A 458    12401  18613   9939  -1982   3318
-403       C
ATOM   3401  CG  LEU A 458      -9.885 -19.988  63.354  1.00105.74
C
ANISOU 3401  CG  LEU A 458    11927  18493   9757  -2035   3187
-213       C
ATOM   3402  CD1 LEU A 458     -10.006 -20.523  61.946  1.00 97.22
C
ANISOU 3402  CD1 LEU A 458    10785  17243   8911  -1862   3002
-177       C
ATOM   3403  CD2 LEU A 458      -9.701 -21.157  64.318  1.00124.93
C
ANISOU 3403  CD2 LEU A 458    14310  21036  12124  -2359   2967
-19       C
ATOM   3404  N   ALA A 459     -10.529 -18.210  66.136  1.00132.53
N
ANISOU 3404  N   ALA A 459    15466  22305  12583  -2315   3841
-381       N
ATOM   3405  CA  ALA A 459     -11.042 -18.573  67.449  1.00117.02
C
ANISOU 3405  CA  ALA A 459    13451  20592  10419  -2600   3894
```

-279 C
ATOM 3406 C ALA A 459 -11.886 -19.835 67.342 1.00105.42
C
ANISOU 3406 C ALA A 459 11744 19249 9061 -2680 3657
-46 C
ATOM 3407 O ALA A 459 -12.638 -20.012 66.379 1.00105.99
O
ANISOU 3407 O ALA A 459 11666 19295 9309 -2471 3621
2 O
ATOM 3408 CB ALA A 459 -11.873 -17.436 68.048 1.00124.97
C
ANISOU 3408 CB ALA A 459 14504 21726 11253 -2525 4334
-413 C
ATOM 3409 N VAL A 460 -11.760 -20.708 68.337 1.00106.50
N
ANISOU 3409 N VAL A 460 11851 19530 9083 -3006 3490
108 N
ATOM 3410 CA VAL A 460 -12.585 -21.904 68.441 1.00107.31
C
ANISOU 3410 CA VAL A 460 11751 19774 9249 -3138 3286
333 C
ATOM 3411 C VAL A 460 -13.589 -21.717 69.567 1.00111.35
C
ANISOU 3411 C VAL A 460 12180 20579 9549 -3298 3510
374 C
ATOM 3412 O VAL A 460 -13.359 -20.967 70.523 1.00136.57
O
ANISOU 3412 O VAL A 460 15511 23861 12518 -3419 3747
261 O
ATOM 3413 CB VAL A 460 -11.746 -23.180 68.667 1.00106.75
C
ANISOU 3413 CB VAL A 460 11691 19633 9235 -3381 2912
517 C
ATOM 3414 CG1 VAL A 460 -11.060 -23.599 67.377 1.00104.45
C
ANISOU 3414 CG1 VAL A 460 11433 19053 9202 -3193 2692
515 C
ATOM 3415 CG2 VAL A 460 -10.729 -22.965 69.778 1.00107.41
C
ANISOU 3415 CG2 VAL A 460 11927 19767 9118 -3634 2917
504 C
ATOM 3416 N ALA A 461 -14.717 -22.411 69.444 1.00110.92
N
ANISOU 3416 N ALA A 461 11907 20682 9557 -3315 3442
537 N
ATOM 3417 CA ALA A 461 -15.775 -22.353 70.440 1.00120.09
C
ANISOU 3417 CA ALA A 461 12952 22138 10540 -3458 3643
605 C
ATOM 3418 C ALA A 461 -16.445 -23.716 70.513 1.00147.14
C
ANISOU 3418 C ALA A 461 16179 25705 14022 -3655 3360
859 C
ATOM 3419 O ALA A 461 -16.365 -24.520 69.581 1.00132.05

```
O
ANISOU 3419  O   ALA A 461    14207  23659  12307  -3605   3080
958       O
ATOM   3420  CB  ALA A 461     -16.803 -21.262  70.116  1.00114.67
C
ANISOU 3420  CB  ALA A 461    12173  21535   9862  -3162   4026
504       C
ATOM   3421  N   ARG A 462     -17.114 -23.966  71.637  1.00145.16
N
ANISOU 3421  N   ARG A 462    15847  25723  13585  -3894   3450
959       N
ATOM   3422  CA  ARG A 462     -17.734 -25.256  71.894  1.00135.63
C
ANISOU 3422  CA  ARG A 462    14471  24672  12392  -4133   3193
1205       C
ATOM   3423  C   ARG A 462     -19.050 -25.048  72.623  1.00128.48
C
ANISOU 3423  C   ARG A 462    13384  24093  11338  -4198   3444
1272       C
ATOM   3424  O   ARG A 462     -19.176 -24.138  73.447  1.00142.84
O
ANISOU 3424  O   ARG A 462    15277  26027  12970  -4205   3782
1145       O
ATOM   3425  CB  ARG A 462     -16.818 -26.150  72.745  1.00127.39
C
ANISOU 3425  CB  ARG A 462    13541  23604  11257  -4478   2927
1320       C
ATOM   3426  CG  ARG A 462     -17.382 -27.528  73.058  1.00128.71
C
ANISOU 3426  CG  ARG A 462    13563  23905  11435  -4745   2656
1581       C
ATOM   3427  CD  ARG A 462     -16.342 -28.410  73.726  1.00130.14
C
ANISOU 3427  CD  ARG A 462    13861  24007  11577  -5034   2376
1716       C
ATOM   3428  NE  ARG A 462     -16.012 -27.915  75.060  1.00120.23
N
ANISOU 3428  NE  ARG A 462    12701  22930  10051  -5264   2534
1677       N
ATOM   3429  CZ  ARG A 462     -15.304 -28.591  75.959  1.00146.39
C
ANISOU 3429  CZ  ARG A 462    16080  26286  13258  -5580   2334
1830       C
ATOM   3430  NH1 ARG A 462     -14.764 -29.759  75.644  1.00133.56
N
ANISOU 3430  NH1 ARG A 462    14444  24499  11802  -5663   1985
2029       N
ATOM   3431  NH2 ARG A 462     -15.069 -28.058  77.150  1.00174.26
N
ANISOU 3431  NH2 ARG A 462    19701  30001  16508  -5812   2496
1784       N
ATOM   3432  N   GLN A 463     -20.028 -25.895  72.314  1.00121.19
N
ANISOU 3432  N   GLN A 463    12234  23320  10492  -4257   3290
```

```
1472       N
ATOM   3433  CA  GLN A 463     -21.271 -25.929  73.064  1.00123.99
C
ANISOU 3433  CA  GLN A 463    12388  24015  10710  -4370   3475
1588       C
ATOM   3434  C   GLN A 463     -21.817 -27.348  73.058  1.00135.34
C
ANISOU 3434  C   GLN A 463    13664  25576  12182  -4632   3139
1848       C
ATOM   3435  O   GLN A 463     -21.346 -28.220  72.324  1.00123.54
O
ANISOU 3435  O   GLN A 463    12213  23886  10840  -4677   2800
1926       O
ATOM   3436  CB  GLN A 463     -22.317 -24.974  72.482  1.00125.00
C
ANISOU 3436  CB  GLN A 463    12330  24259  10906  -4034   3802
1547       C
ATOM   3437  CG  GLN A 463     -22.834 -25.406  71.118  1.00124.72
C
ANISOU 3437  CG  GLN A 463    12111  24183  11096  -3867   3594
1668       C
ATOM   3438  CD  GLN A 463     -23.936 -24.508  70.594  1.00155.50
C
ANISOU 3438  CD  GLN A 463    15778  28245  15061  -3551   3903
1689       C
ATOM   3439  OE1 GLN A 463     -24.528 -24.779  69.549  1.00158.31
O
ANISOU 3439  OE1 GLN A 463    15945  28638  15569  -3435   3764
1817       O
ATOM   3440  NE2 GLN A 463     -24.222 -23.435  71.322  1.00197.45
N
ANISOU 3440  NE2 GLN A 463    21108  33657  20256  -3418   4335
1576       N
ATOM   3441  N   VAL A 464     -22.824 -27.563  73.894  1.00127.30
N
ANISOU 3441  N   VAL A 464    12473  24879  11015  -4809   3255
1980       N
ATOM   3442  CA  VAL A 464     -23.598 -28.795  73.911  1.00128.49
C
ANISOU 3442  CA  VAL A 464    12438  25204  11177  -5055   2992
2235       C
ATOM   3443  C   VAL A 464     -25.047 -28.386  73.685  1.00150.97
C
ANISOU 3443  C   VAL A 464    14985  28351  14028  -4910   3225
2326       C
ATOM   3444  O   VAL A 464     -25.465 -27.293  74.080  1.00150.59
O
ANISOU 3444  O   VAL A 464    14886  28432  13901  -4724   3624
2220       O
ATOM   3445  CB  VAL A 464     -23.417 -29.583  75.230  1.00129.94
C
ANISOU 3445  CB  VAL A 464    12681  25525  11165  -5463   2873
2353       C
ATOM   3446  CG1 VAL A 464     -24.033 -28.833  76.405  1.00151.82
```

```
C
ANISOU 3446  CG1 VAL A 464    15396  28588  13702  -5527   3253
2304       C
ATOM   3447  CG2 VAL A 464     -23.996 -30.987  75.113  1.00130.78
C
ANISOU 3447  CG2 VAL A 464    12650  25729  11310  -5730   2540
2615       C
ATOM   3448  N   VAL A 465     -25.813 -29.250  73.029  1.00155.55
N
ANISOU 3448  N   VAL A 465    15365  29038  14699  -4995   2989
2533       N
ATOM   3449  CA  VAL A 465     -27.230 -28.982  72.815  1.00141.95
C
ANISOU 3449  CA  VAL A 465    13312  27646  12978  -4894   3168
2679       C
ATOM   3450  C   VAL A 465     -28.042 -29.953  73.672  1.00139.71
C
ANISOU 3450  C   VAL A 465    12865  27673  12546  -5264   3056
2910       C
ATOM   3451  O   VAL A 465     -27.986 -31.174  73.463  1.00135.60
O
ANISOU 3451  O   VAL A 465    12365  27109  12049  -5539   2686
3060       O
ATOM   3452  CB  VAL A 465     -27.620 -29.041  71.329  1.00136.84
C
ANISOU 3452  CB  VAL A 465    12523  26941  12528  -4698   3022
2751       C
ATOM   3453  CG1 VAL A 465     -27.212 -30.344  70.688  1.00131.16
C
ANISOU 3453  CG1 VAL A 465    11911  26036  11888  -4942   2567
2849       C
ATOM   3454  CG2 VAL A 465     -29.121 -28.817  71.172  1.00173.24
C
ANISOU 3454  CG2 VAL A 465    16748  31946  17131  -4624   3190
2958       C
ATOM   3455  N   PRO A 466     -28.779 -29.459  74.669  1.00156.85
N
ANISOU 3455  N   PRO A 466    14940  30053  14603  -5240   3333
2908       N
ATOM   3456  CA  PRO A 466     -29.531 -30.368  75.543  1.00163.89
C
ANISOU 3456  CA  PRO A 466    15730  31155  15386  -5550   3186
3089       C
ATOM   3457  C   PRO A 466     -30.710 -31.018  74.844  1.00142.48
C
ANISOU 3457  C   PRO A 466    12710  28680  12746  -5606   3015
3344       C
ATOM   3458  O   PRO A 466     -31.109 -32.124  75.228  1.00143.73
O
ANISOU 3458  O   PRO A 466    12813  28970  12825  -5945   2757
3525       O
ATOM   3459  CB  PRO A 466     -29.991 -29.450  76.683  1.00165.74
C
ANISOU 3459  CB  PRO A 466    15994  31446  15535  -5403   3556
```

```
2959       C
ATOM   3460  CG  PRO A 466     -28.923 -28.410  76.755  1.00166.69
C
ANISOU 3460  CG  PRO A 466    16383  31299  15651  -5197   3786
2683       C
ATOM   3461  CD  PRO A 466     -28.568 -28.148  75.306  1.00154.99
C
ANISOU 3461  CD  PRO A 466    14850  29704  14336  -4966   3731
2666       C
ATOM   3462  N   GLU A 467     -31.279 -30.371  73.829  1.00142.57
N
ANISOU 3462  N   GLU A 467    12524  28750  12897  -5298   3139
3373       N
ATOM   3463  CA  GLU A 467     -32.516 -30.828  73.214  1.00191.54
C
ANISOU 3463  CA  GLU A 467    18407  35214  19156  -5333   3007
3627       C
ATOM   3464  C   GLU A 467     -32.270 -31.822  72.085  1.00168.83
C
ANISOU 3464  C   GLU A 467    15518  32303  16326  -5566   2628
3766       C
ATOM   3465  O   GLU A 467     -33.213 -32.173  71.368  1.00186.45
O
ANISOU 3465  O   GLU A 467    17504  34736  18601  -5608   2490
3973       O
ATOM   3466  CB  GLU A 467     -33.323 -29.629  72.709  1.00200.92
C
ANISOU 3466  CB  GLU A 467    19369  36495  20476  -4888   3326
3629       C
ATOM   3467  CG  GLU A 467     -33.796 -28.708  73.829  1.00217.20
C
ANISOU 3467  CG  GLU A 467    21430  38591  22505  -4674   3730
3525       C
ATOM   3468  CD  GLU A 467     -34.824 -27.691  73.372  1.00195.71
C
ANISOU 3468  CD  GLU A 467    18441  35983  19935  -4261   4033
3603       C
ATOM   3469  OE1 GLU A 467     -35.150 -27.673  72.166  1.00179.74
O
ANISOU 3469  OE1 GLU A 467    16223  34033  18037  -4137   3910
3743       O
ATOM   3470  OE2 GLU A 467     -35.307 -26.913  74.223  1.00156.98
O
ANISOU 3470  OE2 GLU A 467    13530  31087  15028  -4067   4401
3535       O
ATOM   3471  N   LYS A 468     -31.025 -32.282  71.933  1.00151.20
N
ANISOU 3471  N   LYS A 468    13579  29771  14098  -5707   2444
3647       N
ATOM   3472  CA  LYS A 468     -30.659 -33.420  71.085  1.00148.88
C
ANISOU 3472  CA  LYS A 468    13431  29252  13883  -5926   2021
3713       C
ATOM   3473  C   LYS A 468     -31.299 -33.375  69.696  1.00165.21
```

```
C
ANISOU 3473  C   LYS A 468    15319  31384  16068  -5814   1917
3817       C
ATOM   3474  O   LYS A 468     -31.835 -34.369  69.201  1.00176.45
O
ANISOU 3474  O   LYS A 468    16674  32899  17472  -6101   1635
4007       O
ATOM   3475  CB  LYS A 468     -30.981 -34.735  71.794  1.00143.34
C
ANISOU 3475  CB  LYS A 468    12733  28680  13050  -6388   1771
3909       C
ATOM   3476  CG  LYS A 468     -30.181 -34.936  73.074  1.00159.85
C
ANISOU 3476  CG  LYS A 468    15041  30663  15030  -6543   1799
3823       C
ATOM   3477  CD  LYS A 468     -30.546 -36.234  73.774  1.00161.72
C
ANISOU 3477  CD  LYS A 468    15270  31036  15139  -6998   1555
4038       C
ATOM   3478  CE  LYS A 468     -30.286 -37.435  72.880  1.00147.52
C
ANISOU 3478  CE  LYS A 468    13630  28982  13439  -7211   1164
4129       C
ATOM   3479  NZ  LYS A 468     -30.531 -38.716  73.598  1.00151.26
N
ANISOU 3479  NZ  LYS A 468    14143  29536  13793  -7657    931
4331       N
ATOM   3480  N   THR A 469     -31.238 -32.207  69.059  1.00168.83
N
ANISOU 3480  N   THR A 469    15712  31796  16638  -5412   2148
3698       N
ATOM   3481  CA  THR A 469     -31.950 -31.992  67.807  1.00177.57
C
ANISOU 3481  CA  THR A 469    16596  33031  17841  -5283   2094
3825       C
ATOM   3482  C   THR A 469     -31.152 -31.017  66.955  1.00163.56
C
ANISOU 3482  C   THR A 469    14975  30947  16224  -4890   2209
3598       C
ATOM   3483  O   THR A 469     -30.504 -30.102  67.470  1.00162.18
O
ANISOU 3483  O   THR A 469    14932  30622  16067  -4630   2481
3388       O
ATOM   3484  CB  THR A 469     -33.368 -31.452  68.035  1.00224.38
C
ANISOU 3484  CB  THR A 469    22075  39461  23719  -5193   2338
4060       C
ATOM   3485  OG1 THR A 469     -33.384 -30.612  69.196  1.00234.35
O
ANISOU 3485  OG1 THR A 469    23336  40770  24938  -4986   2703
3943       O
ATOM   3486  CG2 THR A 469     -34.356 -32.595  68.224  1.00237.07
C
ANISOU 3486  CG2 THR A 469    23491  41375  25209  -5604   2085
```

```
4344       C
ATOM   3487  N   LYS A 470     -31.207 -31.233  65.638  1.00164.94
N
ANISOU 3487  N   LYS A 470    15144  31032  16495  -4877   1998
3645       N
ATOM   3488  CA  LYS A 470     -30.452 -30.403  64.701  1.00165.38
C
ANISOU 3488  CA  LYS A 470    15350  30787  16699  -4533   2067
3443       C
ATOM   3489  C   LYS A 470     -30.856 -28.934  64.769  1.00170.24
C
ANISOU 3489  C   LYS A 470    15751  31563  17371  -4104   2473
3414       C
ATOM   3490  O   LYS A 470     -30.000 -28.046  64.688  1.00178.40
O
ANISOU 3490  O   LYS A 470    16979  32320  18484  -3802   2658
3172       O
ATOM   3491  CB  LYS A 470     -30.609 -30.940  63.277  1.00178.17
C
ANISOU 3491  CB  LYS A 470    16973  32340  18382  -4642   1770
3532       C
ATOM   3492  CG  LYS A 470     -29.927 -32.284  63.051  1.00173.75
C
ANISOU 3492  CG  LYS A 470    16734  31480  17803  -5004   1410
3488       C
ATOM   3493  CD  LYS A 470     -29.988 -32.722  61.593  1.00166.90
C
ANISOU 3493  CD  LYS A 470    15929  30499  16987  -5106   1157
3530       C
ATOM   3494  CE  LYS A 470     -31.413 -32.980  61.131  1.00191.55
C
ANISOU 3494  CE  LYS A 470    18687  34076  20018  -5305   1062
3844       C
ATOM   3495  NZ  LYS A 470     -31.441 -33.522  59.742  1.00174.95
N
ANISOU 3495  NZ  LYS A 470    16689  31858  17925  -5488    789
3883       N
ATOM   3496  N   GLU A 471     -32.147 -28.653  64.915  1.00172.62
N
ANISOU 3496  N   GLU A 471    15652  32301  17635  -4067   2629
3667       N
ATOM   3497  CA  GLU A 471     -32.653 -27.301  64.720  1.00181.39
C
ANISOU 3497  CA  GLU A 471    16524  33562  18835  -3634   3006
3695       C
ATOM   3498  C   GLU A 471     -32.773 -26.497  66.010  1.00179.00
C
ANISOU 3498  C   GLU A 471    16187  33355  18471  -3456   3439
3611       C
ATOM   3499  O   GLU A 471     -33.191 -25.337  65.958  1.00188.01
O
ANISOU 3499  O   GLU A 471    17200  34521  19714  -3055   3775
3601       O
ATOM   3500  CB  GLU A 471     -34.005 -27.350  63.998  1.00209.35
```

```
C
ANISOU 3500  CB  GLU A 471    19614  37530  22398  -3647   2954
4052       C
ATOM   3501  CG  GLU A 471     -33.899 -27.858  62.562  1.00210.87
C
ANISOU 3501  CG  GLU A 471    19847  37626  22650  -3769   2588
4118       C
ATOM   3502  CD  GLU A 471     -35.245 -27.998  61.877  1.00226.26
C
ANISOU 3502  CD  GLU A 471    21343  40039  24586  -3856   2494
4505       C
ATOM   3503  OE1 GLU A 471     -35.978 -26.991  61.788  1.00234.26
O
ANISOU 3503  OE1 GLU A 471    22052  41263  25692  -3499   2771
4646       O
ATOM   3504  OE2 GLU A 471     -35.566 -29.114  61.416  1.00225.55
O
ANISOU 3504  OE2 GLU A 471    21240  40053  24404  -4270   2124
4660       O
ATOM   3505  N   SER A 472     -32.424 -27.077  67.166  1.00170.99
N
ANISOU 3505  N   SER A 472    15345  32309  17315  -3722   3415
3529       N
ATOM   3506  CA  SER A 472     -32.537 -26.292  68.393  1.00183.18
C
ANISOU 3506  CA  SER A 472    16953  33828  18817  -3541   3786
3398       C
ATOM   3507  C   SER A 472     -31.174 -25.734  68.775  1.00171.19
C
ANISOU 3507  C   SER A 472    15803  31980  17261  -3452   3945
3073       C
ATOM   3508  O   SER A 472     -30.201 -26.499  68.848  1.00160.61
O
ANISOU 3508  O   SER A 472    14737  30415  15874  -3705   3660
2961       O
ATOM   3509  CB  SER A 472     -33.085 -27.146  69.533  1.00201.19
C
ANISOU 3509  CB  SER A 472    19197  36290  20958  -3872   3689
3508       C
ATOM   3510  OG  SER A 472     -33.375 -26.360  70.678  1.00202.13
O
ANISOU 3510  OG  SER A 472    19368  36384  21049  -3689   4056
3395       O
ATOM   3511  N   PRO A 473     -31.060 -24.408  68.995  1.00160.47
N
ANISOU 3511  N   PRO A 473    14540  30454  15976  -3058   4337
2888       N
ATOM   3512  CA  PRO A 473     -29.887 -23.846  69.688  1.00139.60
C
ANISOU 3512  CA  PRO A 473    12271  27518  13253  -3020   4520
2575       C
ATOM   3513  C   PRO A 473     -29.391 -24.657  70.878  1.00155.86
C
ANISOU 3513  C   PRO A 473    14521  29578  15118  -3417   4387
```

```
2522       C
ATOM   3514  O   PRO A 473     -30.181 -25.080  71.728  1.00192.26
O
ANISOU 3514  O   PRO A 473    19018  34378  19654  -3581   4394
2647       O
ATOM   3515  CB  PRO A 473     -30.381 -22.459  70.123  1.00138.92
C
ANISOU 3515  CB  PRO A 473    12207  27327  13251  -2618   4996
2463       C
ATOM   3516  CG  PRO A 473     -31.378 -22.096  69.073  1.00140.37
C
ANISOU 3516  CG  PRO A 473    12058  27653  13623  -2330   5031
2677       C
ATOM   3517  CD  PRO A 473     -32.098 -23.387  68.769  1.00172.53
C
ANISOU 3517  CD  PRO A 473    15845  32053  17655  -2657   4647
2978       C
ATOM   3518  N   GLY A 474     -28.079 -24.872  70.945  1.00133.74
N
ANISOU 3518  N   GLY A 474    12021  26543  12252  -3561   4247
2342       N
ATOM   3519  CA  GLY A 474     -27.450 -25.472  72.103  1.00133.62
C
ANISOU 3519  CA  GLY A 474    12217  26497  12057  -3906   4148
2281       C
ATOM   3520  C   GLY A 474     -27.278 -24.519  73.275  1.00136.69
C
ANISOU 3520  C   GLY A 474    12814  26782  12341  -3811   4509
2072       C
ATOM   3521  O   GLY A 474     -27.777 -23.393  73.297  1.00139.04
O
ANISOU 3521  O   GLY A 474    13097  27016  12716  -3470   4873
1979       O
ATOM   3522  N   ALA A 475     -26.547 -25.009  74.275  1.00140.44
N
ANISOU 3522  N   ALA A 475    13508  27207  12646  -4133   4394
2007       N
ATOM   3523  CA  ALA A 475     -26.158 -24.209  75.428  1.00145.28
C
ANISOU 3523  CA  ALA A 475    14390  27679  13130  -4124   4675
1791       C
ATOM   3524  C   ALA A 475     -25.267 -23.034  75.015  1.00139.43
C
ANISOU 3524  C   ALA A 475    13896  26646  12436  -3845   4899
1528       C
ATOM   3525  O   ALA A 475     -24.498 -23.138  74.054  1.00142.96
O
ANISOU 3525  O   ALA A 475    14379  26983  12957  -3791   4730
1495       O
ATOM   3526  CB  ALA A 475     -25.432 -25.076  76.456  1.00149.61
C
ANISOU 3526  CB  ALA A 475    15112  28245  13486  -4560   4430
1809       C
ATOM   3527  N   PRO A 476     -25.391 -21.881  75.704  1.00136.15
```

```
N
ANISOU 3527  N   PRO A 476    13663  26084  11983  -3657   5300
1339       N
ATOM   3528  CA  PRO A 476     -24.447 -20.763  75.520  1.00144.81
C
ANISOU 3528  CA  PRO A 476    15064  26868  13088  -3452   5513
1071       C
ATOM   3529  C   PRO A 476     -22.984 -21.151  75.316  1.00163.45
C
ANISOU 3529  C   PRO A 476    17635  29099  15372  -3664   5221
978       C
ATOM   3530  O   PRO A 476     -22.389 -21.851  76.142  1.00168.41
O
ANISOU 3530  O   PRO A 476    18378  29776  15835  -4032   5007
1005       O
ATOM   3531  CB  PRO A 476     -24.632 -19.951  76.809  1.00137.80
C
ANISOU 3531  CB  PRO A 476    14411  25877  12069  -3460   5877
921       C
ATOM   3532  CG  PRO A 476     -26.063 -20.181  77.163  1.00140.88
C
ANISOU 3532  CG  PRO A 476    14528  26498  12501  -3413   6015
1106       C
ATOM   3533  CD  PRO A 476     -26.316 -21.632  76.825  1.00139.77
C
ANISOU 3533  CD  PRO A 476    14103  26643  12362  -3686   5567
1361       C
ATOM   3534  N   TRP A 477     -22.412 -20.694  74.202  1.00129.29
N
ANISOU 3534  N   TRP A 477    13346  24608  11171  -3426   5214
884       N
ATOM   3535  CA  TRP A 477     -21.091 -21.129  73.757  1.00126.23
C
ANISOU 3535  CA  TRP A 477    13098  24112  10751  -3585   4928
832       C
ATOM   3536  C   TRP A 477     -19.983 -20.844  74.768  1.00134.16
C
ANISOU 3536  C   TRP A 477    14443  24962  11569  -3814   4936
665       C
ATOM   3537  O   TRP A 477     -19.958 -19.794  75.416  1.00127.85
O
ANISOU 3537  O   TRP A 477    13867  24006  10704  -3710   5246
478       O
ATOM   3538  CB  TRP A 477     -20.730 -20.436  72.443  1.00143.57
C
ANISOU 3538  CB  TRP A 477    15318  26093  13137  -3224   4977
717       C
ATOM   3539  CG  TRP A 477     -21.598 -20.775  71.284  1.00123.66
C
ANISOU 3539  CG  TRP A 477    12508  23627  10851  -2994   4846
886       C
ATOM   3540  CD1 TRP A 477     -22.744 -20.144  70.899  1.00125.44
C
ANISOU 3540  CD1 TRP A 477    12505  23998  11160  -2709   5137
```

```
953       C
ATOM   3541  CD2 TRP A 477     -21.369 -21.813  70.327  1.00122.38
C
ANISOU 3541  CD2 TRP A 477    12260  23373  10866  -3041   4397
1020       C
ATOM   3542  NE1 TRP A 477     -23.252 -20.736  69.767  1.00124.58
N
ANISOU 3542  NE1 TRP A 477    12159  23923  11253  -2603   4868
1132       N
ATOM   3543  CE2 TRP A 477     -22.424 -21.763  69.396  1.00122.20
C
ANISOU 3543  CE2 TRP A 477    11961  23466  11003  -2812   4420
1159       C
ATOM   3544  CE3 TRP A 477     -20.376 -22.784  70.172  1.00119.75
C
ANISOU 3544  CE3 TRP A 477    12061  22867  10570  -3257   3991
1045       C
ATOM   3545  CZ2 TRP A 477     -22.513 -22.648  68.324  1.00139.06
C
ANISOU 3545  CZ2 TRP A 477    13980  25551  13306  -2830   4048
1301       C
ATOM   3546  CZ3 TRP A 477     -20.466 -23.661  69.110  1.00119.77
C
ANISOU 3546  CZ3 TRP A 477    11956  22791  10760  -3242   3651
1177       C
ATOM   3547  CH2 TRP A 477     -21.527 -23.588  68.200  1.00129.53
C
ANISOU 3547  CH2 TRP A 477    12945  24146  12126  -3048   3679
1292       C
ATOM   3548  N   GLU A 478     -19.066 -21.805  74.896  1.00131.95
N
ANISOU 3548  N   GLU A 478    14204  24723  11207  -4138   4590
758       N
ATOM   3549  CA  GLU A 478     -17.774 -21.588  75.538  1.00124.08
C
ANISOU 3549  CA  GLU A 478    13501  23579  10063  -4345   4521
633       C
ATOM   3550  C   GLU A 478     -16.823 -20.931  74.543  1.00121.51
C
ANISOU 3550  C   GLU A 478    13325  22980   9864  -4099   4518
465       C
ATOM   3551  O   GLU A 478     -16.685 -21.403  73.411  1.00135.57
O
ANISOU 3551  O   GLU A 478    15005  24607  11899  -3910   4260
534       O
ATOM   3552  CB  GLU A 478     -17.173 -22.902  76.036  1.00149.15
C
ANISOU 3552  CB  GLU A 478    16654  26839  13178  -4730   4107
841       C
ATOM   3553  CG  GLU A 478     -17.898 -23.553  77.197  1.00172.80
C
ANISOU 3553  CG  GLU A 478    19555  30093  16010  -5038   4083
996       C
ATOM   3554  CD  GLU A 478     -17.279 -24.885  77.576  1.00176.49
```

```
C
ANISOU 3554  CD  GLU A 478    19990  30624  16444  -5397   3662
1232       C
ATOM   3555  OE1 GLU A 478     -16.864 -25.038  78.744  1.00140.46
O
ANISOU 3555  OE1 GLU A 478    15548  26145  11675  -5716   3612
1263       O
ATOM   3556  OE2 GLU A 478     -17.199 -25.774  76.702  1.00192.46
O
ANISOU 3556  OE2 GLU A 478    21899  32516  18711  -5308   3337
1375       O
ATOM   3557  N   PHE A 479     -16.165 -19.849  74.954  1.00121.89
N
ANISOU 3557  N   PHE A 479    13654  22860   9799  -4064   4744
238        N
ATOM   3558  CA  PHE A 479     -15.090 -19.281  74.137  1.00133.54
C
ANISOU 3558  CA  PHE A 479    15289  24114  11336  -3915   4728
85       C
ATOM   3559  C   PHE A 479     -13.811 -20.048  74.458  1.00118.29
C
ANISOU 3559  C   PHE A 479    13456  22141   9348  -4223   4352
176        C
ATOM   3560  O   PHE A 479     -13.024 -19.665  75.325  1.00140.66
O
ANISOU 3560  O   PHE A 479    16506  24975  11963  -4468   4398
94       O
ATOM   3561  CB  PHE A 479     -14.941 -17.788  74.403  1.00139.96
C
ANISOU 3561  CB  PHE A 479    16387  24670  12123  -3705   5073
-180       C
ATOM   3562  CG  PHE A 479     -14.130 -17.061  73.366  1.00127.97
C
ANISOU 3562  CG  PHE A 479    14999  22908  10716  -3457   5109
-342       C
ATOM   3563  CD1 PHE A 479     -14.757 -16.288  72.400  1.00117.93
C
ANISOU 3563  CD1 PHE A 479    13671  21504   9633  -3034   5341
-427       C
ATOM   3564  CD2 PHE A 479     -12.748 -17.143  73.356  1.00116.72
C
ANISOU 3564  CD2 PHE A 479    13743  21394   9211  -3644   4908
-391       C
ATOM   3565  CE1 PHE A 479     -14.024 -15.613  71.445  1.00115.88
C
ANISOU 3565  CE1 PHE A 479    13538  21013   9479  -2806   5368
-576       C
ATOM   3566  CE2 PHE A 479     -12.008 -16.472  72.401  1.00114.68
C
ANISOU 3566  CE2 PHE A 479    13604  20913   9056  -3417   4941
-542       C
ATOM   3567  CZ  PHE A 479     -12.648 -15.705  71.445  1.00114.21
C
ANISOU 3567  CZ  PHE A 479    13505  20707   9183  -2998   5169
```

```
-644       C
ATOM   3568  N   VAL A 480      -13.617 -21.161  73.743  1.00145.78
N
ANISOU 3568  N   VAL A 480    17848  25610  11931   4178   6973
914        N
ATOM   3569  CA  VAL A 480      -12.562 -22.118  74.078  1.00143.64
C
ANISOU 3569  CA  VAL A 480    17838  25211  11529   3982   6823
782        C
ATOM   3570  C   VAL A 480      -11.169 -21.502  73.950  1.00140.21
C
ANISOU 3570  C   VAL A 480    17788  24587  10898   3984   6447
389        C
ATOM   3571  O   VAL A 480      -10.297 -21.736  74.796  1.00140.23
O
ANISOU 3571  O   VAL A 480    18189  24438  10654   4066   6336
232        O
ATOM   3572  CB  VAL A 480      -12.708 -23.379  73.205  1.00141.64
C
ANISOU 3572  CB  VAL A 480    17210  25065  11541   3527   6830
956        C
ATOM   3573  CG1 VAL A 480      -11.518 -24.299  73.390  1.00139.03
C
ANISOU 3573  CG1 VAL A 480    17164  24590  11069   3330   6636
795        C
ATOM   3574  CG2 VAL A 480      -13.998 -24.104  73.544  1.00145.67
C
ANISOU 3574  CG2 VAL A 480    17393  25741  12212   3506   7196
1351       C
ATOM   3575  N   GLY A 481      -10.935 -20.713  72.913  1.00139.10
N
ANISOU 3575  N   GLY A 481    17531  24456  10864   3890   6238
231        N
ATOM   3576  CA  GLY A 481       -9.626 -20.117  72.725  1.00139.21
C
ANISOU 3576  CA  GLY A 481    17877  24303  10713   3859   5874
-127       C
ATOM   3577  C   GLY A 481       -9.425 -19.658  71.296  1.00130.81
C
ANISOU 3577  C   GLY A 481    16556  23294   9850   3626   5659
-237       C
ATOM   3578  O   GLY A 481      -10.274 -19.859  70.427  1.00137.46
O
ANISOU 3578  O   GLY A 481    16958  24306  10964   3473   5777
-37       O
ATOM   3579  N   LEU A 482       -8.273 -19.024  71.072  1.00128.28
N
ANISOU 3579  N   LEU A 482    16515  22832   9393   3593   5326
-555       N
ATOM   3580  CA  LEU A 482       -7.928 -18.538  69.742  1.00124.81
C
ANISOU 3580  CA  LEU A 482    15879  22427   9115   3378   5089
-693       C
ATOM   3581  C   LEU A 482       -6.422 -18.626  69.515  1.00121.53
```

```
C
ANISOU 3581  C   LEU A 482    15707  21880   8588   3187   4712
-976       C
ATOM   3582  O   LEU A 482      -5.631 -18.408  70.437  1.00122.43
O
ANISOU 3582  O   LEU A 482    16228  21840   8448   3322   4587
-1147       O
ATOM   3583  CB  LEU A 482      -8.454 -17.108  69.546  1.00126.18
C
ANISOU 3583  CB  LEU A 482    16086  22589   9267   3628   5116
-765       C
ATOM   3584  CG  LEU A 482      -8.007 -15.944  70.437  1.00128.19
C
ANISOU 3584  CG  LEU A 482    16846  22647   9214   3950   5023
-993       C
ATOM   3585  CD1 LEU A 482      -6.646 -15.390  70.047  1.00125.27
C
ANISOU 3585  CD1 LEU A 482    16745  22122   8730   3798   4618
-1335       C
ATOM   3586  CD2 LEU A 482      -9.063 -14.854  70.444  1.00131.05
C
ANISOU 3586  CD2 LEU A 482    17170  23039   9583   4263   5219
-908       C
ATOM   3587  N   LEU A 483      -6.042 -18.964  68.278  1.00122.12
N
ANISOU 3587  N   LEU A 483    15513  22026   8862   2868   4524
-1012       N
ATOM   3588  CA  LEU A 483      -4.733 -19.436  67.842  1.00117.24
C
ANISOU 3588  CA  LEU A 483    14982  21340   8222   2615   4199
-1190       C
ATOM   3589  C   LEU A 483      -4.109 -18.527  66.780  1.00111.93
C
ANISOU 3589  C   LEU A 483    14262  20650   7616   2484   3899
-1418       C
ATOM   3590  O   LEU A 483      -4.824 -17.892  65.999  1.00111.70
O
ANISOU 3590  O   LEU A 483    13991  20710   7738   2484   3959
-1381       O
ATOM   3591  CB  LEU A 483      -4.835 -20.875  67.312  1.00113.13
C
ANISOU 3591  CB  LEU A 483    14183  20919   7881   2328   4255
-993       C
ATOM   3592  CG  LEU A 483      -6.018 -21.252  66.414  1.00113.12
C
ANISOU 3592  CG  LEU A 483    13714  21102   8163   2170   4452
-739       C
ATOM   3593  CD1 LEU A 483      -5.904 -20.673  65.015  1.00145.34
C
ANISOU 3593  CD1 LEU A 483    17539  25256  12429   1975   4243
-845       C
ATOM   3594  CD2 LEU A 483      -6.189 -22.765  66.361  1.00112.97
C
ANISOU 3594  CD2 LEU A 483    13550  21134   8239   1942   4563
```

```
-519       C
ATOM   3595  N   PRO A 484      -2.765 -18.422  66.751  1.00116.37
N
ANISOU 3595  N   PRO A 484    15048  21100   8067   2375   3568
-1651       N
ATOM   3596  CA  PRO A 484      -2.095 -17.522  65.797  1.00116.74
C
ANISOU 3596  CA  PRO A 484    15070  21117   8168   2239   3271
-1876       C
ATOM   3597  C   PRO A 484      -1.639 -18.224  64.521  1.00111.07
C
ANISOU 3597  C   PRO A 484    14036  20491   7676   1890   3099
-1861       C
ATOM   3598  O   PRO A 484      -0.919 -19.224  64.585  1.00102.99
O
ANISOU 3598  O   PRO A 484    13029  19453   6648   1743   2999
-1834       O
ATOM   3599  CB  PRO A 484      -0.897 -17.005  66.602  1.00153.03
C
ANISOU 3599  CB  PRO A 484    20091  25541  12513   2314   3010
-2114       C
ATOM   3600  CG  PRO A 484      -0.552 -18.151  67.497  1.00175.51
C
ANISOU 3600  CG  PRO A 484    23059  28369  15257   2332   3070
-2012       C
ATOM   3601  CD  PRO A 484      -1.855 -18.852  67.828  1.00149.32
C
ANISOU 3601  CD  PRO A 484    19569  25149  12016   2442   3465
-1732       C
ATOM   3602  N   LEU A 485      -2.027 -17.696  63.358  1.00102.65
N
ANISOU 3602  N   LEU A 485    12701  19506   6796   1765   3059
-1883       N
ATOM   3603  CA  LEU A 485      -1.738 -18.296  62.057  1.00 99.53
C
ANISOU 3603  CA  LEU A 485    11999  19196   6623   1434   2915
-1860       C
ATOM   3604  C   LEU A 485      -0.828 -17.396  61.226  1.00 97.42
C
ANISOU 3604  C   LEU A 485    11766  18864   6384   1297   2599
-2110       C
ATOM   3605  O   LEU A 485      -1.121 -16.208  61.052  1.00 98.09
O
ANISOU 3605  O   LEU A 485    11905  18919   6447   1410   2585
-2228       O
ATOM   3606  CB  LEU A 485      -3.045 -18.538  61.300  1.00 99.69
C
ANISOU 3606  CB  LEU A 485    11632  19380   6866   1361   3140
-1644       C
ATOM   3607  CG  LEU A 485      -3.985 -19.658  61.739  1.00101.39
C
ANISOU 3607  CG  LEU A 485    11687  19693   7144   1365   3432
-1345       C
ATOM   3608  CD1 LEU A 485      -5.421 -19.238  61.501  1.00103.31
```

```
C
ANISOU 3608  CD1 LEU A 485    11643  20075   7534   1464   3687
-1158       C
ATOM   3609  CD2 LEU A 485      -3.691 -20.911  60.953  1.00 99.22
C
ANISOU 3609  CD2 LEU A 485    11224  19464   7012   1031   3346
-1245       C
ATOM   3610  N   PHE A 486       0.272 -17.960  60.715  1.00 95.08
N
ANISOU 3610  N   PHE A 486    11450  18539   6138   1062   2355
-2179       N
ATOM   3611  CA  PHE A 486       1.213 -17.228  59.872  1.00 93.12
C
ANISOU 3611  CA  PHE A 486    11203  18233   5945    894   2054
-2387       C
ATOM   3612  C   PHE A 486       0.772 -17.238  58.406  1.00137.41
C
ANISOU 3612  C   PHE A 486    16483  23931  11794    674   2047
-2347       C
ATOM   3613  O   PHE A 486       0.211 -18.220  57.912  1.00 96.26
O
ANISOU 3613  O   PHE A 486    11040  18817   6718    548   2174
-2161       O
ATOM   3614  CB  PHE A 486       2.617 -17.826  60.003  1.00 91.94
C
ANISOU 3614  CB  PHE A 486    11163  18023   5748    765   1807
-2449       C
ATOM   3615  CG  PHE A 486       3.717 -16.930  59.495  1.00104.47
C
ANISOU 3615  CG  PHE A 486    12813  19534   7346    636   1492
-2663       C
ATOM   3616  CD1 PHE A 486       3.536 -15.558  59.415  1.00115.35
C
ANISOU 3616  CD1 PHE A 486    14302  20844   8682    699   1431
-2828       C
ATOM   3617  CD2 PHE A 486       4.934 -17.460  59.108  1.00111.86
C
ANISOU 3617  CD2 PHE A 486    13705  20463   8333    457   1266
-2683       C
ATOM   3618  CE1 PHE A 486       4.545 -14.734  58.950  1.00 91.01
C
ANISOU 3618  CE1 PHE A 486    11284  17676   5618    548   1139
-3012       C
ATOM   3619  CE2 PHE A 486       5.947 -16.640  58.643  1.00107.92
C
ANISOU 3619  CE2 PHE A 486    13233  19909   7862    321    986
-2851       C
ATOM   3620  CZ  PHE A 486       5.750 -15.275  58.563  1.00 89.48
C
ANISOU 3620  CZ  PHE A 486    11007  17495   5494    348    917
-3017       C
ATOM   3621  N   ASP A 487       1.056 -16.130  57.712  1.00123.93
N
ANISOU 3621  N   ASP A 487    14782  22175  10130    617   1883
```

```
-2526       N
ATOM   3622  CA  ASP A 487       0.643 -15.975  56.317  1.00 95.58
C
ANISOU 3622  CA  ASP A 487    10924  18570   6822    424   1840
-2480       C
ATOM   3623  C   ASP A 487       1.167 -17.027  55.339  1.00121.85
C
ANISOU 3623  C   ASP A 487    14070  21899  10330    143   1719
-2390       C
ATOM   3624  O   ASP A 487       0.390 -17.442  54.461  1.00 85.02
O
ANISOU 3624  O   ASP A 487     9167  17245   5893     17   1787
-2238       O
ATOM   3625  CB  ASP A 487       1.027 -14.571  55.832  1.00118.28
C
ANISOU 3625  CB  ASP A 487    13909  21301   9729    415   1650
-2683       C
ATOM   3626  CG  ASP A 487       0.032 -13.515  56.261  1.00147.33
C
ANISOU 3626  CG  ASP A 487    17685  24956  13337    678   1815
-2706       C
ATOM   3627  OD1 ASP A 487       0.363 -12.313  56.167  1.00162.50
O
ANISOU 3627  OD1 ASP A 487    19801  26739  15203    723   1678
-2890       O
ATOM   3628  OD2 ASP A 487      -1.081 -13.885  56.687  1.00170.07
O
ANISOU 3628  OD2 ASP A 487    20453  27954  16212    843   2088
-2530       O
ATOM   3629  N   PRO A 488       2.426 -17.505  55.417  1.00135.03
N
ANISOU 3629  N   PRO A 488    15840  23560  11904     45   1541
-2464       N
ATOM   3630  CA  PRO A 488       3.641 -16.743  55.077  1.00 83.83
C
ANISOU 3630  CA  PRO A 488     9456  16991   5403    -48   1266
-2657       C
ATOM   3631  C   PRO A 488       3.763 -16.243  53.610  1.00101.43
C
ANISOU 3631  C   PRO A 488    11528  19114   7898   -248   1122
-2682       C
ATOM   3632  O   PRO A 488       3.055 -16.733  52.731  1.00155.06
O
ANISOU 3632  O   PRO A 488    18126  25899  14890   -347   1199
-2542       O
ATOM   3633  CB  PRO A 488       4.748 -17.750  55.402  1.00 83.26
C
ANISOU 3633  CB  PRO A 488     9441  16934   5258   -104   1155
-2612       C
ATOM   3634  CG  PRO A 488       4.162 -19.061  55.050  1.00 82.49
C
ANISOU 3634  CG  PRO A 488     9201  16903   5239   -169   1319
-2407       C
ATOM   3635  CD  PRO A 488       2.678 -18.959  55.376  1.00 85.37
```

```
C
ANISOU 3635  CD  PRO A 488     9492  17336   5608    -54   1584
-2313       C
ATOM   3636  N   PRO A 489       4.655 -15.268  53.380  1.00 81.63
N
ANISOU 3636  N   PRO A 489     9119  16519   5379   -312    907
-2856       N
ATOM   3637  CA  PRO A 489       4.983 -14.831  52.011  1.00 98.50
C
ANISOU 3637  CA  PRO A 489    11131  18551   7744   -504    761
-2880       C
ATOM   3638  C   PRO A 489       5.429 -15.935  51.056  1.00115.94
C
ANISOU 3638  C   PRO A 489    13176  20786  10092   -675    719
-2746       C
ATOM   3639  O   PRO A 489       6.043 -16.926  51.456  1.00134.13
O
ANISOU 3639  O   PRO A 489    15497  23181  12285   -677    714
-2683       O
ATOM   3640  CB  PRO A 489       6.099 -13.801  52.233  1.00 80.47
C
ANISOU 3640  CB  PRO A 489     9009  16197   5367   -554    534
-3073       C
ATOM   3641  CG  PRO A 489       6.751 -14.240  53.478  1.00 81.83
C
ANISOU 3641  CG  PRO A 489     9327  16476   5288   -463    501
-3110       C
ATOM   3642  CD  PRO A 489       5.602 -14.693  54.351  1.00 83.10
C
ANISOU 3642  CD  PRO A 489     9536  16703   5333   -247    758
-3024       C
ATOM   3643  N   ARG A 490       5.105 -15.742  49.773  1.00137.27
N
ANISOU 3643  N   ARG A 490    15743  23393  13018   -797    688
-2703       N
ATOM   3644  CA  ARG A 490       5.714 -16.513  48.696  1.00139.98
C
ANISOU 3644  CA  ARG A 490    15987  23713  13484   -955    605
-2611       C
ATOM   3645  C   ARG A 490       7.179 -16.110  48.514  1.00122.52
C
ANISOU 3645  C   ARG A 490    13821  21495  11237  -1038    409
-2707       C
ATOM   3646  O   ARG A 490       7.600 -15.008  48.877  1.00167.44
O
ANISOU 3646  O   ARG A 490    19596  27147  16876  -1033    304
-2857       O
ATOM   3647  CB  ARG A 490       4.966 -16.285  47.382  1.00128.29
C
ANISOU 3647  CB  ARG A 490    14385  22121  12237  -1048    609
-2552       C
ATOM   3648  CG  ARG A 490       3.477 -16.572  47.429  1.00123.92
C
ANISOU 3648  CG  ARG A 490    13735  21592  11758   -995    777
```

```
-2439       C
ATOM   3649  CD  ARG A 490       2.846 -16.325  46.067  1.00 84.84
C
ANISOU 3649  CD  ARG A 490     8666  16536   7035  -1096    736
-2387       C
ATOM   3650  NE  ARG A 490       1.391 -16.433  46.096  1.00 89.61
N
ANISOU 3650  NE  ARG A 490     9137  17185   7727  -1050    874
-2274       N
ATOM   3651  CZ  ARG A 490       0.722 -17.575  45.988  1.00 77.98
C
ANISOU 3651  CZ  ARG A 490     7563  15763   6302  -1126    951
-2097       C
ATOM   3652  NH1 ARG A 490       1.375 -18.718  45.814  1.00 73.29
N
ANISOU 3652  NH1 ARG A 490     7032  15156   5661  -1233    908
-2025       N
ATOM   3653  NH2 ARG A 490      -0.603 -17.571  46.028  1.00 75.53
N
ANISOU 3653  NH2 ARG A 490     7093  15518   6088  -1096   1068
-1982       N
ATOM   3654  N   HIS A 491       7.964 -17.030  47.945  1.00110.71
N
ANISOU 3654  N   HIS A 491    12271  20034   9760  -1113    361
-2607       N
ATOM   3655  CA  HIS A 491       9.381 -16.758  47.696  1.00105.41
C
ANISOU 3655  CA  HIS A 491    11587  19392   9072  -1189    191
-2653       C
ATOM   3656  C   HIS A 491       9.611 -15.602  46.720  1.00 72.27
C
ANISOU 3656  C   HIS A 491     7350  15066   5045  -1308     80
-2730       C
ATOM   3657  O   HIS A 491      10.485 -14.757  46.949  1.00 73.15
O
ANISOU 3657  O   HIS A 491     7484  15183   5125  -1371    -64
-2834       O
ATOM   3658  CB  HIS A 491      10.079 -18.036  47.217  1.00 98.89
C
ANISOU 3658  CB  HIS A 491    10717  18633   8223  -1199    199
-2500       C
ATOM   3659  CG  HIS A 491      10.031 -18.252  45.736  1.00104.02
C
ANISOU 3659  CG  HIS A 491    11302  19168   9053  -1289    196
-2406       C
ATOM   3660  ND1 HIS A 491       8.964 -18.853  45.103  1.00 86.61
N
ANISOU 3660  ND1 HIS A 491     9100  16866   6940  -1305    300
-2310       N
ATOM   3661  CD2 HIS A 491      10.928 -17.962  44.764  1.00 69.58
C
ANISOU 3661  CD2 HIS A 491     6882  14771   4786  -1366    100
-2384       C
ATOM   3662  CE1 HIS A 491       9.204 -18.917  43.805  1.00 68.68
```

```
C
ANISOU 3662  CE1 HIS A 491     6805  14491   4799  -1378    255
-2248       C
ATOM   3663  NE2 HIS A 491      10.389 -18.382  43.573  1.00 90.85
N
ANISOU 3663  NE2 HIS A 491     9576  17335   7610  -1404    150
-2288       N
ATOM   3664  N   ASP A 492       8.847 -15.540  45.630  1.00 83.17
N
ANISOU 3664  N   ASP A 492     8677  16323   6602  -1352    135
-2676       N
ATOM   3665  CA  ASP A 492       9.138 -14.590  44.559  1.00 97.67
C
ANISOU 3665  CA  ASP A 492    10485  18028   8597  -1459     42
-2723       C
ATOM   3666  C   ASP A 492       8.274 -13.335  44.569  1.00109.25
C
ANISOU 3666  C   ASP A 492    12012  19372  10125  -1438     51
-2845       C
ATOM   3667  O   ASP A 492       8.514 -12.439  43.753  1.00164.90
O
ANISOU 3667  O   ASP A 492    19070  26296  17291  -1521    -26
-2897       O
ATOM   3668  CB  ASP A 492       9.003 -15.282  43.194  1.00109.90
C
ANISOU 3668  CB  ASP A 492    11969  19500  10287  -1510     72
-2585       C
ATOM   3669  CG  ASP A 492       7.651 -15.952  43.004  1.00 90.53
C
ANISOU 3669  CG  ASP A 492     9502  17012   7882  -1466    192
-2500       C
ATOM   3670  OD1 ASP A 492       7.412 -16.515  41.915  1.00113.17
O
ANISOU 3670  OD1 ASP A 492    12352  19795  10852  -1514    197
-2395       O
ATOM   3671  OD2 ASP A 492       6.827 -15.918  43.941  1.00115.54
O
ANISOU 3671  OD2 ASP A 492    12681  20237  10982  -1386    278
-2528       O
ATOM   3672  N   SER A 493       7.279 -13.247  45.449  1.00105.81
N
ANISOU 3672  N   SER A 493    11628  18967   9607  -1311    158
-2879       N
ATOM   3673  CA  SER A 493       6.529 -12.003  45.603  1.00136.40
C
ANISOU 3673  CA  SER A 493    15592  22737  13497  -1242    177
-2996       C
ATOM   3674  C   SER A 493       7.408 -10.855  46.092  1.00 74.59
C
ANISOU 3674  C   SER A 493     7919  14845   5577  -1288     30
-3163       C
ATOM   3675  O   SER A 493       7.245  -9.708  45.660  1.00 75.06
O
ANISOU 3675  O   SER A 493     8068  14752   5698  -1310    -17
```

```
-3257       O
ATOM   3676  CB  SER A 493       5.350 -12.224  46.549  1.00 74.44
C
ANISOU 3676  CB  SER A 493     7764  14966   5553  -1063    345
-2971       C
ATOM   3677  OG  SER A 493       5.780 -12.777  47.779  1.00 75.40
O
ANISOU 3677  OG  SER A 493     7957  15215   5476   -996    365
-2983       O
ATOM   3678  N   ALA A 494       8.328 -11.141  47.011  1.00 75.45
N
ANISOU 3678  N   ALA A 494     8079  15061   5528  -1306    -53
-3200       N
ATOM   3679  CA  ALA A 494       9.311 -10.146  47.434  1.00 82.11
C
ANISOU 3679  CA  ALA A 494     9057  15851   6289  -1405   -239
-3344       C
ATOM   3680  C   ALA A 494      10.157  -9.606  46.282  1.00 97.42
C
ANISOU 3680  C   ALA A 494    10922  17696   8398  -1607   -371
-3337       C
ATOM   3681  O   ALA A 494      10.427  -8.401  46.222  1.00133.64
O
ANISOU 3681  O   ALA A 494    15644  22140  12992  -1696   -485
-3459       O
ATOM   3682  CB  ALA A 494      10.209 -10.745  48.515  1.00 87.31
C
ANISOU 3682  CB  ALA A 494     9735  16675   6762  -1401   -325
-3351       C
ATOM   3683  N   GLU A 495      10.623 -10.473  45.383  1.00 80.72
N
ANISOU 3683  N   GLU A 495     8618  15648   6404  -1678   -355
-3190       N
ATOM   3684  CA  GLU A 495      11.223  -9.990  44.138  1.00105.07
C
ANISOU 3684  CA  GLU A 495    11630  18630   9662  -1832   -427
-3155       C
ATOM   3685  C   GLU A 495      10.231  -9.251  43.243  1.00100.00
C
ANISOU 3685  C   GLU A 495    11047  17792   9155  -1804   -357
-3185       C
ATOM   3686  O   GLU A 495      10.563  -8.209  42.665  1.00 89.37
O
ANISOU 3686  O   GLU A 495     9767  16298   7892  -1915   -438
-3248       O
ATOM   3687  CB  GLU A 495      11.825 -11.161  43.361  1.00124.19
C
ANISOU 3687  CB  GLU A 495    13872  21159  12154  -1856   -392
-2976       C
ATOM   3688  CG  GLU A 495      13.062 -11.788  43.972  1.00 95.36
C
ANISOU 3688  CG  GLU A 495    10132  17707   8395  -1892   -479
-2919       C
ATOM   3689  CD  GLU A 495      13.697 -12.807  43.044  1.00134.15
```

```
C
ANISOU 3689  CD  GLU A 495    14897  22699  13375  -1889   -431
-2732       C
ATOM   3690  OE1 GLU A 495      13.075 -13.137  42.012  1.00149.26
O
ANISOU 3690  OE1 GLU A 495    16810  24503  15400  -1854   -329
-2655       O
ATOM   3691  OE2 GLU A 495      14.814 -13.280  43.341  1.00165.98
O
ANISOU 3691  OE2 GLU A 495    18825  26903  17335  -1907   -497
-2656       O
ATOM   3692  N   THR A 496       9.015  -9.781  43.114  1.00105.85
N
ANISOU 3692  N   THR A 496    11763  18535   9922  -1663   -210
-3131       N
ATOM   3693  CA  THR A 496       8.058  -9.267  42.135  1.00 95.44
C
ANISOU 3693  CA  THR A 496    10456  17064   8744  -1625   -148
-3124       C
ATOM   3694  C   THR A 496       7.674  -7.802  42.359  1.00 85.79
C
ANISOU 3694  C   THR A 496     9418  15684   7494  -1588   -177
-3279       C
ATOM   3695  O   THR A 496       7.558  -7.039  41.393  1.00136.11
O
ANISOU 3695  O   THR A 496    15835  21896  13985  -1630   -200
-3298       O
ATOM   3696  CB  THR A 496       6.814 -10.156  42.130  1.00116.87
C
ANISOU 3696  CB  THR A 496    13081  19846  11478  -1495     -4
-3023       C
ATOM   3697  OG1 THR A 496       7.177 -11.477  41.711  1.00129.50
O
ANISOU 3697  OG1 THR A 496    14562  21536  13105  -1545     12
-2878       O
ATOM   3698  CG2 THR A 496       5.787  -9.622  41.163  1.00117.55
C
ANISOU 3698  CG2 THR A 496    13159  19802  11704  -1446     40
-3009       C
ATOM   3699  N   ILE A 497       7.483  -7.380  43.613  1.00 74.93
N
ANISOU 3699  N   ILE A 497     8188  14335   5947  -1495   -175
-3389       N
ATOM   3700  CA  ILE A 497       7.288  -5.954  43.888  1.00 76.74
C
ANISOU 3700  CA  ILE A 497     8661  14388   6110  -1459   -221
-3546       C
ATOM   3701  C   ILE A 497       8.454  -5.111  43.377  1.00 77.25
C
ANISOU 3701  C   ILE A 497     8807  14315   6228  -1685   -396
-3613       C
ATOM   3702  O   ILE A 497       8.247  -4.055  42.766  1.00117.98
O
ANISOU 3702  O   ILE A 497    14107  19275  11446  -1705   -417
```

```
-3679       O
ATOM   3703  CB  ILE A 497       7.030  -5.729  45.392  1.00 78.79
C
ANISOU 3703  CB  ILE A 497     9105  14695   6136  -1314   -200
-3651       C
ATOM   3704  CG1 ILE A 497       7.166  -4.246  45.768  1.00143.82
C
ANISOU 3704  CG1 ILE A 497    17666  22720  14257  -1313   -298
-3831       C
ATOM   3705  CG2 ILE A 497       7.941  -6.597  46.232  1.00 84.76
C
ANISOU 3705  CG2 ILE A 497     9795  15628   6781  -1388   -275
-3628       C
ATOM   3706  CD1 ILE A 497       6.162  -3.322  45.102  1.00 81.42
C
ANISOU 3706  CD1 ILE A 497     9878  14640   6416  -1175   -204
-3860       C
ATOM   3707  N   ARG A 498       9.690  -5.552  43.610  1.00 77.29
N
ANISOU 3707  N   ARG A 498     8720  14431   6216  -1857   -520
-3584       N
ATOM   3708  CA  ARG A 498      10.841  -4.827  43.075  1.00111.05
C
ANISOU 3708  CA  ARG A 498    13017  18609  10569  -2099   -681
-3607       C
ATOM   3709  C   ARG A 498      10.830  -4.808  41.548  1.00127.21
C
ANISOU 3709  C   ARG A 498    14946  20562  12825  -2159   -627
-3501       C
ATOM   3710  O   ARG A 498      11.043  -3.765  40.920  1.00 78.11
O
ANISOU 3710  O   ARG A 498     8848  14152   6681  -2268   -684
-3552       O
ATOM   3711  CB  ARG A 498      12.136  -5.453  43.593  1.00 95.58
C
ANISOU 3711  CB  ARG A 498    10914  16841   8560  -2249   -811
-3554       C
ATOM   3712  CG  ARG A 498      12.301  -5.403  45.103  1.00122.99
C
ANISOU 3712  CG  ARG A 498    14528  20394  11808  -2208   -903
-3668       C
ATOM   3713  CD  ARG A 498      13.200  -6.532  45.583  1.00154.36
C
ANISOU 3713  CD  ARG A 498    18296  24627  15728  -2245   -956
-3564       C
ATOM   3714  NE  ARG A 498      14.497  -6.520  44.912  1.00171.53
N
ANISOU 3714  NE  ARG A 498    20282  26865  18026  -2474  -1083
-3467       N
ATOM   3715  CZ  ARG A 498      15.335  -7.551  44.882  1.00163.13
C
ANISOU 3715  CZ  ARG A 498    18981  26031  16971  -2493  -1097
-3322       C
ATOM   3716  NH1 ARG A 498      15.085  -8.633  45.605  1.00137.65
```

```
N
ANISOU 3716  NH1 ARG A 498    15713  22974  13613  -2320  -1019
-3281       N
ATOM   3717  NH2 ARG A 498      16.485  -7.450  44.231  1.00181.19
N
ANISOU 3717  NH2 ARG A 498    21084  28384  19376  -2683  -1193
-3214       N
ATOM   3718  N   ARG A 499      10.586  -5.969  40.942  1.00119.19
N
ANISOU 3718  N   ARG A 499    13728  19666  11893  -2087   -520
-3352       N
ATOM   3719  CA  ARG A 499      10.472  -6.085  39.491  1.00 79.89
C
ANISOU 3719  CA  ARG A 499     8669  14598   7090  -2106   -463
-3245       C
ATOM   3720  C   ARG A 499       9.356  -5.209  38.926  1.00 73.25
C
ANISOU 3720  C   ARG A 499     7972  13556   6303  -2001   -402
-3313       C
ATOM   3721  O   ARG A 499       9.552  -4.499  37.934  1.00110.75
O
ANISOU 3721  O   ARG A 499    12781  18139  11160  -2074   -423
-3309       O
ATOM   3722  CB  ARG A 499      10.249  -7.553  39.129  1.00 81.01
C
ANISOU 3722  CB  ARG A 499     8631  14887   7261  -2021   -370
-3088       C
ATOM   3723  CG  ARG A 499      10.384  -7.900  37.661  1.00 88.30
C
ANISOU 3723  CG  ARG A 499     9483  15736   8330  -2045   -332
-2958       C
ATOM   3724  CD  ARG A 499      10.311  -9.408  37.507  1.00100.01
C
ANISOU 3724  CD  ARG A 499    10846  17359   9794  -1973   -264
-2813       C
ATOM   3725  NE  ARG A 499      11.508 -10.049  38.042  1.00115.76
N
ANISOU 3725  NE  ARG A 499    12743  19529  11710  -2031   -304
-2748       N
ATOM   3726  CZ  ARG A 499      11.517 -11.223  38.663  1.00127.47
C
ANISOU 3726  CZ  ARG A 499    14167  21176  13091  -1956   -263
-2680       C
ATOM   3727  NH1 ARG A 499      10.418 -11.963  38.706  1.00131.76
N
ANISOU 3727  NH1 ARG A 499    14729  21718  13617  -1849   -178
-2647       N
ATOM   3728  NH2 ARG A 499      12.656 -11.712  39.133  1.00123.18
N
ANISOU 3728  NH2 ARG A 499    13536  20797  12468  -1990   -307
-2619       N
ATOM   3729  N   ALA A 500       8.172  -5.253  39.543  1.00 73.07
N
ANISOU 3729  N   ALA A 500     8001  13557   6203  -1813   -316
```

```
-3361       N
ATOM   3730  CA  ALA A 500       7.092  -4.341  39.168  1.00 73.62
C
ANISOU 3730  CA  ALA A 500     8209  13463   6298  -1676   -256
-3425       C
ATOM   3731  C   ALA A 500       7.494  -2.874  39.296  1.00 75.50
C
ANISOU 3731  C   ALA A 500     8708  13493   6485  -1746   -343
-3572       C
ATOM   3732  O   ALA A 500       7.179  -2.062  38.419  1.00 82.97
O
ANISOU 3732  O   ALA A 500     9764  14252   7509  -1727   -332
-3592       O
ATOM   3733  CB  ALA A 500       5.857  -4.624  40.023  1.00 92.04
C
ANISOU 3733  CB  ALA A 500    10535  15900   8535  -1453   -139
-3435       C
ATOM   3734  N   LEU A 501       8.172  -2.509  40.384  1.00 77.16
N
ANISOU 3734  N   LEU A 501     9045  13717   6554  -1830   -439
-3676       N
ATOM   3735  CA  LEU A 501       8.612  -1.126  40.550  1.00 79.31
C
ANISOU 3735  CA  LEU A 501     9605  13767   6762  -1934   -550
-3818       C
ATOM   3736  C   LEU A 501       9.567  -0.700  39.439  1.00109.05
C
ANISOU 3736  C   LEU A 501    13341  17409  10683  -2174   -634
-3767       C
ATOM   3737  O   LEU A 501       9.442   0.401  38.890  1.00137.18
O
ANISOU 3737  O   LEU A 501    17117  20734  14272  -2200   -650
-3832       O
ATOM   3738  CB  LEU A 501       9.267  -0.939  41.919  1.00114.84
C
ANISOU 3738  CB  LEU A 501    14245  18315  11076  -2015   -676
-3928       C
ATOM   3739  CG  LEU A 501       9.737   0.491  42.194  1.00107.27
C
ANISOU 3739  CG  LEU A 501    13633  17100  10023  -2152   -824
-4084       C
ATOM   3740  CD1 LEU A 501       8.561   1.458  42.132  1.00102.32
C
ANISOU 3740  CD1 LEU A 501    13304  16259   9315  -1910   -717
-4182       C
ATOM   3741  CD2 LEU A 501      10.440   0.586  43.538  1.00151.72
C
ANISOU 3741  CD2 LEU A 501    19403  22784  15461  -2253   -986
-4189       C
ATOM   3742  N   ASN A 502      10.531  -1.557  39.101  1.00 94.23
N
ANISOU 3742  N   ASN A 502    11211  15692   8901  -2333   -674
-3640       N
ATOM   3743  CA  ASN A 502      11.392  -1.307  37.947  1.00132.38
```

```
C
ANISOU 3743  CA  ASN A 502    15968  20439  13891  -2525   -710
-3547       C
ATOM   3744  C   ASN A 502      10.598  -1.176  36.650  1.00108.59
C
ANISOU 3744  C   ASN A 502    12972  17285  11000  -2397   -590
-3488       C
ATOM   3745  O   ASN A 502      10.934  -0.348  35.794  1.00111.00
O
ANISOU 3745  O   ASN A 502    13385  17399  11392  -2502   -608
-3483       O
ATOM   3746  CB  ASN A 502      12.434  -2.418  37.836  1.00142.98
C
ANISOU 3746  CB  ASN A 502    17018  22016  15292  -2640   -734
-3391       C
ATOM   3747  CG  ASN A 502      13.457  -2.366  38.954  1.00128.69
C
ANISOU 3747  CG  ASN A 502    15181  20335  13382  -2815   -892
-3436       C
ATOM   3748  OD1 ASN A 502      13.633  -1.333  39.604  1.00 89.65
O
ANISOU 3748  OD1 ASN A 502    10456  15256   8350  -2928  -1017
-3577       O
ATOM   3749  ND2 ASN A 502      14.134  -3.483  39.190  1.00141.00
N
ANISOU 3749  ND2 ASN A 502    16492  22145  14935  -2831   -898
-3314       N
ATOM   3750  N   LEU A 503       9.546  -1.974  36.478  1.00 93.86
N
ANISOU 3750  N   LEU A 503    11009  15510   9145  -2180   -476
-3437       N
ATOM   3751  CA  LEU A 503       8.711  -1.881  35.288  1.00 73.44
C
ANISOU 3751  CA  LEU A 503     8440  12803   6663  -2050   -390
-3383       C
ATOM   3752  C   LEU A 503       7.576  -0.874  35.444  1.00 74.60
C
ANISOU 3752  C   LEU A 503     8808  12790   6748  -1870   -349
-3504       C
ATOM   3753  O   LEU A 503       6.631  -0.889  34.647  1.00 83.99
O
ANISOU 3753  O   LEU A 503     9987  13924   8000  -1711   -278
-3461       O
ATOM   3754  CB  LEU A 503       8.153  -3.258  34.928  1.00 71.42
C
ANISOU 3754  CB  LEU A 503     7966  12716   6455  -1935   -315
-3251       C
ATOM   3755  CG  LEU A 503       9.200  -4.287  34.499  1.00 70.25
C
ANISOU 3755  CG  LEU A 503     7636  12693   6361  -2058   -329
-3107       C
ATOM   3756  CD1 LEU A 503       8.597  -5.678  34.418  1.00 68.67
C
ANISOU 3756  CD1 LEU A 503     7283  12646   6161  -1944   -268
```

```
-2998       C
ATOM   3757  CD2 LEU A 503       9.819  -3.894  33.167  1.00102.70
C
ANISOU 3757  CD2 LEU A 503    11782  16650  10588  -2143   -328
-3029       C
ATOM   3758  N   GLY A 504       7.648  -0.010  36.456  1.00 76.63
N
ANISOU 3758  N   GLY A 504     9278  12972   6867  -1879   -398
-3648       N
ATOM   3759  CA  GLY A 504       6.744   1.118  36.587  1.00 89.28
C
ANISOU 3759  CA  GLY A 504    11154  14385   8382  -1700   -358
-3765       C
ATOM   3760  C   GLY A 504       5.317   0.798  36.970  1.00 78.10
C
ANISOU 3760  C   GLY A 504     9687  13080   6908  -1396   -233
-3755       C
ATOM   3761  O   GLY A 504       4.419   1.592  36.674  1.00 79.06
O
ANISOU 3761  O   GLY A 504     9971  13067   7001  -1197   -169
-3797       O
ATOM   3762  N   VAL A 505       5.070  -0.336  37.618  1.00 94.14
N
ANISOU 3762  N   VAL A 505    11492  15357   8919  -1346   -190
-3687       N
ATOM   3763  CA  VAL A 505       3.776  -0.620  38.227  1.00121.45
C
ANISOU 3763  CA  VAL A 505    14892  18947  12307  -1077    -65
-3668       C
ATOM   3764  C   VAL A 505       3.961  -0.670  39.737  1.00140.75
C
ANISOU 3764  C   VAL A 505    17432  21485  14562  -1040    -63
-3752       C
ATOM   3765  O   VAL A 505       4.756  -1.470  40.247  1.00121.50
O
ANISOU 3765  O   VAL A 505    14872  19184  12107  -1193   -122
-3727       O
ATOM   3766  CB  VAL A 505       3.169  -1.929  37.696  1.00 92.83
C
ANISOU 3766  CB  VAL A 505    10938  15521   8813  -1041     -3
-3500       C
ATOM   3767  CG1 VAL A 505       2.344  -1.661  36.457  1.00 75.20
C
ANISOU 3767  CG1 VAL A 505     8668  13199   6706   -935     26
-3435       C
ATOM   3768  CG2 VAL A 505       4.262  -2.927  37.375  1.00 81.74
C
ANISOU 3768  CG2 VAL A 505     9370  14200   7487  -1277    -79
-3421       C
ATOM   3769  N   ASN A 506       3.237   0.191  40.450  1.00162.71
N
ANISOU 3769  N   ASN A 506    20453  24187  17184   -815      6
-3849       N
ATOM   3770  CA  ASN A 506       3.304   0.234  41.906  1.00145.90
```

```
C
ANISOU 3770  CA  ASN A 506    18475  22120  14840   -731      18
-3935       C
ATOM   3771  C   ASN A 506       2.371  -0.827  42.476  1.00133.32
C
ANISOU 3771  C   ASN A 506    16629  20793  13234   -539    175
-3814       C
ATOM   3772  O   ASN A 506       1.157  -0.771  42.257  1.00162.60
O
ANISOU 3772  O   ASN A 506    20254  24557  16971   -297    320
-3738       O
ATOM   3773  CB  ASN A 506       2.930   1.619  42.426  1.00106.08
C
ANISOU 3773  CB  ASN A 506    13850  16855   9600   -543      36
-4085       C
ATOM   3774  CG  ASN A 506       3.132   1.750  43.921  1.00 98.51
C
ANISOU 3774  CG  ASN A 506    13123  15917   8389   -466      21
-4192       C
ATOM   3775  OD1 ASN A 506       2.354   2.409  44.610  1.00113.32
O
ANISOU 3775  OD1 ASN A 506    15260  17726  10071   -174    130
-4255       O
ATOM   3776  ND2 ASN A 506       4.182   1.117  44.434  1.00113.27
N
ANISOU 3776  ND2 ASN A 506    14911  17884  10244   -706   -111
-4206       N
ATOM   3777  N   VAL A 507       2.927  -1.791  43.202  1.00 93.41
N
ANISOU 3777  N   VAL A 507    11445  15910   8137   -646    148
-3782       N
ATOM   3778  CA  VAL A 507       2.108  -2.802  43.863  1.00 81.62
C
ANISOU 3778  CA  VAL A 507     9744  14657   6613   -483    301
-3666       C
ATOM   3779  C   VAL A 507       1.632  -2.275  45.213  1.00 84.26
C
ANISOU 3779  C   VAL A 507    10324  14995   6695   -230    400
-3751       C
ATOM   3780  O   VAL A 507       2.443  -1.897  46.064  1.00115.37
O
ANISOU 3780  O   VAL A 507    14517  18858  10459   -292    295
-3885       O
ATOM   3781  CB  VAL A 507       2.878  -4.121  44.027  1.00 79.87
C
ANISOU 3781  CB  VAL A 507     9299  14608   6439   -681    246
-3583       C
ATOM   3782  CG1 VAL A 507       1.982  -5.185  44.638  1.00 79.83
C
ANISOU 3782  CG1 VAL A 507     9089  14832   6412   -532    413
-3446       C
ATOM   3783  CG2 VAL A 507       3.421  -4.592  42.694  1.00 77.62
C
ANISOU 3783  CG2 VAL A 507     8827  14296   6369   -902    154
```

```
-3499       C
ATOM   3784  N   LYS A 508       0.316  -2.247  45.408  1.00 85.35
N
ANISOU 3784  N   LYS A 508    10393  15228   6809     62    599
-3664       N
ATOM   3785  CA  LYS A 508      -0.290  -1.787  46.651  1.00 94.49
C
ANISOU 3785  CA  LYS A 508    11778  16405   7718    367    742
-3711       C
ATOM   3786  C   LYS A 508      -0.996  -2.969  47.299  1.00 97.77
C
ANISOU 3786  C   LYS A 508    11911  17102   8134    483    921
-3542       C
ATOM   3787  O   LYS A 508      -1.823  -3.631  46.663  1.00 87.04
O
ANISOU 3787  O   LYS A 508    10213  15898   6962    502   1022
-3365       O
ATOM   3788  CB  LYS A 508      -1.284  -0.645  46.419  1.00 90.12
C
ANISOU 3788  CB  LYS A 508    11403  15733   7106    670    862
-3731       C
ATOM   3789  CG  LYS A 508      -0.691   0.635  45.849  1.00 90.72
C
ANISOU 3789  CG  LYS A 508    11823  15496   7148    588    708
-3900       C
ATOM   3790  CD  LYS A 508      -1.721   1.761  45.916  1.00 93.36
C
ANISOU 3790  CD  LYS A 508    12410  15716   7346    966    859
-3924       C
ATOM   3791  CE  LYS A 508      -1.250   3.036  45.229  1.00 94.05
C
ANISOU 3791  CE  LYS A 508    12853  15474   7410    894    722
-4075       C
ATOM   3792  NZ  LYS A 508       0.049   3.512  45.773  1.00107.11
N
ANISOU 3792  NZ  LYS A 508    14864  16913   8918    642    510
-4265       N
ATOM   3793  N   MET A 509      -0.659  -3.234  48.558  1.00107.32
N
ANISOU 3793  N   MET A 509    13275  18371   9132    547    950
-3593       N
ATOM   3794  CA  MET A 509      -1.338  -4.265  49.330  1.00112.00
C
ANISOU 3794  CA  MET A 509    13657  19212   9684    687   1145
-3435       C
ATOM   3795  C   MET A 509      -2.812  -3.945  49.548  1.00 99.09
C
ANISOU 3795  C   MET A 509    11952  17680   8016   1048   1409
-3308       C
ATOM   3796  O   MET A 509      -3.189  -2.800  49.807  1.00 94.04
O
ANISOU 3796  O   MET A 509    11604  16908   7220   1306   1470
-3397       O
ATOM   3797  CB  MET A 509      -0.640  -4.439  50.680  1.00140.76
```

```
C
ANISOU 3797  CB  MET A 509    17552  22865  13066    719   1117
-3536       C
ATOM   3798  CG  MET A 509      -1.400  -5.272  51.695  1.00117.79
C
ANISOU 3798  CG  MET A 509    14529  20178  10046    938   1357
-3390       C
ATOM   3799  SD  MET A 509      -0.483  -5.415  53.236  1.00 93.67
S
ANISOU 3799  SD  MET A 509    11822  17107   6661    981   1290
-3529       S
ATOM   3800  CE  MET A 509       0.933  -6.368  52.692  1.00 90.55
C
ANISOU 3800  CE  MET A 509    11239  16744   6422    544   1024
-3549       C
ATOM   3801  N   ILE A 510      -3.651  -4.972  49.434  1.00 99.60
N
ANISOU 3801  N   ILE A 510    11632  17986   8227   1067   1565
-3085       N
ATOM   3802  CA  ILE A 510      -5.035  -4.918  49.885  1.00 97.35
C
ANISOU 3802  CA  ILE A 510    11210  17877   7901   1407   1846
-2914       C
ATOM   3803  C   ILE A 510      -5.231  -6.092  50.832  1.00129.49
C
ANISOU 3803  C   ILE A 510    15120  22167  11914   1420   1998
-2770       C
ATOM   3804  O   ILE A 510      -4.891  -7.229  50.485  1.00141.36
O
ANISOU 3804  O   ILE A 510    16377  23759  13573   1136   1920
-2683       O
ATOM   3805  CB  ILE A 510      -6.030  -4.983  48.710  1.00 93.38
C
ANISOU 3805  CB  ILE A 510    10342  17472   7665   1404   1888
-2741       C
ATOM   3806  CG1 ILE A 510      -5.888  -3.745  47.820  1.00 93.27
C
ANISOU 3806  CG1 ILE A 510    10529  17230   7679   1440   1761
-2882       C
ATOM   3807  CG2 ILE A 510      -7.455  -5.116  49.222  1.00118.89
C
ANISOU 3807  CG2 ILE A 510    13344  20950  10880   1728   2184
-2514       C
ATOM   3808  CD1 ILE A 510      -6.224  -2.447  48.520  1.00 96.31
C
ANISOU 3808  CD1 ILE A 510    11301  17492   7802   1823   1884
-2991       C
ATOM   3809  N   THR A 511      -5.781  -5.831  52.018  1.00143.57
N
ANISOU 3809  N   THR A 511    17065  24025  13459   1762   2225
-2737       N
ATOM   3810  CA  THR A 511      -6.231  -6.927  52.865  1.00134.80
C
ANISOU 3810  CA  THR A 511    15759  23148  12311   1819   2428
```

```
-2550       C
ATOM   3811  C   THR A 511      -7.287  -6.445  53.847  1.00136.21
C
ANISOU 3811  C   THR A 511    16026  23442  12286   2279   2749
-2441       C
ATOM   3812  O   THR A 511      -7.308  -5.276  54.240  1.00127.97
O
ANISOU 3812  O   THR A 511    15355  22247  11022   2565   2782
-2583       O
ATOM   3813  CB  THR A 511      -5.062  -7.558  53.635  1.00115.75
C
ANISOU 3813  CB  THR A 511    13553  20686   9742   1651   2306
-2672       C
ATOM   3814  OG1 THR A 511      -5.522  -8.708  54.356  1.00 97.69
O
ANISOU 3814  OG1 THR A 511    11064  18618   7436   1687   2508
-2472       O
ATOM   3815  CG2 THR A 511      -4.454  -6.555  54.611  1.00 98.87
C
ANISOU 3815  CG2 THR A 511    11935  18363   7268   1863   2260
-2903       C
ATOM   3816  N   GLY A 512      -8.180  -7.367  54.217  1.00141.73
N
ANISOU 3816  N   GLY A 512    16385  24409  13059   2348   2991
-2174       N
ATOM   3817  CA  GLY A 512      -9.112  -7.136  55.306  1.00114.56
C
ANISOU 3817  CA  GLY A 512    13002  21117   9410   2786   3335
-2032       C
ATOM   3818  C   GLY A 512      -8.511  -7.270  56.690  1.00107.96
C
ANISOU 3818  C   GLY A 512    12555  20226   8240   2937   3405
-2139       C
ATOM   3819  O   GLY A 512      -9.113  -6.801  57.660  1.00111.34
O
ANISOU 3819  O   GLY A 512    13182  20704   8416   3360   3671
-2083       O
ATOM   3820  N   ASP A 513      -7.346  -7.911  56.802  1.00105.53
N
ANISOU 3820  N   ASP A 513    12363  19823   7913   2623   3177
-2281       N
ATOM   3821  CA  ASP A 513      -6.592  -7.920  58.051  1.00106.63
C
ANISOU 3821  CA  ASP A 513    12924  19873   7717   2745   3168
-2430       C
ATOM   3822  C   ASP A 513      -6.301  -6.508  58.551  1.00115.65
C
ANISOU 3822  C   ASP A 513    14604  20780   8558   3030   3112
-2667       C
ATOM   3823  O   ASP A 513      -6.082  -5.576  57.773  1.00108.02
O
ANISOU 3823  O   ASP A 513    13750  19634   7657   2969   2935
-2812       O
ATOM   3824  CB  ASP A 513      -5.282  -8.690  57.878  1.00103.56
```

```
C
ANISOU 3824  CB  ASP A 513    12560  19409   7379   2342   2873
-2561       C
ATOM   3825  CG  ASP A 513      -5.505 -10.163  57.600  1.00107.11
C
ANISOU 3825  CG  ASP A 513    12587  20060   8050   2096   2943
-2335       C
ATOM   3826  OD1 ASP A 513      -4.573 -10.823  57.096  1.00173.68
O
ANISOU 3826  OD1 ASP A 513    20952  28442  16595   1750   2706
-2403       O
ATOM   3827  OD2 ASP A 513      -6.612 -10.662  57.892  1.00103.75
O
ANISOU 3827  OD2 ASP A 513    11904  19840   7675   2251   3238
-2080       O
ATOM   3828  N   GLN A 514      -6.309  -6.367  59.877  1.00118.52
N
ANISOU 3828  N   GLN A 514    15334  21030   8668   3291   3225
-2664       N
ATOM   3829  CA  GLN A 514      -5.882  -5.136  60.531  1.00113.81
C
ANISOU 3829  CA  GLN A 514    15333  20115   7793   3496   3114
-2882       C
ATOM   3830  C   GLN A 514      -4.454  -4.748  60.150  1.00111.62
C
ANISOU 3830  C   GLN A 514    15317  19639   7454   3171   2708
-3193       C
ATOM   3831  O   GLN A 514      -3.618  -5.587  59.800  1.00108.75
O
ANISOU 3831  O   GLN A 514    14750  19366   7203   2820   2517
-3239       O
ATOM   3832  CB  GLN A 514      -6.014  -5.241  62.057  1.00133.08
C
ANISOU 3832  CB  GLN A 514    18112  22453   9998   3759   3265
-2817       C
ATOM   3833  CG  GLN A 514      -5.306  -6.409  62.736  1.00148.27
C
ANISOU 3833  CG  GLN A 514    20006  24437  11894   3560   3196
-2796       C
ATOM   3834  CD  GLN A 514      -6.129  -7.685  62.728  1.00168.30
C
ANISOU 3834  CD  GLN A 514    22047  27267  14632   3541   3470
-2484       C
ATOM   3835  OE1 GLN A 514      -7.146  -7.779  62.040  1.00201.95
O
ANISOU 3835  OE1 GLN A 514    25911  31720  19100   3598   3667
-2286       O
ATOM   3836  NE2 GLN A 514      -5.712  -8.663  63.526  1.00125.72
N
ANISOU 3836  NE2 GLN A 514    16688  21900   9180   3458   3478
-2428       N
ATOM   3837  N   LEU A 515      -4.206  -3.437  60.221  1.00130.79
N
ANISOU 3837  N   LEU A 515    18203  21781   9711   3290   2578
```

```
-3391       N
ATOM   3838  CA  LEU A 515      -3.025  -2.798  59.641  1.00120.63
C
ANISOU 3838  CA  LEU A 515    17143  20285   8406   2982   2198
-3669       C
ATOM   3839  C   LEU A 515      -1.720  -3.408  60.144  1.00116.30
C
ANISOU 3839  C   LEU A 515    16699  19689   7800   2670   1920
-3788       C
ATOM   3840  O   LEU A 515      -0.779  -3.607  59.366  1.00112.32
O
ANISOU 3840  O   LEU A 515    16040  19200   7437   2294   1645
-3904       O
ATOM   3841  CB  LEU A 515      -3.067  -1.302  59.954  1.00129.44
C
ANISOU 3841  CB  LEU A 515    18825  21054   9302   3198   2138
-3827       C
ATOM   3842  CG  LEU A 515      -1.956  -0.383  59.444  1.00128.55
C
ANISOU 3842  CG  LEU A 515    19039  20656   9150   2906   1757
-4105       C
ATOM   3843  CD1 LEU A 515      -1.983  -0.277  57.931  1.00111.22
C
ANISOU 3843  CD1 LEU A 515    16492  18550   7217   2698   1681
-4128       C
ATOM   3844  CD2 LEU A 515      -2.073   0.991  60.083  1.00135.34
C
ANISOU 3844  CD2 LEU A 515    20526  21145   9752   3143   1732
-4226       C
ATOM   3845  N   ALA A 516      -1.642  -3.697  61.443  1.00112.78
N
ANISOU 3845  N   ALA A 516    16509  19187   7155   2826   1987
-3749       N
ATOM   3846  CA  ALA A 516      -0.457  -4.321  62.028  1.00112.06
C
ANISOU 3846  CA  ALA A 516    16515  19067   6994   2574   1739
-3834       C
ATOM   3847  C   ALA A 516      -0.046  -5.621  61.338  1.00124.30
C
ANISOU 3847  C   ALA A 516    17551  20883   8793   2265   1686
-3736       C
ATOM   3848  O   ALA A 516       1.148  -5.851  61.117  1.00166.40
O
ANISOU 3848  O   ALA A 516    22872  26183  14171   1940   1377
-3853       O
ATOM   3849  CB  ALA A 516      -0.694  -4.573  63.518  1.00115.16
C
ANISOU 3849  CB  ALA A 516    17199  19405   7151   2843   1894
-3759       C
ATOM   3850  N   ILE A 517      -1.002  -6.486  60.996  1.00111.31
N
ANISOU 3850  N   ILE A 517    15476  19497   7319   2348   1976
-3505       N
ATOM   3851  CA  ILE A 517      -0.676  -7.646  60.163  1.00103.80
```

```
C
ANISOU 3851  CA  ILE A 517    14052  18768   6620   2030   1920
-3412       C
ATOM   3852  C   ILE A 517      -0.140  -7.221  58.799  1.00114.75
C
ANISOU 3852  C   ILE A 517    15271  20116   8214   1721   1682
-3535       C
ATOM   3853  O   ILE A 517       0.877  -7.741  58.326  1.00120.47
O
ANISOU 3853  O   ILE A 517    15858  20848   9067   1387   1443
-3580       O
ATOM   3854  CB  ILE A 517      -1.900  -8.568  60.008  1.00103.59
C
ANISOU 3854  CB  ILE A 517    13614  18998   6749   2150   2269
-3127       C
ATOM   3855  CG1 ILE A 517      -2.399  -9.045  61.370  1.00144.65
C
ANISOU 3855  CG1 ILE A 517    18972  24207  11782   2427   2511
-2974       C
ATOM   3856  CG2 ILE A 517      -1.570  -9.751  59.107  1.00100.23
C
ANISOU 3856  CG2 ILE A 517    12751  18756   6577   1801   2197
-3031       C
ATOM   3857  CD1 ILE A 517      -3.704  -9.798  61.294  1.00155.76
C
ANISOU 3857  CD1 ILE A 517    19993  25843  13345   2556   2868
-2671       C
ATOM   3858  N   GLY A 518      -0.815  -6.278  58.144  1.00101.69
N
ANISOU 3858  N   GLY A 518    13627  18360   6649   1823   1741
-3545       N
ATOM   3859  CA  GLY A 518      -0.396  -5.885  56.807  1.00 99.31
C
ANISOU 3859  CA  GLY A 518    13165  17949   6618   1524   1529
-3603       C
ATOM   3860  C   GLY A 518       0.972  -5.229  56.747  1.00 99.03
C
ANISOU 3860  C   GLY A 518    13419  17706   6504   1275   1171
-3843       C
ATOM   3861  O   GLY A 518       1.792  -5.556  55.887  1.00 97.19
O
ANISOU 3861  O   GLY A 518    12975  17470   6485    931    963
-3857       O
ATOM   3862  N   LYS A 519       1.238  -4.289  57.656  1.00101.90
N
ANISOU 3862  N   LYS A 519    14275  17891   6553   1445   1092
-4025       N
ATOM   3863  CA  LYS A 519       2.582  -3.727  57.788  1.00102.26
C
ANISOU 3863  CA  LYS A 519    14607  17753   6495   1185    728
-4244       C
ATOM   3864  C   LYS A 519       3.637  -4.770  58.158  1.00100.99
C
ANISOU 3864  C   LYS A 519    14298  17723   6352    946    557
```

```
-4221       C
ATOM   3865  O   LYS A 519       4.724  -4.796  57.570  1.00 99.49
O
ANISOU 3865  O   LYS A 519    14000  17501   6299    600    281
-4283       O
ATOM   3866  CB  LYS A 519       2.573  -2.588  58.808  1.00106.08
C
ANISOU 3866  CB  LYS A 519    15692  17967   6648   1415    676
-4400       C
ATOM   3867  CG  LYS A 519       1.775  -1.374  58.356  1.00122.05
C
ANISOU 3867  CG  LYS A 519    17940  19814   8621   1628    782
-4467       C
ATOM   3868  CD  LYS A 519       1.929  -0.200  59.313  1.00144.45
C
ANISOU 3868  CD  LYS A 519    21431  22297  11157   1787    685
-4611       C
ATOM   3869  CE  LYS A 519       1.778  -0.626  60.766  1.00160.60
C
ANISOU 3869  CE  LYS A 519    23700  24346  12973   2016    804
-4543       C
ATOM   3870  NZ  LYS A 519       1.989   0.519  61.697  1.00145.44
N
ANISOU 3870  NZ  LYS A 519    22418  22074  10768   2123    686
-4689       N
ATOM   3871  N   GLU A 520       3.343  -5.634  59.133  1.00101.78
N
ANISOU 3871  N   GLU A 520    14390  17943   6339   1132    724
-4096       N
ATOM   3872  CA  GLU A 520       4.316  -6.651  59.533  1.00100.80
C
ANISOU 3872  CA  GLU A 520    14147  17915   6237    944    574
-4045       C
ATOM   3873  C   GLU A 520       4.621  -7.641  58.414  1.00108.92
C
ANISOU 3873  C   GLU A 520    14683  19146   7557    671    553
-3931       C
ATOM   3874  O   GLU A 520       5.789  -7.935  58.136  1.00147.17
O
ANISOU 3874  O   GLU A 520    19433  23992  12493    395    299
-3958       O
ATOM   3875  CB  GLU A 520       3.821  -7.393  60.775  1.00112.69
C
ANISOU 3875  CB  GLU A 520    15756  19496   7563   1222    794
-3921       C
ATOM   3876  CG  GLU A 520       4.780  -8.458  61.287  1.00110.32
C
ANISOU 3876  CG  GLU A 520    15375  19287   7257   1078    656
-3863       C
ATOM   3877  CD  GLU A 520       6.228  -8.002  61.288  1.00103.85
C
ANISOU 3877  CD  GLU A 520    14702  18342   6415    802    252
-4018       C
ATOM   3878  OE1 GLU A 520       6.525  -6.936  61.869  1.00114.08
```

```
O
ANISOU 3878  OE1 GLU A 520    16414  19414   7516    838     84
-4178       O
ATOM   3879  OE2 GLU A 520       7.071  -8.715  60.705  1.00102.40
O
ANISOU 3879  OE2 GLU A 520    14220  18278   6409    547    102
-3964       O
ATOM   3880  N   THR A 521       3.588  -8.175  57.762  1.00108.15
N
ANISOU 3880  N   THR A 521    14269  19217   7605    747    818
-3788       N
ATOM   3881  CA  THR A 521       3.810  -8.929  56.530  1.00119.73
C
ANISOU 3881  CA  THR A 521    15327  20773   9391    475    777
-3666       C
ATOM   3882  C   THR A 521       4.447  -8.063  55.449  1.00 91.39
C
ANISOU 3882  C   THR A 521    11727  17021   5977    227    542
-3776       C
ATOM   3883  O   THR A 521       5.424  -8.465  54.807  1.00 89.56
O
ANISOU 3883  O   THR A 521    11325  16818   5887    -46    354
-3769       O
ATOM   3884  CB  THR A 521       2.493  -9.526  56.031  1.00 91.65
C
ANISOU 3884  CB  THR A 521    11473  17332   6019    585   1073
-3452       C
ATOM   3885  OG1 THR A 521       2.071 -10.567  56.921  1.00 92.40
O
ANISOU 3885  OG1 THR A 521    11517  17599   5992    738   1278
-3314       O
ATOM   3886  CG2 THR A 521       2.663 -10.107  54.640  1.00 88.63
C
ANISOU 3886  CG2 THR A 521    10737  16980   5958    308   1002
-3347       C
ATOM   3887  N   GLY A 522       3.893  -6.872  55.227  1.00 92.64
N
ANISOU 3887  N   GLY A 522    12072  17007   6121    338    568
-3864       N
ATOM   3888  CA  GLY A 522       4.448  -5.972  54.229  1.00 91.88
C
ANISOU 3888  CA  GLY A 522    12004  16731   6175    114    361
-3967       C
ATOM   3889  C   GLY A 522       5.911  -5.617  54.429  1.00 92.39
C
ANISOU 3889  C   GLY A 522    12230  16712   6161   -143     34
-4118       C
ATOM   3890  O   GLY A 522       6.658  -5.485  53.456  1.00146.41
O
ANISOU 3890  O   GLY A 522    18920  23508  13202   -422   -136
-4123       O
ATOM   3891  N   ARG A 523       6.346  -5.446  55.681  1.00 94.79
N
ANISOU 3891  N   ARG A 523    12841  17000   6172    -55    -63
```

```
-4234       N
ATOM   3892  CA  ARG A 523       7.769  -5.187  55.896  1.00112.62
C
ANISOU 3892  CA  ARG A 523    15210  19164   8418   -324   -400
-4316       C
ATOM   3893  C   ARG A 523       8.639  -6.389  55.529  1.00 96.79
C
ANISOU 3893  C   ARG A 523    12831  17358   6588   -531   -478
-4175       C
ATOM   3894  O   ARG A 523       9.714  -6.216  54.944  1.00 92.77
O
ANISOU 3894  O   ARG A 523    12208  16815   6224   -817   -710
-4180       O
ATOM   3895  CB  ARG A 523       8.023  -4.731  57.335  1.00126.17
C
ANISOU 3895  CB  ARG A 523    17367  20736   9836   -179   -508
-4416       C
ATOM   3896  CG  ARG A 523       9.461  -4.262  57.567  1.00114.63
C
ANISOU 3896  CG  ARG A 523    16050  19143   8363   -473   -892
-4501       C
ATOM   3897  CD  ARG A 523      10.326  -5.273  58.302  1.00143.98
C
ANISOU 3897  CD  ARG A 523    19649  23017  12039   -523  -1006
-4413       C
ATOM   3898  NE  ARG A 523      10.012  -5.309  59.727  1.00162.33
N
ANISOU 3898  NE  ARG A 523    22336  25284  14056   -255   -957
-4454       N
ATOM   3899  CZ  ARG A 523       9.183  -6.170  60.304  1.00149.56
C
ANISOU 3899  CZ  ARG A 523    20674  23807  12344     32   -670
-4351       C
ATOM   3900  NH1 ARG A 523       8.775  -7.245  59.648  1.00157.90
N
ANISOU 3900  NH1 ARG A 523    21312  25092  13593     45   -455
-4191       N
ATOM   3901  NH2 ARG A 523       8.951  -6.075  61.607  1.00138.72
N
ANISOU 3901  NH2 ARG A 523    19676  22347  10685    269   -632
-4390       N
ATOM   3902  N   ARG A 524       8.210  -7.614  55.858  1.00103.21
N
ANISOU 3902  N   ARG A 524    13454  18372   7389   -386   -279
-4031       N
ATOM   3903  CA  ARG A 524       9.018  -8.758  55.438  1.00 97.27
C
ANISOU 3903  CA  ARG A 524    12381  17790   6789   -557   -341
-3893       C
ATOM   3904  C   ARG A 524       8.833  -9.060  53.957  1.00 93.50
C
ANISOU 3904  C   ARG A 524    11577  17381   6567   -713   -273
-3811       C
ATOM   3905  O   ARG A 524       9.749  -9.590  53.317  1.00133.08
```

```
O
ANISOU 3905  O   ARG A 524    16371  22467  11728   -910   -392
-3730       O
ATOM   3906  CB  ARG A 524       8.717 -10.008  56.273  1.00103.69
C
ANISOU 3906  CB  ARG A 524    13139  18764   7496   -370   -169
-3761       C
ATOM   3907  CG  ARG A 524       7.279 -10.488  56.276  1.00 89.56
C
ANISOU 3907  CG  ARG A 524    11273  17061   5695   -149    164
-3665       C
ATOM   3908  CD  ARG A 524       7.189 -11.896  56.870  1.00 89.86
C
ANISOU 3908  CD  ARG A 524    11202  17252   5689    -47    310
-3497       C
ATOM   3909  NE  ARG A 524       7.664 -11.980  58.249  1.00 91.46
N
ANISOU 3909  NE  ARG A 524    11669  17417   5664     90    240
-3530       N
ATOM   3910  CZ  ARG A 524       7.065 -11.422  59.296  1.00102.76
C
ANISOU 3910  CZ  ARG A 524    13409  18757   6879    331    334
-3595       C
ATOM   3911  NH1 ARG A 524       5.906 -10.793  59.155  1.00104.75
N
ANISOU 3911  NH1 ARG A 524    13717  18969   7114    491    535
-3614       N
ATOM   3912  NH2 ARG A 524       7.597 -11.554  60.504  1.00 96.08
N
ANISOU 3912  NH2 ARG A 524    12816  17868   5822    437    245
-3624       N
ATOM   3913  N   LEU A 525       7.663  -8.738  53.403  1.00101.71
N
ANISOU 3913  N   LEU A 525    12588  18363   7694   -603    -80
-3790       N
ATOM   3914  CA  LEU A 525       7.490  -8.757  51.954  1.00113.85
C
ANISOU 3914  CA  LEU A 525    13883  19853   9520   -748    -56
-3702       C
ATOM   3915  C   LEU A 525       8.429  -7.765  51.281  1.00 90.03
C
ANISOU 3915  C   LEU A 525    10925  16688   6594   -984   -294
-3810       C
ATOM   3916  O   LEU A 525       9.057  -8.076  50.261  1.00 82.82
O
ANISOU 3916  O   LEU A 525     9794  15799   5877  -1180   -369
-3731       O
ATOM   3917  CB  LEU A 525       6.037  -8.439  51.609  1.00108.11
C
ANISOU 3917  CB  LEU A 525    13143  19063   8871   -563    171
-3652       C
ATOM   3918  CG  LEU A 525       5.681  -8.247  50.138  1.00 84.72
C
ANISOU 3918  CG  LEU A 525     9988  16020   6182   -675    190
```

```
-3582       C
ATOM   3919  CD1 LEU A 525       5.806  -9.558  49.384  1.00123.96
C
ANISOU 3919  CD1 LEU A 525    14653  21122  11323   -793    231
-3407       C
ATOM   3920  CD2 LEU A 525       4.282  -7.673  50.016  1.00 85.53
C
ANISOU 3920  CD2 LEU A 525    10127  16061   6309   -457    381
-3561       C
ATOM   3921  N   GLY A 526       8.527  -6.568  51.836  1.00107.39
N
ANISOU 3921  N   GLY A 526    13439  18722   8644   -963   -408
-3982       N
ATOM   3922  CA  GLY A 526       9.430  -5.538  51.386  1.00 98.08
C
ANISOU 3922  CA  GLY A 526    12371  17380   7515  -1204   -650
-4094       C
ATOM   3923  C   GLY A 526       8.747  -4.339  50.771  1.00 90.04
C
ANISOU 3923  C   GLY A 526    11532  16121   6560  -1165   -607
-4172       C
ATOM   3924  O   GLY A 526       9.380  -3.629  49.979  1.00 97.29
O
ANISOU 3924  O   GLY A 526    12459  16901   7606  -1392   -760
-4212       O
ATOM   3925  N   MET A 527       7.484  -4.093  51.110  1.00 89.03
N
ANISOU 3925  N   MET A 527    11542  15943   6340   -873   -395
-4181       N
ATOM   3926  CA  MET A 527       6.612  -3.219  50.342  1.00 88.98
C
ANISOU 3926  CA  MET A 527    11618  15762   6429   -774   -289
-4198       C
ATOM   3927  C   MET A 527       6.610  -1.787  50.873  1.00 91.98
C
ANISOU 3927  C   MET A 527    12465  15881   6604   -709   -389
-4390       C
ATOM   3928  O   MET A 527       6.073  -0.890  50.213  1.00103.58
O
ANISOU 3928  O   MET A 527    14061  17164   8131   -648   -340
-4425       O
ATOM   3929  CB  MET A 527       5.198  -3.821  50.336  1.00 88.24
C
ANISOU 3929  CB  MET A 527    11362  15796   6371   -484      9
-4061       C
ATOM   3930  CG  MET A 527       4.104  -2.993  49.693  1.00 95.94
C
ANISOU 3930  CG  MET A 527    12402  16638   7414   -304    150
-4055       C
ATOM   3931  SD  MET A 527       2.586  -3.942  49.531  1.00 92.00
S
ANISOU 3931  SD  MET A 527    11572  16361   7022    -48    464
-3838       S
ATOM   3932  CE  MET A 527       2.961  -4.913  48.080  1.00 84.19
```

```
C
ANISOU 3932  CE  MET A 527    10167  15458   6362   -338    396
-3691       C
ATOM   3933  N   GLY A 528       7.250  -1.539  52.014  1.00 94.31
N
ANISOU 3933  N   GLY A 528    13043  16139   6649   -730   -549
-4518       N
ATOM   3934  CA  GLY A 528       7.093  -0.276  52.709  1.00 97.60
C
ANISOU 3934  CA  GLY A 528    13977  16297   6809   -611   -623
-4701       C
ATOM   3935  C   GLY A 528       5.818  -0.203  53.525  1.00125.61
C
ANISOU 3935  C   GLY A 528    17730  19855  10142   -169   -355
-4695       C
ATOM   3936  O   GLY A 528       4.821  -0.849  53.189  1.00162.10
O
ANISOU 3936  O   GLY A 528    22064  24640  14885     33    -86
-4538       O
ATOM   3937  N   THR A 529       5.839   0.575  54.608  1.00118.11
N
ANISOU 3937  N   THR A 529    17281  18732   8863    -15   -428
-4856       N
ATOM   3938  CA  THR A 529       4.804   0.524  55.633  1.00111.77
C
ANISOU 3938  CA  THR A 529    16699  17969   7798    426   -177
-4843       C
ATOM   3939  C   THR A 529       3.939   1.779  55.646  1.00120.62
C
ANISOU 3939  C   THR A 529    18243  18832   8754    722    -55
-4927       C
ATOM   3940  O   THR A 529       3.184   1.993  56.600  1.00109.47
O
ANISOU 3940  O   THR A 529    17135  17399   7061   1113    130
-4943       O
ATOM   3941  CB  THR A 529       5.429   0.305  57.013  1.00119.79
C
ANISOU 3941  CB  THR A 529    18002  18980   8531    450   -317
-4922       C
ATOM   3942  OG1 THR A 529       6.410   1.320  57.266  1.00134.61
O
ANISOU 3942  OG1 THR A 529    20302  20546  10298    206   -656
-5085       O
ATOM   3943  CG2 THR A 529       6.090  -1.068  57.086  1.00104.35
C
ANISOU 3943  CG2 THR A 529    15616  17304   6728    256   -368
-4791       C
ATOM   3944  N   ASN A 530       4.034   2.611  54.608  1.00159.46
N
ANISOU 3944  N   ASN A 530    23204  23553  13829    566   -142
-4971       N
ATOM   3945  CA  ASN A 530       3.197   3.803  54.460  1.00152.32
C
ANISOU 3945  CA  ASN A 530    22686  22399  12788    855    -17
```

```
-5036       C
ATOM   3946  C   ASN A 530       1.817   3.400  53.934  1.00119.90
C
ANISOU 3946  C   ASN A 530    18234  18491   8834   1189    346
-4836       C
ATOM   3947  O   ASN A 530       1.439   3.667  52.791  1.00111.95
O
ANISOU 3947  O   ASN A 530    17029  17452   8056   1151    400
-4769       O
ATOM   3948  CB  ASN A 530       3.875   4.814  53.544  1.00151.33
C
ANISOU 3948  CB  ASN A 530    22743  21984  12772    544   -254
-5153       C
ATOM   3949  CG  ASN A 530       3.132   6.137  53.481  1.00138.89
C
ANISOU 3949  CG  ASN A 530    21670  20101  11002    842   -156
-5246       C
ATOM   3950  OD1 ASN A 530       2.197   6.374  54.245  1.00126.57
O
ANISOU 3950  OD1 ASN A 530    20376  18531   9183   1294     69
-5240       O
ATOM   3951  ND2 ASN A 530       3.543   7.004  52.563  1.00143.23
N
ANISOU 3951  ND2 ASN A 530    22359  20396  11665    607   -310
-5319       N
ATOM   3952  N   MET A 531       1.051   2.741  54.803  1.00108.22
N
ANISOU 3952  N   MET A 531    16675  17224   7218   1519    593
-4728       N
ATOM   3953  CA  MET A 531      -0.231   2.171  54.413  1.00107.18
C
ANISOU 3953  CA  MET A 531    16137  17340   7245   1798    928
-4502       C
ATOM   3954  C   MET A 531      -1.250   2.455  55.510  1.00120.09
C
ANISOU 3954  C   MET A 531    18060  19004   8563   2323   1214
-4463       C
ATOM   3955  O   MET A 531      -0.896   2.741  56.656  1.00113.14
O
ANISOU 3955  O   MET A 531    17630  18007   7352   2447   1158
-4594       O
ATOM   3956  CB  MET A 531      -0.104   0.671  54.110  1.00103.87
C
ANISOU 3956  CB  MET A 531    15123  17245   7098   1578    963
-4324       C
ATOM   3957  CG  MET A 531       0.652  -0.140  55.138  1.00106.64
C
ANISOU 3957  CG  MET A 531    15505  17708   7306   1471    870
-4357       C
ATOM   3958  SD  MET A 531       0.670  -1.885  54.681  1.00117.65
S
ANISOU 3958  SD  MET A 531    16235  19455   9010   1254    945
-4130       S
ATOM   3959  CE  MET A 531      -0.950  -2.409  55.233  1.00123.11
```

```
C
ANISOU 3959  CE  MET A 531    16742  20387   9649   1703   1373
-3902       C
ATOM   3960  N   TYR A 532      -2.528   2.376  55.143  1.00138.19
N
ANISOU 3960  N   TYR A 532    20091  21460  10955   2640   1522
-4271       N
ATOM   3961  CA  TYR A 532      -3.591   2.978  55.935  1.00114.52
C
ANISOU 3961  CA  TYR A 532    17400  18448   7663   3192   1816
-4224       C
ATOM   3962  C   TYR A 532      -4.797   2.053  56.011  1.00114.33
C
ANISOU 3962  C   TYR A 532    16865  18801   7774   3452   2171
-3930       C
ATOM   3963  O   TYR A 532      -5.060   1.295  55.070  1.00121.64
O
ANISOU 3963  O   TYR A 532    17226  19936   9055   3237   2187
-3767       O
ATOM   3964  CB  TYR A 532      -4.011   4.320  55.314  1.00116.25
C
ANISOU 3964  CB  TYR A 532    17943  18410   7819   3389   1830
-4302       C
ATOM   3965  CG  TYR A 532      -2.880   5.322  55.261  1.00117.02
C
ANISOU 3965  CG  TYR A 532    18588  18102   7772   3127   1487
-4581       C
ATOM   3966  CD1 TYR A 532      -2.665   6.219  56.295  1.00120.82
C
ANISOU 3966  CD1 TYR A 532    19681  18235   7991   3239   1426
-4674       C
ATOM   3967  CD2 TYR A 532      -2.001   5.341  54.185  1.00114.14
C
ANISOU 3967  CD2 TYR A 532    18042  17641   7685   2641   1206
-4655       C
ATOM   3968  CE1 TYR A 532      -1.622   7.130  56.244  1.00130.61
C
ANISOU 3968  CE1 TYR A 532    21377  19095   9152   2924   1094
-4893       C
ATOM   3969  CE2 TYR A 532      -0.951   6.236  54.129  1.00115.08
C
ANISOU 3969  CE2 TYR A 532    18628  17407   7691   2365    892
-4888       C
ATOM   3970  CZ  TYR A 532      -0.768   7.131  55.158  1.00136.32
C
ANISOU 3970  CZ  TYR A 532    21949  19783  10064   2500    831
-5011       C
ATOM   3971  OH  TYR A 532       0.280   8.023  55.101  1.00137.89
O
ANISOU 3971  OH  TYR A 532    22563  19618  10210   2150    503
-5200       O
ATOM   3972  N   PRO A 533      -5.542   2.087  57.118  1.00117.55
N
ANISOU 3972  N   PRO A 533    17450  19261   7952   3870   2442
```

```
-3819       N
ATOM   3973  CA  PRO A 533      -6.915   1.560  57.103  1.00118.45
C
ANISOU 3973  CA  PRO A 533    17119  19699   8185   4194   2817
-3512       C
ATOM   3974  C   PRO A 533      -7.778   2.236  56.044  1.00157.67
C
ANISOU 3974  C   PRO A 533    21922  24711  13275   4391   2930
-3436       C
ATOM   3975  O   PRO A 533      -7.865   3.465  55.977  1.00141.48
O
ANISOU 3975  O   PRO A 533    20313  22359  11083   4582   2895
-3542       O
ATOM   3976  CB  PRO A 533      -7.422   1.853  58.523  1.00122.66
C
ANISOU 3976  CB  PRO A 533    18011  20115   8480   4572   3023
-3414       C
ATOM   3977  CG  PRO A 533      -6.177   2.148  59.325  1.00174.88
C
ANISOU 3977  CG  PRO A 533    25160  26415  14872   4361   2733
-3667       C
ATOM   3978  CD  PRO A 533      -5.271   2.827  58.358  1.00160.22
C
ANISOU 3978  CD  PRO A 533    23476  24340  13060   4039   2403
-3917       C
ATOM   3979  N   SER A 534      -8.419   1.409  55.213  1.00176.75
N
ANISOU 3979  N   SER A 534    23683  27433  16040   4267   3026
-3205       N
ATOM   3980  CA  SER A 534      -9.344   1.901  54.194  1.00163.68
C
ANISOU 3980  CA  SER A 534    21775  25848  14570   4427   3126
-3071       C
ATOM   3981  C   SER A 534     -10.498   2.698  54.795  1.00158.54
C
ANISOU 3981  C   SER A 534    21324  25244  13668   5056   3468
-2944       C
ATOM   3982  O   SER A 534     -10.925   3.710  54.226  1.00177.36
O
ANISOU 3982  O   SER A 534    23877  27489  16024   5280   3488
-2966       O
ATOM   3983  CB  SER A 534      -9.882   0.730  53.371  1.00141.82
C
ANISOU 3983  CB  SER A 534    18261  23415  12209   4174   3162
-2814       C
ATOM   3984  OG  SER A 534     -10.477  -0.248  54.206  1.00148.09
O
ANISOU 3984  OG  SER A 534    18760  24517  12991   4300   3415
-2589       O
ATOM   3985  N   SER A 535     -11.024   2.249  55.938  1.00161.79
N
ANISOU 3985  N   SER A 535    21708  25760  14003   5271   3694
-2752       N
ATOM   3986  CA  SER A 535     -12.148   2.934  56.573  1.00159.10
```

```
C
ANISOU 3986  CA  SER A 535    21510  25396  13546   5785   3978
-2550       C
ATOM   3987  C   SER A 535     -11.826   4.385  56.914  1.00133.37
C
ANISOU 3987  C   SER A 535    18990  21686   9999   6006   3878
-2763       C
ATOM   3988  O   SER A 535     -12.698   5.257  56.821  1.00134.48
O
ANISOU 3988  O   SER A 535    19245  21772  10081   6403   4039
-2647       O
ATOM   3989  CB  SER A 535     -12.575   2.176  57.831  1.00179.29
C
ANISOU 3989  CB  SER A 535    23973  28104  16047   5931   4203
-2340       C
ATOM   3990  OG  SER A 535     -11.483   2.004  58.717  1.00221.26
O
ANISOU 3990  OG  SER A 535    29714  33193  21164   5739   4028
-2556       O
ATOM   3991  N   ALA A 536     -10.585   4.665  57.321  1.00130.42
N
ANISOU 3991  N   ALA A 536    19120  20985   9448   5744   3600
-3060       N
ATOM   3992  CA  ALA A 536     -10.173   6.050  57.538  1.00154.03
C
ANISOU 3992  CA  ALA A 536    22815  23517  12191   5860   3449
-3275       C
ATOM   3993  C   ALA A 536     -10.269   6.877  56.260  1.00161.56
C
ANISOU 3993  C   ALA A 536    23791  24355  13239   5847   3355
-3357       C
ATOM   3994  O   ALA A 536     -10.677   8.044  56.297  1.00162.06
O
ANISOU 3994  O   ALA A 536    24261  24163  13151   6157   3403
-3368       O
ATOM   3995  CB  ALA A 536      -8.751   6.095  58.098  1.00131.86
C
ANISOU 3995  CB  ALA A 536    20457  20414   9229   5485   3121
-3566       C
ATOM   3996  N   LEU A 537      -9.902   6.292  55.121  1.00127.79
N
ANISOU 3996  N   LEU A 537    19093  20258   9202   5502   3219
-3408       N
ATOM   3997  CA  LEU A 537      -9.827   7.016  53.857  1.00126.54
C
ANISOU 3997  CA  LEU A 537    18973  19971   9136   5432   3090
-3515       C
ATOM   3998  C   LEU A 537     -11.061   6.845  52.978  1.00143.11
C
ANISOU 3998  C   LEU A 537    20523  22410  11441   5697   3323
-3258       C
ATOM   3999  O   LEU A 537     -11.171   7.531  51.956  1.00126.17
O
ANISOU 3999  O   LEU A 537    18425  20159   9356   5726   3252
```

```
-3318       O
ATOM   4000  CB  LEU A 537      -8.590   6.564  53.076  1.00122.14
C
ANISOU 4000  CB  LEU A 537    18319  19378   8708   4882   2760
-3744       C
ATOM   4001  CG  LEU A 537      -7.255   6.784  53.786  1.00122.03
C
ANISOU 4001  CG  LEU A 537    18813  19030   8524   4554   2468
-4001       C
ATOM   4002  CD1 LEU A 537      -6.143   6.137  52.989  1.00117.71
C
ANISOU 4002  CD1 LEU A 537    17981  18490   8253   3946   2149
-4107       C
ATOM   4003  CD2 LEU A 537      -6.989   8.266  53.993  1.00125.18
C
ANISOU 4003  CD2 LEU A 537    19920  18935   8709   4653   2350
-4163       C
ATOM   4004  N   LEU A 538     -11.977   5.951  53.341  1.00156.99
N
ANISOU 4004  N   LEU A 538    21759  24569  13321   5870   3586
-2966       N
ATOM   4005  CA  LEU A 538     -13.272   5.870  52.676  1.00142.39
C
ANISOU 4005  CA  LEU A 538    19392  23047  11661   6156   3816
-2675       C
ATOM   4006  C   LEU A 538     -14.056   7.172  52.814  1.00175.09
C
ANISOU 4006  C   LEU A 538    23912  26982  15632   6655   3960
-2604       C
ATOM   4007  O   LEU A 538     -14.212   7.708  53.915  1.00173.75
O
ANISOU 4007  O   LEU A 538    24181  26614  15221   6938   4067
-2586       O
ATOM   4008  CB  LEU A 538     -14.080   4.705  53.245  1.00152.81
C
ANISOU 4008  CB  LEU A 538    20133  24795  13133   6220   4062
-2353       C
ATOM   4009  CG  LEU A 538     -15.413   4.415  52.555  1.00142.42
C
ANISOU 4009  CG  LEU A 538    18160  23880  12073   6425   4268
-2011       C
ATOM   4010  CD1 LEU A 538     -15.190   3.596  51.292  1.00126.15
C
ANISOU 4010  CD1 LEU A 538    15541  22038  10352   5979   4078
-2005       C
ATOM   4011  CD2 LEU A 538     -16.365   3.706  53.505  1.00159.94
C
ANISOU 4011  CD2 LEU A 538    20031  26391  14349   6626   4553
-1667       C
ATOM   4012  N   GLY A 539     -14.551   7.674  51.684  1.00181.39
N
ANISOU 4012  N   GLY A 539    24544  27828  16548   6770   3955
-2560       N
ATOM   4013  CA  GLY A 539     -15.223   8.958  51.603  1.00190.59
```

```
C
ANISOU 4013  CA  GLY A 539    26074  28782  17559   7220   4056
-2509       C
ATOM   4014  C   GLY A 539     -14.487  10.164  52.149  1.00195.84
C
ANISOU 4014  C   GLY A 539    27606  28892  17912   7274   3913
-2772       C
ATOM   4015  O   GLY A 539     -13.262  10.145  52.309  1.00186.91
O
ANISOU 4015  O   GLY A 539    26822  27486  16708   6878   3659
-3054       O
ATOM   4016  N   THR A 540     -15.239  11.229  52.438  1.00208.35
N
ANISOU 4016  N   THR A 540    29536  30314  19313   7756   4062
-2666       N
ATOM   4017  CA  THR A 540     -14.683  12.472  52.960  1.00199.78
C
ANISOU 4017  CA  THR A 540    29288  28694  17924   7848   3937
-2877       C
ATOM   4018  C   THR A 540     -15.206  12.824  54.347  1.00171.28
C
ANISOU 4018  C   THR A 540    26017  25009  14053   8269   4135
-2739       C
ATOM   4019  O   THR A 540     -14.929  13.924  54.840  1.00180.30
O
ANISOU 4019  O   THR A 540    27862  25721  14923   8427   4059
-2867       O
ATOM   4020  CB  THR A 540     -14.966  13.631  51.997  1.00185.38
C
ANISOU 4020  CB  THR A 540    27726  26642  16070   8040   3891
-2913       C
ATOM   4021  OG1 THR A 540     -16.373  13.704  51.729  1.00201.35
O
ANISOU 4021  OG1 THR A 540    29349  28997  18159   8535   4170
-2582       O
ATOM   4022  CG2 THR A 540     -14.214  13.432  50.685  1.00147.55
C
ANISOU 4022  CG2 THR A 540    22761  21810  11490   7583   3644
-3109       C
ATOM   4023  N   HIS A 541     -15.954  11.930  54.988  1.00168.54
N
ANISOU 4023  N   HIS A 541    25201  25058  13778   8452   4382
-2471       N
ATOM   4024  CA  HIS A 541     -16.678  12.249  56.211  1.00209.97
C
ANISOU 4024  CA  HIS A 541    30693  30293  18792   8935   4621
-2274       C
ATOM   4025  C   HIS A 541     -15.982  11.731  57.462  1.00197.57
C
ANISOU 4025  C   HIS A 541    29373  28619  17077   8766   4572
-2378       C
ATOM   4026  O   HIS A 541     -16.500  11.922  58.567  1.00180.65
O
ANISOU 4026  O   HIS A 541    27459  26453  14725   9150   4762
```

```
-2224       O
ATOM   4027  CB  HIS A 541     -18.107  11.700  56.141  1.00222.23
C
ANISOU 4027  CB  HIS A 541    31570  32356  20513   9304   4952
-1857       C
ATOM   4028  CG  HIS A 541     -18.883  12.187  54.956  1.00217.61
C
ANISOU 4028  CG  HIS A 541    30701  31913  20066   9507   5003
-1721       C
ATOM   4029  ND1 HIS A 541     -19.097  13.525  54.708  1.00217.90
N
ANISOU 4029  ND1 HIS A 541    31263  31632  19895   9845   4986
-1767       N
ATOM   4030  CD2 HIS A 541     -19.492  11.517  53.950  1.00203.50
C
ANISOU 4030  CD2 HIS A 541    28169  30550  18601   9418   5057
-1534       C
ATOM   4031  CE1 HIS A 541     -19.806  13.659  53.602  1.00211.33
C
ANISOU 4031  CE1 HIS A 541    30022  31029  19246   9972   5034
-1618       C
ATOM   4032  NE2 HIS A 541     -20.059  12.455  53.122  1.00200.84
N
ANISOU 4032  NE2 HIS A 541    27913  30154  18242   9714   5071
-1476       N
ATOM   4033  N   LYS A 542     -14.827  11.085  57.316  1.00204.45
N
ANISOU 4033  N   LYS A 542    30210  29429  18043   8216   4321
-2625       N
ATOM   4034  CA  LYS A 542     -14.148  10.419  58.419  1.00204.92
C
ANISOU 4034  CA  LYS A 542    30408  29451  18000   8017   4261
-2710       C
ATOM   4035  C   LYS A 542     -12.969  11.228  58.947  1.00219.17
C
ANISOU 4035  C   LYS A 542    32990  30739  19546   7813   3960
-3041       C
ATOM   4036  O   LYS A 542     -12.218  10.732  59.794  1.00226.63
O
ANISOU 4036  O   LYS A 542    34092  31616  20402   7586   3843
-3158       O
ATOM   4037  CB  LYS A 542     -13.688   9.025  57.989  1.00174.13
C
ANISOU 4037  CB  LYS A 542    25899  25883  14381   7549   4190
-2721       C
ATOM   4038  CG  LYS A 542     -14.835   8.097  57.620  1.00165.86
C
ANISOU 4038  CG  LYS A 542    24060  25359  13601   7699   4474
-2370       C
ATOM   4039  CD  LYS A 542     -15.775   7.899  58.800  1.00160.22
C
ANISOU 4039  CD  LYS A 542    23301  24804  12771   8129   4786
-2080       C
ATOM   4040  CE  LYS A 542     -16.766   6.776  58.543  1.00170.38
```

```
C
ANISOU 4040  CE  LYS A 542    23760  26621  14356   8152   5031
-1726       C
ATOM   4041  NZ  LYS A 542     -17.703   7.106  57.434  1.00193.89
N
ANISOU 4041  NZ  LYS A 542    26329  29816  17526   8335   5125
-1538       N
ATOM   4042  N   ASP A 543     -12.800  12.462  58.466  1.00223.32
N
ANISOU 4042  N   ASP A 543    33996  30901  19955   7881   3825
-3181       N
ATOM   4043  CA  ASP A 543     -11.716  13.356  58.863  1.00223.60
C
ANISOU 4043  CA  ASP A 543    34771  30423  19764   7662   3517
-3478       C
ATOM   4044  C   ASP A 543     -10.343  12.703  58.728  1.00229.46
C
ANISOU 4044  C   ASP A 543    35457  31113  20614   7016   3191
-3738       C
ATOM   4045  O   ASP A 543      -9.950  12.301  57.627  1.00217.45
O
ANISOU 4045  O   ASP A 543    33572  29713  19335   6657   3066
-3817       O
ATOM   4046  CB  ASP A 543     -11.948  13.845  60.298  1.00203.19
C
ANISOU 4046  CB  ASP A 543    32724  27630  16848   8043   3612
-3421       C
ATOM   4047  CG  ASP A 543     -11.079  15.036  60.663  1.00198.50
C
ANISOU 4047  CG  ASP A 543    32952  26476  15995   7926   3320
-3677       C
ATOM   4048  OD1 ASP A 543     -10.434  15.605  59.758  1.00199.01
O
ANISOU 4048  OD1 ASP A 543    33170  26308  16138   7599   3077
-3869       O
ATOM   4049  OD2 ASP A 543     -11.041  15.401  61.856  1.00197.76
O
ANISOU 4049  OD2 ASP A 543    33351  26170  15620   8155   3328
-3676       O
ATOM   4050  N   ALA A 544      -9.605  12.624  59.840  1.00224.00
N
ANISOU 4050  N   ALA A 544    35138  30239  19734   6881   3044
-3865       N
ATOM   4051  CA  ALA A 544      -8.239  12.092  59.878  1.00203.25
C
ANISOU 4051  CA  ALA A 544    32520  27538  17166   6287   2709
-4106       C
ATOM   4052  C   ALA A 544      -7.277  12.960  59.066  1.00200.87
C
ANISOU 4052  C   ALA A 544    32554  26875  16892   5889   2365
-4361       C
ATOM   4053  O   ALA A 544      -6.448  12.454  58.307  1.00174.86
O
ANISOU 4053  O   ALA A 544    28987  23654  13799   5401   2149
```

```
-4492       O
ATOM   4054  CB  ALA A 544      -8.188  10.632  59.413  1.00170.70
C
ANISOU 4054  CB  ALA A 544    27668  23870  13321   6029   2771
-4024       C
ATOM   4055  N   ASN A 545      -7.412  14.283  59.214  1.00207.60
N
ANISOU 4055  N   ASN A 545    34005  27333  17540   6108   2321
-4411       N
ATOM   4056  CA  ASN A 545      -6.531  15.273  58.582  1.00185.73
C
ANISOU 4056  CA  ASN A 545    31647  24159  14764   5756   2000
-4635       C
ATOM   4057  C   ASN A 545      -6.702  15.297  57.061  1.00180.06
C
ANISOU 4057  C   ASN A 545    30565  23544  14305   5620   2013
-4625       C
ATOM   4058  O   ASN A 545      -5.728  15.381  56.312  1.00190.08
O
ANISOU 4058  O   ASN A 545    31827  24680  15714   5121   1731
-4803       O
ATOM   4059  CB  ASN A 545      -5.067  15.049  58.972  1.00174.34
C
ANISOU 4059  CB  ASN A 545    30369  22557  13313   5175   1615
-4870       C
ATOM   4060  CG  ASN A 545      -4.886  14.901  60.471  1.00184.35
C
ANISOU 4060  CG  ASN A 545    31952  23759  14334   5305   1594
-4876       C
ATOM   4061  OD1 ASN A 545      -4.437  13.862  60.956  1.00185.57
O
ANISOU 4061  OD1 ASN A 545    31816  24150  14541   5098   1545
-4889       O
ATOM   4062  ND2 ASN A 545      -5.243  15.942  61.214  1.00159.22
N
ANISOU 4062  ND2 ASN A 545    29384  20244  10867   5666   1632
-4859       N
ATOM   4063  N   LEU A 546      -7.955  15.210  56.612  1.00175.88
N
ANISOU 4063  N   LEU A 546    29716  23267  13844   6072   2341
-4400       N
ATOM   4064  CA  LEU A 546      -8.268  15.252  55.185  1.00180.73
C
ANISOU 4064  CA  LEU A 546    29983  23996  14689   6021   2377
-4364       C
ATOM   4065  C   LEU A 546      -7.814  16.558  54.538  1.00191.58
C
ANISOU 4065  C   LEU A 546    31875  24912  16004   5892   2172
-4518       C
ATOM   4066  O   LEU A 546      -7.330  16.562  53.400  1.00154.03
O
ANISOU 4066  O   LEU A 546    26953  20126  11447   5549   2019
-4615       O
ATOM   4067  CB  LEU A 546      -9.770  15.044  54.983  1.00170.48
```

```
C
ANISOU 4067  CB  LEU A 546    28291  23044  13440   6587   2764
-4068       C
ATOM   4068  CG  LEU A 546     -10.328  14.947  53.561  1.00179.74
C
ANISOU 4068  CG  LEU A 546    29009  24428  14856   6627   2849
-3976       C
ATOM   4069  CD1 LEU A 546     -11.489  13.977  53.543  1.00165.09
C
ANISOU 4069  CD1 LEU A 546    26472  23109  13146   6956   3174
-3681       C
ATOM   4070  CD2 LEU A 546     -10.777  16.309  53.044  1.00170.66
C
ANISOU 4070  CD2 LEU A 546    28289  22961  13592   6922   2872
-3965       C
ATOM   4071  N   ALA A 547      -7.981  17.676  55.246  1.00196.35
N
ANISOU 4071  N   ALA A 547    33122  25150  16331   6173   2173
-4529       N
ATOM   4072  CA  ALA A 547      -7.506  18.971  54.760  1.00191.71
C
ANISOU 4072  CA  ALA A 547    33095  24086  15660   6040   1968
-4668       C
ATOM   4073  C   ALA A 547      -5.995  19.009  54.544  1.00200.33
C
ANISOU 4073  C   ALA A 547    34339  24937  16839   5346   1569
-4924       C
ATOM   4074  O   ALA A 547      -5.525  19.580  53.552  1.00191.24
O
ANISOU 4074  O   ALA A 547    33287  23573  15804   5066   1406
-5021       O
ATOM   4075  CB  ALA A 547      -7.928  20.074  55.733  1.00177.96
C
ANISOU 4075  CB  ALA A 547    32044  21999  13573   6476   2035
-4620       C
ATOM   4076  N   SER A 548      -5.215  18.423  55.455  1.00220.77
N
ANISOU 4076  N   SER A 548    36942  27563  19380   5063   1406
-5022       N
ATOM   4077  CA  SER A 548      -3.764  18.404  55.274  1.00220.60
C
ANISOU 4077  CA  SER A 548    37002  27361  19456   4397   1017
-5239       C
ATOM   4078  C   SER A 548      -3.355  17.625  54.026  1.00197.53
C
ANISOU 4078  C   SER A 548    33501  24689  16861   4005    946
-5269       C
ATOM   4079  O   SER A 548      -2.529  18.097  53.234  1.00192.70
O
ANISOU 4079  O   SER A 548    32993  23854  16372   3585    703
-5394       O
ATOM   4080  CB  SER A 548      -3.095  17.815  56.517  1.00213.94
C
ANISOU 4080  CB  SER A 548    36220  26569  18497   4219    873
```

```
-5311       C
ATOM   4081  OG  SER A 548      -3.248  18.674  57.633  1.00218.35
O
ANISOU 4081  OG  SER A 548    37414  26809  18739   4503    862
-5317       O
ATOM   4082  N   ILE A 549      -3.919  16.438  53.827  1.00178.26
N
ANISOU 4082  N   ILE A 549    30457  22707  14567   4136   1156
-5144       N
ATOM   4083  CA  ILE A 549      -3.709  15.683  52.596  1.00177.31
C
ANISOU 4083  CA  ILE A 549    29791  22839  14741   3852   1125
-5143       C
ATOM   4084  C   ILE A 549      -5.054  15.139  52.134  1.00200.22
C
ANISOU 4084  C   ILE A 549    32230  26115  17731   4324   1484
-4926       C
ATOM   4085  O   ILE A 549      -5.672  14.340  52.851  1.00176.34
O
ANISOU 4085  O   ILE A 549    28926  23407  14668   4591   1694
-4787       O
ATOM   4086  CB  ILE A 549      -2.692  14.544  52.779  1.00161.69
C
ANISOU 4086  CB  ILE A 549    27466  21078  12891   3371    921
-5228       C
ATOM   4087  CG1 ILE A 549      -1.390  15.060  53.400  1.00136.68
C
ANISOU 4087  CG1 ILE A 549    24721  17583   9628   2929    562
-5415       C
ATOM   4088  CG2 ILE A 549      -2.409  13.863  51.447  1.00183.01
C
ANISOU 4088  CG2 ILE A 549    29667  23989  15880   3076    861
-5229       C
ATOM   4089  CD1 ILE A 549      -0.369  13.977  53.657  1.00132.21
C
ANISOU 4089  CD1 ILE A 549    23824  17238   9173   2479    349
-5483       C
ATOM   4090  N   PRO A 550      -5.560  15.561  50.973  1.00211.10
N
ANISOU 4090  N   PRO A 550    33506  27474  19228   4447   1564
-4873       N
ATOM   4091  CA  PRO A 550      -6.777  14.946  50.427  1.00190.12
C
ANISOU 4091  CA  PRO A 550    30314  25226  16695   4842   1870
-4658       C
ATOM   4092  C   PRO A 550      -6.682  13.427  50.419  1.00160.43
C
ANISOU 4092  C   PRO A 550    25915  21929  13113   4667   1905
-4597       C
ATOM   4093  O   PRO A 550      -5.615  12.856  50.193  1.00126.47
O
ANISOU 4093  O   PRO A 550    21491  17637   8924   4176   1661
-4737       O
ATOM   4094  CB  PRO A 550      -6.850  15.518  49.006  1.00132.21
```

```
C
ANISOU 4094  CB  PRO A 550    22961  17767   9506   4786   1819
-4681       C
ATOM   4095  CG  PRO A 550      -6.168  16.842  49.113  1.00135.12
C
ANISOU 4095  CG  PRO A 550    24019  17595   9724   4625   1614
-4840       C
ATOM   4096  CD  PRO A 550      -5.034  16.615  50.085  1.00188.40
C
ANISOU 4096  CD  PRO A 550    30985  24210  16389   4219   1373
-4997       C
ATOM   4097  N   VAL A 551      -7.819  12.780  50.679  1.00160.38
N
ANISOU 4097  N   VAL A 551    25487  22315  13134   5077   2212
-4367       N
ATOM   4098  CA  VAL A 551      -7.863  11.320  50.721  1.00138.74
C
ANISOU 4098  CA  VAL A 551    22115  20039  10561   4948   2277
-4272       C
ATOM   4099  C   VAL A 551      -7.421  10.722  49.390  1.00125.38
C
ANISOU 4099  C   VAL A 551    19966  18498   9176   4559   2113
-4306       C
ATOM   4100  O   VAL A 551      -6.675   9.736  49.353  1.00126.50
O
ANISOU 4100  O   VAL A 551    19749  18776   9538   4101   1945
-4309       O
ATOM   4101  CB  VAL A 551      -9.265  10.832  51.133  1.00141.70
C
ANISOU 4101  CB  VAL A 551    22085  20806  10950   5449   2648
-3974       C
ATOM   4102  CG1 VAL A 551      -9.512  11.137  52.603  1.00132.34
C
ANISOU 4102  CG1 VAL A 551    21276  19497   9511   5731   2775
-3920       C
ATOM   4103  CG2 VAL A 551     -10.338  11.488  50.277  1.00147.79
C
ANISOU 4103  CG2 VAL A 551    22750  21627  11778   5836   2824
-3824       C
ATOM   4104  N   GLU A 552      -7.856  11.318  48.277  1.00130.04
N
ANISOU 4104  N   GLU A 552    20510  19010   9889   4651   2127
-4263       N
ATOM   4105  CA  GLU A 552      -7.391  10.886  46.961  1.00150.89
C
ANISOU 4105  CA  GLU A 552    22727  21686  12920   4191   1926
-4239       C
ATOM   4106  C   GLU A 552      -5.880  11.036  46.816  1.00154.86
C
ANISOU 4106  C   GLU A 552    23504  21877  13459   3627   1589
-4466       C
ATOM   4107  O   GLU A 552      -5.207  10.132  46.307  1.00112.85
O
ANISOU 4107  O   GLU A 552    17751  16691   8434   3168   1424
```

```
-4436       O
ATOM   4108  CB  GLU A 552      -8.115  11.689  45.880  1.00151.02
C
ANISOU 4108  CB  GLU A 552    22772  21617  12990   4448   2001
-4176       C
ATOM   4109  CG  GLU A 552      -9.622  11.482  45.869  1.00150.30
C
ANISOU 4109  CG  GLU A 552    22300  21889  12918   4977   2315
-3913       C
ATOM   4110  CD  GLU A 552     -10.315  12.280  44.785  1.00164.91
C
ANISOU 4110  CD  GLU A 552    24178  23666  14815   5242   2370
-3850       C
ATOM   4111  OE1 GLU A 552     -11.545  12.125  44.628  1.00166.55
O
ANISOU 4111  OE1 GLU A 552    24016  24193  15071   5654   2601
-3615       O
ATOM   4112  OE2 GLU A 552      -9.633  13.067  44.094  1.00162.51
O
ANISOU 4112  OE2 GLU A 552    24261  22990  14496   5041   2184
-4023       O
ATOM   4113  N   GLU A 553      -5.331  12.173  47.251  1.00168.91
N
ANISOU 4113  N   GLU A 553    26006  23239  14935   3654   1482
-4683       N
ATOM   4114  CA  GLU A 553      -3.882  12.363  47.237  1.00165.00
C
ANISOU 4114  CA  GLU A 553    25782  22456  14455   3110   1153
-4886       C
ATOM   4115  C   GLU A 553      -3.175  11.368  48.151  1.00129.59
C
ANISOU 4115  C   GLU A 553    21101  18155   9983   2832   1046
-4908       C
ATOM   4116  O   GLU A 553      -2.169  10.757  47.772  1.00135.44
O
ANISOU 4116  O   GLU A 553    21567  18937  10958   2324    821
-4935       O
ATOM   4117  CB  GLU A 553      -3.551  13.798  47.659  1.00122.03
C
ANISOU 4117  CB  GLU A 553    21160  16520   8686   3194   1062
-5079       C
ATOM   4118  CG  GLU A 553      -2.109  14.218  47.412  1.00121.54
C
ANISOU 4118  CG  GLU A 553    21378  16123   8676   2607    710
-5265       C
ATOM   4119  CD  GLU A 553      -1.807  14.432  45.941  1.00157.12
C
ANISOU 4119  CD  GLU A 553    25731  20535  13434   2351    612
-5264       C
ATOM   4120  OE1 GLU A 553      -0.968  13.687  45.392  1.00144.64
O
ANISOU 4120  OE1 GLU A 553    23716  19082  12159   1867    440
-5230       O
ATOM   4121  OE2 GLU A 553      -2.404  15.347  45.334  1.00156.94
```

```
O
ANISOU 4121  OE2 GLU A 553    25984  20307  13340   2623    716
-5258       O
ATOM   4122  N   LEU A 554      -3.700  11.194  49.365  1.00119.07
N
ANISOU 4122  N   LEU A 554    19915  16939   8386   3190   1219
-4886       N
ATOM   4123  CA  LEU A 554      -3.160  10.227  50.318  1.00152.49
C
ANISOU 4123  CA  LEU A 554    23978  21365  12597   3003   1152
-4892       C
ATOM   4124  C   LEU A 554      -3.243   8.789  49.803  1.00149.68
C
ANISOU 4124  C   LEU A 554    22823  21427  12622   2776   1192
-4695       C
ATOM   4125  O   LEU A 554      -2.291   8.015  49.954  1.00149.34
O
ANISOU 4125  O   LEU A 554    22574  21466  12702   2365   1000
-4729       O
ATOM   4126  CB  LEU A 554      -3.888  10.387  51.657  1.00130.16
C
ANISOU 4126  CB  LEU A 554    21437  18560   9458   3470   1379
-4841       C
ATOM   4127  CG  LEU A 554      -3.284   9.841  52.956  1.00122.50
C
ANISOU 4127  CG  LEU A 554    20569  17621   8354   3336   1293
-4883       C
ATOM   4128  CD1 LEU A 554      -3.402   8.337  53.059  1.00119.44
C
ANISOU 4128  CD1 LEU A 554    19574  17704   8103   3290   1392
-4764       C
ATOM   4129  CD2 LEU A 554      -1.825  10.277  53.096  1.00129.18
C
ANISOU 4129  CD2 LEU A 554    21774  18144   9164   2823    902
-5104       C
ATOM   4130  N   ILE A 555      -4.373   8.414  49.197  1.00113.30
N
ANISOU 4130  N   ILE A 555    17771  17084   8193   3038   1429
-4481       N
ATOM   4131  CA  ILE A 555      -4.524   7.082  48.605  1.00109.72
C
ANISOU 4131  CA  ILE A 555    16593  16995   8099   2814   1453
-4288       C
ATOM   4132  C   ILE A 555      -3.474   6.807  47.533  1.00140.21
C
ANISOU 4132  C   ILE A 555    20255  20768  12250   2277   1177
-4344       C
ATOM   4133  O   ILE A 555      -2.885   5.719  47.494  1.00132.52
O
ANISOU 4133  O   ILE A 555    18909  19980  11462   1947   1077
-4293       O
ATOM   4134  CB  ILE A 555      -5.952   6.910  48.047  1.00110.06
C
ANISOU 4134  CB  ILE A 555    16241  17300   8278   3180   1720
```

```
-4053       C
ATOM   4135  CG1 ILE A 555      -6.950   6.661  49.179  1.00112.80
C
ANISOU 4135  CG1 ILE A 555    16559  17882   8417   3647   2023
-3915       C
ATOM   4136  CG2 ILE A 555      -6.010   5.797  47.012  1.00106.35
C
ANISOU 4136  CG2 ILE A 555    15108  17092   8209   2868   1661
-3887       C
ATOM   4137  CD1 ILE A 555      -8.388   6.934  48.796  1.00114.70
C
ANISOU 4137  CD1 ILE A 555    16575  18315   8689   4112   2299
-3705       C
ATOM   4138  N   GLU A 556      -3.219   7.773  46.649  1.00173.31
N
ANISOU 4138  N   GLU A 556    24700  24676  16474   2196   1065
-4439       N
ATOM   4139  CA  GLU A 556      -2.128   7.615  45.689  1.00155.44
C
ANISOU 4139  CA  GLU A 556    22306  22300  14454   1695    813
-4494       C
ATOM   4140  C   GLU A 556      -0.775   7.419  46.368  1.00131.28
C
ANISOU 4140  C   GLU A 556    19416  19142  11322   1310    577
-4639       C
ATOM   4141  O   GLU A 556       0.007   6.551  45.960  1.00102.58
O
ANISOU 4141  O   GLU A 556    15422  15639   7915    932    440
-4593       O
ATOM   4142  CB  GLU A 556      -2.078   8.828  44.760  1.00118.97
C
ANISOU 4142  CB  GLU A 556    18023  17351   9829   1704    749
-4581       C
ATOM   4143  CG  GLU A 556      -0.955   8.790  43.736  1.00115.80
C
ANISOU 4143  CG  GLU A 556    17524  16810   9664   1210    514
-4625       C
ATOM   4144  CD  GLU A 556      -0.914  10.037  42.873  1.00148.83
C
ANISOU 4144  CD  GLU A 556    22085  20645  13819   1230    467
-4708       C
ATOM   4145  OE1 GLU A 556       0.118  10.741  42.893  1.00169.96
O
ANISOU 4145  OE1 GLU A 556    25137  23016  16423    933    270
-4860       O
ATOM   4146  OE2 GLU A 556      -1.912  10.316  42.177  1.00175.66
O
ANISOU 4146  OE2 GLU A 556    25402  24075  17264   1538    621
-4613       O
ATOM   4147  N   LYS A 557      -0.478   8.212  47.399  1.00106.62
N
ANISOU 4147  N   LYS A 557    16849  15789   7874   1408    519
-4807       N
ATOM   4148  CA  LYS A 557       0.746   8.008  48.174  1.00118.53
```

```
C
ANISOU 4148  CA  LYS A 557    18515  17232   9288   1065    280
-4937       C
ATOM   4149  C   LYS A 557       0.764   6.673  48.916  1.00139.79
C
ANISOU 4149  C   LYS A 557    20818  20278  12019   1050    340
-4835       C
ATOM   4150  O   LYS A 557       1.817   6.030  49.015  1.00139.42
O
ANISOU 4150  O   LYS A 557    20593  20307  12073    673    143
-4856       O
ATOM   4151  CB  LYS A 557       0.943   9.163  49.157  1.00135.51
C
ANISOU 4151  CB  LYS A 557    21394  19044  11049   1206    198
-5139       C
ATOM   4152  CG  LYS A 557       1.747  10.337  48.605  1.00148.42
C
ANISOU 4152  CG  LYS A 557    23468  20271  12654    921    -38
-5299       C
ATOM   4153  CD  LYS A 557       1.092  10.969  47.388  1.00133.16
C
ANISOU 4153  CD  LYS A 557    21520  18217  10856   1065     85
-5238       C
ATOM   4154  CE  LYS A 557       1.786  12.264  47.005  1.00151.05
C
ANISOU 4154  CE  LYS A 557    24337  20031  13025    841   -118
-5404       C
ATOM   4155  NZ  LYS A 557       3.206  12.037  46.616  1.00163.87
N
ANISOU 4155  NZ  LYS A 557    25803  21608  14854    233   -415
-5443       N
ATOM   4156  N   ALA A 558      -0.379   6.246  49.451  1.00129.07
N
ANISOU 4156  N   ALA A 558    19323  19141  10577   1463    616
-4710       N
ATOM   4157  CA  ALA A 558      -0.425   5.049  50.286  1.00126.36
C
ANISOU 4157  CA  ALA A 558    18683  19104  10223   1484    696
-4615       C
ATOM   4158  C   ALA A 558       0.110   3.808  49.579  1.00101.26
C
ANISOU 4158  C   ALA A 558    14929  16165   7380   1114    608
-4492       C
ATOM   4159  O   ALA A 558      -0.129   3.594  48.388  1.00 99.05
O
ANISOU 4159  O   ALA A 558    14319  15943   7374   1010    626
-4387       O
ATOM   4160  CB  ALA A 558      -1.860   4.794  50.748  1.00106.52
C
ANISOU 4160  CB  ALA A 558    16040  16809   7623   1980   1042
-4452       C
ATOM   4161  N   ASP A 559       0.845   2.989  50.336  1.00100.63
N
ANISOU 4161  N   ASP A 559    14764  16218   7254    933    510
```

```
-4507       N
ATOM   4162  CA  ASP A 559       1.504   1.795  49.824  1.00 97.33
C
ANISOU 4162  CA  ASP A 559    13875  16009   7098    594    413
-4405       C
ATOM   4163  C   ASP A 559       0.656   0.542  50.006  1.00100.69
C
ANISOU 4163  C   ASP A 559    13871  16767   7620    754    643
-4199       C
ATOM   4164  O   ASP A 559       1.144  -0.565  49.753  1.00 93.56
O
ANISOU 4164  O   ASP A 559    12619  16043   6888    515    585
-4107       O
ATOM   4165  CB  ASP A 559       2.858   1.598  50.513  1.00 97.58
C
ANISOU 4165  CB  ASP A 559    14049  16005   7024    298    159
-4527       C
ATOM   4166  CG  ASP A 559       3.844   2.704  50.197  1.00122.64
C
ANISOU 4166  CG  ASP A 559    17565  18871  10161     30   -109
-4702       C
ATOM   4167  OD1 ASP A 559       4.884   2.784  50.886  1.00 99.92
O
ANISOU 4167  OD1 ASP A 559    14879  15935   7152   -185   -337
-4817       O
ATOM   4168  OD2 ASP A 559       3.580   3.493  49.264  1.00134.80
O
ANISOU 4168  OD2 ASP A 559    19183  20231  11804     27   -100
-4716       O
ATOM   4169  N   GLY A 560      -0.587   0.692  50.436  1.00 99.56
N
ANISOU 4169  N   GLY A 560    13753  16706   7368   1151    904
-4113       N
ATOM   4170  CA  GLY A 560      -1.446  -0.434  50.738  1.00 97.20
C
ANISOU 4170  CA  GLY A 560    13070  16719   7141   1306   1133
-3904       C
ATOM   4171  C   GLY A 560      -2.584  -0.027  51.644  1.00100.38
C
ANISOU 4171  C   GLY A 560    13650  17179   7312   1780   1410
-3849       C
ATOM   4172  O   GLY A 560      -2.559   1.014  52.300  1.00103.17
O
ANISOU 4172  O   GLY A 560    14491  17325   7385   2001   1411
-3997       O
ATOM   4173  N   PHE A 561      -3.626  -0.856  51.650  1.00107.19
N
ANISOU 4173  N   PHE A 561    14113  18323   8290   1944   1652
-3618       N
ATOM   4174  CA  PHE A 561      -4.847  -0.566  52.392  1.00103.34
C
ANISOU 4174  CA  PHE A 561    13689  17950   7626   2418   1963
-3501       C
ATOM   4175  C   PHE A 561      -5.248  -1.766  53.237  1.00110.86
```

C
ANISOU 4175 C   PHE A 561   14373 19191   8560   2488   2156
-3322   C
ATOM   4176 O   PHE A 561   -5.342 -2.886 52.723 1.00101.27
O
ANISOU 4176 O   PHE A 561   12700 18175   7602   2249   2153
-3163   O
ATOM   4177 CB PHE A 561   -5.968 -0.156 51.437 1.00103.75
C
ANISOU 4177 CB PHE A 561   13497 18071   7854   2605   2103
-3355   C
ATOM   4178 CG PHE A 561   -5.635   1.060 50.629 1.00103.80
C
ANISOU 4178 CG PHE A 561   13804 17781   7856   2573   1939
-3524   C
ATOM   4179 CD1 PHE A 561   -5.320   0.954 49.287 1.00123.92
C
ANISOU 4179 CD1 PHE A 561   16110 20280 10694   2258   1759
-3519   C
ATOM   4180 CD2 PHE A 561   -5.615   2.312 51.222 1.00106.82
C
ANISOU 4180 CD2 PHE A 561   14751 17910   7924   2861   1964
-3687   C
ATOM   4181 CE1 PHE A 561   -4.999   2.074 48.548 1.00126.74
C
ANISOU 4181 CE1 PHE A 561   16759 20354 11042   2227   1620
-3667   C
ATOM   4182 CE2 PHE A 561   -5.300   3.435 50.488 1.00107.06
C
ANISOU 4182 CE2 PHE A 561   15092 17645   7942   2819   1815
-3840   C
ATOM   4183 CZ PHE A 561   -4.991   3.316 49.150 1.00104.22
C
ANISOU 4183 CZ PHE A 561   14463 17251   7886   2499   1648
-3826   C
ATOM   4184 N   ALA A 562   -5.479 -1.533 54.527 1.00113.85
N
ANISOU 4184 N   ALA A 562   15067 19572   8619   2819   2325
-3345   N
ATOM   4185 CA ALA A 562   -5.864 -2.582 55.458 1.00111.68
C
ANISOU 4185 CA ALA A 562   14604 19550   8279   2930   2537
-3176   C
ATOM   4186 C   ALA A 562   -7.343 -2.473 55.809 1.00136.34
C
ANISOU 4186 C   ALA A 562   17569 22881 11353   3383   2918
-2939   C
ATOM   4187 O   ALA A 562   -7.972 -1.424 55.639 1.00154.44
O
ANISOU 4187 O   ALA A 562   20040 25087 13554   3698   3023
-2950   O
ATOM   4188 CB ALA A 562   -5.023 -2.516 56.735 1.00118.90
C
ANISOU 4188 CB ALA A 562   15981 20342   8853   2984   2461

```
-3351       C
ATOM   4189  N   GLY A 563      -7.889  -3.578  56.314  1.00110.57
N
ANISOU 4189  N   GLY A 563    13967  19899   8146   3420   3133
-2711       N
ATOM   4190  CA  GLY A 563      -9.284  -3.616  56.726  1.00113.67
C
ANISOU 4190  CA  GLY A 563    14143  20540   8507   3831   3518
-2441       C
ATOM   4191  C   GLY A 563     -10.244  -3.355  55.589  1.00136.25
C
ANISOU 4191  C   GLY A 563    16600  23523  11644   3866   3580
-2270       C
ATOM   4192  O   GLY A 563     -11.345  -2.842  55.812  1.00143.48
O
ANISOU 4192  O   GLY A 563    17459  24573  12483   4292   3864
-2103       O
ATOM   4193  N   VAL A 564      -9.847  -3.703  54.368  1.00150.41
N
ANISOU 4193  N   VAL A 564    18117  25282  13751   3444   3321
-2299       N
ATOM   4194  CA  VAL A 564     -10.615  -3.403  53.164  1.00112.77
C
ANISOU 4194  CA  VAL A 564    13010  20592   9247   3436   3310
-2175       C
ATOM   4195  C   VAL A 564     -11.886  -4.248  53.136  1.00111.42
C
ANISOU 4195  C   VAL A 564    12266  20797   9272   3519   3567
-1813       C
ATOM   4196  O   VAL A 564     -11.826  -5.474  53.018  1.00115.26
O
ANISOU 4196  O   VAL A 564    12406  21436   9950   3196   3530
-1679       O
ATOM   4197  CB  VAL A 564      -9.777  -3.624  51.901  1.00119.35
C
ANISOU 4197  CB  VAL A 564    13735  21273  10339   2956   2959
-2305       C
ATOM   4198  CG1 VAL A 564     -10.600  -3.312  50.664  1.00175.12
C
ANISOU 4198  CG1 VAL A 564    20472  28412  17656   2967   2941
-2176       C
ATOM   4199  CG2 VAL A 564      -8.523  -2.764  51.947  1.00122.49
C
ANISOU 4199  CG2 VAL A 564    14668  21317  10555   2857   2713
-2636       C
ATOM   4200  N   PHE A 565     -13.031  -3.596  53.249  1.00114.80
N
ANISOU 4200  N   PHE A 565    12598  21375   9646   3950   3827
-1643       N
ATOM   4201  CA  PHE A 565     -14.315  -4.224  52.976  1.00129.28
C
ANISOU 4201  CA  PHE A 565    13824  23580  11716   4011   4036
-1278       C
ATOM   4202  C   PHE A 565     -14.643  -4.052  51.501  1.00151.09
```

```
C
ANISOU 4202  C   PHE A 565    16267  26357  14785   3813   3835
-1235       C
ATOM   4203  O   PHE A 565     -13.980  -3.290  50.793  1.00115.35
O
ANISOU 4203  O   PHE A 565    12031  21551  10245   3727   3598
-1479       O
ATOM   4204  CB  PHE A 565     -15.394  -3.617  53.873  1.00143.09
C
ANISOU 4204  CB  PHE A 565    15607  25511  13248   4607   4433
-1085       C
ATOM   4205  CG  PHE A 565     -15.353  -4.113  55.293  1.00139.35
C
ANISOU 4205  CG  PHE A 565    15335  24989  12622   4703   4602
-1001       C
ATOM   4206  CD1 PHE A 565     -14.795  -3.343  56.301  1.00124.67
C
ANISOU 4206  CD1 PHE A 565    14121  22827  10420   4967   4623
-1209       C
ATOM   4207  CD2 PHE A 565     -15.875  -5.356  55.617  1.00147.39
C
ANISOU 4207  CD2 PHE A 565    15915  26247  13840   4515   4725
-712       C
ATOM   4208  CE1 PHE A 565     -14.762  -3.805  57.607  1.00129.82
C
ANISOU 4208  CE1 PHE A 565    14963  23435  10927   5064   4772
-1133       C
ATOM   4209  CE2 PHE A 565     -15.845  -5.823  56.919  1.00150.97
C
ANISOU 4209  CE2 PHE A 565    16563  26651  14147   4614   4890
-634       C
ATOM   4210  CZ  PHE A 565     -15.287  -5.047  57.915  1.00143.13
C
ANISOU 4210  CZ  PHE A 565    16201  25374  12808   4900   4916
-847       C
ATOM   4211  N   PRO A 566     -15.659  -4.750  50.983  1.00171.69
N
ANISOU 4211  N   PRO A 566    18277  29281  17676   3722   3910
-922       N
ATOM   4212  CA  PRO A 566     -15.839  -4.743  49.524  1.00153.75
C
ANISOU 4212  CA  PRO A 566    15717  27001  15701   3459   3657
-899       C
ATOM   4213  C   PRO A 566     -16.296  -3.398  48.991  1.00138.48
C
ANISOU 4213  C   PRO A 566    13925  24992  13698   3826   3678
-953       C
ATOM   4214  O   PRO A 566     -15.901  -3.018  47.882  1.00113.61
O
ANISOU 4214  O   PRO A 566    10843  21652  10670   3636   3411
-1101       O
ATOM   4215  CB  PRO A 566     -16.902  -5.826  49.303  1.00138.86
C
ANISOU 4215  CB  PRO A 566    13170  25492  14097   3300   3752
```

-526 C
ATOM 4216 CG PRO A 566 -16.661 -6.785 50.408 1.00146.23
C
ANISOU 4216 CG PRO A 566 14111 26511 14938 3205 3907
-453 C
ATOM 4217 CD PRO A 566 -16.344 -5.904 51.595 1.00154.19
C
ANISOU 4217 CD PRO A 566 15634 27391 15561 3656 4125
-612 C
ATOM 4218 N GLU A 567 -17.119 -2.670 49.746 1.00128.40
N
ANISOU 4218 N GLU A 567 12714 23857 12215 4369 4001
-827 N
ATOM 4219 CA GLU A 567 -17.460 -1.301 49.373 1.00137.26
C
ANISOU 4219 CA GLU A 567 14088 24865 13202 4779 4041
-905 C
ATOM 4220 C GLU A 567 -16.216 -0.421 49.301 1.00122.13
C
ANISOU 4220 C GLU A 567 12850 22484 11070 4724 3828
-1307 C
ATOM 4221 O GLU A 567 -16.074 0.399 48.386 1.00118.76
O
ANISOU 4221 O GLU A 567 12586 21863 10674 4741 3662
-1440 O
ATOM 4222 CB GLU A 567 -18.462 -0.727 50.376 1.00131.59
C
ANISOU 4222 CB GLU A 567 13395 24363 12241 5410 4458
-704 C
ATOM 4223 CG GLU A 567 -19.791 -1.462 50.422 1.00130.40
C
ANISOU 4223 CG GLU A 567 12577 24613 12354 5444 4636
-259 C
ATOM 4224 CD GLU A 567 -20.821 -0.746 51.276 1.00134.99
C
ANISOU 4224 CD GLU A 567 13275 25218 12798 5979 4922
-32 C
ATOM 4225 OE1 GLU A 567 -20.556 -0.542 52.480 1.00136.53
O
ANISOU 4225 OE1 GLU A 567 13908 25234 12734 6187 5075
-96 O
ATOM 4226 OE2 GLU A 567 -21.895 -0.389 50.748 1.00151.65
O
ANISOU 4226 OE2 GLU A 567 15042 27527 15051 6194 4985
215 O
ATOM 4227 N HIS A 568 -15.307 -0.579 50.266 1.00129.62
N
ANISOU 4227 N HIS A 568 14199 23252 11798 4652 3824
-1495 N
ATOM 4228 CA HIS A 568 -14.036 0.140 50.244 1.00123.92
C
ANISOU 4228 CA HIS A 568 14088 22102 10892 4519 3587
-1865 C
ATOM 4229 C HIS A 568 -13.194 -0.221 49.025 1.00126.77

C
ANISOU 4229 C   HIS A 568   14341 22301 11524   3981   3220
-2003   C
ATOM   4230 O   HIS A 568   -12.609   0.660 48.384 1.00149.77
O
ANISOU 4230 O   HIS A 568   17592 24917 14395   3933   3031
-2220   O
ATOM   4231 CB HIS A 568   -13.240 -0.151 51.520 1.00116.63
C
ANISOU 4231 CB HIS A 568   13534 21070   9710   4497   3627
-2006   C
ATOM   4232 CG HIS A 568   -14.000   0.076 52.792 1.00135.52
C
ANISOU 4232 CG HIS A 568   16059 23617 11817   5020   3999
-1864   C
ATOM   4233 ND1 HIS A 568   -13.417 -0.053 54.033 1.00166.93
N
ANISOU 4233 ND1 HIS A 568   20427 27494 15505   5100   4068
-1982   N
ATOM   4234 CD2 HIS A 568   -15.289   0.427 53.019 1.00144.82
C
ANISOU 4234 CD2 HIS A 568   17037 25042 12946   5505   4325
-1603   C
ATOM   4235 CE1 HIS A 568   -14.313   0.203 54.969 1.00165.12
C
ANISOU 4235 CE1 HIS A 568   20270 27313 15156   5518   4353
-1772   C
ATOM   4236 NE2 HIS A 568   -15.457   0.497 54.380 1.00161.94
N
ANISOU 4236 NE2 HIS A 568   19509 27109 14912   5756   4506
-1531   N
ATOM   4237 N   LYS A 569   -13.120 -1.511 48.691 1.00116.75
N
ANISOU 4237 N   LYS A 569   12629 21211 10520   3581   3124
-1873   N
ATOM   4238 CA LYS A 569   -12.287 -1.957 47.576 1.00131.83
C
ANISOU 4238 CA LYS A 569   14455 22968 12664   3086   2792
-1990   C
ATOM   4239 C   LYS A 569   -12.704 -1.324 46.252 1.00151.43
C
ANISOU 4239 C   LYS A 569   16824 25397 15317   3104   2668
-1976   C
ATOM   4240 O   LYS A 569   -11.854 -0.876 45.474 1.00165.17
O
ANISOU 4240 O   LYS A 569   18812 26857 17089   2895   2428
-2184   O
ATOM   4241 CB LYS A 569   -12.341 -3.483 47.478 1.00136.24
C
ANISOU 4241 CB LYS A 569   14562 23749 13456   2718   2749
-1811   C
ATOM   4242 CG LYS A 569   -11.318 -4.095 46.538 1.00134.14
C
ANISOU 4242 CG LYS A 569   14279 23312 13374   2221   2429

```
-1939       C
ATOM   4243  CD  LYS A 569     -11.125  -5.574  46.837  1.00148.35
C
ANISOU 4243  CD  LYS A 569    15812  25266  15287   1904   2414
-1813       C
ATOM   4244  CE  LYS A 569     -10.097  -6.200  45.911  1.00163.11
C
ANISOU 4244  CE  LYS A 569    17694  26966  17314   1452   2115
-1929       C
ATOM   4245  NZ  LYS A 569      -9.948  -7.660  46.160  1.00175.20
N
ANISOU 4245  NZ  LYS A 569    18995  28633  18940   1163   2104
-1797       N
ATOM   4246  N   TYR A 570     -14.007  -1.290  45.970  1.00127.76
N
ANISOU 4246  N   TYR A 570    13437  22674  12431   3353   2826
-1717       N
ATOM   4247  CA  TYR A 570     -14.499  -0.543  44.814  1.00110.65
C
ANISOU 4247  CA  TYR A 570    11200  20465  10377   3462   2733
-1699       C
ATOM   4248  C   TYR A 570     -14.112   0.933  44.875  1.00119.89
C
ANISOU 4248  C   TYR A 570    12951  21317  11285   3768   2743
-1936       C
ATOM   4249  O   TYR A 570     -13.716   1.521  43.863  1.00113.87
O
ANISOU 4249  O   TYR A 570    12356  20325  10586   3659   2543
-2076       O
ATOM   4250  CB  TYR A 570     -16.017  -0.715  44.704  1.00127.49
C
ANISOU 4250  CB  TYR A 570    12810  22991  12639   3735   2930
-1357       C
ATOM   4251  CG  TYR A 570     -16.708   0.202  43.713  1.00148.03
C
ANISOU 4251  CG  TYR A 570    15354  25594  15298   3985   2891
-1311       C
ATOM   4252  CD1 TYR A 570     -17.105   1.486  44.074  1.00161.24
C
ANISOU 4252  CD1 TYR A 570    17359  27192  16714   4518   3086
-1355       C
ATOM   4253  CD2 TYR A 570     -16.960  -0.219  42.413  1.00136.86
C
ANISOU 4253  CD2 TYR A 570    13585  24241  14173   3704   2655
-1223       C
ATOM   4254  CE1 TYR A 570     -17.733   2.322  43.170  1.00176.24
C
ANISOU 4254  CE1 TYR A 570    19221  29092  18651   4771   3056
-1307       C
ATOM   4255  CE2 TYR A 570     -17.589   0.611  41.501  1.00137.35
C
ANISOU 4255  CE2 TYR A 570    13600  24308  14279   3944   2610
-1179       C
ATOM   4256  CZ  TYR A 570     -17.973   1.881  41.885  1.00156.52
```

```
C
ANISOU 4256  CZ  TYR A 570    16344  26673  16453   4481   2815
-1219       C
ATOM   4257  OH  TYR A 570     -18.602   2.710  40.983  1.00144.09
O
ANISOU 4257  OH  TYR A 570    14738  25104  14906   4747   2778
-1170       O
ATOM   4258  N   GLU A 571     -14.269   1.563  46.039  1.00152.67
N
ANISOU 4258  N   GLU A 571    17434  25443  15130   4168   2981
-1973       N
ATOM   4259  CA  GLU A 571     -13.881   2.964  46.192  1.00147.73
C
ANISOU 4259  CA  GLU A 571    17433  24479  14219   4455   2986
-2206       C
ATOM   4260  C   GLU A 571     -12.382   3.191  45.995  1.00138.18
C
ANISOU 4260  C   GLU A 571    16668  22874  12958   4066   2701
-2527       C
ATOM   4261  O   GLU A 571     -11.976   4.172  45.361  1.00145.08
O
ANISOU 4261  O   GLU A 571    17901  23450  13772   4081   2569
-2702       O
ATOM   4262  CB  GLU A 571     -14.320   3.477  47.565  1.00160.52
C
ANISOU 4262  CB  GLU A 571    19355  26142  15493   4955   3296
-2176       C
ATOM   4263  CG  GLU A 571     -14.329   4.991  47.672  1.00160.11
C
ANISOU 4263  CG  GLU A 571    19912  25791  15132   5376   3359
-2343       C
ATOM   4264  CD  GLU A 571     -15.455   5.619  46.875  1.00153.52
C
ANISOU 4264  CD  GLU A 571    18864  25099  14367   5744   3475
-2165       C
ATOM   4265  OE1 GLU A 571     -15.169   6.477  46.013  1.00144.11
O
ANISOU 4265  OE1 GLU A 571    17953  23635  13167   5734   3316
-2317       O
ATOM   4266  OE2 GLU A 571     -16.626   5.253  47.109  1.00167.04
O
ANISOU 4266  OE2 GLU A 571    20119  27205  16144   6044   3726
-1860       O
ATOM   4267  N   ILE A 572     -11.546   2.301  46.535  1.00127.21
N
ANISOU 4267  N   ILE A 572    15258  21485  11591   3721   2608
-2594       N
ATOM   4268  CA  ILE A 572     -10.093   2.426  46.393  1.00115.31
C
ANISOU 4268  CA  ILE A 572    14110  19653  10051   3337   2337
-2867       C
ATOM   4269  C   ILE A 572      -9.645   2.421  44.932  1.00151.25
C
ANISOU 4269  C   ILE A 572    18534  24073  14861   2995   2083
```

```
-2918       C
ATOM   4270  O   ILE A 572      -8.876   3.291  44.506  1.00171.21
O
ANISOU 4270  O   ILE A 572    21459  26277  17318   2903   1924
-3126       O
ATOM   4271  CB  ILE A 572      -9.386   1.318  47.194  1.00104.30
C
ANISOU 4271  CB  ILE A 572    12623  18350   8656   3050   2295
-2880       C
ATOM   4272  CG1 ILE A 572      -9.579   1.534  48.696  1.00107.28
C
ANISOU 4272  CG1 ILE A 572    13290  18761   8710   3392   2515
-2894       C
ATOM   4273  CG2 ILE A 572      -7.913   1.253  46.835  1.00157.03
C
ANISOU 4273  CG2 ILE A 572    19524  24766  15376   2604   1995
-3106       C
ATOM   4274  CD1 ILE A 572      -9.372   0.285  49.520  1.00106.43
C
ANISOU 4274  CD1 ILE A 572    12955  18863   8621   3226   2571
-2803       C
ATOM   4275  N   VAL A 573     -10.109   1.452  44.142  1.00159.50
N
ANISOU 4275  N   VAL A 573    19049  25354  16201   2798   2038
-2727       N
ATOM   4276  CA  VAL A 573      -9.831   1.487  42.704  1.00133.91
C
ANISOU 4276  CA  VAL A 573    15702  21993  13186   2539   1818
-2755       C
ATOM   4277  C   VAL A 573     -10.430   2.727  42.044  1.00128.33
C
ANISOU 4277  C   VAL A 573    15179  21158  12421   2860   1854
-2775       C
ATOM   4278  O   VAL A 573      -9.778   3.381  41.221  1.00124.44
O
ANISOU 4278  O   VAL A 573    14952  20382  11948   2725   1685
-2931       O
ATOM   4279  CB  VAL A 573     -10.292   0.183  42.021  1.00138.49
C
ANISOU 4279  CB  VAL A 573    15714  22841  14065   2283   1754
-2538       C
ATOM   4280  CG1 VAL A 573     -11.742  -0.127  42.317  1.00153.06
C
ANISOU 4280  CG1 VAL A 573    17153  25041  15963   2574   1970
-2265       C
ATOM   4281  CG2 VAL A 573     -10.060   0.259  40.517  1.00141.47
C
ANISOU 4281  CG2 VAL A 573    16024  23082  14647   2061   1530
-2565       C
ATOM   4282  N   LYS A 574     -11.669   3.076  42.393  1.00129.13
N
ANISOU 4282  N   LYS A 574    15149  21469  12447   3301   2086
-2607       N
ATOM   4283  CA  LYS A 574     -12.306   4.254  41.808  1.00133.80
```

```
C
ANISOU 4283  CA  LYS A 574    15918  21959  12961   3662   2141
-2609       C
ATOM   4284  C   LYS A 574     -11.524   5.539  42.079  1.00143.73
C
ANISOU 4284  C   LYS A 574    17866  22803  13941   3779   2109
-2880       C
ATOM   4285  O   LYS A 574     -11.331   6.356  41.172  1.00150.22
O
ANISOU 4285  O   LYS A 574    18923  23383  14771   3786   1997
-2980       O
ATOM   4286  CB  LYS A 574     -13.738   4.374  42.333  1.00137.23
C
ANISOU 4286  CB  LYS A 574    16091  22724  13327   4157   2430
-2362       C
ATOM   4287  CG  LYS A 574     -14.492   5.608  41.859  1.00136.40
C
ANISOU 4287  CG  LYS A 574    16175  22549  13103   4618   2527
-2339       C
ATOM   4288  CD  LYS A 574     -15.974   5.495  42.192  1.00142.07
C
ANISOU 4288  CD  LYS A 574    16468  23683  13829   5065   2798
-2027       C
ATOM   4289  CE  LYS A 574     -16.779   6.646  41.606  1.00146.23
C
ANISOU 4289  CE  LYS A 574    17123  24182  14257   5539   2886
-1974       C
ATOM   4290  NZ  LYS A 574     -16.370   7.960  42.175  1.00145.56
N
ANISOU 4290  NZ  LYS A 574    17780  23729  13796   5883   2990
-2198       N
ATOM   4291  N   LYS A 575     -11.071   5.743  43.320  1.00175.89
N
ANISOU 4291  N   LYS A 575    22297  26778  17757   3866   2198
-2999       N
ATOM   4292  CA  LYS A 575     -10.220   6.897  43.614  1.00161.29
C
ANISOU 4292  CA  LYS A 575    21133  24508  15643   3899   2120
-3269       C
ATOM   4293  C   LYS A 575      -8.906   6.876  42.834  1.00158.78
C
ANISOU 4293  C   LYS A 575    20957  23911  15463   3382   1819
-3455       C
ATOM   4294  O   LYS A 575      -8.478   7.909  42.305  1.00178.41
O
ANISOU 4294  O   LYS A 575    23856  26065  17865   3379   1719
-3609       O
ATOM   4295  CB  LYS A 575      -9.942   6.970  45.117  1.00125.42
C
ANISOU 4295  CB  LYS A 575    16927  19925  10802   4046   2237
-3356       C
ATOM   4296  CG  LYS A 575     -11.163   7.298  45.962  1.00116.02
C
ANISOU 4296  CG  LYS A 575    15757  18934   9392   4641   2568
```

```
-3198       C
ATOM   4297  CD  LYS A 575     -11.946   8.458  45.367  1.00117.89
C
ANISOU 4297  CD  LYS A 575    16202  19065   9526   5070   2674
-3168       C
ATOM   4298  CE  LYS A 575     -12.771   9.170  46.426  1.00124.42
C
ANISOU 4298  CE  LYS A 575    17352  19926   9994   5699   2986
-3107       C
ATOM   4299  NZ  LYS A 575     -13.470  10.366  45.878  1.00140.08
N
ANISOU 4299  NZ  LYS A 575    19608  21777  11838   6152   3091
-3090       N
ATOM   4300  N   LEU A 576      -8.248   5.717  42.755  1.00153.70
N
ANISOU 4300  N   LEU A 576    19985  23394  15022   2954   1683
-3431       N
ATOM   4301  CA  LEU A 576      -7.028   5.598  41.956  1.00157.84
C
ANISOU 4301  CA  LEU A 576    20575  23700  15696   2481   1420
-3566       C
ATOM   4302  C   LEU A 576      -7.288   5.809  40.468  1.00174.07
C
ANISOU 4302  C   LEU A 576    22475  25701  17963   2424   1331
-3508       C
ATOM   4303  O   LEU A 576      -6.518   6.495  39.786  1.00166.36
O
ANISOU 4303  O   LEU A 576    21797  24421  16992   2248   1182
-3650       O
ATOM   4304  CB  LEU A 576      -6.372   4.238  42.194  1.00154.79
C
ANISOU 4304  CB  LEU A 576    19850  23495  15467   2097   1324
-3520       C
ATOM   4305  CG  LEU A 576      -5.691   4.055  43.550  1.00153.11
C
ANISOU 4305  CG  LEU A 576    19867  23261  15048   2041   1332
-3630       C
ATOM   4306  CD1 LEU A 576      -5.276   2.608  43.743  1.00150.09
C
ANISOU 4306  CD1 LEU A 576    19090  23111  14826   1734   1277
-3538       C
ATOM   4307  CD2 LEU A 576      -4.488   4.982  43.677  1.00152.25
C
ANISOU 4307  CD2 LEU A 576    20280  22782  14785   1854   1150
-3876       C
ATOM   4308  N   GLN A 577      -8.367   5.222  39.952  1.00190.09
N
ANISOU 4308  N   GLN A 577    24039  28021  20165   2563   1413
-3292       N
ATOM   4309  CA  GLN A 577      -8.755   5.412  38.557  1.00159.78
C
ANISOU 4309  CA  GLN A 577    20048  24153  16510   2557   1326
-3222       C
ATOM   4310  C   GLN A 577      -9.084   6.867  38.240  1.00136.33
```

```
C
ANISOU 4310  C   GLN A 577    17500  20935  13365   2899   1385
-3309       C
ATOM   4311  O   GLN A 577      -8.696   7.383  37.185  1.00147.05
O
ANISOU 4311  O   GLN A 577    19020  22061  14792   2783   1253
-3382       O
ATOM   4312  CB  GLN A 577      -9.949   4.510  38.243  1.00140.15
C
ANISOU 4312  CB  GLN A 577    16982  22053  14216   2661   1398
-2961       C
ATOM   4313  CG  GLN A 577     -10.379   4.475  36.795  1.00137.37
C
ANISOU 4313  CG  GLN A 577    16410  21713  14071   2615   1272
-2868       C
ATOM   4314  CD  GLN A 577     -11.448   3.429  36.553  1.00135.66
C
ANISOU 4314  CD  GLN A 577    15601  21885  14059   2624   1294
-2606       C
ATOM   4315  OE1 GLN A 577     -12.631   3.749  36.439  1.00124.83
O
ANISOU 4315  OE1 GLN A 577    14031  20711  12688   2970   1407
-2448       O
ATOM   4316  NE2 GLN A 577     -11.038   2.167  36.493  1.00130.79
N
ANISOU 4316  NE2 GLN A 577    14701  21384  13610   2244   1183
-2550       N
ATOM   4317  N   GLU A 578      -9.797   7.545  39.139  1.00133.38
N
ANISOU 4317  N   GLU A 578    17332  20597  12749   3340   1593
-3295       N
ATOM   4318  CA  GLU A 578      -9.978   8.990  39.020  1.00134.84
C
ANISOU 4318  CA  GLU A 578    18036  20493  12703   3680   1658
-3406       C
ATOM   4319  C   GLU A 578      -8.654   9.748  39.047  1.00130.06
C
ANISOU 4319  C   GLU A 578    18005  19444  11969   3408   1498
-3669       C
ATOM   4320  O   GLU A 578      -8.501  10.755  38.345  1.00142.18
O
ANISOU 4320  O   GLU A 578    19903  20681  13438   3475   1447
-3763       O
ATOM   4321  CB  GLU A 578     -10.911   9.496  40.121  1.00135.77
C
ANISOU 4321  CB  GLU A 578    18307  20731  12549   4218   1927
-3340       C
ATOM   4322  CG  GLU A 578     -12.368   9.120  39.898  1.00139.62
C
ANISOU 4322  CG  GLU A 578    18273  21628  13147   4578   2104
-3058       C
ATOM   4323  CD  GLU A 578     -13.305   9.768  40.896  1.00167.95
C
ANISOU 4323  CD  GLU A 578    22046  25323  16445   5177   2401
```

```
-2975       C
ATOM   4324  OE1 GLU A 578     -13.967   9.031  41.657  1.00172.09
O
ANISOU 4324  OE1 GLU A 578    22193  26204  16990   5318   2576
-2788       O
ATOM   4325  OE2 GLU A 578     -13.384  11.014  40.917  1.00184.09
O
ANISOU 4325  OE2 GLU A 578    24628  27087  18229   5517   2472
-3086       O
ATOM   4326  N   ARG A 579      -7.684   9.293  39.841  1.00143.40
N
ANISOU 4326  N   ARG A 579    19783  21085  13619   3092   1412
-3780       N
ATOM   4327  CA  ARG A 579      -6.341   9.864  39.757  1.00142.80
C
ANISOU 4327  CA  ARG A 579    20151  20628  13479   2740   1218
-4000       C
ATOM   4328  C   ARG A 579      -5.543   9.414  38.532  1.00151.18
C
ANISOU 4328  C   ARG A 579    20991  21625  14825   2293   1016
-3997       C
ATOM   4329  O   ARG A 579      -4.316   9.292  38.602  1.00165.04
O
ANISOU 4329  O   ARG A 579    22871  23222  16613   1889    850
-4115       O
ATOM   4330  CB  ARG A 579      -5.561   9.524  41.032  1.00164.68
C
ANISOU 4330  CB  ARG A 579    23075  23389  16107   2559   1175
-4108       C
ATOM   4331  CG  ARG A 579      -6.033  10.281  42.274  1.00179.55
C
ANISOU 4331  CG  ARG A 579    25405  25188  17630   2975   1338
-4179       C
ATOM   4332  CD  ARG A 579      -5.710  11.770  42.234  1.00173.68
C
ANISOU 4332  CD  ARG A 579    25367  23993  16632   3089   1290
-4367       C
ATOM   4333  NE  ARG A 579      -4.274  12.030  42.245  1.00172.08
N
ANISOU 4333  NE  ARG A 579    25472  23486  16425   2600   1033
-4558       N
ATOM   4334  CZ  ARG A 579      -3.706  13.056  42.871  1.00142.57
C
ANISOU 4334  CZ  ARG A 579    22393  19377  12400   2592    950
-4754       C
ATOM   4335  NH1 ARG A 579      -4.460  13.988  43.440  1.00116.12
N
ANISOU 4335  NH1 ARG A 579    19519  15872   8727   3077   1113
-4797       N
ATOM   4336  NH2 ARG A 579      -2.389  13.205  42.842  1.00123.40
N
ANISOU 4336  NH2 ARG A 579    20163  16715  10008   2100    697
-4899       N
ATOM   4337  N   LYS A 580      -6.237   9.140  37.421  1.00156.32
```

```
N
ANISOU 4337  N   LYS A 580    21307  22413  15675   2373   1029
-3852       N
ATOM   4338  CA  LYS A 580      -5.669   8.883  36.095  1.00145.53
C
ANISOU 4338  CA  LYS A 580    19795  20953  14545   2052    865
-3836       C
ATOM   4339  C   LYS A 580      -4.877   7.581  35.971  1.00118.67
C
ANISOU 4339  C   LYS A 580    16018  17714  11358   1616    737
-3790       C
ATOM   4340  O   LYS A 580      -4.650   7.102  34.854  1.00115.10
O
ANISOU 4340  O   LYS A 580    15347  17271  11116   1411    632
-3722       O
ATOM   4341  CB  LYS A 580      -4.794  10.062  35.644  1.00160.59
C
ANISOU 4341  CB  LYS A 580    22236  22426  16353   1924    769
-4010       C
ATOM   4342  CG  LYS A 580      -3.288   9.837  35.723  1.00181.60
C
ANISOU 4342  CG  LYS A 580    24999  24926  19077   1425    595
-4122       C
ATOM   4343  CD  LYS A 580      -2.529  11.099  35.336  1.00177.60
C
ANISOU 4343  CD  LYS A 580    25031  23987  18463   1309    514
-4273       C
ATOM   4344  CE  LYS A 580      -1.025  10.885  35.382  1.00167.16
C
ANISOU 4344  CE  LYS A 580    23757  22535  17221    798    338
-4354       C
ATOM   4345  NZ  LYS A 580      -0.286  12.119  34.994  1.00166.52
N
ANISOU 4345  NZ  LYS A 580    24187  22033  17051    647    255
-4482       N
ATOM   4346  N   HIS A 581      -4.447   7.004  37.090  1.00151.92
N
ANISOU 4346  N   HIS A 581    20178  22042  15502   1493    745
-3822       N
ATOM   4347  CA  HIS A 581      -3.706   5.749  37.043  1.00137.23
C
ANISOU 4347  CA  HIS A 581    17982  20342  13819   1117    637
-3771       C
ATOM   4348  C   HIS A 581      -4.533   4.632  36.417  1.00 92.63
C
ANISOU 4348  C   HIS A 581    11817  14995   8383   1138    661
-3568       C
ATOM   4349  O   HIS A 581      -5.749   4.548  36.611  1.00107.45
O
ANISOU 4349  O   HIS A 581    13503  17077  10247   1447    794
-3447       O
ATOM   4350  CB  HIS A 581      -3.265   5.333  38.449  1.00159.13
C
ANISOU 4350  CB  HIS A 581    20796  23214  16454   1051    661
```

```
-3829       C
ATOM   4351  CG  HIS A 581      -2.210   6.213  39.045  1.00125.13
C
ANISOU 4351  CG  HIS A 581    16965  18616  11964    909    567
-4028       C
ATOM   4352  ND1 HIS A 581      -2.431   7.535  39.362  1.00122.68
N
ANISOU 4352  ND1 HIS A 581    17140  18045  11426   1147    613
-4152       N
ATOM   4353  CD2 HIS A 581      -0.929   5.951  39.395  1.00117.75
C
ANISOU 4353  CD2 HIS A 581    16098  17609  11032    547    415
-4118       C
ATOM   4354  CE1 HIS A 581      -1.329   8.052  39.876  1.00121.90
C
ANISOU 4354  CE1 HIS A 581    17401  17711  11203    908    478
-4316       C
ATOM   4355  NE2 HIS A 581      -0.402   7.112  39.908  1.00119.06
N
ANISOU 4355  NE2 HIS A 581    16774  17477  10988    540    353
-4293       N
ATOM   4356  N   ILE A 582      -3.862   3.768  35.660  1.00 86.17
N
ANISOU 4356  N   ILE A 582    10781  14203   7758    806    529
-3521       N
ATOM   4357  CA  ILE A 582      -4.485   2.562  35.125  1.00 84.67
C
ANISOU 4357  CA  ILE A 582    10139  14274   7759    754    513
-3339       C
ATOM   4358  C   ILE A 582      -4.284   1.478  36.174  1.00114.60
C
ANISOU 4358  C   ILE A 582    13717  18289  11536    629    549
-3293       C
ATOM   4359  O   ILE A 582      -3.149   1.079  36.459  1.00101.76
O
ANISOU 4359  O   ILE A 582    12154  16606   9904    358    467
-3364       O
ATOM   4360  CB  ILE A 582      -3.879   2.151  33.773  1.00 82.33
C
ANISOU 4360  CB  ILE A 582     9770  13871   7643    492    360
-3307       C
ATOM   4361  CG1 ILE A 582      -4.155   3.199  32.685  1.00114.62
C
ANISOU 4361  CG1 ILE A 582    14069  17739  11741    637    334
-3338       C
ATOM   4362  CG2 ILE A 582      -4.418   0.796  33.341  1.00 80.93
C
ANISOU 4362  CG2 ILE A 582     9175  13939   7635    397    318
-3130       C
ATOM   4363  CD1 ILE A 582      -3.198   4.383  32.669  1.00129.56
C
ANISOU 4363  CD1 ILE A 582    16410  19300  13518    573    310
-3510       C
ATOM   4364  N   VAL A 583      -5.383   0.992  36.741  1.00149.65
```

```
N
ANISOU 4364  N   VAL A 583    17894  22994  15973    831    675
-3159       N
ATOM   4365  CA  VAL A 583      -5.354   0.216  37.975  1.00125.45
C
ANISOU 4365  CA  VAL A 583    14703  20127  12835    807    762
-3124       C
ATOM   4366  C   VAL A 583      -5.609  -1.253  37.674  1.00108.53
C
ANISOU 4366  C   VAL A 583    12147  18215  10875    609    721
-2950       C
ATOM   4367  O   VAL A 583      -6.704  -1.625  37.235  1.00 84.41
O
ANISOU 4367  O   VAL A 583     8797  15338   7937    712    755
-2783       O
ATOM   4368  CB  VAL A 583      -6.384   0.741  38.985  1.00147.05
C
ANISOU 4368  CB  VAL A 583    17473  22994  15406   1195    970
-3085       C
ATOM   4369  CG1 VAL A 583      -6.219   0.039  40.322  1.00178.03
C
ANISOU 4369  CG1 VAL A 583    21342  27082  19220   1177   1067
-3069       C
ATOM   4370  CG2 VAL A 583      -6.260   2.250  39.136  1.00171.98
C
ANISOU 4370  CG2 VAL A 583    21089  25888  18367   1425   1005
-3249       C
ATOM   4371  N   GLY A 584      -4.602  -2.085  37.927  1.00109.41
N
ANISOU 4371  N   GLY A 584    12245  18324  11001    326    642
-2982       N
ATOM   4372  CA  GLY A 584      -4.826  -3.515  38.012  1.00107.89
C
ANISOU 4372  CA  GLY A 584    11727  18349  10918    168    638
-2827       C
ATOM   4373  C   GLY A 584      -5.281  -3.850  39.423  1.00122.63
C
ANISOU 4373  C   GLY A 584    13517  20417  12659    313    809
-2777       C
ATOM   4374  O   GLY A 584      -4.783  -3.300  40.405  1.00150.01
O
ANISOU 4374  O   GLY A 584    17237  23819  15940    402    868
-2908       O
ATOM   4375  N   MET A 585      -6.241  -4.767  39.524  1.00148.91
N
ANISOU 4375  N   MET A 585    16505  23989  16085    328    882
-2580       N
ATOM   4376  CA  MET A 585      -6.719  -5.163  40.843  1.00138.86
C
ANISOU 4376  CA  MET A 585    15139  22922  14700    468   1069
-2502       C
ATOM   4377  C   MET A 585      -7.080  -6.639  40.877  1.00124.49
C
ANISOU 4377  C   MET A 585    12986  21308  13008    273   1072
```

```
-2309       C
ATOM   4378  O   MET A 585      -7.871  -7.114  40.055  1.00100.86
O
ANISOU 4378  O   MET A 585     9717  18413  10193    202   1018
-2149       O
ATOM   4379  CB  MET A 585      -7.928  -4.317  41.258  1.00105.19
C
ANISOU 4379  CB  MET A 585    10830  18772  10364    851   1257
-2431       C
ATOM   4380  CG  MET A 585      -8.577  -4.767  42.555  1.00114.74
C
ANISOU 4380  CG  MET A 585    11904  20222  11468   1028   1483
-2305       C
ATOM   4381  SD  MET A 585      -9.718  -3.538  43.211  1.00142.46
S
ANISOU 4381  SD  MET A 585    15492  23822  14814   1551   1737
-2260       S
ATOM   4382  CE  MET A 585      -8.654  -2.662  44.355  1.00164.83
C
ANISOU 4382  CE  MET A 585    18891  26415  17323   1680   1772
-2531       C
ATOM   4383  N   THR A 586      -6.489  -7.350  41.831  1.00 83.44
N
ANISOU 4383  N   THR A 586     7836  16161   7704    181   1123
-2326       N
ATOM   4384  CA  THR A 586      -6.743  -8.768  42.018  1.00 83.05
C
ANISOU 4384  CA  THR A 586     7533  16284   7739     -3   1143
-2152       C
ATOM   4385  C   THR A 586      -8.046  -8.964  42.782  1.00 85.85
C
ANISOU 4385  C   THR A 586     7629  16897   8091    198   1362
-1955       C
ATOM   4386  O   THR A 586      -8.488  -8.090  43.533  1.00 92.88
O
ANISOU 4386  O   THR A 586     8607  17838   8846    509   1533
-1983       O
ATOM   4387  CB  THR A 586      -5.592  -9.431  42.778  1.00103.71
C
ANISOU 4387  CB  THR A 586    10320  18860  10227   -148   1124
-2240       C
ATOM   4388  OG1 THR A 586      -5.647  -9.059  44.161  1.00151.03
O
ANISOU 4388  OG1 THR A 586    16442  24928  16015     78   1302
-2288       O
ATOM   4389  CG2 THR A 586      -4.256  -8.987  42.210  1.00100.81
C
ANISOU 4389  CG2 THR A 586    10213  18258   9832   -288    942
-2436       C
ATOM   4390  N   GLY A 587      -8.666 -10.123  42.583  1.00 86.09
N
ANISOU 4390  N   GLY A 587     7353  17090   8267     20   1361
-1744       N
ATOM   4391  CA  GLY A 587      -9.870 -10.448  43.324  1.00148.58
```

```
C
ANISOU 4391  CA  GLY A 587    14978  25275  16199    166   1577
-1519       C
ATOM   4392  C   GLY A 587     -10.053 -11.944  43.436  1.00175.32
C
ANISOU 4392  C   GLY A 587    18148  28783  19683   -114   1568
-1333       C
ATOM   4393  O   GLY A 587      -9.462 -12.726  42.684  1.00168.65
O
ANISOU 4393  O   GLY A 587    17342  27814  18923   -415   1369
-1353       O
ATOM   4394  N   ASP A 588     -10.893 -12.335  44.395  1.00166.10
N
ANISOU 4394  N   ASP A 588    16769  27852  18488      2   1798
-1140       N
ATOM   4395  CA  ASP A 588     -11.076 -13.748  44.714  1.00163.00
C
ANISOU 4395  CA  ASP A 588    16205  27570  18156   -250   1829
-955       C
ATOM   4396  C   ASP A 588     -12.508 -14.150  45.042  1.00146.55
C
ANISOU 4396  C   ASP A 588    13701  25784  16197   -209   2008
-640       C
ATOM   4397  O   ASP A 588     -12.860 -15.310  44.806  1.00137.91
O
ANISOU 4397  O   ASP A 588    12401  24760  15238   -509   1948
-453       O
ATOM   4398  CB  ASP A 588     -10.137 -14.155  45.871  1.00170.77
C
ANISOU 4398  CB  ASP A 588    17465  28502  18917   -221   1937
-1065       C
ATOM   4399  CG  ASP A 588     -10.800 -14.101  47.245  1.00174.61
C
ANISOU 4399  CG  ASP A 588    17876  29201  19269     48   2254
-936       C
ATOM   4400  OD1 ASP A 588     -11.636 -14.979  47.554  1.00184.83
O
ANISOU 4400  OD1 ASP A 588    18882  30690  20654    -45   2384
-676       O
ATOM   4401  OD2 ASP A 588     -10.461 -13.188  48.032  1.00172.34
O
ANISOU 4401  OD2 ASP A 588    17839  28872  18772    351   2374
-1091       O
ATOM   4402  N   GLY A 589     -13.352 -13.259  45.565  1.00140.23
N
ANISOU 4402  N   GLY A 589    12766  25161  15352    147   2227
-560       N
ATOM   4403  CA  GLY A 589     -14.620 -13.646  46.138  1.00160.52
C
ANISOU 4403  CA  GLY A 589    14938  28048  18005    231   2459
-242       C
ATOM   4404  C   GLY A 589     -15.836 -13.054  45.442  1.00123.28
C
ANISOU 4404  C   GLY A 589     9844  23527  13471    357   2458
```

```
-57       C
ATOM   4405  O   GLY A 589     -15.738 -12.306  44.466  1.00102.83
O
ANISOU 4405  O   GLY A 589     7318  20814  10941    393   2271
-184       O
ATOM   4406  N   VAL A 590     -16.999 -13.429  45.981  1.00107.63
N
ANISOU 4406  N   VAL A 590     7452  21866  11575    428   2680
265       N
ATOM   4407  CA  VAL A 590     -18.291 -13.018  45.432  1.00110.79
C
ANISOU 4407  CA  VAL A 590     7399  22530  12166    544   2703
511       C
ATOM   4408  C   VAL A 590     -18.441 -11.500  45.419  1.00111.60
C
ANISOU 4408  C   VAL A 590     7645  22621  12137   1028   2807
383       C
ATOM   4409  O   VAL A 590     -19.048 -10.934  44.501  1.00117.01
O
ANISOU 4409  O   VAL A 590     8126  23374  12960   1095   2683
441       O
ATOM   4410  CB  VAL A 590     -19.439 -13.697  46.205  1.00124.82
C
ANISOU 4410  CB  VAL A 590     8710  24679  14038    554   2972
901       C
ATOM   4411  CG1 VAL A 590     -19.395 -15.204  46.000  1.00114.46
C
ANISOU 4411  CG1 VAL A 590     7246  23356  12887     22   2819
1047       C
ATOM   4412  CG2 VAL A 590     -19.369 -13.361  47.691  1.00119.15
C
ANISOU 4412  CG2 VAL A 590     8169  24044  13057    954   3364
903       C
ATOM   4413  N   ASN A 591     -17.903 -10.815  46.426  1.00124.07
N
ANISOU 4413  N   ASN A 591     9602  24104  13436   1376   3024
208       N
ATOM   4414  CA  ASN A 591     -18.066  -9.370  46.528  1.00115.90
C
ANISOU 4414  CA  ASN A 591     8764  23036  12238   1858   3146
91       C
ATOM   4415  C   ASN A 591     -16.897  -8.592  45.941  1.00109.15
C
ANISOU 4415  C   ASN A 591     8408  21797  11269   1836   2910
-288       C
ATOM   4416  O   ASN A 591     -16.887  -7.360  46.022  1.00118.46
O
ANISOU 4416  O   ASN A 591     9841  22883  12284   2212   2988
-423       O
ATOM   4417  CB  ASN A 591     -18.272  -8.970  47.994  1.00115.38
C
ANISOU 4417  CB  ASN A 591     8849  23085  11905   2296   3532
139       C
ATOM   4418  CG  ASN A 591     -19.538  -9.565  48.589  1.00119.57
```

```
C
ANISOU 4418  CG  ASN A 591     8861  24027  12542   2394   3821
550       C
ATOM   4419  OD1 ASN A 591     -20.499  -9.847  47.874  1.00121.43
O
ANISOU 4419  OD1 ASN A 591     8600  24495  13041   2269   3755
813       O
ATOM   4420  ND2 ASN A 591     -19.540  -9.765  49.903  1.00137.23
N
ANISOU 4420  ND2 ASN A 591    11258  26292  14593   2594   4102
614       N
ATOM   4421  N   ASP A 592     -15.915  -9.279  45.355  1.00105.49
N
ANISOU 4421  N   ASP A 592     8098  21105  10878   1412   2633
-450       N
ATOM   4422  CA  ASP A 592     -14.888  -8.622  44.557  1.00109.97
C
ANISOU 4422  CA  ASP A 592     9045  21337  11400   1334   2383
-758       C
ATOM   4423  C   ASP A 592     -15.401  -8.232  43.176  1.00136.69
C
ANISOU 4423  C   ASP A 592    12237  24714  14983   1280   2178
-715       C
ATOM   4424  O   ASP A 592     -14.780  -7.396  42.509  1.00117.10
O
ANISOU 4424  O   ASP A 592    10057  21982  12452   1325   2026
-941       O
ATOM   4425  CB  ASP A 592     -13.660  -9.524  44.412  1.00 98.70
C
ANISOU 4425  CB  ASP A 592     7832  19695   9975    930   2181
-917       C
ATOM   4426  CG  ASP A 592     -12.834  -9.598  45.680  1.00111.32
C
ANISOU 4426  CG  ASP A 592     9758  21215  11324   1021   2326
-1056       C
ATOM   4427  OD1 ASP A 592     -12.405 -10.713  46.044  1.00129.54
O
ANISOU 4427  OD1 ASP A 592    12046  23535  13640    765   2307
-1015       O
ATOM   4428  OD2 ASP A 592     -12.612  -8.542  46.310  1.00122.95
O
ANISOU 4428  OD2 ASP A 592    11532  22603  12580   1353   2448
-1208       O
ATOM   4429  N   ALA A 593     -16.511  -8.826  42.745  1.00144.21
N
ANISOU 4429  N   ALA A 593    12699  25936  16158   1174   2163
-422       N
ATOM   4430  CA  ALA A 593     -17.012  -8.642  41.385  1.00104.42
C
ANISOU 4430  CA  ALA A 593     7451  20903  11321   1070   1924
-363       C
ATOM   4431  C   ALA A 593     -17.223  -7.190  40.965  1.00114.39
C
ANISOU 4431  C   ALA A 593     8872  22093  12499   1460   1947
```

```
-474        C
ATOM   4432  O   ALA A 593     -16.854  -6.856  39.826  1.00168.14
O
ANISOU 4432  O   ALA A 593    15817  28695  19375   1344   1696
-611        O
ATOM   4433  CB  ALA A 593     -18.312  -9.442  41.211  1.00112.92
C
ANISOU 4433  CB  ALA A 593     7938  22335  12632    936   1935
7       C
ATOM   4434  N   PRO A 594     -17.791  -6.292  41.779  1.00108.21
N
ANISOU 4434  N   PRO A 594     8109  21450  11554   1933   2236
-421        N
ATOM   4435  CA  PRO A 594     -17.925  -4.908  41.290  1.00108.97
C
ANISOU 4435  CA  PRO A 594     8421  21431  11553   2297   2237
-543        C
ATOM   4436  C   PRO A 594     -16.598  -4.177  41.214  1.00105.91
C
ANISOU 4436  C   PRO A 594     8639  20626  10975   2292   2137
-911        C
ATOM   4437  O   PRO A 594     -16.398  -3.361  40.305  1.00114.75
O
ANISOU 4437  O   PRO A 594     9954  21550  12095   2358   1988
-1048       O
ATOM   4438  CB  PRO A 594     -18.869  -4.260  42.316  1.00113.21
C
ANISOU 4438  CB  PRO A 594     8846  22231  11936   2819   2605
-368        C
ATOM   4439  CG  PRO A 594     -18.764  -5.121  43.530  1.00113.67
C
ANISOU 4439  CG  PRO A 594     8839  22419  11930   2737   2804
-275        C
ATOM   4440  CD  PRO A 594     -18.576  -6.506  43.006  1.00111.60
C
ANISOU 4440  CD  PRO A 594     8299  22191  11913   2181   2575
-187        C
ATOM   4441  N   ALA A 595     -15.686  -4.437  42.149  1.00104.41
N
ANISOU 4441  N   ALA A 595     8750  20299  10622   2211   2210
-1064       N
ATOM   4442  CA  ALA A 595     -14.342  -3.877  42.055  1.00117.05
C
ANISOU 4442  CA  ALA A 595    10882  21519  12071   2122   2075
-1396       C
ATOM   4443  C   ALA A 595     -13.605  -4.391  40.820  1.00122.00
C
ANISOU 4443  C   ALA A 595    11520  21953  12880   1693   1752
-1494       C
ATOM   4444  O   ALA A 595     -12.965  -3.620  40.096  1.00 96.56
O
ANISOU 4444  O   ALA A 595     8604  18462   9624   1685   1606
-1692       O
ATOM   4445  CB  ALA A 595     -13.555  -4.197  43.326  1.00113.36
```

```
C
ANISOU 4445  CB  ALA A 595    10676  20989  11407   2095   2195
-1509       C
ATOM   4446  N   LEU A 596     -13.685  -5.702  40.569  1.00 97.02
N
ANISOU 4446  N   LEU A 596     8049  18915   9899   1340   1647
-1349       N
ATOM   4447  CA  LEU A 596     -13.142  -6.283  39.342  1.00 94.22
C
ANISOU 4447  CA  LEU A 596     7688  18400   9713    962   1351
-1401       C
ATOM   4448  C   LEU A 596     -13.786  -5.708  38.086  1.00 94.93
C
ANISOU 4448  C   LEU A 596     7657  18477   9935   1037   1205
-1351       C
ATOM   4449  O   LEU A 596     -13.088  -5.373  37.120  1.00110.07
O
ANISOU 4449  O   LEU A 596     9812  20135  11873    918   1008
-1513       O
ATOM   4450  CB  LEU A 596     -13.320  -7.802  39.365  1.00 93.76
C
ANISOU 4450  CB  LEU A 596     7326  18492   9805    610   1284
-1220       C
ATOM   4451  CG  LEU A 596     -12.471  -8.611  40.347  1.00 92.32
C
ANISOU 4451  CG  LEU A 596     7293  18273   9512    445   1359
-1282       C
ATOM   4452  CD1 LEU A 596     -12.983 -10.041  40.432  1.00 93.46
C
ANISOU 4452  CD1 LEU A 596     7100  18608   9802    161   1339
-1046       C
ATOM   4453  CD2 LEU A 596     -11.002  -8.589  39.954  1.00 88.87
C
ANISOU 4453  CD2 LEU A 596     7240  17522   9003    249   1184
-1536       C
ATOM   4454  N   LYS A 597     -15.112  -5.578  38.075  1.00140.19
N
ANISOU 4454  N   LYS A 597    13015  24496  15754   1245   1301
-1117       N
ATOM   4455  CA  LYS A 597     -15.784  -5.013  36.908  1.00130.61
C
ANISOU 4455  CA  LYS A 597    11672  23297  14656   1346   1157
-1057       C
ATOM   4456  C   LYS A 597     -15.327  -3.581  36.645  1.00121.67
C
ANISOU 4456  C   LYS A 597    10962  21909  13359   1645   1181
-1283       C
ATOM   4457  O   LYS A 597     -15.046  -3.210  35.500  1.00160.33
O
ANISOU 4457  O   LYS A 597    16001  26607  18311   1573    978
-1381       O
ATOM   4458  CB  LYS A 597     -17.299  -5.087  37.096  1.00107.65
C
ANISOU 4458  CB  LYS A 597     8257  20788  11857   1553   1282
```

```
-743       C
ATOM   4459  CG  LYS A 597     -18.107  -4.689  35.871  1.00104.65
C
ANISOU 4459  CG  LYS A 597     7660  20481  11622   1627   1101
-637       C
ATOM   4460  CD  LYS A 597     -19.594  -4.920  36.102  1.00119.29
C
ANISOU 4460  CD  LYS A 597     8937  22779  13608   1782   1211
-286       C
ATOM   4461  CE  LYS A 597     -20.422  -4.483  34.905  1.00122.99
C
ANISOU 4461  CE  LYS A 597     9175  23344  14211   1881   1016
-172       C
ATOM   4462  NZ  LYS A 597     -20.167  -5.351  33.722  1.00116.78
N
ANISOU 4462  NZ  LYS A 597     8335  22425  13608   1413    640
-183       N
ATOM   4463  N   LYS A 598     -15.266  -2.755  37.690  1.00111.24
N
ANISOU 4463  N   LYS A 598     9873  20575  11820   1986   1430
-1364       N
ATOM   4464  CA  LYS A 598     -14.931  -1.347  37.514  1.00100.52
C
ANISOU 4464  CA  LYS A 598     8942  18966  10284   2287   1465
-1564       C
ATOM   4465  C   LYS A 598     -13.431  -1.104  37.367  1.00 97.22
C
ANISOU 4465  C   LYS A 598     9007  18161   9771   2056   1333
-1859       C
ATOM   4466  O   LYS A 598     -13.039  -0.077  36.803  1.00100.32
O
ANISOU 4466  O   LYS A 598     9741  18295  10081   2172   1272
-2023       O
ATOM   4467  CB  LYS A 598     -15.485  -0.529  38.685  1.00103.77
C
ANISOU 4467  CB  LYS A 598     9461  19493  10473   2763   1777
-1530       C
ATOM   4468  CG  LYS A 598     -15.670   0.956  38.390  1.00110.46
C
ANISOU 4468  CG  LYS A 598    10648  20169  11154   3172   1839
-1639       C
ATOM   4469  CD  LYS A 598     -14.496   1.779  38.886  1.00121.25
C
ANISOU 4469  CD  LYS A 598    12639  21155  12275   3199   1854
-1944       C
ATOM   4470  CE  LYS A 598     -14.782   3.265  38.780  1.00131.03
C
ANISOU 4470  CE  LYS A 598    14254  22222  13309   3647   1956
-2035       C
ATOM   4471  NZ  LYS A 598     -14.926   3.693  37.362  1.00105.98
N
ANISOU 4471  NZ  LYS A 598    11068  18934  10266   3619   1769
-2047       N
ATOM   4472  N   ALA A 599     -12.589  -2.016  37.848  1.00 95.00
```

```
N
ANISOU 4472  N   ALA A 599     8756  17841   9498   1736   1290
-1916       N
ATOM   4473  CA  ALA A 599     -11.152  -1.910  37.615  1.00102.53
C
ANISOU 4473  CA  ALA A 599    10092  18470  10394   1482   1143
-2158       C
ATOM   4474  C   ALA A 599     -10.817  -1.900  36.126  1.00 94.92
C
ANISOU 4474  C   ALA A 599     9158  17326   9580   1277    903
-2201       C
ATOM   4475  O   ALA A 599     -11.501  -2.516  35.303  1.00 90.14
O
ANISOU 4475  O   ALA A 599     8235  16859   9157   1176    790
-2038       O
ATOM   4476  CB  ALA A 599     -10.416  -3.061  38.301  1.00148.95
C
ANISOU 4476  CB  ALA A 599    15921  24393  16278   1179   1129
-2165       C
ATOM   4477  N   ASP A 600      -9.744  -1.175  35.788  1.00114.97
N
ANISOU 4477  N   ASP A 600    12102  19549  12034   1212    821
-2418       N
ATOM   4478  CA  ASP A 600      -9.226  -1.173  34.422  1.00 95.77
C
ANISOU 4478  CA  ASP A 600     9754  16913   9720   1009    612
-2470       C
ATOM   4479  C   ASP A 600      -8.789  -2.565  33.981  1.00 84.14
C
ANISOU 4479  C   ASP A 600     8083  15489   8398    635    462
-2395       C
ATOM   4480  O   ASP A 600      -9.068  -2.984  32.852  1.00 83.50
O
ANISOU 4480  O   ASP A 600     7869  15398   8461    517    304
-2311       O
ATOM   4481  CB  ASP A 600      -8.058  -0.193  34.303  1.00 85.48
C
ANISOU 4481  CB  ASP A 600     8913  15273   8292    979    578
-2700       C
ATOM   4482  CG  ASP A 600      -8.440   1.223  34.688  1.00 87.97
C
ANISOU 4482  CG  ASP A 600     9508  15484   8431   1342    712
-2789       C
ATOM   4483  OD1 ASP A 600      -8.187   1.616  35.847  1.00 89.05
O
ANISOU 4483  OD1 ASP A 600     9843  15601   8390   1455    840
-2879       O
ATOM   4484  OD2 ASP A 600      -8.993   1.944  33.832  1.00 96.22
O
ANISOU 4484  OD2 ASP A 600    10605  16456   9499   1530    686
-2769       O
ATOM   4485  N   ILE A 601      -8.109  -3.299  34.857  1.00 83.03
N
ANISOU 4485  N   ILE A 601     7947  15392   8208    460    504
```

```
-2424       N
ATOM   4486  CA  ILE A 601      -7.873  -4.727  34.680  1.00 81.39
C
ANISOU 4486  CA  ILE A 601     7537  15274   8113    154    405
-2321       C
ATOM   4487  C   ILE A 601      -8.368  -5.432  35.934  1.00 82.60
C
ANISOU 4487  C   ILE A 601     7474  15684   8227    185    560
-2209       C
ATOM   4488  O   ILE A 601      -7.752  -5.316  37.000  1.00 88.54
O
ANISOU 4488  O   ILE A 601     8386  16422   8834    217    670
-2308       O
ATOM   4489  CB  ILE A 601      -6.394  -5.066  34.420  1.00104.24
C
ANISOU 4489  CB  ILE A 601    10667  17958  10982   -108    299
-2452       C
ATOM   4490  CG1 ILE A 601      -5.742  -4.117  33.402  1.00 77.81
C
ANISOU 4490  CG1 ILE A 601     7597  14335   7633   -102    201
-2583       C
ATOM   4491  CG2 ILE A 601      -6.257  -6.510  33.952  1.00111.72
C
ANISOU 4491  CG2 ILE A 601    11442  18965  12041   -385    180
-2331       C
ATOM   4492  CD1 ILE A 601      -5.232  -2.796  33.972  1.00 78.64
C
ANISOU 4492  CD1 ILE A 601     8013  14282   7585     58    290
-2760       C
ATOM   4493  N   GLY A 602      -9.473  -6.161  35.810  1.00 98.83
N
ANISOU 4493  N   GLY A 602     9170  17972  10410    167    562
-1996       N
ATOM   4494  CA  GLY A 602      -9.842  -7.124  36.827  1.00136.29
C
ANISOU 4494  CA  GLY A 602    13688  22947  15150    105    683
-1858       C
ATOM   4495  C   GLY A 602      -9.081  -8.424  36.679  1.00 88.88
C
ANISOU 4495  C   GLY A 602     7689  16889   9191   -246    564
-1842       C
ATOM   4496  O   GLY A 602      -9.104  -9.050  35.615  1.00126.78
O
ANISOU 4496  O   GLY A 602    12425  21625  14120   -458    374
-1779       O
ATOM   4497  N   ILE A 603      -8.396  -8.838  37.740  1.00 81.97
N
ANISOU 4497  N   ILE A 603     6920  16032   8193   -290    669
-1898       N
ATOM   4498  CA  ILE A 603      -7.538 -10.016  37.722  1.00 79.94
C
ANISOU 4498  CA  ILE A 603     6720  15716   7938   -578    579
-1898       C
ATOM   4499  C   ILE A 603      -8.109 -11.009  38.722  1.00 81.28
```

```
C
ANISOU 4499  C   ILE A 603     6673  16113   8098   -623    716
-1731       C
ATOM   4500  O   ILE A 603      -8.060 -10.775  39.936  1.00120.19
O
ANISOU 4500  O   ILE A 603    11646  21134  12887   -460    901
-1763       O
ATOM   4501  CB  ILE A 603      -6.082  -9.668  38.061  1.00 83.10
C
ANISOU 4501  CB  ILE A 603     7448  15932   8192   -606    566
-2108       C
ATOM   4502  CG1 ILE A 603      -5.558  -8.581  37.121  1.00 77.13
C
ANISOU 4502  CG1 ILE A 603     6906  14952   7448   -559    457
-2258       C
ATOM   4503  CG2 ILE A 603      -5.201 -10.908  37.983  1.00 78.11
C
ANISOU 4503  CG2 ILE A 603     6866  15257   7556   -868    480
-2088       C
ATOM   4504  CD1 ILE A 603      -4.256  -7.958  37.583  1.00 76.09
C
ANISOU 4504  CD1 ILE A 603     7073  14663   7174   -560    459
-2456       C
ATOM   4505  N   ALA A 604      -8.659 -12.112  38.223  1.00 81.55
N
ANISOU 4505  N   ALA A 604     6493  16223   8268   -844    622
-1551       N
ATOM   4506  CA  ALA A 604      -9.125 -13.176  39.099  1.00 82.81
C
ANISOU 4506  CA  ALA A 604     6466  16573   8426   -940    740
-1379       C
ATOM   4507  C   ALA A 604      -7.969 -14.099  39.454  1.00 84.67
C
ANISOU 4507  C   ALA A 604     6920  16697   8555  -1123    711
-1450       C
ATOM   4508  O   ALA A 604      -7.278 -14.611  38.567  1.00 78.86
O
ANISOU 4508  O   ALA A 604     6329  15786   7849  -1320    529
-1494       O
ATOM   4509  CB  ALA A 604     -10.247 -13.971  38.437  1.00 84.43
C
ANISOU 4509  CB  ALA A 604     6355  16902   8822  -1129    636
-1141       C
ATOM   4510  N   VAL A 605      -7.759 -14.306  40.757  1.00 82.24
N
ANISOU 4510  N   VAL A 605     6648  16493   8105  -1031    899
-1455       N
ATOM   4511  CA  VAL A 605      -6.744 -15.248  41.216  1.00 89.51
C
ANISOU 4511  CA  VAL A 605     7752  17346   8911  -1177    890
-1497       C
ATOM   4512  C   VAL A 605      -7.127 -16.670  40.808  1.00 80.08
C
ANISOU 4512  C   VAL A 605     6440  16171   7815  -1456    808
```

```
-1306       C
ATOM   4513  O   VAL A 605      -8.288 -16.976  40.515  1.00 81.96
O
ANISOU 4513  O   VAL A 605     6412  16530   8200  -1536    800
-1117       O
ATOM   4514  CB  VAL A 605      -6.541 -15.131  42.737  1.00132.02
C
ANISOU 4514  CB  VAL A 605    13204  22846  14111   -995   1107
-1535       C
ATOM   4515  CG1 VAL A 605      -7.678 -15.809  43.487  1.00153.49
C
ANISOU 4515  CG1 VAL A 605    15663  25792  16865   -985   1289
-1303       C
ATOM   4516  CG2 VAL A 605      -5.179 -15.688  43.154  1.00160.59
C
ANISOU 4516  CG2 VAL A 605    17081  26361  17575  -1079   1066
-1654       C
ATOM   4517  N   ALA A 606      -6.114 -17.540  40.743  1.00 83.59
N
ANISOU 4517  N   ALA A 606     7098  16489   8173  -1609    733
-1350       N
ATOM   4518  CA  ALA A 606      -6.303 -18.868  40.165  1.00117.99
C
ANISOU 4518  CA  ALA A 606    11447  20790  12596  -1882    616
-1198       C
ATOM   4519  C   ALA A 606      -7.427 -19.631  40.858  1.00145.91
C
ANISOU 4519  C   ALA A 606    14737  24516  16186  -1969    743
-970       C
ATOM   4520  O   ALA A 606      -8.205 -20.334  40.203  1.00157.64
O
ANISOU 4520  O   ALA A 606    16085  26002  17810  -2193    626
-800       O
ATOM   4521  CB  ALA A 606      -4.995 -19.658  40.232  1.00121.11
C
ANISOU 4521  CB  ALA A 606    12134  21041  12839  -1962    571
-1276       C
ATOM   4522  N   ASP A 607      -7.530 -19.509  42.182  1.00158.86
N
ANISOU 4522  N   ASP A 607    16328  26315  17716  -1803    976
-955       N
ATOM   4523  CA  ASP A 607      -8.572 -20.175  42.950  1.00157.67
C
ANISOU 4523  CA  ASP A 607    15937  26363  17607  -1858   1141
-723       C
ATOM   4524  C   ASP A 607      -9.774 -19.273  43.225  1.00146.33
C
ANISOU 4524  C   ASP A 607    14178  25145  16276  -1669   1280
-621       C
ATOM   4525  O   ASP A 607     -10.577 -19.578  44.113  1.00174.87
O
ANISOU 4525  O   ASP A 607    17583  28964  19894  -1623   1488
-437       O
ATOM   4526  CB  ASP A 607      -7.995 -20.697  44.267  1.00172.36
```

```
C
ANISOU 4526  CB  ASP A 607    17954  28272  19265  -1772   1335
-739       C
ATOM   4527  CG  ASP A 607      -7.039 -21.857  44.065  1.00196.79
C
ANISOU 4527  CG  ASP A 607    21317  31193  22260  -1970   1225
-762       C
ATOM   4528  OD1 ASP A 607      -7.221 -22.619  43.093  1.00202.01
O
ANISOU 4528  OD1 ASP A 607    21989  31737  23027  -2230   1046
-670       O
ATOM   4529  OD2 ASP A 607      -6.106 -22.006  44.882  1.00197.64
O
ANISOU 4529  OD2 ASP A 607    21642  31280  22171  -1853   1312
-870       O
ATOM   4530  N   ALA A 608      -9.906 -18.169  42.492  1.00137.48
N
ANISOU 4530  N   ALA A 608    13019  23987  15228  -1538   1186
-725       N
ATOM   4531  CA  ALA A 608     -11.065 -17.295  42.633  1.00125.14
C
ANISOU 4531  CA  ALA A 608    11157  22631  13761  -1330   1309
-618       C
ATOM   4532  C   ALA A 608     -12.369 -18.034  42.348  1.00109.57
C
ANISOU 4532  C   ALA A 608     8796  20844  11990  -1535   1291
-315       C
ATOM   4533  O   ALA A 608     -12.407 -19.022  41.610  1.00115.03
O
ANISOU 4533  O   ALA A 608     9479  21443  12785  -1870   1091
-224       O
ATOM   4534  CB  ALA A 608     -10.945 -16.086  41.705  1.00126.07
C
ANISOU 4534  CB  ALA A 608    11336  22640  13926  -1187   1173
-777       C
ATOM   4535  N   THR A 609     -13.445 -17.541  42.958  1.00103.01
N
ANISOU 4535  N   THR A 609     7650  20280  11208  -1326   1504
-147       N
ATOM   4536  CA  THR A 609     -14.786 -17.999  42.623  1.00117.01
C
ANISOU 4536  CA  THR A 609     8980  22276  13201  -1491   1481
160       C
ATOM   4537  C   THR A 609     -15.098 -17.719  41.155  1.00103.11
C
ANISOU 4537  C   THR A 609     7127  20434  11618  -1631   1175
150       C
ATOM   4538  O   THR A 609     -14.593 -16.764  40.558  1.00 95.99
O
ANISOU 4538  O   THR A 609     6417  19383  10671  -1459   1076
-66       O
ATOM   4539  CB  THR A 609     -15.823 -17.321  43.520  1.00133.71
C
ANISOU 4539  CB  THR A 609    10773  24711  15321  -1161   1790
```

337 C
ATOM 4540 OG1 THR A 609 -15.753 -15.900 43.350 1.00144.85
O
ANISOU 4540 OG1 THR A 609 12271 26102 16663 -792 1826
168 O
ATOM 4541 CG2 THR A 609 -15.571 -17.667 44.976 1.00142.55
C
ANISOU 4541 CG2 THR A 609 11999 25909 16253 -1021 2096
367 C
ATOM 4542 N ASP A 610 -15.932 -18.584 40.572 1.00108.11
N
ANISOU 4542 N ASP A 610 7478 21154 12447 -1962 1013
390 N
ATOM 4543 CA ASP A 610 -16.363 -18.418 39.186 1.00116.95
C
ANISOU 4543 CA ASP A 610 8486 22215 13737 -2115 701
413 C
ATOM 4544 C ASP A 610 -17.074 -17.088 38.951 1.00101.64
C
ANISOU 4544 C ASP A 610 6309 20451 11858 -1770 760
424 C
ATOM 4545 O ASP A 610 -16.956 -16.506 37.866 1.00100.49
O
ANISOU 4545 O ASP A 610 6254 20170 11758 -1743 537
303 O
ATOM 4546 CB ASP A 610 -17.281 -19.575 38.788 1.00128.19
C
ANISOU 4546 CB ASP A 610 9609 23742 15355 -2533 530
706 C
ATOM 4547 CG ASP A 610 -16.536 -20.885 38.635 1.00146.72
C
ANISOU 4547 CG ASP A 610 12279 25829 17637 -2904 381
670 C
ATOM 4548 OD1 ASP A 610 -17.007 -21.902 39.187 1.00151.42
O
ANISOU 4548 OD1 ASP A 610 12723 26528 18282 -3153 448
893 O
ATOM 4549 OD2 ASP A 610 -15.480 -20.899 37.968 1.00172.69
O
ANISOU 4549 OD2 ASP A 610 15982 28812 20819 -2934 209
431 O
ATOM 4550 N ALA A 611 -17.822 -16.601 39.941 1.00104.38
N
ANISOU 4550 N ALA A 611 6371 21096 12194 -1484 1067
576 N
ATOM 4551 CA ALA A 611 -18.361 -15.244 39.882 1.00149.00
C
ANISOU 4551 CA ALA A 611 11880 26895 17840 -1069 1179
560 C
ATOM 4552 C ALA A 611 -17.271 -14.187 39.728 1.00102.43
C
ANISOU 4552 C ALA A 611 6445 20722 11752 -799 1174
206 C
ATOM 4553 O ALA A 611 -17.420 -13.245 38.941 1.00102.28

```
O
ANISOU 4553  O   ALA A 611     6447  20655  11762   -624   1065
120       O
ATOM   4554  CB  ALA A 611     -19.199 -14.965  41.129  1.00157.88
C
ANISOU 4554  CB  ALA A 611    12697  28360  18928   -766   1559
775       C
ATOM   4555  N   ALA A 612     -16.175 -14.312  40.477  1.00140.58
N
ANISOU 4555  N   ALA A 612    11651  25376  16388   -766   1288
8       N
ATOM   4556  CA  ALA A 612     -15.020 -13.447  40.247  1.00140.20
C
ANISOU 4556  CA  ALA A 612    12048  25042  16178   -605   1232
-322       C
ATOM   4557  C   ALA A 612     -14.406 -13.645  38.863  1.00156.46
C
ANISOU 4557  C   ALA A 612    14296  26835  18317   -864    894
-454       C
ATOM   4558  O   ALA A 612     -14.039 -12.669  38.197  1.00153.65
O
ANISOU 4558  O   ALA A 612    14130  26322  17928   -707    803
-632       O
ATOM   4559  CB  ALA A 612     -13.972 -13.682  41.334  1.00162.88
C
ANISOU 4559  CB  ALA A 612    15245  27803  18837   -565   1389
-479       C
ATOM   4560  N   ARG A 613     -14.273 -14.896  38.416  1.00156.24
N
ANISOU 4560  N   ARG A 613    14251  26736  18376  -1250    713
-368       N
ATOM   4561  CA  ARG A 613     -13.790 -15.160  37.061  1.00116.57
C
ANISOU 4561  CA  ARG A 613     9412  21462  13417  -1482    394
-464       C
ATOM   4562  C   ARG A 613     -14.685 -14.539  35.992  1.00141.46
C
ANISOU 4562  C   ARG A 613    12347  24678  16723  -1428    223
-386       C
ATOM   4563  O   ARG A 613     -14.186 -14.002  34.996  1.00157.77
O
ANISOU 4563  O   ARG A 613    14640  26527  18779  -1398     45
-549       O
ATOM   4564  CB  ARG A 613     -13.650 -16.666  36.833  1.00119.74
C
ANISOU 4564  CB  ARG A 613     9843  21786  13869  -1888    241
-351       C
ATOM   4565  CG  ARG A 613     -12.718 -17.350  37.818  1.00118.03
C
ANISOU 4565  CG  ARG A 613     9864  21495  13487  -1938    394
-425       C
ATOM   4566  CD  ARG A 613     -12.196 -18.673  37.284  1.00120.87
C
ANISOU 4566  CD  ARG A 613    10426  21656  13842  -2298    196
```

```
-400       C
ATOM   4567  NE  ARG A 613     -13.266 -19.560  36.838  1.00135.74
N
ANISOU 4567  NE  ARG A 613    12043  23636  15897  -2600     43
-146       N
ATOM   4568  CZ  ARG A 613     -13.337 -20.086  35.619  1.00146.90
C
ANISOU 4568  CZ  ARG A 613    13551  24875  17390  -2860   -265
-119       C
ATOM   4569  NH1 ARG A 613     -12.393 -19.827  34.726  1.00161.29
N
ANISOU 4569  NH1 ARG A 613    15726  26424  19134  -2826   -424
-325       N
ATOM   4570  NH2 ARG A 613     -14.346 -20.885  35.300  1.00146.29
N
ANISOU 4570  NH2 ARG A 613    13226  24892  17466  -3158   -417
121       N
ATOM   4571  N   GLY A 614     -16.006 -14.599  36.173  1.00111.69
N
ANISOU 4571  N   GLY A 614     8129  21213  13097  -1407    274
-125       N
ATOM   4572  CA  GLY A 614     -16.901 -13.963  35.216  1.00107.39
C
ANISOU 4572  CA  GLY A 614     7347  20765  12691  -1320    114
-37       C
ATOM   4573  C   GLY A 614     -16.764 -12.452  35.175  1.00 98.05
C
ANISOU 4573  C   GLY A 614     6296  19549  11408   -889    234
-205       C
ATOM   4574  O   GLY A 614     -16.805 -11.845  34.102  1.00132.71
O
ANISOU 4574  O   GLY A 614    10765  23822  15838   -828     42
-283       O
ATOM   4575  N   ALA A 615     -16.598 -11.826  36.338  1.00 98.34
N
ANISOU 4575  N   ALA A 615     6395  19671  11298   -579    547
-264       N
ATOM   4576  CA  ALA A 615     -16.548 -10.372  36.426  1.00 98.50
C
ANISOU 4576  CA  ALA A 615     6567  19658  11201   -153    682
-409       C
ATOM   4577  C   ALA A 615     -15.221  -9.789  35.956  1.00 94.89
C
ANISOU 4577  C   ALA A 615     6617  18828  10608   -135    584
-731       C
ATOM   4578  O   ALA A 615     -15.175  -8.609  35.593  1.00 94.90
O
ANISOU 4578  O   ALA A 615     6777  18735  10545    141    597
-856       O
ATOM   4579  CB  ALA A 615     -16.822  -9.922  37.861  1.00127.76
C
ANISOU 4579  CB  ALA A 615    10216  23559  14768    177   1045
-361       C
ATOM   4580  N   SER A 616     -14.151 -10.580  35.951  1.00 92.04
```

```
N
ANISOU 4580  N   SER A 616     6511  18260  10202   -417    493
-852       N
ATOM   4581  CA  SER A 616     -12.809 -10.070  35.710  1.00126.95
C
ANISOU 4581  CA  SER A 616    11384  22365  14488   -401    443
-1133       C
ATOM   4582  C   SER A 616     -12.458 -10.071  34.227  1.00117.73
C
ANISOU 4582  C   SER A 616    10362  20969  13401   -563    160
-1207       C
ATOM   4583  O   SER A 616     -12.796 -11.002  33.490  1.00127.63
O
ANISOU 4583  O   SER A 616    11480  22231  14782   -831    -40
-1079       O
ATOM   4584  CB  SER A 616     -11.785 -10.903  36.480  1.00128.65
C
ANISOU 4584  CB  SER A 616    11790  22493  14597   -586    502
-1213       C
ATOM   4585  OG  SER A 616     -12.074 -12.285  36.357  1.00129.22
O
ANISOU 4585  OG  SER A 616    11681  22643  14775   -902    403
-1039       O
ATOM   4586  N   ASP A 617     -11.773  -9.005  33.802  1.00119.77
N
ANISOU 4586  N   ASP A 617    10925  21010  13571   -396    146
-1412       N
ATOM   4587  CA  ASP A 617     -11.285  -8.899  32.430  1.00121.60
C
ANISOU 4587  CA  ASP A 617    11358  20995  13848   -513    -89
-1502       C
ATOM   4588  C   ASP A 617     -10.305 -10.014  32.083  1.00129.76
C
ANISOU 4588  C   ASP A 617    12569  21856  14876   -836   -223
-1540       C
ATOM   4589  O   ASP A 617     -10.360 -10.578  30.984  1.00170.17
O
ANISOU 4589  O   ASP A 617    17712  26868  20076  -1020   -445
-1490       O
ATOM   4590  CB  ASP A 617     -10.625  -7.536  32.222  1.00110.47
C
ANISOU 4590  CB  ASP A 617    10268  19378  12328   -280    -35
-1712       C
ATOM   4591  CG  ASP A 617     -11.436  -6.405  32.812  1.00126.00
C
ANISOU 4591  CG  ASP A 617    12145  21490  14240     84    148
-1697       C
ATOM   4592  OD1 ASP A 617     -11.513  -6.333  34.056  1.00132.10
O
ANISOU 4592  OD1 ASP A 617    12875  22399  14918    208    358
-1689       O
ATOM   4593  OD2 ASP A 617     -11.995  -5.595  32.044  1.00133.20
O
ANISOU 4593  OD2 ASP A 617    13051  22375  15184    269     88
```

```
-1689       O
ATOM   4594  N   ILE A 618      -9.401 -10.347  33.002  1.00 99.63
N
ANISOU 4594  N   ILE A 618     8897  18009  10949   -890    -96
-1626       N
ATOM   4595  CA  ILE A 618      -8.426 -11.410  32.791  1.00 78.12
C
ANISOU 4595  CA  ILE A 618     6347  15142   8194  -1153   -192
-1653       C
ATOM   4596  C   ILE A 618      -8.450 -12.366  33.974  1.00 78.55
C
ANISOU 4596  C   ILE A 618     6282  15360   8203  -1253    -61
-1564       C
ATOM   4597  O   ILE A 618      -8.635 -11.951  35.124  1.00103.99
O
ANISOU 4597  O   ILE A 618     9428  18731  11354  -1084    142
-1576       O
ATOM   4598  CB  ILE A 618      -7.005 -10.837  32.581  1.00 75.74
C
ANISOU 4598  CB  ILE A 618     6395  14600   7781  -1121   -188
-1861       C
ATOM   4599  CG1 ILE A 618      -6.040 -11.930  32.116  1.00 96.05
C
ANISOU 4599  CG1 ILE A 618     9143  17026  10326  -1358   -300
-1861       C
ATOM   4600  CG2 ILE A 618      -6.488 -10.157  33.842  1.00114.39
C
ANISOU 4600  CG2 ILE A 618    11373  19545  12545   -963     13
-1983       C
ATOM   4601  CD1 ILE A 618      -4.702 -11.400  31.643  1.00129.69
C
ANISOU 4601  CD1 ILE A 618    13702  21062  14510  -1340   -320
-2021       C
ATOM   4602  N   VAL A 619      -8.292 -13.655  33.684  1.00 77.99
N
ANISOU 4602  N   VAL A 619     6224  15249   8161  -1517   -177
-1469       N
ATOM   4603  CA  VAL A 619      -8.371 -14.712  34.683  1.00 78.56
C
ANISOU 4603  CA  VAL A 619     6198  15457   8195  -1643    -72
-1361       C
ATOM   4604  C   VAL A 619      -7.046 -15.460  34.690  1.00 88.66
C
ANISOU 4604  C   VAL A 619     7772  16561   9356  -1777   -105
-1451       C
ATOM   4605  O   VAL A 619      -6.657 -16.051  33.675  1.00121.72
O
ANISOU 4605  O   VAL A 619    12122  20568  13558  -1936   -284
-1443       O
ATOM   4606  CB  VAL A 619      -9.534 -15.674  34.404  1.00 80.71
C
ANISOU 4606  CB  VAL A 619     6203  15857   8606  -1852   -177
-1126       C
ATOM   4607  CG1 VAL A 619      -9.502 -16.835  35.382  1.00 81.23
```

```
C
ANISOU 4607  CG1 VAL A 619     6223  16022   8621  -2007    -68
-1014       C
ATOM   4608  CG2 VAL A 619     -10.857 -14.936  34.483  1.00 83.40
C
ANISOU 4608  CG2 VAL A 619     6198  16424   9066  -1695   -125
-1007       C
ATOM   4609  N   LEU A 620      -6.355 -15.428  35.824  1.00 78.01
N
ANISOU 4609  N   LEU A 620     6502  15264   7874  -1692     67
-1530       N
ATOM   4610  CA  LEU A 620      -5.160 -16.239  36.002  1.00 73.85
C
ANISOU 4610  CA  LEU A 620     6207  14626   7226  -1801     56
-1583       C
ATOM   4611  C   LEU A 620      -5.532 -17.710  36.140  1.00 81.59
C
ANISOU 4611  C   LEU A 620     7140  15645   8214  -2018     25
-1409       C
ATOM   4612  O   LEU A 620      -6.589 -18.060  36.671  1.00 86.90
O
ANISOU 4612  O   LEU A 620     7573  16491   8953  -2063     94
-1259       O
ATOM   4613  CB  LEU A 620      -4.375 -15.794  37.236  1.00 95.14
C
ANISOU 4613  CB  LEU A 620     8988  17388   9773  -1651    230
-1707       C
ATOM   4614  CG  LEU A 620      -3.989 -14.323  37.396  1.00 98.23
C
ANISOU 4614  CG  LEU A 620     9458  17741  10123  -1445    280
-1883       C
ATOM   4615  CD1 LEU A 620      -3.156 -14.142  38.653  1.00 72.86
C
ANISOU 4615  CD1 LEU A 620     6360  14581   6743  -1352    409
-1991       C
ATOM   4616  CD2 LEU A 620      -3.225 -13.826  36.182  1.00 90.30
C
ANISOU 4616  CD2 LEU A 620     8633  16523   9153  -1478    131
-1984       C
ATOM   4617  N   THR A 621      -4.650 -18.579  35.652  1.00 93.68
N
ANISOU 4617  N   THR A 621     8913  17011   9671  -2149    -72
-1419       N
ATOM   4618  CA  THR A 621      -4.743 -20.001  35.955  1.00 91.74
C
ANISOU 4618  CA  THR A 621     8714  16768   9375  -2335    -77
-1280       C
ATOM   4619  C   THR A 621      -3.531 -20.468  36.746  1.00 85.44
C
ANISOU 4619  C   THR A 621     8117  15954   8393  -2279     35
-1351       C
ATOM   4620  O   THR A 621      -3.341 -21.677  36.926  1.00 91.32
O
ANISOU 4620  O   THR A 621     8985  16656   9055  -2407     30
```

```
-1256       O
ATOM   4621  CB  THR A 621      -4.897 -20.836  34.680  1.00 97.18
C
ANISOU 4621  CB  THR A 621     9551  17263  10109  -2540   -303
-1193       C
ATOM   4622  OG1 THR A 621      -5.044 -22.217  35.033  1.00104.73
O
ANISOU 4622  OG1 THR A 621    10583  18205  11004  -2731   -306
-1053       O
ATOM   4623  CG2 THR A 621      -3.675 -20.688  33.787  1.00128.71
C
ANISOU 4623  CG2 THR A 621    13851  21039  14015  -2478   -391
-1317       C
ATOM   4624  N   GLU A 622      -2.708 -19.533  37.225  1.00 90.56
N
ANISOU 4624  N   GLU A 622     8809  16631   8967  -2093    124
-1511       N
ATOM   4625  CA  GLU A 622      -1.468 -19.814  37.917  1.00 77.66
C
ANISOU 4625  CA  GLU A 622     7346  14998   7164  -2026    201
-1589       C
ATOM   4626  C   GLU A 622      -1.326 -18.735  38.982  1.00 92.71
C
ANISOU 4626  C   GLU A 622     9166  17040   9020  -1843    337
-1712       C
ATOM   4627  O   GLU A 622      -1.596 -17.557  38.704  1.00 83.67
O
ANISOU 4627  O   GLU A 622     7953  15886   7952  -1747    322
-1800       O
ATOM   4628  CB  GLU A 622      -0.264 -19.786  36.968  1.00103.33
C
ANISOU 4628  CB  GLU A 622    10820  18074  10368  -2022     92
-1665       C
ATOM   4629  CG  GLU A 622       0.827 -20.768  37.326  1.00145.99
C
ANISOU 4629  CG  GLU A 622    16412  23456  15601  -2025    125
-1643       C
ATOM   4630  CD  GLU A 622       0.371 -22.205  37.151  1.00129.89
C
ANISOU 4630  CD  GLU A 622    14472  21356  13526  -2174     92
-1482       C
ATOM   4631  OE1 GLU A 622       0.334 -22.683  35.996  1.00101.99
O
ANISOU 4631  OE1 GLU A 622    11086  17645  10019  -2267    -43
-1424       O
ATOM   4632  OE2 GLU A 622       0.039 -22.854  38.164  1.00119.82
O
ANISOU 4632  OE2 GLU A 622    13151  20193  12183  -2199    199
-1411       O
ATOM   4633  N   PRO A 623      -0.913 -19.092  40.197  1.00111.65
N
ANISOU 4633  N   PRO A 623    11598  19549  11274  -1780    462
-1723       N
ATOM   4634  CA  PRO A 623      -1.079 -18.175  41.329  1.00 71.86
```

```
C
ANISOU 4634  CA  PRO A 623     6487  14644   6174  -1605    596
-1814       C
ATOM   4635  C   PRO A 623       0.134 -17.296  41.592  1.00 70.88
C
ANISOU 4635  C   PRO A 623     6506  14478   5947  -1502    567
-2000       C
ATOM   4636  O   PRO A 623       1.280 -17.651  41.309  1.00 69.61
O
ANISOU 4636  O   PRO A 623     6479  14249   5720  -1550    490
-2037       O
ATOM   4637  CB  PRO A 623      -1.300 -19.135  42.508  1.00 73.02
C
ANISOU 4637  CB  PRO A 623     6617  14923   6202  -1601    741
-1712       C
ATOM   4638  CG  PRO A 623      -0.507 -20.334  42.131  1.00 71.94
C
ANISOU 4638  CG  PRO A 623     6641  14696   5996  -1729    666
-1655       C
ATOM   4639  CD  PRO A 623      -0.694 -20.478  40.646  1.00 71.13
C
ANISOU 4639  CD  PRO A 623     6554  14436   6035  -1869    503
-1610       C
ATOM   4640  N   GLY A 624      -0.147 -16.129  42.152  1.00 71.78
N
ANISOU 4640  N   GLY A 624     6592  14637   6044  -1354    628
-2105       N
ATOM   4641  CA  GLY A 624       0.839 -15.254  42.748  1.00 73.93
C
ANISOU 4641  CA  GLY A 624     7005  14890   6193  -1260    612
-2278       C
ATOM   4642  C   GLY A 624       1.004 -13.949  41.978  1.00 71.25
C
ANISOU 4642  C   GLY A 624     6715  14420   5938  -1234    521
-2402       C
ATOM   4643  O   GLY A 624       0.669 -13.834  40.795  1.00 70.57
O
ANISOU 4643  O   GLY A 624     6581  14236   5998  -1300    445
-2361       O
ATOM   4644  N   LEU A 625       1.532 -12.949  42.689  1.00 71.92
N
ANISOU 4644  N   LEU A 625     6924  14490   5912  -1137    522
-2555       N
ATOM   4645  CA  LEU A 625       1.842 -11.655  42.086  1.00 72.69
C
ANISOU 4645  CA  LEU A 625     7119  14441   6059  -1124    435
-2686       C
ATOM   4646  C   LEU A 625       2.852 -11.797  40.951  1.00110.24
C
ANISOU 4646  C   LEU A 625    11908  19077  10899  -1284    297
-2688       C
ATOM   4647  O   LEU A 625       2.795 -11.063  39.958  1.00184.20
O
ANISOU 4647  O   LEU A 625    21303  28308  20375  -1305    233
```

```
-2721       O
ATOM   4648  CB  LEU A 625       2.368 -10.701  43.158  1.00 77.17
C
ANISOU 4648  CB  LEU A 625     7861  14998   6462  -1028    438
-2848       C
ATOM   4649  CG  LEU A 625       2.627  -9.259  42.731  1.00 73.56
C
ANISOU 4649  CG  LEU A 625     7555  14370   6025  -1010    358
-2993       C
ATOM   4650  CD1 LEU A 625       1.308  -8.564  42.487  1.00 74.65
C
ANISOU 4650  CD1 LEU A 625     7664  14480   6221   -841    453
-2975       C
ATOM   4651  CD2 LEU A 625       3.444  -8.516  43.775  1.00 74.87
C
ANISOU 4651  CD2 LEU A 625     7932  14505   6010   -987    305
-3152       C
ATOM   4652  N   SER A 626       3.795 -12.730  41.102  1.00 69.27
N
ANISOU 4652  N   SER A 626     6728  13943   5649  -1374    262
-2642       N
ATOM   4653  CA  SER A 626       4.807 -13.002  40.083  1.00 76.78
C
ANISOU 4653  CA  SER A 626     7705  14810   6659  -1493    161
-2611       C
ATOM   4654  C   SER A 626       4.202 -13.327  38.720  1.00111.81
C
ANISOU 4654  C   SER A 626    12101  19138  11244  -1540    132
-2512       C
ATOM   4655  O   SER A 626       4.710 -12.879  37.686  1.00113.22
O
ANISOU 4655  O   SER A 626    12332  19187  11501  -1589     58
-2527       O
ATOM   4656  CB  SER A 626       5.705 -14.150  40.548  1.00 69.92
C
ANISOU 4656  CB  SER A 626     6839  14050   5679  -1531    161
-2541       C
ATOM   4657  OG  SER A 626       6.672 -14.475  39.565  1.00126.46
O
ANISOU 4657  OG  SER A 626    14022  21146  12882  -1609     89
-2484       O
ATOM   4658  N   VAL A 627       3.125 -14.112  38.698  1.00109.11
N
ANISOU 4658  N   VAL A 627    11674  18846  10939  -1533    183
-2402       N
ATOM   4659  CA  VAL A 627       2.406 -14.383  37.454  1.00 75.84
C
ANISOU 4659  CA  VAL A 627     7427  14528   6860  -1585    126
-2311       C
ATOM   4660  C   VAL A 627       1.844 -13.110  36.823  1.00 67.10
C
ANISOU 4660  C   VAL A 627     6314  13324   5859  -1524     95
-2386       C
ATOM   4661  O   VAL A 627       1.946 -12.914  35.606  1.00 66.39
```

```
O
ANISOU 4661  O   VAL A 627     6278  13091   5857  -1563       9
-2371       O
ATOM   4662  CB  VAL A 627       1.289 -15.412  37.716  1.00 76.75
C
ANISOU 4662  CB  VAL A 627     7431  14735   6995  -1618     174
-2171       C
ATOM   4663  CG1 VAL A 627       0.354 -15.510  36.525  1.00 67.55
C
ANISOU 4663  CG1 VAL A 627     6213  13477   5975  -1677      88
-2086       C
ATOM   4664  CG2 VAL A 627       1.885 -16.773  38.046  1.00101.25
C
ANISOU 4664  CG2 VAL A 627    10599  17880   9992  -1690     189
-2082       C
ATOM   4665  N   ILE A 628       1.253 -12.225  37.625  1.00 68.37
N
ANISOU 4665  N   ILE A 628     6436  13547   5994  -1406     169
-2463       N
ATOM   4666  CA  ILE A 628       0.720 -10.971  37.086  1.00 68.96
C
ANISOU 4666  CA  ILE A 628     6536  13524   6144  -1314     152
-2536       C
ATOM   4667  C   ILE A 628       1.809 -10.093  36.469  1.00 94.15
C
ANISOU 4667  C   ILE A 628     9889  16545   9337  -1354      74
-2649       C
ATOM   4668  O   ILE A 628       1.643  -9.569  35.362  1.00 90.33
O
ANISOU 4668  O   ILE A 628     9450  15921   8948  -1352      14
-2651       O
ATOM   4669  CB  ILE A 628      -0.063 -10.210  38.171  1.00 70.77
C
ANISOU 4669  CB  ILE A 628     6733  13850   6304  -1140     269
-2593       C
ATOM   4670  CG1 ILE A 628      -1.176 -11.090  38.732  1.00100.86
C
ANISOU 4670  CG1 ILE A 628    10349  17845  10130  -1105     366
-2448       C
ATOM   4671  CG2 ILE A 628      -0.667  -8.948  37.594  1.00 71.59
C
ANISOU 4671  CG2 ILE A 628     6885  13848   6468  -1014     260
-2656       C
ATOM   4672  CD1 ILE A 628      -1.940 -10.450  39.861  1.00128.07
C
ANISOU 4672  CD1 ILE A 628    13762  21410  13489   -900     517
-2473       C
ATOM   4673  N   ILE A 629       2.940  -9.921  37.160  1.00 68.16
N
ANISOU 4673  N   ILE A 629     6685  13268   5944  -1399      68
-2731       N
ATOM   4674  CA  ILE A 629       4.036  -9.128  36.594  1.00 67.78
C
ANISOU 4674  CA  ILE A 629     6764  13076   5912  -1474      -8
```

```
-2812       C
ATOM   4675  C   ILE A 629       4.599  -9.753  35.322  1.00 66.90
C
ANISOU 4675  C   ILE A 629     6652  12882   5884  -1570    -67
-2711       C
ATOM   4676  O   ILE A 629       4.877  -9.048  34.344  1.00 66.14
O
ANISOU 4676  O   ILE A 629     6640  12629   5862  -1590   -111
-2732       O
ATOM   4677  CB  ILE A 629       5.135  -8.892  37.650  1.00 68.36
C
ANISOU 4677  CB  ILE A 629     6901  13211   5861  -1528    -27
-2901       C
ATOM   4678  CG1 ILE A 629       4.866  -7.598  38.422  1.00 70.03
C
ANISOU 4678  CG1 ILE A 629     7246  13366   5997  -1444    -16
-3053       C
ATOM   4679  CG2 ILE A 629       6.519  -8.852  37.015  1.00 67.68
C
ANISOU 4679  CG2 ILE A 629     6847  13064   5806  -1669   -107
-2887       C
ATOM   4680  CD1 ILE A 629       3.521  -7.523  39.108  1.00 79.73
C
ANISOU 4680  CD1 ILE A 629     8444  14672   7179  -1261     93
-3054       C
ATOM   4681  N   SER A 630       4.778 -11.074  35.306  1.00 65.58
N
ANISOU 4681  N   SER A 630     6423  12803   5691  -1614    -61
-2595       N
ATOM   4682  CA  SER A 630       5.204 -11.735  34.075  1.00 64.48
C
ANISOU 4682  CA  SER A 630     6328  12569   5604  -1667   -107
-2489       C
ATOM   4683  C   SER A 630       4.216 -11.510  32.935  1.00 96.53
C
ANISOU 4683  C   SER A 630    10411  16493   9773  -1634   -151
-2455       C
ATOM   4684  O   SER A 630       4.619 -11.227  31.801  1.00 73.44
O
ANISOU 4684  O   SER A 630     7585  13419   6902  -1645   -195
-2435       O
ATOM   4685  CB  SER A 630       5.402 -13.231  34.323  1.00 63.98
C
ANISOU 4685  CB  SER A 630     6238  12605   5466  -1696    -89
-2370       C
ATOM   4686  OG  SER A 630       6.344 -13.455  35.357  1.00111.39
O
ANISOU 4686  OG  SER A 630    12219  18745  11358  -1707    -57
-2396       O
ATOM   4687  N   ALA A 631       2.917 -11.630  33.219  1.00119.48
N
ANISOU 4687  N   ALA A 631    13222  19462  12714  -1587   -138
-2435       N
ATOM   4688  CA  ALA A 631       1.889 -11.303  32.233  1.00 65.37
```

```
C
ANISOU 4688  CA  ALA A 631     6361  12510   5965  -1546   -197
-2404       C
ATOM   4689  C   ALA A 631       1.919  -9.842  31.790  1.00 65.77
C
ANISOU 4689  C   ALA A 631     6497  12432   6060  -1467   -203
-2513       C
ATOM   4690  O   ALA A 631       1.796  -9.554  30.594  1.00133.76
O
ANISOU 4690  O   ALA A 631    15193  20892  14738  -1452   -269
-2492       O
ATOM   4691  CB  ALA A 631       0.512 -11.650  32.797  1.00 66.48
C
ANISOU 4691  CB  ALA A 631     6330  12793   6136  -1510   -171
-2344       C
ATOM   4692  N   VAL A 632       2.074  -8.905  32.729  1.00 66.58
N
ANISOU 4692  N   VAL A 632     6614  12573   6109  -1409   -139
-2630       N
ATOM   4693  CA  VAL A 632       2.263  -7.497  32.365  1.00108.86
C
ANISOU 4693  CA  VAL A 632    12107  17774  11481  -1351   -144
-2740       C
ATOM   4694  C   VAL A 632       3.501  -7.297  31.493  1.00101.77
C
ANISOU 4694  C   VAL A 632    11339  16723  10604  -1452   -188
-2742       C
ATOM   4695  O   VAL A 632       3.449  -6.620  30.459  1.00 79.05
O
ANISOU 4695  O   VAL A 632     8573  13677   7787  -1421   -218
-2751       O
ATOM   4696  CB  VAL A 632       2.328  -6.619  33.630  1.00 94.76
C
ANISOU 4696  CB  VAL A 632    10368  16036   9599  -1287    -79
-2868       C
ATOM   4697  CG1 VAL A 632       2.753  -5.194  33.277  1.00 69.13
C
ANISOU 4697  CG1 VAL A 632     7322  12595   6350  -1268    -97
-2986       C
ATOM   4698  CG2 VAL A 632       0.984  -6.604  34.340  1.00102.09
C
ANISOU 4698  CG2 VAL A 632    11183  17099  10507  -1130     -5
-2851       C
ATOM   4699  N   LEU A 633       4.628  -7.887  31.890  1.00 89.96
N
ANISOU 4699  N   LEU A 633     9828  15297   9058  -1561   -182
-2718       N
ATOM   4700  CA  LEU A 633       5.849  -7.793  31.091  1.00102.01
C
ANISOU 4700  CA  LEU A 633    11436  16718  10606  -1649   -202
-2681       C
ATOM   4701  C   LEU A 633       5.699  -8.409  29.701  1.00 96.56
C
ANISOU 4701  C   LEU A 633    10799  15918   9972  -1625   -232
```

```
-2564       C
ATOM   4702  O   LEU A 633       6.103  -7.809  28.699  1.00105.76
O
ANISOU 4702  O   LEU A 633    12080  16919  11184  -1623   -239
-2554       O
ATOM   4703  CB  LEU A 633       7.004  -8.450  31.848  1.00128.38
C
ANISOU 4703  CB  LEU A 633    14706  20202  13872  -1743   -189
-2649       C
ATOM   4704  CG  LEU A 633       8.394  -8.356  31.218  1.00108.67
C
ANISOU 4704  CG  LEU A 633    12240  17656  11393  -1831   -191
-2586       C
ATOM   4705  CD1 LEU A 633       8.789  -6.907  31.021  1.00102.07
C
ANISOU 4705  CD1 LEU A 633    11500  16677  10605  -1892   -208
-2679       C
ATOM   4706  CD2 LEU A 633       9.416  -9.081  32.079  1.00122.97
C
ANISOU 4706  CD2 LEU A 633    13945  19656  13122  -1896   -184
-2541       C
ATOM   4707  N   THR A 634       5.128  -9.611  29.622  1.00 89.12
N
ANISOU 4707  N   THR A 634     9799  15050   9015  -1611   -252
-2471       N
ATOM   4708  CA  THR A 634       4.824 -10.217  28.326  1.00 79.30
C
ANISOU 4708  CA  THR A 634     8648  13679   7802  -1584   -309
-2369       C
ATOM   4709  C   THR A 634       3.823  -9.408  27.501  1.00 80.98
C
ANISOU 4709  C   THR A 634     8918  13756   8093  -1505   -365
-2404       C
ATOM   4710  O   THR A 634       4.005  -9.240  26.289  1.00106.64
O
ANISOU 4710  O   THR A 634    12315  16835  11368  -1471   -401
-2365       O
ATOM   4711  CB  THR A 634       4.301 -11.641  28.543  1.00 75.17
C
ANISOU 4711  CB  THR A 634     8072  13252   7237  -1612   -341
-2271       C
ATOM   4712  OG1 THR A 634       5.383 -12.496  28.931  1.00 76.51
O
ANISOU 4712  OG1 THR A 634     8255  13501   7316  -1652   -292
-2210       O
ATOM   4713  CG2 THR A 634       3.669 -12.191  27.276  1.00 85.28
C
ANISOU 4713  CG2 THR A 634     9472  14387   8545  -1594   -441
-2183       C
ATOM   4714  N   SER A 635       2.773  -8.885  28.135  1.00 65.20
N
ANISOU 4714  N   SER A 635     6814  11834   6123  -1450   -364
-2469       N
ATOM   4715  CA  SER A 635       1.808  -8.045  27.423  1.00 65.30
```

```
C
ANISOU 4715  CA  SER A 635     6866  11743   6201  -1343   -411
-2498       C
ATOM   4716  C   SER A 635       2.421  -6.791  26.800  1.00 65.50
C
ANISOU 4716  C   SER A 635     7067  11580   6240  -1300   -385
-2575       C
ATOM   4717  O   SER A 635       2.075  -6.428  25.670  1.00112.60
O
ANISOU 4717  O   SER A 635    13148  17391  12244  -1228   -439
-2555       O
ATOM   4718  CB  SER A 635       0.675  -7.654  28.371  1.00 99.30
C
ANISOU 4718  CB  SER A 635    11014  16197  10517  -1258   -378
-2541       C
ATOM   4719  OG  SER A 635      -0.369  -6.996  27.678  1.00106.69
O
ANISOU 4719  OG  SER A 635    11954  17070  11512  -1131   -430
-2541       O
ATOM   4720  N   ARG A 636       3.323  -6.110  27.509  1.00 65.69
N
ANISOU 4720  N   ARG A 636     7127  11606   6227  -1352   -314
-2659       N
ATOM   4721  CA  ARG A 636       4.083  -5.022  26.888  1.00 83.94
C
ANISOU 4721  CA  ARG A 636     9615  13726   8554  -1363   -290
-2709       C
ATOM   4722  C   ARG A 636       4.909  -5.462  25.680  1.00107.87
C
ANISOU 4722  C   ARG A 636    12748  16632  11604  -1403   -298
-2603       C
ATOM   4723  O   ARG A 636       4.971  -4.740  24.677  1.00128.57
O
ANISOU 4723  O   ARG A 636    15530  19063  14256  -1351   -297
-2602       O
ATOM   4724  CB  ARG A 636       4.966  -4.325  27.926  1.00 66.67
C
ANISOU 4724  CB  ARG A 636     7441  11570   6322  -1460   -241
-2805       C
ATOM   4725  CG  ARG A 636       4.159  -3.505  28.928  1.00101.24
C
ANISOU 4725  CG  ARG A 636    11826  15986  10654  -1370   -222
-2929       C
ATOM   4726  CD  ARG A 636       5.033  -2.586  29.777  1.00 68.99
C
ANISOU 4726  CD  ARG A 636     7846  11859   6510  -1470   -206
-3041       C
ATOM   4727  NE  ARG A 636       4.264  -1.945  30.842  1.00 70.34
N
ANISOU 4727  NE  ARG A 636     8058  12069   6597  -1356   -180
-3155       N
ATOM   4728  CZ  ARG A 636       4.711  -1.715  32.072  1.00 71.21
C
ANISOU 4728  CZ  ARG A 636     8194  12249   6613  -1419   -178
```

```
-3243       C
ATOM   4729  NH1 ARG A 636       5.997  -1.859  32.354  1.00 94.83
N
ANISOU 4729  NH1 ARG A 636    11175  15261   9595  -1620   -220
-3241       N
ATOM   4730  NH2 ARG A 636       3.908  -1.153  32.967  1.00 72.59
N
ANISOU 4730  NH2 ARG A 636     8442  12447   6693  -1268   -139
-3335       N
ATOM   4731  N   ALA A 637       5.556  -6.625  25.748  1.00112.08
N
ANISOU 4731  N   ALA A 637    13214  17265  12106  -1473   -291
-2506       N
ATOM   4732  CA  ALA A 637       6.274  -7.139  24.581  1.00106.80
C
ANISOU 4732  CA  ALA A 637    12666  16482  11432  -1465   -281
-2386       C
ATOM   4733  C   ALA A 637       5.363  -7.400  23.380  1.00108.49
C
ANISOU 4733  C   ALA A 637    13011  16552  11658  -1354   -362
-2336       C
ATOM   4734  O   ALA A 637       5.731  -7.092  22.240  1.00123.95
O
ANISOU 4734  O   ALA A 637    15146  18330  13620  -1298   -349
-2287       O
ATOM   4735  CB  ALA A 637       7.027  -8.415  24.960  1.00105.10
C
ANISOU 4735  CB  ALA A 637    12371  16410  11152  -1520   -255
-2285       C
ATOM   4736  N   ILE A 638       4.178  -7.965  23.608  1.00 95.91
N
ANISOU 4736  N   ILE A 638    11333  15039  10071  -1327   -452
-2338       N
ATOM   4737  CA  ILE A 638       3.158  -8.045  22.559  1.00 92.71
C
ANISOU 4737  CA  ILE A 638    11026  14511   9688  -1237   -568
-2305       C
ATOM   4738  C   ILE A 638       2.696  -6.662  22.102  1.00 96.84
C
ANISOU 4738  C   ILE A 638    11631  14906  10259  -1131   -567
-2388       C
ATOM   4739  O   ILE A 638       2.571  -6.395  20.901  1.00110.95
O
ANISOU 4739  O   ILE A 638    13602  16508  12045  -1044   -616
-2357       O
ATOM   4740  CB  ILE A 638       1.971  -8.898  23.043  1.00 82.18
C
ANISOU 4740  CB  ILE A 638     9530  13327   8368  -1265   -669
-2275       C
ATOM   4741  CG1 ILE A 638       2.427 -10.333  23.314  1.00 87.69
C
ANISOU 4741  CG1 ILE A 638    10219  14100   8999  -1363   -677
-2182       C
ATOM   4742  CG2 ILE A 638       0.847  -8.879  22.023  1.00 86.29
```

```
C
ANISOU 4742  CG2 ILE A 638    10116  13747   8923  -1189   -820
-2241       C
ATOM   4743  CD1 ILE A 638       3.030 -11.026  22.110  1.00100.21
C
ANISOU 4743  CD1 ILE A 638    12055  15507  10515  -1336   -718
-2084       C
ATOM   4744  N   PHE A 639       2.434  -5.765  23.052  1.00 95.59
N
ANISOU 4744  N   PHE A 639    11370  14829  10122  -1119   -510
-2493       N
ATOM   4745  CA  PHE A 639       1.963  -4.420  22.724  1.00 79.53
C
ANISOU 4745  CA  PHE A 639     9440  12667   8110   -998   -499
-2576       C
ATOM   4746  C   PHE A 639       2.963  -3.618  21.891  1.00103.23
C
ANISOU 4746  C   PHE A 639    12672  15445  11106   -999   -434
-2583       C
ATOM   4747  O   PHE A 639       2.561  -2.822  21.034  1.00147.97
O
ANISOU 4747  O   PHE A 639    18502  20940  16779   -877   -453
-2602       O
ATOM   4748  CB  PHE A 639       1.624  -3.677  24.013  1.00 77.34
C
ANISOU 4748  CB  PHE A 639     9056  12509   7822   -977   -434
-2685       C
ATOM   4749  CG  PHE A 639       1.065  -2.302  23.797  1.00 88.88
C
ANISOU 4749  CG  PHE A 639    10650  13840   9279   -823   -413
-2771       C
ATOM   4750  CD1 PHE A 639      -0.226  -2.132  23.317  1.00 69.71
C
ANISOU 4750  CD1 PHE A 639     8188  11426   6873   -650   -484
-2749       C
ATOM   4751  CD2 PHE A 639       1.822  -1.178  24.094  1.00100.45
C
ANISOU 4751  CD2 PHE A 639    12281  15173  10711   -853   -330
-2868       C
ATOM   4752  CE1 PHE A 639      -0.750  -0.866  23.129  1.00 71.13
C
ANISOU 4752  CE1 PHE A 639     8506  11490   7030   -472   -454
-2823       C
ATOM   4753  CE2 PHE A 639       1.306   0.090  23.909  1.00 97.54
C
ANISOU 4753  CE2 PHE A 639    12084  14660  10316   -699   -305
-2949       C
ATOM   4754  CZ  PHE A 639       0.020   0.247  23.426  1.00 71.68
C
ANISOU 4754  CZ  PHE A 639     8783  11401   7050   -490   -357
-2927       C
ATOM   4755  N   GLN A 640       4.263  -3.797  22.137  1.00 92.37
N
ANISOU 4755  N   GLN A 640    11301  14077   9717  -1133   -353
```

```
-2555       N
ATOM   4756  CA  GLN A 640       5.290  -3.092  21.366  1.00108.66
C
ANISOU 4756  CA  GLN A 640    13548  15951  11787  -1159   -274
-2528       C
ATOM   4757  C   GLN A 640       5.156  -3.250  19.851  1.00120.95
C
ANISOU 4757  C   GLN A 640    15303  17318  13334  -1039   -302
-2439       C
ATOM   4758  O   GLN A 640       5.293  -2.268  19.111  1.00110.93
O
ANISOU 4758  O   GLN A 640    14225  15849  12073   -976   -260
-2454       O
ATOM   4759  CB  GLN A 640       6.679  -3.557  21.804  1.00116.44
C
ANISOU 4759  CB  GLN A 640    14445  17030  12765  -1318   -195
-2462       C
ATOM   4760  CG  GLN A 640       7.307  -2.709  22.892  1.00152.42
C
ANISOU 4760  CG  GLN A 640    18938  21642  17334  -1458   -150
-2555       C
ATOM   4761  CD  GLN A 640       8.139  -1.575  22.324  1.00111.35
C
ANISOU 4761  CD  GLN A 640    13897  16244  12165  -1525    -80
-2550       C
ATOM   4762  OE1 GLN A 640       7.603  -0.611  21.774  1.00 73.29
O
ANISOU 4762  OE1 GLN A 640     9263  11232   7351  -1436    -80
-2609       O
ATOM   4763  NE2 GLN A 640       9.458  -1.689  22.444  1.00 83.04
N
ANISOU 4763  NE2 GLN A 640    10238  12714   8599  -1681    -17
-2464       N
ATOM   4764  N   ARG A 641       4.890  -4.461  19.362  1.00 82.16
N
ANISOU 4764  N   ARG A 641    10384  12443   8389  -1002   -377
-2345       N
ATOM   4765  CA  ARG A 641       4.853  -4.654  17.914  1.00 65.89
C
ANISOU 4765  CA  ARG A 641     8560  10183   6291   -881   -411
-2259       C
ATOM   4766  C   ARG A 641       3.590  -4.122  17.245  1.00 75.49
C
ANISOU 4766  C   ARG A 641     9879  11287   7515   -733   -532
-2309       C
ATOM   4767  O   ARG A 641       3.620  -3.848  16.041  1.00136.18
O
ANISOU 4767  O   ARG A 641    17806  18768  15167   -615   -544
-2265       O
ATOM   4768  CB  ARG A 641       5.015  -6.138  17.578  1.00 74.30
C
ANISOU 4768  CB  ARG A 641     9648  11293   7290   -886   -466
-2143       C
ATOM   4769  CG  ARG A 641       3.807  -6.986  17.919  1.00 91.42
```

```
C
ANISOU 4769  CG  ARG A 641    11700  13580   9457   -898   -633
-2157       C
ATOM   4770  CD  ARG A 641       4.198  -8.438  18.126  1.00117.36
C
ANISOU 4770  CD  ARG A 641    14966  16953  12672   -967   -652
-2062       C
ATOM   4771  NE  ARG A 641       4.975  -8.621  19.348  1.00116.00
N
ANISOU 4771  NE  ARG A 641    14590  16975  12511  -1084   -535
-2075       N
ATOM   4772  CZ  ARG A 641       5.673  -9.713  19.640  1.00102.02
C
ANISOU 4772  CZ  ARG A 641    12806  15290  10669  -1129   -493
-1989       C
ATOM   4773  NH1 ARG A 641       5.544 -10.803  18.898  1.00105.75
N
ANISOU 4773  NH1 ARG A 641    13463  15670  11048  -1078   -571
-1891       N
ATOM   4774  NH2 ARG A 641       6.367  -9.770  20.770  1.00123.06
N
ANISOU 4774  NH2 ARG A 641    15277  18137  13341  -1223   -397
-2007       N
ATOM   4775  N   MET A 642       2.491  -3.964  17.983  1.00 67.02
N
ANISOU 4775  N   MET A 642     8631  10354   6479   -718   -614
-2387       N
ATOM   4776  CA  MET A 642       1.331  -3.260  17.439  1.00 68.40
C
ANISOU 4776  CA  MET A 642     8872  10452   6666   -556   -711
-2430       C
ATOM   4777  C   MET A 642       1.640  -1.796  17.137  1.00106.17
C
ANISOU 4777  C   MET A 642    13843  15051  11447   -468   -604
-2501       C
ATOM   4778  O   MET A 642       1.255  -1.279  16.081  1.00142.33
O
ANISOU 4778  O   MET A 642    18628  19450  16002   -314   -649
-2491       O
ATOM   4779  CB  MET A 642       0.138  -3.384  18.389  1.00 90.01
C
ANISOU 4779  CB  MET A 642    11343  13412   9444   -544   -789
-2473       C
ATOM   4780  CG  MET A 642      -0.225  -4.829  18.720  1.00109.96
C
ANISOU 4780  CG  MET A 642    13688  16114  11978   -657   -895
-2394       C
ATOM   4781  SD  MET A 642      -1.787  -5.022  19.610  1.00 69.14
S
ANISOU 4781  SD  MET A 642     8190  11209   6869   -632   -993
-2395       S
ATOM   4782  CE  MET A 642      -2.959  -4.821  18.273  1.00 91.55
C
ANISOU 4782  CE  MET A 642    11120  13945   9720   -473  -1196
```

```
-2345       C
ATOM   4783  N   LYS A 643       2.327  -1.113  18.052  1.00 69.05
N
ANISOU 4783  N   LYS A 643     9096  10379   6761   -567   -474
-2572       N
ATOM   4784  CA  LYS A 643       2.825   0.233  17.773  1.00 95.73
C
ANISOU 4784  CA  LYS A 643    12691  13550  10130   -535   -368
-2629       C
ATOM   4785  C   LYS A 643       3.827   0.268  16.620  1.00 99.69
C
ANISOU 4785  C   LYS A 643    13414  13845  10619   -549   -294
-2533       C
ATOM   4786  O   LYS A 643       3.714   1.103  15.717  1.00 95.07
O
ANISOU 4786  O   LYS A 643    13073  13040  10009   -424   -269
-2535       O
ATOM   4787  CB  LYS A 643       3.467   0.825  19.029  1.00 96.00
C
ANISOU 4787  CB  LYS A 643    12645  13654  10175   -688   -272
-2716       C
ATOM   4788  CG  LYS A 643       2.515   1.080  20.180  1.00 83.96
C
ANISOU 4788  CG  LYS A 643    10972  12291   8636   -629   -304
-2819       C
ATOM   4789  CD  LYS A 643       3.275   1.551  21.412  1.00 78.38
C
ANISOU 4789  CD  LYS A 643    10229  11636   7915   -792   -228
-2903       C
ATOM   4790  CE  LYS A 643       3.909   2.919  21.184  1.00 95.80
C
ANISOU 4790  CE  LYS A 643    12709  13591  10100   -829   -152
-2966       C
ATOM   4791  NZ  LYS A 643       2.918   4.026  21.311  1.00 74.19
N
ANISOU 4791  NZ  LYS A 643    10143  10746   7301   -624   -149
-3071       N
ATOM   4792  N   ASN A 644       4.825  -0.620  16.638  1.00 69.36
N
ANISOU 4792  N   ASN A 644     9499  10074   6781   -681   -243
-2435       N
ATOM   4793  CA  ASN A 644       5.829  -0.627  15.571  1.00121.60
C
ANISOU 4793  CA  ASN A 644    16308  16516  13377   -672   -142
-2315       C
ATOM   4794  C   ASN A 644       5.248  -0.929  14.191  1.00107.44
C
ANISOU 4794  C   ASN A 644    14746  14554  11521   -469   -219
-2250       C
ATOM   4795  O   ASN A 644       5.644  -0.297  13.204  1.00 79.43
O
ANISOU 4795  O   ASN A 644    11448  10785   7947   -381   -136
-2200       O
ATOM   4796  CB  ASN A 644       6.943  -1.622  15.902  1.00106.63
```

```
C
ANISOU 4796  CB  ASN A 644    14267  14765  11482   -811    -69
-2205       C
ATOM   4797  CG  ASN A 644       7.716  -1.241  17.150  1.00122.44
C
ANISOU 4797  CG  ASN A 644    16069  16914  13539  -1020      4
-2253       C
ATOM   4798  OD1 ASN A 644       7.699  -0.085  17.576  1.00 93.39
O
ANISOU 4798  OD1 ASN A 644    12430  13162   9891  -1081     31
-2353       O
ATOM   4799  ND2 ASN A 644       8.409  -2.210  17.736  1.00123.43
N
ANISOU 4799  ND2 ASN A 644    16004  17235  13661  -1126     27
-2182       N
ATOM   4800  N   TYR A 645       4.322  -1.885  14.084  1.00 68.83
N
ANISOU 4800  N   TYR A 645     9794   9757   6602   -401   -385
-2244       N
ATOM   4801  CA  TYR A 645       3.613  -2.048  12.816  1.00 69.39
C
ANISOU 4801  CA  TYR A 645    10100   9659   6607   -212   -507
-2204       C
ATOM   4802  C   TYR A 645       2.802  -0.813  12.440  1.00 70.63
C
ANISOU 4802  C   TYR A 645    10385   9682   6768    -60   -541
-2290       C
ATOM   4803  O   TYR A 645       2.780  -0.415  11.269  1.00 80.19
O
ANISOU 4803  O   TYR A 645    11881  10669   7918     97   -542
-2251       O
ATOM   4804  CB  TYR A 645       2.705  -3.275  12.841  1.00 93.75
C
ANISOU 4804  CB  TYR A 645    13084  12871   9667   -209   -714
-2181       C
ATOM   4805  CG  TYR A 645       2.098  -3.532  11.479  1.00 92.74
C
ANISOU 4805  CG  TYR A 645    13229  12555   9453    -37   -868
-2130       C
ATOM   4806  CD1 TYR A 645       2.845  -4.114  10.464  1.00102.16
C
ANISOU 4806  CD1 TYR A 645    14702  13572  10541     32   -828
-2019       C
ATOM   4807  CD2 TYR A 645       0.802  -3.125  11.186  1.00 70.84
C
ANISOU 4807  CD2 TYR A 645    10450   9775   6690     81  -1048
-2186       C
ATOM   4808  CE1 TYR A 645       2.286  -4.378   9.231  1.00163.74
C
ANISOU 4808  CE1 TYR A 645    22792  21183  18239    196   -988
-1978       C
ATOM   4809  CE2 TYR A 645       0.252  -3.339   9.938  1.00 71.81
C
ANISOU 4809  CE2 TYR A 645    10832   9726   6727    235  -1216
```

```
-2141       C
ATOM   4810  CZ  TYR A 645       0.992  -3.978   8.968  1.00152.38
C
ANISOU 4810  CZ  TYR A 645    21339  19742  16818    286  -1194
-2045       C
ATOM   4811  OH  TYR A 645       0.460  -4.141   7.712  1.00116.26
O
ANISOU 4811  OH  TYR A 645    17068  14972  12135    453  -1370
-2008       O
ATOM   4812  N   THR A 646       2.116  -0.203  13.406  1.00 70.96
N
ANISOU 4812  N   THR A 646    10243   9854   6863    -77   -564
-2400       N
ATOM   4813  CA  THR A 646       1.371   1.018  13.115  1.00100.06
C
ANISOU 4813  CA  THR A 646    14066  13416  10535     98   -576
-2478       C
ATOM   4814  C   THR A 646       2.285   2.136  12.624  1.00 99.58
C
ANISOU 4814  C   THR A 646    14280  13104  10454    110   -399
-2481       C
ATOM   4815  O   THR A 646       1.933   2.867  11.691  1.00 74.25
O
ANISOU 4815  O   THR A 646    11331   9688   7193    296   -406
-2483       O
ATOM   4816  CB  THR A 646       0.584   1.468  14.344  1.00124.91
C
ANISOU 4816  CB  THR A 646    16983  16754  13723     96   -595
-2584       C
ATOM   4817  OG1 THR A 646      -0.289   0.411  14.760  1.00 80.94
O
ANISOU 4817  OG1 THR A 646    11145  11425   8183     75   -750
-2556       O
ATOM   4818  CG2 THR A 646      -0.256   2.689  14.003  1.00139.83
C
ANISOU 4818  CG2 THR A 646    19035  18520  15575    325   -606
-2652       C
ATOM   4819  N   ILE A 647       3.459   2.288  13.238  1.00 72.71
N
ANISOU 4819  N   ILE A 647    10821   9716   7090    -93   -245
-2471       N
ATOM   4820  CA  ILE A 647       4.470   3.189  12.686  1.00 80.62
C
ANISOU 4820  CA  ILE A 647    12063  10484   8086   -131    -76
-2431       C
ATOM   4821  C   ILE A 647       4.844   2.778  11.265  1.00102.15
C
ANISOU 4821  C   ILE A 647    15022  13036  10756    -10    -47
-2296       C
ATOM   4822  O   ILE A 647       4.885   3.609  10.351  1.00 74.89
O
ANISOU 4822  O   ILE A 647    11861   9336   7258    121     20
-2277       O
ATOM   4823  CB  ILE A 647       5.712   3.217  13.596  1.00 85.18
```

```
C
ANISOU 4823  CB  ILE A 647    12483  11155   8728   -405     53
-2413       C
ATOM   4824  CG1 ILE A 647       5.364   3.737  14.990  1.00113.17
C
ANISOU 4824  CG1 ILE A 647    15868  14832  12301   -509     23
-2558       C
ATOM   4825  CG2 ILE A 647       6.819   4.052  12.967  1.00 74.56
C
ANISOU 4825  CG2 ILE A 647    11351   9584   7393   -482    227
-2334       C
ATOM   4826  CD1 ILE A 647       6.453   3.488  16.018  1.00 83.38
C
ANISOU 4826  CD1 ILE A 647    11887  11212   8582   -783     88
-2544       C
ATOM   4827  N   TYR A 648       5.107   1.486  11.059  1.00 75.81
N
ANISOU 4827  N   TYR A 648    11592   9810   7401    -35    -91
-2199       N
ATOM   4828  CA  TYR A 648       5.428   0.980   9.726  1.00 72.78
C
ANISOU 4828  CA  TYR A 648    11463   9259   6932    111    -70
-2066       C
ATOM   4829  C   TYR A 648       4.301   1.219   8.725  1.00105.78
C
ANISOU 4829  C   TYR A 648    15886  13275  11029    361   -224
-2100       C
ATOM   4830  O   TYR A 648       4.534   1.740   7.628  1.00 93.41
O
ANISOU 4830  O   TYR A 648    14635  11463   9393    514   -149
-2041       O
ATOM   4831  CB  TYR A 648       5.766  -0.509   9.806  1.00 71.60
C
ANISOU 4831  CB  TYR A 648    11193   9260   6751     58   -116
-1972       C
ATOM   4832  CG  TYR A 648       5.973  -1.154   8.456  1.00 74.26
C
ANISOU 4832  CG  TYR A 648    11834   9417   6963    241   -119
-1841       C
ATOM   4833  CD1 TYR A 648       7.137  -0.934   7.729  1.00 72.68
C
ANISOU 4833  CD1 TYR A 648    11823   9068   6725    286    104
-1699       C
ATOM   4834  CD2 TYR A 648       5.010  -1.995   7.915  1.00 73.50
C
ANISOU 4834  CD2 TYR A 648    11847   9300   6780    367   -347
-1849       C
ATOM   4835  CE1 TYR A 648       7.327  -1.518   6.491  1.00 93.45
C
ANISOU 4835  CE1 TYR A 648    14770  11522   9214    490    118
-1574       C
ATOM   4836  CE2 TYR A 648       5.194  -2.590   6.683  1.00 95.64
C
ANISOU 4836  CE2 TYR A 648    14984  11914   9441    544   -366
```

```
-1738       C
ATOM   4837  CZ  TYR A 648       6.354  -2.350   5.974  1.00134.05
C
ANISOU 4837  CZ  TYR A 648    20060  16622  14250    624   -124
-1602       C
ATOM   4838  OH  TYR A 648       6.542  -2.943   4.746  1.00181.75
O
ANISOU 4838  OH  TYR A 648    26469  22463  20123    835   -127
-1485       O
ATOM   4839  N   ALA A 649       3.074   0.840   9.086  1.00100.07
N
ANISOU 4839  N   ALA A 649    15012  12695  10313    407   -442
-2182       N
ATOM   4840  CA  ALA A 649       1.928   0.995   8.191  1.00 74.43
C
ANISOU 4840  CA  ALA A 649    11945   9339   6994    635   -629
-2204       C
ATOM   4841  C   ALA A 649       1.665   2.452   7.819  1.00 75.87
C
ANISOU 4841  C   ALA A 649    12337   9333   7159    792   -554
-2267       C
ATOM   4842  O   ALA A 649       1.398   2.768   6.654  1.00 94.92
O
ANISOU 4842  O   ALA A 649    15057  11532   9478   1002   -595
-2234       O
ATOM   4843  CB  ALA A 649       0.685   0.372   8.832  1.00106.19
C
ANISOU 4843  CB  ALA A 649    15685  13604  11058    614   -864
-2262       C
ATOM   4844  N   VAL A 650       1.704   3.352   8.799  1.00 76.08
N
ANISOU 4844  N   VAL A 650    12233   9422   7254    707   -451
-2360       N
ATOM   4845  CA  VAL A 650       1.627   4.783   8.504  1.00 77.63
C
ANISOU 4845  CA  VAL A 650    12677   9402   7415    838   -347
-2416       C
ATOM   4846  C   VAL A 650       2.814   5.251   7.662  1.00 82.21
C
ANISOU 4846  C   VAL A 650    13560   9715   7961    818   -142
-2324       C
ATOM   4847  O   VAL A 650       2.646   5.981   6.678  1.00123.33
O
ANISOU 4847  O   VAL A 650    19097  14676  13088   1017   -108
-2307       O
ATOM   4848  CB  VAL A 650       1.511   5.590   9.809  1.00 92.81
C
ANISOU 4848  CB  VAL A 650    14435  11430   9397    734   -278
-2536       C
ATOM   4849  CG1 VAL A 650       1.645   7.079   9.528  1.00 83.29
C
ANISOU 4849  CG1 VAL A 650    13547   9958   8140    835   -144
-2587       C
ATOM   4850  CG2 VAL A 650       0.183   5.294  10.486  1.00 93.28
```

```
C
ANISOU 4850  CG2 VAL A 650    14234  11736   9473    830   -458
-2607       C
ATOM   4851  N   SER A 651       4.027   4.836   8.028  1.00 77.33
N
ANISOU 4851  N   SER A 651    12829   9151   7402    588      5
-2247       N
ATOM   4852  CA  SER A 651       5.221   5.254   7.291  1.00 81.83
C
ANISOU 4852  CA  SER A 651    13631   9504   7958    550    224
-2125       C
ATOM   4853  C   SER A 651       5.233   4.798   5.829  1.00 95.75
C
ANISOU 4853  C   SER A 651    15690  11084   9605    778    217
-2004       C
ATOM   4854  O   SER A 651       5.576   5.583   4.935  1.00 85.54
O
ANISOU 4854  O   SER A 651    14716   9529   8255    894    354
-1944       O
ATOM   4855  CB  SER A 651       6.473   4.753   8.013  1.00 77.31
C
ANISOU 4855  CB  SER A 651    12812   9086   7475    268    361
-2043       C
ATOM   4856  OG  SER A 651       7.651   5.298   7.441  1.00 82.49
O
ANISOU 4856  OG  SER A 651    13635   9564   8142    199    590
-1910       O
ATOM   4857  N   ILE A 652       4.880   3.538   5.554  1.00102.63
N
ANISOU 4857  N   ILE A 652    16497  12069  10427    845     61
-1962       N
ATOM   4858  CA  ILE A 652       4.745   3.112   4.156  1.00 78.20
C
ANISOU 4858  CA  ILE A 652    13742   8780   7192   1088     14
-1867       C
ATOM   4859  C   ILE A 652       3.658   3.896   3.427  1.00 79.56
C
ANISOU 4859  C   ILE A 652    14171   8776   7282   1341   -120
-1945       C
ATOM   4860  O   ILE A 652       3.825   4.267   2.258  1.00102.42
O
ANISOU 4860  O   ILE A 652    17439  11412  10065   1544    -51
-1873       O
ATOM   4861  CB  ILE A 652       4.517   1.589   4.054  1.00 77.13
C
ANISOU 4861  CB  ILE A 652    13524   8781   7001   1095   -161
-1819       C
ATOM   4862  CG1 ILE A 652       3.208   1.161   4.720  1.00127.62
C
ANISOU 4862  CG1 ILE A 652    19672  15382  13435   1066   -450
-1945       C
ATOM   4863  CG2 ILE A 652       5.701   0.833   4.623  1.00 76.08
C
ANISOU 4863  CG2 ILE A 652    13194   8794   6917    901      4
```

```
-1715       C
ATOM   4864  CD1 ILE A 652       2.920  -0.320   4.604  1.00121.40
C
ANISOU 4864  CD1 ILE A 652    18837  14701  12589   1044   -644
-1898       C
ATOM   4865  N   THR A 653       2.534   4.157   4.092  1.00 79.52
N
ANISOU 4865  N   THR A 653    13975   8917   7322   1354   -305
-2081       N
ATOM   4866  CA  THR A 653       1.480   4.966   3.487  1.00 81.05
C
ANISOU 4866  CA  THR A 653    14378   8978   7440   1613   -429
-2150       C
ATOM   4867  C   THR A 653       1.971   6.357   3.095  1.00 82.50
C
ANISOU 4867  C   THR A 653    14861   8893   7591   1696   -202
-2150       C
ATOM   4868  O   THR A 653       1.727   6.818   1.975  1.00168.39
O
ANISOU 4868  O   THR A 653    26101  19532  18349   1944   -209
-2118       O
ATOM   4869  CB  THR A 653       0.291   5.074   4.443  1.00135.39
C
ANISOU 4869  CB  THR A 653    20946  16105  14391   1607   -614
-2274       C
ATOM   4870  OG1 THR A 653      -0.168   3.761   4.783  1.00140.26
O
ANISOU 4870  OG1 THR A 653    21288  16962  15041   1506   -823
-2258       O
ATOM   4871  CG2 THR A 653      -0.845   5.850   3.790  1.00 82.82
C
ANISOU 4871  CG2 THR A 653    14479   9343   7645   1909   -754
-2325       C
ATOM   4872  N   ILE A 654       2.673   7.036   4.003  1.00 82.35
N
ANISOU 4872  N   ILE A 654    14721   8897   7671   1481     -9
-2183       N
ATOM   4873  CA  ILE A 654       3.175   8.383   3.723  1.00 83.98
C
ANISOU 4873  CA  ILE A 654    15220   8834   7853   1509    203
-2182       C
ATOM   4874  C   ILE A 654       4.183   8.392   2.575  1.00 84.72
C
ANISOU 4874  C   ILE A 654    15624   8680   7884   1554    397
-2019       C
ATOM   4875  O   ILE A 654       4.114   9.245   1.682  1.00154.85
O
ANISOU 4875  O   ILE A 654    24882  17285  16670   1751    481
-1994       O
ATOM   4876  CB  ILE A 654       3.762   9.016   5.002  1.00122.12
C
ANISOU 4876  CB  ILE A 654    19856  13743  12800   1221    336
-2249       C
ATOM   4877  CG1 ILE A 654       2.673   9.686   5.858  1.00108.77
```

```
C
ANISOU 4877  CG1 ILE A 654    18079  12142  11107   1303    218
-2416       C
ATOM   4878  CG2 ILE A 654       4.858  10.019   4.661  1.00108.13
C
ANISOU 4878  CG2 ILE A 654    18352  11699  11035   1109    598
-2174       C
ATOM   4879  CD1 ILE A 654       1.404   8.890   6.088  1.00114.84
C
ANISOU 4879  CD1 ILE A 654    18601  13167  11867   1453    -39
-2474       C
ATOM   4880  N   ARG A 655       5.133   7.452   2.571  1.00 83.65
N
ANISOU 4880  N   ARG A 655    15350   8643   7792   1397    485
-1893       N
ATOM   4881  CA  ARG A 655       6.133   7.443   1.502  1.00124.43
C
ANISOU 4881  CA  ARG A 655    20793  13593  12892   1463    704
-1710       C
ATOM   4882  C   ARG A 655       5.518   7.181   0.127  1.00132.63
C
ANISOU 4882  C   ARG A 655    22207  14436  13751   1817    599
-1667       C
ATOM   4883  O   ARG A 655       5.907   7.815  -0.861  1.00 90.07
O
ANISOU 4883  O   ARG A 655    17186   8768   8270   1975    769
-1572       O
ATOM   4884  CB  ARG A 655       7.246   6.432   1.834  1.00110.85
C
ANISOU 4884  CB  ARG A 655    18824  12053  11242   1258    823
-1571       C
ATOM   4885  CG  ARG A 655       7.035   4.942   1.492  1.00 82.23
C
ANISOU 4885  CG  ARG A 655    15151   8557   7536   1368    671
-1517       C
ATOM   4886  CD  ARG A 655       7.319   4.601   0.024  1.00 83.35
C
ANISOU 4886  CD  ARG A 655    15702   8462   7505   1647    751
-1362       C
ATOM   4887  NE  ARG A 655       8.635   5.052  -0.415  1.00 84.63
N
ANISOU 4887  NE  ARG A 655    15982   8491   7681   1605   1096
-1170       N
ATOM   4888  CZ  ARG A 655       9.771   4.400  -0.189  1.00131.91
C
ANISOU 4888  CZ  ARG A 655    21785  14618  13719   1472   1282
-1006       C
ATOM   4889  NH1 ARG A 655      10.898   4.821  -0.741  1.00161.55
N
ANISOU 4889  NH1 ARG A 655    25657  18247  17475   1471   1598
-801       N
ATOM   4890  NH2 ARG A 655       9.762   3.269   0.504  1.00136.44
N
ANISOU 4890  NH2 ARG A 655    22071  15449  14320   1369   1157
```

```
-1025       N
ATOM   4891  N   ILE A 656       4.565   6.252   0.039  1.00144.66
N
ANISOU 4891  N   ILE A 656    23655  16093  15215   1937    313
-1730       N
ATOM   4892  CA  ILE A 656       3.868   6.003  -1.224  1.00 85.57
C
ANISOU 4892  CA  ILE A 656    16534   8429   7551   2263    152
-1707       C
ATOM   4893  C   ILE A 656       3.061   7.220  -1.666  1.00 87.27
C
ANISOU 4893  C   ILE A 656    17005   8454   7698   2485    107
-1795       C
ATOM   4894  O   ILE A 656       3.150   7.662  -2.817  1.00 88.86
O
ANISOU 4894  O   ILE A 656    17630   8376   7757   2729    184
-1725       O
ATOM   4895  CB  ILE A 656       2.979   4.753  -1.106  1.00 84.53
C
ANISOU 4895  CB  ILE A 656    16234   8497   7388   2285   -182
-1758       C
ATOM   4896  CG1 ILE A 656       3.840   3.506  -0.915  1.00 83.22
C
ANISOU 4896  CG1 ILE A 656    15936   8451   7234   2131   -118
-1648       C
ATOM   4897  CG2 ILE A 656       2.101   4.606  -2.332  1.00 85.95
C
ANISOU 4897  CG2 ILE A 656    16779   8497   7383   2603   -411
-1759       C
ATOM   4898  CD1 ILE A 656       4.671   3.151  -2.127  1.00 84.19
C
ANISOU 4898  CD1 ILE A 656    16460   8334   7196   2316     46
-1475       C
ATOM   4899  N   VAL A 657       2.267   7.780  -0.754  1.00106.82
N
ANISOU 4899  N   VAL A 657    19247  11079  10261   2427     -6
-1942       N
ATOM   4900  CA  VAL A 657       1.433   8.938  -1.078  1.00 96.78
C
ANISOU 4900  CA  VAL A 657    18206   9652   8913   2666    -52
-2028       C
ATOM   4901  C   VAL A 657       2.264  10.154  -1.475  1.00113.57
C
ANISOU 4901  C   VAL A 657    20670  11472  11009   2683    259
-1974       C
ATOM   4902  O   VAL A 657       1.990  10.797  -2.493  1.00158.03
O
ANISOU 4902  O   VAL A 657    26707  16841  16497   2963    283
-1950       O
ATOM   4903  CB  VAL A 657       0.492   9.265   0.095  1.00118.77
C
ANISOU 4903  CB  VAL A 657    20654  12680  11793   2607   -198
-2179       C
ATOM   4904  CG1 VAL A 657      -0.158  10.631  -0.109  1.00 90.58
```

```
C
ANISOU 4904  CG1 VAL A 657    17343   8934   8141   2850   -169
-2256       C
ATOM   4905  CG2 VAL A 657      -0.576   8.185   0.227  1.00119.65
C
ANISOU 4905  CG2 VAL A 657    20491  13060  11910   2657   -534
-2214       C
ATOM   4906  N   PHE A 658       3.292  10.487  -0.696  1.00 89.93
N
ANISOU 4906  N   PHE A 658    17525   8500   8146   2378    494
-1946       N
ATOM   4907  CA  PHE A 658       4.178  11.581  -1.093  1.00 91.65
C
ANISOU 4907  CA  PHE A 658    18054   8421   8347   2340    791
-1866       C
ATOM   4908  C   PHE A 658       4.976  11.286  -2.361  1.00109.55
C
ANISOU 4908  C   PHE A 658    20633  10473  10517   2463    965
-1674       C
ATOM   4909  O   PHE A 658       5.065  12.139  -3.250  1.00 94.67
O
ANISOU 4909  O   PHE A 658    19166   8284   8522   2654   1104
-1620       O
ATOM   4910  CB  PHE A 658       5.115  11.949   0.057  1.00105.47
C
ANISOU 4910  CB  PHE A 658    19546  10262  10266   1945    968
-1868       C
ATOM   4911  CG  PHE A 658       4.516  12.927   1.024  1.00106.03
C
ANISOU 4911  CG  PHE A 658    19572  10344  10370   1888    915
-2040       C
ATOM   4912  CD1 PHE A 658       3.661  12.504   2.027  1.00156.64
C
ANISOU 4912  CD1 PHE A 658    25642  17044  16831   1862    698
-2181       C
ATOM   4913  CD2 PHE A 658       4.795  14.282   0.912  1.00112.61
C
ANISOU 4913  CD2 PHE A 658    20737  10884  11166   1877   1092
-2051       C
ATOM   4914  CE1 PHE A 658       3.105  13.415   2.913  1.00187.34
C
ANISOU 4914  CE1 PHE A 658    29522  20936  20723   1853    670
-2329       C
ATOM   4915  CE2 PHE A 658       4.244  15.197   1.793  1.00114.26
C
ANISOU 4915  CE2 PHE A 658    20967  11075  11372   1854   1048
-2210       C
ATOM   4916  CZ  PHE A 658       3.397  14.764   2.795  1.00168.46
C
ANISOU 4916  CZ  PHE A 658    27494  18237  18276   1857    842
-2349       C
ATOM   4917  N   GLY A 659       5.560  10.098  -2.475  1.00 91.18
N
ANISOU 4917  N   GLY A 659    18140   8290   8212   2382    973
```

```
-1562       N
ATOM   4918  CA  GLY A 659       6.530   9.884  -3.538  1.00137.00
C
ANISOU 4918  CA  GLY A 659    24222  13901  13932   2477   1209
-1351       C
ATOM   4919  C   GLY A 659       6.078   9.967  -4.987  1.00120.51
C
ANISOU 4919  C   GLY A 659    22633  11535  11622   2879   1174
-1298       C
ATOM   4920  O   GLY A 659       6.619  10.765  -5.759  1.00113.79
O
ANISOU 4920  O   GLY A 659    22133  10401  10699   2985   1423
-1184       O
ATOM   4921  N   PHE A 660       5.092   9.155  -5.370  1.00 93.14
N
ANISOU 4921  N   PHE A 660    19211   8138   8038   3096    860
-1373       N
ATOM   4922  CA  PHE A 660       4.651   9.101  -6.764  1.00 94.71
C
ANISOU 4922  CA  PHE A 660    19899   8082   8006   3483    782
-1324       C
ATOM   4923  C   PHE A 660       3.983  10.394  -7.232  1.00122.58
C
ANISOU 4923  C   PHE A 660    23755  11374  11446   3709    777
-1401       C
ATOM   4924  O   PHE A 660       4.188  10.823  -8.372  1.00140.84
O
ANISOU 4924  O   PHE A 660    26535  13386  13591   3973    912
-1302       O
ATOM   4925  CB  PHE A 660       3.736   7.895  -6.977  1.00 93.84
C
ANISOU 4925  CB  PHE A 660    19738   8115   7801   3609    401
-1390       C
ATOM   4926  CG  PHE A 660       4.369   6.586  -6.592  1.00 92.17
C
ANISOU 4926  CG  PHE A 660    19278   8101   7641   3422    405
-1310       C
ATOM   4927  CD1 PHE A 660       3.632   5.591  -5.973  1.00 90.65
C
ANISOU 4927  CD1 PHE A 660    18778   8170   7495   3313     82
-1413       C
ATOM   4928  CD2 PHE A 660       5.709   6.354  -6.855  1.00 92.37
C
ANISOU 4928  CD2 PHE A 660    19378   8054   7664   3365    743
-1117       C
ATOM   4929  CE1 PHE A 660       4.222   4.389  -5.626  1.00 89.27
C
ANISOU 4929  CE1 PHE A 660    18414   8156   7348   3156     93
-1337       C
ATOM   4930  CE2 PHE A 660       6.303   5.157  -6.509  1.00 91.04
C
ANISOU 4930  CE2 PHE A 660    18999   8070   7523   3230    759
-1033       C
ATOM   4931  CZ  PHE A 660       5.560   4.173  -5.895  1.00 89.45
```

```
C
ANISOU 4931  CZ  PHE A 660    18531   8102   7352   3129    432
-1151       C
ATOM   4932  N   MET A 661       3.152  11.011  -6.394  1.00 97.30
N
ANISOU 4932  N   MET A 661    20340   8299   8332   3646    627
-1572       N
ATOM   4933  CA  MET A 661       2.580  12.309  -6.750  1.00117.81
C
ANISOU 4933  CA  MET A 661    23255  10670  10839   3868    654
-1640       C
ATOM   4934  C   MET A 661       3.634  13.413  -6.853  1.00141.53
C
ANISOU 4934  C   MET A 661    26495  13408  13872   3757   1048
-1542       C
ATOM   4935  O   MET A 661       3.543  14.274  -7.736  1.00191.34
O
ANISOU 4935  O   MET A 661    33261  19406  20033   4012   1159
-1504       O
ATOM   4936  CB  MET A 661       1.480  12.690  -5.751  1.00138.03
C
ANISOU 4936  CB  MET A 661    25522  13447  13479   3842    427
-1830       C
ATOM   4937  CG  MET A 661       1.956  13.160  -4.380  1.00118.89
C
ANISOU 4937  CG  MET A 661    22761  11163  11250   3485    576
-1893       C
ATOM   4938  SD  MET A 661       2.370  14.912  -4.241  1.00111.33
S
ANISOU 4938  SD  MET A 661    22134   9889  10279   3461    872
-1912       S
ATOM   4939  CE  MET A 661       0.735  15.638  -4.212  1.00100.62
C
ANISOU 4939  CE  MET A 661    20894   8545   8794   3836    618
-2082       C
ATOM   4940  N   LEU A 662       4.631  13.417  -5.965  1.00124.47
N
ANISOU 4940  N   LEU A 662    24035  11360  11900   3371   1253
-1492       N
ATOM   4941  CA  LEU A 662       5.661  14.456  -6.009  1.00138.10
C
ANISOU 4941  CA  LEU A 662    25949  12846  13675   3204   1608
-1383       C
ATOM   4942  C   LEU A 662       6.504  14.417  -7.286  1.00125.89
C
ANISOU 4942  C   LEU A 662    24782  11037  12012   3362   1870
-1158       C
ATOM   4943  O   LEU A 662       6.798  15.470  -7.865  1.00141.33
O
ANISOU 4943  O   LEU A 662    27123  12680  13897   3446   2092
-1087       O
ATOM   4944  CB  LEU A 662       6.556  14.343  -4.773  1.00124.39
C
ANISOU 4944  CB  LEU A 662    23771  11324  12169   2737   1727
```

```
-1367       C
ATOM   4945  CG  LEU A 662       7.580  15.450  -4.523  1.00122.33
C
ANISOU 4945  CG  LEU A 662    23613  10865  12003   2458   2040
-1276       C
ATOM   4946  CD1 LEU A 662       6.920  16.818  -4.598  1.00103.27
C
ANISOU 4946  CD1 LEU A 662    21570   8174   9492   2600   2041
-1398       C
ATOM   4947  CD2 LEU A 662       8.231  15.254  -3.165  1.00100.00
C
ANISOU 4947  CD2 LEU A 662    20304   8300   9393   2004   2055
-1303       C
ATOM   4948  N   ILE A 663       6.910  13.230  -7.744  1.00101.22
N
ANISOU 4948  N   ILE A 663    21586   8021   8852   3417   1865
-1033       N
ATOM   4949  CA  ILE A 663       7.553  13.138  -9.058  1.00102.97
C
ANISOU 4949  CA  ILE A 663    22223   7986   8914   3658   2099
-818       C
ATOM   4950  C   ILE A 663       6.609  13.583 -10.173  1.00122.80
C
ANISOU 4950  C   ILE A 663    25257  10221  11180   4106   1968
-876       C
ATOM   4951  O   ILE A 663       7.021  14.274 -11.113  1.00156.80
O
ANISOU 4951  O   ILE A 663    29913  14284  15381   4241   2193
-705       O
ATOM   4952  CB  ILE A 663       8.112  11.721  -9.307  1.00101.75
C
ANISOU 4952  CB  ILE A 663    21925   8001   8736   3672   2105
-680       C
ATOM   4953  CG1 ILE A 663       7.006  10.668  -9.335  1.00100.12
C
ANISOU 4953  CG1 ILE A 663    21655   7958   8430   3849   1688
-832       C
ATOM   4954  CG2 ILE A 663       9.151  11.367  -8.250  1.00100.51
C
ANISOU 4954  CG2 ILE A 663    21265   8109   8814   3248   2270
-595       C
ATOM   4955  CD1 ILE A 663       7.489   9.282  -9.699  1.00 99.31
C
ANISOU 4955  CD1 ILE A 663    21525   7959   8251   3911   1681
-700       C
ATOM   4956  N   ALA A 664       5.335  13.194 -10.092  1.00103.69
N
ANISOU 4956  N   ALA A 664    22720   7974   8702   4250   1559
-1033       N
ATOM   4957  CA  ALA A 664       4.335  13.674 -11.044  1.00105.42
C
ANISOU 4957  CA  ALA A 664    23145   8154   8757   4532   1336
-997       C
ATOM   4958  C   ALA A 664       4.178  15.191 -11.001  1.00125.89
```

```
C
ANISOU 4958  C   ALA A 664    25890  10587  11356   4531   1468
-1010       C
ATOM   4959  O   ALA A 664       4.041  15.836 -12.047  1.00165.16
O
ANISOU 4959  O   ALA A 664    31172  15413  16168   4732   1516
-877       O
ATOM   4960  CB  ALA A 664       2.993  12.990 -10.781  1.00104.22
C
ANISOU 4960  CB  ALA A 664    22783   8236   8581   4646    876
-1168       C
ATOM   4961  N   LEU A 665       4.177  15.777  -9.805  1.00144.14
N
ANISOU 4961  N   LEU A 665    28014  12927  13826   4309   1523
-1172       N
ATOM   4962  CA  LEU A 665       4.138  17.233  -9.689  1.00147.05
C
ANISOU 4962  CA  LEU A 665    28566  13112  14194   4281   1671
-1180       C
ATOM   4963  C   LEU A 665       5.334  17.894 -10.368  1.00128.94
C
ANISOU 4963  C   LEU A 665    26611  10515  11866   4212   2077
-974       C
ATOM   4964  O   LEU A 665       5.174  18.860 -11.123  1.00146.00
O
ANISOU 4964  O   LEU A 665    29073  12502  13898   4359   2155
-877       O
ATOM   4965  CB  LEU A 665       4.070  17.637  -8.216  1.00164.70
C
ANISOU 4965  CB  LEU A 665    30564  15423  16593   4021   1673
-1387       C
ATOM   4966  CG  LEU A 665       4.049  19.143  -7.943  1.00169.47
C
ANISOU 4966  CG  LEU A 665    31377  15822  17192   3961   1818
-1412       C
ATOM   4967  CD1 LEU A 665       2.858  19.803  -8.627  1.00158.96
C
ANISOU 4967  CD1 LEU A 665    30198  14501  15699   4304   1611
-1400       C
ATOM   4968  CD2 LEU A 665       4.040  19.419  -6.447  1.00152.60
C
ANISOU 4968  CD2 LEU A 665    29018  13767  15196   3690   1808
-1618       C
ATOM   4969  N   ILE A 666       6.539  17.391 -10.117  1.00117.91
N
ANISOU 4969  N   ILE A 666    25179   9045  10575   4001   2354
-898       N
ATOM   4970  CA  ILE A 666       7.742  18.078 -10.584  1.00129.00
C
ANISOU 4970  CA  ILE A 666    26890  10136  11988   3896   2786
-704       C
ATOM   4971  C   ILE A 666       8.134  17.655 -11.996  1.00123.80
C
ANISOU 4971  C   ILE A 666    26477   9406  11157   4151   2914
```

```
-449       C
ATOM   4972  O   ILE A 666       8.460  18.503 -12.830  1.00137.08
O
ANISOU 4972  O   ILE A 666    28492  10865  12729   4238   3134
-289       O
ATOM   4973  CB  ILE A 666       8.897  17.851  -9.590  1.00129.27
C
ANISOU 4973  CB  ILE A 666    26539  10305  12273   3422   2990
-638       C
ATOM   4974  CG1 ILE A 666       8.483  18.294  -8.186  1.00133.65
C
ANISOU 4974  CG1 ILE A 666    26767  11030  12984   3118   2813
-855       C
ATOM   4975  CG2 ILE A 666      10.135  18.611 -10.035  1.00138.16
C
ANISOU 4975  CG2 ILE A 666    27872  11176  13448   3241   3414
-386       C
ATOM   4976  CD1 ILE A 666       8.131  19.765  -8.090  1.00139.38
C
ANISOU 4976  CD1 ILE A 666    27858  11450  13650   3135   2876
-952       C
ATOM   4977  N   TRP A 667       8.110  16.358 -12.303  1.00128.17
N
ANISOU 4977  N   TRP A 667    26896  10144  11660   4277   2782
-406       N
ATOM   4978  CA  TRP A 667       8.626  15.865 -13.576  1.00127.15
C
ANISOU 4978  CA  TRP A 667    27010   9952  11350   4504   2935
-157       C
ATOM   4979  C   TRP A 667       7.564  15.164 -14.418  1.00135.72
C
ANISOU 4979  C   TRP A 667    28132  11220  12214   4798   2540
-173       C
ATOM   4980  O   TRP A 667       7.911  14.417 -15.339  1.00136.26
O
ANISOU 4980  O   TRP A 667    28356  11302  12114   4974   2591
-7       O
ATOM   4981  CB  TRP A 667       9.829  14.949 -13.353  1.00112.57
C
ANISOU 4981  CB  TRP A 667    25070   8093   9607   4403   3225
-21       C
ATOM   4982  CG  TRP A 667      10.973  15.648 -12.682  1.00113.51
C
ANISOU 4982  CG  TRP A 667    25029   8141   9957   4022   3596
95       C
ATOM   4983  CD1 TRP A 667      11.837  16.536 -13.258  1.00126.90
C
ANISOU 4983  CD1 TRP A 667    26998   9567  11651   3985   3995
320       C
ATOM   4984  CD2 TRP A 667      11.383  15.522 -11.315  1.00121.66
C
ANISOU 4984  CD2 TRP A 667    25481   9482  11263   3543   3555
36       C
ATOM   4985  NE1 TRP A 667      12.756  16.971 -12.336  1.00121.05
```

```
N
ANISOU 4985  NE1 TRP A 667    25868   8950  11175   3489   4186
407       N
ATOM   4986  CE2 TRP A 667      12.501  16.362 -11.135  1.00134.08
C
ANISOU 4986  CE2 TRP A 667    26999  10953  12991   3219   3916
231       C
ATOM   4987  CE3 TRP A 667      10.914  14.780 -10.227  1.00150.70
C
ANISOU 4987  CE3 TRP A 667    28687  13508  15066   3353   3246
-154       C
ATOM   4988  CZ2 TRP A 667      13.155  16.482  -9.910  1.00143.75
C
ANISOU 4988  CZ2 TRP A 667    27722  12413  14484   2714   3949
230       C
ATOM   4989  CZ3 TRP A 667      11.566  14.900  -9.011  1.00132.69
C
ANISOU 4989  CZ3 TRP A 667    25921  11453  13040   2878   3302
-156       C
ATOM   4990  CH2 TRP A 667      12.674  15.744  -8.863  1.00138.99
C
ANISOU 4990  CH2 TRP A 667    26684  12144  13982   2564   3639
30       C
ATOM   4991  N   GLU A 668       6.283  15.359 -14.088  1.00140.59
N
ANISOU 4991  N   GLU A 668    28618  11974  12827   4852   2152
-370       N
ATOM   4992  CA  GLU A 668       5.141  14.875 -14.880  1.00112.60
C
ANISOU 4992  CA  GLU A 668    25145   8559   9079   5125   1758
-398       C
ATOM   4993  C   GLU A 668       5.266  13.391 -15.229  1.00111.38
C
ANISOU 4993  C   GLU A 668    24946   8531   8841   5199   1617
-343       C
ATOM   4994  O   GLU A 668       4.915  12.955 -16.327  1.00112.61
O
ANISOU 4994  O   GLU A 668    25339   8696   8753   5433   1463
-254       O
ATOM   4995  CB  GLU A 668       4.900  15.736 -16.134  1.00115.61
C
ANISOU 4995  CB  GLU A 668    25916   8797   9215   5366   1812
-269       C
ATOM   4996  CG  GLU A 668       6.032  15.867 -17.149  1.00144.50
C
ANISOU 4996  CG  GLU A 668    29902  12280  12721   5435   2206
-21       C
ATOM   4997  CD  GLU A 668       5.741  16.902 -18.220  1.00140.69
C
ANISOU 4997  CD  GLU A 668    29792  11660  12005   5641   2271
67       C
ATOM   4998  OE1 GLU A 668       5.587  18.093 -17.871  1.00139.65
O
ANISOU 4998  OE1 GLU A 668    29709  11409  11941   5571   2363
```

```
17       O
ATOM   4999  OE2 GLU A 668       5.671  16.525 -19.409  1.00132.45
O
ANISOU 4999  OE2 GLU A 668    29012  10624  10688   5874   2232
180       O
ATOM   5000  N   PHE A 669       5.772  12.605 -14.278  1.00110.10
N
ANISOU 5000  N   PHE A 669    24504   8466   8862   4997   1666
-405       N
ATOM   5001  CA  PHE A 669       6.099  11.202 -14.505  1.00108.03
C
ANISOU 5001  CA  PHE A 669    24216   8301   8528   5042   1599
-335       C
ATOM   5002  C   PHE A 669       4.996  10.329 -13.914  1.00142.32
C
ANISOU 5002  C   PHE A 669    28307  12855  12913   5023   1135
-544       C
ATOM   5003  O   PHE A 669       4.793  10.306 -12.695  1.00146.96
O
ANISOU 5003  O   PHE A 669    28566  13565  13706   4818   1067
-730       O
ATOM   5004  CB  PHE A 669       7.452  10.867 -13.880  1.00107.12
C
ANISOU 5004  CB  PHE A 669    23982   8149   8569   4853   1989
-247       C
ATOM   5005  CG  PHE A 669       7.996   9.520 -14.272  1.00106.64
C
ANISOU 5005  CG  PHE A 669    23970   8154   8395   4948   2014
-114       C
ATOM   5006  CD1 PHE A 669       8.878   9.389 -15.330  1.00175.29
C
ANISOU 5006  CD1 PHE A 669    32965  16731  16905   5125   2319
150       C
ATOM   5007  CD2 PHE A 669       7.643   8.385 -13.556  1.00104.32
C
ANISOU 5007  CD2 PHE A 669    23427   8047   8163   4864   1750
-252       C
ATOM   5008  CE1 PHE A 669       9.384   8.144 -15.675  1.00189.36
C
ANISOU 5008  CE1 PHE A 669    34810  18584  18557   5235   2354
278       C
ATOM   5009  CE2 PHE A 669       8.145   7.140 -13.897  1.00104.06
C
ANISOU 5009  CE2 PHE A 669    23467   8062   8007   4958   1772
-127       C
ATOM   5010  CZ  PHE A 669       9.016   7.019 -14.956  1.00112.98
C
ANISOU 5010  CZ  PHE A 669    24907   9076   8945   5151   2073
142       C
ATOM   5011  N   ASP A 670       4.283   9.622 -14.789  1.00141.82
N
ANISOU 5011  N   ASP A 670    28421  12828  12636   5234    819
-517       N
ATOM   5012  CA  ASP A 670       3.205   8.725 -14.389  1.00116.06
```

```
C
ANISOU 5012  CA  ASP A 670    24984   9729   9385   5239    360
-695       C
ATOM   5013  C   ASP A 670       3.790   7.370 -14.001  1.00103.69
C
ANISOU 5013  C   ASP A 670    23306   8246   7846   5127    364
-676       C
ATOM   5014  O   ASP A 670       4.441   6.715 -14.822  1.00121.98
O
ANISOU 5014  O   ASP A 670    25875  10491   9980   5241    479
-492       O
ATOM   5015  CB  ASP A 670       2.194   8.582 -15.525  1.00109.51
C
ANISOU 5015  CB  ASP A 670    24446   8868   8297   5510      6
-678       C
ATOM   5016  CG  ASP A 670       1.612   9.919 -15.963  1.00110.23
C
ANISOU 5016  CG  ASP A 670    24671   8882   8328   5654      9
-688       C
ATOM   5017  OD1 ASP A 670       1.523  10.838 -15.122  1.00131.50
O
ANISOU 5017  OD1 ASP A 670    27154  11594  11216   5535    125
-787       O
ATOM   5018  OD2 ASP A 670       1.250  10.054 -17.151  1.00111.60
O
ANISOU 5018  OD2 ASP A 670    25185   8978   8238   5890   -103
-601       O
ATOM   5019  N   PHE A 671       3.565   6.945 -12.763  1.00101.39
N
ANISOU 5019  N   PHE A 671    22653   8119   7751   4916    249
-865       N
ATOM   5020  CA  PHE A 671       4.092   5.665 -12.309  1.00102.67
C
ANISOU 5020  CA  PHE A 671    22701   8378   7931   4800    250
-863       C
ATOM   5021  C   PHE A 671       3.121   4.536 -12.648  1.00 99.79
C
ANISOU 5021  C   PHE A 671    22415   8075   7425   4889   -222
-933       C
ATOM   5022  O   PHE A 671       1.911   4.745 -12.766  1.00100.41
O
ANISOU 5022  O   PHE A 671    22489   8187   7477   4968   -586
-1065       O
ATOM   5023  CB  PHE A 671       4.375   5.690 -10.806  1.00 98.52
C
ANISOU 5023  CB  PHE A 671    21703   8068   7662   4473    351
-1008       C
ATOM   5024  CG  PHE A 671       5.241   4.552 -10.338  1.00130.22
C
ANISOU 5024  CG  PHE A 671    25489  12257  11733   4283    461
-900       C
ATOM   5025  CD1 PHE A 671       4.683   3.394  -9.832  1.00125.99
C
ANISOU 5025  CD1 PHE A 671    24721  11935  11212   4157    133
```

```
-988       C
ATOM   5026  CD2 PHE A 671       6.621   4.644 -10.411  1.00159.79
C
ANISOU 5026  CD2 PHE A 671    29246  15954  15511   4235    900
-696       C
ATOM   5027  CE1 PHE A 671       5.485   2.345  -9.408  1.00 93.34
C
ANISOU 5027  CE1 PHE A 671    20409   7948   7107   4008    243
-887       C
ATOM   5028  CE2 PHE A 671       7.426   3.601  -9.987  1.00136.33
C
ANISOU 5028  CE2 PHE A 671    26061  13160  12578   4095   1010
-583       C
ATOM   5029  CZ  PHE A 671       6.856   2.451  -9.484  1.00 93.64
C
ANISOU 5029  CZ  PHE A 671    20461   7949   7169   3992    682
-686       C
ATOM   5030  N   SER A 672       3.670   3.330 -12.808  1.00 99.37
N
ANISOU 5030  N   SER A 672    22458   8026   7274   4885   -214
-839       N
ATOM   5031  CA  SER A 672       2.863   2.155 -13.129  1.00121.59
C
ANISOU 5031  CA  SER A 672    25397  10860   9941   4939   -657
-893       C
ATOM   5032  C   SER A 672       2.019   1.723 -11.931  1.00 97.73
C
ANISOU 5032  C   SER A 672    21994   8063   7075   4706   -971
-1158       C
ATOM   5033  O   SER A 672       2.551   1.411 -10.861  1.00 95.68
O
ANISOU 5033  O   SER A 672    21301   8040   7013   4415   -819
-1158       O
ATOM   5034  CB  SER A 672       3.768   1.012 -13.580  1.00131.57
C
ANISOU 5034  CB  SER A 672    26891  12060  11041   4998   -525
-706       C
ATOM   5035  OG  SER A 672       3.023  -0.173 -13.801  1.00173.97
O
ANISOU 5035  OG  SER A 672    32398  17427  16277   5011   -961
-761       O
ATOM   5036  N   ALA A 673       0.699   1.698 -12.127  1.00 98.63
N
ANISOU 5036  N   ALA A 673    22128   8206   7141   4767  -1421
-1295       N
ATOM   5037  CA  ALA A 673      -0.220   1.158 -11.127  1.00 97.34
C
ANISOU 5037  CA  ALA A 673    21472   8363   7152   4484  -1774
-1434       C
ATOM   5038  C   ALA A 673       0.039  -0.316 -10.825  1.00130.92
C
ANISOU 5038  C   ALA A 673    25660  12708  11373   4298  -1904
-1396       C
ATOM   5039  O   ALA A 673      -0.009  -0.735  -9.663  1.00140.86
```

```
O
ANISOU 5039  O   ALA A 673    26410  14264  12845   3981  -1932
-1443       O
ATOM   5040  CB  ALA A 673      -1.662   1.358 -11.590  1.00 99.94
C
ANISOU 5040  CB  ALA A 673    21878   8691   7404   4622  -2242
-1546       C
ATOM   5041  N   PHE A 674       0.299  -1.119 -11.857  1.00 97.87
N
ANISOU 5041  N   PHE A 674    22021   8261   6906   4508  -1987
-1312       N
ATOM   5042  CA  PHE A 674       0.574  -2.540 -11.657  1.00 97.31
C
ANISOU 5042  CA  PHE A 674    21983   8230   6760   4368  -2104
-1269       C
ATOM   5043  C   PHE A 674       1.822  -2.790 -10.817  1.00115.81
C
ANISOU 5043  C   PHE A 674    24034  10722   9246   4192  -1674
-1168       C
ATOM   5044  O   PHE A 674       1.833  -3.688  -9.967  1.00160.94
O
ANISOU 5044  O   PHE A 674    29436  16651  15063   3929  -1769
-1188       O
ATOM   5045  CB  PHE A 674       0.703  -3.249 -13.003  1.00 99.69
C
ANISOU 5045  CB  PHE A 674    22847   8213   6818   4626  -2216
-1093       C
ATOM   5046  CG  PHE A 674       0.708  -4.748 -12.894  1.00103.02
C
ANISOU 5046  CG  PHE A 674    23324   8627   7192   4488  -2428
-1053       C
ATOM   5047  CD1 PHE A 674      -0.479  -5.453 -12.781  1.00109.08
C
ANISOU 5047  CD1 PHE A 674    23984   9457   8004   4307  -2943
-1164       C
ATOM   5048  CD2 PHE A 674       1.903  -5.451 -12.886  1.00108.82
C
ANISOU 5048  CD2 PHE A 674    24194   9296   7858   4528  -2098
-892       C
ATOM   5049  CE1 PHE A 674      -0.473  -6.833 -12.675  1.00113.26
C
ANISOU 5049  CE1 PHE A 674    24555   9950   8526   4149  -3117
-1121       C
ATOM   5050  CE2 PHE A 674       1.916  -6.831 -12.779  1.00 99.35
C
ANISOU 5050  CE2 PHE A 674    23059   8061   6631   4414  -2272
-857       C
ATOM   5051  CZ  PHE A 674       0.726  -7.523 -12.673  1.00100.11
C
ANISOU 5051  CZ  PHE A 674    23061   8189   6788   4212  -2778
-977       C
ATOM   5052  N   MET A 675       2.886  -2.023 -11.046  1.00 95.18
N
ANISOU 5052  N   MET A 675    21519   8006   6641   4328  -1204
```

```
-1048       N
ATOM   5053  CA  MET A 675       4.077  -2.143 -10.208  1.00124.40
C
ANISOU 5053  CA  MET A 675    24881  11883  10503   4144   -799
-942       C
ATOM   5054  C   MET A 675       3.798  -1.860  -8.731  1.00139.82
C
ANISOU 5054  C   MET A 675    26138  14201  12787   3762   -830
-1061       C
ATOM   5055  O   MET A 675       4.289  -2.585  -7.858  1.00104.99
O
ANISOU 5055  O   MET A 675    21405  10002   8483   3537   -753
-1032       O
ATOM   5056  CB  MET A 675       5.176  -1.223 -10.738  1.00109.14
C
ANISOU 5056  CB  MET A 675    23142   9784   8544   4334   -311
-782       C
ATOM   5057  CG  MET A 675       5.765  -1.681 -12.068  1.00118.63
C
ANISOU 5057  CG  MET A 675    25004  10657   9413   4716   -171
-610       C
ATOM   5058  SD  MET A 675       6.769  -3.178 -11.927  1.00132.26
S
ANISOU 5058  SD  MET A 675    26809  12436  11008   4726    -15
-444       S
ATOM   5059  CE  MET A 675       5.617  -4.437 -12.475  1.00 97.18
C
ANISOU 5059  CE  MET A 675    22664   7883   6379   4764   -610
-500       C
ATOM   5060  N   VAL A 676       3.023  -0.816  -8.425  1.00153.54
N
ANISOU 5060  N   VAL A 676    27656  16011  14672   3707   -932
-1190       N
ATOM   5061  CA  VAL A 676       2.601  -0.587  -7.041  1.00122.27
C
ANISOU 5061  CA  VAL A 676    23079  12386  10993   3379  -1001
-1312       C
ATOM   5062  C   VAL A 676       1.749  -1.739  -6.516  1.00 88.49
C
ANISOU 5062  C   VAL A 676    18583   8301   6736   3195  -1397
-1396       C
ATOM   5063  O   VAL A 676       1.880  -2.149  -5.356  1.00 86.69
O
ANISOU 5063  O   VAL A 676    17902   8346   6689   2912  -1372
-1428       O
ATOM   5064  CB  VAL A 676       1.847   0.750  -6.925  1.00 89.70
C
ANISOU 5064  CB  VAL A 676    18839   8271   6973   3422  -1046
-1427       C
ATOM   5065  CG1 VAL A 676       1.590   1.079  -5.471  1.00 87.93
C
ANISOU 5065  CG1 VAL A 676    18016   8371   7020   3114  -1039
-1534       C
ATOM   5066  CG2 VAL A 676       2.618   1.865  -7.587  1.00107.30
```

```
C
ANISOU 5066  CG2 VAL A 676    21367  10254   9148   3614   -683
-1339       C
ATOM   5067  N   LEU A 677       0.860  -2.270  -7.354  1.00 90.11
N
ANISOU 5067  N   LEU A 677    19117   8366   6757   3342  -1775
-1429       N
ATOM   5068  CA  LEU A 677       0.100  -3.469  -7.005  1.00 89.98
C
ANISOU 5068  CA  LEU A 677    18968   8487   6733   3157  -2166
-1481       C
ATOM   5069  C   LEU A 677       0.999  -4.664  -6.683  1.00 88.94
C
ANISOU 5069  C   LEU A 677    18867   8380   6546   3040  -2035
-1387       C
ATOM   5070  O   LEU A 677       0.733  -5.410  -5.734  1.00120.32
O
ANISOU 5070  O   LEU A 677    22479  12593  10646   2765  -2172
-1428       O
ATOM   5071  CB  LEU A 677      -0.869  -3.798  -8.146  1.00 92.47
C
ANISOU 5071  CB  LEU A 677    19724   8589   6822   3352  -2591
-1509       C
ATOM   5072  CG  LEU A 677      -1.852  -4.976  -8.080  1.00 93.32
C
ANISOU 5072  CG  LEU A 677    19803   8772   6882   3178  -3090
-1557       C
ATOM   5073  CD1 LEU A 677      -1.225  -6.299  -8.522  1.00 93.98
C
ANISOU 5073  CD1 LEU A 677    20308   8663   6736   3197  -3118
-1469       C
ATOM   5074  CD2 LEU A 677      -2.461  -5.102  -6.698  1.00113.88
C
ANISOU 5074  CD2 LEU A 677    21727  11768   9776   2827  -3198
-1634       C
ATOM   5075  N   ILE A 678       2.062  -4.865  -7.462  1.00 89.59
N
ANISOU 5075  N   ILE A 678    19386   8223   6432   3266  -1758
-1248       N
ATOM   5076  CA  ILE A 678       3.091  -5.851  -7.122  1.00 88.70
C
ANISOU 5076  CA  ILE A 678    19285   8150   6267   3204  -1543
-1132       C
ATOM   5077  C   ILE A 678       3.758  -5.562  -5.777  1.00107.04
C
ANISOU 5077  C   ILE A 678    21011  10791   8869   2935  -1250
-1128       C
ATOM   5078  O   ILE A 678       3.978  -6.478  -4.974  1.00119.75
O
ANISOU 5078  O   ILE A 678    22384  12583  10533   2736  -1271
-1118       O
ATOM   5079  CB  ILE A 678       4.128  -5.936  -8.258  1.00115.54
C
ANISOU 5079  CB  ILE A 678    23255  11244   9402   3553  -1247
```

```
-958       C
ATOM   5080  CG1 ILE A 678       3.495  -6.537  -9.514  1.00103.59
C
ANISOU 5080  CG1 ILE A 678    22388   9407   7565   3807  -1583
-964       C
ATOM   5081  CG2 ILE A 678       5.336  -6.757  -7.830  1.00142.30
C
ANISOU 5081  CG2 ILE A 678    26587  14718  12761   3523   -937
-810       C
ATOM   5082  CD1 ILE A 678       3.119  -7.996  -9.366  1.00 92.90
C
ANISOU 5082  CD1 ILE A 678    21154   8052   6094   3681  -1909
-981       C
ATOM   5083  N   ILE A 679       4.095  -4.300  -5.506  1.00 85.87
N
ANISOU 5083  N   ILE A 679    18104   8168   6356   2921   -984
-1136       N
ATOM   5084  CA  ILE A 679       4.633  -3.932  -4.193  1.00 91.59
C
ANISOU 5084  CA  ILE A 679    18267   9186   7346   2644   -755
-1152       C
ATOM   5085  C   ILE A 679       3.672  -4.293  -3.063  1.00101.47
C
ANISOU 5085  C   ILE A 679    19061  10720   8771   2355  -1050
-1302       C
ATOM   5086  O   ILE A 679       4.078  -4.856  -2.039  1.00 81.05
O
ANISOU 5086  O   ILE A 679    16127   8366   6302   2132   -975
-1295       O
ATOM   5087  CB  ILE A 679       4.977  -2.431  -4.157  1.00103.08
C
ANISOU 5087  CB  ILE A 679    19620  10611   8933   2667   -485
-1156       C
ATOM   5088  CG1 ILE A 679       6.036  -2.083  -5.200  1.00147.19
C
ANISOU 5088  CG1 ILE A 679    25616  15941  14369   2926   -144
-975       C
ATOM   5089  CG2 ILE A 679       5.450  -2.019  -2.771  1.00 86.53
C
ANISOU 5089  CG2 ILE A 679    16972   8805   7101   2361   -301
-1192       C
ATOM   5090  CD1 ILE A 679       6.229  -0.593  -5.391  1.00153.59
C
ANISOU 5090  CD1 ILE A 679    26438  16648  15271   2972     79
-977       C
ATOM   5091  N   ALA A 680       2.387  -3.981  -3.232  1.00140.01
N
ANISOU 5091  N   ALA A 680    23929  15598  13669   2369  -1382
-1427       N
ATOM   5092  CA  ALA A 680       1.384  -4.298  -2.215  1.00130.72
C
ANISOU 5092  CA  ALA A 680    22312  14701  12657   2116  -1660
-1547       C
ATOM   5093  C   ALA A 680       1.260  -5.795  -1.931  1.00129.38
```

```
C
ANISOU 5093  C   ALA A 680    22129  14607  12422   1965  -1865
-1521       C
ATOM   5094  O   ALA A 680       1.214  -6.209  -0.767  1.00140.06
O
ANISOU 5094  O   ALA A 680    23059  16226  13932   1709  -1867
-1556       O
ATOM   5095  CB  ALA A 680       0.029  -3.726  -2.635  1.00108.60
C
ANISOU 5095  CB  ALA A 680    19534  11873   9855   2207  -1984
-1648       C
ATOM   5096  N   ILE A 681       1.208  -6.624  -2.974  1.00 83.55
N
ANISOU 5096  N   ILE A 681    16817   8557   6372   2124  -2039
-1459       N
ATOM   5097  CA  ILE A 681       1.190  -8.075  -2.778  1.00122.63
C
ANISOU 5097  CA  ILE A 681    21846  13524  11222   1993  -2217
-1424       C
ATOM   5098  C   ILE A 681       2.459  -8.588  -2.101  1.00129.34
C
ANISOU 5098  C   ILE A 681    22572  14478  12094   1923  -1869
-1328       C
ATOM   5099  O   ILE A 681       2.390  -9.415  -1.184  1.00193.21
O
ANISOU 5099  O   ILE A 681    30391  22762  20258   1693  -1940
-1342       O
ATOM   5100  CB  ILE A 681       0.943  -8.790  -4.119  1.00 97.63
C
ANISOU 5100  CB  ILE A 681    19326  10022   7749   2209  -2467
-1376       C
ATOM   5101  CG1 ILE A 681      -0.442  -8.434  -4.661  1.00 94.00
C
ANISOU 5101  CG1 ILE A 681    18922   9512   7282   2227  -2890
-1474       C
ATOM   5102  CG2 ILE A 681       1.072 -10.302  -3.949  1.00 85.98
C
ANISOU 5102  CG2 ILE A 681    18017   8516   6136   2090  -2615
-1328       C
ATOM   5103  CD1 ILE A 681      -0.694  -8.935  -6.064  1.00146.98
C
ANISOU 5103  CD1 ILE A 681    26305  15863  13677   2463  -3150
-1438       C
ATOM   5104  N   LEU A 682       3.632  -8.119  -2.531  1.00103.40
N
ANISOU 5104  N   LEU A 682    19468  11076   8744   2121  -1487
-1216       N
ATOM   5105  CA  LEU A 682       4.864  -8.498  -1.838  1.00121.67
C
ANISOU 5105  CA  LEU A 682    21593  13534  11102   2056  -1146
-1109       C
ATOM   5106  C   LEU A 682       4.880  -8.045  -0.381  1.00105.14
C
ANISOU 5106  C   LEU A 682    18862  11787   9301   1757  -1053
```

```
-1189       C
ATOM   5107  O   LEU A 682       5.295  -8.804   0.503  1.00 77.59
O
ANISOU 5107  O   LEU A 682    15131   8489   5861   1594   -992
-1162       O
ATOM   5108  CB  LEU A 682       6.077  -7.945  -2.585  1.00123.67
C
ANISOU 5108  CB  LEU A 682    22105  13625  11258   2314   -748
-952       C
ATOM   5109  CG  LEU A 682       6.340  -8.590  -3.947  1.00 95.03
C
ANISOU 5109  CG  LEU A 682    19145   9662   7299   2649   -759
-831       C
ATOM   5110  CD1 LEU A 682       7.421  -7.837  -4.708  1.00143.69
C
ANISOU 5110  CD1 LEU A 682    25528  15675  13391   2914   -350
-668       C
ATOM   5111  CD2 LEU A 682       6.719 -10.060  -3.776  1.00 83.56
C
ANISOU 5111  CD2 LEU A 682    17856   8215   5678   2652   -797
-751       C
ATOM   5112  N   ASN A 683       4.447  -6.814  -0.109  1.00103.12
N
ANISOU 5112  N   ASN A 683    18357  11601   9221   1700  -1036
-1287       N
ATOM   5113  CA  ASN A 683       4.289  -6.368   1.274  1.00101.61
C
ANISOU 5113  CA  ASN A 683    17607  11716   9282   1431   -994
-1384       C
ATOM   5114  C   ASN A 683       3.292  -7.235   2.041  1.00116.02
C
ANISOU 5114  C   ASN A 683    19186  13731  11167   1221  -1310
-1473       C
ATOM   5115  O   ASN A 683       3.586  -7.713   3.143  1.00131.19
O
ANISOU 5115  O   ASN A 683    20770  15884  13192   1019  -1243
-1479       O
ATOM   5116  CB  ASN A 683       3.860  -4.900   1.299  1.00101.73
C
ANISOU 5116  CB  ASN A 683    17500  11724   9430   1453   -954
-1477       C
ATOM   5117  CG  ASN A 683       3.589  -4.397   2.702  1.00150.49
C
ANISOU 5117  CG  ASN A 683    23155  18188  15835   1206   -932
-1587       C
ATOM   5118  OD1 ASN A 683       4.449  -4.482   3.577  1.00171.96
O
ANISOU 5118  OD1 ASN A 683    25615  21073  18650   1050   -722
-1555       O
ATOM   5119  ND2 ASN A 683       2.388  -3.875   2.924  1.00152.11
N
ANISOU 5119  ND2 ASN A 683    23211  18463  16120   1185  -1151
-1710       N
ATOM   5120  N   ASP A 684       2.104  -7.446   1.467  1.00114.08
```

```
N
ANISOU 5120  N   ASP A 684    19098  13392  10854   1263  -1659
-1533       N
ATOM   5121  CA  ASP A 684       1.071  -8.261   2.105  1.00104.44
C
ANISOU 5121  CA  ASP A 684    17643  12346   9695   1049  -1980
-1596       C
ATOM   5122  C   ASP A 684       1.540  -9.688   2.381  1.00112.41
C
ANISOU 5122  C   ASP A 684    18743  13369  10600    944  -2000
-1521       C
ATOM   5123  O   ASP A 684       1.211 -10.265   3.425  1.00155.68
O
ANISOU 5123  O   ASP A 684    23882  19079  16192    708  -2081
-1553       O
ATOM   5124  CB  ASP A 684      -0.190  -8.267   1.239  1.00102.65
C
ANISOU 5124  CB  ASP A 684    17622  11989   9391   1126  -2365
-1640       C
ATOM   5125  CG  ASP A 684      -1.306  -9.111   1.828  1.00112.49
C
ANISOU 5125  CG  ASP A 684    18613  13418  10710    883  -2714
-1678       C
ATOM   5126  OD1 ASP A 684      -1.258 -10.354   1.692  1.00109.12
O
ANISOU 5126  OD1 ASP A 684    18389  12916  10157    794  -2867
-1626       O
ATOM   5127  OD2 ASP A 684      -2.233  -8.528   2.428  1.00145.00
O
ANISOU 5127  OD2 ASP A 684    22336  17753  15006    786  -2829
-1750       O
ATOM   5128  N   GLY A 685       2.298 -10.280   1.458  1.00 85.61
N
ANISOU 5128  N   GLY A 685    15828   9724   6974   1137  -1919
-1415       N
ATOM   5129  CA  GLY A 685       2.857 -11.602   1.686  1.00 77.61
C
ANISOU 5129  CA  GLY A 685    14957   8701   5830   1087  -1897
-1331       C
ATOM   5130  C   GLY A 685       3.908 -11.681   2.775  1.00108.06
C
ANISOU 5130  C   GLY A 685    18467  12792   9798    986  -1567
-1283       C
ATOM   5131  O   GLY A 685       4.141 -12.769   3.312  1.00 75.46
O
ANISOU 5131  O   GLY A 685    14321   8739   5612    882  -1586
-1242       O
ATOM   5132  N   THR A 686       4.547 -10.563   3.116  1.00124.66
N
ANISOU 5132  N   THR A 686    20310  15003  12050   1007  -1277
-1286       N
ATOM   5133  CA  THR A 686       5.654 -10.574   4.063  1.00116.98
C
ANISOU 5133  CA  THR A 686    19031  14244  11173    921   -968
```

```
-1225       C
ATOM   5134  C   THR A 686       5.322 -10.019   5.443  1.00106.19
C
ANISOU 5134  C   THR A 686    17106  13183  10061    659   -960
-1341       C
ATOM   5135  O   THR A 686       6.127 -10.201   6.363  1.00 99.99
O
ANISOU 5135  O   THR A 686    16049  12596   9346    552   -766
-1304       O
ATOM   5136  CB  THR A 686       6.848  -9.795   3.492  1.00101.03
C
ANISOU 5136  CB  THR A 686    17133  12129   9125   1113   -616
-1108       C
ATOM   5137  OG1 THR A 686       6.392  -8.558   2.931  1.00119.23
O
ANISOU 5137  OG1 THR A 686    19492  14314  11497   1186   -634
-1176       O
ATOM   5138  CG2 THR A 686       7.542 -10.612   2.413  1.00 93.61
C
ANISOU 5138  CG2 THR A 686    16701  10953   7913   1386   -521
-945       C
ATOM   5139  N   ILE A 687       4.175  -9.353   5.629  1.00 72.22
N
ANISOU 5139  N   ILE A 687    12628   8928   5882    573  -1163
-1472       N
ATOM   5140  CA  ILE A 687       3.919  -8.662   6.892  1.00 78.12
C
ANISOU 5140  CA  ILE A 687    12890   9943   6850    378  -1114
-1576       C
ATOM   5141  C   ILE A 687       3.304  -9.556   7.965  1.00 87.53
C
ANISOU 5141  C   ILE A 687    13790  11362   8107    159  -1276
-1620       C
ATOM   5142  O   ILE A 687       3.003  -9.064   9.062  1.00 78.38
O
ANISOU 5142  O   ILE A 687    12241  10429   7113     10  -1246
-1705       O
ATOM   5143  CB  ILE A 687       3.069  -7.385   6.674  1.00 71.69
C
ANISOU 5143  CB  ILE A 687    12011   9092   6135    428  -1193
-1681       C
ATOM   5144  CG1 ILE A 687       1.821  -7.606   5.791  1.00102.17
C
ANISOU 5144  CG1 ILE A 687    16088  12815   9916    517  -1525
-1712       C
ATOM   5145  CG2 ILE A 687       3.937  -6.260   6.129  1.00 72.00
C
ANISOU 5145  CG2 ILE A 687    12198   8984   6174    569   -930
-1647       C
ATOM   5146  CD1 ILE A 687       0.882  -8.740   6.165  1.00117.28
C
ANISOU 5146  CD1 ILE A 687    17891  14844  11826    356  -1823
-1724       C
ATOM   5147  N   MET A 688       3.104 -10.845   7.697  1.00 95.99
```

```
N
ANISOU 5147  N   MET A 688    15062  12367   9042    139  -1442
-1562       N
ATOM   5148  CA  MET A 688       2.692 -11.740   8.770  1.00 79.28
C
ANISOU 5148  CA  MET A 688    12677  10462   6982    -80  -1549
-1582       C
ATOM   5149  C   MET A 688       3.891 -12.301   9.519  1.00 94.62
C
ANISOU 5149  C   MET A 688    14526  12528   8898   -120  -1302
-1512       C
ATOM   5150  O   MET A 688       3.723 -13.126  10.423  1.00149.01
O
ANISOU 5150  O   MET A 688    21231  19580  15806   -282  -1354
-1514       O
ATOM   5151  CB  MET A 688       1.826 -12.875   8.217  1.00 91.76
C
ANISOU 5151  CB  MET A 688    14514  11914   8437   -128  -1872
-1554       C
ATOM   5152  CG  MET A 688       0.532 -12.377   7.603  1.00136.71
C
ANISOU 5152  CG  MET A 688    20226  17542  14177   -121  -2160
-1616       C
ATOM   5153  SD  MET A 688      -0.487 -11.472   8.785  1.00 88.72
S
ANISOU 5153  SD  MET A 688    13552  11790   8367   -282  -2197
-1723       S
ATOM   5154  CE  MET A 688      -1.745 -10.821   7.692  1.00139.77
C
ANISOU 5154  CE  MET A 688    20147  18127  14831   -166  -2498
-1758       C
ATOM   5155  N   THR A 689       5.091 -11.850   9.153  1.00 79.87
N
ANISOU 5155  N   THR A 689    12770  10593   6985     29  -1032
-1438       N
ATOM   5156  CA  THR A 689       6.249 -11.891  10.039  1.00 89.53
C
ANISOU 5156  CA  THR A 689    13757  12008   8254    -23   -768
-1386       C
ATOM   5157  C   THR A 689       6.001 -11.065  11.295  1.00117.39
C
ANISOU 5157  C   THR A 689    16822  15788  11994   -213   -729
-1504       C
ATOM   5158  O   THR A 689       6.358 -11.480  12.404  1.00158.71
O
ANISOU 5158  O   THR A 689    21792  21238  17273   -345   -660
-1507       O
ATOM   5159  CB  THR A 689       7.494 -11.401   9.300  1.00112.72
C
ANISOU 5159  CB  THR A 689    16873  14831  11126    167   -498
-1266       C
ATOM   5160  OG1 THR A 689       7.694 -12.199   8.128  1.00 93.66
O
ANISOU 5160  OG1 THR A 689    14931  12171   8484    382   -524
```

```
-1150       O
ATOM   5161  CG2 THR A 689       8.721 -11.515  10.192  1.00 67.82
C
ANISOU 5161  CG2 THR A 689    10919   9365   5483    106   -247
-1187       C
ATOM   5162  N   ILE A 690       5.398  -9.883  11.129  1.00 97.29
N
ANISOU 5162  N   ILE A 690    14204  13204   9559   -207   -771
-1600       N
ATOM   5163  CA  ILE A 690       5.406  -8.838  12.151  1.00 90.90
C
ANISOU 5163  CA  ILE A 690    13046  12574   8920   -330   -676
-1702       C
ATOM   5164  C   ILE A 690       4.767  -9.330  13.443  1.00119.89
C
ANISOU 5164  C   ILE A 690    16394  16491  12669   -510   -775
-1775       C
ATOM   5165  O   ILE A 690       5.257  -9.038  14.542  1.00120.24
O
ANISOU 5165  O   ILE A 690    16168  16724  12794   -624   -650
-1813       O
ATOM   5166  CB  ILE A 690       4.679  -7.585  11.624  1.00 94.44
C
ANISOU 5166  CB  ILE A 690    13550  12899   9433   -251   -734
-1790       C
ATOM   5167  CG1 ILE A 690       5.250  -7.164  10.272  1.00123.16
C
ANISOU 5167  CG1 ILE A 690    17548  16271  12976    -58   -638
-1708       C
ATOM   5168  CG2 ILE A 690       4.777  -6.440  12.626  1.00 67.10
C
ANISOU 5168  CG2 ILE A 690     9806   9576   6113   -354   -619
-1893       C
ATOM   5169  CD1 ILE A 690       6.715  -6.910  10.298  1.00139.94
C
ANISOU 5169  CD1 ILE A 690    19674  18399  15099    -54   -359
-1607       C
ATOM   5170  N   SER A 691       3.658 -10.063  13.338  1.00113.43
N
ANISOU 5170  N   SER A 691    15599  15674  11826   -545  -1007
-1789       N
ATOM   5171  CA  SER A 691       3.033 -10.631  14.529  1.00 97.08
C
ANISOU 5171  CA  SER A 691    13228  13834   9822   -715  -1089
-1830       C
ATOM   5172  C   SER A 691       3.997 -11.539  15.285  1.00106.67
C
ANISOU 5172  C   SER A 691    14377  15173  10981   -791   -958
-1766       C
ATOM   5173  O   SER A 691       3.985 -11.576  16.521  1.00166.58
O
ANISOU 5173  O   SER A 691    21670  22981  18640   -916   -905
-1812       O
ATOM   5174  CB  SER A 691       1.768 -11.399  14.143  1.00 96.48
```

```
C
ANISOU 5174  CB  SER A 691    13216  13722   9722   -760  -1368
-1816       C
ATOM   5175  OG  SER A 691       0.838 -10.556  13.487  1.00138.24
O
ANISOU 5175  OG  SER A 691    18532  18930  15064   -677  -1503
-1867       O
ATOM   5176  N   LYS A 692       4.844 -12.270  14.564  1.00101.97
N
ANISOU 5176  N   LYS A 692    14064  14440  10240   -693   -896
-1654       N
ATOM   5177  CA  LYS A 692       5.704 -13.293  15.144  1.00113.29
C
ANISOU 5177  CA  LYS A 692    15484  15976  11585   -724   -788
-1571       C
ATOM   5178  C   LYS A 692       7.110 -12.784  15.463  1.00136.43
C
ANISOU 5178  C   LYS A 692    18307  18998  14532   -679   -527
-1523       C
ATOM   5179  O   LYS A 692       8.002 -13.591  15.740  1.00155.91
O
ANISOU 5179  O   LYS A 692    20797  21539  16903   -648   -414
-1423       O
ATOM   5180  CB  LYS A 692       5.769 -14.497  14.202  1.00 99.63
C
ANISOU 5180  CB  LYS A 692    14153  14045   9658   -621   -878
-1460       C
ATOM   5181  CG  LYS A 692       4.388 -14.999  13.797  1.00 89.80
C
ANISOU 5181  CG  LYS A 692    13031  12691   8396   -698  -1176
-1497       C
ATOM   5182  CD  LYS A 692       4.447 -16.313  13.036  1.00107.81
C
ANISOU 5182  CD  LYS A 692    15738  14765  10462   -635  -1291
-1396       C
ATOM   5183  CE  LYS A 692       3.054 -16.753  12.593  1.00127.75
C
ANISOU 5183  CE  LYS A 692    18381  17177  12981   -752  -1626
-1427       C
ATOM   5184  NZ  LYS A 692       2.524 -15.923  11.472  1.00130.16
N
ANISOU 5184  NZ  LYS A 692    18850  17303  13301   -641  -1751
-1463       N
ATOM   5185  N   ASP A 693       7.324 -11.470  15.427  1.00126.72
N
ANISOU 5185  N   ASP A 693    16965  17765  13417   -678   -435
-1581       N
ATOM   5186  CA  ASP A 693       8.573 -10.855  15.866  1.00114.41
C
ANISOU 5186  CA  ASP A 693    15245  16316  11908   -700   -218
-1542       C
ATOM   5187  C   ASP A 693       8.791 -11.054  17.373  1.00133.95
C
ANISOU 5187  C   ASP A 693    17387  19064  14443   -862   -189
```

```
-1595       C
ATOM   5188  O   ASP A 693       7.950 -11.601  18.093  1.00191.53
O
ANISOU 5188  O   ASP A 693    24570  26458  21743   -948   -314
-1663       O
ATOM   5189  CB  ASP A 693       8.579  -9.372  15.497  1.00108.77
C
ANISOU 5189  CB  ASP A 693    14522  15503  11302   -695   -163
-1607       C
ATOM   5190  CG  ASP A 693       9.980  -8.795  15.411  1.00112.27
C
ANISOU 5190  CG  ASP A 693    14916  15971  11771   -694     59
-1508       C
ATOM   5191  OD1 ASP A 693      10.947  -9.530  15.702  1.00111.50
O
ANISOU 5191  OD1 ASP A 693    14747  16003  11615   -686    170
-1388       O
ATOM   5192  OD2 ASP A 693      10.114  -7.604  15.058  1.00112.68
O
ANISOU 5192  OD2 ASP A 693    14995  15918  11901   -702    121
-1540       O
ATOM   5193  N   ARG A 694       9.950 -10.593  17.850  1.00117.12
N
ANISOU 5193  N   ARG A 694    15093  17056  12353   -908    -25
-1553       N
ATOM   5194  CA  ARG A 694      10.460 -10.817  19.202  1.00127.99
C
ANISOU 5194  CA  ARG A 694    16185  18690  13757  -1038     19
-1575       C
ATOM   5195  C   ARG A 694      10.975  -9.504  19.794  1.00123.05
C
ANISOU 5195  C   ARG A 694    15359  18137  13256  -1168     87
-1649       C
ATOM   5196  O   ARG A 694      12.034  -9.450  20.427  1.00109.04
O
ANISOU 5196  O   ARG A 694    13406  16528  11497  -1245    182
-1593       O
ATOM   5197  CB  ARG A 694      11.563 -11.878  19.175  1.00151.39
C
ANISOU 5197  CB  ARG A 694    19172  21749  16600   -954    135
-1404       C
ATOM   5198  CG  ARG A 694      11.999 -12.485  20.512  1.00147.14
C
ANISOU 5198  CG  ARG A 694    18389  21476  16042  -1046    153
-1407       C
ATOM   5199  CD  ARG A 694      10.868 -13.117  21.304  1.00136.94
C
ANISOU 5199  CD  ARG A 694    17053  20243  14734  -1122      9
-1516       C
ATOM   5200  NE  ARG A 694      11.394 -13.787  22.492  1.00176.00
N
ANISOU 5200  NE  ARG A 694    21812  25427  19632  -1175     48
-1496       N
ATOM   5201  CZ  ARG A 694      10.649 -14.369  23.426  1.00146.94
```

```
C
ANISOU 5201  CZ  ARG A 694    18048  21850  15932  -1251    -36
-1570       C
ATOM   5202  NH1 ARG A 694      11.227 -14.986  24.448  1.00107.45
N
ANISOU 5202  NH1 ARG A 694    12905  17055  10868  -1274     12
-1540       N
ATOM   5203  NH2 ARG A 694       9.328 -14.365  23.323  1.00131.51
N
ANISOU 5203  NH2 ARG A 694    16147  19802  14019  -1295   -165
-1657       N
ATOM   5204  N   VAL A 695      10.231  -8.415  19.576  1.00126.81
N
ANISOU 5204  N   VAL A 695    15882  18485  13817  -1192     29
-1769       N
ATOM   5205  CA  VAL A 695      10.589  -7.118  20.143  1.00129.64
C
ANISOU 5205  CA  VAL A 695    16115  18866  14276  -1322     72
-1858       C
ATOM   5206  C   VAL A 695      10.618  -7.156  21.667  1.00117.19
C
ANISOU 5206  C   VAL A 695    14292  17515  12719  -1467     35
-1954       C
ATOM   5207  O   VAL A 695       9.811  -7.840  22.310  1.00126.71
O
ANISOU 5207  O   VAL A 695    15431  18823  13892  -1460    -49
-2013       O
ATOM   5208  CB  VAL A 695       9.618  -6.036  19.642  1.00156.55
C
ANISOU 5208  CB  VAL A 695    19664  22080  17737  -1280     10
-1975       C
ATOM   5209  CG1 VAL A 695       9.786  -5.829  18.145  1.00193.44
C
ANISOU 5209  CG1 VAL A 695    24594  26519  22385  -1141     63
-1876       C
ATOM   5210  CG2 VAL A 695       8.182  -6.420  19.971  1.00169.54
C
ANISOU 5210  CG2 VAL A 695    21289  23757  19372  -1234   -137
-2077       C
ATOM   5211  N   LYS A 696      11.576  -6.408  22.259  1.00145.68
N
ANISOU 5211  N   LYS A 696    17771  21202  16379  -1610     94
-1963       N
ATOM   5212  CA  LYS A 696      11.711  -6.337  23.710  1.00147.83
C
ANISOU 5212  CA  LYS A 696    17842  21671  16654  -1748     49
-2059       C
ATOM   5213  C   LYS A 696      10.650  -5.394  24.284  1.00107.67
C
ANISOU 5213  C   LYS A 696    12794  16516  11599  -1774    -31
-2250       C
ATOM   5214  O   LYS A 696      10.348  -4.359  23.681  1.00 89.89
O
ANISOU 5214  O   LYS A 696    10692  14071   9393  -1755    -27
```

```
-2303       O
ATOM   5215  CB  LYS A 696      13.114  -5.872  24.109  1.00107.77
C
ANISOU 5215  CB  LYS A 696    12628  16702  11618  -1906    108
-1993       C
ATOM   5216  CG  LYS A 696      13.527  -4.504  23.588  1.00108.01
C
ANISOU 5216  CG  LYS A 696    12749  16556  11735  -2007    144
-2007       C
ATOM   5217  CD  LYS A 696      14.888  -4.109  24.151  1.00130.54
C
ANISOU 5217  CD  LYS A 696    15418  19548  14635  -2215    168
-1935       C
ATOM   5218  CE  LYS A 696      15.339  -2.744  23.656  1.00152.57
C
ANISOU 5218  CE  LYS A 696    18306  22149  17514  -2357    200
-1934       C
ATOM   5219  NZ  LYS A 696      16.646  -2.347  24.255  1.00 73.31
N
ANISOU 5219  NZ  LYS A 696     8062  12257   7533  -2605    192
-1856       N
ATOM   5220  N   PRO A 697      10.057  -5.737  25.429  1.00 85.09
N
ANISOU 5220  N   PRO A 697     9820  13808   8702  -1792    -91
-2346       N
ATOM   5221  CA  PRO A 697       9.058  -4.850  26.043  1.00 86.43
C
ANISOU 5221  CA  PRO A 697    10028  13930   8881  -1778   -143
-2511       C
ATOM   5222  C   PRO A 697       9.591  -3.465  26.391  1.00 67.36
C
ANISOU 5222  C   PRO A 697     7683  11418   6492  -1898   -138
-2606       C
ATOM   5223  O   PRO A 697      10.749  -3.297  26.778  1.00111.06
O
ANISOU 5223  O   PRO A 697    13144  17019  12035  -2055   -127
-2573       O
ATOM   5224  CB  PRO A 697       8.643  -5.617  27.303  1.00103.20
C
ANISOU 5224  CB  PRO A 697    11996  16271  10946  -1784   -175
-2556       C
ATOM   5225  CG  PRO A 697       9.851  -6.429  27.648  1.00103.14
C
ANISOU 5225  CG  PRO A 697    11864  16421  10903  -1873   -146
-2454       C
ATOM   5226  CD  PRO A 697      10.429  -6.848  26.321  1.00 77.02
C
ANISOU 5226  CD  PRO A 697     8632  13015   7616  -1823    -94
-2299       C
ATOM   5227  N   SER A 698       8.715  -2.474  26.248  1.00 68.00
N
ANISOU 5227  N   SER A 698     7916  11340   6582  -1821   -156
-2719       N
ATOM   5228  CA  SER A 698       8.944  -1.155  26.823  1.00 69.63
```

```
C
ANISOU 5228  CA   SER A 698     8240  11439   6777  -1917   -170
-2845       C
ATOM   5229  C    SER A 698       8.968  -1.256  28.349  1.00 78.58
C
ANISOU 5229  C    SER A 698     9273  12745   7837  -1986   -215
-2943       C
ATOM   5230  O    SER A 698       8.136  -1.956  28.937  1.00 89.89
O
ANISOU 5230  O    SER A 698    10605  14323   9225  -1873   -222
-2965       O
ATOM   5231  CB   SER A 698       7.862  -0.174  26.368  1.00 70.29
C
ANISOU 5231  CB   SER A 698     8532  11322   6855  -1761   -169
-2939       C
ATOM   5232  OG   SER A 698       8.031   1.096  26.978  1.00 72.08
O
ANISOU 5232  OG   SER A 698     8928  11419   7041  -1838   -183
-3069       O
ATOM   5233  N    PRO A 699       9.903  -0.584  29.023  1.00 92.63
N
ANISOU 5233  N    PRO A 699    11082  14514   9599  -2177   -253
-2996       N
ATOM   5234  CA   PRO A 699       9.947  -0.676  30.491  1.00 91.34
C
ANISOU 5234  CA   PRO A 699    10858  14503   9342  -2231   -311
-3096       C
ATOM   5235  C    PRO A 699       8.860   0.137  31.184  1.00 75.43
C
ANISOU 5235  C    PRO A 699     9025  12397   7237  -2095   -321
-3261       C
ATOM   5236  O    PRO A 699       8.150  -0.386  32.049  1.00 72.88
O
ANISOU 5236  O    PRO A 699     8626  12225   6840  -1975   -312
-3303       O
ATOM   5237  CB   PRO A 699      11.342  -0.137  30.839  1.00100.14
C
ANISOU 5237  CB   PRO A 699    11968  15611  10471  -2499   -377
-3089       C
ATOM   5238  CG   PRO A 699      11.748   0.696  29.670  1.00 80.62
C
ANISOU 5238  CG   PRO A 699     9631  12912   8090  -2574   -342
-3035       C
ATOM   5239  CD   PRO A 699      11.061   0.134  28.462  1.00108.18
C
ANISOU 5239  CD   PRO A 699    13115  16354  11633  -2370   -250
-2940       C
ATOM   5240  N    THR A 700       8.717   1.410  30.821  1.00 74.64
N
ANISOU 5240  N    THR A 700     9180  12049   7131  -2095   -324
-3343       N
ATOM   5241  CA   THR A 700       7.558   2.180  31.246  1.00 75.68
C
ANISOU 5241  CA   THR A 700     9511  12076   7168  -1893   -304
```

```
-3475       C
ATOM   5242  C   THR A 700       6.284   1.568  30.660  1.00 98.16
C
ANISOU 5242  C   THR A 700    12252  14995  10051  -1635   -236
-3413       C
ATOM   5243  O   THR A 700       6.307   1.014  29.556  1.00116.64
O
ANISOU 5243  O   THR A 700    14498  17330  12491  -1623   -219
-3296       O
ATOM   5244  CB  THR A 700       7.698   3.642  30.820  1.00 98.77
C
ANISOU 5244  CB  THR A 700    12764  14692  10072  -1933   -314
-3559       C
ATOM   5245  OG1 THR A 700       6.531   4.379  31.209  1.00101.15
O
ANISOU 5245  OG1 THR A 700    13279  14894  10259  -1684   -277
-3676       O
ATOM   5246  CG2 THR A 700       7.892   3.748  29.310  1.00 81.97
C
ANISOU 5246  CG2 THR A 700    10657  12421   8066  -1940   -269
-3449       C
ATOM   5247  N   PRO A 701       5.164   1.627  31.383  1.00 93.52
N
ANISOU 5247  N   PRO A 701    11673  14482   9377  -1426   -202
-3478       N
ATOM   5248  CA  PRO A 701       3.861   1.509  30.717  1.00 89.13
C
ANISOU 5248  CA  PRO A 701    11072  13937   8857  -1178   -152
-3430       C
ATOM   5249  C   PRO A 701       3.738   2.464  29.537  1.00141.98
C
ANISOU 5249  C   PRO A 701    17983  20374  15590  -1114   -146
-3437       C
ATOM   5250  O   PRO A 701       4.380   3.516  29.488  1.00168.24
O
ANISOU 5250  O   PRO A 701    21563  23483  18878  -1210   -157
-3517       O
ATOM   5251  CB  PRO A 701       2.864   1.848  31.833  1.00 87.30
C
ANISOU 5251  CB  PRO A 701    10880  13789   8500   -968    -99
-3513       C
ATOM   5252  CG  PRO A 701       3.640   2.722  32.769  1.00 77.54
C
ANISOU 5252  CG  PRO A 701     9888  12432   7140  -1081   -129
-3651       C
ATOM   5253  CD  PRO A 701       5.021   2.106  32.768  1.00 76.46
C
ANISOU 5253  CD  PRO A 701     9631  12352   7068  -1387   -205
-3606       C
ATOM   5254  N   ASP A 702       2.881   2.092  28.585  1.00131.66
N
ANISOU 5254  N   ASP A 702    16588  19085  14353   -956   -138
-3351       N
ATOM   5255  CA  ASP A 702       2.591   2.917  27.420  1.00123.43
```

```
C
ANISOU 5255  CA  ASP A 702    15747  17816  13336   -845   -132
-3348       C
ATOM   5256  C   ASP A 702       1.124   2.763  27.047  1.00122.44
C
ANISOU 5256  C   ASP A 702    15526  17776  13220   -569   -127
-3302       C
ATOM   5257  O   ASP A 702       0.565   1.668  27.148  1.00 92.00
O
ANISOU 5257  O   ASP A 702    11400  14142   9415   -546   -153
-3213       O
ATOM   5258  CB  ASP A 702       3.495   2.537  26.237  1.00118.24
C
ANISOU 5258  CB  ASP A 702    15090  17057  12777  -1003   -156
-3248       C
ATOM   5259  CG  ASP A 702       3.305   3.445  25.037  1.00135.76
C
ANISOU 5259  CG  ASP A 702    17555  19018  15010   -893   -141
-3246       C
ATOM   5260  OD1 ASP A 702       4.282   4.122  24.654  1.00153.80
O
ANISOU 5260  OD1 ASP A 702    20028  21104  17304  -1042   -119
-3257       O
ATOM   5261  OD2 ASP A 702       2.195   3.474  24.464  1.00177.97
O
ANISOU 5261  OD2 ASP A 702    22900  24363  20357   -664   -155
-3222       O
ATOM   5262  N   SER A 703       0.500   3.862  26.619  1.00128.46
N
ANISOU 5262  N   SER A 703    16512  18366  13932   -363    -99
-3354       N
ATOM   5263  CA  SER A 703      -0.948   3.907  26.469  1.00125.78
C
ANISOU 5263  CA  SER A 703    16075  18135  13582    -68    -89
-3314       C
ATOM   5264  C   SER A 703      -1.399   3.961  25.011  1.00138.82
C
ANISOU 5264  C   SER A 703    17767  19679  15301     44   -146
-3237       C
ATOM   5265  O   SER A 703      -2.563   4.286  24.752  1.00182.53
O
ANISOU 5265  O   SER A 703    23272  25263  20817    310   -148
-3209       O
ATOM   5266  CB  SER A 703      -1.518   5.103  27.237  1.00150.56
C
ANISOU 5266  CB  SER A 703    19433  21198  16575    164     -4
-3422       C
ATOM   5267  OG  SER A 703      -1.153   5.054  28.606  1.00160.30
O
ANISOU 5267  OG  SER A 703    20664  22522  17722     82     39
-3497       O
ATOM   5268  N   TRP A 704      -0.500   3.690  24.056  1.00147.15
N
ANISOU 5268  N   TRP A 704    26923  13836  15149   -680   3594
```

```
1874       N
ATOM   5269  CA  TRP A 704      -0.815   3.710  22.620  1.00123.80
C
ANISOU 5269  CA  TRP A 704    23506  10986  12545   -642   3589
1887       C
ATOM   5270  C   TRP A 704      -1.115   5.126  22.121  1.00110.65
C
ANISOU 5270  C   TRP A 704    21827   9273  10941   -596   3601
1819       C
ATOM   5271  O   TRP A 704      -2.152   5.378  21.506  1.00 96.12
O
ANISOU 5271  O   TRP A 704    19832   7383   9305   -554   3878
1756       O
ATOM   5272  CB  TRP A 704      -1.958   2.750  22.275  1.00113.49
C
ANISOU 5272  CB  TRP A 704    22003   9663  11457   -642   3970
1856       C
ATOM   5273  CG  TRP A 704      -1.878   2.218  20.878  1.00123.37
C
ANISOU 5273  CG  TRP A 704    22782  11054  13038   -618   3850
1914       C
ATOM   5274  CD1 TRP A 704      -1.131   2.719  19.851  1.00102.79
C
ANISOU 5274  CD1 TRP A 704    19925   8577  10555   -594   3516
1972       C
ATOM   5275  CD2 TRP A 704      -2.526   1.046  20.368  1.00134.06
C
ANISOU 5275  CD2 TRP A 704    23879  12427  14631   -628   4064
1919       C
ATOM   5276  NE1 TRP A 704      -1.297   1.949  18.725  1.00129.73
N
ANISOU 5276  NE1 TRP A 704    22955  12089  14246   -583   3518
2009       N
ATOM   5277  CE2 TRP A 704      -2.146   0.913  19.017  1.00129.00
C
ANISOU 5277  CE2 TRP A 704    22849  11927  14238   -599   3845
1974       C
ATOM   5278  CE3 TRP A 704      -3.400   0.103  20.918  1.00124.68
C
ANISOU 5278  CE3 TRP A 704    22758  11141  13474   -667   4432
1877       C
ATOM   5279  CZ2 TRP A 704      -2.609  -0.125  18.210  1.00 85.81
C
ANISOU 5279  CZ2 TRP A 704    17067   6493   9043   -598   3977
1981       C
ATOM   5280  CZ3 TRP A 704      -3.858  -0.927  20.115  1.00110.76
C
ANISOU 5280  CZ3 TRP A 704    20663   9411  12008   -672   4560
1885       C
ATOM   5281  CH2 TRP A 704      -3.462  -1.032  18.776  1.00 86.92
C
ANISOU 5281  CH2 TRP A 704    17269   6526   9232   -633   4331
1933       C
ATOM   5282  N   LYS A 705      -0.200   6.054  22.393  1.00 91.49
```

```
N
ANISOU 5282  N   LYS A 705    19574   6838   8351   -604   3296
1833       N
ATOM   5283  CA  LYS A 705      -0.313   7.395  21.829  1.00 90.74
C
ANISOU 5283  CA  LYS A 705    19449   6693   8336   -567   3232
1796       C
ATOM   5284  C   LYS A 705      -0.198   7.372  20.307  1.00104.79
C
ANISOU 5284  C   LYS A 705    20761   8621  10434   -544   3071
1868       C
ATOM   5285  O   LYS A 705       0.901   7.252  19.754  1.00115.84
O
ANISOU 5285  O   LYS A 705    21965  10167  11882   -581   2716
1960       O
ATOM   5286  CB  LYS A 705       0.740   8.317  22.454  1.00 98.57
C
ANISOU 5286  CB  LYS A 705    20723   7635   9095   -598   2919
1799       C
ATOM   5287  CG  LYS A 705       2.115   7.681  22.659  1.00126.38
C
ANISOU 5287  CG  LYS A 705    24217  11278  12523   -652   2546
1891       C
ATOM   5288  CD  LYS A 705       2.260   7.094  24.069  1.00150.59
C
ANISOU 5288  CD  LYS A 705    27687  14255  15275   -676   2610
1869       C
ATOM   5289  CE  LYS A 705       2.432   8.172  25.133  1.00122.06
C
ANISOU 5289  CE  LYS A 705    24559  10467  11351   -684   2588
1784       C
ATOM   5290  NZ  LYS A 705       3.807   8.743  25.144  1.00 96.09
N
ANISOU 5290  NZ  LYS A 705    21322   7198   7989   -718   2142
1822       N
ATOM   5291  N   LEU A 706      -1.349   7.485  19.634  1.00114.20
N
ANISOU 5291  N   LEU A 706    21792   9758  11842   -485   3352
1821       N
ATOM   5292  CA  LEU A 706      -1.400   7.466  18.173  1.00 97.83
C
ANISOU 5292  CA  LEU A 706    19313   7805  10054   -453   3233
1885       C
ATOM   5293  C   LEU A 706      -0.600   8.606  17.555  1.00 83.86
C
ANISOU 5293  C   LEU A 706    17486   6075   8300   -461   2883
1946       C
ATOM   5294  O   LEU A 706       0.052   8.428  16.520  1.00 84.02
O
ANISOU 5294  O   LEU A 706    17208   6266   8452   -488   2636
2042       O
ATOM   5295  CB  LEU A 706      -2.852   7.517  17.693  1.00 85.31
C
ANISOU 5295  CB  LEU A 706    17631   6093   8690   -373   3615
```

```
1807       C
ATOM   5296  CG  LEU A 706      -3.771   6.363  18.105  1.00122.65
C
ANISOU 5296  CG  LEU A 706    22345  10775  13481   -381   4018
1739       C
ATOM   5297  CD1 LEU A 706      -5.175   6.574  17.558  1.00139.21
C
ANISOU 5297  CD1 LEU A 706    24325  12711  15855   -302   4400
1645       C
ATOM   5298  CD2 LEU A 706      -3.212   5.027  17.639  1.00135.29
C
ANISOU 5298  CD2 LEU A 706    23672  12568  15163   -426   3873
1826       C
ATOM   5299  N   LYS A 707      -0.667   9.794  18.151  1.00 85.47
N
ANISOU 5299  N   LYS A 707    17985   6112   8380   -446   2878
1889       N
ATOM   5300  CA  LYS A 707       0.043  10.946  17.604  1.00 84.90
C
ANISOU 5300  CA  LYS A 707    17875   6045   8340   -464   2561
1945       C
ATOM   5301  C   LYS A 707       1.552  10.722  17.527  1.00 83.79
C
ANISOU 5301  C   LYS A 707    17640   6078   8119   -566   2192
2036       C
ATOM   5302  O   LYS A 707       2.183  11.071  16.524  1.00108.66
O
ANISOU 5302  O   LYS A 707    20547   9342  11396   -606   1951
2126       O
ATOM   5303  CB  LYS A 707      -0.265  12.198  18.429  1.00 87.23
C
ANISOU 5303  CB  LYS A 707    18550   6090   8505   -435   2627
1853       C
ATOM   5304  CG  LYS A 707      -1.609  12.875  18.136  1.00102.83
C
ANISOU 5304  CG  LYS A 707    20569   7846  10655   -320   2901
1772       C
ATOM   5305  CD  LYS A 707      -2.816  11.981  18.397  1.00136.47
C
ANISOU 5305  CD  LYS A 707    24830  12040  14984   -266   3346
1686       C
ATOM   5306  CE  LYS A 707      -4.110  12.778  18.350  1.00159.88
C
ANISOU 5306  CE  LYS A 707    27907  14706  18134   -146   3643
1560       C
ATOM   5307  NZ  LYS A 707      -4.347  13.375  17.008  1.00 89.56
N
ANISOU 5307  NZ  LYS A 707    18719   5787   9524    -55   3455
1616       N
ATOM   5308  N   GLU A 708       2.151  10.151  18.573  1.00136.92
N
ANISOU 5308  N   GLU A 708    24575  12809  14638   -611   2151
2016       N
ATOM   5309  CA  GLU A 708       3.574   9.812  18.522  1.00154.56
```

```
C
ANISOU 5309  CA  GLU A 708    26726  15177  16825   -696   1818
2093       C
ATOM   5310  C   GLU A 708       3.913   8.769  17.455  1.00131.03
C
ANISOU 5310  C   GLU A 708    23344  12404  14037   -718   1739
2184       C
ATOM   5311  O   GLU A 708       4.889   8.932  16.714  1.00146.78
O
ANISOU 5311  O   GLU A 708    25145  14513  16111   -784   1484
2261       O
ATOM   5312  CB  GLU A 708       4.043   9.339  19.900  1.00128.48
C
ANISOU 5312  CB  GLU A 708    23763  11802  13253   -718   1794
2053       C
ATOM   5313  CG  GLU A 708       4.150  10.456  20.929  1.00 95.18
C
ANISOU 5313  CG  GLU A 708    19975   7388   8801   -725   1765
1972       C
ATOM   5314  CD  GLU A 708       4.854  10.018  22.199  1.00101.83
C
ANISOU 5314  CD  GLU A 708    21166   8171   9353   -756   1657
1951       C
ATOM   5315  OE1 GLU A 708       6.018   9.572  22.112  1.00 91.69
O
ANISOU 5315  OE1 GLU A 708    19795   6978   8066   -808   1362
2017       O
ATOM   5316  OE2 GLU A 708       4.246  10.130  23.283  1.00152.01
O
ANISOU 5316  OE2 GLU A 708    27905  14375  15475   -728   1869
1867       O
ATOM   5317  N   ILE A 709       3.134   7.689  17.357  1.00 96.33
N
ANISOU 5317  N   ILE A 709    18834   8046   9720   -672   1970
2173       N
ATOM   5318  CA  ILE A 709       3.397   6.703  16.305  1.00 79.16
C
ANISOU 5318  CA  ILE A 709    16298   6045   7733   -689   1907
2250       C
ATOM   5319  C   ILE A 709       3.144   7.276  14.910  1.00 91.31
C
ANISOU 5319  C   ILE A 709    17561   7667   9466   -680   1873
2296       C
ATOM   5320  O   ILE A 709       3.910   7.009  13.976  1.00 76.13
O
ANISOU 5320  O   ILE A 709    15394   5896   7637   -735   1688
2378       O
ATOM   5321  CB  ILE A 709       2.614   5.397  16.552  1.00 79.34
C
ANISOU 5321  CB  ILE A 709    16279   6060   7806   -650   2165
2224       C
ATOM   5322  CG1 ILE A 709       1.108   5.624  16.605  1.00 80.16
C
ANISOU 5322  CG1 ILE A 709    16439   6044   7976   -577   2532
```

```
2138       C
ATOM   5323  CG2 ILE A 709       3.100   4.715  17.827  1.00130.47
C
ANISOU 5323  CG2 ILE A 709    23019  12474  14078   -675   2132
2219       C
ATOM   5324  CD1 ILE A 709       0.331   4.401  17.035  1.00108.42
C
ANISOU 5324  CD1 ILE A 709    20023   9580  11590   -561   2825
2100       C
ATOM   5325  N   PHE A 710       2.076   8.059  14.735  1.00101.39
N
ANISOU 5325  N   PHE A 710    18894   8832  10798   -610   2054
2248       N
ATOM   5326  CA  PHE A 710       1.874   8.757  13.464  1.00121.20
C
ANISOU 5326  CA  PHE A 710    21200  11389  13461   -594   1981
2308       C
ATOM   5327  C   PHE A 710       3.021   9.710  13.141  1.00 86.71
C
ANISOU 5327  C   PHE A 710    16825   7075   9047   -684   1669
2384       C
ATOM   5328  O   PHE A 710       3.517   9.737  12.010  1.00 95.13
O
ANISOU 5328  O   PHE A 710    17654   8286  10205   -736   1523
2478       O
ATOM   5329  CB  PHE A 710       0.546   9.522  13.467  1.00 78.38
C
ANISOU 5329  CB  PHE A 710    15897   5775   8107   -486   2211
2240       C
ATOM   5330  CG  PHE A 710      -0.678   8.644  13.458  1.00 84.10
C
ANISOU 5330  CG  PHE A 710    16565   6440   8951   -401   2557
2170       C
ATOM   5331  CD1 PHE A 710      -1.943   9.212  13.487  1.00 79.82
C
ANISOU 5331  CD1 PHE A 710    16127   5687   8512   -292   2817
2090       C
ATOM   5332  CD2 PHE A 710      -0.571   7.261  13.402  1.00111.02
C
ANISOU 5332  CD2 PHE A 710    19813   9970  12400   -429   2634
2177       C
ATOM   5333  CE1 PHE A 710      -3.076   8.419  13.472  1.00 81.20
C
ANISOU 5333  CE1 PHE A 710    16233   5780   8839   -224   3175
2012       C
ATOM   5334  CE2 PHE A 710      -1.701   6.459  13.388  1.00 77.95
C
ANISOU 5334  CE2 PHE A 710    15560   5707   8350   -367   2975
2107       C
ATOM   5335  CZ  PHE A 710      -2.954   7.039  13.422  1.00 83.33
C
ANISOU 5335  CZ  PHE A 710    16332   6186   9145   -270   3260
2021       C
ATOM   5336  N   ALA A 711       3.456  10.503  14.122  1.00 78.56
```

```
N
ANISOU 5336  N   ALA A 711    16068   5915   7865   -713   1582
2341       N
ATOM   5337  CA  ALA A 711       4.621  11.365  13.927  1.00 78.73
C
ANISOU 5337  CA  ALA A 711    16100   5960   7852   -817   1301
2403       C
ATOM   5338  C   ALA A 711       5.885  10.577  13.597  1.00 90.93
C
ANISOU 5338  C   ALA A 711    17472   7682   9398   -920   1111
2470       C
ATOM   5339  O   ALA A 711       6.660  10.978  12.721  1.00105.66
O
ANISOU 5339  O   ALA A 711    19176   9643  11327  -1013    941
2555       O
ATOM   5340  CB  ALA A 711       4.846  12.226  15.169  1.00161.81
C
ANISOU 5340  CB  ALA A 711    26989  16287  18203   -827   1255
2325       C
ATOM   5341  N   THR A 712       6.121   9.463  14.292  1.00 87.09
N
ANISOU 5341  N   THR A 712    17034   7219   8837   -910   1143
2437       N
ATOM   5342  CA  THR A 712       7.253   8.602  13.958  1.00116.58
C
ANISOU 5342  CA  THR A 712    20615  11087  12594   -990    972
2496       C
ATOM   5343  C   THR A 712       7.168   8.057  12.534  1.00131.04
C
ANISOU 5343  C   THR A 712    22101  13096  14594  -1009   1000
2572       C
ATOM   5344  O   THR A 712       8.168   8.043  11.808  1.00148.51
O
ANISOU 5344  O   THR A 712    24169  15416  16841  -1115    833
2640       O
ATOM   5345  CB  THR A 712       7.349   7.452  14.961  1.00119.92
C
ANISOU 5345  CB  THR A 712    21175  11471  12918   -954   1009
2456       C
ATOM   5346  OG1 THR A 712       7.422   7.982  16.290  1.00138.36
O
ANISOU 5346  OG1 THR A 712    23874  13644  15053   -940    985
2388       O
ATOM   5347  CG2 THR A 712       8.597   6.623  14.696  1.00131.79
C
ANISOU 5347  CG2 THR A 712    22567  13062  14444  -1030    797
2514       C
ATOM   5348  N   GLY A 713       5.985   7.605  12.117  1.00143.42
N
ANISOU 5348  N   GLY A 713    23556  14682  16255   -918   1219
2554       N
ATOM   5349  CA  GLY A 713       5.833   7.068  10.771  1.00135.92
C
ANISOU 5349  CA  GLY A 713    22315  13888  15442   -929   1253
```

```
2617       C
ATOM   5350  C   GLY A 713       5.936   8.097   9.657  1.00 72.52
C
ANISOU 5350  C   GLY A 713    14173   5928   7452   -979   1164
2696       C
ATOM   5351  O   GLY A 713       6.613   7.869   8.651  1.00 71.74
O
ANISOU 5351  O   GLY A 713    13887   5987   7384  -1070   1070
2774       O
ATOM   5352  N   VAL A 714       5.265   9.240   9.814  1.00 73.49
N
ANISOU 5352  N   VAL A 714    14430   5926   7568   -927   1198
2683       N
ATOM   5353  CA  VAL A 714       5.261  10.257   8.761  1.00 91.07
C
ANISOU 5353  CA  VAL A 714    16578   8192   9835   -963   1108
2775       C
ATOM   5354  C   VAL A 714       6.662  10.792   8.461  1.00 95.87
C
ANISOU 5354  C   VAL A 714    17152   8880  10393  -1127    890
2852       C
ATOM   5355  O   VAL A 714       7.031  10.955   7.292  1.00128.84
O
ANISOU 5355  O   VAL A 714    21163  13199  14592  -1210    826
2953       O
ATOM   5356  CB  VAL A 714       4.276  11.392   9.117  1.00 85.73
C
ANISOU 5356  CB  VAL A 714    16094   7309   9172   -865   1173
2741       C
ATOM   5357  CG1 VAL A 714       4.746  12.188  10.327  1.00 76.43
C
ANISOU 5357  CG1 VAL A 714    15177   5969   7895   -897   1093
2680       C
ATOM   5358  CG2 VAL A 714       4.081  12.320   7.923  1.00 78.07
C
ANISOU 5358  CG2 VAL A 714    15045   6361   8259   -875   1083
2856       C
ATOM   5359  N   VAL A 715       7.465  11.077   9.490  1.00 74.81
N
ANISOU 5359  N   VAL A 715    14662   6117   7646  -1185    783
2805       N
ATOM   5360  CA  VAL A 715       8.833  11.536   9.241  1.00 77.70
C
ANISOU 5360  CA  VAL A 715    15013   6529   7980  -1356    590
2865       C
ATOM   5361  C   VAL A 715       9.680  10.460   8.565  1.00100.11
C
ANISOU 5361  C   VAL A 715    17650   9555  10832  -1457    546
2906       C
ATOM   5362  O   VAL A 715      10.471  10.757   7.661  1.00116.22
O
ANISOU 5362  O   VAL A 715    19573  11708  12879  -1610    460
2988       O
ATOM   5363  CB  VAL A 715       9.483  12.036  10.545  1.00 94.41
```

```
C
ANISOU 5363  CB  VAL A 715    17401   8471  10000  -1392    473
2793       C
ATOM   5364  CG1 VAL A 715       8.657  13.163  11.147  1.00137.66
C
ANISOU 5364  CG1 VAL A 715    23096  13752  15458  -1308    522
2747       C
ATOM   5365  CG2 VAL A 715       9.648  10.901  11.540  1.00131.14
C
ANISOU 5365  CG2 VAL A 715    22148  13101  14578  -1339    488
2713       C
ATOM   5366  N   LEU A 716       9.541   9.202   8.989  1.00 73.36
N
ANISOU 5366  N   LEU A 716    14236   6192   7445  -1390    612
2850       N
ATOM   5367  CA  LEU A 716      10.230   8.113   8.301  1.00109.28
C
ANISOU 5367  CA  LEU A 716    18605  10895  12022  -1477    580
2884       C
ATOM   5368  C   LEU A 716       9.760   7.954   6.859  1.00109.44
C
ANISOU 5368  C   LEU A 716    18391  11095  12094  -1496    679
2955       C
ATOM   5369  O   LEU A 716      10.578   7.763   5.951  1.00 71.43
O
ANISOU 5369  O   LEU A 716    13439   6435   7266  -1655    615
3015       O
ATOM   5370  CB  LEU A 716      10.047   6.810   9.080  1.00 71.93
C
ANISOU 5370  CB  LEU A 716    13918   6115   7298  -1383    634
2817       C
ATOM   5371  CG  LEU A 716      10.782   6.777  10.423  1.00 73.08
C
ANISOU 5371  CG  LEU A 716    14310   6107   7350  -1390    487
2765       C
ATOM   5372  CD1 LEU A 716      10.451   5.511  11.203  1.00121.03
C
ANISOU 5372  CD1 LEU A 716    20448  12119  13419  -1282    554
2716       C
ATOM   5373  CD2 LEU A 716      12.286   6.927  10.235  1.00 73.70
C
ANISOU 5373  CD2 LEU A 716    14404   6199   7401  -1575    249
2801       C
ATOM   5374  N   GLY A 717       8.449   8.040   6.624  1.00 92.41
N
ANISOU 5374  N   GLY A 717    16209   8920   9984  -1351    832
2946       N
ATOM   5375  CA  GLY A 717       7.944   7.958   5.263  1.00 79.17
C
ANISOU 5375  CA  GLY A 717    14355   7398   8330  -1356    907
3016       C
ATOM   5376  C   GLY A 717       8.312   9.149   4.397  1.00 89.51
C
ANISOU 5376  C   GLY A 717    15642   8777   9590  -1466    807
```

```
3123       C
ATOM   5377  O   GLY A 717       8.623   8.992   3.213  1.00 83.57
O
ANISOU 5377  O   GLY A 717    14740   8219   8793  -1573    801
3200       O
ATOM   5378  N   GLY A 718       8.279  10.353   4.970  1.00106.94
N
ANISOU 5378  N   GLY A 718    18012  10826  11793  -1451    737
3130       N
ATOM   5379  CA  GLY A 718       8.717  11.532   4.235  1.00103.63
C
ANISOU 5379  CA  GLY A 718    17596  10437  11342  -1566    638
3240       C
ATOM   5380  C   GLY A 718      10.175  11.493   3.818  1.00 86.78
C
ANISOU 5380  C   GLY A 718    15383   8435   9154  -1801    540
3286       C
ATOM   5381  O   GLY A 718      10.515  11.848   2.686  1.00 85.09
O
ANISOU 5381  O   GLY A 718    15066   8374   8892  -1930    526
3389       O
ATOM   5382  N   TYR A 719      11.057  11.065   4.723  1.00 87.46
N
ANISOU 5382  N   TYR A 719    15536   8458   9235  -1872    468
3210       N
ATOM   5383  CA  TYR A 719      12.457  10.835   4.370  1.00113.92
C
ANISOU 5383  CA  TYR A 719    18814  11920  12550  -2119    373
3236       C
ATOM   5384  C   TYR A 719      12.609   9.827   3.235  1.00124.06
C
ANISOU 5384  C   TYR A 719    19869  13465  13805  -2215    444
3269       C
ATOM   5385  O   TYR A 719      13.370  10.054   2.288  1.00143.87
O
ANISOU 5385  O   TYR A 719    22257  16147  16260  -2440    426
3339       O
ATOM   5386  CB  TYR A 719      13.226  10.377   5.613  1.00 96.34
C
ANISOU 5386  CB  TYR A 719    16730   9549  10326  -2146    257
3144       C
ATOM   5387  CG  TYR A 719      14.631   9.870   5.354  1.00 83.90
C
ANISOU 5387  CG  TYR A 719    15083   8060   8736  -2408    137
3155       C
ATOM   5388  CD1 TYR A 719      15.730  10.711   5.489  1.00 94.46
C
ANISOU 5388  CD1 TYR A 719    16500   9323  10067  -2634     -3
3181       C
ATOM   5389  CD2 TYR A 719      14.863   8.538   5.028  1.00 74.42
C
ANISOU 5389  CD2 TYR A 719    13650   7039   7586  -2401    154
3079       C
ATOM   5390  CE1 TYR A 719      17.015  10.249   5.267  1.00101.37
```

```
C
ANISOU 5390  CE1 TYR A 719    17160  10342  11013  -2828   -115
3094       C
ATOM   5391  CE2 TYR A 719      16.142   8.067   4.808  1.00 75.09
C
ANISOU 5391  CE2 TYR A 719    13475   7293   7763  -2549     32
2958       C
ATOM   5392  CZ  TYR A 719      17.215   8.925   4.929  1.00104.84
C
ANISOU 5392  CZ  TYR A 719    17283  11011  11540  -2762    -99
2961       C
ATOM   5393  OH  TYR A 719      18.490   8.456   4.712  1.00 77.75
O
ANISOU 5393  OH  TYR A 719    13559   7750   8232  -2910   -216
2808       O
ATOM   5394  N   GLN A 720      11.898   8.701   3.315  1.00 98.26
N
ANISOU 5394  N   GLN A 720    16539  10232  10565  -2070    538
3213       N
ATOM   5395  CA  GLN A 720      11.944   7.716   2.237  1.00 74.29
C
ANISOU 5395  CA  GLN A 720    13181   7494   7553  -2100    605
3163       C
ATOM   5396  C   GLN A 720      11.415   8.270   0.917  1.00 72.86
C
ANISOU 5396  C   GLN A 720    12902   7489   7292  -2127    674
3265       C
ATOM   5397  O   GLN A 720      12.006   8.033  -0.143  1.00 94.29
O
ANISOU 5397  O   GLN A 720    15351  10499   9978  -2261    681
3241       O
ATOM   5398  CB  GLN A 720      11.158   6.469   2.644  1.00 70.41
C
ANISOU 5398  CB  GLN A 720    12595   6992   7167  -1887    696
3040       C
ATOM   5399  CG  GLN A 720      11.781   5.697   3.794  1.00 88.16
C
ANISOU 5399  CG  GLN A 720    14908   9109   9481  -1865    611
2943       C
ATOM   5400  CD  GLN A 720      11.005   4.440   4.145  1.00127.84
C
ANISOU 5400  CD  GLN A 720    19850  14109  14614  -1677    717
2843       C
ATOM   5401  OE1 GLN A 720      11.362   3.717   5.075  1.00176.94
O
ANISOU 5401  OE1 GLN A 720    26149  20202  20878  -1636    655
2783       O
ATOM   5402  NE2 GLN A 720       9.936   4.176   3.403  1.00 94.32
N
ANISOU 5402  NE2 GLN A 720    15451   9972  10416  -1564    867
2824       N
ATOM   5403  N   ALA A 721      10.304   9.006   0.959  1.00 90.82
N
ANISOU 5403  N   ALA A 721    15376   9596   9534  -1990    718
```

```
3360       N
ATOM   5404  CA  ALA A 721       9.814   9.709  -0.226  1.00 95.48
C
ANISOU 5404  CA  ALA A 721    15925  10311  10042  -1998    732
3475       C
ATOM   5405  C   ALA A 721      10.813  10.727  -0.770  1.00 79.27
C
ANISOU 5405  C   ALA A 721    13870   8333   7916  -2213    652
3572       C
ATOM   5406  O   ALA A 721      11.020  10.810  -1.987  1.00127.97
O
ANISOU 5406  O   ALA A 721    19925  14737  13963  -2344    675
3657       O
ATOM   5407  CB  ALA A 721       8.484  10.393   0.091  1.00112.31
C
ANISOU 5407  CB  ALA A 721    18202  12234  12236  -1757    749
3491       C
ATOM   5408  N   ILE A 722      11.438  11.513   0.109  1.00 84.59
N
ANISOU 5408  N   ILE A 722    14680   8810   8652  -2263    569
3559       N
ATOM   5409  CA  ILE A 722      12.490  12.437  -0.320  1.00 85.52
C
ANISOU 5409  CA  ILE A 722    14807   8964   8722  -2501    510
3641       C
ATOM   5410  C   ILE A 722      13.656  11.709  -0.985  1.00 78.31
C
ANISOU 5410  C   ILE A 722    13701   8319   7735  -2788    536
3630       C
ATOM   5411  O   ILE A 722      14.145  12.136  -2.038  1.00120.08
O
ANISOU 5411  O   ILE A 722    18902  13797  12927  -2982    572
3718       O
ATOM   5412  CB  ILE A 722      12.965  13.292   0.870  1.00 81.10
C
ANISOU 5412  CB  ILE A 722    14440   8126   8250  -2516    409
3604       C
ATOM   5413  CG1 ILE A 722      11.897  14.323   1.241  1.00 79.41
C
ANISOU 5413  CG1 ILE A 722    14396   7688   8090  -2306    390
3639       C
ATOM   5414  CG2 ILE A 722      14.279  13.989   0.540  1.00 80.85
C
ANISOU 5414  CG2 ILE A 722    14403   8126   8191  -2822    355
3663       C
ATOM   5415  CD1 ILE A 722      12.113  14.964   2.594  1.00123.76
C
ANISOU 5415  CD1 ILE A 722    20212  13030  13780  -2273    304
3563       C
ATOM   5416  N   MET A 723      14.115  10.602  -0.400  1.00 77.02
N
ANISOU 5416  N   MET A 723    13467   8182   7614  -2832    522
3522       N
ATOM   5417  CA  MET A 723      15.180   9.831  -1.042  1.00 91.35
```

```
C
ANISOU 5417  CA  MET A 723    14918  10322   9469  -2997    541
3379       C
ATOM   5418  C   MET A 723      14.726   9.132  -2.321  1.00136.26
C
ANISOU 5418  C   MET A 723    20321  16357  15094  -2950    657
3336       C
ATOM   5419  O   MET A 723      15.544   8.911  -3.221  1.00151.80
O
ANISOU 5419  O   MET A 723    22027  18626  17025  -3144    708
3274       O
ATOM   5420  CB  MET A 723      15.757   8.815  -0.056  1.00 76.33
C
ANISOU 5420  CB  MET A 723    12895   8377   7731  -2925    455
3171       C
ATOM   5421  CG  MET A 723      16.424   9.466   1.141  1.00115.24
C
ANISOU 5421  CG  MET A 723    18074  13009  12704  -3000    310
3181       C
ATOM   5422  SD  MET A 723      17.834  10.479   0.649  1.00100.29
S
ANISOU 5422  SD  MET A 723    16117  11185  10803  -3374    268
3222       S
ATOM   5423  CE  MET A 723      19.097   9.228   0.433  1.00110.50
C
ANISOU 5423  CE  MET A 723    16943  12778  12264  -3470    222
2939       C
ATOM   5424  N   THR A 724      13.446   8.776  -2.426  1.00107.26
N
ANISOU 5424  N   THR A 724    16681  12657  11413  -2700    706
3346       N
ATOM   5425  CA  THR A 724      12.896   8.331  -3.706  1.00 75.83
C
ANISOU 5425  CA  THR A 724    12478   8986   7347  -2648    793
3325       C
ATOM   5426  C   THR A 724      12.949   9.428  -4.766  1.00 78.00
C
ANISOU 5426  C   THR A 724    12873   9356   7407  -2820    812
3536       C
ATOM   5427  O   THR A 724      13.360   9.187  -5.907  1.00105.48
O
ANISOU 5427  O   THR A 724    16133  13174  10769  -2956    880
3509       O
ATOM   5428  CB  THR A 724      11.458   7.844  -3.515  1.00 74.28
C
ANISOU 5428  CB  THR A 724    12311   8695   7215  -2342    825
3286       C
ATOM   5429  OG1 THR A 724      11.405   6.913  -2.428  1.00120.25
O
ANISOU 5429  OG1 THR A 724    18089  14380  13222  -2205    820
3127       O
ATOM   5430  CG2 THR A 724      10.953   7.168  -4.776  1.00 74.33
C
ANISOU 5430  CG2 THR A 724    12034   9040   7167  -2269    890
```

```
3201       C
ATOM   5431  N   VAL A 725      12.528  10.640  -4.405  1.00 78.84
N
ANISOU 5431  N   VAL A 725    13340   9160   7454  -2816    755
3748       N
ATOM   5432  CA  VAL A 725      12.593  11.787  -5.312  1.00 84.52
C
ANISOU 5432  CA  VAL A 725    14156   9917   8039  -2910    747
3915       C
ATOM   5433  C   VAL A 725      14.028  12.114  -5.719  1.00 97.49
C
ANISOU 5433  C   VAL A 725    15704  11714   9625  -3252    788
3930       C
ATOM   5434  O   VAL A 725      14.307  12.381  -6.895  1.00119.22
O
ANISOU 5434  O   VAL A 725    18383  14709  12208  -3404    856
4008       O
ATOM   5435  CB  VAL A 725      11.897  13.001  -4.669  1.00 81.85
C
ANISOU 5435  CB  VAL A 725    14076   9216   7809  -2707    656
3993       C
ATOM   5436  CG1 VAL A 725      12.399  14.302  -5.284  1.00 84.75
C
ANISOU 5436  CG1 VAL A 725    14546   9549   8108  -2855    632
4141       C
ATOM   5437  CG2 VAL A 725      10.389  12.875  -4.820  1.00 81.02
C
ANISOU 5437  CG2 VAL A 725    14042   9040   7701  -2419    638
4020       C
ATOM   5438  N   ILE A 726      14.958  12.097  -4.764  1.00 82.94
N
ANISOU 5438  N   ILE A 726    13868   9734   7912  -3391    749
3853       N
ATOM   5439  CA  ILE A 726      16.376  12.260  -5.085  1.00 98.74
C
ANISOU 5439  CA  ILE A 726    15742  11889   9885  -3759    797
3841       C
ATOM   5440  C   ILE A 726      16.874  11.182  -6.046  1.00107.79
C
ANISOU 5440  C   ILE A 726    16502  13482  10970  -3876    917
3684       C
ATOM   5441  O   ILE A 726      17.618  11.477  -6.990  1.00129.73
O
ANISOU 5441  O   ILE A 726    19168  16503  13619  -4155   1026
3724       O
ATOM   5442  CB  ILE A 726      17.209  12.277  -3.788  1.00 95.21
C
ANISOU 5442  CB  ILE A 726    15355  11206   9616  -3854    697
3751       C
ATOM   5443  CG1 ILE A 726      16.905  13.533  -2.968  1.00117.96
C
ANISOU 5443  CG1 ILE A 726    18533  13700  12589  -3717    587
3824       C
ATOM   5444  CG2 ILE A 726      18.696  12.189  -4.101  1.00 86.25
```

```
C
ANISOU 5444  CG2 ILE A 726    13954  10277   8541  -4176    742
3625       C
ATOM   5445  CD1 ILE A 726      17.357  13.442  -1.523  1.00122.30
C
ANISOU 5445  CD1 ILE A 726    19203  13979  13285  -3708    457
3725       C
ATOM   5446  N   PHE A 727      16.476   9.927  -5.834  1.00 82.71
N
ANISOU 5446  N   PHE A 727    13089  10423   7912  -3641    908
3465       N
ATOM   5447  CA  PHE A 727      16.816   8.868  -6.785  1.00 82.86
C
ANISOU 5447  CA  PHE A 727    12703  10867   7914  -3677   1011
3260       C
ATOM   5448  C   PHE A 727      16.282   9.146  -8.189  1.00107.79
C
ANISOU 5448  C   PHE A 727    15867  14289  10798  -3722   1115
3392       C
ATOM   5449  O   PHE A 727      17.003   8.977  -9.180  1.00 86.29
O
ANISOU 5449  O   PHE A 727    12919  11913   7953  -3942   1237
3325       O
ATOM   5450  CB  PHE A 727      16.292   7.528  -6.262  1.00100.55
C
ANISOU 5450  CB  PHE A 727    14739  13121  10346  -3386    966
3028       C
ATOM   5451  CG  PHE A 727      16.492   6.375  -7.211  1.00117.38
C
ANISOU 5451  CG  PHE A 727    16449  15663  12488  -3378   1055
2791       C
ATOM   5452  CD1 PHE A 727      17.655   5.621  -7.174  1.00111.81
C
ANISOU 5452  CD1 PHE A 727    15400  15140  11943  -3506   1063
2537       C
ATOM   5453  CD2 PHE A 727      15.498   6.018  -8.113  1.00 95.05
C
ANISOU 5453  CD2 PHE A 727    13555  13029   9532  -3224   1114
2794       C
ATOM   5454  CE1 PHE A 727      17.837   4.553  -8.037  1.00103.25
C
ANISOU 5454  CE1 PHE A 727    13915  14428  10887  -3491   1144
2289       C
ATOM   5455  CE2 PHE A 727      15.673   4.952  -8.978  1.00 89.75
C
ANISOU 5455  CE2 PHE A 727    12493  12736   8873  -3210   1191
2551       C
ATOM   5456  CZ  PHE A 727      16.844   4.218  -8.939  1.00100.13
C
ANISOU 5456  CZ  PHE A 727    13468  14231  10347  -3345   1213
2296       C
ATOM   5457  N   PHE A 728      15.018   9.561  -8.299  1.00131.47
N
ANISOU 5457  N   PHE A 728    19121  17137  13695  -3510   1065
```

```
3567       N
ATOM   5458  CA  PHE A 728      14.448   9.881  -9.608  1.00102.12
C
ANISOU 5458  CA  PHE A 728    15455  13645   9702  -3525   1117
3710       C
ATOM   5459  C   PHE A 728      15.189  11.021 -10.305  1.00 88.86
C
ANISOU 5459  C   PHE A 728    13960  12012   7790  -3868   1184
3948       C
ATOM   5460  O   PHE A 728      15.546  10.911 -11.483  1.00 91.28
O
ANISOU 5460  O   PHE A 728    14131  12673   7877  -4036   1301
3946       O
ATOM   5461  CB  PHE A 728      12.963  10.224  -9.461  1.00 98.50
C
ANISOU 5461  CB  PHE A 728    15251  12953   9222  -3220   1011
3847       C
ATOM   5462  CG  PHE A 728      12.277  10.526 -10.767  1.00 92.61
C
ANISOU 5462  CG  PHE A 728    14575  12414   8198  -3186   1010
3987       C
ATOM   5463  CD1 PHE A 728      12.271  11.815 -11.284  1.00 89.23
C
ANISOU 5463  CD1 PHE A 728    14431  11862   7612  -3265    971
4233       C
ATOM   5464  CD2 PHE A 728      11.639   9.522 -11.477  1.00 97.34
C
ANISOU 5464  CD2 PHE A 728    14910  13307   8766  -2997   1027
3801       C
ATOM   5465  CE1 PHE A 728      11.648  12.097 -12.484  1.00 91.34
C
ANISOU 5465  CE1 PHE A 728    14776  12302   7625  -3201    941
4351       C
ATOM   5466  CE2 PHE A 728      11.010   9.797 -12.678  1.00126.77
C
ANISOU 5466  CE2 PHE A 728    18714  17233  12219  -2954    997
3919       C
ATOM   5467  CZ  PHE A 728      11.015  11.087 -13.182  1.00126.13
C
ANISOU 5467  CZ  PHE A 728    18998  17041  11884  -3102    950
4251       C
ATOM   5468  N   TRP A 729      15.430  12.123  -9.592  1.00 89.59
N
ANISOU 5468  N   TRP A 729    14291  11731   8018  -3870   1104
4053       N
ATOM   5469  CA  TRP A 729      16.167  13.250 -10.163  1.00 92.96
C
ANISOU 5469  CA  TRP A 729    14821  12135   8366  -4085   1154
4178       C
ATOM   5470  C   TRP A 729      17.576  12.850 -10.594  1.00 94.57
C
ANISOU 5470  C   TRP A 729    14754  12656   8524  -4471   1319
4060       C
ATOM   5471  O   TRP A 729      18.046  13.262 -11.661  1.00163.87
```

O
ANISOU 5471 O   TRP A 729   23503 21644 17118 -4666   1440
4121   O
ATOM   5472 CB TRP A 729   16.211 14.400 -9.159 1.00 93.27
C
ANISOU 5472 CB TRP A 729   15118 11722   8598 -4023   1036
4270   C
ATOM   5473 CG TRP A 729   17.073 15.543 -9.576 1.00 99.40
C
ANISOU 5473 CG TRP A 729   15991 12438   9336 -4278   1090
4392   C
ATOM   5474 CD1 TRP A 729   16.703 16.597 -10.358 1.00103.87
C
ANISOU 5474 CD1 TRP A 729   16762 12927   9776 -4247   1082
4592   C
ATOM   5475 CD2 TRP A 729   18.448 15.757 -9.239 1.00110.42
C
ANISOU 5475 CD2 TRP A 729   17289 13834 10830 -4618   1160
4328   C
ATOM   5476 NE1 TRP A 729   17.760 17.457 -10.526 1.00127.61
N
ANISOU 5476 NE1 TRP A 729   19811 15879 12795 -4547   1158
4664   N
ATOM   5477 CE2 TRP A 729   18.845 16.963 -9.850 1.00124.59
C
ANISOU 5477 CE2 TRP A 729   19236 15548 12555 -4783   1211
4496   C
ATOM   5478 CE3 TRP A 729   19.383 15.046 -8.480 1.00111.52
C
ANISOU 5478 CE3 TRP A 729   17229 14028 11117 -4811   1178
4147   C
ATOM   5479 CZ2 TRP A 729   20.135 17.474 -9.726 1.00131.60
C
ANISOU 5479 CZ2 TRP A 729   20068 16410 13525 -5139   1296
4477   C
ATOM   5480 CZ3 TRP A 729   20.664 15.556 -8.357 1.00117.11
C
ANISOU 5480 CZ3 TRP A 729   17873 14715 11908 -5164   1244
4122   C
ATOM   5481 CH2 TRP A 729   21.028 16.758 -8.976 1.00123.50
C
ANISOU 5481 CH2 TRP A 729   18824 15447 12654 -5327   1311
4280   C
ATOM   5482 N   ALA A 730   18.267 12.063 -9.767 1.00 92.84
N
ANISOU 5482 N   ALA A 730   14335 12467   8472 -4595   1328
3887   N
ATOM   5483 CA ALA A 730   19.618 11.606 -10.087 1.00132.16
C
ANISOU 5483 CA ALA A 730   18983 17750 13483 -4938   1478
3704   C
ATOM   5484 C   ALA A 730   19.684 10.844 -11.408 1.00147.33
C
ANISOU 5484 C   ALA A 730   20607 20167 15205 -4998   1646

```
3572       C
ATOM   5485  O   ALA A 730      20.690  10.930 -12.122  1.00112.47
O
ANISOU 5485  O   ALA A 730    16006  16028  10701  -5328   1826
3507       O
ATOM   5486  CB  ALA A 730      20.159  10.738  -8.953  1.00101.45
C
ANISOU 5486  CB  ALA A 730    14838  13770   9939  -4821   1372
3400       C
ATOM   5487  N   ALA A 731      18.631  10.104 -11.754  1.00110.26
N
ANISOU 5487  N   ALA A 731    15855  15596  10441  -4692   1600
3513       N
ATOM   5488  CA  ALA A 731      18.584   9.312 -12.977  1.00 94.77
C
ANISOU 5488  CA  ALA A 731    13611  14104   8292  -4705   1734
3356       C
ATOM   5489  C   ALA A 731      17.642   9.897 -14.027  1.00128.60
C
ANISOU 5489  C   ALA A 731    18177  18481  12203  -4662   1746
3628       C
ATOM   5490  O   ALA A 731      17.160   9.165 -14.898  1.00149.03
O
ANISOU 5490  O   ALA A 731    20587  21396  14640  -4538   1785
3501       O
ATOM   5491  CB  ALA A 731      18.189   7.870 -12.654  1.00 91.80
C
ANISOU 5491  CB  ALA A 731    12892  13839   8148  -4397   1667
3023       C
ATOM   5492  N   HIS A 732      17.365  11.199 -13.964  1.00103.00
N
ANISOU 5492  N   HIS A 732    15320  14899   8916  -4633   1659
3902       N
ATOM   5493  CA  HIS A 732      16.523  11.819 -14.981  1.00100.02
C
ANISOU 5493  CA  HIS A 732    15180  14542   8282  -4488   1614
4091       C
ATOM   5494  C   HIS A 732      17.062  13.162 -15.465  1.00109.04
C
ANISOU 5494  C   HIS A 732    16564  15530   9336  -4659   1655
4295       C
ATOM   5495  O   HIS A 732      17.703  13.228 -16.518  1.00127.90
O
ANISOU 5495  O   HIS A 732    18871  18217  11507  -4889   1820
4286       O
ATOM   5496  CB  HIS A 732      15.097  12.001 -14.454  1.00110.77
C
ANISOU 5496  CB  HIS A 732    16782  15589   9715  -4110   1402
4212       C
ATOM   5497  CG  HIS A 732      14.175  12.672 -15.426  1.00113.15
C
ANISOU 5497  CG  HIS A 732    17337  15880   9774  -3947   1316
4411       C
ATOM   5498  ND1 HIS A 732      13.621  12.016 -16.504  1.00106.63
```

N
ANISOU 5498 ND1 HIS A 732   16417 15427   8670 -3881   1332
4369   N
ATOM   5499 CD2 HIS A 732   13.720 13.946 -15.488 1.00117.24
C
ANISOU 5499 CD2 HIS A 732   18196 16066 10282 -3839   1200
4650   C
ATOM   5500 CE1 HIS A 732   12.858 12.855 -17.183 1.00110.85
C
ANISOU 5500 CE1 HIS A 732   17247 15847   9023 -3736   1213
4587   C
ATOM   5501 NE2 HIS A 732   12.902 14.033 -16.588 1.00110.69
N
ANISOU 5501 NE2 HIS A 732   17489 15398   9170 -3709   1135
4763   N
ATOM   5502 N   LYS A 733   16.798 14.238 -14.722 1.00117.24
N
ANISOU 5502 N   LYS A 733   17893 16112 10542 -4552   1519
4471   N
ATOM   5503 CA LYS A 733   17.298 15.554 -15.115 1.00131.11
C
ANISOU 5503 CA LYS A 733   19877 17697 12242 -4719   1557
4673   C
ATOM   5504 C   LYS A 733   18.824 15.615 -15.146 1.00160.31
C
ANISOU 5504 C   LYS A 733   23384 21535 15990 -5126   1753
4573   C
ATOM   5505 O   LYS A 733   19.411 16.138 -16.100 1.00188.07
O
ANISOU 5505 O   LYS A 733   26941 25198 19321 -5357   1901
4663   O
ATOM   5506 CB LYS A 733   16.737 16.619 -14.167 1.00120.77
C
ANISOU 5506 CB LYS A 733   18862 15880 11146 -4521   1372
4831   C
ATOM   5507 CG LYS A 733   17.441 17.970 -14.229 1.00124.06
C
ANISOU 5507 CG LYS A 733   19474 16064 11598 -4729   1409
5007   C
ATOM   5508 CD LYS A 733   17.296 18.634 -15.587 1.00118.97
C
ANISOU 5508 CD LYS A 733   19009 15538 10657 -4795   1470
5220   C
ATOM   5509 CE LYS A 733   18.104 19.921 -15.641 1.00131.55
C
ANISOU 5509 CE LYS A 733   20777 16909 12297 -5046   1536
5393   C
ATOM   5510 NZ LYS A 733   18.013 20.603 -16.961 1.00146.43
N
ANISOU 5510 NZ LYS A 733   22866 18891 13882 -5129   1605
5626   N
ATOM   5511 N   THR A 734   19.485 15.083 -14.125 1.00162.08
N
ANISOU 5511 N   THR A 734   23402 21718 16462 -5228   1760

```
4385       N
ATOM   5512  CA  THR A 734      20.940  15.117 -14.062  1.00170.62
C
ANISOU 5512  CA  THR A 734    24272  22922  17633  -5621   1931
4262       C
ATOM   5513  C   THR A 734      21.568  13.781 -14.453  1.00168.90
C
ANISOU 5513  C   THR A 734    23621  23181  17372  -5791   2100
3973       C
ATOM   5514  O   THR A 734      20.907  12.743 -14.541  1.00146.79
O
ANISOU 5514  O   THR A 734    20673  20584  14516  -5598   2065
3854       O
ATOM   5515  CB  THR A 734      21.411  15.528 -12.663  1.00175.40
C
ANISOU 5515  CB  THR A 734    24930  23157  18556  -5667   1816
4236       C
ATOM   5516  OG1 THR A 734      22.807  15.854 -12.704  1.00164.91
O
ANISOU 5516  OG1 THR A 734    23443  21899  17315  -6070   1975
4155       O
ATOM   5517  CG2 THR A 734      21.200  14.394 -11.682  1.00177.78
C
ANISOU 5517  CG2 THR A 734    25049  23469  19030  -5526   1717
4041       C
ATOM   5518  N   ASP A 735      22.879  13.832 -14.689  1.00191.00
N
ANISOU 5518  N   ASP A 735    26191  26161  20219  -6164   2294
3841       N
ATOM   5519  CA  ASP A 735      23.653  12.732 -15.248  1.00168.09
C
ANISOU 5519  CA  ASP A 735    22837  23747  17284  -6374   2502
3533       C
ATOM   5520  C   ASP A 735      24.342  11.920 -14.160  1.00141.59
C
ANISOU 5520  C   ASP A 735    19148  20398  14252  -6475   2473
3265       C
ATOM   5521  O   ASP A 735      25.017  10.933 -14.466  1.00117.86
O
ANISOU 5521  O   ASP A 735    15699  17781  11301  -6631   2627
2953       O
ATOM   5522  CB  ASP A 735      24.703  13.268 -16.229  1.00161.62
C
ANISOU 5522  CB  ASP A 735    21929  23141  16340  -6728   2757
3510       C
ATOM   5523  CG  ASP A 735      24.098  14.108 -17.338  1.00151.98
C
ANISOU 5523  CG  ASP A 735    21056  21904  14785  -6661   2787
3796       C
ATOM   5524  OD1 ASP A 735      24.830  14.936 -17.923  1.00146.14
O
ANISOU 5524  OD1 ASP A 735    20398  21166  13964  -6930   2952
3892       O
ATOM   5525  OD2 ASP A 735      22.895  13.940 -17.627  1.00138.02
```

```
O
ANISOU 5525  OD2 ASP A 735    19477  20120  12844  -6347   2646
3922       O
ATOM   5526  N   PHE A 736      24.182  12.336 -12.901  1.00148.36
N
ANISOU 5526  N   PHE A 736    20208  20826  15335  -6382   2270
3367       N
ATOM   5527  CA  PHE A 736      25.006  11.861 -11.791  1.00148.38
C
ANISOU 5527  CA  PHE A 736    19951  20727  15698  -6465   2188
3119       C
ATOM   5528  C   PHE A 736      24.960  10.344 -11.637  1.00111.21
C
ANISOU 5528  C   PHE A 736    14822  16253  11181  -6185   2118
2733       C
ATOM   5529  O   PHE A 736      25.997   9.703 -11.425  1.00104.25
O
ANISOU 5529  O   PHE A 736    13526  15521  10565  -6289   2142
2386       O
ATOM   5530  CB  PHE A 736      24.545  12.549 -10.502  1.00173.60
C
ANISOU 5530  CB  PHE A 736    23511  23392  19058  -6285   1932
3301       C
ATOM   5531  CG  PHE A 736      25.202  12.030  -9.253  1.00155.23
C
ANISOU 5531  CG  PHE A 736    20966  20887  17127  -6182   1745
3010       C
ATOM   5532  CD1 PHE A 736      26.560  11.757  -9.216  1.00160.21
C
ANISOU 5532  CD1 PHE A 736    21204  21681  17988  -6443   1819
2711       C
ATOM   5533  CD2 PHE A 736      24.447  11.809  -8.113  1.00121.09
C
ANISOU 5533  CD2 PHE A 736    16835  16233  12940  -5821   1491
3027       C
ATOM   5534  CE1 PHE A 736      27.150  11.277  -8.063  1.00143.76
C
ANISOU 5534  CE1 PHE A 736    18936  19422  16264  -6325   1603
2443       C
ATOM   5535  CE2 PHE A 736      25.031  11.330  -6.960  1.00121.67
C
ANISOU 5535  CE2 PHE A 736    16753  16139  13337  -5719   1300
2779       C
ATOM   5536  CZ  PHE A 736      26.383  11.064  -6.935  1.00131.57
C
ANISOU 5536  CZ  PHE A 736    17627  17546  14817  -5961   1336
2491       C
ATOM   5537  N   PHE A 737      23.769   9.752 -11.745  1.00106.09
N
ANISOU 5537  N   PHE A 737    14261  15624  10424  -5829   2022
2776       N
ATOM   5538  CA  PHE A 737      23.618   8.318 -11.510  1.00121.43
C
ANISOU 5538  CA  PHE A 737    15841  17722  12574  -5542   1935
```

```
2432       C
ATOM   5539  C   PHE A 737      24.450   7.480 -12.476  1.00100.43
C
ANISOU 5539  C   PHE A 737    12689  15554   9914  -5718   2137
2097       C
ATOM   5540  O   PHE A 737      25.009   6.449 -12.086  1.00110.66
O
ANISOU 5540  O   PHE A 737    13590  16941  11515  -5622   2069
1733       O
ATOM   5541  CB  PHE A 737      22.145   7.927 -11.595  1.00 95.58
C
ANISOU 5541  CB  PHE A 737    12755  14394   9168  -5169   1834
2552       C
ATOM   5542  CG  PHE A 737      21.806   6.704 -10.801  1.00101.79
C
ANISOU 5542  CG  PHE A 737    13328  15102  10245  -4826   1666
2292       C
ATOM   5543  CD1 PHE A 737      21.372   6.818  -9.490  1.00112.65
C
ANISOU 5543  CD1 PHE A 737    14939  16051  11810  -4606   1457
2374       C
ATOM   5544  CD2 PHE A 737      21.939   5.441 -11.352  1.00119.84
C
ANISOU 5544  CD2 PHE A 737    15188  17731  12612  -4733   1722
1964       C
ATOM   5545  CE1 PHE A 737      21.064   5.695  -8.745  1.00157.85
C
ANISOU 5545  CE1 PHE A 737    20504  21688  17785  -4309   1316
2161       C
ATOM   5546  CE2 PHE A 737      21.631   4.311 -10.614  1.00162.68
C
ANISOU 5546  CE2 PHE A 737    20436  23053  18321  -4427   1564
1741       C
ATOM   5547  CZ  PHE A 737      21.193   4.439  -9.308  1.00173.52
C
ANISOU 5547  CZ  PHE A 737    22072  23993  19864  -4221   1365
1854       C
ATOM   5548  N   SER A 738      24.541   7.894 -13.736  1.00112.67
N
ANISOU 5548  N   SER A 738    14262  17423  11122  -5972   2382
2205       N
ATOM   5549  CA  SER A 738      25.340   7.171 -14.717  1.00147.98
C
ANISOU 5549  CA  SER A 738    18278  22390  15559  -6169   2613
1876       C
ATOM   5550  C   SER A 738      26.722   7.774 -14.924  1.00173.81
C
ANISOU 5550  C   SER A 738    21384  25769  18886  -6626   2823
1796       C
ATOM   5551  O   SER A 738      27.559   7.153 -15.587  1.00192.20
O
ANISOU 5551  O   SER A 738    23273  28493  21262  -6810   3022
1458       O
ATOM   5552  CB  SER A 738      24.606   7.111 -16.061  1.00138.37
```

```
C
ANISOU 5552  CB  SER A 738    17152  21524  13900  -6167   2776
1993       C
ATOM   5553  OG  SER A 738      24.223   8.404 -16.500  1.00137.59
O
ANISOU 5553  OG  SER A 738    17537  21301  13440  -6349   2848
2441       O
ATOM   5554  N   ASP A 739      26.973   8.965 -14.380  1.00143.18
N
ANISOU 5554  N   ASP A 739    17833  21548  15023  -6818   2791
2078       N
ATOM   5555  CA  ASP A 739      28.327   9.509 -14.347  1.00119.09
C
ANISOU 5555  CA  ASP A 739    14595  18527  12127  -7239   2955
1965       C
ATOM   5556  C   ASP A 739      29.284   8.611 -13.568  1.00134.07
C
ANISOU 5556  C   ASP A 739    15996  20437  14510  -7176   2833
1496       C
ATOM   5557  O   ASP A 739      30.416   8.375 -14.007  1.00154.81
O
ANISOU 5557  O   ASP A 739    18202  23351  17270  -7467   3033
1186       O
ATOM   5558  CB  ASP A 739      28.305  10.916 -13.745  1.00115.02
C
ANISOU 5558  CB  ASP A 739    14540  17570  11591  -7399   2885
2346       C
ATOM   5559  CG  ASP A 739      29.665  11.589 -13.774  1.00143.32
C
ANISOU 5559  CG  ASP A 739    17957  21143  15354  -7798   3044
2231       C
ATOM   5560  OD1 ASP A 739      30.518  11.182 -14.590  1.00182.95
O
ANISOU 5560  OD1 ASP A 739    22589  26541  20384  -7995   3283
1942       O
ATOM   5561  OD2 ASP A 739      29.882  12.526 -12.977  1.00134.46
O
ANISOU 5561  OD2 ASP A 739    17086  19621  14384  -7883   2922
2403       O
ATOM   5562  N   THR A 740      28.854   8.100 -12.416  1.00151.27
N
ANISOU 5562  N   THR A 740    18214  22304  16957  -6801   2503
1432       N
ATOM   5563  CA  THR A 740      29.627   7.122 -11.654  1.00159.23
C
ANISOU 5563  CA  THR A 740    18785  23302  18412  -6670   2326
1000       C
ATOM   5564  C   THR A 740      29.185   5.693 -11.966  1.00163.15
C
ANISOU 5564  C   THR A 740    18970  24053  18966  -6357   2278
720       C
ATOM   5565  O   THR A 740      28.088   5.273 -11.579  1.00154.13
O
ANISOU 5565  O   THR A 740    18043  22743  17776  -5996   2099
```

```
847        O
ATOM   5566  CB  THR A 740      29.512   7.401 -10.156  1.00128.52
C
ANISOU 5566  CB  THR A 740    15143  18910  14779  -6464   1987
1090       C
ATOM   5567  OG1 THR A 740      30.035   8.704  -9.868  1.00127.33
O
ANISOU 5567  OG1 THR A 740    15231  18528  14622  -6771   2027
1296       O
ATOM   5568  CG2 THR A 740      30.294   6.366  -9.362  1.00120.29
C
ANISOU 5568  CG2 THR A 740    13683  17842  14181  -6307   1764
660        C
ATOM   5569  N   PHE A 741      30.049   4.955 -12.668  1.00165.59
N
ANISOU 5569  N   PHE A 741    18758  24762  19397  -6507   2448
320        N
ATOM   5570  CA  PHE A 741      29.860   3.529 -12.952  1.00168.36
C
ANISOU 5570  CA  PHE A 741    18724  25361  19884  -6239   2398
-33        C
ATOM   5571  C   PHE A 741      28.545   3.263 -13.689  1.00181.98
C
ANISOU 5571  C   PHE A 741    20668  27228  21247  -6030   2462
166        C
ATOM   5572  O   PHE A 741      27.705   2.470 -13.256  1.00204.99
O
ANISOU 5572  O   PHE A 741    23619  30010  24258  -5654   2257
140        O
ATOM   5573  CB  PHE A 741      29.963   2.708 -11.660  1.00166.43
C
ANISOU 5573  CB  PHE A 741    18357  24800  20080  -5906   2029
-236       C
ATOM   5574  CG  PHE A 741      29.881   1.219 -11.870  1.00178.31
C
ANISOU 5574  CG  PHE A 741    19444  26510  21794  -5640   1953
-619       C
ATOM   5575  CD1 PHE A 741      30.771   0.579 -12.717  1.00180.89
C
ANISOU 5575  CD1 PHE A 741    19226  27258  22244  -5808   2141
-1047       C
ATOM   5576  CD2 PHE A 741      28.928   0.460 -11.209  1.00172.82
C
ANISOU 5576  CD2 PHE A 741    18895  25577  21192  -5230   1704
-566       C
ATOM   5577  CE1 PHE A 741      30.705  -0.788 -12.913  1.00184.04
C
ANISOU 5577  CE1 PHE A 741    19234  27830  22863  -5558   2060
-1418       C
ATOM   5578  CE2 PHE A 741      28.857  -0.910 -11.400  1.00162.24
C
ANISOU 5578  CE2 PHE A 741    17176  24398  20070  -4993   1630
-915       C
ATOM   5579  CZ  PHE A 741      29.748  -1.534 -12.252  1.00175.72
```

```
C
ANISOU 5579  CZ  PHE A 741    18340  26515  21910  -5150   1796
-1344       C
ATOM   5580  N   GLY A 742      28.357   3.955 -14.809  1.00158.44
N
ANISOU 5580  N   GLY A 742    17848  24508  17842  -6283   2744
372       N
ATOM   5581  CA  GLY A 742      27.163   3.761 -15.609  1.00149.29
C
ANISOU 5581  CA  GLY A 742    16890  23516  16319  -6105   2797
544       C
ATOM   5582  C   GLY A 742      27.245   2.533 -16.494  1.00163.62
C
ANISOU 5582  C   GLY A 742    18240  25786  18143  -6023   2914
137       C
ATOM   5583  O   GLY A 742      27.637   2.630 -17.661  1.00188.06
O
ANISOU 5583  O   GLY A 742    21185  29305  20963  -6293   3208
45       O
ATOM   5584  N   VAL A 743      26.882   1.368 -15.948  1.00162.23
N
ANISOU 5584  N   VAL A 743    17841  25522  18279  -5659   2691
-114       N
ATOM   5585  CA  VAL A 743      26.738   0.169 -16.772  1.00179.50
C
ANISOU 5585  CA  VAL A 743    19626  28099  20478  -5524   2766
-484       C
ATOM   5586  C   VAL A 743      25.664   0.375 -17.832  1.00197.05
C
ANISOU 5586  C   VAL A 743    22101  30552  22217  -5477   2890
-266       C
ATOM   5587  O   VAL A 743      25.835  -0.009 -18.996  1.00243.63
O
ANISOU 5587  O   VAL A 743    27755  36917  27898  -5596   3109
-484       O
ATOM   5588  CB  VAL A 743      26.435  -1.053 -15.885  1.00147.55
C
ANISOU 5588  CB  VAL A 743    15365  23828  16868  -5128   2474
-732       C
ATOM   5589  CG1 VAL A 743      26.063  -2.259 -16.738  1.00140.55
C
ANISOU 5589  CG1 VAL A 743    14120  23298  15986  -4952   2530
-1076       C
ATOM   5590  CG2 VAL A 743      27.633  -1.370 -15.006  1.00124.27
C
ANISOU 5590  CG2 VAL A 743    12105  20724  14388  -5183   2344
-1015       C
ATOM   5591  N   ARG A 744      24.549   0.987 -17.452  1.00162.52
N
ANISOU 5591  N   ARG A 744    18216  25864  17670  -5299   2744
148       N
ATOM   5592  CA  ARG A 744      23.648   1.630 -18.393  1.00137.58
C
ANISOU 5592  CA  ARG A 744    15410  22854  14011  -5326   2846
```

```
460       C
ATOM   5593  C   ARG A 744      23.552   3.114 -18.067  1.00125.39
C
ANISOU 5593  C   ARG A 744    14384  21002  12256  -5512   2846
947       C
ATOM   5594  O   ARG A 744      24.085   3.593 -17.063  1.00107.33
O
ANISOU 5594  O   ARG A 744    12181  18377  10221  -5587   2755
1030       O
ATOM   5595  CB  ARG A 744      22.257   0.979 -18.371  1.00123.03
C
ANISOU 5595  CB  ARG A 744    13660  20927  12157  -4912   2658
485       C
ATOM   5596  CG  ARG A 744      22.229  -0.455 -18.883  1.00129.57
C
ANISOU 5596  CG  ARG A 744    14002  22083  13148  -4735   2670
18       C
ATOM   5597  CD  ARG A 744      20.817  -0.884 -19.262  1.00124.97
C
ANISOU 5597  CD  ARG A 744    13542  21519  12424  -4408   2554
73       C
ATOM   5598  NE  ARG A 744      20.750  -2.304 -19.596  1.00138.47
N
ANISOU 5598  NE  ARG A 744    14781  23470  14361  -4211   2532
-387       N
ATOM   5599  CZ  ARG A 744      19.623  -2.965 -19.842  1.00171.66
C
ANISOU 5599  CZ  ARG A 744    18966  27691  18567  -3904   2417
-457       C
ATOM   5600  NH1 ARG A 744      19.651  -4.274 -20.051  1.00188.75
N
ANISOU 5600  NH1 ARG A 744    20684  30034  20999  -3736   2389
-894       N
ATOM   5601  NH2 ARG A 744      18.460  -2.328 -19.821  1.00181.76
N
ANISOU 5601  NH2 ARG A 744    20659  28778  19624  -3755   2314
-106       N
ATOM   5602  N   SER A 745      22.864   3.847 -18.938  1.00124.79
N
ANISOU 5602  N   SER A 745    14661  21038  11713  -5579   2933
1263       N
ATOM   5603  CA  SER A 745      22.599   5.259 -18.706  1.00119.24
C
ANISOU 5603  CA  SER A 745    14489  20020  10797  -5717   2907
1749       C
ATOM   5604  C   SER A 745      21.227   5.600 -19.261  1.00112.50
C
ANISOU 5604  C   SER A 745    14017  19139   9587  -5501   2807
2049       C
ATOM   5605  O   SER A 745      20.929   5.285 -20.417  1.00124.15
O
ANISOU 5605  O   SER A 745    15430  21007  10733  -5511   2917
1980       O
ATOM   5606  CB  SER A 745      23.668   6.137 -19.364  1.00122.51
```

```
C
ANISOU 5606  CB  SER A 745    14948  20627  10972  -6209   3193
1854       C
ATOM   5607  OG  SER A 745      23.510   7.494 -18.989  1.00122.81
O
ANISOU 5607  OG  SER A 745    15483  20241  10938  -6277   3106
2279       O
ATOM   5608  N   ILE A 746      20.401   6.243 -18.438  1.00113.91
N
ANISOU 5608  N   ILE A 746    14584  18859   9838  -5301   2589
2360       N
ATOM   5609  CA  ILE A 746      19.017   6.530 -18.789  1.00103.80
C
ANISOU 5609  CA  ILE A 746    13642  17486   8310  -5037   2441
2614       C
ATOM   5610  C   ILE A 746      18.637   7.888 -18.221  1.00103.81
C
ANISOU 5610  C   ILE A 746    14163  17034   8246  -5072   2326
3062       C
ATOM   5611  O   ILE A 746      19.125   8.306 -17.167  1.00102.63
O
ANISOU 5611  O   ILE A 746    14080  16545   8371  -5142   2274
3117       O
ATOM   5612  CB  ILE A 746      18.032   5.451 -18.282  1.00 99.93
C
ANISOU 5612  CB  ILE A 746    12971  16904   8095  -4593   2242
2393       C
ATOM   5613  CG1 ILE A 746      18.232   5.196 -16.786  1.00115.93
C
ANISOU 5613  CG1 ILE A 746    14930  18527  10591  -4460   2105
2309       C
ATOM   5614  CG2 ILE A 746      18.172   4.166 -19.089  1.00100.43
C
ANISOU 5614  CG2 ILE A 746    12569  17433   8156  -4529   2338
1978       C
ATOM   5615  CD1 ILE A 746      17.121   4.385 -16.152  1.00 93.00
C
ANISOU 5615  CD1 ILE A 746    11974  15428   7935  -4041   1914
2197       C
ATOM   5616  N   ARG A 747      17.762   8.580 -18.943  1.00105.41
N
ANISOU 5616  N   ARG A 747    14722  17186   8144  -4958   2243
3334       N
ATOM   5617  CA  ARG A 747      17.137   9.808 -18.472  1.00130.72
C
ANISOU 5617  CA  ARG A 747    18393  19882  11394  -4812   2054
3678       C
ATOM   5618  C   ARG A 747      15.651   9.733 -18.786  1.00166.96
C
ANISOU 5618  C   ARG A 747    23180  24430  15827  -4471   1864
3801       C
ATOM   5619  O   ARG A 747      14.815   9.673 -17.879  1.00175.90
O
ANISOU 5619  O   ARG A 747    24417  25268  17149  -4230   1694
```

3861   O
ATOM   5620 CB ARG A 747   17.761 11.054 -19.113 1.00129.06
C
ANISOU 5620 CB ARG A 747   18440 19563 11036 -5021   2134
3888   C
ATOM   5621 CG ARG A 747   19.141 11.462 -18.596 1.00132.78
C
ANISOU 5621 CG ARG A 747   18802 19937 11713 -5348   2279
3832   C
ATOM   5622 CD ARG A 747   20.286 10.688 -19.220 1.00164.10
C
ANISOU 5622 CD ARG A 747   22351 24377 15622 -5650   2542
3532   C
ATOM   5623 NE ARG A 747   21.562 11.345 -18.952 1.00172.19
N
ANISOU 5623 NE ARG A 747   23333 25296 16794 -5983   2682
3526   N
ATOM   5624 CZ ARG A 747   22.733 10.960 -19.449 1.00179.67
C
ANISOU 5624 CZ ARG A 747   23942 26586 17736 -6293   2929
3279   C
ATOM   5625 NH1 ARG A 747   22.855   9.780 -20.035 1.00199.88
N
ANISOU 5625 NH1 ARG A 747   26119 29611 20215 -6298   3053
2962   N
ATOM   5626 NH2 ARG A 747   23.823 11.658 -19.164 1.00154.30
N
ANISOU 5626 NH2 ARG A 747   20715 23236 14676 -6589   3043
3292   N
ATOM   5627 N   ASP A 748   15.321   9.733 -20.076 1.00139.47
N
ANISOU 5627 N   ASP A 748   19749 21249 11994 -4455   1891
3829   N
ATOM   5628 CA ASP A 748   13.947   9.733 -20.554 1.00121.19
C
ANISOU 5628 CA ASP A 748   17628 18937   9482 -4153   1696
3947   C
ATOM   5629 C   ASP A 748   13.461   8.344 -20.960 1.00126.31
C
ANISOU 5629 C   ASP A 748   17918 20035 10038 -4024   1712
3653   C
ATOM   5630 O   ASP A 748   12.395   8.226 -21.572 1.00143.15
O
ANISOU 5630 O   ASP A 748   20143 22250 12000 -3767   1553
3676   O
ATOM   5631 CB ASP A 748   13.810 10.707 -21.726 1.00130.15
C
ANISOU 5631 CB ASP A 748   19094 20082 10277 -4184   1672
4183   C
ATOM   5632 CG ASP A 748   14.731 10.361 -22.877 1.00153.26
C
ANISOU 5632 CG ASP A 748   21830 23476 12925 -4453   1910
4031   C
ATOM   5633 OD1 ASP A 748   14.238 10.244 -24.018 1.00187.03

```
O
ANISOU 5633  OD1 ASP A 748    26187  28032  16845  -4381   1879
4048       O
ATOM   5634  OD2 ASP A 748      15.949  10.208 -22.639  1.00159.69
O
ANISOU 5634  OD2 ASP A 748    22414  24381  13880  -4737   2122
3883       O
ATOM   5635  N   ASN A 749      14.219   7.294 -20.637  1.00121.88
N
ANISOU 5635  N   ASN A 749    16898  19664   9747  -4077   1852
3276       N
ATOM   5636  CA  ASN A 749      13.822   5.911 -20.919  1.00136.26
C
ANISOU 5636  CA  ASN A 749    18294  21771  11709  -3832   1832
2865       C
ATOM   5637  C   ASN A 749      12.858   5.436 -19.826  1.00143.86
C
ANISOU 5637  C   ASN A 749    19199  22362  13098  -3451   1633
2773       C
ATOM   5638  O   ASN A 749      13.185   4.626 -18.956  1.00145.82
O
ANISOU 5638  O   ASN A 749    19153  22510  13743  -3380   1652
2519       O
ATOM   5639  CB  ASN A 749      15.055   5.027 -21.036  1.00133.00
C
ANISOU 5639  CB  ASN A 749    17422  21687  11423  -4050   2053
2503       C
ATOM   5640  CG  ASN A 749      14.745   3.658 -21.602  1.00123.47
C
ANISOU 5640  CG  ASN A 749    15783  20841  10288  -3849   2056
2078       C
ATOM   5641  OD1 ASN A 749      13.591   3.326 -21.871  1.00117.20
O
ANISOU 5641  OD1 ASN A 749    15016  20049   9468  -3541   1892
2041       O
ATOM   5642  ND2 ASN A 749      15.785   2.867 -21.819  1.00147.63
N
ANISOU 5642  ND2 ASN A 749    18427  24214  13452  -4024   2241
1733       N
ATOM   5643  N   ASN A 750      11.637   5.976 -19.895  1.00137.45
N
ANISOU 5643  N   ASN A 750    18689  21340  12193  -3207   1436
2988       N
ATOM   5644  CA  ASN A 750      10.682   5.896 -18.790  1.00103.74
C
ANISOU 5644  CA  ASN A 750    14479  16644   8291  -2891   1267
2992       C
ATOM   5645  C   ASN A 750      10.358   4.466 -18.360  1.00 92.48
C
ANISOU 5645  C   ASN A 750    12619  15279   7240  -2651   1262
2588       C
ATOM   5646  O   ASN A 750      10.290   4.184 -17.158  1.00139.12
O
ANISOU 5646  O   ASN A 750    18475  20864  13521  -2543   1238
```

```
2525       O
ATOM   5647  CB  ASN A 750       9.395   6.633 -19.161  1.00 95.73
C
ANISOU 5647  CB  ASN A 750    13802  15468   7102  -2660   1057
3231       C
ATOM   5648  CG  ASN A 750       9.597   8.128 -19.289  1.00 98.27
C
ANISOU 5648  CG  ASN A 750    14610  15580   7147  -2856   1019
3670       C
ATOM   5649  OD1 ASN A 750      10.719   8.624 -19.196  1.00101.59
O
ANISOU 5649  OD1 ASN A 750    15112  16010   7478  -3192   1172
3798       O
ATOM   5650  ND2 ASN A 750       8.507   8.858 -19.491  1.00106.81
N
ANISOU 5650  ND2 ASN A 750    16009  16454   8120  -2644    806
3893       N
ATOM   5651  N   HIS A 751      10.141   3.549 -19.309  1.00 92.62
N
ANISOU 5651  N   HIS A 751    12335  15693   7162  -2564   1281
2310       N
ATOM   5652  CA  HIS A 751       9.841   2.171 -18.913  1.00 90.07
C
ANISOU 5652  CA  HIS A 751    11595  15405   7222  -2345   1276
1923       C
ATOM   5653  C   HIS A 751      11.000   1.495 -18.185  1.00102.07
C
ANISOU 5653  C   HIS A 751    12834  16914   9032  -2502   1410
1719       C
ATOM   5654  O   HIS A 751      10.781   0.777 -17.202  1.00124.72
O
ANISOU 5654  O   HIS A 751    15547  19540  12302  -2335   1373
1563       O
ATOM   5655  CB  HIS A 751       9.430   1.335 -20.129  1.00 91.70
C
ANISOU 5655  CB  HIS A 751    11521  16050   7270  -2233   1264
1641       C
ATOM   5656  CG  HIS A 751       8.233   1.863 -20.857  1.00134.43
C
ANISOU 5656  CG  HIS A 751    17171  21483  12426  -2034   1087
1790       C
ATOM   5657  ND1 HIS A 751       7.150   2.414 -20.208  1.00151.59
N
ANISOU 5657  ND1 HIS A 751    19593  23253  14753  -1800    919
1977       N
ATOM   5658  CD2 HIS A 751       7.929   1.884 -22.176  1.00140.32
C
ANISOU 5658  CD2 HIS A 751    17930  22605  12781  -2019   1036
1754       C
ATOM   5659  CE1 HIS A 751       6.243   2.777 -21.098  1.00145.31
C
ANISOU 5659  CE1 HIS A 751    18945  22571  13693  -1643    755
2052       C
ATOM   5660  NE2 HIS A 751       6.691   2.466 -22.299  1.00134.33
```

```
N
ANISOU 5660  NE2 HIS A 751    17432  21653  11955  -1770    813
1930       N
ATOM   5661  N   GLU A 752      12.234   1.696 -18.644  1.00107.03
N
ANISOU 5661  N   GLU A 752    13394  17798   9473  -2822   1563
1709       N
ATOM   5662  CA  GLU A 752      13.384   1.301 -17.833  1.00128.77
C
ANISOU 5662  CA  GLU A 752    15940  20473  12515  -2981   1654
1563       C
ATOM   5663  C   GLU A 752      13.503   2.117 -16.548  1.00137.23
C
ANISOU 5663  C   GLU A 752    17322  21060  13757  -3010   1589
1834       C
ATOM   5664  O   GLU A 752      13.811   1.565 -15.485  1.00138.16
O
ANISOU 5664  O   GLU A 752    17308  20951  14237  -2944   1556
1702       O
ATOM   5665  CB  GLU A 752      14.677   1.380 -18.646  1.00135.77
C
ANISOU 5665  CB  GLU A 752    16659  21750  13178  -3330   1847
1466       C
ATOM   5666  CG  GLU A 752      14.825   0.255 -19.662  1.00136.27
C
ANISOU 5666  CG  GLU A 752    16293  22292  13193  -3302   1934
1069       C
ATOM   5667  CD  GLU A 752      16.150   0.294 -20.397  1.00154.82
C
ANISOU 5667  CD  GLU A 752    18444  25031  15351  -3659   2157
931       C
ATOM   5668  OE1 GLU A 752      16.142   0.400 -21.641  1.00167.88
O
ANISOU 5668  OE1 GLU A 752    20093  27090  16602  -3772   2265
904       O
ATOM   5669  OE2 GLU A 752      17.204   0.218 -19.730  1.00152.21
O
ANISOU 5669  OE2 GLU A 752    17959  24606  15269  -3828   2225
837       O
ATOM   5670  N   LEU A 753      13.276   3.432 -16.626  1.00126.43
N
ANISOU 5670  N   LEU A 753    16382  19526  12130  -3108   1558
2210       N
ATOM   5671  CA  LEU A 753      13.423   4.293 -15.453  1.00 92.00
C
ANISOU 5671  CA  LEU A 753    12329  14714   7912  -3152   1497
2458       C
ATOM   5672  C   LEU A 753      12.490   3.914 -14.303  1.00121.92
C
ANISOU 5672  C   LEU A 753    16167  18123  12034  -2836   1364
2424       C
ATOM   5673  O   LEU A 753      12.927   3.859 -13.148  1.00143.45
O
ANISOU 5673  O   LEU A 753    18916  20569  15019  -2848   1343
```

```
2410       O
ATOM   5674  CB  LEU A 753      13.204   5.752 -15.857  1.00 94.25
C
ANISOU 5674  CB  LEU A 753    13065  14883   7862  -3286   1471
2860       C
ATOM   5675  CG  LEU A 753      13.459   6.818 -14.790  1.00 89.49
C
ANISOU 5675  CG  LEU A 753    12803  13837   7363  -3380   1418
3128       C
ATOM   5676  CD1 LEU A 753      14.891   6.734 -14.296  1.00 89.75
C
ANISOU 5676  CD1 LEU A 753    12669  13890   7541  -3661   1529
3019       C
ATOM   5677  CD2 LEU A 753      13.170   8.200 -15.347  1.00 92.15
C
ANISOU 5677  CD2 LEU A 753    13575  14070   7369  -3498   1381
3515       C
ATOM   5678  N   MET A 754      11.209   3.644 -14.581  1.00 83.84
N
ANISOU 5678  N   MET A 754    11361  13283   7211  -2555   1277
2399       N
ATOM   5679  CA  MET A 754      10.306   3.320 -13.477  1.00 83.01
C
ANISOU 5679  CA  MET A 754    11306  12811   7423  -2279   1190
2366       C
ATOM   5680  C   MET A 754      10.501   1.911 -12.925  1.00 89.50
C
ANISOU 5680  C   MET A 754    11758  13657   8590  -2170   1223
2033       C
ATOM   5681  O   MET A 754      10.090   1.644 -11.790  1.00112.95
O
ANISOU 5681  O   MET A 754    14791  16293  11832  -2018   1185
2020       O
ATOM   5682  CB  MET A 754       8.839   3.499 -13.877  1.00 81.19
C
ANISOU 5682  CB  MET A 754    11191  12522   7134  -2010   1086
2423       C
ATOM   5683  CG  MET A 754       8.309   2.485 -14.862  1.00106.32
C
ANISOU 5683  CG  MET A 754    14040  16054  10302  -1861   1083
2149       C
ATOM   5684  SD  MET A 754       6.568   2.797 -15.204  1.00144.91
S
ANISOU 5684  SD  MET A 754    19071  20819  15170  -1531    926
2209       S
ATOM   5685  CE  MET A 754       6.683   4.140 -16.381  1.00203.15
C
ANISOU 5685  CE  MET A 754    26794  28362  22030  -1688    849
2537       C
ATOM   5686  N   GLY A 755      11.109   1.007 -13.692  1.00 79.88
N
ANISOU 5686  N   GLY A 755    10168  12815   7367  -2246   1293
1764       N
ATOM   5687  CA  GLY A 755      11.546  -0.260 -13.123  1.00 78.34
```

```
C
ANISOU 5687  CA  GLY A 755     9634  12614   7516  -2184   1311
1461       C
ATOM   5688  C   GLY A 755      12.603  -0.090 -12.048  1.00 98.55
C
ANISOU 5688  C   GLY A 755    12262  14945  10237  -2335   1306
1509       C
ATOM   5689  O   GLY A 755      12.576  -0.772 -11.021  1.00121.78
O
ANISOU 5689  O   GLY A 755    15147  17637  13485  -2210   1257
1408       O
ATOM   5690  N   ALA A 756      13.553   0.817 -12.278  1.00106.10
N
ANISOU 5690  N   ALA A 756    13346  15980  10987  -2610   1352
1661       N
ATOM   5691  CA  ALA A 756      14.506   1.203 -11.241  1.00 83.93
C
ANISOU 5691  CA  ALA A 756    10652  12925   8314  -2758   1322
1735       C
ATOM   5692  C   ALA A 756      13.827   1.845 -10.033  1.00 93.33
C
ANISOU 5692  C   ALA A 756    12229  13646   9586  -2623   1227
1972       C
ATOM   5693  O   ALA A 756      14.167   1.530  -8.885  1.00 83.22
O
ANISOU 5693  O   ALA A 756    10976  12103   8540  -2579   1160
1925       O
ATOM   5694  CB  ALA A 756      15.545   2.159 -11.829  1.00 86.03
C
ANISOU 5694  CB  ALA A 756    10988  13366   8333  -3095   1408
1861       C
ATOM   5695  N   VAL A 757      12.876   2.752 -10.266  1.00 77.50
N
ANISOU 5695  N   VAL A 757    10533  11528   7386  -2553   1211
2219       N
ATOM   5696  CA  VAL A 757      12.123   3.360  -9.167  1.00 99.71
C
ANISOU 5696  CA  VAL A 757    13697  13908  10282  -2407   1135
2412       C
ATOM   5697  C   VAL A 757      11.355   2.313  -8.360  1.00106.67
C
ANISOU 5697  C   VAL A 757    14473  14608  11448  -2139   1113
2245       C
ATOM   5698  O   VAL A 757      11.339   2.358  -7.123  1.00 99.43
O
ANISOU 5698  O   VAL A 757    13735  13358  10686  -2080   1072
2292       O
ATOM   5699  CB  VAL A 757      11.184   4.454  -9.709  1.00 77.12
C
ANISOU 5699  CB  VAL A 757    11138  10982   7184  -2356   1109
2668       C
ATOM   5700  CG1 VAL A 757      10.298   4.996  -8.598  1.00 78.09
C
ANISOU 5700  CG1 VAL A 757    11579  10669   7424  -2176   1043
```

2813 C
ATOM 5701 CG2 VAL A 757 11.991 5.573 -10.349 1.00 79.61
C
ANISOU 5701 CG2 VAL A 757 11624 11402 7222 -2648 1134
2882 C
ATOM 5702 N TYR A 758 10.711 1.361 -9.035 1.00 73.51
N
ANISOU 5702 N TYR A 758 9995 10619 7316 -1983 1144
2046 N
ATOM 5703 CA TYR A 758 10.076 0.242 -8.338 1.00 71.70
C
ANISOU 5703 CA TYR A 758 9624 10235 7384 -1761 1145
1864 C
ATOM 5704 C TYR A 758 11.038 -0.522 -7.433 1.00 70.92
C
ANISOU 5704 C TYR A 758 9410 10026 7511 -1815 1118
1732 C
ATOM 5705 O TYR A 758 10.723 -0.795 -6.269 1.00119.36
O
ANISOU 5705 O TYR A 758 15693 15836 13824 -1700 1096
1756 O
ATOM 5706 CB TYR A 758 9.470 -0.720 -9.356 1.00 71.94
C
ANISOU 5706 CB TYR A 758 9308 10561 7465 -1629 1178
1626 C
ATOM 5707 CG TYR A 758 9.265 -2.118 -8.810 1.00 70.59
C
ANISOU 5707 CG TYR A 758 8881 10305 7635 -1477 1192
1376 C
ATOM 5708 CD1 TYR A 758 8.210 -2.416 -7.962 1.00 91.68
C
ANISOU 5708 CD1 TYR A 758 11666 12663 10505 -1277 1212
1390 C
ATOM 5709 CD2 TYR A 758 10.149 -3.142 -9.137 1.00 99.16
C
ANISOU 5709 CD2 TYR A 758 12142 14147 11387 -1542 1193
1120 C
ATOM 5710 CE1 TYR A 758 8.037 -3.700 -7.460 1.00108.42
C
ANISOU 5710 CE1 TYR A 758 13574 14683 12936 -1160 1236
1183 C
ATOM 5711 CE2 TYR A 758 9.984 -4.423 -8.644 1.00104.62
C
ANISOU 5711 CE2 TYR A 758 12613 14731 12405 -1404 1189
902 C
ATOM 5712 CZ TYR A 758 8.927 -4.698 -7.805 1.00 93.56
C
ANISOU 5712 CZ TYR A 758 11356 13006 11186 -1219 1213
949 C
ATOM 5713 OH TYR A 758 8.759 -5.977 -7.319 1.00 83.93
O
ANISOU 5713 OH TYR A 758 9940 11660 10291 -1100 1220
753 O
ATOM 5714 N LEU A 759 12.211 -0.884 -7.951 1.00 90.94

```
N
ANISOU 5714  N   LEU A 759    11683  12830  10041  -1987   1117
1582       N
ATOM   5715  CA  LEU A 759      13.180  -1.634  -7.155  1.00 96.90
C
ANISOU 5715  CA  LEU A 759    12298  13487  11032  -2024   1053
1430       C
ATOM   5716  C   LEU A 759      13.685  -0.844  -5.948  1.00102.89
C
ANISOU 5716  C   LEU A 759    13400  13910  11784  -2105    973
1626       C
ATOM   5717  O   LEU A 759      13.798  -1.392  -4.845  1.00118.36
O
ANISOU 5717  O   LEU A 759    15427  15606  13941  -2011    895
1587       O
ATOM   5718  CB  LEU A 759      14.337  -2.081  -8.054  1.00 79.95
C
ANISOU 5718  CB  LEU A 759     9774  11719   8885  -2199   1073
1204       C
ATOM   5719  CG  LEU A 759      15.257  -3.217  -7.587  1.00 79.79
C
ANISOU 5719  CG  LEU A 759     9455  11692   9169  -2188    992
935       C
ATOM   5720  CD1 LEU A 759      16.464  -2.719  -6.815  1.00 73.90
C
ANISOU 5720  CD1 LEU A 759     8814  10802   8462  -2357    896
987       C
ATOM   5721  CD2 LEU A 759      14.469  -4.227  -6.759  1.00 71.53
C
ANISOU 5721  CD2 LEU A 759     8420  10371   8388  -1935    938
867       C
ATOM   5722  N   GLN A 760      14.001   0.439  -6.135  1.00 72.36
N
ANISOU 5722  N   GLN A 760     9765  10042   7688  -2280    983
1837       N
ATOM   5723  CA  GLN A 760      14.423   1.288  -5.020  1.00 72.30
C
ANISOU 5723  CA  GLN A 760    10094   9711   7668  -2356    900
2015       C
ATOM   5724  C   GLN A 760      13.355   1.421  -3.934  1.00 98.07
C
ANISOU 5724  C   GLN A 760    13684  12597  10983  -2149    881
2147       C
ATOM   5725  O   GLN A 760      13.648   1.279  -2.741  1.00 70.25
O
ANISOU 5725  O   GLN A 760    10321   8800   7571  -2113    796
2155       O
ATOM   5726  CB  GLN A 760      14.813   2.666  -5.565  1.00 73.94
C
ANISOU 5726  CB  GLN A 760    10484   9984   7625  -2583    932
2221       C
ATOM   5727  CG  GLN A 760      15.441   3.624  -4.561  1.00108.35
C
ANISOU 5727  CG  GLN A 760    15147  14046  11973  -2708    842
```

```
2376       C
ATOM   5728  CD  GLN A 760      14.410   4.430  -3.787  1.00105.24
C
ANISOU 5728  CD  GLN A 760    15172  13299  11515  -2569    820
2598       C
ATOM   5729  OE1 GLN A 760      13.295   4.654  -4.260  1.00 73.13
O
ANISOU 5729  OE1 GLN A 760    11193   9240   7355  -2438    881
2691       O
ATOM   5730  NE2 GLN A 760      14.788   4.884  -2.597  1.00 75.01
N
ANISOU 5730  NE2 GLN A 760    11597   9164   7741  -2591    722
2663       N
ATOM   5731  N   VAL A 761      12.110   1.690  -4.329  1.00 92.96
N
ANISOU 5731  N   VAL A 761    13136  11933  10252  -2011    956
2236       N
ATOM   5732  CA  VAL A 761      11.001   1.784  -3.378  1.00 69.14
C
ANISOU 5732  CA  VAL A 761    10388   8583   7300  -1814    975
2325       C
ATOM   5733  C   VAL A 761      10.772   0.481  -2.615  1.00 77.01
C
ANISOU 5733  C   VAL A 761    11267   9455   8537  -1659    984
2159       C
ATOM   5734  O   VAL A 761      10.569   0.493  -1.394  1.00104.70
O
ANISOU 5734  O   VAL A 761    15033  12646  12103  -1588    967
2223       O
ATOM   5735  CB  VAL A 761       9.724   2.242  -4.105  1.00 95.34
C
ANISOU 5735  CB  VAL A 761    13758  11947  10521  -1689   1045
2403       C
ATOM   5736  CG1 VAL A 761       8.513   2.106  -3.195  1.00106.26
C
ANISOU 5736  CG1 VAL A 761    15323  13022  12027  -1473   1099
2419       C
ATOM   5737  CG2 VAL A 761       9.882   3.681  -4.565  1.00 70.60
C
ANISOU 5737  CG2 VAL A 761    10862   8815   7148  -1829   1011
2628       C
ATOM   5738  N   SER A 762      10.801  -0.657  -3.310  1.00 67.85
N
ANISOU 5738  N   SER A 762     9733   8533   7512  -1609   1010
1945       N
ATOM   5739  CA  SER A 762      10.531  -1.942  -2.663  1.00 67.01
C
ANISOU 5739  CA  SER A 762     9511   8294   7657  -1462   1019
1793       C
ATOM   5740  C   SER A 762      11.530  -2.284  -1.558  1.00 90.64
C
ANISOU 5740  C   SER A 762    12608  11086  10746  -1514    897
1783       C
ATOM   5741  O   SER A 762      11.130  -2.688  -0.460  1.00 76.19
```

```
O
ANISOU 5741  O   SER A 762    10981   8957   9011  -1405    899
1822       O
ATOM   5742  CB  SER A 762      10.506  -3.052  -3.715  1.00 78.12
C
ANISOU 5742  CB  SER A 762    10470  10008   9203  -1416   1050
1544       C
ATOM   5743  OG  SER A 762      10.230  -4.310  -3.121  1.00102.73
O
ANISOU 5743  OG  SER A 762    13474  12972  12587  -1280   1056
1401       O
ATOM   5744  N   ILE A 763      12.830  -2.143  -1.824  1.00 68.04
N
ANISOU 5744  N   ILE A 763     9609   8382   7860  -1681    788
1724       N
ATOM   5745  CA  ILE A 763      13.840  -2.491  -0.819  1.00 83.14
C
ANISOU 5745  CA  ILE A 763    11591  10115   9885  -1717    627
1685       C
ATOM   5746  C   ILE A 763      13.732  -1.609   0.423  1.00112.44
C
ANISOU 5746  C   ILE A 763    15770  13479  13471  -1719    576
1899       C
ATOM   5747  O   ILE A 763      13.745  -2.101   1.558  1.00115.08
O
ANISOU 5747  O   ILE A 763    16296  13542  13888  -1627    498
1913       O
ATOM   5748  CB  ILE A 763      15.252  -2.419  -1.427  1.00100.99
C
ANISOU 5748  CB  ILE A 763    13575  12632  12164  -1907    529
1548       C
ATOM   5749  CG1 ILE A 763      15.409  -3.431  -2.559  1.00102.64
C
ANISOU 5749  CG1 ILE A 763    13307  13180  12512  -1893    578
1291       C
ATOM   5750  CG2 ILE A 763      16.308  -2.655  -0.356  1.00143.27
C
ANISOU 5750  CG2 ILE A 763    19013  17783  17641  -1934    322
1503       C
ATOM   5751  CD1 ILE A 763      16.706  -3.288  -3.309  1.00146.81
C
ANISOU 5751  CD1 ILE A 763    18601  19075  18106  -2098    536
1135       C
ATOM   5752  N   ILE A 764      13.625  -0.295   0.227  1.00111.84
N
ANISOU 5752  N   ILE A 764    15901  13403  13190  -1823    614
2068       N
ATOM   5753  CA  ILE A 764      13.471   0.632   1.348  1.00 91.70
C
ANISOU 5753  CA  ILE A 764    13792  10531  10519  -1823    572
2251       C
ATOM   5754  C   ILE A 764      12.180   0.383   2.127  1.00 67.86
C
ANISOU 5754  C   ILE A 764    11018   7253   7512  -1629    684
```

```
2321       C
ATOM   5755  O   ILE A 764      12.167   0.446   3.363  1.00 67.92
O
ANISOU 5755  O   ILE A 764    11340   6972   7494  -1581    633
2387       O
ATOM   5756  CB  ILE A 764      13.561   2.083   0.843  1.00123.95
C
ANISOU 5756  CB  ILE A 764    18019  14667  14409  -1974    596
2408       C
ATOM   5757  CG1 ILE A 764      14.959   2.356   0.288  1.00145.30
C
ANISOU 5757  CG1 ILE A 764    20517  17583  17109  -2202    500
2341       C
ATOM   5758  CG2 ILE A 764      13.261   3.056   1.960  1.00 69.55
C
ANISOU 5758  CG2 ILE A 764    11577   7439   7410  -1954    563
2576       C
ATOM   5759  CD1 ILE A 764      16.062   2.111   1.288  1.00 92.05
C
ANISOU 5759  CD1 ILE A 764    13821  10687  10468  -2252    311
2260       C
ATOM   5760  N   SER A 765      11.081   0.089   1.430  1.00 88.85
N
ANISOU 5760  N   SER A 765    13536  10014  10207  -1519    842
2292       N
ATOM   5761  CA  SER A 765       9.844  -0.281   2.119  1.00 78.10
C
ANISOU 5761  CA  SER A 765    12344   8425   8905  -1344    978
2315       C
ATOM   5762  C   SER A 765       9.994  -1.538   2.975  1.00 74.95
C
ANISOU 5762  C   SER A 765    11940   7869   8667  -1264    953
2227       C
ATOM   5763  O   SER A 765       9.511  -1.576   4.112  1.00107.12
O
ANISOU 5763  O   SER A 765    16332  11655  12716  -1191   1006
2305       O
ATOM   5764  CB  SER A 765       8.719  -0.463   1.101  1.00113.87
C
ANISOU 5764  CB  SER A 765    16651  13123  13491  -1243   1127
2254       C
ATOM   5765  OG  SER A 765       8.509   0.728   0.361  1.00 99.42
O
ANISOU 5765  OG  SER A 765    14879  11401  11495  -1299   1129
2362       O
ATOM   5766  N   GLN A 766      10.650  -2.577   2.454  1.00 68.50
N
ANISOU 5766  N   GLN A 766    10781   7232   8014  -1276    874
2065       N
ATOM   5767  CA  GLN A 766      10.898  -3.768   3.267  1.00 66.71
C
ANISOU 5767  CA  GLN A 766    10566   6829   7952  -1201    808
1994       C
ATOM   5768  C   GLN A 766      11.857  -3.488   4.422  1.00 67.58
```

C
ANISOU 5768 C   GLN A 766   10984   6722   7974 -1257   614
2079   C
ATOM   5769 O   GLN A 766   11.646 -3.971   5.541 1.00 68.02
O
ANISOU 5769 O   GLN A 766   11312   6496   8037 -1178   601
2139   O
ATOM   5770 CB GLN A 766   11.439 -4.896   2.387 1.00 66.83
C
ANISOU 5770 CB GLN A 766   10120   7084   8187 -1196   742
1776   C
ATOM   5771 CG GLN A 766   11.095 -6.302   2.867 1.00 66.99
C
ANISOU 5771 CG GLN A 766   10074   6936   8441 -1069   759
1685   C
ATOM   5772 CD GLN A 766   9.641 -6.659   2.652 1.00 66.35
C
ANISOU 5772 CD GLN A 766   9956   6811   8444   -958   1000
1674   C
ATOM   5773 OE1 GLN A 766   9.107 -6.497   1.555 1.00129.43
O
ANISOU 5773 OE1 GLN A 766   17683 15046 16447   -947   1102
1587   O
ATOM   5774 NE2 GLN A 766   8.994 -7.157   3.697 1.00102.30
N
ANISOU 5774 NE2 GLN A 766   14771 11047 13050   -878   1093
1753   N
ATOM   5775 N   ALA A 767   12.919 -2.721   4.169 1.00 87.55
N
ANISOU 5775 N   ALA A 767   13477   9373 10413 -1397   461
2082   N
ATOM   5776 CA ALA A 767   13.884 -2.372   5.213 1.00111.59
C
ANISOU 5776 CA ALA A 767   16788 12226 13384 -1453   246
2135   C
ATOM   5777 C   ALA A 767   13.261 -1.596   6.372 1.00 92.46
C
ANISOU 5777 C   ALA A 767   14869   9500 10762 -1413   299
2317   C
ATOM   5778 O   ALA A 767   13.701 -1.739   7.519 1.00101.67
O
ANISOU 5778 O   ALA A 767   16322 10432 11875 -1386   148
2358   O
ATOM   5779 CB ALA A 767   15.036 -1.572   4.607 1.00156.77
C
ANISOU 5779 CB ALA A 767   22346 18153 19066 -1634   113
2090   C
ATOM   5780 N   LEU A 768   12.253 -0.766   6.092 1.00 73.82
N
ANISOU 5780 N   LEU A 768   12623   7139   8287 -1403   499
2415   N
ATOM   5781 CA LEU A 768   11.553 -0.014   7.133 1.00 68.91
C
ANISOU 5781 CA LEU A 768   12450   6240   7491 -1357   583

```
2555       C
ATOM   5782  C   LEU A 768      11.007  -0.880   8.269  1.00 69.32
C
ANISOU 5782  C   LEU A 768    12755   6032   7550  -1234    649
2578       C
ATOM   5783  O   LEU A 768      10.892  -0.401   9.403  1.00 83.84
O
ANISOU 5783  O   LEU A 768    15008   7628   9220  -1219    638
2671       O
ATOM   5784  CB  LEU A 768      10.418   0.782   6.491  1.00 83.71
C
ANISOU 5784  CB  LEU A 768    14320   8172   9314  -1329    796
2612       C
ATOM   5785  CG  LEU A 768       9.562   1.671   7.387  1.00 70.23
C
ANISOU 5785  CG  LEU A 768    13022   6206   7456  -1275    915
2722       C
ATOM   5786  CD1 LEU A 768      10.402   2.776   8.023  1.00 69.57
C
ANISOU 5786  CD1 LEU A 768    13227   6000   7208  -1383    740
2800       C
ATOM   5787  CD2 LEU A 768       8.433   2.253   6.566  1.00123.92
C
ANISOU 5787  CD2 LEU A 768    19721  13088  14274  -1220   1098
2737       C
ATOM   5788  N   ILE A 769      10.663  -2.140   8.000  1.00 71.67
N
ANISOU 5788  N   ILE A 769    12828   6370   8032  -1153    724
2495       N
ATOM   5789  CA  ILE A 769      10.284  -3.043   9.088  1.00 69.79
C
ANISOU 5789  CA  ILE A 769    12843   5869   7803  -1063    773
2533       C
ATOM   5790  C   ILE A 769      11.400  -3.177  10.117  1.00 81.95
C
ANISOU 5790  C   ILE A 769    14649   7240   9247  -1086    492
2573       C
ATOM   5791  O   ILE A 769      11.157  -3.137  11.329  1.00110.82
O
ANISOU 5791  O   ILE A 769    18739  10634  12736  -1048    509
2677       O
ATOM   5792  CB  ILE A 769       9.887  -4.424   8.536  1.00 69.41
C
ANISOU 5792  CB  ILE A 769    12468   5891   8015   -989    865
2424       C
ATOM   5793  CG1 ILE A 769       8.640  -4.328   7.658  1.00 68.30
C
ANISOU 5793  CG1 ILE A 769    12101   5883   7968   -945   1143
2372       C
ATOM   5794  CG2 ILE A 769       9.731  -5.412   9.672  1.00 70.70
C
ANISOU 5794  CG2 ILE A 769    12907   5763   8192   -922    872
2483       C
ATOM   5795  CD1 ILE A 769       8.321  -5.599   6.902  1.00115.80
```

```
C
ANISOU 5795  CD1 ILE A 769    17722  12015  14262   -884   1212
2224       C
ATOM   5796  N   PHE A 770      12.638  -3.326   9.651  1.00 85.07
N
ANISOU 5796  N   PHE A 770    14790   7789   9742  -1146    227
2476       N
ATOM   5797  CA  PHE A 770      13.783  -3.405  10.553  1.00 75.53
C
ANISOU 5797  CA  PHE A 770    13790   6435   8472  -1160    -90
2481       C
ATOM   5798  C   PHE A 770      13.989  -2.119  11.350  1.00 73.78
C
ANISOU 5798  C   PHE A 770    13963   6081   7989  -1220   -159
2582       C
ATOM   5799  O   PHE A 770      14.347  -2.168  12.533  1.00109.91
O
ANISOU 5799  O   PHE A 770    18915  10425  12419  -1184   -326
2641       O
ATOM   5800  CB  PHE A 770      15.048  -3.742   9.759  1.00106.43
C
ANISOU 5800  CB  PHE A 770    17279  10572  12590  -1222   -339
2313       C
ATOM   5801  CG  PHE A 770      14.976  -5.044   9.003  1.00112.61
C
ANISOU 5801  CG  PHE A 770    17658  11481  13648  -1157   -308
2176       C
ATOM   5802  CD1 PHE A 770      15.708  -6.141   9.428  1.00 99.39
C
ANISOU 5802  CD1 PHE A 770    15930   9693  12139  -1083   -556
2095       C
ATOM   5803  CD2 PHE A 770      14.214  -5.161   7.850  1.00147.40
C
ANISOU 5803  CD2 PHE A 770    21734  16115  18157  -1162    -60
2114       C
ATOM   5804  CE1 PHE A 770      15.661  -7.337   8.735  1.00 94.28
C
ANISOU 5804  CE1 PHE A 770    14905   9146  11772  -1019   -540
1950       C
ATOM   5805  CE2 PHE A 770      14.161  -6.354   7.154  1.00143.75
C
ANISOU 5805  CE2 PHE A 770    20893  15770  17956  -1101    -41
1962       C
ATOM   5806  CZ  PHE A 770      14.889  -7.442   7.596  1.00115.22
C
ANISOU 5806  CZ  PHE A 770    17223  12032  14523  -1032   -275
1876       C
ATOM   5807  N   VAL A 771      13.778  -0.962  10.725  1.00 72.91
N
ANISOU 5807  N   VAL A 771    13784   6105   7812  -1309    -50
2600       N
ATOM   5808  CA  VAL A 771      13.827   0.306  11.452  1.00 73.65
C
ANISOU 5808  CA  VAL A 771    14252   6053   7680  -1362    -87
```

```
2688       C
ATOM   5809  C   VAL A 771      12.701   0.415  12.478  1.00 90.35
C
ANISOU 5809  C   VAL A 771    16697   7987   9645  -1242    117
2744       C
ATOM   5810  O   VAL A 771      12.944   0.706  13.656  1.00 82.75
O
ANISOU 5810  O   VAL A 771    15995   6906   8540  -1196      3
2721       O
ATOM   5811  CB  VAL A 771      13.794   1.485  10.463  1.00153.11
C
ANISOU 5811  CB  VAL A 771    24135  16299  17740  -1478     -9
2694       C
ATOM   5812  CG1 VAL A 771      13.727   2.811  11.212  1.00 73.69
C
ANISOU 5812  CG1 VAL A 771    14463   6060   7476  -1523    -32
2777       C
ATOM   5813  CG2 VAL A 771      15.009   1.439   9.545  1.00172.80
C
ANISOU 5813  CG2 VAL A 771    26232  19037  20386  -1606   -196
2583       C
ATOM   5814  N   THR A 772      11.456   0.184  12.054  1.00 96.00
N
ANISOU 5814  N   THR A 772    17262   8775  10440  -1166    417
2733       N
ATOM   5815  CA  THR A 772      10.316   0.382  12.949  1.00 73.56
C
ANISOU 5815  CA  THR A 772    14548   5875   7528  -1050    627
2691       C
ATOM   5816  C   THR A 772      10.306  -0.583  14.132  1.00 75.09
C
ANISOU 5816  C   THR A 772    14954   5918   7660   -974    599
2689       C
ATOM   5817  O   THR A 772       9.973  -0.182  15.253  1.00109.52
O
ANISOU 5817  O   THR A 772    19560  10196  11858   -929    639
2659       O
ATOM   5818  CB  THR A 772       9.010   0.260  12.164  1.00 72.29
C
ANISOU 5818  CB  THR A 772    14177   5798   7493  -1001    928
2673       C
ATOM   5819  OG1 THR A 772       9.041  -0.927  11.363  1.00 76.67
O
ANISOU 5819  OG1 THR A 772    14544   6377   8211  -1010    973
2696       O
ATOM   5820  CG2 THR A 772       8.823   1.470  11.262  1.00119.20
C
ANISOU 5820  CG2 THR A 772    19989  11864  13437  -1049    959
2679       C
ATOM   5821  N   ARG A 773      10.657  -1.853  13.915  1.00 75.10
N
ANISOU 5821  N   ARG A 773    14890   5865   7780   -964    531
2724       N
ATOM   5822  CA  ARG A 773      10.723  -2.792  15.035  1.00 83.67
```

```
C
ANISOU 5822  CA  ARG A 773    16194   6792   8806   -894    473
2743       C
ATOM   5823  C   ARG A 773      11.785  -2.400  16.058  1.00106.09
C
ANISOU 5823  C   ARG A 773    19331   9532  11448   -901    162
2747       C
ATOM   5824  O   ARG A 773      11.587  -2.585  17.265  1.00109.87
O
ANISOU 5824  O   ARG A 773    20082   9904  11761   -845    166
2749       O
ATOM   5825  CB  ARG A 773      10.985  -4.214  14.537  1.00 76.77
C
ANISOU 5825  CB  ARG A 773    15195   5842   8130   -879    416
2781       C
ATOM   5826  CG  ARG A 773      12.380  -4.434  13.984  1.00 84.23
C
ANISOU 5826  CG  ARG A 773    16100   6752   9150   -945     62
2800       C
ATOM   5827  CD  ARG A 773      12.493  -5.765  13.287  1.00160.00
C
ANISOU 5827  CD  ARG A 773    25356  16396  19043   -903     32
2728       C
ATOM   5828  NE  ARG A 773      12.407  -6.865  14.242  1.00 81.71
N
ANISOU 5828  NE  ARG A 773    15733   6204   9111   -825    -21
2818       N
ATOM   5829  CZ  ARG A 773      13.453  -7.382  14.876  1.00 80.89
C
ANISOU 5829  CZ  ARG A 773    15773   5961   8999   -782   -387
2826       C
ATOM   5830  NH1 ARG A 773      14.656  -6.843  14.730  1.00130.69
N
ANISOU 5830  NH1 ARG A 773    21960  12382  15315   -815   -721
2731       N
ATOM   5831  NH2 ARG A 773      13.281  -8.389  15.721  1.00 82.06
N
ANISOU 5831  NH2 ARG A 773    16176   5871   9131   -704   -419
2915       N
ATOM   5832  N   SER A 774      12.913  -1.861  15.603  1.00144.76
N
ANISOU 5832  N   SER A 774    24199  14452  16350   -983   -112
2747       N
ATOM   5833  CA  SER A 774      14.041  -1.608  16.489  1.00115.92
C
ANISOU 5833  CA  SER A 774    20822  10677  12545   -993   -465
2742       C
ATOM   5834  C   SER A 774      13.904  -0.277  17.215  1.00112.85
C
ANISOU 5834  C   SER A 774    20641  10292  11943  -1010   -438
2694       C
ATOM   5835  O   SER A 774      13.499   0.732  16.631  1.00125.37
O
ANISOU 5835  O   SER A 774    22096  11989  13550  -1065   -285
```

```
2670       O
ATOM   5836  CB  SER A 774      15.348  -1.626  15.696  1.00123.91
C
ANISOU 5836  CB  SER A 774    21722  11676  13681  -1099   -802
2755       C
ATOM   5837  OG  SER A 774      15.256  -0.799  14.548  1.00109.34
O
ANISOU 5837  OG  SER A 774    19608  10011  11924  -1217   -676
2738       O
ATOM   5838  N   ARG A 775      14.246  -0.286  18.504  1.00119.19
N
ANISOU 5838  N   ARG A 775    21791  10957  12537   -960   -604
2680       N
ATOM   5839  CA  ARG A 775      14.260   0.936  19.294  1.00160.24
C
ANISOU 5839  CA  ARG A 775    27250  16117  17519   -980   -632
2623       C
ATOM   5840  C   ARG A 775      15.634   1.590  19.288  1.00147.21
C
ANISOU 5840  C   ARG A 775    25700  14397  15835  -1066  -1028
2605       C
ATOM   5841  O   ARG A 775      15.729   2.822  19.327  1.00144.15
O
ANISOU 5841  O   ARG A 775    25384  14013  15374  -1135  -1039
2562       O
ATOM   5842  CB  ARG A 775      13.847   0.632  20.739  1.00151.64
C
ANISOU 5842  CB  ARG A 775    26531  14904  16180   -894   -590
2611       C
ATOM   5843  CG  ARG A 775      12.357   0.379  20.944  1.00156.58
C
ANISOU 5843  CG  ARG A 775    27132  15571  16791   -844   -165
2608       C
ATOM   5844  CD  ARG A 775      11.496   1.596  20.636  1.00165.83
C
ANISOU 5844  CD  ARG A 775    28229  16817  17960   -872     90
2544       C
ATOM   5845  NE  ARG A 775      10.094   1.347  20.966  1.00174.81
N
ANISOU 5845  NE  ARG A 775    29396  17953  19070   -824    466
2525       N
ATOM   5846  CZ  ARG A 775       9.350   2.136  21.736  1.00147.35
C
ANISOU 5846  CZ  ARG A 775    26169  14411  15406   -813    649
2456       C
ATOM   5847  NH1 ARG A 775       9.893   3.188  22.330  1.00150.24
N
ANISOU 5847  NH1 ARG A 775    26800  14702  15582   -837    481
2404       N
ATOM   5848  NH2 ARG A 775       8.080   1.829  21.972  1.00117.13
N
ANISOU 5848  NH2 ARG A 775    22363  10568  11573   -781    999
2434       N
ATOM   5849  N   SER A 776      16.695   0.782  19.231  1.00129.63
```

```
N
ANISOU 5849  N   SER A 776    23483  12085  13685  -1067  -1374
2633       N
ATOM   5850  CA  SER A 776      18.055   1.255  18.999  1.00141.12
C
ANISOU 5850  CA  SER A 776    24967  13462  15188  -1171  -1788
2618       C
ATOM   5851  C   SER A 776      18.400   1.103  17.524  1.00188.51
C
ANISOU 5851  C   SER A 776    30543  19604  21480  -1287  -1776
2628       C
ATOM   5852  O   SER A 776      17.508   1.142  16.670  1.00222.13
O
ANISOU 5852  O   SER A 776    34590  23993  25818  -1315  -1429
2676       O
ATOM   5853  CB  SER A 776      19.055   0.488  19.868  1.00144.21
C
ANISOU 5853  CB  SER A 776    25611  13664  15518  -1089  -2238
2612       C
ATOM   5854  OG  SER A 776      20.338   1.090  19.825  1.00151.22
O
ANISOU 5854  OG  SER A 776    26462  14515  16480  -1172  -2646
2518       O
ATOM   5855  N   TRP A 777      19.687   0.942  17.211  1.00196.53
N
ANISOU 5855  N   TRP A 777    31256  20713  22704  -1331  -2117
2477       N
ATOM   5856  CA  TRP A 777      20.085   0.714  15.829  1.00188.70
C
ANISOU 5856  CA  TRP A 777    29671  19996  22033  -1424  -2061
2365       C
ATOM   5857  C   TRP A 777      19.473  -0.577  15.294  1.00172.04
C
ANISOU 5857  C   TRP A 777    27346  17954  20069  -1327  -1885
2393       C
ATOM   5858  O   TRP A 777      19.411  -1.596  15.987  1.00184.57
O
ANISOU 5858  O   TRP A 777    29127  19374  21628  -1191  -2003
2434       O
ATOM   5859  CB  TRP A 777      21.609   0.662  15.703  1.00163.02
C
ANISOU 5859  CB  TRP A 777    26130  16816  18993  -1482  -2462
2165       C
ATOM   5860  CG  TRP A 777      22.317   1.884  16.197  1.00155.26
C
ANISOU 5860  CG  TRP A 777    25309  15764  17919  -1589  -2664
2103       C
ATOM   5861  CD1 TRP A 777      22.938   2.046  17.401  1.00177.76
C
ANISOU 5861  CD1 TRP A 777    28513  18400  20627  -1522  -3018
2061       C
ATOM   5862  CD2 TRP A 777      22.514   3.107  15.476  1.00140.37
C
ANISOU 5862  CD2 TRP A 777    23230  14016  16090  -1787  -2540
```

```
2067       C
ATOM   5863  NE1 TRP A 777      23.491   3.302  17.482  1.00181.94
N
ANISOU 5863  NE1 TRP A 777    29064  18926  21139  -1666  -3117
1983       N
ATOM   5864  CE2 TRP A 777      23.246   3.973  16.313  1.00140.80
C
ANISOU 5864  CE2 TRP A 777    23523  13918  16055  -1836  -2820
1995       C
ATOM   5865  CE3 TRP A 777      22.134   3.557  14.208  1.00148.03
C
ANISOU 5865  CE3 TRP A 777    23866  15210  17166  -1927  -2227
2096       C
ATOM   5866  CZ2 TRP A 777      23.606   5.262  15.922  1.00126.50
C
ANISOU 5866  CZ2 TRP A 777    21618  12159  14287  -2032  -2782
1952       C
ATOM   5867  CZ3 TRP A 777      22.491   4.837  13.822  1.00136.42
C
ANISOU 5867  CZ3 TRP A 777    22331  13791  15711  -2119  -2194
2077       C
ATOM   5868  CH2 TRP A 777      23.219   5.674  14.676  1.00125.40
C
ANISOU 5868  CH2 TRP A 777    21168  12226  14254  -2176  -2462
2006       C
ATOM   5869  N   SER A 778      19.017  -0.513  14.043  1.00118.92
N
ANISOU 5869  N   SER A 778    20225  11464  13493  -1402  -1608
2373       N
ATOM   5870  CA  SER A 778      18.408  -1.662  13.379  1.00109.32
C
ANISOU 5870  CA  SER A 778    18746  10343  12447  -1324  -1423
2367       C
ATOM   5871  C   SER A 778      19.377  -2.834  13.247  1.00127.85
C
ANISOU 5871  C   SER A 778    20804  12722  15051  -1262  -1717
2215       C
ATOM   5872  O   SER A 778      18.989  -3.995  13.422  1.00141.06
O
ANISOU 5872  O   SER A 778    22493  14299  16805  -1138  -1697
2241       O
ATOM   5873  CB  SER A 778      17.885  -1.238  12.009  1.00 90.99
C
ANISOU 5873  CB  SER A 778    16047   8298  10228  -1424  -1124
2345       C
ATOM   5874  OG  SER A 778      17.193  -2.300  11.386  1.00156.57
O
ANISOU 5874  OG  SER A 778    24112  16691  18687  -1343   -934
2327       O
ATOM   5875  N   PHE A 779      20.640  -2.546  12.930  1.00132.16
N
ANISOU 5875  N   PHE A 779    21070  13393  15751  -1350  -1989
2041       N
ATOM   5876  CA  PHE A 779      21.685  -3.568  12.931  1.00139.68
```

```
C
ANISOU 5876  CA  PHE A 779    21756  14349  16965  -1279  -2326
1860       C
ATOM   5877  C   PHE A 779      21.894  -4.189  14.309  1.00162.83
C
ANISOU 5877  C   PHE A 779    25129  16952  19786  -1115  -2636
1932       C
ATOM   5878  O   PHE A 779      22.056  -5.410  14.425  1.00167.79
O
ANISOU 5878  O   PHE A 779    25676  17492  20584   -983  -2790
1888       O
ATOM   5879  CB  PHE A 779      22.992  -2.960  12.418  1.00151.75
C
ANISOU 5879  CB  PHE A 779    22921  16065  18671  -1425  -2542
1645       C
ATOM   5880  CG  PHE A 779      24.134  -3.934  12.347  1.00174.71
C
ANISOU 5880  CG  PHE A 779    25490  18998  21894  -1355  -2898
1407       C
ATOM   5881  CD1 PHE A 779      24.151  -4.942  11.395  1.00179.30
C
ANISOU 5881  CD1 PHE A 779    25609  19763  22755  -1322  -2817
1264       C
ATOM   5882  CD2 PHE A 779      25.196  -3.837  13.232  1.00184.63
C
ANISOU 5882  CD2 PHE A 779    26878  20091  23183  -1313  -3333
1303       C
ATOM   5883  CE1 PHE A 779      25.207  -5.837  11.328  1.00172.87
C
ANISOU 5883  CE1 PHE A 779    24462  18960  22260  -1246  -3155
1017       C
ATOM   5884  CE2 PHE A 779      26.253  -4.728  13.172  1.00192.84
C
ANISOU 5884  CE2 PHE A 779    27587  21139  24543  -1231  -3689
1061       C
ATOM   5885  CZ  PHE A 779      26.259  -5.730  12.219  1.00178.29
C
ANISOU 5885  CZ  PHE A 779    25276  19473  22992  -1197  -3595
916       C
ATOM   5886  N   VAL A 780      21.909  -3.371  15.362  1.00163.52
N
ANISOU 5886  N   VAL A 780    25696  16849  19585  -1118  -2743
2040       N
ATOM   5887  CA  VAL A 780      22.168  -3.882  16.708  1.00135.92
C
ANISOU 5887  CA  VAL A 780    22666  13047  15930   -967  -3065
2112       C
ATOM   5888  C   VAL A 780      21.094  -4.871  17.158  1.00131.66
C
ANISOU 5888  C   VAL A 780    22428  12322  15274   -838  -2867
2305       C
ATOM   5889  O   VAL A 780      21.409  -5.932  17.711  1.00136.66
O
ANISOU 5889  O   VAL A 780    23187  12770  15969   -699  -3128
```

2320 O
ATOM 5890 CB VAL A 780 22.303 -2.711 17.700 1.00150.54
C
ANISOU 5890 CB VAL A 780 24980 14756 17461 -1007 -3178
2177 C
ATOM 5891 CG1 VAL A 780 22.377 -3.230 19.128 1.00171.71
C
ANISOU 5891 CG1 VAL A 780 28225 17122 19895 -847 -3464
2286 C
ATOM 5892 CG2 VAL A 780 23.533 -1.880 17.370 1.00164.59
C
ANISOU 5892 CG2 VAL A 780 26459 16672 19404 -1131 -3444
1963 C
ATOM 5893 N GLU A 781 19.820 -4.558 16.926 1.00128.48
N
ANISOU 5893 N GLU A 781 22139 11953 14724 -882 -2412
2451 N
ATOM 5894 CA GLU A 781 18.739 -5.473 17.295 1.00115.25
C
ANISOU 5894 CA GLU A 781 20713 10110 12968 -787 -2170
2619 C
ATOM 5895 C GLU A 781 18.526 -6.544 16.226 1.00109.09
C
ANISOU 5895 C GLU A 781 19439 9469 12539 -760 -2042
2532 C
ATOM 5896 O GLU A 781 18.108 -6.244 15.102 1.00116.79
O
ANISOU 5896 O GLU A 781 20020 10698 13657 -844 -1767
2459 O
ATOM 5897 CB GLU A 781 17.455 -4.690 17.553 1.00112.15
C
ANISOU 5897 CB GLU A 781 20636 9683 12295 -840 -1742
2776 C
ATOM 5898 CG GLU A 781 17.486 -3.894 18.851 1.00132.89
C
ANISOU 5898 CG GLU A 781 23673 12235 14586 -815 -1803
2783 C
ATOM 5899 CD GLU A 781 16.208 -3.118 19.097 1.00137.31
C
ANISOU 5899 CD GLU A 781 24259 12930 14980 -832 -1337
2774 C
ATOM 5900 OE1 GLU A 781 15.125 -3.739 19.096 1.00153.83
O
ANISOU 5900 OE1 GLU A 781 26279 15059 17112 -784 -999
2807 O
ATOM 5901 OE2 GLU A 781 16.287 -1.887 19.296 1.00111.53
O
ANISOU 5901 OE2 GLU A 781 21089 9714 11572 -893 -1330
2728 O
ATOM 5902 N ARG A 782 18.827 -7.790 16.588 1.00106.44
N
ANISOU 5902 N ARG A 782 19142 8959 12341 -637 -2263
2535 N
ATOM 5903 CA ARG A 782 18.863 -8.893 15.636 1.00111.43

```
C
ANISOU 5903  CA  ARG A 782    19284   9701  13354   -595  -2237
2406       C
ATOM   5904  C   ARG A 782      17.477  -9.124  15.030  1.00104.21
C
ANISOU 5904  C   ARG A 782    18270   8859  12467   -626  -1742
2484       C
ATOM   5905  O   ARG A 782      16.472  -9.091  15.749  1.00154.30
O
ANISOU 5905  O   ARG A 782    25044  15013  18571   -615  -1489
2682       O
ATOM   5906  CB  ARG A 782      19.366 -10.164  16.327  1.00141.55
C
ANISOU 5906  CB  ARG A 782    23256  13239  17287   -443  -2584
2428       C
ATOM   5907  CG  ARG A 782      19.941 -11.222  15.394  1.00137.20
C
ANISOU 5907  CG  ARG A 782    22135  12801  17193   -383  -2742
2205       C
ATOM   5908  CD  ARG A 782      20.526 -12.385  16.188  1.00140.75
C
ANISOU 5908  CD  ARG A 782    22790  12930  17757   -217  -3153
2235       C
ATOM   5909  NE  ARG A 782      21.279 -13.315  15.349  1.00152.84
N
ANISOU 5909  NE  ARG A 782    23754  14562  19754   -147  -3380
1972       N
ATOM   5910  CZ  ARG A 782      22.576 -13.193  15.079  1.00134.09
C
ANISOU 5910  CZ  ARG A 782    21039  12310  17598   -126  -3767
1718       C
ATOM   5911  NH1 ARG A 782      23.260 -12.156  15.545  1.00 95.28
N
ANISOU 5911  NH1 ARG A 782    16281   7436  12485   -181  -3966
1696       N
ATOM   5912  NH2 ARG A 782      23.182 -14.090  14.313  1.00142.00
N
ANISOU 5912  NH2 ARG A 782    21515  13402  19038    -58  -3940
1458       N
ATOM   5913  N   PRO A 783      17.386  -9.370  13.716  1.00 96.78
N
ANISOU 5913  N   PRO A 783    16763   8193  11815   -665  -1591
2315       N
ATOM   5914  CA  PRO A 783      16.070  -9.484  13.062  1.00 88.13
C
ANISOU 5914  CA  PRO A 783    15537   7192  10754   -692  -1139
2358       C
ATOM   5915  C   PRO A 783      15.353 -10.802  13.332  1.00 85.71
C
ANISOU 5915  C   PRO A 783    15314   6665  10587   -598  -1023
2429       C
ATOM   5916  O   PRO A 783      14.124 -10.833  13.462  1.00 80.88
O
ANISOU 5916  O   PRO A 783    14870   5977   9885   -610   -657
```

```
2549       O
ATOM   5917  CB  PRO A 783      16.409  -9.342  11.571  1.00128.67
C
ANISOU 5917  CB  PRO A 783    20042  12706  16142   -760  -1092
2130       C
ATOM   5918  CG  PRO A 783      17.841  -9.769  11.472  1.00148.43
C
ANISOU 5918  CG  PRO A 783    22291  15251  18856   -733  -1509
1946       C
ATOM   5919  CD  PRO A 783      18.481  -9.284  12.736  1.00136.70
C
ANISOU 5919  CD  PRO A 783    21279  13528  17135   -712  -1803
2061       C
ATOM   5920  N   GLY A 784      16.110 -11.889  13.417  1.00 83.28
N
ANISOU 5920  N   GLY A 784    14880   6243  10521   -506  -1333
2345       N
ATOM   5921  CA  GLY A 784      15.589 -13.236  13.314  1.00 84.00
C
ANISOU 5921  CA  GLY A 784    14879   6174  10862   -426  -1250
2347       C
ATOM   5922  C   GLY A 784      15.372 -13.690  11.879  1.00 82.14
C
ANISOU 5922  C   GLY A 784    14004   6229  10978   -439  -1091
2113       C
ATOM   5923  O   GLY A 784      15.210 -12.899  10.951  1.00 82.90
O
ANISOU 5923  O   GLY A 784    13793   6649  11057   -524   -914
2004       O
ATOM   5924  N   ALA A 785      15.375 -15.018  11.715  1.00108.06
N
ANISOU 5924  N   ALA A 785    17104   9374  14580   -350  -1171
2035       N
ATOM   5925  CA  ALA A 785      15.371 -15.635  10.389  1.00132.11
C
ANISOU 5925  CA  ALA A 785    19519  12678  17998   -339  -1103
1765       C
ATOM   5926  C   ALA A 785      14.135 -15.291   9.559  1.00130.96
C
ANISOU 5926  C   ALA A 785    19174  12747  17837   -408   -651
1742       C
ATOM   5927  O   ALA A 785      14.251 -15.000   8.362  1.00137.10
O
ANISOU 5927  O   ALA A 785    19476  13886  18729   -452   -579
1531       O
ATOM   5928  CB  ALA A 785      15.498 -17.153  10.530  1.00157.85
C
ANISOU 5928  CB  ALA A 785    22690  15680  21605   -220  -1270
1706       C
ATOM   5929  N   LEU A 786      12.947 -15.322  10.167  1.00107.16
N
ANISOU 5929  N   LEU A 786    16515   9519  14680   -421   -346
1947       N
ATOM   5930  CA  LEU A 786      11.715 -15.129   9.401  1.00 90.91
```

```
C
ANISOU 5930  CA  LEU A 786    14243   7632  12666   -463     62
1898       C
ATOM   5931  C   LEU A 786      11.657 -13.767   8.718  1.00100.83
C
ANISOU 5931  C   LEU A 786    15364   9232  13715   -545    175
1854       C
ATOM   5932  O   LEU A 786      11.289 -13.667   7.542  1.00117.29
O
ANISOU 5932  O   LEU A 786    17022  11614  15930   -563    325
1682       O
ATOM   5933  CB  LEU A 786      10.497 -15.319  10.307  1.00 84.73
C
ANISOU 5933  CB  LEU A 786    13894   6541  11760   -475    374
2119       C
ATOM   5934  CG  LEU A 786       9.150 -15.216   9.584  1.00100.66
C
ANISOU 5934  CG  LEU A 786    15678   8696  13873   -505    789
2046       C
ATOM   5935  CD1 LEU A 786       9.038 -16.289   8.514  1.00 98.87
C
ANISOU 5935  CD1 LEU A 786    14907   8593  14068   -455    793
1797       C
ATOM   5936  CD2 LEU A 786       7.986 -15.300  10.561  1.00117.57
C
ANISOU 5936  CD2 LEU A 786    18255  10533  15883   -536   1119
2250       C
ATOM   5937  N   LEU A 787      12.008 -12.704   9.440  1.00 75.78
N
ANISOU 5937  N   LEU A 787    12567   6013  10215   -595     98
2011       N
ATOM   5938  CA  LEU A 787      12.042 -11.374   8.838  1.00106.55
C
ANISOU 5938  CA  LEU A 787    16365  10197  13922   -679    174
1987       C
ATOM   5939  C   LEU A 787      13.076 -11.255   7.721  1.00117.13
C
ANISOU 5939  C   LEU A 787    17218  11878  15408   -718    -25
1763       C
ATOM   5940  O   LEU A 787      12.788 -10.689   6.660  1.00113.85
O
ANISOU 5940  O   LEU A 787    16505  11767  14985   -774    128
1668       O
ATOM   5941  CB  LEU A 787      12.301 -10.322   9.913  1.00113.80
C
ANISOU 5941  CB  LEU A 787    17790  10961  14488   -723     98
2182       C
ATOM   5942  CG  LEU A 787      12.315  -8.895   9.370  1.00128.59
C
ANISOU 5942  CG  LEU A 787    19607  13082  16171   -816    173
2179       C
ATOM   5943  CD1 LEU A 787      11.007  -8.583   8.657  1.00 71.55
C
ANISOU 5943  CD1 LEU A 787    12240   5988   8959   -820    535
```

```
2169       C
ATOM   5944  CD2 LEU A 787      12.575  -7.899  10.476  1.00148.15
C
ANISOU 5944  CD2 LEU A 787    22582  15382  18326   -854     86
2349       C
ATOM   5945  N   MET A 788      14.286 -11.772   7.940  1.00100.51
N
ANISOU 5945  N   MET A 788    15029   9726  13433   -691   -366
1670       N
ATOM   5946  CA  MET A 788      15.284 -11.817   6.873  1.00126.77
C
ANISOU 5946  CA  MET A 788    17847  13375  16943   -732   -532
1415       C
ATOM   5947  C   MET A 788      14.822 -12.631   5.667  1.00 86.51
C
ANISOU 5947  C   MET A 788    12248   8499  12121   -700   -375
1199       C
ATOM   5948  O   MET A 788      14.946 -12.180   4.523  1.00104.91
O
ANISOU 5948  O   MET A 788    14215  11191  14456   -775   -287
1048       O
ATOM   5949  CB  MET A 788      16.600 -12.375   7.416  1.00132.41
C
ANISOU 5949  CB  MET A 788    18549  13956  17805   -682   -938
1325       C
ATOM   5950  CG  MET A 788      17.366 -11.405   8.299  1.00129.57
C
ANISOU 5950  CG  MET A 788    18545  13494  17193   -735  -1153
1448       C
ATOM   5951  SD  MET A 788      17.827  -9.881   7.447  1.00163.30
S
ANISOU 5951  SD  MET A 788    22615  18142  21290   -919  -1071
1385       S
ATOM   5952  CE  MET A 788      18.729 -10.528   6.041  1.00124.21
C
ANISOU 5952  CE  MET A 788    16945  13560  16688   -953  -1162
1020       C
ATOM   5953  N   ILE A 789      14.268 -13.823   5.898  1.00 74.71
N
ANISOU 5953  N   ILE A 789    10744   6792  10852   -596   -333
1183       N
ATOM   5954  CA  ILE A 789      13.750 -14.618   4.785  1.00 74.92
C
ANISOU 5954  CA  ILE A 789    10301   7010  11154   -559   -182
961      C
ATOM   5955  C   ILE A 789      12.624 -13.895   4.054  1.00 97.51
C
ANISOU 5955  C   ILE A 789    13092  10091  13867   -609    156
989      C
ATOM   5956  O   ILE A 789      12.591 -13.866   2.818  1.00113.63
O
ANISOU 5956  O   ILE A 789    14705  12479  15990   -634    230
783      O
ATOM   5957  CB  ILE A 789      13.297 -16.003   5.286  1.00 88.82
```

C
ANISOU 5957 CB ILE A 789   12113   8441 13192   -447   -190
963   C
ATOM   5958 CG1 ILE A 789   14.497 -16.810   5.782 1.00108.22
C
ANISOU 5958 CG1 ILE A 789   14550 10714 15854   -373   -578
884   C
ATOM   5959 CG2 ILE A 789   12.559 -16.756   4.188 1.00 86.95
C
ANISOU 5959 CG2 ILE A 789   11421   8380 13235   -410   4
738   C
ATOM   5960 CD1 ILE A 789   15.514 -17.112   4.706 1.00147.23
C
ANISOU 5960 CD1 ILE A 789   18921 15979 21042   -371   -766
542   C
ATOM   5961 N   ALA A 790   11.698 -13.287   4.795 1.00 83.85
N
ANISOU 5961 N   ALA A 790   11780   8167 11911   -621   355
1230   N
ATOM   5962 CA ALA A 790   10.659 -12.480   4.161 1.00 91.64
C
ANISOU 5962 CA ALA A 790   12721   9342 12757   -655   638
1256   C
ATOM   5963 C   ALA A 790   11.233 -11.304   3.375 1.00107.37
C
ANISOU 5963 C   ALA A 790   14578 11674 14544   -754   588
1223   C
ATOM   5964 O   ALA A 790   10.804 -11.037   2.247 1.00143.00
O
ANISOU 5964 O   ALA A 790   18790 16483 19059   -771   717
1107   O
ATOM   5965 CB ALA A 790   9.669 -11.983   5.215 1.00 70.14
C
ANISOU 5965 CB ALA A 790   10486   6330   9834   -650   843
1505   C
ATOM   5966 N   PHE A 791   12.201 -10.591   3.952 1.00 69.70
N
ANISOU 5966 N   PHE A 791   10034   6857   9592   -824   396
1325   N
ATOM   5967 CA PHE A 791   12.907 -9.539   3.222 1.00 71.98
C
ANISOU 5967 CA PHE A 791   10182   7449   9719   -944   336
1288   C
ATOM   5968 C   PHE A 791   13.665 -10.065   2.004 1.00 96.96
C
ANISOU 5968 C   PHE A 791   12810 10957 13073   -978   249
1010   C
ATOM   5969 O   PHE A 791   13.494 -9.559   0.889 1.00 95.88
O
ANISOU 5969 O   PHE A 791   12430 11144 12854 -1045   368
933   O
ATOM   5970 CB PHE A 791   13.860 -8.817   4.182 1.00 71.87
C
ANISOU 5970 CB PHE A 791   10500   7278   9529 -1011   125

```
1421       C
ATOM   5971  CG  PHE A 791      14.774  -7.817   3.518  1.00 88.57
C
ANISOU 5971  CG  PHE A 791    12461   9669  11522  -1159     40
1371       C
ATOM   5972  CD1 PHE A 791      14.420  -6.479   3.447  1.00 74.42
C
ANISOU 5972  CD1 PHE A 791    10888   7925   9463  -1250    160
1534       C
ATOM   5973  CD2 PHE A 791      16.007  -8.205   3.009  1.00104.26
C
ANISOU 5973  CD2 PHE A 791    14092  11847  13674  -1216   -157
1157       C
ATOM   5974  CE1 PHE A 791      15.255  -5.551   2.850  1.00 82.18
C
ANISOU 5974  CE1 PHE A 791    11751   9135  10337  -1407     98
1507       C
ATOM   5975  CE2 PHE A 791      16.848  -7.283   2.410  1.00120.12
C
ANISOU 5975  CE2 PHE A 791    15959  14106  15575  -1380   -200
1108       C
ATOM   5976  CZ  PHE A 791      16.472  -5.953   2.332  1.00102.24
C
ANISOU 5976  CZ  PHE A 791    13935  11878  13035  -1483    -69
1297       C
ATOM   5977  N   LEU A 792      14.511 -11.079   2.197  1.00126.75
N
ANISOU 5977  N   LEU A 792    16401  14663  17094   -930     36
851       N
ATOM   5978  CA  LEU A 792      15.345 -11.581   1.103  1.00112.13
C
ANISOU 5978  CA  LEU A 792    14028  13137  15438   -964    -55
549       C
ATOM   5979  C   LEU A 792      14.534 -12.181  -0.045  1.00 74.02
C
ANISOU 5979  C   LEU A 792     8831   8550  10746   -916    140
367       C
ATOM   5980  O   LEU A 792      14.798 -11.890  -1.217  1.00142.39
O
ANISOU 5980  O   LEU A 792    17154  17590  19356   -998    202
202       O
ATOM   5981  CB  LEU A 792      16.349 -12.602   1.642  1.00 99.26
C
ANISOU 5981  CB  LEU A 792    12289  11336  14089   -892   -345
402       C
ATOM   5982  CG  LEU A 792      17.419 -12.022   2.572  1.00 97.98
C
ANISOU 5982  CG  LEU A 792    12388  11017  13824   -945   -602
501       C
ATOM   5983  CD1 LEU A 792      18.203 -13.130   3.261  1.00118.81
C
ANISOU 5983  CD1 LEU A 792    14989  13405  16750   -826   -917
386       C
ATOM   5984  CD2 LEU A 792      18.348 -11.083   1.817  1.00100.10
```

```
C
ANISOU 5984  CD2 LEU A 792    12422  11628  13982  -1116   -626
388       C
ATOM   5985  N   ILE A 793      13.547 -13.022   0.266  1.00 70.68
N
ANISOU 5985  N   ILE A 793     8464   7907  10486   -791    241
386       N
ATOM   5986  CA  ILE A 793      12.702 -13.601  -0.780  1.00 70.27
C
ANISOU 5986  CA  ILE A 793     8061   8059  10579   -734    415
197       C
ATOM   5987  C   ILE A 793      11.922 -12.527  -1.533  1.00 69.06
C
ANISOU 5987  C   ILE A 793     7932   8156  10150   -794    623
279       C
ATOM   5988  O   ILE A 793      11.861 -12.537  -2.768  1.00 93.96
O
ANISOU 5988  O   ILE A 793    10729  11670  13299   -817    686
85       O
ATOM   5989  CB  ILE A 793      11.764 -14.666  -0.183  1.00100.31
C
ANISOU 5989  CB  ILE A 793    11952  11531  14631   -605    495
218       C
ATOM   5990  CG1 ILE A 793      12.576 -15.845   0.358  1.00123.34
C
ANISOU 5990  CG1 ILE A 793    14788  14221  17854   -536    259
106       C
ATOM   5991  CG2 ILE A 793      10.758 -15.143  -1.220  1.00 70.10
C
ANISOU 5991  CG2 ILE A 793     7785   7901  10949   -548    686
26       C
ATOM   5992  CD1 ILE A 793      13.342 -16.603  -0.707  1.00 80.96
C
ANISOU 5992  CD1 ILE A 793     8868   9144  12749   -519    135
-261       C
ATOM   5993  N   ALA A 794      11.312 -11.587  -0.808  1.00 68.24
N
ANISOU 5993  N   ALA A 794     8257   7862   9809   -813    720
560       N
ATOM   5994  CA  ALA A 794      10.584 -10.509  -1.474  1.00 96.69
C
ANISOU 5994  CA  ALA A 794    11910  11665  13161   -855    882
650       C
ATOM   5995  C   ALA A 794      11.491  -9.636  -2.335  1.00 73.60
C
ANISOU 5995  C   ALA A 794     8847   9090  10026   -998    815
616       C
ATOM   5996  O   ALA A 794      11.129  -9.284  -3.463  1.00 97.18
O
ANISOU 5996  O   ALA A 794    11633  12386  12904  -1023    908
540       O
ATOM   5997  CB  ALA A 794       9.853  -9.652  -0.440  1.00104.11
C
ANISOU 5997  CB  ALA A 794    13339  12309  13911   -845    981
```

```
938       C
ATOM   5998  N   GLN A 795      12.668  -9.272  -1.827  1.00 68.18
N
ANISOU 5998  N   GLN A 795     8272   8357   9277  -1100    653
669       N
ATOM   5999  CA  GLN A 795      13.585  -8.437  -2.598  1.00 81.72
C
ANISOU 5999  CA  GLN A 795     9856  10386  10810  -1267    612
635       C
ATOM   6000  C   GLN A 795      14.366  -9.181  -3.676  1.00107.46
C
ANISOU 6000  C   GLN A 795    12609  13995  14224  -1310    568
315       C
ATOM   6001  O   GLN A 795      14.799  -8.548  -4.645  1.00 90.11
O
ANISOU 6001  O   GLN A 795    10251  12135  11850  -1450    621
263       O
ATOM   6002  CB  GLN A 795      14.550  -7.713  -1.659  1.00 69.23
C
ANISOU 6002  CB  GLN A 795     8558   8623   9122  -1371    460
786       C
ATOM   6003  CG  GLN A 795      13.828  -6.765  -0.719  1.00 90.87
C
ANISOU 6003  CG  GLN A 795    11798  11072  11655  -1354    520
1088       C
ATOM   6004  CD  GLN A 795      12.680  -6.046  -1.411  1.00 84.52
C
ANISOU 6004  CD  GLN A 795    11055  10388  10673  -1341    719
1192       C
ATOM   6005  OE1 GLN A 795      12.895  -5.248  -2.320  1.00 67.85
O
ANISOU 6005  OE1 GLN A 795     8855   8543   8384  -1459    760
1208       O
ATOM   6006  NE2 GLN A 795      11.453  -6.340  -0.992  1.00 66.75
N
ANISOU 6006  NE2 GLN A 795     8952   7933   8475  -1197    842
1259       N
ATOM   6007  N   LEU A 796      14.570 -10.491  -3.542  1.00134.32
N
ANISOU 6007  N   LEU A 796    15764  17324  17949  -1201    478
97       N
ATOM   6008  CA  LEU A 796      15.024 -11.275  -4.688  1.00 85.94
C
ANISOU 6008  CA  LEU A 796     9124  11544  11987  -1210    476
-247       C
ATOM   6009  C   LEU A 796      14.010 -11.244  -5.826  1.00 75.09
C
ANISOU 6009  C   LEU A 796     7577  10445  10508  -1176    664
-321       C
ATOM   6010  O   LEU A 796      14.369 -11.013  -6.986  1.00114.42
O
ANISOU 6010  O   LEU A 796    12285  15828  15360  -1276    724
-479       O
ATOM   6011  CB  LEU A 796      15.296 -12.718  -4.262  1.00105.84
```

```
C
ANISOU 6011  CB  LEU A 796    11434  13873  14906  -1072    332
-461       C
ATOM   6012  CG  LEU A 796      15.700 -13.687  -5.377  1.00 73.43
C
ANISOU 6012  CG  LEU A 796     6777  10091  11034  -1050    321
-862       C
ATOM   6013  CD1 LEU A 796      16.972 -13.216  -6.069  1.00101.48
C
ANISOU 6013  CD1 LEU A 796    10072  13996  14491  -1224    280
-1033       C
ATOM   6014  CD2 LEU A 796      15.868 -15.098  -4.839  1.00 98.09
C
ANISOU 6014  CD2 LEU A 796     9744  12946  14580   -895    160
-1043       C
ATOM   6015  N   ILE A 797      12.734 -11.470  -5.509  1.00 69.93
N
ANISOU 6015  N   ILE A 797     7084   9584   9903  -1038    760
-217       N
ATOM   6016  CA  ILE A 797      11.670 -11.346  -6.504  1.00 93.10
C
ANISOU 6016  CA  ILE A 797     9891  12746  12738   -987    912
-274       C
ATOM   6017  C   ILE A 797      11.586  -9.929  -7.063  1.00 90.18
C
ANISOU 6017  C   ILE A 797     9707  12587  11969  -1110    984
-79       C
ATOM   6018  O   ILE A 797      11.463  -9.735  -8.279  1.00103.02
O
ANISOU 6018  O   ILE A 797    11118  14583  13442  -1149   1046
-199       O
ATOM   6019  CB  ILE A 797      10.328 -11.795  -5.898  1.00 84.31
C
ANISOU 6019  CB  ILE A 797     8931  11319  11785   -825   1002
-195       C
ATOM   6020  CG1 ILE A 797      10.368 -13.288  -5.569  1.00 94.65
C
ANISOU 6020  CG1 ILE A 797    10011  12450  13503   -713    943
-412       C
ATOM   6021  CG2 ILE A 797       9.174 -11.479  -6.842  1.00 69.97
C
ANISOU 6021  CG2 ILE A 797     7023   9712   9852   -763   1134
-234       C
ATOM   6022  CD1 ILE A 797       9.249 -13.742  -4.659  1.00133.97
C
ANISOU 6022  CD1 ILE A 797    15210  17034  18658   -594   1038
-291       C
ATOM   6023  N   ALA A 798      11.649  -8.920  -6.193  1.00 68.88
N
ANISOU 6023  N   ALA A 798     7424   9653   9092  -1173    971
223       N
ATOM   6024  CA  ALA A 798      11.660  -7.535  -6.661  1.00 69.04
C
ANISOU 6024  CA  ALA A 798     7646   9830   8755  -1302   1020
```

```
424       C
ATOM   6025  C   ALA A 798      12.845  -7.236  -7.572  1.00 70.50
C
ANISOU 6025  C   ALA A 798     7603  10387   8797  -1494    998
308       C
ATOM   6026  O   ALA A 798      12.691  -6.552  -8.590  1.00 71.26
O
ANISOU 6026  O   ALA A 798     7672  10773   8629  -1579   1074
344       O
ATOM   6027  CB  ALA A 798      11.657  -6.582  -5.466  1.00116.39
C
ANISOU 6027  CB  ALA A 798    14112  15481  14632  -1339    990
733       C
ATOM   6028  N   THR A 799      14.037  -7.714  -7.215  1.00 71.20
N
ANISOU 6028  N   THR A 799     7536  10466   9051  -1570    893
168       N
ATOM   6029  CA  THR A 799      15.197  -7.567  -8.093  1.00114.04
C
ANISOU 6029  CA  THR A 799    12678  16260  14392  -1760    898
-4       C
ATOM   6030  C   THR A 799      15.002  -8.258  -9.443  1.00132.29
C
ANISOU 6030  C   THR A 799    14576  18977  16710  -1735    986
-296       C
ATOM   6031  O   THR A 799      15.283  -7.676 -10.497  1.00125.79
O
ANISOU 6031  O   THR A 799    13660  18514  15620  -1888   1083
-319       O
ATOM   6032  CB  THR A 799      16.447  -8.106  -7.396  1.00125.39
C
ANISOU 6032  CB  THR A 799    13977  17583  16082  -1806    745
-153       C
ATOM   6033  OG1 THR A 799      16.625  -7.430  -6.145  1.00 73.61
O
ANISOU 6033  OG1 THR A 799     7823  10658   9487  -1826    647
112       O
ATOM   6034  CG2 THR A 799      17.676  -7.882  -8.262  1.00146.95
C
ANISOU 6034  CG2 THR A 799    16400  20690  18743  -2024    775
-349       C
ATOM   6035  N   LEU A 800      14.520  -9.503  -9.426  1.00 81.05
N
ANISOU 6035  N   LEU A 800     7847  12431  10519  -1549    956
-523       N
ATOM   6036  CA  LEU A 800      14.281 -10.246 -10.665  1.00 80.83
C
ANISOU 6036  CA  LEU A 800     7413  12771  10526  -1503   1024
-838       C
ATOM   6037  C   LEU A 800      13.268  -9.578 -11.592  1.00 80.52
C
ANISOU 6037  C   LEU A 800     7476  12952  10168  -1489   1138
-728       C
ATOM   6038  O   LEU A 800      13.467  -9.545 -12.811  1.00 76.27
```

```
O
ANISOU 6038  O   LEU A 800     6705  12834   9440  -1570   1209
-899       O
ATOM   6039  CB  LEU A 800      13.837 -11.674 -10.343  1.00 74.17
C
ANISOU 6039  CB  LEU A 800     6335  11749  10097  -1295    959
-1075       C
ATOM   6040  CG  LEU A 800      14.888 -12.599  -9.725  1.00 74.85
C
ANISOU 6040  CG  LEU A 800     6212  11688  10538  -1282    814
-1280       C
ATOM   6041  CD1 LEU A 800      14.255 -13.913  -9.293  1.00 74.41
C
ANISOU 6041  CD1 LEU A 800     6022  11370  10881  -1071    752
-1436       C
ATOM   6042  CD2 LEU A 800      16.035 -12.841 -10.695  1.00 76.97
C
ANISOU 6042  CD2 LEU A 800     6058  12376  10811  -1416    821
-1610       C
ATOM   6043  N   ILE A 801      12.169  -9.059 -11.048  1.00 95.53
N
ANISOU 6043  N   ILE A 801     9717  14577  12002  -1381   1151
-461       N
ATOM   6044  CA  ILE A 801      11.240  -8.274 -11.862  1.00 78.80
C
ANISOU 6044  CA  ILE A 801     7734  12631   9574  -1360   1219
-329       C
ATOM   6045  C   ILE A 801      11.914  -7.038 -12.452  1.00 74.78
C
ANISOU 6045  C   ILE A 801     7394  12357   8663  -1587   1261
-142       C
ATOM   6046  O   ILE A 801      11.750  -6.729 -13.637  1.00 79.06
O
ANISOU 6046  O   ILE A 801     7853  13255   8930  -1639   1314
-189       O
ATOM   6047  CB  ILE A 801       9.996  -7.897 -11.039  1.00115.72
C
ANISOU 6047  CB  ILE A 801    12743  16928  14295  -1203   1220
-88       C
ATOM   6048  CG1 ILE A 801       9.252  -9.159 -10.597  1.00121.22
C
ANISOU 6048  CG1 ILE A 801    13252  17422  15386  -1001   1217
-288       C
ATOM   6049  CG2 ILE A 801       9.074  -6.991 -11.842  1.00 72.38
C
ANISOU 6049  CG2 ILE A 801     7410  11590   8500  -1170   1251
55       C
ATOM   6050  CD1 ILE A 801       8.991 -10.137 -11.723  1.00118.11
C
ANISOU 6050  CD1 ILE A 801    12419  17352  15106   -917   1227
-648       C
ATOM   6051  N   ALA A 802      12.678  -6.311 -11.640  1.00 74.48
N
ANISOU 6051  N   ALA A 802     7604  12117   8576  -1730   1235
```

```
72        N
ATOM   6052  CA  ALA A 802      13.367  -5.122 -12.139  1.00 75.94
C
ANISOU 6052  CA  ALA A 802     7955  12488   8412  -1972   1286
257        C
ATOM   6053  C   ALA A 802      14.376  -5.426 -13.248  1.00 91.80
C
ANISOU 6053  C   ALA A 802     9607  14957  10316  -2154   1363
-1        C
ATOM   6054  O   ALA A 802      14.428  -4.711 -14.255  1.00131.25
O
ANISOU 6054  O   ALA A 802    14659  20253  14957  -2298   1451
81        O
ATOM   6055  CB  ALA A 802      14.060  -4.407 -10.979  1.00124.90
C
ANISOU 6055  CB  ALA A 802    14444  18365  14646  -2088   1230
483        C
ATOM   6056  N   VAL A 803      15.185  -6.473 -13.083  1.00112.31
N
ANISOU 6056  N   VAL A 803    11844  17614  13215  -2153   1333
-315        N
ATOM   6057  CA  VAL A 803      16.137  -6.868 -14.125  1.00145.09
C
ANISOU 6057  CA  VAL A 803    15605  22213  17309  -2315   1422
-623        C
ATOM   6058  C   VAL A 803      15.465  -7.415 -15.389  1.00108.46
C
ANISOU 6058  C   VAL A 803    10723  17948  12539  -2224   1493
-846        C
ATOM   6059  O   VAL A 803      15.902  -7.106 -16.504  1.00114.68
O
ANISOU 6059  O   VAL A 803    11394  19153  13028  -2401   1616
-932        O
ATOM   6060  CB  VAL A 803      17.186  -7.848 -13.560  1.00138.13
C
ANISOU 6060  CB  VAL A 803    14389  21265  16830  -2315   1341
-925        C
ATOM   6061  CG1 VAL A 803      17.945  -7.202 -12.406  1.00 87.32
C
ANISOU 6061  CG1 VAL A 803     8199  14505  10474  -2424   1254
-714        C
ATOM   6062  CG2 VAL A 803      16.557  -9.152 -13.121  1.00148.83
C
ANISOU 6062  CG2 VAL A 803    15557  22423  18570  -2041   1233
-1132        C
ATOM   6063  N   TYR A 804      14.426  -8.248 -15.265  1.00112.14
N
ANISOU 6063  N   TYR A 804    11102  18286  13221  -1963   1424
-961        N
ATOM   6064  CA  TYR A 804      13.990  -9.029 -16.423  1.00110.90
C
ANISOU 6064  CA  TYR A 804    10609  18495  13033  -1870   1464
-1283        C
ATOM   6065  C   TYR A 804      12.601  -8.734 -16.983  1.00117.29
```

```
C
ANISOU 6065  C   TYR A 804    11584  19347  13634  -1712   1452
-1166       C
ATOM   6066  O   TYR A 804      12.257  -9.307 -18.022  1.00131.42
O
ANISOU 6066  O   TYR A 804    13106  21472  15354  -1642   1473
-1440       O
ATOM   6067  CB  TYR A 804      14.045 -10.531 -16.095  1.00108.16
C
ANISOU 6067  CB  TYR A 804     9870  18051  13174  -1695   1389
-1657       C
ATOM   6068  CG  TYR A 804      15.425 -11.071 -15.795  1.00105.81
C
ANISOU 6068  CG  TYR A 804     9292  17786  13125  -1812   1371
-1892       C
ATOM   6069  CD1 TYR A 804      16.542 -10.591 -16.459  1.00101.15
C
ANISOU 6069  CD1 TYR A 804     8578  17547  12306  -2067   1479
-1976       C
ATOM   6070  CD2 TYR A 804      15.604 -12.086 -14.864  1.00124.54
C
ANISOU 6070  CD2 TYR A 804    11513  19834  15974  -1668   1242
-2043       C
ATOM   6071  CE1 TYR A 804      17.804 -11.080 -16.181  1.00120.93
C
ANISOU 6071  CE1 TYR A 804    10791  20080  15076  -2167   1453
-2226       C
ATOM   6072  CE2 TYR A 804      16.860 -12.590 -14.587  1.00127.82
C
ANISOU 6072  CE2 TYR A 804    11661  20263  16643  -1750   1186
-2275       C
ATOM   6073  CZ  TYR A 804      17.957 -12.082 -15.246  1.00133.33
C
ANISOU 6073  CZ  TYR A 804    12211  21317  17133  -1994   1288
-2380       C
ATOM   6074  OH  TYR A 804      19.209 -12.590 -14.983  1.00145.45
O
ANISOU 6074  OH  TYR A 804    13440  22868  18955  -2069   1225
-2650       O
ATOM   6075  N   ALA A 805      11.788  -7.882 -16.363  1.00140.79
N
ANISOU 6075  N   ALA A 805    14970  22007  16517  -1644   1408
-803       N
ATOM   6076  CA  ALA A 805      10.400  -7.730 -16.813  1.00159.65
C
ANISOU 6076  CA  ALA A 805    17467  24397  18796  -1452   1365
-743       C
ATOM   6077  C   ALA A 805      10.297  -6.713 -17.948  1.00126.83
C
ANISOU 6077  C   ALA A 805    13494  20573  14122  -1569   1396
-582       C
ATOM   6078  O   ALA A 805      10.348  -5.500 -17.726  1.00 98.09
O
ANISOU 6078  O   ALA A 805    10241  16806  10223  -1683   1399
```

```
-218       O
ATOM   6079  CB  ALA A 805       9.500  -7.336 -15.647  1.00190.07
C
ANISOU 6079  CB  ALA A 805    21645  27758  22814  -1307   1307
-468       C
ATOM   6080  N   ASN A 806      10.162  -7.217 -19.177  1.00152.71
N
ANISOU 6080  N   ASN A 806    16505  24276  17244  -1542   1413
-858       N
ATOM   6081  CA  ASN A 806       9.799  -6.415 -20.349  1.00187.24
C
ANISOU 6081  CA  ASN A 806    21057  28971  21116  -1593   1410
-732       C
ATOM   6082  C   ASN A 806       8.290  -6.530 -20.549  1.00179.67
C
ANISOU 6082  C   ASN A 806    20148  27915  20205  -1305   1272
-744       C
ATOM   6083  O   ASN A 806       7.813  -7.387 -21.294  1.00190.56
O
ANISOU 6083  O   ASN A 806    21220  29540  21645  -1161   1232
-1079       O
ATOM   6084  CB  ASN A 806      10.555  -6.871 -21.593  1.00195.92
C
ANISOU 6084  CB  ASN A 806    21853  30615  21974  -1739   1512
-1039       C
ATOM   6085  CG  ASN A 806      12.052  -6.663 -21.485  1.00182.53
C
ANISOU 6085  CG  ASN A 806    20093  29043  20217  -2045   1665
-1041       C
ATOM   6086  OD1 ASN A 806      12.520  -5.782 -20.764  1.00182.46
O
ANISOU 6086  OD1 ASN A 806    20377  28782  20168  -2195   1691
-718       O
ATOM   6087  ND2 ASN A 806      12.814  -7.467 -22.219  1.00163.26
N
ANISOU 6087  ND2 ASN A 806    17251  26997  17784  -2138   1768
-1432       N
ATOM   6088  N   TRP A 807       7.525  -5.665 -19.884  1.00162.17
N
ANISOU 6088  N   TRP A 807    18300  25335  17981  -1216   1194
-405       N
ATOM   6089  CA  TRP A 807       6.066  -5.710 -19.972  1.00159.15
C
ANISOU 6089  CA  TRP A 807    17959  24820  17693   -938   1060
-422       C
ATOM   6090  C   TRP A 807       5.518  -4.351 -20.386  1.00150.35
C
ANISOU 6090  C   TRP A 807    17268  23685  16172   -937    961
-63       C
ATOM   6091  O   TRP A 807       5.520  -3.408 -19.588  1.00146.79
O
ANISOU 6091  O   TRP A 807    17169  22903  15703   -990    957
280       O
ATOM   6092  CB  TRP A 807       5.426  -6.142 -18.652  1.00181.27
```

```
C
ANISOU 6092  CB  TRP A 807    20750  27140  20985   -769   1051
-420       C
ATOM   6093  CG  TRP A 807       5.866  -7.466 -18.119  1.00195.29
C
ANISOU 6093  CG  TRP A 807    22160  28849  23192   -749   1120
-730       C
ATOM   6094  CD1 TRP A 807       6.248  -8.561 -18.838  1.00197.53
C
ANISOU 6094  CD1 TRP A 807    22023  29452  23576   -744   1143
-1123       C
ATOM   6095  CD2 TRP A 807       5.906  -7.856 -16.742  1.00172.84
C
ANISOU 6095  CD2 TRP A 807    19351  25573  20747   -714   1162
-678       C
ATOM   6096  NE1 TRP A 807       6.557  -9.598 -17.990  1.00176.59
N
ANISOU 6096  NE1 TRP A 807    19141  26573  21382   -709   1183
-1308       N
ATOM   6097  CE2 TRP A 807       6.351  -9.191 -16.698  1.00163.75
C
ANISOU 6097  CE2 TRP A 807    17802  24485  19929   -692   1194
-1029       C
ATOM   6098  CE3 TRP A 807       5.621  -7.199 -15.541  1.00156.15
C
ANISOU 6098  CE3 TRP A 807    17580  23022  18727   -701   1171
-372       C
ATOM   6099  CZ2 TRP A 807       6.518  -9.882 -15.501  1.00161.01
C
ANISOU 6099  CZ2 TRP A 807    17416  23769  19992   -658   1223
-1053       C
ATOM   6100  CZ3 TRP A 807       5.787  -7.886 -14.354  1.00161.07
C
ANISOU 6100  CZ3 TRP A 807    18164  23306  19731   -674   1217
-408       C
ATOM   6101  CH2 TRP A 807       6.231  -9.214 -14.342  1.00167.84
C
ANISOU 6101  CH2 TRP A 807    18645  24223  20902   -653   1237
-732       C
ATOM   6102  N   GLU A 808       5.055  -4.254 -21.635  1.00158.27
N
ANISOU 6102  N   GLU A 808    18249  25037  16849   -870    866
-147       N
ATOM   6103  CA  GLU A 808       4.476  -3.006 -22.124  1.00167.82
C
ANISOU 6103  CA  GLU A 808    19869  26229  17666   -842    731
188       C
ATOM   6104  C   GLU A 808       3.197  -2.667 -21.368  1.00185.35
C
ANISOU 6104  C   GLU A 808    22242  28020  20162   -581    591
305       C
ATOM   6105  O   GLU A 808       2.894  -1.492 -21.132  1.00196.68
O
ANISOU 6105  O   GLU A 808    24075  29226  21428   -583    508
```

```
661       O
ATOM   6106  CB  GLU A 808       4.208  -3.097 -23.625  1.00136.76
C
ANISOU 6106  CB  GLU A 808    15868  22766  13326   -799    632
40       C
ATOM   6107  CG  GLU A 808       5.429  -3.462 -24.445  1.00127.18
C
ANISOU 6107  CG  GLU A 808    14484  22017  11823  -1061    798
-118       C
ATOM   6108  CD  GLU A 808       6.682  -2.750 -23.967  1.00120.95
C
ANISOU 6108  CD  GLU A 808    13906  21149  10900  -1390    979
163       C
ATOM   6109  OE1 GLU A 808       7.636  -3.441 -23.554  1.00113.67
O
ANISOU 6109  OE1 GLU A 808    12705  20272  10213  -1532   1143
-37       O
ATOM   6110  OE2 GLU A 808       6.712  -1.501 -24.003  1.00113.42
O
ANISOU 6110  OE2 GLU A 808    13391  20075   9629  -1503    944
573       O
ATOM   6111  N   PHE A 809       2.426  -3.693 -20.999  1.00169.40
N
ANISOU 6111  N   PHE A 809    19897  25883  18583   -357    570
-7       N
ATOM   6112  CA  PHE A 809       1.229  -3.502 -20.184  1.00148.86
C
ANISOU 6112  CA  PHE A 809    17382  22868  16310   -123    486
50       C
ATOM   6113  C   PHE A 809       1.555  -2.780 -18.880  1.00131.56
C
ANISOU 6113  C   PHE A 809    15498  20250  14241   -228    579
369       C
ATOM   6114  O   PHE A 809       0.855  -1.839 -18.488  1.00134.49
O
ANISOU 6114  O   PHE A 809    16169  20338  14593   -132    489
611       O
ATOM   6115  CB  PHE A 809       0.580  -4.867 -19.918  1.00160.63
C
ANISOU 6115  CB  PHE A 809    18436  24306  18291     66    516
-359       C
ATOM   6116  CG  PHE A 809      -0.178  -4.956 -18.618  1.00163.49
C
ANISOU 6116  CG  PHE A 809    18834  24182  19103    197    569
-320       C
ATOM   6117  CD1 PHE A 809      -1.201  -4.066 -18.318  1.00160.54
C
ANISOU 6117  CD1 PHE A 809    18711  23540  18745    353    462
-135       C
ATOM   6118  CD2 PHE A 809       0.143  -5.937 -17.694  1.00170.30
C
ANISOU 6118  CD2 PHE A 809    19485  24852  20370    161    729
-475       C
ATOM   6119  CE1 PHE A 809      -1.881  -4.154 -17.121  1.00184.78
```

```
C
ANISOU 6119  CE1 PHE A 809    21807  26183  22217    456    546
-121       C
ATOM   6120  CE2 PHE A 809      -0.538  -6.031 -16.498  1.00189.08
C
ANISOU 6120  CE2 PHE A 809    21922  26795  23125    259    805
-431       C
ATOM   6121  CZ  PHE A 809      -1.551  -5.140 -16.210  1.00196.99
C
ANISOU 6121  CZ  PHE A 809    23162  27556  24130    400    731
-262       C
ATOM   6122  N   ALA A 810       2.625  -3.187 -18.206  1.00120.26
N
ANISOU 6122  N   ALA A 810    13995  18764  12933   -419    743
361       N
ATOM   6123  CA  ALA A 810       2.990  -2.598 -16.926  1.00128.28
C
ANISOU 6123  CA  ALA A 810    15285  19384  14072   -517    822
628       C
ATOM   6124  C   ALA A 810       3.966  -1.439 -17.073  1.00130.75
C
ANISOU 6124  C   ALA A 810    15937  19748  13995   -771    838
963       C
ATOM   6125  O   ALA A 810       4.487  -0.950 -16.065  1.00141.29
O
ANISOU 6125  O   ALA A 810    17485  20794  15404   -890    902
1167       O
ATOM   6126  CB  ALA A 810       3.582  -3.667 -16.004  1.00151.31
C
ANISOU 6126  CB  ALA A 810    17963  22165  17363   -567    959
443       C
ATOM   6127  N   LYS A 811       4.229  -1.004 -18.305  1.00135.06
N
ANISOU 6127  N   LYS A 811    16539  20652  14124   -865    784
1018       N
ATOM   6128  CA  LYS A 811       5.081   0.149 -18.595  1.00130.45
C
ANISOU 6128  CA  LYS A 811    16293  20130  13143  -1123    807
1349       C
ATOM   6129  C   LYS A 811       6.500  -0.034 -18.070  1.00118.78
C
ANISOU 6129  C   LYS A 811    14742  18671  11716  -1399    977
1348       C
ATOM   6130  O   LYS A 811       7.157   0.936 -17.686  1.00124.45
O
ANISOU 6130  O   LYS A 811    15758  19238  12289  -1600   1014
1639       O
ATOM   6131  CB  LYS A 811       4.480   1.445 -18.042  1.00131.45
C
ANISOU 6131  CB  LYS A 811    16861  19872  13210  -1065    700
1714       C
ATOM   6132  CG  LYS A 811       3.122   1.804 -18.614  1.00134.71
C
ANISOU 6132  CG  LYS A 811    17375  20256  13552   -795    497
```

```
1738       C
ATOM   6133  CD  LYS A 811       2.665   3.162 -18.109  1.00135.96
C
ANISOU 6133  CD  LYS A 811    17977  20036  13646   -759    386
2101       C
ATOM   6134  CE  LYS A 811       1.247   3.469 -18.556  1.00151.62
C
ANISOU 6134  CE  LYS A 811    20027  21941  15639   -451    159
2087       C
ATOM   6135  NZ  LYS A 811       1.150   3.602 -20.036  1.00173.10
N
ANISOU 6135  NZ  LYS A 811    22776  25054  17942   -444     16
2093       N
ATOM   6136  N   ILE A 812       6.991  -1.270 -18.044  1.00111.20
N
ANISOU 6136  N   ILE A 812    13379  17883  10988  -1408   1067
1007       N
ATOM   6137  CA  ILE A 812       8.234  -1.595 -17.359  1.00129.13
C
ANISOU 6137  CA  ILE A 812    15536  20109  13420  -1612   1192
954       C
ATOM   6138  C   ILE A 812       9.124  -2.376 -18.319  1.00112.80
C
ANISOU 6138  C   ILE A 812    13105  18508  11243  -1757   1290
657       C
ATOM   6139  O   ILE A 812       8.650  -3.251 -19.054  1.00106.77
O
ANISOU 6139  O   ILE A 812    12047  18002  10519  -1613   1264
360       O
ATOM   6140  CB  ILE A 812       7.980  -2.369 -16.042  1.00120.64
C
ANISOU 6140  CB  ILE A 812    14344  18663  12829  -1460   1192
836       C
ATOM   6141  CG1 ILE A 812       9.190  -2.269 -15.103  1.00130.23
C
ANISOU 6141  CG1 ILE A 812    15609  19709  14165  -1661   1261
909       C
ATOM   6142  CG2 ILE A 812       7.569  -3.819 -16.301  1.00111.97
C
ANISOU 6142  CG2 ILE A 812    12814  17707  12024  -1280   1193
436       C
ATOM   6143  CD1 ILE A 812      10.183  -3.405 -15.231  1.00126.56
C
ANISOU 6143  CD1 ILE A 812    14734  19466  13888  -1748   1327
578       C
ATOM   6144  N   ARG A 813      10.406  -2.021 -18.348  1.00 87.25
N
ANISOU 6144  N   ARG A 813     9887  15393   7871  -2047   1404
720       N
ATOM   6145  CA  ARG A 813      11.386  -2.689 -19.193  1.00 93.17
C
ANISOU 6145  CA  ARG A 813    10286  16584   8529  -2219   1528
425       C
ATOM   6146  C   ARG A 813      12.575  -3.097 -18.335  1.00 98.09
```

```
C
ANISOU 6146  C   ARG A 813    10726  17090   9453  -2368   1602
307       C
ATOM   6147  O   ARG A 813      13.069  -2.297 -17.534  1.00 90.52
O
ANISOU 6147  O   ARG A 813    10027  15859   8509  -2506   1608
567       O
ATOM   6148  CB  ARG A 813      11.824  -1.792 -20.359  1.00142.63
C
ANISOU 6148  CB  ARG A 813    16733  23202  14258  -2464   1615
593       C
ATOM   6149  CG  ARG A 813      12.626  -2.522 -21.436  1.00141.89
C
ANISOU 6149  CG  ARG A 813    16264  23634  14014  -2616   1761
245       C
ATOM   6150  CD  ARG A 813      12.859  -1.656 -22.683  1.00 97.46
C
ANISOU 6150  CD  ARG A 813    10861  18374   7797  -2841   1856
424       C
ATOM   6151  NE  ARG A 813      11.667  -0.978 -23.196  1.00125.74
N
ANISOU 6151  NE  ARG A 813    14792  21911  11073  -2675   1698
682       N
ATOM   6152  CZ  ARG A 813      10.484  -1.537 -23.458  1.00125.77
C
ANISOU 6152  CZ  ARG A 813    14705  21924  11160  -2355   1532
531       C
ATOM   6153  NH1 ARG A 813       9.437  -0.765 -23.712  1.00 98.38
N
ANISOU 6153  NH1 ARG A 813    11586  18323   7470  -2201   1359
804       N
ATOM   6154  NH2 ARG A 813      10.316  -2.852 -23.395  1.00 96.31
N
ANISOU 6154  NH2 ARG A 813    10530  18289   7776  -2178   1520
105       N
ATOM   6155  N   GLY A 814      13.026  -4.338 -18.503  1.00 89.33
N
ANISOU 6155  N   GLY A 814     9168  16176   8598  -2330   1639
-99       N
ATOM   6156  CA  GLY A 814      14.107  -4.890 -17.707  1.00 97.90
C
ANISOU 6156  CA  GLY A 814    10031  17146  10020  -2424   1666
-265       C
ATOM   6157  C   GLY A 814      15.405  -4.108 -17.756  1.00119.93
C
ANISOU 6157  C   GLY A 814    12893  20044  12633  -2759   1782
-153       C
ATOM   6158  O   GLY A 814      15.943  -3.846 -18.837  1.00132.05
O
ANISOU 6158  O   GLY A 814    14340  21986  13847  -2969   1923
-220       O
ATOM   6159  N   ILE A 815      15.920  -3.729 -16.588  1.00 85.92
N
ANISOU 6159  N   ILE A 815     8743  15374   8529  -2820   1730
```

```
8       N
ATOM   6160  CA  ILE A 815      17.126  -2.913 -16.475  1.00112.51
C
ANISOU 6160  CA  ILE A 815    12192  18774  11782  -3136   1822
126       C
ATOM   6161  C   ILE A 815      18.168  -3.676 -15.658  1.00127.25
C
ANISOU 6161  C   ILE A 815    13755  20528  14066  -3162   1774
-138       C
ATOM   6162  O   ILE A 815      17.910  -4.067 -14.513  1.00149.66
O
ANISOU 6162  O   ILE A 815    16656  22982  17224  -2975   1621
-115       O
ATOM   6163  CB  ILE A 815      16.825  -1.530 -15.871  1.00136.27
C
ANISOU 6163  CB  ILE A 815    15719  21446  14610  -3208   1779
589       C
ATOM   6164  CG1 ILE A 815      18.110  -0.713 -15.730  1.00131.78
C
ANISOU 6164  CG1 ILE A 815    15209  20894  13966  -3549   1875
687       C
ATOM   6165  CG2 ILE A 815      16.063  -1.648 -14.548  1.00103.30
C
ANISOU 6165  CG2 ILE A 815    11749  16778  10722  -2952   1610
719       C
ATOM   6166  CD1 ILE A 815      17.873   0.781 -15.749  1.00130.02
C
ANISOU 6166  CD1 ILE A 815    15471  20508  13421  -3700   1896
1128       C
ATOM   6167  N   GLY A 816      19.329  -3.911 -16.269  1.00 89.48
N
ANISOU 6167  N   GLY A 816     8637  16084   9277  -3389   1903
-403       N
ATOM   6168  CA  GLY A 816      20.319  -4.890 -15.852  1.00110.10
C
ANISOU 6168  CA  GLY A 816    10828  18710  12296  -3388   1856
-779       C
ATOM   6169  C   GLY A 816      20.737  -4.999 -14.395  1.00144.18
C
ANISOU 6169  C   GLY A 816    15221  22575  16985  -3310   1666
-729       C
ATOM   6170  O   GLY A 816      20.573  -4.069 -13.599  1.00161.11
O
ANISOU 6170  O   GLY A 816    17767  24393  19055  -3343   1601
-381       O
ATOM   6171  N   TRP A 817      21.298  -6.167 -14.061  1.00135.54
N
ANISOU 6171  N   TRP A 817    13737  21468  16296  -3199   1564
-1095       N
ATOM   6172  CA  TRP A 817      21.768  -6.460 -12.708  1.00126.10
C
ANISOU 6172  CA  TRP A 817    12577  19862  15471  -3102   1350
-1098       C
ATOM   6173  C   TRP A 817      22.803  -5.455 -12.215  1.00157.84
```

```
C
ANISOU 6173  C   TRP A 817    16736  23784  19451  -3359   1348
-958       C
ATOM   6174  O   TRP A 817      22.788  -5.068 -11.040  1.00190.46
O
ANISOU 6174  O   TRP A 817    21168  27511  23688  -3298   1183
-740       O
ATOM   6175  CB  TRP A 817      22.366  -7.867 -12.666  1.00114.40
C
ANISOU 6175  CB  TRP A 817    10603  18447  14416  -2975   1244
-1556       C
ATOM   6176  CG  TRP A 817      21.373  -8.976 -12.660  1.00113.10
C
ANISOU 6176  CG  TRP A 817    10343  18194  14435  -2676   1164
-1678       C
ATOM   6177  CD1 TRP A 817      20.882  -9.648 -13.740  1.00 86.30
C
ANISOU 6177  CD1 TRP A 817     6671  15132  10987  -2605   1274
-1922       C
ATOM   6178  CD2 TRP A 817      20.801  -9.598 -11.504  1.00152.86
C
ANISOU 6178  CD2 TRP A 817    15543  22775  19761  -2416    958
-1589       C
ATOM   6179  NE1 TRP A 817      20.007 -10.625 -13.329  1.00 84.93
N
ANISOU 6179  NE1 TRP A 817     6471  14723  11076  -2318   1149
-1987       N
ATOM   6180  CE2 TRP A 817      19.944 -10.618 -11.961  1.00152.55
C
ANISOU 6180  CE2 TRP A 817    15311  22802  19851  -2207    967
-1776       C
ATOM   6181  CE3 TRP A 817      20.918  -9.381 -10.128  1.00131.68
C
ANISOU 6181  CE3 TRP A 817    13170  19636  17227  -2348    773
-1367       C
ATOM   6182  CZ2 TRP A 817      19.207 -11.419 -11.089  1.00132.56
C
ANISOU 6182  CZ2 TRP A 817    12880  19884  17602  -1951    819
-1736       C
ATOM   6183  CZ3 TRP A 817      20.187 -10.178  -9.264  1.00117.30
C
ANISOU 6183  CZ3 TRP A 817    11472  17447  15650  -2090    626
-1319       C
ATOM   6184  CH2 TRP A 817      19.342 -11.183  -9.748  1.00108.97
C
ANISOU 6184  CH2 TRP A 817    10217  16456  14731  -1903    660
-1496       C
ATOM   6185  N   GLY A 818      23.713  -5.027 -13.091  1.00172.17
N
ANISOU 6185  N   GLY A 818    18335  25967  21116  -3655   1538
-1094       N
ATOM   6186  CA  GLY A 818      24.684  -4.015 -12.700  1.00171.25
C
ANISOU 6186  CA  GLY A 818    18336  25767  20966  -3929   1561
```

```
-967       C
ATOM   6187  C   GLY A 818      24.049  -2.708 -12.269  1.00178.10
C
ANISOU 6187  C   GLY A 818    19768  26362  21538  -3987   1563
-473       C
ATOM   6188  O   GLY A 818      24.430  -2.125 -11.251  1.00198.66
O
ANISOU 6188  O   GLY A 818    22593  28635  24255  -4028   1427
-315       O
ATOM   6189  N   TRP A 819      23.074  -2.226 -13.040  1.00183.28
N
ANISOU 6189  N   TRP A 819    20664  27150  21822  -3982   1698
-238       N
ATOM   6190  CA  TRP A 819      22.303  -1.061 -12.616  1.00170.87
C
ANISOU 6190  CA  TRP A 819    19633  25286  20004  -3980   1669
220       C
ATOM   6191  C   TRP A 819      21.528  -1.342 -11.331  1.00150.07
C
ANISOU 6191  C   TRP A 819    17247  22190  17584  -3675   1439
352       C
ATOM   6192  O   TRP A 819      21.512  -0.519 -10.408  1.00174.65
O
ANISOU 6192  O   TRP A 819    20710  24955  20693  -3697   1342
611       O
ATOM   6193  CB  TRP A 819      21.357  -0.644 -13.744  1.00145.58
C
ANISOU 6193  CB  TRP A 819    16602  22323  16391  -3988   1820
403       C
ATOM   6194  CG  TRP A 819      20.542   0.576 -13.455  1.00146.54
C
ANISOU 6194  CG  TRP A 819    17258  22164  16256  -3982   1788
857       C
ATOM   6195  CD1 TRP A 819      19.387   0.637 -12.734  1.00158.67
C
ANISOU 6195  CD1 TRP A 819    19095  23357  17835  -3705   1640
1058       C
ATOM   6196  CD2 TRP A 819      20.808   1.911 -13.902  1.00147.50
C
ANISOU 6196  CD2 TRP A 819    17671  22319  16055  -4269   1912
1155       C
ATOM   6197  NE1 TRP A 819      18.922   1.929 -12.693  1.00172.88
N
ANISOU 6197  NE1 TRP A 819    21341  24973  19373  -3784   1649
1443       N
ATOM   6198  CE2 TRP A 819      19.776   2.730 -13.403  1.00151.77
C
ANISOU 6198  CE2 TRP A 819    18682  22512  16471  -4128   1804
1523       C
ATOM   6199  CE3 TRP A 819      21.821   2.493 -14.669  1.00161.16
C
ANISOU 6199  CE3 TRP A 819    19309  24327  17598  -4642   2114
1141       C
ATOM   6200  CZ2 TRP A 819      19.728   4.100 -13.648  1.00150.85
```

```
C
ANISOU 6200  CZ2 TRP A 819    18951  22300  16064  -4331   1863
1881       C
ATOM   6201  CZ3 TRP A 819      21.771   3.852 -14.910  1.00173.32
C
ANISOU 6201  CZ3 TRP A 819    21248  25772  18835  -4863   2192
1518       C
ATOM   6202  CH2 TRP A 819      20.732   4.641 -14.402  1.00150.69
C
ANISOU 6202  CH2 TRP A 819    18853  22539  15862  -4699   2053
1886       C
ATOM   6203  N   ALA A 820      20.871  -2.503 -11.260  1.00126.11
N
ANISOU 6203  N   ALA A 820    14037  19144  14735  -3396   1361
168       N
ATOM   6204  CA  ALA A 820      20.138  -2.896 -10.057  1.00111.81
C
ANISOU 6204  CA  ALA A 820    12442  16907  13133  -3117   1174
270       C
ATOM   6205  C   ALA A 820      21.036  -2.977  -8.825  1.00 84.35
C
ANISOU 6205  C   ALA A 820     8986  13129   9935  -3129    991
224       C
ATOM   6206  O   ALA A 820      20.624  -2.599  -7.722  1.00 80.64
O
ANISOU 6206  O   ALA A 820     8878  12268   9492  -3021    865
452       O
ATOM   6207  CB  ALA A 820      19.439  -4.235 -10.294  1.00119.35
C
ANISOU 6207  CB  ALA A 820    13137  17932  14279  -2857   1145
31       C
ATOM   6208  N   GLY A 821      22.261  -3.476  -8.991  1.00 83.40
N
ANISOU 6208  N   GLY A 821     8476  13187  10025  -3253    965
-89       N
ATOM   6209  CA  GLY A 821      23.239  -3.426  -7.912  1.00 83.87
C
ANISOU 6209  CA  GLY A 821     8543  12988  10334  -3290    769
-146       C
ATOM   6210  C   GLY A 821      23.512  -2.032  -7.374  1.00 84.24
C
ANISOU 6210  C   GLY A 821     8967  12834  10205  -3478    761
153       C
ATOM   6211  O   GLY A 821      23.668  -1.846  -6.165  1.00119.46
O
ANISOU 6211  O   GLY A 821    13666  16932  14792  -3399    560
254       O
ATOM   6212  N   VAL A 822      23.581  -1.037  -8.259  1.00 85.69
N
ANISOU 6212  N   VAL A 822     9224  13243  10092  -3730    972
299       N
ATOM   6213  CA  VAL A 822      23.663   0.355  -7.818  1.00 92.91
C
ANISOU 6213  CA  VAL A 822    10537  13940  10824  -3902    977
```

```
619       C
ATOM   6214  C   VAL A 822      22.415   0.775  -7.048  1.00 88.80
C
ANISOU 6214  C   VAL A 822    10501  13056  10182  -3684    891
948       C
ATOM   6215  O   VAL A 822      22.505   1.478  -6.034  1.00124.92
O
ANISOU 6215  O   VAL A 822    15394  17293  14776  -3690    764
1126       O
ATOM   6216  CB  VAL A 822      23.918   1.278  -9.025  1.00116.45
C
ANISOU 6216  CB  VAL A 822    13511  17236  13498  -4219   1235
725       C
ATOM   6217  CG1 VAL A 822      24.195   2.699  -8.558  1.00118.13
C
ANISOU 6217  CG1 VAL A 822    14093  17206  13584  -4430   1230
1021       C
ATOM   6218  CG2 VAL A 822      25.078   0.751  -9.860  1.00103.71
C
ANISOU 6218  CG2 VAL A 822    11381  16025  11999  -4433   1367
358       C
ATOM   6219  N   ILE A 823      21.235   0.366  -7.515  1.00 88.97
N
ANISOU 6219  N   ILE A 823    10575  13145  10085  -3489    962
1012       N
ATOM   6220  CA  ILE A 823      20.007   0.617  -6.760  1.00129.36
C
ANISOU 6220  CA  ILE A 823    16093  17921  15136  -3260    891
1267       C
ATOM   6221  C   ILE A 823      20.049  -0.050  -5.388  1.00125.79
C
ANISOU 6221  C   ILE A 823    15717  17124  14954  -3059    686
1200       C
ATOM   6222  O   ILE A 823      19.655   0.549  -4.380  1.00 96.06
O
ANISOU 6222  O   ILE A 823    12339  13009  11150  -2988    597
1415       O
ATOM   6223  CB  ILE A 823      18.779   0.155  -7.560  1.00122.55
C
ANISOU 6223  CB  ILE A 823    15192  17218  14154  -3082    997
1280       C
ATOM   6224  CG1 ILE A 823      18.808   0.720  -8.975  1.00112.15
C
ANISOU 6224  CG1 ILE A 823    13796  16273  12542  -3277   1179
1327       C
ATOM   6225  CG2 ILE A 823      17.510   0.619  -6.872  1.00122.15
C
ANISOU 6225  CG2 ILE A 823    15556  16831  14024  -2884    957
1547       C
ATOM   6226  CD1 ILE A 823      17.723   0.148  -9.850  1.00106.89
C
ANISOU 6226  CD1 ILE A 823    13029  15811  11772  -3099   1253
1275       C
ATOM   6227  N   TRP A 824      20.515  -1.299  -5.327  1.00125.43
```

```
N
ANISOU 6227  N   TRP A 824    15319  17164  15174  -2961    603
902       N
ATOM   6228  CA  TRP A 824      20.746  -1.957  -4.042  1.00 82.03
C
ANISOU 6228  CA  TRP A 824     9894  11343   9929  -2793    381
835       C
ATOM   6229  C   TRP A 824      21.726  -1.178  -3.169  1.00 87.09
C
ANISOU 6229  C   TRP A 824    10707  11779  10603  -2937    228
894       C
ATOM   6230  O   TRP A 824      21.476  -0.960  -1.978  1.00119.16
O
ANISOU 6230  O   TRP A 824    15120  15482  14675  -2824     82
1044       O
ATOM   6231  CB  TRP A 824      21.273  -3.376  -4.278  1.00 77.92
C
ANISOU 6231  CB  TRP A 824     8924  10974   9710  -2697    302
481       C
ATOM   6232  CG  TRP A 824      20.222  -4.445  -4.372  1.00 76.34
C
ANISOU 6232  CG  TRP A 824     8660  10742   9604  -2446    331
425       C
ATOM   6233  CD1 TRP A 824      19.622  -4.903  -5.509  1.00 97.60
C
ANISOU 6233  CD1 TRP A 824    11106  13733  12245  -2411    497
312       C
ATOM   6234  CD2 TRP A 824      19.676  -5.215  -3.293  1.00133.12
C
ANISOU 6234  CD2 TRP A 824    16031  17581  16967  -2207    192
465       C
ATOM   6235  NE1 TRP A 824      18.724  -5.897  -5.205  1.00107.03
N
ANISOU 6235  NE1 TRP A 824    12295  14774  13597  -2166    470
269       N
ATOM   6236  CE2 TRP A 824      18.738  -6.108  -3.851  1.00132.17
C
ANISOU 6236  CE2 TRP A 824    15745  17556  16918  -2047    297
371       C
ATOM   6237  CE3 TRP A 824      19.881  -5.231  -1.910  1.00136.41
C
ANISOU 6237  CE3 TRP A 824    16749  17612  17470  -2120     -9
572       C
ATOM   6238  CZ2 TRP A 824      18.008  -7.006  -3.074  1.00129.03
C
ANISOU 6238  CZ2 TRP A 824    15464  16867  16693  -1821    230
390       C
ATOM   6239  CZ3 TRP A 824      19.154  -6.124  -1.141  1.00 89.87
C
ANISOU 6239  CZ3 TRP A 824    10999  11440  11709  -1893    -75
605       C
ATOM   6240  CH2 TRP A 824      18.230  -6.999  -1.724  1.00 85.73
C
ANISOU 6240  CH2 TRP A 824    10296  11006  11272  -1755     55
```

```
518       C
ATOM   6241  N   LEU A 825      22.851  -0.753  -3.746  1.00 92.99
N
ANISOU 6241  N   LEU A 825    11207  12757  11369  -3195    266
762       N
ATOM   6242  CA  LEU A 825      23.817   0.070  -3.019  1.00100.09
C
ANISOU 6242  CA  LEU A 825    12235  13481  12313  -3361    130
796       C
ATOM   6243  C   LEU A 825      23.224   1.401  -2.559  1.00 87.20
C
ANISOU 6243  C   LEU A 825    11100  11603  10430  -3418    166
1149       C
ATOM   6244  O   LEU A 825      23.425   1.817  -1.412  1.00139.74
O
ANISOU 6244  O   LEU A 825    18035  17934  17125  -3380    -17
1235       O
ATOM   6245  CB  LEU A 825      25.051   0.296  -3.899  1.00106.30
C
ANISOU 6245  CB  LEU A 825    12623  14598  13167  -3657    227
575       C
ATOM   6246  CG  LEU A 825      26.302   0.984  -3.337  1.00 97.63
C
ANISOU 6246  CG  LEU A 825    11500  13394  12203  -3868     94
499       C
ATOM   6247  CD1 LEU A 825      26.195   2.511  -3.351  1.00 87.74
C
ANISOU 6247  CD1 LEU A 825    10601  12034  10702  -4092    205
802       C
ATOM   6248  CD2 LEU A 825      26.598   0.479  -1.929  1.00109.19
C
ANISOU 6248  CD2 LEU A 825    13081  14507  13900  -3657   -247
422       C
ATOM   6249  N   TYR A 826      22.495   2.087  -3.440  1.00 81.27
N
ANISOU 6249  N   TYR A 826    10468  10993   9417  -3502    384
1347       N
ATOM   6250  CA  TYR A 826      21.819   3.327  -3.059  1.00 91.31
C
ANISOU 6250  CA  TYR A 826    12209  12015  10469  -3528    412
1677       C
ATOM   6251  C   TYR A 826      20.826   3.120  -1.918  1.00109.13
C
ANISOU 6251  C   TYR A 826    14815  13915  12734  -3244    296
1807       C
ATOM   6252  O   TYR A 826      20.763   3.928  -0.985  1.00116.79
O
ANISOU 6252  O   TYR A 826    16144  14578  13654  -3245    199
1967       O
ATOM   6253  CB  TYR A 826      21.124   3.923  -4.284  1.00107.41
C
ANISOU 6253  CB  TYR A 826    14295  14276  12240  -3622    639
1851       C
ATOM   6254  CG  TYR A 826      20.356   5.199  -4.023  1.00112.98
```

```
C
ANISOU 6254  CG  TYR A 826    15466  14729  12734  -3634    663
2186       C
ATOM   6255  CD1 TYR A 826      21.003   6.429  -4.030  1.00 82.72
C
ANISOU 6255  CD1 TYR A 826    11798  10813   8817  -3899    677
2329       C
ATOM   6256  CD2 TYR A 826      18.985   5.180  -3.802  1.00 95.70
C
ANISOU 6256  CD2 TYR A 826    13532  12378  10453  -3386    675
2342       C
ATOM   6257  CE1 TYR A 826      20.313   7.602  -3.782  1.00 82.67
C
ANISOU 6257  CE1 TYR A 826    12216  10550   8645  -3901    680
2624       C
ATOM   6258  CE2 TYR A 826      18.282   6.350  -3.571  1.00105.85
C
ANISOU 6258  CE2 TYR A 826    15222  13424  11572  -3383    685
2621       C
ATOM   6259  CZ  TYR A 826      18.952   7.558  -3.558  1.00 80.70
C
ANISOU 6259  CZ  TYR A 826    12209  10144   8309  -3635    678
2764       C
ATOM   6260  OH  TYR A 826      18.254   8.728  -3.352  1.00 82.64
O
ANISOU 6260  OH  TYR A 826    12853  10133   8412  -3625    675
3032       O
ATOM   6261  N   SER A 827      20.037   2.049  -1.983  1.00112.57
N
ANISOU 6261  N   SER A 827    15150  14387  13234  -3010    320
1731       N
ATOM   6262  CA  SER A 827      19.129   1.698  -0.892  1.00119.22
C
ANISOU 6262  CA  SER A 827    16291  14903  14105  -2754    240
1824       C
ATOM   6263  C   SER A 827      19.859   1.403   0.419  1.00132.69
C
ANISOU 6263  C   SER A 827    18109  16340  15966  -2701      3
1748       C
ATOM   6264  O   SER A 827      19.412   1.826   1.492  1.00117.28
O
ANISOU 6264  O   SER A 827    16552  14068  13942  -2605    -71
1903       O
ATOM   6265  CB  SER A 827      18.270   0.500  -1.298  1.00 88.27
C
ANISOU 6265  CB  SER A 827    12175  11093  10269  -2545    322
1718       C
ATOM   6266  OG  SER A 827      17.578   0.754  -2.509  1.00134.32
O
ANISOU 6266  OG  SER A 827    17907  17180  15948  -2576    511
1772       O
ATOM   6267  N   ILE A 828      20.974   0.672   0.354  1.00113.18
N
ANISOU 6267  N   ILE A 828    15299  13996  13709  -2753   -129
```

```
1498       N
ATOM   6268  CA  ILE A 828      21.787   0.407   1.543  1.00 77.54
C
ANISOU 6268  CA  ILE A 828    10874   9239   9349  -2704   -402
1407       C
ATOM   6269  C   ILE A 828      22.292   1.689   2.205  1.00 78.65
C
ANISOU 6269  C   ILE A 828    11312   9187   9385  -2855   -498
1536       C
ATOM   6270  O   ILE A 828      22.195   1.844   3.428  1.00 85.19
O
ANISOU 6270  O   ILE A 828    12487   9698  10183  -2745   -668
1618       O
ATOM   6271  CB  ILE A 828      22.958  -0.525   1.180  1.00 79.27
C
ANISOU 6271  CB  ILE A 828    10614   9660   9844  -2747   -532
1082       C
ATOM   6272  CG1 ILE A 828      22.452  -1.940   0.896  1.00 78.06
C
ANISOU 6272  CG1 ILE A 828    10230   9585   9844  -2540   -512
942       C
ATOM   6273  CG2 ILE A 828      23.998  -0.538   2.288  1.00112.10
C
ANISOU 6273  CG2 ILE A 828    14844  13592  14159  -2741   -848
978       C
ATOM   6274  CD1 ILE A 828      21.935  -2.666   2.117  1.00 85.81
C
ANISOU 6274  CD1 ILE A 828    11504  10216  10886  -2289   -679
1013       C
ATOM   6275  N   VAL A 829      22.824   2.633   1.429  1.00 79.95
N
ANISOU 6275  N   VAL A 829    11363   9531   9484  -3115   -389
1555       N
ATOM   6276  CA  VAL A 829      23.314   3.854   2.072  1.00 83.30
C
ANISOU 6276  CA  VAL A 829    12062   9747   9840  -3266   -486
1664       C
ATOM   6277  C   VAL A 829      22.192   4.817   2.469  1.00 81.23
C
ANISOU 6277  C   VAL A 829    12278   9249   9339  -3205   -393
1962       C
ATOM   6278  O   VAL A 829      22.365   5.627   3.388  1.00 83.74
O
ANISOU 6278  O   VAL A 829    12913   9297   9609  -3232   -523
2048       O
ATOM   6279  CB  VAL A 829      24.346   4.545   1.164  1.00 89.01
C
ANISOU 6279  CB  VAL A 829    12503  10713  10605  -3593   -397
1579       C
ATOM   6280  CG1 VAL A 829      25.490   3.591   0.846  1.00 84.96
C
ANISOU 6280  CG1 VAL A 829    11492  10425  10362  -3646   -494
1238       C
ATOM   6281  CG2 VAL A 829      23.689   5.041  -0.116  1.00133.64
```

```
C
ANISOU 6281  CG2 VAL A 829    18122  16602  16052  -3714    -99
1738       C
ATOM   6282  N   THR A 830      21.047   4.762   1.792  1.00 92.45
N
ANISOU 6282  N   THR A 830    13743  10763  10620  -3117   -183
2099       N
ATOM   6283  CA  THR A 830      19.844   5.455   2.252  1.00102.83
C
ANISOU 6283  CA  THR A 830    15483  11835  11753  -2994   -109
2339       C
ATOM   6284  C   THR A 830      19.338   4.918   3.591  1.00109.78
C
ANISOU 6284  C   THR A 830    16643  12417  12651  -2752   -233
2343       C
ATOM   6285  O   THR A 830      18.912   5.687   4.462  1.00134.72
O
ANISOU 6285  O   THR A 830    20194  15296  15696  -2706   -271
2480       O
ATOM   6286  CB  THR A 830      18.748   5.349   1.188  1.00105.37
C
ANISOU 6286  CB  THR A 830    15731  12344  11962  -2928    116
2436       C
ATOM   6287  OG1 THR A 830      19.170   6.029   0.000  1.00114.83
O
ANISOU 6287  OG1 THR A 830    16764  13788  13076  -3167    233
2480       O
ATOM   6288  CG2 THR A 830      17.452   5.974   1.681  1.00 93.12
C
ANISOU 6288  CG2 THR A 830    14577  10536  10268  -2773    184
2643       C
ATOM   6289  N   TYR A 831      19.392   3.598   3.767  1.00103.70
N
ANISOU 6289  N   TYR A 831    15681  11700  12021  -2604   -294
2191       N
ATOM   6290  CA  TYR A 831      18.860   2.922   4.952  1.00127.55
C
ANISOU 6290  CA  TYR A 831    18963  14454  15046  -2379   -384
2208       C
ATOM   6291  C   TYR A 831      19.478   3.375   6.278  1.00120.47
C
ANISOU 6291  C   TYR A 831    18389  13269  14114  -2381   -620
2219       C
ATOM   6292  O   TYR A 831      18.749   3.561   7.261  1.00 75.68
O
ANISOU 6292  O   TYR A 831    13114   7332   8310  -2249   -616
2337       O
ATOM   6293  CB  TYR A 831      19.036   1.417   4.749  1.00114.51
C
ANISOU 6293  CB  TYR A 831    16994  12925  13588  -2259   -431
2028       C
ATOM   6294  CG  TYR A 831      19.073   0.564   5.991  1.00111.15
C
ANISOU 6294  CG  TYR A 831    16771  12237  13222  -2080   -614
```

```
1996       C
ATOM   6295  CD1 TYR A 831      20.266   0.001   6.422  1.00123.78
C
ANISOU 6295  CD1 TYR A 831    18230  13813  14989  -2088   -893
1825       C
ATOM   6296  CD2 TYR A 831      17.916   0.280   6.704  1.00102.49
C
ANISOU 6296  CD2 TYR A 831    15999  10921  12023  -1905   -509
2130       C
ATOM   6297  CE1 TYR A 831      20.316  -0.787   7.548  1.00153.30
C
ANISOU 6297  CE1 TYR A 831    22186  17299  18763  -1919  -1089
1815       C
ATOM   6298  CE2 TYR A 831      17.954  -0.520   7.830  1.00117.48
C
ANISOU 6298  CE2 TYR A 831    18116  12576  13946  -1758   -664
2124       C
ATOM   6299  CZ  TYR A 831      19.160  -1.048   8.247  1.00154.69
C
ANISOU 6299  CZ  TYR A 831    22720  17256  18800  -1762   -967
1979       C
ATOM   6300  OH  TYR A 831      19.216  -1.856   9.355  1.00167.12
O
ANISOU 6300  OH  TYR A 831    24541  18575  20381  -1609  -1150
1991       O
ATOM   6301  N   PHE A 832      20.798   3.536   6.353  1.00108.88
N
ANISOU 6301  N   PHE A 832    16757  11847  12763  -2524   -826
2079       N
ATOM   6302  CA  PHE A 832      21.407   3.847   7.649  1.00 88.08
C
ANISOU 6302  CA  PHE A 832    14417   8941  10107  -2500  -1094
2058       C
ATOM   6303  C   PHE A 832      20.905   5.112   8.348  1.00112.03
C
ANISOU 6303  C   PHE A 832    17903  11730  12932  -2525  -1065
2233       C
ATOM   6304  O   PHE A 832      20.685   5.047   9.570  1.00127.57
O
ANISOU 6304  O   PHE A 832    20238  13435  14796  -2387  -1196
2268       O
ATOM   6305  CB  PHE A 832      22.938   3.877   7.533  1.00 81.34
C
ANISOU 6305  CB  PHE A 832    13263   8191   9452  -2662  -1327
1845       C
ATOM   6306  CG  PHE A 832      23.554   2.525   7.336  1.00 88.54
C
ANISOU 6306  CG  PHE A 832    13809   9238  10595  -2576  -1466
1628       C
ATOM   6307  CD1 PHE A 832      23.536   1.597   8.365  1.00100.64
C
ANISOU 6307  CD1 PHE A 832    15518  10561  12160  -2358  -1689
1592       C
ATOM   6308  CD2 PHE A 832      24.171   2.185   6.146  1.00124.76
```

```
C
ANISOU 6308  CD2 PHE A 832    17890  14149  15364  -2713  -1381
1455       C
ATOM   6309  CE1 PHE A 832      24.104   0.349   8.206  1.00106.64
C
ANISOU 6309  CE1 PHE A 832    15950  11409  13157  -2264  -1845
1392       C
ATOM   6310  CE2 PHE A 832      24.745   0.935   5.982  1.00110.14
C
ANISOU 6310  CE2 PHE A 832    15687  12409  13754  -2621  -1521
1225       C
ATOM   6311  CZ  PHE A 832      24.710   0.017   7.014  1.00 98.12
C
ANISOU 6311  CZ  PHE A 832    14339  10651  12290  -2389  -1766
1195       C
ATOM   6312  N   PRO A 833      20.698   6.259   7.691  1.00 99.62
N
ANISOU 6312  N   PRO A 833    16354  10212  11286  -2686   -907
2344       N
ATOM   6313  CA  PRO A 833      20.155   7.413   8.428  1.00104.96
C
ANISOU 6313  CA  PRO A 833    17471  10621  11789  -2679   -894
2492       C
ATOM   6314  C   PRO A 833      18.673   7.325   8.773  1.00115.43
C
ANISOU 6314  C   PRO A 833    19089  11811  12959  -2478   -703
2636       C
ATOM   6315  O   PRO A 833      18.149   8.268   9.378  1.00 79.53
O
ANISOU 6315  O   PRO A 833    14897   7043   8277  -2458   -676
2737       O
ATOM   6316  CB  PRO A 833      20.405   8.584   7.466  1.00 80.55
C
ANISOU 6316  CB  PRO A 833    14272   7634   8699  -2920   -783
2569       C
ATOM   6317  CG  PRO A 833      21.580   8.162   6.672  1.00 88.07
C
ANISOU 6317  CG  PRO A 833    14778   8853   9833  -3096   -842
2406       C
ATOM   6318  CD  PRO A 833      21.353   6.689   6.443  1.00 80.20
C
ANISOU 6318  CD  PRO A 833    13536   8019   8916  -2925   -817
2302       C
ATOM   6319  N   LEU A 834      17.972   6.249   8.407  1.00123.18
N
ANISOU 6319  N   LEU A 834    19919  12909  13973  -2334   -565
2629       N
ATOM   6320  CA  LEU A 834      16.603   6.063   8.885  1.00116.68
C
ANISOU 6320  CA  LEU A 834    19365  11935  13033  -2142   -388
2730       C
ATOM   6321  C   LEU A 834      16.537   5.937  10.403  1.00105.96
C
ANISOU 6321  C   LEU A 834    18413  10288  11558  -2021   -513
```

```
2730       C
ATOM   6322  O   LEU A 834      15.647   6.508  11.045  1.00 82.25
O
ANISOU 6322  O   LEU A 834    15743   7098   8412  -1936   -397
2811       O
ATOM   6323  CB  LEU A 834      15.982   4.822   8.238  1.00108.00
C
ANISOU 6323  CB  LEU A 834    17993  11011  12031  -2023   -236
2691       C
ATOM   6324  CG  LEU A 834      15.541   4.843   6.776  1.00 96.11
C
ANISOU 6324  CG  LEU A 834    16156   9780  10583  -2073    -48
2707       C
ATOM   6325  CD1 LEU A 834      15.317   3.423   6.290  1.00127.05
C
ANISOU 6325  CD1 LEU A 834    19762  13869  14642  -1967     16
2598       C
ATOM   6326  CD2 LEU A 834      14.256   5.637   6.647  1.00 94.95
C
ANISOU 6326  CD2 LEU A 834    16221   9534  10320  -2003    151
2846       C
ATOM   6327  N   ASP A 835      17.481   5.200  10.994  1.00 94.85
N
ANISOU 6327  N   ASP A 835    16976   8853  10212  -2004   -757
2621       N
ATOM   6328  CA  ASP A 835      17.603   5.129  12.448  1.00 78.62
C
ANISOU 6328  CA  ASP A 835    15331   6528   8012  -1905   -929
2621       C
ATOM   6329  C   ASP A 835      17.866   6.486  13.093  1.00 80.03
C
ANISOU 6329  C   ASP A 835    15820   6530   8056  -1991  -1031
2643       C
ATOM   6330  O   ASP A 835      17.266   6.811  14.123  1.00 97.31
O
ANISOU 6330  O   ASP A 835    18409   8508  10055  -1889   -997
2686       O
ATOM   6331  CB  ASP A 835      18.706   4.138  12.819  1.00 83.04
C
ANISOU 6331  CB  ASP A 835    15760   7102   8689  -1876  -1229
2489       C
ATOM   6332  CG  ASP A 835      18.277   2.696  12.626  1.00 78.82
C
ANISOU 6332  CG  ASP A 835    15069   6628   8250  -1737  -1150
2479       C
ATOM   6333  OD1 ASP A 835      17.075   2.404  12.793  1.00 77.78
O
ANISOU 6333  OD1 ASP A 835    15110   6421   8020  -1628   -899
2585       O
ATOM   6334  OD2 ASP A 835      19.141   1.854  12.303  1.00106.62
O
ANISOU 6334  OD2 ASP A 835    18279  10264  11967  -1739  -1336
2348       O
ATOM   6335  N   VAL A 836      18.764   7.287  12.516  1.00 80.79
```

```
N
ANISOU 6335  N   VAL A 836    15717   6720   8260  -2179  -1143
2591       N
ATOM   6336  CA  VAL A 836      18.999   8.631  13.044  1.00 87.50
C
ANISOU 6336  CA  VAL A 836    16837   7390   9018  -2276  -1232
2606       C
ATOM   6337  C   VAL A 836      17.730   9.475  12.996  1.00103.88
C
ANISOU 6337  C   VAL A 836    19142   9363  10965  -2225   -968
2737       C
ATOM   6338  O   VAL A 836      17.401  10.181  13.958  1.00118.63
O
ANISOU 6338  O   VAL A 836    21264  11089  12723  -2131   -982
2677       O
ATOM   6339  CB  VAL A 836      20.157   9.304  12.285  1.00109.14
C
ANISOU 6339  CB  VAL A 836    19274  10260  11934  -2516  -1356
2531       C
ATOM   6340  CG1 VAL A 836      20.368  10.729  12.785  1.00105.05
C
ANISOU 6340  CG1 VAL A 836    19028   9535  11351  -2628  -1440
2547       C
ATOM   6341  CG2 VAL A 836      21.434   8.483  12.437  1.00 91.60
C
ANISOU 6341  CG2 VAL A 836    16800   8130   9872  -2545  -1638
2349       C
ATOM   6342  N   PHE A 837      17.001   9.421  11.880  1.00104.75
N
ANISOU 6342  N   PHE A 837    18971   9673  11155  -2212   -717
2793       N
ATOM   6343  CA  PHE A 837      15.678  10.038  11.810  1.00 88.65
C
ANISOU 6343  CA  PHE A 837    16907   7675   9102  -2047   -463
2784       C
ATOM   6344  C   PHE A 837      14.704   9.437  12.820  1.00116.29
C
ANISOU 6344  C   PHE A 837    20551  11114  12519  -1807   -347
2711       C
ATOM   6345  O   PHE A 837      13.950  10.167  13.475  1.00137.77
O
ANISOU 6345  O   PHE A 837    23438  13738  15172  -1705   -256
2664       O
ATOM   6346  CB  PHE A 837      15.130   9.925  10.387  1.00 77.61
C
ANISOU 6346  CB  PHE A 837    15192   6495   7803  -2070   -267
2861       C
ATOM   6347  CG  PHE A 837      15.566  11.047   9.488  1.00129.95
C
ANISOU 6347  CG  PHE A 837    21729  13172  14476  -2264   -285
2936       C
ATOM   6348  CD1 PHE A 837      14.641  11.939   8.971  1.00122.70
C
ANISOU 6348  CD1 PHE A 837    20755  12283  13582  -2186   -135
```

2977 C
ATOM 6349 CD2 PHE A 837 16.906 11.221 9.177 1.00121.42
C
ANISOU 6349 CD2 PHE A 837 20628 12086 13422 -2540 -463
2972 C
ATOM 6350 CE1 PHE A 837 15.042 12.980 8.152 1.00113.08
C
ANISOU 6350 CE1 PHE A 837 19473 11093 12399 -2359 -154
3061 C
ATOM 6351 CE2 PHE A 837 17.315 12.259 8.359 1.00122.91
C
ANISOU 6351 CE2 PHE A 837 20731 12316 13651 -2745 -453
3047 C
ATOM 6352 CZ PHE A 837 16.381 13.141 7.845 1.00129.06
C
ANISOU 6352 CZ PHE A 837 21469 13129 14438 -2642 -296
3097 C
ATOM 6353 N LYS A 838 14.701 8.110 12.954 1.00101.98
N
ANISOU 6353 N LYS A 838 18688 9345 10714 -1734 -342
2705 N
ATOM 6354 CA LYS A 838 13.868 7.446 13.956 1.00 90.04
C
ANISOU 6354 CA LYS A 838 17325 7768 9119 -1542 -228
2647 C
ATOM 6355 C LYS A 838 14.199 7.893 15.378 1.00100.67
C
ANISOU 6355 C LYS A 838 19046 8920 10284 -1512 -382
2579 C
ATOM 6356 O LYS A 838 13.296 8.166 16.178 1.00110.26
O
ANISOU 6356 O LYS A 838 20437 10064 11392 -1396 -231
2524 O
ATOM 6357 CB LYS A 838 14.010 5.928 13.823 1.00 90.35
C
ANISOU 6357 CB LYS A 838 17258 7858 9214 -1496 -240
2667 C
ATOM 6358 CG LYS A 838 13.283 5.140 14.900 1.00 92.05
C
ANISOU 6358 CG LYS A 838 17644 7992 9340 -1331 -135
2622 C
ATOM 6359 CD LYS A 838 13.605 3.660 14.812 1.00102.97
C
ANISOU 6359 CD LYS A 838 18952 9379 10792 -1298 -200
2653 C
ATOM 6360 CE LYS A 838 14.971 3.356 15.397 1.00123.16
C
ANISOU 6360 CE LYS A 838 21704 11806 13286 -1357 -568
2656 C
ATOM 6361 NZ LYS A 838 15.448 2.001 15.007 1.00142.68
N
ANISOU 6361 NZ LYS A 838 24059 14269 15882 -1351 -689
2696 N
ATOM 6362 N PHE A 839 15.487 7.964 15.716 1.00113.98

```
N
ANISOU 6362  N   PHE A 839    20879  10503  11924  -1626   -693
2579       N
ATOM   6363  CA  PHE A 839      15.894   8.495  17.016  1.00 93.29
C
ANISOU 6363  CA  PHE A 839    18644   7686   9116  -1610   -884
2512       C
ATOM   6364  C   PHE A 839      15.444   9.940  17.209  1.00 89.99
C
ANISOU 6364  C   PHE A 839    18354   7192   8648  -1634   -798
2474       C
ATOM   6365  O   PHE A 839      14.926  10.307  18.270  1.00133.49
O
ANISOU 6365  O   PHE A 839    24158  12580  13984  -1541   -750
2403       O
ATOM   6366  CB  PHE A 839      17.415   8.401  17.166  1.00 99.81
C
ANISOU 6366  CB  PHE A 839    19581   8396   9946  -1750  -1283
2521       C
ATOM   6367  CG  PHE A 839      17.960   7.003  17.064  1.00108.59
C
ANISOU 6367  CG  PHE A 839    20612   9529  11117  -1720  -1438
2553       C
ATOM   6368  CD1 PHE A 839      19.269   6.791  16.662  1.00119.67
C
ANISOU 6368  CD1 PHE A 839    21826  10963  12680  -1846  -1748
2505       C
ATOM   6369  CD2 PHE A 839      17.172   5.904  17.367  1.00119.00
C
ANISOU 6369  CD2 PHE A 839    21909  10905  12402  -1548  -1263
2555       C
ATOM   6370  CE1 PHE A 839      19.785   5.513  16.564  1.00133.10
C
ANISOU 6370  CE1 PHE A 839    23317  12747  14505  -1776  -1898
2449       C
ATOM   6371  CE2 PHE A 839      17.683   4.621  17.270  1.00139.65
C
ANISOU 6371  CE2 PHE A 839    24461  13508  15093  -1514  -1425
2588       C
ATOM   6372  CZ  PHE A 839      18.990   4.426  16.867  1.00145.16
C
ANISOU 6372  CZ  PHE A 839    24974  14228  15952  -1619  -1759
2534       C
ATOM   6373  N   ALA A 840      15.641  10.774  16.189  1.00 83.83
N
ANISOU 6373  N   ALA A 840    17371   6468   8011  -1768   -779
2523       N
ATOM   6374  CA  ALA A 840      15.274  12.186  16.264  1.00 84.67
C
ANISOU 6374  CA  ALA A 840    17587   6477   8108  -1799   -724
2501       C
ATOM   6375  C   ALA A 840      13.783  12.419  16.513  1.00 96.16
C
ANISOU 6375  C   ALA A 840    19072   7939   9523  -1625   -435
```

```
2463       C
ATOM   6376  O   ALA A 840      13.418  13.268  17.335  1.00114.32
O
ANISOU 6376  O   ALA A 840    21654  10074  11708  -1586   -425
2394       O
ATOM   6377  CB  ALA A 840      15.708  12.902  14.985  1.00 84.82
C
ANISOU 6377  CB  ALA A 840    17350   6575   8303  -1976   -735
2586       C
ATOM   6378  N   ILE A 841      12.904  11.690  15.822  1.00102.00
N
ANISOU 6378  N   ILE A 841    19548   8846  10363  -1531   -207
2499       N
ATOM   6379  CA  ILE A 841      11.471  11.882  16.051  1.00125.86
C
ANISOU 6379  CA  ILE A 841    22607  11846  13367  -1383     54
2457       C
ATOM   6380  C   ILE A 841      11.033  11.375  17.422  1.00151.81
C
ANISOU 6380  C   ILE A 841    26203  15022  16455  -1273    122
2369       C
ATOM   6381  O   ILE A 841      10.178  11.991  18.070  1.00127.64
O
ANISOU 6381  O   ILE A 841    23363  11836  13298  -1200    264
2301       O
ATOM   6382  CB  ILE A 841      10.650  11.237  14.918  1.00 88.19
C
ANISOU 6382  CB  ILE A 841    17485   7260   8762  -1323    256
2517       C
ATOM   6383  CG1 ILE A 841       9.183  11.675  15.008  1.00 80.33
C
ANISOU 6383  CG1 ILE A 841    16533   6205   7782  -1196    498
2475       C
ATOM   6384  CG2 ILE A 841      10.778   9.716  14.923  1.00 96.10
C
ANISOU 6384  CG2 ILE A 841    18368   8371   9776  -1284    283
2527       C
ATOM   6385  CD1 ILE A 841       8.985  13.179  14.925  1.00 81.26
C
ANISOU 6385  CD1 ILE A 841    16769   6189   7916  -1222    465
2472       C
ATOM   6386  N   ARG A 842      11.586  10.264  17.896  1.00135.18
N
ANISOU 6386  N   ARG A 842    24146  12943  14274  -1262     27
2371       N
ATOM   6387  CA  ARG A 842      11.119   9.712  19.160  1.00111.79
C
ANISOU 6387  CA  ARG A 842    21484   9884  11106  -1163    111
2306       C
ATOM   6388  C   ARG A 842      11.973  10.157  20.339  1.00124.24
C
ANISOU 6388  C   ARG A 842    23475  11284  12448  -1200   -137
2251       C
ATOM   6389  O   ARG A 842      11.728   9.723  21.469  1.00131.24
```

O
ANISOU 6389 O   ARG A 842   24670 12081 13116 -1130   -101
2202   O
ATOM   6390 CB ARG A 842   11.027   8.182 19.094 1.00 84.00
C
ANISOU 6390 CB ARG A 842   17819   6468   7629 -1107   173
2346   C
ATOM   6391 CG ARG A 842   12.324   7.414 19.204 1.00 84.38
C
ANISOU 6391 CG ARG A 842   17884   6516   7660 -1159   -125
2387   C
ATOM   6392 CD ARG A 842   12.128   6.028 18.608 1.00105.68
C
ANISOU 6392 CD ARG A 842   20312   9337 10505 -1116   -36
2447   C
ATOM   6393 NE ARG A 842   10.960   5.372 19.185 1.00146.12
N
ANISOU 6393 NE ARG A 842   25510 14443 15566 -1010   240
2424   N
ATOM   6394 CZ ARG A 842   10.214   4.459 18.570 1.00126.15
C
ANISOU 6394 CZ ARG A 842   22727 12009 13194   -964   449
2458   C
ATOM   6395 NH1 ARG A 842   9.050   4.095 19.078 1.00139.13
N
ANISOU 6395 NH1 ARG A 842   24456 13617 14789   -893   728
2427   N
ATOM   6396 NH2 ARG A 842   10.563   4.027 17.366 1.00 81.82
N
ANISOU 6396 NH2 ARG A 842   16779   6520   7790 -1004   400
2515   N
ATOM   6397 N   TYR A 843   12.969 11.011 20.096 1.00 93.63
N
ANISOU 6397 N   TYR A 843   19627   7344   8605 -1318   -391
2259   N
ATOM   6398 CA TYR A 843   13.549 11.812 21.167 1.00103.65
C
ANISOU 6398 CA TYR A 843   21320   8404   9660 -1355   -606
2186   C
ATOM   6399 C   TYR A 843   12.516 12.745 21.790 1.00157.51
C
ANISOU 6399 C   TYR A 843   28401 15095 16352 -1290   -398
2105   C
ATOM   6400 O   TYR A 843   12.470 12.902 23.016 1.00209.86
O
ANISOU 6400 O   TYR A 843   35461 21567 22712 -1247   -438
2025   O
ATOM   6401 CB TYR A 843   14.734 12.609 20.620 1.00113.60
C
ANISOU 6401 CB TYR A 843   22518   9607 11039 -1520   -894
2214   C
ATOM   6402 CG TYR A 843   15.432 13.489 21.630 1.00133.24
C
ANISOU 6402 CG TYR A 843   25433 11851 13341 -1577 -1164

```
2136       C
ATOM   6403  CD1 TYR A 843      15.137  14.845  21.715  1.00126.92
C
ANISOU 6403  CD1 TYR A 843    24774  10905  12545  -1621  -1128
2089       C
ATOM   6404  CD2 TYR A 843      16.392  12.971  22.490  1.00144.45
C
ANISOU 6404  CD2 TYR A 843    27129  13164  14590  -1582  -1484
2107       C
ATOM   6405  CE1 TYR A 843      15.770  15.658  22.636  1.00147.50
C
ANISOU 6405  CE1 TYR A 843    27788  13264  14991  -1676  -1387
2007       C
ATOM   6406  CE2 TYR A 843      17.035  13.778  23.413  1.00156.62
C
ANISOU 6406  CE2 TYR A 843    29085  14466  15956  -1629  -1766
2028       C
ATOM   6407  CZ  TYR A 843      16.718  15.121  23.482  1.00165.91
C
ANISOU 6407  CZ  TYR A 843    30397  15498  17142  -1681  -1709
1974       C
ATOM   6408  OH  TYR A 843      17.356  15.931  24.394  1.00158.45
O
ANISOU 6408  OH  TYR A 843    29877  14289  16037  -1731  -2002
1883       O
ATOM   6409  N   ILE A 844      11.675  13.366  20.967  1.00113.78
N
ANISOU 6409  N   ILE A 844    22636   9603  10991  -1278   -184
2122       N
ATOM   6410  CA  ILE A 844      10.711  14.344  21.462  1.00155.87
C
ANISOU 6410  CA  ILE A 844    28214  14777  16234  -1219     -4
2043       C
ATOM   6411  C   ILE A 844       9.334  13.713  21.658  1.00128.27
C
ANISOU 6411  C   ILE A 844    24708  11327  12703  -1089    352
2013       C
ATOM   6412  O   ILE A 844       8.926  12.831  20.902  1.00 89.12
O
ANISOU 6412  O   ILE A 844    19408   6546   7906  -1054    489
2075       O
ATOM   6413  CB  ILE A 844      10.626  15.568  20.526  1.00110.25
C
ANISOU 6413  CB  ILE A 844    22257   8961  10673  -1282    -22
2076       C
ATOM   6414  CG1 ILE A 844       9.632  16.595  21.076  1.00102.46
C
ANISOU 6414  CG1 ILE A 844    21554   7767   9609  -1208    143
1985       C
ATOM   6415  CG2 ILE A 844      10.247  15.143  19.118  1.00 88.48
C
ANISOU 6415  CG2 ILE A 844    19016   6423   8180  -1277    102
2179       C
ATOM   6416  CD1 ILE A 844      10.004  17.138  22.440  1.00125.52
```

```
C
ANISOU 6416  CD1 ILE A 844    24997  10448  12249  -1219     16
1873       C
TER    6417      ILE A 844
ATOM   6418  N   VAL B  12      19.962 -69.215  74.672  1.00257.54
N
ANISOU 6418  N   VAL B  12    54866  20710  22277   2392 -12586
-1703       N
ATOM   6419  CA  VAL B  12      18.559 -68.895  74.908  1.00249.42
C
ANISOU 6419  CA  VAL B  12    53727  19727  21313   1305 -12044
-1294       C
ATOM   6420  C   VAL B  12      17.671 -69.678  73.949  1.00258.66
C
ANISOU 6420  C   VAL B  12    55247  20631  22401    853 -12095
-1213       C
ATOM   6421  O   VAL B  12      18.163 -70.371  73.059  1.00268.79
O
ANISOU 6421  O   VAL B  12    56768  21743  23618   1406 -12517
-1469       O
ATOM   6422  CB  VAL B  12      18.299 -67.383  74.777  1.00241.12
C
ANISOU 6422  CB  VAL B  12    51306  19552  20758   1068 -11332
-1202       C
ATOM   6423  CG1 VAL B  12      19.151 -66.611  75.771  1.00223.29
C
ANISOU 6423  CG1 VAL B  12    48734  17539  18566   1475 -11280
-1266       C
ATOM   6424  CG2 VAL B  12      18.568 -66.914  73.354  1.00216.78
C
ANISOU 6424  CG2 VAL B  12    47247  17064  18055   1468 -11180
-1436       C
ATOM   6425  N   ASP B  13      16.354 -69.562  74.136  1.00251.74
N
ANISOU 6425  N   ASP B  13    54387  19739  21523   -155 -11663
-868       N
ATOM   6426  CA  ASP B  13      15.409 -70.377  73.383  1.00237.58
C
ANISOU 6426  CA  ASP B  13    53046  17626  19596   -697 -11715
-760       C
ATOM   6427  C   ASP B  13      15.225 -69.886  71.954  1.00230.93
C
ANISOU 6427  C   ASP B  13    51195  17374  19172   -568 -11437
-891       C
ATOM   6428  O   ASP B  13      14.812 -70.669  71.092  1.00229.31
O
ANISOU 6428  O   ASP B  13    51356  16899  18871   -691 -11633
-920       O
ATOM   6429  CB  ASP B  13      14.058 -70.412  74.098  1.00234.76
C
ANISOU 6429  CB  ASP B  13    53028  17094  19078  -1843 -11338
-371       C
ATOM   6430  CG  ASP B  13      14.126 -71.131  75.431  1.00237.85
C
```

```
ANISOU 6430  CG  ASP B  13    54628  16770  18973  -2062 -11670
-226       C
ATOM   6431  OD1 ASP B  13      15.150 -71.793  75.700  1.00241.43
O
ANISOU 6431  OD1 ASP B  13    55810  16759  19165  -1330 -12269
-432       O
ATOM   6432  OD2 ASP B  13      13.155 -71.033  76.210  1.00238.63
O
ANISOU 6432  OD2 ASP B  13    54956  16781  18933  -2960 -11338
81       O
ATOM   6433  N   LEU B  14      15.526 -68.616  71.685  1.00225.48
N
ANISOU 6433  N   LEU B  14    49280  17465  18927   -327 -10996
-971       N
ATOM   6434  CA  LEU B  14      15.451 -68.091  70.329  1.00245.55
C
ANISOU 6434  CA  LEU B  14    50858  20580  21862   -145 -10743
-1117       C
ATOM   6435  C   LEU B  14      16.809 -68.261  69.665  1.00251.45
C
ANISOU 6435  C   LEU B  14    51450  21422  22666    930 -11171
-1514       C
ATOM   6436  O   LEU B  14      17.074 -69.293  69.039  1.00245.02
O
ANISOU 6436  O   LEU B  14    51266  20184  21646   1278 -11661
-1681       O
ATOM   6437  CB  LEU B  14      15.040 -66.615  70.330  1.00216.02
C
ANISOU 6437  CB  LEU B  14    45893  17633  18550   -465 -10046
-1003       C
ATOM   6438  CG  LEU B  14      13.586 -66.228  70.628  1.00209.36
C
ANISOU 6438  CG  LEU B  14    44861  16925  17759  -1513  -9515
-665       C
ATOM   6439  CD1 LEU B  14      12.639 -66.834  69.600  1.00211.27
C
ANISOU 6439  CD1 LEU B  14    45236  17040  17996  -1963  -9488
-619       C
ATOM   6440  CD2 LEU B  14      13.167 -66.596  72.047  1.00212.54
C
ANISOU 6440  CD2 LEU B  14    46088  16860  17808  -2066  -9553
-407       C
ATOM   6441  N   GLU B  15      17.669 -67.251  69.801  1.00237.75
N
ANISOU 6441  N   GLU B  15    48879  20257  21198   1450 -10992
-1677       N
ATOM   6442  CA  GLU B  15      19.067 -67.308  69.367  1.00231.44
C
ANISOU 6442  CA  GLU B  15    47882  19619  20435   2485 -11378
-2081       C
ATOM   6443  C   GLU B  15      19.100 -67.562  67.864  1.00219.45
C
ANISOU 6443  C   GLU B  15    46010  18292  19077   2770 -11454
-2288       C
```

```
ATOM   6444  O   GLU B  15      18.559 -66.738  67.106  1.00208.21
O
ANISOU 6444  O   GLU B  15    43677  17422  18010   2463 -10954
-2224       O
ATOM   6445  CB  GLU B  15      19.811 -68.327  70.235  1.00246.94
C
ANISOU 6445  CB  GLU B  15    50980  20895  21951   2939 -12032
-2196       C
ATOM   6446  CG  GLU B  15      21.311 -68.089  70.323  1.00252.66
C
ANISOU 6446  CG  GLU B  15    51417  21879  22705   3956 -12343
-2587       C
ATOM   6447  CD  GLU B  15      21.963 -68.883  71.438  1.00260.52
C
ANISOU 6447  CD  GLU B  15    53476  22238  23270   4327 -12910
-2663       C
ATOM   6448  OE1 GLU B  15      22.879 -69.680  71.146  1.00262.36
O
ANISOU 6448  OE1 GLU B  15    53955  22221  23509   5098 -13324
-2907       O
ATOM   6449  OE2 GLU B  15      21.559 -68.711  72.608  1.00260.36
O
ANISOU 6449  OE2 GLU B  15    53813  21988  23122   3841 -12761
-2391       O
ATOM   6450  N   LYS B  16      19.704 -68.649  67.390  1.00223.02
N
ANISOU 6450  N   LYS B  16    47143  18317  19278   3353 -12061
-2540       N
ATOM   6451  CA  LYS B  16      19.811 -68.943  65.969  1.00239.28
C
ANISOU 6451  CA  LYS B  16    48906  20540  21469   3685 -12175
-2759       C
ATOM   6452  C   LYS B  16      18.691 -69.843  65.452  1.00249.85
C
ANISOU 6452  C   LYS B  16    50893  21385  22653   3078 -12254
-2556       C
ATOM   6453  O   LYS B  16      18.754 -70.283  64.299  1.00245.94
O
ANISOU 6453  O   LYS B  16    50334  20904  22208   3358 -12432
-2732       O
ATOM   6454  CB  LYS B  16      21.170 -69.590  65.687  1.00240.52
C
ANISOU 6454  CB  LYS B  16    49376  20568  21442   4737 -12808
-3194       C
ATOM   6455  CG  LYS B  16      22.349 -68.662  65.937  1.00224.57
C
ANISOU 6455  CG  LYS B  16    46554  19157  19614   5400 -12717
-3466       C
ATOM   6456  CD  LYS B  16      23.673 -69.334  65.618  1.00228.03
C
ANISOU 6456  CD  LYS B  16    47142  19517  19981   6429 -13239
-3872       C
ATOM   6457  CE  LYS B  16      24.840 -68.399  65.894  1.00219.55
C
```

```
ANISOU 6457  CE  LYS B  16    45252  19081  19088   7046 -13131
-4158       C
ATOM   6458  NZ  LYS B  16      26.153 -69.047  65.624  1.00230.52
N
ANISOU 6458  NZ  LYS B  16    46622  20433  20533   8027 -13512
-4521       N
ATOM   6459  N   ILE B  17      17.680 -70.132  66.265  1.00251.19
N
ANISOU 6459  N   ILE B  17    51681  21134  22627   2250 -12126
-2202       N
ATOM   6460  CA  ILE B  17      16.545 -70.949  65.853  1.00247.31
C
ANISOU 6460  CA  ILE B  17    51797  20195  21973   1573 -12160
-1993       C
ATOM   6461  C   ILE B  17      15.316 -70.044  65.765  1.00239.70
C
ANISOU 6461  C   ILE B  17    50079  19684  21311    677 -11444
-1699       C
ATOM   6462  O   ILE B  17      14.859 -69.525  66.795  1.00218.27
O
ANISOU 6462  O   ILE B  17    47320  17032  18582    143 -11120
-1458       O
ATOM   6463  CB  ILE B  17      16.306 -72.116  66.820  1.00252.90
C
ANISOU 6463  CB  ILE B  17    53911  20024  22155   1280 -12616
-1837       C
ATOM   6464  CG1 ILE B  17      17.592 -72.922  67.016  1.00258.05
C
ANISOU 6464  CG1 ILE B  17    55186  20274  22586   2242 -13265
-2102       C
ATOM   6465  CG2 ILE B  17      15.185 -73.011  66.308  1.00239.55
C
ANISOU 6465  CG2 ILE B  17    52866  17874  20277    596 -12682
-1649       C
ATOM   6466  CD1 ILE B  17      18.148 -73.513  65.739  1.00235.89
C
ANISOU 6466  CD1 ILE B  17    52226  17485  19916   2940 -13559
-2356       C
ATOM   6467  N   PRO B  18      14.746 -69.823  64.572  1.00250.57
N
ANISOU 6467  N   PRO B  18    50855  21391  22957    500 -11186
-1719       N
ATOM   6468  CA  PRO B  18      13.691 -68.806  64.441  1.00242.67
C
ANISOU 6468  CA  PRO B  18    48996  20926  22280   -231 -10497
-1498       C
ATOM   6469  C   PRO B  18      12.369 -69.280  65.029  1.00255.40
C
ANISOU 6469  C   PRO B  18    51228  22149  23664  -1242 -10346
-1163       C
ATOM   6470  O   PRO B  18      11.750 -68.567  65.826  1.00230.04
O
ANISOU 6470  O   PRO B  18    47707  19177  20520  -1829  -9907
-942       O
```

```
ATOM   6471  CB  PRO B  18      13.588 -68.591  62.924  1.00208.97
C
ANISOU 6471  CB  PRO B  18    44031  17048  18320    -14 -10373
-1667       C
ATOM   6472  CG  PRO B  18      14.148 -69.839  62.319  1.00213.59
C
ANISOU 6472  CG  PRO B  18    45407  17102  18647    525 -11013
-1875       C
ATOM   6473  CD  PRO B  18      15.210 -70.318  63.265  1.00217.17
C
ANISOU 6473  CD  PRO B  18    46551  17171  18792   1092 -11496
-1996       C
ATOM   6474  N   ILE B  19      11.943 -70.487  64.631  1.00264.23
N
ANISOU 6474  N   ILE B  19    53221  22677  24497  -1447 -10714
-1137       N
ATOM   6475  CA  ILE B  19      10.774 -71.213  65.138  1.00255.99
C
ANISOU 6475  CA  ILE B  19    52980  21144  23141  -2382 -10692
-853       C
ATOM   6476  C   ILE B  19       9.581 -70.281  65.380  1.00265.67
C
ANISOU 6476  C   ILE B  19    53476  22870  24594  -3259 -10001
-613       C
ATOM   6477  O   ILE B  19       8.962 -70.281  66.451  1.00265.86
O
ANISOU 6477  O   ILE B  19    53866  22734  24415  -3932  -9827
-375       O
ATOM   6478  CB  ILE B  19      11.164 -72.057  66.376  1.00239.71
C
ANISOU 6478  CB  ILE B  19    52116  18370  20591  -2379 -11142
-771       C
ATOM   6479  CG1 ILE B  19      10.038 -73.020  66.782  1.00233.64
C
ANISOU 6479  CG1 ILE B  19    52348  16999  19426  -3315 -11217
-508       C
ATOM   6480  CG2 ILE B  19      11.703 -71.206  67.523  1.00241.18
C
ANISOU 6480  CG2 ILE B  19    51984  18815  20838  -2228 -10946
-728       C
ATOM   6481  CD1 ILE B  19       9.557 -73.908  65.656  1.00236.07
C
ANISOU 6481  CD1 ILE B  19    53023  17017  19654  -3437 -11451
-559       C
ATOM   6482  N   GLU B  20       9.271 -69.467  64.369  1.00260.05
N
ANISOU 6482  N   GLU B  20    51721  22785  24300  -3232  -9611
-694       N
ATOM   6483  CA  GLU B  20       8.178 -68.498  64.448  1.00254.26
C
ANISOU 6483  CA  GLU B  20    50188  22603  23818  -3960  -8964
-520       C
ATOM   6484  C   GLU B  20       6.820 -69.157  64.687  1.00270.89
C
```

```
ANISOU 6484  C   GLU B  20    52859  24394  25670  -4968  -8854
-288        C
ATOM   6485  O   GLU B  20       6.022 -68.678  65.502  1.00271.23
O
ANISOU 6485  O   GLU B  20    52739  24631  25685  -5671  -8459
-87        O
ATOM   6486  CB  GLU B  20       8.136 -67.668  63.163  1.00247.06
C
ANISOU 6486  CB  GLU B  20    48184  22331  23357  -3675  -8663
-685        C
ATOM   6487  CG  GLU B  20       6.830 -66.919  62.941  1.00257.66
C
ANISOU 6487  CG  GLU B  20    48819  24160  24921  -4447  -8079
-542        C
ATOM   6488  CD  GLU B  20       6.981 -65.743  61.996  1.00270.92
C
ANISOU 6488  CD  GLU B  20    49311  26563  27063  -4087  -7721
-698        C
ATOM   6489  OE1 GLU B  20       7.625 -64.745  62.382  1.00266.72
O
ANISOU 6489  OE1 GLU B  20    48174  26447  26722  -3725  -7525
-744        O
ATOM   6490  OE2 GLU B  20       6.453 -65.815  60.866  1.00275.61
O
ANISOU 6490  OE2 GLU B  20    49599  27300  27819  -4176  -7640
-775        O
ATOM   6491  N   GLU B  21       6.550 -70.258  63.983  1.00279.79
N
ANISOU 6491  N   GLU B  21    54653  25050  26602  -5055  -9201
-324        N
ATOM   6492  CA  GLU B  21       5.232 -70.896  63.990  1.00298.44
C
ANISOU 6492  CA  GLU B  21    57485  27162  28747  -6017  -9089
-137        C
ATOM   6493  C   GLU B  21       4.753 -71.323  65.378  1.00312.04
C
ANISOU 6493  C   GLU B  21    60023  28480  30058  -6732  -9091
107        C
ATOM   6494  O   GLU B  21       3.586 -71.105  65.724  1.00322.17
O
ANISOU 6494  O   GLU B  21    61146  29959  31304  -7631  -8683
283        O
ATOM   6495  CB  GLU B  21       5.248 -72.098  63.044  1.00307.26
C
ANISOU 6495  CB  GLU B  21    59309  27755  29683  -5860  -9564
-234        C
ATOM   6496  CG  GLU B  21       3.972 -72.922  63.055  1.00318.45
C
ANISOU 6496  CG  GLU B  21    61363  28822  30813  -6841  -9530
-52        C
ATOM   6497  CD  GLU B  21       3.977 -74.017  62.006  1.00320.99
C
ANISOU 6497  CD  GLU B  21    62284  28678  30998  -6657  -9976
-158        C
```

ATOM   6498 OE1 GLU B 21   3.132 -73.966 61.088 1.00318.23
O
ANISOU 6498 OE1 GLU B 21   61508 28577 30829 -7025 -9748
-172   O
ATOM   6499 OE2 GLU B 21   4.826 -74.928 62.100 1.00323.23
O
ANISOU 6499 OE2 GLU B 21   63473 28351 30988 -6127 -10568
-237   O
ATOM   6500 N   VAL B 22   5.620 -71.924 66.193 1.00301.03
N
ANISOU 6500 N   VAL B 22   59502 26536 28340 -6358 -9540
110   N
ATOM   6501 CA VAL B 22   5.191 -72.302 67.540 1.00287.73
C
ANISOU 6501 CA VAL B 22   58615 24458 26252 -7037 -9538
344   C
ATOM   6502 C   VAL B 22   5.217 -71.129 68.518 1.00282.61
C
ANISOU 6502 C   VAL B 22   57279 24319 25781 -7156 -9083
441   C
ATOM   6503 O   VAL B 22   4.340 -71.016 69.383 1.00298.92
O
ANISOU 6503 O   VAL B 22   59491 26412 27674 -7998 -8774
656   O
ATOM   6504 CB VAL B 22   6.060 -73.472 68.041 1.00277.76
C
ANISOU 6504 CB VAL B 22   58660 22347 24529 -6612 -10234
309   C
ATOM   6505 CG1 VAL B 22   5.637 -73.899 69.441 1.00277.34
C
ANISOU 6505 CG1 VAL B 22   59512 21839 24024 -7321 -10252
553   C
ATOM   6506 CG2 VAL B 22   5.980 -74.642 67.069 1.00257.73
C
ANISOU 6506 CG2 VAL B 22   56833 19296 21796 -6518 -10693
218   C
ATOM   6507 N   PHE B 23   6.200 -70.237 68.407 1.00260.47
N
ANISOU 6507 N   PHE B 23   53719 21938 23309 -6344 -9029
282   N
ATOM   6508 CA PHE B 23   6.304 -69.118 69.345 1.00248.34
C
ANISOU 6508 CA PHE B 23   51558 20864 21935 -6402 -8630
369   C
ATOM   6509 C   PHE B 23   5.113 -68.164 69.299 1.00240.57
C
ANISOU 6509 C   PHE B 23   49662 20539 21205 -7143 -7965
493   C
ATOM   6510 O   PHE B 23   4.618 -67.744 70.352 1.00236.83
O
ANISOU 6510 O   PHE B 23   49161 20193 20632 -7693 -7663
673   O
ATOM   6511 CB PHE B 23   7.625 -68.378 69.149 1.00233.14
C

```
ANISOU 6511  CB  PHE B  23    49009  19266  20309  -5376  -8724
153       C
ATOM   6512  CG  PHE B  23       8.761 -68.988  69.920  1.00227.31
C
ANISOU 6512  CG  PHE B  23    49118  17978  19272  -4784  -9253
87       C
ATOM   6513  CD1 PHE B  23       9.164 -70.292  69.693  1.00225.92
C
ANISOU 6513  CD1 PHE B  23    50008  17085  18745  -4510  -9864
2       C
ATOM   6514  CD2 PHE B  23       9.416 -68.250  70.893  1.00218.61
C
ANISOU 6514  CD2 PHE B  23    47764  17073  18225  -4495  -9150
103       C
ATOM   6515  CE1 PHE B  23      10.197 -70.848  70.429  1.00229.28
C
ANISOU 6515  CE1 PHE B  23    51238  17003  18876  -3944 -10373
-80       C
ATOM   6516  CE2 PHE B  23      10.453 -68.798  71.621  1.00221.84
C
ANISOU 6516  CE2 PHE B  23    48946  16986  18355  -3938  -9643
24       C
ATOM   6517  CZ  PHE B  23      10.847 -70.097  71.385  1.00227.21
C
ANISOU 6517  CZ  PHE B  23    50688  16959  18683  -3649 -10261
-76       C
ATOM   6518  N   GLN B  24       4.632 -67.802  68.110  1.00233.08
N
ANISOU 6518  N   GLN B  24    47966  20020  20574  -7160  -7735
390       N
ATOM   6519  CA  GLN B  24       3.383 -67.043  68.048  1.00235.12
C
ANISOU 6519  CA  GLN B  24    47474  20848  21014  -7916  -7151
491       C
ATOM   6520  C   GLN B  24       2.192 -67.847  68.564  1.00258.65
C
ANISOU 6520  C   GLN B  24    51170  23491  23612  -8949  -7091
686       C
ATOM   6521  O   GLN B  24       1.407 -67.351  69.380  1.00263.92
O
ANISOU 6521  O   GLN B  24    51612  24442  24225  -9626  -6693
836       O
ATOM   6522  CB  GLN B  24       3.133 -66.567  66.617  1.00218.25
C
ANISOU 6522  CB  GLN B  24    44466  19190  19270  -7688  -6966
320       C
ATOM   6523  CG  GLN B  24       4.205 -65.630  66.086  1.00212.50
C
ANISOU 6523  CG  GLN B  24    42921  18893  18927  -6765  -6940
129       C
ATOM   6524  CD  GLN B  24       4.685 -64.636  67.131  1.00230.32
C
ANISOU 6524  CD  GLN B  24    44770  21469  21272  -6601  -6710
191       C
```

```
ATOM   6525  OE1 GLN B  24       5.874 -64.574  67.443  1.00240.31
O
ANISOU 6525  OE1 GLN B  24    46169  22603  22534  -5901  -6976
105        O
ATOM   6526  NE2 GLN B  24       3.762 -63.842  67.665  1.00226.74
N
ANISOU 6526  NE2 GLN B  24    43799  21458  20895  -7232  -6220
326        N
ATOM   6527  N   GLN B  25       2.038 -69.090  68.111  1.00271.01
N
ANISOU 6527  N   GLN B  25    53613  24463  24895  -9101  -7484
681        N
ATOM   6528  CA  GLN B  25       0.814 -69.828  68.413  1.00284.48
C
ANISOU 6528  CA  GLN B  25    55917  25913  26259 -10143  -7392
845        C
ATOM   6529  C   GLN B  25       0.776 -70.405  69.824  1.00288.49
C
ANISOU 6529  C   GLN B  25    57417  25906  26288 -10627  -7531
1046       C
ATOM   6530  O   GLN B  25      -0.318 -70.630  70.352  1.00295.73
O
ANISOU 6530  O   GLN B  25    58580  26825  26960 -11601  -7277
1203       O
ATOM   6531  CB  GLN B  25       0.590 -70.947  67.395  1.00295.31
C
ANISOU 6531  CB  GLN B  25    57885  26834  27487 -10193  -7751
775        C
ATOM   6532  CG  GLN B  25       0.292 -70.442  65.994  1.00295.87
C
ANISOU 6532  CG  GLN B  25    57004  27423  27989  -9958  -7544
603        C
ATOM   6533  CD  GLN B  25      -0.013 -71.564  65.023  1.00304.48
C
ANISOU 6533  CD  GLN B  25    58701  28065  28924 -10071  -7885
547        C
ATOM   6534  OE1 GLN B  25      -1.143 -71.704  64.554  1.00307.27
O
ANISOU 6534  OE1 GLN B  25    58877  28592  29279 -10769  -7643
573        O
ATOM   6535  NE2 GLN B  25       0.994 -72.373  64.718  1.00304.88
N
ANISOU 6535  NE2 GLN B  25    59469  27544  28830  -9373  -8460
455        N
ATOM   6536  N   LEU B  26       1.929 -70.645  70.448  1.00286.38
N
ANISOU 6536  N   LEU B  26    57721  25217  25873  -9987  -7923
1034       N
ATOM   6537  CA  LEU B  26       1.953 -71.293  71.755  1.00273.49
C
ANISOU 6537  CA  LEU B  26    57154  23009  23751 -10401  -8120
1217       C
ATOM   6538  C   LEU B  26       2.714 -70.494  72.809  1.00252.02
C
```

ANISOU 6538 C   LEU B 26   54186 20462 21107 -9998 -8028
1252   C
ATOM   6539 O   LEU B 26   2.173 -70.216 73.884 1.00248.72
O
ANISOU 6539 O   LEU B 26   53856 20128 20517 -10637 -7739
1431   O
ATOM   6540 CB LEU B 26   2.551 -72.697 71.631 1.00262.33
C
ANISOU 6540 CB LEU B 26   57053 20693 21926 -10112 -8814
1184   C
ATOM   6541 CG LEU B 26   2.698 -73.499 72.925 1.00249.76
C
ANISOU 6541 CG LEU B 26   56694 18423 19781 -10426 -9106
1367   C
ATOM   6542 CD1 LEU B 26   1.356 -73.634 73.629 1.00252.92
C
ANISOU 6542 CD1 LEU B 26   57133 19062 19903 -11543 -8647
1649   C
ATOM   6543 CD2 LEU B 26   3.291 -74.868 72.634 1.00255.32
C
ANISOU 6543 CD2 LEU B 26   58397 18489 20123 -9914 -9721
1391   C
ATOM   6544 N   LYS B 27   3.968 -70.135 72.518 1.00243.56
N
ANISOU 6544 N   LYS B 27   52813 19452 20278 -8952 -8274
1073   N
ATOM   6545 CA LYS B 27   4.822 -69.503 73.524 1.00257.83
C
ANISOU 6545 CA LYS B 27   54509 21335 22119 -8498 -8272
1088   C
ATOM   6546 C   LYS B 27   4.235 -68.186 74.022 1.00262.11
C
ANISOU 6546 C   LYS B 27   54016 22628 22945 -8889 -7627
1188   C
ATOM   6547 O   LYS B 27   4.244 -67.918 75.229 1.00256.61
O
ANISOU 6547 O   LYS B 27   53529 21887 22083 -9139 -7508
1334   O
ATOM   6548 CB LYS B 27   6.228 -69.291 72.963 1.00241.35
C
ANISOU 6548 CB LYS B 27   52138 19286 20277 -7316 -8613
839   C
ATOM   6549 CG LYS B 27   6.921 -70.579 72.544 1.00236.84
C
ANISOU 6549 CG LYS B 27   52609 17973 19404 -6823 -9299
710   C
ATOM   6550 CD LYS B 27   7.039 -71.553 73.704 1.00240.29
C
ANISOU 6550 CD LYS B 27   54397 17619 19282 -7093 -9691
852   C
ATOM   6551 CE LYS B 27   8.140 -72.570 73.455 1.00244.40
C
ANISOU 6551 CE LYS B 27   55838 17474 19551 -6274 -10427
661   C

```
ATOM   6552  NZ  LYS B  27       7.841 -73.431  72.277  1.00248.42
N
ANISOU 6552  NZ  LYS B  27    56670  17721  19999  -6287 -10693
569       N
ATOM   6553  N   CYS B  28       3.724 -67.350  73.119  1.00245.02
N
ANISOU 6553  N   CYS B  28    50752  21148  21195  -8933  -7217
1107       N
ATOM   6554  CA  CYS B  28       2.917 -66.212  73.541  1.00229.21
C
ANISOU 6554  CA  CYS B  28    47849  19837  19404  -9449  -6605
1205       C
ATOM   6555  C   CYS B  28       1.426 -66.438  73.327  1.00247.59
C
ANISOU 6555  C   CYS B  28    50119  22331  21624 -10465  -6275
1303       C
ATOM   6556  O   CYS B  28       0.619 -65.649  73.831  1.00251.71
O
ANISOU 6556  O   CYS B  28    50047  23368  22223 -11022  -5789
1392       O
ATOM   6557  CB  CYS B  28       3.354 -64.937  72.808  1.00210.60
C
ANISOU 6557  CB  CYS B  28    44232  18204  17581  -8807  -6332
1040       C
ATOM   6558  SG  CYS B  28       3.171 -64.992  71.013  1.00220.28
S
ANISOU 6558  SG  CYS B  28    44888  19679  19129  -8518  -6348
830       S
ATOM   6559  N   SER B  29       1.048 -67.490  72.597  1.00248.72
N
ANISOU 6559  N   SER B  29    50854  22065  21581 -10708  -6535
1273       N
ATOM   6560  CA  SER B  29      -0.350 -67.858  72.361  1.00242.40
C
ANISOU 6560  CA  SER B  29    50101  21366  20634 -11695  -6273
1348       C
ATOM   6561  C   SER B  29      -1.166 -66.727  71.739  1.00231.68
C
ANISOU 6561  C   SER B  29    47456  20880  19691 -11903  -5717
1266       C
ATOM   6562  O   SER B  29      -2.369 -66.612  71.992  1.00222.37
O
ANISOU 6562  O   SER B  29    46080  19999  18411 -12778  -5345
1342       O
ATOM   6563  CB  SER B  29      -1.017 -68.339  73.655  1.00251.72
C
ANISOU 6563  CB  SER B  29    52052  22245  21343 -12599  -6189
1569       C
ATOM   6564  OG  SER B  29      -0.486 -69.585  74.072  1.00265.07
O
ANISOU 6564  OG  SER B  29    55078  23054  22584 -12551  -6734
1641       O
ATOM   6565  N   ARG B  30      -0.545 -65.905  70.895  1.00226.41
N
```

```
ANISOU 6565  N   ARG B  30    45921  20632  19473 -11116  -5665
1096       N
ATOM   6566  CA  ARG B  30      -1.190 -64.693  70.403  1.00215.21
C
ANISOU 6566  CA  ARG B  30    43277  20043  18449 -11212  -5153
1012       C
ATOM   6567  C   ARG B  30      -0.550 -64.272  69.089  1.00208.14
C
ANISOU 6567  C   ARG B  30    41734  19388  17962 -10391  -5244
802       C
ATOM   6568  O   ARG B  30       0.583 -64.646  68.773  1.00209.00
O
ANISOU 6568  O   ARG B  30    42182  19134  18094  -9637  -5650
719       O
ATOM   6569  CB  ARG B  30      -1.106 -63.534  71.413  1.00219.17
C
ANISOU 6569  CB  ARG B  30    43193  21017  19066 -11208  -4797
1087       C
ATOM   6570  CG  ARG B  30      -1.926 -63.697  72.691  1.00232.34
C
ANISOU 6570  CG  ARG B  30    45264  22635  20379 -12098  -4579
1282       C
ATOM   6571  CD  ARG B  30      -1.910 -62.438  73.543  1.00210.58
C
ANISOU 6571  CD  ARG B  30    41785  20436  17789 -12059  -4195
1332       C
ATOM   6572  NE  ARG B  30      -2.720 -62.586  74.749  1.00210.43
N
ANISOU 6572  NE  ARG B  30    42126  20403  17426 -12921  -3973
1507       N
ATOM   6573  CZ  ARG B  30      -2.290 -63.146  75.875  1.00214.02
C
ANISOU 6573  CZ  ARG B  30    43460  20330  17529 -13041  -4188
1668       C
ATOM   6574  NH1 ARG B  30      -3.086 -63.204  76.934  1.00217.12
N
ANISOU 6574  NH1 ARG B  30    44106  20774  17617 -13861  -3942
1819       N
ATOM   6575  NH2 ARG B  30      -1.056 -63.626  75.950  1.00214.58
N
ANISOU 6575  NH2 ARG B  30    44145  19840  17544 -12329  -4651
1663       N
ATOM   6576  N   GLU B  31      -1.304 -63.479  68.332  1.00212.34
N
ANISOU 6576  N   GLU B  31    41325  20554  18802 -10553  -4859
702       N
ATOM   6577  CA  GLU B  31      -0.887 -62.934  67.048  1.00209.90
C
ANISOU 6577  CA  GLU B  31    40289  20570  18892  -9883  -4857
502       C
ATOM   6578  C   GLU B  31      -0.384 -61.503  67.173  1.00205.83
C
ANISOU 6578  C   GLU B  31    38828  20656  18723  -9381  -4570
441       C
```

```
ATOM   6579  O   GLU B  31      -0.180 -60.833  66.157  1.00187.68
O
ANISOU 6579  O   GLU B  31    35799  18744  16768  -8917  -4473
278       O
ATOM   6580  CB  GLU B  31      -2.039 -63.005  66.044  1.00209.24
C
ANISOU 6580  CB  GLU B  31    39814  20770  18917 -10356  -4649
413       C
ATOM   6581  CG  GLU B  31      -2.468 -64.422  65.699  1.00229.76
C
ANISOU 6581  CG  GLU B  31    43313  22778  21207 -10778  -4962
446       C
ATOM   6582  CD  GLU B  31      -3.548 -64.464  64.635  1.00244.49
C
ANISOU 6582  CD  GLU B  31    44747  24943  23206 -11186  -4767
336       C
ATOM   6583  OE1 GLU B  31      -4.672 -64.914  64.942  1.00252.53
O
ANISOU 6583  OE1 GLU B  31    46011  25955  23984 -12062  -4610
409       O
ATOM   6584  OE2 GLU B  31      -3.272 -64.048  63.490  1.00238.70
O
ANISOU 6584  OE2 GLU B  31    43430  24460  22807 -10638  -4771
165       O
ATOM   6585  N   GLY B  32      -0.190 -61.028  68.397  1.00210.96
N
ANISOU 6585  N   GLY B  32    39503  21379  19274  -9481  -4438
570       N
ATOM   6586  CA  GLY B  32       0.400 -59.734  68.657  1.00212.53
C
ANISOU 6586  CA  GLY B  32    38929  22070  19754  -8992  -4215
532       C
ATOM   6587  C   GLY B  32      -0.591 -58.616  68.888  1.00229.48
C
ANISOU 6587  C   GLY B  32    40234  24905  22052  -9449  -3695
547       C
ATOM   6588  O   GLY B  32      -0.210 -57.444  68.788  1.00209.13
O
ANISOU 6588  O   GLY B  32    36890  22810  19761  -9021  -3490
479       O
ATOM   6589  N   LEU B  33      -1.847 -58.942  69.187  1.00244.77
N
ANISOU 6589  N   LEU B  33    42297  26911  23792 -10303  -3487
621       N
ATOM   6590  CA  LEU B  33      -2.925 -57.974  69.325  1.00235.19
C
ANISOU 6590  CA  LEU B  33    40291  26375  22695 -10779  -3008
597       C
ATOM   6591  C   LEU B  33      -3.285 -57.735  70.786  1.00234.00
C
ANISOU 6591  C   LEU B  33    40299  26302  22308 -11289  -2802
769       C
ATOM   6592  O   LEU B  33      -4.228 -56.988  71.068  1.00240.69
O
```

```
ANISOU 6592  O   LEU B  33    40550  27706  23195 -11741  -2410
752       O
ATOM   6593  CB  LEU B  33      -4.159 -58.436  68.542  1.00232.50
C
ANISOU 6593  CB  LEU B  33    39860  26165  22315 -11387  -2885
512       C
ATOM   6594  CG  LEU B  33      -3.908 -58.808  67.077  1.00205.12
C
ANISOU 6594  CG  LEU B  33    36314  22573  19048 -10955  -3102
349       C
ATOM   6595  CD1 LEU B  33      -5.189 -59.286  66.407  1.00188.19
C
ANISOU 6595  CD1 LEU B  33    34109  20555  16841 -11616  -2974
270       C
ATOM   6596  CD2 LEU B  33      -3.304 -57.636  66.314  1.00179.61
C
ANISOU 6596  CD2 LEU B  33    32215  19800  16229 -10207  -2992
197       C
ATOM   6597  N   THR B  34      -2.547 -58.366  71.705  1.00236.78
N
ANISOU 6597  N   THR B  34    41456  26105  22406 -11205  -3075
918       N
ATOM   6598  CA  THR B  34      -2.791 -58.471  73.144  1.00238.37
C
ANISOU 6598  CA  THR B  34    42088  26185  22299 -11716  -2977
1106       C
ATOM   6599  C   THR B  34      -3.592 -57.332  73.767  1.00237.07
C
ANISOU 6599  C   THR B  34    41153  26710  22212 -12100  -2494
1123       C
ATOM   6600  O   THR B  34      -3.226 -56.159  73.639  1.00220.25
O
ANISOU 6600  O   THR B  34    38247  25050  20389 -11607  -2319
1052       O
ATOM   6601  CB  THR B  34      -1.455 -58.598  73.881  1.00224.87
C
ANISOU 6601  CB  THR B  34    40893  24031  20518 -11115  -3289
1196       C
ATOM   6602  OG1 THR B  34      -0.608 -59.517  73.180  1.00222.73
O
ANISOU 6602  OG1 THR B  34    41211  23196  20219 -10607  -3753
1128       O
ATOM   6603  CG2 THR B  34      -1.682 -59.114  75.293  1.00196.15
C
ANISOU 6603  CG2 THR B  34    38006  20049  16474 -11687  -3303
1400       C
ATOM   6604  N   THR B  35      -4.684 -57.675  74.448  1.00242.44
N
ANISOU 6604  N   THR B  35    42061  27459  22598 -12988  -2284
1209       N
ATOM   6605  CA  THR B  35      -5.507 -56.694  75.136  1.00240.12
C
ANISOU 6605  CA  THR B  35    41103  27810  22320 -13410  -1839
1217       C
```

```
ATOM   6606  C   THR B  35      -4.869 -56.290  76.466  1.00223.19
C
ANISOU 6606  C   THR B  35    39166  25570  20066 -13265  -1828
1383       C
ATOM   6607  O   THR B  35      -4.030 -57.001  77.027  1.00221.27
O
ANISOU 6607  O   THR B  35    39744  24701  19628 -13072  -2153
1512       O
ATOM   6608  CB  THR B  35      -6.916 -57.240  75.373  1.00256.53
C
ANISOU 6608  CB  THR B  35    43336  30033  24100 -14439  -1614
1223       C
ATOM   6609  OG1 THR B  35      -7.794 -56.161  75.719  1.00263.08
O
ANISOU 6609  OG1 THR B  35    43309  31630  25017 -14755  -1167
1151       O
ATOM   6610  CG2 THR B  35      -6.916 -58.278  76.492  1.00265.73
C
ANISOU 6610  CG2 THR B  35    45558  30609  24798 -15005  -1769
1430       C
ATOM   6611  N   GLN B  36      -5.265 -55.108  76.951  1.00212.46
N
ANISOU 6611  N   GLN B  36    37041  24846  18836 -13323  -1463
1364       N
ATOM   6612  CA  GLN B  36      -4.581 -54.479  78.081  1.00204.14
C
ANISOU 6612  CA  GLN B  36    35999  23798  17766 -13044  -1431
1497       C
ATOM   6613  C   GLN B  36      -4.608 -55.346  79.336  1.00208.61
C
ANISOU 6613  C   GLN B  36    37506  23858  17899 -13573  -1537
1702       C
ATOM   6614  O   GLN B  36      -3.597 -55.457  80.040  1.00191.82
O
ANISOU 6614  O   GLN B  36    35857  21320  15705 -13167  -1766
1823       O
ATOM   6615  CB  GLN B  36      -5.199 -53.110  78.374  1.00208.24
C
ANISOU 6615  CB  GLN B  36    35554  25111  18457 -13124  -1006
1434       C
ATOM   6616  CG  GLN B  36      -4.792 -51.992  77.413  1.00228.93
C
ANISOU 6616  CG  GLN B  36    37288  28184  21512 -12400   -939
1267       C
ATOM   6617  CD  GLN B  36      -5.067 -52.318  75.955  1.00237.90
C
ANISOU 6617  CD  GLN B  36    38216  29353  22824 -12293  -1019
1088       C
ATOM   6618  OE1 GLN B  36      -6.192 -52.179  75.476  1.00230.27
O
ANISOU 6618  OE1 GLN B  36    36818  28808  21867 -12749   -784
961       O
ATOM   6619  NE2 GLN B  36      -4.033 -52.751  75.241  1.00237.15
N
```

```
ANISOU 6619  NE2 GLN B  36    38417  28827  22862 -11676  -1357
1061       N
ATOM   6620  N   GLU B  37      -5.753 -55.965  79.639  1.00232.00
N
ANISOU 6620  N   GLU B  37    40755  26847  20547 -14487  -1374
1736       N
ATOM   6621  CA  GLU B  37      -5.792 -56.947  80.721  1.00232.94
C
ANISOU 6621  CA  GLU B  37    41894  26404  20211 -15041  -1511
1929       C
ATOM   6622  C   GLU B  37      -4.857 -58.120  80.451  1.00240.26
C
ANISOU 6622  C   GLU B  37    43813  26476  20998 -14698  -2022
1990       C
ATOM   6623  O   GLU B  37      -4.159 -58.585  81.359  1.00224.04
O
ANISOU 6623  O   GLU B  37    42537  23882  18704 -14600  -2263
2143       O
ATOM   6624  CB  GLU B  37      -7.221 -57.442  80.946  1.00214.09
C
ANISOU 6624  CB  GLU B  37    39619  24223  17502 -16124  -1242
1927       C
ATOM   6625  CG  GLU B  37      -8.154 -56.396  81.537  1.00205.68
C
ANISOU 6625  CG  GLU B  37    37730  23955  16463 -16549   -755
1881       C
ATOM   6626  CD  GLU B  37      -9.522 -56.958  81.872  1.00211.16
C
ANISOU 6626  CD  GLU B  37    38482  24938  16810 -17404   -530
1798       C
ATOM   6627  OE1 GLU B  37      -9.767 -58.147  81.577  1.00215.88
O
ANISOU 6627  OE1 GLU B  37    39753  25120  17152 -17704   -736
1793       O
ATOM   6628  OE2 GLU B  37     -10.347 -56.212  82.440  1.00210.92
O
ANISOU 6628  OE2 GLU B  37    37766  25611  16763 -17607   -177
1686       O
ATOM   6629  N   GLY B  38      -4.819 -58.601  79.208  1.00244.73
N
ANISOU 6629  N   GLY B  38    44374  26907  21704 -14484  -2209
1860       N
ATOM   6630  CA  GLY B  38      -3.861 -59.636  78.855  1.00228.11
C
ANISOU 6630  CA  GLY B  38    43140  24033  19498 -14040  -2719
1884       C
ATOM   6631  C   GLY B  38      -2.420 -59.190  79.014  1.00238.07
C
ANISOU 6631  C   GLY B  38    44375  25109  20970 -13057  -2973
1883       C
ATOM   6632  O   GLY B  38      -1.581 -59.941  79.515  1.00242.63
O
ANISOU 6632  O   GLY B  38    45837  25028  21325 -12803  -3358
1970       O
```

```
ATOM   6633  N   GLU B  39      -2.117 -57.958  78.598  1.00238.65
N
ANISOU 6633  N   GLU B  39    43448  25767  21460 -12498  -2767
1771       N
ATOM   6634  CA  GLU B  39      -0.791 -57.389  78.822  1.00239.64
C
ANISOU 6634  CA  GLU B  39    43444  25819  21788 -11615  -2948
1761       C
ATOM   6635  C   GLU B  39      -0.439 -57.337  80.305  1.00220.53
C
ANISOU 6635  C   GLU B  39    41496  23173  19124 -11723  -2965
1941       C
ATOM   6636  O   GLU B  39       0.671 -57.708  80.703  1.00221.85
O
ANISOU 6636  O   GLU B  39    42236  22845  19213 -11192  -3323
1977       O
ATOM   6637  CB  GLU B  39      -0.718 -55.983  78.224  1.00252.79
C
ANISOU 6637  CB  GLU B  39    43933  28215  23900 -11156  -2655
1627       C
ATOM   6638  CG  GLU B  39      -0.769 -55.922  76.713  1.00261.53
C
ANISOU 6638  CG  GLU B  39    44557  29518  25294 -10839  -2689
1433       C
ATOM   6639  CD  GLU B  39      -0.598 -54.509  76.194  1.00262.72
C
ANISOU 6639  CD  GLU B  39    43627  30341  25852 -10358  -2427
1309       C
ATOM   6640  OE1 GLU B  39      -1.518 -53.688  76.392  1.00269.42
O
ANISOU 6640  OE1 GLU B  39    43840  31762  26765 -10756  -2035
1301       O
ATOM   6641  OE2 GLU B  39       0.457 -54.219  75.593  1.00267.67
O
ANISOU 6641  OE2 GLU B  39    44054  30931  26717  -9585  -2618
1208       O
ATOM   6642  N   ASP B  40      -1.372 -56.875  81.138  1.00208.70
N
ANISOU 6642  N   ASP B  40    39756  22046  17497 -12393  -2585
2041       N
ATOM   6643  CA  ASP B  40      -1.169 -56.917  82.583  1.00211.99
C
ANISOU 6643  CA  ASP B  40    40689  22224  17635 -12605  -2586
2224       C
ATOM   6644  C   ASP B  40      -1.027 -58.346  83.101  1.00233.00
C
ANISOU 6644  C   ASP B  40    44632  24058  19839 -12948  -2950
2347       C
ATOM   6645  O   ASP B  40      -0.150 -58.634  83.923  1.00249.46
O
ANISOU 6645  O   ASP B  40    47363  25662  21759 -12629  -3225
2444       O
ATOM   6646  CB  ASP B  40      -2.322 -56.199  83.284  1.00208.78
C
```

```
ANISOU 6646  CB  ASP B  40    39756  22414  17157 -13323  -2093
2285       C
ATOM   6647  CG  ASP B  40      -2.300 -54.700  83.051  1.00192.33
C
ANISOU 6647  CG  ASP B  40    36509  21087  15479 -12904  -1776
2187       C
ATOM   6648  OD1 ASP B  40      -1.216 -54.160  82.745  1.00187.65
O
ANISOU 6648  OD1 ASP B  40    35638  20491  15171 -12065  -1938
2129       O
ATOM   6649  OD2 ASP B  40      -3.368 -54.063  83.168  1.00191.43
O
ANISOU 6649  OD2 ASP B  40    35771  21580  15385 -13415  -1374
2155       O
ATOM   6650  N   ARG B  41      -1.900 -59.253  82.653  1.00234.00
N
ANISOU 6650  N   ARG B  41    45166  24005  19737 -13613  -2963
2342       N
ATOM   6651  CA  ARG B  41      -1.789 -60.649  83.070  1.00240.27
C
ANISOU 6651  CA  ARG B  41    47241  23980  20071 -13959  -3333
2455       C
ATOM   6652  C   ARG B  41      -0.471 -61.280  82.631  1.00246.75
C
ANISOU 6652  C   ARG B  41    48655  24171  20928 -13098  -3887
2392       C
ATOM   6653  O   ARG B  41       0.124 -62.068  83.375  1.00267.83
O
ANISOU 6653  O   ARG B  41    52339  26161  23264 -13033  -4246
2494       O
ATOM   6654  CB  ARG B  41      -2.974 -61.461  82.547  1.00221.98
C
ANISOU 6654  CB  ARG B  41    45189  21629  17524 -14823  -3239
2442       C
ATOM   6655  CG  ARG B  41      -4.302 -61.102  83.197  1.00220.85
C
ANISOU 6655  CG  ARG B  41    44707  22000  17206 -15803  -2743
2507       C
ATOM   6656  CD  ARG B  41      -4.292 -61.435  84.681  1.00225.89
C
ANISOU 6656  CD  ARG B  41    46021  22375  17434 -16135  -2762
2655       C
ATOM   6657  NE  ARG B  41      -4.226 -62.873  84.927  1.00231.49
N
ANISOU 6657  NE  ARG B  41    47846  22422  17687 -16306  -3148
2667       N
ATOM   6658  CZ  ARG B  41      -5.287 -63.671  84.986  1.00236.94
C
ANISOU 6658  CZ  ARG B  41    48762  23238  18028 -16968  -3056
2597       C
ATOM   6659  NH1 ARG B  41      -6.498 -63.185  84.755  1.00236.53
N
ANISOU 6659  NH1 ARG B  41    47872  23947  18053 -17491  -2602
2488       N
```

```
ATOM   6660  NH2 ARG B  41      -5.131 -64.966  85.224  1.00242.95
N
ANISOU 6660  NH2 ARG B  41    50590  23372  18349 -17080  -3435
2624       N
ATOM   6661  N   ILE B  42       0.007 -60.946  81.432  1.00228.90
N
ANISOU 6661  N   ILE B  42    45789  22129  19054 -12422  -3974
2211       N
ATOM   6662  CA  ILE B  42       1.256 -61.512  80.931  1.00222.10
C
ANISOU 6662  CA  ILE B  42    45405  20744  18240 -11574  -4492
2112       C
ATOM   6663  C   ILE B  42       2.430 -60.608  81.297  1.00206.55
C
ANISOU 6663  C   ILE B  42    42994  18949  16535 -10712  -4544
2064       C
ATOM   6664  O   ILE B  42       3.548 -60.781  80.796  1.00207.15
O
ANISOU 6664  O   ILE B  42    43180  18784  16745  -9887  -4911
1931       O
ATOM   6665  CB  ILE B  42       1.172 -61.754  79.409  1.00230.11
C
ANISOU 6665  CB  ILE B  42    46084  21857  19491 -11321  -4588
1931       C
ATOM   6666  CG1 ILE B  42       2.180 -62.819  78.967  1.00224.21
C
ANISOU 6666  CG1 ILE B  42    46190  20393  18608 -10722  -5188
1850       C
ATOM   6667  CG2 ILE B  42       1.374 -60.460  78.633  1.00213.34
C
ANISOU 6667  CG2 ILE B  42    42702  20471  17886 -10781  -4304
1780       C
ATOM   6668  CD1 ILE B  42       1.962 -63.316  77.555  1.00212.46
C
ANISOU 6668  CD1 ILE B  42    44595  18881  17250 -10622  -5320
1700       C
ATOM   6669  N   GLN B  43       2.200 -59.662  82.208  1.00204.31
N
ANISOU 6669  N   GLN B  43    42236  19082  16309 -10902  -4191
2165       N
ATOM   6670  CA  GLN B  43       3.261 -58.770  82.663  1.00200.23
C
ANISOU 6670  CA  GLN B  43    41313  18744  16022 -10153  -4217
2136       C
ATOM   6671  C   GLN B  43       4.112 -59.458  83.726  1.00215.00
C
ANISOU 6671  C   GLN B  43    44200  19928  17563  -9931  -4612
2230       C
ATOM   6672  O   GLN B  43       4.599 -58.813  84.661  1.00232.68
O
ANISOU 6672  O   GLN B  43    46311  22268  19830  -9701  -4542
2298       O
ATOM   6673  CB  GLN B  43       2.694 -57.448  83.184  1.00196.27
C
```

```
ANISOU 6673  CB  GLN B  43    39889  18965  15718 -10403  -3699
2199       C
ATOM   6674  CG  GLN B  43       3.671 -56.283  83.057  1.00190.44
C
ANISOU 6674  CG  GLN B  43    38359  18629  15369  -9567  -3652
2099       C
ATOM   6675  CD  GLN B  43       3.128 -54.985  83.619  1.00186.74
C
ANISOU 6675  CD  GLN B  43    37048  18838  15067  -9797  -3172
2166       C
ATOM   6676  OE1 GLN B  43       3.697 -54.416  84.550  1.00185.30
O
ANISOU 6676  OE1 GLN B  43    36822  18693  14892  -9539  -3150
2243       O
ATOM   6677  NE2 GLN B  43       2.029 -54.505  83.049  1.00185.25
N
ANISOU 6677  NE2 GLN B  43    36184  19194  15009 -10262  -2800
2125       N
ATOM   6678  N   ILE B  44       4.293 -60.777  83.588  1.00222.97
N
ANISOU 6678  N   ILE B  44    46249  20221  18246  -9989  -5046
2228       N
ATOM   6679  CA  ILE B  44       5.129 -61.548  84.506  1.00244.10
C
ANISOU 6679  CA  ILE B  44    49997  22173  20575  -9730  -5494
2289       C
ATOM   6680  C   ILE B  44       6.598 -61.470  84.136  1.00255.96
C
ANISOU 6680  C   ILE B  44    51452  23524  22277  -8626  -5896
2096       C
ATOM   6681  O   ILE B  44       7.442 -62.010  84.863  1.00273.52
O
ANISOU 6681  O   ILE B  44    54483  25190  24252  -8256  -6299
2100       O
ATOM   6682  CB  ILE B  44       4.692 -63.026  84.563  1.00231.24
C
ANISOU 6682  CB  ILE B  44    49596  19802  18461 -10274  -5821
2365       C
ATOM   6683  CG1 ILE B  44       4.672 -63.639  83.161  1.00225.80
C
ANISOU 6683  CG1 ILE B  44    48904  19010  17878 -10071  -6039
2202       C
ATOM   6684  CG2 ILE B  44       3.332 -63.151  85.236  1.00222.99
C
ANISOU 6684  CG2 ILE B  44    48744  18851  17130 -11415  -5441
2569       C
ATOM   6685  CD1 ILE B  44       4.571 -65.148  83.163  1.00227.18
C
ANISOU 6685  CD1 ILE B  44    50391  18352  17576 -10372  -6495
2243       C
ATOM   6686  N   PHE B  45       6.926 -60.815  83.026  1.00229.74
N
ANISOU 6686  N   PHE B  45    47213  20694  19382  -8094  -5803
1911       N
```

```
ATOM   6687  CA  PHE B  45       8.303 -60.532  82.652  1.00211.52
C
ANISOU 6687  CA  PHE B  45    44655  18404  17307  -7061  -6101
1704       C
ATOM   6688  C   PHE B  45       8.743 -59.162  83.151  1.00202.66
C
ANISOU 6688  C   PHE B  45    42649  17860  16490  -6723  -5793
1706       C
ATOM   6689  O   PHE B  45       9.879 -58.749  82.894  1.00201.07
O
ANISOU 6689  O   PHE B  45    42108  17783  16507  -5895  -5974
1530       O
ATOM   6690  CB  PHE B  45       8.462 -60.613  81.130  1.00216.11
C
ANISOU 6690  CB  PHE B  45    44767  19186  18160  -6671  -6184
1492       C
ATOM   6691  CG  PHE B  45       8.019 -61.924  80.542  1.00220.74
C
ANISOU 6691  CG  PHE B  45    46171  19231  18470  -6982  -6486
1482       C
ATOM   6692  CD1 PHE B  45       6.768 -62.044  79.959  1.00227.36
C
ANISOU 6692  CD1 PHE B  45    46799  20271  19318  -7723  -6188
1555       C
ATOM   6693  CD2 PHE B  45       8.848 -63.033  80.572  1.00230.99
C
ANISOU 6693  CD2 PHE B  45    48456  19824  19486  -6527  -7080
1387       C
ATOM   6694  CE1 PHE B  45       6.351 -63.245  79.417  1.00240.84
C
ANISOU 6694  CE1 PHE B  45    49263  21480  20764  -8031  -6464
1549       C
ATOM   6695  CE2 PHE B  45       8.436 -64.239  80.030  1.00242.75
C
ANISOU 6695  CE2 PHE B  45    50733  20798  20704  -6815  -7374
1382       C
ATOM   6696  CZ  PHE B  45       7.185 -64.344  79.452  1.00248.01
C
ANISOU 6696  CZ  PHE B  45    51179  21665  21388  -7582  -7059
1471       C
ATOM   6697  N   GLY B  46       7.856 -58.463  83.856  1.00211.03
N
ANISOU 6697  N   GLY B  46    43340  19287  17556  -7360  -5337
1891       N
ATOM   6698  CA  GLY B  46       8.113 -57.180  84.467  1.00218.07
C
ANISOU 6698  CA  GLY B  46    43471  20700  18684  -7161  -5025
1931       C
ATOM   6699  C   GLY B  46       8.122 -55.992  83.529  1.00230.28
C
ANISOU 6699  C   GLY B  46    43802  22998  20696  -6854  -4701
1801       C
ATOM   6700  O   GLY B  46       7.824 -56.099  82.334  1.00223.68
O
```

ANISOU 6700 O   GLY B 46   42624 22339 20026 -6837 -4668
1679   O
ATOM   6701 N   PRO B 47   8.474 -54.821 84.072 1.00240.19
N
ANISOU 6701 N   PRO B 47   44406 24697 22158 -6605 -4462
1826   N
ATOM   6702 CA PRO B 47   8.333 -53.577 83.308 1.00251.75
C
ANISOU 6702 CA PRO B 47   44723 26901 24028 -6427 -4102
1733   C
ATOM   6703 C   PRO B 47   9.649 -53.013 82.793 1.00268.96
C
ANISOU 6703 C   PRO B 47   46441 29264 26487 -5519 -4273
1532   C
ATOM   6704 O   PRO B 47   9.737 -51.803 82.562 1.00278.03
O
ANISOU 6704 O   PRO B 47   46703 31003 27933 -5326 -3980
1490   O
ATOM   6705 CB PRO B 47   7.703 -52.630 84.335 1.00233.14
C
ANISOU 6705 CB PRO B 47   41980 24932 21672 -6863 -3694
1913   C
ATOM   6706 CG PRO B 47   8.378 -53.047 85.602 1.00216.53
C
ANISOU 6706 CG PRO B 47   40630 22336 19304 -6724 -3952
2018   C
ATOM   6707 CD PRO B 47   8.445 -54.561 85.523 1.00216.15
C
ANISOU 6707 CD PRO B 47   41652 21553 18922 -6842 -4370
2012   C
ATOM   6708 N   ASN B 48   10.680 -53.852 82.648 1.00264.69
N
ANISOU 6708 N   ASN B 48   46495 28238 25836 -4961 -4748
1397   N
ATOM   6709 CA ASN B 48   11.906 -53.475 81.943 1.00239.05
C
ANISOU 6709 CA ASN B 48   42815 25177 22838 -4111 -4937
1154   C
ATOM   6710 C   ASN B 48   12.732 -52.447 82.718 1.00242.25
C
ANISOU 6710 C   ASN B 48   42793 25875 23378 -3690 -4848
1150   C
ATOM   6711 O   ASN B 48   13.845 -52.107 82.301 1.00236.12
O
ANISOU 6711 O   ASN B 48   41682 25257 22775 -2985 -5010
944   O
ATOM   6712 CB ASN B 48   11.588 -52.943 80.538 1.00222.96
C
ANISOU 6712 CB ASN B 48   39960 23640 21114 -4055 -4713
1018   C
ATOM   6713 CG ASN B 48   10.840 -53.951 79.680 1.00222.19
C
ANISOU 6713 CG ASN B 48   40250 23268 20906 -4413 -4820
999   C

```
ATOM   6714  OD1 ASN B  48      11.428 -54.607  78.822  1.00228.54
O
ANISOU 6714  OD1 ASN B  48    41259  23854  21722  -3977  -5142
813       O
ATOM   6715  ND2 ASN B  48       9.533 -54.063  79.896  1.00213.31
N
ANISOU 6715  ND2 ASN B  48    39202  22177  19667  -5209  -4545
1177       N
ATOM   6716  N   LYS B  49      12.196 -51.948  83.840  1.00261.69
N
ANISOU 6716  N   LYS B  49    45255  28423  25752  -4128  -4589
1369       N
ATOM   6717  CA  LYS B  49      12.576 -50.629  84.347  1.00257.91
C
ANISOU 6717  CA  LYS B  49    44068  28440  25487  -3904  -4328
1395       C
ATOM   6718  C   LYS B  49      14.052 -50.539  84.724  1.00275.77
C
ANISOU 6718  C   LYS B  49    46433  30574  27773  -3123  -4647
1245       C
ATOM   6719  O   LYS B  49      14.702 -49.527  84.435  1.00278.40
O
ANISOU 6719  O   LYS B  49    46028  31380  28373  -2689  -4522
1129       O
ATOM   6720  CB  LYS B  49      11.709 -50.255  85.550  1.00241.17
C
ANISOU 6720  CB  LYS B  49    42041  26373  23221  -4529  -4033
1657       C
ATOM   6721  CG  LYS B  49      10.299 -49.811  85.198  1.00217.71
C
ANISOU 6721  CG  LYS B  49    38591  23811  20319  -5229  -3596
1768       C
ATOM   6722  CD  LYS B  49      10.316 -48.462  84.486  1.00178.44
C
ANISOU 6722  CD  LYS B  49    32542  19546  15711  -5000  -3279
1675       C
ATOM   6723  CE  LYS B  49       8.909 -47.945  84.237  1.00166.89
C
ANISOU 6723  CE  LYS B  49    30591  18517  14302  -5662  -2854
1766       C
ATOM   6724  NZ  LYS B  49       8.309 -47.365  85.470  1.00156.92
N
ANISOU 6724  NZ  LYS B  49    29276  17422  12924  -6092  -2584
1966       N
ATOM   6725  N   LEU B  50      14.597 -51.572  85.375  1.00278.17
N
ANISOU 6725  N   LEU B  50    47652  30251  27788  -2938  -5065
1233       N
ATOM   6726  CA  LEU B  50      15.872 -51.472  86.098  1.00260.72
C
ANISOU 6726  CA  LEU B  50    45629  27892  25541  -2290  -5352
1126       C
ATOM   6727  C   LEU B  50      15.773 -50.430  87.220  1.00253.05
C
```

ANISOU 6727 C   LEU B 50   44321 27203 24622 -2452 -5051
1307   C
ATOM   6728 O   LEU B 50   16.449 -49.399 87.224 1.00258.53
O
ANISOU 6728 O   LEU B 50   44333 28343 25553 -2044 -4927
1221   O
ATOM   6729 CB LEU B 50   17.041 -51.186 85.147 1.00245.66
C
ANISOU 6729 CB LEU B 50   43210 26259 23869 -1501 -5533
813   C
ATOM   6730 CG LEU B 50   17.153 -52.145 83.958 1.00231.64
C
ANISOU 6730 CG LEU B 50   41690 24260 22062 -1304 -5818
617   C
ATOM   6731 CD1 LEU B 50   18.201 -51.661 82.970 1.00216.33
C
ANISOU 6731 CD1 LEU B 50   39079 22730 20386   -597 -5899
310   C
ATOM   6732 CD2 LEU B 50   17.466 -53.557 84.433 1.00231.10
C
ANISOU 6732 CD2 LEU B 50   42760 23424 21625 -1178 -6331
584   C
ATOM   6733 N   GLU B 51   14.897 -50.742 88.181 1.00243.37
N
ANISOU 6733 N   GLU B 51   43616 25704 23150 -3085 -4938
1561   N
ATOM   6734 CA GLU B 51   14.166 -49.746 88.965 1.00239.68
C
ANISOU 6734 CA GLU B 51   42711 25616 22742 -3553 -4499
1783   C
ATOM   6735 C   GLU B 51   15.065 -48.761 89.713 1.00251.22
C
ANISOU 6735 C   GLU B 51   43783 27330 24339 -3082 -4464
1766   C
ATOM   6736 O   GLU B 51   14.619 -47.646 90.012 1.00255.39
O
ANISOU 6736 O   GLU B 51   43663 28348 25026 -3308 -4075
1886   O
ATOM   6737 CB GLU B 51   13.240 -50.463 89.948 1.00229.80
C
ANISOU 6737 CB GLU B 51   42246 23932 21136 -4260 -4470
2027   C
ATOM   6738 CG GLU B 51   12.115 -51.230 89.271 1.00225.98
C
ANISOU 6738 CG GLU B 51   42015 23319 20526 -4889 -4390
2079   C
ATOM   6739 CD GLU B 51   11.361 -52.127 90.230 1.00233.69
C
ANISOU 6739 CD GLU B 51   43916 23781 21093 -5558 -4439
2289   C
ATOM   6740 OE1 GLU B 51   11.407 -53.362 90.046 1.00236.22
O
ANISOU 6740 OE1 GLU B 51   45081 23518 21153 -5619 -4787
2255   O

```
ATOM   6741  OE2 GLU B  51      10.725 -51.600  91.166  1.00233.73
O
ANISOU 6741  OE2 GLU B  51    43821  23964  21021  -6030  -4134
2484       O
ATOM   6742  N   GLU B  52      16.288 -49.168  90.084  1.00255.11
N
ANISOU 6742  N   GLU B  52    44695  27486  24750  -2447  -4872
1619       N
ATOM   6743  CA  GLU B  52      17.277 -48.307  90.745  1.00245.31
C
ANISOU 6743  CA  GLU B  52    43108  26466  23634  -1931  -4889
1563       C
ATOM   6744  C   GLU B  52      17.022 -48.178  92.246  1.00239.33
C
ANISOU 6744  C   GLU B  52    42771  25488  22674  -2228  -4820
1801       C
ATOM   6745  O   GLU B  52      17.958 -48.274  93.047  1.00251.30
O
ANISOU 6745  O   GLU B  52    44636  26752  24096  -1777  -5092
1744       O
ATOM   6746  CB  GLU B  52      17.348 -46.909  90.114  1.00242.26
C
ANISOU 6746  CB  GLU B  52    41601  26833  23614  -1796  -4522
1506       C
ATOM   6747  CG  GLU B  52      18.294 -45.961  90.840  1.00232.33
C
ANISOU 6747  CG  GLU B  52    39975  25825  22475  -1338  -4509
1469       C
ATOM   6748  CD  GLU B  52      18.380 -44.596  90.187  1.00224.61
C
ANISOU 6748  CD  GLU B  52    37950  25564  21829  -1220  -4167
1411       C
ATOM   6749  OE1 GLU B  52      19.438 -44.279  89.603  1.00221.45
O
ANISOU 6749  OE1 GLU B  52    37164  25388  21591   -630  -4304
1167       O
ATOM   6750  OE2 GLU B  52      17.390 -43.837  90.261  1.00222.21
O
ANISOU 6750  OE2 GLU B  52    37220  25604  21606  -1721  -3769
1598       O
ATOM   6751  N   LYS B  53      15.768 -47.946  92.633  1.00218.01
N
ANISOU 6751  N   LYS B  53    40026  22905  19903  -2974  -4459
2053       N
ATOM   6752  CA  LYS B  53      15.378 -47.992  94.039  1.00209.54
C
ANISOU 6752  CA  LYS B  53    39455  21574  18586  -3356  -4393
2290       C
ATOM   6753  C   LYS B  53      15.789 -49.316  94.673  1.00214.79
C
ANISOU 6753  C   LYS B  53    41277  21449  18885  -3264  -4856
2283       C
ATOM   6754  O   LYS B  53      15.662 -50.382  94.065  1.00207.45
O
```

```
ANISOU 6754  O   LYS B  53    40887  20133  17800  -3330  -5106
2204       O
ATOM   6755  CB  LYS B  53      13.870 -47.778  94.183  1.00195.61
C
ANISOU 6755  CB  LYS B  53    37535  20033  16755  -4226  -3965
2520       C
ATOM   6756  CG  LYS B  53      13.411 -46.380  93.793  1.00177.64
C
ANISOU 6756  CG  LYS B  53    34172  18526  14798  -4326  -3511
2545       C
ATOM   6757  CD  LYS B  53      11.905 -46.221  93.936  1.00180.14
C
ANISOU 6757  CD  LYS B  53    34342  19079  15024  -5165  -3112
2734       C
ATOM   6758  CE  LYS B  53      11.426 -44.907  93.335  1.00169.24
C
ANISOU 6758  CE  LYS B  53    31896  18454  13955  -5216  -2708
2712       C
ATOM   6759  NZ  LYS B  53      11.822 -43.736  94.164  1.00172.84
N
ANISOU 6759  NZ  LYS B  53    31908  19236  14528  -4982  -2550
2787       N
ATOM   6760  N   LYS B  54      16.285 -49.242  95.909  1.00206.27
N
ANISOU 6760  N   LYS B  54    40604  20113  17655  -3104  -4984
2365       N
ATOM   6761  CA  LYS B  54      16.944 -50.380  96.534  1.00206.97
C
ANISOU 6761  CA  LYS B  54    41768  19458  17413  -2833  -5486
2310       C
ATOM   6762  C   LYS B  54      16.809 -50.301  98.051  1.00211.57
C
ANISOU 6762  C   LYS B  54    42865  19767  17757  -3084  -5454
2529       C
ATOM   6763  O   LYS B  54      16.599 -49.232  98.629  1.00221.86
O
ANISOU 6763  O   LYS B  54    43588  21499  19210  -3216  -5109
2664       O
ATOM   6764  CB  LYS B  54      18.426 -50.421  96.128  1.00202.83
C
ANISOU 6764  CB  LYS B  54    41140  18901  17024  -1875  -5888
1993       C
ATOM   6765  CG  LYS B  54      19.215 -51.644  96.575  1.00200.96
C
ANISOU 6765  CG  LYS B  54    41992  17910  16456  -1462  -6478
1860       C
ATOM   6766  CD  LYS B  54      18.664 -52.935  95.997  1.00187.52
C
ANISOU 6766  CD  LYS B  54    41053  15705  14490  -1783  -6708
1853       C
ATOM   6767  CE  LYS B  54      19.595 -54.100  96.299  1.00192.73
C
ANISOU 6767  CE  LYS B  54    42749  15645  14837  -1233  -7350
1663       C
```

```
ATOM   6768  NZ  LYS B  54      18.922 -55.414  96.115  1.00197.98
N
ANISOU 6768  NZ  LYS B  54    44402  15685  15138  -1689  -7580
1738       N
ATOM   6769  N   GLU B  55      16.936 -51.474  98.681  1.00223.44
N
ANISOU 6769  N   GLU B  55    45506  20525  18866  -3144  -5838
2559       N
ATOM   6770  CA  GLU B  55      16.758 -51.714 100.112  1.00231.88
C
ANISOU 6770  CA  GLU B  55    47325  21167  19613  -3444  -5882
2768       C
ATOM   6771  C   GLU B  55      17.894 -51.184 100.980  1.00240.08
C
ANISOU 6771  C   GLU B  55    48326  22178  20714  -2772  -6071
2691       C
ATOM   6772  O   GLU B  55      17.719 -51.088 102.199  1.00225.49
O
ANISOU 6772  O   GLU B  55    46896  20114  18667  -3017  -6018
2882       O
ATOM   6773  CB  GLU B  55      16.589 -53.216 100.352  1.00213.30
C
ANISOU 6773  CB  GLU B  55    46262  17985  16799  -3685  -6284
2792       C
ATOM   6774  CG  GLU B  55      17.844 -54.026 100.061  1.00197.72
C
ANISOU 6774  CG  GLU B  55    44862  15528  14733  -2849  -6903
2501       C
ATOM   6775  CD  GLU B  55      17.553 -55.499  99.838  1.00203.77
C
ANISOU 6775  CD  GLU B  55    46755  15571  15098  -3099  -7281
2488       C
ATOM   6776  OE1 GLU B  55      17.652 -55.957  98.681  1.00211.52
O
ANISOU 6776  OE1 GLU B  55    47631  16571  16165  -2904  -7437
2308       O
ATOM   6777  OE2 GLU B  55      17.224 -56.197 100.820  1.00208.36
O
ANISOU 6777  OE2 GLU B  55    48142  15624  15402  -3490  -7360
2552       O
ATOM   6778  N   SER B  56      19.039 -50.839 100.399  1.00250.49
N
ANISOU 6778  N   SER B  56    49161  23721  22292  -1952  -6284
2411       N
ATOM   6779  CA  SER B  56      20.146 -50.269 101.157  1.00245.18
C
ANISOU 6779  CA  SER B  56    48360  23093  21704  -1299  -6449
2310       C
ATOM   6780  C   SER B  56      20.134 -48.747 101.187  1.00238.60
C
ANISOU 6780  C   SER B  56    46387  23025  21245  -1267  -5999
2372       C
ATOM   6781  O   SER B  56      20.935 -48.152 101.916  1.00241.44
O
```

```
ANISOU 6781  O   SER B  56    46590  23468  21678   -820  -6071
2328       O
ATOM   6782  CB  SER B  56      21.484 -50.754 100.588  1.00248.41
C
ANISOU 6782  CB  SER B  56    48900  23330  22153   -396  -6960
1936       C
ATOM   6783  OG  SER B  56      21.634 -50.366  99.234  1.00260.74
O
ANISOU 6783  OG  SER B  56    49616  25416  24038   -175  -6837
1746       O
ATOM   6784  N   LYS B  57      19.250 -48.107 100.424  1.00235.01
N
ANISOU 6784  N   LYS B  57    45163  23117  21014  -1720  -5555
2465       N
ATOM   6785  CA  LYS B  57      19.166 -46.654 100.341  1.00222.66
C
ANISOU 6785  CA  LYS B  57    42526  22284  19792  -1711  -5132
2518       C
ATOM   6786  C   LYS B  57      17.813 -46.149 100.835  1.00227.44
C
ANISOU 6786  C   LYS B  57    42940  23128  20349  -2532  -4655
2830       C
ATOM   6787  O   LYS B  57      17.326 -45.110 100.385  1.00219.72
O
ANISOU 6787  O   LYS B  57    41070  22780  19634  -2714  -4257
2878       O
ATOM   6788  CB  LYS B  57      19.434 -46.176  98.916  1.00198.01
C
ANISOU 6788  CB  LYS B  57    38547  19684  17004  -1414  -5037
2303       C
ATOM   6789  CG  LYS B  57      20.869 -46.377  98.463  1.00184.11
C
ANISOU 6789  CG  LYS B  57    36750  17865  15339   -549  -5446
1967       C
ATOM   6790  CD  LYS B  57      21.128 -45.686  97.139  1.00161.07
C
ANISOU 6790  CD  LYS B  57    32880  15554  12764   -295  -5281
1773       C
ATOM   6791  CE  LYS B  57      22.600 -45.741  96.774  1.00161.20
C
ANISOU 6791  CE  LYS B  57    32761  15610  12879    561  -5648
1424       C
ATOM   6792  NZ  LYS B  57      22.877 -45.029  95.498  1.00166.00
N
ANISOU 6792  NZ  LYS B  57    32439  16828  13805    783  -5471
1233       N
ATOM   6793  N   LEU B  58      17.194 -46.894 101.754  1.00221.96
N
ANISOU 6793  N   LEU B  58    43099  21936  19300  -3034  -4702
3028       N
ATOM   6794  CA  LEU B  58      16.023 -46.435 102.494  1.00193.39
C
ANISOU 6794  CA  LEU B  58    39385  18513  15582  -3781  -4281
3314       C
```

```
ATOM   6795  C   LEU B  58      16.197 -45.045 103.102  1.00188.74
C
ANISOU 6795  C   LEU B  58    38048  18446  15219  -3623  -3995
3398       C
ATOM   6796  O   LEU B  58      17.316 -44.534 103.218  1.00206.26
O
ANISOU 6796  O   LEU B  58    39999  20757  17613  -2942  -4168
3260       O
ATOM   6797  CB  LEU B  58      15.675 -47.442 103.595  1.00176.29
C
ANISOU 6797  CB  LEU B  58    38352  15664  12967  -4198  -4457
3485       C
ATOM   6798  CG  LEU B  58      15.397 -48.875 103.138  1.00181.47
C
ANISOU 6798  CG  LEU B  58    39890  15733  13328  -4452  -4747
3438       C
ATOM   6799  CD1 LEU B  58      15.229 -49.795 104.335  1.00186.97
C
ANISOU 6799  CD1 LEU B  58    41647  15753  13640  -4770  -4934
3537       C
ATOM   6800  CD2 LEU B  58      14.170 -48.929 102.245  1.00181.01
C
ANISOU 6800  CD2 LEU B  58    39453  16006  13315  -5125  -4404
3498       C
ATOM   6801  N   LEU B  59      15.072 -44.445 103.501  1.00183.27
N
ANISOU 6801  N   LEU B  59    37027  18103  14504  -4266  -3562
3615       N
ATOM   6802  CA  LEU B  59      15.038 -43.066 103.984  1.00194.69
C
ANISOU 6802  CA  LEU B  59    37707  20106  16162  -4198  -3246
3707       C
ATOM   6803  C   LEU B  59      15.971 -42.822 105.167  1.00211.95
C
ANISOU 6803  C   LEU B  59    40204  22041  18287  -3755  -3449
3744       C
ATOM   6804  O   LEU B  59      16.523 -41.723 105.301  1.00207.04
O
ANISOU 6804  O   LEU B  59    38925  21827  17912  -3357  -3349
3713       O
ATOM   6805  CB  LEU B  59      13.601 -42.694 104.361  1.00187.98
C
ANISOU 6805  CB  LEU B  59    36644  19573  15205  -5008  -2801
3929       C
ATOM   6806  CG  LEU B  59      13.333 -41.285 104.893  1.00177.88
C
ANISOU 6806  CG  LEU B  59    34603  18884  14098  -5041  -2446
4043       C
ATOM   6807  CD1 LEU B  59      13.753 -40.236 103.874  1.00176.25
C
ANISOU 6807  CD1 LEU B  59    33417  19264  14287  -4590  -2336
3884       C
ATOM   6808  CD2 LEU B  59      11.863 -41.127 105.265  1.00174.90
C
```

ANISOU 6808 CD2 LEU B 59   34123 18777 13555 -5867 -2047
4229   C
ATOM   6809 N   LYS B 60   16.158 -43.823 106.030 1.00223.13
N
ANISOU 6809 N   LYS B 60   42633 22782 19365 -3821 -3740
3808   N
ATOM   6810 CA LYS B 60   17.122 -43.705 107.123 1.00218.96
C
ANISOU 6810 CA LYS B 60   42479 21953 18764 -3342 -3990
3814   C
ATOM   6811 C   LYS B 60   18.544 -43.446 106.627 1.00198.41
C
ANISOU 6811 C   LYS B 60   39560 19416 16409 -2444 -4300
3538   C
ATOM   6812 O   LYS B 60   19.253 -42.601 107.187 1.00185.09
O
ANISOU 6812 O   LYS B 60   37521 17934 14871 -2021 -4299
3522   O
ATOM   6813 CB LYS B 60   17.084 -44.969 107.985 1.00242.94
C
ANISOU 6813 CB LYS B 60   46511 24258 21537 -3520 -4243
3773   C
ATOM   6814 CG LYS B 60   18.110 -44.998 109.112 1.00260.36
C
ANISOU 6814 CG LYS B 60   49049 26121 23755 -2986 -4511
3665   C
ATOM   6815 CD LYS B 60   17.936 -43.825 110.067 1.00269.89
C
ANISOU 6815 CD LYS B 60   49680 27737 25128 -3059 -4176
3803   C
ATOM   6816 CE LYS B 60   16.546 -43.815 110.686 1.00281.10
C
ANISOU 6816 CE LYS B 60   51062 29281 26463 -3897 -3772
3975   C
ATOM   6817 NZ LYS B 60   16.403 -42.745 111.711 1.00282.60
N
ANISOU 6817 NZ LYS B 60   50750 29825 26801 -3927 -3486
4087   N
ATOM   6818 N   PHE B 61   18.983 -44.155 105.586 1.00197.07
N
ANISOU 6818 N   PHE B 61   39503 19095 16280 -2146 -4568
3308   N
ATOM   6819 CA PHE B 61   20.248 -43.810 104.938 1.00190.16
C
ANISOU 6819 CA PHE B 61   38161 18425 15666 -1339 -4800
3017   C
ATOM   6820 C   PHE B 61   20.230 -42.400 104.355 1.00186.44
C
ANISOU 6820 C   PHE B 61   36522 18743 15575 -1253 -4429
2998   C
ATOM   6821 O   PHE B 61   21.174 -41.625 104.549 1.00174.51
O
ANISOU 6821 O   PHE B 61   34575 17483 14249   -714 -4486
2885   O

```
ATOM   6822  CB  PHE B  61      20.588 -44.819 103.842  1.00195.76
C
ANISOU 6822  CB  PHE B  61    39158  18880  16343  -1095  -5118
2775       C
ATOM   6823  CG  PHE B  61      21.766 -44.408 103.001  1.00193.58
C
ANISOU 6823  CG  PHE B  61    38273  18930  16349   -339  -5293
2456       C
ATOM   6824  CD1 PHE B  61      23.037 -44.356 103.548  1.00183.28
C
ANISOU 6824  CD1 PHE B  61    37116  17477  15044    364  -5631
2270       C
ATOM   6825  CD2 PHE B  61      21.597 -44.046 101.675  1.00160.14
C
ANISOU 6825  CD2 PHE B  61    33301  15175  12372   -341  -5109
2334       C
ATOM   6826  CE1 PHE B  61      24.121 -43.968 102.784  1.00176.32
C
ANISOU 6826  CE1 PHE B  61    35646  16943  14406   1030  -5777
1957       C
ATOM   6827  CE2 PHE B  61      22.678 -43.655 100.905  1.00158.05
C
ANISOU 6827  CE2 PHE B  61    32473  15234  12346    318  -5252
2036       C
ATOM   6828  CZ  PHE B  61      23.941 -43.617 101.461  1.00158.84
C
ANISOU 6828  CZ  PHE B  61    32709  15207  12435    994  -5580
1843       C
ATOM   6829  N   LEU B  62      19.158 -42.051 103.641  1.00177.61
N
ANISOU 6829  N   LEU B  62    34904  18020  14560  -1785  -4055
3099       N
ATOM   6830  CA  LEU B  62      19.088 -40.757 102.964  1.00151.02
C
ANISOU 6830  CA  LEU B  62    30471  15379  11530  -1713  -3723
3065       C
ATOM   6831  C   LEU B  62      19.155 -39.592 103.945  1.00153.52
C
ANISOU 6831  C   LEU B  62    30403  16001  11926  -1685  -3506
3221       C
ATOM   6832  O   LEU B  62      19.873 -38.614 103.711  1.00152.07
O
ANISOU 6832  O   LEU B  62    29557  16232  11990  -1255  -3459
3112       O
ATOM   6833  CB  LEU B  62      17.803 -40.681 102.137  1.00148.85
C
ANISOU 6833  CB  LEU B  62    29840  15415  11299  -2333  -3379
3154       C
ATOM   6834  CG  LEU B  62      17.581 -39.425 101.295  1.00149.39
C
ANISOU 6834  CG  LEU B  62    28863  16210  11688  -2313  -3042
3112       C
ATOM   6835  CD1 LEU B  62      18.789 -39.150 100.412  1.00141.67
C
```

```
ANISOU 6835  CD1 LEU B  62    27461  15419  10948  -1628  -3240
2834       C
ATOM   6836  CD2 LEU B  62      16.317 -39.558 100.463  1.00150.69
C
ANISOU 6836  CD2 LEU B  62    28798  16598  11858  -2902  -2766
3167       C
ATOM   6837  N   GLY B  63      18.413 -39.677 105.047  1.00162.35
N
ANISOU 6837  N   GLY B  63    31935  16925  12824  -2155  -3371
3471       N
ATOM   6838  CA  GLY B  63      18.500 -38.655 106.080  1.00167.59
C
ANISOU 6838  CA  GLY B  63    32327  17819  13533  -2111  -3201
3624       C
ATOM   6839  C   GLY B  63      19.848 -38.573 106.777  1.00167.08
C
ANISOU 6839  C   GLY B  63    32483  17518  13483  -1439  -3528
3515       C
ATOM   6840  O   GLY B  63      20.359 -37.479 107.029  1.00160.24
O
ANISOU 6840  O   GLY B  63    31045  17030  12810  -1133  -3427
3507       O
ATOM   6841  N   PHE B  64      20.442 -39.724 107.099  1.00182.85
N
ANISOU 6841  N   PHE B  64    35324  18886  15264  -1196  -3936
3417       N
ATOM   6842  CA  PHE B  64      21.808 -39.744 107.624  1.00189.37
C
ANISOU 6842  CA  PHE B  64    36359  19488  16105   -482  -4300
3247       C
ATOM   6843  C   PHE B  64      22.849 -39.196 106.649  1.00195.60
C
ANISOU 6843  C   PHE B  64    36439  20686  17195    139  -4397
2952       C
ATOM   6844  O   PHE B  64      23.700 -38.389 107.041  1.00200.58
O
ANISOU 6844  O   PHE B  64    36694  21549  17970    583  -4434
2878       O
ATOM   6845  CB  PHE B  64      22.167 -41.176 108.029  1.00176.72
C
ANISOU 6845  CB  PHE B  64    35843  17117  14186   -346  -4747
3169       C
ATOM   6846  CG  PHE B  64      23.619 -41.370 108.357  1.00170.92
C
ANISOU 6846  CG  PHE B  64    35346  16139  13456    456  -5188
2916       C
ATOM   6847  CD1 PHE B  64      24.189 -40.755 109.458  1.00178.44
C
ANISOU 6847  CD1 PHE B  64    36297  17086  14414    737  -5228
2971       C
ATOM   6848  CD2 PHE B  64      24.414 -42.177 107.558  1.00168.28
C
ANISOU 6848  CD2 PHE B  64    35231  15596  13113    944  -5573
2603       C
```

```
ATOM   6849  CE1 PHE B  64      25.527 -40.938 109.753  1.00182.13
C
ANISOU 6849  CE1 PHE B  64    36944  17359  14898   1483  -5632
2701       C
ATOM   6850  CE2 PHE B  64      25.750 -42.364 107.848  1.00169.64
C
ANISOU 6850  CE2 PHE B  64    35597  15582  13277   1704  -5990
2332       C
ATOM   6851  CZ  PHE B  64      26.308 -41.744 108.946  1.00177.92
C
ANISOU 6851  CZ  PHE B  64    36630  16637  14334   1974  -6024
2380       C
ATOM   6852  N   MET B  65      22.819 -39.621 105.387  1.00172.11
N
ANISOU 6852  N   MET B  65    33277  17809  14307    173  -4443
2775       N
ATOM   6853  CA  MET B  65      23.939 -39.307 104.502  1.00146.71
C
ANISOU 6853  CA  MET B  65    29524  14898  11320    803  -4605
2456       C
ATOM   6854  C   MET B  65      24.087 -37.803 104.268  1.00141.42
C
ANISOU 6854  C   MET B  65    27866  14924  10942    876  -4278
2473       C
ATOM   6855  O   MET B  65      23.105 -37.088 104.051  1.00138.62
O
ANISOU 6855  O   MET B  65    27051  14939  10679    390  -3884
2668       O
ATOM   6856  CB  MET B  65      23.799 -40.053 103.169  1.00147.92
C
ANISOU 6856  CB  MET B  65    29670  15031  11502    788  -4698
2274       C
ATOM   6857  CG  MET B  65      22.523 -39.804 102.380  1.00155.25
C
ANISOU 6857  CG  MET B  65    30207  16270  12511    155  -4305
2433       C
ATOM   6858  SD  MET B  65      22.601 -38.376 101.282  1.00180.18
S
ANISOU 6858  SD  MET B  65    32148  20268  16045    244  -3954
2342       S
ATOM   6859  CE  MET B  65      23.553 -39.053  99.924  1.00169.66
C
ANISOU 6859  CE  MET B  65    30727  18929  14806    779  -4272
1952       C
ATOM   6860  N   TRP B  66      25.332 -37.326 104.330  1.00173.01
N
ANISOU 6860  N   TRP B  66    31561  19103  15071   1492  -4457
2254       N
ATOM   6861  CA  TRP B  66      25.647 -35.908 104.184  1.00171.49
C
ANISOU 6861  CA  TRP B  66    30499  19532  15128   1613  -4198
2250       C
ATOM   6862  C   TRP B  66      27.133 -35.748 103.874  1.00169.62
C
```

```
ANISOU 6862  C   TRP B  66    29985  19457  15008   2324  -4474
1901       C
ATOM   6863  O   TRP B  66      27.919 -36.694 103.966  1.00139.10
O
ANISOU 6863  O   TRP B  66    26625  15209  11019   2752  -4876
1675       O
ATOM   6864  CB  TRP B  66      25.209 -35.059 105.387  1.00137.35
C
ANISOU 6864  CB  TRP B  66    26125  15284  10776   1363  -3970
2542       C
ATOM   6865  CG  TRP B  66      25.552 -35.545 106.750  1.00149.24
C
ANISOU 6865  CG  TRP B  66    28348  16283  12073   1515  -4214
2623       C
ATOM   6866  CD1 TRP B  66      26.734 -36.076 107.169  1.00170.98
C
ANISOU 6866  CD1 TRP B  66    31494  18719  14753   2106  -4626
2397       C
ATOM   6867  CD2 TRP B  66      24.722 -35.440 107.914  1.00182.37
C
ANISOU 6867  CD2 TRP B  66    32913  20271  16106   1084  -4053
2945       C
ATOM   6868  NE1 TRP B  66      26.671 -36.358 108.515  1.00206.94
N
ANISOU 6868  NE1 TRP B  66    36693  22832  19102   2065  -4743
2568       N
ATOM   6869  CE2 TRP B  66      25.448 -35.973 108.995  1.00192.52
C
ANISOU 6869  CE2 TRP B  66    34862  21068  17218   1429  -4388
2911       C
ATOM   6870  CE3 TRP B  66      23.425 -34.965 108.140  1.00168.23
C
ANISOU 6870  CE3 TRP B  66    30961  18672  14288    442  -3663
3243       C
ATOM   6871  CZ2 TRP B  66      24.922 -36.046 110.284  1.00158.76
C
ANISOU 6871  CZ2 TRP B  66    31107  16474  12742   1134  -4335
3181       C
ATOM   6872  CZ3 TRP B  66      22.904 -35.039 109.419  1.00137.01
C
ANISOU 6872  CZ3 TRP B  66    27492  14433  10133    149  -3606
3498       C
ATOM   6873  CH2 TRP B  66      23.651 -35.574 110.475  1.00139.91
C
ANISOU 6873  CH2 TRP B  66    28533  14298  10329    484  -3935
3474       C
ATOM   6874  N   ASN B  67      27.501 -34.520 103.511  1.00167.97
N
ANISOU 6874  N   ASN B  67    28969  19833  15020   2437  -4257
1851       N
ATOM   6875  CA  ASN B  67      28.803 -34.196 102.938  1.00145.20
C
ANISOU 6875  CA  ASN B  67    25629  17267  12275   3013  -4427
1503       C
```

```
ATOM   6876  C   ASN B  67      29.903 -34.000 103.985  1.00135.12
C
ANISOU 6876  C   ASN B  67    24493  15890  10957   3508  -4673
1391       C
ATOM   6877  O   ASN B  67      29.647 -33.959 105.190  1.00135.73
O
ANISOU 6877  O   ASN B  67    24971  15680  10918   3400  -4682
1613       O
ATOM   6878  CB  ASN B  67      28.656 -32.946 102.068  1.00141.54
C
ANISOU 6878  CB  ASN B  67    24268  17470  12039   2852  -4070
1508       C
ATOM   6879  CG  ASN B  67      28.152 -31.742 102.846  1.00140.90
C
ANISOU 6879  CG  ASN B  67    23884  17640  12012   2561  -3758
1802       C
ATOM   6880  OD1 ASN B  67      27.991 -31.793 104.066  1.00129.92
O
ANISOU 6880  OD1 ASN B  67    22905  15955  10503   2508  -3803
1992       O
ATOM   6881  ND2 ASN B  67      27.900 -30.647 102.138  1.00173.46
N
ANISOU 6881  ND2 ASN B  67    27304  22303  16302   2379  -3451
1835       N
ATOM   6882  N   PRO B  68      31.177 -33.888 103.517  1.00147.91
N
ANISOU 6882  N   PRO B  68    25772  17763  12663   4072  -4886
1021       N
ATOM   6883  CA  PRO B  68      32.281 -33.355 104.337  1.00159.97
C
ANISOU 6883  CA  PRO B  68    27172  19400  14210   4535  -5044
883       C
ATOM   6884  C   PRO B  68      31.990 -32.164 105.239  1.00141.45
C
ANISOU 6884  C   PRO B  68    24551  17263  11930   4304  -4766
1173       C
ATOM   6885  O   PRO B  68      32.428 -32.154 106.393  1.00145.18
O
ANISOU 6885  O   PRO B  68    25351  17495  12316   4532  -4932
1211       O
ATOM   6886  CB  PRO B  68      33.333 -32.998 103.280  1.00150.79
C
ANISOU 6886  CB  PRO B  68    25348  18751  13195   4932  -5101
492       C
ATOM   6887  CG  PRO B  68      33.153 -34.057 102.259  1.00133.75
C
ANISOU 6887  CG  PRO B  68    23414  16419  10986   4953  -5251
319       C
ATOM   6888  CD  PRO B  68      31.670 -34.349 102.205  1.00143.98
C
ANISOU 6888  CD  PRO B  68    25025  17450  12229   4317  -5013
687       C
ATOM   6889  N   LEU B  69      31.288 -31.149 104.734  1.00124.88
N
```

```
ANISOU 6889  N   LEU B  69    21867  15607   9974   3886  -4364
1364       N
ATOM   6890  CA  LEU B  69      30.945 -30.005 105.575  1.00132.75
C
ANISOU 6890  CA  LEU B  69    22620  16801  11017   3663  -4107
1645       C
ATOM   6891  C   LEU B  69      30.140 -30.427 106.799  1.00150.59
C
ANISOU 6891  C   LEU B  69    25543  18567  13106   3391  -4110
1963       C
ATOM   6892  O   LEU B  69      30.402 -29.962 107.915  1.00148.41
O
ANISOU 6892  O   LEU B  69    25375  18219  12795   3499  -4136
2082       O
ATOM   6893  CB  LEU B  69      30.154 -28.977 104.762  1.00126.90
C
ANISOU 6893  CB  LEU B  69    21238  16560  10420   3228  -3697
1800       C
ATOM   6894  CG  LEU B  69      30.746 -28.392 103.478  1.00115.89
C
ANISOU 6894  CG  LEU B  69    19149  15698   9185   3367  -3612
1540       C
ATOM   6895  CD1 LEU B  69      29.897 -27.224 102.995  1.00111.83
C
ANISOU 6895  CD1 LEU B  69    18094  15620   8776   2925  -3212
1754       C
ATOM   6896  CD2 LEU B  69      32.187 -27.962 103.680  1.00116.09
C
ANISOU 6896  CD2 LEU B  69    18890  15957   9260   3886  -3800
1258       C
ATOM   6897  N   SER B  70      29.157 -31.304 106.610  1.00157.91
N
ANISOU 6897  N   SER B  70    26924  19160  13916   3020  -4080
2100       N
ATOM   6898  CA  SER B  70      28.443 -31.898 107.738  1.00157.26
C
ANISOU 6898  CA  SER B  70    27561  18563  13628   2752  -4118
2365       C
ATOM   6899  C   SER B  70      29.349 -32.756 108.621  1.00157.02
C
ANISOU 6899  C   SER B  70    28209  18016  13433   3223  -4548
2216       C
ATOM   6900  O   SER B  70      29.208 -32.751 109.850  1.00142.84
O
ANISOU 6900  O   SER B  70    26843  15920  11508   3172  -4587
2409       O
ATOM   6901  CB  SER B  70      27.266 -32.723 107.226  1.00131.95
C
ANISOU 6901  CB  SER B  70    24691  15129  10316   2248  -4011
2498       C
ATOM   6902  OG  SER B  70      26.398 -31.936 106.431  1.00132.85
O
ANISOU 6902  OG  SER B  70    24190  15716  10571   1829  -3627
2621       O
```

```
ATOM   6903  N   TRP B  71      30.276 -33.509 108.020  1.00147.15
N
ANISOU 6903  N   TRP B  71    27082  16654  12173   3697  -4886
1862       N
ATOM   6904  CA  TRP B  71      31.322 -34.163 108.806  1.00137.11
C
ANISOU 6904  CA  TRP B  71    26349  14980  10764   4259  -5325
1652       C
ATOM   6905  C   TRP B  71      32.162 -33.176 109.615  1.00135.22
C
ANISOU 6905  C   TRP B  71    25768  14992  10618   4597  -5333
1617       C
ATOM   6906  O   TRP B  71      32.483 -33.440 110.779  1.00143.66
O
ANISOU 6906  O   TRP B  71    27367  15672  11545   4804  -5549
1662       O
ATOM   6907  CB  TRP B  71      32.263 -34.955 107.887  1.00139.72
C
ANISOU 6907  CB  TRP B  71    26700  15291  11095   4769  -5672
1221       C
ATOM   6908  CG  TRP B  71      31.665 -36.019 106.989  1.00173.13
C
ANISOU 6908  CG  TRP B  71    31279  19266  15235   4558  -5744
1179       C
ATOM   6909  CD1 TRP B  71      32.039 -36.289 105.702  1.00142.35
C
ANISOU 6909  CD1 TRP B  71    27033  15620  11432   4752  -5813
889       C
ATOM   6910  CD2 TRP B  71      30.672 -37.003 107.324  1.00207.78
C
ANISOU 6910  CD2 TRP B  71    36465  23084  19399   4144  -5785
1410       C
ATOM   6911  NE1 TRP B  71      31.308 -37.333 105.198  1.00144.62
N
ANISOU 6911  NE1 TRP B  71    27822  15539  11587   4483  -5884
940       N
ATOM   6912  CE2 TRP B  71      30.467 -37.796 106.174  1.00195.92
C
ANISOU 6912  CE2 TRP B  71    35033  21526  17882   4098  -5870
1255       C
ATOM   6913  CE3 TRP B  71      29.924 -37.280 108.473  1.00190.69
C
ANISOU 6913  CE3 TRP B  71    34957  20463  17035   3780  -5747
1732       C
ATOM   6914  CZ2 TRP B  71      29.546 -38.842 106.141  1.00167.00
C
ANISOU 6914  CZ2 TRP B  71    32076  17364  14010   3696  -5923
1413       C
ATOM   6915  CZ3 TRP B  71      29.012 -38.324 108.437  1.00195.48
C
ANISOU 6915  CZ3 TRP B  71    36264  20586  17424   3361  -5790
1882       C
ATOM   6916  CH2 TRP B  71      28.830 -39.089 107.279  1.00192.70
C
```

ANISOU 6916 CH2 TRP B 71   35968 20188 17062   3318 -5878
1725   C
ATOM   6917 N   VAL B 72   32.536 -32.040 109.021 1.00131.52
N
ANISOU 6917 N   VAL B 72   24443 15157 10373   4652 -5110
1534   N
ATOM   6918 CA VAL B 72   33.183 -30.968 109.781 1.00135.57
C
ANISOU 6918 CA VAL B 72   24588 15946 10975   4870 -5058
1552   C
ATOM   6919 C   VAL B 72   32.278 -30.395 110.870 1.00130.65
C
ANISOU 6919 C   VAL B 72   24141 15199 10300   4451 -4811
1977   C
ATOM   6920 O   VAL B 72   32.730 -30.119 111.989 1.00127.87
O
ANISOU 6920 O   VAL B 72   23985 14700   9899   4672 -4926
2030   O
ATOM   6921 CB VAL B 72   33.665 -29.866 108.818 1.00125.82
C
ANISOU 6921 CB VAL B 72   22419 15420   9965   4927 -4844
1393   C
ATOM   6922 CG1 VAL B 72   34.129 -28.637 109.588 1.00123.13
C
ANISOU 6922 CG1 VAL B 72   21686 15387   9712   5027 -4727
1478   C
ATOM   6923 CG2 VAL B 72   34.778 -30.390 107.921 1.00129.56
C
ANISOU 6923 CG2 VAL B 72   22709 16044 10473   5428 -5124
926   C
ATOM   6924 N   MET B 73   30.990 -30.219 110.574 1.00125.84
N
ANISOU 6924 N   MET B 73   23465 14657   9693   3852 -4478
2270   N
ATOM   6925 CA MET B 73   30.012 -29.919 111.619 1.00125.06
C
ANISOU 6925 CA MET B 73   23646 14379   9494   3427 -4272
2658   C
ATOM   6926 C   MET B 73   29.914 -31.005 112.685 1.00140.22
C
ANISOU 6926 C   MET B 73   26464 15618 11196   3447 -4516
2724   C
ATOM   6927 O   MET B 73   29.792 -30.700 113.877 1.00137.27
O
ANISOU 6927 O   MET B 73   26177 15105 10876   3334 -4403
2857   O
ATOM   6928 CB MET B 73   28.644 -29.673 110.988 1.00123.09
C
ANISOU 6928 CB MET B 73   23158 14340   9270   2794 -3893
2896   C
ATOM   6929 CG MET B 73   28.532 -28.326 110.305 1.00136.81
C
ANISOU 6929 CG MET B 73   24027 16736 11218   2676 -3578
2922   C

```
ATOM   6930  SD  MET B  73      28.348 -26.994 111.502  1.00115.48
S
ANISOU 6930  SD  MET B  73    21089  14248   8538   2581  -3369
3207       S
ATOM   6931  CE  MET B  73      26.576 -27.033 111.748  1.00115.07
C
ANISOU 6931  CE  MET B  73    21221  14135   8365   1858  -3033
3577       C
ATOM   6932  N   GLU B  74      29.956 -32.275 112.283  1.00155.52
N
ANISOU 6932  N   GLU B  74    28969  17142  12978   3532  -4787
2578       N
ATOM   6933  CA  GLU B  74      30.049 -33.361 113.256  1.00152.63
C
ANISOU 6933  CA  GLU B  74    29367  16127  12499   3573  -5003
2522       C
ATOM   6934  C   GLU B  74      31.320 -33.293 114.099  1.00138.60
C
ANISOU 6934  C   GLU B  74    27611  14232  10821   4137  -5244
2256       C
ATOM   6935  O   GLU B  74      31.280 -33.540 115.310  1.00143.59
O
ANISOU 6935  O   GLU B  74    28639  14493  11424   4074  -5257
2311       O
ATOM   6936  CB  GLU B  74      29.958 -34.705 112.533  1.00165.91
C
ANISOU 6936  CB  GLU B  74    31630  17414  13995   3609  -5280
2375       C
ATOM   6937  CG  GLU B  74      30.070 -35.918 113.440  1.00180.88
C
ANISOU 6937  CG  GLU B  74    34382  18604  15738   3657  -5537
2283       C
ATOM   6938  CD  GLU B  74      29.450 -37.157 112.825  1.00202.09
C
ANISOU 6938  CD  GLU B  74    37689  20886  18211   3406  -5678
2283       C
ATOM   6939  OE1 GLU B  74      28.214 -37.178 112.644  1.00203.79
O
ANISOU 6939  OE1 GLU B  74    37970  21117  18343   2772  -5394
2578       O
ATOM   6940  OE2 GLU B  74      30.199 -38.108 112.518  1.00212.39
O
ANISOU 6940  OE2 GLU B  74    39402  21868  19430   3841  -6072
1972       O
ATOM   6941  N   MET B  75      32.454 -32.952 113.484  1.00139.74
N
ANISOU 6941  N   MET B  75    27310  14709  11075   4678  -5426
1947       N
ATOM   6942  CA  MET B  75      33.679 -32.724 114.249  1.00146.36
C
ANISOU 6942  CA  MET B  75    28048  15536  12027   5192  -5611
1679       C
ATOM   6943  C   MET B  75      33.555 -31.568 115.238  1.00135.54
C
```

```
ANISOU 6943  C   MET B  75    26309  14386  10803   5012  -5322
1899       C
ATOM   6944  O   MET B  75      34.022 -31.671 116.378  1.00137.17
O
ANISOU 6944  O   MET B  75    26771  14314  11032   5180  -5413
1827       O
ATOM   6945  CB  MET B  75      34.848 -32.482 113.293  1.00138.55
C
ANISOU 6945  CB  MET B  75    26537  14971  11134   5740  -5807
1296       C
ATOM   6946  CG  MET B  75      36.185 -32.292 113.992  1.00158.27
C
ANISOU 6946  CG  MET B  75    28901  17494  13740   6284  -6006
961       C
ATOM   6947  SD  MET B  75      37.330 -31.277 113.039  1.00160.74
S
ANISOU 6947  SD  MET B  75    28252  18586  14238   6695  -6000
640       S
ATOM   6948  CE  MET B  75      36.597 -29.659 113.259  1.00144.46
C
ANISOU 6948  CE  MET B  75    25566  17006  12317   6200  -5520
1066       C
ATOM   6949  N   ALA B  76      32.935 -30.461 114.827  1.00131.18
N
ANISOU 6949  N   ALA B  76    25168  14329  10345   4678  -4980
2153       N
ATOM   6950  CA  ALA B  76      32.679 -29.364 115.760  1.00128.25
C
ANISOU 6950  CA  ALA B  76    24466  14156  10108   4459  -4693
2385       C
ATOM   6951  C   ALA B  76      31.812 -29.785 116.944  1.00129.89
C
ANISOU 6951  C   ALA B  76    25206  13916  10232   4071  -4579
2632       C
ATOM   6952  O   ALA B  76      32.110 -29.432 118.092  1.00141.43
O
ANISOU 6952  O   ALA B  76    26688  15277  11773   4138  -4549
2652       O
ATOM   6953  CB  ALA B  76      32.031 -28.194 115.019  1.00123.65
C
ANISOU 6953  CB  ALA B  76    23219  14155   9607   4135  -4355
2608       C
ATOM   6954  N   ALA B  77      30.736 -30.532 116.693  1.00131.42
N
ANISOU 6954  N   ALA B  77    25819  13852  10262   3644  -4508
2810       N
ATOM   6955  CA  ALA B  77      29.948 -31.094 117.789  1.00138.18
C
ANISOU 6955  CA  ALA B  77    27230  14263  11009   3271  -4426
2992       C
ATOM   6956  C   ALA B  77      30.762 -32.051 118.657  1.00144.87
C
ANISOU 6956  C   ALA B  77    28716  14552  11775   3634  -4768
2746       C
```

```
ATOM   6957  O   ALA B  77      30.695 -31.996 119.890  1.00150.65
O
ANISOU 6957  O   ALA B  77    29664  15062  12512   3553  -4717
2813       O
ATOM   6958  CB  ALA B  77      28.711 -31.797 117.229  1.00135.05
C
ANISOU 6958  CB  ALA B  77    27165  13712  10437   2743  -4303
3179       C
ATOM   6959  N   ILE B  78      31.529 -32.944 118.027  1.00141.03
N
ANISOU 6959  N   ILE B  78    28541  13838  11204   4045  -5125
2439       N
ATOM   6960  CA  ILE B  78      32.435 -33.826 118.762  1.00146.79
C
ANISOU 6960  CA  ILE B  78    29849  14072  11853   4472  -5487
2137       C
ATOM   6961  C   ILE B  78      33.479 -33.043 119.554  1.00156.06
C
ANISOU 6961  C   ILE B  78    30669  15428  13198   4883  -5526
1977       C
ATOM   6962  O   ILE B  78      33.748 -33.349 120.720  1.00146.38
O
ANISOU 6962  O   ILE B  78    29849  13841  11926   4969  -5625
1910       O
ATOM   6963  CB  ILE B  78      33.095 -34.832 117.800  1.00148.40
C
ANISOU 6963  CB  ILE B  78    30350  14087  11949   4880  -5866
1803       C
ATOM   6964  CG1 ILE B  78      32.064 -35.849 117.304  1.00150.81
C
ANISOU 6964  CG1 ILE B  78    31221  14038  12043   4449  -5874
1948       C
ATOM   6965  CG2 ILE B  78      34.258 -35.543 118.476  1.00152.46
C
ANISOU 6965  CG2 ILE B  78    31312  14208  12407   5443  -6257
1413       C
ATOM   6966  CD1 ILE B  78      31.513 -36.745 118.396  1.00154.78
C
ANISOU 6966  CD1 ILE B  78    32538  13920  12352   4145  -5922
2032       C
ATOM   6967  N   MET B  79      34.079 -32.021 118.944  1.00164.44
N
ANISOU 6967  N   MET B  79    30978  17056  14446   5121  -5447
1904       N
ATOM   6968  CA  MET B  79      34.972 -31.135 119.688  1.00138.88
C
ANISOU 6968  CA  MET B  79    27338  14048  11381   5422  -5426
1790       C
ATOM   6969  C   MET B  79      34.279 -30.421 120.846  1.00136.90
C
ANISOU 6969  C   MET B  79    27019  13799  11198   5025  -5123
2112       C
ATOM   6970  O   MET B  79      34.869 -30.262 121.921  1.00137.74
O
```

ANISOU 6970 O   MET B 79   27218 13762 11353   5225 -5188
2012   O
ATOM   6971 CB MET B 79   35.595 -30.109 118.740 1.00146.19
C
ANISOU 6971 CB MET B 79   27445 15620 12480   5643 -5350
1685   C
ATOM   6972 CG MET B 79   36.598 -29.179 119.408 1.00133.98
C
ANISOU 6972 CG MET B 79   25455 14342 11109   5948 -5335
1538   C
ATOM   6973 SD MET B 79   37.162 -27.851 118.326 1.00165.04
S
ANISOU 6973 SD MET B 79   28412 19068 15229   6068 -5180
1464   S
ATOM   6974 CE MET B 79   35.696 -26.825 118.262 1.00131.85
C
ANISOU 6974 CE MET B 79   23872 15146 11078   5386 -4721
1988   C
ATOM   6975 N   ALA B 80   33.034 -29.980 120.655 1.00134.38
N
ANISOU 6975 N   ALA B 80   26521 13660 10878   4472 -4792
2478   N
ATOM   6976 CA ALA B 80   32.303 -29.335 121.745 1.00132.73
C
ANISOU 6976 CA ALA B 80   26229 13477 10727   4090 -4503
2767   C
ATOM   6977 C   ALA B 80   32.089 -30.248 122.952 1.00141.58
C
ANISOU 6977 C   ALA B 80   28084 14010 11700   3997 -4613
2760   C
ATOM   6978 O   ALA B 80   32.324 -29.841 124.095 1.00136.51
O
ANISOU 6978 O   ALA B 80   27429 13310 11129   4042 -4565
2779   O
ATOM   6979 CB ALA B 80   30.958 -28.823 121.227 1.00129.89
C
ANISOU 6979 CB ALA B 80   25566 13423 10364   3522 -4148
3113   C
ATOM   6980 N   ILE B 81   31.636 -31.481 122.731 1.00140.45
N
ANISOU 6980 N   ILE B 81   28600 13424 11341   3851 -4763
2730   N
ATOM   6981 CA ILE B 81   31.573 -32.446 123.830 1.00144.85
C
ANISOU 6981 CA ILE B 81   29924 13388 11723   3807 -4922
2663   C
ATOM   6982 C   ILE B 81   32.966 -32.824 124.339 1.00147.45
C
ANISOU 6982 C   ILE B 81   30513 13461 12051   4426 -5286
2282   C
ATOM   6983 O   ILE B 81   33.214 -32.829 125.551 1.00148.82
O
ANISOU 6983 O   ILE B 81   30931 13407 12208   4487 -5321
2241   O

```
ATOM   6984  CB  ILE B  81      30.738 -33.681 123.435  1.00148.45
C
ANISOU 6984  CB  ILE B  81    31047  13428  11930   3462  -4998
2715       C
ATOM   6985  CG1 ILE B  81      30.575 -34.620 124.635  1.00153.08
C
ANISOU 6985  CG1 ILE B  81    32438  13412  12311   3342  -5136
2664       C
ATOM   6986  CG2 ILE B  81      31.314 -34.410 122.235  1.00165.33
C
ANISOU 6986  CG2 ILE B  81    33342  15478  13997   3798  -5296
2465       C
ATOM   6987  CD1 ILE B  81      29.476 -35.647 124.467  1.00156.31
C
ANISOU 6987  CD1 ILE B  81    33458  13460  12474   2820  -5104
2797       C
ATOM   6988  N   ALA B  82      33.892 -33.146 123.432  1.00165.50
N
ANISOU 6988  N   ALA B  82    32738  15801  14344   4900  -5563
1976       N
ATOM   6989  CA  ALA B  82      35.230 -33.575 123.843  1.00185.16
C
ANISOU 6989  CA  ALA B  82    35470  18072  16809   5513  -5927
1559       C
ATOM   6990  C   ALA B  82      36.037 -32.476 124.530  1.00188.55
C
ANISOU 6990  C   ALA B  82    35358  18837  17447   5774  -5842
1490       C
ATOM   6991  O   ALA B  82      36.622 -32.708 125.595  1.00167.87
O
ANISOU 6991  O   ALA B  82    33066  15937  14779   6000  -5996
1315       O
ATOM   6992  CB  ALA B  82      36.000 -34.116 122.638  1.00189.05
C
ANISOU 6992  CB  ALA B  82    35933  18626  17271   5959  -6225
1229       C
ATOM   6993  N   LEU B  83      36.085 -31.273 123.956  1.00193.62
N
ANISOU 6993  N   LEU B  83    35189  20072  18304   5736  -5603
1617       N
ATOM   6994  CA  LEU B  83      37.047 -30.283 124.428  1.00154.69
C
ANISOU 6994  CA  LEU B  83    29738  15473  13564   6045  -5573
1482       C
ATOM   6995  C   LEU B  83      36.459 -28.981 124.955  1.00157.70
C
ANISOU 6995  C   LEU B  83    29578  16216  14124   5672  -5187
1830       C
ATOM   6996  O   LEU B  83      37.222 -28.146 125.454  1.00192.71
O
ANISOU 6996  O   LEU B  83    33618  20894  18711   5886  -5154
1736       O
ATOM   6997  CB  LEU B  83      38.051 -29.960 123.313  1.00146.97
C
```

```
ANISOU 6997  CB  LEU B  83    28240  14916  12687   6477  -5709
1188       C
ATOM   6998  CG  LEU B  83      38.820 -31.164 122.764  1.00164.21
C
ANISOU 6998  CG  LEU B  83    30859  16817  14715   6943  -6120
769        C
ATOM   6999  CD1 LEU B  83      39.696 -30.756 121.591  1.00151.09
C
ANISOU 6999  CD1 LEU B  83    28583  15662  13163   7313  -6204
494        C
ATOM   7000  CD2 LEU B  83      39.651 -31.814 123.860  1.00149.32
C
ANISOU 7000  CD2 LEU B  83    29504  14507  12723   7314  -6408
455        C
ATOM   7001  N   ALA B  84      35.148 -28.771 124.875  1.00159.84
N
ANISOU 7001  N   ALA B  84    29810  16544  14378   5126  -4899
2208       N
ATOM   7002  CA  ALA B  84      34.559 -27.544 125.395  1.00185.61
C
ANISOU 7002  CA  ALA B  84    32561  20159  17804   4786  -4547
2516       C
ATOM   7003  C   ALA B  84      33.983 -27.747 126.791  1.00191.71
C
ANISOU 7003  C   ALA B  84    33732  20594  18516   4531  -4458
2672       C
ATOM   7004  O   ALA B  84      33.301 -26.859 127.311  1.00194.07
O
ANISOU 7004  O   ALA B  84    33677  21137  18923   4200  -4164
2944       O
ATOM   7005  CB  ALA B  84      33.485 -27.010 124.443  1.00128.21
C
ANISOU 7005  CB  ALA B  84    24886  13263  10564   4357  -4262
2811       C
ATOM   7006  N   ASN B  85      34.256 -28.900 127.405  1.00151.22
N
ANISOU 7006  N   ASN B  85    29335  14916  13204   4687  -4719
2484       N
ATOM   7007  CA  ASN B  85      34.039 -29.124 128.834  1.00156.50
C
ANISOU 7007  CA  ASN B  85    30415  15244  13804   4565  -4704
2537       C
ATOM   7008  C   ASN B  85      35.166 -28.454 129.625  1.00172.25
C
ANISOU 7008  C   ASN B  85    32153  17351  15945   4954  -4782
2345       C
ATOM   7009  O   ASN B  85      36.025 -29.097 130.232  1.00196.52
O
ANISOU 7009  O   ASN B  85    35671  20078  18918   5317  -5065
2049       O
ATOM   7010  CB  ASN B  85      33.945 -30.616 129.129  1.00150.88
C
ANISOU 7010  CB  ASN B  85    30611  13902  12814   4582  -4974
2383       C
```

ATOM   7011 CG ASN B 85   33.555 -30.905 130.567 1.00162.33
C
ANISOU 7011 CG ASN B 85   32529 14995 14153   4378 -4938
2459   C
ATOM   7012 OD1 ASN B 85   33.228 -29.996 131.332 1.00158.07
O
ANISOU 7012 OD1 ASN B 85   31617 14691 13753   4189 -4688
2658   O
ATOM   7013 ND2 ASN B 85   33.588 -32.178 130.943 1.00158.35
N
ANISOU 7013 ND2 ASN B 85   32857 13922 13386   4415 -5200
2291   N
ATOM   7014 N   GLY B 86   35.149 -27.124 129.603 1.00150.39
N
ANISOU 7014 N   GLY B 86   28654 15082 13404   4864 -4528
2510   N
ATOM   7015 CA GLY B 86   36.010 -26.335 130.460 1.00156.54
C
ANISOU 7015 CA GLY B 86   29145 16000 14335   5109 -4531
2401   C
ATOM   7016 C   GLY B 86   35.191 -25.285 131.177 1.00180.49
C
ANISOU 7016 C   GLY B 86   31779 19289 17510   4712 -4191
2738   C
ATOM   7017 O   GLY B 86   35.660 -24.632 132.114 1.00178.25
O
ANISOU 7017 O   GLY B 86   31311 19072 17341   4810 -4152
2714   O
ATOM   7018 N   ASP B 87   33.946 -25.126 130.727 1.00186.74
N
ANISOU 7018 N   ASP B 87   32428 20237 18287   4262 -3949
3043   N
ATOM   7019 CA ASP B 87   33.016 -24.133 131.246 1.00177.23
C
ANISOU 7019 CA ASP B 87   30801 19332 17204   3865 -3622
3363   C
ATOM   7020 C   ASP B 87   31.858 -24.811 131.972 1.00197.84
C
ANISOU 7020 C   ASP B 87   33886 21639 19646   3482 -3520
3552   C
ATOM   7021 O   ASP B 87   30.779 -24.227 132.120 1.00185.32
O
ANISOU 7021 O   ASP B 87   32001 20308 18102   3086 -3242
3830   O
ATOM   7022 CB ASP B 87   32.510 -23.226 130.124 1.00156.49
C
ANISOU 7022 CB ASP B 87   27531 17230 14699   3655 -3407
3542   C
ATOM   7023 CG ASP B 87   31.963 -21.909 130.643 1.00158.00
C
ANISOU 7023 CG ASP B 87   27145 17817 15069   3389 -3127
3784   C
ATOM   7024 OD1 ASP B 87   32.196 -21.594 131.830 1.00158.90
O

```
ANISOU 7024  OD1 ASP B  87    27286  17837  15251   3435  -3115
3785       O
ATOM   7025  OD2 ASP B  87      31.306 -21.186 129.865  1.00169.43
O
ANISOU 7025  OD2 ASP B  87    28120  19669  16586   3139  -2933
3959       O
ATOM   7026  N   GLY B  88      32.081 -26.041 132.428  1.00211.91
N
ANISOU 7026  N   GLY B  88    36410  22883  21222   3597  -3754
3382       N
ATOM   7027  CA  GLY B  88      31.128 -26.914 133.074  1.00174.66
C
ANISOU 7027  CA  GLY B  88    32282  17789  16292   3255  -3718
3497       C
ATOM   7028  C   GLY B  88      30.072 -27.539 132.170  1.00199.40
C
ANISOU 7028  C   GLY B  88    35599  20892  19273   2879  -3627
3643       C
ATOM   7029  O   GLY B  88      30.096 -27.461 130.940  1.00200.40
O
ANISOU 7029  O   GLY B  88    35469  21240  19435   2911  -3627
3636       O
ATOM   7030  N   ARG B  89      29.124 -28.182 132.849  1.00181.52
N
ANISOU 7030  N   ARG B  89    33803  18342  16822   2500  -3547
3769       N
ATOM   7031  CA  ARG B  89      28.094 -28.981 132.186  1.00204.13
C
ANISOU 7031  CA  ARG B  89    36990  21080  19491   2094  -3479
3881       C
ATOM   7032  C   ARG B  89      27.190 -28.267 131.179  1.00203.75
C
ANISOU 7032  C   ARG B  89    36328  21550  19536   1779  -3189
4110       C
ATOM   7033  O   ARG B  89      26.930 -28.861 130.118  1.00242.28
O
ANISOU 7033  O   ARG B  89    41360  26382  24313   1674  -3237
4090       O
ATOM   7034  CB  ARG B  89      27.235 -29.690 133.245  1.00201.28
C
ANISOU 7034  CB  ARG B  89    37210  20356  18912   1707  -3420
3972       C
ATOM   7035  CG  ARG B  89      27.821 -31.000 133.786  1.00149.59
C
ANISOU 7035  CG  ARG B  89    31569  13144  12123   1881  -3762
3723       C
ATOM   7036  CD  ARG B  89      29.162 -30.865 134.492  1.00149.99
C
ANISOU 7036  CD  ARG B  89    31705  13030  12254   2426  -4011
3472       C
ATOM   7037  NE  ARG B  89      29.087 -30.044 135.696  1.00177.49
N
ANISOU 7037  NE  ARG B  89    34914  16668  15857   2391  -3846
3579       N
```

```
ATOM   7038  CZ  ARG B  89      30.114 -29.825 136.511  1.00185.69
C
ANISOU 7038  CZ  ARG B  89    35995  17594  16964   2791  -4010
3391       C
ATOM   7039  NH1 ARG B  89      31.323 -30.266 136.193  1.00185.60
N
ANISOU 7039  NH1 ARG B  89    36210  17379  16931   3280  -4332
3078       N
ATOM   7040  NH2 ARG B  89      29.951 -29.080 137.596  1.00188.17
N
ANISOU 7040  NH2 ARG B  89    36058  18055  17384   2718  -3845
3509       N
ATOM   7041  N   PRO B  90      26.685 -27.054 131.409  1.00195.32
N
ANISOU 7041  N   PRO B  90    34594  20974  18645   1627  -2904
4312       N
ATOM   7042  CA  PRO B  90      25.631 -26.527 130.515  1.00198.22
C
ANISOU 7042  CA  PRO B  90    34483  21793  19039   1272  -2628
4518       C
ATOM   7043  C   PRO B  90      26.119 -26.189 129.114  1.00221.57
C
ANISOU 7043  C   PRO B  90    37054  25035  22098   1475  -2681
4450       C
ATOM   7044  O   PRO B  90      25.402 -26.482 128.146  1.00227.41
O
ANISOU 7044  O   PRO B  90    37776  25886  22745   1213  -2587
4521       O
ATOM   7045  CB  PRO B  90      25.137 -25.270 131.252  1.00179.67
C
ANISOU 7045  CB  PRO B  90    31535  19878  16854   1152  -2362
4705       C
ATOM   7046  CG  PRO B  90      25.447 -25.526 132.678  1.00170.29
C
ANISOU 7046  CG  PRO B  90    30724  18348  15631   1237  -2452
4655       C
ATOM   7047  CD  PRO B  90      26.752 -26.281 132.663  1.00188.48
C
ANISOU 7047  CD  PRO B  90    33502  20214  17899   1665  -2801
4382       C
ATOM   7048  N   PRO B  91      27.307 -25.599 128.922  1.00226.14
N
ANISOU 7048  N   PRO B  91    37324  25748  22852   1917  -2825
4301       N
ATOM   7049  CA  PRO B  91      27.749 -25.417 127.527  1.00205.07
C
ANISOU 7049  CA  PRO B  91    34355  23322  20238   2092  -2892
4216       C
ATOM   7050  C   PRO B  91      28.125 -26.728 126.858  1.00197.04
C
ANISOU 7050  C   PRO B  91    33938  21886  19042   2233  -3163
4025       C
ATOM   7051  O   PRO B  91      27.864 -26.920 125.664  1.00178.66
O
```

```
ANISOU 7051  O   PRO B  91    31519  19697  16666   2152  -3153
4031       O
ATOM   7052  CB  PRO B  91      28.961 -24.482 127.661  1.00201.42
C
ANISOU 7052  CB  PRO B  91    33451  23086  19994   2513  -2983
4081       C
ATOM   7053  CG  PRO B  91      28.719 -23.738 128.916  1.00205.55
C
ANISOU 7053  CG  PRO B  91    33775  23702  20623   2406  -2829
4211       C
ATOM   7054  CD  PRO B  91      28.117 -24.769 129.834  1.00219.66
C
ANISOU 7054  CD  PRO B  91    36224  25023  22214   2200  -2855
4247       C
ATOM   7055  N   ASP B  92      28.737 -27.633 127.620  1.00208.63
N
ANISOU 7055  N   ASP B  92    36029  22840  20402   2451  -3418
3843       N
ATOM   7056  CA  ASP B  92      29.075 -28.974 127.150  1.00197.88
C
ANISOU 7056  CA  ASP B  92    35332  21010  18843   2592  -3713
3642       C
ATOM   7057  C   ASP B  92      27.849 -29.748 126.659  1.00187.49
C
ANISOU 7057  C   ASP B  92    34354  19553  17329   2092  -3599
3797       C
ATOM   7058  O   ASP B  92      27.863 -30.328 125.567  1.00171.79
O
ANISOU 7058  O   ASP B  92    32504  17511  15257   2114  -3714
3722       O
ATOM   7059  CB  ASP B  92      29.798 -29.723 128.278  1.00170.76
C
ANISOU 7059  CB  ASP B  92    32521  17051  15308   2859  -3978
3434       C
ATOM   7060  CG  ASP B  92      29.940 -31.217 128.023  1.00182.96
C
ANISOU 7060  CG  ASP B  92    34882  18034  16600   2925  -4283
3240       C
ATOM   7061  OD1 ASP B  92      29.918 -31.653 126.852  1.00210.72
O
ANISOU 7061  OD1 ASP B  92    38432  21572  20061   2952  -4373
3183       O
ATOM   7062  OD2 ASP B  92      30.084 -31.964 129.015  1.00147.97
O
ANISOU 7062  OD2 ASP B  92    31078  13129  12013   2954  -4444
3134       O
ATOM   7063  N   TRP B  93      26.774 -29.760 127.452  1.00195.12
N
ANISOU 7063  N   TRP B  93    35442  20478  18216   1630  -3371
4003       N
ATOM   7064  CA  TRP B  93      25.501 -30.332 127.008  1.00189.50
C
ANISOU 7064  CA  TRP B  93    34941  19730  17330   1087  -3201
4162       C
```

ATOM   7065 C   TRP B 93   24.870 -29.593 125.826 1.00170.62
C
ANISOU 7065 C   TRP B 93   31910 17884 15035   886 -2958
4309   C
ATOM   7066 O   TRP B 93   24.545 -30.209 124.805 1.00196.97
O
ANISOU 7066 O   TRP B 93   35413 21168 18260   735 -2996
4290   O
ATOM   7067 CB TRP B 93   24.503 -30.341 128.169 1.00190.77
C
ANISOU 7067 CB TRP B 93   35264 19821 17402   649 -2984
4334   C
ATOM   7068 CG TRP B 93   24.871 -31.169 129.362 1.00195.63
C
ANISOU 7068 CG TRP B 93   36587 19876 17866   727 -3190
4211   C
ATOM   7069 CD1 TRP B 93   25.748 -32.213 129.408 1.00201.74
C
ANISOU 7069 CD1 TRP B 93   38029 20118 18503   1039 -3557
3962   C
ATOM   7070 CD2 TRP B 93   24.340 -31.031 130.686 1.00208.52
C
ANISOU 7070 CD2 TRP B 93   38346 21432 19451   487 -3049
4317   C
ATOM   7071 NE1 TRP B 93   25.805 -32.725 130.685 1.00222.80
N
ANISOU 7071 NE1 TRP B 93   41246 22374 21032   1002 -3653
3905   N
ATOM   7072 CE2 TRP B 93   24.949 -32.017 131.487 1.00220.39
C
ANISOU 7072 CE2 TRP B 93   40619 22339 20781   657 -3340
4126   C
ATOM   7073 CE3 TRP B 93   23.412 -30.163 131.272 1.00199.21
C
ANISOU 7073 CE3 TRP B 93   36704 20641 18348   166 -2715
4540   C
ATOM   7074 CZ2 TRP B 93   24.658 -32.160 132.845 1.00190.22
C
ANISOU 7074 CZ2 TRP B 93   37123 18293 16859   492 -3298
4162   C
ATOM   7075 CZ3 TRP B 93   23.126 -30.305 132.619 1.00199.63
C
ANISOU 7075 CZ3 TRP B 93   37060 20480 18311   16 -2675
4576   C
ATOM   7076 CH2 TRP B 93   23.747 -31.295 133.390 1.00176.20
C
ANISOU 7076 CH2 TRP B 93   34867 16911 15170   168 -2960
4394   C
ATOM   7077 N   GLN B 94   24.689 -28.276 125.944 1.00165.45
N
ANISOU 7077 N   GLN B 94   30535 17752 14576   881 -2717
4446   N
ATOM   7078 CA GLN B 94   23.919 -27.533 124.942 1.00181.16
C

```
ANISOU 7078  CA  GLN B  94    31932  20272  16630    637  -2457
4591       C
ATOM   7079  C   GLN B  94      24.556 -27.511 123.552  1.00169.11
C
ANISOU 7079  C   GLN B  94    30211  18895  15147    897  -2595
4470       C
ATOM   7080  O   GLN B  94      23.858 -27.694 122.548  1.00159.37
O
ANISOU 7080  O   GLN B  94    28896  17820  13837    628  -2486
4528       O
ATOM   7081  CB  GLN B  94      23.671 -26.104 125.430  1.00184.19
C
ANISOU 7081  CB  GLN B  94    31616  21162  17208    628  -2207
4735       C
ATOM   7082  CG  GLN B  94      22.720 -26.020 126.617  1.00184.70
C
ANISOU 7082  CG  GLN B  94    31758  21204  17215    292  -2001
4886       C
ATOM   7083  CD  GLN B  94      21.451 -26.823 126.406  1.00202.24
C
ANISOU 7083  CD  GLN B  94    34298  23320  19222   -230  -1851
4971       C
ATOM   7084  OE1 GLN B  94      21.196 -27.794 127.118  1.00195.04
O
ANISOU 7084  OE1 GLN B  94    34003  21968  18137   -427  -1925
4949       O
ATOM   7085  NE2 GLN B  94      20.647 -26.421 125.428  1.00207.05
N
ANISOU 7085  NE2 GLN B  94    34496  24337  19836   -471  -1640
5051       N
ATOM   7086  N   ASP B  95      25.864 -27.276 123.459  1.00170.62
N
ANISOU 7086  N   ASP B  95    30306  19063  15457   1411  -2829
4288       N
ATOM   7087  CA  ASP B  95      26.497 -27.216 122.141  1.00136.45
C
ANISOU 7087  CA  ASP B  95    25760  14918  11166   1677  -2962
4153       C
ATOM   7088  C   ASP B  95      26.435 -28.539 121.378  1.00127.71
C
ANISOU 7088  C   ASP B  95    25229  13433   9862   1643  -3167
4039       C
ATOM   7089  O   ASP B  95      26.172 -28.544 120.170  1.00126.56
O
ANISOU 7089  O   ASP B  95    24893  13507   9687   1558  -3129
4044       O
ATOM   7090  CB  ASP B  95      27.946 -26.750 122.276  1.00138.50
C
ANISOU 7090  CB  ASP B  95    25813  15231  11582   2240  -3180
3941       C
ATOM   7091  CG  ASP B  95      28.052 -25.298 122.696  1.00139.55
C
ANISOU 7091  CG  ASP B  95    25272  15825  11926   2261  -2976
4048       C
```

```
ATOM   7092  OD1 ASP B  95      29.133 -24.895 123.174  1.00181.79
O
ANISOU 7092  OD1 ASP B  95    30493  21176  17403   2640  -3116
3897       O
ATOM   7093  OD2 ASP B  95      27.057 -24.558 122.538  1.00116.49
O
ANISOU 7093  OD2 ASP B  95    21949  13272   9040   1897  -2682
4266       O
ATOM   7094  N   PHE B  96      26.686 -29.666 122.046  1.00132.06
N
ANISOU 7094  N   PHE B  96    26495  13412  10268   1712  -3396
3923       N
ATOM   7095  CA  PHE B  96      26.584 -30.955 121.362  1.00135.66
C
ANISOU 7095  CA  PHE B  96    27543  13477  10525   1658  -3605
3813       C
ATOM   7096  C   PHE B  96      25.181 -31.231 120.825  1.00135.92
C
ANISOU 7096  C   PHE B  96    27615  13599  10430   1044  -3351
4019       C
ATOM   7097  O   PHE B  96      25.019 -31.611 119.659  1.00190.64
O
ANISOU 7097  O   PHE B  96    34557  20583  17293    986  -3398
3984       O
ATOM   7098  CB  PHE B  96      27.027 -32.071 122.311  1.00140.50
C
ANISOU 7098  CB  PHE B  96    28949  13448  10988   1804  -3887
3654       C
ATOM   7099  CG  PHE B  96      26.858 -33.459 121.753  1.00144.74
C
ANISOU 7099  CG  PHE B  96    30180  13521  11295   1713  -4115
3542       C
ATOM   7100  CD1 PHE B  96      27.871 -34.049 121.016  1.00146.25
C
ANISOU 7100  CD1 PHE B  96    30572  13536  11460   2208  -4472
3264       C
ATOM   7101  CD2 PHE B  96      25.706 -34.189 122.001  1.00147.49
C
ANISOU 7101  CD2 PHE B  96    30984  13610  11447   1137  -3985
3693       C
ATOM   7102  CE1 PHE B  96      27.730 -35.330 120.515  1.00150.27
C
ANISOU 7102  CE1 PHE B  96    31735  13605  11755   2143  -4703
3152       C
ATOM   7103  CE2 PHE B  96      25.559 -35.470 121.503  1.00151.56
C
ANISOU 7103  CE2 PHE B  96    32161  13681  11743   1032  -4203
3587       C
ATOM   7104  CZ  PHE B  96      26.574 -36.042 120.762  1.00152.91
C
ANISOU 7104  CZ  PHE B  96    32546  13661  11893   1544  -4570
3321       C
ATOM   7105  N   VAL B  97      24.152 -31.052 121.655  1.00136.20
N
```

ANISOU 7105 N   VAL B 97   27655 13668 10426   582 -3082
4217   N
ATOM   7106 CA VAL B 97   22.776 -31.195 121.177 1.00136.33
C
ANISOU 7106 CA VAL B 97   27610 13853 10337   -17 -2804
4392   C
ATOM   7107 C   VAL B 97   22.394 -30.099 120.181 1.00136.44
C
ANISOU 7107 C   VAL B 97   26847 14505 10491   -90 -2557
4492   C
ATOM   7108 O   VAL B 97   21.725 -30.369 119.178 1.00173.05
O
ANISOU 7108 O   VAL B 97   31435 19269 15048   -381 -2462
4527   O
ATOM   7109 CB VAL B 97   21.791 -31.268 122.360 1.00138.10
C
ANISOU 7109 CB VAL B 97   28006 13988 10478   -469 -2585
4540   C
ATOM   7110 CG1 VAL B 97   22.145 -32.440 123.264 1.00143.09
C
ANISOU 7110 CG1 VAL B 97   29476 13959 10933   -429 -2845
4424   C
ATOM   7111 CG2 VAL B 97   21.768 -29.968 123.149 1.00134.59
C
ANISOU 7111 CG2 VAL B 97   26963 13950 10226   -378 -2373
4652   C
ATOM   7112 N   GLY B 98   22.839 -28.863 120.414 1.00127.85
N
ANISOU 7112 N   GLY B 98   25157 13816   9605   175 -2463
4523   N
ATOM   7113 CA GLY B 98   22.491 -27.769 119.514 1.00140.92
C
ANISOU 7113 CA GLY B 98   26090 16072 11380   115 -2238
4603   C
ATOM   7114 C   GLY B 98   22.983 -27.931 118.087 1.00123.99
C
ANISOU 7114 C   GLY B 98   23851 14035   9226   310 -2383
4481   C
ATOM   7115 O   GLY B 98   22.225 -27.740 117.133 1.00123.73
O
ANISOU 7115 O   GLY B 98   23549 14303   9160   23 -2204
4545   O
ATOM   7116 N   ILE B 99   24.261 -28.266 117.918 1.00123.47
N
ANISOU 7116 N   ILE B 99   23980 13748   9187   816 -2710
4281   N
ATOM   7117 CA ILE B 99   24.798 -28.522 116.581 1.00123.07
C
ANISOU 7117 CA ILE B 99   23874 13779   9109   1050 -2886
4129   C
ATOM   7118 C   ILE B 99   24.089 -29.695 115.907 1.00150.13
C
ANISOU 7118 C   ILE B 99   27786 16921 12337   722 -2930
4134   C

```
ATOM   7119  O   ILE B  99      23.709 -29.618 114.733  1.00178.99
O
ANISOU 7119  O   ILE B  99    31216  20833  15960    578  -2854
4142       O
ATOM   7120  CB  ILE B  99      26.320 -28.744 116.652  1.00124.04
C
ANISOU 7120  CB  ILE B  99    24129  13710   9292   1690  -3249
3866       C
ATOM   7121  CG1 ILE B  99      27.010 -27.493 117.200  1.00120.83
C
ANISOU 7121  CG1 ILE B  99    23171  13647   9094   1968  -3180
3857       C
ATOM   7122  CG2 ILE B  99      26.873 -29.106 115.284  1.00159.04
C
ANISOU 7122  CG2 ILE B  99    28520  18227  13681   1960  -3455
3674       C
ATOM   7123  CD1 ILE B  99      28.518 -27.587 117.229  1.00121.61
C
ANISOU 7123  CD1 ILE B  99    23287  13651   9269   2587  -3505
3567       C
ATOM   7124  N   ILE B 100      23.894 -30.793 116.637  1.00130.25
N
ANISOU 7124  N   ILE B 100    25950  13864   9675    582  -3055
4121       N
ATOM   7125  CA  ILE B 100      23.116 -31.918 116.116  1.00133.41
C
ANISOU 7125  CA  ILE B 100    26843  13970   9875    191  -3076
4142       C
ATOM   7126  C   ILE B 100      21.671 -31.529 115.804  1.00131.96
C
ANISOU 7126  C   ILE B 100    26332  14142   9666   -440  -2680
4350       C
ATOM   7127  O   ILE B 100      21.107 -31.970 114.796  1.00134.01
O
ANISOU 7127  O   ILE B 100    26637  14451   9830   -711  -2637
4355       O
ATOM   7128  CB  ILE B 100      23.189 -33.108 117.090  1.00138.38
C
ANISOU 7128  CB  ILE B 100    28285  13947  10346    143  -3281
4081       C
ATOM   7129  CG1 ILE B 100      24.585 -33.729 117.040  1.00140.44
C
ANISOU 7129  CG1 ILE B 100    28923  13841  10597    782  -3724
3808       C
ATOM   7130  CG2 ILE B 100      22.126 -34.151 116.761  1.00141.69
C
ANISOU 7130  CG2 ILE B 100    29183  14100  10552   -414  -3218
4149       C
ATOM   7131  CD1 ILE B 100      24.838 -34.724 118.130  1.00158.44
C
ANISOU 7131  CD1 ILE B 100    31963  15498  12738    832  -3948
3713       C
ATOM   7132  N   CYS B 101      21.045 -30.708 116.651  1.00141.29
N
```

ANISOU 7132 N   CYS B 101   27165 15588 10931   -672 -2393
4502   N
ATOM   7133 CA CYS B 101   19.723 -30.178 116.312 1.00147.83
C
ANISOU 7133 CA CYS B 101   27560 16847 11761 -1193 -2019
4651   C
ATOM   7134 C   CYS B 101   19.710 -29.422 114.985 1.00146.05
C
ANISOU 7134 C   CYS B 101   26749 17118 11624 -1127 -1922
4635   C
ATOM   7135 O   CYS B 101   18.789 -29.599 114.179 1.00171.21
O
ANISOU 7135 O   CYS B 101   29824 20485 14743 -1528 -1751
4670   O
ATOM   7136 CB CYS B 101   19.202 -29.276 117.434 1.00147.99
C
ANISOU 7136 CB CYS B 101   27229 17120 11880 -1322 -1762
4779   C
ATOM   7137 SG CYS B 101   18.734 -30.139 118.954 1.00211.29
S
ANISOU 7137 SG CYS B 101   35880 24648 19751 -1612 -1767
4827   S
ATOM   7138 N   LEU B 102   20.712 -28.578 114.735 1.00123.83
N
ANISOU 7138 N   LEU B 102   23553 14541   8957   -644 -2026
4566   N
ATOM   7139 CA LEU B 102   20.828 -27.937 113.425 1.00118.67
C
ANISOU 7139 CA LEU B 102   22408 14319   8361   -555 -1979
4519   C
ATOM   7140 C   LEU B 102   20.973 -28.951 112.293 1.00139.34
C
ANISOU 7140 C   LEU B 102   25379 16721 10842   -562 -2177
4406   C
ATOM   7141 O   LEU B 102   20.276 -28.862 111.275 1.00150.46
O
ANISOU 7141 O   LEU B 102   26484 18416 12269   -849 -2006
4398   O
ATOM   7142 CB LEU B 102   22.001 -26.949 113.429 1.00115.62
C
ANISOU 7142 CB LEU B 102   21618 14177   8137   -20 -2094
4434   C
ATOM   7143 CG LEU B 102   22.314 -26.108 112.177 1.00114.06
C
ANISOU 7143 CG LEU B 102   20875 14455   8008   138 -2062
4363   C
ATOM   7144 CD1 LEU B 102   23.173 -24.923 112.557 1.00109.17
C
ANISOU 7144 CD1 LEU B 102   19795 14126   7560   524 -2070
4329   C
ATOM   7145 CD2 LEU B 102   23.027 -26.889 111.071 1.00115.10
C
ANISOU 7145 CD2 LEU B 102   21035 14472   8225   398 -2273
4082   C

```
ATOM   7146  N   LEU B 103      21.881 -29.917 112.445  1.00123.60
N
ANISOU 7146  N   LEU B 103    23946  14242   8774   -216  -2530
4264       N
ATOM   7147  CA  LEU B 103      22.055 -30.951 111.425  1.00150.13
C
ANISOU 7147  CA  LEU B 103    27573  17382  12087   -169  -2722
4085       C
ATOM   7148  C   LEU B 103      20.779 -31.752 111.172  1.00161.70
C
ANISOU 7148  C   LEU B 103    29366  18694  13379   -792  -2566
4205       C
ATOM   7149  O   LEU B 103      20.430 -32.020 110.016  1.00130.51
O
ANISOU 7149  O   LEU B 103    25180  14901   9506   -936  -2500
4093       O
ATOM   7150  CB  LEU B 103      23.203 -31.880 111.822  1.00148.11
C
ANISOU 7150  CB  LEU B 103    27942  16592  11741    325  -3156
3911       C
ATOM   7151  CG  LEU B 103      24.591 -31.232 111.789  1.00127.19
C
ANISOU 7151  CG  LEU B 103    24916  14132   9278    988  -3347
3700       C
ATOM   7152  CD1 LEU B 103      25.646 -32.163 112.370  1.00130.86
C
ANISOU 7152  CD1 LEU B 103    26054  14052   9613   1475  -3788
3528       C
ATOM   7153  CD2 LEU B 103      24.957 -30.807 110.375  1.00124.71
C
ANISOU 7153  CD2 LEU B 103    23907  14280   9199   1165  -3288
3474       C
ATOM   7154  N   VAL B 104      20.075 -32.149 112.233  1.00171.20
N
ANISOU 7154  N   VAL B 104    30975  19626  14448  -1164  -2467
4358       N
ATOM   7155  CA  VAL B 104      18.803 -32.854 112.070  1.00133.08
C
ANISOU 7155  CA  VAL B 104    26382  14705   9476  -1805  -2279
4446       C
ATOM   7156  C   VAL B 104      17.782 -32.003 111.320  1.00140.82
C
ANISOU 7156  C   VAL B 104    26686  16291  10529  -2192  -1913
4534       C
ATOM   7157  O   VAL B 104      17.097 -32.487 110.410  1.00177.87
O
ANISOU 7157  O   VAL B 104    31412  21038  15133  -2541  -1838
4526       O
ATOM   7158  CB  VAL B 104      18.268 -33.304 113.443  1.00136.20
C
ANISOU 7158  CB  VAL B 104    27178  14779   9793  -2108  -2200
4527       C
ATOM   7159  CG1 VAL B 104      16.805 -33.717 113.338  1.00138.11
C
```

ANISOU 7159 CG1 VAL B 104   27457 15099   9917 -2822 -1918
4616   C
ATOM   7160 CG2 VAL B 104   19.114 -34.447 113.990 1.00143.96
C
ANISOU 7160 CG2 VAL B 104   28962 15086 10650 -1821 -2585
4408   C
ATOM   7161 N   ILE B 105   17.669 -30.723 111.681 1.00126.35
N
ANISOU 7161 N   ILE B 105   24228 14919   8860 -2120 -1691
4602   N
ATOM   7162 CA ILE B 105   16.829 -29.787 110.932 1.00127.12
C
ANISOU 7162 CA ILE B 105   23631 15621   9050 -2381 -1379
4642   C
ATOM   7163 C   ILE B 105   17.321 -29.602 109.499 1.00140.21
C
ANISOU 7163 C   ILE B 105   24910 17516 10849 -2132 -1455
4474   C
ATOM   7164 O   ILE B 105   16.521 -29.582 108.554 1.00144.78
O
ANISOU 7164 O   ILE B 105   25162 18374 11473 -2437 -1272
4428   O
ATOM   7165 CB ILE B 105   16.743 -28.441 111.678 1.00119.87
C
ANISOU 7165 CB ILE B 105   22142 15101   8302 -2254 -1174
4710   C
ATOM   7166 CG1 ILE B 105   16.011 -28.620 113.008 1.00122.09
C
ANISOU 7166 CG1 ILE B 105   22614 15230   8545 -2537 -1026
4798   C
ATOM   7167 CG2 ILE B 105   16.043 -27.393 110.829 1.00116.38
C
ANISOU 7167 CG2 ILE B 105   20971 15277   7971 -2405   -906
4704   C
ATOM   7168 CD1 ILE B 105   14.567 -29.055 112.858 1.00147.03
C
ANISOU 7168 CD1 ILE B 105   25762 18496 11607 -3152   -775
4821   C
ATOM   7169 N   ASN B 106   18.633 -29.456 109.311 1.00119.92
N
ANISOU 7169 N   ASN B 106   22269 14869   8427 -1549 -1701
4317   N
ATOM   7170 CA ASN B 106   19.192 -29.334 107.965 1.00118.23
C
ANISOU 7170 CA ASN B 106   21619 14880   8422 -1264 -1766
4082   C
ATOM   7171 C   ASN B 106   18.821 -30.510 107.064 1.00120.95
C
ANISOU 7171 C   ASN B 106   22257 14987   8710 -1472 -1841
3969   C
ATOM   7172 O   ASN B 106   18.393 -30.314 105.922 1.00119.45
O
ANISOU 7172 O   ASN B 106   21631 15116   8640 -1603 -1700
3876   O

```
ATOM   7173  CB  ASN B 106      20.713 -29.196 108.051  1.00139.98
C
ANISOU 7173  CB  ASN B 106    24362  17530  11293   -619  -2052
3910       C
ATOM   7174  CG  ASN B 106      21.373 -29.163 106.687  1.00136.91
C
ANISOU 7174  CG  ASN B 106    23566  17359  11095   -321  -2136
3644       C
ATOM   7175  OD1 ASN B 106      20.853 -28.562 105.747  1.00113.99
O
ANISOU 7175  OD1 ASN B 106    20121  14877   8312   -497  -1917
3616       O
ATOM   7176  ND2 ASN B 106      22.522 -29.820 106.569  1.00151.92
N
ANISOU 7176  ND2 ASN B 106    25734  18978  13011    142  -2462
3433       N
ATOM   7177  N   SER B 107      18.967 -31.740 107.559  1.00125.06
N
ANISOU 7177  N   SER B 107    23543  14936   9036  -1505  -2074
3976       N
ATOM   7178  CA  SER B 107      18.554 -32.910 106.783  1.00128.00
C
ANISOU 7178  CA  SER B 107    24273  15041   9321  -1739  -2157
3888       C
ATOM   7179  C   SER B 107      17.058 -32.917 106.477  1.00145.09
C
ANISOU 7179  C   SER B 107    26292  17429  11406  -2410  -1832
4021       C
ATOM   7180  O   SER B 107      16.651 -33.230 105.352  1.00179.92
O
ANISOU 7180  O   SER B 107    30511  21967  15885  -2564  -1776
3911       O
ATOM   7181  CB  SER B 107      18.946 -34.190 107.519  1.00143.08
C
ANISOU 7181  CB  SER B 107    27113  16260  10990  -1673  -2480
3892       C
ATOM   7182  OG  SER B 107      20.309 -34.507 107.297  1.00181.40
O
ANISOU 7182  OG  SER B 107    32099  20902  15924  -1029  -2837
3663       O
ATOM   7183  N   THR B 108      16.226 -32.583 107.463  1.00164.67
N
ANISOU 7183  N   THR B 108    28854  19978  13737  -2811  -1618
4243       N
ATOM   7184  CA  THR B 108      14.778 -32.552 107.258  1.00188.78
C
ANISOU 7184  CA  THR B 108    31739  23296  16694  -3458  -1300
4348       C
ATOM   7185  C   THR B 108      14.361 -31.598 106.139  1.00186.48
C
ANISOU 7185  C   THR B 108    30604  23620  16628  -3468  -1069
4253       C
ATOM   7186  O   THR B 108      13.559 -31.960 105.272  1.00183.74
O
```

```
ANISOU 7186  O   THR B 108    30132  23404  16277  -3810   -946
4193       O
ATOM   7187  CB  THR B 108      14.076 -32.176 108.563  1.00225.01
C
ANISOU 7187  CB  THR B 108    36467  27933  21093  -3815  -1107
4579       C
ATOM   7188  OG1 THR B 108      14.444 -33.103 109.592  1.00240.17
O
ANISOU 7188  OG1 THR B 108    39119  29258  22876  -3782  -1314
4607       O
ATOM   7189  CG2 THR B 108      12.565 -32.206 108.383  1.00241.85
C
ANISOU 7189  CG2 THR B 108    38338  30367  23188  -4448   -775
4599       C
ATOM   7190  N   ILE B 109      14.896 -30.378 106.137  1.00162.92
N
ANISOU 7190  N   ILE B 109    27060  21011  13833  -3099  -1016
4233       N
ATOM   7191  CA  ILE B 109      14.580 -29.424 105.074  1.00135.74
C
ANISOU 7191  CA  ILE B 109    22860  18126  10588  -3072   -825
4135       C
ATOM   7192  C   ILE B 109      15.293 -29.747 103.760  1.00120.69
C
ANISOU 7192  C   ILE B 109    20797  16201   8860  -2751   -987
3905       C
ATOM   7193  O   ILE B 109      14.725 -29.548 102.680  1.00142.91
O
ANISOU 7193  O   ILE B 109    23208  19324  11769  -2907   -845
3811       O
ATOM   7194  CB  ILE B 109      14.876 -27.986 105.548  1.00113.85
C
ANISOU 7194  CB  ILE B 109    19590  15746   7922  -2819   -720
4201       C
ATOM   7195  CG1 ILE B 109      14.193 -26.969 104.629  1.00110.67
C
ANISOU 7195  CG1 ILE B 109    18476  15928   7645  -2930   -480
4141       C
ATOM   7196  CG2 ILE B 109      16.376 -27.724 105.655  1.00112.55
C
ANISOU 7196  CG2 ILE B 109    19425  15446   7892  -2211   -968
4108       C
ATOM   7197  CD1 ILE B 109      14.180 -25.560 105.179  1.00107.44
C
ANISOU 7197  CD1 ILE B 109    17630  15914   7279  -2796   -348
4235       C
ATOM   7198  N   SER B 110      16.534 -30.234 103.812  1.00117.90
N
ANISOU 7198  N   SER B 110    20742  15506   8549  -2291  -1287
3795       N
ATOM   7199  CA  SER B 110      17.265 -30.537 102.582  1.00117.47
C
ANISOU 7199  CA  SER B 110    20523  15459   8652  -1963  -1447
3556       C
```

```
ATOM   7200  C   SER B 110      16.585 -31.615 101.742  1.00135.50
C
ANISOU 7200  C   SER B 110    23054  17559  10870  -2283  -1459
3488       C
ATOM   7201  O   SER B 110      16.395 -31.444 100.532  1.00180.80
O
ANISOU 7201  O   SER B 110    28381  23568  16745  -2291  -1379
3354       O
ATOM   7202  CB  SER B 110      18.693 -30.966 102.924  1.00118.69
C
ANISOU 7202  CB  SER B 110    21006  15268   8823  -1413  -1789
3432       C
ATOM   7203  OG  SER B 110      19.435 -31.285 101.760  1.00121.19
O
ANISOU 7203  OG  SER B 110    21160  15612   9276  -1082  -1952
3180       O
ATOM   7204  N   PHE B 111      16.216 -32.732 102.359  1.00132.51
N
ANISOU 7204  N   PHE B 111    23368  16709  10271  -2556  -1563
3579       N
ATOM   7205  CA  PHE B 111      15.713 -33.877 101.608  1.00138.26
C
ANISOU 7205  CA  PHE B 111    24433  17185  10915  -2828  -1629
3506       C
ATOM   7206  C   PHE B 111      14.222 -33.839 101.286  1.00150.58
C
ANISOU 7206  C   PHE B 111    25803  19004  12407  -3464  -1319
3600       C
ATOM   7207  O   PHE B 111      13.807 -34.479 100.314  1.00190.26
O
ANISOU 7207  O   PHE B 111    30856  23993  17443  -3644  -1325
3499       O
ATOM   7208  CB  PHE B 111      16.082 -35.167 102.344  1.00131.39
C
ANISOU 7208  CB  PHE B 111    24461  15647   9814  -2812  -1927
3536       C
ATOM   7209  CG  PHE B 111      17.567 -35.391 102.417  1.00140.13
C
ANISOU 7209  CG  PHE B 111    25765  16492  10986  -2142  -2282
3370       C
ATOM   7210  CD1 PHE B 111      18.291 -35.664 101.268  1.00131.34
C
ANISOU 7210  CD1 PHE B 111    24469  15412  10023  -1758  -2461
3122       C
ATOM   7211  CD2 PHE B 111      18.243 -35.294 103.620  1.00165.40
C
ANISOU 7211  CD2 PHE B 111    29299  19448  14098  -1885  -2434
3444       C
ATOM   7212  CE1 PHE B 111      19.658 -35.855 101.321  1.00131.85
C
ANISOU 7212  CE1 PHE B 111    24668  15292  10138  -1133  -2787
2934       C
ATOM   7213  CE2 PHE B 111      19.612 -35.484 103.678  1.00148.61
C
```

```
ANISOU 7213  CE2 PHE B 111    27319  17118  12028  -1251  -2768
3259       C
ATOM   7214  CZ  PHE B 111      20.319 -35.766 102.527  1.00132.68
C
ANISOU 7214  CZ  PHE B 111    25100  15161  10152   -875  -2944
2995       C
ATOM   7215  N   ILE B 112      13.405 -33.118 102.057  1.00145.11
N
ANISOU 7215  N   ILE B 112    24910  18587  11639  -3799  -1055
3773       N
ATOM   7216  CA  ILE B 112      12.029 -32.863 101.627  1.00160.47
C
ANISOU 7216  CA  ILE B 112    26504  20915  13552  -4334   -745
3808       C
ATOM   7217  C   ILE B 112      11.992 -32.022 100.351  1.00170.46
C
ANISOU 7217  C   ILE B 112    27030  22673  15064  -4150   -629
3651       C
ATOM   7218  O   ILE B 112      11.206 -32.298  99.436  1.00173.79
O
ANISOU 7218  O   ILE B 112    27280  23249  15502  -4442   -516
3570       O
ATOM   7219  CB  ILE B 112      11.205 -32.226 102.767  1.00147.35
C
ANISOU 7219  CB  ILE B 112    24763  19480  11744  -4690   -501
4001       C
ATOM   7220  CG1 ILE B 112       9.713 -32.271 102.428  1.00167.25
C
ANISOU 7220  CG1 ILE B 112    27061  22324  14162  -5309   -213
4015       C
ATOM   7221  CG2 ILE B 112      11.646 -30.795 103.066  1.00129.94
C
ANISOU 7221  CG2 ILE B 112    21997  17678   9697  -4326   -416
4028       C
ATOM   7222  CD1 ILE B 112       9.172 -33.670 102.215  1.00178.27
C
ANISOU 7222  CD1 ILE B 112    29033  23334  15367  -5753   -274
4007       C
ATOM   7223  N   GLU B 113      12.829 -30.985 100.268  1.00150.10
N
ANISOU 7223  N   GLU B 113    24020  20344  12667  -3679   -656
3602       N
ATOM   7224  CA  GLU B 113      12.869 -30.149  99.070  1.00120.39
C
ANISOU 7224  CA  GLU B 113    19599  17025   9117  -3496   -559
3453       C
ATOM   7225  C   GLU B 113      13.341 -30.928  97.846  1.00115.98
C
ANISOU 7225  C   GLU B 113    19112  16293   8662  -3319   -730
3258       C
ATOM   7226  O   GLU B 113      12.753 -30.822  96.764  1.00135.78
O
ANISOU 7226  O   GLU B 113    21279  19058  11253  -3468   -611
3154       O
```

```
ATOM   7227  CB  GLU B 113      13.784 -28.945  99.313  1.00111.67
C
ANISOU 7227  CB  GLU B 113    18109  16169   8153  -3039   -580
3446       C
ATOM   7228  CG  GLU B 113      13.591 -27.783  98.349  1.00107.98
C
ANISOU 7228  CG  GLU B 113    16947  16232   7848  -2947   -417
3348       C
ATOM   7229  CD  GLU B 113      12.385 -26.929  98.683  1.00106.73
C
ANISOU 7229  CD  GLU B 113    16458  16478   7617  -3294   -144
3456       C
ATOM   7230  OE1 GLU B 113      12.199 -26.599  99.873  1.00106.96
O
ANISOU 7230  OE1 GLU B 113    16606  16502   7531  -3382    -87
3621       O
ATOM   7231  OE2 GLU B 113      11.626 -26.581  97.755  1.00139.01
O
ANISOU 7231  OE2 GLU B 113    20164  20898  11757  -3463      4
3365       O
ATOM   7232  N   GLU B 114      14.406 -31.718  98.000  1.00117.81
N
ANISOU 7232  N   GLU B 114    19789  16093   8881  -2982  -1022
3192       N
ATOM   7233  CA  GLU B 114      14.815 -32.645  96.946  1.00119.34
C
ANISOU 7233  CA  GLU B 114    20155  16061   9129  -2830  -1216
3008       C
ATOM   7234  C   GLU B 114      13.753 -33.695  96.630  1.00126.71
C
ANISOU 7234  C   GLU B 114    21440  16785   9921  -3333  -1172
3037       C
ATOM   7235  O   GLU B 114      13.528 -34.016  95.457  1.00175.23
O
ANISOU 7235  O   GLU B 114    27424  23004  16151  -3373  -1170
2898       O
ATOM   7236  CB  GLU B 114      16.136 -33.313  97.332  1.00121.17
C
ANISOU 7236  CB  GLU B 114    20844  15861   9335  -2358  -1563
2922       C
ATOM   7237  CG  GLU B 114      17.338 -32.391  97.175  1.00129.22
C
ANISOU 7237  CG  GLU B 114    21430  17135  10534  -1807  -1636
2798       C
ATOM   7238  CD  GLU B 114      18.651 -33.140  97.063  1.00126.49
C
ANISOU 7238  CD  GLU B 114    21413  16451  10196  -1297  -1994
2608       C
ATOM   7239  OE1 GLU B 114      18.631 -34.387  97.129  1.00123.74
O
ANISOU 7239  OE1 GLU B 114    21672  15637   9707  -1347  -2206
2580       O
ATOM   7240  OE2 GLU B 114      19.700 -32.479  96.907  1.00118.09
O
```

```
ANISOU 7240  OE2 GLU B 114    20007  15599   9265   -847  -2070
2474       O
ATOM   7241  N   ASN B 115      13.098 -34.253  97.650  1.00129.60
N
ANISOU 7241  N   ASN B 115    22296  16886  10060  -3736  -1136
3211       N
ATOM   7242  CA  ASN B 115      11.953 -35.128  97.399  1.00136.73
C
ANISOU 7242  CA  ASN B 115    23483  17661  10806  -4306  -1044
3249       C
ATOM   7243  C   ASN B 115      10.851 -34.423  96.615  1.00148.08
C
ANISOU 7243  C   ASN B 115    24285  19642  12336  -4631   -732
3215       C
ATOM   7244  O   ASN B 115      10.301 -34.982  95.659  1.00156.38
O
ANISOU 7244  O   ASN B 115    25317  20700  13401  -4850   -709
3117       O
ATOM   7245  CB  ASN B 115      11.400 -35.665  98.720  1.00150.26
C
ANISOU 7245  CB  ASN B 115    25779  19073  12239  -4729  -1013
3451       C
ATOM   7246  CG  ASN B 115      10.171 -36.533  98.525  1.00154.29
C
ANISOU 7246  CG  ASN B 115    26578  19486  12558  -5385   -893
3493       C
ATOM   7247  OD1 ASN B 115      10.274 -37.695  98.130  1.00155.92
O
ANISOU 7247  OD1 ASN B 115    27307  19268  12666  -5458  -1094
3436       O
ATOM   7248  ND2 ASN B 115       8.999 -35.971  98.800  1.00142.39
N
ANISOU 7248  ND2 ASN B 115    24732  18387  10985  -5866   -570
3579       N
ATOM   7249  N   ASN B 116      10.505 -33.197  97.012  1.00158.49
N
ANISOU 7249  N   ASN B 116    25094  21415  13709  -4653   -505
3285       N
ATOM   7250  CA  ASN B 116       9.500 -32.434  96.276  1.00166.97
C
ANISOU 7250  CA  ASN B 116    25550  23026  14864  -4896   -236
3226       C
ATOM   7251  C   ASN B 116       9.940 -32.140  94.844  1.00182.82
C
ANISOU 7251  C   ASN B 116    27139  25224  17102  -4563   -288
3027       C
ATOM   7252  O   ASN B 116       9.153 -32.289  93.902  1.00202.14
O
ANISOU 7252  O   ASN B 116    29364  27858  19582  -4807   -178
2928       O
ATOM   7253  CB  ASN B 116       9.187 -31.139  97.028  1.00156.16
C
ANISOU 7253  CB  ASN B 116    23757  22081  13496  -4898    -32
3328       C
```

ATOM   7254 CG ASN B 116   7.947 -30.441 96.501 1.00171.11
C
ANISOU 7254 CG ASN B 116   25103 24512 15400 -5220   243
3273   C
ATOM   7255 OD1 ASN B 116   7.187 -31.006 95.716 1.00165.68
O
ANISOU 7255 OD1 ASN B 116   24391 23869 14692 -5528   307
3180   O
ATOM   7256 ND2 ASN B 116   7.739 -29.202 96.933 1.00164.95
N
ANISOU 7256 ND2 ASN B 116   23887 24143 14642 -5134   393
3320   N
ATOM   7257 N   ALA B 117   11.193 -31.716 94.663 1.00174.71
N
ANISOU 7257 N   ALA B 117   25994 24165 16225 -4014   -452
2955   N
ATOM   7258 CA ALA B 117   11.754 -31.540 93.324 1.00163.42
C
ANISOU 7258 CA ALA B 117   24230 22871 14992 -3686   -525
2756   C
ATOM   7259 C   ALA B 117   11.782 -32.841 92.524 1.00167.21
C
ANISOU 7259 C   ALA B 117   25077 23000 15454 -3747   -692
2644   C
ATOM   7260 O   ALA B 117   11.442 -32.853 91.335 1.00162.33
O
ANISOU 7260 O   ALA B 117   24176 22561 14940 -3787   -638
2508   O
ATOM   7261 CB ALA B 117   13.160 -30.948 93.426 1.00146.73
C
ANISOU 7261 CB ALA B 117   21982 20765 13003 -3118   -680
2696   C
ATOM   7262 N   GLY B 118   12.176 -33.947 93.158 1.00175.24
N
ANISOU 7262 N   GLY B 118   26752 23503 16329 -3747   -910
2696   N
ATOM   7263 CA GLY B 118   12.139 -35.239 92.483 1.00163.46
C
ANISOU 7263 CA GLY B 118   25686 21640 14783 -3830 -1089
2601   C
ATOM   7264 C   GLY B 118   10.745 -35.693 92.091 1.00164.14
C
ANISOU 7264 C   GLY B 118   25795 21798 14774 -4422   -906
2633   C
ATOM   7265 O   GLY B 118   10.553 -36.280 91.022 1.00164.79
O
ANISOU 7265 O   GLY B 118   25882 21823 14908 -4467   -961
2505   O
ATOM   7266 N   ASN B 119   9.756 -35.434 92.947 1.00189.74
N
ANISOU 7266 N   ASN B 119   29041 25181 17870 -4884   -686
2792   N
ATOM   7267 CA ASN B 119   8.367 -35.724 92.601 1.00189.24
C

```
ANISOU 7267  CA  ASN B 119    28902  25288  17713  -5471   -476
2800       C
ATOM   7268  C   ASN B 119       7.906 -34.936  91.376  1.00163.89
C
ANISOU 7268  C   ASN B 119    24995  22578  14698  -5424   -306
2648       C
ATOM   7269  O   ASN B 119       7.236 -35.484  90.493  1.00176.35
O
ANISOU 7269  O   ASN B 119    26552  24173  16278  -5682   -270
2553       O
ATOM   7270  CB  ASN B 119       7.469 -35.430  93.806  1.00174.44
C
ANISOU 7270  CB  ASN B 119    27080  23557  15644  -5933   -258
2975       C
ATOM   7271  CG  ASN B 119       6.074 -35.998  93.648  1.00160.38
C
ANISOU 7271  CG  ASN B 119    25367  21871  13697  -6602    -72
2981       C
ATOM   7272  OD1 ASN B 119       5.820 -36.810  92.760  1.00181.15
O
ANISOU 7272  OD1 ASN B 119    28152  24346  16332  -6746   -144
2881       O
ATOM   7273  ND2 ASN B 119       5.158 -35.572  94.513  1.00141.91
N
ANISOU 7273  ND2 ASN B 119    22908  19807  11206  -7021    168
3088       N
ATOM   7274  N   ALA B 120       8.252 -33.649  91.308  1.00151.29
N
ANISOU 7274  N   ALA B 120    22848  21378  13255  -5102   -209
2622       N
ATOM   7275  CA  ALA B 120       8.045 -32.866  90.090  1.00152.93
C
ANISOU 7275  CA  ALA B 120    22447  22009  13651  -4958   -100
2462       C
ATOM   7276  C   ALA B 120       8.847 -33.396  88.901  1.00180.66
C
ANISOU 7276  C   ALA B 120    26005  25329  17310  -4616   -301
2295       C
ATOM   7277  O   ALA B 120       8.339 -33.439  87.774  1.00178.28
O
ANISOU 7277  O   ALA B 120    25446  25199  17092  -4704   -239
2162       O
ATOM   7278  CB  ALA B 120       8.394 -31.401  90.351  1.00143.64
C
ANISOU 7278  CB  ALA B 120    20771  21231  12574  -4661      5
2480       C
ATOM   7279  N   ALA B 121      10.098 -33.805  89.126  1.00180.20
N
ANISOU 7279  N   ALA B 121    26265  24927  17277  -4212   -549
2283       N
ATOM   7280  CA  ALA B 121      10.969 -34.245  88.036  1.00167.43
C
ANISOU 7280  CA  ALA B 121    24658  23165  15792  -3832   -751
2101       C
```

```
ATOM   7281  C   ALA B 121      10.621 -35.621  87.469  1.00145.68
C
ANISOU 7281  C   ALA B 121    22347  20042  12961  -4044   -889
2043       C
ATOM   7282  O   ALA B 121      11.088 -35.951  86.374  1.00145.86
O
ANISOU 7282  O   ALA B 121    22303  20019  13098  -3791  -1017
1875       O
ATOM   7283  CB  ALA B 121      12.425 -34.246  88.504  1.00196.20
C
ANISOU 7283  CB  ALA B 121    28485  26593  19469  -3316   -985
2075       C
ATOM   7284  N   ALA B 122       9.833 -36.430  88.178  1.00152.03
N
ANISOU 7284  N   ALA B 122    23617  20585  13562  -4507   -871
2175       N
ATOM   7285  CA  ALA B 122       9.371 -37.700  87.619  1.00158.15
C
ANISOU 7285  CA  ALA B 122    24818  21029  14243  -4777   -984
2128       C
ATOM   7286  C   ALA B 122       8.570 -37.514  86.330  1.00165.89
C
ANISOU 7286  C   ALA B 122    25354  22332  15345  -4951   -829
1996       C
ATOM   7287  O   ALA B 122       8.735 -38.281  85.374  1.00161.25
O
ANISOU 7287  O   ALA B 122    24921  21544  14800  -4864   -982
1870       O
ATOM   7288  CB  ALA B 122       8.540 -38.456  88.656  1.00160.23
C
ANISOU 7288  CB  ALA B 122    25621  21019  14239  -5332   -942
2302       C
ATOM   7289  N   ALA B 123       7.694 -36.508  86.286  1.00157.74
N
ANISOU 7289  N   ALA B 123    23780  21795  14360  -5180   -541
2011       N
ATOM   7290  CA  ALA B 123       6.997 -36.164  85.047  1.00132.22
C
ANISOU 7290  CA  ALA B 123    20072  18909  11255  -5273   -400
1863       C
ATOM   7291  C   ALA B 123       7.955 -35.745  83.933  1.00129.72
C
ANISOU 7291  C   ALA B 123    19440  18691  11157  -4736   -510
1693       C
ATOM   7292  O   ALA B 123       7.763 -36.120  82.770  1.00120.28
O
ANISOU 7292  O   ALA B 123    18155  17500  10047  -4727   -545
1552       O
ATOM   7293  CB  ALA B 123       5.978 -35.056  85.315  1.00129.77
C
ANISOU 7293  CB  ALA B 123    19244  19126  10937  -5541    -99
1893       C
ATOM   7294  N   LEU B 124       8.982 -34.960  84.265  1.00158.68
N
```

```
ANISOU 7294  N   LEU B 124    22933  22450  14909  -4303   -560
1700       N
ATOM   7295  CA  LEU B 124       9.992 -34.573  83.280  1.00117.03
C
ANISOU 7295  CA  LEU B 124    17379  17273   9813  -3807   -666
1532       C
ATOM   7296  C   LEU B 124      10.725 -35.780  82.696  1.00132.98
C
ANISOU 7296  C   LEU B 124    19808  18876  11840  -3584   -945
1421       C
ATOM   7297  O   LEU B 124      10.930 -35.857  81.478  1.00150.96
O
ANISOU 7297  O   LEU B 124    21886  21231  14239  -3405   -989
1252       O
ATOM   7298  CB  LEU B 124      10.978 -33.592  83.923  1.00112.94
C
ANISOU 7298  CB  LEU B 124    16661  16905   9345  -3433   -676
1570       C
ATOM   7299  CG  LEU B 124      11.942 -32.776  83.056  1.00122.86
C
ANISOU 7299  CG  LEU B 124    17498  18416  10769  -2978   -704
1410       C
ATOM   7300  CD1 LEU B 124      12.374 -31.532  83.814  1.00141.15
C
ANISOU 7300  CD1 LEU B 124    19529  21004  13099  -2814   -605
1492       C
ATOM   7301  CD2 LEU B 124      13.168 -33.587  82.658  1.00151.53
C
ANISOU 7301  CD2 LEU B 124    21392  21744  14439  -2577   -981
1274       C
ATOM   7302  N   MET B 125      11.131 -36.728  83.542  1.00154.00
N
ANISOU 7302  N   MET B 125    23063  21090  14360  -3578  -1147
1505       N
ATOM   7303  CA  MET B 125      11.671 -37.997  83.054  1.00165.29
C
ANISOU 7303  CA  MET B 125    24968  22084  15751  -3408  -1437
1400       C
ATOM   7304  C   MET B 125      10.665 -38.769  82.200  1.00189.59
C
ANISOU 7304  C   MET B 125    28159  25076  18801  -3783  -1404
1357       C
ATOM   7305  O   MET B 125      11.022 -39.305  81.144  1.00198.21
O
ANISOU 7305  O   MET B 125    29281  26062  19970  -3568  -1555
1196       O
ATOM   7306  CB  MET B 125      12.131 -38.851  84.236  1.00160.63
C
ANISOU 7306  CB  MET B 125    25054  21008  14969  -3387  -1660
1513       C
ATOM   7307  CG  MET B 125      12.475 -40.289  83.876  1.00161.29
C
ANISOU 7307  CG  MET B 125    25755  20579  14950  -3295  -1979
1427       C
```

```
ATOM   7308  SD  MET B 125      13.774 -40.416  82.633  1.00183.84
S
ANISOU 7308  SD  MET B 125    28412  23461  17977  -2625  -2225
1141       S
ATOM   7309  CE  MET B 125      13.617 -42.136  82.157  1.00158.92
C
ANISOU 7309  CE  MET B 125    25989  19731  14664  -2714  -2538
1075       C
ATOM   7310  N   ALA B 126       9.408 -38.840  82.641  1.00183.20
N
ANISOU 7310  N   ALA B 126    27404  24328  17876  -4345  -1207
1488       N
ATOM   7311  CA  ALA B 126       8.360 -39.487  81.851  1.00188.46
C
ANISOU 7311  CA  ALA B 126    28124  24971  18513  -4748  -1145
1441       C
ATOM   7312  C   ALA B 126       8.175 -38.841  80.479  1.00169.38
C
ANISOU 7312  C   ALA B 126    25112  22940  16304  -4600  -1028
1267       C
ATOM   7313  O   ALA B 126       7.953 -39.541  79.484  1.00160.53
O
ANISOU 7313  O   ALA B 126    24083  21694  15218  -4641  -1114
1154       O
ATOM   7314  CB  ALA B 126       7.042 -39.475  82.626  1.00203.23
C
ANISOU 7314  CB  ALA B 126    30047  26951  20218  -5383   -912
1590       C
ATOM   7315  N   GLY B 127       8.258 -37.512  80.404  1.00158.97
N
ANISOU 7315  N   GLY B 127    23211  22077  15114  -4430   -843
1244       N
ATOM   7316  CA  GLY B 127       8.163 -36.834  79.119  1.00160.53
C
ANISOU 7316  CA  GLY B 127    22879  22624  15491  -4262   -746
1076       C
ATOM   7317  C   GLY B 127       9.198 -37.254  78.090  1.00148.07
C
ANISOU 7317  C   GLY B 127    21343  20886  14030  -3812   -965
906       C
ATOM   7318  O   GLY B 127       8.901 -37.292  76.892  1.00151.58
O
ANISOU 7318  O   GLY B 127    21567  21450  14576  -3797   -939
764       O
ATOM   7319  N   LEU B 128      10.413 -37.575  78.525  1.00122.30
N
ANISOU 7319  N   LEU B 128    18354  17366  10747  -3431  -1185
900       N
ATOM   7320  CA  LEU B 128      11.471 -38.007  77.617  1.00135.69
C
ANISOU 7320  CA  LEU B 128    20091  18931  12533  -2975  -1409
713       C
ATOM   7321  C   LEU B 128      11.666 -39.520  77.575  1.00147.95
C
```

```
ANISOU 7321  C   LEU B 128    22279  19961  13972  -2966  -1697
687       C
ATOM   7322  O   LEU B 128      12.548 -39.991  76.850  1.00166.70
O
ANISOU 7322  O   LEU B 128    24732  22206  16400  -2569  -1915
517       O
ATOM   7323  CB  LEU B 128      12.794 -37.313  77.980  1.00136.14
C
ANISOU 7323  CB  LEU B 128    19972  19108  12647  -2492  -1484
663       C
ATOM   7324  CG  LEU B 128      13.481 -37.532  79.335  1.00139.04
C
ANISOU 7324  CG  LEU B 128    20711  19224  12895  -2358  -1627
777       C
ATOM   7325  CD1 LEU B 128      14.285 -38.829  79.390  1.00126.40
C
ANISOU 7325  CD1 LEU B 128    19681  17143  11203  -2087  -1981
691       C
ATOM   7326  CD2 LEU B 128      14.366 -36.341  79.672  1.00140.64
C
ANISOU 7326  CD2 LEU B 128    20509  19750  13179  -2029  -1555
755       C
ATOM   7327  N   ALA B 129      10.884 -40.283  78.332  1.00130.11
N
ANISOU 7327  N   ALA B 129    20489  17407  11540  -3393  -1711
842       N
ATOM   7328  CA  ALA B 129      11.106 -41.720  78.429  1.00139.58
C
ANISOU 7328  CA  ALA B 129    22384  18062  12589  -3393  -2012
835       C
ATOM   7329  C   ALA B 129      10.981 -42.400  77.064  1.00145.21
C
ANISOU 7329  C   ALA B 129    23113  18687  13371  -3330  -2125
668       C
ATOM   7330  O   ALA B 129      10.103 -42.045  76.267  1.00133.54
O
ANISOU 7330  O   ALA B 129    21261  17489  11990  -3586  -1917
633       O
ATOM   7331  CB  ALA B 129      10.114 -42.344  79.412  1.00147.52
C
ANISOU 7331  CB  ALA B 129    23869  18805  13376  -3967  -1960
1037       C
ATOM   7332  N   PRO B 130      11.871 -43.369  76.758  1.00159.97
N
ANISOU 7332  N   PRO B 130    25411  20180  15191  -2960  -2467
547       N
ATOM   7333  CA  PRO B 130      11.747 -44.203  75.549  1.00177.24
C
ANISOU 7333  CA  PRO B 130    27731  22203  17408  -2908  -2620
399       C
ATOM   7334  C   PRO B 130      10.339 -44.677  75.218  1.00193.90
C
ANISOU 7334  C   PRO B 130    29958  24255  19459  -3507  -2481
482       C
```

```
ATOM   7335  O   PRO B 130       9.568 -45.024  76.118  1.00205.38
O
ANISOU 7335  O   PRO B 130    31762  25534  20740  -3985  -2415
661       O
ATOM   7336  CB  PRO B 130      12.666 -45.389  75.868  1.00162.00
C
ANISOU 7336  CB  PRO B 130    26494  19741  15317  -2574  -3037
338       C
ATOM   7337  CG  PRO B 130      13.743 -44.772  76.690  1.00140.43
C
ANISOU 7337  CG  PRO B 130    23654  17105  12597  -2167  -3092
327       C
ATOM   7338  CD  PRO B 130      13.024 -43.777  77.577  1.00138.53
C
ANISOU 7338  CD  PRO B 130    23112  17154  12368  -2559  -2753
533       C
ATOM   7339  N   LYS B 131       9.994 -44.699  73.931  1.00196.53
N
ANISOU 7339  N   LYS B 131    30002  24745  19925  -3498  -2434
345       N
ATOM   7340  CA  LYS B 131       8.659 -45.063  73.478  1.00193.25
C
ANISOU 7340  CA  LYS B 131    29608  24339  19477  -4042  -2289
388       C
ATOM   7341  C   LYS B 131       8.726 -46.206  72.472  1.00186.64
C
ANISOU 7341  C   LYS B 131    29128  23167  18620  -3950  -2546
262       C
ATOM   7342  O   LYS B 131       9.658 -46.290  71.665  1.00185.08
O
ANISOU 7342  O   LYS B 131    28834  22959  18529  -3432  -2726
86       O
ATOM   7343  CB  LYS B 131       7.940 -43.868  72.849  1.00184.07
C
ANISOU 7343  CB  LYS B 131    27695  23743  18499  -4182  -1945
342       C
ATOM   7344  CG  LYS B 131       7.457 -42.844  73.856  1.00171.47
C
ANISOU 7344  CG  LYS B 131    25795  22472  16882  -4436  -1668
486       C
ATOM   7345  CD  LYS B 131       6.868 -41.631  73.163  1.00164.40
C
ANISOU 7345  CD  LYS B 131    24180  22127  16159  -4478  -1377
407       C
ATOM   7346  CE  LYS B 131       6.080 -40.774  74.139  1.00180.77
C
ANISOU 7346  CE  LYS B 131    26004  24509  18171  -4833  -1105
545       C
ATOM   7347  NZ  LYS B 131       5.438 -39.615  73.463  1.00181.70
N
ANISOU 7347  NZ  LYS B 131    25463  25148  18428  -4868   -851
451       N
ATOM   7348  N   THR B 132       7.725 -47.082  72.537  1.00198.18
N
```

```
ANISOU 7348  N   THR B 132    31002  24368  19928  -4470  -2558
347       N
ATOM   7349  CA  THR B 132       7.536 -48.191  71.614  1.00189.68
C
ANISOU 7349  CA  THR B 132    30292  22968  18809  -4502  -2776
253       C
ATOM   7350  C   THR B 132       6.249 -47.986  70.825  1.00158.09
C
ANISOU 7350  C   THR B 132    25924  19249  14892  -4959  -2520
228       C
ATOM   7351  O   THR B 132       5.317 -47.318  71.285  1.00154.02
O
ANISOU 7351  O   THR B 132    25091  19056  14374  -5394  -2213
321       O
ATOM   7352  CB  THR B 132       7.496 -49.544  72.341  1.00207.00
C
ANISOU 7352  CB  THR B 132    33394  24545  20711  -4735  -3062
365       C
ATOM   7353  OG1 THR B 132       7.477 -50.606  71.378  1.00197.20
O
ANISOU 7353  OG1 THR B 132    32522  22975  19432  -4672  -3317
255       O
ATOM   7354  CG2 THR B 132       6.259 -49.648  73.221  1.00207.07
C
ANISOU 7354  CG2 THR B 132    33590  24546  20541  -5487  -2840
560       C
ATOM   7355  N   LYS B 133       6.212 -48.553  69.621  1.00144.84
N
ANISOU 7355  N   LYS B 133    24279  17466  13287  -4834  -2655
85       N
ATOM   7356  CA  LYS B 133       5.115 -48.347  68.679  1.00158.80
C
ANISOU 7356  CA  LYS B 133    25660  19512  15165  -5164  -2444
16       C
ATOM   7357  C   LYS B 133       4.560 -49.706  68.275  1.00170.99
C
ANISOU 7357  C   LYS B 133    27791  20621  16558  -5480  -2645
18       C
ATOM   7358  O   LYS B 133       5.204 -50.447  67.526  1.00197.12
O
ANISOU 7358  O   LYS B 133    31387  23631  19880  -5119  -2935
-94       O
ATOM   7359  CB  LYS B 133       5.582 -47.561  67.451  1.00175.60
C
ANISOU 7359  CB  LYS B 133    27172  21993  17555  -4691  -2383
-177       C
ATOM   7360  CG  LYS B 133       6.214 -46.210  67.770  1.00177.78
C
ANISOU 7360  CG  LYS B 133    26895  22685  17966  -4362  -2205
-189       C
ATOM   7361  CD  LYS B 133       7.723 -46.319  67.945  1.00173.46
C
ANISOU 7361  CD  LYS B 133    26518  21964  17426  -3758  -2456
-249       C
```

ATOM   7362 CE LYS B 133   8.320 -45.015 68.449 1.00135.78
C
ANISOU 7362 CE LYS B 133   21258 17581 12751 -3507 -2277
-235   C
ATOM   7363 NZ LYS B 133   9.799 -45.103 68.609 1.00136.36
N
ANISOU 7363 NZ LYS B 133   21455 17530 12826 -2924 -2515
-321   N
ATOM   7364 N   VAL B 134   3.368 -50.029 68.774 1.00176.31
N
ANISOU 7364 N   VAL B 134   28643 21272 17074 -6161 -2493
134   N
ATOM   7365 CA VAL B 134   2.901 -51.406 68.891 1.00192.76
C
ANISOU 7365 CA VAL B 134   31474 22851 18915 -6568 -2702
202   C
ATOM   7366 C   VAL B 134   1.414 -51.454 68.556 1.00205.78
C
ANISOU 7366 C   VAL B 134   32918 24732 20538 -7238 -2450
198   C
ATOM   7367 O   VAL B 134   0.653 -50.545 68.905 1.00207.36
O
ANISOU 7367 O   VAL B 134   32595 25404 20790 -7545 -2113
220   O
ATOM   7368 CB VAL B 134   3.183 -51.975 70.300 1.00172.93
C
ANISOU 7368 CB VAL B 134   29645 19938 16122 -6759 -2841
387   C
ATOM   7369 CG1 VAL B 134   2.492 -51.140 71.372 1.00157.56
C
ANISOU 7369 CG1 VAL B 134   27391 18351 14124 -7199 -2496
523   C
ATOM   7370 CG2 VAL B 134   2.765 -53.439 70.390 1.00176.64
C
ANISOU 7370 CG2 VAL B 134   30971 19836 16307 -7173 -3091
455   C
ATOM   7371 N   LEU B 135   1.011 -52.516 67.860 1.00211.82
N
ANISOU 7371 N   LEU B 135   34089 25176 21216 -7447 -2626
153   N
ATOM   7372 CA LEU B 135   -0.368 -52.716 67.420 1.00209.30
C
ANISOU 7372 CA LEU B 135   33619 25042 20862 -8074 -2430
119   C
ATOM   7373 C   LEU B 135   -1.222 -53.192 68.590 1.00220.73
C
ANISOU 7373 C   LEU B 135   35494 26368 22006 -8828 -2322
288   C
ATOM   7374 O   LEU B 135   -1.032 -54.305 69.093 1.00225.94
O
ANISOU 7374 O   LEU B 135   36970 26475 22400 -9024 -2577
400   O
ATOM   7375 CB LEU B 135   -0.410 -53.718 66.270 1.00209.50
C

```
ANISOU 7375  CB  LEU B 135    33970  24736  20894  -8007  -2682
13        C
ATOM   7376  CG  LEU B 135      -1.776 -54.023  65.655  1.00210.71
C
ANISOU 7376  CG  LEU B 135    33986  25051  21022  -8614  -2519
-51        C
ATOM   7377  CD1 LEU B 135      -2.500 -52.736  65.293  1.00207.92
C
ANISOU 7377  CD1 LEU B 135    32710  25396  20893  -8676  -2137
-165        C
ATOM   7378  CD2 LEU B 135      -1.622 -54.917  64.433  1.00191.93
C
ANISOU 7378  CD2 LEU B 135    31891  22344  18688  -8412  -2794
-165        C
ATOM   7379  N   ARG B 136      -2.166 -52.357  69.028  1.00221.90
N
ANISOU 7379  N   ARG B 136    35109  27033  22169  -9252  -1952
298        N
ATOM   7380  CA  ARG B 136      -3.005 -52.653  70.185  1.00210.87
C
ANISOU 7380  CA  ARG B 136    34013  25623  20484  -9985  -1797
443        C
ATOM   7381  C   ARG B 136      -4.473 -52.601  69.784  1.00205.87
C
ANISOU 7381  C   ARG B 136    33010  25387  19823 -10634  -1523
344        C
ATOM   7382  O   ARG B 136      -4.980 -51.540  69.405  1.00208.68
O
ANISOU 7382  O   ARG B 136    32574  26335  20380 -10574  -1252
215        O
ATOM   7383  CB  ARG B 136      -2.726 -51.668  71.325  1.00196.86
C
ANISOU 7383  CB  ARG B 136    31957  24129  18711  -9898  -1605
546        C
ATOM   7384  CG  ARG B 136      -1.266 -51.601  71.784  1.00196.48
C
ANISOU 7384  CG  ARG B 136    32201  23753  18699  -9244  -1854
626        C
ATOM   7385  CD  ARG B 136      -0.833 -52.846  72.560  1.00203.06
C
ANISOU 7385  CD  ARG B 136    34034  23902  19218  -9407  -2162
779        C
ATOM   7386  NE  ARG B 136      -0.734 -54.032  71.713  1.00216.71
N
ANISOU 7386  NE  ARG B 136    36309  25158  20874  -9383  -2471
720        N
ATOM   7387  CZ  ARG B 136      -0.232 -55.196  72.105  1.00219.13
C
ANISOU 7387  CZ  ARG B 136    37531  24808  20921  -9395  -2822
812        C
ATOM   7388  NH1 ARG B 136       0.233 -55.342  73.338  1.00222.21
N
ANISOU 7388  NH1 ARG B 136    38404  24924  21102  -9426  -2910
967        N
```

```
ATOM   7389  NH2 ARG B 136      -0.192 -56.215  71.258  1.00229.80
N
ANISOU 7389  NH2 ARG B 136    39336  25764  22213  -9359  -3101
744        N
ATOM   7390  N   ASP B 137      -5.149 -53.750  69.889  1.00195.37
N
ANISOU 7390  N   ASP B 137    32275  23730  18225 -11256  -1606
395        N
ATOM   7391  CA  ASP B 137      -6.585 -53.887  69.621  1.00200.90
C
ANISOU 7391  CA  ASP B 137    32725  24771  18838 -11976  -1362
300        C
ATOM   7392  C   ASP B 137      -6.968 -53.281  68.270  1.00212.73
C
ANISOU 7392  C   ASP B 137    33487  26696  20644 -11701  -1257
68        C
ATOM   7393  O   ASP B 137      -7.960 -52.559  68.139  1.00220.02
O
ANISOU 7393  O   ASP B 137    33756  28209  21632 -11994   -950
-63        O
ATOM   7394  CB  ASP B 137      -7.407 -53.272  70.757  1.00190.48
C
ANISOU 7394  CB  ASP B 137    31114  23898  17362 -12532  -1016
355        C
ATOM   7395  CG  ASP B 137      -8.875 -53.653  70.693  1.00208.98
C
ANISOU 7395  CG  ASP B 137    33359  26526  19519 -13385   -794
267        C
ATOM   7396  OD1 ASP B 137      -9.704 -52.926  71.280  1.00203.66
O
ANISOU 7396  OD1 ASP B 137    32177  26412  18793 -13768   -465
218        O
ATOM   7397  OD2 ASP B 137      -9.198 -54.679  70.058  1.00224.24
O
ANISOU 7397  OD2 ASP B 137    35717  28136  21349 -13671   -954
234        O
ATOM   7398  N   GLY B 138      -6.163 -53.570  67.252  1.00197.77
N
ANISOU 7398  N   GLY B 138    31705  24507  18932 -11113  -1524
3        N
ATOM   7399  CA  GLY B 138      -6.393 -53.049  65.921  1.00183.38
C
ANISOU 7399  CA  GLY B 138    29261  23018  17399 -10794  -1462
-211        C
ATOM   7400  C   GLY B 138      -6.013 -51.600  65.729  1.00193.30
C
ANISOU 7400  C   GLY B 138    29734  24776  18935 -10246  -1283
-303        C
ATOM   7401  O   GLY B 138      -6.303 -51.034  64.668  1.00188.64
O
ANISOU 7401  O   GLY B 138    28586  24518  18571 -10015  -1198
-489        O
ATOM   7402  N   LYS B 139      -5.371 -50.982  66.717  1.00205.92
N
```

```
ANISOU 7402  N   LYS B 139    31300  26427  20513 -10035  -1231
-178       N
ATOM   7403  CA  LYS B 139      -4.914 -49.600  66.638  1.00203.05
C
ANISOU 7403  CA  LYS B 139    30263  26501  20386  -9515  -1080
-243       C
ATOM   7404  C   LYS B 139      -3.424 -49.586  66.938  1.00221.01
C
ANISOU 7404  C   LYS B 139    32834  28427  22713  -8896  -1311
-138       C
ATOM   7405  O   LYS B 139      -3.007 -49.979  68.032  1.00236.97
O
ANISOU 7405  O   LYS B 139    35342  30151  24544  -9008  -1397
36       O
ATOM   7406  CB  LYS B 139      -5.677 -48.711  67.623  1.00195.29
C
ANISOU 7406  CB  LYS B 139    28861  26018  19322  -9887   -756
-215       C
ATOM   7407  CG  LYS B 139      -5.207 -47.267  67.654  1.00190.29
C
ANISOU 7407  CG  LYS B 139    27591  25814  18898  -9373   -612
-265       C
ATOM   7408  CD  LYS B 139      -6.006 -46.456  68.661  1.00204.44
C
ANISOU 7408  CD  LYS B 139    29010  28086  20582  -9752   -314
-241       C
ATOM   7409  CE  LYS B 139      -5.511 -45.020  68.737  1.00207.21
C
ANISOU 7409  CE  LYS B 139    28784  28830  21116  -9238   -194
-279       C
ATOM   7410  NZ  LYS B 139      -6.277 -44.221  69.735  1.00211.83
N
ANISOU 7410  NZ  LYS B 139    29015  29885  21587  -9575     77
-262       N
ATOM   7411  N   TRP B 140      -2.626 -49.137  65.976  1.00212.95
N
ANISOU 7411  N   TRP B 140    31524  27452  21935  -8252  -1411
-254       N
ATOM   7412  CA  TRP B 140      -1.186 -49.067  66.180  1.00211.68
C
ANISOU 7412  CA  TRP B 140    31569  27028  21831  -7636  -1623
-196       C
ATOM   7413  C   TRP B 140      -0.867 -47.880  67.083  1.00214.75
C
ANISOU 7413  C   TRP B 140    31569  27764  22262  -7482  -1427
-126       C
ATOM   7414  O   TRP B 140      -1.161 -46.730  66.737  1.00213.44
O
ANISOU 7414  O   TRP B 140    30741  28097  22262  -7358  -1202
-225       O
ATOM   7415  CB  TRP B 140      -0.468 -48.969  64.837  1.00190.58
C
ANISOU 7415  CB  TRP B 140    28690  24334  19386  -7040  -1775
-362       C
```

```
ATOM   7416  CG  TRP B 140       0.833 -49.693  64.833  1.00182.77
C
ANISOU 7416  CG  TRP B 140    28221  22862  18362  -6562  -2120
-331       C
ATOM   7417  CD1 TRP B 140       1.943 -49.396  65.566  1.00204.07
C
ANISOU 7417  CD1 TRP B 140    31024  25467  21045  -6163  -2216
-260       C
ATOM   7418  CD2 TRP B 140       1.137 -50.891  64.111  1.00168.12
C
ANISOU 7418  CD2 TRP B 140    26881  20542  16455  -6439  -2434
-384       C
ATOM   7419  NE1 TRP B 140       2.935 -50.313  65.315  1.00200.31
N
ANISOU 7419  NE1 TRP B 140    31069  24523  20515  -5772  -2574
-286       N
ATOM   7420  CE2 TRP B 140       2.463 -51.244  64.427  1.00171.98
C
ANISOU 7420  CE2 TRP B 140    27752  20693  16900  -5927  -2719
-359       C
ATOM   7421  CE3 TRP B 140       0.421 -51.690  63.214  1.00167.43
C
ANISOU 7421  CE3 TRP B 140    26964  20301  16351  -6695  -2512
-461       C
ATOM   7422  CZ2 TRP B 140       3.087 -52.360  63.879  1.00169.14
C
ANISOU 7422  CZ2 TRP B 140    27939  19854  16472  -5647  -3087
-417       C
ATOM   7423  CZ3 TRP B 140       1.042 -52.796  62.672  1.00175.75
C
ANISOU 7423  CZ3 TRP B 140    28574  20860  17342  -6433  -2872
-499       C
ATOM   7424  CH2 TRP B 140       2.362 -53.122  63.006  1.00177.87
C
ANISOU 7424  CH2 TRP B 140    29220  20804  17560  -5905  -3160
-480       C
ATOM   7425  N   SER B 141      -0.269 -48.159  68.239  1.00198.37
N
ANISOU 7425  N   SER B 141    29932  25410  20027  -7482  -1527
42       N
ATOM   7426  CA  SER B 141      -0.270 -47.244  69.372  1.00178.28
C
ANISOU 7426  CA  SER B 141    27142  23151  17444  -7551  -1322
150       C
ATOM   7427  C   SER B 141       1.158 -46.940  69.798  1.00165.21
C
ANISOU 7427  C   SER B 141    25596  21330  15845  -6953  -1488
205       C
ATOM   7428  O   SER B 141       1.972 -47.856  69.955  1.00146.01
O
ANISOU 7428  O   SER B 141    23757  18405  13317  -6746  -1785
252       O
ATOM   7429  CB  SER B 141      -1.054 -47.831  70.549  1.00179.78
C
```

```
ANISOU 7429  CB  SER B 141    27755  23203  17350  -8224  -1245
311       C
ATOM   7430  OG  SER B 141      -2.415 -48.027  70.210  1.00185.15
O
ANISOU 7430  OG  SER B 141    28265  24115  17967  -8813  -1061
239       O
ATOM   7431  N   GLU B 142       1.456 -45.657  69.984  1.00169.46
N
ANISOU 7431  N   GLU B 142    25575  22288  16526  -6673  -1307
186       N
ATOM   7432  CA  GLU B 142       2.727 -45.228  70.554  1.00173.62
C
ANISOU 7432  CA  GLU B 142    26144  22733  17090  -6172  -1416
242       C
ATOM   7433  C   GLU B 142       2.614 -45.210  72.074  1.00187.60
C
ANISOU 7433  C   GLU B 142    28189  24425  18667  -6468  -1350
437       C
ATOM   7434  O   GLU B 142       1.800 -44.464  72.631  1.00196.68
O
ANISOU 7434  O   GLU B 142    28992  25950  19788  -6803  -1077
487       O
ATOM   7435  CB  GLU B 142       3.113 -43.847  70.025  1.00170.64
C
ANISOU 7435  CB  GLU B 142    25064  22833  16940  -5755  -1257
133       C
ATOM   7436  CG  GLU B 142       4.369 -43.262  70.649  1.00189.77
C
ANISOU 7436  CG  GLU B 142    27455  25250  19401  -5278  -1331
183       C
ATOM   7437  CD  GLU B 142       4.662 -41.857  70.157  1.00211.68
C
ANISOU 7437  CD  GLU B 142    29557  28506  22366  -4943  -1156
83       C
ATOM   7438  OE1 GLU B 142       4.958 -41.694  68.954  1.00203.00
O
ANISOU 7438  OE1 GLU B 142    28212  27502  21417  -4646  -1198
-79       O
ATOM   7439  OE2 GLU B 142       4.587 -40.913  70.971  1.00221.76
O
ANISOU 7439  OE2 GLU B 142    30572  30059  23629  -4987   -981
168       O
ATOM   7440  N   GLN B 143       3.429 -46.026  72.742  1.00197.81
N
ANISOU 7440  N   GLN B 143    30110  25234  19816  -6328  -1611
535       N
ATOM   7441  CA  GLN B 143       3.336 -46.203  74.184  1.00186.83
C
ANISOU 7441  CA  GLN B 143    29105  23674  18207  -6630  -1589
726       C
ATOM   7442  C   GLN B 143       4.737 -46.314  74.767  1.00171.31
C
ANISOU 7442  C   GLN B 143    27467  21402  16222  -6101  -1841
768       C
```

```
ATOM   7443  O   GLN B 143       5.718 -46.513  74.047  1.00188.12
O
ANISOU 7443  O   GLN B 143    29627  23387  18464  -5561  -2068
642       O
ATOM   7444  CB  GLN B 143       2.511 -47.446  74.559  1.00176.73
C
ANISOU 7444  CB  GLN B 143    28476  22014  16660  -7249  -1661
821       C
ATOM   7445  CG  GLN B 143       1.029 -47.372  74.207  1.00174.22
C
ANISOU 7445  CG  GLN B 143    27857  22025  16315  -7871  -1390
783       C
ATOM   7446  CD  GLN B 143       0.314 -46.186  74.837  1.00172.91
C
ANISOU 7446  CD  GLN B 143    27098  22422  16176  -8116  -1034
816       C
ATOM   7447  OE1 GLN B 143       0.790 -45.592  75.807  1.00160.73
O
ANISOU 7447  OE1 GLN B 143    25516  20945  14606  -7971   -984
924       O
ATOM   7448  NE2 GLN B 143      -0.843 -45.841  74.287  1.00175.03
N
ANISOU 7448  NE2 GLN B 143    26906  23106  16491  -8479   -797
710       N
ATOM   7449  N   GLU B 144       4.819 -46.167  76.089  1.00144.96
N
ANISOU 7449  N   GLU B 144    24355  17989  12732  -6254  -1798
931       N
ATOM   7450  CA  GLU B 144       6.096 -46.296  76.778  1.00161.73
C
ANISOU 7450  CA  GLU B 144    26825  19815  14812  -5781  -2042
971       C
ATOM   7451  C   GLU B 144       6.685 -47.691  76.580  1.00181.82
C
ANISOU 7451  C   GLU B 144    30136  21742  17206  -5618  -2440
936       C
ATOM   7452  O   GLU B 144       5.988 -48.700  76.714  1.00174.42
O
ANISOU 7452  O   GLU B 144    29747  20464  16058  -6089  -2519
1007       O
ATOM   7453  CB  GLU B 144       5.911 -45.988  78.263  1.00150.95
C
ANISOU 7453  CB  GLU B 144    25627  18447  13281  -6058  -1920
1165       C
ATOM   7454  CG  GLU B 144       5.538 -44.534  78.529  1.00141.93
C
ANISOU 7454  CG  GLU B 144    23736  17906  12283  -6096  -1572
1188       C
ATOM   7455  CD  GLU B 144       5.132 -44.279  79.967  1.00172.26
C
ANISOU 7455  CD  GLU B 144    27736  21773  15944  -6464  -1423
1381       C
ATOM   7456  OE1 GLU B 144       5.984 -44.437  80.866  1.00184.38
O
```

```
ANISOU 7456  OE1 GLU B 144    29651  23019  17386  -6223  -1592
1470       O
ATOM   7457  OE2 GLU B 144       3.959 -43.916  80.197  1.00189.21
O
ANISOU 7457  OE2 GLU B 144    29616  24241  18033  -6985  -1139
1429       O
ATOM   7458  N   ALA B 145       7.980 -47.735  76.256  1.00197.45
N
ANISOU 7458  N   ALA B 145    32154  23590  19280  -4948  -2700
812        N
ATOM   7459  CA  ALA B 145       8.656 -48.997  75.959  1.00206.99
C
ANISOU 7459  CA  ALA B 145    34044  24245  20357  -4674  -3116
733        C
ATOM   7460  C   ALA B 145       8.739 -49.922  77.171  1.00222.58
C
ANISOU 7460  C   ALA B 145    36883  25671  22015  -4886  -3337
887        C
ATOM   7461  O   ALA B 145       8.827 -51.144  77.012  1.00219.72
O
ANISOU 7461  O   ALA B 145    37229  24792  21464  -4911  -3653
866        O
ATOM   7462  CB  ALA B 145      10.056 -48.716  75.416  1.00214.78
C
ANISOU 7462  CB  ALA B 145    34800  25297  21508  -3886  -3324
538        C
ATOM   7463  N   ALA B 146       8.715 -49.358  78.381  1.00223.48
N
ANISOU 7463  N   ALA B 146    36982  25874  22055  -5034  -3189
1040       N
ATOM   7464  CA  ALA B 146       8.639 -50.157  79.603  1.00211.47
C
ANISOU 7464  CA  ALA B 146    36278  23856  20214  -5328  -3351
1208       C
ATOM   7465  C   ALA B 146       7.377 -51.012  79.673  1.00216.23
C
ANISOU 7465  C   ALA B 146    37346  24226  20586  -6097  -3283
1327       C
ATOM   7466  O   ALA B 146       7.428 -52.168  80.109  1.00230.62
O
ANISOU 7466  O   ALA B 146    40045  25464  22118  -6262  -3569
1391       O
ATOM   7467  CB  ALA B 146       8.728 -49.241  80.823  1.00211.41
C
ANISOU 7467  CB  ALA B 146    36051  24076  20199  -5382  -3142
1351       C
ATOM   7468  N   ILE B 147       6.236 -50.460  79.258  1.00209.12
N
ANISOU 7468  N   ILE B 147    35887  23779  19790  -6578  -2915
1346       N
ATOM   7469  CA  ILE B 147       4.954 -51.152  79.402  1.00225.37
C
ANISOU 7469  CA  ILE B 147    38296  25711  21623  -7376  -2794
1450       C
```

```
ATOM   7470  C   ILE B 147       4.923 -52.457  78.610  1.00212.20
C
ANISOU 7470  C   ILE B 147    37245  23557  19826  -7425  -3107
1377       C
ATOM   7471  O   ILE B 147       4.427 -53.481  79.098  1.00235.90
O
ANISOU 7471  O   ILE B 147    41010  26112  22511  -7930  -3230
1488       O
ATOM   7472  CB  ILE B 147       3.806 -50.214  78.986  1.00218.59
C
ANISOU 7472  CB  ILE B 147    36613  25509  20933  -7784  -2351
1427       C
ATOM   7473  CG1 ILE B 147       3.712 -49.033  79.953  1.00200.29
C
ANISOU 7473  CG1 ILE B 147    33817  23616  18669  -7829  -2058
1526       C
ATOM   7474  CG2 ILE B 147       2.487 -50.968  78.931  1.00230.84
C
ANISOU 7474  CG2 ILE B 147    38460  26981  22267  -8591  -2234
1482       C
ATOM   7475  CD1 ILE B 147       3.364 -49.434  81.373  1.00201.61
C
ANISOU 7475  CD1 ILE B 147    34564  23523  18517  -8323  -2030
1728       C
ATOM   7476  N   LEU B 148       5.450 -52.449  77.391  1.00192.58
N
ANISOU 7476  N   LEU B 148    34465  21142  17566  -6916  -3246
1190       N
ATOM   7477  CA  LEU B 148       5.228 -53.550  76.460  1.00186.08
C
ANISOU 7477  CA  LEU B 148    34075  19965  16661  -7003  -3484
1106       C
ATOM   7478  C   LEU B 148       5.798 -54.879  76.953  1.00174.87
C
ANISOU 7478  C   LEU B 148    33734  17790  14917  -6927  -3937
1153       C
ATOM   7479  O   LEU B 148       6.868 -54.936  77.564  1.00175.16
O
ANISOU 7479  O   LEU B 148    34080  17575  14896  -6429  -4187
1146       O
ATOM   7480  CB  LEU B 148       5.829 -53.211  75.102  1.00194.46
C
ANISOU 7480  CB  LEU B 148    34595  21260  18031  -6390  -3557
887       C
ATOM   7481  CG  LEU B 148       5.443 -54.215  74.022  1.00194.42
C
ANISOU 7481  CG  LEU B 148    34909  20982  17980  -6513  -3745
793       C
ATOM   7482  CD1 LEU B 148       3.936 -54.219  73.814  1.00188.72
C
ANISOU 7482  CD1 LEU B 148    33994  20494  17217  -7303  -3427
858       C
ATOM   7483  CD2 LEU B 148       6.166 -53.882  72.757  1.00195.57
C
```

```
ANISOU 7483  CD2 LEU B 148    34557  21339  18414  -5857  -3837
574       C
ATOM   7484  N   VAL B 149       5.058 -55.951  76.674  1.00179.02
N
ANISOU 7484  N   VAL B 149    34846  17954  15220  -7430  -4051
1191       N
ATOM   7485  CA  VAL B 149       5.384 -57.324  77.057  1.00184.51
C
ANISOU 7485  CA  VAL B 149    36659  17890  15556  -7476  -4490
1242       C
ATOM   7486  C   VAL B 149       5.858 -58.105  75.833  1.00188.05
C
ANISOU 7486  C   VAL B 149    37336  18059  16057  -7039  -4844
1060       C
ATOM   7487  O   VAL B 149       5.376 -57.861  74.717  1.00183.41
O
ANISOU 7487  O   VAL B 149    36190  17805  15693  -7083  -4682
955       O
ATOM   7488  CB  VAL B 149       4.179 -58.012  77.715  1.00189.04
C
ANISOU 7488  CB  VAL B 149    37818  18226  15783  -8429  -4370
1429       C
ATOM   7489  CG1 VAL B 149       3.948 -57.453  79.110  1.00189.04
C
ANISOU 7489  CG1 VAL B 149    37819  18356  15652  -8777  -4128
1608       C
ATOM   7490  CG2 VAL B 149       2.938 -57.822  76.851  1.00188.17
C
ANISOU 7490  CG2 VAL B 149    37162  18536  15799  -8983  -4038
1392       C
ATOM   7491  N   PRO B 150       6.823 -59.034  75.995  1.00197.72
N
ANISOU 7491  N   PRO B 150    39369  18680  17078  -6576  -5344
1005       N
ATOM   7492  CA  PRO B 150       7.117 -60.036  74.955  1.00199.65
C
ANISOU 7492  CA  PRO B 150    40047  18542  17269  -6285  -5730
855       C
ATOM   7493  C   PRO B 150       5.921 -60.623  74.214  1.00203.99
C
ANISOU 7493  C   PRO B 150    40710  19046  17750  -6943  -5623
891       C
ATOM   7494  O   PRO B 150       4.878 -60.911  74.809  1.00210.89
O
ANISOU 7494  O   PRO B 150    41903  19830  18395  -7758  -5437
1068       O
ATOM   7495  CB  PRO B 150       7.848 -61.128  75.746  1.00196.19
C
ANISOU 7495  CB  PRO B 150    40748  17350  16446  -6073  -6241
886       C
ATOM   7496  CG  PRO B 150       8.589 -60.362  76.780  1.00194.17
C
ANISOU 7496  CG  PRO B 150    40294  17245  16236  -5735  -6180
922       C
```

```
ATOM   7497  CD  PRO B 150       7.620 -59.274  77.210  1.00190.90
C
ANISOU 7497  CD  PRO B 150    39135  17419  15980  -6336  -5597
1079       C
ATOM   7498  N   GLY B 151       6.082 -60.808  72.900  1.00191.94
N
ANISOU 7498  N   GLY B 151    38918  17595  16415  -6593  -5741
712        N
ATOM   7499  CA  GLY B 151       5.111 -61.475  72.056  1.00194.03
C
ANISOU 7499  CA  GLY B 151    39340  17759  16622  -7093  -5721
709        C
ATOM   7500  C   GLY B 151       4.425 -60.561  71.064  1.00201.25
C
ANISOU 7500  C   GLY B 151    39213  19349  17904  -7210  -5305
623        C
ATOM   7501  O   GLY B 151       3.843 -61.049  70.085  1.00190.50
O
ANISOU 7501  O   GLY B 151    37862  17948  16571  -7412  -5333
556        O
ATOM   7502  N   ASP B 152       4.485 -59.255  71.291  1.00226.89
N
ANISOU 7502  N   ASP B 152    41590  23196  21420  -7079  -4938
618        N
ATOM   7503  CA  ASP B 152       3.742 -58.266  70.527  1.00233.03
C
ANISOU 7503  CA  ASP B 152    41387  24638  22514  -7248  -4512
551        C
ATOM   7504  C   ASP B 152       4.510 -57.814  69.289  1.00192.97
C
ANISOU 7504  C   ASP B 152    35728  19820  17771  -6504  -4588
330        C
ATOM   7505  O   ASP B 152       5.744 -57.809  69.263  1.00175.96
O
ANISOU 7505  O   ASP B 152    33659  17533  15665  -5796  -4856
229        O
ATOM   7506  CB  ASP B 152       3.415 -57.069  71.418  1.00215.88
C
ANISOU 7506  CB  ASP B 152    38614  22973  20438  -7475  -4097
654        C
ATOM   7507  CG  ASP B 152       2.329 -57.383  72.425  1.00223.65
C
ANISOU 7507  CG  ASP B 152    39978  23867  21132  -8351  -3908
848        C
ATOM   7508  OD1 ASP B 152       1.291 -57.951  72.023  1.00234.79
O
ANISOU 7508  OD1 ASP B 152    41548  25232  22430  -8953  -3833
861        O
ATOM   7509  OD2 ASP B 152       2.513 -57.073  73.620  1.00226.68
O
ANISOU 7509  OD2 ASP B 152    40507  24233  21389  -8451  -3834
982        O
ATOM   7510  N   ILE B 153       3.762 -57.433  68.257  1.00174.58
N
```

```
ANISOU 7510  N   ILE B 153    32802  17875  15657  -6678  -4349 
240       N
ATOM   7511  CA  ILE B 153       4.340 -56.953  67.006  1.00180.68 
C
ANISOU 7511  CA  ILE B 153    32981  18930  16740  -6058  -4373 
32       C
ATOM   7512  C   ILE B 153       4.591 -55.456  67.138  1.00178.15 
C
ANISOU 7512  C   ILE B 153    31765  19232  16690  -5807  -4030 
-2       C
ATOM   7513  O   ILE B 153       3.663 -54.680  67.391  1.00163.93 
O
ANISOU 7513  O   ILE B 153    29472  17852  14961  -6267  -3646 
66       O
ATOM   7514  CB  ILE B 153       3.426 -57.255  65.809  1.00187.76 
C
ANISOU 7514  CB  ILE B 153    33691  19921  17730  -6347  -4295 
-55       C
ATOM   7515  CG1 ILE B 153       3.211 -58.762  65.665  1.00190.79 
C
ANISOU 7515  CG1 ILE B 153    35004  19660  17827  -6592  -4659 
-20       C
ATOM   7516  CG2 ILE B 153       4.006 -56.663  64.529  1.00167.21 
C
ANISOU 7516  CG2 ILE B 153    30441  17642  15448  -5719  -4288 
-268       C
ATOM   7517  CD1 ILE B 153       2.124 -59.128  64.680  1.00185.48 
C
ANISOU 7517  CD1 ILE B 153    34219  19057  17199  -7032  -4559 
-71       C
ATOM   7518  N   VAL B 154       5.848 -55.053  66.966  1.00193.84 
N
ANISOU 7518  N   VAL B 154    33550  21289  18814  -5078  -4177 
-120       N
ATOM   7519  CA  VAL B 154       6.261 -53.659  67.075  1.00182.39 
C
ANISOU 7519  CA  VAL B 154    31311  20388  17602  -4780  -3898 
-161       C
ATOM   7520  C   VAL B 154       6.708 -53.125  65.722  1.00177.86 
C
ANISOU 7520  C   VAL B 154    30130  20143  17307  -4284  -3869 
-376       C
ATOM   7521  O   VAL B 154       7.270 -53.848  64.893  1.00180.27 
O
ANISOU 7521  O   VAL B 154    30686  20195  17615  -3903  -4167 
-518       O
ATOM   7522  CB  VAL B 154       7.392 -53.490  68.108  1.00158.66 
C
ANISOU 7522  CB  VAL B 154    28519  17252  14512  -4371  -4054 
-122       C
ATOM   7523  CG1 VAL B 154       6.893 -53.854  69.468  1.00161.60 
C
ANISOU 7523  CG1 VAL B 154    29425  17355  14619  -4889  -4031 
98       C
```

```
ATOM   7524  CG2 VAL B 154       8.594 -54.349  67.734  1.00160.75
C
ANISOU 7524  CG2 VAL B 154    29260  17113  14704  -3736  -4516
-274       C
ATOM   7525  N   SER B 155       6.439 -51.841  65.503  1.00174.77
N
ANISOU 7525  N   SER B 155    28950  20317  17135  -4295  -3510
-403       N
ATOM   7526  CA  SER B 155       6.934 -51.124  64.337  1.00166.68
C
ANISOU 7526  CA  SER B 155    27308  19654  16367  -3826  -3442
-598       C
ATOM   7527  C   SER B 155       8.221 -50.417  64.740  1.00166.69
C
ANISOU 7527  C   SER B 155    27074  19825  16436  -3265  -3483
-655       C
ATOM   7528  O   SER B 155       8.257 -49.716  65.757  1.00145.96
O
ANISOU 7528  O   SER B 155    24301  17370  13786  -3374  -3313
-534       O
ATOM   7529  CB  SER B 155       5.904 -50.116  63.824  1.00166.02
C
ANISOU 7529  CB  SER B 155    26539  20080  16462  -4150  -3050
-612       C
ATOM   7530  OG  SER B 155       6.368 -49.462  62.655  1.00192.65
O
ANISOU 7530  OG  SER B 155    29376  23764  20058  -3716  -2998
-800       O
ATOM   7531  N   ILE B 156       9.270 -50.603  63.947  1.00182.89
N
ANISOU 7531  N   ILE B 156    29083  21843  18564  -2672  -3707
-849       N
ATOM   7532  CA  ILE B 156      10.596 -50.084  64.259  1.00176.34
C
ANISOU 7532  CA  ILE B 156    28071  21156  17775  -2108  -3793
-944       C
ATOM   7533  C   ILE B 156      11.020 -49.134  63.145  1.00179.43
C
ANISOU 7533  C   ILE B 156    27753  22023  18401  -1762  -3629
-1132       C
ATOM   7534  O   ILE B 156      11.220 -49.557  61.998  1.00188.55
O
ANISOU 7534  O   ILE B 156    28883  23139  19620  -1518  -3764
-1301       O
ATOM   7535  CB  ILE B 156      11.614 -51.213  64.449  1.00172.83
C
ANISOU 7535  CB  ILE B 156    28264  20244  17158  -1673  -4247
-1037       C
ATOM   7536  CG1 ILE B 156      11.225 -52.076  65.650  1.00171.62
C
ANISOU 7536  CG1 ILE B 156    28859  19605  16744  -2031  -4407
-838       C
ATOM   7537  CG2 ILE B 156      12.966 -50.639  64.693  1.00166.14
C
```

```
ANISOU 7537  CG2 ILE B 156    27157  19610  16361  -1087  -4322
-1173       C
ATOM   7538  CD1 ILE B 156      12.063 -53.326  65.796  1.00164.91
C
ANISOU 7538  CD1 ILE B 156    28754  18220  15684  -1646  -4898
-930       C
ATOM   7539  N   LYS B 157      11.162 -47.854  63.483  1.00177.88
N
ANISOU 7539  N   LYS B 157    27007  22265  18315  -1745  -3344
-1102       N
ATOM   7540  CA  LYS B 157      11.467 -46.796  62.533  1.00171.10
C
ANISOU 7540  CA  LYS B 157    25476  21881  17653  -1503  -3144
-1253       C
ATOM   7541  C   LYS B 157      12.977 -46.603  62.367  1.00143.81
C
ANISOU 7541  C   LYS B 157    21884  18539  14218   -882  -3307
-1441       C
ATOM   7542  O   LYS B 157      13.797 -47.254  63.020  1.00138.90
O
ANISOU 7542  O   LYS B 157    21664  17647  13466   -607  -3582
-1464       O
ATOM   7543  CB  LYS B 157      10.791 -45.498  62.969  1.00186.26
C
ANISOU 7543  CB  LYS B 157    26904  24210  19655  -1826  -2763
-1130       C
ATOM   7544  CG  LYS B 157       9.295 -45.647  63.199  1.00194.08
C
ANISOU 7544  CG  LYS B 157    27982  25149  20608  -2440  -2592
-973       C
ATOM   7545  CD  LYS B 157       8.604 -44.301  63.321  1.00189.13
C
ANISOU 7545  CD  LYS B 157    26788  24992  20080  -2685  -2228
-918       C
ATOM   7546  CE  LYS B 157       9.146 -43.506  64.496  1.00180.67
C
ANISOU 7546  CE  LYS B 157    25600  24079  18969  -2610  -2133
-808       C
ATOM   7547  NZ  LYS B 157       8.445 -42.202  64.647  1.00183.01
N
ANISOU 7547  NZ  LYS B 157    25380  24818  19338  -2841  -1801
-755       N
ATOM   7548  N   LEU B 158      13.339 -45.694  61.454  1.00134.53
N
ANISOU 7548  N   LEU B 158    20137  17784  13195   -664  -3140
-1593       N
ATOM   7549  CA  LEU B 158      14.742 -45.367  61.204  1.00133.97
C
ANISOU 7549  CA  LEU B 158    19835  17920  13146   -118  -3242
-1797       C
ATOM   7550  C   LEU B 158      15.443 -44.877  62.467  1.00141.54
C
ANISOU 7550  C   LEU B 158    20788  18955  14035     -6  -3232
-1721       C
```

```
ATOM   7551  O   LEU B 158      16.580 -45.275  62.747  1.00147.04
O
ANISOU 7551  O   LEU B 158    21646  19567  14654    425  -3479
-1856       O
ATOM   7552  CB  LEU B 158      14.823 -44.316  60.090  1.00145.55
C
ANISOU 7552  CB  LEU B 158    20681  19853  14770    -33  -2997
-1936       C
ATOM   7553  CG  LEU B 158      16.145 -43.721  59.586  1.00147.07
C
ANISOU 7553  CG  LEU B 158    20491  20391  14999    444  -3008
-2168       C
ATOM   7554  CD1 LEU B 158      16.662 -42.589  60.471  1.00128.23
C
ANISOU 7554  CD1 LEU B 158    17793  18319  12608    460  -2823
-2104       C
ATOM   7555  CD2 LEU B 158      17.194 -44.806  59.444  1.00152.00
C
ANISOU 7555  CD2 LEU B 158    21443  20782  15528    917  -3384
-2364       C
ATOM   7556  N   GLY B 159      14.788 -44.011  63.237  1.00150.01
N
ANISOU 7556  N   GLY B 159    21668  20197  15129   -370  -2958
-1522       N
ATOM   7557  CA  GLY B 159      15.410 -43.367  64.377  1.00146.06
C
ANISOU 7557  CA  GLY B 159    21088  19825  14584   -278  -2906
-1446       C
ATOM   7558  C   GLY B 159      15.224 -44.084  65.692  1.00145.41
C
ANISOU 7558  C   GLY B 159    21556  19346  14346   -440  -3060
-1264       C
ATOM   7559  O   GLY B 159      15.833 -43.700  66.695  1.00133.19
O
ANISOU 7559  O   GLY B 159    20015  17845  12744   -316  -3068
-1210       O
ATOM   7560  N   ASP B 160      14.388 -45.121  65.699  1.00159.32
N
ANISOU 7560  N   ASP B 160    23797  20714  16022   -730  -3182
-1170       N
ATOM   7561  CA  ASP B 160      13.975 -45.782  66.930  1.00158.96
C
ANISOU 7561  CA  ASP B 160    24309  20283  15807  -1011  -3287
-968       C
ATOM   7562  C   ASP B 160      15.154 -46.373  67.695  1.00164.24
C
ANISOU 7562  C   ASP B 160    25388  20680  16337   -585  -3614
-1034       C
ATOM   7563  O   ASP B 160      16.124 -46.863  67.110  1.00145.37
O
ANISOU 7563  O   ASP B 160    23071  18227  13937    -93  -3877
-1259       O
ATOM   7564  CB  ASP B 160      12.965 -46.884  66.611  1.00153.77
C
```

ANISOU 7564 CB ASP B 160   24119 19243 15062 -1379 -3392
-897   C
ATOM   7565 CG ASP B 160   11.597 -46.335 66.266 1.00175.87
C
ANISOU 7565 CG ASP B 160   26593 22278 17953 -1912 -3058
-786   C
ATOM   7566 OD1 ASP B 160   10.706 -47.136 65.914 1.00176.96
O
ANISOU 7566 OD1 ASP B 160   27032 22170 18034 -2249 -3105
-744   O
ATOM   7567 OD2 ASP B 160   11.415 -45.101 66.343 1.00181.97
O
ANISOU 7567 OD2 ASP B 160   26814 23484 18841 -1988 -2759
-755   O
ATOM   7568 N   ILE B 161   15.051 -46.320 69.019 1.00169.46
N
ANISOU 7568 N   ILE B 161   26315 21191 16880   -769 -3601
-849   N
ATOM   7569 CA ILE B 161   15.818 -47.177 69.915 1.00166.35
C
ANISOU 7569 CA ILE B 161   26528 20382 16297   -503 -3949
-858   C
ATOM   7570 C   ILE B 161   15.009 -48.446 70.146 1.00166.22
C
ANISOU 7570 C   ILE B 161   27245 19820 16092   -859 -4140
-732   C
ATOM   7571 O   ILE B 161   13.821 -48.381 70.484 1.00160.48
O
ANISOU 7571 O   ILE B 161   26584 19058 15335 -1447 -3922
-522   O
ATOM   7572 CB ILE B 161   16.123 -46.460 71.240 1.00173.11
C
ANISOU 7572 CB ILE B 161   27322 21340 17111   -527 -3839
-719   C
ATOM   7573 CG1 ILE B 161   16.972 -45.212 70.987 1.00179.42
C
ANISOU 7573 CG1 ILE B 161   27418 22675 18078   -182 -3664
-854   C
ATOM   7574 CG2 ILE B 161   16.824 -47.402 72.208 1.00191.28
C
ANISOU 7574 CG2 ILE B 161   30317 23166 19194   -277 -4214
-723   C
ATOM   7575 CD1 ILE B 161   16.993 -44.245 72.151 1.00201.43
C
ANISOU 7575 CD1 ILE B 161   30007 25661 20868   -318 -3454
-685   C
ATOM   7576 N   ILE B 162   15.643 -49.598 69.953 1.00169.32
N
ANISOU 7576 N   ILE B 162   28192 19799 16343   -510 -4554
-871   N
ATOM   7577 CA ILE B 162   14.967 -50.885 70.104 1.00167.36
C
ANISOU 7577 CA ILE B 162   28715 18988 15885   -820 -4784
-769   C

```
ATOM   7578  C   ILE B 162      14.483 -51.078  71.538  1.00179.86
C
ANISOU 7578  C   ILE B 162    30792  20276  17272  -1234  -4762
-515       C
ATOM   7579  O   ILE B 162      15.290 -51.070  72.479  1.00189.07
O
ANISOU 7579  O   ILE B 162    32190  21315  18333   -946  -4926
-518       O
ATOM   7580  CB  ILE B 162      15.889 -52.042  69.688  1.00181.74
C
ANISOU 7580  CB  ILE B 162    31068  20416  17568   -279  -5278
-990       C
ATOM   7581  CG1 ILE B 162      16.366 -51.861  68.246  1.00174.69
C
ANISOU 7581  CG1 ILE B 162    29676  19837  16862    124  -5293
-1252       C
ATOM   7582  CG2 ILE B 162      15.173 -53.375  69.846  1.00205.08
C
ANISOU 7582  CG2 ILE B 162    34881  22759  20281   -624  -5532
-875       C
ATOM   7583  CD1 ILE B 162      15.253 -51.869  67.229  1.00174.67
C
ANISOU 7583  CD1 ILE B 162    29460  19930  16978   -297  -5078
-1198       C
ATOM   7584  N   PRO B 163      13.174 -51.252  71.743  1.00171.93
N
ANISOU 7584  N   PRO B 163    29952  19172  16203  -1916  -4561
-304       N
ATOM   7585  CA  PRO B 163      12.634 -51.201  73.114  1.00179.54
C
ANISOU 7585  CA  PRO B 163    31245  19974  16997  -2373  -4449
-56       C
ATOM   7586  C   PRO B 163      12.962 -52.439  73.930  1.00182.63
C
ANISOU 7586  C   PRO B 163    32612  19705  17073  -2336  -4855
-5       C
ATOM   7587  O   PRO B 163      13.353 -52.329  75.098  1.00172.94
O
ANISOU 7587  O   PRO B 163    31650  18342  15720  -2287  -4912
90       O
ATOM   7588  CB  PRO B 163      11.123 -51.050  72.889  1.00167.21
C
ANISOU 7588  CB  PRO B 163    29510  18562  15461  -3104  -4113
102       C
ATOM   7589  CG  PRO B 163      10.877 -51.607  71.527  1.00161.97
C
ANISOU 7589  CG  PRO B 163    28816  17853  14875  -3051  -4212
-45       C
ATOM   7590  CD  PRO B 163      12.117 -51.353  70.722  1.00158.52
C
ANISOU 7590  CD  PRO B 163    28043  17589  14600  -2307  -4382
-296       C
ATOM   7591  N   ALA B 164      12.810 -53.619  73.334  1.00196.45
N
```

```
ANISOU 7591  N   ALA B 164    34932  21028  18680  -2351  -5152
-70       N
ATOM   7592  CA  ALA B 164      12.974 -54.902  74.000  1.00208.34
C
ANISOU 7592  CA  ALA B 164    37462  21849  19847  -2379  -5563
-20       C
ATOM   7593  C   ALA B 164      13.651 -55.844  73.019  1.00206.72
C
ANISOU 7593  C   ALA B 164    37592  21355  19598  -1864  -5988
-258       C
ATOM   7594  O   ALA B 164      13.693 -55.579  71.815  1.00213.82
O
ANISOU 7594  O   ALA B 164    37958  22568  20716  -1663  -5907
-415       O
ATOM   7595  CB  ALA B 164      11.633 -55.479  74.474  1.00219.93
C
ANISOU 7595  CB  ALA B 164    39436  23026  21102  -3221  -5439
227       C
ATOM   7596  N   ASP B 165      14.202 -56.944  73.534  1.00204.23
N
ANISOU 7596  N   ASP B 165    38178  20437  18985  -1627  -6457
-295       N
ATOM   7597  CA  ASP B 165      14.697 -57.963  72.620  1.00197.03
C
ANISOU 7597  CA  ASP B 165    37683  19197  17984  -1196  -6888
-511       C
ATOM   7598  C   ASP B 165      13.556 -58.473  71.756  1.00192.54
C
ANISOU 7598  C   ASP B 165    37210  18532  17416  -1734  -6785
-433       C
ATOM   7599  O   ASP B 165      12.418 -58.618  72.211  1.00180.46
O
ANISOU 7599  O   ASP B 165    35922  16875  15771  -2482  -6572
-192       O
ATOM   7600  CB  ASP B 165      15.321 -59.129  73.388  1.00206.20
C
ANISOU 7600  CB  ASP B 165    39911  19664  18774   -925  -7430
-546       C
ATOM   7601  CG  ASP B 165      16.611 -58.752  74.073  1.00225.07
C
ANISOU 7601  CG  ASP B 165    42223  22134  21161   -252  -7620
-699       C
ATOM   7602  OD1 ASP B 165      17.047 -57.592  73.922  1.00232.11
O
ANISOU 7602  OD1 ASP B 165    42224  23627  22340     -6  -7326
-779       O
ATOM   7603  OD2 ASP B 165      17.191 -59.616  74.764  1.00233.54
O
ANISOU 7603  OD2 ASP B 165    44142  22664  21929     30  -8072
-749       O
ATOM   7604  N   ALA B 166      13.875 -58.749  70.497  1.00195.11
N
ANISOU 7604  N   ALA B 166    37334  18934  17863  -1347  -6937
-650       N
```

```
ATOM   7605  CA  ALA B 166      12.846 -59.032  69.514  1.00186.91
C
ANISOU 7605  CA  ALA B 166    36199  17923  16897  -1793  -6791
-607       C
ATOM   7606  C   ALA B 166      13.433 -59.879  68.400  1.00193.39
C
ANISOU 7606  C   ALA B 166    37267  18521  17693  -1263  -7195
-854       C
ATOM   7607  O   ALA B 166      14.652 -59.984  68.245  1.00204.93
O
ANISOU 7607  O   ALA B 166    38732  19978  19153   -517  -7503
-1091       O
ATOM   7608  CB  ALA B 166      12.245 -57.742  68.946  1.00173.47
C
ANISOU 7608  CB  ALA B 166    33445  16919  15547  -2042  -6246
-577       C
ATOM   7609  N   ARG B 167      12.540 -60.483  67.631  1.00182.20
N
ANISOU 7609  N   ARG B 167    36046  16934  16248  -1661  -7191
-808       N
ATOM   7610  CA  ARG B 167      12.886 -61.150  66.388  1.00183.33
C
ANISOU 7610  CA  ARG B 167    36278  16957  16420  -1240  -7487
-1029       C
ATOM   7611  C   ARG B 167      12.219 -60.392  65.246  1.00207.86
C
ANISOU 7611  C   ARG B 167    38520  20597  19859  -1444  -7081
-1059       C
ATOM   7612  O   ARG B 167      11.031 -60.059  65.324  1.00236.43
O
ANISOU 7612  O   ARG B 167    41933  24364  23536  -2145  -6716
-865       O
ATOM   7613  CB  ARG B 167      12.468 -62.622  66.444  1.00189.04
C
ANISOU 7613  CB  ARG B 167    38085  16949  16791  -1491  -7899
-961       C
ATOM   7614  CG  ARG B 167      10.971 -62.846  66.557  1.00190.07
C
ANISOU 7614  CG  ARG B 167    38413  16962  16844  -2434  -7627
-702       C
ATOM   7615  CD  ARG B 167      10.631 -64.317  66.718  1.00229.70
C
ANISOU 7615  CD  ARG B 167    44582  21223  21469  -2701  -8056
-629       C
ATOM   7616  NE  ARG B 167       9.204 -64.554  66.528  1.00265.43
N
ANISOU 7616  NE  ARG B 167    49197  25705  25948  -3578  -7793
-436       N
ATOM   7617  CZ  ARG B 167       8.608 -64.665  65.346  1.00257.70
C
ANISOU 7617  CZ  ARG B 167    47911  24875  25128  -3721  -7697
-501       C
ATOM   7618  NH1 ARG B 167       9.317 -64.602  64.227  1.00242.68
N
```

ANISOU 7618 NH1 ARG B 167   45630 23145 23431 -3046 -7852
-744   N
ATOM   7619 NH2 ARG B 167   7.301 -64.868 65.286 1.00248.48
N
ANISOU 7619 NH2 ARG B 167   46830 23683 23898 -4546 -7454
-333   N
ATOM   7620 N   LEU B 168   12.994 -60.094 64.205 1.00211.57
N
ANISOU 7620 N   LEU B 168   38475 21377 20535   -827 -7144
-1316   N
ATOM   7621 CA LEU B 168   12.460 -59.378 63.053 1.00185.52
C
ANISOU 7621 CA LEU B 168   34381 18568 17541   -951 -6793
-1371   C
ATOM   7622 C   LEU B 168   11.380 -60.183 62.344 1.00185.10
C
ANISOU 7622 C   LEU B 168   34673 18231 17425 -1426 -6830
-1297   C
ATOM   7623 O   LEU B 168   11.477 -61.405 62.202 1.00199.13
O
ANISOU 7623 O   LEU B 168   37234 19461 18964 -1336 -7253
-1333   O
ATOM   7624 CB LEU B 168   13.580 -59.015 62.079 1.00170.97
C
ANISOU 7624 CB LEU B 168   32010 17065 15885   -184 -6895
-1676   C
ATOM   7625 CG LEU B 168   14.673 -58.129 62.675 1.00186.03
C
ANISOU 7625 CG LEU B 168   33477 19332 17874   283 -6828
-1779   C
ATOM   7626 CD1 LEU B 168   15.690 -57.759 61.615 1.00174.10
C
ANISOU 7626 CD1 LEU B 168   31403 18208 16539   962 -6891
-2093   C
ATOM   7627 CD2 LEU B 168   14.067 -56.880 63.301 1.00194.45
C
ANISOU 7627 CD2 LEU B 168   33950 20829 19105   -184 -6315
-1578   C
ATOM   7628 N   LEU B 169   10.342 -59.480 61.898 1.00183.08
N
ANISOU 7628 N   LEU B 169   33833 18354 17375 -1927 -6395
-1203   N
ATOM   7629 CA LEU B 169   9.123 -60.120 61.428 1.00204.11
C
ANISOU 7629 CA LEU B 169   36777 20801 19974 -2526 -6351
-1099   C
ATOM   7630 C   LEU B 169   9.152 -60.208 59.907 1.00218.58
C
ANISOU 7630 C   LEU B 169   38267 22784 21999 -2230 -6396
-1295   C
ATOM   7631 O   LEU B 169   9.386 -61.285 59.348 1.00211.99
O
ANISOU 7631 O   LEU B 169   37995 21528 21025 -2005 -6789
-1391   O

```
ATOM   7632  CB  LEU B 169       7.891 -59.353  61.915  1.00199.67
C
ANISOU 7632  CB  LEU B 169    35812  20561  19491  -3280  -5862
-895       C
ATOM   7633  CG  LEU B 169       6.526 -59.946  61.568  1.00209.57
C
ANISOU 7633  CG  LEU B 169    37314  21651  20663  -3993  -5770
-786       C
ATOM   7634  CD1 LEU B 169       6.400 -61.358  62.122  1.00190.27
C
ANISOU 7634  CD1 LEU B 169    35957  18497  17841  -4248  -6162
-680       C
ATOM   7635  CD2 LEU B 169       5.409 -59.058  62.095  1.00198.96
C
ANISOU 7635  CD2 LEU B 169    35471  20723  19403  -4664  -5276
-630       C
ATOM   7636  N   GLU B 170       8.921 -59.085  59.231  1.00225.73
N
ANISOU 7636  N   GLU B 170    38279  24275  23211  -2218  -6009
-1360       N
ATOM   7637  CA  GLU B 170       8.937 -59.056  57.774  1.00228.07
C
ANISOU 7637  CA  GLU B 170    38200  24754  23703  -1944  -6015
-1547       C
ATOM   7638  C   GLU B 170       9.179 -57.628  57.302  1.00220.18
C
ANISOU 7638  C   GLU B 170    36229  24419  23009  -1729  -5635
-1646       C
ATOM   7639  O   GLU B 170       9.030 -56.667  58.062  1.00230.77
O
ANISOU 7639  O   GLU B 170    37182  26082  24418  -1932  -5323
-1538       O
ATOM   7640  CB  GLU B 170       7.643 -59.627  57.184  1.00232.53
C
ANISOU 7640  CB  GLU B 170    38956  25149  24246  -2525  -5953
-1466       C
ATOM   7641  CG  GLU B 170       6.393 -58.825  57.481  1.00237.34
C
ANISOU 7641  CG  GLU B 170    39106  26112  24962  -3217  -5480
-1310       C
ATOM   7642  CD  GLU B 170       5.136 -59.532  57.012  1.00248.58
C
ANISOU 7642  CD  GLU B 170    40803  27330  26315  -3814  -5461
-1243       C
ATOM   7643  OE1 GLU B 170       4.822 -60.611  57.559  1.00256.78
O
ANISOU 7643  OE1 GLU B 170    42636  27857  27073  -4152  -5704
-1123       O
ATOM   7644  OE2 GLU B 170       4.468 -59.015  56.093  1.00252.14
O
ANISOU 7644  OE2 GLU B 170    40695  28127  26981  -3950  -5212
-1317       O
ATOM   7645  N   GLY B 171       9.554 -57.509  56.035  1.00201.65
N
```

```
ANISOU 7645  N   GLY B 171    33524  22264  20831  -1321  -5673
-1854       N
ATOM   7646  CA  GLY B 171      10.020 -56.269  55.448  1.00199.24
C
ANISOU 7646  CA  GLY B 171    32383  22539  20780  -1007  -5392
-1991       C
ATOM   7647  C   GLY B 171      11.467 -56.369  55.004  1.00200.40
C
ANISOU 7647  C   GLY B 171    32467  22743  20932   -238  -5658
-2237       C
ATOM   7648  O   GLY B 171      12.141 -57.379  55.201  1.00190.52
O
ANISOU 7648  O   GLY B 171    31811  21090  19489     96  -6076
-2312       O
ATOM   7649  N   ASP B 172      11.934 -55.291  54.379  1.00212.62
N
ANISOU 7649  N   ASP B 172    33283  24813  22689     38  -5414
-2379       N
ATOM   7650  CA  ASP B 172      13.328 -55.236  53.961  1.00214.35
C
ANISOU 7650  CA  ASP B 172    33334  25192  22917    741  -5611
-2636       C
ATOM   7651  C   ASP B 172      14.262 -55.288  55.169  1.00232.89
C
ANISOU 7651  C   ASP B 172    35923  27452  25110   1005  -5782
-2630       C
ATOM   7652  O   ASP B 172      13.964 -54.697  56.215  1.00241.64
O
ANISOU 7652  O   ASP B 172    36958  28645  26209    681  -5567
-2442       O
ATOM   7653  CB  ASP B 172      13.595 -53.969  53.144  1.00199.27
C
ANISOU 7653  CB  ASP B 172    30591  23883  21239    896  -5273
-2770       C
ATOM   7654  CG  ASP B 172      12.923 -54.000  51.783  1.00164.75
C
ANISOU 7654  CG  ASP B 172    26002  19588  17009    796  -5178
-2845       C
ATOM   7655  OD1 ASP B 172      12.813 -52.931  51.147  1.00144.49
O
ANISOU 7655  OD1 ASP B 172    22806  17471  14623    770  -4857
-2903       O
ATOM   7656  OD2 ASP B 172      12.510 -55.096  51.348  1.00162.20
O
ANISOU 7656  OD2 ASP B 172    26161  18861  16605    745  -5435
-2847       O
ATOM   7657  N   PRO B 173      15.389 -56.001  55.062  1.00243.08
N
ANISOU 7657  N   PRO B 173    37514  28575  26270   1604  -6181
-2846       N
ATOM   7658  CA  PRO B 173      16.516 -55.820  55.992  1.00240.81
C
ANISOU 7658  CA  PRO B 173    37262  28357  25878   2000  -6320
-2929       C
```

```
ATOM   7659  C   PRO B 173      16.844 -54.385  56.383  1.00226.96
C
ANISOU 7659  C   PRO B 173    34795  27159  24279   1970  -5928
-2911       C
ATOM   7660  O   PRO B 173      16.689 -53.454  55.585  1.00238.56
O
ANISOU 7660  O   PRO B 173    35618  29077  25949   1910  -5605
-2965       O
ATOM   7661  CB  PRO B 173      17.686 -56.457  55.230  1.00249.75
C
ANISOU 7661  CB  PRO B 173    38477  29479  26938   2724  -6704
-3269       C
ATOM   7662  CG  PRO B 173      17.039 -57.509  54.400  1.00255.49
C
ANISOU 7662  CG  PRO B 173    39654  29810  27611   2630  -6930
-3270       C
ATOM   7663  CD  PRO B 173      15.737 -56.902  53.951  1.00250.31
C
ANISOU 7663  CD  PRO B 173    38651  29308  27146   2001  -6509
-3066       C
ATOM   7664  N   LEU B 174      17.304 -54.207  57.621  1.00209.85
N
ANISOU 7664  N   LEU B 174    32780  24946  22007   2015  -5969
-2835       N
ATOM   7665  CA  LEU B 174      17.703 -52.910  58.148  1.00177.65
C
ANISOU 7665  CA  LEU B 174    28105  21351  18043   2011  -5644
-2812       C
ATOM   7666  C   LEU B 174      18.968 -53.118  58.971  1.00169.83
C
ANISOU 7666  C   LEU B 174    27303  20320  16903   2518  -5926
-2960       C
ATOM   7667  O   LEU B 174      19.372 -54.252  59.244  1.00177.73
O
ANISOU 7667  O   LEU B 174    28942  20885  17704   2805  -6358
-3043       O
ATOM   7668  CB  LEU B 174      16.609 -52.250  59.001  1.00169.74
C
ANISOU 7668  CB  LEU B 174    27031  20371  17089   1358  -5293
-2488       C
ATOM   7669  CG  LEU B 174      16.374 -52.725  60.440  1.00181.10
C
ANISOU 7669  CG  LEU B 174    29057  21412  18339   1115  -5420
-2272       C
ATOM   7670  CD1 LEU B 174      15.287 -51.880  61.087  1.00186.37
C
ANISOU 7670  CD1 LEU B 174    29484  22240  19086    481  -5009
-1991       C
ATOM   7671  CD2 LEU B 174      16.011 -54.204  60.516  1.00167.16
C
ANISOU 7671  CD2 LEU B 174    28136  19014  16364   1041  -5805
-2227       C
ATOM   7672  N   LYS B 175      19.600 -52.019  59.369  1.00172.97
N
```

```
ANISOU 7672  N   LYS B 175    27162  21172  17386   2638  -5695
-3004       N
ATOM   7673  CA  LYS B 175      20.847 -52.082  60.119  1.00181.76
C
ANISOU 7673  CA  LYS B 175    28355  22330  18376   3134  -5932
-3174       C
ATOM   7674  C   LYS B 175      20.671 -51.465  61.500  1.00170.53
C
ANISOU 7674  C   LYS B 175    26956  20909  16929   2845  -5754
-2937       C
ATOM   7675  O   LYS B 175      19.987 -50.449  61.658  1.00153.02
O
ANISOU 7675  O   LYS B 175    24323  18962  14854   2404  -5343
-2739       O
ATOM   7676  CB  LYS B 175      21.980 -51.376  59.365  1.00179.38
C
ANISOU 7676  CB  LYS B 175    27388  22599  18168   3612  -5860
-3497       C
ATOM   7677  CG  LYS B 175      22.176 -51.879  57.943  1.00177.29
C
ANISOU 7677  CG  LYS B 175    27028  22398  17936   3899  -5999
-3745       C
ATOM   7678  CD  LYS B 175      23.278 -51.120  57.220  1.00180.12
C
ANISOU 7678  CD  LYS B 175    26704  23363  18370   4314  -5891
-4064       C
ATOM   7679  CE  LYS B 175      23.383 -51.561  55.767  1.00162.05
C
ANISOU 7679  CE  LYS B 175    24295  21158  16119   4552  -5990
-4297       C
ATOM   7680  NZ  LYS B 175      24.330 -50.717  54.986  1.00147.09
N
ANISOU 7680  NZ  LYS B 175    21693  19896  14298   4853  -5816
-4590       N
ATOM   7681  N   VAL B 176      21.286 -52.094  62.498  1.00179.29
N
ANISOU 7681  N   VAL B 176    28574  21704  17845   3111  -6082
-2966       N
ATOM   7682  CA  VAL B 176      21.229 -51.651  63.887  1.00193.22
C
ANISOU 7682  CA  VAL B 176    30450  23410  19554   2900  -5978
-2759       C
ATOM   7683  C   VAL B 176      22.655 -51.419  64.373  1.00205.44
C
ANISOU 7683  C   VAL B 176    31840  25180  21037   3493  -6162
-3011       C
ATOM   7684  O   VAL B 176      23.549 -52.231  64.109  1.00238.24
O
ANISOU 7684  O   VAL B 176    36260  29202  25059   4052  -6567
-3296       O
ATOM   7685  CB  VAL B 176      20.474 -52.651  64.785  1.00201.45
C
ANISOU 7685  CB  VAL B 176    32342  23803  20395   2569  -6191
-2515       C
```

```
ATOM   7686  CG1 VAL B 176      19.050 -52.848  64.277  1.00185.42
C
ANISOU 7686  CG1 VAL B 176    30414  21610  18427   1953  -5989
-2288       C
ATOM   7687  CG2 VAL B 176      21.197 -53.988  64.862  1.00212.82
C
ANISOU 7687  CG2 VAL B 176    34498  24771  21594   3067  -6756
-2715       C
ATOM   7688  N   ASP B 177      22.875 -50.292  65.042  1.00183.75
N
ANISOU 7688  N   ASP B 177    28639  22798  18380   3387  -5869
-2927       N
ATOM   7689  CA  ASP B 177      24.204 -49.915  65.507  1.00182.18
C
ANISOU 7689  CA  ASP B 177    28197  22885  18137   3903  -5988
-3166       C
ATOM   7690  C   ASP B 177      24.423 -50.492  66.902  1.00203.38
C
ANISOU 7690  C   ASP B 177    31519  25131  20623   3990  -6268
-3069       C
ATOM   7691  O   ASP B 177      23.735 -50.109  67.855  1.00205.22
O
ANISOU 7691  O   ASP B 177    31881  25235  20859   3543  -6067
-2760       O
ATOM   7692  CB  ASP B 177      24.362 -48.394  65.506  1.00175.40
C
ANISOU 7692  CB  ASP B 177    26545  22636  17462   3743  -5541
-3130       C
ATOM   7693  CG  ASP B 177      25.799 -47.950  65.724  1.00178.93
C
ANISOU 7693  CG  ASP B 177    26630  23477  17878   4281  -5638
-3434       C
ATOM   7694  OD1 ASP B 177      26.632 -48.771  66.163  1.00188.79
O
ANISOU 7694  OD1 ASP B 177    28273  24503  18957   4764  -6050
-3639       O
ATOM   7695  OD2 ASP B 177      26.095 -46.765  65.461  1.00167.58
O
ANISOU 7695  OD2 ASP B 177    24518  22581  16574   4218  -5308
-3477       O
ATOM   7696  N   GLN B 178      25.379 -51.413  67.018  1.00209.91
N
ANISOU 7696  N   GLN B 178    32755  25735  21267   4577  -6741
-3346       N
ATOM   7697  CA  GLN B 178      25.678 -52.085  68.275  1.00219.08
C
ANISOU 7697  CA  GLN B 178    34603  26433  22205   4740  -7080
-3299       C
ATOM   7698  C   GLN B 178      26.808 -51.415  69.050  1.00210.19
C
ANISOU 7698  C   GLN B 178    33153  25641  21068   5134  -7098
-3469       C
ATOM   7699  O   GLN B 178      27.303 -51.993  70.023  1.00196.34
O
```

```
ANISOU 7699  O   GLN B 178    31940  23542  19117   5416  -7440
-3516       O
ATOM   7700  CB  GLN B 178      26.041 -53.550  68.012  1.00226.99
C
ANISOU 7700  CB  GLN B 178    36336  26936  22974   5178  -7642
-3512       C
ATOM   7701  CG  GLN B 178      24.906 -54.408  67.467  1.00225.37
C
ANISOU 7701  CG  GLN B 178    36641  26267  22720   4773  -7699
-3320       C
ATOM   7702  CD  GLN B 178      23.703 -54.454  68.387  1.00228.23
C
ANISOU 7702  CD  GLN B 178    37451  26234  23031   4071  -7519
-2889       C
ATOM   7703  OE1 GLN B 178      22.597 -54.081  67.998  1.00216.58
O
ANISOU 7703  OE1 GLN B 178    35759  24834  21698   3483  -7160
-2646       O
ATOM   7704  NE2 GLN B 178      23.912 -54.917  69.615  1.00230.23
N
ANISOU 7704  NE2 GLN B 178    38336  26073  23068   4132  -7773
-2808       N
ATOM   7705  N   SER B 179      27.226 -50.218  68.640  1.00212.08
N
ANISOU 7705  N   SER B 179    32548  26531  21500   5154  -6750
-3566       N
ATOM   7706  CA  SER B 179      28.083 -49.383  69.473  1.00200.10
C
ANISOU 7706  CA  SER B 179    30675  25357  19997   5363  -6667
-3646       C
ATOM   7707  C   SER B 179      27.408 -49.055  70.800  1.00193.10
C
ANISOU 7707  C   SER B 179    30081  24200  19087   4919  -6511
-3267       C
ATOM   7708  O   SER B 179      26.192 -48.850  70.859  1.00189.02
O
ANISOU 7708  O   SER B 179    29643  23530  18644   4311  -6232
-2923       O
ATOM   7709  CB  SER B 179      28.448 -48.093  68.739  1.00190.95
C
ANISOU 7709  CB  SER B 179    28581  24927  19045   5323  -6265
-3761       C
ATOM   7710  OG  SER B 179      27.287 -47.397  68.322  1.00183.82
O
ANISOU 7710  OG  SER B 179    27402  24126  18315   4699  -5831
-3454       O
ATOM   7711  N   ALA B 180      28.214 -49.055  71.869  1.00211.86
N
ANISOU 7711  N   ALA B 180    32636  26517  21345   5247  -6714
-3353       N
ATOM   7712  CA  ALA B 180      27.802 -48.912  73.266  1.00243.29
C
ANISOU 7712  CA  ALA B 180    37000  30188  25252   4956  -6668
-3049       C
```

```
ATOM   7713  C   ALA B 180      27.227 -50.215  73.807  1.00257.37
C
ANISOU 7713  C   ALA B 180    39766  31217  26808   4855  -7018
-2900       C
ATOM   7714  O   ALA B 180      27.609 -50.659  74.894  1.00272.74
O
ANISOU 7714  O   ALA B 180    42249  32810  28569   5056  -7300
-2894       O
ATOM   7715  CB  ALA B 180      26.804 -47.763  73.457  1.00237.78
C
ANISOU 7715  CB  ALA B 180    35867  29736  24741   4289  -6127
-2685       C
ATOM   7716  N   LEU B 181      26.303 -50.828  73.063  1.00243.40
N
ANISOU 7716  N   LEU B 181    38259  29186  25037   4531  -7006
-2781       N
ATOM   7717  CA  LEU B 181      25.780 -52.132  73.463  1.00237.44
C
ANISOU 7717  CA  LEU B 181    38471  27707  24039   4424  -7361
-2662       C
ATOM   7718  C   LEU B 181      26.891 -53.172  73.520  1.00247.91
C
ANISOU 7718  C   LEU B 181    40312  28747  25134   5161  -7952
-3020       C
ATOM   7719  O   LEU B 181      26.963 -53.965  74.466  1.00252.76
O
ANISOU 7719  O   LEU B 181    41727  28810  25500   5253  -8299
-2966       O
ATOM   7720  CB  LEU B 181      24.668 -52.582  72.511  1.00223.61
C
ANISOU 7720  CB  LEU B 181    36839  25788  22336   3977  -7245
-2515       C
ATOM   7721  CG  LEU B 181      23.308 -51.868  72.540  1.00205.57
C
ANISOU 7721  CG  LEU B 181    34280  23620  20208   3182  -6738
-2137       C
ATOM   7722  CD1 LEU B 181      22.701 -51.921  73.939  1.00196.66
C
ANISOU 7722  CD1 LEU B 181    33659  22126  18939   2754  -6687
-1814       C
ATOM   7723  CD2 LEU B 181      23.366 -50.433  72.022  1.00206.04
C
ANISOU 7723  CD2 LEU B 181    33343  24392  20554   3087  -6269
-2151       C
ATOM   7724  N   THR B 182      27.771 -53.181  72.518  1.00247.19
N
ANISOU 7724  N   THR B 182    39785  29030  25107   5699  -8084
-3403       N
ATOM   7725  CA  THR B 182      28.963 -54.024  72.549  1.00245.52
C
ANISOU 7725  CA  THR B 182    39929  28673  24685   6483  -8639
-3811       C
ATOM   7726  C   THR B 182      30.199 -53.393  71.922  1.00238.84
C
```

```
ANISOU 7726  C   THR B 182    38278  28516  23955   7059  -8622
-4234       C
ATOM   7727  O   THR B 182      31.311 -53.805  72.268  1.00240.07
O
ANISOU 7727  O   THR B 182    38602  28666  23946   7719  -9028
-4580       O
ATOM   7728  CB  THR B 182      28.687 -55.373  71.865  1.00233.77
C
ANISOU 7728  CB  THR B 182    39129  26677  23015   6623  -9047
-3903       C
ATOM   7729  OG1 THR B 182      29.659 -56.336  72.290  1.00245.29
O
ANISOU 7729  OG1 THR B 182    41208  27799  24192   7319  -9653
-4220       O
ATOM   7730  CG2 THR B 182      28.732 -55.240  70.349  1.00218.03
C
ANISOU 7730  CG2 THR B 182    36550  25098  21192   6727  -8919
-4103       C
ATOM   7731  N   GLY B 183      30.066 -52.423  71.027  1.00223.37
N
ANISOU 7731  N   GLY B 183    35471  27150  22249   6842  -8183
-4239       N
ATOM   7732  CA  GLY B 183      31.185 -51.656  70.531  1.00207.24
C
ANISOU 7732  CA  GLY B 183    32620  25810  20313   7272  -8088
-4602       C
ATOM   7733  C   GLY B 183      31.546 -51.881  69.071  1.00203.32
C
ANISOU 7733  C   GLY B 183    31745  25640  19869   7564  -8142
-4924       C
ATOM   7734  O   GLY B 183      32.701 -51.637  68.697  1.00192.85
O
ANISOU 7734  O   GLY B 183    29934  24809  18532   8092  -8241
-5336       O
ATOM   7735  N   GLU B 184      30.607 -52.339  68.248  1.00212.69
N
ANISOU 7735  N   GLU B 184    33131  26581  21102   7236  -8082
-4762       N
ATOM   7736  CA  GLU B 184      30.837 -52.462  66.815  1.00213.47
C
ANISOU 7736  CA  GLU B 184    32835  27001  21272   7445  -8078
-5029       C
ATOM   7737  C   GLU B 184      31.128 -51.106  66.179  1.00203.51
C
ANISOU 7737  C   GLU B 184    30579  26506  20238   7303  -7595
-5104       C
ATOM   7738  O   GLU B 184      30.603 -50.070  66.594  1.00195.23
O
ANISOU 7738  O   GLU B 184    29192  25644  19342   6804  -7164
-4809       O
ATOM   7739  CB  GLU B 184      29.627 -53.106  66.136  1.00211.25
C
ANISOU 7739  CB  GLU B 184    32954  26294  21018   7024  -8052
-4779       C
```

```
ATOM   7740  CG  GLU B 184      29.398 -54.554  66.531  1.00210.34
C
ANISOU 7740  CG  GLU B 184    33849  25425  20645   7192  -8570
-4755       C
ATOM   7741  CD  GLU B 184      30.601 -55.429  66.242  1.00210.33
C
ANISOU 7741  CD  GLU B 184    34068  25409  20438   8027  -9119
-5241       C
ATOM   7742  OE1 GLU B 184      31.131 -56.041  67.192  1.00205.87
O
ANISOU 7742  OE1 GLU B 184    34079  24494  19647   8392  -9526
-5340       O
ATOM   7743  OE2 GLU B 184      31.015 -55.505  65.065  1.00208.96
O
ANISOU 7743  OE2 GLU B 184    33500  25578  20317   8328  -9153
-5535       O
ATOM   7744  N   SER B 185      31.980 -51.125  65.154  1.00198.84
N
ANISOU 7744  N   SER B 185    29539  26362  19650   7747  -7680
-5513       N
ATOM   7745  CA  SER B 185      32.418 -49.919  64.465  1.00188.84
C
ANISOU 7745  CA  SER B 185    27358  25840  18551   7668  -7269
-5652       C
ATOM   7746  C   SER B 185      31.635 -49.676  63.183  1.00175.42
C
ANISOU 7746  C   SER B 185    25365  24274  17012   7299  -6972
-5545       C
ATOM   7747  O   SER B 185      31.908 -48.701  62.475  1.00155.48
O
ANISOU 7747  O   SER B 185    22124  22336  14613   7192  -6626
-5650       O
ATOM   7748  CB  SER B 185      33.916 -50.000  64.152  1.00202.90
C
ANISOU 7748  CB  SER B 185    28761  28114  20219   8370  -7517
-6202       C
ATOM   7749  OG  SER B 185      34.680 -50.106  65.340  1.00216.25
O
ANISOU 7749  OG  SER B 185    30654  29741  21771   8721  -7771
-6324       O
ATOM   7750  N   LEU B 186      30.668 -50.542  62.874  1.00186.72
N
ANISOU 7750  N   LEU B 186    27355  25165  18426   7092  -7105
-5344       N
ATOM   7751  CA  LEU B 186      29.808 -50.465  61.710  1.00178.32
C
ANISOU 7751  CA  LEU B 186    26123  24126  17502   6740  -6870
-5223       C
ATOM   7752  C   LEU B 186      28.467 -51.025  62.161  1.00187.69
C
ANISOU 7752  C   LEU B 186    27962  24664  18687   6245  -6881
-4803       C
ATOM   7753  O   LEU B 186      28.444 -52.050  62.858  1.00205.59
O
```

```
ANISOU 7753  O   LEU B 186    30955  26389  20773   6414  -7274 
-4772       O
ATOM   7754  CB  LEU B 186      30.358 -51.267  60.525  1.00162.51 
C
ANISOU 7754  CB  LEU B 186    24117  22203  15426   7228  -7165 
-5602       C
ATOM   7755  CG  LEU B 186      29.655 -51.151  59.169  1.00176.57 
C
ANISOU 7755  CG  LEU B 186    25643  24093  17351   6952  -6934 
-5551       C
ATOM   7756  CD1 LEU B 186      29.971 -49.815  58.509  1.00162.60 
C
ANISOU 7756  CD1 LEU B 186    23005  23033  15741   6792  -6475 
-5641       C
ATOM   7757  CD2 LEU B 186      30.042 -52.309  58.259  1.00192.04 
C
ANISOU 7757  CD2 LEU B 186    27881  25897  19187   7450  -7351 
-5868       C
ATOM   7758  N   PRO B 187      27.350 -50.391  61.803  1.00176.15 
N
ANISOU 7758  N   PRO B 187    26284  23243  17402   5634  -6470 
-4491       N
ATOM   7759  CA  PRO B 187      26.047 -51.020  62.047  1.00186.98 
C
ANISOU 7759  CA  PRO B 187    28248  24036  18759   5160  -6488 
-4141       C
ATOM   7760  C   PRO B 187      25.929 -52.366  61.346  1.00184.26 
C
ANISOU 7760  C   PRO B 187    28450  23268  18292   5405  -6890 
-4273       C
ATOM   7761  O   PRO B 187      26.416 -52.553  60.228  1.00177.08 
O
ANISOU 7761  O   PRO B 187    27279  22597  17407   5744  -6978 
-4561       O
ATOM   7762  CB  PRO B 187      25.054 -49.993  61.493  1.00181.13 
C
ANISOU 7762  CB  PRO B 187    27006  23575  18241   4572  -5972 
-3899       C
ATOM   7763  CG  PRO B 187      25.808 -49.321  60.397  1.00176.03 
C
ANISOU 7763  CG  PRO B 187    25663  23523  17695   4842  -5818 
-4197       C
ATOM   7764  CD  PRO B 187      27.232 -49.222  60.913  1.00159.73 
C
ANISOU 7764  CD  PRO B 187    23438  21732  15519   5394  -6019 
-4504       C
ATOM   7765  N   VAL B 188      25.271 -53.307  62.019  1.00198.51 
N
ANISOU 7765  N   VAL B 188    31039  24434  19951   5216  -7135 
-4060       N
ATOM   7766  CA  VAL B 188      25.162 -54.686  61.553  1.00197.10 
C
ANISOU 7766  CA  VAL B 188    31527  23756  19607   5444  -7578 
-4162       C
```

```
ATOM   7767  C   VAL B 188      23.827 -54.878  60.845  1.00189.11
C
ANISOU 7767  C   VAL B 188    30624  22530  18697   4882  -7381
-3909       C
ATOM   7768  O   VAL B 188      22.773 -54.477  61.357  1.00187.73
O
ANISOU 7768  O   VAL B 188    30494  22239  18595   4256  -7079
-3559       O
ATOM   7769  CB  VAL B 188      25.338 -55.685  62.712  1.00199.87
C
ANISOU 7769  CB  VAL B 188    32753  23504  19686   5609  -8022
-4114       C
ATOM   7770  CG1 VAL B 188      26.767 -55.635  63.233  1.00196.63
C
ANISOU 7770  CG1 VAL B 188    32240  23312  19157   6288  -8294
-4451       C
ATOM   7771  CG2 VAL B 188      24.364 -55.388  63.841  1.00200.32
C
ANISOU 7771  CG2 VAL B 188    33088  23282  19742   4962  -7784
-3690       C
ATOM   7772  N   THR B 189      23.879 -55.463  59.652  1.00185.30
N
ANISOU 7772  N   THR B 189    30154  22030  18222   5114  -7547
-4103       N
ATOM   7773  CA  THR B 189      22.679 -55.767  58.883  1.00188.45
C
ANISOU 7773  CA  THR B 189    30687  22210  18705   4647  -7416
-3908       C
ATOM   7774  C   THR B 189      21.845 -56.859  59.551  1.00201.23
C
ANISOU 7774  C   THR B 189    33220  23109  20130   4325  -7678
-3661       C
ATOM   7775  O   THR B 189      22.365 -57.909  59.944  1.00204.46
O
ANISOU 7775  O   THR B 189    34306  23087  20294   4704  -8164
-3784       O
ATOM   7776  CB  THR B 189      23.050 -56.164  57.451  1.00190.64
C
ANISOU 7776  CB  THR B 189    30773  22640  19022   5036  -7565
-4203       C
ATOM   7777  OG1 THR B 189      21.858 -56.417  56.695  1.00185.50
O
ANISOU 7777  OG1 THR B 189    30230  21788  18465   4571  -7425
-4012       O
ATOM   7778  CG2 THR B 189      23.958 -57.388  57.421  1.00195.76
C
ANISOU 7778  CG2 THR B 189    31992  22968  19421   5699  -8164
-4501       C
ATOM   7779  N   LYS B 190      20.550 -56.591  59.711  1.00210.47
N
ANISOU 7779  N   LYS B 190    34419  24160  21391   3613  -7356
-3321       N
ATOM   7780  CA  LYS B 190      19.623 -57.487  60.393  1.00213.53
C
```

```
ANISOU 7780  CA  LYS B 190    35617  23919  21595   3165  -7516
-3052       C
ATOM   7781  C   LYS B 190      18.440 -57.764  59.477  1.00222.74
C
ANISOU 7781  C   LYS B 190    36812  24965  22853   2695  -7369
-2917       C
ATOM   7782  O   LYS B 190      17.859 -56.833  58.908  1.00230.61
O
ANISOU 7782  O   LYS B 190    37153  26378  24088   2378  -6936
-2843       O
ATOM   7783  CB  LYS B 190      19.144 -56.896  61.725  1.00191.25
C
ANISOU 7783  CB  LYS B 190    32838  21071  18756   2690  -7259
-2757       C
ATOM   7784  CG  LYS B 190      20.232 -56.729  62.770  1.00166.83
C
ANISOU 7784  CG  LYS B 190    29832  18010  15546   3115  -7436
-2861       C
ATOM   7785  CD  LYS B 190      20.575 -58.075  63.393  1.00172.30
C
ANISOU 7785  CD  LYS B 190    31510  18044  15911   3377  -7994
-2908       C
ATOM   7786  CE  LYS B 190      21.516 -57.929  64.576  1.00173.54
C
ANISOU 7786  CE  LYS B 190    31825  18178  15934   3735  -8169
-2979       C
ATOM   7787  NZ  LYS B 190      21.687 -59.219  65.301  1.00178.97
N
ANISOU 7787  NZ  LYS B 190    33561  18163  16276   3902  -8700
-2980       N
ATOM   7788  N   HIS B 191      18.087 -59.038  59.342  1.00226.53
N
ANISOU 7788  N   HIS B 191    38072  24870  23131   2656  -7742
-2893       N
ATOM   7789  CA  HIS B 191      17.165 -59.523  58.328  1.00233.74
C
ANISOU 7789  CA  HIS B 191    39086  25626  24098   2350  -7715
-2839       C
ATOM   7790  C   HIS B 191      15.940 -60.177  58.959  1.00220.91
C
ANISOU 7790  C   HIS B 191    38129  23490  22316   1650  -7718
-2524       C
ATOM   7791  O   HIS B 191      15.977 -60.591  60.123  1.00211.82
O
ANISOU 7791  O   HIS B 191    37567  21973  20943   1525  -7888
-2390       O
ATOM   7792  CB  HIS B 191      17.875 -60.533  57.413  1.00245.76
C
ANISOU 7792  CB  HIS B 191    40945  26929  25506   2960  -8190
-3132       C
ATOM   7793  CG  HIS B 191      19.080 -59.978  56.718  1.00257.90
C
ANISOU 7793  CG  HIS B 191    41835  28986  27170   3640  -8201
-3475       C
```

```
ATOM   7794  ND1 HIS B 191      19.151 -59.832  55.350  1.00255.34
N
ANISOU 7794  ND1 HIS B 191    41050  28957  27009   3841  -8126
-3661       N
ATOM   7795  CD2 HIS B 191      20.263 -59.534  57.205  1.00258.20
C
ANISOU 7795  CD2 HIS B 191    41606  29316  27183   4148  -8274
-3676       C
ATOM   7796  CE1 HIS B 191      20.325 -59.321  55.024  1.00258.20
C
ANISOU 7796  CE1 HIS B 191    40891  29782  27433   4424  -8143
-3962       C
ATOM   7797  NE2 HIS B 191      21.018 -59.130  56.131  1.00261.04
N
ANISOU 7797  NE2 HIS B 191    41345  30157  27682   4619  -8232
-3982       N
ATOM   7798  N   PRO B 192      14.810 -60.244  58.221  1.00219.22
N
ANISOU 7798  N   PRO B 192    37821  23265  22209   1153  -7512
-2402       N
ATOM   7799  CA  PRO B 192      13.650 -61.034  58.670  1.00216.65
C
ANISOU 7799  CA  PRO B 192    38177  22440  21701    488  -7559
-2143       C
ATOM   7800  C   PRO B 192      13.987 -62.360  59.337  1.00218.72
C
ANISOU 7800  C   PRO B 192    39497  22009  21596    631  -8095
-2133       C
ATOM   7801  O   PRO B 192      14.762 -63.157  58.800  1.00223.52
O
ANISOU 7801  O   PRO B 192    40464  22379  22086   1212  -8537
-2360       O
ATOM   7802  CB  PRO B 192      12.863 -61.250  57.370  1.00203.55
C
ANISOU 7802  CB  PRO B 192    36336  20824  20179    280  -7470
-2173       C
ATOM   7803  CG  PRO B 192      13.157 -60.027  56.584  1.00200.63
C
ANISOU 7803  CG  PRO B 192    34974  21124  20132    514  -7110
-2314       C
ATOM   7804  CD  PRO B 192      14.605 -59.713  56.861  1.00209.25
C
ANISOU 7804  CD  PRO B 192    35878  22418  21210   1220  -7277
-2530       C
ATOM   7805  N   GLY B 193      13.408 -62.602  60.514  1.00220.60
N
ANISOU 7805  N   GLY B 193    40259  21922  21636    107  -8068
-1880       N
ATOM   7806  CA  GLY B 193      13.675 -63.801  61.279  1.00229.38
C
ANISOU 7806  CA  GLY B 193    42435  22351  22369    177  -8561
-1844       C
ATOM   7807  C   GLY B 193      14.886 -63.739  62.182  1.00231.40
C
```

```
ANISOU 7807  C   GLY B 193    42878  22551  22493    750  -8817
-1963       C
ATOM   7808  O   GLY B 193      15.120 -64.687  62.943  1.00240.08
O
ANISOU 7808  O   GLY B 193    44902  23065  23254    811  -9233
-1928       O
ATOM   7809  N   GLN B 194      15.660 -62.661  62.129  1.00219.45
N
ANISOU 7809  N   GLN B 194    40545  21618  21220   1163  -8592
-2108       N
ATOM   7810  CA  GLN B 194      16.866 -62.529  62.930  1.00209.46
C
ANISOU 7810  CA  GLN B 194    39365  20368  19851   1744  -8822
-2256       C
ATOM   7811  C   GLN B 194      16.545 -61.824  64.242  1.00201.51
C
ANISOU 7811  C   GLN B 194    38284  19446  18837   1305  -8516
-2006       C
ATOM   7812  O   GLN B 194      15.631 -60.998  64.320  1.00196.12
O
ANISOU 7812  O   GLN B 194    37091  19083  18341    709  -8021
-1794       O
ATOM   7813  CB  GLN B 194      17.947 -61.760  62.169  1.00208.48
C
ANISOU 7813  CB  GLN B 194    38399  20844  19968   2433  -8757
-2572       C
ATOM   7814  CG  GLN B 194      19.359 -61.989  62.679  1.00218.79
C
ANISOU 7814  CG  GLN B 194    39909  22105  21117   3207  -9159
-2841       C
ATOM   7815  CD  GLN B 194      20.412 -61.445  61.733  1.00226.82
C
ANISOU 7815  CD  GLN B 194    40163  23690  22329   3887  -9151
-3199       C
ATOM   7816  OE1 GLN B 194      20.090 -60.833  60.713  1.00220.53
O
ANISOU 7816  OE1 GLN B 194    38680  23321  21791   3766  -8823
-3230       O
ATOM   7817  NE2 GLN B 194      21.678 -61.667  62.065  1.00236.51
N
ANISOU 7817  NE2 GLN B 194    41511  24934  23418   4602  -9515
-3487       N
ATOM   7818  N   GLU B 195      17.309 -62.157  65.277  1.00196.73
N
ANISOU 7818  N   GLU B 195    38197  18551  18002   1625  -8828
-2045       N
ATOM   7819  CA  GLU B 195      17.066 -61.628  66.610  1.00186.44
C
ANISOU 7819  CA  GLU B 195    36951  17244  16644   1244  -8605
-1810       C
ATOM   7820  C   GLU B 195      17.753 -60.276  66.761  1.00189.35
C
ANISOU 7820  C   GLU B 195    36373  18284  17287   1544  -8274
-1906       C
```

```
ATOM   7821  O   GLU B 195      18.890 -60.088  66.319  1.00184.89           
O
ANISOU 7821  O   GLU B 195    35465  17987  16799   2255  -8446  
-2209       O
ATOM   7822  CB  GLU B 195      17.570 -62.606  67.674  1.00191.62           
C
ANISOU 7822  CB  GLU B 195    38641  17254  16913   1451  -9104  
-1808       C
ATOM   7823  CG  GLU B 195      17.400 -62.128  69.109  1.00195.89           
C
ANISOU 7823  CG  GLU B 195    39314  17747  17368   1101  -8916  
-1576       C
ATOM   7824  CD  GLU B 195      17.746 -63.202  70.125  1.00208.69           
C
ANISOU 7824  CD  GLU B 195    42075  18647  18569   1224  -9424  
-1551       C
ATOM   7825  OE1 GLU B 195      16.848 -63.613  70.889  1.00211.57           
O
ANISOU 7825  OE1 GLU B 195    43066  18602  18718    546  -9370  
-1263       O
ATOM   7826  OE2 GLU B 195      18.916 -63.638  70.158  1.00206.34           
O
ANISOU 7826  OE2 GLU B 195    42058  18199  18142   1998  -9883  
-1831       O
ATOM   7827  N   VAL B 196      17.049 -59.336  67.393  1.00202.69           
N
ANISOU 7827  N   VAL B 196    37647  20257  19110    985  -7799  
-1654       N
ATOM   7828  CA  VAL B 196      17.558 -57.997  67.654  1.00204.13           
C
ANISOU 7828  CA  VAL B 196    36976  21050  19533   1160  -7454  
-1693       C
ATOM   7829  C   VAL B 196      17.408 -57.699  69.140  1.00209.61           
C
ANISOU 7829  C   VAL B 196    37929  21611  20103    857  -7351  
-1465       C
ATOM   7830  O   VAL B 196      16.574 -58.282  69.836  1.00209.05           
O
ANISOU 7830  O   VAL B 196    38505  21101  19824    311  -7378  
-1223       O
ATOM   7831  CB  VAL B 196      16.845 -56.920  66.808  1.00169.16           
C
ANISOU 7831  CB  VAL B 196    31631  17209  15434    814  -6928  
-1633       C
ATOM   7832  CG1 VAL B 196      17.159 -57.113  65.333  1.00168.58           
C
ANISOU 7832  CG1 VAL B 196    31243  17315  15495   1194  -7028  
-1888       C
ATOM   7833  CG2 VAL B 196      15.343 -56.962  67.049  1.00169.05           
C
ANISOU 7833  CG2 VAL B 196    31775  17058  15398    -39  -6637  
-1322       C
ATOM   7834  N   PHE B 197      18.235 -56.776  69.621  1.00208.37           
N
```

```
ANISOU 7834  N   PHE B 197    37258  21847  20065   1205  -7228
-1550       N
ATOM   7835  CA  PHE B 197      18.475 -56.598  71.045  1.00210.35
C
ANISOU 7835  CA  PHE B 197    37813  21939  20173   1149  -7253
-1413       C
ATOM   7836  C   PHE B 197      18.062 -55.199  71.483  1.00207.23
C
ANISOU 7836  C   PHE B 197    36654  22077  20006    784  -6713
-1233       C
ATOM   7837  O   PHE B 197      18.258 -54.222  70.753  1.00210.95
O
ANISOU 7837  O   PHE B 197    36278  23124  20749    922  -6425
-1339       O
ATOM   7838  CB  PHE B 197      19.948 -56.851  71.375  1.00216.34
C
ANISOU 7838  CB  PHE B 197    38751  22630  20821   1960  -7669
-1699       C
ATOM   7839  CG  PHE B 197      20.429 -58.212  70.955  1.00217.65
C
ANISOU 7839  CG  PHE B 197    39675  22281  20741   2399  -8241
-1912       C
ATOM   7840  CD1 PHE B 197      19.883 -59.356  71.513  1.00220.89
C
ANISOU 7840  CD1 PHE B 197    41114  21984  20831   2099  -8544
-1752       C
ATOM   7841  CD2 PHE B 197      21.416 -58.348  69.992  1.00213.37
C
ANISOU 7841  CD2 PHE B 197    38834  21968  20269   3100  -8480
-2278       C
ATOM   7842  CE1 PHE B 197      20.318 -60.612  71.126  1.00214.32
C
ANISOU 7842  CE1 PHE B 197    41027  20654  19751   2512  -9097
-1948       C
ATOM   7843  CE2 PHE B 197      21.856 -59.602  69.601  1.00213.41
C
ANISOU 7843  CE2 PHE B 197    39543  21506  20036   3534  -9028
-2488       C
ATOM   7844  CZ  PHE B 197      21.306 -60.734  70.169  1.00214.30
C
ANISOU 7844  CZ  PHE B 197    40708  20888  19828   3250  -9347
-2320       C
ATOM   7845  N   SER B 198      17.480 -55.124  72.679  1.00213.30
N
ANISOU 7845  N   SER B 198    37763  22639  20644    311  -6590
-963       N
ATOM   7846  CA  SER B 198      17.035 -53.862  73.256  1.00222.43
C
ANISOU 7846  CA  SER B 198    38295  24246  21973    -55  -6111
-774       C
ATOM   7847  C   SER B 198      18.175 -52.855  73.374  1.00215.77
C
ANISOU 7847  C   SER B 198    36804  23877  21302    500  -6040
-949       C
```

```
ATOM   7848  O   SER B 198      19.334 -53.208  73.607  1.00217.35
O
ANISOU 7848  O   SER B 198    37232  23947  21404   1112  -6396
-1161       O
ATOM   7849  CB  SER B 198      16.416 -54.103  74.633  1.00240.93
C
ANISOU 7849  CB  SER B 198    41224  26226  24091   -558  -6077
-490       C
ATOM   7850  OG  SER B 198      17.417 -54.277  75.620  1.00260.37
O
ANISOU 7850  OG  SER B 198    44072  28459  26396   -121  -6367
-553       O
ATOM   7851  N   GLY B 199      17.821 -51.579  73.205  1.00206.28
N
ANISOU 7851  N   GLY B 199    34791  23240  20347    274  -5581
-869       N
ATOM   7852  CA  GLY B 199      18.745 -50.469  73.309  1.00195.09
C
ANISOU 7852  CA  GLY B 199    32702  22324  19100    675  -5438
-998       C
ATOM   7853  C   GLY B 199      19.532 -50.156  72.056  1.00199.34
C
ANISOU 7853  C   GLY B 199    32668  23256  19814   1171  -5464
-1302       C
ATOM   7854  O   GLY B 199      20.084 -49.054  71.950  1.00193.38
O
ANISOU 7854  O   GLY B 199    31232  23014  19231   1366  -5242
-1388       O
ATOM   7855  N   SER B 200      19.610 -51.085  71.108  1.00201.69
N
ANISOU 7855  N   SER B 200    33235  23334  20062   1369  -5730
-1470       N
ATOM   7856  CA  SER B 200      20.250 -50.817  69.829  1.00187.28
C
ANISOU 7856  CA  SER B 200    30864  21895  18400   1788  -5732
-1756       C
ATOM   7857  C   SER B 200      19.510 -49.712  69.082  1.00172.56
C
ANISOU 7857  C   SER B 200    28248  20531  16785   1411  -5251
-1666       C
ATOM   7858  O   SER B 200      18.306 -49.508  69.265  1.00152.74
O
ANISOU 7858  O   SER B 200    25752  17975  14305    814  -4986
-1408       O
ATOM   7859  CB  SER B 200      20.295 -52.085  68.978  1.00191.37
C
ANISOU 7859  CB  SER B 200    31873  22036  18802   2006  -6105
-1919       C
ATOM   7860  OG  SER B 200      21.132 -51.908  67.849  1.00194.32
O
ANISOU 7860  OG  SER B 200    31768  22770  19295   2512  -6170
-2238       O
ATOM   7861  N   THR B 201      20.238 -48.986  68.236  1.00174.31
N
```

```
ANISOU 7861  N   THR B 201    27815  21246  17171   1764  -5142
-1894       N
ATOM   7862  CA  THR B 201      19.668 -47.864  67.502  1.00165.26
C
ANISOU 7862  CA  THR B 201    25962  20586  16245   1471  -4710
-1840       C
ATOM   7863  C   THR B 201      19.693 -48.204  66.018  1.00170.86
C
ANISOU 7863  C   THR B 201    26491  21391  17037   1643  -4768
-2046       C
ATOM   7864  O   THR B 201      20.744 -48.554  65.471  1.00170.81
O
ANISOU 7864  O   THR B 201    26439  21457  17003   2185  -5018
-2337       O
ATOM   7865  CB  THR B 201      20.441 -46.568  67.767  1.00160.70
C
ANISOU 7865  CB  THR B 201    24747  20534  15778   1663  -4483
-1910       C
ATOM   7866  OG1 THR B 201      21.845 -46.848  67.817  1.00174.20
O
ANISOU 7866  OG1 THR B 201    26476  22300  17413   2289  -4781
-2197       O
ATOM   7867  CG2 THR B 201      20.009 -45.954  69.091  1.00139.38
C
ANISOU 7867  CG2 THR B 201    22092  17818  13049   1319  -4288
-1636       C
ATOM   7868  N   CYS B 202      18.530 -48.097  65.382  1.00184.05
N
ANISOU 7868  N   CYS B 202    28050  23076  18803   1187  -4541
-1908       N
ATOM   7869  CA  CYS B 202      18.392 -48.321  63.949  1.00186.73
C
ANISOU 7869  CA  CYS B 202    28190  23519  19240   1282  -4548
-2074       C
ATOM   7870  C   CYS B 202      19.140 -47.301  63.101  1.00169.27
C
ANISOU 7870  C   CYS B 202    25255  21875  17183   1579  -4360
-2289       C
ATOM   7871  O   CYS B 202      19.271 -46.126  63.456  1.00166.61
O
ANISOU 7871  O   CYS B 202    24449  21923  16931   1486  -4074
-2231       O
ATOM   7872  CB  CYS B 202      16.921 -48.342  63.554  1.00166.22
C
ANISOU 7872  CB  CYS B 202    25613  20835  16710    695  -4324
-1872       C
ATOM   7873  SG  CYS B 202      16.103 -49.816  64.143  1.00172.13
S
ANISOU 7873  SG  CYS B 202    27265  20892  17246    369  -4606
-1694       S
ATOM   7874  N   LYS B 203      19.630 -47.784  61.962  1.00143.08
N
ANISOU 7874  N   LYS B 203    21879  18600  13885   1929  -4532
-2542       N
```

```
ATOM   7875  CA  LYS B 203      20.191 -46.963  60.903  1.00137.77
C
ANISOU 7875  CA  LYS B 203    20564  18437  13345   2151  -4354
-2759       C
ATOM   7876  C   LYS B 203      19.827 -47.600  59.570  1.00160.09
C
ANISOU 7876  C   LYS B 203    23440  21168  16217   2193  -4443
-2882       C
ATOM   7877  O   LYS B 203      19.803 -48.827  59.447  1.00161.76
O
ANISOU 7877  O   LYS B 203    24184  20957  16319   2340  -4781
-2933       O
ATOM   7878  CB  LYS B 203      21.718 -46.827  61.016  1.00137.41
C
ANISOU 7878  CB  LYS B 203    20319  18651  13238   2727  -4525
-3046       C
ATOM   7879  CG  LYS B 203      22.206 -46.007  62.201  1.00157.31
C
ANISOU 7879  CG  LYS B 203    22669  21365  15736   2723  -4403
-2962       C
ATOM   7880  CD  LYS B 203      23.721 -45.869  62.181  1.00170.12
C
ANISOU 7880  CD  LYS B 203    24041  23299  17299   3294  -4566
-3288       C
ATOM   7881  CE  LYS B 203      24.211 -44.907  63.253  1.00166.73
C
ANISOU 7881  CE  LYS B 203    23358  23127  16867   3271  -4403
-3214       C
ATOM   7882  NZ  LYS B 203      25.688 -44.990  63.440  1.00147.58
N
ANISOU 7882  NZ  LYS B 203    20798  20929  14346   3841  -4631
-3540       N
ATOM   7883  N   GLN B 204      19.550 -46.756  58.579  1.00175.67
N
ANISOU 7883  N   GLN B 204    24884  23521  18342   2068  -4151
-2930       N
ATOM   7884  CA  GLN B 204      19.410 -47.179  57.185  1.00194.01
C
ANISOU 7884  CA  GLN B 204    27138  25854  20722   2183  -4211
-3096       C
ATOM   7885  C   GLN B 204      18.311 -48.225  56.973  1.00193.41
C
ANISOU 7885  C   GLN B 204    27573  25293  20621   1901  -4352
-2954       C
ATOM   7886  O   GLN B 204      18.495 -49.191  56.229  1.00208.77
O
ANISOU 7886  O   GLN B 204    29785  27017  22520   2154  -4627
-3109       O
ATOM   7887  CB  GLN B 204      20.747 -47.695  56.647  1.00222.00
C
ANISOU 7887  CB  GLN B 204    30658  29502  24191   2804  -4503
-3442       C
ATOM   7888  CG  GLN B 204      21.898 -46.715  56.812  1.00242.69
C
```

```
ANISOU 7888  CG  GLN B 204    32763  32633  26817   3081  -4373
-3620       C
ATOM   7889  CD  GLN B 204      23.202 -47.243  56.247  1.00249.93
C
ANISOU 7889  CD  GLN B 204    33617  33702  27644   3692  -4660
-3999       C
ATOM   7890  OE1 GLN B 204      23.225 -48.263  55.557  1.00255.21
O
ANISOU 7890  OE1 GLN B 204    34578  34127  28264   3925  -4937
-4138       O
ATOM   7891  NE2 GLN B 204      24.297 -46.551  56.540  1.00256.42
N
ANISOU 7891  NE2 GLN B 204    34052  34944  28433   3958  -4601
-4180       N
ATOM   7892  N   GLY B 205      17.166 -48.052  57.620  1.00189.36
N
ANISOU 7892  N   GLY B 205    27200  24623  20125   1372  -4172
-2668       N
ATOM   7893  CA  GLY B 205      16.051 -48.939  57.341  1.00192.06
C
ANISOU 7893  CA  GLY B 205    27960  24569  20445   1037  -4258
-2542       C
ATOM   7894  C   GLY B 205      14.960 -48.889  58.387  1.00181.00
C
ANISOU 7894  C   GLY B 205    26798  22973  19001    477  -4112
-2240       C
ATOM   7895  O   GLY B 205      15.084 -48.257  59.436  1.00205.80
O
ANISOU 7895  O   GLY B 205    29847  26231  22116    369  -3977
-2114       O
ATOM   7896  N   GLU B 206      13.868 -49.586  58.060  1.00165.45
N
ANISOU 7896  N   GLU B 206    25137  20711  17016    110  -4141
-2136       N
ATOM   7897  CA  GLU B 206      12.632 -49.617  58.833  1.00152.08
C
ANISOU 7897  CA  GLU B 206    23647  18859  15278   -499  -3981
-1874       C
ATOM   7898  C   GLU B 206      12.002 -51.001  58.714  1.00151.75
C
ANISOU 7898  C   GLU B 206    24262  18295  15102   -708  -4246
-1817       C
ATOM   7899  O   GLU B 206      12.029 -51.597  57.635  1.00156.72
O
ANISOU 7899  O   GLU B 206    24970  18814  15764   -529  -4405
-1961       O
ATOM   7900  CB  GLU B 206      11.649 -48.545  58.343  1.00155.19
C
ANISOU 7900  CB  GLU B 206    23477  19647  15841   -869  -3579
-1815       C
ATOM   7901  CG  GLU B 206      10.290 -48.568  59.024  1.00179.15
C
ANISOU 7901  CG  GLU B 206    26656  22584  18828  -1508  -3399
-1585       C
```

ATOM   7902 CD GLU B 206   9.302 -47.612 58.381 1.00195.05
C
ANISOU 7902 CD GLU B 206   28129 24982 20999 -1812 -3053
-1581   C
ATOM   7903 OE1 GLU B 206   8.259 -48.081 57.879 1.00202.73
O
ANISOU 7903 OE1 GLU B 206   29210 25838 21981 -2155 -3026
-1558   O
ATOM   7904 OE2 GLU B 206   9.568 -46.391 58.376 1.00200.64
O
ANISOU 7904 OE2 GLU B 206   28320 26106 21808 -1707 -2819
-1610   O
ATOM   7905 N   ILE B 207   11.434 -51.513 59.810 1.00173.12
N
ANISOU 7905 N   ILE B 207   27455 20676 17646 -1099 -4295
-1609   N
ATOM   7906 CA ILE B 207   10.955 -52.895 59.813 1.00185.59
C
ANISOU 7906 CA ILE B 207   29757 21712 19048 -1297 -4587
-1553   C
ATOM   7907 C   ILE B 207   9.911 -53.105 60.907 1.00189.50
C
ANISOU 7907 C   ILE B 207   30601 22001 19399 -1950 -4469
-1292   C
ATOM   7908 O   ILE B 207   9.697 -52.232 61.756 1.00202.51
O
ANISOU 7908 O   ILE B 207   31962 23907 21075 -2173 -4201
-1164   O
ATOM   7909 CB ILE B 207   12.130 -53.877 59.989 1.00204.33
C
ANISOU 7909 CB ILE B 207   32685 23702 21250   -756 -5051
-1684   C
ATOM   7910 CG1 ILE B 207   11.812 -55.232 59.351 1.00218.23
C
ANISOU 7910 CG1 ILE B 207   35058 24978 22881   -782 -5385
-1727   C
ATOM   7911 CG2 ILE B 207   12.459 -54.057 61.466 1.00215.76
C
ANISOU 7911 CG2 ILE B 207   34565 24909 22506   -811 -5162
-1541   C
ATOM   7912 CD1 ILE B 207   13.015 -56.140 59.225 1.00223.40
C
ANISOU 7912 CD1 ILE B 207   36176 25318 23387   -142 -5863
-1923   C
ATOM   7913 N   GLU B 208   9.252 -54.264 60.886 1.00187.73
N
ANISOU 7913 N   GLU B 208   31003 21318 19008 -2272 -4670
-1218   N
ATOM   7914 CA GLU B 208   8.433 -54.761 61.982 1.00177.17
C
ANISOU 7914 CA GLU B 208   30182 19677 17457 -2865 -4652
-989   C
ATOM   7915 C   GLU B 208   9.142 -55.914 62.686 1.00180.88
C

ANISOU 7915 C   GLU B 208   31511 19563 17650 -2656 -5094
-966   C
ATOM   7916 O   GLU B 208   9.943 -56.632 62.081 1.00184.64
O
ANISOU 7916 O   GLU B 208   32283 19790 18081 -2152 -5453
-1135   O
ATOM   7917 CB GLU B 208   7.061 -55.221 61.479 1.00158.65
C
ANISOU 7917 CB GLU B 208   27943 17245 15091 -3476 -4538
-917   C
ATOM   7918 CG GLU B 208   6.155 -54.089 61.018 1.00153.80
C
ANISOU 7918 CG GLU B 208   26544 17189 14703 -3785 -4092
-918   C
ATOM   7919 CD GLU B 208   4.981 -54.581 60.192 1.00179.46
C
ANISOU 7919 CD GLU B 208   29831 20387 17968 -4230 -4031
-931   C
ATOM   7920 OE1 GLU B 208   5.193 -54.969 59.025 1.00203.19
O
ANISOU 7920 OE1 GLU B 208   32817 23321 21066 -3934 -4190
-1086   O
ATOM   7921 OE2 GLU B 208   3.845 -54.582 60.710 1.00183.02
O
ANISOU 7921 OE2 GLU B 208   30322 20886 18333 -4880 -3825
-797   O
ATOM   7922 N   ALA B 209   8.837 -56.092 63.972 1.00195.36
N
ANISOU 7922 N   ALA B 209   33764 21179 19284 -3038 -5076
-767   N
ATOM   7923 CA ALA B 209   9.453 -57.153 64.757 1.00191.28
C
ANISOU 7923 CA ALA B 209   34123 20082 18474 -2876 -5496
-732   C
ATOM   7924 C   ALA B 209   8.485 -57.631 65.830 1.00200.19
C
ANISOU 7924 C   ALA B 209   35816 20899 19349 -3608 -5432
-480   C
ATOM   7925 O   ALA B 209   7.491 -56.970 66.140 1.00213.95
O
ANISOU 7925 O   ALA B 209   37175 22956 21159 -4176 -5036
-341   O
ATOM   7926 CB ALA B 209   10.768 -56.693 65.396 1.00192.19
C
ANISOU 7926 CB ALA B 209   34136 20289 18599 -2269 -5619
-817   C
ATOM   7927 N   VAL B 210   8.789 -58.800 66.391 1.00209.88
N
ANISOU 7927 N   VAL B 210   37976 21505 20263 -3587 -5837
-435   N
ATOM   7928 CA VAL B 210   8.048 -59.374 67.510 1.00186.47
C
ANISOU 7928 CA VAL B 210   35691 18164 16996 -4246 -5839
-201   C

```
ATOM   7929  C   VAL B 210       8.962 -59.344  68.729  1.00179.65
C
ANISOU 7929  C   VAL B 210    35196  17093  15971  -3939  -6021
-155       C
ATOM   7930  O   VAL B 210       9.980 -60.045  68.766  1.00182.10
O
ANISOU 7930  O   VAL B 210    36040  17012  16137  -3373  -6469
-279       O
ATOM   7931  CB  VAL B 210       7.570 -60.801  67.208  1.00183.74
C
ANISOU 7931  CB  VAL B 210    36233  17210  16370  -4547  -6173
-170       C
ATOM   7932  CG1 VAL B 210       6.936 -61.423  68.446  1.00187.96
C
ANISOU 7932  CG1 VAL B 210    37550  17321  16545  -5214  -6205
66       C
ATOM   7933  CG2 VAL B 210       6.590 -60.798  66.041  1.00182.66
C
ANISOU 7933  CG2 VAL B 210    35707  17299  16396  -4895  -5970
-211       C
ATOM   7934  N   VAL B 211       8.588 -58.550  69.737  1.00186.40
N
ANISOU 7934  N   VAL B 211    35781  18206  16837  -4302  -5689
13       N
ATOM   7935  CA  VAL B 211       9.324 -58.531  70.997  1.00204.59
C
ANISOU 7935  CA  VAL B 211    38473  20292  18968  -4099  -5839
85       C
ATOM   7936  C   VAL B 211       9.249 -59.892  71.676  1.00196.69
C
ANISOU 7936  C   VAL B 211    38645  18540  17549  -4337  -6235
187       C
ATOM   7937  O   VAL B 211       8.219 -60.578  71.631  1.00206.20
O
ANISOU 7937  O   VAL B 211    40289  19488  18568  -4998  -6205
314       O
ATOM   7938  CB  VAL B 211       8.793 -57.420  71.918  1.00198.76
C
ANISOU 7938  CB  VAL B 211    37218  19985  18318  -4512  -5381
259       C
ATOM   7939  CG1 VAL B 211       9.141 -56.054  71.351  1.00215.76
C
ANISOU 7939  CG1 VAL B 211    38302  22826  20851  -4138  -5069
139       C
ATOM   7940  CG2 VAL B 211       7.289 -57.556  72.108  1.00197.49
C
ANISOU 7940  CG2 VAL B 211    37126  19862  18051  -5427  -5080
445       C
ATOM   7941  N   ILE B 212      10.355 -60.297  72.302  1.00187.36
N
ANISOU 7941  N   ILE B 212    37995  16995  16198  -3795  -6623
120       N
ATOM   7942  CA  ILE B 212      10.508 -61.684  72.726  1.00193.36
C
```

```
ANISOU 7942  CA  ILE B 212    39935  16983  16550  -3835  -7112
152       C
ATOM   7943  C   ILE B 212      11.209 -61.795  74.078  1.00195.44
C
ANISOU 7943  C   ILE B 212    40756  16924  16576  -3639  -7324
218       C
ATOM   7944  O   ILE B 212      11.091 -62.823  74.755  1.00203.55
O
ANISOU 7944  O   ILE B 212    42818  17308  17215  -3881  -7642
321       O
ATOM   7945  CB  ILE B 212      11.251 -62.499  71.652  1.00195.09
C
ANISOU 7945  CB  ILE B 212    40438  16933  16755  -3192  -7573
-98       C
ATOM   7946  CG1 ILE B 212      10.829 -63.968  71.716  1.00205.04
C
ANISOU 7946  CG1 ILE B 212    42855  17441  17611  -3524  -7974
-26       C
ATOM   7947  CG2 ILE B 212      12.759 -62.357  71.806  1.00194.58
C
ANISOU 7947  CG2 ILE B 212    40334  16871  16726  -2255  -7900
-327       C
ATOM   7948  CD1 ILE B 212       9.370 -64.200  71.394  1.00215.64
C
ANISOU 7948  CD1 ILE B 212    44226  18795  18912  -4429  -7681
155       C
ATOM   7949  N   ALA B 213      11.938 -60.758  74.492  1.00194.46
N
ANISOU 7949  N   ALA B 213    39995  17224  16666  -3213  -7160
161       N
ATOM   7950  CA  ALA B 213      12.432 -60.720  75.863  1.00193.23
C
ANISOU 7950  CA  ALA B 213    40285  16825  16307  -3135  -7276
256       C
ATOM   7951  C   ALA B 213      12.551 -59.285  76.356  1.00192.17
C
ANISOU 7951  C   ALA B 213    39235  17324  16456  -3100  -6835
310       C
ATOM   7952  O   ALA B 213      13.029 -58.406  75.634  1.00205.38
O
ANISOU 7952  O   ALA B 213    40028  19541  18466  -2665  -6664
149       O
ATOM   7953  CB  ALA B 213      13.789 -61.425  75.981  1.00196.12
C
ANISOU 7953  CB  ALA B 213    41252  16764  16503  -2298  -7864
32       C
ATOM   7954  N   THR B 214      12.106 -59.065  77.593  1.00188.90
N
ANISOU 7954  N   THR B 214    39064  16825  15883  -3576  -6656
539       N
ATOM   7955  CA  THR B 214      12.220 -57.766  78.246  1.00191.54
C
ANISOU 7955  CA  THR B 214    38656  17687  16432  -3561  -6275
613       C
```

```
ATOM   7956  C   THR B 214      13.674 -57.340  78.436  1.00204.28
C
ANISOU 7956  C   THR B 214    40048  19410  18159  -2687  -6500
413       C
ATOM   7957  O   THR B 214      14.585 -58.168  78.528  1.00206.48
O
ANISOU 7957  O   THR B 214    40973  19233  18246  -2155  -6993
259       O
ATOM   7958  CB  THR B 214      11.515 -57.788  79.603  1.00215.95
C
ANISOU 7958  CB  THR B 214    42197  20579  19273  -4221  -6109
889       C
ATOM   7959  OG1 THR B 214      11.964 -58.917  80.365  1.00229.42
O
ANISOU 7959  OG1 THR B 214    45035  21548  20587  -4125  -6579
915       O
ATOM   7960  CG2 THR B 214      10.012 -57.854  79.422  1.00206.14
C
ANISOU 7960  CG2 THR B 214    40879  19459  17986  -5129  -5752
1069       C
ATOM   7961  N   GLY B 215      13.873 -56.017  78.472  1.00211.24
N
ANISOU 7961  N   GLY B 215    39993  20922  19346  -2542  -6135
401       N
ATOM   7962  CA  GLY B 215      15.209 -55.436  78.548  1.00194.99
C
ANISOU 7962  CA  GLY B 215    37554  19095  17437  -1755  -6273
197       C
ATOM   7963  C   GLY B 215      16.091 -56.027  79.633  1.00181.86
C
ANISOU 7963  C   GLY B 215    36662  16936  15500  -1382  -6686
168       C
ATOM   7964  O   GLY B 215      17.313 -56.092  79.480  1.00179.58
O
ANISOU 7964  O   GLY B 215    36346  16645  15240   -627  -7000
-85       O
ATOM   7965  N   VAL B 216      15.489 -56.459  80.745  1.00182.29
N
ANISOU 7965  N   VAL B 216    37412  16579  15273  -1902  -6692
411       N
ATOM   7966  CA  VAL B 216      16.254 -56.957  81.885  1.00187.60
C
ANISOU 7966  CA  VAL B 216    38845  16766  15667  -1593  -7063
406       C
ATOM   7967  C   VAL B 216      16.878 -58.314  81.586  1.00195.24
C
ANISOU 7967  C   VAL B 216    40725  17101  16358  -1159  -7671
217       C
ATOM   7968  O   VAL B 216      17.876 -58.692  82.213  1.00193.23
O
ANISOU 7968  O   VAL B 216    40973  16519  15928   -602  -8076
82       O
ATOM   7969  CB  VAL B 216      15.352 -57.017  83.135  1.00206.64
C
```

ANISOU 7969 CB VAL B 216   41748 18925 17842 -2337 -6869
730   C
ATOM   7970 CG1 VAL B 216   14.207 -58.004 82.926 1.00193.74
C
ANISOU 7970 CG1 VAL B 216   40779 16880 15953 -3061 -6887
888   C
ATOM   7971 CG2 VAL B 216   16.160 -57.356 84.388 1.00190.40
C
ANISOU 7971 CG2 VAL B 216   40405 16417 15522 -2008 -7209
735   C
ATOM   7972 N   HIS B 217   16.333 -59.045 80.620 1.00207.33
N
ANISOU 7972 N   HIS B 217   42475 18459 17843 -1368 -7762
185   N
ATOM   7973 CA HIS B 217   16.901 -60.314 80.192 1.00216.82
C
ANISOU 7973 CA HIS B 217   44501 19090 18792   -931 -8347
-13   C
ATOM   7974 C   HIS B 217   17.920 -60.144 79.074 1.00221.55
C
ANISOU 7974 C   HIS B 217   44529 20020 19629   -108 -8533
-367   C
ATOM   7975 O   HIS B 217   18.676 -61.081 78.793 1.00226.71
O
ANISOU 7975 O   HIS B 217   45787 20265 20086   454 -9066
-598   O
ATOM   7976 CB HIS B 217   15.786 -61.260 79.733 1.00214.20
C
ANISOU 7976 CB HIS B 217   44765 18364 18257 -1594 -8376
135   C
ATOM   7977 CG HIS B 217   14.813 -61.613 80.814 1.00216.68
C
ANISOU 7977 CG HIS B 217   45772 18292 18267 -2422 -8251
456   C
ATOM   7978 ND1 HIS B 217   13.512 -61.161 80.820 1.00211.25
N
ANISOU 7978 ND1 HIS B 217   44705 17898 17664 -3285 -7738
701   N
ATOM   7979 CD2 HIS B 217   14.952 -62.370 81.928 1.00216.73
C
ANISOU 7979 CD2 HIS B 217   46828 17653 17866 -2526 -8573
558   C
ATOM   7980 CE1 HIS B 217   12.890 -61.627 81.889 1.00207.20
C
ANISOU 7980 CE1 HIS B 217   44958 16961 16810 -3906 -7733
939   C
ATOM   7981 NE2 HIS B 217   13.742 -62.364 82.578 1.00211.92
N
ANISOU 7981 NE2 HIS B 217   46450 16968 17101 -3471 -8234
869   N
ATOM   7982 N   THR B 218   17.944 -58.979 78.429 1.00218.61
N
ANISOU 7982 N   THR B 218   43030 20379 19655   -33 -8112
-423   N

ATOM   7983 CA THR B 218   18.970 -58.676 77.441 1.00229.82
C
ANISOU 7983 CA THR B 218   43831 22187 21303   731 -8239
-764   C
ATOM   7984 C   THR B 218   20.365 -58.747 78.053 1.00234.19
C
ANISOU 7984 C   THR B 218   44582 22646 21755   1527 -8630
-1008   C
ATOM   7985 O   THR B 218   20.582 -58.348 79.201 1.00254.80
O
ANISOU 7985 O   THR B 218   47294 25221 24299   1498 -8576
-898   O
ATOM   7986 CB THR B 218   18.738 -57.274 76.868 1.00237.66
C
ANISOU 7986 CB THR B 218   43613 23977 22709   602 -7681
-744   C
ATOM   7987 OG1 THR B 218   17.406 -57.179 76.354 1.00250.80
O
ANISOU 7987 OG1 THR B 218   45088 25744 24461   -134 -7320
-530   O
ATOM   7988 CG2 THR B 218   19.727 -56.972 75.745 1.00244.47
C
ANISOU 7988 CG2 THR B 218   43831 25261 23795   1316 -7783
-1095   C
ATOM   7989 N   PHE B 219   21.311 -59.270 77.264 1.00231.34
N
ANISOU 7989 N   PHE B 219   44267 22256 21374   2250 -9034
-1358   N
ATOM   7990 CA PHE B 219   22.667 -59.544 77.737 1.00235.29
C
ANISOU 7990 CA PHE B 219   45043 22625 21731   3072 -9496
-1655   C
ATOM   7991 C   PHE B 219   23.327 -58.297 78.322 1.00248.59
C
ANISOU 7991 C   PHE B 219   45948 24867 23638   3308 -9217
-1698   C
ATOM   7992 O   PHE B 219   24.037 -58.374 79.331 1.00261.05
O
ANISOU 7992 O   PHE B 219   47890 26244 25053   3652 -9465
-1764   O
ATOM   7993 CB PHE B 219   23.510 -60.130 76.598 1.00218.17
C
ANISOU 7993 CB PHE B 219   42825 20506 19564   3792 -9890
-2054   C
ATOM   7994 CG PHE B 219   23.856 -59.139 75.521 1.00214.16
C
ANISOU 7994 CG PHE B 219   41149 20784 19440   4023 -9551
-2239   C
ATOM   7995 CD1 PHE B 219   25.082 -58.492 75.522 1.00217.00
C
ANISOU 7995 CD1 PHE B 219   40903 21619 19928   4699 -9602
-2548   C
ATOM   7996 CD2 PHE B 219   22.954 -58.853 74.512 1.00214.81
C

```
ANISOU 7996  CD2 PHE B 219    40752  21129  19737   3550  -9184
-2113       C
ATOM   7997  CE1 PHE B 219      25.399 -57.578  74.536  1.00217.69
C
ANISOU 7997  CE1 PHE B 219    39950  22422  20338   4867  -9284
-2716       C
ATOM   7998  CE2 PHE B 219      23.266 -57.941  73.523  1.00219.56
C
ANISOU 7998  CE2 PHE B 219    40330  22426  20668   3747  -8880
-2280       C
ATOM   7999  CZ  PHE B 219      24.490 -57.302  73.535  1.00216.67
C
ANISOU 7999  CZ  PHE B 219    39391  22520  20414   4392  -8926
-2576       C
ATOM   8000  N   PHE B 220      23.108 -57.142  77.689  1.00226.64
N
ANISOU 8000  N   PHE B 220    42115  22779  21219   3133  -8713
-1668       N
ATOM   8001  CA  PHE B 220      23.633 -55.875  78.194  1.00220.59
C
ANISOU 8001  CA  PHE B 220    40577  22566  20670   3275  -8402
-1681       C
ATOM   8002  C   PHE B 220      23.139 -55.570  79.605  1.00217.96
C
ANISOU 8002  C   PHE B 220    40565  22020  20230   2815  -8232
-1359       C
ATOM   8003  O   PHE B 220      23.917 -55.129  80.459  1.00198.35
O
ANISOU 8003  O   PHE B 220    38004  19630  17732   3156  -8298
-1428       O
ATOM   8004  CB  PHE B 220      23.245 -54.755  77.228  1.00206.62
C
ANISOU 8004  CB  PHE B 220    37739  21498  19269   3033  -7879
-1653       C
ATOM   8005  CG  PHE B 220      23.713 -53.392  77.644  1.00210.87
C
ANISOU 8005  CG  PHE B 220    37461  22628  20033   3125  -7534
-1653       C
ATOM   8006  CD1 PHE B 220      22.803 -52.433  78.059  1.00184.25
C
ANISOU 8006  CD1 PHE B 220    33680  19515  16813   2494  -7030
-1332       C
ATOM   8007  CD2 PHE B 220      25.057 -53.061  77.602  1.00219.23
C
ANISOU 8007  CD2 PHE B 220    38152  24003  21141   3840  -7717
-1988       C
ATOM   8008  CE1 PHE B 220      23.225 -51.173  78.434  1.00175.48
C
ANISOU 8008  CE1 PHE B 220    31850  18930  15895   2577  -6727
-1327       C
ATOM   8009  CE2 PHE B 220      25.487 -51.801  77.977  1.00203.88
C
ANISOU 8009  CE2 PHE B 220    35472  22601  19393   3897  -7400
-1985       C
```

```
ATOM   8010  CZ  PHE B 220      24.569 -50.856  78.394  1.00186.71
C
ANISOU 8010  CZ  PHE B 220    32934  20643  17364   3266  -6910
-1646       C
ATOM   8011  N   GLY B 221      21.858 -55.798  79.873  1.00202.00
N
ANISOU 8011  N   GLY B 221    38898  19727  18126   2041  -8013
-1018       N
ATOM   8012  CA  GLY B 221      21.294 -55.466  81.168  1.00180.39
C
ANISOU 8012  CA  GLY B 221    36416  16835  15288   1542  -7805
-704       C
ATOM   8013  C   GLY B 221      21.356 -56.608  82.163  1.00185.96
C
ANISOU 8013  C   GLY B 221    38307  16763  15585   1536  -8249
-637       C
ATOM   8014  O   GLY B 221      21.387 -56.386  83.378  1.00186.47
O
ANISOU 8014  O   GLY B 221    38652  16669  15530   1408  -8219
-480       O
ATOM   8015  N   LYS B 222      21.371 -57.839  81.648  1.00190.27
N
ANISOU 8015  N   LYS B 222    39586  16806  15901   1674  -8673
-755       N
ATOM   8016  CA  LYS B 222      21.440 -59.021  82.503  1.00196.10
C
ANISOU 8016  CA  LYS B 222    41555  16745  16208   1684  -9150
-710       C
ATOM   8017  C   LYS B 222      22.743 -59.083  83.298  1.00205.30
C
ANISOU 8017  C   LYS B 222    42991  17768  17245   2447  -9551
-944       C
ATOM   8018  O   LYS B 222      22.740 -59.470  84.472  1.00207.72
O
ANISOU 8018  O   LYS B 222    44069  17585  17268   2336  -9741
-810       O
ATOM   8019  CB  LYS B 222      21.269 -60.280  81.652  1.00200.19
C
ANISOU 8019  CB  LYS B 222    42751  16794  16519   1747  -9545
-826       C
ATOM   8020  CG  LYS B 222      21.208 -61.574  82.440  1.00206.59
C
ANISOU 8020  CG  LYS B 222    44925  16725  16845   1676 -10048
-761       C
ATOM   8021  CD  LYS B 222      20.024 -61.566  83.395  1.00207.32
C
ANISOU 8021  CD  LYS B 222    45452  16550  16770    737  -9740
-344       C
ATOM   8022  CE  LYS B 222      19.843 -62.916  84.068  1.00220.90
C
ANISOU 8022  CE  LYS B 222    48593  17362  17976    562 -10227
-261       C
ATOM   8023  NZ  LYS B 222      20.986 -63.251  84.961  1.00217.11
N
```

```
ANISOU 8023  NZ  LYS B 222    48724  16508  17259   1261 -10722
-448       N
ATOM   8024  N   ALA B 223      23.867 -58.722  82.675  1.00204.83
N
ANISOU 8024  N   ALA B 223    42320  18132  17376   3222  -9692
-1306       N
ATOM   8025  CA  ALA B 223      25.163 -58.788  83.346  1.00217.25
C
ANISOU 8025  CA  ALA B 223    44090  19625  18831   4002 -10093
-1585       C
ATOM   8026  C   ALA B 223      25.200 -57.925  84.605  1.00233.78
C
ANISOU 8026  C   ALA B 223    46001  21854  20971   3819  -9831
-1388       C
ATOM   8027  O   ALA B 223      24.650 -56.820  84.644  1.00224.35
O
ANISOU 8027  O   ALA B 223    44019  21167  20055   3363  -9272
-1172       O
ATOM   8028  CB  ALA B 223      26.271 -58.356  82.383  1.00197.94
C
ANISOU 8028  CB  ALA B 223    40817  17766  16625   4768 -10176
-2006       C
ATOM   8029  N   ALA B 224      25.862 -58.447  85.641  1.00255.41
N
ANISOU 8029  N   ALA B 224    49502  24121  23422   4202 -10259
-1474       N
ATOM   8030  CA  ALA B 224      25.945 -57.822  86.957  1.00258.99
C
ANISOU 8030  CA  ALA B 224    49984  24570  23852   4073 -10105
-1294       C
ATOM   8031  C   ALA B 224      27.390 -57.804  87.440  1.00258.02
C
ANISOU 8031  C   ALA B 224    49896  24476  23662   4982 -10522
-1660       C
ATOM   8032  O   ALA B 224      28.041 -58.852  87.489  1.00249.98
O
ANISOU 8032  O   ALA B 224    49671  22963  22347   5532 -11122
-1922       O
ATOM   8033  CB  ALA B 224      25.060 -58.556  87.971  1.00257.05
C
ANISOU 8033  CB  ALA B 224    50822  23596  23249   3448 -10187
-951       C
ATOM   8034  N   HIS B 225      27.882 -56.621  87.811  1.00256.21
N
ANISOU 8034  N   HIS B 225    48832  24823  23695   5142 -10219
-1688       N
ATOM   8035  CA  HIS B 225      29.277 -56.432  88.198  1.00244.69
C
ANISOU 8035  CA  HIS B 225    47216  23531  22224   5996 -10549
-2061       C
ATOM   8036  C   HIS B 225      29.371 -55.263  89.174  1.00249.99
C
ANISOU 8036  C   HIS B 225    47370  24550  23065   5838 -10181
-1884       C
```

```
ATOM   8037  O   HIS B 225      28.368 -54.631  89.517  1.00254.89
O
ANISOU 8037  O   HIS B 225    47766  25278  23803   5099  -9692
-1487       O
ATOM   8038  CB  HIS B 225      30.190 -56.230  86.980  1.00226.92
C
ANISOU 8038  CB  HIS B 225    44192  21849  20178   6640 -10642
-2498       C
ATOM   8039  CG  HIS B 225      30.180 -57.377  86.018  1.00228.68
C
ANISOU 8039  CG  HIS B 225    44913  21745  20231   6868 -11036
-2701       C
ATOM   8040  ND1 HIS B 225      29.511 -57.341  84.814  1.00236.74
N
ANISOU 8040  ND1 HIS B 225    45510  23010  21430   6517 -10768
-2634       N
ATOM   8041  CD2 HIS B 225      30.760 -58.599  86.088  1.00232.17
C
ANISOU 8041  CD2 HIS B 225    46266  21620  20327   7430 -11697
-2976       C
ATOM   8042  CE1 HIS B 225      29.682 -58.489  84.183  1.00237.07
C
ANISOU 8042  CE1 HIS B 225    46168  22654  21252   6844 -11238
-2851       C
ATOM   8043  NE2 HIS B 225      30.435 -59.270  84.935  1.00239.35
N
ANISOU 8043  NE2 HIS B 225    47280  22446  21215   7403 -11812
-3063       N
ATOM   8044  N   LEU B 226      30.596 -54.992  89.629  1.00248.19
N
ANISOU 8044  N   LEU B 226    46960  24505  22837   6556 -10436
-2199       N
ATOM   8045  CA  LEU B 226      30.857 -53.891  90.551  1.00229.75
C
ANISOU 8045  CA  LEU B 226    44128  22510  20655   6514 -10143
-2084       C
ATOM   8046  C   LEU B 226      30.395 -52.539  90.009  1.00256.98
C
ANISOU 8046  C   LEU B 226    46419  26717  24504   6068  -9482
-1906       C
ATOM   8047  O   LEU B 226      30.440 -52.267  88.806  1.00269.53
O
ANISOU 8047  O   LEU B 226    47335  28769  26303   6119  -9318
-2055       O
ATOM   8048  CB  LEU B 226      32.349 -53.828  90.888  1.00199.63
C
ANISOU 8048  CB  LEU B 226    40188  18863  16799   7429 -10542
-2532       C
ATOM   8049  CG  LEU B 226      32.964 -55.057  91.561  1.00199.69
C
ANISOU 8049  CG  LEU B 226    41314  18146  16415   7982 -11226
-2758       C
ATOM   8050  CD1 LEU B 226      34.454 -54.856  91.790  1.00201.02
C
```

ANISOU 8050 CD1 LEU B 226   41032 18597 16751   8836 -11421
-3234   C
ATOM   8051 CD2 LEU B 226   32.260 -55.365 92.872 1.00203.22
C
ANISOU 8051 CD2 LEU B 226   42634 17929 16650   7490 -11212
-2371   C
ATOM   8052 N   VAL B 227   29.940 -51.696 90.942 1.00254.71
N
ANISOU 8052 N   VAL B 227   45945 26531 24300   5632 -9116
-1583   N
ATOM   8053 CA VAL B 227   29.307 -50.421 90.622 1.00251.58
C
ANISOU 8053 CA VAL B 227   44598 26757 24234   5110 -8485
-1342   C
ATOM   8054 C   VAL B 227   30.240 -49.500 89.841 1.00245.69
C
ANISOU 8054 C   VAL B 227   42805 26771 23773   5570 -8350
-1658   C
ATOM   8055 O   VAL B 227   31.456 -49.459 90.073 1.00251.65
O
ANISOU 8055 O   VAL B 227   43452 27664 24498   6253 -8646
-2001   O
ATOM   8056 CB VAL B 227   28.809 -49.751 91.915 1.00240.42
C
ANISOU 8056 CB VAL B 227   43266 25271 22810   4675 -8206
-984   C
ATOM   8057 CG1 VAL B 227   29.979 -49.336 92.801 1.00238.63
C
ANISOU 8057 CG1 VAL B 227   42952 25147 22569   5273 -8412
-1185   C
ATOM   8058 CG2 VAL B 227   27.914 -48.558 91.598 1.00230.42
C
ANISOU 8058 CG2 VAL B 227   41159 24555 21834   4047 -7569
-693   C
ATOM   8059 N   ASP B 228   29.666 -48.771 88.884 1.00236.07
N
ANISOU 8059 N   ASP B 228   40823 26056 22816   5187 -7905
-1557   N
ATOM   8060 CA ASP B 228   30.361 -47.732 88.130 1.00224.95
C
ANISOU 8060 CA ASP B 228   38378 25407 21685   5456 -7673
-1786   C
ATOM   8061 C   ASP B 228   29.540 -46.455 88.255 1.00228.48
C
ANISOU 8061 C   ASP B 228   38176 26273 22365   4835 -7080
-1440   C
ATOM   8062 O   ASP B 228   28.367 -46.436 87.869 1.00233.56
O
ANISOU 8062 O   ASP B 228   38820 26863 23059   4228 -6791
-1161   O
ATOM   8063 CB ASP B 228   30.536 -48.132 86.663 1.00206.68
C
ANISOU 8063 CB ASP B 228   35779 23307 19443   5660 -7755
-2058   C

```
ATOM   8064  CG  ASP B 228      31.403 -47.158  85.891  1.00186.42
C
ANISOU 8064  CG  ASP B 228    32210  21507  17115   5993  -7571
-2348       C
ATOM   8065  OD1 ASP B 228      31.460 -47.271  84.649  1.00186.60
O
ANISOU 8065  OD1 ASP B 228    31871  21797  17232   6074  -7539
-2535       O
ATOM   8066  OD2 ASP B 228      32.034 -46.285  86.524  1.00186.90
O
ANISOU 8066  OD2 ASP B 228    31857  21902  17256   6161  -7460
-2393       O
ATOM   8067  N   SER B 229      30.146 -45.392  88.789  1.00215.18
N
ANISOU 8067  N   SER B 229    35944  25007  20809   4991  -6909
-1468       N
ATOM   8068  CA  SER B 229      29.417 -44.158  89.084  1.00198.44
C
ANISOU 8068  CA  SER B 229    33282  23243  18874   4446  -6389
-1138       C
ATOM   8069  C   SER B 229      29.421 -43.224  87.876  1.00192.98
C
ANISOU 8069  C   SER B 229    31661  23222  18443   4356  -6040
-1230       C
ATOM   8070  O   SER B 229      30.443 -42.610  87.552  1.00185.30
O
ANISOU 8070  O   SER B 229    30106  22722  17577   4769  -6046
-1511       O
ATOM   8071  CB  SER B 229      30.019 -43.467  90.304  1.00195.99
C
ANISOU 8071  CB  SER B 229    32896  23016  18556   4631  -6383
-1098       C
ATOM   8072  OG  SER B 229      29.934 -44.297  91.448  1.00205.39
O
ANISOU 8072  OG  SER B 229    34979  23565  19497   4668  -6686
-986       O
ATOM   8073  N   THR B 230      28.268 -43.127  87.216  1.00204.52
N
ANISOU 8073  N   THR B 230    33001  24720  19988   3801  -5739
-1002       N
ATOM   8074  CA  THR B 230      28.089 -42.274  86.046  1.00220.51
C
ANISOU 8074  CA  THR B 230    34222  27322  22241   3643  -5397
-1052       C
ATOM   8075  C   THR B 230      28.290 -40.793  86.367  1.00243.79
C
ANISOU 8075  C   THR B 230    36465  30808  25358   3544  -5043
-966       C
ATOM   8076  O   THR B 230      27.667 -40.258  87.289  1.00261.33
O
ANISOU 8076  O   THR B 230    38754  32965  27576   3182  -4833
-657       O
ATOM   8077  CB  THR B 230      26.696 -42.492  85.454  1.00224.67
C
```

ANISOU 8077 CB THR B 230   34845 27720 22801   3037 -5157
-796   C
ATOM   8078 OG1 THR B 230   26.514 -43.882 85.156 1.00225.89
O
ANISOU 8078 OG1 THR B 230   35687 27358 22784   3112 -5496
-873   O
ATOM   8079 CG2 THR B 230   26.527 -41.681 84.177 1.00222.73
C
ANISOU 8079 CG2 THR B 230   33820 28034 22773   2912 -4841
-873   C
ATOM   8080 N   ASN B 231   29.167 -40.134 85.602 1.00247.31
N
ANISOU 8080 N   ASN B 231   36245 31788 25934   3860 -4983
-1245   N
ATOM   8081 CA ASN B 231   29.405 -38.689 85.724 1.00248.62
C
ANISOU 8081 CA ASN B 231   35711 32502 26251   3760 -4647
-1191   C
ATOM   8082 C   ASN B 231   29.893 -38.295 87.121 1.00260.90
C
ANISOU 8082 C   ASN B 231   37406 33980 27744   3904 -4713
-1107   C
ATOM   8083 O   ASN B 231   29.463 -37.290 87.692 1.00279.79
O
ANISOU 8083 O   ASN B 231   39541 36558 30208   3585 -4413
-853   O
ATOM   8084 CB ASN B 231   28.151 -37.899 85.331 1.00244.90
C
ANISOU 8084 CB ASN B 231   34922 32221 25906   3136 -4207
-878   C
ATOM   8085 CG ASN B 231   28.418 -36.414 85.170 1.00239.51
C
ANISOU 8085 CG ASN B 231   33501 32132 25370   3053 -3879
-866   C
ATOM   8086 OD1 ASN B 231   27.748 -35.580 85.778 1.00245.24
O
ANISOU 8086 OD1 ASN B 231   34104 32940 26136   2687 -3608
-577   O
ATOM   8087 ND2 ASN B 231   29.402 -36.078 84.344 1.00232.52
N
ANISOU 8087 ND2 ASN B 231   32131 31667 24547   3387 -3906
-1188   N
ATOM   8088 N   GLN B 232   30.786 -39.109 87.684 1.00236.98
N
ANISOU 8088 N   GLN B 232   34812 30662 24569   4406 -5126
-1330   N
ATOM   8089 CA GLN B 232   31.328 -38.838 89.014 1.00202.21
C
ANISOU 8089 CA GLN B 232   30589 26149 20092   4604 -5237
-1285   C
ATOM   8090 C   GLN B 232   32.059 -37.497 89.076 1.00187.91
C
ANISOU 8090 C   GLN B 232   28032 24947 18420   4710 -5013
-1375   C

```
ATOM   8091  O   GLN B 232      31.917 -36.749  90.050  1.00167.66
O
ANISOU 8091  O   GLN B 232    25416  22414  15872   4544  -4856
-1150       O
ATOM   8092  CB  GLN B 232      32.262 -39.973  89.437  1.00189.58
C
ANISOU 8092  CB  GLN B 232    29559  24169  18305   5201  -5760
-1582       C
ATOM   8093  CG  GLN B 232      33.003 -39.709  90.739  1.00178.26
C
ANISOU 8093  CG  GLN B 232    28280  22655  16794   5504  -5919
-1609       C
ATOM   8094  CD  GLN B 232      33.868 -40.876  91.168  1.00173.69
C
ANISOU 8094  CD  GLN B 232    28326  21662  16006   6111  -6467
-1913       C
ATOM   8095  OE1 GLN B 232      35.096 -40.790  91.158  1.00176.29
O
ANISOU 8095  OE1 GLN B 232    28394  22266  16324   6678  -6689
-2293       O
ATOM   8096  NE2 GLN B 232      33.231 -41.977  91.550  1.00174.60
N
ANISOU 8096  NE2 GLN B 232    29286  21118  15938   5992  -6696
-1761       N
ATOM   8097  N   VAL B 233      32.837 -37.172  88.044  1.00207.65
N
ANISOU 8097  N   VAL B 233    29957  27935  21004   4968  -4993
-1702       N
ATOM   8098  CA  VAL B 233      33.716 -36.003  88.039  1.00196.15
C
ANISOU 8098  CA  VAL B 233    27819  27068  19642   5116  -4831
-1858       C
ATOM   8099  C   VAL B 233      33.050 -34.763  87.447  1.00201.03
C
ANISOU 8099  C   VAL B 233    27848  28119  20417   4615  -4359
-1642       C
ATOM   8100  O   VAL B 233      33.729 -33.775  87.147  1.00207.56
O
ANISOU 8100  O   VAL B 233    28070  29477  21316   4680  -4197
-1788       O
ATOM   8101  CB  VAL B 233      35.022 -36.316  87.286  1.00169.01
C
ANISOU 8101  CB  VAL B 233    24075  23963  16178   5679  -5078
-2371       C
ATOM   8102  CG1 VAL B 233      35.788 -37.425  87.993  1.00155.10
C
ANISOU 8102  CG1 VAL B 233    22881  21807  14244   6243  -5575
-2618       C
ATOM   8103  CG2 VAL B 233      34.724 -36.700  85.840  1.00154.07
C
ANISOU 8103  CG2 VAL B 233    22004  22202  14335   5596  -5023
-2503       C
ATOM   8104  N   GLY B 234      31.731 -34.810  87.262  1.00184.11
N
```

```
ANISOU 8104  N   GLY B 234    25884  25758  18309   4115  -4147
-1311       N
ATOM   8105  CA  GLY B 234      30.970 -33.686  86.737  1.00154.32
C
ANISOU 8105  CA  GLY B 234    21627  22342  14664   3641  -3725
-1096       C
ATOM   8106  C   GLY B 234      31.260 -32.305  87.303  1.00147.90
C
ANISOU 8106  C   GLY B 234    20372  21914  13908   3542  -3488
-991       C
ATOM   8107  O   GLY B 234      31.451 -32.126  88.509  1.00156.82
O
ANISOU 8107  O   GLY B 234    21706  22891  14988   3614  -3554
-869       O
ATOM   8108  N   HIS B 235      31.298 -31.323  86.395  1.00156.01
N
ANISOU 8108  N   HIS B 235    20810  23437  15030   3373  -3217
-1045       N
ATOM   8109  CA  HIS B 235      31.750 -29.966  86.705  1.00169.49
C
ANISOU 8109  CA  HIS B 235    22046  25578  16775   3310  -3008
-1012       C
ATOM   8110  C   HIS B 235      30.902 -29.286  87.777  1.00180.90
C
ANISOU 8110  C   HIS B 235    23632  26880  18223   2971  -2824
-614       C
ATOM   8111  O   HIS B 235      31.430 -28.546  88.616  1.00210.47
O
ANISOU 8111  O   HIS B 235    27245  30771  21953   3055  -2800
-574       O
ATOM   8112  CB  HIS B 235      31.751 -29.125  85.428  1.00179.28
C
ANISOU 8112  CB  HIS B 235    22722  27308  18087   3119  -2749
-1113       C
ATOM   8113  CG  HIS B 235      32.412 -27.791  85.578  1.00190.55
C
ANISOU 8113  CG  HIS B 235    23666  29211  19524   3090  -2571
-1151       C
ATOM   8114  ND1 HIS B 235      31.702 -26.611  85.593  1.00188.61
N
ANISOU 8114  ND1 HIS B 235    23197  29154  19311   2687  -2263
-885       N
ATOM   8115  CD2 HIS B 235      33.714 -27.447  85.713  1.00194.53
C
ANISOU 8115  CD2 HIS B 235    23876  30046  19992   3406  -2665
-1434       C
ATOM   8116  CE1 HIS B 235      32.537 -25.597  85.733  1.00186.52
C
ANISOU 8116  CE1 HIS B 235    22553  29290  19026   2742  -2178
-985       C
ATOM   8117  NE2 HIS B 235      33.765 -26.077  85.809  1.00192.68
N
ANISOU 8117  NE2 HIS B 235    23266  30176  19767   3162  -2407
-1318       N
```

```
ATOM   8118  N   PHE B 236      29.588 -29.511  87.755  1.00178.53
N
ANISOU 8118  N   PHE B 236    23582  26318  17932   2583  -2692
-331       N
ATOM   8119  CA  PHE B 236      28.709 -28.963  88.787  1.00169.31
C
ANISOU 8119  CA  PHE B 236    22570  25012  16749   2259  -2527
34       C
ATOM   8120  C   PHE B 236      29.113 -29.420  90.186  1.00159.62
C
ANISOU 8120  C   PHE B 236    21774  23440  15433   2481  -2750
98       C
ATOM   8121  O   PHE B 236      29.118 -28.623  91.130  1.00167.70
O
ANISOU 8121  O   PHE B 236    22738  24533  16447   2413  -2653
275       O
ATOM   8122  CB  PHE B 236      27.263 -29.360  88.483  1.00162.45
C
ANISOU 8122  CB  PHE B 236    21928  23911  15883   1834  -2389
267       C
ATOM   8123  CG  PHE B 236      26.274 -28.911  89.519  1.00159.70
C
ANISOU 8123  CG  PHE B 236    21753  23427  15500   1487  -2225
621       C
ATOM   8124  CD1 PHE B 236      25.411 -29.818  90.111  1.00148.69
C
ANISOU 8124  CD1 PHE B 236    20885  21587  14024   1284  -2291
807       C
ATOM   8125  CD2 PHE B 236      26.191 -27.578  89.885  1.00158.49
C
ANISOU 8125  CD2 PHE B 236    21244  23602  15375   1350  -2004
762       C
ATOM   8126  CE1 PHE B 236      24.494 -29.405  91.061  1.00141.06
C
ANISOU 8126  CE1 PHE B 236    20054  20531  13009    951  -2128
1115       C
ATOM   8127  CE2 PHE B 236      25.274 -27.158  90.832  1.00143.55
C
ANISOU 8127  CE2 PHE B 236    19495  21609  13440   1050  -1859
1071       C
ATOM   8128  CZ  PHE B 236      24.426 -28.073  91.421  1.00128.40
C
ANISOU 8128  CZ  PHE B 236    18069  19274  11444    849  -1914
1242       C
ATOM   8129  N   GLN B 237      29.412 -30.706  90.349  1.00155.82
N
ANISOU 8129  N   GLN B 237    21767  22562  14876   2744  -3058
-36       N
ATOM   8130  CA  GLN B 237      29.890 -31.210  91.635  1.00162.75
C
ANISOU 8130  CA  GLN B 237    23102  23085  15649   3005  -3312
-14       C
ATOM   8131  C   GLN B 237      31.234 -30.601  92.038  1.00145.60
C
```

```
ANISOU 8131  C   GLN B 237    20621  21212  13488   3416  -3417
-237       C
ATOM   8132  O   GLN B 237      31.437 -30.240  93.204  1.00180.53
O
ANISOU 8132  O   GLN B 237    25179  25543  17873   3477  -3447
-107       O
ATOM   8133  CB  GLN B 237      29.989 -32.735  91.574  1.00177.04
C
ANISOU 8133  CB  GLN B 237    25503  24412  17351   3232  -3654
-155       C
ATOM   8134  CG  GLN B 237      29.660 -33.457  92.873  1.00170.90
C
ANISOU 8134  CG  GLN B 237    25426  23076  16430   3213  -3838
42       C
ATOM   8135  CD  GLN B 237      30.544 -33.031  94.025  1.00176.86
C
ANISOU 8135  CD  GLN B 237    26206  23844  17147   3529  -3964
8       C
ATOM   8136  OE1 GLN B 237      31.708 -33.421  94.104  1.00178.07
O
ANISOU 8136  OE1 GLN B 237    26404  23997  17258   4045  -4262
-306       O
ATOM   8137  NE2 GLN B 237      29.995 -32.227  94.928  1.00180.34
N
ANISOU 8137  NE2 GLN B 237    26615  24309  17598   3235  -3746
318       N
ATOM   8138  N   LYS B 238      32.159 -30.475  91.085  1.00143.90
N
ANISOU 8138  N   LYS B 238    19984  21374  13318   3691  -3467
-582       N
ATOM   8139  CA  LYS B 238      33.458 -29.847  91.333  1.00153.13
C
ANISOU 8139  CA  LYS B 238    20780  22911  14492   4049  -3542
-835       C
ATOM   8140  C   LYS B 238      33.372 -28.390  91.791  1.00142.71
C
ANISOU 8140  C   LYS B 238    19064  21932  13226   3797  -3251
-630       C
ATOM   8141  O   LYS B 238      34.052 -27.996  92.746  1.00149.24
O
ANISOU 8141  O   LYS B 238    19881  22801  14024   4003  -3336
-649       O
ATOM   8142  CB  LYS B 238      34.316 -29.937  90.069  1.00170.86
C
ANISOU 8142  CB  LYS B 238    22596  25556  16765   4295  -3594
-1239       C
ATOM   8143  CG  LYS B 238      34.715 -31.347  89.673  1.00184.58
C
ANISOU 8143  CG  LYS B 238    24688  27012  18430   4678  -3947
-1526       C
ATOM   8144  CD  LYS B 238      35.674 -31.956  90.679  1.00191.70
C
ANISOU 8144  CD  LYS B 238    25921  27691  19226   5189  -4319
-1727       C
```

ATOM   8145 CE LYS B 238   36.207 -33.290 90.184 1.00198.89
C
ANISOU 8145 CE LYS B 238   27140 28384 20045   5634 -4699
-2076   C
ATOM   8146 NZ LYS B 238   37.197 -33.886 91.124 1.00193.49
N
ANISOU 8146 NZ LYS B 238   26783 27496 19239   6186 -5097
-2319   N
ATOM   8147 N   VAL B 239   32.557 -27.568 91.127 1.00131.07
N
ANISOU 8147 N   VAL B 239   17278 20701 11821   3368 -2925
-443   N
ATOM   8148 CA VAL B 239   32.408 -26.173 91.559 1.00123.92
C
ANISOU 8148 CA VAL B 239   16046 20093 10944   3125 -2667
-237   C
ATOM   8149 C   VAL B 239   31.792 -26.050 92.953 1.00124.81
C
ANISOU 8149 C   VAL B 239   16528 19875 11018   2994 -2656
101   C
ATOM   8150 O   VAL B 239   32.232 -25.219 93.757 1.00115.47
O
ANISOU 8150 O   VAL B 239   15207 18841   9825   3050 -2618
169   O
ATOM   8151 CB VAL B 239   31.626 -25.361 90.509 1.00122.30
C
ANISOU 8151 CB VAL B 239   15479 20191 10799   2717 -2350
-127   C
ATOM   8152 CG1 VAL B 239   32.393 -25.331 89.197 1.00154.70
C
ANISOU 8152 CG1 VAL B 239   19180 24672 14926   2855 -2349
-475   C
ATOM   8153 CG2 VAL B 239   30.253 -25.941 90.285 1.00123.88
C
ANISOU 8153 CG2 VAL B 239   15991 20068 11011   2395 -2268
108   C
ATOM   8154 N   LEU B 240   30.776 -26.855 93.270 1.00144.90
N
ANISOU 8154 N   LEU B 240   19545 21980 13529   2804 -2685
315   N
ATOM   8155 CA LEU B 240   30.256 -26.869 94.638 1.00143.76
C
ANISOU 8155 CA LEU B 240   19796 21504 13323   2696 -2699
609   C
ATOM   8156 C   LEU B 240   31.320 -27.258 95.661 1.00158.88
C
ANISOU 8156 C   LEU B 240   21966 23228 15174   3136 -2991
472   C
ATOM   8157 O   LEU B 240   31.402 -26.656 96.738 1.00159.84
O
ANISOU 8157 O   LEU B 240   22133 23328 15272   3136 -2960
640   O
ATOM   8158 CB LEU B 240   29.053 -27.808 94.739 1.00126.91
C

```
ANISOU 8158  CB  LEU B 240    18153  18931  11135   2408  -2699
816       C
ATOM   8159  CG  LEU B 240      27.669 -27.188  94.528  1.00128.83
C
ANISOU 8159  CG  LEU B 240    18264  19278  11405   1885  -2375
1112       C
ATOM   8160  CD1 LEU B 240      27.617 -26.352  93.263  1.00142.30
C
ANISOU 8160  CD1 LEU B 240    19402  21456  13208   1759  -2162
1010       C
ATOM   8161  CD2 LEU B 240      26.593 -28.264  94.511  1.00121.10
C
ANISOU 8161  CD2 LEU B 240    17761  17891  10362   1613  -2400
1250       C
ATOM   8162  N   THR B 241      32.138 -28.262  95.347  1.00141.09
N
ANISOU 8162  N   THR B 241    19891  20835  12884   3533  -3290
156       N
ATOM   8163  CA  THR B 241      33.277 -28.598  96.200  1.00144.01
C
ANISOU 8163  CA  THR B 241    20448  21081  13188   4017  -3595
-46       C
ATOM   8164  C   THR B 241      34.281 -27.454  96.314  1.00147.06
C
ANISOU 8164  C   THR B 241    20284  21966  13626   4193  -3521
-195       C
ATOM   8165  O   THR B 241      34.777 -27.164  97.409  1.00142.92
O
ANISOU 8165  O   THR B 241    19863  21373  13066   4380  -3620
-158       O
ATOM   8166  CB  THR B 241      33.968 -29.858  95.679  1.00142.85
C
ANISOU 8166  CB  THR B 241    20548  20748  12980   4435  -3939
-404       C
ATOM   8167  OG1 THR B 241      33.016 -30.924  95.591  1.00141.77
O
ANISOU 8167  OG1 THR B 241    20965  20124  12777   4242  -4013
-252       O
ATOM   8168  CG2 THR B 241      35.097 -30.272  96.616  1.00141.05
C
ANISOU 8168  CG2 THR B 241    20569  20360  12664   4967  -4289
-630       C
ATOM   8169  N   ALA B 242      34.598 -26.794  95.197  1.00160.57
N
ANISOU 8169  N   ALA B 242    21422  24176  15409   4121  -3350
-367       N
ATOM   8170  CA  ALA B 242      35.477 -25.627  95.243  1.00159.52
C
ANISOU 8170  CA  ALA B 242    20762  24543  15307   4200  -3244
-490       C
ATOM   8171  C   ALA B 242      34.905 -24.497  96.095  1.00124.12
C
ANISOU 8171  C   ALA B 242    16218  20103  10837   3889  -3013
-125       C
```

```
ATOM   8172  O   ALA B 242      35.644 -23.854  96.851  1.00112.08
O
ANISOU 8172  O   ALA B 242    14553  18735   9298   4056  -3052
-161       O
ATOM   8173  CB  ALA B 242      35.759 -25.138  93.824  1.00175.10
C
ANISOU 8173  CB  ALA B 242    22185  27016  17330   4093  -3074
-708       C
ATOM   8174  N   ILE B 243      33.603 -24.226  95.984  1.00118.70
N
ANISOU 8174  N   ILE B 243    15626  19301  10174   3446  -2779
214       N
ATOM   8175  CA  ILE B 243      32.980 -23.271  96.900  1.00109.05
C
ANISOU 8175  CA  ILE B 243    14418  18071   8944   3182  -2594
564       C
ATOM   8176  C   ILE B 243      33.114 -23.752  98.340  1.00134.73
C
ANISOU 8176  C   ILE B 243    18143  20914  12135   3385  -2796
681       C
ATOM   8177  O   ILE B 243      33.447 -22.978  99.244  1.00175.78
O
ANISOU 8177  O   ILE B 243    23270  26198  17321   3439  -2774
785       O
ATOM   8178  CB  ILE B 243      31.507 -23.044  96.512  1.00100.53
C
ANISOU 8178  CB  ILE B 243    13382  16932   7883   2701  -2337
867       C
ATOM   8179  CG1 ILE B 243      31.408 -22.485  95.091  1.00 98.85
C
ANISOU 8179  CG1 ILE B 243    12709  17126   7723   2515  -2144
745       C
ATOM   8180  CG2 ILE B 243      30.832 -22.107  97.504  1.00 98.11
C
ANISOU 8180  CG2 ILE B 243    13108  16617   7551   2457  -2168
1213       C
ATOM   8181  CD1 ILE B 243      30.055 -22.697  94.448  1.00 97.81
C
ANISOU 8181  CD1 ILE B 243    12668  16888   7610   2136  -1971
930       C
ATOM   8182  N   GLY B 244      32.853 -25.040  98.568  1.00132.92
N
ANISOU 8182  N   GLY B 244    18430  20221  11852   3495  -3003
666       N
ATOM   8183  CA  GLY B 244      33.132 -25.651  99.860  1.00135.64
C
ANISOU 8183  CA  GLY B 244    19283  20142  12112   3746  -3248
717       C
ATOM   8184  C   GLY B 244      34.580 -25.529 100.299  1.00110.88
C
ANISOU 8184  C   GLY B 244    16011  17153   8965   4242  -3481
418       C
ATOM   8185  O   GLY B 244      34.868 -25.256 101.465  1.00110.96
O
```

ANISOU 8185 O   GLY B 244   16193 17030   8937   4377 -3562
521   O
ATOM   8186 N   ASN B 245   35.511 -25.772 99.378 1.00112.31
N
ANISOU 8186 N   ASN B 245   15889 17614   9172   4529 -3602
29   N
ATOM   8187 CA ASN B 245   36.932 -25.620 99.676 1.00113.93
C
ANISOU 8187 CA ASN B 245   15879 18049   9362   4999 -3814
-312   C
ATOM   8188 C   ASN B 245   37.312 -24.195 100.077 1.00111.15
C
ANISOU 8188 C   ASN B 245   15070 18109   9051   4895 -3617
-227   C
ATOM   8189 O   ASN B 245   38.039 -23.995 101.056 1.00111.97
O
ANISOU 8189 O   ASN B 245   15235 18185   9123   5176 -3770
-286   O
ATOM   8190 CB ASN B 245   37.749 -26.067 98.461 1.00122.82
C
ANISOU 8190 CB ASN B 245   16687 19480 10499   5259 -3930
-751   C
ATOM   8191 CG ASN B 245   39.243 -25.985 98.696 1.00152.63
C
ANISOU 8191 CG ASN B 245   20205 23543 14243   5760 -4161
-1163   C
ATOM   8192 OD1 ASN B 245   39.955 -25.279 97.981 1.00142.15
O
ANISOU 8192 OD1 ASN B 245   18291 22770 12950   5773 -4047
-1403   O
ATOM   8193 ND2 ASN B 245   39.728 -26.709 99.698 1.00164.97
N
ANISOU 8193 ND2 ASN B 245   22215 24741 15726   6168 -4491
-1261   N
ATOM   8194 N   PHE B 246   36.833 -23.189 99.342 1.00107.96
N
ANISOU 8194 N   PHE B 246   14233 18078   8707   4499 -3293
-91   N
ATOM   8195 CA PHE B 246   37.211 -21.807 99.648 1.00105.45
C
ANISOU 8195 CA PHE B 246   13503 18157   8407   4385 -3116
-19   C
ATOM   8196 C   PHE B 246   36.761 -21.338 101.031 1.00104.07
C
ANISOU 8196 C   PHE B 246   13608 17724   8209   4300 -3087
330   C
ATOM   8197 O   PHE B 246   37.536 -20.706 101.757 1.00140.52
O
ANISOU 8197 O   PHE B 246   18082 22496 12811   4480 -3143
279   O
ATOM   8198 CB PHE B 246   36.650 -20.862 98.589 1.00102.44
C
ANISOU 8198 CB PHE B 246   12700 18158   8065   3956 -2790
85   C

```
ATOM   8199  CG  PHE B 246      37.050 -19.430  98.793  1.00109.74
C
ANISOU 8199  CG  PHE B 246    13224  19491   8980   3818  -2618
145       C
ATOM   8200  CD1 PHE B 246      36.092 -18.446  98.961  1.00 96.79
C
ANISOU 8200  CD1 PHE B 246    11549  17888   7339   3420  -2366
508       C
ATOM   8201  CD2 PHE B 246      38.385 -19.069  98.828  1.00142.67
C
ANISOU 8201  CD2 PHE B 246    17065  24016  13128   4088  -2720
-173       C
ATOM   8202  CE1 PHE B 246      36.459 -17.130  99.158  1.00110.06
C
ANISOU 8202  CE1 PHE B 246    12911  19918   8990   3296  -2231
568       C
ATOM   8203  CE2 PHE B 246      38.756 -17.754  99.023  1.00132.41
C
ANISOU 8203  CE2 PHE B 246    15427  23082  11801   3932  -2567
-113       C
ATOM   8204  CZ  PHE B 246      37.791 -16.784  99.187  1.00115.25
C
ANISOU 8204  CZ  PHE B 246    13262  20907   9621   3536  -2327
266       C
ATOM   8205  N   CYS B 247      35.518 -21.627 101.414  1.00103.18
N
ANISOU 8205  N   CYS B 247    13881  17239   8085   4020  -2995
678       N
ATOM   8206  CA  CYS B 247      35.061 -21.316 102.769  1.00138.91
C
ANISOU 8206  CA  CYS B 247    18718  21489  12571   3952  -2984
1000       C
ATOM   8207  C   CYS B 247      35.821 -22.089 103.848  1.00112.41
C
ANISOU 8207  C   CYS B 247    15770  17784   9158   4391  -3311
876       C
ATOM   8208  O   CYS B 247      36.224 -21.513 104.866  1.00108.80
O
ANISOU 8208  O   CYS B 247    15329  17326   8683   4516  -3352
959       O
ATOM   8209  CB  CYS B 247      33.553 -21.552 102.878  1.00151.21
C
ANISOU 8209  CB  CYS B 247    20591  22754  14110   3542  -2815
1358       C
ATOM   8210  SG  CYS B 247      32.968 -23.146 102.291  1.00136.83
S
ANISOU 8210  SG  CYS B 247    19215  20518  12257   3521  -2948
1284       S
ATOM   8211  N   ILE B 248      36.018 -23.394 103.652  1.00120.27
N
ANISOU 8211  N   ILE B 248    17124  18461  10112   4638  -3561
676       N
ATOM   8212  CA  ILE B 248      36.821 -24.187 104.585  1.00111.82
C
```

```
ANISOU 8212  CA  ILE B 248    16468  17053   8967   5109  -3916
503       C
ATOM   8213  C   ILE B 248      38.256 -23.672 104.696  1.00114.71
C
ANISOU 8213  C   ILE B 248    16432  17798   9353   5517  -4056
162       C
ATOM   8214  O   ILE B 248      38.814 -23.599 105.798  1.00113.46
O
ANISOU 8214  O   ILE B 248    16463  17493   9155   5794  -4229
151       O
ATOM   8215  CB  ILE B 248      36.786 -25.673 104.181  1.00117.46
C
ANISOU 8215  CB  ILE B 248    17634  17383   9613   5306  -4175
316       C
ATOM   8216  CG1 ILE B 248      35.407 -26.269 104.471  1.00117.05
C
ANISOU 8216  CG1 ILE B 248    18117  16855   9501   4921  -4084
679       C
ATOM   8217  CG2 ILE B 248      37.870 -26.458 104.904  1.00119.16
C
ANISOU 8217  CG2 ILE B 248    18192  17342   9740   5890  -4589
18       C
ATOM   8218  CD1 ILE B 248      35.116 -26.435 105.946  1.00116.11
C
ANISOU 8218  CD1 ILE B 248    18544  16287   9284   4934  -4181
935       C
ATOM   8219  N   CYS B 249      38.874 -23.302 103.573  1.00149.79
N
ANISOU 8219  N   CYS B 249    20318  22745  13850   5547  -3980
-128       N
ATOM   8220  CA  CYS B 249      40.149 -22.584 103.634  1.00133.07
C
ANISOU 8220  CA  CYS B 249    17727  21089  11746   5820  -4038
-426       C
ATOM   8221  C   CYS B 249      40.058 -21.316 104.479  1.00132.74
C
ANISOU 8221  C   CYS B 249    17517  21194  11722   5624  -3855
-149       C
ATOM   8222  O   CYS B 249      40.940 -21.042 105.301  1.00152.10
O
ANISOU 8222  O   CYS B 249    19928  23710  14152   5930  -4011
-279       O
ATOM   8223  CB  CYS B 249      40.634 -22.251 102.222  1.00146.62
C
ANISOU 8223  CB  CYS B 249    18851  23357  13501   5750  -3910
-725       C
ATOM   8224  SG  CYS B 249      41.121 -23.686 101.240  1.00159.63
S
ANISOU 8224  SG  CYS B 249    20601  24937  15116   6114  -4184
-1168       S
ATOM   8225  N   SER B 250      39.001 -20.528 104.287  1.00117.61
N
ANISOU 8225  N   SER B 250    15506  19341   9841   5131  -3537
221       N
```

```
ATOM   8226  CA  SER B 250      38.843 -19.282 105.036  1.00124.14
C
ANISOU 8226  CA  SER B 250    16184  20312  10673   4933  -3363
495       C
ATOM   8227  C   SER B 250      38.702 -19.509 106.540  1.00143.65
C
ANISOU 8227  C   SER B 250    19139  22340  13102   5098  -3516
709       C
ATOM   8228  O   SER B 250      39.267 -18.758 107.343  1.00130.81
O
ANISOU 8228  O   SER B 250    17396  20838  11469   5218  -3542
735       O
ATOM   8229  CB  SER B 250      37.633 -18.511 104.507  1.00 99.78
C
ANISOU 8229  CB  SER B 250    12962  17340   7610   4401  -3025
835       C
ATOM   8230  OG  SER B 250      37.766 -18.235 103.123  1.00137.99
O
ANISOU 8230  OG  SER B 250    17370  22583  12478   4239  -2881
645       O
ATOM   8231  N   ILE B 251      37.946 -20.532 106.942  1.00136.76
N
ANISOU 8231  N   ILE B 251    18827  20948  12190   5089  -3615
866       N
ATOM   8232  CA  ILE B 251      37.869 -20.911 108.354  1.00106.42
C
ANISOU 8232  CA  ILE B 251    15510  16641   8283   5268  -3791
1035       C
ATOM   8233  C   ILE B 251      39.218 -21.377 108.898  1.00109.52
C
ANISOU 8233  C   ILE B 251    15989  16982   8643   5840  -4141
675       C
ATOM   8234  O   ILE B 251      39.680 -20.897 109.939  1.00149.45
O
ANISOU 8234  O   ILE B 251    21058  21946  13781   5914  -4138
706       O
ATOM   8235  CB  ILE B 251      36.797 -21.998 108.548  1.00107.76
C
ANISOU 8235  CB  ILE B 251    16286  16272   8388   5103  -3828
1244       C
ATOM   8236  CG1 ILE B 251      35.413 -21.461 108.190  1.00114.25
C
ANISOU 8236  CG1 ILE B 251    17020  17159   9231   4539  -3480
1610       C
ATOM   8237  CG2 ILE B 251      36.813 -22.516 109.978  1.00109.61
C
ANISOU 8237  CG2 ILE B 251    17122  15996   8527   5310  -4043
1377       C
ATOM   8238  CD1 ILE B 251      34.449 -22.537 107.753  1.00116.64
C
ANISOU 8238  CD1 ILE B 251    17714  17115   9488   4320  -3473
1696       C
ATOM   8239  N   ALA B 252      39.871 -22.311 108.203  1.00112.54
N
```

```
ANISOU 8239  N   ALA B 252    16382  17368   9009   6154  -4365
289       N
ATOM   8240  CA  ALA B 252      41.148 -22.855 108.670  1.00134.71
C
ANISOU 8240  CA  ALA B 252    19267  20100  11816   6691  -4684
-113       C
ATOM   8241  C   ALA B 252      42.224 -21.792 108.887  1.00128.37
C
ANISOU 8241  C   ALA B 252    17907  19740  11128   6773  -4585
-316       C
ATOM   8242  O   ALA B 252      42.949 -21.833 109.888  1.00116.67
O
ANISOU 8242  O   ALA B 252    16561  18069   9699   7025  -4706
-441       O
ATOM   8243  CB  ALA B 252      41.649 -23.912 107.687  1.00119.15
C
ANISOU 8243  CB  ALA B 252    17312  18181   9779   7021  -4939
-517       C
ATOM   8244  N   ILE B 253      42.349 -20.837 107.966  1.00127.01
N
ANISOU 8244  N   ILE B 253    17126  20149  10984   6545  -4362
-358       N
ATOM   8245  CA  ILE B 253      43.170 -19.651 108.214  1.00122.26
C
ANISOU 8245  CA  ILE B 253    16031  19949  10473   6490  -4208
-456       C
ATOM   8246  C   ILE B 253      42.678 -18.847 109.416  1.00139.33
C
ANISOU 8246  C   ILE B 253    18370  21870  12699   6249  -4049
-59       C
ATOM   8247  O   ILE B 253      43.483 -18.334 110.204  1.00133.55
O
ANISOU 8247  O   ILE B 253    17525  21178  12039   6375  -4062
-164       O
ATOM   8248  CB  ILE B 253      43.232 -18.782 106.943  1.00122.05
C
ANISOU 8248  CB  ILE B 253    15386  20547  10438   6202  -3972
-530       C
ATOM   8249  CG1 ILE B 253      43.893 -19.557 105.800  1.00145.83
C
ANISOU 8249  CG1 ILE B 253    18170  23832  13407   6461  -4127
-986       C
ATOM   8250  CG2 ILE B 253      43.982 -17.483 107.208  1.00122.79
C
ANISOU 8250  CG2 ILE B 253    15026  21026  10602   6067  -3790
-585       C
ATOM   8251  CD1 ILE B 253      45.223 -20.188 106.174  1.00116.21
C
ANISOU 8251  CD1 ILE B 253    14409  20064   9682   6966  -4390
-1467       C
ATOM   8252  N   GLY B 254      41.362 -18.723 109.584  1.00135.86
N
ANISOU 8252  N   GLY B 254    18200  21187  12233   5896  -3892
384       N
```

```
ATOM   8253  CA  GLY B 254      40.832 -18.089 110.786  1.00115.89
C
ANISOU 8253  CA  GLY B 254    15881  18378   9771   5674  -3754
746       C
ATOM   8254  C   GLY B 254      41.201 -18.767 112.096  1.00131.77
C
ANISOU 8254  C   GLY B 254    18363  19892  11811   5964  -3965
707       C
ATOM   8255  O   GLY B 254      41.649 -18.112 113.039  1.00164.64
O
ANISOU 8255  O   GLY B 254    22471  24022  16065   5976  -3921
740       O
ATOM   8256  N   MET B 255      41.026 -20.083 112.178  1.00120.90
N
ANISOU 8256  N   MET B 255    17476  18108  10351   6191  -4198
628       N
ATOM   8257  CA  MET B 255      41.314 -20.810 113.428  1.00140.07
C
ANISOU 8257  CA  MET B 255    20422  20012  12786   6447  -4401
589       C
ATOM   8258  C   MET B 255      42.757 -21.288 113.576  1.00127.65
C
ANISOU 8258  C   MET B 255    18791  18483  11227   6970  -4674
92       C
ATOM   8259  O   MET B 255      43.486 -20.825 114.458  1.00119.84
O
ANISOU 8259  O   MET B 255    17728  17492  10314   7105  -4686
-1       O
ATOM   8260  CB  MET B 255      40.344 -21.986 113.590  1.00146.90
C
ANISOU 8260  CB  MET B 255    21937  20341  13537   6382  -4510
774       C
ATOM   8261  CG  MET B 255      40.070 -22.793 112.335  1.00144.03
C
ANISOU 8261  CG  MET B 255    21618  20046  13061   6434  -4618
645       C
ATOM   8262  SD  MET B 255      39.095 -24.268 112.694  1.00159.26
S
ANISOU 8262  SD  MET B 255    24411  21263  14838   6378  -4788
823       S
ATOM   8263  CE  MET B 255      39.541 -25.319 111.314  1.00148.59
C
ANISOU 8263  CE  MET B 255    23058  20014  13384   6702  -5057
429       C
ATOM   8264  N   VAL B 256      43.177 -22.206 112.706  1.00142.02
N
ANISOU 8264  N   VAL B 256    20635  20355  12971   7266  -4890
-248       N
ATOM   8265  CA  VAL B 256      44.456 -22.898 112.857  1.00163.55
C
ANISOU 8265  CA  VAL B 256    23402  23052  15688   7796  -5183
-754       C
ATOM   8266  C   VAL B 256      45.644 -21.957 112.690  1.00172.27
C
```

```
ANISOU 8266  C   VAL B 256    23859  24722  16876   7919  -5107
-1063       C
ATOM   8267  O   VAL B 256      46.716 -22.193 113.261  1.00168.66
O
ANISOU 8267  O   VAL B 256    23421  24230  16430   8299  -5285
-1416       O
ATOM   8268  CB  VAL B 256      44.520 -24.079 111.867  1.00163.16
C
ANISOU 8268  CB  VAL B 256    23515  22942  15536   8045  -5416
-1036       C
ATOM   8269  CG1 VAL B 256      45.844 -24.832 111.983  1.00144.80
C
ANISOU 8269  CG1 VAL B 256    21233  20596  13189   8615  -5727
-1600       C
ATOM   8270  CG2 VAL B 256      43.344 -25.020 112.094  1.00167.00
C
ANISOU 8270  CG2 VAL B 256    24699  22833  15919   7889  -5493
-720       C
ATOM   8271  N   ILE B 257      45.486 -20.877 111.933  1.00178.74
N
ANISOU 8271  N   ILE B 257    24116  26060  17737   7588  -4840
-949       N
ATOM   8272  CA  ILE B 257      46.409 -19.754 112.053  1.00167.38
C
ANISOU 8272  CA  ILE B 257    22139  25086  16373   7564  -4703
-1108       C
ATOM   8273  C   ILE B 257      46.029 -18.762 113.151  1.00134.74
C
ANISOU 8273  C   ILE B 257    18071  20807  12317   7282  -4513
-725       C
ATOM   8274  O   ILE B 257      46.439 -18.917 114.307  1.00131.90
O
ANISOU 8274  O   ILE B 257    17990  20135  11991   7483  -4626
-761       O
ATOM   8275  CB  ILE B 257      46.553 -19.032 110.702  1.00185.32
C
ANISOU 8275  CB  ILE B 257    23775  28003  18637   7333  -4507
-1218       C
ATOM   8276  CG1 ILE B 257      46.772 -20.047 109.579  1.00190.24
C
ANISOU 8276  CG1 ILE B 257    24348  28746  19187   7570  -4677
-1564       C
ATOM   8277  CG2 ILE B 257      47.718 -18.051 110.746  1.00194.40
C
ANISOU 8277  CG2 ILE B 257    24389  29644  19830   7357  -4408
-1488       C
ATOM   8278  CD1 ILE B 257      48.013 -20.899 109.758  1.00179.50
C
ANISOU 8278  CD1 ILE B 257    23031  27361  17808   8113  -4966
-2106       C
ATOM   8279  N   GLU B 258      45.233 -17.751 112.795  1.00131.20
N
ANISOU 8279  N   GLU B 258    17381  20574  11895   6817  -4227
-369       N
```

```
ATOM   8280  CA  GLU B 258      45.128 -16.527 113.591  1.00125.20
C
ANISOU 8280  CA  GLU B 258    16496  19854  11219   6539  -4019
-98       C
ATOM   8281  C   GLU B 258      44.739 -16.765 115.052  1.00124.50
C
ANISOU 8281  C   GLU B 258    16918  19205  11182   6565  -4073
149       C
ATOM   8282  O   GLU B 258      45.401 -16.254 115.963  1.00130.98
O
ANISOU 8282  O   GLU B 258    17697  20002  12069   6653  -4079
93       O
ATOM   8283  CB  GLU B 258      44.119 -15.582 112.934  1.00148.30
C
ANISOU 8283  CB  GLU B 258    19196  23005  14148   6032  -3723
260       C
ATOM   8284  CG  GLU B 258      44.594 -14.955 111.634  1.00144.04
C
ANISOU 8284  CG  GLU B 258    18082  23079  13568   5912  -3599
44       C
ATOM   8285  CD  GLU B 258      45.842 -14.115 111.814  1.00161.74
C
ANISOU 8285  CD  GLU B 258    19918  25693  15841   5993  -3570
-227       C
ATOM   8286  OE1 GLU B 258      46.867 -14.424 111.171  1.00164.05
O
ANISOU 8286  OE1 GLU B 258    19905  26334  16093   6240  -3674
-668       O
ATOM   8287  OE2 GLU B 258      45.795 -13.147 112.602  1.00153.39
O
ANISOU 8287  OE2 GLU B 258    18849  24584  14847   5798  -3439
-8       O
ATOM   8288  N   ILE B 259      43.675 -17.529 115.306  1.00138.31
N
ANISOU 8288  N   ILE B 259    19150  20507  12892   6466  -4104
416       N
ATOM   8289  CA  ILE B 259      43.350 -17.891 116.687  1.00143.15
C
ANISOU 8289  CA  ILE B 259    20268  20584  13537   6491  -4164
605       C
ATOM   8290  C   ILE B 259      44.422 -18.778 117.313  1.00136.39
C
ANISOU 8290  C   ILE B 259    19675  19491  12655   6996  -4461
221       C
ATOM   8291  O   ILE B 259      44.888 -18.515 118.427  1.00113.92
O
ANISOU 8291  O   ILE B 259    16938  16478   9868   7093  -4487
204       O
ATOM   8292  CB  ILE B 259      41.965 -18.557 116.765  1.00141.47
C
ANISOU 8292  CB  ILE B 259    20516  19972  13265   6236  -4118
955       C
ATOM   8293  CG1 ILE B 259      40.882 -17.605 116.260  1.00104.93
C
```

```
ANISOU 8293  CG1 ILE B 259    15628  15577   8664   5729  -3811
1323       C
ATOM   8294  CG2 ILE B 259      41.668 -18.993 118.192  1.00170.87
C
ANISOU 8294  CG2 ILE B 259    24760  23157  17006   6247  -4175
1116       C
ATOM   8295  CD1 ILE B 259      39.573 -18.284 115.994  1.00104.72
C
ANISOU 8295  CD1 ILE B 259    15954  15286   8549   5479  -3759
1598       C
ATOM   8296  N   ILE B 260      44.823 -19.841 116.617  1.00133.01
N
ANISOU 8296  N   ILE B 260    19363  19038  12135   7327  -4694
-107       N
ATOM   8297  CA  ILE B 260      45.796 -20.772 117.185  1.00127.73
C
ANISOU 8297  CA  ILE B 260    18999  18111  11424   7826  -5000
-504       C
ATOM   8298  C   ILE B 260      47.149 -20.105 117.412  1.00122.11
C
ANISOU 8298  C   ILE B 260    17855  17774  10768   8070  -5026
-858       C
ATOM   8299  O   ILE B 260      47.843 -20.407 118.386  1.00124.07
O
ANISOU 8299  O   ILE B 260    18346  17782  11013   8362  -5187
-1056       O
ATOM   8300  CB  ILE B 260      45.908 -22.032 116.305  1.00152.81
C
ANISOU 8300  CB  ILE B 260    22383  21192  14486   8119  -5249
-798       C
ATOM   8301  CG1 ILE B 260      44.672 -22.915 116.500  1.00124.45
C
ANISOU 8301  CG1 ILE B 260    19422  17051  10811   7935  -5286
-471       C
ATOM   8302  CG2 ILE B 260      47.173 -22.819 116.625  1.00154.09
C
ANISOU 8302  CG2 ILE B 260    22689  21260  14599   8680  -5564
-1330       C
ATOM   8303  CD1 ILE B 260      44.602 -23.578 117.859  1.00126.68
C
ANISOU 8303  CD1 ILE B 260    20353  16724  11057   8057  -5432
-417       C
ATOM   8304  N   VAL B 261      47.567 -19.217 116.510  1.00126.54
N
ANISOU 8304  N   VAL B 261    17784  18932  11362   7947  -4871
-966       N
ATOM   8305  CA  VAL B 261      48.702 -18.341 116.805  1.00126.11
C
ANISOU 8305  CA  VAL B 261    17301  19256  11360   8048  -4826
-1212       C
ATOM   8306  C   VAL B 261      48.446 -17.463 118.033  1.00145.66
C
ANISOU 8306  C   VAL B 261    19882  21537  13925   7815  -4675
-875       C
```

```
ATOM   8307  O   VAL B 261      49.315 -17.319 118.900  1.00191.44
O
ANISOU 8307  O   VAL B 261    25703  27291  19745   8042  -4764
-1075       O
ATOM   8308  CB  VAL B 261      49.047 -17.489 115.569  1.00118.66
C
ANISOU 8308  CB  VAL B 261    15683  18987  10417   7858  -4647
-1338       C
ATOM   8309  CG1 VAL B 261      50.026 -16.385 115.935  1.00118.19
C
ANISOU 8309  CG1 VAL B 261    15199  19306  10402   7831  -4542
-1487       C
ATOM   8310  CG2 VAL B 261      49.615 -18.368 114.461  1.00145.35
C
ANISOU 8310  CG2 VAL B 261    18900  22609  13716   8161  -4818
-1790       C
ATOM   8311  N   MET B 262      47.255 -16.870 118.129  1.00137.15
N
ANISOU 8311  N   MET B 262    18868  20346  12897   7362  -4445
-379       N
ATOM   8312  CA  MET B 262      46.879 -16.095 119.315  1.00125.35
C
ANISOU 8312  CA  MET B 262    17505  18628  11495   7117  -4299
-44       C
ATOM   8313  C   MET B 262      46.802 -16.937 120.589  1.00146.32
C
ANISOU 8313  C   MET B 262    20743  20706  14146   7320  -4465
-21       C
ATOM   8314  O   MET B 262      47.221 -16.486 121.661  1.00113.77
O
ANISOU 8314  O   MET B 262    16671  16470  10085   7354  -4454
-8       O
ATOM   8315  CB  MET B 262      45.552 -15.372 119.054  1.00149.02
C
ANISOU 8315  CB  MET B 262    20450  21634  14537   6592  -4027
441       C
ATOM   8316  CG  MET B 262      44.850 -14.766 120.278  1.00192.77
C
ANISOU 8316  CG  MET B 262    26205  26861  20177   6296  -3877
827       C
ATOM   8317  SD  MET B 262      43.903 -15.893 121.326  1.00193.09
S
ANISOU 8317  SD  MET B 262    26937  26232  20195   6291  -3964
1055       S
ATOM   8318  CE  MET B 262      42.363 -15.992 120.417  1.00137.64
C
ANISOU 8318  CE  MET B 262    19953  19216  13129   5868  -3786
1411       C
ATOM   8319  N   TYR B 263      46.272 -18.153 120.492  1.00169.45
N
ANISOU 8319  N   TYR B 263    24132  23261  16991   7442  -4619
-21       N
ATOM   8320  CA  TYR B 263      45.871 -18.942 121.660  1.00161.30
C
```

```
ANISOU 8320  CA  TYR B 263    23721  21635  15931   7507  -4729
94       C
ATOM   8321  C   TYR B 263      46.952 -19.162 122.723  1.00150.56
C
ANISOU 8321  C   TYR B 263    22538  20102  14567   7881  -4917
-212       C
ATOM   8322  O   TYR B 263      46.654 -18.949 123.910  1.00119.74
O
ANISOU 8322  O   TYR B 263    18889  15896  10710   7749  -4852
0       O
ATOM   8323  CB  TYR B 263      45.294 -20.253 121.100  1.00155.36
C
ANISOU 8323  CB  TYR B 263    23397  20578  15053   7606  -4894
62       C
ATOM   8324  CG  TYR B 263      44.844 -21.302 122.086  1.00137.32
C
ANISOU 8324  CG  TYR B 263    21827  17660  12688   7675  -5042
137       C
ATOM   8325  CD1 TYR B 263      45.738 -22.234 122.598  1.00126.75
C
ANISOU 8325  CD1 TYR B 263    20861  16039  11260   8142  -5349
-256       C
ATOM   8326  CD2 TYR B 263      43.508 -21.403 122.453  1.00120.83
C
ANISOU 8326  CD2 TYR B 263    20051  15267  10590   7265  -4879
576       C
ATOM   8327  CE1 TYR B 263      45.324 -23.207 123.486  1.00129.29
C
ANISOU 8327  CE1 TYR B 263    21876  15768  11481   8185  -5491
-200       C
ATOM   8328  CE2 TYR B 263      43.083 -22.378 123.333  1.00123.38
C
ANISOU 8328  CE2 TYR B 263    21036  15024  10819   7291  -5004
635       C
ATOM   8329  CZ  TYR B 263      43.996 -23.276 123.847  1.00148.34
C
ANISOU 8329  CZ  TYR B 263    24587  17888  13885   7747  -5312
251       C
ATOM   8330  OH  TYR B 263      43.581 -24.259 124.712  1.00150.75
O
ANISOU 8330  OH  TYR B 263    25591  17617  14071   7757  -5446
295       O
ATOM   8331  N   PRO B 264      48.201 -19.569 122.403  1.00131.08
N
ANISOU 8331  N   PRO B 264    19942  17822  12039   8339  -5146
-720       N
ATOM   8332  CA  PRO B 264      49.146 -19.903 123.479  1.00132.01
C
ANISOU 8332  CA  PRO B 264    20313  17721  12124   8702  -5346
-1020       C
ATOM   8333  C   PRO B 264      50.209 -18.833 123.695  1.00134.31
C
ANISOU 8333  C   PRO B 264    20098  18447  12489   8771  -5273
-1212       C
```

```
ATOM   8334  O   PRO B 264      50.897 -18.826 124.721  1.00141.07
O
ANISOU 8334  O   PRO B 264    21112  19152  13337   8979  -5377
-1379       O
ATOM   8335  CB  PRO B 264      49.780 -21.204 122.981  1.00131.39
C
ANISOU 8335  CB  PRO B 264    20491  17529  11901   9183  -5678
-1493       C
ATOM   8336  CG  PRO B 264      49.875 -20.970 121.529  1.00130.48
C
ANISOU 8336  CG  PRO B 264    19865  17915  11795   9144  -5611
-1605       C
ATOM   8337  CD  PRO B 264      48.624 -20.207 121.145  1.00153.06
C
ANISOU 8337  CD  PRO B 264    22556  20859  14741   8594  -5299
-1065       C
ATOM   8338  N   ILE B 265      50.344 -17.924 122.727  1.00125.98
N
ANISOU 8338  N   ILE B 265    18446  17933  11487   8579  -5095
-1193       N
ATOM   8339  CA  ILE B 265      51.233 -16.774 122.877  1.00139.47
C
ANISOU 8339  CA  ILE B 265    19662  20073  13257   8549  -4986
-1315       C
ATOM   8340  C   ILE B 265      50.755 -15.840 123.983  1.00135.98
C
ANISOU 8340  C   ILE B 265    19294  19446  12927   8219  -4791
-909       C
ATOM   8341  O   ILE B 265      51.553 -15.390 124.814  1.00169.44
O
ANISOU 8341  O   ILE B 265    23474  23717  17190   8344  -4823
-1050       O
ATOM   8342  CB  ILE B 265      51.379 -16.040 121.532  1.00123.70
C
ANISOU 8342  CB  ILE B 265    17051  18683  11268   8363  -4828
-1369       C
ATOM   8343  CG1 ILE B 265      51.956 -16.992 120.483  1.00125.05
C
ANISOU 8343  CG1 ILE B 265    17119  19061  11334   8713  -5026
-1826       C
ATOM   8344  CG2 ILE B 265      52.248 -14.804 121.687  1.00119.83
C
ANISOU 8344  CG2 ILE B 265    16077  18631  10821   8277  -4701
-1471       C
ATOM   8345  CD1 ILE B 265      53.362 -17.463 120.790  1.00142.15
C
ANISOU 8345  CD1 ILE B 265    19244  21339  13426   9213  -5270
-2403       C
ATOM   8346  N   GLN B 266      49.460 -15.539 124.028  1.00121.23
N
ANISOU 8346  N   GLN B 266    17549  17391  11123   7795  -4592
-421       N
ATOM   8347  CA  GLN B 266      48.942 -14.795 125.167  1.00113.56
C
```

```
ANISOU 8347  CA  GLN B 266    16697  16194  10258   7497  -4427
-59       C
ATOM   8348  C   GLN B 266      48.472 -15.718 126.285  1.00147.39
C
ANISOU 8348  C   GLN B 266    21591  19895  14517   7584  -4538
36       C
ATOM   8349  O   GLN B 266      48.250 -15.251 127.408  1.00160.52
O
ANISOU 8349  O   GLN B 266    23381  21351  16258   7421  -4444
245       O
ATOM   8350  CB  GLN B 266      47.797 -13.879 124.713  1.00109.16
C
ANISOU 8350  CB  GLN B 266    15925  15767   9783   6967  -4142
394       C
ATOM   8351  CG  GLN B 266      47.266 -12.912 125.769  1.00132.08
C
ANISOU 8351  CG  GLN B 266    18849  18522  12815   6617  -3950
751       C
ATOM   8352  CD  GLN B 266      48.362 -12.089 126.425  1.00142.12
C
ANISOU 8352  CD  GLN B 266    19899  19965  14135   6734  -3963
579       C
ATOM   8353  OE1 GLN B 266      48.718 -11.015 125.940  1.00146.87
O
ANISOU 8353  OE1 GLN B 266    20081  20955  14769   6574  -3843
580       O
ATOM   8354  NE2 GLN B 266      48.888 -12.579 127.544  1.00153.56
N
ANISOU 8354  NE2 GLN B 266    21651  21119  15576   7000  -4108
431       N
ATOM   8355  N   ARG B 267      48.359 -17.018 126.006  1.00146.31
N
ANISOU 8355  N   ARG B 267    21837  19492  14263   7839  -4744
-135       N
ATOM   8356  CA  ARG B 267      48.125 -18.046 127.022  1.00126.34
C
ANISOU 8356  CA  ARG B 267    19939  16405  11659   7992  -4906
-148       C
ATOM   8357  C   ARG B 267      46.853 -17.774 127.818  1.00118.32
C
ANISOU 8357  C   ARG B 267    19153  15087  10716   7548  -4692
345       C
ATOM   8358  O   ARG B 267      46.800 -17.980 129.032  1.00119.62
O
ANISOU 8358  O   ARG B 267    19665  14908  10878   7562  -4725
396       O
ATOM   8359  CB  ARG B 267      49.343 -18.177 127.937  1.00156.71
C
ANISOU 8359  CB  ARG B 267    23898  20178  15468   8380  -5100
-516       C
ATOM   8360  CG  ARG B 267      49.608 -19.580 128.442  1.00169.99
C
ANISOU 8360  CG  ARG B 267    26201  21398  16989   8759  -5408
-800       C
```

```
ATOM   8361  CD  ARG B 267      51.046 -19.679 128.907  1.00177.87
C
ANISOU 8361  CD  ARG B 267    27167  22494  17922   9217  -5636
-1292       C
ATOM   8362  NE  ARG B 267      51.942 -19.533 127.763  1.00185.66
N
ANISOU 8362  NE  ARG B 267    27671  23983  18889   9462  -5712
-1662       N
ATOM   8363  CZ  ARG B 267      53.240 -19.264 127.843  1.00182.03
C
ANISOU 8363  CZ  ARG B 267    26933  23833  18399   9786  -5835
-2090       C
ATOM   8364  NH1 ARG B 267      53.855 -19.284 129.016  1.00194.87
N
ANISOU 8364  NH1 ARG B 267    28793  25263  19987   9973  -5950
-2244       N
ATOM   8365  NH2 ARG B 267      53.952 -19.127 126.731  1.00180.77
N
ANISOU 8365  NH2 ARG B 267    26298  24167  18218   9955  -5873
-2415       N
ATOM   8366  N   ARG B 268      45.823 -17.297 127.113  1.00122.72
N
ANISOU 8366  N   ARG B 268    19496  15804  11330   7146  -4471
688       N
ATOM   8367  CA  ARG B 268      44.665 -16.689 127.759  1.00116.03
C
ANISOU 8367  CA  ARG B 268    18676  14830  10579   6674  -4220
1143       C
ATOM   8368  C   ARG B 268      43.968 -17.687 128.671  1.00127.56
C
ANISOU 8368  C   ARG B 268    20740  15767  11962   6647  -4288
1259       C
ATOM   8369  O   ARG B 268      43.548 -17.347 129.784  1.00136.90
O
ANISOU 8369  O   ARG B 268    22036  16768  13210   6444  -4174
1467       O
ATOM   8370  CB  ARG B 268      43.701 -16.151 126.704  1.00151.89
C
ANISOU 8370  CB  ARG B 268    22912  19642  15159   6289  -4012
1425       C
ATOM   8371  CG  ARG B 268      44.340 -15.158 125.750  1.00173.37
C
ANISOU 8371  CG  ARG B 268    25062  22881  17931   6280  -3939
1310       C
ATOM   8372  CD  ARG B 268      43.315 -14.542 124.817  1.00166.93
C
ANISOU 8372  CD  ARG B 268    23977  22304  17145   5861  -3721
1602       C
ATOM   8373  NE  ARG B 268      43.924 -13.599 123.883  1.00161.07
N
ANISOU 8373  NE  ARG B 268    22722  22050  16427   5834  -3651
1479       N
ATOM   8374  CZ  ARG B 268      44.194 -12.329 124.169  1.00174.99
C
```

```
ANISOU 8374  CZ  ARG B 268    24168  24029  18292   5642  -3511
1556       C
ATOM   8375  NH1 ARG B 268      44.760 -11.550 123.258  1.00163.64
N
ANISOU 8375  NH1 ARG B 268    22302  23030  16843   5616  -3455
1424       N
ATOM   8376  NH2 ARG B 268      43.910 -11.839 125.369  1.00190.80
N
ANISOU 8376  NH2 ARG B 268    26287  25808  20399   5469  -3432
1753       N
ATOM   8377  N   LYS B 269      43.847 -18.930 128.208  1.00142.26
N
ANISOU 8377  N   LYS B 269    22998  17382  13674   6846  -4479
1112       N
ATOM   8378  CA  LYS B 269      43.004 -19.963 128.791  1.00119.11
C
ANISOU 8378  CA  LYS B 269    20669  13959  10630   6754  -4534
1247       C
ATOM   8379  C   LYS B 269      43.226 -21.231 127.980  1.00122.66
C
ANISOU 8379  C   LYS B 269    21468  14226  10910   7057  -4797
980       C
ATOM   8380  O   LYS B 269      43.986 -21.215 127.006  1.00123.16
O
ANISOU 8380  O   LYS B 269    21244  14587  10966   7321  -4909
708       O
ATOM   8381  CB  LYS B 269      41.530 -19.554 128.764  1.00121.19
C
ANISOU 8381  CB  LYS B 269    20861  14238  10946   6217  -4251
1710       C
ATOM   8382  CG  LYS B 269      41.002 -19.278 127.362  1.00113.91
C
ANISOU 8382  CG  LYS B 269    19599  13648  10032   6025  -4138
1830       C
ATOM   8383  CD  LYS B 269      39.792 -18.357 127.378  1.00122.62
C
ANISOU 8383  CD  LYS B 269    20415  14938  11238   5496  -3825
2248       C
ATOM   8384  CE  LYS B 269      40.209 -16.897 127.438  1.00145.62
C
ANISOU 8384  CE  LYS B 269    22761  18244  14323   5380  -3670
2295       C
ATOM   8385  NZ  LYS B 269      39.059 -16.006 127.752  1.00156.12
N
ANISOU 8385  NZ  LYS B 269    23876  19685  15760   4883  -3403
2667       N
ATOM   8386  N   TYR B 270      42.586 -22.332 128.361  1.00125.29
N
ANISOU 8386  N   TYR B 270    22420  14084  11100   7018  -4902
1037       N
ATOM   8387  CA  TYR B 270      42.747 -23.559 127.591  1.00154.46
C
ANISOU 8387  CA  TYR B 270    26492  17569  14627   7284  -5165
790       C
```

```
ATOM   8388  C   TYR B 270      41.414 -24.085 127.074  1.00151.82
C
ANISOU 8388  C   TYR B 270    26412  17060  14213   6913  -5058
1101       C
ATOM   8389  O   TYR B 270      41.063 -23.852 125.913  1.00162.15
O
ANISOU 8389  O   TYR B 270    27394  18669  15548   6785  -4965
1188       O
ATOM   8390  CB  TYR B 270      43.471 -24.620 128.420  1.00166.49
C
ANISOU 8390  CB  TYR B 270    28630  18634  15992   7680  -5489
446       C
ATOM   8391  CG  TYR B 270      44.906 -24.253 128.727  1.00182.92
C
ANISOU 8391  CG  TYR B 270    30464  20922  18115   8113  -5645
53       C
ATOM   8392  CD1 TYR B 270      45.362 -24.165 130.037  1.00166.33
C
ANISOU 8392  CD1 TYR B 270    28583  18614  16003   8219  -5699
-30       C
ATOM   8393  CD2 TYR B 270      45.805 -23.984 127.702  1.00185.19
C
ANISOU 8393  CD2 TYR B 270    30283  21639  18444   8404  -5731
-248       C
ATOM   8394  CE1 TYR B 270      46.679 -23.840 130.316  1.00167.76
C
ANISOU 8394  CE1 TYR B 270    28543  18994  16203   8607  -5843
-402       C
ATOM   8395  CE2 TYR B 270      47.119 -23.647 127.970  1.00188.19
C
ANISOU 8395  CE2 TYR B 270    30417  22241  18846   8782  -5865
-627       C
ATOM   8396  CZ  TYR B 270      47.552 -23.579 129.279  1.00169.21
C
ANISOU 8396  CZ  TYR B 270    28256  19613  16423   8883  -5924
-702       C
ATOM   8397  OH  TYR B 270      48.857 -23.232 129.549  1.00155.12
O
ANISOU 8397  OH  TYR B 270    26226  18066  14647   9246  -6055
-1084       O
ATOM   8398  N   ARG B 271      40.663 -24.790 127.918  1.00147.03
N
ANISOU 8398  N   ARG B 271    26385  15985  13494   6722  -5066
1260       N
ATOM   8399  CA  ARG B 271      39.420 -25.405 127.472  1.00147.97
C
ANISOU 8399  CA  ARG B 271    26809  15909  13505   6363  -4980
1524       C
ATOM   8400  C   ARG B 271      38.294 -24.394 127.296  1.00148.43
C
ANISOU 8400  C   ARG B 271    26414  16294  13688   5840  -4602
1958       C
ATOM   8401  O   ARG B 271      37.290 -24.711 126.648  1.00152.04
O
```

```
ANISOU 8401  O   ARG B 271    26962  16735  14072   5531  -4498
2169       O
ATOM   8402  CB  ARG B 271      39.011 -26.517 128.440  1.00155.12
C
ANISOU 8402  CB  ARG B 271    28507  16209  14223   6310  -5118
1525       C
ATOM   8403  CG  ARG B 271      40.027 -27.644 128.501  1.00153.52
C
ANISOU 8403  CG  ARG B 271    28828  15649  13853   6820  -5525
1077       C
ATOM   8404  CD  ARG B 271      39.378 -28.965 128.866  1.00146.63
C
ANISOU 8404  CD  ARG B 271    28795  14182  12737   6694  -5682
1096       C
ATOM   8405  NE  ARG B 271      38.792 -28.942 130.203  1.00182.32
N
ANISOU 8405  NE  ARG B 271    33636  18424  17215   6409  -5556
1300       N
ATOM   8406  CZ  ARG B 271      39.478 -29.165 131.321  1.00172.59
C
ANISOU 8406  CZ  ARG B 271    32727  16933  15917   6658  -5724
1088       C
ATOM   8407  NH1 ARG B 271      38.859 -29.172 132.493  1.00146.69
N
ANISOU 8407  NH1 ARG B 271    29740  13408  12587   6369  -5601
1286       N
ATOM   8408  NH2 ARG B 271      40.780 -29.415 131.265  1.00166.77
N
ANISOU 8408  NH2 ARG B 271    32028  16192  15146   7200  -6023
658       N
ATOM   8409  N   ASP B 272      38.435 -23.188 127.851  1.00159.82
N
ANISOU 8409  N   ASP B 272    27379  18037  15309   5732  -4404
2081       N
ATOM   8410  CA  ASP B 272      37.496 -22.119 127.528  1.00178.23
C
ANISOU 8410  CA  ASP B 272    29204  20751  17765   5286  -4074
2436       C
ATOM   8411  C   ASP B 272      37.741 -21.567 126.128  1.00145.89
C
ANISOU 8411  C   ASP B 272    24595  17109  13727   5325  -4035
2383       C
ATOM   8412  O   ASP B 272      36.814 -21.046 125.496  1.00133.68
O
ANISOU 8412  O   ASP B 272    22760  15823  12211   4960  -3814
2644       O
ATOM   8413  CB  ASP B 272      37.596 -21.002 128.567  1.00172.15
C
ANISOU 8413  CB  ASP B 272    28106  20140  17164   5162  -3901
2563       C
ATOM   8414  CG  ASP B 272      37.053 -21.416 129.919  1.00198.39
C
ANISOU 8414  CG  ASP B 272    31867  23080  20432   5013  -3871
2689       C
```

```
ATOM   8415  OD1 ASP B 272      36.025 -22.125 129.959  1.00211.21
O
ANISOU 8415  OD1 ASP B 272    33856  24469  21926   4749  -3819
2862       O
ATOM   8416  OD2 ASP B 272      37.656 -21.033 130.944  1.00200.16
O
ANISOU 8416  OD2 ASP B 272    32072  23248  20733   5148  -3896
2609       O
ATOM   8417  N   GLY B 273      38.977 -21.664 125.635  1.00161.04
N
ANISOU 8417  N   GLY B 273    26387  19150  15652   5761  -4246
2030       N
ATOM   8418  CA  GLY B 273      39.380 -21.094 124.357  1.00167.35
C
ANISOU 8418  CA  GLY B 273    26666  20417  16503   5834  -4219
1928       C
ATOM   8419  C   GLY B 273      38.873 -21.847 123.143  1.00142.65
C
ANISOU 8419  C   GLY B 273    23662  17290  13249   5798  -4280
1922       C
ATOM   8420  O   GLY B 273      38.943 -21.314 122.030  1.00114.40
O
ANISOU 8420  O   GLY B 273    19633  14128   9705   5760  -4205
1901       O
ATOM   8421  N   ILE B 274      38.363 -23.066 123.338  1.00130.19
N
ANISOU 8421  N   ILE B 274    22699  15250  11516   5791  -4413
1937       N
ATOM   8422  CA  ILE B 274      37.782 -23.850 122.251  1.00120.52
C
ANISOU 8422  CA  ILE B 274    21661  13970  10159   5713  -4471
1955       C
ATOM   8423  C   ILE B 274      36.540 -23.170 121.688  1.00116.97
C
ANISOU 8423  C   ILE B 274    20903  13803   9736   5201  -4154
2329       C
ATOM   8424  O   ILE B 274      36.228 -23.311 120.498  1.00116.36
O
ANISOU 8424  O   ILE B 274    20687  13923   9600   5133  -4139
2333       O
ATOM   8425  CB  ILE B 274      37.469 -25.275 122.758  1.00124.79
C
ANISOU 8425  CB  ILE B 274    22996  13899  10519   5762  -4676
1906       C
ATOM   8426  CG1 ILE B 274      38.753 -25.971 123.214  1.00128.57
C
ANISOU 8426  CG1 ILE B 274    23785  14116  10951   6310  -5021
1481       C
ATOM   8427  CG2 ILE B 274      36.775 -26.104 121.688  1.00154.83
C
ANISOU 8427  CG2 ILE B 274    27042  17609  14178   5625  -4726
1954       C
ATOM   8428  CD1 ILE B 274      39.848 -25.995 122.166  1.00129.22
C
```

```
ANISOU 8428  CD1 ILE B 274    23526  14515  11055   6749  -5218
1102       C
ATOM   8429  N   ASP B 275      35.824 -22.416 122.524  1.00114.67
N
ANISOU 8429  N   ASP B 275    20486  13554   9531   4845  -3903
2626       N
ATOM   8430  CA  ASP B 275      34.631 -21.697 122.080  1.00124.09
C
ANISOU 8430  CA  ASP B 275    21361  15038  10751   4362  -3602
2956       C
ATOM   8431  C   ASP B 275      34.916 -20.786 120.889  1.00112.87
C
ANISOU 8431  C   ASP B 275    19336  14144   9407   4368  -3517
2906       C
ATOM   8432  O   ASP B 275      34.091 -20.675 119.974  1.00118.25
O
ANISOU 8432  O   ASP B 275    19870  15025  10033   4095  -3377
3053       O
ATOM   8433  CB  ASP B 275      34.055 -20.888 123.243  1.00126.74
C
ANISOU 8433  CB  ASP B 275    21564  15394  11197   4061  -3382
3208       C
ATOM   8434  CG  ASP B 275      33.334 -21.756 124.261  1.00145.13
C
ANISOU 8434  CG  ASP B 275    24460  17267  13416   3897  -3382
3337       C
ATOM   8435  OD1 ASP B 275      33.463 -22.997 124.186  1.00157.77
O
ANISOU 8435  OD1 ASP B 275    26613  18474  14860   4055  -3590
3198       O
ATOM   8436  OD2 ASP B 275      32.644 -21.199 125.141  1.00147.81
O
ANISOU 8436  OD2 ASP B 275    24699  17642  13820   3606  -3185
3561       O
ATOM   8437  N   ASN B 276      36.074 -20.120 120.884  1.00107.62
N
ANISOU 8437  N   ASN B 276    18321  13713   8855   4660  -3591
2686       N
ATOM   8438  CA  ASN B 276      36.485 -19.332 119.723  1.00130.80
C
ANISOU 8438  CA  ASN B 276    20715  17141  11841   4693  -3533
2582       C
ATOM   8439  C   ASN B 276      36.589 -20.177 118.455  1.00149.22
C
ANISOU 8439  C   ASN B 276    23127  19529  14040   4867  -3681
2402       C
ATOM   8440  O   ASN B 276      36.095 -19.780 117.393  1.00152.64
O
ANISOU 8440  O   ASN B 276    23259  20288  14450   4662  -3544
2483       O
ATOM   8441  CB  ASN B 276      37.823 -18.649 120.012  1.00151.68
C
ANISOU 8441  CB  ASN B 276    23040  19988  14602   5004  -3616
2329       C
```

ATOM   8442 CG ASN B 276   37.719 -17.602 121.099 1.00153.05
C
ANISOU 8442 CG ASN B 276   23047 20184 14923   4793 -3450
2510   C
ATOM   8443 OD1 ASN B 276   37.495 -16.424 120.822 1.00163.22
O
ANISOU 8443 OD1 ASN B 276   23909 21799 16307   4530 -3257
2634   O
ATOM   8444 ND2 ASN B 276   37.878 -18.027 122.347 1.00138.13
N
ANISOU 8444 ND2 ASN B 276   21511 17926 13045   4901 -3533
2511   N
ATOM   8445 N   LEU B 277   37.234 -21.343 118.541 1.00140.67
N
ANISOU 8445 N   LEU B 277   22453 18128 12868   5248 -3970
2138   N
ATOM   8446 CA LEU B 277   37.279 -22.258 117.401 1.00134.84
C
ANISOU 8446 CA LEU B 277   21848 17384 11999   5412 -4139
1958   C
ATOM   8447 C   LEU B 277   35.886 -22.744 117.016 1.00149.93
C
ANISOU 8447 C   LEU B 277   24045 19130 13790   5011 -4010
2254   C
ATOM   8448 O   LEU B 277   35.524 -22.770 115.834 1.00125.57
O
ANISOU 8448 O   LEU B 277   20774 16294 10643   4910 -3962
2258   O
ATOM   8449 CB LEU B 277   38.192 -23.444 117.716 1.00117.21
C
ANISOU 8449 CB LEU B 277   20065 14777   9694   5892 -4495
1607   C
ATOM   8450 CG LEU B 277   39.598 -23.107 118.215 1.00121.77
C
ANISOU 8450 CG LEU B 277   20422 15476 10368   6314 -4642
1272   C
ATOM   8451 CD1 LEU B 277   40.372 -24.372 118.553 1.00164.09
C
ANISOU 8451 CD1 LEU B 277   26293 20423 15631   6776 -5002
917   C
ATOM   8452 CD2 LEU B 277   40.347 -22.278 117.184 1.00116.43
C
ANISOU 8452 CD2 LEU B 277   19060 15412   9766   6436 -4593
1062   C
ATOM   8453 N   LEU B 278   35.095 -23.137 118.014 1.00134.46
N
ANISOU 8453 N   LEU B 278   22531 16769 11790   4764 -3945
2489   N
ATOM   8454 CA LEU B 278   33.739 -23.625 117.782 1.00139.70
C
ANISOU 8454 CA LEU B 278   23487 17264 12328   4340 -3804
2766   C
ATOM   8455 C   LEU B 278   32.834 -22.573 117.148 1.00127.66
C

ANISOU 8455 C   LEU B 278   21464 16192 10848   3927 -3482
3022   C
ATOM   8456 O   LEU B 278   32.011 -22.897 116.284 1.00138.00
O
ANISOU 8456 O   LEU B 278   22826 17561 12046   3687 -3400
3126   O
ATOM   8457 CB LEU B 278   33.139 -24.102 119.105 1.00131.59
C
ANISOU 8457 CB LEU B 278   22958 15779 11259   4138 -3768
2945   C
ATOM   8458 CG LEU B 278   31.856 -24.931 119.068 1.00158.93
C
ANISOU 8458 CG LEU B 278   26880 18950 14557   3730 -3677
3166   C
ATOM   8459 CD1 LEU B 278   32.023 -26.150 118.175 1.00177.97
C
ANISOU 8459 CD1 LEU B 278   29704 21110 16807   3901 -3929
2978   C
ATOM   8460 CD2 LEU B 278   31.457 -25.334 120.480 1.00144.71
C
ANISOU 8460 CD2 LEU B 278   25531 16732 12722   3574 -3652
3288   C
ATOM   8461 N   VAL B 279   32.974 -21.312 117.557 1.00112.02
N
ANISOU 8461 N   VAL B 279   19014 14523   9027   3834 -3306
3109   N
ATOM   8462 CA VAL B 279   32.294 -20.206 116.880 1.00105.20
C
ANISOU 8462 CA VAL B 279   17640 14112   8218   3496 -3037
3284   C
ATOM   8463 C   VAL B 279   32.653 -20.127 115.396 1.00101.98
C
ANISOU 8463 C   VAL B 279   16929 14054   7766   3628 -3075
3107   C
ATOM   8464 O   VAL B 279   31.772 -19.978 114.541 1.00102.90
O
ANISOU 8464 O   VAL B 279   16911 14372   7814   3340 -2911
3237   O
ATOM   8465 CB VAL B 279   32.621 -18.880 117.593 1.00110.12
C
ANISOU 8465 CB VAL B 279   17853 14955   9031   3430 -2908
3338   C
ATOM   8466 CG1 VAL B 279   32.466 -17.702 116.643 1.00113.76
C
ANISOU 8466 CG1 VAL B 279   17765 15895   9563   3239 -2729
3355   C
ATOM   8467 CG2 VAL B 279   31.754 -18.697 118.819 1.00138.04
C
ANISOU 8467 CG2 VAL B 279   21548 18293 12609   3119 -2764
3598   C
ATOM   8468 N   LEU B 280   33.941 -20.231 115.063 1.00108.16
N
ANISOU 8468 N   LEU B 280   17576 14940   8579   4064 -3286
2787   N

ATOM   8469 CA LEU B 280   34.347 -20.298 113.657 1.00122.15
C
ANISOU 8469 CA LEU B 280   19060 17055 10296   4222 -3346
2572   C
ATOM   8470 C   LEU B 280   33.747 -21.490 112.918 1.00135.54
C
ANISOU 8470 C   LEU B 280   21140 18541 11817   4198 -3450
2566   C
ATOM   8471 O   LEU B 280   33.205 -21.338 111.817 1.00125.16
O
ANISOU 8471 O   LEU B 280   19607 17510 10437   4020 -3328
2608   O
ATOM   8472 CB LEU B 280   35.870 -20.309 113.543 1.00115.00
C
ANISOU 8472 CB LEU B 280   17948 16295   9450   4705 -3572
2186   C
ATOM   8473 CG LEU B 280   36.474 -18.918 113.334 1.00110.22
C
ANISOU 8473 CG LEU B 280   16722 16180   8977   4665 -3415
2123   C
ATOM   8474 CD1 LEU B 280   36.265 -18.010 114.542 1.00 99.69
C
ANISOU 8474 CD1 LEU B 280   15357 14747   7773   4452 -3257
2347   C
ATOM   8475 CD2 LEU B 280   37.938 -19.026 112.961 1.00104.45
C
ANISOU 8475 CD2 LEU B 280   15738 15677   8271   5113 -3625
1698   C
ATOM   8476 N   LEU B 281   33.841 -22.685 113.498 1.00108.57
N
ANISOU 8476 N   LEU B 281   18315 14617   8320   4365 -3679
2503   N
ATOM   8477 CA LEU B 281   33.270 -23.864 112.853 1.00118.17
C
ANISOU 8477 CA LEU B 281   19969 15565   9364   4311 -3798
2496   C
ATOM   8478 C   LEU B 281   31.755 -23.769 112.698 1.00124.13
C
ANISOU 8478 C   LEU B 281   20827 16302 10035   3762 -3520
2852   C
ATOM   8479 O   LEU B 281   31.216 -24.100 111.636 1.00109.25
O
ANISOU 8479 O   LEU B 281   18864 14524   8122   3566 -3439
2830   O
ATOM   8480 CB LEU B 281   33.654 -25.122 113.630 1.00115.08
C
ANISOU 8480 CB LEU B 281   20239 14584   8903   4561 -4093
2362   C
ATOM   8481 CG LEU B 281   35.148 -25.449 113.611 1.00117.36
C
ANISOU 8481 CG LEU B 281   20471 14878   9244   5149 -4410
1937   C
ATOM   8482 CD1 LEU B 281   35.476 -26.513 114.641 1.00163.65
C

```
ANISOU 8482  CD1 LEU B 281    26997  20136  15048   5367  -4665
1830       C
ATOM   8483  CD2 LEU B 281      35.585 -25.890 112.220  1.00118.27
C
ANISOU 8483  CD2 LEU B 281    20402  15237   9299   5392  -4578
1651       C
ATOM   8484  N   ILE B 282      31.049 -23.320 113.738  1.00119.26
N
ANISOU 8484  N   ILE B 282    20272  15572   9467   3441  -3315
3125       N
ATOM   8485  CA  ILE B 282      29.591 -23.413 113.709  1.00123.86
C
ANISOU 8485  CA  ILE B 282    21003  16101   9957   2920  -3071
3424       C
ATOM   8486  C   ILE B 282      28.972 -22.427 112.721  1.00104.13
C
ANISOU 8486  C   ILE B 282    17961  14127   7479   2663  -2805
3529       C
ATOM   8487  O   ILE B 282      27.957 -22.740 112.086  1.00104.10
O
ANISOU 8487  O   ILE B 282    18025  14146   7383   2319  -2661
3641       O
ATOM   8488  CB  ILE B 282      29.022 -23.230 115.133  1.00143.56
C
ANISOU 8488  CB  ILE B 282    23671  18372  12502   2668  -2931
3646       C
ATOM   8489  CG1 ILE B 282      27.554 -23.657 115.197  1.00160.23
C
ANISOU 8489  CG1 ILE B 282    26035  20357  14487   2152  -2722
3894       C
ATOM   8490  CG2 ILE B 282      29.179 -21.797 115.618  1.00140.92
C
ANISOU 8490  CG2 ILE B 282    22782  18408  12353   2624  -2749
3730       C
ATOM   8491  CD1 ILE B 282      27.283 -24.997 114.573  1.00172.90
C
ANISOU 8491  CD1 ILE B 282    28169  21617  15907   2098  -2878
3826       C
ATOM   8492  N   GLY B 283      29.552 -21.239 112.559  1.00101.31
N
ANISOU 8492  N   GLY B 283    17038  14187   7269   2780  -2721
3465       N
ATOM   8493  CA  GLY B 283      28.934 -20.254 111.690  1.00 98.10
C
ANISOU 8493  CA  GLY B 283    16126  14254   6893   2513  -2459
3556       C
ATOM   8494  C   GLY B 283      29.647 -19.950 110.391  1.00 97.13
C
ANISOU 8494  C   GLY B 283    15546  14518   6841   2695  -2479
3299       C
ATOM   8495  O   GLY B 283      29.015 -19.516 109.423  1.00124.92
O
ANISOU 8495  O   GLY B 283    18718  18340  10405   2416  -2269
3319       O
```

```
ATOM   8496  N   GLY B 284      30.962 -20.160 110.353  1.00 98.46
N
ANISOU 8496  N   GLY B 284    15668  14687   7056   3127  -2716
3020       N
ATOM   8497  CA  GLY B 284      31.704 -19.884 109.134  1.00160.86
C
ANISOU 8497  CA  GLY B 284    23087  22972  15060   3257  -2718
2729       C
ATOM   8498  C   GLY B 284      31.369 -20.833 107.998  1.00133.78
C
ANISOU 8498  C   GLY B 284    19710  19475  11646   3153  -2725
2575       C
ATOM   8499  O   GLY B 284      31.278 -20.420 106.839  1.00 97.84
O
ANISOU 8499  O   GLY B 284    14733  15279   7165   3012  -2578
2474       O
ATOM   8500  N   ILE B 285      31.181 -22.108 108.312  1.00132.49
N
ANISOU 8500  N   ILE B 285    20092  18844  11405   3216  -2904
2557       N
ATOM   8501  CA  ILE B 285      30.891 -23.108 107.278  1.00104.18
C
ANISOU 8501  CA  ILE B 285    16617  15146   7820   3138  -2945
2406       C
ATOM   8502  C   ILE B 285      29.501 -22.866 106.695  1.00102.32
C
ANISOU 8502  C   ILE B 285    16260  15029   7588   2618  -2643
2634       C
ATOM   8503  O   ILE B 285      28.542 -22.618 107.450  1.00127.80
O
ANISOU 8503  O   ILE B 285    19671  18143  10745   2317  -2493
2934       O
ATOM   8504  CB  ILE B 285      31.026 -24.525 107.856  1.00111.34
C
ANISOU 8504  CB  ILE B 285    18213  15481   8610   3330  -3235
2344       C
ATOM   8505  CG1 ILE B 285      31.225 -25.551 106.741  1.00127.15
C
ANISOU 8505  CG1 ILE B 285    20287  17402  10620   3433  -3377
2075       C
ATOM   8506  CG2 ILE B 285      29.849 -24.902 108.757  1.00108.83
C
ANISOU 8506  CG2 ILE B 285    18409  14778   8163   2968  -3144
2686       C
ATOM   8507  CD1 ILE B 285      31.697 -26.889 107.255  1.00161.51
C
ANISOU 8507  CD1 ILE B 285    25299  21219  14846   3754  -3740
1928       C
ATOM   8508  N   PRO B 286      29.349 -22.844 105.301  1.00143.82
N
ANISOU 8508  N   PRO B 286    21156  20565  12924   2498  -2534
2483       N
ATOM   8509  CA  PRO B 286      28.031 -22.662 104.662  1.00121.63
C
```

```
ANISOU 8509  CA  PRO B 286    18224  17875  10116   2027  -2266
2661       C
ATOM   8510  C   PRO B 286      27.295 -23.992 104.550  1.00102.74
C
ANISOU 8510  C   PRO B 286    16323  15070   7642   1847  -2333
2694       C
ATOM   8511  O   PRO B 286      27.174 -24.594 103.480  1.00103.61
O
ANISOU 8511  O   PRO B 286    16386  15192   7787   1799  -2348
2538       O
ATOM   8512  CB  PRO B 286      28.406 -22.088 103.289  1.00 98.21
C
ANISOU 8512  CB  PRO B 286    14699  15357   7261   2050  -2169
2450       C
ATOM   8513  CG  PRO B 286      29.741 -21.472 103.483  1.00 97.78
C
ANISOU 8513  CG  PRO B 286    14378  15524   7251   2424  -2291
2265       C
ATOM   8514  CD  PRO B 286      30.431 -22.442 104.398  1.00141.29
C
ANISOU 8514  CD  PRO B 286    20367  20616  12700   2770  -2590
2175       C
ATOM   8515  N   ILE B 287      26.806 -24.497 105.683  1.00134.29
N
ANISOU 8515  N   ILE B 287    20835  18677  11514   1736  -2383
2898       N
ATOM   8516  CA  ILE B 287      25.977 -25.693 105.644  1.00116.09
C
ANISOU 8516  CA  ILE B 287    19029  15981   9098   1476  -2417
2966       C
ATOM   8517  C   ILE B 287      24.687 -25.405 104.885  1.00105.43
C
ANISOU 8517  C   ILE B 287    17418  14873   7767    990  -2119
3097       C
ATOM   8518  O   ILE B 287      24.109 -24.314 104.991  1.00106.29
O
ANISOU 8518  O   ILE B 287    17159  15321   7905    778  -1877
3257       O
ATOM   8519  CB  ILE B 287      25.707 -26.208 107.068  1.00156.46
C
ANISOU 8519  CB  ILE B 287    24758  20643  14047   1416  -2514
3169       C
ATOM   8520  CG1 ILE B 287      25.114 -27.620 107.022  1.00156.45
C
ANISOU 8520  CG1 ILE B 287    25372  20170  13901   1206  -2624
3185       C
ATOM   8521  CG2 ILE B 287      24.807 -25.252 107.842  1.00160.99
C
ANISOU 8521  CG2 ILE B 287    25185  21401  14582   1081  -2245
3472       C
ATOM   8522  CD1 ILE B 287      26.010 -28.633 106.342  1.00120.60
C
ANISOU 8522  CD1 ILE B 287    21048  15404   9371   1576  -2932
2880       C
```

```
ATOM   8523  N   ALA B 288      24.270 -26.369 104.061  1.00118.74
N
ANISOU 8523  N   ALA B 288    19284  16399   9434    841  -2153
3004       N
ATOM   8524  CA  ALA B 288      23.160 -26.300 103.108  1.00168.70
C
ANISOU 8524  CA  ALA B 288    25369  22941  15789    431  -1916
3052       C
ATOM   8525  C   ALA B 288      23.468 -25.468 101.867  1.00171.94
C
ANISOU 8525  C   ALA B 288    25141  23830  16361    525  -1804
2885       C
ATOM   8526  O   ALA B 288      22.578 -25.294 101.026  1.00152.36
O
ANISOU 8526  O   ALA B 288    22418  21560  13912    214  -1609
2910       O
ATOM   8527  CB  ALA B 288      21.861 -25.781 103.743  1.00160.60
C
ANISOU 8527  CB  ALA B 288    24327  22017  14677    -29  -1649
3339       C
ATOM   8528  N   MET B 289      24.683 -24.937 101.722  1.00157.37
N
ANISOU 8528  N   MET B 289    23018  22171  14604    926  -1918
2710       N
ATOM   8529  CA  MET B 289      25.044 -24.274 100.471  1.00100.30
C
ANISOU 8529  CA  MET B 289    15239  15367   7502   1000  -1829
2525       C
ATOM   8530  C   MET B 289      24.937 -25.199  99.261  1.00101.85
C
ANISOU 8530  C   MET B 289    15474  15491   7735    973  -1888
2334       C
ATOM   8531  O   MET B 289      24.439 -24.740  98.218  1.00131.14
O
ANISOU 8531  O   MET B 289    18811  19505  11512    778  -1707
2300       O
ATOM   8532  CB  MET B 289      26.434 -23.629 100.607  1.00 99.48
C
ANISOU 8532  CB  MET B 289    14869  15469   7461   1418  -1953
2351       C
ATOM   8533  CG  MET B 289      27.084 -23.099  99.324  1.00 98.06
C
ANISOU 8533  CG  MET B 289    14174  15699   7387   1544  -1910
2104       C
ATOM   8534  SD  MET B 289      27.835 -24.323  98.227  1.00100.91
S
ANISOU 8534  SD  MET B 289    14613  15942   7785   1818  -2135
1747       S
ATOM   8535  CE  MET B 289      29.170 -24.939  99.251  1.00103.75
C
ANISOU 8535  CE  MET B 289    15302  16025   8093   2332  -2482
1580       C
ATOM   8536  N   PRO B 290      25.362 -26.467  99.307  1.00105.17
N
```

```
ANISOU 8536  N   PRO B 290    16337  15520   8103   1166  -2143
2200       N
ATOM   8537  CA  PRO B 290      25.212 -27.302  98.108  1.00134.62
C
ANISOU 8537  CA  PRO B 290    20090  19196  11864   1134  -2196
2023       C
ATOM   8538  C   PRO B 290      23.774 -27.709  97.849  1.00162.27
C
ANISOU 8538  C   PRO B 290    23756  22583  15314    646  -2022
2204       C
ATOM   8539  O   PRO B 290      23.381 -27.855  96.685  1.00173.78
O
ANISOU 8539  O   PRO B 290    25006  24189  16835    511  -1937
2107       O
ATOM   8540  CB  PRO B 290      26.079 -28.524  98.436  1.00110.27
C
ANISOU 8540  CB  PRO B 290    17506  15688   8706   1506  -2553
1843       C
ATOM   8541  CG  PRO B 290      27.150 -27.972  99.282  1.00109.92
C
ANISOU 8541  CG  PRO B 290    17385  15713   8667   1874  -2675
1784       C
ATOM   8542  CD  PRO B 290      26.393 -27.031 100.198  1.00107.58
C
ANISOU 8542  CD  PRO B 290    17014  15514   8346   1567  -2439
2103       C
ATOM   8543  N   THR B 291      22.981 -27.903  98.904  1.00112.02
N
ANISOU 8543  N   THR B 291    17759  15973   8831    372  -1964
2455       N
ATOM   8544  CA  THR B 291      21.571 -28.247  98.747  1.00108.18
C
ANISOU 8544  CA  THR B 291    17407  15421   8275   -132  -1779
2622       C
ATOM   8545  C   THR B 291      20.800 -27.167  97.993  1.00110.84
C
ANISOU 8545  C   THR B 291    17163  16248   8704   -391  -1480
2669       C
ATOM   8546  O   THR B 291      20.040 -27.465  97.064  1.00166.69
O
ANISOU 8546  O   THR B 291    24139  23396  15799   -644  -1375
2629       O
ATOM   8547  CB  THR B 291      20.940 -28.483 100.121  1.00125.57
C
ANISOU 8547  CB  THR B 291    20058  17337  10315   -381  -1751
2876       C
ATOM   8548  OG1 THR B 291      21.514 -29.651 100.722  1.00127.79
O
ANISOU 8548  OG1 THR B 291    20977  17099  10478   -186  -2047
2828       O
ATOM   8549  CG2 THR B 291      19.431 -28.662  99.993  1.00134.84
C
ANISOU 8549  CG2 THR B 291    21278  18548  11408   -943  -1516
3040       C
```

```
ATOM   8550  N   VAL B 292      20.984 -25.904  98.383  1.00102.00
N
ANISOU 8550  N   VAL B 292    15673  15456   7627   -321  -1355
2746       N
ATOM   8551  CA  VAL B 292      20.282 -24.795  97.737  1.00 98.92
C
ANISOU 8551  CA  VAL B 292    14768  15521   7297   -533  -1098
2787       C
ATOM   8552  C   VAL B 292      20.621 -24.710  96.252  1.00113.82
C
ANISOU 8552  C   VAL B 292    16306  17636   9304   -424  -1094
2558       C
ATOM   8553  O   VAL B 292      19.735 -24.555  95.403  1.00110.31
O
ANISOU 8553  O   VAL B 292    15650  17380   8883   -688   -932
2551       O
ATOM   8554  CB  VAL B 292      20.601 -23.474  98.460  1.00 96.29
C
ANISOU 8554  CB  VAL B 292    14159  15458   6971   -418  -1017
2896       C
ATOM   8555  CG1 VAL B 292      20.040 -22.287  97.686  1.00 93.22
C
ANISOU 8555  CG1 VAL B 292    13257  15532   6630   -568   -799
2898       C
ATOM   8556  CG2 VAL B 292      20.053 -23.502  99.879  1.00129.69
C
ANISOU 8556  CG2 VAL B 292    18704  19498  11074   -584   -982
3139       C
ATOM   8557  N   LEU B 293      21.906 -24.801  95.913  1.00 98.23
N
ANISOU 8557  N   LEU B 293    14259  15665   7398    -32  -1271
2354       N
ATOM   8558  CA  LEU B 293      22.296 -24.849  94.506  1.00102.57
C
ANISOU 8558  CA  LEU B 293    14517  16409   8046     78  -1282
2117       C
ATOM   8559  C   LEU B 293      21.751 -26.084  93.791  1.00125.59
C
ANISOU 8559  C   LEU B 293    17695  19070  10953    -65  -1343
2043       C
ATOM   8560  O   LEU B 293      21.279 -25.989  92.652  1.00143.41
O
ANISOU 8560  O   LEU B 293    19702  21519  13270   -209  -1231
1959       O
ATOM   8561  CB  LEU B 293      23.818 -24.785  94.389  1.00101.61
C
ANISOU 8561  CB  LEU B 293    14282  16351   7976    527  -1471
1891       C
ATOM   8562  CG  LEU B 293      24.443 -23.512  94.966  1.00 95.82
C
ANISOU 8562  CG  LEU B 293    13249  15905   7252    663  -1410
1939       C
ATOM   8563  CD1 LEU B 293      25.965 -23.554  94.895  1.00 96.69
C
```

```
ANISOU 8563  CD1 LEU B 293    13255  16084   7397   1096  -1607
1687       C
ATOM   8564  CD2 LEU B 293      23.896 -22.278  94.258  1.00 92.67
C
ANISOU 8564  CD2 LEU B 293    12397  15928   6885    441  -1165
1988       C
ATOM   8565  N   SER B 294      21.817 -27.250  94.435  1.00129.87
N
ANISOU 8565  N   SER B 294    18768  19167  11410    -25  -1533
2069       N
ATOM   8566  CA  SER B 294      21.269 -28.473  93.847  1.00130.94
C
ANISOU 8566  CA  SER B 294    19225  19016  11510   -185  -1609
2016       C
ATOM   8567  C   SER B 294      19.771 -28.379  93.557  1.00142.67
C
ANISOU 8567  C   SER B 294    20644  20595  12967   -686  -1365
2173       C
ATOM   8568  O   SER B 294      19.319 -28.746  92.466  1.00156.10
O
ANISOU 8568  O   SER B 294    22245  22350  14715   -817  -1321
2072       O
ATOM   8569  CB  SER B 294      21.556 -29.660  94.770  1.00119.96
C
ANISOU 8569  CB  SER B 294    18489  17101   9991    -82  -1863
2047       C
ATOM   8570  OG  SER B 294      21.058 -30.870  94.225  1.00136.24
O
ANISOU 8570  OG  SER B 294    20915  18856  11995   -241  -1959
1998       O
ATOM   8571  N   VAL B 295      18.980 -27.895  94.517  1.00134.70
N
ANISOU 8571  N   VAL B 295    19679  19624  11877   -965  -1207
2405       N
ATOM   8572  CA  VAL B 295      17.552 -27.692  94.268  1.00125.81
C
ANISOU 8572  CA  VAL B 295    18423  18666  10715  -1430   -965
2524       C
ATOM   8573  C   VAL B 295      17.270 -26.557  93.284  1.00124.12
C
ANISOU 8573  C   VAL B 295    17612  18937  10610  -1456   -773
2452       C
ATOM   8574  O   VAL B 295      16.311 -26.636  92.508  1.00144.11
O
ANISOU 8574  O   VAL B 295    20003  21602  13152  -1736   -638
2428       O
ATOM   8575  CB  VAL B 295      16.797 -27.486  95.598  1.00142.22
C
ANISOU 8575  CB  VAL B 295    20700  20681  12656  -1712   -852
2771       C
ATOM   8576  CG1 VAL B 295      16.999 -28.687  96.518  1.00118.55
C
ANISOU 8576  CG1 VAL B 295    18360  17161   9521  -1730  -1045
2842       C
```

ATOM   8577 CG2 VAL B 295   17.215 -26.195 96.281 1.00146.02
C
ANISOU 8577 CG2 VAL B 295   20880 21436 13163 -1532   -779
2856   C
ATOM   8578 N   THR B 296   18.079 -25.496 93.289 1.00116.19
N
ANISOU 8578 N   THR B 296   16273 18199   9674 -1177   -765
2409   N
ATOM   8579 CA THR B 296   17.873 -24.393 92.348 1.00 95.09
C
ANISOU 8579 CA THR B 296   13090 15960   7077 -1196   -605
2336   C
ATOM   8580 C   THR B 296   17.977 -24.830 90.887 1.00108.01
C
ANISOU 8580 C   THR B 296   14587 17647   8804 -1155   -632
2124   C
ATOM   8581 O   THR B 296   17.155 -24.425 90.055 1.00 94.05
O
ANISOU 8581 O   THR B 296   12559 16116   7059 -1359   -479
2095   O
ATOM   8582 CB THR B 296   18.871 -23.270 92.637 1.00121.33
C
ANISOU 8582 CB THR B 296   16149 19515 10435   -904   -621
2318   C
ATOM   8583 OG1 THR B 296   18.677 -22.794 93.975 1.00113.45
O
ANISOU 8583 OG1 THR B 296   15263 18488   9353   -954   -584
2525   O
ATOM   8584 CG2 THR B 296   18.674 -22.115 91.665 1.00 90.03
C
ANISOU 8584 CG2 THR B 296   11719 15973   6517   -938   -470
2245   C
ATOM   8585 N   MET B 297   18.972 -25.652 90.549 1.00136.05
N
ANISOU 8585 N   MET B 297   18308 20985 12400   -877   -836
1961   N
ATOM   8586 CA MET B 297   18.995 -26.289 89.232 1.00104.02
C
ANISOU 8586 CA MET B 297   14196 16914   8412   -853   -882
1766   C
ATOM   8587 C   MET B 297   17.783 -27.183 88.987 1.00100.92
C
ANISOU 8587 C   MET B 297   14043 16326   7976 -1209   -832
1829   C
ATOM   8588 O   MET B 297   17.207 -27.165 87.892 1.00122.62
O
ANISOU 8588 O   MET B 297   16586 19226 10777 -1350   -738
1740   O
ATOM   8589 CB MET B 297   20.283 -27.089 89.048 1.00 99.70
C
ANISOU 8589 CB MET B 297   13827 16159   7895   -465 -1138
1570   C
ATOM   8590 CG MET B 297   21.510 -26.237 88.804 1.00 98.06
C

```
ANISOU 8590  CG  MET B 297    13269  16241   7747   -128  -1171
1418       C
ATOM   8591  SD  MET B 297      22.901 -27.238  88.258  1.00113.85
S
ANISOU 8591  SD  MET B 297    15394  18085   9780    319  -1462
1110       S
ATOM   8592  CE  MET B 297      24.003 -25.973  87.632  1.00158.26
C
ANISOU 8592  CE  MET B 297    20452  24210  15468    554  -1388
922       C
ATOM   8593  N   ALA B 298      17.385 -27.979  89.980  1.00106.48
N
ANISOU 8593  N   ALA B 298    15194  16691   8573  -1370   -896
1975       N
ATOM   8594  CA  ALA B 298      16.223 -28.847  89.805  1.00121.08
C
ANISOU 8594  CA  ALA B 298    17292  18357  10354  -1757   -842
2036       C
ATOM   8595  C   ALA B 298      14.947 -28.062  89.515  1.00123.07
C
ANISOU 8595  C   ALA B 298    17200  18959  10603  -2118   -574
2112       C
ATOM   8596  O   ALA B 298      14.176 -28.434  88.622  1.00139.58
O
ANISOU 8596  O   ALA B 298    19224  21095  12714  -2337   -505
2042       O
ATOM   8597  CB  ALA B 298      16.038 -29.724  91.044  1.00138.07
C
ANISOU 8597  CB  ALA B 298    20005  20099  12358  -1901   -945
2194       C
ATOM   8598  N   ILE B 299      14.700 -26.977  90.249  1.00114.56
N
ANISOU 8598  N   ILE B 299    15901  18134   9490  -2167   -433
2242       N
ATOM   8599  CA  ILE B 299      13.523 -26.158  89.963  1.00113.50
C
ANISOU 8599  CA  ILE B 299    15422  18365   9338  -2455   -202
2282       C
ATOM   8600  C   ILE B 299      13.694 -25.293  88.714  1.00101.43
C
ANISOU 8600  C   ILE B 299    13439  17180   7922  -2301   -140
2122       C
ATOM   8601  O   ILE B 299      12.702 -24.944  88.062  1.00136.94
O
ANISOU 8601  O   ILE B 299    17697  21919  12417  -2520      5
2082       O
ATOM   8602  CB  ILE B 299      13.163 -25.301  91.191  1.00142.99
C
ANISOU 8602  CB  ILE B 299    19098  22254  12978  -2548    -90
2467       C
ATOM   8603  CG1 ILE B 299      14.319 -24.373  91.571  1.00176.48
C
ANISOU 8603  CG1 ILE B 299    23186  26600  17268  -2171   -158
2476       C
```

```
ATOM   8604  CG2 ILE B 299      12.782 -26.193  92.365  1.00148.44
C
ANISOU 8604  CG2 ILE B 299    20252  22617  13532  -2784   -121
2627       C
ATOM   8605  CD1 ILE B 299      14.171 -22.951  91.073  1.00167.45
C
ANISOU 8605  CD1 ILE B 299    21571  25895  16158  -2105    -28
2439       C
ATOM   8606  N   GLY B 300      14.926 -24.931  88.355  1.00 94.51
N
ANISOU 8606  N   GLY B 300    12441  16341   7128  -1936   -246
2016       N
ATOM   8607  CA  GLY B 300      15.152 -24.267  87.078  1.00 92.49
C
ANISOU 8607  CA  GLY B 300    11817  16366   6960  -1813   -200
1848       C
ATOM   8608  C   GLY B 300      14.899 -25.145  85.865  1.00111.01
C
ANISOU 8608  C   GLY B 300    14195  18612   9370  -1875   -237
1690       C
ATOM   8609  O   GLY B 300      14.301 -24.702  84.881  1.00 92.49
O
ANISOU 8609  O   GLY B 300    11582  16502   7057  -1978   -128
1602       O
ATOM   8610  N   SER B 301      15.362 -26.395  85.912  1.00141.92
N
ANISOU 8610  N   SER B 301    18458  22170  13295  -1795   -408
1645       N
ATOM   8611  CA  SER B 301      15.068 -27.361  84.855  1.00107.12
C
ANISOU 8611  CA  SER B 301    14148  17620   8934  -1868   -463
1510       C
ATOM   8612  C   SER B 301      13.571 -27.601  84.671  1.00 98.54
C
ANISOU 8612  C   SER B 301    13069  16572   7801  -2289   -315
1575       C
ATOM   8613  O   SER B 301      13.086 -27.681  83.536  1.00106.03
O
ANISOU 8613  O   SER B 301    13848  17633   8806  -2374   -263
1452       O
ATOM   8614  CB  SER B 301      15.781 -28.681  85.151  1.00119.82
C
ANISOU 8614  CB  SER B 301    16206  18799  10522  -1709   -700
1471       C
ATOM   8615  OG  SER B 301      15.426 -29.677  84.208  1.00149.86
O
ANISOU 8615  OG  SER B 301    20155  22433  14353  -1799   -767
1358       O
ATOM   8616  N   HIS B 302      12.824 -27.725  85.769  1.00 99.62
N
ANISOU 8616  N   HIS B 302    13396  16630   7826  -2562   -246
1754       N
ATOM   8617  CA  HIS B 302      11.367 -27.808  85.680  1.00100.28
C
```

```
ANISOU 8617  CA  HIS B 302    13423  16834   7845  -2984    -81
1800       C
ATOM   8618  C   HIS B 302      10.727 -26.587  85.022  1.00137.13
C
ANISOU 8618  C   HIS B 302    17603  21955  12546  -3026     95
1735       C
ATOM   8619  O   HIS B 302       9.819 -26.732  84.196  1.00167.27
O
ANISOU 8619  O   HIS B 302    21283  25896  16375  -3234    179
1643       O
ATOM   8620  CB  HIS B 302      10.785 -28.007  87.081  1.00101.86
C
ANISOU 8620  CB  HIS B 302    13881  16924   7899  -3257    -24
1998       C
ATOM   8621  CG  HIS B 302       9.295 -28.142  87.104  1.00103.01
C
ANISOU 8621  CG  HIS B 302    13967  17220   7952  -3718    150
2031       C
ATOM   8622  ND1 HIS B 302       8.650 -29.345  86.915  1.00121.91
N
ANISOU 8622  ND1 HIS B 302    16665  19370  10283  -4036    128
2019       N
ATOM   8623  CD2 HIS B 302       8.321 -27.221  87.293  1.00113.94
C
ANISOU 8623  CD2 HIS B 302    15018  18994   9280  -3913    343
2056       C
ATOM   8624  CE1 HIS B 302       7.344 -29.159  86.989  1.00128.75
C
ANISOU 8624  CE1 HIS B 302    17365  20491  11064  -4428    315
2032       C
ATOM   8625  NE2 HIS B 302       7.118 -27.878  87.218  1.00123.93
N
ANISOU 8625  NE2 HIS B 302    16358  20273  10456  -4345    443
2045       N
ATOM   8626  N   ARG B 303      11.167 -25.378  85.375  1.00 95.03
N
ANISOU 8626  N   ARG B 303    12025  16867   7215  -2830    140
1774       N
ATOM   8627  CA  ARG B 303      10.705 -24.191  84.654  1.00 92.49
C
ANISOU 8627  CA  ARG B 303    11288  16945   6909  -2813    264
1691       C
ATOM   8628  C   ARG B 303      11.080 -24.193  83.172  1.00 91.61
C
ANISOU 8628  C   ARG B 303    11009  16893   6907  -2654    224
1493       C
ATOM   8629  O   ARG B 303      10.298 -23.724  82.337  1.00 90.67
O
ANISOU 8629  O   ARG B 303    10646  17014   6791  -2756    319
1394       O
ATOM   8630  CB  ARG B 303      11.184 -22.919  85.355  1.00 90.19
C
ANISOU 8630  CB  ARG B 303    10827  16865   6575  -2630    295
1779       C
```

```
ATOM   8631  CG  ARG B 303      10.421 -22.688  86.658  1.00123.34
C
ANISOU 8631  CG  ARG B 303    15095  21120  10649  -2842    383
1955       C
ATOM   8632  CD  ARG B 303      10.405 -21.229  87.096  1.00134.15
C
ANISOU 8632  CD  ARG B 303    16208  22809  11956  -2727    451
2013       C
ATOM   8633  NE  ARG B 303      11.687 -20.759  87.606  1.00153.12
N
ANISOU 8633  NE  ARG B 303    18657  25138  14385  -2428    356
2075       N
ATOM   8634  CZ  ARG B 303      12.051 -20.840  88.883  1.00134.83
C
ANISOU 8634  CZ  ARG B 303    16537  22676  12017  -2396    320
2236       C
ATOM   8635  NH1 ARG B 303      11.303 -21.515  89.746  1.00140.92
N
ANISOU 8635  NH1 ARG B 303    17524  23311  12707  -2653    363
2354       N
ATOM   8636  NH2 ARG B 303      13.227 -20.367  89.268  1.00142.29
N
ANISOU 8636  NH2 ARG B 303    17492  23583  12990  -2113    230
2268       N
ATOM   8637  N   LEU B 304      12.267 -24.692  82.816  1.00121.35
N
ANISOU 8637  N   LEU B 304    14894  20460  10752  -2392     79
1416       N
ATOM   8638  CA  LEU B 304      12.546 -24.942  81.400  1.00108.34
C
ANISOU 8638  CA  LEU B 304    13133  18832   9200  -2280     38
1221       C
ATOM   8639  C   LEU B 304      11.585 -25.952  80.781  1.00 93.71
C
ANISOU 8639  C   LEU B 304    11400  16844   7363  -2533     48
1165       C
ATOM   8640  O   LEU B 304      11.174 -25.792  79.627  1.00104.93
O
ANISOU 8640  O   LEU B 304    12625  18410   8833  -2562     96
1026       O
ATOM   8641  CB  LEU B 304      13.989 -25.395  81.198  1.00 92.30
C
ANISOU 8641  CB  LEU B 304    11210  16625   7233  -1951   -129
1129       C
ATOM   8642  CG  LEU B 304      15.041 -24.349  81.547  1.00 90.36
C
ANISOU 8642  CG  LEU B 304    10789  16563   6979  -1693   -137
1133       C
ATOM   8643  CD1 LEU B 304      16.396 -24.893  81.197  1.00131.84
C
ANISOU 8643  CD1 LEU B 304    16112  21687  12293  -1381   -301
988       C
ATOM   8644  CD2 LEU B 304      14.777 -23.054  80.802  1.00 87.75
C
```

```
ANISOU 8644  CD2 LEU B 304    10105  16598   6637  -1707      -5
1071       C
ATOM   8645  N   SER B 305      11.227 -27.004  81.518  1.00 96.29
N
ANISOU 8645  N   SER B 305    12068  16882   7637  -2726      -2
1267       N
ATOM   8646  CA  SER B 305      10.182 -27.904  81.035  1.00 98.30
C
ANISOU 8646  CA  SER B 305    12441  17030   7878  -3034      27
1229       C
ATOM   8647  C   SER B 305       8.865 -27.165  80.824  1.00 97.29
C
ANISOU 8647  C   SER B 305    12012  17248   7706  -3303     218
1216       C
ATOM   8648  O   SER B 305       8.157 -27.412  79.840  1.00103.78
O
ANISOU 8648  O   SER B 305    12723  18145   8564  -3437     259
1091       O
ATOM   8649  CB  SER B 305       9.999 -29.067  82.009  1.00110.63
C
ANISOU 8649  CB  SER B 305    14460  18227   9347  -3241     -51
1360       C
ATOM   8650  OG  SER B 305       9.066 -30.008  81.507  1.00140.02
O
ANISOU 8650  OG  SER B 305    18332  21825  13045  -3556     -37
1317       O
ATOM   8651  N   GLN B 306       8.518 -26.256  81.737  1.00 96.16
N
ANISOU 8651  N   GLN B 306    11732  17325   7478  -3367     325
1329       N
ATOM   8652  CA  GLN B 306       7.425 -25.322  81.482  1.00104.56
C
ANISOU 8652  CA  GLN B 306    12457  18779   8493  -3518     481
1278       C
ATOM   8653  C   GLN B 306       7.712 -24.435  80.274  1.00123.69
C
ANISOU 8653  C   GLN B 306    14576  21430  10992  -3276     483
1117       C
ATOM   8654  O   GLN B 306       6.821 -24.185  79.453  1.00105.40
O
ANISOU 8654  O   GLN B 306    12052  19321   8673  -3388     554
988       O
ATOM   8655  CB  GLN B 306       7.178 -24.464  82.723  1.00 94.05
C
ANISOU 8655  CB  GLN B 306    11053  17634   7048  -3562     564
1424       C
ATOM   8656  CG  GLN B 306       6.755 -25.249  83.951  1.00 96.59
C
ANISOU 8656  CG  GLN B 306    11667  17768   7265  -3847     588
1584       C
ATOM   8657  CD  GLN B 306       6.413 -24.349  85.121  1.00136.47
C
ANISOU 8657  CD  GLN B 306    16611  23045  12198  -3897     682
1714       C
```

```
ATOM   8658  OE1 GLN B 306       6.271 -23.136  84.965  1.00156.25
O
ANISOU 8658  OE1 GLN B 306    18806  25877  14686  -3754    737
1671       O
ATOM   8659  NE2 GLN B 306       6.289 -24.938  86.304  1.00111.29
N
ANISOU 8659  NE2 GLN B 306    13706  19669   8911  -4096    692
1872       N
ATOM   8660  N   GLN B 307       8.949 -23.941  80.156  1.00163.00
N
ANISOU 8660  N   GLN B 307    19530  26381  16022  -2953    404
1112       N
ATOM   8661  CA  GLN B 307       9.315 -23.096  79.021  1.00124.44
C
ANISOU 8661  CA  GLN B 307    14398  21699  11186  -2746    406
962       C
ATOM   8662  C   GLN B 307       9.130 -23.824  77.695  1.00109.41
C
ANISOU 8662  C   GLN B 307    12489  19709   9372  -2769    373
792       C
ATOM   8663  O   GLN B 307       8.667 -23.231  76.714  1.00104.16
O
ANISOU 8663  O   GLN B 307    11608  19255   8712  -2760    423
658       O
ATOM   8664  CB  GLN B 307      10.762 -22.620  79.170  1.00139.93
C
ANISOU 8664  CB  GLN B 307    16364  23627  13176  -2437    326
979       C
ATOM   8665  CG  GLN B 307      11.226 -21.623  78.113  1.00167.55
C
ANISOU 8665  CG  GLN B 307    19628  27349  16685  -2253    340
839       C
ATOM   8666  CD  GLN B 307      10.591 -20.257  78.273  1.00178.82
C
ANISOU 8666  CD  GLN B 307    20855  29087  18000  -2288    431
865       C
ATOM   8667  OE1 GLN B 307      11.040 -19.443  79.079  1.00185.03
O
ANISOU 8667  OE1 GLN B 307    21621  29964  18716  -2195    433
971       O
ATOM   8668  NE2 GLN B 307       9.546 -19.994  77.496  1.00189.68
N
ANISOU 8668  NE2 GLN B 307    22094  30627  19349  -2405    490
754       N
ATOM   8669  N   GLY B 308       9.480 -25.106  77.646  1.00105.40
N
ANISOU 8669  N   GLY B 308    12239  18883   8925  -2789    275
791       N
ATOM   8670  CA  GLY B 308       9.314 -25.877  76.431  1.00102.66
C
ANISOU 8670  CA  GLY B 308    11916  18430   8661  -2807    229
637       C
ATOM   8671  C   GLY B 308      10.561 -26.653  76.062  1.00112.58
C
```

ANISOU 8671 C   GLY B 308   13352 19425   9997 -2556   71
575   C
ATOM   8672 O   GLY B 308   10.642 -27.231 74.974 1.00129.49
O
ANISOU 8672 O   GLY B 308   15505 21484 12213 -2503   13
432   O
ATOM   8673 N   ALA B 309   11.545 -26.668 76.959 1.00122.86
N
ANISOU 8673 N   ALA B 309   14792 20610 11280 -2382   -9
667   N
ATOM   8674 CA ALA B 309   12.793 -27.389 76.748 1.00116.02
C
ANISOU 8674 CA ALA B 309   14093 19521 10470 -2104   -180
587   C
ATOM   8675 C   ALA B 309   13.003 -28.378 77.887 1.00137.13
C
ANISOU 8675 C   ALA B 309   17156 21854 13093 -2139   -302
716   C
ATOM   8676 O   ALA B 309   13.114 -27.979 79.052 1.00 96.60
O
ANISOU 8676 O   ALA B 309   12081 16729   7894 -2157   -276
865   O
ATOM   8677 CB ALA B 309   13.972 -26.419 76.648 1.00102.63
C
ANISOU 8677 CB ALA B 309   12181 18028   8785 -1806   -188
525   C
ATOM   8678 N   ILE B 310   13.055 -29.660 77.546 1.00132.86
N
ANISOU 8678 N   ILE B 310   16909 21003 12571 -2144   -444
657   N
ATOM   8679 CA ILE B 310   13.205 -30.743 78.510 1.00102.17
C
ANISOU 8679 CA ILE B 310   13476 16734   8612 -2189   -591
763   C
ATOM   8680 C   ILE B 310   14.686 -31.093 78.578 1.00118.61
C
ANISOU 8680 C   ILE B 310   15686 18662 10717 -1770   -800
663   C
ATOM   8681 O   ILE B 310   15.241 -31.666 77.634 1.00126.56
O
ANISOU 8681 O   ILE B 310   16716 19590 11782 -1562   -929
486   O
ATOM   8682 CB ILE B 310   12.355 -31.961 78.125 1.00104.82
C
ANISOU 8682 CB ILE B 310   14106 16799   8922 -2457   -644
754   C
ATOM   8683 CG1 ILE B 310   10.877 -31.572 78.040 1.00114.92
C
ANISOU 8683 CG1 ILE B 310   15204 18289 10172 -2873   -429
818   C
ATOM   8684 CG2 ILE B 310   12.563 -33.095 79.113 1.00109.01
C
ANISOU 8684 CG2 ILE B 310   15176 16898   9346 -2506   -820
861   C

```
ATOM   8685  CD1 ILE B 310       9.969 -32.703  77.602  1.00128.12           
C
ANISOU 8685  CD1 ILE B 310    17126  19740  11812  -3182   -460  -
797       C
ATOM   8686  N   THR B 311      15.334 -30.755  79.691  1.00117.29           
N
ANISOU 8686  N   THR B 311    15596  18468  10500  -1633   -841  -
760       N
ATOM   8687  CA  THR B 311      16.776 -30.933  79.833  1.00110.34           
C
ANISOU 8687  CA  THR B 311    14781  17510   9634  -1213  -1032  -
643       C
ATOM   8688  C   THR B 311      17.051 -32.336  80.362  1.00109.57           
C
ANISOU 8688  C   THR B 311    15223  16945   9462  -1139  -1280  -
651       C
ATOM   8689  O   THR B 311      16.831 -32.618  81.545  1.00110.45           
O
ANISOU 8689  O   THR B 311    15655  16835   9477  -1263  -1316  -
821       O
ATOM   8690  CB  THR B 311      17.362 -29.874  80.764  1.00117.62           
C
ANISOU 8690  CB  THR B 311    15526  18631  10533  -1089   -969  -
731       C
ATOM   8691  OG1 THR B 311      16.741 -29.972  82.051  1.00141.42           
O
ANISOU 8691  OG1 THR B 311    18798  21479  13454  -1317   -933  -
957       O
ATOM   8692  CG2 THR B 311      17.123 -28.482  80.203  1.00120.11           
C
ANISOU 8692  CG2 THR B 311    15358  19384  10894  -1153   -751  -
716       C
ATOM   8693  N   LYS B 312      17.527 -33.216  79.477  1.00131.32           
N
ANISOU 8693  N   LYS B 312    18106  19543  12248   -933  -1462  -
461       N
ATOM   8694  CA  LYS B 312      17.925 -34.559  79.889  1.00120.50           
C
ANISOU 8694  CA  LYS B 312    17282  17715  10789   -793  -1746  -
431       C
ATOM   8695  C   LYS B 312      19.057 -34.523  80.910  1.00117.09           
C
ANISOU 8695  C   LYS B 312    17003  17187  10300   -452  -1916  -
418       C
ATOM   8696  O   LYS B 312      19.025 -35.250  81.909  1.00119.17           
O
ANISOU 8696  O   LYS B 312    17756  17079  10444   -486  -2068  -
529       O
ATOM   8697  CB  LYS B 312      18.333 -35.376  78.662  1.00132.85           
C
ANISOU 8697  CB  LYS B 312    18893  19185  12397   -570  -1920  -
198       C
ATOM   8698  CG  LYS B 312      18.953 -36.730  78.973  1.00125.83           
C
```

```
ANISOU 8698  CG  LYS B 312    18567  17838  11405   -319  -2267
113       C
ATOM   8699  CD  LYS B 312      17.979 -37.636  79.707  1.00154.01
C
ANISOU 8699  CD  LYS B 312    22696  20980  14840   -693  -2318
317       C
ATOM   8700  CE  LYS B 312      18.510 -39.057  79.789  1.00173.14
C
ANISOU 8700  CE  LYS B 312    25731  22914  17141   -454  -2688
212       C
ATOM   8701  NZ  LYS B 312      19.744 -39.137  80.615  1.00184.93
N
ANISOU 8701  NZ  LYS B 312    27400  24296  18568     -9  -2921
128       N
ATOM   8702  N   ARG B 313      20.064 -33.683  80.677  1.00123.64
N
ANISOU 8702  N   ARG B 313    17433  18345  11198   -133  -1896
276       N
ATOM   8703  CA  ARG B 313      21.048 -33.359  81.704  1.00160.24
C
ANISOU 8703  CA  ARG B 313    22108  22985  15791    149  -1999
276       C
ATOM   8704  C   ARG B 313      20.528 -32.264  82.626  1.00150.94
C
ANISOU 8704  C   ARG B 313    20754  21993  14604   -104  -1766
513       C
ATOM   8705  O   ARG B 313      20.141 -31.184  82.168  1.00149.73
O
ANISOU 8705  O   ARG B 313    20162  22210  14517   -261  -1525
546       O
ATOM   8706  CB  ARG B 313      22.370 -32.934  81.071  1.00149.57
C
ANISOU 8706  CB  ARG B 313    20392  21937  14501    576  -2075
3       C
ATOM   8707  CG  ARG B 313      23.212 -34.084  80.546  1.00135.67
C
ANISOU 8707  CG  ARG B 313    18871  19966  12712    958  -2386
-260       C
ATOM   8708  CD  ARG B 313      24.447 -33.550  79.846  1.00153.60
C
ANISOU 8708  CD  ARG B 313    20695  22628  15037   1332  -2414
-548       C
ATOM   8709  NE  ARG B 313      25.229 -32.705  80.743  1.00162.03
N
ANISOU 8709  NE  ARG B 313    21576  23901  16088   1491  -2386
-536       N
ATOM   8710  CZ  ARG B 313      25.674 -31.491  80.440  1.00168.28
C
ANISOU 8710  CZ  ARG B 313    21855  25152  16931   1497  -2192
-594       C
ATOM   8711  NH1 ARG B 313      25.614 -31.052  79.191  1.00178.64
N
ANISOU 8711  NH1 ARG B 313    22794  26772  18308   1425  -2045
-721       N
```

ATOM   8712 NH2 ARG B 313   26.352 -30.797 81.344 1.00166.66
N
ANISOU 8712 NH2 ARG B 313   21532 25092 16698   1626 -2184
-569   N
ATOM   8713 N   MET B 314   20.519 -32.554 83.929 1.00119.35
N
ANISOU 8713 N   MET B 314   17123 17722 10504   -132 -1852
673   N
ATOM   8714 CA MET B 314   20.170 -31.548 84.926 1.00145.08
C
ANISOU 8714 CA MET B 314   20240 21143 13741   -312 -1665
888   C
ATOM   8715 C   MET B 314   21.149 -30.379 84.922 1.00125.13
C
ANISOU 8715 C   MET B 314   17267 19000 11279   -39 -1606
795   C
ATOM   8716 O   MET B 314   20.752 -29.237 85.184 1.00116.14
O
ANISOU 8716 O   MET B 314   15824 18147 10156   -213 -1387
928   O
ATOM   8717 CB MET B 314   20.112 -32.187 86.313 1.00183.23
C
ANISOU 8717 CB MET B 314   25598 25580 18443   -353 -1801
1053   C
ATOM   8718 CG MET B 314   19.028 -33.243 86.462 1.00172.50
C
ANISOU 8718 CG MET B 314   24707 23852 16983   -721 -1824
1182   C
ATOM   8719 SD MET B 314   17.361 -32.576 86.283 1.00168.77
S
ANISOU 8719 SD MET B 314   23965 23638 16522 -1305 -1467
1379   S
ATOM   8720 CE MET B 314   16.381 -34.054 86.534 1.00181.27
C
ANISOU 8720 CE MET B 314   26185 24736 17954 -1691 -1560
1482   C
ATOM   8721 N   THR B 315   22.422 -30.637 84.623 1.00124.06
N
ANISOU 8721 N   THR B 315   17085 18889 11162   382 -1803
557   N
ATOM   8722 CA THR B 315   23.490 -29.659 84.793 1.00160.11
C
ANISOU 8722 CA THR B 315   21289 23785 15758   650 -1782
454   C
ATOM   8723 C   THR B 315   23.725 -28.803 83.552 1.00132.33
C
ANISOU 8723 C   THR B 315   17247 20710 12323   657 -1622
295   C
ATOM   8724 O   THR B 315   24.691 -28.033 83.518 1.00133.92
O
ANISOU 8724 O   THR B 315   17134 21213 12537   867 -1607
170   O
ATOM   8725 CB THR B 315   24.791 -30.367 85.181 1.00169.21
C

```
ANISOU 8725  CB  THR B 315    22650  24773  16869   1112  -2086
247       C
ATOM   8726  OG1 THR B 315      25.092 -31.382  84.215  1.00180.92
O
ANISOU 8726  OG1 THR B 315    24249  26130  18360   1301  -2268
13       O
ATOM   8727  CG2 THR B 315      24.654 -31.014  86.552  1.00147.23
C
ANISOU 8727  CG2 THR B 315    20394  21563  13982   1120  -2243
416       C
ATOM   8728  N   ALA B 316      22.869 -28.924  82.536  1.00104.79
N
ANISOU 8728  N   ALA B 316    13672  17265   8880    421  -1503
293       N
ATOM   8729  CA  ALA B 316      23.062 -28.211  81.278  1.00107.45
C
ANISOU 8729  CA  ALA B 316    13568  17980   9279    423  -1367
132       C
ATOM   8730  C   ALA B 316      22.833 -26.712  81.430  1.00 97.60
C
ANISOU 8730  C   ALA B 316    11968  17087   8028    246  -1128
254       C
ATOM   8731  O   ALA B 316      23.212 -25.942  80.542  1.00101.85
O
ANISOU 8731  O   ALA B 316    12149  17961   8588    272  -1024
118       O
ATOM   8732  CB  ALA B 316      22.134 -28.779  80.204  1.00118.23
C
ANISOU 8732  CB  ALA B 316    14975  19260  10686    217  -1317
105       C
ATOM   8733  N   ILE B 317      22.205 -26.298  82.532  1.00 96.21
N
ANISOU 8733  N   ILE B 317    11911  16835   7809     61  -1048
505       N
ATOM   8734  CA  ILE B 317      21.901 -24.892  82.794  1.00 93.34
C
ANISOU 8734  CA  ILE B 317    11268  16775   7422   -101   -845
639       C
ATOM   8735  C   ILE B 317      23.148 -24.016  82.719  1.00121.75
C
ANISOU 8735  C   ILE B 317    14573  20678  11009    130   -849
500       C
ATOM   8736  O   ILE B 317      23.117 -22.909  82.166  1.00129.15
O
ANISOU 8736  O   ILE B 317    15204  21937  11930     29   -693
484       O
ATOM   8737  CB  ILE B 317      21.236 -24.764  84.178  1.00 95.12
C
ANISOU 8737  CB  ILE B 317    11717  16835   7589   -262   -810
908       C
ATOM   8738  CG1 ILE B 317      20.035 -25.700  84.292  1.00 94.67
C
ANISOU 8738  CG1 ILE B 317    11965  16487   7520   -526   -804
1029       C
```

```
ATOM   8739  CG2 ILE B 317      20.819 -23.324  84.442  1.00134.43
C
ANISOU 8739  CG2 ILE B 317    16428  22119  12529   -423   -615
1047       C
ATOM   8740  CD1 ILE B 317      19.036 -25.518  83.222  1.00 93.64
C
ANISOU 8740  CD1 ILE B 317    11656  16503   7421   -774   -654
1004       C
ATOM   8741  N   GLU B 318      24.258 -24.490  83.286  1.00150.43
N
ANISOU 8741  N   GLU B 318    18309  24215  14631    436  -1034
390       N
ATOM   8742  CA  GLU B 318      25.528 -23.767  83.214  1.00185.43
C
ANISOU 8742  CA  GLU B 318    22453  28956  19044    660  -1051
220       C
ATOM   8743  C   GLU B 318      26.016 -23.563  81.779  1.00158.54
C
ANISOU 8743  C   GLU B 318    18734  25844  15659    704  -1000
-37       C
ATOM   8744  O   GLU B 318      26.395 -22.450  81.393  1.00150.08
O
ANISOU 8744  O   GLU B 318    17351  25123  14550    640   -866
-87       O
ATOM   8745  CB  GLU B 318      26.576 -24.510  84.045  1.00196.02
C
ANISOU 8745  CB  GLU B 318    23988  30119  20370   1010  -1291
109       C
ATOM   8746  CG  GLU B 318      28.008 -24.083  83.791  1.00204.97
C
ANISOU 8746  CG  GLU B 318    24817  31578  21484   1289  -1352
-159       C
ATOM   8747  CD  GLU B 318      28.971 -24.635  84.824  1.00164.52
C
ANISOU 8747  CD  GLU B 318    19879  26299  16332   1637  -1587
-247       C
ATOM   8748  OE1 GLU B 318      29.741 -23.843  85.409  1.00157.30
O
ANISOU 8748  OE1 GLU B 318    18779  25605  15383   1738  -1572
-272       O
ATOM   8749  OE2 GLU B 318      28.955 -25.863  85.054  1.00166.69
O
ANISOU 8749  OE2 GLU B 318    20507  26219  16608   1812  -1797
-297       O
ATOM   8750  N   GLU B 319      26.014 -24.624  80.971  1.00138.05
N
ANISOU 8750  N   GLU B 319    16239  23107  13107    802  -1109
-202       N
ATOM   8751  CA  GLU B 319      26.389 -24.497  79.564  1.00139.24
C
ANISOU 8751  CA  GLU B 319    16108  23523  13273    828  -1056
-443       C
ATOM   8752  C   GLU B 319      25.430 -23.607  78.777  1.00124.08
C
```

```
ANISOU 8752  C   GLU B 319    14013  21779  11353    495   -822
-336       C
ATOM   8753  O   GLU B 319      25.867 -22.765  77.984  1.00112.57
O
ANISOU 8753  O   GLU B 319    12255  20657   9858    453   -707
-464       O
ATOM   8754  CB  GLU B 319      26.469 -25.881  78.924  1.00162.20
C
ANISOU 8754  CB  GLU B 319    19204  26199  16225   1005  -1237
-621       C
ATOM   8755  CG  GLU B 319      27.582 -26.755  79.468  1.00180.26
C
ANISOU 8755  CG  GLU B 319    21641  28359  18489   1403  -1504
-811       C
ATOM   8756  CD  GLU B 319      27.092 -28.129  79.869  1.00189.54
C
ANISOU 8756  CD  GLU B 319    23292  29051  19673   1477  -1710
-748       C
ATOM   8757  OE1 GLU B 319      27.565 -29.123  79.280  1.00211.90
O
ANISOU 8757  OE1 GLU B 319    26229  31778  22506   1729  -1905
-979       O
ATOM   8758  OE2 GLU B 319      26.238 -28.215  80.776  1.00193.40
O
ANISOU 8758  OE2 GLU B 319    24065  29267  20152   1278  -1682
-472       O
ATOM   8759  N   MET B 320      24.124 -23.781  78.978  1.00125.52
N
ANISOU 8759  N   MET B 320    14386  21745  11559    252   -754
-117       N
ATOM   8760  CA  MET B 320      23.139 -22.898  78.355  1.00121.86
C
ANISOU 8760  CA  MET B 320    13774  21446  11082    -42   -552
-17       C
ATOM   8761  C   MET B 320      23.340 -21.436  78.742  1.00118.50
C
ANISOU 8761  C   MET B 320    13146  21305  10573   -130   -418
75       C
ATOM   8762  O   MET B 320      23.263 -20.547  77.887  1.00124.74
O
ANISOU 8762  O   MET B 320    13725  22352  11317   -250   -291
14       O
ATOM   8763  CB  MET B 320      21.731 -23.358  78.730  1.00153.93
C
ANISOU 8763  CB  MET B 320    18072  25246  15168   -274   -517
193       C
ATOM   8764  CG  MET B 320      21.258 -24.576  77.957  1.00115.32
C
ANISOU 8764  CG  MET B 320    13344  20129  10344   -290   -596
101       C
ATOM   8765  SD  MET B 320      20.723 -24.206  76.280  1.00 90.03
S
ANISOU 8765  SD  MET B 320     9902  17136   7169   -431   -463
-40       S
```

```
ATOM   8766  CE  MET B 320      19.029 -23.704  76.582  1.00 88.46
C
ANISOU 8766  CE  MET B 320     9747  16917   6948   -791   -302
195       C
ATOM   8767  N   ALA B 321      23.586 -21.164  80.025  1.00127.00
N
ANISOU 8767  N   ALA B 321    14312  22325  11618    -76   -455
223       N
ATOM   8768  CA  ALA B 321      23.864 -19.794  80.457  1.00 98.56
C
ANISOU 8768  CA  ALA B 321    10541  18981   7927   -140   -352
309       C
ATOM   8769  C   ALA B 321      25.104 -19.211  79.786  1.00 85.19
C
ANISOU 8769  C   ALA B 321     8582  17605   6182    -27   -339
82       C
ATOM   8770  O   ALA B 321      25.110 -18.036  79.399  1.00107.72
O
ANISOU 8770  O   ALA B 321    11267  20717   8945   -174   -212
93       O
ATOM   8771  CB  ALA B 321      24.012 -19.751  81.977  1.00113.17
C
ANISOU 8771  CB  ALA B 321    12551  20691   9760    -66   -417
490       C
ATOM   8772  N   GLY B 322      26.163 -20.011  79.637  1.00112.16
N
ANISOU 8772  N   GLY B 322    11965  21015   9634    228   -476
-139       N
ATOM   8773  CA  GLY B 322      27.396 -19.516  79.044  1.00148.68
C
ANISOU 8773  CA  GLY B 322    16312  25982  14198    330   -462
-385       C
ATOM   8774  C   GLY B 322      27.424 -19.479  77.528  1.00159.13
C
ANISOU 8774  C   GLY B 322    17461  27494  15509    247   -382
-586       C
ATOM   8775  O   GLY B 322      28.260 -18.770  76.959  1.00123.61
O
ANISOU 8775  O   GLY B 322    12718  23329  10920    224   -312
-758       O
ATOM   8776  N   MET B 323      26.548 -20.240  76.871  1.00160.99
N
ANISOU 8776  N   MET B 323    17823  27524  15822    191   -391
-575       N
ATOM   8777  CA  MET B 323      26.475 -20.372  75.415  1.00129.14
C
ANISOU 8777  CA  MET B 323    13662  23615  11791    131   -332
-762       C
ATOM   8778  C   MET B 323      26.799 -19.099  74.639  1.00124.28
C
ANISOU 8778  C   MET B 323    12809  23361  11050    -49   -168
-841       C
ATOM   8779  O   MET B 323      26.187 -18.048  74.848  1.00122.72
O
```

```
ANISOU 8779  O   MET B 323    12631  23221  10777   -264    -50
-659       O
ATOM   8780  CB  MET B 323      25.094 -20.875  74.999  1.00123.14
C
ANISOU 8780  CB  MET B 323    13083  22600  11107    -24   -303
-632       C
ATOM   8781  CG  MET B 323      25.032 -21.198  73.505  1.00102.16
C
ANISOU 8781  CG  MET B 323    10322  20024   8469    -46   -271
-835       C
ATOM   8782  SD  MET B 323      23.390 -21.572  72.874  1.00103.47
S
ANISOU 8782  SD  MET B 323    10650  19956   8707   -262   -213
-706       S
ATOM   8783  CE  MET B 323      23.031 -23.090  73.751  1.00116.59
C
ANISOU 8783  CE  MET B 323    12624  21198  10478   -132   -396
-613       C
ATOM   8784  N   ASP B 324      27.779 -19.210  73.738  1.00150.57
N
ANISOU 8784  N   ASP B 324    15931  26938  14339     42   -170
-1127       N
ATOM   8785  CA  ASP B 324      28.343 -18.090  72.999  1.00162.00
C
ANISOU 8785  CA  ASP B 324    17159  28755  15638   -128    -26
-1248       C
ATOM   8786  C   ASP B 324      28.038 -18.169  71.509  1.00150.33
C
ANISOU 8786  C   ASP B 324    15619  27357  14144   -240     53
-1397       C
ATOM   8787  O   ASP B 324      28.075 -17.140  70.826  1.00155.77
O
ANISOU 8787  O   ASP B 324    16219  28272  14693   -462    194
-1428       O
ATOM   8788  CB  ASP B 324      29.865 -18.025  73.197  1.00174.30
C
ANISOU 8788  CB  ASP B 324    18487  30616  17125     36    -72
-1489       C
ATOM   8789  CG  ASP B 324      30.464 -16.712  72.728  1.00178.63
C
ANISOU 8789  CG  ASP B 324    18840  31548  17483   -198     87
-1570       C
ATOM   8790  OD1 ASP B 324      31.697 -16.653  72.552  1.00179.23
O
ANISOU 8790  OD1 ASP B 324    18678  31943  17478   -117     82
-1827       O
ATOM   8791  OD2 ASP B 324      29.705 -15.738  72.546  1.00181.14
O
ANISOU 8791  OD2 ASP B 324    19253  31855  17715   -466    210
-1387       O
ATOM   8792  N   VAL B 325      27.731 -19.359  71.000  1.00135.28
N
ANISOU 8792  N   VAL B 325    13784  25252  12363    -95    -42
-1487       N
```

```
ATOM   8793  CA  VAL B 325      27.429 -19.585  69.590  1.00115.27
C
ANISOU 8793  CA  VAL B 325    11203  22761   9832   -167     14
-1635       C
ATOM   8794  C   VAL B 325      26.426 -20.728  69.508  1.00110.24
C
ANISOU 8794  C   VAL B 325    10780  21753   9353    -92    -86
-1549       C
ATOM   8795  O   VAL B 325      26.501 -21.691  70.277  1.00140.57
O
ANISOU 8795  O   VAL B 325    14755  25366  13289    108   -241
-1515       O
ATOM   8796  CB  VAL B 325      28.710 -19.877  68.777  1.00130.81
C
ANISOU 8796  CB  VAL B 325    12926  25034  11743    -23     -7
-1983       C
ATOM   8797  CG1 VAL B 325      29.477 -21.037  69.385  1.00133.19
C
ANISOU 8797  CG1 VAL B 325    13222  25252  12132    331   -209
-2124       C
ATOM   8798  CG2 VAL B 325      28.377 -20.160  67.321  1.00144.23
C
ANISOU 8798  CG2 VAL B 325    14591  26760  13448    -88     46
-2132       C
ATOM   8799  N   LEU B 326      25.477 -20.616  68.579  1.00 88.26
N
ANISOU 8799  N   LEU B 326     8051  18902   6583   -262     -4
-1516       N
ATOM   8800  CA  LEU B 326      24.479 -21.652  68.336  1.00 88.65
C
ANISOU 8800  CA  LEU B 326     8286  18629   6768   -235    -81
-1454       C
ATOM   8801  C   LEU B 326      24.538 -22.096  66.881  1.00 95.94
C
ANISOU 8801  C   LEU B 326     9133  19611   7708   -209    -73
-1676       C
ATOM   8802  O   LEU B 326      24.323 -21.289  65.971  1.00 88.33
O
ANISOU 8802  O   LEU B 326     8082  18818   6660   -384     61
-1723       O
ATOM   8803  CB  LEU B 326      23.080 -21.145  68.689  1.00 86.66
C
ANISOU 8803  CB  LEU B 326     8182  18219   6526   -466      0
-1193       C
ATOM   8804  CG  LEU B 326      21.903 -22.058  68.347  1.00 86.96
C
ANISOU 8804  CG  LEU B 326     8392  17968   6682   -514    -45
-1131       C
ATOM   8805  CD1 LEU B 326      22.080 -23.423  68.994  1.00 90.52
C
ANISOU 8805  CD1 LEU B 326     9012  18143   7238   -326   -220
-1124       C
ATOM   8806  CD2 LEU B 326      20.600 -21.421  68.800  1.00 85.15
C
```

```
ANISOU 8806  CD2 LEU B 326     8257  17663   6434   -740     43
-901       C
ATOM   8807  N   CYS B 327      24.825 -23.381  66.670  1.00 91.56
N
ANISOU 8807  N   CYS B 327     8638  18901   7250     18   -228
-1811       N
ATOM   8808  CA  CYS B 327      24.798 -23.995  65.344  1.00 92.53
C
ANISOU 8808  CA  CYS B 327     8722  19028   7408     75   -250
-2012       C
ATOM   8809  C   CYS B 327      23.427 -24.630  65.126  1.00 98.10
C
ANISOU 8809  C   CYS B 327     9650  19398   8224    -29   -278
-1865       C
ATOM   8810  O   CYS B 327      23.093 -25.635  65.761  1.00 93.25
O
ANISOU 8810  O   CYS B 327     9245  18485   7700     68   -421
-1779       O
ATOM   8811  CB  CYS B 327      25.909 -25.032  65.213  1.00 95.28
C
ANISOU 8811  CB  CYS B 327     9014  19410   7778    404   -425
-2266       C
ATOM   8812  SG  CYS B 327      27.567 -24.337  65.022  1.00 96.32
S
ANISOU 8812  SG  CYS B 327     8797  20038   7760    513   -372
-2547       S
ATOM   8813  N   SER B 328      22.637 -24.044  64.229  1.00102.41
N
ANISOU 8813  N   SER B 328    10166  19995   8750   -236   -146
-1845       N
ATOM   8814  CA  SER B 328      21.239 -24.410  64.030  1.00 92.50
C
ANISOU 8814  CA  SER B 328     9083  18481   7581   -380   -142
-1707       C
ATOM   8815  C   SER B 328      21.044 -25.066  62.669  1.00 90.74
C
ANISOU 8815  C   SER B 328     8856  18208   7412   -334   -172
-1885       C
ATOM   8816  O   SER B 328      21.412 -24.485  61.642  1.00 90.31
O
ANISOU 8816  O   SER B 328     8650  18379   7284   -364    -81
-2043       O
ATOM   8817  CB  SER B 328      20.340 -23.180  64.151  1.00 93.05
C
ANISOU 8817  CB  SER B 328     9140  18638   7578   -638     12
-1543       C
ATOM   8818  OG  SER B 328      18.978 -23.526  63.987  1.00105.62
O
ANISOU 8818  OG  SER B 328    10866  20021   9245   -773     16
-1437       O
ATOM   8819  N   ASP B 329      20.481 -26.274  62.661  1.00 92.06
N
ANISOU 8819  N   ASP B 329     9208  18075   7694   -272   -301
-1862       N
```

```
ATOM   8820  CA  ASP B 329      19.972 -26.839  61.417  1.00 92.52
C
ANISOU 8820  CA  ASP B 329     9296  18042   7813   -276   -320
-1982       C
ATOM   8821  C   ASP B 329      18.935 -25.900  60.811  1.00100.77
C
ANISOU 8821  C   ASP B 329    10296  19166   8827   -525   -161
-1917       C
ATOM   8822  O   ASP B 329      18.079 -25.358  61.515  1.00119.37
O
ANISOU 8822  O   ASP B 329    12706  21481  11170   -704    -94
-1726       O
ATOM   8823  CB  ASP B 329      19.360 -28.220  61.654  1.00 94.21
C
ANISOU 8823  CB  ASP B 329     9764  17892   8141   -227   -484
-1924       C
ATOM   8824  CG  ASP B 329      20.362 -29.223  62.186  1.00120.24
C
ANISOU 8824  CG  ASP B 329    13163  21073  11448     55   -683
-2012       C
ATOM   8825  OD1 ASP B 329      21.335 -29.531  61.466  1.00134.31
O
ANISOU 8825  OD1 ASP B 329    14833  22989  13210    284   -754
-2249       O
ATOM   8826  OD2 ASP B 329      20.174 -29.710  63.320  1.00136.50
O
ANISOU 8826  OD2 ASP B 329    15427  22913  13525     54   -777
-1856       O
ATOM   8827  N   LYS B 330      19.020 -25.703  59.493  1.00107.70
N
ANISOU 8827  N   LYS B 330    11080  20161   9680   -521   -109
-2092       N
ATOM   8828  CA  LYS B 330      18.121 -24.770  58.819  1.00102.83
C
ANISOU 8828  CA  LYS B 330    10440  19622   9010   -723     23
-2065       C
ATOM   8829  C   LYS B 330      16.671 -25.246  58.865  1.00136.48
C
ANISOU 8829  C   LYS B 330    14843  23642  13370   -851     -3
-1948       C
ATOM   8830  O   LYS B 330      15.783 -24.526  59.337  1.00127.48
O
ANISOU 8830  O   LYS B 330    13723  22522  12192  -1021     72
-1808       O
ATOM   8831  CB  LYS B 330      18.572 -24.564  57.371  1.00 94.25
C
ANISOU 8831  CB  LYS B 330     9253  18689   7867   -682     70
-2291       C
ATOM   8832  CG  LYS B 330      17.696 -23.597  56.591  1.00120.68
C
ANISOU 8832  CG  LYS B 330    12615  22099  11137   -866    184
-2289       C
ATOM   8833  CD  LYS B 330      18.357 -23.159  55.297  1.00 87.08
C
```

```
ANISOU 8833  CD  LYS B 330     8273  18037   6775   -851    252
-2503       C
ATOM   8834  CE  LYS B 330      18.605 -24.345  54.387  1.00 88.74
C
ANISOU 8834  CE  LYS B 330     8476  18147   7096   -684    159
-2677       C
ATOM   8835  NZ  LYS B 330      17.332 -25.016  54.006  1.00140.27
N
ANISOU 8835  NZ  LYS B 330    15135  24406  13755   -718     97
-2629       N
ATOM   8836  N   THR B 331      16.410 -26.457  58.374  1.00158.87
N
ANISOU 8836  N   THR B 331    17777  26264  16321   -773   -114
-2016       N
ATOM   8837  CA  THR B 331      15.049 -26.982  58.362  1.00142.49
C
ANISOU 8837  CA  THR B 331    15832  23975  14334   -920   -137
-1925       C
ATOM   8838  C   THR B 331      14.499 -27.160  59.773  1.00167.30
C
ANISOU 8838  C   THR B 331    19082  26992  17492  -1040   -154
-1708       C
ATOM   8839  O   THR B 331      15.156 -27.735  60.645  1.00179.43
O
ANISOU 8839  O   THR B 331    20705  28428  19043   -938   -245
-1648       O
ATOM   8840  CB  THR B 331      15.004 -28.315  57.616  1.00128.32
C
ANISOU 8840  CB  THR B 331    14150  21960  12648   -810   -271
-2039       C
ATOM   8841  OG1 THR B 331      16.067 -29.158  58.076  1.00146.36
O
ANISOU 8841  OG1 THR B 331    16503  24157  14950   -598   -409
-2078       O
ATOM   8842  CG2 THR B 331      15.149 -28.091  56.119  1.00121.02
C
ANISOU 8842  CG2 THR B 331    13127  21144  11712   -747   -233
-2241       C
ATOM   8843  N   GLY B 332      13.285 -26.661  59.987  1.00172.25
N
ANISOU 8843  N   GLY B 332    19707  27635  18105  -1249    -73
-1607       N
ATOM   8844  CA  GLY B 332      12.557 -26.868  61.223  1.00161.99
C
ANISOU 8844  CA  GLY B 332    18505  26231  16815  -1406    -74
-1415       C
ATOM   8845  C   GLY B 332      12.826 -25.834  62.296  1.00136.92
C
ANISOU 8845  C   GLY B 332    15259  23224  13542  -1439      4
-1279       C
ATOM   8846  O   GLY B 332      11.903 -25.162  62.768  1.00134.85
O
ANISOU 8846  O   GLY B 332    14960  23049  13228  -1603     83
-1181       O
```

```
ATOM   8847  N   THR B 333      14.093 -25.699  62.691  1.00122.10
N
ANISOU 8847  N   THR B 333    13356  21406  11631  -1273    -27
-1287       N
ATOM   8848  CA  THR B 333      14.470 -24.663  63.649  1.00142.01
C
ANISOU 8848  CA  THR B 333    15806  24097  14053  -1290     42
-1169       C
ATOM   8849  C   THR B 333      14.285 -23.269  63.060  1.00127.24
C
ANISOU 8849  C   THR B 333    13799  22479  12069  -1353    160
-1218       C
ATOM   8850  O   THR B 333      13.719 -22.380  63.707  1.00111.82
O
ANISOU 8850  O   THR B 333    11824  20629  10034  -1465    226
-1100       O
ATOM   8851  CB  THR B 333      15.910 -24.867  64.112  1.00164.91
C
ANISOU 8851  CB  THR B 333    18695  27021  16942  -1089    -25
-1201       C
ATOM   8852  OG1 THR B 333      16.092 -26.232  64.508  1.00179.00
O
ANISOU 8852  OG1 THR B 333    20655  28540  18816   -996   -170
-1188       O
ATOM   8853  CG2 THR B 333      16.199 -23.971  65.308  1.00168.79
C
ANISOU 8853  CG2 THR B 333    19149  27636  17346  -1122     29
-1048       C
ATOM   8854  N   LEU B 334      14.759 -23.059  61.830  1.00136.78
N
ANISOU 8854  N   LEU B 334    14935  23783  13252  -1279    177
-1399       N
ATOM   8855  CA  LEU B 334      14.685 -21.752  61.192  1.00105.14
C
ANISOU 8855  CA  LEU B 334    10854  19990   9105  -1339    273
-1460       C
ATOM   8856  C   LEU B 334      13.506 -21.627  60.242  1.00 86.30
C
ANISOU 8856  C   LEU B 334     8491  17574   6724  -1429    293
-1536       C
ATOM   8857  O   LEU B 334      13.112 -20.503  59.911  1.00 81.22
O
ANISOU 8857  O   LEU B 334     7841  17071   5949  -1495    352
-1558       O
ATOM   8858  CB  LEU B 334      15.974 -21.465  60.410  1.00 90.89
C
ANISOU 8858  CB  LEU B 334     8966  18340   7230  -1234    294
-1624       C
ATOM   8859  CG  LEU B 334      17.318 -21.586  61.132  1.00 83.89
C
ANISOU 8859  CG  LEU B 334     8014  17535   6324  -1114    268
-1619       C
ATOM   8860  CD1 LEU B 334      18.457 -21.228  60.190  1.00 92.65
C
```

```
ANISOU 8860  CD1 LEU B 334     9010  18854   7338  -1052    311
-1820       C
ATOM   8861  CD2 LEU B 334      17.353 -20.707  62.369  1.00 82.78
C
ANISOU 8861  CD2 LEU B 334     7876  17482   6096  -1184    309
-1439       C
ATOM   8862  N   THR B 335      12.944 -22.747  59.801  1.00130.85
N
ANISOU 8862  N   THR B 335    14181  23032  12504  -1426    232
-1585       N
ATOM   8863  CA  THR B 335      11.944 -22.778  58.748  1.00131.88
C
ANISOU 8863  CA  THR B 335    14322  23130  12655  -1485    238
-1696       C
ATOM   8864  C   THR B 335      10.677 -23.423  59.288  1.00136.65
C
ANISOU 8864  C   THR B 335    14971  23602  13346  -1616    212
-1606       C
ATOM   8865  O   THR B 335      10.722 -24.224  60.227  1.00186.39
O
ANISOU 8865  O   THR B 335    21333  29768  19718  -1647    169
-1484       O
ATOM   8866  CB  THR B 335      12.436 -23.552  57.518  1.00116.72
C
ANISOU 8866  CB  THR B 335    12410  21128  10812  -1372    191
-1872       C
ATOM   8867  OG1 THR B 335      12.520 -24.948  57.830  1.00135.63
O
ANISOU 8867  OG1 THR B 335    14877  23305  13350  -1323     93
-1842       O
ATOM   8868  CG2 THR B 335      13.806 -23.051  57.085  1.00 84.59
C
ANISOU 8868  CG2 THR B 335     8274  17217   6649  -1259    223
-1973       C
ATOM   8869  N   LEU B 336       9.539 -23.061  58.694  1.00 99.54
N
ANISOU 8869  N   LEU B 336    10249  18948   8622  -1701    235
-1680       N
ATOM   8870  CA  LEU B 336       8.294 -23.740  59.037  1.00 99.54
C
ANISOU 8870  CA  LEU B 336    10266  18857   8700  -1849    217
-1639       C
ATOM   8871  C   LEU B 336       8.381 -25.226  58.719  1.00 96.75
C
ANISOU 8871  C   LEU B 336    10003  18260   8496  -1847    137
-1666       C
ATOM   8872  O   LEU B 336       7.795 -26.053  59.427  1.00124.03
O
ANISOU 8872  O   LEU B 336    13528  21586  12012  -1985    110
-1570       O
ATOM   8873  CB  LEU B 336       7.117 -23.100  58.301  1.00118.08
C
ANISOU 8873  CB  LEU B 336    12553  21321  10990  -1906    240
-1765       C
```

```
ATOM   8874  CG  LEU B 336       6.707 -21.696  58.751  1.00139.36
C
ANISOU 8874  CG  LEU B 336    15194  24240  13516  -1920    287
-1740       C
ATOM   8875  CD1 LEU B 336       5.679 -21.105  57.799  1.00164.77
C
ANISOU 8875  CD1 LEU B 336    18382  27557  16666  -1913    275
-1917       C
ATOM   8876  CD2 LEU B 336       6.169 -21.721  60.176  1.00170.74
C
ANISOU 8876  CD2 LEU B 336    19133  28265  17475  -2049    313
-1575       C
ATOM   8877  N   ASN B 337       9.068 -25.569  57.626  1.00 86.86
N
ANISOU 8877  N   ASN B 337     8768  16945   7288  -1702     95
-1804       N
ATOM   8878  CA  ASN B 337       9.335 -26.937  57.191  1.00 88.63
C
ANISOU 8878  CA  ASN B 337     9098  16939   7639  -1645     -5
-1853       C
ATOM   8879  C   ASN B 337       8.074 -27.515  56.562  1.00103.20
C
ANISOU 8879  C   ASN B 337    10970  18685   9556  -1776    -27
-1925       C
ATOM   8880  O   ASN B 337       7.864 -28.733  56.558  1.00 93.62
O
ANISOU 8880  O   ASN B 337     9882  17252   8437  -1823   -113
-1912       O
ATOM   8881  CB  ASN B 337       9.850 -27.817  58.336  1.00 90.05
C
ANISOU 8881  CB  ASN B 337     9403  16951   7859  -1643    -78
-1708       C
ATOM   8882  CG  ASN B 337      10.496 -29.096  57.838  1.00 91.89
C
ANISOU 8882  CG  ASN B 337     9772  16958   8184  -1500   -213
-1785       C
ATOM   8883  OD1 ASN B 337      11.063 -29.134  56.746  1.00100.72
O
ANISOU 8883  OD1 ASN B 337    10844  18106   9319  -1339   -238
-1945       O
ATOM   8884  ND2 ASN B 337      10.409 -30.153  58.635  1.00 94.06
N
ANISOU 8884  ND2 ASN B 337    10235  17001   8501  -1558   -310
-1677       N
ATOM   8885  N   LYS B 338       7.227 -26.637  56.033  1.00 99.65
N
ANISOU 8885  N   LYS B 338    10418  18397   9047  -1834     38
-2009       N
ATOM   8886  CA  LYS B 338       6.038 -27.029  55.285  1.00117.94
C
ANISOU 8886  CA  LYS B 338    12724  20668  11420  -1935     19
-2121       C
ATOM   8887  C   LYS B 338       6.204 -26.461  53.883  1.00119.72
C
```

```
ANISOU 8887  C   LYS B 338    12911  20960  11617  -1789     22
-2309       C
ATOM   8888  O   LYS B 338       6.054 -25.252  53.672  1.00130.14
O
ANISOU 8888  O   LYS B 338    14167  22465  12815  -1757     78
-2362       O
ATOM   8889  CB  LYS B 338       4.764 -26.521  55.959  1.00155.84
C
ANISOU 8889  CB  LYS B 338    17434  25618  16159  -2127     78
-2083       C
ATOM   8890  CG  LYS B 338       4.506 -27.158  57.319  1.00153.46
C
ANISOU 8890  CG  LYS B 338    17186  25246  15875  -2312     84
-1903       C
ATOM   8891  CD  LYS B 338       3.380 -26.469  58.072  1.00141.79
C
ANISOU 8891  CD  LYS B 338    15582  23985  14305  -2486    158
-1874       C
ATOM   8892  CE  LYS B 338       2.077 -26.515  57.292  1.00128.26
C
ANISOU 8892  CE  LYS B 338    13772  22359  12603  -2585    158
-2050       C
ATOM   8893  NZ  LYS B 338       0.979 -25.805  58.007  1.00161.91
N
ANISOU 8893  NZ  LYS B 338    17881  26881  16758  -2727    222
-2061       N
ATOM   8894  N   LEU B 339       6.515 -27.337  52.933  1.00111.53
N
ANISOU 8894  N   LEU B 339    11940  19758  10677  -1699    -47
-2411       N
ATOM   8895  CA  LEU B 339       7.125 -26.939  51.675  1.00108.04
C
ANISOU 8895  CA  LEU B 339    11490  19356  10204  -1531    -47
-2571       C
ATOM   8896  C   LEU B 339       6.069 -26.555  50.647  1.00102.20
C
ANISOU 8896  C   LEU B 339    10725  18655   9451  -1554    -43
-2730       C
ATOM   8897  O   LEU B 339       5.003 -27.172  50.569  1.00 88.78
O
ANISOU 8897  O   LEU B 339     9025  16876   7833  -1667    -80
-2758       O
ATOM   8898  CB  LEU B 339       8.002 -28.070  51.135  1.00115.95
C
ANISOU 8898  CB  LEU B 339    12571  20177  11307  -1394   -134
-2622       C
ATOM   8899  CG  LEU B 339       9.087 -28.594  52.082  1.00 93.61
C
ANISOU 8899  CG  LEU B 339     9787  17289   8491  -1324   -175
-2502       C
ATOM   8900  CD1 LEU B 339       9.816 -29.785  51.474  1.00 91.94
C
ANISOU 8900  CD1 LEU B 339     9668  16895   8370  -1159   -296
-2589       C
```

```
ATOM   8901  CD2 LEU B 339      10.072 -27.491  52.447  1.00 88.94
C
ANISOU 8901  CD2 LEU B 339     9107  16916   7772  -1251    -91
-2477       C
ATOM   8902  N   SER B 340       6.378 -25.528  49.857  1.00111.59
N
ANISOU 8902  N   SER B 340    11906  19969  10522  -1456     -4
-2843       N
ATOM   8903  CA  SER B 340       5.498 -25.043  48.804  1.00 86.15
C
ANISOU 8903  CA  SER B 340     8695  16780   7259  -1437    -16
-3015       C
ATOM   8904  C   SER B 340       6.196 -25.184  47.461  1.00 86.19
C
ANISOU 8904  C   SER B 340     8764  16717   7268  -1294    -35
-3164       C
ATOM   8905  O   SER B 340       7.374 -24.833  47.330  1.00 97.83
O
ANISOU 8905  O   SER B 340    10255  18252   8665  -1216      5
-3160       O
ATOM   8906  CB  SER B 340       5.113 -23.579  49.033  1.00107.49
C
ANISOU 8906  CB  SER B 340    11388  19681   9773  -1450     29
-3031       C
ATOM   8907  OG  SER B 340       4.335 -23.427  50.204  1.00154.57
O
ANISOU 8907  OG  SER B 340    17273  25732  15724  -1572     43
-2921       O
ATOM   8908  N   VAL B 341       5.472 -25.698  46.471  1.00110.96
N
ANISOU 8908  N   VAL B 341    11928  19743  10487  -1269    -93
-3305       N
ATOM   8909  CA  VAL B 341       5.984 -25.861  45.116  1.00103.25
C
ANISOU 8909  CA  VAL B 341    11020  18695   9517  -1135   -116
-3462       C
ATOM   8910  C   VAL B 341       5.103 -25.036  44.195  1.00107.06
C
ANISOU 8910  C   VAL B 341    11553  19222   9904  -1110   -125
-3627       C
ATOM   8911  O   VAL B 341       3.884 -25.242  44.150  1.00123.14
O
ANISOU 8911  O   VAL B 341    13558  21230  11999  -1164   -173
-3683       O
ATOM   8912  CB  VAL B 341       5.988 -27.332  44.669  1.00 88.48
C
ANISOU 8912  CB  VAL B 341     9171  16614   7833  -1093   -202
-3492       C
ATOM   8913  CG1 VAL B 341       6.524 -27.453  43.249  1.00 88.61
C
ANISOU 8913  CG1 VAL B 341     9249  16575   7845   -941   -224
-3663       C
ATOM   8914  CG2 VAL B 341       6.797 -28.174  45.617  1.00 89.38
C
```

```
ANISOU 8914  CG2 VAL B 341     9279  16660   8022  -1096   -226
-3341       C
ATOM   8915  N   ASP B 342       5.708 -24.112  43.458  1.00111.39
N
ANISOU 8915  N   ASP B 342    12190  19844  10290  -1035    -87
-3719       N
ATOM   8916  CA  ASP B 342       5.024 -23.499  42.331  1.00130.08
C
ANISOU 8916  CA  ASP B 342    14665  22196  12565   -974   -122
-3907       C
ATOM   8917  C   ASP B 342       5.340 -24.311  41.080  1.00138.89
C
ANISOU 8917  C   ASP B 342    15828  23163  13779   -873   -159
-4038       C
ATOM   8918  O   ASP B 342       6.508 -24.461  40.705  1.00138.51
O
ANISOU 8918  O   ASP B 342    15801  23123  13705   -822   -117
-4044       O
ATOM   8919  CB  ASP B 342       5.426 -22.031  42.162  1.00129.92
C
ANISOU 8919  CB  ASP B 342    14778  22307  12281   -967    -75
-3942       C
ATOM   8920  CG  ASP B 342       6.912 -21.848  41.921  1.00 98.04
C
ANISOU 8920  CG  ASP B 342    10779  18321   8150   -962      7
-3917       C
ATOM   8921  OD1 ASP B 342       7.269 -21.050  41.026  1.00 83.79
O
ANISOU 8921  OD1 ASP B 342     9135  16543   6158   -943     31
-4034       O
ATOM   8922  OD2 ASP B 342       7.720 -22.493  42.624  1.00 84.84
O
ANISOU 8922  OD2 ASP B 342     8986  16673   6576   -981     43
-3795       O
ATOM   8923  N   LYS B 343       4.301 -24.851  40.450  1.00113.07
N
ANISOU 8923  N   LYS B 343    12563  19776  10621   -844   -239
-4152       N
ATOM   8924  CA  LYS B 343       4.486 -25.792  39.354  1.00 98.52
C
ANISOU 8924  CA  LYS B 343    10759  17771   8904   -748   -291
-4262       C
ATOM   8925  C   LYS B 343       4.904 -25.093  38.069  1.00107.66
C
ANISOU 8925  C   LYS B 343    12065  18929   9914   -645   -275
-4428       C
ATOM   8926  O   LYS B 343       5.311 -25.764  37.114  1.00123.95
O
ANISOU 8926  O   LYS B 343    14165  20882  12049   -552   -301
-4521       O
ATOM   8927  CB  LYS B 343       3.218 -26.628  39.150  1.00118.48
C
ANISOU 8927  CB  LYS B 343    13243  20174  11599   -773   -383
-4327       C
```

```
ATOM   8928  CG  LYS B 343       1.982 -25.861  38.728  1.00133.82
C
ANISOU 8928  CG  LYS B 343    15215  22169  13460   -762   -425
-4480       C
ATOM   8929  CD  LYS B 343       0.761 -26.765  38.818  1.00142.77
C
ANISOU 8929  CD  LYS B 343    16251  23233  14764   -836   -501
-4524       C
ATOM   8930  CE  LYS B 343      -0.498 -26.066  38.346  1.00154.28
C
ANISOU 8930  CE  LYS B 343    17710  24767  16144   -796   -559
-4720       C
ATOM   8931  NZ  LYS B 343      -1.704 -26.903  38.590  1.00191.05
N
ANISOU 8931  NZ  LYS B 343    22228  29415  20948   -912   -615
-4768       N
ATOM   8932  N   ASN B 344       4.801 -23.762  38.038  1.00113.74
N
ANISOU 8932  N   ASN B 344    12942  19813  10462   -664   -240
-4468       N
ATOM   8933  CA  ASN B 344       5.281 -22.973  36.909  1.00128.47
C
ANISOU 8933  CA  ASN B 344    15000  21679  12134   -606   -216
-4610       C
ATOM   8934  C   ASN B 344       6.762 -23.216  36.634  1.00131.20
C
ANISOU 8934  C   ASN B 344    15333  22074  12444   -605   -128
-4587       C
ATOM   8935  O   ASN B 344       7.169 -23.334  35.472  1.00183.57
O
ANISOU 8935  O   ASN B 344    22062  28651  19035   -538   -123
-4725       O
ATOM   8936  CB  ASN B 344       5.018 -21.489  37.175  1.00145.43
C
ANISOU 8936  CB  ASN B 344    17300  23875  14081   -642   -199
-4564       C
ATOM   8937  CG  ASN B 344       5.280 -20.622  35.962  1.00158.20
C
ANISOU 8937  CG  ASN B 344    19187  25267  15654   -580   -188
-4547       C
ATOM   8938  OD1 ASN B 344       5.301 -21.105  34.831  1.00186.11
O
ANISOU 8938  OD1 ASN B 344    22789  28682  19241   -502   -214
-4672       O
ATOM   8939  ND2 ASN B 344       5.477 -19.328  36.192  1.00155.92
N
ANISOU 8939  ND2 ASN B 344    19080  24902  15259   -620   -158
-4390       N
ATOM   8940  N   LEU B 345       7.585 -23.293  37.678  1.00113.03
N
ANISOU 8940  N   LEU B 345    12906  19893  10148   -674    -61
-4430       N
ATOM   8941  CA  LEU B 345       9.014 -23.525  37.500  1.00112.14
C
```

```
ANISOU 8941  CA  LEU B 345    12740  19875   9992   -664     17
-4433       C
ATOM   8942  C   LEU B 345       9.415 -24.995  37.601  1.00 90.74
C
ANISOU 8942  C   LEU B 345     9879  17081   7515   -566    -35
-4409       C
ATOM   8943  O   LEU B 345      10.613 -25.294  37.591  1.00 88.41
O
ANISOU 8943  O   LEU B 345     9506  16890   7196   -528     11
-4422       O
ATOM   8944  CB  LEU B 345       9.815 -22.708  38.519  1.00107.37
C
ANISOU 8944  CB  LEU B 345    12096  19468   9232   -779    112
-4304       C
ATOM   8945  CG  LEU B 345       9.754 -21.184  38.392  1.00108.97
C
ANISOU 8945  CG  LEU B 345    12491  19717   9197   -878    165
-4288       C
ATOM   8946  CD1 LEU B 345      10.653 -20.521  39.427  1.00 91.08
C
ANISOU 8946  CD1 LEU B 345    10166  17595   6843   -988    254
-4119       C
ATOM   8947  CD2 LEU B 345      10.143 -20.752  36.986  1.00124.54
C
ANISOU 8947  CD2 LEU B 345    14647  21527  11147   -855    195
-4343       C
ATOM   8948  N   VAL B 346       8.457 -25.913  37.706  1.00 88.11
N
ANISOU 8948  N   VAL B 346     9515  16574   7390   -526   -139
-4386       N
ATOM   8949  CA  VAL B 346       8.762 -27.334  37.553  1.00 89.42
C
ANISOU 8949  CA  VAL B 346     9614  16609   7752   -418   -220
-4392       C
ATOM   8950  C   VAL B 346       9.154 -27.581  36.098  1.00 90.04
C
ANISOU 8950  C   VAL B 346     9762  16638   7809   -293   -235
-4582       C
ATOM   8951  O   VAL B 346       8.317 -27.478  35.197  1.00123.51
O
ANISOU 8951  O   VAL B 346    14105  20764  12061   -266   -277
-4694       O
ATOM   8952  CB  VAL B 346       7.580 -28.218  37.966  1.00 89.89
C
ANISOU 8952  CB  VAL B 346     9660  16484   8009   -450   -326
-4326       C
ATOM   8953  CG1 VAL B 346       7.898 -29.681  37.694  1.00 91.43
C
ANISOU 8953  CG1 VAL B 346     9851  16509   8380   -335   -434
-4343       C
ATOM   8954  CG2 VAL B 346       7.259 -28.017  39.435  1.00134.76
C
ANISOU 8954  CG2 VAL B 346    15270  22231  13701   -586   -302
-4140       C
```

```
ATOM   8955  N   GLU B 347      10.422 -27.908  35.862  1.00116.08
N
ANISOU 8955  N   GLU B 347    12998  20037  11068   -207   -204
-4635       N
ATOM   8956  CA  GLU B 347      10.897 -28.231  34.524  1.00134.76
C
ANISOU 8956  CA  GLU B 347    15409  22385  13411    -81   -215
-4821       C
ATOM   8957  C   GLU B 347      10.889 -29.741  34.331  1.00144.40
C
ANISOU 8957  C   GLU B 347    16596  23424  14844     84   -359
-4843       C
ATOM   8958  O   GLU B 347      11.312 -30.496  35.211  1.00123.53
O
ANISOU 8958  O   GLU B 347    13872  20769  12294    131   -419
-4745       O
ATOM   8959  CB  GLU B 347      12.301 -27.671  34.283  1.00133.45
C
ANISOU 8959  CB  GLU B 347    15182  22478  13046    -89    -94
-4905       C
ATOM   8960  CG  GLU B 347      12.351 -26.153  34.180  1.00145.81
C
ANISOU 8960  CG  GLU B 347    16849  24195  14356   -266     41
-4915       C
ATOM   8961  CD  GLU B 347      11.567 -25.622  32.994  1.00161.84
C
ANISOU 8961  CD  GLU B 347    19086  26101  16304   -277     33
-5041       C
ATOM   8962  OE1 GLU B 347      12.027 -25.801  31.846  1.00179.71
O
ANISOU 8962  OE1 GLU B 347    21399  28373  18509   -209     51
-5205       O
ATOM   8963  OE2 GLU B 347      10.489 -25.028  33.209  1.00167.44
O
ANISOU 8963  OE2 GLU B 347    19910  26711  16998   -341      2
-4990       O
ATOM   8964  N   VAL B 348      10.405 -30.174  33.169  1.00148.78
N
ANISOU 8964  N   VAL B 348    17241  23823  15463    179   -427
-4975       N
ATOM   8965  CA  VAL B 348      10.140 -31.578  32.899  1.00146.09
C
ANISOU 8965  CA  VAL B 348    16920  23263  15324    322   -587
-4995       C
ATOM   8966  C   VAL B 348      10.883 -32.008  31.643  1.00133.71
C
ANISOU 8966  C   VAL B 348    15369  21709  13727    507   -612
-5187       C
ATOM   8967  O   VAL B 348      11.246 -31.195  30.790  1.00138.60
O
ANISOU 8967  O   VAL B 348    16017  22461  14185    492   -506
-5315       O
ATOM   8968  CB  VAL B 348       8.631 -31.865  32.755  1.00163.90
C
```

ANISOU 8968 CB VAL B 348   19267 25289 17719   258   -673
-4968   C
ATOM   8969 CG1 VAL B 348   7.911 -31.599 34.066 1.00187.01
C
ANISOU 8969 CG1 VAL B 348   22151 28224 20679   78   -656
-4789   C
ATOM   8970 CG2 VAL B 348   8.037 -31.033 31.628 1.00165.44
C
ANISOU 8970 CG2 VAL B 348   19561 25475 17824   248   -627
-5113   C
ATOM   8971 N   PHE B 349   11.115 -33.313 31.546 1.00125.73
N
ANISOU 8971 N   PHE B 349   14359 20556 12858   679   -763
-5208   N
ATOM   8972 CA PHE B 349   11.854 -33.913 30.448 1.00137.32
C
ANISOU 8972 CA PHE B 349   15827 22035 14312   892   -819
-5393   C
ATOM   8973 C   PHE B 349   10.999 -35.028 29.863 1.00155.34
C
ANISOU 8973 C   PHE B 349   18237 24003 16782   1001   -998
-5417   C
ATOM   8974 O   PHE B 349   9.965 -35.399 30.427 1.00169.82
O
ANISOU 8974 O   PHE B 349   20140 25637 18748   896 -1075
-5288   O
ATOM   8975 CB PHE B 349   13.212 -34.463 30.902 1.00139.37
C
ANISOU 8975 CB PHE B 349   15960 22465 14527   1052   -855
-5432   C
ATOM   8976 CG PHE B 349   14.084 -33.447 31.584 1.00146.60
C
ANISOU 8976 CG PHE B 349   16734 23701 15267   934   -687
-5405   C
ATOM   8977 CD1 PHE B 349   14.359 -33.547 32.938 1.00150.64
C
ANISOU 8977 CD1 PHE B 349   17177 24260 15801   892   -704
-5255   C
ATOM   8978 CD2 PHE B 349   14.639 -32.400 30.868 1.00169.13
C
ANISOU 8978 CD2 PHE B 349   19543 26801 17917   853   -513
-5534   C
ATOM   8979 CE1 PHE B 349   15.167 -32.616 33.566 1.00154.72
C
ANISOU 8979 CE1 PHE B 349   17560 25071 16155   784   -554
-5234   C
ATOM   8980 CE2 PHE B 349   15.447 -31.467 31.491 1.00154.12
C
ANISOU 8980 CE2 PHE B 349   17524 25197 15839   718   -359
-5512   C
ATOM   8981 CZ PHE B 349   15.711 -31.575 32.841 1.00155.51
C
ANISOU 8981 CZ PHE B 349   17611 25424 16054   691   -381
-5362   C

```
ATOM   8982  N   CYS B 350      11.425 -35.521  28.695  1.00173.85
N
ANISOU 8982  N   CYS B 350    20612  26321  19122   1196  -1057
-5593       N
ATOM   8983  CA  CYS B 350      10.738 -36.546  27.908  1.00169.70
C
ANISOU 8983  CA  CYS B 350    20219  25508  18752   1326  -1229
-5651       C
ATOM   8984  C   CYS B 350       9.604 -35.928  27.095  1.00159.15
C
ANISOU 8984  C   CYS B 350    18980  24060  17432   1218  -1180
-5698       C
ATOM   8985  O   CYS B 350       9.451 -36.239  25.910  1.00157.52
O
ANISOU 8985  O   CYS B 350    18854  23744  17251   1350  -1237
-5841       O
ATOM   8986  CB  CYS B 350      10.210 -37.696  28.773  1.00145.43
C
ANISOU 8986  CB  CYS B 350    17226  22184  15848   1326  -1412
-5503       C
ATOM   8987  SG  CYS B 350      11.454 -38.495  29.808  1.00142.98
S
ANISOU 8987  SG  CYS B 350    16861  21952  15512   1483  -1518
-5451       S
ATOM   8988  N   LYS B 351       8.791 -35.082  27.734  1.00153.86
N
ANISOU 8988  N   LYS B 351    18305  23411  16741   1000  -1090
-5589       N
ATOM   8989  CA  LYS B 351       7.696 -34.316  27.125  1.00160.45
C
ANISOU 8989  CA  LYS B 351    19226  24179  17559    901  -1046
-5645       C
ATOM   8990  C   LYS B 351       6.484 -35.193  26.834  1.00172.91
C
ANISOU 8990  C   LYS B 351    20889  25478  19331    912  -1201
-5648       C
ATOM   8991  O   LYS B 351       5.560 -34.750  26.134  1.00170.86
O
ANISOU 8991  O   LYS B 351    20703  25140  19074    886  -1202
-5741       O
ATOM   8992  CB  LYS B 351       8.122 -33.556  25.859  1.00140.60
C
ANISOU 8992  CB  LYS B 351    16780  21757  14883    973   -955
-5828       C
ATOM   8993  CG  LYS B 351       8.911 -32.271  26.145  1.00114.92
C
ANISOU 8993  CG  LYS B 351    13486  18785  11392    852   -768
-5825       C
ATOM   8994  CD  LYS B 351      10.323 -32.524  26.671  1.00112.42
C
ANISOU 8994  CD  LYS B 351    13021  18688  11007    900   -717
-5804       C
ATOM   8995  CE  LYS B 351      11.058 -31.214  26.937  1.00104.61
C
```

```
ANISOU 8995  CE  LYS B 351    11997  17982   9769    743   -525
-5805       C
ATOM   8996  NZ  LYS B 351      11.256 -30.408  25.700  1.00 96.29
N
ANISOU 8996  NZ  LYS B 351    11074  16992   8519    719   -428
-5975       N
ATOM   8997  N   GLY B 352       6.453 -36.418  27.348  1.00164.17
N
ANISOU 8997  N   GLY B 352    19791  24215  18371    946  -1342
-5559       N
ATOM   8998  CA  GLY B 352       5.272 -37.254  27.286  1.00143.83
C
ANISOU 8998  CA  GLY B 352    17297  21388  15964    890  -1482
-5536       C
ATOM   8999  C   GLY B 352       4.345 -36.996  28.457  1.00146.54
C
ANISOU 8999  C   GLY B 352    17585  21751  16342    636  -1447
-5389       C
ATOM   9000  O   GLY B 352       3.152 -37.305  28.400  1.00123.02
O
ANISOU 9000  O   GLY B 352    14636  18640  13465    522  -1512
-5398       O
ATOM   9001  N   VAL B 353       4.892 -36.429  29.530  1.00157.04
N
ANISOU 9001  N   VAL B 353    18823  23265  17582    541  -1342
-5265       N
ATOM   9002  CA  VAL B 353       4.157 -36.164  30.762  1.00159.63
C
ANISOU 9002  CA  VAL B 353    19086  23643  17923    304  -1298
-5115       C
ATOM   9003  C   VAL B 353       4.261 -34.676  31.075  1.00133.53
C
ANISOU 9003  C   VAL B 353    15689  20589  14457    228  -1129
-5113       C
ATOM   9004  O   VAL B 353       5.361 -34.110  31.095  1.00125.68
O
ANISOU 9004  O   VAL B 353    14661  19754  13337    302  -1039
-5116       O
ATOM   9005  CB  VAL B 353       4.672 -37.027  31.930  1.00158.69
C
ANISOU 9005  CB  VAL B 353    18981  23466  17849    254  -1363
-4941       C
ATOM   9006  CG1 VAL B 353       4.336 -38.489  31.685  1.00157.96
C
ANISOU 9006  CG1 VAL B 353    19036  23086  17896    288  -1555
-4935       C
ATOM   9007  CG2 VAL B 353       6.175 -36.867  32.099  1.00148.82
C
ANISOU 9007  CG2 VAL B 353    17689  22359  16498    417  -1321
-4934       C
ATOM   9008  N   GLU B 354       3.111 -34.042  31.290  1.00120.57
N
ANISOU 9008  N   GLU B 354    14013  18992  12806     81  -1094
-5126       N
```

```
ATOM   9009  CA  GLU B 354       3.042 -32.596  31.429  1.00118.83
C
ANISOU 9009  CA  GLU B 354    13755  18978  12417     34   -968
-5154       C
ATOM   9010  C   GLU B 354       3.469 -32.164  32.835  1.00160.13
C
ANISOU 9010  C   GLU B 354    18890  24372  17578   -100   -879
-4972       C
ATOM   9011  O   GLU B 354       3.490 -32.954  33.783  1.00167.24
O
ANISOU 9011  O   GLU B 354    19752  25220  18573   -191   -917
-4826       O
ATOM   9012  CB  GLU B 354       1.628 -32.105  31.091  1.00121.60
C
ANISOU 9012  CB  GLU B 354    14109  19322  12773    -27   -995
-5270       C
ATOM   9013  CG  GLU B 354       1.467 -30.588  31.021  1.00131.03
C
ANISOU 9013  CG  GLU B 354    15327  20692  13767    -28   -908
-5342       C
ATOM   9014  CD  GLU B 354       0.144 -30.161  30.413  1.00138.60
C
ANISOU 9014  CD  GLU B 354    16318  21628  14717     -7   -973
-5521       C
ATOM   9015  OE1 GLU B 354      -0.687 -31.042  30.111  1.00155.45
O
ANISOU 9015  OE1 GLU B 354    18420  23633  17011    -21  -1070
-5583       O
ATOM   9016  OE2 GLU B 354      -0.062 -28.943  30.234  1.00142.41
O
ANISOU 9016  OE2 GLU B 354    16870  22220  15019     26   -939
-5610       O
ATOM   9017  N   LYS B 355       3.825 -30.880  32.945  1.00161.65
N
ANISOU 9017  N   LYS B 355    19077  24754  17589   -114   -765
-4983       N
ATOM   9018  CA  LYS B 355       4.302 -30.299  34.200  1.00136.21
C
ANISOU 9018  CA  LYS B 355    15772  21704  14277   -226   -672
-4822       C
ATOM   9019  C   LYS B 355       3.297 -30.466  35.337  1.00133.24
C
ANISOU 9019  C   LYS B 355    15312  21339  13975   -400   -691
-4704       C
ATOM   9020  O   LYS B 355       3.678 -30.755  36.477  1.00120.59
O
ANISOU 9020  O   LYS B 355    13647  19776  12396   -490   -667
-4535       O
ATOM   9021  CB  LYS B 355       4.596 -28.814  33.980  1.00145.84
C
ANISOU 9021  CB  LYS B 355    17043  23099  15271   -228   -567
-4879       C
ATOM   9022  CG  LYS B 355       5.722 -28.535  32.997  1.00165.86
C
```

ANISOU 9022 CG LYS B 355   19659 25673 17687   -113   -515
-4985   C
ATOM   9023 CD LYS B 355   5.612 -27.128 32.419 1.00164.93
C
ANISOU 9023 CD LYS B 355   19680 25649 17337   -130   -449
-5091   C
ATOM   9024 CE LYS B 355   5.762 -26.056 33.483 1.00157.19
C
ANISOU 9024 CE LYS B 355   18679 24850 16196   -260   -364
-4972   C
ATOM   9025 NZ LYS B 355   7.180 -25.879 33.895 1.00176.84
N
ANISOU 9025 NZ LYS B 355   21112 27494 18585   -296   -259
-4891   N
ATOM   9026 N   ASP B 356   2.012 -30.261 35.051 1.00153.44
N
ANISOU 9026 N   ASP B 356   17863 23880 16557   -450   -732
-4805   N
ATOM   9027 CA ASP B 356   0.954 -30.573 36.011 1.00148.72
C
ANISOU 9027 CA ASP B 356   17165 23309 16035   -633   -753
-4729   C
ATOM   9028 C   ASP B 356   0.905 -32.057 36.369 1.00130.39
C
ANISOU 9028 C   ASP B 356   14849 20806 13889   -717   -832
-4630   C
ATOM   9029 O   ASP B 356   0.733 -32.414 37.541 1.00126.69
O
ANISOU 9029 O   ASP B 356   14325 20361 13449   -889   -817
-4475   O
ATOM   9030 CB ASP B 356   -0.395 -30.111 35.458 1.00151.00
C
ANISOU 9030 CB ASP B 356   17430 23638 16307   -640   -796
-4912   C
ATOM   9031 CG ASP B 356   -1.508 -30.213 36.481 1.00147.94
C
ANISOU 9031 CG ASP B 356   16899 23360 15951   -844   -795
-4867   C
ATOM   9032 OD1 ASP B 356   -2.659 -30.496 36.086 1.00146.27
O
ANISOU 9032 OD1 ASP B 356   16635 23137 15802   -889   -860
-5011   O
ATOM   9033 OD2 ASP B 356   -1.230 -30.005 37.680 1.00157.94
O
ANISOU 9033 OD2 ASP B 356   18097 24739 17173   -966   -726
-4698   O
ATOM   9034 N   GLN B 357   1.051 -32.933 35.374 1.00118.50
N
ANISOU 9034 N   GLN B 357   13435 19105 12485   -601   -925
-4717   N
ATOM   9035 CA GLN B 357   0.965 -34.376 35.598 1.00103.11
C
ANISOU 9035 CA GLN B 357   11548 16946 10683   -670 -1031
-4638   C

```
ATOM   9036  C   GLN B 357       2.048 -34.914  36.534  1.00108.28           C
ANISOU 9036  C   GLN B 357    12244  17561  11335   -671  -1036  -4455       C
ATOM   9037  O   GLN B 357       1.770 -35.786  37.365  1.00125.71           O
ANISOU 9037  O   GLN B 357    14498  19657  13608   -830  -1093  -4327       O
ATOM   9038  CB  GLN B 357       1.023 -35.100  34.253  1.00113.86           C
ANISOU 9038  CB  GLN B 357    13018  18109  12132   -501  -1141  -4782       C
ATOM   9039  CG  GLN B 357       0.976 -36.614  34.341  1.00112.16           C
ANISOU 9039  CG  GLN B 357    12921  17644  12051   -546  -1281  -4715       C
ATOM   9040  CD  GLN B 357       0.939 -37.267  32.974  1.00138.80           C
ANISOU 9040  CD  GLN B 357    16402  20828  15508   -370  -1396  -4868       C
ATOM   9041  OE1 GLN B 357       1.148 -36.609  31.954  1.00134.64           O
ANISOU 9041  OE1 GLN B 357    15866  20358  14935   -194  -1362  -5016       O
ATOM   9042  NE2 GLN B 357       0.670 -38.567  32.945  1.00155.21           N
ANISOU 9042  NE2 GLN B 357    18609  22669  17696   -424  -1538  -4831       N
ATOM   9043  N   VAL B 358       3.285 -34.423  36.420  1.00 96.78           N
ANISOU 9043  N   VAL B 358    10784  16198   9792   -504   -982  -4449       N
ATOM   9044  CA  VAL B 358       4.387 -35.057  37.145  1.00118.57           C
ANISOU 9044  CA  VAL B 358    13586  18910  12554   -448  -1018  -4320       C
ATOM   9045  C   VAL B 358       4.293 -34.913  38.664  1.00138.68           C
ANISOU 9045  C   VAL B 358    16088  21534  15070   -638   -966  -4129       C
ATOM   9046  O   VAL B 358       4.957 -35.665  39.388  1.00 98.71           O
ANISOU 9046  O   VAL B 358    11100  16376  10028   -620  -1033  -4013       O
ATOM   9047  CB  VAL B 358       5.735 -34.493  36.660  1.00135.38           C
ANISOU 9047  CB  VAL B 358    15681  21177  14579   -238   -959  -4390       C
ATOM   9048  CG1 VAL B 358       5.969 -34.863  35.205  1.00155.03           C
ANISOU 9048  CG1 VAL B 358    18235  23570  17099    -42  -1025  -4571       C
ATOM   9049  CG2 VAL B 358       5.801 -32.989  36.869  1.00134.05           C
```

```
ANISOU 9049  CG2 VAL B 358    15411  21262  14260   -299   -799
-4393       C
ATOM   9050  N   LEU B 359       3.502 -33.964  39.170  1.00156.82
N
ANISOU 9050  N   LEU B 359    18277  24000  17307   -801   -861
-4103       N
ATOM   9051  CA  LEU B 359       3.254 -33.873  40.610  1.00104.67
C
ANISOU 9051  CA  LEU B 359    11627  17466  10676  -1001   -814
-3926       C
ATOM   9052  C   LEU B 359       2.625 -35.141  41.184  1.00 99.14
C
ANISOU 9052  C   LEU B 359    11031  16561  10077  -1183   -914
-3831       C
ATOM   9053  O   LEU B 359       2.914 -35.511  42.329  1.00120.29
O
ANISOU 9053  O   LEU B 359    13759  19203  12741  -1290   -920
-3665       O
ATOM   9054  CB  LEU B 359       2.369 -32.664  40.915  1.00110.39
C
ANISOU 9054  CB  LEU B 359    12217  18415  11313  -1126   -704
-3952       C
ATOM   9055  CG  LEU B 359       2.818 -31.330  40.317  1.00113.97
C
ANISOU 9055  CG  LEU B 359    12623  19048  11631   -979   -618
-4054       C
ATOM   9056  CD1 LEU B 359       1.836 -30.227  40.677  1.00116.45
C
ANISOU 9056  CD1 LEU B 359    12842  19558  11847  -1086   -551
-4089       C
ATOM   9057  CD2 LEU B 359       4.222 -30.980  40.785  1.00101.13
C
ANISOU 9057  CD2 LEU B 359    10998  17504   9922   -886   -561
-3957       C
ATOM   9058  N   LEU B 360       1.762 -35.809  40.416  1.00116.57
N
ANISOU 9058  N   LEU B 360    13292  18627  12373  -1234   -995
-3934       N
ATOM   9059  CA  LEU B 360       1.183 -37.082  40.845  1.00120.53
C
ANISOU 9059  CA  LEU B 360    13935  18910  12953  -1432  -1101
-3854       C
ATOM   9060  C   LEU B 360       2.237 -38.151  41.120  1.00125.55
C
ANISOU 9060  C   LEU B 360    14783  19310  13609  -1316  -1233
-3752       C
ATOM   9061  O   LEU B 360       2.126 -38.892  42.103  1.00157.51
O
ANISOU 9061  O   LEU B 360    18970  23226  17651  -1497  -1288
-3604       O
ATOM   9062  CB  LEU B 360       0.175 -37.587  39.808  1.00102.94
C
ANISOU 9062  CB  LEU B 360    11729  16574  10811  -1481  -1174
-4008       C
```

```
ATOM   9063  CG  LEU B 360      -1.221 -36.948  39.776  1.00102.64
C
ANISOU 9063  CG  LEU B 360    11511  16728  10759  -1677  -1092
-4112       C
ATOM   9064  CD1 LEU B 360      -1.192 -35.474  39.379  1.00100.30
C
ANISOU 9064  CD1 LEU B 360    11044  16688  10378  -1520   -983
-4228       C
ATOM   9065  CD2 LEU B 360      -2.137 -37.727  38.842  1.00110.10
C
ANISOU 9065  CD2 LEU B 360    12507  17525  11800  -1740  -1192
-4254       C
ATOM   9066  N   PHE B 361       3.269 -38.246  40.279  1.00103.13
N
ANISOU 9066  N   PHE B 361    11984  16422  10777  -1012  -1293
-3841       N
ATOM   9067  CA  PHE B 361       4.338 -39.211  40.540  1.00112.20
C
ANISOU 9067  CA  PHE B 361    13323  17382  11928   -848  -1437
-3779       C
ATOM   9068  C   PHE B 361       5.036 -38.957  41.873  1.00121.73
C
ANISOU 9068  C   PHE B 361    14524  18672  13054   -882  -1392
-3619       C
ATOM   9069  O   PHE B 361       5.358 -39.906  42.598  1.00126.75
O
ANISOU 9069  O   PHE B 361    15369  19107  13685   -903  -1522
-3509       O
ATOM   9070  CB  PHE B 361       5.356 -39.192  39.398  1.00123.03
C
ANISOU 9070  CB  PHE B 361    14679  18767  13298   -503  -1486
-3936       C
ATOM   9071  CG  PHE B 361       4.753 -39.408  38.040  1.00105.27
C
ANISOU 9071  CG  PHE B 361    12444  16430  11123   -440  -1533
-4098       C
ATOM   9072  CD1 PHE B 361       5.063 -38.567  36.985  1.00102.71
C
ANISOU 9072  CD1 PHE B 361    11986  16270  10771   -266  -1446
-4256       C
ATOM   9073  CD2 PHE B 361       3.896 -40.472  37.812  1.00121.26
C
ANISOU 9073  CD2 PHE B 361    14641  18199  13234   -565  -1669
-4095       C
ATOM   9074  CE1 PHE B 361       4.512 -38.771  35.733  1.00112.76
C
ANISOU 9074  CE1 PHE B 361    13287  17447  12108   -196  -1496
-4409       C
ATOM   9075  CE2 PHE B 361       3.344 -40.683  36.563  1.00113.65
C
ANISOU 9075  CE2 PHE B 361    13690  17149  12342   -498  -1719
-4248       C
ATOM   9076  CZ  PHE B 361       3.652 -39.832  35.522  1.00122.15
C
```

```
ANISOU 9076  CZ  PHE B 361    14626  18386  13399   -301  -1634
-4406       C
ATOM   9077  N   ALA B 362       5.293 -37.693  42.212  1.00102.41
N
ANISOU 9077  N   ALA B 362    11869  16507  10536   -880  -1223
-3605       N
ATOM   9078  CA  ALA B 362       5.807 -37.379  43.543  1.00102.18
C
ANISOU 9078  CA  ALA B 362    11821  16569  10434   -944  -1169
-3446       C
ATOM   9079  C   ALA B 362       4.825 -37.792  44.637  1.00103.30
C
ANISOU 9079  C   ALA B 362    12051  16617  10582  -1268  -1169
-3288       C
ATOM   9080  O   ALA B 362       5.214 -38.397  45.643  1.00104.67
O
ANISOU 9080  O   ALA B 362    12382  16661  10726  -1318  -1241
-3148       O
ATOM   9081  CB  ALA B 362       6.125 -35.886  43.638  1.00 99.75
C
ANISOU 9081  CB  ALA B 362    11284  16578  10038   -910   -988
-3466       C
ATOM   9082  N   ALA B 363       3.544 -37.467  44.453  1.00137.71
N
ANISOU 9082  N   ALA B 363    16311  21050  14962  -1494  -1092
-3324       N
ATOM   9083  CA  ALA B 363       2.495 -37.905  45.373  1.00131.07
C
ANISOU 9083  CA  ALA B 363    15531  20152  14116  -1841  -1081
-3208       C
ATOM   9084  C   ALA B 363       2.379 -39.426  45.446  1.00136.55
C
ANISOU 9084  C   ALA B 363    16530  20503  14851  -1938  -1260
-3153       C
ATOM   9085  O   ALA B 363       2.148 -39.986  46.523  1.00156.27
O
ANISOU 9085  O   ALA B 363    19186  22887  17303  -2172  -1288
-3000       O
ATOM   9086  CB  ALA B 363       1.159 -37.286  44.962  1.00130.36
C
ANISOU 9086  CB  ALA B 363    15249  20245  14039  -2024   -979
-3319       C
ATOM   9087  N   MET B 364       2.528 -40.109  44.308  1.00122.57
N
ANISOU 9087  N   MET B 364    14869  18552  13150  -1768  -1391
-3275       N
ATOM   9088  CA  MET B 364       2.561 -41.571  44.294  1.00128.02
C
ANISOU 9088  CA  MET B 364    15897  18886  13860  -1809  -1595
-3231       C
ATOM   9089  C   MET B 364       3.669 -42.144  45.172  1.00128.88
C
ANISOU 9089  C   MET B 364    16238  18828  13903  -1678  -1717
-3103       C
```

```
ATOM   9090  O   MET B 364       3.445 -43.122  45.895  1.00146.41
O
ANISOU 9090  O   MET B 364    18757  20794  16079  -1871  -1837
-2982       O
ATOM   9091  CB  MET B 364       2.694 -42.083  42.860  1.00159.58
C
ANISOU 9091  CB  MET B 364    19954  22741  17937  -1578  -1720
-3399       C
ATOM   9092  CG  MET B 364       1.426 -41.915  42.042  1.00160.96
C
ANISOU 9092  CG  MET B 364    20000  22979  18179  -1752  -1660
-3521       C
ATOM   9093  SD  MET B 364       1.656 -42.220  40.283  1.00130.39
S
ANISOU 9093  SD  MET B 364    16147  18998  14400  -1435  -1772
-3732       S
ATOM   9094  CE  MET B 364      -0.016 -41.973  39.686  1.00123.02
C
ANISOU 9094  CE  MET B 364    15056  18158  13527  -1713  -1692
-3853       C
ATOM   9095  N   ALA B 365       4.861 -41.554  45.145  1.00133.82
N
ANISOU 9095  N   ALA B 365    16746  19596  14504  -1362  -1693
-3137       N
ATOM   9096  CA  ALA B 365       5.953 -42.050  45.971  1.00151.51
C
ANISOU 9096  CA  ALA B 365    19183  21708  16675  -1199  -1818
-3047       C
ATOM   9097  C   ALA B 365       5.993 -41.408  47.355  1.00156.52
C
ANISOU 9097  C   ALA B 365    19746  22488  17237  -1371  -1691
-2883       C
ATOM   9098  O   ALA B 365       7.031 -41.459  48.025  1.00154.15
O
ANISOU 9098  O   ALA B 365    19518  22173  16877  -1187  -1753
-2832       O
ATOM   9099  CB  ALA B 365       7.290 -41.839  45.256  1.00168.73
C
ANISOU 9099  CB  ALA B 365    21268  23987  18854   -765  -1873
-3193       C
ATOM   9100  N   SER B 366       4.889 -40.814  47.796  1.00146.57
N
ANISOU 9100  N   SER B 366    18340  21377  15975  -1706  -1522
-2814       N
ATOM   9101  CA  SER B 366       4.761 -40.275  49.143  1.00129.12
C
ANISOU 9101  CA  SER B 366    16078  19293  13690  -1907  -1405
-2651       C
ATOM   9102  C   SER B 366       3.773 -41.126  49.932  1.00133.64
C
ANISOU 9102  C   SER B 366    16891  19664  14224  -2303  -1446
-2521       C
ATOM   9103  O   SER B 366       2.696 -41.458  49.426  1.00169.62
O
```

```
ANISOU 9103  O   SER B 366    21449  24183  18817  -2535  -1435
-2576       O
ATOM   9104  CB  SER B 366       4.293 -38.820  49.103  1.00142.96
C
ANISOU 9104  CB  SER B 366    17460  21419  15440  -1975  -1175
-2688       C
ATOM   9105  OG  SER B 366       5.233 -37.999  48.432  1.00154.81
O
ANISOU 9105  OG  SER B 366    18775  23101  16945  -1654  -1129
-2798       O
ATOM   9106  N   ARG B 367       4.135 -41.477  51.163  1.00115.25
N
ANISOU 9106  N   ARG B 367    14771  17210  11808  -2393  -1493
-2356       N
ATOM   9107  CA  ARG B 367       3.213 -42.193  52.036  1.00130.77
C
ANISOU 9107  CA  ARG B 367    16977  19007  13702  -2817  -1508
-2219       C
ATOM   9108  C   ARG B 367       2.039 -41.302  52.436  1.00131.93
C
ANISOU 9108  C   ARG B 367    16820  19471  13835  -3159  -1273
-2200       C
ATOM   9109  O   ARG B 367       2.166 -40.081  52.561  1.00161.53
O
ANISOU 9109  O   ARG B 367    20242  23540  17592  -3057  -1113
-2222       O
ATOM   9110  CB  ARG B 367       3.933 -42.747  53.265  1.00151.39
C
ANISOU 9110  CB  ARG B 367    19917  21398  16207  -2814  -1622
-2052       C
ATOM   9111  CG  ARG B 367       4.893 -43.874  52.910  1.00141.44
C
ANISOU 9111  CG  ARG B 367    19033  19776  14930  -2513  -1905
-2090       C
ATOM   9112  CD  ARG B 367       5.219 -44.778  54.092  1.00156.57
C
ANISOU 9112  CD  ARG B 367    21413  21365  16711  -2616  -2071
-1928       C
ATOM   9113  NE  ARG B 367       6.179 -44.193  55.022  1.00180.22
N
ANISOU 9113  NE  ARG B 367    24347  24470  19659  -2411  -2043
-1855       N
ATOM   9114  CZ  ARG B 367       6.869 -44.897  55.914  1.00196.56
C
ANISOU 9114  CZ  ARG B 367    26808  26260  21616  -2323  -2232
-1761       C
ATOM   9115  NH1 ARG B 367       6.632 -46.194  56.062  1.00206.24
N
ANISOU 9115  NH1 ARG B 367    28547  27068  22747  -2462  -2455
-1710       N
ATOM   9116  NH2 ARG B 367       7.733 -44.293  56.718  1.00197.71
N
ANISOU 9116  NH2 ARG B 367    26857  26536  21728  -2124  -2197
-1712       N
```

```
ATOM   9117  N   VAL B 368       0.882 -41.936  52.643  1.00116.75
N
ANISOU 9117  N   VAL B 368    15017  17466  11875  -3574  -1262
-2171       N
ATOM   9118  CA  VAL B 368      -0.388 -41.223  52.755  1.00116.13
C
ANISOU 9118  CA  VAL B 368    14624  17713  11788  -3891  -1062
-2222       C
ATOM   9119  C   VAL B 368      -0.900 -41.111  54.192  1.00153.44
C
ANISOU 9119  C   VAL B 368    19379  22532  16389  -4264   -949
-2063       C
ATOM   9120  O   VAL B 368      -1.882 -40.388  54.429  1.00167.77
O
ANISOU 9120  O   VAL B 368    20892  24676  18176  -4498   -773
-2113       O
ATOM   9121  CB  VAL B 368      -1.459 -41.889  51.860  1.00140.12
C
ANISOU 9121  CB  VAL B 368    17686  20684  14868  -4118  -1099
-2352       C
ATOM   9122  CG1 VAL B 368      -1.994 -43.157  52.514  1.00166.01
C
ANISOU 9122  CG1 VAL B 368    21354  23675  18047  -4538  -1191
-2234       C
ATOM   9123  CG2 VAL B 368      -2.581 -40.911  51.507  1.00151.57
C
ANISOU 9123  CG2 VAL B 368    18708  22539  16344  -4257   -914
-2503       C
ATOM   9124  N   GLU B 369      -0.264 -41.774  55.160  1.00153.01
N
ANISOU 9124  N   GLU B 369    19678  22212  16248  -4309  -1049
-1887       N
ATOM   9125  CA  GLU B 369      -0.812 -41.805  56.513  1.00152.68
C
ANISOU 9125  CA  GLU B 369    19716  22222  16071  -4707   -948
-1732       C
ATOM   9126  C   GLU B 369       0.262 -41.498  57.553  1.00141.21
C
ANISOU 9126  C   GLU B 369    18377  20713  14563  -4509   -969
-1574       C
ATOM   9127  O   GLU B 369       0.133 -40.536  58.318  1.00117.88
O
ANISOU 9127  O   GLU B 369    15173  18046  11571  -4563   -804
-1518       O
ATOM   9128  CB  GLU B 369      -1.461 -43.162  56.792  1.00124.27
C
ANISOU 9128  CB  GLU B 369    16537  18308  12373  -5131  -1053
-1663       C
ATOM   9129  CG  GLU B 369      -2.081 -43.283  58.173  1.00126.24
C
ANISOU 9129  CG  GLU B 369    16904  18603  12459  -5601   -944
-1508       C
ATOM   9130  CD  GLU B 369      -2.952 -44.517  58.316  1.00140.89
C
```

```
ANISOU 9130  CD  GLU B 369    19125  20212  14194  -6109  -1009
-1473       C
ATOM   9131  OE1 GLU B 369      -4.178 -44.362  58.499  1.00174.09
O
ANISOU 9131  OE1 GLU B 369    23120  24687  18340  -6551   -840
-1529       O
ATOM   9132  OE2 GLU B 369      -2.413 -45.642  58.246  1.00156.39
O
ANISOU 9132  OE2 GLU B 369    21592  21720  16107  -6070  -1236
-1402       O
ATOM   9133  N   ASN B 370       1.320 -42.307  57.601  1.00125.34
N
ANISOU 9133  N   ASN B 370    16743  18339  12539  -4266  -1182
-1514       N
ATOM   9134  CA  ASN B 370       2.489 -42.010  58.429  1.00119.71
C
ANISOU 9134  CA  ASN B 370    16118  17577  11789  -3991  -1230
-1405       C
ATOM   9135  C   ASN B 370       3.404 -41.055  57.657  1.00124.27
C
ANISOU 9135  C   ASN B 370    16357  18376  12482  -3512  -1201
-1530       C
ATOM   9136  O   ASN B 370       4.555 -41.348  57.338  1.00117.90
O
ANISOU 9136  O   ASN B 370    15681  17412  11702  -3132  -1361
-1572       O
ATOM   9137  CB  ASN B 370       3.205 -43.297  58.819  1.00123.19
C
ANISOU 9137  CB  ASN B 370    17119  17546  12143  -3922  -1492
-1319       C
ATOM   9138  CG  ASN B 370       4.329 -43.069  59.814  1.00132.59
C
ANISOU 9138  CG  ASN B 370    18429  18677  13274  -3671  -1552
-1210       C
ATOM   9139  OD1 ASN B 370       4.465 -41.984  60.381  1.00165.84
O
ANISOU 9139  OD1 ASN B 370    22327  23189  17494  -3631  -1379
-1165       O
ATOM   9140  ND2 ASN B 370       5.147 -44.094  60.023  1.00127.04
N
ANISOU 9140  ND2 ASN B 370    18192  17579  12499  -3483  -1816
-1180       N
ATOM   9141  N   GLN B 371       2.855 -39.877  57.370  1.00128.83
N
ANISOU 9141  N   GLN B 371    16499  19342  13108  -3546   -995
-1602       N
ATOM   9142  CA  GLN B 371       3.391 -38.995  56.340  1.00139.10
C
ANISOU 9142  CA  GLN B 371    17485  20860  14507  -3185   -952
-1755       C
ATOM   9143  C   GLN B 371       4.633 -38.255  56.814  1.00116.06
C
ANISOU 9143  C   GLN B 371    14475  18050  11574  -2864   -940
-1711       C
```

```
ATOM   9144  O   GLN B 371       4.612 -37.585  57.852  1.00108.40
O
ANISOU 9144  O   GLN B 371    13412  17236  10538  -2963   -828
-1592       O
ATOM   9145  CB  GLN B 371       2.327 -37.990  55.907  1.00148.69
C
ANISOU 9145  CB  GLN B 371    18318  22430  15747  -3336   -760
-1855       C
ATOM   9146  CG  GLN B 371       1.011 -38.607  55.495  1.00156.22
C
ANISOU 9146  CG  GLN B 371    19299  23348  16709  -3677   -749
-1923       C
ATOM   9147  CD  GLN B 371       0.156 -37.649  54.692  1.00138.83
C
ANISOU 9147  CD  GLN B 371    16717  21481  14551  -3685   -614
-2095       C
ATOM   9148  OE1 GLN B 371      -0.582 -36.838  55.253  1.00166.88
O
ANISOU 9148  OE1 GLN B 371    20034  25329  18046  -3864   -463
-2092       O
ATOM   9149  NE2 GLN B 371       0.258 -37.728  53.370  1.00116.37
N
ANISOU 9149  NE2 GLN B 371    13822  18594  11800  -3472   -679
-2257       N
ATOM   9150  N   ASP B 372       5.716 -38.386  56.050  1.00113.50
N
ANISOU 9150  N   ASP B 372    14169  17654  11300  -2484  -1056
-1816       N
ATOM   9151  CA  ASP B 372       6.817 -37.440  56.137  1.00115.23
C
ANISOU 9151  CA  ASP B 372    14186  18083  11515  -2176  -1004
-1841       C
ATOM   9152  C   ASP B 372       6.322 -36.047  55.756  1.00130.15
C
ANISOU 9152  C   ASP B 372    15693  20342  13415  -2215   -797
-1903       C
ATOM   9153  O   ASP B 372       5.369 -35.893  54.988  1.00148.51
O
ANISOU 9153  O   ASP B 372    17897  22749  15781  -2346   -736
-1998       O
ATOM   9154  CB  ASP B 372       7.958 -37.876  55.216  1.00111.20
C
ANISOU 9154  CB  ASP B 372    13733  17472  11048  -1789  -1158
-1987       C
ATOM   9155  CG  ASP B 372       9.267 -37.175  55.526  1.00124.53
C
ANISOU 9155  CG  ASP B 372    15282  19333  12701  -1490  -1141
-2007       C
ATOM   9156  OD1 ASP B 372       9.335 -36.448  56.538  1.00159.21
O
ANISOU 9156  OD1 ASP B 372    19583  23872  17039  -1577  -1033
-1884       O
ATOM   9157  OD2 ASP B 372      10.232 -37.354  54.753  1.00129.10
O
```

```
ANISOU 9157  OD2 ASP B 372    15834  19917  13301  -1172  -1237
-2156       O
ATOM   9158  N   ALA B 373       6.970 -35.022  56.314  1.00137.60
N
ANISOU 9158  N   ALA B 373    16466  21504  14311  -2097   -702
-1856       N
ATOM   9159  CA  ALA B 373       6.505 -33.651  56.117  1.00134.85
C
ANISOU 9159  CA  ALA B 373    15811  21488  13937  -2140   -524
-1898       C
ATOM   9160  C   ALA B 373       6.494 -33.252  54.642  1.00128.74
C
ANISOU 9160  C   ALA B 373    14886  20818  13211  -1982   -507
-2094       C
ATOM   9161  O   ALA B 373       5.545 -32.616  54.169  1.00154.67
O
ANISOU 9161  O   ALA B 373    18011  24266  16491  -2098   -413
-2169       O
ATOM   9162  CB  ALA B 373       7.373 -32.685  56.925  1.00146.50
C
ANISOU 9162  CB  ALA B 373    17175  23147  15341  -2015   -452
-1814       C
ATOM   9163  N   ILE B 374       7.544 -33.613  53.902  1.00107.13
N
ANISOU 9163  N   ILE B 374    12200  17996  10507  -1709   -603
-2195       N
ATOM   9164  CA  ILE B 374       7.556 -33.445  52.448  1.00155.06
C
ANISOU 9164  CA  ILE B 374    18175  24118  16621  -1566   -605
-2385       C
ATOM   9165  C   ILE B 374       6.480 -34.291  51.763  1.00101.31
C
ANISOU 9165  C   ILE B 374    11465  17136   9891  -1712   -668
-2449       C
ATOM   9166  O   ILE B 374       5.813 -33.828  50.829  1.00 98.20
O
ANISOU 9166  O   ILE B 374    10943  16849   9519  -1733   -611
-2575       O
ATOM   9167  CB  ILE B 374       8.968 -33.741  51.898  1.00155.80
C
ANISOU 9167  CB  ILE B 374    18301  24178  16720  -1248   -698
-2487       C
ATOM   9168  CG1 ILE B 374       9.069 -33.371  50.416  1.00 97.13
C
ANISOU 9168  CG1 ILE B 374    10751  16846   9309  -1106   -671
-2684       C
ATOM   9169  CG2 ILE B 374       9.379 -35.190  52.148  1.00147.59
C
ANISOU 9169  CG2 ILE B 374    17529  22828  15721  -1164   -896
-2459       C
ATOM   9170  CD1 ILE B 374      10.493 -33.198  49.935  1.00 96.96
C
ANISOU 9170  CD1 ILE B 374    10663  16931   9246   -822   -694
-2801       C
```

```
ATOM   9171  N   ASP B 375       6.298 -35.538  52.203  1.00101.47
N
ANISOU 9171  N   ASP B 375    11733  16880   9942  -1816   -797
-2370       N
ATOM   9172  CA  ASP B 375       5.231 -36.381  51.664  1.00113.48
C
ANISOU 9172  CA  ASP B 375    13365  18230  11521  -2006   -856
-2416       C
ATOM   9173  C   ASP B 375       3.845 -35.780  51.881  1.00135.15
C
ANISOU 9173  C   ASP B 375    15942  21162  14247  -2309   -715
-2410       C
ATOM   9174  O   ASP B 375       3.011 -35.770  50.968  1.00164.08
O
ANISOU 9174  O   ASP B 375    19521  24863  17958  -2371   -700
-2543       O
ATOM   9175  CB  ASP B 375       5.304 -37.772  52.296  1.00113.49
C
ANISOU 9175  CB  ASP B 375    13713  17892  11515  -2107  -1022
-2306       C
ATOM   9176  CG  ASP B 375       6.490 -38.575  51.811  1.00140.67
C
ANISOU 9176  CG  ASP B 375    17347  21121  14978  -1777  -1212
-2373       C
ATOM   9177  OD1 ASP B 375       7.418 -37.978  51.225  1.00168.99
O
ANISOU 9177  OD1 ASP B 375    20767  24868  18574  -1480  -1191
-2482       O
ATOM   9178  OD2 ASP B 375       6.493 -39.807  52.018  1.00144.15
O
ANISOU 9178  OD2 ASP B 375    18117  21243  15410  -1820  -1390
-2326       O
ATOM   9179  N   ALA B 376       3.579 -35.282  53.088  1.00151.95
N
ANISOU 9179  N   ALA B 376    18013  23420  16301  -2489   -617
-2272       N
ATOM   9180  CA  ALA B 376       2.302 -34.633  53.382  1.00135.47
C
ANISOU 9180  CA  ALA B 376    15734  21564  14174  -2753   -484
-2286       C
ATOM   9181  C   ALA B 376       2.033 -33.410  52.507  1.00131.45
C
ANISOU 9181  C   ALA B 376    14959  21328  13657  -2610   -393
-2448       C
ATOM   9182  O   ALA B 376       0.907 -33.221  52.032  1.00173.09
O
ANISOU 9182  O   ALA B 376    20107  26721  18938  -2745   -353
-2568       O
ATOM   9183  CB  ALA B 376       2.246 -34.246  54.861  1.00145.04
C
ANISOU 9183  CB  ALA B 376    16932  22882  15294  -2922   -399
-2110       C
ATOM   9184  N   ALA B 377       3.043 -32.569  52.283  1.00121.80
N
```

```
ANISOU 9184  N   ALA B 377    13663  20210  12405  -2345   -366
-2465       N
ATOM   9185  CA  ALA B 377       2.899 -31.458  51.342  1.00136.79
C
ANISOU 9185  CA  ALA B 377    15384  22317  14273  -2199   -303
-2624       C
ATOM   9186  C   ALA B 377       2.626 -31.915  49.907  1.00118.59
C
ANISOU 9186  C   ALA B 377    13106  19904  12050  -2105   -374
-2806       C
ATOM   9187  O   ALA B 377       1.725 -31.389  49.244  1.00101.21
O
ANISOU 9187  O   ALA B 377    10787  17833   9837  -2134   -341
-2950       O
ATOM   9188  CB  ALA B 377       4.151 -30.581  51.390  1.00 93.75
C
ANISOU 9188  CB  ALA B 377     9894  16974   8755  -1969   -265
-2599       C
ATOM   9189  N   MET B 378       3.394 -32.883  49.403  1.00109.00
N
ANISOU 9189  N   MET B 378    12049  18454  10910  -1971   -483
-2817       N
ATOM   9190  CA  MET B 378       3.186 -33.347  48.030  1.00124.99
C
ANISOU 9190  CA  MET B 378    14110  20367  13014  -1866   -557
-2986       C
ATOM   9191  C   MET B 378       1.823 -34.003  47.819  1.00 98.94
C
ANISOU 9191  C   MET B 378    10827  16993   9773  -2101   -588
-3041       C
ATOM   9192  O   MET B 378       1.176 -33.771  46.791  1.00124.81
O
ANISOU 9192  O   MET B 378    14024  20319  13078  -2065   -589
-3210       O
ATOM   9193  CB  MET B 378       4.319 -34.276  47.598  1.00133.06
C
ANISOU 9193  CB  MET B 378    15301  21166  14091  -1656   -683
-2993       C
ATOM   9194  CG  MET B 378       5.624 -33.528  47.383  1.00119.94
C
ANISOU 9194  CG  MET B 378    13566  19638  12367  -1399   -643
-3026       C
ATOM   9195  SD  MET B 378       7.004 -34.556  46.866  1.00147.49
S
ANISOU 9195  SD  MET B 378    17204  22937  15899  -1113   -795
-3082       S
ATOM   9196  CE  MET B 378       8.286 -33.316  46.698  1.00146.77
C
ANISOU 9196  CE  MET B 378    16940  23130  15696   -908   -683
-3143       C
ATOM   9197  N   VAL B 379       1.372 -34.834  48.760  1.00100.82
N
ANISOU 9197  N   VAL B 379    11178  17113  10017  -2354   -616
-2909       N
```

```
ATOM   9198  CA  VAL B 379       0.040 -35.426  48.631  1.00102.54
C
ANISOU 9198  CA  VAL B 379    11394  17299  10266  -2632   -628
-2967       C
ATOM   9199  C   VAL B 379      -1.032 -34.342  48.564  1.00117.56
C
ANISOU 9199  C   VAL B 379    13029  19524  12116  -2727   -509
-3087       C
ATOM   9200  O   VAL B 379      -1.986 -34.442  47.783  1.00114.75
O
ANISOU 9200  O   VAL B 379    12591  19211  11796  -2794   -524
-3252       O
ATOM   9201  CB  VAL B 379      -0.219 -36.418  49.780  1.00127.92
C
ANISOU 9201  CB  VAL B 379    14798  20350  13455  -2933   -662
-2793       C
ATOM   9202  CG1 VAL B 379      -1.680 -36.853  49.796  1.00124.42
C
ANISOU 9202  CG1 VAL B 379    14306  19957  13009  -3289   -635
-2859       C
ATOM   9203  CG2 VAL B 379       0.696 -37.629  49.646  1.00117.14
C
ANISOU 9203  CG2 VAL B 379    13750  18625  12134  -2814   -830
-2721       C
ATOM   9204  N   GLY B 380      -0.890 -33.287  49.369  1.00126.90
N
ANISOU 9204  N   GLY B 380    14075  20938  13203  -2715   -406
-3020       N
ATOM   9205  CA  GLY B 380      -1.861 -32.204  49.354  1.00132.58
C
ANISOU 9205  CA  GLY B 380    14557  21970  13846  -2762   -318
-3146       C
ATOM   9206  C   GLY B 380      -1.711 -31.236  48.199  1.00119.52
C
ANISOU 9206  C   GLY B 380    12820  20421  12172  -2482   -323
-3327       C
ATOM   9207  O   GLY B 380      -2.643 -30.475  47.921  1.00118.59
O
ANISOU 9207  O   GLY B 380    12542  20523  11994  -2489   -295
-3486       O
ATOM   9208  N   MET B 381      -0.555 -31.246  47.530  1.00119.93
N
ANISOU 9208  N   MET B 381    12988  20326  12256  -2236   -365
-3319       N
ATOM   9209  CA  MET B 381      -0.251 -30.245  46.508  1.00142.43
C
ANISOU 9209  CA  MET B 381    15798  23270  15051  -1988   -357
-3470       C
ATOM   9210  C   MET B 381      -1.255 -30.266  45.360  1.00120.26
C
ANISOU 9210  C   MET B 381    12943  20473  12279  -1962   -406
-3698       C
ATOM   9211  O   MET B 381      -1.626 -29.210  44.833  1.00125.80
O
```

```
ANISOU 9211  O   MET B 381    13574  21337  12887  -1843   -391
-3847       O
ATOM   9212  CB  MET B 381       1.162 -30.461  45.968  1.00139.67
C
ANISOU 9212  CB  MET B 381    15576  22765  14729  -1770   -391
-3436       C
ATOM   9213  CG  MET B 381       1.705 -29.284  45.176  1.00132.60
C
ANISOU 9213  CG  MET B 381    14663  21991  13728  -1558   -353
-3547       C
ATOM   9214  SD  MET B 381       3.097 -29.748  44.131  1.00189.82
S
ANISOU 9214  SD  MET B 381    22031  29075  21018  -1324   -400
-3597       S
ATOM   9215  CE  MET B 381       4.128 -30.621  45.306  1.00190.20
C
ANISOU 9215  CE  MET B 381    22136  29016  21113  -1353   -421
-3384       C
ATOM   9216  N   LEU B 382      -1.709 -31.451  44.960  1.00 96.48
N
ANISOU 9216  N   LEU B 382     9990  17279   9388  -2067   -478
-3734       N
ATOM   9217  CA  LEU B 382      -2.515 -31.616  43.756  1.00136.82
C
ANISOU 9217  CA  LEU B 382    15076  22359  14552  -2018   -540
-3953       C
ATOM   9218  C   LEU B 382      -4.014 -31.444  43.997  1.00145.10
C
ANISOU 9218  C   LEU B 382    15949  23611  15571  -2216   -522
-4082       C
ATOM   9219  O   LEU B 382      -4.808 -31.721  43.091  1.00137.18
O
ANISOU 9219  O   LEU B 382    14913  22589  14622  -2209   -582
-4273       O
ATOM   9220  CB  LEU B 382      -2.224 -32.982  43.120  1.00141.90
C
ANISOU 9220  CB  LEU B 382    15882  22700  15334  -2016   -641
-3942       C
ATOM   9221  CG  LEU B 382      -2.058 -34.223  44.010  1.00107.47
C
ANISOU 9221  CG  LEU B 382    11650  18150  11031  -2231   -679
-3754       C
ATOM   9222  CD1 LEU B 382      -3.279 -34.498  44.865  1.00102.03
C
ANISOU 9222  CD1 LEU B 382    10866  17579  10320  -2580   -637
-3734       C
ATOM   9223  CD2 LEU B 382      -1.716 -35.444  43.166  1.00113.46
C
ANISOU 9223  CD2 LEU B 382    12605  18601  11904  -2157   -810
-3779       C
ATOM   9224  N   ALA B 383      -4.411 -31.036  45.202  1.00125.16
N
ANISOU 9224  N   ALA B 383    13303  21292  12961  -2392   -444
-3994       N
```

```
ATOM   9225  CA  ALA B 383      -5.744 -30.539  45.563  1.00135.39
C
ANISOU 9225  CA  ALA B 383    14381  22882  14178  -2540   -410
-4140       C
ATOM   9226  C   ALA B 383      -6.757 -31.656  45.768  1.00142.91
C
ANISOU 9226  C   ALA B 383    15277  23821  15201  -2863   -420
-4178       C
ATOM   9227  O   ALA B 383      -7.967 -31.373  45.827  1.00122.59
O
ANISOU 9227  O   ALA B 383    12495  21509  12573  -2985   -404
-4363       O
ATOM   9228  CB  ALA B 383      -6.303 -29.543  44.540  1.00101.34
C
ANISOU 9228  CB  ALA B 383     9983  18724   9798  -2305   -456
-4409       C
ATOM   9229  N   ASP B 384      -6.314 -32.908  45.889  1.00132.41
N
ANISOU 9229  N   ASP B 384    14133  22205  13972  -3009   -455
-4024       N
ATOM   9230  CA  ASP B 384      -7.186 -34.025  46.228  1.00112.43
C
ANISOU 9230  CA  ASP B 384    11606  19632  11479  -3375   -463
-4021       C
ATOM   9231  C   ASP B 384      -6.311 -35.223  46.577  1.00106.83
C
ANISOU 9231  C   ASP B 384    11191  18562  10837  -3471   -517
-3789       C
ATOM   9232  O   ASP B 384      -5.874 -35.958  45.682  1.00107.13
O
ANISOU 9232  O   ASP B 384    11409  18322  10974  -3345   -624
-3808       O
ATOM   9233  CB  ASP B 384      -8.136 -34.361  45.075  1.00123.95
C
ANISOU 9233  CB  ASP B 384    12989  21103  13004  -3382   -532
-4276       C
ATOM   9234  CG  ASP B 384      -9.122 -35.464  45.427  1.00163.60
C
ANISOU 9234  CG  ASP B 384    17999  26119  18042  -3806   -530
-4293       C
ATOM   9235  OD1 ASP B 384      -9.869 -35.901  44.526  1.00184.75
O
ANISOU 9235  OD1 ASP B 384    20633  28782  20781  -3848   -592
-4488       O
ATOM   9236  OD2 ASP B 384      -9.169 -35.879  46.604  1.00166.66
O
ANISOU 9236  OD2 ASP B 384    18429  26524  18369  -4112   -465
-4119       O
ATOM   9237  N   PRO B 385      -6.057 -35.454  47.868  1.00129.04
N
ANISOU 9237  N   PRO B 385    14074  21368  13587  -3680   -461
-3579       N
ATOM   9238  CA  PRO B 385      -5.264 -36.622  48.294  1.00141.42
C
```

```
ANISOU 9238  CA  PRO B 385    15962  22580  15192  -3771   -538
-3367       C
ATOM   9239  C   PRO B 385      -5.684 -37.954  47.688  1.00111.42
C
ANISOU 9239  C   PRO B 385    12358  18513  11464  -3948   -651
-3408       C
ATOM   9240  O   PRO B 385      -4.845 -38.853  47.543  1.00132.61
O
ANISOU 9240  O   PRO B 385    15341  20851  14194  -3862   -771
-3292       O
ATOM   9241  CB  PRO B 385      -5.459 -36.614  49.816  1.00148.20
C
ANISOU 9241  CB  PRO B 385    16818  23550  15942  -4072   -443
-3193       C
ATOM   9242  CG  PRO B 385      -5.581 -35.157  50.144  1.00107.68
C
ANISOU 9242  CG  PRO B 385    11401  18779  10735  -3930   -329
-3251       C
ATOM   9243  CD  PRO B 385      -6.392 -34.576  49.003  1.00107.05
C
ANISOU 9243  CD  PRO B 385    11098  18894  10681  -3799   -337
-3529       C
ATOM   9244  N   LYS B 386      -6.961 -38.110  47.337  1.00112.95
N
ANISOU 9244  N   LYS B 386    12395  18863  11656  -4187   -626
-3582       N
ATOM   9245  CA  LYS B 386      -7.416 -39.323  46.663  1.00117.29
C
ANISOU 9245  CA  LYS B 386    13125  19170  12271  -4360   -737
-3640       C
ATOM   9246  C   LYS B 386      -6.736 -39.524  45.311  1.00114.52
C
ANISOU 9246  C   LYS B 386    12893  18579  12040  -3979   -868
-3724       C
ATOM   9247  O   LYS B 386      -6.477 -40.665  44.908  1.00131.47
O
ANISOU 9247  O   LYS B 386    15323  20389  14241  -4012  -1003
-3675       O
ATOM   9248  CB  LYS B 386      -8.935 -39.282  46.505  1.00164.64
C
ANISOU 9248  CB  LYS B 386    18867  25451  18236  -4670   -671
-3850       C
ATOM   9249  CG  LYS B 386      -9.658 -39.096  47.828  1.00167.84
C
ANISOU 9249  CG  LYS B 386    19128  26137  18505  -5068   -532
-3792       C
ATOM   9250  CD  LYS B 386      -9.296 -40.196  48.810  1.00159.98
C
ANISOU 9250  CD  LYS B 386    18483  24855  17448  -5396   -559
-3536       C
ATOM   9251  CE  LYS B 386      -9.978 -39.986  50.150  1.00161.18
C
ANISOU 9251  CE  LYS B 386    18501  25291  17450  -5804   -408
-3473       C
```

```
ATOM   9252  NZ  LYS B 386     -11.458 -40.068  50.043  1.00171.46
N
ANISOU 9252  NZ  LYS B 386    19533  26920  18694  -6178   -327
-3697       N
ATOM   9253  N   GLU B 387      -6.453 -38.437  44.589  1.00118.50
N
ANISOU 9253  N   GLU B 387    13206  19246  12572  -3621   -838
-3856       N
ATOM   9254  CA  GLU B 387      -5.725 -38.555  43.326  1.00139.24
C
ANISOU 9254  CA  GLU B 387    15945  21665  15296  -3257   -947
-3934       C
ATOM   9255  C   GLU B 387      -4.369 -39.222  43.521  1.00147.63
C
ANISOU 9255  C   GLU B 387    17299  22414  16380  -3092  -1041
-3744       C
ATOM   9256  O   GLU B 387      -3.935 -40.022  42.684  1.00151.20
O
ANISOU 9256  O   GLU B 387    17949  22592  16908  -2941  -1178
-3772       O
ATOM   9257  CB  GLU B 387      -5.532 -37.175  42.694  1.00138.74
C
ANISOU 9257  CB  GLU B 387    15668  21832  15214  -2929   -884
-4080       C
ATOM   9258  CG  GLU B 387      -6.767 -36.562  42.064  1.00131.34
C
ANISOU 9258  CG  GLU B 387    14488  21148  14269  -2956   -855
-4336       C
ATOM   9259  CD  GLU B 387      -6.435 -35.302  41.289  1.00130.03
C
ANISOU 9259  CD  GLU B 387    14216  21120  14068  -2593   -835
-4477       C
ATOM   9260  OE1 GLU B 387      -5.627 -35.386  40.340  1.00110.05
O
ANISOU 9260  OE1 GLU B 387    11824  18396  11594  -2320   -903
-4501       O
ATOM   9261  OE2 GLU B 387      -6.969 -34.227  41.638  1.00129.78
O
ANISOU 9261  OE2 GLU B 387    13983  21391  13936  -2585   -759
-4569       O
ATOM   9262  N   ALA B 388      -3.686 -38.894  44.618  1.00148.84
N
ANISOU 9262  N   ALA B 388    17479  22614  16460  -3100   -980
-3566       N
ATOM   9263  CA  ALA B 388      -2.368 -39.456  44.896  1.00136.45
C
ANISOU 9263  CA  ALA B 388    16165  20785  14894  -2918  -1076
-3408       C
ATOM   9264  C   ALA B 388      -2.384 -40.980  44.966  1.00128.32
C
ANISOU 9264  C   ALA B 388    15478  19394  13885  -3081  -1239
-3325       C
ATOM   9265  O   ALA B 388      -1.428 -41.634  44.532  1.00113.00
O
```

ANISOU 9265 O   ALA B 388   13764 17191 11979 -2833 -1386
-3300   O
ATOM   9266 CB ALA B 388   -1.822 -38.874 46.199 1.00149.67
C
ANISOU 9266 CB ALA B 388   17803 22588 16478 -2955   -981
-3235   C
ATOM   9267 N   ARG B 389   -3.451 -41.569 45.507 1.00143.05
N
ANISOU 9267 N   ARG B 389   17397 21244 15710 -3501 -1223
-3292   N
ATOM   9268 CA ARG B 389   -3.536 -43.017 45.661 1.00162.24
C
ANISOU 9268 CA ARG B 389   20205 23314 18123 -3716 -1381
-3201   C
ATOM   9269 C   ARG B 389   -4.531 -43.680 44.711 1.00160.70
C
ANISOU 9269 C   ARG B 389   20035 23037 17987 -3884 -1449
-3352   C
ATOM   9270 O   ARG B 389   -4.864 -44.854 44.906 1.00164.72
O
ANISOU 9270 O   ARG B 389   20853 23276 18457 -4162 -1564
-3283   O
ATOM   9271 CB ARG B 389   -3.909 -43.360 47.106 1.00185.25
C
ANISOU 9271 CB ARG B 389   23252 26223 20913 -4130 -1326
-3022   C
ATOM   9272 CG ARG B 389   -3.027 -42.716 48.180 1.00166.37
C
ANISOU 9272 CG ARG B 389   20838 23920 18456 -4005 -1254
-2864   C
ATOM   9273 CD ARG B 389   -1.539 -42.740 47.856 1.00171.44
C
ANISOU 9273 CD ARG B 389   21618 24381 19138 -3536 -1377
-2829   C
ATOM   9274 NE ARG B 389   -1.009 -44.088 47.684 1.00174.94
N
ANISOU 9274 NE ARG B 389   22497 24400 19572 -3481 -1609
-2771   N
ATOM   9275 CZ ARG B 389   0.197 -44.355 47.193 1.00160.04
C
ANISOU 9275 CZ ARG B 389   20753 22335 17719 -3064 -1759
-2791   C
ATOM   9276 NH1 ARG B 389   1.025 -43.365 46.889 1.00158.32
N
ANISOU 9276 NH1 ARG B 389   20277 22335 17542 -2707 -1681
-2853   N
ATOM   9277 NH2 ARG B 389   0.599 -45.611 47.063 1.00168.12
N
ANISOU 9277 NH2 ARG B 389   22192 22971 18714 -3010 -1994
-2753   N
ATOM   9278 N   ALA B 390   -5.017 -42.965 43.697 1.00169.71
N
ANISOU 9278 N   ALA B 390   20883 24392 19209 -3729 -1389
-3559   N

```
ATOM   9279  CA  ALA B 390      -6.117 -43.442  42.862  1.00179.45
C
ANISOU 9279  CA  ALA B 390    22074  25614  20493  -3913  -1428
-3727       C
ATOM   9280  C   ALA B 390      -5.820 -44.745  42.122  1.00186.90
C
ANISOU 9280  C   ALA B 390    23381  26143  21491  -3856  -1643
-3715       C
ATOM   9281  O   ALA B 390      -5.047 -44.756  41.158  1.00193.55
O
ANISOU 9281  O   ALA B 390    24281  26845  22415  -3454  -1745
-3780       O
ATOM   9282  CB  ALA B 390      -6.507 -42.362  41.853  1.00174.94
C
ANISOU 9282  CB  ALA B 390    21155  25322  19994  -3663  -1350
-3960       C
ATOM   9283  N   GLY B 391      -6.431 -45.846  42.566  1.00186.25
N
ANISOU 9283  N   GLY B 391    23558  25863  21345  -4266  -1717
-3639       N
ATOM   9284  CA  GLY B 391      -6.338 -47.122  41.875  1.00184.78
C
ANISOU 9284  CA  GLY B 391    23741  25278  21188  -4265  -1936
-3638       C
ATOM   9285  C   GLY B 391      -4.973 -47.770  41.867  1.00186.08
C
ANISOU 9285  C   GLY B 391    24279  25079  21344  -3930  -2131
-3507       C
ATOM   9286  O   GLY B 391      -4.733 -48.674  41.061  1.00190.86
O
ANISOU 9286  O   GLY B 391    25160  25371  21989  -3789  -2332
-3543       O
ATOM   9287  N   ILE B 392      -4.067 -47.321  42.735  1.00188.27
N
ANISOU 9287  N   ILE B 392    24565  25403  21564  -3780  -2086
-3372       N
ATOM   9288  CA  ILE B 392      -2.663 -47.719  42.650  1.00192.86
C
ANISOU 9288  CA  ILE B 392    25412  25724  22141  -3369  -2260
-3299       C
ATOM   9289  C   ILE B 392      -2.495 -49.225  42.839  1.00190.27
C
ANISOU 9289  C   ILE B 392    25631  24933  21731  -3491  -2518
-3195       C
ATOM   9290  O   ILE B 392      -1.769 -49.881  42.082  1.00175.86
O
ANISOU 9290  O   ILE B 392    24042  22841  19937  -3153  -2732
-3242       O
ATOM   9291  CB  ILE B 392      -1.823 -46.919  43.661  1.00203.29
C
ANISOU 9291  CB  ILE B 392    26618  27217  23404  -3235  -2150
-3181       C
ATOM   9292  CG1 ILE B 392      -1.692 -45.469  43.192  1.00182.42
C
```

```
ANISOU 9292  CG1 ILE B 392    23506  24967  20839  -2986  -1955
-3305       C
ATOM   9293  CG2 ILE B 392      -0.462 -47.557  43.871  1.00220.32
C
ANISOU 9293  CG2 ILE B 392    29110  29085  25515  -2901  -2355
-3096       C
ATOM   9294  CD1 ILE B 392      -0.698 -44.664  43.979  1.00169.63
C
ANISOU 9294  CD1 ILE B 392    21776  23504  19171  -2783  -1868
-3210       C
ATOM   9295  N   ARG B 393      -3.174 -49.795  43.835  1.00183.20
N
ANISOU 9295  N   ARG B 393    24962  23936  20710  -3980  -2509
-3060       N
ATOM   9296  CA  ARG B 393      -3.047 -51.213  44.186  1.00162.76
C
ANISOU 9296  CA  ARG B 393    22964  20882  17994  -4155  -2760
-2938       C
ATOM   9297  C   ARG B 393      -1.571 -51.557  44.414  1.00156.84
C
ANISOU 9297  C   ARG B 393    22514  19879  17200  -3697  -2965
-2864       C
ATOM   9298  O   ARG B 393      -0.988 -52.444  43.786  1.00154.82
O
ANISOU 9298  O   ARG B 393    22601  19288  16936  -3423  -3230
-2900       O
ATOM   9299  CB  ARG B 393      -3.689 -52.113  43.123  1.00152.26
C
ANISOU 9299  CB  ARG B 393    21824  19323  16705  -4253  -2915
-3045       C
ATOM   9300  CG  ARG B 393      -3.853 -53.571  43.551  1.00152.83
C
ANISOU 9300  CG  ARG B 393    22534  18924  16612  -4568  -3158
-2916       C
ATOM   9301  CD  ARG B 393      -4.583 -54.401  42.502  1.00144.06
C
ANISOU 9301  CD  ARG B 393    21588  17611  15538  -4706  -3297
-3024       C
ATOM   9302  NE  ARG B 393      -4.755 -55.786  42.937  1.00149.06
N
ANISOU 9302  NE  ARG B 393    22875  17777  15983  -5039  -3539
-2894       N
ATOM   9303  CZ  ARG B 393      -5.378 -56.725  42.232  1.00160.17
C
ANISOU 9303  CZ  ARG B 393    24563  18925  17368  -5241  -3700
-2947       C
ATOM   9304  NH1 ARG B 393      -5.489 -57.955  42.717  1.00155.21
N
ANISOU 9304  NH1 ARG B 393    24581  17856  16537  -5563  -3927
-2814       N
ATOM   9305  NH2 ARG B 393      -5.894 -56.438  41.045  1.00171.90
N
ANISOU 9305  NH2 ARG B 393    25712  20579  19025  -5128  -3645
-3132       N
```

```
ATOM   9306  N   GLU B 394      -0.974 -50.797  45.333  1.00151.46
N
ANISOU 9306  N   GLU B 394    21675  19389  16483  -3603  -2841
-2775       N
ATOM   9307  CA  GLU B 394       0.458 -50.857  45.606  1.00160.07
C
ANISOU 9307  CA  GLU B 394    22927  20353  17539  -3141  -2989
-2737       C
ATOM   9308  C   GLU B 394       0.902 -52.231  46.097  1.00172.03
C
ANISOU 9308  C   GLU B 394    25098  21370  18894  -3158  -3304
-2628       C
ATOM   9309  O   GLU B 394       0.196 -52.904  46.854  1.00170.89
O
ANISOU 9309  O   GLU B 394    25295  21022  18614  -3629  -3339
-2496       O
ATOM   9310  CB  GLU B 394       0.836 -49.789  46.633  1.00161.20
C
ANISOU 9310  CB  GLU B 394    22787  20802  17659  -3132  -2780
-2649       C
ATOM   9311  CG  GLU B 394       0.165 -49.959  47.984  1.00159.86
C
ANISOU 9311  CG  GLU B 394    22790  20599  17350  -3643  -2694
-2468       C
ATOM   9312  CD  GLU B 394       0.267 -48.717  48.843  1.00173.84
C
ANISOU 9312  CD  GLU B 394    24176  22751  19125  -3672  -2438
-2407       C
ATOM   9313  OE1 GLU B 394       0.947 -48.771  49.889  1.00176.02
O
ANISOU 9313  OE1 GLU B 394    24644  22936  19300  -3627  -2482
-2272       O
ATOM   9314  OE2 GLU B 394      -0.331 -47.687  48.469  1.00177.95
O
ANISOU 9314  OE2 GLU B 394    24217  23653  19742  -3726  -2208
-2501       O
ATOM   9315  N   VAL B 395       2.080 -52.651  45.639  1.00184.06
N
ANISOU 9315  N   VAL B 395    26813  22706  20418  -2638  -3543
-2698       N
ATOM   9316  CA  VAL B 395       2.622 -53.960  45.978  1.00191.76
C
ANISOU 9316  CA  VAL B 395    28438  23192  21229  -2545  -3894
-2633       C
ATOM   9317  C   VAL B 395       3.934 -53.872  46.754  1.00178.10
C
ANISOU 9317  C   VAL B 395    26832  21424  19413  -2158  -4013
-2597       C
ATOM   9318  O   VAL B 395       4.283 -54.831  47.464  1.00182.66
O
ANISOU 9318  O   VAL B 395    27978  21614  19812  -2172  -4274
-2502       O
ATOM   9319  CB  VAL B 395       2.801 -54.830  44.712  1.00211.82
C
```

```
ANISOU 9319  CB  VAL B 395    31206  25468  23808  -2257  -4160
-2775       C
ATOM   9320  CG1 VAL B 395       3.846 -54.232  43.780  1.00206.85
C
ANISOU 9320  CG1 VAL B 395    30214  25071  23309  -1643  -4163
-2962       C
ATOM   9321  CG2 VAL B 395       3.134 -56.280  45.066  1.00227.92
C
ANISOU 9321  CG2 VAL B 395    33996  26957  25647  -2235  -4550
-2703       C
ATOM   9322  N   HIS B 396       4.661 -52.756  46.686  1.00166.30
N
ANISOU 9322  N   HIS B 396    24850  20314  18021  -1830  -3835
-2674       N
ATOM   9323  CA  HIS B 396       5.869 -52.573  47.480  1.00165.16
C
ANISOU 9323  CA  HIS B 396    24765  20191  17798  -1491  -3918
-2651       C
ATOM   9324  C   HIS B 396       6.161 -51.084  47.617  1.00170.10
C
ANISOU 9324  C   HIS B 396    24774  21322  18535  -1394  -3597
-2681       C
ATOM   9325  O   HIS B 396       5.779 -50.284  46.758  1.00157.37
O
ANISOU 9325  O   HIS B 396    22716  20013  17065  -1391  -3389
-2782       O
ATOM   9326  CB  HIS B 396       7.062 -53.301  46.844  1.00156.18
C
ANISOU 9326  CB  HIS B 396    23869  18852  16622   -906  -4250
-2810       C
ATOM   9327  CG  HIS B 396       8.395 -52.853  47.359  1.00174.40
C
ANISOU 9327  CG  HIS B 396    26049  21325  18889   -467  -4290
-2869       C
ATOM   9328  ND1 HIS B 396       8.905 -53.271  48.570  1.00191.19
N
ANISOU 9328  ND1 HIS B 396    28551  23240  20854   -445  -4449
-2760       N
ATOM   9329  CD2 HIS B 396       9.321 -52.020  46.829  1.00175.23
C
ANISOU 9329  CD2 HIS B 396    25697  21798  19083    -48  -4190
-3034       C
ATOM   9330  CE1 HIS B 396      10.089 -52.718  48.761  1.00192.77
C
ANISOU 9330  CE1 HIS B 396    28508  23680  21056    -12  -4450
-2865       C
ATOM   9331  NE2 HIS B 396      10.365 -51.954  47.719  1.00188.34
N
ANISOU 9331  NE2 HIS B 396    27437  23482  20642    219  -4288
-3032       N
ATOM   9332  N   PHE B 397       6.843 -50.723  48.708  1.00177.22
N
ANISOU 9332  N   PHE B 397    25684  22294  19359  -1315  -3572
-2594       N
```

```
ATOM   9333  CA  PHE B 397       7.284 -49.352  48.936  1.00176.47
C
ANISOU 9333  CA  PHE B 397    25062  22648  19340  -1191  -3304
-2616       C
ATOM   9334  C   PHE B 397       8.676 -49.336  49.554  1.00165.06
C
ANISOU 9334  C   PHE B 397    23694  21209  17814   -777  -3443
-2646       C
ATOM   9335  O   PHE B 397       8.967 -50.101  50.478  1.00137.81
O
ANISOU 9335  O   PHE B 397    20689  17451  14221   -789  -3648
-2547       O
ATOM   9336  CB  PHE B 397       6.308 -48.598  49.849  1.00170.55
C
ANISOU 9336  CB  PHE B 397    24122  22091  18588  -1684  -3020
-2448       C
ATOM   9337  CG  PHE B 397       6.653 -47.147  50.037  1.00167.72
C
ANISOU 9337  CG  PHE B 397    23239  22187  18300  -1580  -2748
-2468       C
ATOM   9338  CD1 PHE B 397       6.312 -46.208  49.077  1.00184.88
C
ANISOU 9338  CD1 PHE B 397    24957  24688  20600  -1545  -2542
-2589       C
ATOM   9339  CD2 PHE B 397       7.327 -46.723  51.172  1.00150.84
C
ANISOU 9339  CD2 PHE B 397    21092  20131  16088  -1514  -2711
-2369       C
ATOM   9340  CE1 PHE B 397       6.631 -44.872  49.246  1.00169.39
C
ANISOU 9340  CE1 PHE B 397    22566  23120  18676  -1461  -2310
-2605       C
ATOM   9341  CE2 PHE B 397       7.650 -45.389  51.345  1.00137.91
C
ANISOU 9341  CE2 PHE B 397    18997  18900  14502  -1430  -2471
-2383       C
ATOM   9342  CZ  PHE B 397       7.301 -44.462  50.381  1.00142.41
C
ANISOU 9342  CZ  PHE B 397    19141  19786  15185  -1410  -2273
-2499       C
ATOM   9343  N   LEU B 398       9.529 -48.450  49.037  1.00194.45
N
ANISOU 9343  N   LEU B 398    26986  25285  21613   -419  -3333
-2793       N
ATOM   9344  CA  LEU B 398      10.897 -48.262  49.512  1.00198.54
C
ANISOU 9344  CA  LEU B 398    27463  25908  22065     -8  -3428
-2865       C
ATOM   9345  C   LEU B 398      10.991 -46.951  50.284  1.00204.93
C
ANISOU 9345  C   LEU B 398    27878  27087  22897   -136  -3134
-2776       C
ATOM   9346  O   LEU B 398      10.846 -45.876  49.681  1.00211.73
O
```

```
ANISOU 9346  O   LEU B 398    28283  28308  23857   -158  -2882
-2840       O
ATOM   9347  CB  LEU B 398      11.883 -48.274  48.339  1.00176.42
C
ANISOU 9347  CB  LEU B 398    24471  23257  19304    489  -3531
-3123       C
ATOM   9348  CG  LEU B 398      13.389 -48.097  48.582  1.00163.14
C
ANISOU 9348  CG  LEU B 398    22679  21755  17553    969  -3639
-3273       C
ATOM   9349  CD1 LEU B 398      13.815 -46.633  48.563  1.00170.17
C
ANISOU 9349  CD1 LEU B 398    23008  23146  18503    992  -3325
-3317       C
ATOM   9350  CD2 LEU B 398      13.824 -48.777  49.877  1.00154.37
C
ANISOU 9350  CD2 LEU B 398    21991  20365  16297   1024  -3863
-3171       C
ATOM   9351  N   PRO B 399      11.219 -46.970  51.595  1.00207.24
N
ANISOU 9351  N   PRO B 399    28350  27299  23094   -222  -3159
-2631       N
ATOM   9352  CA  PRO B 399      11.211 -45.729  52.374  1.00197.50
C
ANISOU 9352  CA  PRO B 399    26762  26400  21878   -373  -2881
-2530       C
ATOM   9353  C   PRO B 399      12.567 -45.034  52.415  1.00166.42
C
ANISOU 9353  C   PRO B 399    22537  22762  17932     39  -2861
-2662       C
ATOM   9354  O   PRO B 399      13.612 -45.610  52.105  1.00152.07
O
ANISOU 9354  O   PRO B 399    20835  20877  16068    456  -3091
-2827       O
ATOM   9355  CB  PRO B 399      10.816 -46.210  53.779  1.00226.79
C
ANISOU 9355  CB  PRO B 399    30854  29843  25475   -665  -2943
-2311       C
ATOM   9356  CG  PRO B 399      11.364 -47.596  53.844  1.00233.24
C
ANISOU 9356  CG  PRO B 399    32211  30229  26178   -429  -3318
-2359       C
ATOM   9357  CD  PRO B 399      11.168 -48.163  52.459  1.00219.34
C
ANISOU 9357  CD  PRO B 399    30475  28379  24486   -301  -3430
-2516       C
ATOM   9358  N   PHE B 400      12.514 -43.761  52.809  1.00153.69
N
ANISOU 9358  N   PHE B 400    20541  21501  16354    -92  -2583
-2599       N
ATOM   9359  CA  PHE B 400      13.678 -42.880  52.788  1.00149.10
C
ANISOU 9359  CA  PHE B 400    19616  21271  15764    216  -2501
-2720       C
```

```
ATOM   9360  C   PHE B 400      14.807 -43.394  53.672  1.00146.96
C
ANISOU 9360  C   PHE B 400    19563  20888  15387    519  -2720
-2747       C
ATOM   9361  O   PHE B 400      14.580 -43.895  54.778  1.00153.00
O
ANISOU 9361  O   PHE B 400    20664  21391  16079    378  -2821
-2583       O
ATOM   9362  CB  PHE B 400      13.282 -41.468  53.228  1.00155.49
C
ANISOU 9362  CB  PHE B 400    20059  22417  16603    -38  -2182
-2608       C
ATOM   9363  CG  PHE B 400      14.358 -40.441  53.009  1.00167.11
C
ANISOU 9363  CG  PHE B 400    21152  24280  18062    215  -2061
-2741       C
ATOM   9364  CD1 PHE B 400      14.469 -39.777  51.800  1.00166.15
C
ANISOU 9364  CD1 PHE B 400    20718  24422  17988    294  -1929
-2900       C
ATOM   9365  CD2 PHE B 400      15.262 -40.140  54.015  1.00184.48
C
ANISOU 9365  CD2 PHE B 400    23324  26582  20189    357  -2081
-2710       C
ATOM   9366  CE1 PHE B 400      15.462 -38.836  51.599  1.00180.72
C
ANISOU 9366  CE1 PHE B 400    22238  26632  19795    480  -1811
-3025       C
ATOM   9367  CE2 PHE B 400      16.256 -39.199  53.818  1.00193.68
C
ANISOU 9367  CE2 PHE B 400    24138  28123  21327    556  -1966
-2840       C
ATOM   9368  CZ  PHE B 400      16.354 -38.546  52.609  1.00193.75
C
ANISOU 9368  CZ  PHE B 400    23846  28398  21370    602  -1826
-2996       C
ATOM   9369  N   ASN B 401      16.036 -43.264  53.171  1.00145.50
N
ANISOU 9369  N   ASN B 401    19186  20916  15180    937  -2797
-2971       N
ATOM   9370  CA  ASN B 401      17.237 -43.662  53.892  1.00148.15
C
ANISOU 9370  CA  ASN B 401    19658  21220  15411   1295  -3011
-3060       C
ATOM   9371  C   ASN B 401      18.263 -42.541  53.729  1.00133.60
C
ANISOU 9371  C   ASN B 401    17330  19865  13566   1499  -2840
-3212       C
ATOM   9372  O   ASN B 401      18.589 -42.160  52.592  1.00126.82
O
ANISOU 9372  O   ASN B 401    16170  19272  12745   1631  -2754
-3400       O
ATOM   9373  CB  ASN B 401      17.757 -45.002  53.356  1.00167.74
C
```

```
ANISOU 9373  CB  ASN B 401    22484  23418  17832   1661  -3374
-3242       C
ATOM   9374  CG  ASN B 401      19.127 -45.377  53.902  1.00185.30
C
ANISOU 9374  CG  ASN B 401    24793  25674  19938   2123  -3619
-3413       C
ATOM   9375  OD1 ASN B 401      19.616 -44.798  54.872  1.00188.02
O
ANISOU 9375  OD1 ASN B 401    25027  26171  20240   2137  -3547
-3358       O
ATOM   9376  ND2 ASN B 401      19.746 -46.374  53.280  1.00188.45
N
ANISOU 9376  ND2 ASN B 401    25398  25929  20275   2522  -3928
-3636       N
ATOM   9377  N   PRO B 402      18.762 -41.971  54.836  1.00132.84
N
ANISOU 9377  N   PRO B 402    17154  19903  13417   1502  -2778
-3133       N
ATOM   9378  CA  PRO B 402      19.790 -40.917  54.751  1.00135.62
C
ANISOU 9378  CA  PRO B 402    17062  20723  13746   1676  -2623
-3280       C
ATOM   9379  C   PRO B 402      20.947 -41.179  53.796  1.00158.38
C
ANISOU 9379  C   PRO B 402    19762  23830  16587   2105  -2751
-3614       C
ATOM   9380  O   PRO B 402      21.465 -40.220  53.208  1.00156.70
O
ANISOU 9380  O   PRO B 402    19131  24037  16372   2130  -2550
-3748       O
ATOM   9381  CB  PRO B 402      20.277 -40.822  56.203  1.00147.72
C
ANISOU 9381  CB  PRO B 402    18709  22215  15203   1721  -2684
-3172       C
ATOM   9382  CG  PRO B 402      19.041 -41.091  56.993  1.00150.53
C
ANISOU 9382  CG  PRO B 402    19387  22224  15582   1336  -2655
-2874       C
ATOM   9383  CD  PRO B 402      18.328 -42.194  56.224  1.00145.23
C
ANISOU 9383  CD  PRO B 402    19033  21209  14941   1296  -2822
-2891       C
ATOM   9384  N   VAL B 403      21.384 -42.430  53.623  1.00179.90
N
ANISOU 9384  N   VAL B 403    22795  26301  19259   2440  -3084
-3763       N
ATOM   9385  CA  VAL B 403      22.516 -42.698  52.743  1.00183.39
C
ANISOU 9385  CA  VAL B 403    23045  26991  19645   2876  -3219
-4108       C
ATOM   9386  C   VAL B 403      22.077 -43.064  51.333  1.00163.15
C
ANISOU 9386  C   VAL B 403    20462  24386  17143   2885  -3225
-4218       C
```

```
ATOM   9387  O   VAL B 403      22.931 -43.257  50.456  1.00173.03
O
ANISOU 9387  O   VAL B 403    21528  25867  18350   3222  -3313
-4512       O
ATOM   9388  CB  VAL B 403      23.428 -43.805  53.311  1.00198.91
C
ANISOU 9388  CB  VAL B 403    25323  28762  21490   3329  -3611
-4273       C
ATOM   9389  CG1 VAL B 403      23.969 -43.397  54.675  1.00197.80
C
ANISOU 9389  CG1 VAL B 403    25176  28694  21285   3358  -3607
-4195       C
ATOM   9390  CG2 VAL B 403      22.684 -45.125  53.392  1.00211.62
C
ANISOU 9390  CG2 VAL B 403    27515  29801  23089   3321  -3898
-4162       C
ATOM   9391  N   ASP B 404      20.773 -43.166  51.086  1.00144.14
N
ANISOU 9391  N   ASP B 404    18227  21709  14830   2527  -3135
-4003       N
ATOM   9392  CA  ASP B 404      20.228 -43.399  49.753  1.00156.15
C
ANISOU 9392  CA  ASP B 404    19715  23191  16423   2489  -3109
-4083       C
ATOM   9393  C   ASP B 404      19.528 -42.174  49.193  1.00145.71
C
ANISOU 9393  C   ASP B 404    18049  22127  15188   2134  -2746
-3993       C
ATOM   9394  O   ASP B 404      19.661 -41.879  48.003  1.00141.23
O
ANISOU 9394  O   ASP B 404    17250  21767  14644   2201  -2654
-4160       O
ATOM   9395  CB  ASP B 404      19.253 -44.580  49.779  1.00159.85
C
ANISOU 9395  CB  ASP B 404    20680  23136  16921   2373  -3324
-3943       C
ATOM   9396  CG  ASP B 404      19.946 -45.903  50.046  1.00167.26
C
ANISOU 9396  CG  ASP B 404    22021  23781  17750   2774  -3734
-4075       C
ATOM   9397  OD1 ASP B 404      21.141 -46.029  49.702  1.00165.20
O
ANISOU 9397  OD1 ASP B 404    21600  23754  17414   3211  -3869
-4355       O
ATOM   9398  OD2 ASP B 404      19.299 -46.815  50.600  1.00182.05
O
ANISOU 9398  OD2 ASP B 404    24381  25196  19594   2650  -3929
-3912       O
ATOM   9399  N   LYS B 405      18.769 -41.472  50.037  1.00124.34
N
ANISOU 9399  N   LYS B 405    15329  19401  12515   1765  -2551
-3739       N
ATOM   9400  CA  LYS B 405      18.042 -40.258  49.665  1.00135.78
C
```

```
ANISOU 9400  CA  LYS B 405    16490  21076  14024   1431  -2227
-3643       C
ATOM   9401  C   LYS B 405      16.997 -40.515  48.580  1.00115.55
C
ANISOU 9401  C   LYS B 405    13991  18361  11553   1273  -2196
-3640       C
ATOM   9402  O   LYS B 405      16.906 -39.785  47.592  1.00113.34
O
ANISOU 9402  O   LYS B 405    13452  18316  11297   1229  -2022
-3740       O
ATOM   9403  CB  LYS B 405      19.007 -39.145  49.245  1.00135.26
C
ANISOU 9403  CB  LYS B 405    16006  21488  13900   1541  -2039
-3812       C
ATOM   9404  CG  LYS B 405      20.062 -38.818  50.290  1.00124.56
C
ANISOU 9404  CG  LYS B 405    14549  20326  12453   1690  -2057
-3834       C
ATOM   9405  CD  LYS B 405      21.022 -37.746  49.797  1.00113.69
C
ANISOU 9405  CD  LYS B 405    12759  19438  10998   1764  -1867
-4020       C
ATOM   9406  CE  LYS B 405      21.875 -37.207  50.935  1.00115.75
C
ANISOU 9406  CE  LYS B 405    12895  19909  11177   1820  -1834
-3999       C
ATOM   9407  NZ  LYS B 405      22.730 -38.266  51.536  1.00128.37
N
ANISOU 9407  NZ  LYS B 405    14670  21379  12725   2189  -2133
-4119       N
ATOM   9408  N   ARG B 406      16.208 -41.574  48.760  1.00131.31
N
ANISOU 9408  N   ARG B 406    16353  19952  13587   1180  -2373
-3531       N
ATOM   9409  CA  ARG B 406      15.281 -41.987  47.717  1.00127.75
C
ANISOU 9409  CA  ARG B 406    15987  19332  13218   1064  -2385
-3553       C
ATOM   9410  C   ARG B 406      14.069 -42.652  48.356  1.00128.44
C
ANISOU 9410  C   ARG B 406    16412  19051  13339    740  -2450
-3333       C
ATOM   9411  O   ARG B 406      14.178 -43.307  49.395  1.00144.22
O
ANISOU 9411  O   ARG B 406    18707  20814  15276    730  -2607
-3219       O
ATOM   9412  CB  ARG B 406      15.960 -42.939  46.720  1.00156.22
C
ANISOU 9412  CB  ARG B 406    19700  22847  16811   1441  -2624
-3786       C
ATOM   9413  CG  ARG B 406      15.032 -43.536  45.669  1.00170.11
C
ANISOU 9413  CG  ARG B 406    21602  24380  18654   1349  -2681
-3808       C
```

ATOM   9414 CD ARG B 406   15.800 -44.302 44.599 1.00152.49
C
ANISOU 9414 CD ARG B 406   19415 22125 16401   1752 -2892
-4060   C
ATOM   9415 NE ARG B 406   16.374 -45.544 45.111 1.00156.21
N
ANISOU 9415 NE ARG B 406   20258 22305 16791   2028 -3235
-4099   N
ATOM   9416 CZ ARG B 406   17.655 -45.704 45.429 1.00152.44
C
ANISOU 9416 CZ ARG B 406   19735 21975 16211   2405 -3377
-4263   C
ATOM   9417 NH1 ARG B 406   18.490 -44.675 45.373 1.00152.02
N
ANISOU 9417 NH1 ARG B 406   19263 22370 16125   2502 -3181
-4382   N
ATOM   9418 NH2 ARG B 406   18.085 -46.875 45.879 1.00153.39
N
ANISOU 9418 NH2 ARG B 406   20245 21793 16244   2669 -3722
-4305   N
ATOM   9419 N   THR B 407   12.913 -42.477 47.719 1.00137.73
N
ANISOU 9419 N   THR B 407   17546 20187 14599   468 -2329
-3287   N
ATOM   9420 CA THR B 407   11.728 -43.285 47.960 1.00149.54
C
ANISOU 9420 CA THR B 407   19354 21340 16123   169 -2411
-3142   C
ATOM   9421 C   THR B 407   11.302 -43.923 46.645 1.00181.21
C
ANISOU 9421 C   THR B 407   23443 25207 20203   234 -2511
-3273   C
ATOM   9422 O   THR B 407   11.578 -43.383 45.571 1.00209.69
O
ANISOU 9422 O   THR B 407   26784 29036 23854   396 -2420
-3436   O
ATOM   9423 CB THR B 407   10.576 -42.449 48.537 1.00137.45
C
ANISOU 9423 CB THR B 407   17677 19924 14622   -264 -2163
-2968   C
ATOM   9424 OG1 THR B 407   10.233 -41.402 47.620 1.00115.61
O
ANISOU 9424 OG1 THR B 407   14556 17451 11919   -303 -1948
-3063   O
ATOM   9425 CG2 THR B 407   10.978 -41.835 49.867 1.00155.21
C
ANISOU 9425 CG2 THR B 407   19866 22305 16802   -328 -2071
-2830   C
ATOM   9426 N   ALA B 408   10.635 -45.074 46.718 1.00182.33
N
ANISOU 9426 N   ALA B 408   23968 24969 20341   98 -2700
-3202   N
ATOM   9427 CA ALA B 408   10.089 -45.657 45.500 1.00178.94
C

```
ANISOU 9427  CA  ALA B 408    23617  24390  19982    115  -2786 
-3309       C
ATOM   9428  C   ALA B 408       8.844 -46.477  45.806  1.00175.64           
C
ANISOU 9428  C   ALA B 408    23527  23643  19567   -271  -2859 
-3164       C
ATOM   9429  O   ALA B 408       8.738 -47.111  46.859  1.00187.47           
O
ANISOU 9429  O   ALA B 408    25355  24898  20976   -427  -2976 
-3015       O
ATOM   9430  CB  ALA B 408      11.127 -46.533  44.787  1.00183.91           
C
ANISOU 9430  CB  ALA B 408    24415  24887  20576    572  -3060 
-3495       C
ATOM   9431  N   LEU B 409       7.904 -46.454  44.862  1.00163.54           
N
ANISOU 9431  N   LEU B 409    21905  22108  18125   -433  -2789 
-3218       N
ATOM   9432  CA  LEU B 409       6.651 -47.195  44.943  1.00154.12           
C
ANISOU 9432  CA  LEU B 409    20970  20649  16939   -820  -2839 
-3119       C
ATOM   9433  C   LEU B 409       6.578 -48.154  43.763  1.00162.86           
C
ANISOU 9433  C   LEU B 409    22286  21508  18087   -652  -3053 
-3250       C
ATOM   9434  O   LEU B 409       6.867 -47.760  42.629  1.00191.26           
O
ANISOU 9434  O   LEU B 409    25639  25269  21763   -397  -3011 
-3419       O
ATOM   9435  CB  LEU B 409       5.446 -46.251  44.942  1.00142.23           
C
ANISOU 9435  CB  LEU B 409    19142  19397  15501  -1200  -2554 
-3078       C
ATOM   9436  CG  LEU B 409       4.068 -46.916  44.987  1.00133.56           
C
ANISOU 9436  CG  LEU B 409    18235  18103  14407  -1641  -2571 
-3008       C
ATOM   9437  CD1 LEU B 409       3.752 -47.403  46.394  1.00133.78           
C
ANISOU 9437  CD1 LEU B 409    18555  17960  14316  -1982  -2602 
-2807       C
ATOM   9438  CD2 LEU B 409       2.991 -45.964  44.486  1.00138.18           
C
ANISOU 9438  CD2 LEU B 409    18433  18989  15081  -1866  -2325 
-3075       C
ATOM   9439  N   THR B 410       6.190 -49.401  44.019  1.00147.80           
N
ANISOU 9439  N   THR B 410    20847  19199  16111   -805  -3284 
-3173       N
ATOM   9440  CA  THR B 410       5.935 -50.376  42.965  1.00168.41           
C
ANISOU 9440  CA  THR B 410    23702  21536  18750   -711  -3496 
-3274       C
```

```
ATOM   9441  C   THR B 410       4.472 -50.798  43.000  1.00175.10
C
ANISOU 9441  C   THR B 410    24694  22224  19611  -1225  -3452
-3181       C
ATOM   9442  O   THR B 410       3.938 -51.097  44.073  1.00162.53
O
ANISOU 9442  O   THR B 410    23327  20503  17924  -1606  -3441
-3012       O
ATOM   9443  CB  THR B 410       6.842 -51.603  43.113  1.00157.90
C
ANISOU 9443  CB  THR B 410    22861  19835  17298   -396  -3864
-3295       C
ATOM   9444  OG1 THR B 410       8.195 -51.177  43.319  1.00156.96
O
ANISOU 9444  OG1 THR B 410    22586  19909  17144     44  -3892
-3384       O
ATOM   9445  CG2 THR B 410       6.776 -52.469  41.862  1.00156.27
C
ANISOU 9445  CG2 THR B 410    22849  19398  17128   -193  -4086
-3436       C
ATOM   9446  N   TYR B 411       3.820 -50.801  41.836  1.00159.07
N
ANISOU 9446  N   TYR B 411    22527  20222  17690  -1249  -3421
-3300       N
ATOM   9447  CA  TYR B 411       2.369 -50.932  41.796  1.00158.42
C
ANISOU 9447  CA  TYR B 411    22445  20111  17638  -1745  -3318
-3252       C
ATOM   9448  C   TYR B 411       1.922 -51.565  40.483  1.00165.33
C
ANISOU 9448  C   TYR B 411    23411  20816  18592  -1674  -3452
-3387       C
ATOM   9449  O   TYR B 411       2.673 -51.627  39.506  1.00174.66
O
ANISOU 9449  O   TYR B 411    24548  21986  19831  -1234  -3561
-3531       O
ATOM   9450  CB  TYR B 411       1.679 -49.578  42.007  1.00159.86
C
ANISOU 9450  CB  TYR B 411    22121  20726  17891  -1973  -2974
-3256       C
ATOM   9451  CG  TYR B 411       1.894 -48.565  40.904  1.00155.68
C
ANISOU 9451  CG  TYR B 411    21160  20504  17487  -1671  -2829
-3435       C
ATOM   9452  CD1 TYR B 411       0.879 -48.262  40.006  1.00160.73
C
ANISOU 9452  CD1 TYR B 411    21586  21259  18224  -1812  -2728
-3553       C
ATOM   9453  CD2 TYR B 411       3.101 -47.890  40.780  1.00146.35
C
ANISOU 9453  CD2 TYR B 411    19792  19507  16309  -1261  -2794
-3494       C
ATOM   9454  CE1 TYR B 411       1.064 -47.320  39.010  1.00176.24
C
```

ANISOU 9454 CE1 TYR B 411   23202 23480 20280 -1543 -2604
-3715   C
ATOM   9455 CE2 TYR B 411   3.299 -46.956 39.781 1.00152.98
C
ANISOU 9455 CE2 TYR B 411   20275 20616 17232 -1024 -2658
-3654   C
ATOM   9456 CZ TYR B 411   2.276 -46.671 38.902 1.00161.39
C
ANISOU 9456 CZ TYR B 411   21172 21760 18387 -1163 -2567
-3758   C
ATOM   9457 OH TYR B 411   2.474 -45.750 37.897 1.00147.05
O
ANISOU 9457 OH TYR B 411   19054 20183 16634   -928 -2446
-3918   O
ATOM   9458 N   ILE B 412   0.676 -52.038 40.486 1.00164.74
N
ANISOU 9458 N   ILE B 412   23458 20623 18512 -2128 -3438
-3346   N
ATOM   9459 CA ILE B 412   0.108 -52.876 39.432 1.00169.74
C
ANISOU 9459 CA ILE B 412   24284 21017 19194 -2158 -3601
-3440   C
ATOM   9460 C   ILE B 412   -1.067 -52.130 38.812 1.00175.12
C
ANISOU 9460 C   ILE B 412   24549 21991 19998 -2404 -3364
-3547   C
ATOM   9461 O   ILE B 412   -1.992 -51.724 39.527 1.00167.79
O
ANISOU 9461 O   ILE B 412   23462 21248 19042 -2845 -3172
-3483   O
ATOM   9462 CB ILE B 412   -0.342 -54.247 39.964 1.00158.46
C
ANISOU 9462 CB ILE B 412   23437 19150 17620 -2502 -3834
-3311   C
ATOM   9463 CG1 ILE B 412   0.757 -54.887 40.814 1.00167.22
C
ANISOU 9463 CG1 ILE B 412   24980 19983 18574 -2290 -4063
-3197   C
ATOM   9464 CG2 ILE B 412   -0.737 -55.162 38.812 1.00153.69
C
ANISOU 9464 CG2 ILE B 412   23069 18268 17057 -2457 -4043
-3414   C
ATOM   9465 CD1 ILE B 412   0.376 -56.241 41.377 1.00159.92
C
ANISOU 9465 CD1 ILE B 412   24708 18585 17468 -2624 -4318
-3064   C
ATOM   9466 N   ASP B 413   -1.035 -51.945 37.493 1.00176.33
N
ANISOU 9466 N   ASP B 413   24525 22196 20275 -2112 -3384
-3723   N
ATOM   9467 CA ASP B 413   -2.051 -51.136 36.835 1.00151.09
C
ANISOU 9467 CA ASP B 413   20923 19291 17193 -2264 -3174
-3855   C

```
ATOM   9468  C   ASP B 413      -2.304 -51.674  35.431  1.00130.02
C
ANISOU 9468  C   ASP B 413    18328  16453  14620  -2087  -3321
-4012       C
ATOM   9469  O   ASP B 413      -1.623 -52.587  34.956  1.00131.53
O
ANISOU 9469  O   ASP B 413    18853  16329  14792  -1817  -3574
-4020       O
ATOM   9470  CB  ASP B 413      -1.637 -49.658  36.793  1.00154.48
C
ANISOU 9470  CB  ASP B 413    20886  20133  17677  -2035  -2932
-3924       C
ATOM   9471  CG  ASP B 413      -2.809 -48.727  36.539  1.00147.21
C
ANISOU 9471  CG  ASP B 413    19573  19539  16823  -2274  -2704
-4033       C
ATOM   9472  OD1 ASP B 413      -2.579 -47.510  36.371  1.00142.05
O
ANISOU 9472  OD1 ASP B 413    18571  19199  16201  -2089  -2526
-4108       O
ATOM   9473  OD2 ASP B 413      -3.960 -49.212  36.510  1.00144.83
O
ANISOU 9473  OD2 ASP B 413    19321  19182  16524  -2646  -2713
-4055       O
ATOM   9474  N   GLY B 414      -3.297 -51.080  34.771  1.00139.45
N
ANISOU 9474  N   GLY B 414    19205  17866  15911  -2224  -3170
-4150       N
ATOM   9475  CA  GLY B 414      -3.730 -51.528  33.455  1.00147.99
C
ANISOU 9475  CA  GLY B 414    20330  18810  17092  -2108  -3288
-4307       C
ATOM   9476  C   GLY B 414      -4.205 -52.967  33.464  1.00162.90
C
ANISOU 9476  C   GLY B 414    22670  20301  18925  -2370  -3524
-4241       C
ATOM   9477  O   GLY B 414      -5.091 -53.350  34.236  1.00160.55
O
ANISOU 9477  O   GLY B 414    22476  19974  18551  -2872  -3488
-4153       O
ATOM   9478  N   SER B 415      -3.608 -53.783  32.597  1.00173.61
N
ANISOU 9478  N   SER B 415    24312  21349  20303  -2037  -3773
-4290       N
ATOM   9479  CA  SER B 415      -3.965 -55.189  32.464  1.00169.59
C
ANISOU 9479  CA  SER B 415    24287  20419  19731  -2227  -4039
-4237       C
ATOM   9480  C   SER B 415      -3.125 -56.092  33.356  1.00174.47
C
ANISOU 9480  C   SER B 415    25395  20710  20186  -2193  -4261
-4061       C
ATOM   9481  O   SER B 415      -3.130 -57.312  33.166  1.00172.57
O
```

```
ANISOU 9481  O   SER B 415    25641  20062  19867  -2228  -4543 
-4021       O
ATOM   9482  CB  SER B 415      -3.839 -55.634  31.004  1.00141.57 
C
ANISOU 9482  CB  SER B 415    20815  16692  16284  -1875  -4217 
-4395       C
ATOM   9483  OG  SER B 415      -4.697 -54.882  30.164  1.00135.64 
O
ANISOU 9483  OG  SER B 415    19662  16203  15672  -1921  -4039 
-4566       O
ATOM   9484  N   GLY B 416      -2.410 -55.520  34.320  1.00169.60 
N
ANISOU 9484  N   GLY B 416    24682  20252  19505  -2115  -4154 
-3963       N
ATOM   9485  CA  GLY B 416      -1.597 -56.289  35.237  1.00158.20 
C
ANISOU 9485  CA  GLY B 416    23694  18520  17897  -2062  -4362 
-3810       C
ATOM   9486  C   GLY B 416      -0.120 -56.012  35.055  1.00139.58 
C
ANISOU 9486  C   GLY B 416    21284  16209  15541  -1456  -4447 
-3865       C
ATOM   9487  O   GLY B 416       0.724 -56.870  35.334  1.00141.77 
O
ANISOU 9487  O   GLY B 416    21989  16182  15697  -1220  -4725 
-3817       O
ATOM   9488  N   ASN B 417       0.202 -54.811  34.585  1.00146.09 
N
ANISOU 9488  N   ASN B 417    21596  17425  16486  -1201  -4216 
-3980       N
ATOM   9489  CA  ASN B 417       1.589 -54.405  34.429  1.00152.55 
C
ANISOU 9489  CA  ASN B 417    22294  18369  17297   -671  -4248 
-4051       C
ATOM   9490  C   ASN B 417       2.105 -53.880  35.759  1.00134.21 
C
ANISOU 9490  C   ASN B 417    19907  16204  14885   -748  -4129 
-3917       C
ATOM   9491  O   ASN B 417       1.393 -53.178  36.481  1.00133.32 
O
ANISOU 9491  O   ASN B 417    19569  16305  14780  -1127  -3885 
-3828       O
ATOM   9492  CB  ASN B 417       1.728 -53.333  33.349  1.00161.29 
C
ANISOU 9492  CB  ASN B 417    22917  19820  18544   -401  -4049 
-4230       C
ATOM   9493  CG  ASN B 417       1.498 -53.876  31.951  1.00146.43 
C
ANISOU 9493  CG  ASN B 417    21118  17771  16746   -205  -4200 
-4382       C
ATOM   9494  OD1 ASN B 417       1.457 -55.089  31.742  1.00147.76 
O
ANISOU 9494  OD1 ASN B 417    21715  17564  16865   -181  -4485 
-4364       O
```

```
ATOM   9495  ND2 ASN B 417       1.354 -52.977  30.984  1.00129.07
N
ANISOU 9495  ND2 ASN B 417    18537  15838  14663    -59  -4021
-4534       N
ATOM   9496  N   TRP B 418       3.350 -54.212  36.080  1.00142.36
N
ANISOU 9496  N   TRP B 418    21123  17143  15823   -369  -4307
-3918       N
ATOM   9497  CA  TRP B 418       4.012 -53.639  37.243  1.00158.75
C
ANISOU 9497  CA  TRP B 418    23102  19395  17822   -356  -4199
-3820       C
ATOM   9498  C   TRP B 418       4.714 -52.342  36.864  1.00145.91
C
ANISOU 9498  C   TRP B 418    20954  18211  16275    -61  -3963
-3939       C
ATOM   9499  O   TRP B 418       5.584 -52.329  35.986  1.00130.91
O
ANISOU 9499  O   TRP B 418    18966  16377  14398    384  -4048
-4104       O
ATOM   9500  CB  TRP B 418       5.017 -54.620  37.844  1.00161.24
C
ANISOU 9500  CB  TRP B 418    23881  19405  17980    -88  -4519
-3780       C
ATOM   9501  CG  TRP B 418       4.419 -55.728  38.647  1.00155.05
C
ANISOU 9501  CG  TRP B 418    23655  18198  17059   -451  -4722
-3611       C
ATOM   9502  CD1 TRP B 418       3.190 -56.296  38.483  1.00180.97
C
ANISOU 9502  CD1 TRP B 418    27145  21268  20346   -899  -4740
-3543       C
ATOM   9503  CD2 TRP B 418       5.021 -56.388  39.766  1.00158.00
C
ANISOU 9503  CD2 TRP B 418    24470  18312  17250   -420  -4934
-3495       C
ATOM   9504  NE1 TRP B 418       2.996 -57.282  39.421  1.00169.16
N
ANISOU 9504  NE1 TRP B 418    26213  19391  18671  -1176  -4946
-3384       N
ATOM   9505  CE2 TRP B 418       4.105 -57.356  40.222  1.00168.40
C
ANISOU 9505  CE2 TRP B 418    26285  19243  18455   -878  -5075
-3349       C
ATOM   9506  CE3 TRP B 418       6.249 -56.259  40.423  1.00165.44
C
ANISOU 9506  CE3 TRP B 418    25445  19313  18101    -50  -5026
-3512       C
ATOM   9507  CZ2 TRP B 418       4.378 -58.190  41.305  1.00184.17
C
ANISOU 9507  CZ2 TRP B 418    28846  20886  20244   -980  -5308
-3209       C
ATOM   9508  CZ3 TRP B 418       6.517 -57.086  41.497  1.00190.85
C
```

```
ANISOU 9508  CZ3 TRP B 418    29202  22187  21126   -118  -5266
-3384       C
ATOM   9509  CH2 TRP B 418       5.587 -58.040  41.928  1.00191.10
C
ANISOU 9509  CH2 TRP B 418    29761  21810  21040   -580  -5407
-3229       C
ATOM   9510  N   HIS B 419       4.331 -51.257  37.528  1.00165.51
N
ANISOU 9510  N   HIS B 419    23104  20999  18782   -321  -3669
-3861       N
ATOM   9511  CA  HIS B 419       4.867 -49.931  37.271  1.00166.82
C
ANISOU 9511  CA  HIS B 419    22799  21584  19001   -125  -3425
-3952       C
ATOM   9512  C   HIS B 419       5.578 -49.471  38.535  1.00150.00
C
ANISOU 9512  C   HIS B 419    20621  19594  16779   -122  -3350
-3837       C
ATOM   9513  O   HIS B 419       5.207 -49.867  39.644  1.00136.77
O
ANISOU 9513  O   HIS B 419    19175  17762  15030   -414  -3386
-3668       O
ATOM   9514  CB  HIS B 419       3.758 -48.942  36.892  1.00153.53
C
ANISOU 9514  CB  HIS B 419    20764  20154  17418   -411  -3150
-3980       C
ATOM   9515  CG  HIS B 419       3.053 -49.277  35.615  1.00136.23
C
ANISOU 9515  CG  HIS B 419    18581  17856  15323   -399  -3208
-4111       C
ATOM   9516  ND1 HIS B 419       3.367 -48.686  34.411  1.00139.10
N
ANISOU 9516  ND1 HIS B 419    18702  18392  15758   -105  -3141
-4292       N
ATOM   9517  CD2 HIS B 419       2.043 -50.142  35.355  1.00124.73
C
ANISOU 9517  CD2 HIS B 419    17358  16139  13896   -657  -3326
-4092       C
ATOM   9518  CE1 HIS B 419       2.583 -49.172  33.465  1.00144.50
C
ANISOU 9518  CE1 HIS B 419    19466  18917  16521   -159  -3221
-4377       C
ATOM   9519  NE2 HIS B 419       1.770 -50.058  34.012  1.00130.51
N
ANISOU 9519  NE2 HIS B 419    17973  16887  14728   -493  -3334
-4261       N
ATOM   9520  N   ARG B 420       6.608 -48.644  38.380  1.00153.26
N
ANISOU 9520  N   ARG B 420    20746  20302  17185    194  -3247
-3933       N
ATOM   9521  CA  ARG B 420       7.356 -48.205  39.548  1.00153.26
C
ANISOU 9521  CA  ARG B 420    20691  20443  17098    226  -3187
-3838       C
```

```
ATOM   9522  C   ARG B 420       7.782 -46.752  39.382  1.00165.10
C
ANISOU 9522  C   ARG B 420    21727  22382  18621    312  -2909
-3908       C
ATOM   9523  O   ARG B 420       8.255 -46.353  38.314  1.00179.19
O
ANISOU 9523  O   ARG B 420    23310  24336  20439    572  -2866
-4082       O
ATOM   9524  CB  ARG B 420       8.559 -49.132  39.791  1.00153.40
C
ANISOU 9524  CB  ARG B 420    21003  20268  17013    605  -3478
-3887       C
ATOM   9525  CG  ARG B 420       9.450 -48.750  40.957  1.00157.08
C
ANISOU 9525  CG  ARG B 420    21425  20874  17384    697  -3449
-3819       C
ATOM   9526  CD  ARG B 420      10.601 -49.738  41.112  1.00160.23
C
ANISOU 9526  CD  ARG B 420    22131  21076  17672   1112  -3775
-3908       C
ATOM   9527  NE  ARG B 420      10.145 -51.073  41.481  1.00161.35
N
ANISOU 9527  NE  ARG B 420    22822  20740  17743   1001  -4065
-3808       N
ATOM   9528  CZ  ARG B 420      10.867 -52.181  41.357  1.00164.46
C
ANISOU 9528  CZ  ARG B 420    23594  20857  18036   1353  -4418
-3902       C
ATOM   9529  NH1 ARG B 420      11.954 -52.188  40.600  1.00182.40
N
ANISOU 9529  NH1 ARG B 420    25711  23298  20294   1849  -4523
-4133       N
ATOM   9530  NH2 ARG B 420      10.360 -53.336  41.768  1.00144.44
N
ANISOU 9530  NH2 ARG B 420    21603  17865  15414   1189  -4673
-3788       N
ATOM   9531  N   VAL B 421       7.612 -45.967  40.449  1.00162.27
N
ANISOU 9531  N   VAL B 421    21224  22202  18231     81  -2724
-3770       N
ATOM   9532  CA  VAL B 421       7.908 -44.540  40.456  1.00154.81
C
ANISOU 9532  CA  VAL B 421    19881  21654  17285    100  -2460
-3806       C
ATOM   9533  C   VAL B 421       8.701 -44.216  41.715  1.00147.38
C
ANISOU 9533  C   VAL B 421    18920  20824  16254    130  -2428
-3698       C
ATOM   9534  O   VAL B 421       8.629 -44.926  42.720  1.00156.43
O
ANISOU 9534  O   VAL B 421    20338  21747  17350     14  -2554
-3558       O
ATOM   9535  CB  VAL B 421       6.632 -43.673  40.386  1.00164.70
C
```

```
ANISOU 9535  CB  VAL B 421    20926  23056  18596   -249  -2233
-3761       C
ATOM   9536  CG1 VAL B 421       5.999 -43.771  39.004  1.00181.13
C
ANISOU 9536  CG1 VAL B 421    22965  25094  20764   -213  -2244
-3910       C
ATOM   9537  CG2 VAL B 421       5.647 -44.094  41.470  1.00164.90
C
ANISOU 9537  CG2 VAL B 421    21123  22923  18606   -643  -2237
-3575       C
ATOM   9538  N   SER B 422       9.467 -43.127  41.652  1.00137.31
N
ANISOU 9538  N   SER B 422    17337  19889  14946    276  -2260
-3767       N
ATOM   9539  CA  SER B 422      10.440 -42.849  42.698  1.00130.32
C
ANISOU 9539  CA  SER B 422    16414  19128  13972    382  -2252
-3707       C
ATOM   9540  C   SER B 422      10.657 -41.349  42.853  1.00124.37
C
ANISOU 9540  C   SER B 422    15302  18762  13189    316  -1979
-3708       C
ATOM   9541  O   SER B 422      10.574 -40.588  41.885  1.00112.58
O
ANISOU 9541  O   SER B 422    13594  17468  11715    347  -1842
-3828       O
ATOM   9542  CB  SER B 422      11.778 -43.539  42.401  1.00129.30
C
ANISOU 9542  CB  SER B 422    16374  18968  13785    809  -2463
-3863       C
ATOM   9543  OG  SER B 422      12.749 -43.219  43.381  1.00125.74
O
ANISOU 9543  OG  SER B 422    15858  18671  13247    929  -2454
-3833       O
ATOM   9544  N   LYS B 423      10.937 -40.942  44.092  1.00162.78
N
ANISOU 9544  N   LYS B 423    20138  23715  17994    224  -1915
-3571       N
ATOM   9545  CA  LYS B 423      11.414 -39.607  44.426  1.00150.27
C
ANISOU 9545  CA  LYS B 423    18259  22485  16352    206  -1698
-3565       C
ATOM   9546  C   LYS B 423      12.794 -39.711  45.058  1.00140.02
C
ANISOU 9546  C   LYS B 423    16947  21285  14969    465  -1778
-3601       C
ATOM   9547  O   LYS B 423      13.110 -40.703  45.722  1.00141.23
O
ANISOU 9547  O   LYS B 423    17352  21207  15104    564  -1981
-3552       O
ATOM   9548  CB  LYS B 423      10.492 -38.900  45.425  1.00139.70
C
ANISOU 9548  CB  LYS B 423    16868  21203  15010   -141  -1536
-3370       C
```

```
ATOM   9549  CG  LYS B 423       9.123 -38.512  44.924  1.00148.85
C
ANISOU 9549  CG  LYS B 423    17963  22364  16229   -403  -1422
-3357       C
ATOM   9550  CD  LYS B 423       8.435 -37.678  45.990  1.00148.97
C
ANISOU 9550  CD  LYS B 423    17878  22515  16209   -689  -1260
-3196       C
ATOM   9551  CE  LYS B 423       8.323 -38.455  47.293  1.00147.41
C
ANISOU 9551  CE  LYS B 423    17901  22115  15992   -832  -1357
-3016       C
ATOM   9552  NZ  LYS B 423       7.633 -37.683  48.361  1.00182.68
N
ANISOU 9552  NZ  LYS B 423    22267  26724  20419  -1115  -1199
-2861       N
ATOM   9553  N   GLY B 424      13.616 -38.687  44.863  1.00127.89
N
ANISOU 9553  N   GLY B 424    15133  20092  13366    568  -1626
-3696       N
ATOM   9554  CA  GLY B 424      14.814 -38.583  45.674  1.00138.60
C
ANISOU 9554  CA  GLY B 424    16430  21596  14636    750  -1662
-3713       C
ATOM   9555  C   GLY B 424      15.591 -37.320  45.381  1.00125.70
C
ANISOU 9555  C   GLY B 424    14473  20371  12917    789  -1457
-3818       C
ATOM   9556  O   GLY B 424      15.180 -36.473  44.580  1.00120.10
O
ANISOU 9556  O   GLY B 424    13615  19815  12204    663  -1286
-3864       O
ATOM   9557  N   ALA B 425      16.727 -37.207  46.065  1.00111.53
N
ANISOU 9557  N   ALA B 425    12592  18747  11039    958  -1486
-3861       N
ATOM   9558  CA  ALA B 425      17.671 -36.134  45.795  1.00114.46
C
ANISOU 9558  CA  ALA B 425    12664  19523  11302   1010  -1316
-3991       C
ATOM   9559  C   ALA B 425      18.161 -36.236  44.352  1.00126.12
C
ANISOU 9559  C   ALA B 425    14027  21143  12752   1189  -1318
-4245       C
ATOM   9560  O   ALA B 425      18.394 -37.345  43.855  1.00140.00
O
ANISOU 9560  O   ALA B 425    15909  22738  14546   1432  -1523
-4370       O
ATOM   9561  CB  ALA B 425      18.854 -36.201  46.762  1.00157.96
C
ANISOU 9561  CB  ALA B 425    18107  25181  16728   1196  -1386
-4027       C
ATOM   9562  N   PRO B 426      18.330 -35.111  43.651  1.00137.60
N
```

```
ANISOU 9562  N   PRO B 426    15267  22889  14125   1076  -1102
-4329       N
ATOM   9563  CA  PRO B 426      18.343 -35.186  42.182  1.00143.28
C
ANISOU 9563  CA  PRO B 426    15940  23661  14837   1157  -1083
-4523       C
ATOM   9564  C   PRO B 426      19.579 -35.869  41.622  1.00146.36
C
ANISOU 9564  C   PRO B 426    16235  24211  15165   1497  -1212
-4788       C
ATOM   9565  O   PRO B 426      19.459 -36.674  40.691  1.00181.46
O
ANISOU 9565  O   PRO B 426    20768  28517  19662   1665  -1340
-4915       O
ATOM   9566  CB  PRO B 426      18.285 -33.710  41.768  1.00142.31
C
ANISOU 9566  CB  PRO B 426    15652  23815  14604    924   -819
-4531       C
ATOM   9567  CG  PRO B 426      17.519 -33.057  42.863  1.00142.99
C
ANISOU 9567  CG  PRO B 426    15790  23827  14711    671   -733
-4281       C
ATOM   9568  CD  PRO B 426      17.990 -33.758  44.124  1.00140.27
C
ANISOU 9568  CD  PRO B 426    15497  23399  14400    791   -875
-4190       C
ATOM   9569  N   GLU B 427      20.767 -35.568  42.153  1.00145.54
N
ANISOU 9569  N   GLU B 427    15943  24412  14944   1613  -1186
-4891       N
ATOM   9570  CA  GLU B 427      21.963 -36.296  41.740  1.00135.14
C
ANISOU 9570  CA  GLU B 427    14517  23273  13557   1970  -1336
-5170       C
ATOM   9571  C   GLU B 427      21.849 -37.786  42.048  1.00112.39
C
ANISOU 9571  C   GLU B 427    11896  20036  10770   2250  -1656
-5168       C
ATOM   9572  O   GLU B 427      22.283 -38.627  41.251  1.00114.42
O
ANISOU 9572  O   GLU B 427    12175  20282  11018   2540  -1824
-5383       O
ATOM   9573  CB  GLU B 427      23.207 -35.699  42.401  1.00161.12
C
ANISOU 9573  CB  GLU B 427    17550  26966  16702   2030  -1257
-5282       C
ATOM   9574  CG  GLU B 427      23.550 -34.297  41.915  1.00165.44
C
ANISOU 9574  CG  GLU B 427    17848  27905  17106   1777   -959
-5346       C
ATOM   9575  CD  GLU B 427      24.923 -33.835  42.369  1.00169.13
C
ANISOU 9575  CD  GLU B 427    18032  28820  17410   1859   -894
-5524       C
```

```
ATOM   9576  OE1 GLU B 427      25.000 -32.848  43.130  1.00177.98
O
ANISOU 9576  OE1 GLU B 427    19073  30086  18465   1626   -732
-5390       O
ATOM   9577  OE2 GLU B 427      25.927 -34.457  41.960  1.00165.81
O
ANISOU 9577  OE2 GLU B 427    17461  28620  16919   2162  -1011
-5810       O
ATOM   9578  N   GLN B 428      21.286 -38.131  43.209  1.00112.22
N
ANISOU 9578  N   GLN B 428    12094  19722  10823   2166  -1753
-4932       N
ATOM   9579  CA  GLN B 428      21.038 -39.534  43.534  1.00114.41
C
ANISOU 9579  CA  GLN B 428    12698  19602  11170   2372  -2061
-4897       C
ATOM   9580  C   GLN B 428      20.057 -40.178  42.558  1.00131.89
C
ANISOU 9580  C   GLN B 428    15113  21510  13489   2329  -2138
-4877       C
ATOM   9581  O   GLN B 428      20.261 -41.317  42.121  1.00143.07
O
ANISOU 9581  O   GLN B 428    16710  22734  14915   2611  -2393
-5005       O
ATOM   9582  CB  GLN B 428      20.510 -39.648  44.966  1.00114.07
C
ANISOU 9582  CB  GLN B 428    12868  19305  11167   2206  -2108
-4624       C
ATOM   9583  CG  GLN B 428      21.424 -39.066  46.036  1.00114.07
C
ANISOU 9583  CG  GLN B 428    12702  19566  11074   2253  -2053
-4625       C
ATOM   9584  CD  GLN B 428      22.881 -39.403  45.812  1.00143.49
C
ANISOU 9584  CD  GLN B 428    16256  23580  14685   2657  -2188
-4938       C
ATOM   9585  OE1 GLN B 428      23.706 -38.517  45.592  1.00173.93
O
ANISOU 9585  OE1 GLN B 428    19771  27873  18444   2659  -2010
-5089       O
ATOM   9586  NE2 GLN B 428      23.209 -40.688  45.875  1.00178.35
N
ANISOU 9586  NE2 GLN B 428    20914  27760  19091   2998  -2511
-5050       N
ATOM   9587  N   ILE B 429      18.984 -39.463  42.209  1.00145.87
N
ANISOU 9587  N   ILE B 429    16863  23231  15330   1992  -1934
-4727       N
ATOM   9588  CA  ILE B 429      18.086 -39.892  41.136  1.00135.79
C
ANISOU 9588  CA  ILE B 429    15716  21732  14146   1942  -1970
-4742       C
ATOM   9589  C   ILE B 429      18.815 -39.961  39.799  1.00150.34
C
```

```
ANISOU 9589  C   ILE B 429    17399  23790  15934   2187  -1977
-5026       C
ATOM   9590  O   ILE B 429      18.586 -40.874  38.996  1.00141.54
O
ANISOU 9590  O   ILE B 429    16442  22465  14873   2353  -2150
-5119       O
ATOM   9591  CB  ILE B 429      16.864 -38.958  41.063  1.00109.03
C
ANISOU 9591  CB  ILE B 429    12294  18312  10819   1552  -1740
-4560       C
ATOM   9592  CG1 ILE B 429      16.044 -39.049  42.349  1.00146.59
C
ANISOU 9592  CG1 ILE B 429    17224  22844  15631   1311  -1754
-4292       C
ATOM   9593  CG2 ILE B 429      15.991 -39.306  39.867  1.00108.82
C
ANISOU 9593  CG2 ILE B 429    12367  18100  10881   1515  -1766
-4610       C
ATOM   9594  CD1 ILE B 429      15.499 -40.429  42.633  1.00143.36
C
ANISOU 9594  CD1 ILE B 429    17159  22015  15297   1355  -2011
-4211       C
ATOM   9595  N   LEU B 430      19.703 -38.999  39.541  1.00158.33
N
ANISOU 9595  N   LEU B 430    18105  25228  16826   2198  -1788
-5172       N
ATOM   9596  CA  LEU B 430      20.465 -38.972  38.295  1.00130.15
C
ANISOU 9596  CA  LEU B 430    14355  21921  13175   2398  -1763
-5457       C
ATOM   9597  C   LEU B 430      21.276 -40.246  38.065  1.00132.66
C
ANISOU 9597  C   LEU B 430    14745  22192  13469   2829  -2057
-5672       C
ATOM   9598  O   LEU B 430      21.516 -40.622  36.912  1.00160.32
O
ANISOU 9598  O   LEU B 430    18210  25752  16953   3015  -2113
-5879       O
ATOM   9599  CB  LEU B 430      21.389 -37.752  38.288  1.00130.19
C
ANISOU 9599  CB  LEU B 430    14031  22414  13021   2304  -1516
-5573       C
ATOM   9600  CG  LEU B 430      21.873 -37.232  36.936  1.00128.40
C
ANISOU 9600  CG  LEU B 430    13605  22495  12685   2317  -1366
-5813       C
ATOM   9601  CD1 LEU B 430      20.691 -36.727  36.126  1.00146.61
C
ANISOU 9601  CD1 LEU B 430    16030  24614  15059   2059  -1233
-5700       C
ATOM   9602  CD2 LEU B 430      22.908 -36.135  37.124  1.00111.98
C
ANISOU 9602  CD2 LEU B 430    11223  20905  10418   2211  -1146
-5930       C
```

```
ATOM   9603  N   GLU B 431      21.706 -40.920  39.128  1.00117.78
N
ANISOU 9603  N   GLU B 431    12980  20198  11572   3011  -2261
-5639       N
ATOM   9604  CA  GLU B 431      22.427 -42.182  39.000  1.00121.06
C
ANISOU 9604  CA  GLU B 431    13522  20527  11948   3453  -2588
-5845       C
ATOM   9605  C   GLU B 431      21.533 -43.422  39.014  1.00122.23
C
ANISOU 9605  C   GLU B 431    14101  20133  12206   3516  -2864
-5714       C
ATOM   9606  O   GLU B 431      22.058 -44.539  38.954  1.00125.13
O
ANISOU 9606  O   GLU B 431    14648  20366  12530   3893  -3176
-5870       O
ATOM   9607  CB  GLU B 431      23.475 -42.292  40.112  1.00122.72
C
ANISOU 9607  CB  GLU B 431    13660  20914  12055   3666  -2699
-5921       C
ATOM   9608  CG  GLU B 431      24.624 -41.303  39.971  1.00122.59
C
ANISOU 9608  CG  GLU B 431    13202  21481  11894   3693  -2486
-6139       C
ATOM   9609  CD  GLU B 431      25.752 -41.575  40.945  1.00173.33
C
ANISOU 9609  CD  GLU B 431    19544  28101  18211   3982  -2644
-6281       C
ATOM   9610  OE1 GLU B 431      26.052 -40.685  41.769  1.00170.37
O
ANISOU 9610  OE1 GLU B 431    18992  27950  17791   3795  -2464
-6196       O
ATOM   9611  OE2 GLU B 431      26.341 -42.675  40.884  1.00191.19
O
ANISOU 9611  OE2 GLU B 431    21929  30290  20426   4411  -2961
-6487       O
ATOM   9612  N   LEU B 432      20.207 -43.266  39.086  1.00136.75
N
ANISOU 9612  N   LEU B 432    16119  21667  14171   3162  -2770
-5451       N
ATOM   9613  CA  LEU B 432      19.314 -44.400  38.844  1.00155.59
C
ANISOU 9613  CA  LEU B 432    18890  23574  16651   3178  -3003
-5358       C
ATOM   9614  C   LEU B 432      19.440 -44.915  37.415  1.00173.89
C
ANISOU 9614  C   LEU B 432    21201  25892  18977   3415  -3102
-5579       C
ATOM   9615  O   LEU B 432      19.556 -46.124  37.185  1.00182.49
O
ANISOU 9615  O   LEU B 432    22565  26715  20059   3699  -3414
-5666       O
ATOM   9616  CB  LEU B 432      17.866 -44.011  39.138  1.00161.15
C
```

```
ANISOU 9616  CB  LEU B 432    19718  24035  17478   2724  -2846
-5066       C
ATOM   9617  CG  LEU B 432      17.478 -43.775  40.595  1.00176.76
C
ANISOU 9617  CG  LEU B 432    21801  25898  19460   2470  -2800
-4812       C
ATOM   9618  CD1 LEU B 432      16.017 -43.370  40.677  1.00159.97
C
ANISOU 9618  CD1 LEU B 432    19745  23593  17444   2036  -2635
-4579       C
ATOM   9619  CD2 LEU B 432      17.744 -45.017  41.434  1.00206.92
C
ANISOU 9619  CD2 LEU B 432    25993  29390  23236   2669  -3131
-4775       C
ATOM   9620  N   ALA B 433      19.427 -44.006  36.444  1.00192.88
N
ANISOU 9620  N   ALA B 433    23318  28585  21383   3304  -2848
-5675       N
ATOM   9621  CA  ALA B 433      19.804 -44.304  35.065  1.00159.86
C
ANISOU 9621  CA  ALA B 433    19050  24515  17174   3550  -2900
-5929       C
ATOM   9622  C   ALA B 433      20.670 -43.145  34.602  1.00142.73
C
ANISOU 9622  C   ALA B 433    16465  22877  14887   3526  -2625
-6110       C
ATOM   9623  O   ALA B 433      20.189 -42.012  34.499  1.00141.78
O
ANISOU 9623  O   ALA B 433    16203  22889  14780   3186  -2336
-5999       O
ATOM   9624  CB  ALA B 433      18.581 -44.477  34.165  1.00129.37
C
ANISOU 9624  CB  ALA B 433    15354  20358  13443   3370  -2878
-5836       C
ATOM   9625  N   LYS B 434      21.939 -43.436  34.328  1.00130.73
N
ANISOU 9625  N   LYS B 434    14768  21668  13236   3883  -2725
-6400       N
ATOM   9626  CA  LYS B 434      22.945 -42.391  34.217  1.00128.93
C
ANISOU 9626  CA  LYS B 434    14142  21985  12859   3847  -2481
-6576       C
ATOM   9627  C   LYS B 434      22.589 -41.376  33.138  1.00148.72
C
ANISOU 9627  C   LYS B 434    16485  24675  15348   3575  -2182
-6602       C
ATOM   9628  O   LYS B 434      22.181 -41.735  32.029  1.00120.46
O
ANISOU 9628  O   LYS B 434    12992  20964  11815   3641  -2224
-6679       O
ATOM   9629  CB  LYS B 434      24.305 -43.029  33.928  1.00138.60
C
ANISOU 9629  CB  LYS B 434    15201  23520  13940   4306  -2667
-6933       C
```

```
ATOM   9630  CG  LYS B 434      25.464 -42.061  33.771  1.00145.56
C
ANISOU 9630  CG  LYS B 434    15647  25018  14640   4283  -2430
-7168       C
ATOM   9631  CD  LYS B 434      26.767 -42.831  33.593  1.00129.70
C
ANISOU 9631  CD  LYS B 434    13475  23316  12489   4774  -2657
-7542       C
ATOM   9632  CE  LYS B 434      27.970 -41.906  33.531  1.00130.37
C
ANISOU 9632  CE  LYS B 434    13103  24057  12376   4734  -2424
-7799       C
ATOM   9633  NZ  LYS B 434      27.907 -40.991  32.361  1.00129.02
N
ANISOU 9633  NZ  LYS B 434    12730  24158  12132   4457  -2110
-7879       N
ATOM   9634  N   ALA B 435      22.743 -40.103  33.490  1.00160.35
N
ANISOU 9634  N   ALA B 435    17749  26435  16742   3271  -1891
-6534       N
ATOM   9635  CA  ALA B 435      22.540 -38.984  32.584  1.00158.74
C
ANISOU 9635  CA  ALA B 435    17407  26439  16469   2993  -1599
-6567       C
ATOM   9636  C   ALA B 435      23.438 -39.093  31.356  1.00146.05
C
ANISOU 9636  C   ALA B 435    15605  25165  14725   3201  -1576
-6905       C
ATOM   9637  O   ALA B 435      24.464 -39.779  31.354  1.00152.19
O
ANISOU 9637  O   ALA B 435    16249  26156  15420   3544  -1736
-7147       O
ATOM   9638  CB  ALA B 435      22.797 -37.663  33.305  1.00114.87
C
ANISOU 9638  CB  ALA B 435    11673  21169  10805   2676  -1331
-6468       C
ATOM   9639  N   SER B 436      23.038 -38.395  30.300  1.00141.00
N
ANISOU 9639  N   SER B 436    14953  24575  14045   2993  -1379
-6934       N
ATOM   9640  CA  SER B 436      23.746 -38.424  29.031  1.00181.68
C
ANISOU 9640  CA  SER B 436    19946  30022  19061   3133  -1327
-7238       C
ATOM   9641  C   SER B 436      24.836 -37.362  28.968  1.00205.98
C
ANISOU 9641  C   SER B 436    22704  33664  21894   2966  -1064
-7419       C
ATOM   9642  O   SER B 436      25.392 -37.127  27.890  1.00248.59
O
ANISOU 9642  O   SER B 436    27957  39357  27139   2978   -950
-7664       O
ATOM   9643  CB  SER B 436      22.765 -38.237  27.871  1.00180.88
C
```

```
ANISOU 9643  CB  SER B 436    20027  29673  19026   2995  -1257
-7189       C
ATOM   9644  OG  SER B 436      22.061 -37.013  27.995  1.00169.80
O
ANISOU 9644  OG  SER B 436    18684  28231  17601   2586  -1013
-6996       O
ATOM   9645  N   ASN B 437      25.105 -36.698  30.100  1.00184.26
N
ANISOU 9645  N   ASN B 437    19856  31060  19095   2785   -959
-7295       N
ATOM   9646  CA  ASN B 437      26.023 -35.571  30.263  1.00176.36
C
ANISOU 9646  CA  ASN B 437    18583  30561  17863   2549   -697
-7409       C
ATOM   9647  C   ASN B 437      25.370 -34.282  29.783  1.00175.96
C
ANISOU 9647  C   ASN B 437    18638  30486  17732   2108   -419
-7270       C
ATOM   9648  O   ASN B 437      25.396 -33.268  30.487  1.00176.41
O
ANISOU 9648  O   ASN B 437    18662  30663  17703   1810   -244
-7132       O
ATOM   9649  CB  ASN B 437      27.356 -35.809  29.548  1.00178.72
C
ANISOU 9649  CB  ASN B 437    18572  31344  17990   2750   -683
-7775       C
ATOM   9650  CG  ASN B 437      28.050 -37.068  30.022  1.00180.51
C
ANISOU 9650  CG  ASN B 437    18704  31642  18241   3241   -985
-7983       C
ATOM   9651  OD1 ASN B 437      27.713 -37.617  31.070  1.00173.34
O
ANISOU 9651  OD1 ASN B 437    17944  30436  17483   3372  -1179
-7799       O
ATOM   9652  ND2 ASN B 437      29.026 -37.534  29.251  1.00184.50
N
ANISOU 9652  ND2 ASN B 437    18974  32433  18696   3477  -1045
-8238       N
ATOM   9653  N   ASP B 438      24.789 -34.310  28.582  1.00194.90
N
ANISOU 9653  N   ASP B 438    21185  32722  20145   2078   -392
-7312       N
ATOM   9654  CA  ASP B 438      23.943 -33.205  28.145  1.00190.30
C
ANISOU 9654  CA  ASP B 438    20792  31963  19549   1697   -194
-7113       C
ATOM   9655  C   ASP B 438      22.751 -33.022  29.079  1.00157.46
C
ANISOU 9655  C   ASP B 438    16855  27433  15539   1570   -243
-6817       C
ATOM   9656  O   ASP B 438      22.431 -31.900  29.487  1.00163.57
O
ANISOU 9656  O   ASP B 438    17690  28234  16226   1252    -71
-6666       O
```

```
ATOM   9657  CB  ASP B 438      23.476 -33.457  26.708  1.00172.36
C
ANISOU 9657  CB  ASP B 438    18664  29494  17332   1744   -210
-7174       C
ATOM   9658  CG  ASP B 438      22.738 -32.275  26.116  1.00175.59
C
ANISOU 9658  CG  ASP B 438    19281  29716  17719   1371    -17
-6978       C
ATOM   9659  OD1 ASP B 438      23.139 -31.124  26.383  1.00181.28
O
ANISOU 9659  OD1 ASP B 438    19955  30584  18338   1050    179
-6845       O
ATOM   9660  OD2 ASP B 438      21.751 -32.500  25.383  1.00183.59
O
ANISOU 9660  OD2 ASP B 438    20522  30417  18816   1410    -81
-6959       O
ATOM   9661  N   LEU B 439      22.082 -34.124  29.428  1.00145.68
N
ANISOU 9661  N   LEU B 439    15496  25568  14288   1803   -486
-6703       N
ATOM   9662  CA  LEU B 439      21.045 -34.091  30.457  1.00171.40
C
ANISOU 9662  CA  LEU B 439    18926  28482  17716   1686   -549
-6400       C
ATOM   9663  C   LEU B 439      21.598 -33.704  31.827  1.00154.72
C
ANISOU 9663  C   LEU B 439    16679  26553  15554   1606   -504
-6296       C
ATOM   9664  O   LEU B 439      20.965 -32.937  32.564  1.00106.45
O
ANISOU 9664  O   LEU B 439    10648  20343   9456   1355   -410
-6075       O
ATOM   9665  CB  LEU B 439      20.349 -35.454  30.518  1.00151.48
C
ANISOU 9665  CB  LEU B 439    16575  25552  15429   1940   -824
-6330       C
ATOM   9666  CG  LEU B 439      19.070 -35.610  31.344  1.00118.03
C
ANISOU 9666  CG  LEU B 439    12549  20916  11382   1810   -905
-6038       C
ATOM   9667  CD1 LEU B 439      18.128 -36.596  30.671  1.00131.77
C
ANISOU 9667  CD1 LEU B 439    14502  22266  13301   1941  -1091
-6020       C
ATOM   9668  CD2 LEU B 439      19.389 -36.070  32.759  1.00108.12
C
ANISOU 9668  CD2 LEU B 439    11267  19634  10180   1883  -1021
-5914       C
ATOM   9669  N   SER B 440      22.773 -34.224  32.187  1.00110.68
N
ANISOU 9669  N   SER B 440    10894  21249   9912   1834   -583
-6467       N
ATOM   9670  CA  SER B 440      23.393 -33.877  33.466  1.00110.78
C
```

```
ANISOU 9670  CA  SER B 440    10766  21458   9868   1783   -549
-6396       C
ATOM   9671  C   SER B 440      23.680 -32.384  33.583  1.00109.47
C
ANISOU 9671  C   SER B 440    10497  21595   9503   1421   -263
-6366       C
ATOM   9672  O   SER B 440      23.405 -31.766  34.616  1.00109.25
O
ANISOU 9672  O   SER B 440    10501  21526   9483   1233   -199
-6157       O
ATOM   9673  CB  SER B 440      24.682 -34.679  33.653  1.00167.84
C
ANISOU 9673  CB  SER B 440    17770  28971  17030   2124   -690
-6650       C
ATOM   9674  OG  SER B 440      25.209 -34.501  34.956  1.00155.43
O
ANISOU 9674  OG  SER B 440    16090  27533  15433   2122   -701
-6577       O
ATOM   9675  N   LYS B 441      24.242 -31.789  32.535  1.00141.07
N
ANISOU 9675  N   LYS B 441    14393  25902  13306   1309    -93
-6576       N
ATOM   9676  CA  LYS B 441      24.434 -30.343  32.507  1.00149.38
C
ANISOU 9676  CA  LYS B 441    15420  27178  14159    923    171
-6524       C
ATOM   9677  C   LYS B 441      23.120 -29.574  32.622  1.00130.60
C
ANISOU 9677  C   LYS B 441    13325  24478  11817    664    238
-6274       C
ATOM   9678  O   LYS B 441      23.041 -28.574  33.346  1.00124.43
O
ANISOU 9678  O   LYS B 441    12573  23729  10977    405    359
-6089       O
ATOM   9679  CB  LYS B 441      25.168 -29.947  31.225  1.00164.67
C
ANISOU 9679  CB  LYS B 441    17255  29265  16047    810    298
-6614       C
ATOM   9680  CG  LYS B 441      26.625 -30.384  31.179  1.00174.44
C
ANISOU 9680  CG  LYS B 441    18151  30898  17230    980    275
-6837       C
ATOM   9681  CD  LYS B 441      27.210 -30.236  29.781  1.00176.56
C
ANISOU 9681  CD  LYS B 441    18336  31294  17456    908    363
-6961       C
ATOM   9682  CE  LYS B 441      27.156 -28.798  29.295  1.00177.58
C
ANISOU 9682  CE  LYS B 441    18587  31386  17499    441    597
-6776       C
ATOM   9683  NZ  LYS B 441      28.028 -27.902  30.102  1.00182.66
N
ANISOU 9683  NZ  LYS B 441    19051  32303  18049    187    722
-6706       N
```

```
ATOM   9684  N   LYS B 442      22.082 -30.020  31.915  1.00121.12
N
ANISOU 9684  N   LYS B 442    12336  22919  10765    737    142
-6222       N
ATOM   9685  CA  LYS B 442      20.756 -29.414  32.040  1.00136.31
C
ANISOU 9685  CA  LYS B 442    14515  24516  12761    540    166
-5992       C
ATOM   9686  C   LYS B 442      20.157 -29.515  33.445  1.00124.29
C
ANISOU 9686  C   LYS B 442    13032  22798  11396    520     82
-5732       C
ATOM   9687  O   LYS B 442      19.679 -28.514  33.991  1.00104.52
O
ANISOU 9687  O   LYS B 442    10615  20273   8823    280    186
-5570       O
ATOM   9688  CB  LYS B 442      19.812 -30.055  31.021  1.00139.41
C
ANISOU 9688  CB  LYS B 442    15091  24579  13300    666     52
-6020       C
ATOM   9689  CG  LYS B 442      20.127 -29.686  29.581  1.00145.56
C
ANISOU 9689  CG  LYS B 442    15907  25495  13905    616    162
-6236       C
ATOM   9690  CD  LYS B 442      19.877 -28.215  29.308  1.00148.50
C
ANISOU 9690  CD  LYS B 442    16436  25792  14196    268    345
-6042       C
ATOM   9691  CE  LYS B 442      20.075 -27.897  27.836  1.00138.91
C
ANISOU 9691  CE  LYS B 442    15321  24512  12948    202    422
-6096       C
ATOM   9692  NZ  LYS B 442      19.831 -26.461  27.538  1.00145.25
N
ANISOU 9692  NZ  LYS B 442    16344  25189  13657   -122    568
-5906       N
ATOM   9693  N   VAL B 443      20.161 -30.707  34.048  1.00135.87
N
ANISOU 9693  N   VAL B 443    14456  24111  13056    769   -114
-5692       N
ATOM   9694  CA  VAL B 443      19.630 -30.848  35.409  1.00123.28
C
ANISOU 9694  CA  VAL B 443    12912  22335  11595    735   -190
-5450       C
ATOM   9695  C   VAL B 443      20.436 -30.043  36.429  1.00101.33
C
ANISOU 9695  C   VAL B 443     9979  19848   8673    603    -70
-5399       C
ATOM   9696  O   VAL B 443      19.866 -29.359  37.285  1.00 99.64
O
ANISOU 9696  O   VAL B 443     9836  19559   8463    413    -12
-5191       O
ATOM   9697  CB  VAL B 443      19.514 -32.335  35.806  1.00104.78
C
```

```
ANISOU 9697  CB  VAL B 443    10609  19746   9457   1017   -439
-5427       C
ATOM   9698  CG1 VAL B 443      18.581 -33.066  34.850  1.00120.47
C
ANISOU 9698  CG1 VAL B 443    12772  21415  11586   1107   -556
-5449       C
ATOM   9699  CG2 VAL B 443      20.865 -33.024  35.867  1.00140.05
C
ANISOU 9699  CG2 VAL B 443    14888  24461  13864   1287   -528
-5634       C
ATOM   9700  N   LEU B 444      21.765 -30.111  36.366  1.00103.17
N
ANISOU 9700  N   LEU B 444     9992  20434   8773    706    -35
-5598       N
ATOM   9701  CA  LEU B 444      22.587 -29.315  37.277  1.00103.15
C
ANISOU 9701  CA  LEU B 444     9831  20740   8622    569     86
-5573       C
ATOM   9702  C   LEU B 444      22.389 -27.809  37.108  1.00101.51
C
ANISOU 9702  C   LEU B 444     9690  20662   8216    209    312
-5504       C
ATOM   9703  O   LEU B 444      22.357 -27.071  38.099  1.00148.55
O
ANISOU 9703  O   LEU B 444    15650  26670  14123     41    381
-5341       O
ATOM   9704  CB  LEU B 444      24.061 -29.683  37.101  1.00122.38
C
ANISOU 9704  CB  LEU B 444    11992  23574  10933    750     82
-5852       C
ATOM   9705  CG  LEU B 444      24.433 -31.111  37.509  1.00115.15
C
ANISOU 9705  CG  LEU B 444    11018  22562  10174   1141   -171
-5931       C
ATOM   9706  CD1 LEU B 444      25.858 -31.441  37.091  1.00146.26
C
ANISOU 9706  CD1 LEU B 444    14672  26937  13965   1347   -180
-6270       C
ATOM   9707  CD2 LEU B 444      24.249 -31.311  39.008  1.00115.21
C
ANISOU 9707  CD2 LEU B 444    11073  22410  10292   1174   -273
-5713       C
ATOM   9708  N   SER B 445      22.258 -27.332  35.870  1.00101.46
N
ANISOU 9708  N   SER B 445     9768  20700   8082     89    417
-5628       N
ATOM   9709  CA  SER B 445      21.966 -25.917  35.637  1.00107.11
C
ANISOU 9709  CA  SER B 445    10633  21373   8693   -249    584
-5472       C
ATOM   9710  C   SER B 445      20.645 -25.456  36.256  1.00126.59
C
ANISOU 9710  C   SER B 445    13324  23585  11190   -361    559
-5268       C
```

```
ATOM   9711  O   SER B 445      20.593 -24.405  36.906  1.00163.68
O
ANISOU 9711  O   SER B 445    18081  28272  15839   -578    645
-5073       O
ATOM   9712  CB  SER B 445      21.972 -25.635  34.135  1.00120.39
C
ANISOU 9712  CB  SER B 445    12418  22957  10366   -326    647
-5534       C
ATOM   9713  OG  SER B 445      21.775 -24.256  33.873  1.00124.95
O
ANISOU 9713  OG  SER B 445    13186  23412  10876   -638    776
-5347       O
ATOM   9714  N   ILE B 446      19.564 -26.216  36.065  1.00101.30
N
ANISOU 9714  N   ILE B 446    10247  20063   8180   -217    419
-5218       N
ATOM   9715  CA  ILE B 446      18.304 -25.884  36.736  1.00 97.70
C
ANISOU 9715  CA  ILE B 446     9958  19335   7828   -308    374
-4988       C
ATOM   9716  C   ILE B 446      18.390 -26.050  38.254  1.00113.20
C
ANISOU 9716  C   ILE B 446    11825  21300   9886   -293    327
-4803       C
ATOM   9717  O   ILE B 446      17.816 -25.248  39.002  1.00 93.08
O
ANISOU 9717  O   ILE B 446     9360  18707   7298   -455    370
-4631       O
ATOM   9718  CB  ILE B 446      17.130 -26.669  36.115  1.00112.32
C
ANISOU 9718  CB  ILE B 446    11950  20843   9883   -181    239
-4977       C
ATOM   9719  CG1 ILE B 446      17.346 -28.177  36.180  1.00125.20
C
ANISOU 9719  CG1 ILE B 446    13484  22359  11727     85     73
-5021       C
ATOM   9720  CG2 ILE B 446      16.897 -26.230  34.678  1.00131.67
C
ANISOU 9720  CG2 ILE B 446    14544  23273  12212   -232    298
-5134       C
ATOM   9721  CD1 ILE B 446      16.145 -28.978  35.725  1.00138.33
C
ANISOU 9721  CD1 ILE B 446    15293  23673  13594    178    -67
-4984       C
ATOM   9722  N   ILE B 447      19.083 -27.082  38.742  1.00123.45
N
ANISOU 9722  N   ILE B 447    12965  22640  11301    -88    225
-4841       N
ATOM   9723  CA  ILE B 447      19.291 -27.207  40.187  1.00 95.90
C
ANISOU 9723  CA  ILE B 447     9398  19164   7874    -72    182
-4679       C
ATOM   9724  C   ILE B 447      20.022 -25.989  40.742  1.00120.00
C
```

```
ANISOU 9724  C   ILE B 447    12367  22519  10711   -271    344
-4654       C
ATOM   9725  O   ILE B 447      19.638 -25.432  41.777  1.00184.47
O
ANISOU 9725  O   ILE B 447    20574  30641  18875   -393    367
-4458       O
ATOM   9726  CB  ILE B 447      20.040 -28.511  40.522  1.00 97.92
C
ANISOU 9726  CB  ILE B 447     9531  19423   8252    211     25
-4765       C
ATOM   9727  CG1 ILE B 447      19.160 -29.731  40.250  1.00 98.33
C
ANISOU 9727  CG1 ILE B 447     9725  19113   8524    377   -160
-4729       C
ATOM   9728  CG2 ILE B 447      20.513 -28.508  41.968  1.00118.43
C
ANISOU 9728  CG2 ILE B 447    12043  22093  10862    228     -3
-4637       C
ATOM   9729  CD1 ILE B 447      19.941 -31.008  40.065  1.00100.72
C
ANISOU 9729  CD1 ILE B 447     9957  19415   8896    688   -331
-4891       C
ATOM   9730  N   ASP B 448      21.083 -25.555  40.061  1.00 96.56
N
ANISOU 9730  N   ASP B 448     9277  19868   7542   -322    460
-4856       N
ATOM   9731  CA  ASP B 448      21.745 -24.304  40.426  1.00103.98
C
ANISOU 9731  CA  ASP B 448    10169  21099   8240   -565    628
-4846       C
ATOM   9732  C   ASP B 448      20.819 -23.098  40.292  1.00131.56
C
ANISOU 9732  C   ASP B 448    13907  24392  11688   -819    704
-4652       C
ATOM   9733  O   ASP B 448      20.831 -22.204  41.147  1.00164.90
O
ANISOU 9733  O   ASP B 448    18164  28615  15874   -980    755
-4464       O
ATOM   9734  CB  ASP B 448      23.005 -24.113  39.582  1.00113.32
C
ANISOU 9734  CB  ASP B 448    11187  22527   9342   -601    720
-5019       C
ATOM   9735  CG  ASP B 448      24.124 -25.051  39.992  1.00151.41
C
ANISOU 9735  CG  ASP B 448    15729  27646  14155   -357    653
-5226       C
ATOM   9736  OD1 ASP B 448      25.165 -25.076  39.302  1.00159.07
O
ANISOU 9736  OD1 ASP B 448    16520  28823  15096   -347    700
-5384       O
ATOM   9737  OD2 ASP B 448      23.965 -25.760  41.009  1.00157.32
O
ANISOU 9737  OD2 ASP B 448    16445  28297  15033   -172    520
-5146       O
```

```
ATOM   9738  N   LYS B 449      20.015 -23.047  39.227  1.00120.59
N
ANISOU 9738  N   LYS B 449    12701  22785  10333   -834    689
-4676       N
ATOM   9739  CA  LYS B 449      19.009 -21.992  39.114  1.00111.79
C
ANISOU 9739  CA  LYS B 449    11845  21416   9213  -1010    712
-4488       C
ATOM   9740  C   LYS B 449      17.974 -22.040  40.234  1.00105.45
C
ANISOU 9740  C   LYS B 449    11099  20526   8442   -994    640
-4353       C
ATOM   9741  O   LYS B 449      17.613 -20.996  40.787  1.00114.87
O
ANISOU 9741  O   LYS B 449    12413  21628   9606  -1145    672
-4161       O
ATOM   9742  CB  LYS B 449      18.318 -22.055  37.752  1.00122.25
C
ANISOU 9742  CB  LYS B 449    13354  22525  10572   -984    687
-4568       C
ATOM   9743  CG  LYS B 449      17.372 -20.886  37.525  1.00142.69
C
ANISOU 9743  CG  LYS B 449    16232  24844  13139  -1134    696
-4399       C
ATOM   9744  CD  LYS B 449      18.111 -19.558  37.543  1.00125.13
C
ANISOU 9744  CD  LYS B 449    14105  22626  10811  -1357    801
-4265       C
ATOM   9745  CE  LYS B 449      17.175 -18.403  37.228  1.00114.90
C
ANISOU 9745  CE  LYS B 449    13149  21028   9478  -1457    778
-4118       C
ATOM   9746  NZ  LYS B 449      17.905 -17.110  37.105  1.00107.85
N
ANISOU 9746  NZ  LYS B 449    12409  20101   8468  -1670    864
-4004       N
ATOM   9747  N   TYR B 450      17.482 -23.230  40.586  1.00 90.62
N
ANISOU 9747  N   TYR B 450     9150  18524   6759   -806    515
-4349       N
ATOM   9748  CA  TYR B 450      16.601 -23.333  41.749  1.00 97.32
C
ANISOU 9748  CA  TYR B 450    10026  19202   7751   -807    440
-4133       C
ATOM   9749  C   TYR B 450      17.272 -22.837  43.024  1.00134.15
C
ANISOU 9749  C   TYR B 450    14588  24042  12340   -883    493
-4006       C
ATOM   9750  O   TYR B 450      16.649 -22.135  43.829  1.00129.80
O
ANISOU 9750  O   TYR B 450    14116  23449  11755   -995    505
-3842       O
ATOM   9751  CB  TYR B 450      16.126 -24.773  41.946  1.00 91.79
C
```

```
ANISOU 9751  CB  TYR B 450     9279  18273   7324   -616    290
-4100       C
ATOM   9752  CG  TYR B 450      15.136 -25.279  40.924  1.00101.67
C
ANISOU 9752  CG  TYR B 450    10649  19295   8686   -555    216
-4174       C
ATOM   9753  CD1 TYR B 450      14.700 -24.476  39.880  1.00112.10
C
ANISOU 9753  CD1 TYR B 450    12111  20612   9870   -642    275
-4273       C
ATOM   9754  CD2 TYR B 450      14.621 -26.562  41.022  1.00101.15
C
ANISOU 9754  CD2 TYR B 450    10580  19002   8849   -415     76
-4145       C
ATOM   9755  CE1 TYR B 450      13.765 -24.936  38.970  1.00114.43
C
ANISOU 9755  CE1 TYR B 450    12512  20695  10271   -573    198
-4347       C
ATOM   9756  CE2 TYR B 450      13.705 -27.036  40.109  1.00121.65
C
ANISOU 9756  CE2 TYR B 450    13280  21393  11549   -368      5
-4214       C
ATOM   9757  CZ  TYR B 450      13.277 -26.221  39.087  1.00118.30
C
ANISOU 9757  CZ  TYR B 450    12966  20981  11002   -439     68
-4318       C
ATOM   9758  OH  TYR B 450      12.384 -26.706  38.159  1.00133.57
O
ANISOU 9758  OH  TYR B 450    14998  22713  13041   -376    -10
-4402       O
ATOM   9759  N   ALA B 451      18.537 -23.200  43.232  1.00 90.58
N
ANISOU 9759  N   ALA B 451     8887  18733   6794   -810    516
-4094       N
ATOM   9760  CA  ALA B 451      19.280 -22.671  44.371  1.00 90.47
C
ANISOU 9760  CA  ALA B 451     8768  18915   6690   -883    571
-4000       C
ATOM   9761  C   ALA B 451      19.391 -21.148  44.341  1.00100.18
C
ANISOU 9761  C   ALA B 451    10104  20296   7665  -1138    707
-3961       C
ATOM   9762  O   ALA B 451      19.235 -20.490  45.377  1.00 88.45
O
ANISOU 9762  O   ALA B 451     8647  18799   6161  -1232    721
-3776       O
ATOM   9763  CB  ALA B 451      20.668 -23.309  44.415  1.00 92.44
C
ANISOU 9763  CB  ALA B 451     8790  19401   6932   -743    566
-4160       C
ATOM   9764  N   GLU B 452      19.663 -20.567  43.171  1.00108.00
N
ANISOU 9764  N   GLU B 452    11178  21214   8644  -1236    768
-4007       N
```

```
ATOM   9765  CA  GLU B 452      19.574 -19.114  43.036  1.00111.08
C
ANISOU 9765  CA  GLU B 452    11759  21456   8991  -1456    832
-3836       C
ATOM   9766  C   GLU B 452      18.164 -18.591  43.299  1.00113.57
C
ANISOU 9766  C   GLU B 452    12302  21519   9332  -1483    772
-3683       C
ATOM   9767  O   GLU B 452      17.997 -17.550  43.946  1.00 86.33
O
ANISOU 9767  O   GLU B 452     8967  17984   5851  -1601    783
-3502       O
ATOM   9768  CB  GLU B 452      20.060 -18.686  41.651  1.00132.70
C
ANISOU 9768  CB  GLU B 452    14586  24144  11691  -1551    895
-3929       C
ATOM   9769  CG  GLU B 452      19.876 -17.204  41.364  1.00150.06
C
ANISOU 9769  CG  GLU B 452    17060  26134  13823  -1763    936
-3768       C
ATOM   9770  CD  GLU B 452      20.369 -16.808  39.988  1.00177.97
C
ANISOU 9770  CD  GLU B 452    20711  29614  17295  -1870   1000
-3860       C
ATOM   9771  OE1 GLU B 452      19.530 -16.456  39.132  1.00189.18
O
ANISOU 9771  OE1 GLU B 452    22398  30776  18706  -1869    967
-3843       O
ATOM   9772  OE2 GLU B 452      21.597 -16.848  39.765  1.00176.55
O
ANISOU 9772  OE2 GLU B 452    20353  29658  17068  -1953   1078
-3955       O
ATOM   9773  N   ARG B 453      17.136 -19.291  42.807  1.00134.10
N
ANISOU 9773  N   ARG B 453    14964  24001  11986  -1365    697
-3766       N
ATOM   9774  CA  ARG B 453      15.769 -18.927  43.172  1.00124.51
C
ANISOU 9774  CA  ARG B 453    13909  22600  10800  -1368    628
-3651       C
ATOM   9775  C   ARG B 453      15.555 -19.008  44.677  1.00 99.21
C
ANISOU 9775  C   ARG B 453    10604  19497   7596  -1369    604
-3518       C
ATOM   9776  O   ARG B 453      14.894 -18.144  45.267  1.00111.37
O
ANISOU 9776  O   ARG B 453    12263  20925   9126  -1435    585
-3357       O
ATOM   9777  CB  ARG B 453      14.769 -19.828  42.442  1.00158.84
C
ANISOU 9777  CB  ARG B 453    18291  26847  15213  -1242    545
-3795       C
ATOM   9778  CG  ARG B 453      14.766 -19.698  40.919  1.00170.33
C
```

```
ANISOU 9778  CG  ARG B 453    19886  28157  16675  -1230    554
-3912       C
ATOM   9779  CD  ARG B 453      13.998 -18.474  40.426  1.00183.28
C
ANISOU 9779  CD  ARG B 453    21812  29538  18286  -1302    536
-3807       C
ATOM   9780  NE  ARG B 453      14.746 -17.231  40.599  1.00196.94
N
ANISOU 9780  NE  ARG B 453    23660  31241  19926  -1453    608
-3668       N
ATOM   9781  CZ  ARG B 453      14.259 -16.139  41.179  1.00188.80
C
ANISOU 9781  CZ  ARG B 453    22804  30075  18855  -1514    580
-3502       C
ATOM   9782  NH1 ARG B 453      12.992 -16.100  41.567  1.00178.47
N
ANISOU 9782  NH1 ARG B 453    21563  28658  17591  -1432    486
-3463       N
ATOM   9783  NH2 ARG B 453      15.017 -15.057  41.301  1.00185.07
N
ANISOU 9783  NH2 ARG B 453    22453  29569  18295  -1652    637
-3389       N
ATOM   9784  N   GLY B 454      16.102 -20.038  45.314  1.00 85.49
N
ANISOU 9784  N   GLY B 454     8661  17938   5881  -1277    593
-3575       N
ATOM   9785  CA  GLY B 454      16.038 -20.158  46.755  1.00 84.99
C
ANISOU 9785  CA  GLY B 454     8511  17871   5911  -1266    563
-3385       C
ATOM   9786  C   GLY B 454      15.502 -21.504  47.184  1.00 85.46
C
ANISOU 9786  C   GLY B 454     8491  17733   6248  -1117    451
-3323       C
ATOM   9787  O   GLY B 454      15.226 -21.725  48.367  1.00 85.13
O
ANISOU 9787  O   GLY B 454     8410  17637   6300  -1113    412
-3158       O
ATOM   9788  N   LEU B 455      15.354 -22.415  46.226  1.00 86.37
N
ANISOU 9788  N   LEU B 455     8604  17729   6482  -1005    395
-3454       N
ATOM   9789  CA  LEU B 455      14.403 -23.511  46.332  1.00 86.67
C
ANISOU 9789  CA  LEU B 455     8660  17522   6749   -918    280
-3405       C
ATOM   9790  C   LEU B 455      15.137 -24.843  46.382  1.00 88.33
C
ANISOU 9790  C   LEU B 455     8776  17676   7110   -745    195
-3457       C
ATOM   9791  O   LEU B 455      16.034 -25.100  45.571  1.00 89.37
O
ANISOU 9791  O   LEU B 455     8847  17912   7199   -650    206
-3627       O
```

```
ATOM   9792  CB  LEU B 455      13.411 -23.469  45.165  1.00 86.37
C
ANISOU 9792  CB  LEU B 455     8740  17352   6725   -924    256
-3513       C
ATOM   9793  CG  LEU B 455      12.740 -22.097  45.009  1.00 84.99
C
ANISOU 9793  CG  LEU B 455     8695  17234   6362  -1057    313
-3501       C
ATOM   9794  CD1 LEU B 455      11.858 -22.015  43.775  1.00 84.87
C
ANISOU 9794  CD1 LEU B 455     8810  17098   6338  -1036    278
-3641       C
ATOM   9795  CD2 LEU B 455      11.942 -21.756  46.252  1.00 84.08
C
ANISOU 9795  CD2 LEU B 455     8577  17097   6271  -1130    294
-3319       C
ATOM   9796  N   ARG B 456      14.758 -25.672  47.349  1.00 88.75
N
ANISOU 9796  N   ARG B 456     8833  17569   7318   -707    103
-3320       N
ATOM   9797  CA  ARG B 456      15.213 -27.052  47.422  1.00 94.82
C
ANISOU 9797  CA  ARG B 456     9587  18208   8233   -530    -25
-3360       C
ATOM   9798  C   ARG B 456      14.787 -27.828  46.178  1.00 91.30
C
ANISOU 9798  C   ARG B 456     9204  17606   7879   -440    -99
-3502       C
ATOM   9799  O   ARG B 456      13.747 -27.554  45.574  1.00 90.47
O
ANISOU 9799  O   ARG B 456     9174  17409   7791   -532    -79
-3512       O
ATOM   9800  CB  ARG B 456      14.647 -27.704  48.684  1.00150.45
C
ANISOU 9800  CB  ARG B 456    16697  25068  15400   -560   -111
-3166       C
ATOM   9801  CG  ARG B 456      14.755 -29.216  48.763  1.00179.76
C
ANISOU 9801  CG  ARG B 456    20490  28551  19259   -406   -279
-3183       C
ATOM   9802  CD  ARG B 456      13.950 -29.758  49.941  1.00146.12
C
ANISOU 9802  CD  ARG B 456    16346  24083  15091   -512   -347
-2980       C
ATOM   9803  NE  ARG B 456      14.149 -28.994  51.153  1.00 92.07
N
ANISOU 9803  NE  ARG B 456     9449  17364   8170   -606   -271
-2827       N
ATOM   9804  CZ  ARG B 456      15.293 -28.784  51.795  1.00 93.18
C
ANISOU 9804  CZ  ARG B 456     9513  17649   8242   -505   -266
-2824       C
ATOM   9805  NH1 ARG B 456      15.411 -27.750  52.597  1.00113.17
N
```

```
ANISOU 9805  NH1 ARG B 456    11976  20347  10678   -614   -159
-2712       N
ATOM   9806  NH2 ARG B 456      16.373 -29.479  51.466  1.00103.99
N
ANISOU 9806  NH2 ARG B 456    10854  19041   9618   -281   -360
-2970       N
ATOM   9807  N   SER B 457      15.607 -28.803  45.789  1.00 93.03
N
ANISOU 9807  N   SER B 457     9393  17802   8151   -240   -195
-3629       N
ATOM   9808  CA  SER B 457      15.429 -29.527  44.538  1.00104.90
C
ANISOU 9808  CA  SER B 457    10947  19188   9723   -123   -268
-3788       C
ATOM   9809  C   SER B 457      15.208 -31.004  44.833  1.00 96.15
C
ANISOU 9809  C   SER B 457     9947  17805   8780     14   -461
-3754       C
ATOM   9810  O   SER B 457      15.928 -31.596  45.643  1.00126.77
O
ANISOU 9810  O   SER B 457    13822  21672  12673    138   -553
-3723       O
ATOM   9811  CB  SER B 457      16.643 -29.344  43.624  1.00155.35
C
ANISOU 9811  CB  SER B 457    17214  25818  15994      4   -220
-4011       C
ATOM   9812  OG  SER B 457      17.846 -29.649  44.311  1.00141.75
O
ANISOU 9812  OG  SER B 457    15375  24251  14232    144   -260
-4049       O
ATOM   9813  N   LEU B 458      14.211 -31.591  44.172  1.00 95.80
N
ANISOU 9813  N   LEU B 458    10023  17532   8845     -8   -531
-3766       N
ATOM   9814  CA  LEU B 458      13.891 -33.007  44.295  1.00 99.48
C
ANISOU 9814  CA  LEU B 458    10642  17706   9450     91   -722
-3740       C
ATOM   9815  C   LEU B 458      13.468 -33.536  42.932  1.00115.91
C
ANISOU 9815  C   LEU B 458    12785  19658  11596    171   -784
-3888       C
ATOM   9816  O   LEU B 458      12.711 -32.874  42.217  1.00145.93
O
ANISOU 9816  O   LEU B 458    16572  23488  15388     50   -688
-3922       O
ATOM   9817  CB  LEU B 458      12.786 -33.240  45.339  1.00113.27
C
ANISOU 9817  CB  LEU B 458    12507  19257  11274   -115   -748
-3525       C
ATOM   9818  CG  LEU B 458      12.188 -34.640  45.508  1.00122.22
C
ANISOU 9818  CG  LEU B 458    13854  20055  12531   -107   -933
-3470       C
```

```
ATOM   9819  CD1 LEU B 458      11.841 -34.877  46.965  1.00133.47
C
ANISOU 9819  CD1 LEU B 458    15377  21369  13964   -262   -961
-3259       C
ATOM   9820  CD2 LEU B 458      10.947 -34.829  44.644  1.00126.16
C
ANISOU 9820  CD2 LEU B 458    14412  20410  13111   -232   -936
-3505       C
ATOM   9821  N   ALA B 459      13.944 -34.729  42.580  1.00141.13
N
ANISOU 9821  N   ALA B 459    16068  22704  14850    390   -961
-3983       N
ATOM   9822  CA  ALA B 459      13.713 -35.302  41.262  1.00142.72
C
ANISOU 9822  CA  ALA B 459    16328  22790  15107    507  -1038
-4140       C
ATOM   9823  C   ALA B 459      12.819 -36.535  41.348  1.00107.17
C
ANISOU 9823  C   ALA B 459    12053  17924  10741    487  -1220
-4063       C
ATOM   9824  O   ALA B 459      12.827 -37.268  42.341  1.00103.42
O
ANISOU 9824  O   ALA B 459    11718  17281  10297    481  -1343
-3940       O
ATOM   9825  CB  ALA B 459      15.036 -35.674  40.584  1.00139.31
C
ANISOU 9825  CB  ALA B 459    15813  22508  14610    801  -1104
-4354       C
ATOM   9826  N   VAL B 460      12.045 -36.753  40.284  1.00107.31
N
ANISOU 9826  N   VAL B 460    12128  17817  10827    465  -1240
-4141       N
ATOM   9827  CA  VAL B 460      11.128 -37.880  40.168  1.00123.35
C
ANISOU 9827  CA  VAL B 460    14375  19514  12980    417  -1404
-4092       C
ATOM   9828  C   VAL B 460      11.559 -38.745  38.992  1.00137.80
C
ANISOU 9828  C   VAL B 460    16283  21231  14842    680  -1557
-4275       C
ATOM   9829  O   VAL B 460      12.025 -38.233  37.969  1.00104.22
O
ANISOU 9829  O   VAL B 460    11897  17158  10542    806  -1478
-4443       O
ATOM   9830  CB  VAL B 460       9.665 -37.421  39.995  1.00102.08
C
ANISOU 9830  CB  VAL B 460    11678  16764  10345    141  -1307
-4027       C
ATOM   9831  CG1 VAL B 460       9.100 -36.945  41.324  1.00125.95
C
ANISOU 9831  CG1 VAL B 460    14677  19831  13348   -117  -1217
-3832       C
ATOM   9832  CG2 VAL B 460       9.566 -36.328  38.939  1.00100.27
C
```

```
ANISOU 9832  CG2 VAL B 460    11295  16738  10065    152  -1154 
-4165       C
ATOM   9833  N   ALA B 461      11.403 -40.059  39.143  1.00157.00 
N
ANISOU 9833  N   ALA B 461    18953  23360  17338    757  -1781 
-4245       N
ATOM   9834  CA  ALA B 461      11.811 -41.011  38.122  1.00154.43 
C
ANISOU 9834  CA  ALA B 461    18739  22896  17040   1032  -1966 
-4411       C
ATOM   9835  C   ALA B 461      10.776 -42.121  38.030  1.00157.59 
C
ANISOU 9835  C   ALA B 461    19421  22915  17540    925  -2142 
-4336       C
ATOM   9836  O   ALA B 461       9.977 -42.331  38.946  1.00194.15 
O
ANISOU 9836  O   ALA B 461    24179  27390  22199    661  -2149 
-4156       O
ATOM   9837  CB  ALA B 461      13.197 -41.602  38.416  1.00181.46 
C
ANISOU 9837  CB  ALA B 461    22190  26375  20383   1360  -2123 
-4508       C
ATOM   9838  N   ARG B 462      10.800 -42.836  36.906  1.00141.89 
N
ANISOU 9838  N   ARG B 462    17531  20786  15594   1118  -2283 
-4481       N
ATOM   9839  CA  ARG B 462       9.785 -43.839  36.619  1.00140.23 
C
ANISOU 9839  CA  ARG B 462    17582  20223  15474   1006  -2444 
-4433       C
ATOM   9840  C   ARG B 462      10.411 -45.005  35.869  1.00133.40 
C
ANISOU 9840  C   ARG B 462    16918  19162  14606   1346  -2709 
-4576       C
ATOM   9841  O   ARG B 462      11.322 -44.816  35.058  1.00129.86 
O
ANISOU 9841  O   ARG B 462    16325  18900  14117   1642  -2708 
-4764       O
ATOM   9842  CB  ARG B 462       8.649 -43.254  35.766  1.00144.61 
C
ANISOU 9842  CB  ARG B 462    18026  20809  16112    807  -2299 
-4467       C
ATOM   9843  CG  ARG B 462       7.563 -44.261  35.398  1.00147.55 
C
ANISOU 9843  CG  ARG B 462    18640  20843  16577    671  -2455 
-4438       C
ATOM   9844  CD  ARG B 462       6.410 -43.614  34.655  1.00156.02 
C
ANISOU 9844  CD  ARG B 462    19577  21979  17726    476  -2309 
-4488       C
ATOM   9845  NE  ARG B 462       6.819 -43.212  33.312  1.00168.99 
N
ANISOU 9845  NE  ARG B 462    21096  23740  19374    724  -2271 
-4685       N
```

```
ATOM   9846  CZ  ARG B 462       6.035 -42.582  32.444  1.00149.53
C
ANISOU 9846  CZ  ARG B 462    18520  21340  16955    647  -2161
-4776       C
ATOM   9847  NH1 ARG B 462       4.801 -42.241  32.787  1.00157.24
N
ANISOU 9847  NH1 ARG B 462    19457  22311  17977    344  -2076
-4706       N
ATOM   9848  NH2 ARG B 462       6.497 -42.265  31.242  1.00131.00
N
ANISOU 9848  NH2 ARG B 462    16098  19082  14593    877  -2135
-4953       N
ATOM   9849  N   GLN B 463       9.918 -46.208  36.151  1.00151.26
N
ANISOU 9849  N   GLN B 463    19524  21053  16894   1291  -2940
-4491       N
ATOM   9850  CA  GLN B 463      10.261 -47.384  35.369  1.00159.35
C
ANISOU 9850  CA  GLN B 463    20796  21832  17919   1586  -3220
-4617       C
ATOM   9851  C   GLN B 463       9.095 -48.358  35.428  1.00138.85
C
ANISOU 9851  C   GLN B 463    18539  18837  15382   1337  -3376
-4498       C
ATOM   9852  O   GLN B 463       8.139 -48.175  36.186  1.00159.69
O
ANISOU 9852  O   GLN B 463    21217  21412  18047    946  -3272
-4326       O
ATOM   9853  CB  GLN B 463      11.534 -48.073  35.875  1.00161.90
C
ANISOU 9853  CB  GLN B 463    21267  22119  18128   1939  -3447
-4682       C
ATOM   9854  CG  GLN B 463      11.363 -48.798  37.201  1.00146.99
C
ANISOU 9854  CG  GLN B 463    19717  19951  16182   1793  -3610
-4493       C
ATOM   9855  CD  GLN B 463      12.586 -49.609  37.585  1.00142.70
C
ANISOU 9855  CD  GLN B 463    19380  19325  15514   2198  -3894
-4591       C
ATOM   9856  OE1 GLN B 463      12.556 -50.382  38.543  1.00149.53
O
ANISOU 9856  OE1 GLN B 463    20604  19903  16305   2151  -4093
-4468       O
ATOM   9857  NE2 GLN B 463      13.667 -49.443  36.832  1.00151.58
N
ANISOU 9857  NE2 GLN B 463    20290  20707  16598   2602  -3922
-4828       N
ATOM   9858  N   VAL B 464       9.184 -49.399  34.611  1.00123.48
N
ANISOU 9858  N   VAL B 464    16838  16635  13445   1560  -3627
-4602       N
ATOM   9859  CA  VAL B 464       8.269 -50.527  34.689  1.00125.90
C
```

```
ANISOU 9859  CA  VAL B 464    17542  16521  13772   1360  -3835
-4501       C
ATOM   9860  C   VAL B 464       9.126 -51.777  34.802  1.00133.67
C
ANISOU 9860  C   VAL B 464    18913  17225  14650   1704  -4198
-4553       C
ATOM   9861  O   VAL B 464      10.271 -51.807  34.341  1.00147.48
O
ANISOU 9861  O   VAL B 464    20568  19120  16346   2141  -4291
-4732       O
ATOM   9862  CB  VAL B 464       7.321 -50.609  33.473  1.00131.08
C
ANISOU 9862  CB  VAL B 464    18166  17095  14545   1267  -3808
-4578       C
ATOM   9863  CG1 VAL B 464       6.438 -49.374  33.406  1.00122.08
C
ANISOU 9863  CG1 VAL B 464    16669  16226  13490    949  -3477
-4545       C
ATOM   9864  CG2 VAL B 464       8.117 -50.772  32.185  1.00133.96
C
ANISOU 9864  CG2 VAL B 464    18458  17522  14918   1715  -3911
-4805       C
ATOM   9865  N   VAL B 465       8.573 -52.813  35.421  1.00154.72
N
ANISOU 9865  N   VAL B 465    22028  19493  17264   1503  -4413
-4411       N
ATOM   9866  CA  VAL B 465       9.310 -54.067  35.544  1.00161.66
C
ANISOU 9866  CA  VAL B 465    23357  20050  18017   1831  -4802
-4460       C
ATOM   9867  C   VAL B 465       8.534 -55.201  34.882  1.00156.16
C
ANISOU 9867  C   VAL B 465    23065  18935  17333   1747  -5046
-4451       C
ATOM   9868  O   VAL B 465       7.495 -55.643  35.397  1.00159.18
O
ANISOU 9868  O   VAL B 465    23722  19052  17707   1307  -5065
-4275       O
ATOM   9869  CB  VAL B 465       9.670 -54.366  37.013  1.00140.83
C
ANISOU 9869  CB  VAL B 465    20988  17278  15241   1750  -4907
-4311       C
ATOM   9870  CG1 VAL B 465       8.452 -54.343  37.916  1.00145.62
C
ANISOU 9870  CG1 VAL B 465    21744  17728  15857   1156  -4778
-4065       C
ATOM   9871  CG2 VAL B 465      10.394 -55.706  37.120  1.00140.37
C
ANISOU 9871  CG2 VAL B 465    21456  16849  15028   2111  -5350
-4377       C
ATOM   9872  N   PRO B 466       8.986 -55.682  33.722  1.00148.98
N
ANISOU 9872  N   PRO B 466    22196  17973  16438   2142  -5230
-4644       N
```

```
ATOM   9873  CA  PRO B 466       8.226 -56.730  33.030  1.00162.49
C
ANISOU 9873  CA  PRO B 466    24288  19287  18166   2062  -5461
-4638       C
ATOM   9874  C   PRO B 466       8.315 -58.058  33.750  1.00165.86
C
ANISOU 9874  C   PRO B 466    25360  19236  18422   2067  -5844
-4541       C
ATOM   9875  O   PRO B 466       7.410 -58.891  33.614  1.00171.40
O
ANISOU 9875  O   PRO B 466    26448  19566  19111   1790  -5997
-4447       O
ATOM   9876  CB  PRO B 466       8.892 -56.789  31.649  1.00173.58
C
ANISOU 9876  CB  PRO B 466    25527  20811  19613   2551  -5550
-4887       C
ATOM   9877  CG  PRO B 466       9.399 -55.402  31.437  1.00159.99
C
ANISOU 9877  CG  PRO B 466    23214  19611  17963   2641  -5203
-4984       C
ATOM   9878  CD  PRO B 466       9.898 -54.978  32.804  1.00143.15
C
ANISOU 9878  CD  PRO B 466    21033  17616  15741   2554  -5118
-4871       C
ATOM   9879  N   GLU B 467       9.395 -58.274  34.507  1.00165.99
N
ANISOU 9879  N   GLU B 467    25520  19254  18294   2377  -6013
-4571       N
ATOM   9880  CA  GLU B 467       9.555 -59.500  35.278  1.00164.92
C
ANISOU 9880  CA  GLU B 467    26047  18650  17963   2408  -6399
-4486       C
ATOM   9881  C   GLU B 467       8.419 -59.671  36.276  1.00177.20
C
ANISOU 9881  C   GLU B 467    27883  19961  19486   1762  -6312
-4214       C
ATOM   9882  O   GLU B 467       7.999 -60.802  36.551  1.00178.41
O
ANISOU 9882  O   GLU B 467    28647  19637  19504   1600  -6607
-4116       O
ATOM   9883  CB  GLU B 467      10.904 -59.491  36.000  1.00167.28
C
ANISOU 9883  CB  GLU B 467    26374  19066  18120   2846  -6548
-4580       C
ATOM   9884  CG  GLU B 467      12.102 -59.395  35.066  1.00182.80
C
ANISOU 9884  CG  GLU B 467    28067  21306  20083   3493  -6653
-4880       C
ATOM   9885  CD  GLU B 467      12.074 -60.444  33.975  1.00196.29
C
ANISOU 9885  CD  GLU B 467    30108  22715  21760   3785  -6988
-5017       C
ATOM   9886  OE1 GLU B 467      12.043 -61.649  34.303  1.00203.62
O
```

```
ANISOU 9886  OE1 GLU B 467    31678  23164  22524   3856  -7387
-4973       O
ATOM   9887  OE2 GLU B 467      12.084 -60.061  32.785  1.00199.31
O
ANISOU 9887  OE2 GLU B 467    30130  23329  22268   3942  -6861
-5169       O
ATOM   9888  N   LYS B 468       7.926 -58.563  36.839  1.00190.89
N
ANISOU 9888  N   LYS B 468    29192  22020  21320   1385  -5917
-4098       N
ATOM   9889  CA  LYS B 468       6.815 -58.582  37.791  1.00183.71
C
ANISOU 9889  CA  LYS B 468    28454  20965  20381    747  -5782
-3859       C
ATOM   9890  C   LYS B 468       7.192 -59.324  39.071  1.00186.53
C
ANISOU 9890  C   LYS B 468    29357  20996  20520    695  -6026
-3724       C
ATOM   9891  O   LYS B 468       6.414 -60.115  39.607  1.00180.57
O
ANISOU 9891  O   LYS B 468    29095  19864  19648    276  -6154
-3562       O
ATOM   9892  CB  LYS B 468       5.542 -59.157  37.164  1.00177.10
C
ANISOU 9892  CB  LYS B 468    27815  19881  19596    352  -5809
-3808       C
ATOM   9893  CG  LYS B 468       5.024 -58.337  35.990  1.00164.87
C
ANISOU 9893  CG  LYS B 468    25723  18658  18263    347  -5542
-3931       C
ATOM   9894  CD  LYS B 468       3.863 -59.018  35.285  1.00161.41
C
ANISOU 9894  CD  LYS B 468    25503  17957  17868     27  -5618
-3915       C
ATOM   9895  CE  LYS B 468       2.780 -59.436  36.268  1.00178.16
C
ANISOU 9895  CE  LYS B 468    27935  19864  19893   -626  -5584
-3706       C
ATOM   9896  NZ  LYS B 468       1.558 -59.928  35.573  1.00169.98
N
ANISOU 9896  NZ  LYS B 468    27010  18661  18912   -996  -5598
-3708       N
ATOM   9897  N   THR B 469       8.401 -59.057  39.565  1.00191.54
N
ANISOU 9897  N   THR B 469    29910  21782  21084   1115  -6092
-3800       N
ATOM   9898  CA  THR B 469       8.929 -59.689  40.765  1.00170.76
C
ANISOU 9898  CA  THR B 469    27778  18864  18238   1163  -6342
-3706       C
ATOM   9899  C   THR B 469       9.638 -58.631  41.597  1.00151.18
C
ANISOU 9899  C   THR B 469    24881  16779  15780   1267  -6106
-3697       C
```

```
ATOM   9900  O   THR B 469      10.332 -57.764  41.058  1.00148.48
O
ANISOU 9900  O   THR B 469    23995  16866  15553   1601  -5934
-3861       O
ATOM   9901  CB  THR B 469       9.898 -60.835  40.437  1.00176.48
C
ANISOU 9901  CB  THR B 469    29005  19256  18794   1732  -6835
-3868       C
ATOM   9902  OG1 THR B 469      10.870 -60.387  39.484  1.00201.69
O
ANISOU 9902  OG1 THR B 469    31749  22804  22081   2309  -6825
-4130       O
ATOM   9903  CG2 THR B 469       9.144 -62.034  39.871  1.00173.81
C
ANISOU 9903  CG2 THR B 469    29235  18424  18380   1564  -7121
-3829       C
ATOM   9904  N   LYS B 470       9.440 -58.708  42.916  1.00165.81
N
ANISOU 9904  N   LYS B 470    27007  18481  17511    952  -6095
-3504       N
ATOM   9905  CA  LYS B 470       9.916 -57.667  43.824  1.00176.23
C
ANISOU 9905  CA  LYS B 470    27944  20159  18856    948  -5841
-3454       C
ATOM   9906  C   LYS B 470      11.431 -57.494  43.763  1.00181.78
C
ANISOU 9906  C   LYS B 470    28487  21067  19515   1609  -5988
-3668       C
ATOM   9907  O   LYS B 470      11.934 -56.367  43.842  1.00166.78
O
ANISOU 9907  O   LYS B 470    26025  19629  17714   1725  -5713
-3728       O
ATOM   9908  CB  LYS B 470       9.459 -57.973  45.251  1.00172.92
C
ANISOU 9908  CB  LYS B 470    27947  19472  18284    515  -5862
-3213       C
ATOM   9909  CG  LYS B 470       7.948 -57.899  45.427  1.00168.88
C
ANISOU 9909  CG  LYS B 470    27456  18894  17818   -197  -5629
-3015       C
ATOM   9910  CD  LYS B 470       7.547 -57.903  46.892  1.00180.57
C
ANISOU 9910  CD  LYS B 470    29205  20244  19159   -646  -5556
-2786       C
ATOM   9911  CE  LYS B 470       7.988 -59.181  47.584  1.00203.69
C
ANISOU 9911  CE  LYS B 470    32948  22624  21822   -546  -5989
-2731       C
ATOM   9912  NZ  LYS B 470       7.532 -59.222  49.001  1.00220.25
N
ANISOU 9912  NZ  LYS B 470    35350  24569  23766  -1029  -5913
-2499       N
ATOM   9913  N   GLU B 471      12.176 -58.588  43.624  1.00191.77
N
```

```
ANISOU 9913  N   GLU B 471    30238  22007  20618   2047  -6428
-3798       N
ATOM   9914  CA  GLU B 471      13.631 -58.525  43.679  1.00193.26
C
ANISOU 9914  CA  GLU B 471    30310  22389  20730   2685  -6606
-4026       C
ATOM   9915  C   GLU B 471      14.276 -58.295  42.317  1.00210.27
C
ANISOU 9915  C   GLU B 471    32046  24860  22986   3153  -6606
-4309       C
ATOM   9916  O   GLU B 471      15.506 -58.336  42.215  1.00225.29
O
ANISOU 9916  O   GLU B 471    33841  26948  24811   3712  -6779
-4545       O
ATOM   9917  CB  GLU B 471      14.194 -59.804  44.306  1.00172.87
C
ANISOU 9917  CB  GLU B 471    28478  19317  17888   2978  -7112
-4057       C
ATOM   9918  CG  GLU B 471      13.821 -60.001  45.766  1.00165.09
C
ANISOU 9918  CG  GLU B 471    27925  18041  16762   2591  -7136
-3807       C
ATOM   9919  CD  GLU B 471      14.520 -61.194  46.390  1.00193.44
C
ANISOU 9919  CD  GLU B 471    32268  21158  20071   2950  -7657
-3867       C
ATOM   9920  OE1 GLU B 471      15.765 -61.173  46.486  1.00193.98
O
ANISOU 9920  OE1 GLU B 471    32245  21396  20064   3548  -7849
-4096       O
ATOM   9921  OE2 GLU B 471      13.826 -62.155  46.785  1.00209.15
O
ANISOU 9921  OE2 GLU B 471    34956  22612  21900   2631  -7884
-3699       O
ATOM   9922  N   SER B 472      13.482 -58.057  41.271  1.00193.09
N
ANISOU 9922  N   SER B 472    29631  22763  20971   2941  -6419
-4305       N
ATOM   9923  CA  SER B 472      14.077 -57.707  39.986  1.00173.04
C
ANISOU 9923  CA  SER B 472    26657  20561  18530   3347  -6370
-4565       C
ATOM   9924  C   SER B 472      14.109 -56.195  39.836  1.00178.78
C
ANISOU 9924  C   SER B 472    26660  21848  19421   3199  -5897
-4574       C
ATOM   9925  O   SER B 472      13.067 -55.544  40.007  1.00196.18
O
ANISOU 9925  O   SER B 472    28686  24111  21744   2687  -5586
-4381       O
ATOM   9926  CB  SER B 472      13.287 -58.327  38.837  1.00167.29
C
ANISOU 9926  CB  SER B 472    26099  19592  17873   3263  -6465
-4585       C
```

```
ATOM   9927  OG  SER B 472      13.944 -58.131  37.596  1.00149.36
O
ANISOU 9927  OG  SER B 472    23479  17605  15666   3702  -6469
-4852       O
ATOM   9928  N   PRO B 473      15.268 -55.593  39.541  1.00157.60
N
ANISOU 9928  N   PRO B 473    23557  19592  16731   3621  -5835
-4802       N
ATOM   9929  CA  PRO B 473      15.280 -54.341  38.769  1.00170.37
C
ANISOU 9929  CA  PRO B 473    24529  21705  18498   3555  -5443
-4885       C
ATOM   9930  C   PRO B 473      14.315 -54.347  37.593  1.00167.40
C
ANISOU 9930  C   PRO B 473    24089  21256  18261   3356  -5338
-4875       C
ATOM   9931  O   PRO B 473      14.179 -55.356  36.894  1.00165.62
O
ANISOU 9931  O   PRO B 473    24194  20726  18008   3536  -5621
-4953       O
ATOM   9932  CB  PRO B 473      16.740 -54.232  38.307  1.00162.33
C
ANISOU 9932  CB  PRO B 473    23241  21039  17397   4138  -5546
-5202       C
ATOM   9933  CG  PRO B 473      17.503 -54.987  39.353  1.00159.79
C
ANISOU 9933  CG  PRO B 473    23296  20519  16897   4419  -5878
-5230       C
ATOM   9934  CD  PRO B 473      16.620 -56.157  39.682  1.00147.05
C
ANISOU 9934  CD  PRO B 473    22349  18293  15231   4218  -6163
-5041       C
ATOM   9935  N   GLY B 474      13.645 -53.219  37.365  1.00138.19
N
ANISOU 9935  N   GLY B 474    19978  17828  14701   3002  -4948
-4789       N
ATOM   9936  CA  GLY B 474      12.855 -53.022  36.170  1.00161.96
C
ANISOU 9936  CA  GLY B 474    22839  20855  17843   2867  -4817
-4825       C
ATOM   9937  C   GLY B 474      13.685 -52.636  34.959  1.00173.43
C
ANISOU 9937  C   GLY B 474    23942  22640  19313   3261  -4768
-5098       C
ATOM   9938  O   GLY B 474      14.911 -52.755  34.927  1.00156.04
O
ANISOU 9938  O   GLY B 474    21655  20626  17005   3689  -4897
-5293       O
ATOM   9939  N   ALA B 475      12.977 -52.167  33.936  1.00170.50
N
ANISOU 9939  N   ALA B 475    23366  22351  19067   3104  -4579
-5123       N
ATOM   9940  CA  ALA B 475      13.622 -51.518  32.805  1.00148.38
C
```

ANISOU 9940 CA ALA B 475   20179 19915 16283   3367 -4439
-5355   C
ATOM   9941 C   ALA B 475   14.313 -50.232 33.257 1.00156.70
C
ANISOU 9941 C   ALA B 475   20797 21442 17301   3333 -4140
-5386   C
ATOM   9942 O   ALA B 475   13.828 -49.561 34.175 1.00176.53
O
ANISOU 9942 O   ALA B 475   23231 24003 19838   2985 -3943
-5192   O
ATOM   9943 CB ALA B 475   12.597 -51.214 31.710 1.00136.96
C
ANISOU 9943 CB ALA B 475   18638 18428 14973   3157 -4288
-5352   C
ATOM   9944 N   PRO B 476   15.473 -49.889 32.666 1.00156.51
N
ANISOU 9944 N   PRO B 476   20490 21779 17199   3685 -4108
-5633   N
ATOM   9945 CA PRO B 476   16.164 -48.627 32.987 1.00157.21
C
ANISOU 9945 CA PRO B 476   20158 22341 17236   3633 -3813
-5681   C
ATOM   9946 C   PRO B 476   15.270 -47.417 33.237 1.00164.59
C
ANISOU 9946 C   PRO B 476   20892 23392 18255   3168 -3462
-5493   C
ATOM   9947 O   PRO B 476   14.398 -47.094 32.424 1.00181.11
O
ANISOU 9947 O   PRO B 476   22945 25429 20441   2981 -3336
-5471   O
ATOM   9948 CB PRO B 476   17.052 -48.415 31.755 1.00164.97
C
ANISOU 9948 CB PRO B 476   20872 23650 18157   3959 -3778
-5980   C
ATOM   9949 CG PRO B 476   17.408 -49.805 31.348 1.00171.11
C
ANISOU 9949 CG PRO B 476   21953 24168 18893   4360 -4167
-6123   C
ATOM   9950 CD PRO B 476   16.156 -50.623 31.586 1.00165.98
C
ANISOU 9950 CD PRO B 476   21733 22986 18348   4128 -4332
-5894   C
ATOM   9951 N   TRP B 477   15.483 -46.761 34.378 1.00153.60
N
ANISOU 9951 N   TRP B 477   19385 22156 16821   3002 -3323
-5368   N
ATOM   9952 CA TRP B 477   14.624 -45.670 34.828 1.00160.06
C
ANISOU 9952 CA TRP B 477   20053 23063 17700   2574 -3028
-5176   C
ATOM   9953 C   TRP B 477   14.548 -44.512 33.838 1.00164.77
C
ANISOU 9953 C   TRP B 477   20334 23967 18304   2484 -2749
-5280   C

```
ATOM   9954  O   TRP B 477      15.528 -44.162  33.176  1.00170.04
O
ANISOU 9954  O   TRP B 477    20785  24937  18884   2712  -2691
-5491       O
ATOM   9955  CB  TRP B 477      15.118 -45.136  36.173  1.00190.95
C
ANISOU 9955  CB  TRP B 477    23870  27140  21541   2483  -2939
-5063       C
ATOM   9956  CG  TRP B 477      15.107 -46.125  37.288  1.00186.04
C
ANISOU 9956  CG  TRP B 477    23584  26207  20896   2513  -3186
-4932       C
ATOM   9957  CD1 TRP B 477      16.091 -47.008  37.623  1.00170.41
C
ANISOU 9957  CD1 TRP B 477    21770  24159  18820   2877  -3462
-5046       C
ATOM   9958  CD2 TRP B 477      14.073 -46.286  38.262  1.00159.87
C
ANISOU 9958  CD2 TRP B 477    20491  22621  17632   2156  -3179
-4670       C
ATOM   9959  NE1 TRP B 477      15.717 -47.733  38.730  1.00158.45
N
ANISOU 9959  NE1 TRP B 477    20612  22304  17289   2766  -3639
-4859       N
ATOM   9960  CE2 TRP B 477      14.483 -47.305  39.143  1.00152.51
C
ANISOU 9960  CE2 TRP B 477    19891  21430  16625   2305  -3457
-4622       C
ATOM   9961  CE3 TRP B 477      12.833 -45.674  38.467  1.00156.34
C
ANISOU 9961  CE3 TRP B 477    19996  22135  17270   1728  -2971
-4487       C
ATOM   9962  CZ2 TRP B 477      13.697 -47.725  40.210  1.00157.74
C
ANISOU 9962  CZ2 TRP B 477    20852  21789  17291   2006  -3517
-4384       C
ATOM   9963  CZ3 TRP B 477      12.057 -46.092  39.524  1.00167.34
C
ANISOU 9963  CZ3 TRP B 477    21643  23266  18675   1441  -3024
-4267       C
ATOM   9964  CH2 TRP B 477      12.492 -47.103  40.385  1.00163.12
C
ANISOU 9964  CH2 TRP B 477    21450  22469  18060   1563  -3286
-4209       C
ATOM   9965  N   GLU B 478      13.356 -43.923  33.747  1.00166.07
N
ANISOU 9965  N   GLU B 478    20488  24054  18557   2143  -2582
-5142       N
ATOM   9966  CA  GLU B 478      13.127 -42.659  33.058  1.00164.93
C
ANISOU 9966  CA  GLU B 478    20093  24174  18400   1995  -2306
-5197       C
ATOM   9967  C   GLU B 478      13.347 -41.513  34.040  1.00163.40
C
```

```
ANISOU 9967  C   GLU B 478    19705  24245  18133   1791  -2085
-5087       C
ATOM   9968  O   GLU B 478      12.764 -41.509  35.128  1.00160.96
O
ANISOU 9968  O   GLU B 478    19480  23817  17861   1573  -2078
-4887       O
ATOM   9969  CB  GLU B 478      11.709 -42.596  32.487  1.00164.34
C
ANISOU 9969  CB  GLU B 478    20113  23886  18443   1762  -2265
-5130       C
ATOM   9970  CG  GLU B 478      11.443 -43.554  31.339  1.00167.39
C
ANISOU 9970  CG  GLU B 478    20666  24035  18901   1947  -2452
-5253       C
ATOM   9971  CD  GLU B 478       9.978 -43.596  30.951  1.00170.86
C
ANISOU 9971  CD  GLU B 478    21210  24248  19462   1699  -2434
-5177       C
ATOM   9972  OE1 GLU B 478       9.630 -43.054  29.881  1.00165.50
O
ANISOU 9972  OE1 GLU B 478    20441  23639  18802   1701  -2330
-5292       O
ATOM   9973  OE2 GLU B 478       9.174 -44.166  31.718  1.00184.05
O
ANISOU 9973  OE2 GLU B 478    23055  25679  21195   1495  -2524
-5015       O
ATOM   9974  N   PHE B 479      14.180 -40.545  33.663  1.00164.77
N
ANISOU 9974  N   PHE B 479    19632  24780  18193   1845  -1904
-5216       N
ATOM   9975  CA  PHE B 479      14.374 -39.344  34.478  1.00161.97
C
ANISOU 9975  CA  PHE B 479    19099  24686  17754   1637  -1683
-5120       C
ATOM   9976  C   PHE B 479      13.339 -38.307  34.059  1.00146.13
C
ANISOU 9976  C   PHE B 479    17056  22701  15765   1361  -1487
-5060       C
ATOM   9977  O   PHE B 479      13.502 -37.616  33.051  1.00146.04
O
ANISOU 9977  O   PHE B 479    16951  22854  15682   1372  -1361
-5201       O
ATOM   9978  CB  PHE B 479      15.796 -38.817  34.316  1.00164.52
C
ANISOU 9978  CB  PHE B 479    19193  25395  17920   1803  -1590
-5295       C
ATOM   9979  CG  PHE B 479      16.183 -37.774  35.328  1.00147.71
C
ANISOU 9979  CG  PHE B 479    16907  23521  15696   1626  -1410
-5193       C
ATOM   9980  CD1 PHE B 479      16.962 -38.110  36.423  1.00132.84
C
ANISOU 9980  CD1 PHE B 479    14993  21704  13775   1734  -1496
-5155       C
```

```
ATOM   9981  CD2 PHE B 479      15.784 -36.455  35.175  1.00136.23
C
ANISOU 9981  CD2 PHE B 479    15357  22229  14175   1365  -1171
-5145       C
ATOM   9982  CE1 PHE B 479      17.327 -37.153  37.353  1.00114.47
C
ANISOU 9982  CE1 PHE B 479    12522  19609  11361   1575  -1334
-5062       C
ATOM   9983  CE2 PHE B 479      16.144 -35.493  36.101  1.00124.45
C
ANISOU 9983  CE2 PHE B 479    13739  20961  12584   1205  -1018
-5049       C
ATOM   9984  CZ  PHE B 479      16.917 -35.842  37.192  1.00120.33
C
ANISOU 9984  CZ  PHE B 479    13167  20512  12041   1305  -1093
-5004       C
ATOM   9985  N   VAL B 480      12.265 -38.200  34.845  1.00159.10
N
ANISOU 9985  N   VAL B 480    20076  25181  15194   2397  -2159
-6115       N
ATOM   9986  CA  VAL B 480      11.078 -37.470  34.408  1.00141.29
C
ANISOU 9986  CA  VAL B 480    17755  23182  12747   2200  -2058
-5863       C
ATOM   9987  C   VAL B 480      11.247 -35.958  34.560  1.00129.60
C
ANISOU 9987  C   VAL B 480    16150  22005  11088   2151  -1823
-5449       C
ATOM   9988  O   VAL B 480      10.786 -35.186  33.712  1.00107.59
O
ANISOU 9988  O   VAL B 480    13190  19624   8065   2111  -1687
-5260       O
ATOM   9989  CB  VAL B 480       9.847 -37.990  35.171  1.00119.24
C
ANISOU 9989  CB  VAL B 480    15218  19981  10109   1956  -2235
-5844       C
ATOM   9990  CG1 VAL B 480       8.636 -37.133  34.877  1.00104.96
C
ANISOU 9990  CG1 VAL B 480    13322  18450   8109   1772  -2129
-5557       C
ATOM   9991  CG2 VAL B 480       9.578 -39.445  34.807  1.00118.70
C
ANISOU 9991  CG2 VAL B 480    15274  19650  10176   1966  -2470
-6234       C
ATOM   9992  N   GLY B 481      11.917 -35.509  35.612  1.00116.68
N
ANISOU 9992  N   GLY B 481    14607  20170   9557   2155  -1783
-5299       N
ATOM   9993  CA  GLY B 481      11.965 -34.092  35.917  1.00 99.41
C
ANISOU 9993  CA  GLY B 481    12362  18183   7226   2074  -1589
-4893       C
ATOM   9994  C   GLY B 481      12.235 -33.852  37.388  1.00 96.44
C
```

```
ANISOU 9994  C   GLY B 481    12182  17429   7033   2009  -1619
-4757       C
ATOM   9995  O   GLY B 481      12.351 -34.783  38.185  1.00119.57
O
ANISOU 9995  O   GLY B 481    15295  19936  10200   2014  -1798
-4960       O
ATOM   9996  N   LEU B 482      12.344 -32.567  37.733  1.00 94.17
N
ANISOU 9996  N   LEU B 482    11863  17291   6628   1942  -1452
-4399       N
ATOM   9997  CA  LEU B 482      12.631 -32.177  39.108  1.00 91.35
C
ANISOU 9997  CA  LEU B 482    11665  16604   6439   1871  -1450
-4223       C
ATOM   9998  C   LEU B 482      11.994 -30.828  39.433  1.00 98.36
C
ANISOU 9998  C   LEU B 482    12563  17530   7281   1720  -1301
-3760       C
ATOM   9999  O   LEU B 482      11.944 -29.934  38.583  1.00 89.77
O
ANISOU 9999  O   LEU B 482    11338  16841   5927   1739  -1169
-3582       O
ATOM  10000  CB  LEU B 482      14.149 -32.176  39.351  1.00109.48
C
ANISOU10000  CB  LEU B 482    13897  18914   8786   2006  -1388
-4297       C
ATOM  10001  CG  LEU B 482      15.096 -31.337  38.489  1.00 97.52
C
ANISOU10001  CG  LEU B 482    12146  17918   6991   2085  -1190
-4204       C
ATOM  10002  CD1 LEU B 482      15.160 -29.895  38.961  1.00103.81
C
ANISOU10002  CD1 LEU B 482    12956  18728   7759   1926  -1008
-3722       C
ATOM  10003  CD2 LEU B 482      16.484 -31.963  38.451  1.00 94.71
C
ANISOU10003  CD2 LEU B 482    11678  17609   6699   2267  -1205
-4477       C
ATOM  10004  N   LEU B 483      11.499 -30.699  40.673  1.00 96.08
N
ANISOU10004  N   LEU B 483    12439  16821   7244   1576  -1338
-3572       N
ATOM  10005  CA  LEU B 483      10.597 -29.664  41.175  1.00 94.44
C
ANISOU10005  CA  LEU B 483    12276  16558   7050   1444  -1261
-3211       C
ATOM  10006  C   LEU B 483      11.244 -28.793  42.256  1.00 82.45
C
ANISOU10006  C   LEU B 483    10832  14796   5698   1387  -1152
-2922       C
ATOM  10007  O   LEU B 483      12.162 -29.237  42.953  1.00 81.93
O
ANISOU10007  O   LEU B 483    10824  14492   5812   1400  -1178
-3011       O
```

```
ATOM  10008  CB  LEU B 483       9.308 -30.295  41.721  1.00 90.28
C
ANISOU10008  CB  LEU B 483    11852  15806   6646   1304  -1407
-3272       C
ATOM  10009  CG  LEU B 483       9.371 -31.469  42.704  1.00 95.88
C
ANISOU10009  CG  LEU B 483    12726  16076   7628   1208  -1577
-3460       C
ATOM  10010  CD1 LEU B 483       9.702 -31.033  44.127  1.00181.71
C
ANISOU10010  CD1 LEU B 483    23713  26596  18732   1108  -1534
-3225       C
ATOM  10011  CD2 LEU B 483       8.057 -32.240  42.671  1.00 86.57
C
ANISOU10011  CD2 LEU B 483    11594  14851   6448   1056  -1734
-3592       C
ATOM  10012  N   PRO B 484      10.781 -27.532  42.410  1.00 81.31
N
ANISOU10012  N   PRO B 484    10692  14707   5496   1334  -1042
-2583       N
ATOM  10013  CA  PRO B 484      11.361 -26.620  43.412  1.00100.90
C
ANISOU10013  CA  PRO B 484    13252  16958   8128   1272   -943
-2316       C
ATOM  10014  C   PRO B 484      10.550 -26.513  44.704  1.00 89.80
C
ANISOU10014  C   PRO B 484    11973  15207   6939   1154   -994
-2202       C
ATOM  10015  O   PRO B 484       9.323 -26.377  44.659  1.00100.32
O
ANISOU10015  O   PRO B 484    13302  16593   8223   1123  -1039
-2157       O
ATOM  10016  CB  PRO B 484      11.388 -25.286  42.664  1.00110.42
C
ANISOU10016  CB  PRO B 484    14400  18441   9115   1299   -814
-2029       C
ATOM  10017  CG  PRO B 484      10.153 -25.341  41.821  1.00 81.28
C
ANISOU10017  CG  PRO B 484    10652  14989   5243   1342   -874
-2058       C
ATOM  10018  CD  PRO B 484       9.975 -26.797  41.419  1.00 82.42
C
ANISOU10018  CD  PRO B 484    10745  15190   5380   1371   -998
-2440       C
ATOM  10019  N   LEU B 485      11.222 -26.577  45.859  1.00 75.87
N
ANISOU10019  N   LEU B 485    10299  13132   5395   1088   -989
-2165       N
ATOM  10020  CA  LEU B 485      10.579 -26.573  47.174  1.00 74.14
C
ANISOU10020  CA  LEU B 485    10187  12610   5374    961  -1037
-2080       C
ATOM  10021  C   LEU B 485      11.044 -25.372  47.998  1.00 72.64
C
```

```
ANISOU10021  C   LEU B 485    10047  12282   5270    927   -920
-1816       C
ATOM  10022  O   LEU B 485      12.248 -25.195  48.209  1.00 72.91
O
ANISOU10022  O   LEU B 485    10088  12255   5361    937   -858
-1788       O
ATOM  10023  CB  LEU B 485      10.913 -27.877  47.901  1.00 74.02
C
ANISOU10023  CB  LEU B 485    10255  12322   5548    893  -1173
-2289       C
ATOM  10024  CG  LEU B 485      10.272 -29.148  47.337  1.00 75.65
C
ANISOU10024  CG  LEU B 485    10466  12559   5717    879  -1334
-2558       C
ATOM  10025  CD1 LEU B 485      11.190 -30.342  47.528  1.00 76.54
C
ANISOU10025  CD1 LEU B 485    10657  12457   5969    920  -1463
-2798       C
ATOM  10026  CD2 LEU B 485       8.947 -29.416  48.003  1.00150.80
C
ANISOU10026  CD2 LEU B 485    20036  21975  15286    699  -1423
-2521       C
ATOM  10027  N   PHE B 486      10.094 -24.552  48.467  1.00 71.89
N
ANISOU10027  N   PHE B 486     9981  12153   5183    894   -900
-1644       N
ATOM  10028  CA  PHE B 486      10.391 -23.381  49.295  1.00 70.74
C
ANISOU10028  CA  PHE B 486     9903  11847   5128    868   -812
-1418       C
ATOM  10029  C   PHE B 486      10.429 -23.773  50.773  1.00 69.19
C
ANISOU10029  C   PHE B 486     9778  11365   5146    742   -854
-1448       C
ATOM  10030  O   PHE B 486       9.601 -24.566  51.229  1.00 83.34
O
ANISOU10030  O   PHE B 486    11571  13112   6983    661   -952
-1559       O
ATOM  10031  CB  PHE B 486       9.352 -22.288  49.044  1.00 71.18
C
ANISOU10031  CB  PHE B 486     9955  12009   5080    940   -791
-1248       C
ATOM  10032  CG  PHE B 486       9.698 -20.966  49.656  1.00 70.64
C
ANISOU10032  CG  PHE B 486     9978  11777   5084    946   -715
-1023       C
ATOM  10033  CD1 PHE B 486      11.019 -20.578  49.792  1.00 70.40
C
ANISOU10033  CD1 PHE B 486     9997  11645   5108    894   -635
-934       C
ATOM  10034  CD2 PHE B 486       8.704 -20.082  50.032  1.00 93.75
C
ANISOU10034  CD2 PHE B 486    12936  14672   8013   1013   -734
-915       C
```

```
ATOM  10035  CE1 PHE B 486      11.345 -19.349  50.338  1.00 70.21
C
ANISOU10035  CE1 PHE B 486    10075  11450   5151    874   -579
-733       C
ATOM  10036  CE2 PHE B 486       9.022 -18.848  50.572  1.00134.65
C
ANISOU10036  CE2 PHE B 486    18226  19667  13268   1033   -687
-730       C
ATOM  10037  CZ  PHE B 486      10.344 -18.483  50.728  1.00 84.52
C
ANISOU10037  CZ  PHE B 486    11948  13180   6983    947   -611
-633       C
ATOM  10038  N   ASP B 487      11.394 -23.211  51.522  1.00105.78
N
ANISOU10038  N   ASP B 487    14469  15826   9896    703   -784
-1341       N
ATOM  10039  CA  ASP B 487      11.650 -23.696  52.881  1.00116.33
C
ANISOU10039  CA  ASP B 487    15866  16915  11421    583   -829
-1379       C
ATOM  10040  C   ASP B 487      10.475 -23.629  53.862  1.00 75.39
C
ANISOU10040  C   ASP B 487    10698  11658   6289    492   -876
-1354       C
ATOM  10041  O   ASP B 487      10.217 -24.647  54.527  1.00 66.12
O
ANISOU10041  O   ASP B 487     9545  10383   5192    370   -976
-1457       O
ATOM  10042  CB  ASP B 487      12.870 -22.961  53.451  1.00141.25
C
ANISOU10042  CB  ASP B 487    19061  19945  14664    558   -737
-1261       C
ATOM  10043  CG  ASP B 487      14.180 -23.637  53.089  1.00142.87
C
ANISOU10043  CG  ASP B 487    19228  20176  14879    587   -739
-1371       C
ATOM  10044  OD1 ASP B 487      15.236 -22.977  53.180  1.00143.73
O
ANISOU10044  OD1 ASP B 487    19326  20288  14997    574   -648
-1278       O
ATOM  10045  OD2 ASP B 487      14.154 -24.830  52.717  1.00145.43
O
ANISOU10045  OD2 ASP B 487    19530  20525  15200    625   -840
-1563       O
ATOM  10046  N   PRO B 488       9.718 -22.514  54.014  1.00 66.29
N
ANISOU10046  N   PRO B 488     9537  10562   5089    542   -826
-1232       N
ATOM  10047  CA  PRO B 488      10.060 -21.085  54.096  1.00 66.24
C
ANISOU10047  CA  PRO B 488     9580  10500   5087    618   -732
-1058       C
ATOM  10048  C   PRO B 488      10.367 -20.683  55.567  1.00 72.21
C
```

```
ANISOU10048  C   PRO B 488    10394  11043   5999    520   -710
-1016       C
ATOM  10049  O   PRO B 488      10.032 -21.454  56.466  1.00104.11
O
ANISOU10049  O   PRO B 488    14415  15035  10106    403   -769
-1104       O
ATOM  10050  CB  PRO B 488       8.791 -20.417  53.584  1.00 67.23
C
ANISOU10050  CB  PRO B 488     9665  10791   5088    744   -750
-1014       C
ATOM  10051  CG  PRO B 488       7.714 -21.276  54.148  1.00 77.63
C
ANISOU10051  CG  PRO B 488    10904  12188   6404    661   -832
-1152       C
ATOM  10052  CD  PRO B 488       8.256 -22.701  54.098  1.00104.10
C
ANISOU10052  CD  PRO B 488    14258  15492   9804    528   -890
-1286       C
ATOM  10053  N   PRO B 489      10.988 -19.519  55.804  1.00 81.52
N
ANISOU10053  N   PRO B 489    11648  12104   7223    546   -637
-881       N
ATOM  10054  CA  PRO B 489      11.267 -19.087  57.187  1.00 64.18
C
ANISOU10054  CA  PRO B 489     9501   9724   5162    457   -618
-861       C
ATOM  10055  C   PRO B 489      10.046 -18.956  58.093  1.00 92.42
C
ANISOU10055  C   PRO B 489    13037  13332   8746    459   -662
-922       C
ATOM  10056  O   PRO B 489       8.956 -18.583  57.654  1.00102.42
O
ANISOU10056  O   PRO B 489    14261  14738   9917    583   -688
-932       O
ATOM  10057  CB  PRO B 489      11.945 -17.725  56.994  1.00 64.81
C
ANISOU10057  CB  PRO B 489     9680   9689   5256    500   -548
-705       C
ATOM  10058  CG  PRO B 489      11.358 -17.208  55.739  1.00 66.21
C
ANISOU10058  CG  PRO B 489     9866   9996   5294    645   -556
-625       C
ATOM  10059  CD  PRO B 489      11.254 -18.429  54.847  1.00129.97
C
ANISOU10059  CD  PRO B 489    17837  18274  13272    650   -584
-729       C
ATOM  10060  N   ARG B 490      10.247 -19.276  59.378  1.00 90.40
N
ANISOU10060  N   ARG B 490    12779  12984   8587    321   -672
-969       N
ATOM  10061  CA  ARG B 490       9.301 -18.897  60.427  1.00 63.34
C
ANISOU10061  CA  ARG B 490     9303   9606   5157    309   -689
-1021       C
```

```
ATOM  10062  C   ARG B 490       9.338 -17.390  60.675  1.00 66.06
C
ANISOU10062  C   ARG B 490     9721   9842   5536    442   -646
-964       C
ATOM  10063  O   ARG B 490      10.348 -16.722  60.438  1.00114.86
O
ANISOU10063  O   ARG B 490    16012  15849  11780    451   -599
-864       O
ATOM  10064  CB  ARG B 490       9.597 -19.636  61.732  1.00 62.48
C
ANISOU10064  CB  ARG B 490     9174   9445   5121    104   -715
-1068       C
ATOM  10065  CG  ARG B 490       9.635 -21.145  61.621  1.00 62.23
C
ANISOU10065  CG  ARG B 490     9119   9444   5081    -43   -794
-1114       C
ATOM  10066  CD  ARG B 490       9.893 -21.774  62.982  1.00 75.93
C
ANISOU10066  CD  ARG B 490    10858  11114   6879   -251   -839
-1122       C
ATOM  10067  NE  ARG B 490       9.824 -23.232  62.944  1.00 95.59
N
ANISOU10067  NE  ARG B 490    13362  13591   9367   -404   -953
-1154       N
ATOM  10068  CZ  ARG B 490       8.743 -23.936  63.267  1.00 86.20
C
ANISOU10068  CZ  ARG B 490    12112  12548   8093   -558  -1031
-1191       C
ATOM  10069  NH1 ARG B 490       7.629 -23.312  63.622  1.00 76.52
N
ANISOU10069  NH1 ARG B 490    10767  11548   6759   -553   -993
-1220       N
ATOM  10070  NH2 ARG B 490       8.767 -25.261  63.207  1.00 72.11
N
ANISOU10070  NH2 ARG B 490    10384  10691   6323   -715  -1158
-1207       N
ATOM  10071  N   HIS B 491       8.218 -16.854  61.165  1.00 64.81
N
ANISOU10071  N   HIS B 491     9499   9795   5330    543   -672
-1038       N
ATOM  10072  CA  HIS B 491       8.182 -15.459  61.604  1.00 82.58
C
ANISOU10072  CA  HIS B 491    11834  11911   7633    684   -662
-1028       C
ATOM  10073  C   HIS B 491       9.128 -15.190  62.776  1.00 85.86
C
ANISOU10073  C   HIS B 491    12313  12140   8170    541   -623
-1034       C
ATOM  10074  O   HIS B 491       9.861 -14.193  62.781  1.00 97.95
O
ANISOU10074  O   HIS B 491    13987  13446   9783    577   -600
-960       O
ATOM  10075  CB  HIS B 491       6.741 -15.056  61.945  1.00 82.63
C
```

```
ANISOU10075  CB  HIS B 491    11721  12126   7548    852   -712
-1159       C
ATOM  10076  CG  HIS B 491       6.259 -15.550  63.275  1.00110.10
C
ANISOU10076  CG  HIS B 491    15056  15776  11003    712   -713
-1300       C
ATOM  10077  ND1 HIS B 491       5.808 -16.836  63.474  1.00119.40
N
ANISOU10077  ND1 HIS B 491    16093  17177  12097    516   -731
-1345       N
ATOM  10078  CD2 HIS B 491       6.148 -14.925  64.471  1.00125.68
C
ANISOU10078  CD2 HIS B 491    17005  17743  13007    725   -706
-1404       C
ATOM  10079  CE1 HIS B 491       5.445 -16.984  64.736  1.00109.54
C
ANISOU10079  CE1 HIS B 491    14736  16070  10816    393   -731
-1445       C
ATOM  10080  NE2 HIS B 491       5.641 -15.839  65.363  1.00122.21
N
ANISOU10080  NE2 HIS B 491    16394  17557  12483    528   -709
-1495       N
ATOM  10081  N   ASP B 492       9.120 -16.069  63.782  1.00 97.36
N
ANISOU10081  N   ASP B 492    13671  13691   9630    357   -625
-1111       N
ATOM  10082  CA  ASP B 492       9.908 -15.847  64.995  1.00102.14
C
ANISOU10082  CA  ASP B 492    14313  14170  10327    221   -597
-1130       C
ATOM  10083  C   ASP B 492      11.412 -15.947  64.756  1.00122.67
C
ANISOU10083  C   ASP B 492    17020  16566  13026    111   -558
-1017       C
ATOM  10084  O   ASP B 492      12.186 -15.178  65.338  1.00163.69
O
ANISOU10084  O   ASP B 492    22294  21598  18303     70   -526
-1000       O
ATOM  10085  CB  ASP B 492       9.467 -16.816  66.098  1.00 87.30
C
ANISOU10085  CB  ASP B 492    12297  12476   8395     37   -625
-1214       C
ATOM  10086  CG  ASP B 492       9.791 -18.269  65.784  1.00 89.39
C
ANISOU10086  CG  ASP B 492    12543  12769   8651   -133   -663
-1160       C
ATOM  10087  OD1 ASP B 492       9.615 -19.122  66.681  1.00 84.65
O
ANISOU10087  OD1 ASP B 492    11876  12267   8021   -326   -705
-1184       O
ATOM  10088  OD2 ASP B 492      10.202 -18.566  64.643  1.00114.94
O
ANISOU10088  OD2 ASP B 492    15834  15934  11902    -75   -663
-1097       O
```

```
ATOM  10089  N   SER B 493      11.844 -16.890  63.918  1.00126.43
N
ANISOU10089  N   SER B 493    17484  17069  13484     63   -564
-960       N
ATOM  10090  CA  SER B 493      13.272 -17.088  63.675  1.00 95.17
C
ANISOU10090  CA  SER B 493    13581  12986   9593    -25   -530
-884       C
ATOM  10091  C   SER B 493      13.953 -15.837  63.129  1.00 76.43
C
ANISOU10091  C   SER B 493    11320  10471   7248     31   -474
-783       C
ATOM  10092  O   SER B 493      15.101 -15.546  63.486  1.00 76.74
O
ANISOU10092  O   SER B 493    11400  10404   7353    -80   -435
-739       O
ATOM  10093  CB  SER B 493      13.478 -18.269  62.724  1.00 67.65
C
ANISOU10093  CB  SER B 493    10054   9585   6065    -29   -560
-881       C
ATOM  10094  OG  SER B 493      12.811 -18.057  61.493  1.00 74.44
O
ANISOU10094  OG  SER B 493    10912  10536   6833    113   -559
-858       O
ATOM  10095  N   ALA B 494      13.279 -15.096  62.250  1.00 63.41
N
ANISOU10095  N   ALA B 494     9726   8825   5543    186   -480
-733       N
ATOM  10096  CA  ALA B 494      13.866 -13.864  61.728  1.00 64.67
C
ANISOU10096  CA  ALA B 494    10028   8820   5723    212   -451
-603       C
ATOM  10097  C   ALA B 494      14.155 -12.850  62.833  1.00 65.20
C
ANISOU10097  C   ALA B 494    10191   8690   5892    161   -451
-629       C
ATOM  10098  O   ALA B 494      15.224 -12.231  62.854  1.00 65.75
O
ANISOU10098  O   ALA B 494    10355   8613   6013     39   -416
-538       O
ATOM  10099  CB  ALA B 494      12.941 -13.259  60.673  1.00 75.05
C
ANISOU10099  CB  ALA B 494    11400  10162   6954    407   -491
-537       C
ATOM  10100  N   GLU B 495      13.206 -12.650  63.750  1.00101.29
N
ANISOU10100  N   GLU B 495    14727  13278  10479    246   -491
-765       N
ATOM  10101  CA  GLU B 495      13.478 -11.857  64.950  1.00121.91
C
ANISOU10101  CA  GLU B 495    17401  15740  13180    194   -495
-843       C
ATOM  10102  C   GLU B 495      14.516 -12.511  65.857  1.00 81.47
C
```

```
ANISOU10102  C   GLU B 495    12209  10639   8106    -33   -451
-867       C
ATOM  10103  O   GLU B 495      15.391 -11.831  66.407  1.00 64.97
O
ANISOU10103  O   GLU B 495    10201   8394   6090   -146   -431
-853       O
ATOM  10104  CB  GLU B 495      12.182 -11.616  65.727  1.00136.75
C
ANISOU10104  CB  GLU B 495    19209  17718  15030    350   -545
-1020       C
ATOM  10105  CG  GLU B 495      11.256 -10.587  65.099  1.00148.76
C
ANISOU10105  CG  GLU B 495    20831  19159  16533    618   -614
-1027       C
ATOM  10106  CD  GLU B 495      10.140 -10.164  66.034  1.00159.33
C
ANISOU10106  CD  GLU B 495    22087  20603  17847    790   -667
-1246       C
ATOM  10107  OE1 GLU B 495      10.122 -10.636  67.190  1.00159.07
O
ANISOU10107  OE1 GLU B 495    21922  20717  17801    667   -638
-1378       O
ATOM  10108  OE2 GLU B 495       9.283  -9.358  65.617  1.00160.87
O
ANISOU10108  OE2 GLU B 495    22343  20757  18024   1057   -744
-1290       O
ATOM  10109  N   THR B 496      14.415 -13.828  66.037  1.00100.48
N
ANISOU10109  N   THR B 496    14475  13232  10469   -103   -453
-902       N
ATOM  10110  CA  THR B 496      15.199 -14.540  67.045  1.00108.10
C
ANISOU10110  CA  THR B 496    15370  14232  11470   -286   -445
-935       C
ATOM  10111  C   THR B 496      16.710 -14.413  66.850  1.00 92.52
C
ANISOU10111  C   THR B 496    13441  12160   9554   -413   -402
-845       C
ATOM  10112  O   THR B 496      17.451 -14.252  67.829  1.00 61.92
O
ANISOU10112  O   THR B 496     9556   8244   5727   -544   -392
-875       O
ATOM  10113  CB  THR B 496      14.791 -16.014  67.047  1.00 61.05
C
ANISOU10113  CB  THR B 496     9295   8445   5456   -329   -488
-959       C
ATOM  10114  OG1 THR B 496      13.408 -16.128  67.402  1.00 61.36
O
ANISOU10114  OG1 THR B 496     9263   8630   5421   -267   -524
-1049       O
ATOM  10115  CG2 THR B 496      15.606 -16.786  68.040  1.00 60.32
C
ANISOU10115  CG2 THR B 496     9154   8367   5399   -502   -510
-967       C
```

```
ATOM  10116  N   ILE B 497      17.191 -14.468  65.606  1.00 62.25
N
ANISOU10116  N   ILE B 497     9633   8327   5691   -383   -375
-741       N
ATOM  10117  CA  ILE B 497      18.623 -14.287  65.352  1.00 62.59
C
ANISOU10117  CA  ILE B 497     9685   8342   5755   -512   -325
-662       C
ATOM  10118  C   ILE B 497      19.133 -12.935  65.847  1.00 70.45
C
ANISOU10118  C   ILE B 497    10797   9162   6809   -612   -300
-628       C
ATOM  10119  O   ILE B 497      20.244 -12.842  66.383  1.00 63.93
O
ANISOU10119  O   ILE B 497     9943   8332   6016   -774   -272
-623       O
ATOM  10120  CB  ILE B 497      18.927 -14.491  63.856  1.00 84.38
C
ANISOU10120  CB  ILE B 497    12434  11191   8435   -460   -296
-565       C
ATOM  10121  CG1 ILE B 497      17.960 -13.681  62.996  1.00110.32
C
ANISOU10121  CG1 ILE B 497    15829  14413  11675   -327   -306
-493       C
ATOM  10122  CG2 ILE B 497      18.861 -15.966  63.500  1.00 62.10
C
ANISOU10122  CG2 ILE B 497     9489   8532   5574   -400   -331
-632       C
ATOM  10123  CD1 ILE B 497      18.382 -13.570  61.553  1.00138.96
C
ANISOU10123  CD1 ILE B 497    19466  18130  15203   -316   -270
-364       C
ATOM  10124  N   ARG B 498      18.345 -11.869  65.687  1.00 79.37
N
ANISOU10124  N   ARG B 498    12065  10139   7954   -514   -324
-614       N
ATOM  10125  CA  ARG B 498      18.723 -10.590  66.290  1.00 86.47
C
ANISOU10125  CA  ARG B 498    13108  10819   8928   -603   -331
-614       C
ATOM  10126  C   ARG B 498      18.669 -10.635  67.813  1.00 79.45
C
ANISOU10126  C   ARG B 498    12163   9932   8093   -657   -348
-778       C
ATOM  10127  O   ARG B 498      19.553 -10.090  68.484  1.00 66.59
O
ANISOU10127  O   ARG B 498    10572   8212   6515   -824   -334
-793       O
ATOM  10128  CB  ARG B 498      17.859  -9.450  65.745  1.00 82.03
C
ANISOU10128  CB  ARG B 498    12732  10056   8379   -444   -388
-572       C
ATOM  10129  CG  ARG B 498      18.055  -9.194  64.256  1.00104.61
C
```

```
ANISOU10129  CG  ARG B 498    15677  12899  11170   -434   -379
-370       C
ATOM  10130  CD  ARG B 498      16.898  -9.681  63.411  1.00124.13
C
ANISOU10130  CD  ARG B 498    18102  15500  13562   -204   -409
-367       C
ATOM  10131  NE  ARG B 498      15.674  -8.940  63.691  1.00144.15
N
ANISOU10131  NE  ARG B 498    20736  17904  16131     19   -496
-453       N
ATOM  10132  CZ  ARG B 498      14.606  -8.912  62.902  1.00123.72
C
ANISOU10132  CZ  ARG B 498    18159  15374  13476    243   -548
-433       C
ATOM  10133  NH1 ARG B 498      14.465  -9.806  61.934  1.00119.29
N
ANISOU10133  NH1 ARG B 498    17482  15034  12808    268   -515
-366       N
ATOM  10134  NH2 ARG B 498      13.568  -8.163  63.245  1.00112.50
N
ANISOU10134  NH2 ARG B 498    16816  13842  12086    467   -638
-536       N
ATOM  10135  N   ARG B 499      17.642 -11.270  68.377  1.00 99.23
N
ANISOU10135  N   ARG B 499    14566  12569  10567   -542   -378
-901       N
ATOM  10136  CA  ARG B 499      17.562 -11.426  69.827  1.00117.02
C
ANISOU10136  CA  ARG B 499    16737  14892  12832   -613   -392
-1048       C
ATOM  10137  C   ARG B 499      18.754 -12.200  70.380  1.00108.86
C
ANISOU10137  C   ARG B 499    15604  13953  11806   -812   -367
-1018       C
ATOM  10138  O   ARG B 499      19.346 -11.810  71.394  1.00125.19
O
ANISOU10138  O   ARG B 499    17667  15995  13906   -940   -364
-1085       O
ATOM  10139  CB  ARG B 499      16.264 -12.143  70.195  1.00124.47
C
ANISOU10139  CB  ARG B 499    17560  16032  13701   -496   -425
-1152       C
ATOM  10140  CG  ARG B 499      15.994 -12.238  71.687  1.00 76.70
C
ANISOU10140  CG  ARG B 499    11412  10108   7622   -569   -441
-1304       C
ATOM  10141  CD  ARG B 499      15.009 -13.361  71.978  1.00103.19
C
ANISOU10141  CD  ARG B 499    14612  13725  10871   -561   -469
-1344       C
ATOM  10142  NE  ARG B 499      13.726 -13.167  71.314  1.00156.44
N
ANISOU10142  NE  ARG B 499    21342  20539  17558   -361   -488
-1390       N
```

```
ATOM  10143  CZ  ARG B 499      12.878 -14.151  71.032  1.00132.89
C
ANISOU10143  CZ  ARG B 499    18247  17762  14481   -351   -511
-1379       C
ATOM  10144  NH1 ARG B 499      11.801 -13.914  70.299  1.00112.34
N
ANISOU10144  NH1 ARG B 499    15627  15231  11824   -163   -529
-1416       N
ATOM  10145  NH2 ARG B 499      13.167 -15.394  71.392  1.00 95.29
N
ANISOU10145  NH2 ARG B 499    13408  13115   9685   -533   -532
-1317       N
ATOM  10146  N   ALA B 500      19.116 -13.305  69.727  1.00 68.99
N
ANISOU10146  N   ALA B 500    10471   9020   6722   -823   -361
-935       N
ATOM  10147  CA  ALA B 500      20.310 -14.054  70.112  1.00 61.99
C
ANISOU10147  CA  ALA B 500     9491   8220   5843   -964   -359
-909       C
ATOM  10148  C   ALA B 500      21.577 -13.213  69.994  1.00 63.03
C
ANISOU10148  C   ALA B 500     9665   8275   6009  -1102   -309
-856       C
ATOM  10149  O   ALA B 500      22.423 -13.217  70.897  1.00 72.49
O
ANISOU10149  O   ALA B 500    10805   9513   7224  -1243   -310
-893       O
ATOM  10150  CB  ALA B 500      20.420 -15.318  69.259  1.00 61.14
C
ANISOU10150  CB  ALA B 500     9308   8224   5699   -898   -382
-855       C
ATOM  10151  N   LEU B 501      21.731 -12.487  68.884  1.00 64.00
N
ANISOU10151  N   LEU B 501     9884   8307   6126  -1086   -270
-758       N
ATOM  10152  CA  LEU B 501      22.863 -11.573  68.746  1.00 78.37
C
ANISOU10152  CA  LEU B 501    11759  10056   7962  -1268   -227
-689       C
ATOM  10153  C   LEU B 501      22.849 -10.490  69.820  1.00 84.53
C
ANISOU10153  C   LEU B 501    12646  10661   8810  -1367   -247
-772       C
ATOM  10154  O   LEU B 501      23.897 -10.158  70.387  1.00 85.20
O
ANISOU10154  O   LEU B 501    12704  10760   8910  -1565   -229
-783       O
ATOM  10155  CB  LEU B 501      22.865 -10.944  67.352  1.00112.53
C
ANISOU10155  CB  LEU B 501    16195  14313  12249  -1253   -198
-541       C
ATOM  10156  CG  LEU B 501      24.028  -9.995  67.054  1.00 94.29
C
```

```
ANISOU10156  CG  LEU B 501    13950  11949   9927  -1493   -156
-429       C
ATOM  10157  CD1 LEU B 501      25.354 -10.732  67.173  1.00121.29
C
ANISOU10157  CD1 LEU B 501    17161  15631  13291  -1635   -109
-438       C
ATOM  10158  CD2 LEU B 501      23.878  -9.372  65.677  1.00 92.64
C
ANISOU10158  CD2 LEU B 501    13868  11675   9656  -1490   -143
-253       C
ATOM  10159  N   ASN B 502      21.675  -9.919  70.102  1.00 91.88
N
ANISOU10159  N   ASN B 502    13689  11446   9775  -1224   -291
-852       N
ATOM  10160  CA  ASN B 502      21.546  -8.955  71.193  1.00105.53
C
ANISOU10160  CA  ASN B 502    15509  13021  11565  -1274   -326
-987       C
ATOM  10161  C   ASN B 502      21.951  -9.557  72.535  1.00128.75
C
ANISOU10161  C   ASN B 502    18292  16136  14489  -1380   -325
-1109       C
ATOM  10162  O   ASN B 502      22.568  -8.880  73.366  1.00 75.59
O
ANISOU10162  O   ASN B 502    11593   9338   7791  -1533   -331
-1187       O
ATOM  10163  CB  ASN B 502      20.117  -8.415  71.249  1.00 95.43
C
ANISOU10163  CB  ASN B 502    14332  11624  10304  -1039   -383
-1092       C
ATOM  10164  CG  ASN B 502      19.790  -7.517  70.071  1.00135.32
C
ANISOU10164  CG  ASN B 502    19589  16442  15385   -940   -414
-970       C
ATOM  10165  OD1 ASN B 502      20.685  -7.050  69.365  1.00148.01
O
ANISOU10165  OD1 ASN B 502    21298  17937  17003  -1101   -395
-809       O
ATOM  10166  ND2 ASN B 502      18.504  -7.270  69.853  1.00163.48
N
ANISOU10166  ND2 ASN B 502    23210  19960  18947   -683   -470
-1040       N
ATOM  10167  N   LEU B 503      21.622 -10.824  72.766  1.00131.20
N
ANISOU10167  N   LEU B 503    18444  16665  14743  -1317   -331
-1120       N
ATOM  10168  CA  LEU B 503      22.010 -11.506  73.993  1.00 78.85
C
ANISOU10168  CA  LEU B 503    11671  10210   8079  -1424   -350
-1195       C
ATOM  10169  C   LEU B 503      23.358 -12.205  73.864  1.00 64.43
C
ANISOU10169  C   LEU B 503     9737   8501   6241  -1555   -336
-1103       C
```

```
ATOM  10170  O   LEU B 503      23.714 -13.010  74.731  1.00 86.85
O
ANISOU10170  O   LEU B 503    12455  11499   9047  -1615   -373
-1130       O
ATOM  10171  CB  LEU B 503      20.932 -12.509  74.412  1.00 90.76
C
ANISOU10171  CB  LEU B 503    13082  11882   9522  -1318   -390
-1239       C
ATOM  10172  CG  LEU B 503      19.568 -11.902  74.747  1.00 89.80
C
ANISOU10172  CG  LEU B 503    13001  11744   9376  -1183   -406
-1373       C
ATOM  10173  CD1 LEU B 503      18.520 -12.989  74.955  1.00 84.67
C
ANISOU10173  CD1 LEU B 503    12234  11305   8634  -1120   -440
-1383       C
ATOM  10174  CD2 LEU B 503      19.669 -10.999  75.970  1.00 95.97
C
ANISOU10174  CD2 LEU B 503    13793  12508  10161  -1256   -415
-1541       C
ATOM  10175  N   GLY B 504      24.107 -11.909  72.805  1.00 64.99
N
ANISOU10175  N   GLY B 504     9843   8524   6324  -1597   -292
-996       N
ATOM  10176  CA  GLY B 504      25.494 -12.318  72.700  1.00 65.23
C
ANISOU10176  CA  GLY B 504     9752   8703   6331  -1727   -272
-943       C
ATOM  10177  C   GLY B 504      25.728 -13.782  72.410  1.00 85.18
C
ANISOU10177  C   GLY B 504    12135  11412   8819  -1621   -309
-912       C
ATOM  10178  O   GLY B 504      26.789 -14.312  72.754  1.00106.41
O
ANISOU10178  O   GLY B 504    14690  14258  11485  -1688   -328
-918       O
ATOM  10179  N   VAL B 505      24.769 -14.453  71.781  1.00 87.50
N
ANISOU10179  N   VAL B 505    12455  11686   9104  -1449   -334
-891       N
ATOM  10180  CA  VAL B 505      24.945 -15.825  71.322  1.00 94.39
C
ANISOU10180  CA  VAL B 505    13231  12681   9951  -1334   -388
-871       C
ATOM  10181  C   VAL B 505      24.795 -15.809  69.805  1.00114.19
C
ANISOU10181  C   VAL B 505    15768  15188  12433  -1229   -341
-811       C
ATOM  10182  O   VAL B 505      23.715 -15.514  69.278  1.00118.77
O
ANISOU10182  O   VAL B 505    16446  15670  13011  -1141   -329
-792       O
ATOM  10183  CB  VAL B 505      23.963 -16.800  71.991  1.00102.21
C
```

```
ANISOU10183  CB  VAL B 505    14224  13671  10941  -1261   -481
-902       C
ATOM  10184  CG1 VAL B 505      22.554 -16.225  72.033  1.00123.17
C
ANISOU10184  CG1 VAL B 505    16975  16233  13590  -1216   -460
-928       C
ATOM  10185  CG2 VAL B 505      23.981 -18.147  71.273  1.00112.01
C
ANISOU10185  CG2 VAL B 505    15423  14963  12173  -1128   -556
-882       C
ATOM  10186  N   ASN B 506      25.885 -16.108  69.104  1.00127.60
N
ANISOU10186  N   ASN B 506    17360  17032  14091  -1233   -317
-791       N
ATOM  10187  CA  ASN B 506      25.931 -15.988  67.651  1.00108.69
C
ANISOU10187  CA  ASN B 506    14965  14698  11635  -1168   -260
-734       C
ATOM  10188  C   ASN B 506      25.274 -17.206  67.013  1.00 81.66
C
ANISOU10188  C   ASN B 506    11519  11309   8198   -963   -326
-775       C
ATOM  10189  O   ASN B 506      25.669 -18.344  67.285  1.00107.17
O
ANISOU10189  O   ASN B 506    14661  14618  11442   -880   -412
-845       O
ATOM  10190  CB  ASN B 506      27.375 -15.840  67.175  1.00124.50
C
ANISOU10190  CB  ASN B 506    16825  16915  13566  -1267   -203
-719       C
ATOM  10191  CG  ASN B 506      27.476 -15.615  65.678  1.00117.65
C
ANISOU10191  CG  ASN B 506    15940  16164  12598  -1238   -133
-648       C
ATOM  10192  OD1 ASN B 506      28.188 -16.335  64.976  1.00107.35
O
ANISOU10192  OD1 ASN B 506    14474  15102  11211  -1157   -129
-697       O
ATOM  10193  ND2 ASN B 506      26.763 -14.611  65.181  1.00135.26
N
ANISOU10193  ND2 ASN B 506    18335  18236  14823  -1292    -87
-540       N
ATOM  10194  N   VAL B 507      24.278 -16.974  66.166  1.00 62.68
N
ANISOU10194  N   VAL B 507     9207   8839   5770   -879   -304
-734       N
ATOM  10195  CA  VAL B 507      23.612 -18.056  65.451  1.00 73.14
C
ANISOU10195  CA  VAL B 507    10517  10201   7071   -706   -365
-780       C
ATOM  10196  C   VAL B 507      24.346 -18.312  64.143  1.00 63.19
C
ANISOU10196  C   VAL B 507     9158   9132   5719   -636   -324
-785       C
```

ATOM 10197 O   VAL B 507   24.576 -17.388 63.353 1.00 64.16
O
ANISOU10197 O   VAL B 507   9295   9315   5768   -703   -230
-695   O
ATOM 10198 CB VAL B 507   22.133 -17.721 65.194 1.00 69.46
C
ANISOU10198 CB VAL B 507   10167   9620   6603   -643   -368
-752   C
ATOM 10199 CG1 VAL B 507   21.442 -18.886 64.501 1.00 89.05
C
ANISOU10199 CG1 VAL B 507   12628 12151   9055   -496   -440
-810   C
ATOM 10200 CG2 VAL B 507   21.432 -17.383 66.493 1.00 60.91
C
ANISOU10200 CG2 VAL B 507   9147   8415   5582   -710   -398
-773   C
ATOM 10201 N   LYS B 508   24.711 -19.570 63.913 1.00 63.30
N
ANISOU10201 N   LYS B 508   9078   9247   5728   -503   -406
-894   N
ATOM 10202 CA LYS B 508   25.345 -19.996 62.675 1.00 76.19
C
ANISOU10202 CA LYS B 508   10588 11104   7257   -393   -382
-953   C
ATOM 10203 C   LYS B 508   24.477 -21.081 62.061 1.00 77.37
C
ANISOU10203 C   LYS B 508   10773 11215   7408   -211   -478
-1046   C
ATOM 10204 O   LYS B 508   24.129 -22.057 62.734 1.00111.48
O
ANISOU10204 O   LYS B 508   15140 15399 11817   -150   -610
-1117   O
ATOM 10205 CB LYS B 508   26.762 -20.517 62.932 1.00103.03
C
ANISOU10205 CB LYS B 508   13816 14690 10642   -366   -411
-1051   C
ATOM 10206 CG LYS B 508   27.703 -19.473 63.515 1.00 96.75
C
ANISOU10206 CG LYS B 508   12961 13972   9828   -576   -318
-971   C
ATOM 10207 CD LYS B 508   29.135 -19.981 63.589 1.00 73.01
C
ANISOU10207 CD LYS B 508   9737 11235   6770   -532   -339
-1083   C
ATOM 10208 CE LYS B 508   30.023 -19.016 64.361 1.00 68.40
C
ANISOU10208 CE LYS B 508   9092 10723   6175   -771   -265
-1012   C
ATOM 10209 NZ LYS B 508   30.009 -17.651 63.766 1.00101.33
N
ANISOU10209 NZ LYS B 508   13317 14923 10262   -999   -122
-859   N
ATOM 10210 N   MET B 509   24.129 -20.912 60.790 1.00 69.48
N

```
ANISOU10210  N   MET B 509     9760  10338   6301   -145   -422
-1036       N
ATOM  10211  CA  MET B 509      23.399 -21.946  60.072  1.00 76.65
C
ANISOU10211  CA  MET B 509    10685  11249   7192     23   -512
-1148       C
ATOM  10212  C   MET B 509      24.243 -23.195  59.843  1.00 65.92
C
ANISOU10212  C   MET B 509     9213   9997   5835    190   -617
-1341       C
ATOM  10213  O   MET B 509      25.439 -23.119  59.549  1.00 67.23
O
ANISOU10213  O   MET B 509     9224  10390   5930    219   -570
-1399       O
ATOM  10214  CB  MET B 509      22.899 -21.407  58.733  1.00 65.44
C
ANISOU10214  CB  MET B 509     9258   9974   5630     53   -424
-1093       C
ATOM  10215  CG  MET B 509      22.211 -22.451  57.867  1.00 65.76
C
ANISOU10215  CG  MET B 509     9295  10062   5629    221   -511
-1231       C
ATOM  10216  SD  MET B 509      21.748 -21.817  56.250  1.00 72.03
S
ANISOU10216  SD  MET B 509    10053  11092   6222    259   -410
-1164       S
ATOM  10217  CE  MET B 509      20.565 -20.554  56.718  1.00 92.49
C
ANISOU10217  CE  MET B 509    12814  13473   8857    138   -361
-944       C
ATOM  10218  N   ILE B 510      23.604 -24.352  59.992  1.00 65.70
N
ANISOU10218  N   ILE B 510     9268   9811   5886    297   -774
-1449       N
ATOM  10219  CA  ILE B 510      24.105 -25.621  59.481  1.00 67.19
C
ANISOU10219  CA  ILE B 510     9397  10056   6075    509   -910
-1664       C
ATOM  10220  C   ILE B 510      23.064 -26.126  58.493  1.00 69.49
C
ANISOU10220  C   ILE B 510     9750  10339   6317    593   -954
-1739       C
ATOM  10221  O   ILE B 510      21.879 -26.211  58.835  1.00130.73
O
ANISOU10221  O   ILE B 510    17643  17903  14124    502  -1000
-1665       O
ATOM  10222  CB  ILE B 510      24.344 -26.640  60.611  1.00 67.29
C
ANISOU10222  CB  ILE B 510     9491   9834   6241    550  -1105
-1724       C
ATOM  10223  CG1 ILE B 510      25.521 -26.202  61.484  1.00 67.45
C
ANISOU10223  CG1 ILE B 510     9413   9928   6287    497  -1068
-1680       C
```

```
ATOM  10224  CG2 ILE B 510      24.579 -28.032  60.041  1.00 80.39
C
ANISOU10224  CG2 ILE B 510    11156  11461   7928    793  -1294
-1957       C
ATOM  10225  CD1 ILE B 510      26.850 -26.218  60.769  1.00101.71
C
ANISOU10225  CD1 ILE B 510    13533  14589  10524    647  -1020
-1823       C
ATOM  10226  N   THR B 511      23.492 -26.461  57.275  1.00105.98
N
ANISOU10226  N   THR B 511    14248  15202  10816    758   -940
-1898       N
ATOM  10227  CA  THR B 511      22.579 -27.126  56.357  1.00 84.42
C
ANISOU10227  CA  THR B 511    11570  12467   8040    855  -1013
-2014       C
ATOM  10228  C   THR B 511      23.330 -27.979  55.344  1.00 74.23
C
ANISOU10228  C   THR B 511    10146  11394   6663   1099  -1080
-2287       C
ATOM  10229  O   THR B 511      24.480 -27.698  54.993  1.00146.36
O
ANISOU10229  O   THR B 511    19094  20817  15698   1175   -997
-2353       O
ATOM  10230  CB  THR B 511      21.698 -26.108  55.617  1.00 98.49
C
ANISOU10230  CB  THR B 511    13354  14377   9691    750   -860
-1858       C
ATOM  10231  OG1 THR B 511      20.713 -26.798  54.837  1.00130.47
O
ANISOU10231  OG1 THR B 511    17455  18418  13698    829   -945
-1971       O
ATOM  10232  CG2 THR B 511      22.544 -25.218  54.713  1.00 69.95
C
ANISOU10232  CG2 THR B 511     9570  11116   5893    752   -689
-1811       C
ATOM  10233  N   GLY B 512      22.658 -29.046  54.898  1.00 72.75
N
ANISOU10233  N   GLY B 512    10051  11081   6508   1217  -1240
-2464       N
ATOM  10234  CA  GLY B 512      23.162 -29.908  53.842  1.00 77.81
C
ANISOU10234  CA  GLY B 512    10584  11916   7064   1473  -1324
-2768       C
ATOM  10235  C   GLY B 512      22.871 -29.418  52.439  1.00 76.23
C
ANISOU10235  C   GLY B 512    10250  12079   6634   1497  -1181
-2800       C
ATOM  10236  O   GLY B 512      23.526 -29.859  51.490  1.00 81.26
O
ANISOU10236  O   GLY B 512    10725  13011   7140   1702  -1193
-3047       O
ATOM  10237  N   ASP B 513      21.891 -28.526  52.292  1.00122.44
N
```

```
ANISOU10237  N   ASP B 513    16161  17935  12426   1307  -1058
-2564       N
ATOM  10238  CA  ASP B 513      21.567 -27.866  51.031  1.00134.13
C
ANISOU10238  CA  ASP B 513    17530  19757  13675   1298   -916
-2521       C
ATOM  10239  C   ASP B 513      22.749 -27.157  50.379  1.00 96.78
C
ANISOU10239  C   ASP B 513    12574  15450   8748   1317   -752
-2505       C
ATOM  10240  O   ASP B 513      23.742 -26.831  51.039  1.00109.33
O
ANISOU10240  O   ASP B 513    14095  17063  10383   1276   -705
-2453       O
ATOM  10241  CB  ASP B 513      20.422 -26.871  51.238  1.00136.09
C
ANISOU10241  CB  ASP B 513    17893  19895  13918   1098   -824
-2231       C
ATOM  10242  CG  ASP B 513      19.099 -27.558  51.520  1.00129.74
C
ANISOU10242  CG  ASP B 513    17251  18821  13221   1069   -965
-2270       C
ATOM  10243  OD1 ASP B 513      18.149 -26.869  51.945  1.00143.81
O
ANISOU10243  OD1 ASP B 513    19124  20489  15030    925   -919
-2064       O
ATOM  10244  OD2 ASP B 513      19.006 -28.786  51.310  1.00138.31
O
ANISOU10244  OD2 ASP B 513    18373  19820  14358   1188  -1131
-2517       O
ATOM  10245  N   GLN B 514      22.638 -26.930  49.071  1.00 96.88
N
ANISOU10245  N   GLN B 514    12458  15830   8520   1360   -669
-2548       N
ATOM  10246  CA  GLN B 514      23.591 -26.101  48.345  1.00 93.39
C
ANISOU10246  CA  GLN B 514    11801  15844   7837   1305   -495
-2470       C
ATOM  10247  C   GLN B 514      23.708 -24.710  48.965  1.00 92.06
C
ANISOU10247  C   GLN B 514    11698  15593   7687   1040   -352
-2100       C
ATOM  10248  O   GLN B 514      22.767 -24.183  49.564  1.00 82.28
O
ANISOU10248  O   GLN B 514    10658  14029   6578    917   -358
-1887       O
ATOM  10249  CB  GLN B 514      23.191 -25.978  46.870  1.00144.74
C
ANISOU10249  CB  GLN B 514    18195  22735  14064   1346   -432
-2510       C
ATOM  10250  CG  GLN B 514      23.221 -27.280  46.067  1.00125.72
C
ANISOU10250  CG  GLN B 514    15684  20497  11586   1615   -560
-2918       C
```

```
ATOM  10251  CD  GLN B 514      22.002 -28.157  46.292  1.00135.96
C
ANISOU10251  CD  GLN B 514    17191  21404  13063   1685   -738
-3035       C
ATOM  10252  OE1 GLN B 514      22.076 -29.177  46.977  1.00140.05
O
ANISOU10252  OE1 GLN B 514    17810  21607  13796   1806   -914
-3239       O
ATOM  10253  NE2 GLN B 514      20.876 -27.768  45.703  1.00121.14
N
ANISOU10253  NE2 GLN B 514    15383  19560  11087   1600   -707
-2900       N
ATOM  10254  N   LEU B 515      24.899 -24.126  48.806  1.00 95.14
N
ANISOU10254  N   LEU B 515    11907  16304   7936    954   -230
-2044       N
ATOM  10255  CA  LEU B 515      25.230 -22.841  49.419  1.00 80.40
C
ANISOU10255  CA  LEU B 515    10098  14362   6088    687   -110
-1722       C
ATOM  10256  C   LEU B 515      24.273 -21.729  49.002  1.00108.70
C
ANISOU10256  C   LEU B 515    13838  17867   9596    517    -37
-1409       C
ATOM  10257  O   LEU B 515      23.863 -20.913  49.835  1.00122.32
O
ANISOU10257  O   LEU B 515    15747  19261  11467    365    -24
-1179       O
ATOM  10258  CB  LEU B 515      26.666 -22.455  49.061  1.00 81.72
C
ANISOU10258  CB  LEU B 515    10010  14986   6054    597      9
-1732       C
ATOM  10259  CG  LEU B 515      27.208 -21.153  49.658  1.00 81.01
C
ANISOU10259  CG  LEU B 515     9963  14853   5966    286    126
-1421       C
ATOM  10260  CD1 LEU B 515      27.403 -21.275  51.160  1.00 78.98
C
ANISOU10260  CD1 LEU B 515     9814  14205   5989    269     57
-1429       C
ATOM  10261  CD2 LEU B 515      28.501 -20.726  48.968  1.00 84.05
C
ANISOU10261  CD2 LEU B 515    10067  15803   6067    148    258
-1409       C
ATOM  10262  N   ALA B 516      23.915 -21.671  47.717  1.00108.74
N
ANISOU10262  N   ALA B 516    13771  18181   9366    555     -1
-1405       N
ATOM  10263  CA  ALA B 516      22.989 -20.643  47.245  1.00127.39
C
ANISOU10263  CA  ALA B 516    16284  20477  11642    428     42
-1104       C
ATOM  10264  C   ALA B 516      21.632 -20.722  47.938  1.00149.11
C
```

```
ANISOU10264  C   ALA B 516    19264  22773  14617    489    -60
-1064       C
ATOM  10265  O   ALA B 516      21.026 -19.686  48.237  1.00187.06
O
ANISOU10265  O   ALA B 516    24243  27360  19473    371    -40
-796       O
ATOM  10266  CB  ALA B 516      22.822 -20.746  45.729  1.00151.36
C
ANISOU10266  CB  ALA B 516    19182  23958  14370    486     78
-1139       C
ATOM  10267  N   ILE B 517      21.130 -21.932  48.191  1.00136.77
N
ANISOU10267  N   ILE B 517    17709  21075  13184    669   -180
-1330       N
ATOM  10268  CA  ILE B 517      19.904 -22.077  48.978  1.00112.15
C
ANISOU10268  CA  ILE B 517    14779  17566  10269    689   -275
-1302       C
ATOM  10269  C   ILE B 517      20.081 -21.486  50.372  1.00 92.16
C
ANISOU10269  C   ILE B 517    12371  14697   7949    559   -264
-1157       C
ATOM  10270  O   ILE B 517      19.217 -20.755  50.873  1.00 78.34
O
ANISOU10270  O   ILE B 517    10770  12714   6280    494   -268
-982       O
ATOM  10271  CB  ILE B 517      19.484 -23.556  49.055  1.00115.17
C
ANISOU10271  CB  ILE B 517    15151  17861  10747    853   -420
-1610       C
ATOM  10272  CG1 ILE B 517      19.244 -24.126  47.660  1.00129.42
C
ANISOU10272  CG1 ILE B 517    16837  20000  12337    986   -439
-1783       C
ATOM  10273  CG2 ILE B 517      18.239 -23.714  49.908  1.00132.72
C
ANISOU10273  CG2 ILE B 517    17544  19731  13150    823   -513
-1569       C
ATOM  10274  CD1 ILE B 517      19.100 -25.628  47.633  1.00126.78
C
ANISOU10274  CD1 ILE B 517    16490  19592  12087   1149   -596
-2127       C
ATOM  10275  N   GLY B 518      21.208 -21.795  51.016  1.00106.10
N
ANISOU10275  N   GLY B 518    14063  16457   9795    535   -256
-1246       N
ATOM  10276  CA  GLY B 518      21.491 -21.245  52.335  1.00 93.56
C
ANISOU10276  CA  GLY B 518    12570  14590   8387    403   -244
-1124       C
ATOM  10277  C   GLY B 518      21.558 -19.730  52.373  1.00 78.56
C
ANISOU10277  C   GLY B 518    10756  12656   6439    217   -138
-832       C
```

```
ATOM  10278  O   GLY B 518      20.915 -19.087  53.207  1.00104.36
O
ANISOU10278  O   GLY B 518    14181  15629   9841    149   -154
-704       O
ATOM  10279  N   LYS B 519      22.343 -19.139  51.472  1.00 83.33
N
ANISOU10279  N   LYS B 519    11256  13565   6840    126    -38
-730       N
ATOM  10280  CA  LYS B 519      22.432 -17.683  51.380  1.00 76.00
C
ANISOU10280  CA  LYS B 519    10439  12587   5849    -79     40
-428       C
ATOM  10281  C   LYS B 519      21.093 -17.027  51.045  1.00 73.97
C
ANISOU10281  C   LYS B 519    10366  12152   5586    -36      0
-262       C
ATOM  10282  O   LYS B 519      20.735 -16.006  51.643  1.00 89.19
O
ANISOU10282  O   LYS B 519    12476  13797   7615   -129     -8
-77       O
ATOM  10283  CB  LYS B 519      23.501 -17.302  50.355  1.00 96.81
C
ANISOU10283  CB  LYS B 519    12914  15645   8226   -212    144
-343       C
ATOM  10284  CG  LYS B 519      24.906 -17.732  50.772  1.00 77.51
C
ANISOU10284  CG  LYS B 519    10268  13407   5776   -271    190
-490       C
ATOM  10285  CD  LYS B 519      25.986 -17.163  49.864  1.00 80.51
C
ANISOU10285  CD  LYS B 519    10473  14242   5876   -467    307
-374       C
ATOM  10286  CE  LYS B 519      25.740 -17.520  48.408  1.00126.28
C
ANISOU10286  CE  LYS B 519    16139  20435  11406   -364    330
-423       C
ATOM  10287  NZ  LYS B 519      26.838 -17.031  47.528  1.00123.41
N
ANISOU10287  NZ  LYS B 519    15567  20591  10731   -577    450
-319       N
ATOM  10288  N   GLU B 520      20.346 -17.580  50.089  1.00100.95
N
ANISOU10288  N   GLU B 520    13734  15741   8880    118    -36
-341       N
ATOM  10289  CA  GLU B 520      19.049 -17.001  49.740  1.00112.88
C
ANISOU10289  CA  GLU B 520    15394  17126  10370    187    -86
-199       C
ATOM  10290  C   GLU B 520      18.055 -17.060  50.898  1.00131.01
C
ANISOU10290  C   GLU B 520    17820  19060  12897    260   -166
-253       C
ATOM  10291  O   GLU B 520      17.376 -16.071  51.195  1.00141.13
O
```

```
ANISOU10291  O   GLU B 520    19263  20128  14233    253   -191
-85       O
ATOM  10292  CB  GLU B 520      18.476 -17.713  48.513  1.00118.81
C
ANISOU10292  CB  GLU B 520    16035  18175  10933    336   -113
-310       C
ATOM  10293  CG  GLU B 520      17.190 -17.101  47.971  1.00 85.00
C
ANISOU10293  CG  GLU B 520    11872  13845   6580    419   -167
-156       C
ATOM  10294  CD  GLU B 520      17.299 -15.614  47.714  1.00129.82
C
ANISOU10294  CD  GLU B 520    17705  19440  12182    293   -141
184       C
ATOM  10295  OE1 GLU B 520      18.211 -15.200  46.969  1.00165.59
O
ANISOU10295  OE1 GLU B 520    22181  24213  16525    146    -68
327       O
ATOM  10296  OE2 GLU B 520      16.465 -14.858  48.256  1.00119.36
O
ANISOU10296  OE2 GLU B 520    16558  17813  10979    339   -205
305       O
ATOM  10297  N   THR B 521      17.952 -18.212  51.565  1.00147.67
N
ANISOU10297  N   THR B 521    19864  21107  15135    332   -219
-490       N
ATOM  10298  CA  THR B 521      17.173 -18.289  52.800  1.00110.99
C
ANISOU10298  CA  THR B 521    15316  16165  10689    348   -284
-535       C
ATOM  10299  C   THR B 521      17.748 -17.407  53.905  1.00 83.76
C
ANISOU10299  C   THR B 521    11963  12486   7378    211   -246
-420       C
ATOM  10300  O   THR B 521      17.004 -16.695  54.592  1.00 69.80
O
ANISOU10300  O   THR B 521    10317  10501   5702    214   -274
-347       O
ATOM  10301  CB  THR B 521      17.090 -19.744  53.271  1.00 94.70
C
ANISOU10301  CB  THR B 521    13177  14084   8719    406   -361
-781       C
ATOM  10302  OG1 THR B 521      16.153 -20.459  52.457  1.00 99.75
O
ANISOU10302  OG1 THR B 521    13777  14859   9267    522   -425
-891       O
ATOM  10303  CG2 THR B 521      16.644 -19.825  54.721  1.00 97.96
C
ANISOU10303  CG2 THR B 521    13670  14228   9322    353   -413
-806       C
ATOM  10304  N   GLY B 522      19.069 -17.430  54.080  1.00115.20
N
ANISOU10304  N   GLY B 522    15875  16535  11360     97   -187
-424       N
```

```
ATOM  10305  CA  GLY B 522      19.699 -16.605  55.102  1.00102.02
C
ANISOU10305  CA  GLY B 522    14285  14671   9808    -57   -152
-327       C
ATOM  10306  C   GLY B 522      19.446 -15.115  54.966  1.00100.87
C
ANISOU10306  C   GLY B 522    14308  14376   9641   -142   -130
-92       C
ATOM  10307  O   GLY B 522      19.250 -14.420  55.967  1.00148.82
O
ANISOU10307  O   GLY B 522    20507  20187  15853   -195   -150
-51       O
ATOM  10308  N   ARG B 523      19.453 -14.599  53.736  1.00 71.49
N
ANISOU10308  N   ARG B 523    10603  10816   5743   -155   -102
63       N
ATOM  10309  CA  ARG B 523      19.185 -13.176  53.536  1.00 76.50
C
ANISOU10309  CA  ARG B 523    11441  11268   6359   -230   -116
312       C
ATOM  10310  C   ARG B 523      17.782 -12.773  53.981  1.00 80.77
C
ANISOU10310  C   ARG B 523    12129  11554   7007    -54   -210
304       C
ATOM  10311  O   ARG B 523      17.615 -11.749  54.656  1.00 99.31
O
ANISOU10311  O   ARG B 523    14655  13612   9467    -92   -247
395       O
ATOM  10312  CB  ARG B 523      19.404 -12.797  52.073  1.00118.09
C
ANISOU10312  CB  ARG B 523    16696  16785  11386   -281    -85
499       C
ATOM  10313  CG  ARG B 523      19.648 -11.306  51.865  1.00125.24
C
ANISOU10313  CG  ARG B 523    17820  17508  12257   -459    -99
802       C
ATOM  10314  CD  ARG B 523      19.359 -10.867  50.439  1.00147.56
C
ANISOU10314  CD  ARG B 523    20692  20525  14850   -456   -116
1022       C
ATOM  10315  NE  ARG B 523      20.015 -11.719  49.453  1.00172.52
N
ANISOU10315  NE  ARG B 523    23604  24160  17786   -496    -29
953       N
ATOM  10316  CZ  ARG B 523      19.401 -12.679  48.771  1.00197.71
C
ANISOU10316  CZ  ARG B 523    26645  27599  20876   -290    -39
791       C
ATOM  10317  NH1 ARG B 523      18.095 -12.861  48.911  1.00201.19
N
ANISOU10317  NH1 ARG B 523    27159  27880  21405    -58   -129
715       N
ATOM  10318  NH2 ARG B 523      20.080 -13.412  47.901  1.00205.99
N
```

```
ANISOU10318  NH2 ARG B 523    27466  29084  21718   -320     35
699       N
ATOM  10319  N   ARG B 524      16.757 -13.555  53.629  1.00 86.03
N
ANISOU10319  N   ARG B 524    12716  12338   7635    140   -255
178       N
ATOM  10320  CA  ARG B 524      15.415 -13.170  54.057  1.00100.01
C
ANISOU10320  CA  ARG B 524    14585  13933   9482    311   -342
153       C
ATOM  10321  C   ARG B 524      15.185 -13.473  55.530  1.00111.22
C
ANISOU10321  C   ARG B 524    15990  15182  11085    309   -360
-17       C
ATOM  10322  O   ARG B 524      14.374 -12.801  56.179  1.00129.43
O
ANISOU10322  O   ARG B 524    18396  17307  13474    403   -418
-27       O
ATOM  10323  CB  ARG B 524      14.343 -13.864  53.210  1.00 97.49
C
ANISOU10323  CB  ARG B 524    14170  13832   9042    494   -388
76       C
ATOM  10324  CG  ARG B 524      14.454 -15.378  53.142  1.00 75.79
C
ANISOU10324  CG  ARG B 524    11233  11291   6273    497   -372
-140       C
ATOM  10325  CD  ARG B 524      13.241 -15.994  52.441  1.00 75.18
C
ANISOU10325  CD  ARG B 524    11078  11398   6090    659   -435
-232       C
ATOM  10326  NE  ARG B 524      12.916 -15.372  51.157  1.00 94.35
N
ANISOU10326  NE  ARG B 524    13538  13976   8333    742   -447
-71       N
ATOM  10327  CZ  ARG B 524      13.617 -15.524  50.035  1.00 95.00
C
ANISOU10327  CZ  ARG B 524    13560  14290   8246    692   -399
-4       C
ATOM  10328  NH1 ARG B 524      14.781 -16.158  50.047  1.00 96.29
N
ANISOU10328  NH1 ARG B 524    13626  14552   8407    572   -329
-89       N
ATOM  10329  NH2 ARG B 524      13.213 -14.925  48.924  1.00 92.50
N
ANISOU10329  NH2 ARG B 524    13281  14117   7748    762   -423
163       N
ATOM  10330  N   LEU B 525      15.883 -14.474  56.067  1.00 86.51
N
ANISOU10330  N   LEU B 525    12735  12126   8008    214   -322
-155       N
ATOM  10331  CA  LEU B 525      15.947 -14.649  57.512  1.00 84.81
C
ANISOU10331  CA  LEU B 525    12519  11754   7952    155   -333
-270       C
```

ATOM 10332 C   LEU B 525   16.593 -13.442 58.183 1.00 69.57
C
ANISOU10332 C   LEU B 525   10725   9599   6109   34   -310
-164   C
ATOM 10333 O   LEU B 525   16.121 -12.969 59.224 1.00 82.63
O
ANISOU10333 O   LEU B 525   12447 11078   7871   55   -345
-222   O
ATOM 10334 CB LEU B 525   16.715 -15.932 57.837 1.00 65.30
C
ANISOU10334 CB LEU B 525   9908   9395   5508   81   -318
-406   C
ATOM 10335 CG LEU B 525   16.850 -16.377 59.291 1.00 63.91
C
ANISOU10335 CG LEU B 525   9709   9103   5469   6   -343
-518   C
ATOM 10336 CD1 LEU B 525   15.487 -16.687 59.887 1.00115.46
C
ANISOU10336 CD1 LEU B 525   16235 15609 12026   86   -410
-611   C
ATOM 10337 CD2 LEU B 525   17.759 -17.589 59.373 1.00 72.86
C
ANISOU10337 CD2 LEU B 525   10729 10337   6616   -42   -352
-620   C
ATOM 10338 N   GLY B 526   17.666 -12.924 57.591 1.00 84.43
N
ANISOU10338 N   GLY B 526   12642 11506   7932   -108   -254
-18   N
ATOM 10339 CA GLY B 526   18.357 -11.753 58.088 1.00 91.04
C
ANISOU10339 CA GLY B 526   13624 12130   8837   -269   -241
101   C
ATOM 10340 C   GLY B 526   19.693 -12.074 58.718 1.00 77.81
C
ANISOU10340 C   GLY B 526   11850 10512   7202   -471   -176
55   C
ATOM 10341 O   GLY B 526   20.257 -11.220 59.414 1.00 73.55
O
ANISOU10341 O   GLY B 526   11413   9793   6742   -627   -169
106   O
ATOM 10342 N   MET B 527   20.212 -13.278 58.480 1.00 68.34
N
ANISOU10342 N   MET B 527   10457   9562   5948   -461   -141
-53   N
ATOM 10343 CA MET B 527   21.385 -13.819 59.152 1.00 67.78
C
ANISOU10343 CA MET B 527   10256   9579   5917   -589   -103
-142   C
ATOM 10344 C   MET B 527   22.697 -13.308 58.566 1.00113.07
C
ANISOU10344 C   MET B 527   15941 15475 11544   -791   -24
-19   C
ATOM 10345 O   MET B 527   23.718 -13.312 59.263 1.00127.89
O

```
ANISOU10345  O   MET B 527    17743  17389  13461   -939      7
-58       O
ATOM  10346  CB  MET B 527      21.313 -15.348  59.079  1.00 66.42
C
ANISOU10346  CB  MET B 527     9918   9585   5735   -451   -133
-321       C
ATOM  10347  CG  MET B 527      22.522 -16.112  59.575  1.00124.99
C
ANISOU10347  CG  MET B 527    17183  17133  13175   -515   -122
-430       C
ATOM  10348  SD  MET B 527      22.227 -17.889  59.461  1.00 95.90
S
ANISOU10348  SD  MET B 527    13377  13558   9504   -317   -212
-639       S
ATOM  10349  CE  MET B 527      21.159 -18.146  60.875  1.00 99.70
C
ANISOU10349  CE  MET B 527    13962  13778  10141   -305   -300
-692       C
ATOM  10350  N   GLY B 528      22.687 -12.845  57.326  1.00 71.37
N
ANISOU10350  N   GLY B 528    10690  10317   6111   -819      5
135       N
ATOM  10351  CA  GLY B 528      23.890 -12.439  56.615  1.00 84.01
C
ANISOU10351  CA  GLY B 528    12210  12155   7555  -1037     85
264       C
ATOM  10352  C   GLY B 528      24.513 -13.585  55.842  1.00104.24
C
ANISOU10352  C   GLY B 528    14510  15129   9968   -959    132
131       C
ATOM  10353  O   GLY B 528      24.346 -14.761  56.165  1.00 82.40
O
ANISOU10353  O   GLY B 528    11626  12417   7267   -772     94
-85       O
ATOM  10354  N   THR B 529      25.253 -13.228  54.792  1.00108.52
N
ANISOU10354  N   THR B 529    14964  15973  10297  -1111    205
261       N
ATOM  10355  CA  THR B 529      25.641 -14.176  53.755  1.00 96.60
C
ANISOU10355  CA  THR B 529    13213  14897   8595  -1002    246
139       C
ATOM  10356  C   THR B 529      27.127 -14.523  53.765  1.00 87.99
C
ANISOU10356  C   THR B 529    11860  14179   7393  -1129    321
41       C
ATOM  10357  O   THR B 529      27.601 -15.185  52.837  1.00 79.51
O
ANISOU10357  O   THR B 529    10560  13525   6124  -1052    363
-69       O
ATOM  10358  CB  THR B 529      25.249 -13.634  52.378  1.00143.99
C
ANISOU10358  CB  THR B 529    19264  21068  14379  -1042    271
337       C
```

```
ATOM  10359  OG1 THR B 529      25.738 -12.294  52.237  1.00164.30
O
ANISOU10359  OG1 THR B 529    21970  23583  16873  -1358    309
630       O
ATOM  10360  CG2 THR B 529      23.735 -13.633  52.220  1.00145.36
C
ANISOU10360  CG2 THR B 529    19622  20975  14634   -827    184
359       C
ATOM  10361  N   ASN B 530      27.876 -14.103  54.784  1.00 99.93
N
ANISOU10361  N   ASN B 530    13376  15583   9008  -1309    336
56       N
ATOM  10362  CA  ASN B 530      29.277 -14.506  54.913  1.00 96.15
C
ANISOU10362  CA  ASN B 530    12618  15483   8431  -1401    395
-69       C
ATOM  10363  C   ASN B 530      29.350 -15.935  55.458  1.00116.69
C
ANISOU10363  C   ASN B 530    15068  18121  11149  -1095    322
-382       C
ATOM  10364  O   ASN B 530      29.686 -16.185  56.617  1.00129.07
O
ANISOU10364  O   ASN B 530    16627  19535  12878  -1085    277
-479       O
ATOM  10365  CB  ASN B 530      30.032 -13.524  55.800  1.00132.91
C
ANISOU10365  CB  ASN B 530    17333  20019  13147  -1719    427
61       C
ATOM  10366  CG  ASN B 530      31.519 -13.828  55.879  1.00117.81
C
ANISOU10366  CG  ASN B 530    15104  18561  11098  -1844    494
-53       C
ATOM  10367  OD1 ASN B 530      32.031 -14.679  55.152  1.00 91.62
O
ANISOU10367  OD1 ASN B 530    11516  15681   7615  -1695    523
-220       O
ATOM  10368  ND2 ASN B 530      32.218 -13.132  56.769  1.00107.76
N
ANISOU10368  ND2 ASN B 530    13852  17206   9886  -2108    511
14       N
ATOM  10369  N   MET B 531      29.022 -16.890  54.587  1.00 77.74
N
ANISOU10369  N   MET B 531    10026  13386   6126   -845    295
-540       N
ATOM  10370  CA  MET B 531      29.023 -18.295  54.970  1.00 76.55
C
ANISOU10370  CA  MET B 531     9772  13230   6086   -544    193
-834       C
ATOM  10371  C   MET B 531      29.677 -19.121  53.870  1.00 78.69
C
ANISOU10371  C   MET B 531     9766  13989   6142   -377    212
-1041       C
ATOM  10372  O   MET B 531      29.755 -18.700  52.713  1.00 80.58
O
```

```
ANISOU10372  O   MET B 531     9924  14546   6149   -463    301
-952       O
ATOM  10373  CB  MET B 531      27.603 -18.791  55.287  1.00111.98
C
ANISOU10373  CB  MET B 531    14479  17314  10755   -359     84
-868       C
ATOM  10374  CG  MET B 531      26.523 -18.329  54.329  1.00131.35
C
ANISOU10374  CG  MET B 531    17066  19723  13119   -354    107
-727       C
ATOM  10375  SD  MET B 531      24.895 -18.910  54.855  1.00110.89
S
ANISOU10375  SD  MET B 531    14691  16711  10732   -167    -21
-784       S
ATOM  10376  CE  MET B 531      24.940 -20.616  54.307  1.00129.32
C
ANISOU10376  CE  MET B 531    16872  19224  13039    122   -123
-1106       C
ATOM  10377  N   TYR B 532      30.146 -20.308  54.248  1.00 78.63
N
ANISOU10377  N   TYR B 532     9619  14046   6211   -127    114
-1324       N
ATOM  10378  CA  TYR B 532      31.100 -21.084  53.469  1.00 81.16
C
ANISOU10378  CA  TYR B 532     9629  14866   6343     51    119
-1581       C
ATOM  10379  C   TYR B 532      30.571 -22.491  53.234  1.00 90.14
C
ANISOU10379  C   TYR B 532    10783  15895   7570    424    -38
-1872       C
ATOM  10380  O   TYR B 532      29.876 -23.044  54.093  1.00102.53
O
ANISOU10380  O   TYR B 532    12559  17015   9384    530   -172
-1903       O
ATOM  10381  CB  TYR B 532      32.456 -21.164  54.191  1.00 82.42
C
ANISOU10381  CB  TYR B 532     9568  15252   6496     17    125
-1685       C
ATOM  10382  CG  TYR B 532      33.121 -19.818  54.395  1.00 83.39
C
ANISOU10382  CG  TYR B 532     9649  15527   6510   -384    273
-1424       C
ATOM  10383  CD1 TYR B 532      34.034 -19.322  53.479  1.00 86.51
C
ANISOU10383  CD1 TYR B 532     9777  16494   6599   -563    408
-1391       C
ATOM  10384  CD2 TYR B 532      32.810 -19.032  55.495  1.00 81.48
C
ANISOU10384  CD2 TYR B 532     9638  14864   6458   -603    270
-1213       C
ATOM  10385  CE1 TYR B 532      34.671 -18.114  53.691  1.00119.45
C
ANISOU10385  CE1 TYR B 532    13925  20790  10670   -973    526
-1144       C
```

```
ATOM  10386  CE2 TYR B 532      33.405 -17.801  55.692  1.00 82.66
C
ANISOU10386  CE2 TYR B 532     9778  15107   6520   -983    383
-987       C
ATOM  10387  CZ  TYR B 532      34.344 -17.352  54.795  1.00 85.82
C
ANISOU10387  CZ  TYR B 532     9928  16052   6628  -1180    506
-943       C
ATOM  10388  OH  TYR B 532      34.897 -16.105  54.968  1.00 96.90
O
ANISOU10388  OH  TYR B 532    11353  17521   7942  -1606    605
-693       O
ATOM  10389  N   PRO B 533      30.877 -23.097  52.085  1.00 83.03
N
ANISOU10389  N   PRO B 533     9674  15406   6468    613    -33
-2092       N
ATOM  10390  CA  PRO B 533      30.786 -24.563  51.987  1.00 83.52
C
ANISOU10390  CA  PRO B 533     9706  15406   6621    997   -213
-2447       C
ATOM  10391  C   PRO B 533      31.754 -25.251  52.939  1.00 92.97
C
ANISOU10391  C   PRO B 533    10797  16578   7950   1165   -332
-2642       C
ATOM  10392  O   PRO B 533      32.968 -25.043  52.881  1.00160.47
O
ANISOU10392  O   PRO B 533    19065  25558  16347   1145   -260
-2720       O
ATOM  10393  CB  PRO B 533      31.120 -24.834  50.518  1.00 86.45
C
ANISOU10393  CB  PRO B 533     9823  16327   6696   1129   -151
-2643       C
ATOM  10394  CG  PRO B 533      32.052 -23.732  50.141  1.00108.88
C
ANISOU10394  CG  PRO B 533    12436  19656   9276    842     49
-2465       C
ATOM  10395  CD  PRO B 533      31.666 -22.521  50.981  1.00104.81
C
ANISOU10395  CD  PRO B 533    12159  18773   8891    482    127
-2066       C
ATOM  10396  N   SER B 534      31.195 -26.074  53.831  1.00 82.73
N
ANISOU10396  N   SER B 534     9724  14785   6924   1318   -523
-2709       N
ATOM  10397  CA  SER B 534      31.993 -26.757  54.847  1.00109.60
C
ANISOU10397  CA  SER B 534    13082  18084  10477   1483   -673
-2858       C
ATOM  10398  C   SER B 534      33.042 -27.687  54.243  1.00113.62
C
ANISOU10398  C   SER B 534    13303  19001  10864   1837   -764
-3242       C
ATOM  10399  O   SER B 534      34.178 -27.747  54.728  1.00112.43
O
```

```
ANISOU10399  O   SER B 534    12945  19087  10687   1909   -782
-3342       O
ATOM  10400  CB  SER B 534      31.073 -27.543  55.782  1.00132.99
C
ANISOU10400  CB  SER B 534    16364  20441  13726   1566   -881
-2841       C
ATOM  10401  OG  SER B 534      30.225 -28.411  55.049  1.00149.59
O
ANISOU10401  OG  SER B 534    18587  22396  15854   1757  -1000
-3005       O
ATOM  10402  N   SER B 535      32.680 -28.419  53.187  1.00116.53
N
ANISOU10402  N   SER B 535    13646  19480  11152   2076   -829
-3482       N
ATOM  10403  CA  SER B 535      33.601 -29.376  52.572  1.00150.38
C
ANISOU10403  CA  SER B 535    17666  24146  15327   2467   -940
-3903       C
ATOM  10404  C   SER B 535      34.894 -28.730  52.082  1.00130.26
C
ANISOU10404  C   SER B 535    14696  22317  12480   2405   -753
-3965       C
ATOM  10405  O   SER B 535      35.983 -29.277  52.290  1.00118.77
O
ANISOU10405  O   SER B 535    12998  21138  10991   2667   -846
-4236       O
ATOM  10406  CB  SER B 535      32.900 -30.097  51.420  1.00134.39
C
ANISOU10406  CB  SER B 535    15684  22149  13231   2681  -1011
-4138       C
ATOM  10407  OG  SER B 535      32.333 -29.172  50.508  1.00120.66
O
ANISOU10407  OG  SER B 535    13912  20653  11281   2410   -790
-3930       O
ATOM  10408  N   ALA B 536      34.799 -27.574  51.424  1.00124.15
N
ANISOU10408  N   ALA B 536    13827  21869  11477   2058   -503
-3715       N
ATOM  10409  CA  ALA B 536      35.994 -26.787  51.126  1.00135.18
C
ANISOU10409  CA  ALA B 536    14853  23922  12589   1876   -313
-3682       C
ATOM  10410  C   ALA B 536      36.652 -26.241  52.388  1.00161.17
C
ANISOU10410  C   ALA B 536    18140  27096  16001   1676   -295
-3501       C
ATOM  10411  O   ALA B 536      37.876 -26.318  52.544  1.00177.02
O
ANISOU10411  O   ALA B 536    19820  29559  17880   1755   -282
-3670       O
ATOM  10412  CB  ALA B 536      35.642 -25.645  50.172  1.00 96.27
C
ANISOU10412  CB  ALA B 536     9890  19288   7399   1501    -76
-3398       C
```

```
ATOM  10413  N   LEU B 537      35.852 -25.684  53.300  1.00118.60
N
ANISOU10413  N   LEU B 537    13091  21131  10841   1424   -295
-3175       N
ATOM  10414  CA  LEU B 537      36.402 -24.991  54.464  1.00 90.73
C
ANISOU10414  CA  LEU B 537     9569  17504   7402   1174   -254
-2974       C
ATOM  10415  C   LEU B 537      37.202 -25.913  55.381  1.00 94.93
C
ANISOU10415  C   LEU B 537     9997  17997   8074   1479   -446
-3221       C
ATOM  10416  O   LEU B 537      38.234 -25.504  55.923  1.00115.43
O
ANISOU10416  O   LEU B 537    12374  20887  10598   1364   -393
-3207       O
ATOM  10417  CB  LEU B 537      35.279 -24.307  55.242  1.00 86.98
C
ANISOU10417  CB  LEU B 537     9487  16413   7149    897   -238
-2627       C
ATOM  10418  CG  LEU B 537      35.740 -23.285  56.279  1.00 94.68
C
ANISOU10418  CG  LEU B 537    10483  17317   8173    552   -150
-2379       C
ATOM  10419  CD1 LEU B 537      36.418 -22.113  55.593  1.00 88.06
C
ANISOU10419  CD1 LEU B 537     9434  16972   7053    199     71
-2216       C
ATOM  10420  CD2 LEU B 537      34.556 -22.807  57.093  1.00145.70
C
ANISOU10420  CD2 LEU B 537    17333  23158  14870    372   -172
-2114       C
ATOM  10421  N   LEU B 538      36.751 -27.155  55.578  1.00 95.72
N
ANISOU10421  N   LEU B 538    10261  17734   8373   1859   -684
-3440       N
ATOM  10422  CA  LEU B 538      37.431 -28.026  56.537  1.00135.31
C
ANISOU10422  CA  LEU B 538    15239  22627  13547   2150   -904
-3631       C
ATOM  10423  C   LEU B 538      38.829 -28.463  56.097  1.00156.74
C
ANISOU10423  C   LEU B 538    17513  25988  16054   2443   -930
-3981       C
ATOM  10424  O   LEU B 538      39.609 -28.914  56.943  1.00168.63
O
ANISOU10424  O   LEU B 538    18923  27504  17647   2632  -1078
-4101       O
ATOM  10425  CB  LEU B 538      36.544 -29.235  56.836  1.00 91.54
C
ANISOU10425  CB  LEU B 538    10026  16488   8269   2449  -1176
-3747       C
ATOM  10426  CG  LEU B 538      36.083 -30.099  55.662  1.00 93.12
C
```

```
ANISOU10426  CG  LEU B 538    10240  16724   8417   2752  -1267
-4028       C
ATOM  10427  CD1 LEU B 538      37.058 -31.190  55.290  1.00 97.16
C
ANISOU10427  CD1 LEU B 538    10506  17539   8873   3248  -1456
-4479       C
ATOM  10428  CD2 LEU B 538      34.741 -30.685  56.005  1.00 93.48
C
ANISOU10428  CD2 LEU B 538    10714  16089   8716   2755  -1432
-3927       C
ATOM  10429  N   GLY B 539      39.171 -28.349  54.816  1.00156.99
N
ANISOU10429  N   GLY B 539    17257  26590  15800   2492   -797
-4153       N
ATOM  10430  CA  GLY B 539      40.571 -28.320  54.429  1.00164.72
C
ANISOU10430  CA  GLY B 539    17745  28337  16504   2612   -730
-4404       C
ATOM  10431  C   GLY B 539      41.320 -29.633  54.327  1.00182.66
C
ANISOU10431  C   GLY B 539    19808  30809  18787   3207   -975
-4892       C
ATOM  10432  O   GLY B 539      42.524 -29.661  54.601  1.00197.87
O
ANISOU10432  O   GLY B 539    21375  33225  20583   3327   -984
-5065       O
ATOM  10433  N   THR B 540      40.658 -30.727  53.948  1.00171.32
N
ANISOU10433  N   THR B 540    18581  29013  17500   3592  -1189
-5132       N
ATOM  10434  CA  THR B 540      41.364 -32.002  53.819  1.00177.17
C
ANISOU10434  CA  THR B 540    19152  29900  18266   4199  -1458
-5626       C
ATOM  10435  C   THR B 540      40.814 -32.848  52.671  1.00156.89
C
ANISOU10435  C   THR B 540    16635  27319  15658   4532  -1561
-5955       C
ATOM  10436  O   THR B 540      41.526 -33.100  51.693  1.00172.43
O
ANISOU10436  O   THR B 540    18210  29957  17350   4795  -1520
-6327       O
ATOM  10437  CB  THR B 540      41.363 -32.789  55.140  1.00195.54
C
ANISOU10437  CB  THR B 540    21760  31598  20939   4437  -1769
-5622       C
ATOM  10438  OG1 THR B 540      41.797 -34.134  54.901  1.00217.43
O
ANISOU10438  OG1 THR B 540    24475  34361  23779   5066  -2083
-6103       O
ATOM  10439  CG2 THR B 540      40.005 -32.798  55.813  1.00169.48
C
ANISOU10439  CG2 THR B 540    19003  27447  17943   4193  -1844
-5272       C
```

```
ATOM  10440  N   HIS B 541      39.563 -33.305  52.783  1.00128.20
N
ANISOU10440  N   HIS B 541    13464  22964  12283   4522  -1698
-5840       N
ATOM  10441  CA  HIS B 541      38.985 -34.165  51.752  1.00146.70
C
ANISOU10441  CA  HIS B 541    15890  25237  14611   4829  -1823
-6161       C
ATOM  10442  C   HIS B 541      38.965 -33.470  50.397  1.00155.15
C
ANISOU10442  C   HIS B 541    16665  26971  15312   4644  -1533
-6190       C
ATOM  10443  O   HIS B 541      39.313 -34.076  49.377  1.00116.26
O
ANISOU10443  O   HIS B 541    11493  22486  10195   4995  -1581
-6620       O
ATOM  10444  CB  HIS B 541      37.570 -34.581  52.160  1.00180.25
C
ANISOU10444  CB  HIS B 541    20685  28625  19178   4722  -1980
-5952       C
ATOM  10445  CG  HIS B 541      36.886 -35.474  51.171  1.00207.07
C
ANISOU10445  CG  HIS B 541    24210  31884  22583   4996  -2127
-6265       C
ATOM  10446  ND1 HIS B 541      36.131 -34.986  50.127  1.00195.64
N
ANISOU10446  ND1 HIS B 541    22748  30636  20951   4771  -1924
-6186       N
ATOM  10447  CD2 HIS B 541      36.831 -36.824  51.074  1.00207.14
C
ANISOU10447  CD2 HIS B 541    24380  31558  22765   5471  -2473
-6658       C
ATOM  10448  CE1 HIS B 541      35.643 -35.996  49.428  1.00160.68
C
ANISOU10448  CE1 HIS B 541    18446  26031  16572   5087  -2125
-6530       C
ATOM  10449  NE2 HIS B 541      36.054 -37.122  49.981  1.00195.79
N
ANISOU10449  NE2 HIS B 541    23012  30136  21243   5511  -2463
-6825       N
ATOM  10450  N   LYS B 542      38.567 -32.206  50.368  1.00171.84
N
ANISOU10450  N   LYS B 542    18807  29172  17315   4102  -1244
-5743       N
ATOM  10451  CA  LYS B 542      38.603 -31.398  49.156  1.00168.74
C
ANISOU10451  CA  LYS B 542    18142  29422  16549   3854   -961
-5686       C
ATOM  10452  C   LYS B 542      39.516 -30.193  49.299  1.00135.41
C
ANISOU10452  C   LYS B 542    13599  25773  12077   3467   -696
-5439       C
ATOM  10453  O   LYS B 542      40.234 -29.854  48.352  1.00124.58
O
```

```
ANISOU10453  O   LYS B 542    11811  25196  10327   3427   -524
-5579       O
ATOM  10454  CB  LYS B 542      37.190 -30.938  48.763  1.00167.31
C
ANISOU10454  CB  LYS B 542    18307  28838  16425   3542   -869
-5366       C
ATOM  10455  CG  LYS B 542      37.160 -30.018  47.549  1.00166.47
C
ANISOU10455  CG  LYS B 542    17965  29353  15934   3246   -587
-5235       C
ATOM  10456  CD  LYS B 542      35.759 -29.888  46.976  1.00156.71
C
ANISOU10456  CD  LYS B 542    17042  27758  14743   3096   -562
-5050       C
ATOM  10457  CE  LYS B 542      34.786 -29.320  47.993  1.00167.38
C
ANISOU10457  CE  LYS B 542    18822  28378  16397   2777   -565
-4601       C
ATOM  10458  NZ  LYS B 542      33.418 -29.186  47.418  1.00183.57
N
ANISOU10458  NZ  LYS B 542    21144  30133  18473   2649   -545
-4437       N
ATOM  10459  N   ASP B 543      39.491 -29.532  50.460  1.00108.21
N
ANISOU10459  N   ASP B 543    10338  21955   8821   3156   -661
-5074       N
ATOM  10460  CA  ASP B 543      40.332 -28.378  50.757  1.00118.99
C
ANISOU10460  CA  ASP B 543    11452  23767   9992   2750   -436
-4819       C
ATOM  10461  C   ASP B 543      40.019 -27.207  49.832  1.00139.40
C
ANISOU10461  C   ASP B 543    13976  26700  12291   2278   -153
-4511       C
ATOM  10462  O   ASP B 543      39.087 -27.269  49.024  1.00142.74
O
ANISOU10462  O   ASP B 543    14566  26988  12680   2273   -131
-4474       O
ATOM  10463  CB  ASP B 543      41.815 -28.771  50.666  1.00174.29
C
ANISOU10463  CB  ASP B 543    17949  31497  16777   3029   -463
-5197       C
ATOM  10464  CG  ASP B 543      42.735 -27.783  51.364  1.00167.11
C
ANISOU10464  CG  ASP B 543    16822  30917  15757   2647   -305
-4958       C
ATOM  10465  OD1 ASP B 543      43.970 -27.961  51.281  1.00143.97
O
ANISOU10465  OD1 ASP B 543    13434  28663  12606   2811   -297
-5235       O
ATOM  10466  OD2 ASP B 543      42.224 -26.832  51.992  1.00152.57
O
ANISOU10466  OD2 ASP B 543    15258  28671  14041   2188   -197
-4513       O
```

```
ATOM  10467  N   ALA B 544      40.788 -26.130  49.960  1.00175.59
N
ANISOU10467  N   ALA B 544    18334  31718  16664   1866     49
-4276       N
ATOM  10468  CA  ALA B 544      40.495 -24.874  49.288  1.00186.24
C
ANISOU10468  CA  ALA B 544    19699  33286  17777   1341    295
-3890       C
ATOM  10469  C   ALA B 544      41.799 -24.104  49.140  1.00185.48
C
ANISOU10469  C   ALA B 544    19167  33970  17339   1028    478
-3841       C
ATOM  10470  O   ALA B 544      42.811 -24.430  49.765  1.00186.38
O
ANISOU10470  O   ALA B 544    19015  34349  17452   1180    419
-4053       O
ATOM  10471  CB  ALA B 544      39.452 -24.058  50.058  1.00104.55
C
ANISOU10471  CB  ALA B 544     9839  22171   7712    989    315
-3427       C
ATOM  10472  N   ASN B 545      41.760 -23.071  48.300  1.00179.09
N
ANISOU10472  N   ASN B 545    18285  33536  16226    572    692
-3548       N
ATOM  10473  CA  ASN B 545      42.921 -22.209  48.126  1.00156.28
C
ANISOU10473  CA  ASN B 545    15013  31379  12988    160    877
-3429       C
ATOM  10474  C   ASN B 545      43.101 -21.248  49.292  1.00156.92
C
ANISOU10474  C   ASN B 545    15290  31081  13251   -268    915
-3067       C
ATOM  10475  O   ASN B 545      44.216 -20.760  49.510  1.00147.28
O
ANISOU10475  O   ASN B 545    13741  30400  11821   -535   1012
-3049       O
ATOM  10476  CB  ASN B 545      42.805 -21.424  46.817  1.00144.02
C
ANISOU10476  CB  ASN B 545    13345  30345  11033   -222   1075
-3203       C
ATOM  10477  CG  ASN B 545      41.652 -20.438  46.829  1.00116.23
C
ANISOU10477  CG  ASN B 545    10326  26175   7661   -617   1124
-2691       C
ATOM  10478  OD1 ASN B 545      41.798 -19.296  47.266  1.00118.63
O
ANISOU10478  OD1 ASN B 545    10766  26344   7964  -1125   1217
-2293       O
ATOM  10479  ND2 ASN B 545      40.496 -20.875  46.342  1.00114.12
N
ANISOU10479  ND2 ASN B 545    10335  25504   7521   -375   1047
-2712       N
ATOM  10480  N   LEU B 546      42.033 -20.969  50.040  1.00110.64
N
```

```
ANISOU10480  N   LEU B 546     9940  24336   7760   -341    838
-2797       N
ATOM  10481  CA  LEU B 546      42.075 -20.029  51.159  1.00108.64
C
ANISOU10481  CA  LEU B 546     9917  23664   7698   -736    863
-2465       C
ATOM  10482  C   LEU B 546      42.567 -20.752  52.411  1.00107.62
C
ANISOU10482  C   LEU B 546     9740  23326   7826   -432    705
-2703       C
ATOM  10483  O   LEU B 546      41.796 -21.210  53.257  1.00111.75
O
ANISOU10483  O   LEU B 546    10606  23143   8712   -208    550
-2703       O
ATOM  10484  CB  LEU B 546      40.708 -19.393  51.380  1.00104.77
C
ANISOU10484  CB  LEU B 546     9965  22379   7462   -926    848
-2099       C
ATOM  10485  CG  LEU B 546      40.643 -18.275  52.424  1.00150.42
C
ANISOU10485  CG  LEU B 546    16016  27715  13421  -1361    880
-1745       C
ATOM  10486  CD1 LEU B 546      41.497 -17.086  52.006  1.00106.41
C
ANISOU10486  CD1 LEU B 546    10251  22657   7525  -1929   1056
-1489       C
ATOM  10487  CD2 LEU B 546      39.200 -17.848  52.661  1.00143.20
C
ANISOU10487  CD2 LEU B 546    15620  26009  12780  -1415    830
-1473       C
ATOM  10488  N   ALA B 547      43.891 -20.857  52.522  1.00119.44
N
ANISOU10488  N   ALA B 547    10784  25490   9107   -432    740
-2906       N
ATOM  10489  CA  ALA B 547      44.524 -21.364  53.730  1.00133.73
C
ANISOU10489  CA  ALA B 547    12509  27193  11111   -211    600
-3090       C
ATOM  10490  C   ALA B 547      45.445 -20.333  54.362  1.00123.50
C
ANISOU10490  C   ALA B 547    11044  26189   9694   -709    719
-2887       C
ATOM  10491  O   ALA B 547      46.048 -20.613  55.405  1.00120.59
O
ANISOU10491  O   ALA B 547    10579  25784   9454   -589    617
-3009       O
ATOM  10492  CB  ALA B 547      45.309 -22.648  53.432  1.00123.18
C
ANISOU10492  CB  ALA B 547    10767  26370   9666    368    473
-3605       C
ATOM  10493  N   SER B 548      45.572 -19.152  53.752  1.00119.20
N
ANISOU10493  N   SER B 548    10467  25929   8895  -1277    918
-2574       N
```

```
ATOM  10494  CA  SER B 548      46.272 -18.038  54.384  1.00149.93
C
ANISOU10494  CA  SER B 548    14300  29960  12708  -1836   1020
-2321       C
ATOM  10495  C   SER B 548      45.575 -17.608  55.670  1.00141.80
C
ANISOU10495  C   SER B 548    13742  28058  12076  -1957    930
-2091       C
ATOM  10496  O   SER B 548      46.231 -17.307  56.674  1.00111.04
O
ANISOU10496  O   SER B 548     9769  24178   8242  -2125    908
-2080       O
ATOM  10497  CB  SER B 548      46.379 -16.867  53.407  1.00122.36
C
ANISOU10497  CB  SER B 548    10769  26836   8884  -2436   1222
-1990       C
ATOM  10498  OG  SER B 548      45.110 -16.536  52.871  1.00114.91
O
ANISOU10498  OG  SER B 548    10264  25335   8060  -2491   1228
-1732       O
ATOM  10499  N   ILE B 549      44.248 -17.577  55.655  1.00178.61
N
ANISOU10499  N   ILE B 549    18874  31995  16993  -1871    876
-1923       N
ATOM  10500  CA  ILE B 549      43.450 -17.465  56.873  1.00167.44
C
ANISOU10500  CA  ILE B 549    17887  29762  15969  -1840    760
-1795       C
ATOM  10501  C   ILE B 549      43.135 -18.876  57.360  1.00159.38
C
ANISOU10501  C   ILE B 549    16895  28478  15184  -1223    564
-2109       C
ATOM  10502  O   ILE B 549      42.470 -19.633  56.634  1.00125.95
O
ANISOU10502  O   ILE B 549    12731  24149  10975   -881    508
-2239       O
ATOM  10503  CB  ILE B 549      42.162 -16.672  56.626  1.00130.74
C
ANISOU10503  CB  ILE B 549    13716  24500  11460  -2067    796
-1457       C
ATOM  10504  CG1 ILE B 549      42.472 -15.353  55.917  1.00139.81
C
ANISOU10504  CG1 ILE B 549    14850  25929  12344  -2651    965
-1141       C
ATOM  10505  CG2 ILE B 549      41.462 -16.384  57.943  1.00123.98
C
ANISOU10505  CG2 ILE B 549    13248  22899  10959  -2102    698
-1328       C
ATOM  10506  CD1 ILE B 549      41.240 -14.554  55.554  1.00127.98
C
ANISOU10506  CD1 ILE B 549    13811  23860  10956  -2842    981
-812       C
ATOM  10507  N   PRO B 550      43.613 -19.277  58.540  1.00154.78
N
```

```
ANISOU10507  N   PRO B 550    16259  27787  14763  -1078    443
-2235       N
ATOM  10508  CA  PRO B 550      43.360 -20.641  59.020  1.00139.42
C
ANISOU10508  CA  PRO B 550    14362  25575  13036   -508    226
-2510       C
ATOM  10509  C   PRO B 550      41.876 -20.934  59.186  1.00120.16
C
ANISOU10509  C   PRO B 550    12399  22373  10886   -362    132
-2391       C
ATOM  10510  O   PRO B 550      41.064 -20.043  59.445  1.00126.48
O
ANISOU10510  O   PRO B 550    13524  22737  11797   -686    202
-2095       O
ATOM  10511  CB  PRO B 550      44.096 -20.683  60.364  1.00151.20
C
ANISOU10511  CB  PRO B 550    15764  27051  14636   -520    133
-2555       C
ATOM  10512  CG  PRO B 550      44.073 -19.268  60.837  1.00150.34
C
ANISOU10512  CG  PRO B 550    15800  26805  14516  -1108    278
-2227       C
ATOM  10513  CD  PRO B 550      44.196 -18.423  59.588  1.00149.46
C
ANISOU10513  CD  PRO B 550    15578  27089  14122  -1463    479
-2080       C
ATOM  10514  N   VAL B 551      41.539 -22.217  59.028  1.00131.42
N
ANISOU10514  N   VAL B 551    13856  23653  12426    138    -42
-2644       N
ATOM  10515  CA  VAL B 551      40.146 -22.661  58.977  1.00146.37
C
ANISOU10515  CA  VAL B 551    16147  24921  14546    299   -137
-2577       C
ATOM  10516  C   VAL B 551      39.391 -22.276  60.248  1.00126.95
C
ANISOU10516  C   VAL B 551    14050  21826  12359    121   -194
-2342       C
ATOM  10517  O   VAL B 551      38.205 -21.926  60.201  1.00 85.87
O
ANISOU10517  O   VAL B 551     9176  16171   7280      3   -169
-2149       O
ATOM  10518  CB  VAL B 551      40.091 -24.178  58.708  1.00131.36
C
ANISOU10518  CB  VAL B 551    14203  22986  12723    858   -349
-2918       C
ATOM  10519  CG1 VAL B 551      40.778 -24.496  57.389  1.00 96.25
C
ANISOU10519  CG1 VAL B 551     9385  19202   7984   1039   -281
-3179       C
ATOM  10520  CG2 VAL B 551      40.745 -24.962  59.839  1.00 98.89
C
ANISOU10520  CG2 VAL B 551    10026  18788   8758   1126   -556
-3082       C
```

```
ATOM  10521  N   GLU B 552      40.061 -22.345  61.401  1.00147.91
N
ANISOU10521  N   GLU B 552    16632  24473  15095    113   -275
-2369       N
ATOM  10522  CA  GLU B 552      39.459 -21.926  62.665  1.00 99.87
C
ANISOU10522  CA  GLU B 552    10846  17870   9230    -78   -320
-2164       C
ATOM  10523  C   GLU B 552      39.007 -20.469  62.641  1.00 84.69
C
ANISOU10523  C   GLU B 552     9092  15808   7279   -557   -132
-1869       C
ATOM  10524  O   GLU B 552      37.931 -20.139  63.154  1.00111.87
O
ANISOU10524  O   GLU B 552    12868  18739  10899   -658   -149
-1700       O
ATOM  10525  CB  GLU B 552      40.443 -22.154  63.814  1.00153.89
C
ANISOU10525  CB  GLU B 552    17516  24851  16103    -36   -422
-2251       C
ATOM  10526  CG  GLU B 552      40.767 -23.615  64.086  1.00167.77
C
ANISOU10526  CG  GLU B 552    19193  26604  17947    464   -666
-2512       C
ATOM  10527  CD  GLU B 552      41.985 -23.787  64.974  1.00155.66
C
ANISOU10527  CD  GLU B 552    17394  25380  16371    527   -755
-2625       C
ATOM  10528  OE1 GLU B 552      41.922 -23.390  66.158  1.00118.96
O
ANISOU10528  OE1 GLU B 552    12873  20496  11831    327   -784
-2473       O
ATOM  10529  OE2 GLU B 552      43.004 -24.320  64.488  1.00156.85
O
ANISOU10529  OE2 GLU B 552    17191  26038  16365    789   -799
-2880       O
ATOM  10530  N   GLU B 553      39.815 -19.582  62.060  1.00 88.05
N
ANISOU10530  N   GLU B 553     9293  16686   7475   -859     36
-1806       N
ATOM  10531  CA  GLU B 553      39.450 -18.169  61.977  1.00 86.51
C
ANISOU10531  CA  GLU B 553     9283  16336   7250  -1322    187
-1518       C
ATOM  10532  C   GLU B 553      38.205 -17.926  61.124  1.00 84.93
C
ANISOU10532  C   GLU B 553     9360  15824   7084  -1321    231
-1374       C
ATOM  10533  O   GLU B 553      37.321 -17.156  61.514  1.00 83.00
O
ANISOU10533  O   GLU B 553     9433  15125   6976  -1514    249
-1168       O
ATOM  10534  CB  GLU B 553      40.631 -17.371  61.420  1.00127.80
C
```

```
ANISOU10534  CB  GLU B 553    14203  22157  12197  -1663    340 
-1477       C
ATOM  10535  CG  GLU B 553      40.517 -15.871  61.612  1.00128.52 
C
ANISOU10535  CG  GLU B 553    14487  22071  12272  -2190    456 
-1182       C
ATOM  10536  CD  GLU B 553      40.775 -15.455  63.043  1.00116.38 
C
ANISOU10536  CD  GLU B 553    13033  20298  10889  -2361    404 
-1152       C
ATOM  10537  OE1 GLU B 553      39.860 -14.887  63.674  1.00121.56 
O
ANISOU10537  OE1 GLU B 553    14043  20401  11742  -2472    377 
-1003       O
ATOM  10538  OE2 GLU B 553      41.898 -15.691  63.533  1.00119.86 
O
ANISOU10538  OE2 GLU B 553    13166  21137  11238  -2375    387 
-1294       O
ATOM  10539  N   LEU B 554      38.118 -18.563  59.952  1.00 98.09 
N
ANISOU10539  N   LEU B 554    10902  17748   8619  -1093    241 
-1494       N
ATOM  10540  CA  LEU B 554      36.902 -18.464  59.142  1.00113.57 
C
ANISOU10540  CA  LEU B 554    13112  19432  10608  -1048    262 
-1380       C
ATOM  10541  C   LEU B 554      35.661 -18.990  59.859  1.00108.27 
C
ANISOU10541  C   LEU B 554    12764  18158  10217   -840    127 
-1376       C
ATOM  10542  O   LEU B 554      34.604 -18.350  59.829  1.00 79.43 
O
ANISOU10542  O   LEU B 554     9395  14133   6650   -969    155 
-1183       O
ATOM  10543  CB  LEU B 554      37.069 -19.220  57.820  1.00 87.71 
C
ANISOU10543  CB  LEU B 554     9617  16573   7137   -798    277 
-1564       C
ATOM  10544  CG  LEU B 554      37.948 -18.682  56.690  1.00 89.82 
C
ANISOU10544  CG  LEU B 554     9582  17484   7061  -1011    435 
-1538       C
ATOM  10545  CD1 LEU B 554      37.790 -17.175  56.523  1.00 90.25 
C
ANISOU10545  CD1 LEU B 554     9803  17453   7034  -1515    572 
-1177       C
ATOM  10546  CD2 LEU B 554      39.393 -19.079  56.887  1.00 98.97 
C
ANISOU10546  CD2 LEU B 554    10326  19211   8066   -951    437 
-1765       C
ATOM  10547  N   ILE B 555      35.769 -20.149  60.510  1.00 96.32 
N
ANISOU10547  N   ILE B 555    11210  16552   8836   -524    -31 
-1583       N
```

```
ATOM  10548  CA  ILE B 555      34.622 -20.741  61.198  1.00 77.76
C
ANISOU10548  CA  ILE B 555     9149  13673   6724   -357   -170
-1574       C
ATOM  10549  C   ILE B 555      34.052 -19.831  62.285  1.00 75.75
C
ANISOU10549  C   ILE B 555     9136  13032   6616   -624   -147
-1365       C
ATOM  10550  O   ILE B 555      32.827 -19.694  62.404  1.00119.16
O
ANISOU10550  O   ILE B 555    14894  18151  12230   -628   -168
-1262       O
ATOM  10551  CB  ILE B 555      35.013 -22.121  61.758  1.00 78.13
C
ANISOU10551  CB  ILE B 555     9111  13703   6872     -7   -367
-1811       C
ATOM  10552  CG1 ILE B 555      35.131 -23.130  60.615  1.00 79.79
C
ANISOU10552  CG1 ILE B 555     9180  14150   6986    325   -429
-2045       C
ATOM  10553  CG2 ILE B 555      34.010 -22.602  62.789  1.00 75.63
C
ANISOU10553  CG2 ILE B 555     9085  12863   6789     58   -514
-1752       C
ATOM  10554  CD1 ILE B 555      35.809 -24.418  60.999  1.00107.28
C
ANISOU10554  CD1 ILE B 555    12534  17697  10531    692   -633
-2311       C
ATOM  10555  N   GLU B 556      34.903 -19.190  63.088  1.00112.72
N
ANISOU10555  N   GLU B 556    13723  17823  11282   -846   -106
-1318       N
ATOM  10556  CA  GLU B 556      34.398 -18.142  63.976  1.00112.93
C
ANISOU10556  CA  GLU B 556    13971  17520  11417  -1125    -65
-1134       C
ATOM  10557  C   GLU B 556      33.757 -16.993  63.202  1.00112.63
C
ANISOU10557  C   GLU B 556    14103  17369  11322  -1353     59
-936       C
ATOM  10558  O   GLU B 556      32.649 -16.551  63.529  1.00115.72
O
ANISOU10558  O   GLU B 556    14761  17370  11839  -1389     44
-829       O
ATOM  10559  CB  GLU B 556      35.518 -17.628  64.882  1.00123.31
C
ANISOU10559  CB  GLU B 556    15135  19015  12701  -1344    -43
-1137       C
ATOM  10560  CG  GLU B 556      35.124 -16.400  65.692  1.00147.49
C
ANISOU10560  CG  GLU B 556    18413  21776  15850  -1661      7
-972       C
ATOM  10561  CD  GLU B 556      36.173 -16.006  66.712  1.00152.44
C
```

ANISOU10561 CD GLU B 556   18898 22562 16459 -1866   7
-1002   C
ATOM 10562 OE1 GLU B 556   35.953 -15.012 67.436 1.00141.70
O
ANISOU10562 OE1 GLU B 556   17703 20970 15167 -2126   36
-903   O
ATOM 10563 OE2 GLU B 556   37.212 -16.693 66.794 1.00143.87
O
ANISOU10563 OE2 GLU B 556   17532 21841 15290 -1752   -33
-1142   O
ATOM 10564 N   LYS B 557   34.445 -16.491 62.176 1.00115.96
N
ANISOU10564 N   LYS B 557   14368 18149 11542 -1506   173
-884   N
ATOM 10565 CA LYS B 557   33.910 -15.393 61.372 1.00119.85
C
ANISOU10565 CA LYS B 557   15032 18542 11962 -1733   270
-666   C
ATOM 10566 C   LYS B 557   32.631 -15.779 60.638 1.00125.56
C
ANISOU10566 C   LYS B 557   15930 19050 12727 -1507   237
-646   C
ATOM 10567 O   LYS B 557   31.687 -14.984 60.565 1.00 96.53
O
ANISOU10567 O   LYS B 557   12512 15050   9115 -1603   248
-480   O
ATOM 10568 CB LYS B 557   34.968 -14.884 60.392 1.00127.76
C
ANISOU10568 CB LYS B 557   15806 20035 12703 -1967   391
-602   C
ATOM 10569 CG LYS B 557   35.831 -13.748 60.941 1.00138.66
C
ANISOU10569 CG LYS B 557   17167 21483 14034 -2388   456
-470   C
ATOM 10570 CD LYS B 557   36.462 -14.081 62.284 1.00112.83
C
ANISOU10570 CD LYS B 557   13789 18205 10878 -2365   391
-616   C
ATOM 10571 CE LYS B 557   37.475 -13.026 62.694 1.00103.99
C
ANISOU10571 CE LYS B 557   12590 17256   9666 -2801   461
-516   C
ATOM 10572 NZ LYS B 557   36.843 -11.687 62.861 1.00100.72
N
ANISOU10572 NZ LYS B 557   12525 16406   9339 -3114   479
-282   N
ATOM 10573 N   ALA B 558   32.588 -16.985 60.076 1.00138.18
N
ANISOU10573 N   ALA B 558   17387 20829 14286 -1200   184
-828   N
ATOM 10574 CA ALA B 558   31.489 -17.383 59.204 1.00123.05
C
ANISOU10574 CA ALA B 558   15592 18796 12366 -1007   159
-827   C

```
ATOM  10575  C   ALA B 558      30.136 -17.291  59.899  1.00 83.52
C
ANISOU10575  C   ALA B 558    10877  13292   7563   -949     84
-760       C
ATOM  10576  O   ALA B 558      30.002 -17.536  61.100  1.00 88.80
O
ANISOU10576  O   ALA B 558    11615  13731   8395   -924      8
-809       O
ATOM  10577  CB  ALA B 558      31.696 -18.812  58.706  1.00114.88
C
ANISOU10577  CB  ALA B 558    14373  17985  11290   -670     79
-1083       C
ATOM  10578  N   ASP B 559      29.127 -16.928  59.110  1.00 90.99
N
ANISOU10578  N   ASP B 559    11977  14118   8477   -928    106
-649       N
ATOM  10579  CA  ASP B 559      27.752 -16.779  59.561  1.00102.46
C
ANISOU10579  CA  ASP B 559    13676  15174  10080   -861     45
-593       C
ATOM  10580  C   ASP B 559      26.941 -18.048  59.344  1.00128.15
C
ANISOU10580  C   ASP B 559    16930  18376  13384   -581    -55
-750       C
ATOM  10581  O   ASP B 559      25.733 -18.052  59.601  1.00140.16
O
ANISOU10581  O   ASP B 559    18619  19635  14999   -518   -106
-720       O
ATOM  10582  CB  ASP B 559      27.085 -15.602  58.842  1.00 97.13
C
ANISOU10582  CB  ASP B 559    13174  14392   9340   -987    104
-378       C
ATOM  10583  CG  ASP B 559      27.698 -14.265  59.220  1.00100.01
C
ANISOU10583  CG  ASP B 559    13621  14682   9697  -1291    166
-204       C
ATOM  10584  OD1 ASP B 559      27.623 -13.322  58.406  1.00 94.42
O
ANISOU10584  OD1 ASP B 559    13010  13986   8880  -1440    215
-10       O
ATOM  10585  OD2 ASP B 559      28.263 -14.156  60.329  1.00101.57
O
ANISOU10585  OD2 ASP B 559    13795  14803   9993  -1394    154
-255       O
ATOM  10586  N   GLY B 560      27.579 -19.109  58.874  1.00125.66
N
ANISOU10586  N   GLY B 560    16429  18314  13002   -416    -92
-928       N
ATOM  10587  CA  GLY B 560      26.913 -20.370  58.617  1.00123.22
C
ANISOU10587  CA  GLY B 560    16133  17943  12741   -163   -209
-1095       C
ATOM  10588  C   GLY B 560      27.783 -21.257  57.760  1.00 98.20
C
```

```
ANISOU10588  C   GLY B 560    12743  15122   9447     14   -228
-1295       C
ATOM  10589  O   GLY B 560      28.821 -20.844  57.243  1.00119.03
O
ANISOU10589  O   GLY B 560    15193  18104  11927    -65   -131
-1293       O
ATOM  10590  N   PHE B 561      27.357 -22.514  57.642  1.00 75.52
N
ANISOU10590  N   PHE B 561     9891  12164   6641    253   -368
-1484       N
ATOM  10591  CA  PHE B 561      28.104 -23.521  56.899  1.00 72.89
C
ANISOU10591  CA  PHE B 561     9365  12116   6216    488   -429
-1735       C
ATOM  10592  C   PHE B 561      27.138 -24.350  56.068  1.00 78.73
C
ANISOU10592  C   PHE B 561    10182  12787   6944    672   -517
-1861       C
ATOM  10593  O   PHE B 561      26.159 -24.879  56.602  1.00 88.20
O
ANISOU10593  O   PHE B 561    11573  13641   8297    702   -636
-1858       O
ATOM  10594  CB  PHE B 561      28.910 -24.417  57.849  1.00 73.32
C
ANISOU10594  CB  PHE B 561     9359  12103   6395    630   -573
-1898       C
ATOM  10595  CG  PHE B 561      29.773 -23.649  58.810  1.00 73.20
C
ANISOU10595  CG  PHE B 561     9276  12135   6402    439   -504
-1780       C
ATOM  10596  CD1 PHE B 561      29.379 -23.474  60.125  1.00 71.37
C
ANISOU10596  CD1 PHE B 561     9213  11561   6342    317   -560
-1657       C
ATOM  10597  CD2 PHE B 561      30.971 -23.091  58.395  1.00 87.31
C
ANISOU10597  CD2 PHE B 561    10815  14341   8016    358   -381
-1796       C
ATOM  10598  CE1 PHE B 561      30.162 -22.755  61.009  1.00 71.40
C
ANISOU10598  CE1 PHE B 561     9152  11616   6359    135   -499
-1564       C
ATOM  10599  CE2 PHE B 561      31.759 -22.373  59.277  1.00 81.47
C
ANISOU10599  CE2 PHE B 561    10010  13653   7291    155   -322
-1693       C
ATOM  10600  CZ  PHE B 561      31.353 -22.206  60.583  1.00 75.51
C
ANISOU10600  CZ  PHE B 561     9438  12532   6722     51   -384
-1583       C
ATOM  10601  N   ALA B 562      27.411 -24.462  54.769  1.00 97.83
N
ANISOU10601  N   ALA B 562    12442  15564   9166    772   -459
-1975       N
```

```
ATOM  10602  CA  ALA B 562      26.569 -25.230  53.864  1.00 75.16
C
ANISOU10602  CA  ALA B 562     9619  12684   6256    946   -538
-2122       C
ATOM  10603  C   ALA B 562      27.209 -26.572  53.516  1.00 77.23
C
ANISOU10603  C   ALA B 562     9754  13075   6515   1252   -688
-2475       C
ATOM  10604  O   ALA B 562      28.398 -26.807  53.743  1.00108.21
O
ANISOU10604  O   ALA B 562    13500  17202  10414   1352   -704
-2610       O
ATOM  10605  CB  ALA B 562      26.289 -24.433  52.587  1.00 83.19
C
ANISOU10605  CB  ALA B 562    10561  14014   7035    857   -384
-2012       C
ATOM  10606  N   GLY B 563      26.391 -27.455  52.946  1.00 80.48
N
ANISOU10606  N   GLY B 563    10259  13369   6949   1412   -811
-2639       N
ATOM  10607  CA  GLY B 563      26.854 -28.769  52.529  1.00 92.75
C
ANISOU10607  CA  GLY B 563    11737  14991   8513   1729   -986
-3004       C
ATOM  10608  C   GLY B 563      27.382 -29.616  53.662  1.00 91.18
C
ANISOU10608  C   GLY B 563    11617  14494   8533   1862  -1184
-3114       C
ATOM  10609  O   GLY B 563      28.217 -30.499  53.437  1.00 91.08
O
ANISOU10609  O   GLY B 563    11483  14608   8514   2150  -1317
-3416       O
ATOM  10610  N   VAL B 564      26.898 -29.382  54.879  1.00 91.11
N
ANISOU10610  N   VAL B 564    11810  14100   8709   1674  -1221
-2884       N
ATOM  10611  CA  VAL B 564      27.446 -30.009  56.078  1.00101.27
C
ANISOU10611  CA  VAL B 564    13172  15123  10182   1748  -1395
-2919       C
ATOM  10612  C   VAL B 564      27.085 -31.493  56.084  1.00109.66
C
ANISOU10612  C   VAL B 564    14409  15865  11390   1981  -1684
-3152       C
ATOM  10613  O   VAL B 564      25.910 -31.858  56.171  1.00119.58
O
ANISOU10613  O   VAL B 564    15891  16809  12735   1878  -1773
-3084       O
ATOM  10614  CB  VAL B 564      26.949 -29.311  57.349  1.00111.46
C
ANISOU10614  CB  VAL B 564    14622  16128  11598   1458  -1347
-2606       C
ATOM  10615  CG1 VAL B 564      27.473 -30.025  58.578  1.00124.30
C
```

ANISOU10615 CG1 VAL B 564   16334 17494 13400   1530 -1543
-2631   C
ATOM 10616 CG2 VAL B 564   27.383 -27.849 57.351 1.00110.73
C
ANISOU10616 CG2 VAL B 564   14384 16311 11379   1237 -1089
-2398   C
ATOM 10617 N   PHE B 565   28.091 -32.347 55.988 1.00116.87
N
ANISOU10617 N   PHE B 565   15221 16860 12325   2292 -1844
-3433   N
ATOM 10618 CA PHE B 565   27.897 -33.749 56.307 1.00118.13
C
ANISOU10618 CA PHE B 565   15599 16613 12671   2506 -2171
-3622   C
ATOM 10619 C   PHE B 565   28.089 -33.963 57.802 1.00129.29
C
ANISOU10619 C   PHE B 565   17177 17673 14275   2419 -2317
-3443   C
ATOM 10620 O   PHE B 565   28.758 -33.169 58.471 1.00146.10
O
ANISOU10620 O   PHE B 565   19170 19969 16373   2306 -2181
-3286   O
ATOM 10621 CB PHE B 565   28.866 -34.615 55.505 1.00159.92
C
ANISOU10621 CB PHE B 565   20724 22134 17906   2932 -2313
-4039   C
ATOM 10622 CG PHE B 565   28.472 -34.792 54.064 1.00148.99
C
ANISOU10622 CG PHE B 565   19248 21001 16361   3049 -2258
-4268   C
ATOM 10623 CD1 PHE B 565   29.138 -34.116 53.055 1.00102.64
C
ANISOU10623 CD1 PHE B 565   13036 15735 10226   3111 -2028
-4378   C
ATOM 10624 CD2 PHE B 565   27.425 -35.636 53.722 1.00129.81
C
ANISOU10624 CD2 PHE B 565   17073 18222 14028   3070 -2440
-4367   C
ATOM 10625 CE1 PHE B 565   28.768 -34.280 51.731 1.00153.14
C
ANISOU10625 CE1 PHE B 565   19341 22396 16450   3211 -1980
-4585   C
ATOM 10626 CE2 PHE B 565   27.048 -35.804 52.401 1.00104.98
C
ANISOU10626 CE2 PHE B 565   13839 15325 10724   3174 -2394
-4590   C
ATOM 10627 CZ PHE B 565   27.719 -35.126 51.405 1.00154.25
C
ANISOU10627 CZ PHE B 565   19733 22180 16695   3254 -2163
-4700   C
ATOM 10628 N   PRO B 566   27.478 -35.014 58.365 1.00137.93
N
ANISOU10628 N   PRO B 566   18572 18277 15557   2438 -2599
-3447   N

```
ATOM  10629  CA  PRO B 566      27.734 -35.357  59.779  1.00148.20
C
ANISOU10629  CA  PRO B 566    20035  19252  17023   2377  -2778
-3285       C
ATOM  10630  C   PRO B 566      29.198 -35.338  60.206  1.00130.33
C
ANISOU10630  C   PRO B 566    17568  17201  14752   2612  -2814
-3390       C
ATOM  10631  O   PRO B 566      29.506 -34.869  61.310  1.00118.58
O
ANISOU10631  O   PRO B 566    16075  15677  13302   2447  -2778
-3169       O
ATOM  10632  CB  PRO B 566      27.135 -36.768  59.888  1.00134.46
C
ANISOU10632  CB  PRO B 566    18622  17013  15455   2477  -3135
-3392       C
ATOM  10633  CG  PRO B 566      25.984 -36.731  58.933  1.00121.91
C
ANISOU10633  CG  PRO B 566    17095  15428  13796   2347  -3050
-3424       C
ATOM  10634  CD  PRO B 566      26.490 -35.924  57.755  1.00106.34
C
ANISOU10634  CD  PRO B 566    14791  14000  11615   2475  -2775
-3580       C
ATOM  10635  N   GLU B 567      30.110 -35.832  59.365  1.00104.13
N
ANISOU10635  N   GLU B 567    14060  14137  11368   2997  -2886
-3739       N
ATOM  10636  CA  GLU B 567      31.537 -35.749  59.672  1.00104.85
C
ANISOU10636  CA  GLU B 567    13894  14533  11412   3233  -2900
-3868       C
ATOM  10637  C   GLU B 567      32.020 -34.306  59.790  1.00 95.23
C
ANISOU10637  C   GLU B 567    12390  13772  10023   2974  -2548
-3675       C
ATOM  10638  O   GLU B 567      32.808 -33.981  60.686  1.00122.97
O
ANISOU10638  O   GLU B 567    15798  17376  13548   2942  -2541
-3579       O
ATOM  10639  CB  GLU B 567      32.344 -36.498  58.611  1.00120.04
C
ANISOU10639  CB  GLU B 567    15625  16724  13260   3703  -3020
-4318       C
ATOM  10640  CG  GLU B 567      32.087 -37.996  58.592  1.00147.08
C
ANISOU10640  CG  GLU B 567    19347  19658  16880   4018  -3426
-4552       C
ATOM  10641  CD  GLU B 567      33.010 -38.739  57.645  1.00174.77
C
ANISOU10641  CD  GLU B 567    22644  23445  20315   4544  -3571
-5045       C
ATOM  10642  OE1 GLU B 567      33.793 -39.588  58.122  1.00178.44
O
```

```
ANISOU10642  OE1 GLU B 567    23150  23750  20900   4909  -3872
-5236       O
ATOM  10643  OE2 GLU B 567      32.959 -38.475  56.426  1.00198.25
O
ANISOU10643  OE2 GLU B 567    25405  26819  23101   4607  -3393
-5250       O
ATOM  10644  N   HIS B 568      31.562 -33.427  58.896  1.00 92.92
N
ANISOU10644  N   HIS B 568    11978  13760   9566   2780  -2271
-3612       N
ATOM  10645  CA  HIS B 568      31.907 -32.010  58.989  1.00106.61
C
ANISOU10645  CA  HIS B 568    13500  15858  11147   2487  -1955
-3396       C
ATOM  10646  C   HIS B 568      31.355 -31.378  60.261  1.00107.14
C
ANISOU10646  C   HIS B 568    13756  15619  11332   2139  -1908
-3043       C
ATOM  10647  O   HIS B 568      32.031 -30.574  60.914  1.00 99.12
O
ANISOU10647  O   HIS B 568    12601  14789  10272   1985  -1782
-2910       O
ATOM  10648  CB  HIS B 568      31.366 -31.259  57.769  1.00117.09
C
ANISOU10648  CB  HIS B 568    14731  17467  12289   2343  -1711
-3367       C
ATOM  10649  CG  HIS B 568      31.710 -31.890  56.454  1.00131.79
C
ANISOU10649  CG  HIS B 568    16419  19641  14013   2658  -1750
-3716       C
ATOM  10650  ND1 HIS B 568      31.292 -31.363  55.251  1.00144.63
N
ANISOU10650  ND1 HIS B 568    17941  21566  15445   2572  -1559
-3724       N
ATOM  10651  CD2 HIS B 568      32.413 -33.007  56.150  1.00135.81
C
ANISOU10651  CD2 HIS B 568    16842  20217  14543   3075  -1970
-4083       C
ATOM  10652  CE1 HIS B 568      31.729 -32.124  54.264  1.00162.72
C
ANISOU10652  CE1 HIS B 568    20071  24126  17628   2905  -1645
-4087       C
ATOM  10653  NE2 HIS B 568      32.413 -33.127  54.782  1.00164.85
N
ANISOU10653  NE2 HIS B 568    20349  24256  18031   3227  -1897
-4325       N
ATOM  10654  N   LYS B 569      30.117 -31.724  60.613  1.00132.33
N
ANISOU10654  N   LYS B 569    17250  18373  14654   2002  -2006
-2903       N
ATOM  10655  CA  LYS B 569      29.412 -31.094  61.725  1.00122.76
C
ANISOU10655  CA  LYS B 569    16206  16915  13523   1663  -1944
-2587       C
```

```
ATOM  10656  C   LYS B 569      30.156 -31.254  63.052  1.00113.72
C
ANISOU10656  C   LYS B 569    15069  15671  12470   1659  -2067
-2509       C
ATOM  10657  O   LYS B 569      30.244 -30.305  63.841  1.00 90.01
O
ANISOU10657  O   LYS B 569    12024  12730   9446   1405  -1920
-2304       O
ATOM  10658  CB  LYS B 569      28.002 -31.684  61.797  1.00 98.33
C
ANISOU10658  CB  LYS B 569    13406  13422  10533   1558  -2069
-2507       C
ATOM  10659  CG  LYS B 569      27.044 -31.017  62.752  1.00125.90
C
ANISOU10659  CG  LYS B 569    17048  16722  14068   1207  -1984
-2210       C
ATOM  10660  CD  LYS B 569      25.624 -31.464  62.436  1.00 99.83
C
ANISOU10660  CD  LYS B 569    13956  13176  10801   1102  -2052
-2170       C
ATOM  10661  CE  LYS B 569      24.610 -30.887  63.405  1.00110.72
C
ANISOU10661  CE  LYS B 569    15460  14404  12204    773  -1984
-1907       C
ATOM  10662  NZ  LYS B 569      23.232 -31.344  63.073  1.00 89.93
N
ANISOU10662  NZ  LYS B 569    12995  11594   9581    662  -2050
-1882       N
ATOM  10663  N   TYR B 570      30.691 -32.448  63.316  1.00123.93
N
ANISOU10663  N   TYR B 570    16422  16800  13864   1948  -2353
-2677       N
ATOM  10664  CA  TYR B 570      31.550 -32.669  64.480  1.00104.54
C
ANISOU10664  CA  TYR B 570    13944  14302  11476   2004  -2495
-2629       C
ATOM  10665  C   TYR B 570      32.796 -31.781  64.473  1.00101.50
C
ANISOU10665  C   TYR B 570    13213  14402  10950   2015  -2303
-2676       C
ATOM  10666  O   TYR B 570      33.186 -31.240  65.514  1.00 80.45
O
ANISOU10666  O   TYR B 570    10505  11775   8289   1841  -2260
-2513       O
ATOM  10667  CB  TYR B 570      31.948 -34.148  64.521  1.00 88.80
C
ANISOU10667  CB  TYR B 570    12076  12057   9606   2382  -2862
-2840       C
ATOM  10668  CG  TYR B 570      32.886 -34.562  65.637  1.00102.95
C
ANISOU10668  CG  TYR B 570    13854  13795  11469   2518  -3068
-2818       C
ATOM  10669  CD1 TYR B 570      34.264 -34.435  65.502  1.00111.24
C
```

```
ANISOU10669  CD1 TYR B 570    14586  15252  12428   2779  -3050
-3016       C
ATOM  10670  CD2 TYR B 570      32.395 -35.133  66.803  1.00127.30
C
ANISOU10670  CD2 TYR B 570    17232  16445  14690   2390  -3298
-2603       C
ATOM  10671  CE1 TYR B 570      35.122 -34.817  66.521  1.00144.31
C
ANISOU10671  CE1 TYR B 570    18748  19413  16671   2922  -3251
-2999       C
ATOM  10672  CE2 TYR B 570      33.247 -35.535  67.818  1.00140.73
C
ANISOU10672  CE2 TYR B 570    18928  18099  16443   2522  -3506
-2567       C
ATOM  10673  CZ  TYR B 570      34.609 -35.369  67.676  1.00149.05
C
ANISOU10673  CZ  TYR B 570    19662  19554  17416   2801  -3484
-2770       C
ATOM  10674  OH  TYR B 570      35.458 -35.773  68.683  1.00120.18
O
ANISOU10674  OH  TYR B 570    15987  15872  13803   2953  -3703
-2740       O
ATOM  10675  N   GLU B 571      33.456 -31.651  63.322  1.00117.81
N
ANISOU10675  N   GLU B 571    15019  16868  12874   2206  -2194
-2907       N
ATOM  10676  CA  GLU B 571      34.644 -30.802  63.223  1.00123.67
C
ANISOU10676  CA  GLU B 571    15409  18129  13451   2175  -2003
-2953       C
ATOM  10677  C   GLU B 571      34.347 -29.320  63.451  1.00103.91
C
ANISOU10677  C   GLU B 571    12869  15750  10863   1735  -1703
-2679       C
ATOM  10678  O   GLU B 571      35.145 -28.614  64.081  1.00106.30
O
ANISOU10678  O   GLU B 571    13004  16281  11106   1589  -1606
-2602       O
ATOM  10679  CB  GLU B 571      35.313 -30.998  61.861  1.00164.03
C
ANISOU10679  CB  GLU B 571    20241  23687  18394   2446  -1945
-3258       C
ATOM  10680  CG  GLU B 571      36.752 -30.497  61.802  1.00164.88
C
ANISOU10680  CG  GLU B 571    19948  24375  18323   2500  -1831
-3378       C
ATOM  10681  CD  GLU B 571      37.705 -31.360  62.608  1.00141.82
C
ANISOU10681  CD  GLU B 571    16952  21447  15485   2824  -2095
-3542       C
ATOM  10682  OE1 GLU B 571      38.388 -30.818  63.502  1.00137.17
O
ANISOU10682  OE1 GLU B 571    16235  21012  14871   2675  -2047
-3424       O
```

```
ATOM  10683  OE2 GLU B 571      37.773 -32.579  62.345  1.00144.42
O
ANISOU10683  OE2 GLU B 571    17358  21610  15905   3236  -2364
-3795       O
ATOM  10684  N   ILE B 572      33.212 -28.828  62.947  1.00 87.03
N
ANISOU10684  N   ILE B 572    10889  13460   8718   1530  -1567
-2541       N
ATOM  10685  CA  ILE B 572      32.849 -27.418  63.117  1.00119.98
C
ANISOU10685  CA  ILE B 572    15065  17695  12826   1146  -1312
-2292       C
ATOM  10686  C   ILE B 572      32.750 -27.013  64.587  1.00119.98
C
ANISOU10686  C   ILE B 572    15183  17474  12931    924  -1336
-2091       C
ATOM  10687  O   ILE B 572      33.327 -26.000  65.001  1.00104.92
O
ANISOU10687  O   ILE B 572    13147  15764  10953    705  -1184
-1991       O
ATOM  10688  CB  ILE B 572      31.535 -27.118  62.372  1.00109.55
C
ANISOU10688  CB  ILE B 572    13922  16203  11498   1027  -1217
-2193       C
ATOM  10689  CG1 ILE B 572      31.740 -27.220  60.860  1.00 76.51
C
ANISOU10689  CG1 ILE B 572     9573  12344   7155   1185  -1137
-2369       C
ATOM  10690  CG2 ILE B 572      31.010 -25.743  62.748  1.00113.02
C
ANISOU10690  CG2 ILE B 572    14433  16595  11916    667  -1013
-1933       C
ATOM  10691  CD1 ILE B 572      30.455 -27.296  60.077  1.00 75.34
C
ANISOU10691  CD1 ILE B 572     9601  12017   7009   1160  -1114
-2330       C
ATOM  10692  N   VAL B 573      32.022 -27.779  65.399  1.00146.86
N
ANISOU10692  N   VAL B 573    18831  20482  16490    952  -1530
-2028       N
ATOM  10693  CA  VAL B 573      32.010 -27.506  66.837  1.00135.84
C
ANISOU10693  CA  VAL B 573    17519  18928  15166    761  -1572
-1859       C
ATOM  10694  C   VAL B 573      33.396 -27.680  67.455  1.00100.65
C
ANISOU10694  C   VAL B 573    12859  14699  10684    878  -1654
-1945       C
ATOM  10695  O   VAL B 573      33.814 -26.882  68.303  1.00 74.68
O
ANISOU10695  O   VAL B 573     9494  11516   7365    663  -1563
-1834       O
ATOM  10696  CB  VAL B 573      30.944 -28.364  67.549  1.00114.53
C
```

```
ANISOU10696  CB  VAL B 573    15111  15801  12606    743  -1774
-1761       C
ATOM  10697  CG1 VAL B 573      31.116 -29.837  67.242  1.00121.56
C
ANISOU10697  CG1 VAL B 573    16083  16528  13576   1069  -2053
-1929       C
ATOM  10698  CG2 VAL B 573      30.977 -28.121  69.054  1.00 71.79
C
ANISOU10698  CG2 VAL B 573     9761  10280   7234    544  -1823
-1591       C
ATOM  10699  N   LYS B 574      34.135 -28.710  67.036  1.00111.03
N
ANISOU10699  N   LYS B 574    14074  16109  12004   1232  -1835
-2164       N
ATOM  10700  CA  LYS B 574      35.499 -28.897  67.528  1.00 94.52
C
ANISOU10700  CA  LYS B 574    11750  14294   9869   1391  -1921
-2278       C
ATOM  10701  C   LYS B 574      36.414 -27.712  67.225  1.00 94.88
C
ANISOU10701  C   LYS B 574    11480  14829   9739   1215  -1663
-2292       C
ATOM  10702  O   LYS B 574      37.180 -27.281  68.094  1.00103.68
O
ANISOU10702  O   LYS B 574    12461  16113  10820   1101  -1647
-2243       O
ATOM  10703  CB  LYS B 574      36.089 -30.181  66.941  1.00127.40
C
ANISOU10703  CB  LYS B 574    15850  18500  14059   1854  -2161
-2556       C
ATOM  10704  CG  LYS B 574      37.527 -30.455  67.363  1.00113.85
C
ANISOU10704  CG  LYS B 574    13861  17113  12286   2089  -2274
-2715       C
ATOM  10705  CD  LYS B 574      37.965 -31.857  66.964  1.00 89.14
C
ANISOU10705  CD  LYS B 574    10736  13912   9222   2597  -2581
-2995       C
ATOM  10706  CE  LYS B 574      39.397 -32.137  67.393  1.00 92.29
C
ANISOU10706  CE  LYS B 574    10842  14667   9556   2874  -2711
-3171       C
ATOM  10707  NZ  LYS B 574      40.375 -31.310  66.637  1.00 93.46
N
ANISOU10707  NZ  LYS B 574    10553  15484   9474   2843  -2453
-3330       N
ATOM  10708  N   LYS B 575      36.359 -27.171  66.006  1.00143.86
N
ANISOU10708  N   LYS B 575    17565  21277  15819   1165  -1466
-2350       N
ATOM  10709  CA  LYS B 575      37.096 -25.941  65.712  1.00153.97
C
ANISOU10709  CA  LYS B 575    18592  22987  16924    907  -1214
-2306       C
```

```
ATOM  10710  C   LYS B 575      36.659 -24.775  66.595  1.00126.94
C
ANISOU10710  C   LYS B 575    15302  19393  13536    492  -1074
-2046       C
ATOM  10711  O   LYS B 575      37.499 -24.041  67.128  1.00125.73
O
ANISOU10711  O   LYS B 575    14976  19490  13304    296   -987
-2007       O
ATOM  10712  CB  LYS B 575      36.948 -25.583  64.233  1.00140.01
C
ANISOU10712  CB  LYS B 575    16720  21471  15007    895  -1043
-2368       C
ATOM  10713  CG  LYS B 575      38.107 -26.044  63.353  1.00107.83
C
ANISOU10713  CG  LYS B 575    12287  17926  10756   1166  -1052
-2647       C
ATOM  10714  CD  LYS B 575      38.539 -27.467  63.662  1.00 92.21
C
ANISOU10714  CD  LYS B 575    10284  15873   8877   1625  -1344
-2895       C
ATOM  10715  CE  LYS B 575      39.600 -27.939  62.682  1.00129.78
C
ANISOU10715  CE  LYS B 575    14674  21186  13450   1943  -1356
-3221       C
ATOM  10716  NZ  LYS B 575      40.154 -29.268  63.063  1.00158.78
N
ANISOU10716  NZ  LYS B 575    18313  24792  17225   2426  -1667
-3482       N
ATOM  10717  N   LEU B 576      35.349 -24.580  66.753  1.00 76.03
N
ANISOU10717  N   LEU B 576     9150  12540   7196    357  -1056
-1886       N
ATOM  10718  CA  LEU B 576      34.856 -23.583  67.700  1.00 74.10
C
ANISOU10718  CA  LEU B 576     9049  12105   7001     20   -961
-1679       C
ATOM  10719  C   LEU B 576      35.263 -23.902  69.135  1.00100.47
C
ANISOU10719  C   LEU B 576    12396  15366  10414     11  -1105
-1654       C
ATOM  10720  O   LEU B 576      35.663 -23.007  69.889  1.00 75.23
O
ANISOU10720  O   LEU B 576     9140  12262   7184   -242  -1014
-1573       O
ATOM  10721  CB  LEU B 576      33.337 -23.467  67.583  1.00 86.78
C
ANISOU10721  CB  LEU B 576    10943  13333   8698    -60   -940
-1553       C
ATOM  10722  CG  LEU B 576      32.799 -22.914  66.262  1.00 93.41
C
ANISOU10722  CG  LEU B 576    11802  14231   9458   -106   -782
-1529       C
ATOM  10723  CD1 LEU B 576      31.296 -23.126  66.163  1.00 69.47
C
```

ANISOU10723 CD1 LEU B 576   9031 10846   6518   -100   -817
-1448   C
ATOM 10724 CD2 LEU B 576   33.145 -21.436 66.132 1.00 71.54
C
ANISOU10724 CD2 LEU B 576   8968 11624   6592   -416   -573
-1408   C
ATOM 10725 N   GLN B 577   35.163 -25.174 69.529 1.00148.28
N
ANISOU10725 N   GLN B 577   18536 21241 16563   275 -1345
-1719   N
ATOM 10726 CA GLN B 577   35.625 -25.602 70.848 1.00134.78
C
ANISOU10726 CA GLN B 577   16827 19480 14903   297 -1516
-1687   C
ATOM 10727 C   GLN B 577   37.126 -25.394 71.025 1.00127.68
C
ANISOU10727 C   GLN B 577   15609 19008 13895   348 -1504
-1804   C
ATOM 10728 O   GLN B 577   37.576 -24.947 72.086 1.00114.53
O
ANISOU10728 O   GLN B 577   13885 17421 12212   177 -1504
-1734   O
ATOM 10729 CB GLN B 577   35.251 -27.069 71.059 1.00111.51
C
ANISOU10729 CB GLN B 577   14057 16240 12073   585 -1804
-1725   C
ATOM 10730 CG GLN B 577   35.568 -27.631 72.424 1.00 94.69
C
ANISOU10730 CG GLN B 577   11983 13999   9996   610 -2020
-1650   C
ATOM 10731 CD GLN B 577   34.974 -29.011 72.611 1.00119.40
C
ANISOU10731 CD GLN B 577   15365 16753 13249   822 -2314
-1630   C
ATOM 10732 OE1 GLN B 577   35.695 -30.004 72.696 1.00143.43
O
ANISOU10732 OE1 GLN B 577   18377 19792 16329   1136 -2559
-1742   O
ATOM 10733 NE2 GLN B 577   33.648 -29.083 72.650 1.00103.62
N
ANISOU10733 NE2 GLN B 577   13623 14436 11310   654 -2305
-1493   N
ATOM 10734 N   GLU B 578   37.915 -25.711 69.995 1.00149.89
N
ANISOU10734 N   GLU B 578   18194 22140 16618   578 -1493
-1996   N
ATOM 10735 CA GLU B 578   39.333 -25.355 69.986 1.00129.00
C
ANISOU10735 CA GLU B 578   15191 20000 13825   586 -1436
-2120   C
ATOM 10736 C   GLU B 578   39.554 -23.849 70.070 1.00119.03
C
ANISOU10736 C   GLU B 578   13829 18942 12453   147 -1172
-1997   C

```
ATOM  10737  O   GLU B 578      40.544 -23.404  70.661  1.00131.06
O
ANISOU10737  O   GLU B 578    15132  20777  13888     21  -1145
-2020       O
ATOM  10738  CB  GLU B 578      40.028 -25.934  68.754  1.00146.84
C
ANISOU10738  CB  GLU B 578    17205  22612  15977    907  -1452
-2368       C
ATOM  10739  CG  GLU B 578      40.287 -27.429  68.863  1.00153.92
C
ANISOU10739  CG  GLU B 578    18122  23396  16965   1390  -1763
-2553       C
ATOM  10740  CD  GLU B 578      41.205 -27.947  67.779  1.00161.92
C
ANISOU10740  CD  GLU B 578    18821  24857  17845   1740  -1789
-2858       C
ATOM  10741  OE1 GLU B 578      40.781 -28.843  67.020  1.00164.19
O
ANISOU10741  OE1 GLU B 578    19216  24977  18193   2051  -1916
-3007       O
ATOM  10742  OE2 GLU B 578      42.353 -27.461  67.694  1.00129.68
O
ANISOU10742  OE2 GLU B 578    14371  21318  13584   1699  -1686
-2963       O
ATOM  10743  N   ARG B 579      38.654 -23.050  69.498  1.00108.73
N
ANISOU10743  N   ARG B 579    12695  17462  11157    -89   -995
-1867       N
ATOM  10744  CA  ARG B 579      38.730 -21.604  69.661  1.00102.69
C
ANISOU10744  CA  ARG B 579    11917  16778  10322   -513   -784
-1730       C
ATOM  10745  C   ARG B 579      38.063 -21.126  70.947  1.00104.57
C
ANISOU10745  C   ARG B 579    12383  16672  10676   -733   -805
-1579       C
ATOM  10746  O   ARG B 579      37.817 -19.923  71.094  1.00130.73
O
ANISOU10746  O   ARG B 579    15777  19922  13974  -1066   -652
-1466       O
ATOM  10747  CB  ARG B 579      38.093 -20.911  68.451  1.00136.39
C
ANISOU10747  CB  ARG B 579    16275  21010  14538   -652   -604
-1653       C
ATOM  10748  CG  ARG B 579      38.929 -21.001  67.179  1.00155.41
C
ANISOU10748  CG  ARG B 579    18399  23889  16761   -561   -521
-1783       C
ATOM  10749  CD  ARG B 579      40.135 -20.077  67.204  1.00154.01
C
ANISOU10749  CD  ARG B 579    17941  24171  16406   -856   -385
-1778       C
ATOM  10750  NE  ARG B 579      39.755 -18.677  67.061  1.00178.76
N
```

ANISOU10750 NE   ARG B 579   21225 27184 19512 -1289   -207
-1570   N
ATOM 10751 CZ ARG B 579   40.338 -17.829 66.221 1.00181.35
C
ANISOU10751 CZ ARG B 579   21396 27857 19650 -1563   -44
-1514   C
ATOM 10752 NH1 ARG B 579   41.433 -18.188 65.565 1.00174.50
N
ANISOU10752 NH1 ARG B 579   20161 27561 18581 -1476   -16
-1667   N
ATOM 10753 NH2 ARG B 579   39.910 -16.577 66.144 1.00190.41
N
ANISOU10753 NH2 ARG B 579   22745 28802 20801 -1945   78
-1306   N
ATOM 10754 N   LYS B 580   37.727 -22.064 71.836 1.00115.53
N
ANISOU10754 N   LYS B 580   13889 17833 12175   -547 -1005
-1580   N
ATOM 10755 CA LYS B 580   37.151 -21.829 73.169 1.00126.12
C
ANISOU10755 CA LYS B 580   15410 18913 13598   -717 -1058
-1460   C
ATOM 10756 C   LYS B 580   35.880 -20.981 73.119 1.00121.33
C
ANISOU10756 C   LYS B 580   15054 17997 13049   -943   -927
-1331   C
ATOM 10757 O   LYS B 580   35.738 -19.978 73.822 1.00128.98
O
ANISOU10757 O   LYS B 580   16075 18916 14014 -1220   -836
-1265   O
ATOM 10758 CB LYS B 580   38.173 -21.264 74.173 1.00128.24
C
ANISOU10758 CB LYS B 580   15494 19438 13792   -905 -1048
-1474   C
ATOM 10759 CG LYS B 580   38.993 -20.034 73.817 1.00137.28
C
ANISOU10759 CG LYS B 580   16449 20895 14816 -1195   -850
-1493   C
ATOM 10760 CD LYS B 580   39.935 -19.696 74.967 1.00146.88
C
ANISOU10760 CD LYS B 580   17494 22345 15969 -1355   -887
-1520   C
ATOM 10761 CE LYS B 580   40.824 -18.505 74.653 1.00160.49
C
ANISOU10761 CE LYS B 580   19022 24395 17561 -1686   -708
-1540   C
ATOM 10762 NZ LYS B 580   41.742 -18.205 75.789 1.00171.09
N
ANISOU10762 NZ LYS B 580   20186 25986 18835 -1850   -752
-1583   N
ATOM 10763 N   HIS B 581   34.948 -21.396 72.269 1.00117.94
N
ANISOU10763 N   HIS B 581   14776 17366 12671   -806   -929
-1315   N

```
ATOM  10764  CA  HIS B 581      33.575 -20.920  72.327  1.00110.45
C
ANISOU10764  CA  HIS B 581    14074  16104  11789   -933   -866
-1207       C
ATOM  10765  C   HIS B 581      32.710 -22.002  72.965  1.00100.25
C
ANISOU10765  C   HIS B 581    12949  14557  10585   -797  -1048
-1171       C
ATOM  10766  O   HIS B 581      33.043 -23.190  72.921  1.00 76.14
O
ANISOU10766  O   HIS B 581     9867  11507   7556   -561  -1223
-1228       O
ATOM  10767  CB  HIS B 581      33.038 -20.563  70.938  1.00109.93
C
ANISOU10767  CB  HIS B 581    14060  16007  11700   -908   -740
-1197       C
ATOM  10768  CG  HIS B 581      33.830 -19.507  70.232  1.00106.15
C
ANISOU10768  CG  HIS B 581    13441  15777  11116  -1082   -570
-1194       C
ATOM  10769  ND1 HIS B 581      33.676 -18.163  70.494  1.00119.53
N
ANISOU10769  ND1 HIS B 581    15215  17399  12801  -1376   -440
-1103       N
ATOM  10770  CD2 HIS B 581      34.776 -19.596  69.268  1.00116.75
C
ANISOU10770  CD2 HIS B 581    14569  17447  12343  -1019   -517
-1270       C
ATOM  10771  CE1 HIS B 581      34.496 -17.470  69.724  1.00119.68
C
ANISOU10771  CE1 HIS B 581    15097  17666  12708  -1519   -320
-1094       C
ATOM  10772  NE2 HIS B 581      35.175 -18.315  68.970  1.00134.92
N
ANISOU10772  NE2 HIS B 581    16829  19876  14560  -1314   -353
-1193       N
ATOM  10773  N   ILE B 582      31.599 -21.586  73.562  1.00118.08
N
ANISOU10773  N   ILE B 582    15384  16601  12881   -951  -1018
-1081       N
ATOM  10774  CA  ILE B 582      30.605 -22.517  74.081  1.00135.44
C
ANISOU10774  CA  ILE B 582    17747  18579  15135   -888  -1169
-1022       C
ATOM  10775  C   ILE B 582      29.540 -22.683  73.010  1.00152.41
C
ANISOU10775  C   ILE B 582    20021  20576  17312   -807  -1128
-1016       C
ATOM  10776  O   ILE B 582      28.827 -21.728  72.679  1.00162.79
O
ANISOU10776  O   ILE B 582    21397  21841  18615   -925   -978
-987       O
ATOM  10777  CB  ILE B 582      29.999 -22.017  75.400  1.00150.50
C
```

ANISOU10777 CB ILE B 582   19731 20421 17029 -1106 -1163
-946   C
ATOM 10778 CG1 ILE B 582   31.088 -21.877 76.464 1.00157.71
C
ANISOU10778 CG1 ILE B 582   20510 21512 17900 -1188 -1213
-958   C
ATOM 10779 CG2 ILE B 582   28.927 -22.980 75.879 1.00155.28
C
ANISOU10779 CG2 ILE B 582   20494 20847 17659 -1088 -1312
-866   C
ATOM 10780 CD1 ILE B 582   30.610 -21.228 77.745 1.00160.39
C
ANISOU10780 CD1 ILE B 582   20893 21847 18202 -1416 -1184
-916   C
ATOM 10781 N   VAL B 583   29.424 -23.891 72.471 1.00148.31
N
ANISOU10781 N   VAL B 583   19547 19974 16830   -598 -1276
-1053   N
ATOM 10782 CA VAL B 583   28.725 -24.113 71.213 1.00125.52
C
ANISOU10782 CA VAL B 583   16726 17013 13953   -485 -1237
-1089   C
ATOM 10783 C   VAL B 583   27.440 -24.878 71.488 1.00 95.19
C
ANISOU10783 C   VAL B 583   13075 12936 10157   -502 -1360
-1024   C
ATOM 10784 O   VAL B 583   27.475 -26.053 71.879 1.00 65.66
O
ANISOU10784 O   VAL B 583   9411   9074   6462   -413 -1571
-1016   O
ATOM 10785 CB VAL B 583   29.602 -24.869 70.207 1.00163.26
C
ANISOU10785 CB VAL B 583   21394 21913 18724   -225 -1306
-1226   C
ATOM 10786 CG1 VAL B 583   28.929 -24.915 68.839 1.00186.86
C
ANISOU10786 CG1 VAL B 583   24425 24879 21693   -131 -1235
-1275   C
ATOM 10787 CG2 VAL B 583   30.984 -24.239 70.126 1.00192.86
C
ANISOU10787 CG2 VAL B 583   24915 25962 22401   -232 -1209
-1291   C
ATOM 10788 N   GLY B 584   26.303 -24.220 71.275 1.00132.32
N
ANISOU10788 N   GLY B 584   17856 17579 14839   -622 -1241
-974   N
ATOM 10789 CA GLY B 584   25.056 -24.942 71.125 1.00111.01
C
ANISOU10789 CA GLY B 584   15301 14721 12158   -624 -1332
-940   C
ATOM 10790 C   GLY B 584   24.978 -25.493 69.710 1.00 99.60
C
ANISOU10790 C   GLY B 584   13864 13256 10722   -430 -1350
-1033   C

```
ATOM  10791  O   GLY B 584      25.306 -24.813  68.739  1.00 77.67
O
ANISOU10791  O   GLY B 584    10999  10608   7906   -364  -1205
-1087       O
ATOM  10792  N   MET B 585      24.529 -26.739  69.593  1.00 76.76
N
ANISOU10792  N   MET B 585    11087  10204   7873   -354  -1540
-1049       N
ATOM  10793  CA  MET B 585      24.298 -27.305  68.272  1.00100.36
C
ANISOU10793  CA  MET B 585    14098  13168  10866   -178  -1570
-1160       C
ATOM  10794  C   MET B 585      23.054 -28.176  68.275  1.00131.36
C
ANISOU10794  C   MET B 585    18193  16903  14814   -247  -1709
-1124       C
ATOM  10795  O   MET B 585      22.917 -29.071  69.114  1.00138.61
O
ANISOU10795  O   MET B 585    19232  17653  15780   -317  -1907
-1059       O
ATOM  10796  CB  MET B 585      25.507 -28.120  67.802  1.00132.41
C
ANISOU10796  CB  MET B 585    18091  17257  14962     77  -1698
-1305       C
ATOM  10797  CG  MET B 585      25.312 -28.784  66.450  1.00 79.12
C
ANISOU10797  CG  MET B 585    11356  10498   8207    280  -1747
-1460       C
ATOM  10798  SD  MET B 585      26.830 -29.538  65.842  1.00115.68
S
ANISOU10798  SD  MET B 585    15849  15255  12849    622  -1867
-1686       S
ATOM  10799  CE  MET B 585      27.640 -28.129  65.090  1.00 97.35
C
ANISOU10799  CE  MET B 585    13259  13341  10387    602  -1566
-1714       C
ATOM  10800  N   THR B 586      22.160 -27.910  67.328  1.00102.33
N
ANISOU10800  N   THR B 586    14525  13265  11092   -243  -1614
-1157       N
ATOM  10801  CA  THR B 586      20.950 -28.697  67.175  1.00 76.34
C
ANISOU10801  CA  THR B 586    11368   9838   7801   -323  -1733
-1141       C
ATOM  10802  C   THR B 586      21.270 -30.015  66.477  1.00 87.48
C
ANISOU10802  C   THR B 586    12868  11095   9277   -139  -1942
-1273       C
ATOM  10803  O   THR B 586      22.262 -30.141  65.754  1.00158.00
O
ANISOU10803  O   THR B 586    21716  20094  18223     96  -1942
-1415       O
ATOM  10804  CB  THR B 586      19.889 -27.924  66.391  1.00 72.37
C
```

```
ANISOU10804  CB  THR B 586    10824   9460   7215   -364  -1565
-1142       C
ATOM  10805  OG1 THR B 586      20.393 -27.598  65.090  1.00 85.79
O
ANISOU10805  OG1 THR B 586    12433  11282   8882   -167  -1464
-1255       O
ATOM  10806  CG2 THR B 586      19.526 -26.640  67.119  1.00 61.96
C
ANISOU10806  CG2 THR B 586     9439   8256   5844   -513  -1392
-1038       C
ATOM  10807  N   GLY B 587      20.417 -31.007  66.704  1.00 89.54
N
ANISOU10807  N   GLY B 587    13298  11157   9568   -253  -2131
-1237       N
ATOM  10808  CA  GLY B 587      20.490 -32.235  65.937  1.00 93.96
C
ANISOU10808  CA  GLY B 587    13978  11531  10192    -93  -2343
-1382       C
ATOM  10809  C   GLY B 587      19.115 -32.838  65.764  1.00104.23
C
ANISOU10809  C   GLY B 587    15420  12714  11470   -286  -2441
-1345       C
ATOM  10810  O   GLY B 587      18.178 -32.531  66.508  1.00143.66
O
ANISOU10810  O   GLY B 587    20432  17751  16403   -563  -2395
-1184       O
ATOM  10811  N   ASP B 588      19.008 -33.717  64.768  1.00 71.16
N
ANISOU10811  N   ASP B 588    11318   8404   7317   -140  -2581
-1514       N
ATOM  10812  CA  ASP B 588      17.738 -34.374  64.479  1.00 89.64
C
ANISOU10812  CA  ASP B 588    13792  10636   9632   -330  -2691
-1506       C
ATOM  10813  C   ASP B 588      17.858 -35.843  64.098  1.00 74.90
C
ANISOU10813  C   ASP B 588    12148   8438   7874   -239  -3009
-1641       C
ATOM  10814  O   ASP B 588      16.919 -36.601  64.361  1.00 76.21
O
ANISOU10814  O   ASP B 588    12494   8418   8045   -492  -3181
-1564       O
ATOM  10815  CB  ASP B 588      16.993 -33.610  63.362  1.00 93.58
C
ANISOU10815  CB  ASP B 588    14139  11406  10011   -298  -2471
-1588       C
ATOM  10816  CG  ASP B 588      17.503 -33.940  61.954  1.00132.94
C
ANISOU10816  CG  ASP B 588    19081  16432  14999      6  -2487
-1839       C
ATOM  10817  OD1 ASP B 588      17.292 -35.076  61.475  1.00163.44
O
ANISOU10817  OD1 ASP B 588    23096  20088  18917     50  -2709
-1979       O
```

```
ATOM  10818  OD2 ASP B 588      18.108 -33.049  61.315  1.00151.72
O
ANISOU10818  OD2 ASP B 588    21273  19061  17313    188  -2281
-1901       O
ATOM  10819  N   GLY B 589      18.966 -36.283  63.504  1.00 78.19
N
ANISOU10819  N   GLY B 589    12560   8778   8369    105  -3105
-1849       N
ATOM  10820  CA  GLY B 589      19.071 -37.603  62.930  1.00 93.30
C
ANISOU10820  CA  GLY B 589    14676  10388  10386    259  -3403
-2044       C
ATOM  10821  C   GLY B 589      19.998 -38.526  63.706  1.00 81.69
C
ANISOU10821  C   GLY B 589    13392   8579   9066    390  -3703
-2039       C
ATOM  10822  O   GLY B 589      20.603 -38.166  64.712  1.00 80.96
O
ANISOU10822  O   GLY B 589    13260   8510   8991    357  -3678
-1877       O
ATOM  10823  N   VAL B 590      20.098 -39.754  63.191  1.00 90.72
N
ANISOU10823  N   VAL B 590    14752   9395  10321    556  -4010
-2236       N
ATOM  10824  CA  VAL B 590      20.866 -40.796  63.860  1.00 87.90
C
ANISOU10824  CA  VAL B 590    14634   8638  10125    703  -4366
-2242       C
ATOM  10825  C   VAL B 590      22.361 -40.541  63.741  1.00 88.22
C
ANISOU10825  C   VAL B 590    14480   8844  10194   1136  -4328
-2421       C
ATOM  10826  O   VAL B 590      23.142 -41.070  64.539  1.00 96.72
O
ANISOU10826  O   VAL B 590    15678   9693  11378   1263  -4560
-2372       O
ATOM  10827  CB  VAL B 590      20.500 -42.187  63.307  1.00 91.67
C
ANISOU10827  CB  VAL B 590    15429   8676  10725    765  -4734
-2423       C
ATOM  10828  CG1 VAL B 590      19.019 -42.467  63.517  1.00 99.23
C
ANISOU10828  CG1 VAL B 590    16573   9494  11637    276  -4783
-2222       C
ATOM  10829  CG2 VAL B 590      20.867 -42.294  61.832  1.00143.76
C
ANISOU10829  CG2 VAL B 590    21888  15432  17304   1154  -4686
-2828       C
ATOM  10830  N   ASN B 591      22.782 -39.739  62.764  1.00113.59
N
ANISOU10830  N   ASN B 591    17387  12473  13299   1355  -4048
-2620       N
ATOM  10831  CA  ASN B 591      24.189 -39.421  62.568  1.00130.11
C
```

```
ANISOU10831  CA  ASN B 591    19242  14816  15377   1733  -3980
-2800       C
ATOM  10832  C   ASN B 591      24.573 -38.088  63.196  1.00113.12
C
ANISOU10832  C   ASN B 591    16834  13027  13119   1587  -3660
-2590       C
ATOM  10833  O   ASN B 591      25.746 -37.706  63.146  1.00116.82
O
ANISOU10833  O   ASN B 591    17080  13751  13555   1835  -3579
-2701       O
ATOM  10834  CB  ASN B 591      24.517 -39.398  61.071  1.00156.00
C
ANISOU10834  CB  ASN B 591    22329  18371  18574   2053  -3882
-3159       C
ATOM  10835  CG  ASN B 591      24.323 -40.750  60.408  1.00172.41
C
ANISOU10835  CG  ASN B 591    24648  20097  20764   2269  -4222
-3440       C
ATOM  10836  OD1 ASN B 591      24.582 -41.794  61.006  1.00190.78
O
ANISOU10836  OD1 ASN B 591    27245  21985  23257   2371  -4581
-3458       O
ATOM  10837  ND2 ASN B 591      23.856 -40.735  59.164  1.00148.65
N
ANISOU10837  ND2 ASN B 591    21555  17265  17661   2335  -4124
-3660       N
ATOM  10838  N   ASP B 592      23.613 -37.378  63.788  1.00 95.74
N
ANISOU10838  N   ASP B 592    14652  10867  10858   1190  -3487
-2306       N
ATOM  10839  CA  ASP B 592      23.879 -36.179  64.571  1.00 92.30
C
ANISOU10839  CA  ASP B 592    14033  10694  10345   1020  -3232
-2097       C
ATOM  10840  C   ASP B 592      24.372 -36.501  65.975  1.00121.45
C
ANISOU10840  C   ASP B 592    17835  14193  14116    945  -3410
-1914       C
ATOM  10841  O   ASP B 592      24.881 -35.608  66.660  1.00 83.10
O
ANISOU10841  O   ASP B 592    12814   9556   9205    869  -3236
-1791       O
ATOM  10842  CB  ASP B 592      22.618 -35.314  64.666  1.00113.45
C
ANISOU10842  CB  ASP B 592    16688  13495  12924    662  -3000
-1900       C
ATOM  10843  CG  ASP B 592      22.292 -34.605  63.367  1.00 74.80
C
ANISOU10843  CG  ASP B 592    11616   8890   7913    733  -2757
-2032       C
ATOM  10844  OD1 ASP B 592      21.091 -34.502  63.033  1.00 92.24
O
ANISOU10844  OD1 ASP B 592    13881  11095  10069    541  -2700
-1976       O
```

```
ATOM  10845  OD2 ASP B 592      23.231 -34.148  62.683  1.00 74.90
O
ANISOU10845  OD2 ASP B 592    11424   9164   7871    970  -2627
-2184       O
ATOM  10846  N   ALA B 593      24.214 -37.748  66.411  1.00140.99
N
ANISOU10846  N   ALA B 593    20600  16258  16713    951  -3763
-1887       N
ATOM  10847  CA  ALA B 593      24.546 -38.140  67.778  1.00132.77
C
ANISOU10847  CA  ALA B 593    19705  15007  15734    842  -3968
-1671       C
ATOM  10848  C   ALA B 593      25.968 -37.815  68.228  1.00135.03
C
ANISOU10848  C   ALA B 593    19803  15477  16027   1100  -3952
-1725       C
ATOM  10849  O   ALA B 593      26.123 -37.342  69.366  1.00133.88
O
ANISOU10849  O   ALA B 593    19621  15403  15843    902  -3902
-1499       O
ATOM  10850  CB  ALA B 593      24.261 -39.640  67.953  1.00 96.16
C
ANISOU10850  CB  ALA B 593    15440   9858  11238    861  -4398
-1663       C
ATOM  10851  N   PRO B 594      27.028 -38.024  67.436  1.00115.62
N
ANISOU10851  N   PRO B 594    17197  13140  13593   1524  -3991
-2019       N
ATOM  10852  CA  PRO B 594      28.355 -37.638  67.947  1.00113.71
C
ANISOU10852  CA  PRO B 594    16737  13139  13327   1728  -3959
-2056       C
ATOM  10853  C   PRO B 594      28.562 -36.136  68.003  1.00107.80
C
ANISOU10853  C   PRO B 594    15676  12849  12434   1553  -3556
-1980       C
ATOM  10854  O   PRO B 594      29.185 -35.638  68.950  1.00120.19
O
ANISOU10854  O   PRO B 594    17137  14559  13972   1479  -3507
-1852       O
ATOM  10855  CB  PRO B 594      29.322 -38.303  66.953  1.00114.23
C
ANISOU10855  CB  PRO B 594    16708  13260  13436   2232  -4106
-2430       C
ATOM  10856  CG  PRO B 594      28.512 -38.500  65.717  1.00115.37
C
ANISOU10856  CG  PRO B 594    16903  13366  13565   2246  -4048
-2597       C
ATOM  10857  CD  PRO B 594      27.136 -38.819  66.201  1.00110.37
C
ANISOU10857  CD  PRO B 594    16579  12373  12982   1855  -4134
-2341       C
ATOM  10858  N   ALA B 595      28.062 -35.397  67.011  1.00 98.78
N
```

```
ANISOU10858  N   ALA B 595    14397  11934  11199   1481  -3281
-2054       N
ATOM  10859  CA  ALA B 595      28.128 -33.940  67.062  1.00 99.98
C
ANISOU10859  CA  ALA B 595    14310  12454  11224   1279  -2921
-1954       C
ATOM  10860  C   ALA B 595      27.351 -33.384  68.251  1.00109.74
C
ANISOU10860  C   ALA B 595    15646  13602  12448    891  -2847
-1655       C
ATOM  10861  O   ALA B 595      27.834 -32.495  68.960  1.00 82.23
O
ANISOU10861  O   ALA B 595    12015  10320   8908    768  -2695
-1552       O
ATOM  10862  CB  ALA B 595      27.607 -33.344  65.755  1.00 95.18
C
ANISOU10862  CB  ALA B 595    13590  12051  10522   1272  -2684
-2061       C
ATOM  10863  N   LEU B 596      26.146 -33.909  68.485  1.00110.46
N
ANISOU10863  N   LEU B 596    15976  13416  12578    686  -2960
-1527       N
ATOM  10864  CA  LEU B 596      25.384 -33.584  69.689  1.00144.53
C
ANISOU10864  CA  LEU B 596    20387  17664  16865    326  -2936
-1260       C
ATOM  10865  C   LEU B 596      26.140 -33.946  70.965  1.00155.63
C
ANISOU10865  C   LEU B 596    21839  18987  18306    317  -3121
-1137       C
ATOM  10866  O   LEU B 596      26.191 -33.155  71.915  1.00129.33
O
ANISOU10866  O   LEU B 596    18418  15815  14908    109  -2990
-987        O
ATOM  10867  CB  LEU B 596      24.040 -34.311  69.643  1.00129.32
C
ANISOU10867  CB  LEU B 596    18700  15476  14959    121  -3072
-1169       C
ATOM  10868  CG  LEU B 596      22.983 -33.772  68.674  1.00 80.39
C
ANISOU10868  CG  LEU B 596    12452   9400   8691     25  -2864
-1221       C
ATOM  10869  CD1 LEU B 596      21.849 -34.778  68.512  1.00 74.06
C
ANISOU10869  CD1 LEU B 596    11890   8330   7921   -129  -3064
-1181       C
ATOM  10870  CD2 LEU B 596      22.449 -32.415  69.109  1.00 85.25
C
ANISOU10870  CD2 LEU B 596    12918  10276   9197   -213  -2570
-1092       C
ATOM  10871  N   LYS B 597      26.733 -35.139  71.008  1.00143.12
N
ANISOU10871  N   LYS B 597    20400  17154  16825    554  -3441
-1206       N
```

```
ATOM  10872  CA  LYS B 597      27.521 -35.529  72.175  1.00 87.29
C
ANISOU10872  CA  LYS B 597    13375  10008   9785    586  -3647
-1088       C
ATOM  10873  C   LYS B 597      28.699 -34.586  72.394  1.00 83.63
C
ANISOU10873  C   LYS B 597    12606   9915   9255    704  -3455
-1161       C
ATOM  10874  O   LYS B 597      28.941 -34.125  73.516  1.00 94.10
O
ANISOU10874  O   LYS B 597    13878  11349  10526    525  -3421
-996       O
ATOM  10875  CB  LYS B 597      28.003 -36.973  72.025  1.00 86.88
C
ANISOU10875  CB  LYS B 597    13540   9601   9867    889  -4049
-1185       C
ATOM  10876  CG  LYS B 597      29.008 -37.412  73.083  1.00 94.27
C
ANISOU10876  CG  LYS B 597    14504  10476  10837   1020  -4290
-1098       C
ATOM  10877  CD  LYS B 597      29.359 -38.888  72.935  1.00124.81
C
ANISOU10877  CD  LYS B 597    18644  13922  14857   1331  -4733
-1185       C
ATOM  10878  CE  LYS B 597      30.544 -39.281  73.810  1.00140.56
C
ANISOU10878  CE  LYS B 597    20619  15904  16882   1569  -4978
-1152       C
ATOM  10879  NZ  LYS B 597      30.253 -39.107  75.261  1.00127.22
N
ANISOU10879  NZ  LYS B 597    19026  14190  15122   1198  -5023
-793       N
ATOM  10880  N   LYS B 598      29.447 -34.290  71.331  1.00 93.87
N
ANISOU10880  N   LYS B 598    13690  11437  10537    985  -3330
-1412       N
ATOM  10881  CA  LYS B 598      30.657 -33.485  71.458  1.00117.11
C
ANISOU10881  CA  LYS B 598    16333  14757  13407   1093  -3171
-1498       C
ATOM  10882  C   LYS B 598      30.355 -31.995  71.589  1.00115.97
C
ANISOU10882  C   LYS B 598    16020  14891  13151    785  -2810
-1399       C
ATOM  10883  O   LYS B 598      31.152 -31.260  72.182  1.00106.09
O
ANISOU10883  O   LYS B 598    14582  13892  11837    727  -2699
-1374       O
ATOM  10884  CB  LYS B 598      31.579 -33.747  70.263  1.00110.76
C
ANISOU10884  CB  LYS B 598    15344  14142  12599   1487  -3177
-1806       C
ATOM  10885  CG  LYS B 598      33.054 -33.415  70.495  1.00110.01
C
```

ANISOU10885 CG LYS B 598   14955 14404 12439   1683 -3150
-1926   C
ATOM 10886 CD LYS B 598   33.396 -31.978 70.146 1.00 80.78
C
ANISOU10886 CD LYS B 598   10961 11134   8596   1500 -2777
-1937   C
ATOM 10887 CE LYS B 598   34.898 -31.792 70.047 1.00139.76
C
ANISOU10887 CE LYS B 598   18106 19014 15982   1728 -2758
-2119   C
ATOM 10888 NZ LYS B 598   35.566 -32.012 71.360 1.00157.30
N
ANISOU10888 NZ LYS B 598   20316 21224 18225   1735 -2930
-2019   N
ATOM 10889 N   ALA B 599   29.220 -31.534 71.067 1.00 81.56
N
ANISOU10889 N   ALA B 599   11733 10487   8769   593 -2640
-1349   N
ATOM 10890 CA ALA B 599   28.791 -30.165 71.324 1.00127.79
C
ANISOU10890 CA ALA B 599   17486 16532 14537   306 -2346
-1240   C
ATOM 10891 C   ALA B 599   28.601 -29.916 72.817 1.00118.73
C
ANISOU10891 C   ALA B 599   16393 15348 13369   50 -2380
-1041   C
ATOM 10892 O   ALA B 599   28.214 -30.804 73.580 1.00 72.12
O
ANISOU10892 O   ALA B 599   10675   9220   7506   -11 -2609
-920   O
ATOM 10893 CB ALA B 599   27.493 -29.856 70.578 1.00110.24
C
ANISOU10893 CB ALA B 599   15355 14237 12296   175 -2211
-1216   C
ATOM 10894 N   ASP B 600   28.891 -28.677 73.223 1.00124.10
N
ANISOU10894 N   ASP B 600   16915 16263 13976   -114 -2155
-1010   N
ATOM 10895 CA ASP B 600   28.722 -28.268 74.615 1.00120.48
C
ANISOU10895 CA ASP B 600   16474 15831 13473   -363 -2152
-856   C
ATOM 10896 C   ASP B 600   27.273 -28.404 75.065 1.00 77.28
C
ANISOU10896 C   ASP B 600   11180 10200   7984   -596 -2169
-714   C
ATOM 10897 O   ASP B 600   27.001 -28.874 76.176 1.00 84.08
O
ANISOU10897 O   ASP B 600   12137 10989   8819   -747 -2318
-570   O
ATOM 10898 CB ASP B 600   29.203 -26.828 74.799 1.00128.68
C
ANISOU10898 CB ASP B 600   17323 17128 14440   -498 -1897
-885   C

```
ATOM  10899  CG  ASP B 600      30.661 -26.646  74.423  1.00120.49
C
ANISOU10899  CG  ASP B 600    16077  16316  13388   -324  -1871
-1016       C
ATOM  10900  OD1 ASP B 600      30.947 -25.844  73.507  1.00 69.66
O
ANISOU10900  OD1 ASP B 600     9514  10037   6915   -313  -1675
-1105       O
ATOM  10901  OD2 ASP B 600      31.521 -27.305  75.044  1.00134.93
O
ANISOU10901  OD2 ASP B 600    17858  18183  15225   -206  -2053
-1023       O
ATOM  10902  N   ILE B 601      26.331 -28.003  74.218  1.00 70.29
N
ANISOU10902  N   ILE B 601    10325   9291   7091   -637  -2024
-749       N
ATOM  10903  CA  ILE B 601      24.916 -28.298  74.409  1.00 76.39
C
ANISOU10903  CA  ILE B 601    11245   9940   7838   -820  -2057
-648       C
ATOM  10904  C   ILE B 601      24.416 -28.957  73.134  1.00 76.10
C
ANISOU10904  C   ILE B 601    11297   9762   7854   -671  -2108
-733       C
ATOM  10905  O   ILE B 601      24.335 -28.309  72.083  1.00 69.27
O
ANISOU10905  O   ILE B 601    10349   8991   6981   -577  -1932
-838       O
ATOM  10906  CB  ILE B 601      24.071 -27.053  74.739  1.00106.23
C
ANISOU10906  CB  ILE B 601    14960  13871  11534  -1030  -1828
-620       C
ATOM  10907  CG1 ILE B 601      24.724 -26.144  75.793  1.00 99.14
C
ANISOU10907  CG1 ILE B 601    13941  13142  10584  -1144  -1734
-601       C
ATOM  10908  CG2 ILE B 601      22.680 -27.471  75.205  1.00 64.77
C
ANISOU10908  CG2 ILE B 601     9822   8565   6223  -1239  -1891
-512       C
ATOM  10909  CD1 ILE B 601      25.841 -25.229  75.291  1.00 87.48
C
ANISOU10909  CD1 ILE B 601    12316  11796   9128  -1031  -1581
-712       C
ATOM  10910  N   GLY B 602      24.082 -30.241  73.224  1.00 99.21
N
ANISOU10910  N   GLY B 602    14404  12462  10830   -661  -2360
-681       N
ATOM  10911  CA  GLY B 602      23.358 -30.915  72.168  1.00113.95
C
ANISOU10911  CA  GLY B 602    16384  14178  12735   -586  -2427
-752       C
ATOM  10912  C   GLY B 602      21.864 -30.730  72.321  1.00104.54
C
```

```
ANISOU10912  C   GLY B 602    15254  12999  11467   -855  -2360
-654       C
ATOM  10913  O   GLY B 602      21.277 -31.116  73.335  1.00131.59
O
ANISOU10913  O   GLY B 602    18774  16377  14845  -1102  -2471
-493       O
ATOM  10914  N   ILE B 603      21.242 -30.136  71.309  1.00 66.42
N
ANISOU10914  N   ILE B 603    10358   8271   6608   -813  -2181
-747       N
ATOM  10915  CA  ILE B 603      19.831 -29.773  71.336  1.00 65.56
C
ANISOU10915  CA  ILE B 603    10253   8246   6409  -1025  -2084
-690       C
ATOM  10916  C   ILE B 603      19.120 -30.627  70.296  1.00 89.30
C
ANISOU10916  C   ILE B 603    13369  11122   9439   -977  -2190
-759       C
ATOM  10917  O   ILE B 603      19.340 -30.466  69.090  1.00 86.57
O
ANISOU10917  O   ILE B 603    12972  10803   9117   -764  -2114
-903       O
ATOM  10918  CB  ILE B 603      19.628 -28.278  71.057  1.00 63.68
C
ANISOU10918  CB  ILE B 603     9851   8236   6108  -1008  -1801
-738       C
ATOM  10919  CG1 ILE B 603      20.482 -27.435  72.007  1.00 63.17
C
ANISOU10919  CG1 ILE B 603     9689   8278   6034  -1042  -1709
-702       C
ATOM  10920  CG2 ILE B 603      18.157 -27.902  71.176  1.00 63.13
C
ANISOU10920  CG2 ILE B 603     9767   8284   5937  -1191  -1719
-698       C
ATOM  10921  CD1 ILE B 603      20.497 -25.965  71.663  1.00 61.79
C
ANISOU10921  CD1 ILE B 603     9391   8263   5826  -1004  -1463
-760       C
ATOM  10922  N   ALA B 604      18.270 -31.540  70.754  1.00106.72
N
ANISOU10922  N   ALA B 604    15722  13204  11623  -1198  -2370
-655       N
ATOM  10923  CA  ALA B 604      17.433 -32.308  69.843  1.00119.04
C
ANISOU10923  CA  ALA B 604    17387  14655  13189  -1215  -2470
-718       C
ATOM  10924  C   ALA B 604      16.176 -31.515  69.506  1.00125.89
C
ANISOU10924  C   ALA B 604    18132  15768  13930  -1344  -2272
-726       C
ATOM  10925  O   ALA B 604      15.420 -31.124  70.402  1.00115.12
O
ANISOU10925  O   ALA B 604    16713  14567  12459  -1590  -2213
-611       O
```

```
ATOM  10926  CB  ALA B 604      17.067 -33.657  70.459  1.00 82.73
C
ANISOU10926  CB  ALA B 604    13019   9800   8617  -1432  -2772
-591       C
ATOM  10927  N   VAL B 605      15.971 -31.263  68.209  1.00 93.49
N
ANISOU10927  N   VAL B 605    13972  11723   9826  -1160  -2175
-872       N
ATOM  10928  CA  VAL B 605      14.749 -30.619  67.747  1.00 70.17
C
ANISOU10928  CA  VAL B 605    10914   8993   6756  -1241  -2022
-892       C
ATOM  10929  C   VAL B 605      13.544 -31.470  68.161  1.00108.53
C
ANISOU10929  C   VAL B 605    15858  13840  11538  -1550  -2171
-808       C
ATOM  10930  O   VAL B 605      13.661 -32.667  68.452  1.00105.15
O
ANISOU10930  O   VAL B 605    15613  13170  11168  -1673  -2411
-754       O
ATOM  10931  CB  VAL B 605      14.785 -30.392  66.227  1.00121.08
C
ANISOU10931  CB  VAL B 605    17308  15488  13207   -994  -1936
-1050       C
ATOM  10932  CG1 VAL B 605      14.631 -31.711  65.478  1.00166.63
C
ANISOU10932  CG1 VAL B 605    23223  21063  19027   -969  -2148
-1145       C
ATOM  10933  CG2 VAL B 605      13.739 -29.365  65.793  1.00130.65
C
ANISOU10933  CG2 VAL B 605    18379  16964  14297  -1009  -1742
-1061       C
ATOM  10934  N   ALA B 606      12.376 -30.826  68.219  1.00 82.79
N
ANISOU10934  N   ALA B 606    12465  10852   8139  -1685  -2037
-795       N
ATOM  10935  CA  ALA B 606      11.196 -31.459  68.803  1.00 99.42
C
ANISOU10935  CA  ALA B 606    14596  13054  10127  -2034  -2145
-700       C
ATOM  10936  C   ALA B 606      10.870 -32.791  68.133  1.00101.86
C
ANISOU10936  C   ALA B 606    15082  13142  10476  -2131  -2369
-728       C
ATOM  10937  O   ALA B 606      10.486 -33.754  68.807  1.00120.46
O
ANISOU10937  O   ALA B 606    17576  15387  12805  -2442  -2568
-601       O
ATOM  10938  CB  ALA B 606      10.001 -30.508  68.719  1.00127.59
C
ANISOU10938  CB  ALA B 606    17951  16998  13529  -2085  -1957
-740       C
ATOM  10939  N   ASP B 607      11.013 -32.869  66.811  1.00 96.43
N
```

```
ANISOU10939  N   ASP B 607    14403  12390   9845  -1884  -2352
-891       N
ATOM  10940  CA  ASP B 607      10.741 -34.092  66.065  1.00114.78
C
ANISOU10940  CA  ASP B 607    16898  14497  12216  -1939  -2568
-967       C
ATOM  10941  C   ASP B 607      12.001 -34.903  65.765  1.00120.80
C
ANISOU10941  C   ASP B 607    17852  14884  13162  -1715  -2751
-1044       C
ATOM  10942  O   ASP B 607      11.977 -35.759  64.875  1.00141.43
O
ANISOU10942  O   ASP B 607    20592  17312  15835  -1637  -2907
-1183       O
ATOM  10943  CB  ASP B 607      10.009 -33.756  64.765  1.00161.13
C
ANISOU10943  CB  ASP B 607    22646  20569  18006  -1814  -2454
-1125       C
ATOM  10944  CG  ASP B 607       8.581 -33.302  65.000  1.00188.95
C
ANISOU10944  CG  ASP B 607    26004  24447  21340  -2059  -2352
-1072       C
ATOM  10945  OD1 ASP B 607       7.994 -33.686  66.033  1.00195.99
O
ANISOU10945  OD1 ASP B 607    26921  25391  22155  -2406  -2437
-929       O
ATOM  10946  OD2 ASP B 607       8.045 -32.561  64.148  1.00178.43
O
ANISOU10946  OD2 ASP B 607    24507  23368  19922  -1903  -2193
-1174       O
ATOM  10947  N   ALA B 608      13.098 -34.642  66.474  1.00112.48
N
ANISOU10947  N   ALA B 608    16809  13738  12192  -1594  -2741
-981       N
ATOM  10948  CA  ALA B 608      14.332 -35.400  66.293  1.00103.49
C
ANISOU10948  CA  ALA B 608    15826  12281  11216  -1356  -2927
-1064       C
ATOM  10949  C   ALA B 608      14.126 -36.901  66.504  1.00107.59
C
ANISOU10949  C   ALA B 608    16637  12427  11817  -1530  -3277
-1026       C
ATOM  10950  O   ALA B 608      13.202 -37.349  67.189  1.00 78.02
O
ANISOU10950  O   ALA B 608    12988   8659   7998  -1912  -3386
-856       O
ATOM  10951  CB  ALA B 608      15.417 -34.896  67.244  1.00 92.00
C
ANISOU10951  CB  ALA B 608    14321  10825   9809  -1265  -2875
-969       C
ATOM  10952  N   THR B 609      15.013 -37.678  65.882  1.00111.40
N
ANISOU10952  N   THR B 609    17261  12623  12444  -1242  -3464
-1196       N
```

```
ATOM  10953  CA  THR B 609      15.086 -39.115  66.111  1.00110.40
C
ANISOU10953  CA  THR B 609    17458  12052  12437  -1332  -3844
-1176       C
ATOM  10954  C   THR B 609      15.351 -39.419  67.584  1.00103.98
C
ANISOU10954  C   THR B 609    16784  11077  11646  -1560  -4000
-903       C
ATOM  10955  O   THR B 609      16.002 -38.649  68.296  1.00122.88
O
ANISOU10955  O   THR B 609    19030  13639  14018  -1487  -3850
-812       O
ATOM  10956  CB  THR B 609      16.171 -39.747  65.234  1.00121.28
C
ANISOU10956  CB  THR B 609    18926  13187  13970   -891  -4005
-1448       C
ATOM  10957  OG1 THR B 609      17.420 -39.081  65.451  1.00116.40
O
ANISOU10957  OG1 THR B 609    18141  12698  13386   -582  -3874
-1487       O
ATOM  10958  CG2 THR B 609      15.797 -39.640  63.764  1.00128.74
C
ANISOU10958  CG2 THR B 609    19763  14285  14870   -715  -3896
-1715       C
ATOM  10959  N   ASP B 610      14.822 -40.556  68.039  1.00135.79
N
ANISOU10959  N   ASP B 610    21107  14779  15708  -1865  -4313
-763       N
ATOM  10960  CA  ASP B 610      15.026 -40.994  69.418  1.00139.18
C
ANISOU10960  CA  ASP B 610    21710  15030  16143  -2118  -4510
-475       C
ATOM  10961  C   ASP B 610      16.504 -41.176  69.754  1.00123.98
C
ANISOU10961  C   ASP B 610    19850  12887  14368  -1745  -4643
-514       C
ATOM  10962  O   ASP B 610      16.944 -40.832  70.857  1.00127.87
O
ANISOU10962  O   ASP B 610    20298  13465  14822  -1827  -4618
-316       O
ATOM  10963  CB  ASP B 610      14.280 -42.309  69.656  1.00142.68
C
ANISOU10963  CB  ASP B 610    22507  15094  16610  -2495  -4873
-328       C
ATOM  10964  CG  ASP B 610      12.776 -42.157  69.564  1.00158.51
C
ANISOU10964  CG  ASP B 610    24426  17374  18428  -2948  -4755
-242       C
ATOM  10965  OD1 ASP B 610      12.075 -43.192  69.568  1.00153.47
O
ANISOU10965  OD1 ASP B 610    24055  16464  17792  -3287  -5031
-151       O
ATOM  10966  OD2 ASP B 610      12.295 -41.007  69.482  1.00172.03
O
```

```
ANISOU10966  OD2 ASP B 610    25804  19571  19988  -2965  -4400
-272       O
ATOM  10967  N   ALA B 611      17.284 -41.721  68.818  1.00110.98
N
ANISOU10967  N   ALA B 611    18293  10994  12880  -1326  -4789
-786       N
ATOM  10968  CA  ALA B 611      18.735 -41.797  68.986  1.00 89.88
C
ANISOU10968  CA  ALA B 611    15614   8206  10333   -905  -4884
-884       C
ATOM  10969  C   ALA B 611      19.388 -40.429  69.171  1.00 86.29
C
ANISOU10969  C   ALA B 611    14793   8203   9791   -740  -4515
-905       C
ATOM  10970  O   ALA B 611      20.268 -40.269  70.025  1.00 86.35
O
ANISOU10970  O   ALA B 611    14769   8219   9822   -650  -4554
-802       O
ATOM  10971  CB  ALA B 611      19.355 -42.519  67.789  1.00133.70
C
ANISOU10971  CB  ALA B 611    21258  13512  16031   -461  -5063
-1235       C
ATOM  10972  N   ALA B 612      18.986 -39.434  68.380  1.00 83.41
N
ANISOU10972  N   ALA B 612    14160   8208   9325   -704  -4173
-1032       N
ATOM  10973  CA  ALA B 612      19.443 -38.065  68.614  1.00 80.21
C
ANISOU10973  CA  ALA B 612    13439   8208   8827   -630  -3828
-1014       C
ATOM  10974  C   ALA B 612      18.990 -37.515  69.964  1.00 89.79
C
ANISOU10974  C   ALA B 612    14611   9573   9932   -995  -3737
-724       C
ATOM  10975  O   ALA B 612      19.774 -36.870  70.670  1.00 77.95
O
ANISOU10975  O   ALA B 612    12977   8224   8416   -927  -3633
-661       O
ATOM  10976  CB  ALA B 612      18.963 -37.155  67.485  1.00 97.82
C
ANISOU10976  CB  ALA B 612    15441  10760  10967   -553  -3518
-1176       C
ATOM  10977  N   ARG B 613      17.730 -37.749  70.336  1.00126.68
N
ANISOU10977  N   ARG B 613    19376  14246  14513  -1393  -3771
-558       N
ATOM  10978  CA  ARG B 613      17.238 -37.315  71.643  1.00112.15
C
ANISOU10978  CA  ARG B 613    17485  12588  12540  -1756  -3702
-297       C
ATOM  10979  C   ARG B 613      18.023 -37.928  72.800  1.00132.11
C
ANISOU10979  C   ARG B 613    20176  14899  15119  -1802  -3951
-110       C
```

```
ATOM  10980  O   ARG B 613      18.343 -37.234  73.773  1.00146.77
O
ANISOU10980  O   ARG B 613    21896  16974  16895  -1885  -3828
14       O
ATOM  10981  CB  ARG B 613      15.747 -37.626  71.769  1.00 99.58
C
ANISOU10981  CB  ARG B 613    15957  11058  10819  -2182  -3730
-170       C
ATOM  10982  CG  ARG B 613      14.884 -36.733  70.890  1.00117.26
C
ANISOU10982  CG  ARG B 613    17968  13629  12959  -2165  -3435
-317       C
ATOM  10983  CD  ARG B 613      13.418 -36.780  71.276  1.00119.75
C
ANISOU10983  CD  ARG B 613    18254  14148  13096  -2606  -3408
-179       C
ATOM  10984  NE  ARG B 613      12.756 -38.005  70.845  1.00128.11
N
ANISOU10984  NE  ARG B 613    19552  14942  14182  -2810  -3672
-161       N
ATOM  10985  CZ  ARG B 613      11.438 -38.173  70.848  1.00143.00
C
ANISOU10985  CZ  ARG B 613    21409  17011  15915  -3179  -3663
-93       C
ATOM  10986  NH1 ARG B 613      10.646 -37.201  71.281  1.00131.66
N
ANISOU10986  NH1 ARG B 613    19703  16037  14285  -3348  -3408
-47       N
ATOM  10987  NH2 ARG B 613      10.913 -39.318  70.437  1.00165.11
N
ANISOU10987  NH2 ARG B 613    24446  19539  18749  -3381  -3921
-82       N
ATOM  10988  N   GLY B 614      18.340 -39.219  72.723  1.00129.06
N
ANISOU10988  N   GLY B 614    20093  14079  14866  -1741  -4316
-92       N
ATOM  10989  CA  GLY B 614      19.202 -39.819  73.732  1.00124.15
C
ANISOU10989  CA  GLY B 614    19639  13227  14307  -1714  -4582
76       C
ATOM  10990  C   GLY B 614      20.599 -39.225  73.778  1.00137.81
C
ANISOU10990  C   GLY B 614    21181  15081  16101  -1306  -4481
-61       C
ATOM  10991  O   GLY B 614      21.153 -39.001  74.858  1.00158.86
O
ANISOU10991  O   GLY B 614    23807  17831  18722  -1365  -4505
103       O
ATOM  10992  N   ALA B 615      21.191 -38.970  72.610  1.00100.13
N
ANISOU10992  N   ALA B 615    16277  10355  11414   -906  -4368
-362       N
ATOM  10993  CA  ALA B 615      22.479 -38.281  72.548  1.00 89.84
C
```

```
ANISOU10993  CA   ALA B 615    14739   9258  10137   -552  -4225
-509       C
ATOM  10994  C   ALA B 615      22.409 -36.851  73.081  1.00 86.48
C
ANISOU10994  C   ALA B 615    14024   9267   9569   -713  -3860
-434       C
ATOM  10995  O   ALA B 615      23.306 -36.412  73.812  1.00 79.29
O
ANISOU10995  O   ALA B 615    12992   8494   8641   -637  -3822
-389       O
ATOM  10996  CB  ALA B 615      22.999 -38.284  71.111  1.00 82.74
C
ANISOU10996  CB  ALA B 615    13738   8384   9317   -144  -4161
-845       C
ATOM  10997  N   SER B 616      21.363 -36.109  72.724  1.00114.59
N
ANISOU10997  N   SER B 616    17470  13043  13026   -920  -3604
-433       N
ATOM  10998  CA  SER B 616      21.269 -34.702  73.099  1.00111.85
C
ANISOU10998  CA  SER B 616    16863  13076  12560  -1027  -3268
-406       C
ATOM  10999  C   SER B 616      21.253 -34.492  74.611  1.00124.74
C
ANISOU10999  C   SER B 616    18491  14806  14100  -1300  -3294
-175       C
ATOM  11000  O   SER B 616      20.654 -35.264  75.365  1.00129.42
O
ANISOU11000  O   SER B 616    19267  15256  14651  -1576  -3500
26       O
ATOM  11001  CB  SER B 616      20.015 -34.082  72.481  1.00117.16
C
ANISOU11001  CB  SER B 616    17451  13925  13139  -1190  -3046
-441       C
ATOM  11002  OG  SER B 616      18.840 -34.635  73.047  1.00112.91
O
ANISOU11002  OG  SER B 616    17052  13328  12523  -1550  -3163
-266       O
ATOM  11003  N   ASP B 617      21.931 -33.421  75.039  1.00117.97
N
ANISOU11003  N   ASP B 617    17418  14208  13197  -1238  -3086
-205       N
ATOM  11004  CA  ASP B 617      21.807 -32.920  76.406  1.00 83.88
C
ANISOU11004  CA  ASP B 617    13034  10080   8757  -1505  -3033
-31       C
ATOM  11005  C   ASP B 617      20.396 -32.426  76.702  1.00 71.83
C
ANISOU11005  C   ASP B 617    11461   8747   7086  -1832  -2881
48       C
ATOM  11006  O   ASP B 617      19.870 -32.650  77.799  1.00 72.88
O
ANISOU11006  O   ASP B 617    11637   8957   7097  -2139  -2961
237       O
```

```
ATOM  11007  CB  ASP B 617      22.818 -31.797  76.649  1.00 84.38
C
ANISOU11007  CB  ASP B 617    12871  10382   8809  -1361  -2829
-126       C
ATOM  11008  CG  ASP B 617      24.197 -32.126  76.117  1.00107.73
C
ANISOU11008  CG  ASP B 617    15802  13244  11887  -1005  -2925
-261       C
ATOM  11009  OD1 ASP B 617      24.346 -32.222  74.882  1.00134.19
O
ANISOU11009  OD1 ASP B 617    19135  16536  15314   -772  -2885
-435       O
ATOM  11010  OD2 ASP B 617      25.135 -32.273  76.929  1.00105.83
O
ANISOU11010  OD2 ASP B 617    15535  13028  11648   -954  -3039
-205       O
ATOM  11011  N   ILE B 618      19.768 -31.747  75.743  1.00 70.08
N
ANISOU11011  N   ILE B 618    11135   8638   6854  -1767  -2666
-97       N
ATOM  11012  CA  ILE B 618      18.459 -31.134  75.933  1.00 81.64
C
ANISOU11012  CA  ILE B 618    12509  10336   8175  -2012  -2501
-70       C
ATOM  11013  C   ILE B 618      17.551 -31.542  74.783  1.00 92.50
C
ANISOU11013  C   ILE B 618    13947  11627   9570  -1992  -2510
-146       C
ATOM  11014  O   ILE B 618      17.962 -31.527  73.618  1.00110.72
O
ANISOU11014  O   ILE B 618    16255  13830  11983  -1720  -2474
-300       O
ATOM  11015  CB  ILE B 618      18.556 -29.593  76.001  1.00 67.02
C
ANISOU11015  CB  ILE B 618    10434   8759   6271  -1939  -2203
-184       C
ATOM  11016  CG1 ILE B 618      19.586 -29.148  77.037  1.00 67.04
C
ANISOU11016  CG1 ILE B 618    10365   8844   6264  -1938  -2191
-144       C
ATOM  11017  CG2 ILE B 618      17.195 -28.976  76.300  1.00 66.54
C
ANISOU11017  CG2 ILE B 618    10270   8958   6055  -2154  -2060
-179       C
ATOM  11018  CD1 ILE B 618      19.640 -27.652  77.214  1.00 65.38
C
ANISOU11018  CD1 ILE B 618     9969   8870   6004  -1908  -1927
-255       C
ATOM  11019  N   VAL B 619      16.315 -31.909  75.114  1.00 84.87
N
ANISOU11019  N   VAL B 619    13018  10748   8482  -2296  -2557
-43       N
ATOM  11020  CA  VAL B 619      15.305 -32.277  74.130  1.00 81.78
C
```

```
ANISOU11020  CA  VAL B 619    12664  10332   8075  -2335  -2565
-109       C
ATOM  11021  C   VAL B 619      14.200 -31.231  74.166  1.00 71.49
C
ANISOU11021  C   VAL B 619    11152   9397   6616  -2443  -2323
-162       C
ATOM  11022  O   VAL B 619      13.503 -31.086  75.177  1.00104.31
O
ANISOU11022  O   VAL B 619    15233  13791  10611  -2729  -2304
-55       O
ATOM  11023  CB  VAL B 619      14.738 -33.678  74.398  1.00 98.30
C
ANISOU11023  CB  VAL B 619    14982  12219  10148  -2619  -2853
49       C
ATOM  11024  CG1 VAL B 619      13.567 -33.957  73.471  1.00104.52
C
ANISOU11024  CG1 VAL B 619    15775  13050  10887  -2715  -2841
-25       C
ATOM  11025  CG2 VAL B 619      15.822 -34.729  74.225  1.00106.85
C
ANISOU11025  CG2 VAL B 619    16299  12887  11411  -2441  -3125
65       C
ATOM  11026  N   LEU B 620      14.043 -30.504  73.065  1.00 67.56
N
ANISOU11026  N   LEU B 620    10552   8966   6151  -2205  -2149
-331       N
ATOM  11027  CA  LEU B 620      12.945 -29.559  72.932  1.00 73.59
C
ANISOU11027  CA  LEU B 620    11133  10048   6780  -2250  -1951
-402       C
ATOM  11028  C   LEU B 620      11.611 -30.287  72.806  1.00 85.52
C
ANISOU11028  C   LEU B 620    12659  11669   8166  -2514  -2042
-357       C
ATOM  11029  O   LEU B 620      11.531 -31.406  72.291  1.00 69.57
O
ANISOU11029  O   LEU B 620    10806   9424   6204  -2582  -2229
-326       O
ATOM  11030  CB  LEU B 620      13.159 -28.658  71.717  1.00124.08
C
ANISOU11030  CB  LEU B 620    17449  16452  13244  -1924  -1775
-566       C
ATOM  11031  CG  LEU B 620      14.487 -27.906  71.648  1.00113.41
C
ANISOU11031  CG  LEU B 620    16075  15010  12006  -1683  -1677
-615       C
ATOM  11032  CD1 LEU B 620      14.548 -27.056  70.391  1.00128.83
C
ANISOU11032  CD1 LEU B 620    17962  16995  13993  -1422  -1517
-741       C
ATOM  11033  CD2 LEU B 620      14.670 -27.045  72.884  1.00103.54
C
ANISOU11033  CD2 LEU B 620    14726  13920  10694  -1778  -1580
-577       C
```

```
ATOM  11034  N   THR B 621      10.552 -29.640  73.290  1.00111.42
N
ANISOU11034  N   THR B 621    15755  15312  11266  -2667  -1916
-371       N
ATOM  11035  CA  THR B 621       9.194 -30.087  73.020  1.00114.26
C
ANISOU11035  CA  THR B 621    16058  15878  11477  -2891  -1953
-370       C
ATOM  11036  C   THR B 621       8.373 -29.023  72.307  1.00102.93
C
ANISOU11036  C   THR B 621    14417  14727   9965  -2698  -1751
-540       C
ATOM  11037  O   THR B 621       7.221 -29.285  71.944  1.00 80.56
O
ANISOU11037  O   THR B 621    11498  12111   6999  -2838  -1763
-572       O
ATOM  11038  CB  THR B 621       8.489 -30.498  74.320  1.00147.43
C
ANISOU11038  CB  THR B 621    20203  20338  15476  -3313  -2027
-218       C
ATOM  11039  OG1 THR B 621       7.329 -31.280  74.009  1.00118.63
O
ANISOU11039  OG1 THR B 621    16552  16825  11699  -3597  -2125
-180       O
ATOM  11040  CG2 THR B 621       8.071 -29.270  75.117  1.00155.83
C
ANISOU11040  CG2 THR B 621    21009  21817  16384  -3292  -1824
-298       C
ATOM  11041  N   GLU B 622       8.940 -27.836  72.092  1.00121.68
N
ANISOU11041  N   GLU B 622    16718  17100  12415  -2386  -1583
-645       N
ATOM  11042  CA  GLU B 622       8.398 -26.746  71.306  1.00130.78
C
ANISOU11042  CA  GLU B 622    17730  18423  13537  -2131  -1416
-796       C
ATOM  11043  C   GLU B 622       9.377 -26.406  70.189  1.00132.02
C
ANISOU11043  C   GLU B 622    17989  18300  13873  -1809  -1370
-850       C
ATOM  11044  O   GLU B 622      10.587 -26.353  70.430  1.00162.80
O
ANISOU11044  O   GLU B 622    21979  21979  17897  -1729  -1378
-812       O
ATOM  11045  CB  GLU B 622       8.153 -25.496  72.170  1.00145.58
C
ANISOU11045  CB  GLU B 622    19434  20559  15321  -2070  -1267
-868       C
ATOM  11046  CG  GLU B 622       7.127 -25.660  73.286  1.00165.46
C
ANISOU11046  CG  GLU B 622    21788  23462  17615  -2367  -1282
-851       C
ATOM  11047  CD  GLU B 622       5.700 -25.809  72.782  1.00189.72
C
```

```
ANISOU11047  CD  GLU B 622    24706  26860  20518  -2438  -1277
-924       C
ATOM  11048  OE1 GLU B 622       4.794 -25.983  73.623  1.00203.88
O
ANISOU11048  OE1 GLU B 622    26332  29035  22097  -2701  -1286
-919       O
ATOM  11049  OE2 GLU B 622       5.474 -25.731  71.555  1.00186.03
O
ANISOU11049  OE2 GLU B 622    24263  26310  20111  -2238  -1260
-991       O
ATOM  11050  N   PRO B 623       8.903 -26.175  68.972  1.00106.09
N
ANISOU11050  N   PRO B 623    14674  15054  10583  -1631  -1322
-936       N
ATOM  11051  CA  PRO B 623       9.814 -26.010  67.840  1.00 63.26
C
ANISOU11051  CA  PRO B 623     9341   9403   5293  -1368  -1293
-974       C
ATOM  11052  C   PRO B 623      10.217 -24.553  67.628  1.00102.23
C
ANISOU11052  C   PRO B 623    14220  14362  10262  -1124  -1126
-1018       C
ATOM  11053  O   PRO B 623       9.639 -23.626  68.198  1.00114.21
O
ANISOU11053  O   PRO B 623    15632  16058  11705  -1107  -1039
-1052       O
ATOM  11054  CB  PRO B 623       8.975 -26.524  66.666  1.00 89.78
C
ANISOU11054  CB  PRO B 623    12687  12833   8593  -1337  -1338
-1037       C
ATOM  11055  CG  PRO B 623       7.595 -26.088  67.030  1.00 88.12
C
ANISOU11055  CG  PRO B 623    12309  12961   8212  -1436  -1288
-1070       C
ATOM  11056  CD  PRO B 623       7.501 -26.284  68.530  1.00 94.36
C
ANISOU11056  CD  PRO B 623    13068  13841   8945  -1699  -1323
-995       C
ATOM  11057  N   GLY B 624      11.223 -24.374  66.785  1.00 61.44
N
ANISOU11057  N   GLY B 624     9128   9016   5201   -938  -1094
-1024       N
ATOM  11058  CA  GLY B 624      11.669 -23.066  66.322  1.00 88.28
C
ANISOU11058  CA  GLY B 624    12511  12400   8632   -729   -956
-1039       C
ATOM  11059  C   GLY B 624      12.810 -22.493  67.141  1.00 68.19
C
ANISOU11059  C   GLY B 624     9997   9737   6174   -742   -907
-1001       C
ATOM  11060  O   GLY B 624      13.000 -22.806  68.316  1.00 60.24
O
ANISOU11060  O   GLY B 624     8988   8727   5175   -903   -953
-972       O
```

```
ATOM  11061  N   LEU B 625      13.590 -21.629  66.483  1.00 62.11
N
ANISOU11061  N   LEU B 625     9256   8887   5457   -588   -816
-993       N
ATOM  11062  CA  LEU B 625      14.790 -21.049  67.085  1.00 66.91
C
ANISOU11062  CA  LEU B 625     9889   9389   6143   -608   -766
-961       C
ATOM  11063  C   LEU B 625      14.476 -20.256  68.348  1.00127.86
C
ANISOU11063  C   LEU B 625    17577  17156  13847   -691   -728
-979       C
ATOM  11064  O   LEU B 625      15.263 -20.254  69.301  1.00192.38
O
ANISOU11064  O   LEU B 625    25750  25282  22062   -795   -737
-963       O
ATOM  11065  CB  LEU B 625      15.506 -20.159  66.069  1.00 59.48
C
ANISOU11065  CB  LEU B 625     8978   8393   5228   -469   -672
-936       C
ATOM  11066  CG  LEU B 625      16.917 -19.724  66.467  1.00 80.85
C
ANISOU11066  CG  LEU B 625    11699  11015   8006   -515   -628
-903       C
ATOM  11067  CD1 LEU B 625      17.843 -20.925  66.524  1.00103.38
C
ANISOU11067  CD1 LEU B 625    14534  13847  10899   -546   -711
-914       C
ATOM  11068  CD2 LEU B 625      17.451 -18.670  65.509  1.00 86.05
C
ANISOU11068  CD2 LEU B 625    12391  11648   8659   -434   -531
-853       C
ATOM  11069  N   SER B 626      13.338 -19.560  68.360  1.00 59.98
N
ANISOU11069  N   SER B 626     8938   8672   5179   -628   -692
-1032       N
ATOM  11070  CA  SER B 626      12.952 -18.736  69.504  1.00 60.43
C
ANISOU11070  CA  SER B 626     8948   8805   5206   -665   -658
-1096       C
ATOM  11071  C   SER B 626      12.906 -19.527  70.809  1.00 93.08
C
ANISOU11071  C   SER B 626    13022  13048   9295   -882   -720
-1093       C
ATOM  11072  O   SER B 626      13.288 -19.011  71.866  1.00104.32
O
ANISOU11072  O   SER B 626    14426  14487  10724   -953   -696
-1125       O
ATOM  11073  CB  SER B 626      11.597 -18.083  69.231  1.00 61.29
C
ANISOU11073  CB  SER B 626     8995   9066   5226   -527   -638
-1182       C
ATOM  11074  OG  SER B 626      11.211 -17.237  70.298  1.00110.61
O
```

```
ANISOU11074  OG  SER B 626    15186  15404  11438   -520   -612
-1287       O
ATOM  11075  N   VAL B 627      12.435 -20.774  70.759  1.00 60.65
N
ANISOU11075  N   VAL B 627     8894   9019   5133  -1007   -811
-1050       N
ATOM  11076  CA  VAL B 627      12.375 -21.608  71.960  1.00109.64
C
ANISOU11076  CA  VAL B 627    15064  15317  11276  -1249   -895
-1004       C
ATOM  11077  C   VAL B 627      13.754 -21.832  72.576  1.00 83.18
C
ANISOU11077  C   VAL B 627    11781  11804   8020  -1316   -928
-937       C
ATOM  11078  O   VAL B 627      13.917 -21.745  73.799  1.00 61.10
O
ANISOU11078  O   VAL B 627     8941   9097   5176  -1459   -940
-927       O
ATOM  11079  CB  VAL B 627      11.685 -22.944  71.633  1.00 78.05
C
ANISOU11079  CB  VAL B 627    11074  11367   7214  -1388  -1013
-947       C
ATOM  11080  CG1 VAL B 627      11.748 -23.885  72.829  1.00114.73
C
ANISOU11080  CG1 VAL B 627    15728  16064  11801  -1670  -1129
-851       C
ATOM  11081  CG2 VAL B 627      10.241 -22.692  71.218  1.00 71.17
C
ANISOU11081  CG2 VAL B 627    10091  10738   6214  -1355   -979
-1026       C
ATOM  11082  N   ILE B 628      14.767 -22.116  71.754  1.00 76.74
N
ANISOU11082  N   ILE B 628    11050  10789   7319  -1210   -945
-900       N
ATOM  11083  CA  ILE B 628      16.132 -22.258  72.268  1.00 96.30
C
ANISOU11083  CA  ILE B 628    13562  13149   9878  -1240   -974
-856       C
ATOM  11084  C   ILE B 628      16.635 -20.966  72.907  1.00 83.67
C
ANISOU11084  C   ILE B 628    11924  11574   8294  -1228   -862
-905       C
ATOM  11085  O   ILE B 628      17.151 -20.973  74.031  1.00105.95
O
ANISOU11085  O   ILE B 628    14720  14435  11103  -1351   -889
-889       O
ATOM  11086  CB  ILE B 628      17.082 -22.737  71.154  1.00 61.92
C
ANISOU11086  CB  ILE B 628     9267   8640   5621  -1097  -1003
-846       C
ATOM  11087  CG1 ILE B 628      16.624 -24.085  70.595  1.00 67.42
C
ANISOU11087  CG1 ILE B 628    10024   9280   6314  -1108  -1142
-825       C
```

```
ATOM  11088  CG2 ILE B 628      18.515 -22.826  71.665  1.00 59.75
C
ANISOU11088  CG2 ILE B 628     8993   8295   5415  -1104  -1031
-821       C
ATOM  11089  CD1 ILE B 628      17.378 -24.522  69.358  1.00 76.55
C
ANISOU11089  CD1 ILE B 628    11217  10327   7541   -928  -1166
-866       C
ATOM  11090  N   ILE B 629      16.496 -19.842  72.207  1.00 59.60
N
ANISOU11090  N   ILE B 629     8884   8495   5268  -1086   -751
-963       N
ATOM  11091  CA  ILE B 629      16.945 -18.562  72.753  1.00 64.64
C
ANISOU11091  CA  ILE B 629     9519   9107   5932  -1081   -664
-1020       C
ATOM  11092  C   ILE B 629      16.147 -18.144  73.987  1.00 98.54
C
ANISOU11092  C   ILE B 629    13743  13561  10137  -1167   -659
-1105       C
ATOM  11093  O   ILE B 629      16.718 -17.635  74.959  1.00110.30
O
ANISOU11093  O   ILE B 629    15212  15067  11631  -1250   -642
-1146       O
ATOM  11094  CB  ILE B 629      16.914 -17.484  71.656  1.00 59.95
C
ANISOU11094  CB  ILE B 629     8989   8405   5383   -919   -578
-1038       C
ATOM  11095  CG1 ILE B 629      17.750 -17.946  70.464  1.00 87.55
C
ANISOU11095  CG1 ILE B 629    12521  11815   8928   -857   -577
-960       C
ATOM  11096  CG2 ILE B 629      17.414 -16.147  72.181  1.00 60.59
C
ANISOU11096  CG2 ILE B 629     9110   8403   5508   -932   -514
-1093       C
ATOM  11097  CD1 ILE B 629      19.165 -18.339  70.827  1.00 82.53
C
ANISOU11097  CD1 ILE B 629    11863  11152   8342   -943   -597
-924       C
ATOM  11098  N   SER B 630      14.826 -18.330  73.975  1.00 72.33
N
ANISOU11098  N   SER B 630    10364  10399   6719  -1150   -673
-1150       N
ATOM  11099  CA  SER B 630      14.050 -18.099  75.194  1.00 81.59
C
ANISOU11099  CA  SER B 630    11427  11806   7768  -1248   -674
-1244       C
ATOM  11100  C   SER B 630      14.480 -19.005  76.345  1.00 76.74
C
ANISOU11100  C   SER B 630    10769  11297   7091  -1487   -750
-1165       C
ATOM  11101  O   SER B 630      14.582 -18.551  77.491  1.00110.30
O
```

```
ANISOU11101  O   SER B 630    14951  15682  11276  -1579   -733
-1235       O
ATOM  11102  CB  SER B 630      12.560 -18.284  74.908  1.00101.84
C
ANISOU11102  CB  SER B 630    13900  14584  10210  -1206   -681
-1302       C
ATOM  11103  OG  SER B 630      12.075 -17.265  74.052  1.00147.76
O
ANISOU11103  OG  SER B 630    19744  20335  16063   -962   -623
-1395       O
ATOM  11104  N   ALA B 631      14.733 -20.285  76.067  1.00 68.35
N
ANISOU11104  N   ALA B 631     9756  10170   6044  -1584   -849
-1023       N
ATOM  11105  CA  ALA B 631      15.316 -21.175  77.071  1.00 69.63
C
ANISOU11105  CA  ALA B 631     9921  10365   6170  -1791   -954
-912       C
ATOM  11106  C   ALA B 631      16.697 -20.724  77.544  1.00 61.83
C
ANISOU11106  C   ALA B 631     8958   9267   5269  -1778   -936
-912       C
ATOM  11107  O   ALA B 631      16.991 -20.768  78.744  1.00 62.52
O
ANISOU11107  O   ALA B 631     8993   9481   5282  -1931   -970
-896       O
ATOM  11108  CB  ALA B 631      15.388 -22.600  76.522  1.00 62.13
C
ANISOU11108  CB  ALA B 631     9064   9288   5253  -1851  -1094
-771       C
ATOM  11109  N   VAL B 632      17.559 -20.291  76.622  1.00 74.72
N
ANISOU11109  N   VAL B 632    10654  10699   7038  -1616   -884
-928       N
ATOM  11110  CA  VAL B 632      18.854 -19.719  76.999  1.00115.87
C
ANISOU11110  CA  VAL B 632    15864  15843  12319  -1617   -852
-945       C
ATOM  11111  C   VAL B 632      18.698 -18.494  77.897  1.00119.13
C
ANISOU11111  C   VAL B 632    16221  16361  12683  -1661   -765
-1074       C
ATOM  11112  O   VAL B 632      19.371 -18.371  78.928  1.00119.58
O
ANISOU11112  O   VAL B 632    16232  16493  12709  -1779   -784
-1085       O
ATOM  11113  CB  VAL B 632      19.675 -19.385  75.740  1.00 68.12
C
ANISOU11113  CB  VAL B 632     9873   9618   6393  -1460   -796
-942       C
ATOM  11114  CG1 VAL B 632      20.853 -18.486  76.088  1.00 60.70
C
ANISOU11114  CG1 VAL B 632     8915   8646   5502  -1490   -733
-983       C
```

```
ATOM  11115  CG2 VAL B 632      20.164 -20.655  75.071  1.00 60.23
C
ANISOU11115  CG2 VAL B 632     8908   8536   5439  -1409   -901
-852       C
ATOM  11116  N   LEU B 633      17.814 -17.572  77.523  1.00 61.78
N
ANISOU11116  N   LEU B 633     8961   9104   5409  -1552   -684
-1187       N
ATOM  11117  CA  LEU B 633      17.571 -16.389  78.347  1.00 62.74
C
ANISOU11117  CA  LEU B 633     9044   9305   5490  -1555   -623
-1351       C
ATOM  11118  C   LEU B 633      17.038 -16.729  79.740  1.00 68.14
C
ANISOU11118  C   LEU B 633     9602  10279   6011  -1716   -662
-1399       C
ATOM  11119  O   LEU B 633      17.495 -16.166  80.742  1.00 79.98
O
ANISOU11119  O   LEU B 633    11056  11860   7474  -1799   -648
-1491       O
ATOM  11120  CB  LEU B 633      16.612 -15.451  77.611  1.00 63.12
C
ANISOU11120  CB  LEU B 633     9130   9295   5557  -1361   -564
-1465       C
ATOM  11121  CG  LEU B 633      16.262 -14.120  78.281  1.00122.15
C
ANISOU11121  CG  LEU B 633    16598  16797  13016  -1292   -522
-1675       C
ATOM  11122  CD1 LEU B 633      17.516 -13.304  78.537  1.00 75.80
C
ANISOU11122  CD1 LEU B 633    10812  10738   7250  -1349   -498
-1701       C
ATOM  11123  CD2 LEU B 633      15.281 -13.335  77.424  1.00140.18
C
ANISOU11123  CD2 LEU B 633    18937  18999  15325  -1055   -499
-1767       C
ATOM  11124  N   THR B 634      16.064 -17.637  79.826  1.00123.24
N
ANISOU11124  N   THR B 634    16516  17440  12871  -1787   -714
-1337       N
ATOM  11125  CA  THR B 634      15.614 -18.142  81.125  1.00 75.00
C
ANISOU11125  CA  THR B 634    10281  11644   6572  -1999   -763
-1332       C
ATOM  11126  C   THR B 634      16.710 -18.868  81.905  1.00 65.03
C
ANISOU11126  C   THR B 634     9035  10370   5303  -2179   -851
-1186       C
ATOM  11127  O   THR B 634      16.838 -18.680  83.121  1.00110.42
O
ANISOU11127  O   THR B 634    14690  16337  10928  -2322   -859
-1234       O
ATOM  11128  CB  THR B 634      14.407 -19.061  80.931  1.00 88.91
C
```

```
ANISOU11128  CB  THR B 634    11985  13592   8205  -2085   -814
-1256       C
ATOM  11129  OG1 THR B 634      13.259 -18.271  80.602  1.00140.49
O
ANISOU11129  OG1 THR B 634    18434  20268  14677  -1931   -734
-1438       O
ATOM  11130  CG2 THR B 634      14.110 -19.839  82.203  1.00 66.74
C
ANISOU11130  CG2 THR B 634     9071  11097   5191  -2373   -891
-1170       C
ATOM  11131  N   SER B 635      17.526 -19.677  81.226  1.00 64.12
N
ANISOU11131  N   SER B 635     9028  10021   5312  -2157   -925
-1025       N
ATOM  11132  CA  SER B 635      18.626 -20.375  81.895  1.00 90.73
C
ANISOU11132  CA  SER B 635    12417  13366   8689  -2277  -1031
-893       C
ATOM  11133  C   SER B 635      19.615 -19.435  82.585  1.00 65.95
C
ANISOU11133  C   SER B 635     9232  10255   5572  -2284   -973
-998       C
ATOM  11134  O   SER B 635      20.096 -19.737  83.683  1.00 79.51
O
ANISOU11134  O   SER B 635    10893  12122   7196  -2439  -1043
-946       O
ATOM  11135  CB  SER B 635      19.361 -21.259  80.887  1.00102.48
C
ANISOU11135  CB  SER B 635    14023  14591  10325  -2174  -1117
-762       C
ATOM  11136  OG  SER B 635      20.276 -22.124  81.535  1.00105.98
O
ANISOU11136  OG  SER B 635    14487  15016  10762  -2266  -1261
-626       O
ATOM  11137  N   ARG B 636      19.942 -18.302  81.962  1.00 64.17
N
ANISOU11137  N   ARG B 636     9035   9885   5462  -2138   -858
-1136       N
ATOM  11138  CA  ARG B 636      20.761 -17.289  82.632  1.00 87.94
C
ANISOU11138  CA  ARG B 636    12007  12919   8486  -2176   -803
-1261       C
ATOM  11139  C   ARG B 636      20.127 -16.741  83.912  1.00120.74
C
ANISOU11139  C   ARG B 636    16054  17345  12477  -2286   -780
-1410       C
ATOM  11140  O   ARG B 636      20.839 -16.452  84.882  1.00156.70
O
ANISOU11140  O   ARG B 636    20547  22015  16978  -2401   -793
-1461       O
ATOM  11141  CB  ARG B 636      21.100 -16.156  81.661  1.00112.77
C
ANISOU11141  CB  ARG B 636    15236  15832  11779  -2030   -700
-1362       C
```

```
ATOM  11142  CG  ARG B 636      22.043 -16.598  80.547  1.00135.63
C
ANISOU11142  CG  ARG B 636    18196  18535  14803  -1955   -712
-1234       C
ATOM  11143  CD  ARG B 636      22.422 -15.462  79.613  1.00139.02
C
ANISOU11143  CD  ARG B 636    18707  18766  15348  -1866   -614
-1300       C
ATOM  11144  NE  ARG B 636      23.240 -15.939  78.501  1.00135.74
N
ANISOU11144  NE  ARG B 636    18319  18242  15015  -1800   -618
-1184       N
ATOM  11145  CZ  ARG B 636      23.378 -15.309  77.339  1.00104.54
C
ANISOU11145  CZ  ARG B 636    14444  14136  11140  -1715   -547
-1175       C
ATOM  11146  NH1 ARG B 636      22.863 -14.100  77.171  1.00107.08
N
ANISOU11146  NH1 ARG B 636    14854  14342  11491  -1688   -482
-1264       N
ATOM  11147  NH2 ARG B 636      24.136 -15.838  76.387  1.00107.12
N
ANISOU11147  NH2 ARG B 636    14758  14435  11506  -1662   -551
-1085       N
ATOM  11148  N   ALA B 637      18.803 -16.576  83.939  1.00131.26
N
ANISOU11148  N   ALA B 637    17340  18819  13713  -2248   -746
-1501       N
ATOM  11149  CA  ALA B 637      18.129 -16.163  85.173  1.00133.73
C
ANISOU11149  CA  ALA B 637    17512  19467  13833  -2347   -729
-1662       C
ATOM  11150  C   ALA B 637      18.308 -17.159  86.319  1.00130.16
C
ANISOU11150  C   ALA B 637    16963  19293  13198  -2596   -825
-1515       C
ATOM  11151  O   ALA B 637      18.532 -16.755  87.466  1.00131.44
O
ANISOU11151  O   ALA B 637    17021  19690  13230  -2711   -821
-1624       O
ATOM  11152  CB  ALA B 637      16.642 -15.935  84.899  1.00135.97
C
ANISOU11152  CB  ALA B 637    17733  19904  14027  -2245   -684
-1783       C
ATOM  11153  N   ILE B 638      18.218 -18.457  86.034  1.00123.99
N
ANISOU11153  N   ILE B 638    16228  18482  12399  -2687   -927
-1267       N
ATOM  11154  CA  ILE B 638      18.574 -19.479  87.021  1.00104.92
C
ANISOU11154  CA  ILE B 638    13777  16246   9841  -2924  -1058
-1070       C
ATOM  11155  C   ILE B 638      20.045 -19.397  87.417  1.00 97.56
C
```

```
ANISOU11155  C   ILE B 638    12871  15210   8988  -2935  -1104
-1031       C
ATOM  11156  O   ILE B 638      20.399 -19.565  88.590  1.00117.56
O
ANISOU11156  O   ILE B 638    15319  17981  11367  -3108  -1165
-993       O
ATOM  11157  CB  ILE B 638      18.213 -20.877  86.490  1.00 96.42
C
ANISOU11157  CB  ILE B 638    12800  15066   8770  -2999  -1188
-811       C
ATOM  11158  CG1 ILE B 638      16.730 -20.934  86.127  1.00100.60
C
ANISOU11158  CG1 ILE B 638    13275  15752   9197  -3018  -1138
-861       C
ATOM  11159  CG2 ILE B 638      18.548 -21.945  87.516  1.00 90.81
C
ANISOU11159  CG2 ILE B 638    12091  14502   7911  -3250  -1356
-579       C
ATOM  11160  CD1 ILE B 638      15.814 -20.550  87.266  1.00100.62
C
ANISOU11160  CD1 ILE B 638    13080  16225   8925  -3188  -1089
-982       C
ATOM  11161  N   PHE B 639      20.925 -19.169  86.449  1.00 98.74
N
ANISOU11161  N   PHE B 639    13119  15043   9356  -2761  -1080
-1034       N
ATOM  11162  CA  PHE B 639      22.349 -19.053  86.747  1.00 82.14
C
ANISOU11162  CA  PHE B 639    11011  12879   7321  -2767  -1117
-1015       C
ATOM  11163  C   PHE B 639      22.676 -17.846  87.627  1.00 91.34
C
ANISOU11163  C   PHE B 639    12075  14208   8420  -2829  -1029
-1234       C
ATOM  11164  O   PHE B 639      23.535 -17.935  88.512  1.00151.47
O
ANISOU11164  O   PHE B 639    19622  21965  15965  -2942  -1089
-1208       O
ATOM  11165  CB  PHE B 639      23.121 -18.976  85.435  1.00 84.86
C
ANISOU11165  CB  PHE B 639    11451  12909   7885  -2578  -1088
-998       C
ATOM  11166  CG  PHE B 639      24.485 -19.572  85.499  1.00 93.42
C
ANISOU11166  CG  PHE B 639    12530  13941   9025  -2567  -1193
-885       C
ATOM  11167  CD1 PHE B 639      24.651 -20.888  85.893  1.00 99.02
C
ANISOU11167  CD1 PHE B 639    13265  14680   9678  -2619  -1375
-681       C
ATOM  11168  CD2 PHE B 639      25.598 -18.835  85.141  1.00104.83
C
ANISOU11168  CD2 PHE B 639    13946  15311  10574  -2506  -1125
-979       C
```

```
ATOM  11169  CE1 PHE B 639      25.898 -21.455  85.942  1.00111.53
C
ANISOU11169  CE1 PHE B 639    14839  16223  11314  -2561  -1493
-594       C
ATOM  11170  CE2 PHE B 639      26.849 -19.398  85.186  1.00121.02
C
ANISOU11170  CE2 PHE B 639    15955  17369  12658  -2477  -1223
-895       C
ATOM  11171  CZ  PHE B 639      26.996 -20.714  85.584  1.00127.75
C
ANISOU11171  CZ  PHE B 639    16826  18253  13459  -2478  -1411
-712       C
ATOM  11172  N   GLN B 640      22.019 -16.709  87.391  1.00 69.29
N
ANISOU11172  N   GLN B 640     9282  11391   5655  -2743   -901
-1458       N
ATOM  11173  CA  GLN B 640      22.271 -15.504  88.184  1.00 70.53
C
ANISOU11173  CA  GLN B 640     9371  11661   5766  -2785   -833
-1704       C
ATOM  11174  C   GLN B 640      22.058 -15.708  89.685  1.00 93.69
C
ANISOU11174  C   GLN B 640    12151  14995   8451  -2977   -881
-1743       C
ATOM  11175  O   GLN B 640      22.901 -15.310  90.497  1.00139.38
O
ANISOU11175  O   GLN B 640    17874  20897  14189  -3077   -894
-1823       O
ATOM  11176  CB  GLN B 640      21.376 -14.371  87.679  1.00 85.12
C
ANISOU11176  CB  GLN B 640    11267  13400   7676  -2627   -726
-1938       C
ATOM  11177  CG  GLN B 640      21.362 -13.135  88.563  1.00 79.36
C
ANISOU11177  CG  GLN B 640    10480  12787   6884  -2649   -678
-2238       C
ATOM  11178  CD  GLN B 640      22.537 -12.219  88.305  1.00 72.67
C
ANISOU11178  CD  GLN B 640     9729  11681   6203  -2652   -652
-2321       C
ATOM  11179  OE1 GLN B 640      23.621 -12.409  88.856  1.00 94.74
O
ANISOU11179  OE1 GLN B 640    12471  14554   8973  -2799   -690
-2264       O
ATOM  11180  NE2 GLN B 640      22.328 -11.214  87.461  1.00 92.82
N
ANISOU11180  NE2 GLN B 640    12423  13930   8914  -2502   -596
-2448       N
ATOM  11181  N   ARG B 641      20.936 -16.315  90.077  1.00 72.83
N
ANISOU11181  N   ARG B 641     9437  12606   5630  -3051   -907
-1688       N
ATOM  11182  CA  ARG B 641      20.674 -16.544  91.500  1.00 74.78
C
```

```
ANISOU11182  CA  ARG B 641     9522  13293   5600  -3263   -951
-1706       C
ATOM  11183  C   ARG B 641      21.700 -17.470  92.149  1.00 75.23
C
ANISOU11183  C   ARG B 641     9570  13423   5591  -3432  -1088
-1458       C
ATOM  11184  O   ARG B 641      22.066 -17.276  93.314  1.00129.02
O
ANISOU11184  O   ARG B 641    16262  20533  12228  -3582  -1115
-1520       O
ATOM  11185  CB  ARG B 641      19.254 -17.077  91.698  1.00 88.81
C
ANISOU11185  CB  ARG B 641    11212  15356   7177  -3343   -953
-1666       C
ATOM  11186  CG  ARG B 641      18.896 -18.276  90.861  1.00128.38
C
ANISOU11186  CG  ARG B 641    16335  20194  12251  -3359  -1034
-1375       C
ATOM  11187  CD  ARG B 641      17.397 -18.503  90.870  1.00133.27
C
ANISOU11187  CD  ARG B 641    16857  21087  12692  -3418  -1002
-1405       C
ATOM  11188  NE  ARG B 641      16.717 -17.476  90.083  1.00142.07
N
ANISOU11188  NE  ARG B 641    17970  22092  13920  -3163   -872
-1674       N
ATOM  11189  CZ  ARG B 641      15.425 -17.182  90.183  1.00143.12
C
ANISOU11189  CZ  ARG B 641    17963  22523  13892  -3137   -809
-1839       C
ATOM  11190  NH1 ARG B 641      14.645 -17.879  90.997  1.00176.83
N
ANISOU11190  NH1 ARG B 641    22072  27246  17869  -3387   -849
-1751       N
ATOM  11191  NH2 ARG B 641      14.901 -16.224  89.430  1.00117.42
N
ANISOU11191  NH2 ARG B 641    14728  19123  10762  -2863   -716
-2079       N
ATOM  11192  N   MET B 642      22.171 -18.483  91.420  1.00 74.06
N
ANISOU11192  N   MET B 642     9548  13017   5575  -3394  -1190
-1190       N
ATOM  11193  CA  MET B 642      23.279 -19.300  91.911  1.00101.65
C
ANISOU11193  CA  MET B 642    13054  16522   9048  -3484  -1342
-974       C
ATOM  11194  C   MET B 642      24.556 -18.488  92.118  1.00 74.77
C
ANISOU11194  C   MET B 642     9602  13078   5729  -3439  -1305
-1122       C
ATOM  11195  O   MET B 642      25.259 -18.677  93.117  1.00 81.41
O
ANISOU11195  O   MET B 642    10354  14140   6436  -3570  -1391
-1071       O
```

```
ATOM  11196  CB  MET B 642      23.526 -20.472  90.962  1.00 88.16
C
ANISOU11196  CB  MET B 642    11501  14503   7493  -3390  -1467
-713       C
ATOM  11197  CG  MET B 642      22.333 -21.415  90.845  1.00 73.93
C
ANISOU11197  CG  MET B 642     9758  12740   5591  -3492  -1539
-535       C
ATOM  11198  SD  MET B 642      22.561 -22.745  89.644  1.00 89.85
S
ANISOU11198  SD  MET B 642    11985  14344   7810  -3361  -1698
-280       S
ATOM  11199  CE  MET B 642      23.661 -23.823  90.559  1.00 98.84
C
ANISOU11199  CE  MET B 642    13164  15515   8876  -3463  -1954
-15       C
ATOM  11200  N   LYS B 643      24.882 -17.594  91.183  1.00 73.53
N
ANISOU11200  N   LYS B 643     9503  12655   5780  -3276  -1186
-1290       N
ATOM  11201  CA  LYS B 643      26.009 -16.683  91.380  1.00 87.32
C
ANISOU11201  CA  LYS B 643    11203  14384   7593  -3282  -1138
-1451       C
ATOM  11202  C   LYS B 643      25.835 -15.818  92.624  1.00107.96
C
ANISOU11202  C   LYS B 643    13689  17305  10026  -3425  -1092
-1683       C
ATOM  11203  O   LYS B 643      26.745 -15.713  93.454  1.00185.04
O
ANISOU11203  O   LYS B 643    23355  27251  19702  -3541  -1142
-1707       O
ATOM  11204  CB  LYS B 643      26.177 -15.786  90.153  1.00103.15
C
ANISOU11204  CB  LYS B 643    13311  16056   9826  -3126  -1016
-1584       C
ATOM  11205  CG  LYS B 643      26.578 -16.498  88.882  1.00151.82
C
ANISOU11205  CG  LYS B 643    19573  21949  16163  -2980  -1047
-1399       C
ATOM  11206  CD  LYS B 643      28.081 -16.666  88.819  1.00129.87
C
ANISOU11206  CD  LYS B 643    16739  19164  13441  -2987  -1103
-1341       C
ATOM  11207  CE  LYS B 643      28.800 -15.328  88.763  1.00 94.47
C
ANISOU11207  CE  LYS B 643    12231  14640   9025  -3046   -993
-1549       C
ATOM  11208  NZ  LYS B 643      28.401 -14.501  87.596  1.00173.88
N
ANISOU11208  NZ  LYS B 643    22416  24410  19240  -2947   -871
-1636       N
ATOM  11209  N   ASN B 644      24.670 -15.186  92.766  1.00 76.15
N
```

```
ANISOU11209  N   ASN B 644     9645  13360   5929  -3403  -1002
-1876       N
ATOM  11210  CA  ASN B 644      24.413 -14.319  93.915  1.00 85.64
C
ANISOU11210  CA  ASN B 644    10720  14869   6952  -3504   -958
-2151       C
ATOM  11211  C   ASN B 644      24.421 -15.076  95.241  1.00134.65
C
ANISOU11211  C   ASN B 644    16769  21522  12868  -3716  -1058
-2033       C
ATOM  11212  O   ASN B 644      24.953 -14.579  96.241  1.00 81.51
O
ANISOU11212  O   ASN B 644     9924  15040   6006  -3836  -1066
-2186       O
ATOM  11213  CB  ASN B 644      23.080 -13.592  93.724  1.00 78.48
C
ANISOU11213  CB  ASN B 644     9815  13980   6023  -3386   -859
-2391       C
ATOM  11214  CG  ASN B 644      22.997 -12.858  92.396  1.00 81.95
C
ANISOU11214  CG  ASN B 644    10429  13970   6737  -3173   -782
-2478       C
ATOM  11215  OD1 ASN B 644      21.907 -12.591  91.893  1.00 78.99
O
ANISOU11215  OD1 ASN B 644    10089  13539   6385  -3028   -729
-2574       O
ATOM  11216  ND2 ASN B 644      24.151 -12.509  91.834  1.00148.75
N
ANISOU11216  ND2 ASN B 644    18987  22142  15388  -3162   -779
-2444       N
ATOM  11217  N   TYR B 645      23.827 -16.271  95.277  1.00129.18
N
ANISOU11217  N   TYR B 645    16079  20941  12062  -3786  -1143
-1755       N
ATOM  11218  CA  TYR B 645      23.900 -17.106  96.476  1.00 81.45
C
ANISOU11218  CA  TYR B 645     9919  15292   5739  -4016  -1268
-1569       C
ATOM  11219  C   TYR B 645      25.324 -17.526  96.833  1.00 95.75
C
ANISOU11219  C   TYR B 645    11728  17078   7574  -4069  -1392
-1408       C
ATOM  11220  O   TYR B 645      25.690 -17.534  98.014  1.00147.90
O
ANISOU11220  O   TYR B 645    18199  24043  13955  -4239  -1452
-1417       O
ATOM  11221  CB  TYR B 645      23.024 -18.346  96.302  1.00 81.37
C
ANISOU11221  CB  TYR B 645     9960  15328   5631  -4102  -1359
-1265       C
ATOM  11222  CG  TYR B 645      23.023 -19.235  97.520  1.00 83.64
C
ANISOU11222  CG  TYR B 645    10152  15969   5657  -4343  -1485
-1015       C
```

```
ATOM  11223  CD1 TYR B 645      22.514 -18.790  98.730  1.00 85.93
C
ANISOU11223  CD1 TYR B 645    10236  16640   5775  -4435  -1375
-1133       C
ATOM  11224  CD2 TYR B 645      23.565 -20.513  97.467  1.00130.29
C
ANISOU11224  CD2 TYR B 645    16182  21762  11559  -4426  -1697
-641       C
ATOM  11225  CE1 TYR B 645      22.508 -19.608  99.844  1.00125.67
C
ANISOU11225  CE1 TYR B 645    15180  21947  10620  -4636  -1455
-865       C
ATOM  11226  CE2 TYR B 645      23.580 -21.331  98.580  1.00112.44
C
ANISOU11226  CE2 TYR B 645    13861  19734   9127  -4612  -1799
-369       C
ATOM  11227  CZ  TYR B 645      23.046 -20.876  99.765  1.00106.96
C
ANISOU11227  CZ  TYR B 645    12956  19434   8250  -4733  -1669
-473       C
ATOM  11228  OH  TYR B 645      23.079 -21.683 100.879  1.00127.68
O
ANISOU11228  OH  TYR B 645    15518  22305  10691  -4942  -1767
-191       O
ATOM  11229  N   THR B 646      26.137 -17.892  95.843  1.00 80.23
N
ANISOU11229  N   THR B 646     9891  14735   5859  -3918  -1437
-1269       N
ATOM  11230  CA  THR B 646      27.532 -18.224  96.130  1.00 80.83
C
ANISOU11230  CA  THR B 646     9936  14817   5960  -3930  -1554
-1156       C
ATOM  11231  C   THR B 646      28.298 -17.046  96.724  1.00 89.62
C
ANISOU11231  C   THR B 646    10924  16086   7043  -3988  -1471
-1444       C
ATOM  11232  O   THR B 646      29.084 -17.221  97.663  1.00128.54
O
ANISOU11232  O   THR B 646    15737  21281  11821  -4107  -1568
-1401       O
ATOM  11233  CB  THR B 646      28.229 -18.731  94.870  1.00 97.64
C
ANISOU11233  CB  THR B 646    12194  16548   8355  -3728  -1598
-1015       C
ATOM  11234  OG1 THR B 646      27.478 -19.820  94.320  1.00124.31
O
ANISOU11234  OG1 THR B 646    15704  19764  11764  -3679  -1686
-772       O
ATOM  11235  CG2 THR B 646      29.626 -19.220  95.216  1.00104.69
C
ANISOU11235  CG2 THR B 646    13027  17508   9244  -3717  -1745
-890       C
ATOM  11236  N   ILE B 647      28.088 -15.840  96.194  1.00 86.85
N
```

```
ANISOU11236  N   ILE B 647    10606  15562   6833  -3911  -1308 
-1735       N
ATOM  11237  CA  ILE B 647      28.638 -14.641  96.824  1.00 82.56 
C
ANISOU11237  CA  ILE B 647     9967  15150   6250  -3998  -1238 
-2042       C
ATOM  11238  C   ILE B 647      28.131 -14.484  98.255  1.00 84.81 
C
ANISOU11238  C   ILE B 647    10094  15902   6227  -4171  -1255 
-2170       C
ATOM  11239  O   ILE B 647      28.902 -14.166  99.168  1.00 86.55 
O
ANISOU11239  O   ILE B 647    10189  16349   6349  -4279  -1278 
-2255       O
ATOM  11240  CB  ILE B 647      28.317 -13.398  95.972  1.00 81.78 
C
ANISOU11240  CB  ILE B 647     9982  14729   6360  -3882  -1087 
-2315       C
ATOM  11241  CG1 ILE B 647      28.957 -13.519  94.590  1.00 89.06 
C
ANISOU11241  CG1 ILE B 647    11036  15251   7552  -3748  -1069 
-2181       C
ATOM  11242  CG2 ILE B 647      28.796 -12.131  96.667  1.00 83.68 
C
ANISOU11242  CG2 ILE B 647    10159  15072   6564  -3990  -1036 
-2653       C
ATOM  11243  CD1 ILE B 647      28.405 -12.541  93.575  1.00103.83 
C
ANISOU11243  CD1 ILE B 647    13059  16769   9621  -3621   -944 
-2353       C
ATOM  11244  N   TYR B 648      26.833 -14.702  98.472  1.00135.64 
N
ANISOU11244  N   TYR B 648    16516  22485  12536  -4173  -1221 
-2170       N
ATOM  11245  CA  TYR B 648      26.277 -14.687  99.825  1.00144.60 
C
ANISOU11245  CA  TYR B 648    17472  23999  13470  -4253  -1178 
-2196       C
ATOM  11246  C   TYR B 648      26.957 -15.697 100.747  1.00100.92 
C
ANISOU11246  C   TYR B 648    11849  18708   7787  -4392  -1308 
-1882       C
ATOM  11247  O   TYR B 648      27.361 -15.357 101.865  1.00 90.96 
O
ANISOU11247  O   TYR B 648    10437  17718   6408  -4473  -1286 
-1966       O
ATOM  11248  CB  TYR B 648      24.766 -14.940  99.753  1.00110.66 
C
ANISOU11248  CB  TYR B 648    13157  19801   9089  -4217  -1113 
-2178       C
ATOM  11249  CG  TYR B 648      24.099 -15.311 101.065  1.00111.48 
C
ANISOU11249  CG  TYR B 648    13063  20341   8952  -4341  -1087 
-2095       C
```

```
ATOM  11250  CD1 TYR B 648      23.410 -14.362 101.808  1.00115.48
C
ANISOU11250  CD1 TYR B 648    13408  21100   9370  -4301   -953
-2418       C
ATOM  11251  CD2 TYR B 648      24.102 -16.624 101.528  1.00111.92
C
ANISOU11251  CD2 TYR B 648    13103  20553   8871  -4501  -1204
-1694       C
ATOM  11252  CE1 TYR B 648      22.790 -14.697 102.998  1.00109.93
C
ANISOU11252  CE1 TYR B 648    12496  20840   8432  -4430   -917
-2350       C
ATOM  11253  CE2 TYR B 648      23.484 -16.968 102.716  1.00124.44
C
ANISOU11253  CE2 TYR B 648    14507  22549  10225  -4654  -1173
-1601       C
ATOM  11254  CZ  TYR B 648      22.827 -16.000 103.445  1.00135.45
C
ANISOU11254  CZ  TYR B 648    15708  24235  11520  -4624  -1018
-1932       C
ATOM  11255  OH  TYR B 648      22.205 -16.336 104.625  1.00147.02
O
ANISOU11255  OH  TYR B 648    16963  26155  12741  -4791   -976
-1847       O
ATOM  11256  N   ALA B 649      27.087 -16.946 100.297  1.00 87.84
N
ANISOU11256  N   ALA B 649    10295  16947   6132  -4412  -1458
-1524       N
ATOM  11257  CA  ALA B 649      27.693 -17.991 101.121  1.00 89.45
C
ANISOU11257  CA  ALA B 649    10452  17334   6200  -4524  -1621
-1198       C
ATOM  11258  C   ALA B 649      29.143 -17.678 101.485  1.00123.31
C
ANISOU11258  C   ALA B 649    14666  21672  10513  -4522  -1679
-1260       C
ATOM  11259  O   ALA B 649      29.561 -17.857 102.634  1.00118.26
O
ANISOU11259  O   ALA B 649    13895  21319   9718  -4622  -1723
-1181       O
ATOM  11260  CB  ALA B 649      27.598 -19.336 100.399  1.00 95.61
C
ANISOU11260  CB  ALA B 649    11405  17903   7019  -4511  -1805
-829       C
ATOM  11261  N   VAL B 650      29.934 -17.234 100.512  1.00 88.56
N
ANISOU11261  N   VAL B 650    10334  17016   6296  -4424  -1682
-1391       N
ATOM  11262  CA  VAL B 650      31.287 -16.759 100.793  1.00 89.44
C
ANISOU11262  CA  VAL B 650    10346  17199   6439  -4436  -1706
-1503       C
ATOM  11263  C   VAL B 650      31.289 -15.543 101.718  1.00 91.55
C
```

```
ANISOU11263  C   VAL B 650    10473  17667   6646  -4513  -1551
-1824       C
ATOM  11264  O   VAL B 650      32.076 -15.473 102.670  1.00144.26
O
ANISOU11264  O   VAL B 650    17006  24588  13216  -4589  -1586
-1825       O
ATOM  11265  CB  VAL B 650      32.017 -16.458  99.471  1.00116.49
C
ANISOU11265  CB  VAL B 650    13863  20309  10089  -4329  -1703
-1588       C
ATOM  11266  CG1 VAL B 650      33.331 -15.734  99.733  1.00 88.65
C
ANISOU11266  CG1 VAL B 650    10207  16894   6582  -4389  -1693
-1768       C
ATOM  11267  CG2 VAL B 650      32.254 -17.747  98.695  1.00143.18
C
ANISOU11267  CG2 VAL B 650    17357  23449  13595  -4165  -1844
-1242       C
ATOM  11268  N   SER B 651      30.410 -14.574 101.462  1.00 97.25
N
ANISOU11268  N   SER B 651    11234  18286   7430  -4480  -1392
-2109       N
ATOM  11269  CA  SER B 651      30.361 -13.357 102.274  1.00 96.00
C
ANISOU11269  CA  SER B 651    10968  18273   7233  -4523  -1265
-2453       C
ATOM  11270  C   SER B 651      30.065 -13.610 103.754  1.00111.77
C
ANISOU11270  C   SER B 651    12783  20694   8990  -4620  -1260
-2400       C
ATOM  11271  O   SER B 651      30.728 -13.038 104.627  1.00114.71
O
ANISOU11271  O   SER B 651    13027  21264   9295  -4692  -1238
-2555       O
ATOM  11272  CB  SER B 651      29.327 -12.393 101.689  1.00104.57
C
ANISOU11272  CB  SER B 651    12152  19152   8428  -4424  -1133
-2752       C
ATOM  11273  OG  SER B 651      29.287 -11.181 102.420  1.00128.99
O
ANISOU11273  OG  SER B 651    15167  22338  11503  -4436  -1039
-3111       O
ATOM  11274  N   ILE B 652      29.073 -14.450 104.065  1.00124.84
N
ANISOU11274  N   ILE B 652    14419  22509  10506  -4644  -1277
-2183       N
ATOM  11275  CA  ILE B 652      28.838 -14.819 105.465  1.00118.14
C
ANISOU11275  CA  ILE B 652    13389  22089   9408  -4773  -1280
-2081       C
ATOM  11276  C   ILE B 652      30.034 -15.540 106.085  1.00117.93
C
ANISOU11276  C   ILE B 652    13305  22211   9292  -4863  -1429
-1824       C
```

```
ATOM  11277  O   ILE B 652      30.399 -15.270 107.235  1.00111.99
O
ANISOU11277  O   ILE B 652    12386  21781   8384  -4958  -1409
-1895       O
ATOM  11278  CB  ILE B 652      27.538 -15.638 105.612  1.00 98.59
C
ANISOU11278  CB  ILE B 652    10905  19755   6799  -4824  -1272
-1865       C
ATOM  11279  CG1 ILE B 652      27.598 -16.946 104.825  1.00108.13
C
ANISOU11279  CG1 ILE B 652    12287  20736   8063  -4828  -1435
-1458       C
ATOM  11280  CG2 ILE B 652      26.332 -14.806 105.199  1.00 98.06
C
ANISOU11280  CG2 ILE B 652    10835  19636   6786  -4717  -1116
-2171       C
ATOM  11281  CD1 ILE B 652      26.409 -17.852 105.058  1.00107.18
C
ANISOU11281  CD1 ILE B 652    12164  20763   7799  -4937  -1448
-1200       C
ATOM  11282  N   THR B 653      30.666 -16.457 105.351  1.00 98.06
N
ANISOU11282  N   THR B 653    10915  19477   6865  -4819  -1592
-1538       N
ATOM  11283  CA  THR B 653      31.864 -17.116 105.872  1.00 99.45
C
ANISOU11283  CA  THR B 653    11035  19782   6969  -4856  -1759
-1316       C
ATOM  11284  C   THR B 653      32.957 -16.118 106.236  1.00100.50
C
ANISOU11284  C   THR B 653    11039  20013   7133  -4866  -1701
-1597       C
ATOM  11285  O   THR B 653      33.510 -16.162 107.340  1.00157.34
O
ANISOU11285  O   THR B 653    18088  27525  14171  -4955  -1735
-1566       O
ATOM  11286  CB  THR B 653      32.394 -18.127 104.859  1.00165.31
C
ANISOU11286  CB  THR B 653    19536  27839  15437  -4749  -1951
-1034       C
ATOM  11287  OG1 THR B 653      31.409 -19.144 104.648  1.00161.47
O
ANISOU11287  OG1 THR B 653    19179  27268  14904  -4771  -2031
-743       O
ATOM  11288  CG2 THR B 653      33.678 -18.767 105.375  1.00 99.46
C
ANISOU11288  CG2 THR B 653    11124  19639   7028  -4739  -2144
-835       C
ATOM  11289  N   ILE B 654      33.283 -15.211 105.316  1.00 98.81
N
ANISOU11289  N   ILE B 654    10887  19535   7122  -4796  -1615
-1866       N
ATOM  11290  CA  ILE B 654      34.341 -14.233 105.568  1.00 99.97
C
```

```
ANISOU11290  CA  ILE B 654    10930  19739   7315  -4839  -1563
-2129       C
ATOM  11291  C   ILE B 654      33.971 -13.315 106.729  1.00164.47
C
ANISOU11291  C   ILE B 654    18965  28174  15353  -4933  -1433
-2404       C
ATOM  11292  O   ILE B 654      34.785 -13.058 107.624  1.00196.86
O
ANISOU11292  O   ILE B 654    22916  32531  19349  -5018  -1448
-2468       O
ATOM  11293  CB  ILE B 654      34.650 -13.433 104.287  1.00 97.92
C
ANISOU11293  CB  ILE B 654    10791  19113   7302  -4783  -1495
-2344       C
ATOM  11294  CG1 ILE B 654      35.651 -14.175 103.387  1.00102.62
C
ANISOU11294  CG1 ILE B 654    11417  19577   7996  -4716  -1634
-2131       C
ATOM  11295  CG2 ILE B 654      35.163 -12.036 104.627  1.00 99.50
C
ANISOU11295  CG2 ILE B 654    10926  19326   7553  -4871  -1379
-2716       C
ATOM  11296  CD1 ILE B 654      35.338 -15.626 103.085  1.00113.26
C
ANISOU11296  CD1 ILE B 654    12843  20891   9298  -4620  -1801
-1753       C
ATOM  11297  N   ARG B 655      32.736 -12.815 106.736  1.00130.26
N
ANISOU11297  N   ARG B 655    14672  23804  11018  -4904  -1312
-2582       N
ATOM  11298  CA  ARG B 655      32.276 -11.943 107.816  1.00129.40
C
ANISOU11298  CA  ARG B 655    14424  23960  10782  -4959  -1197
-2874       C
ATOM  11299  C   ARG B 655      32.291 -12.633 109.180  1.00132.83
C
ANISOU11299  C   ARG B 655    14670  24858  10940  -5075  -1240
-2679       C
ATOM  11300  O   ARG B 655      32.790 -12.074 110.164  1.00110.12
O
ANISOU11300  O   ARG B 655    11640  22248   7952  -5156  -1207
-2854       O
ATOM  11301  CB  ARG B 655      30.873 -11.428 107.479  1.00122.54
C
ANISOU11301  CB  ARG B 655    13621  22981   9957  -4863  -1082
-3080       C
ATOM  11302  CG  ARG B 655      30.272 -10.432 108.467  1.00130.92
C
ANISOU11302  CG  ARG B 655    14542  24291  10909  -4868   -968
-3443       C
ATOM  11303  CD  ARG B 655      29.523 -11.082 109.625  1.00109.27
C
ANISOU11303  CD  ARG B 655    11603  22027   7889  -4951   -945
-3298       C
```

ATOM 11304 NE ARG B 655   28.999 -10.087 110.549 1.00112.27
N
ANISOU11304 NE ARG B 655   11823 22671   8164 -4937   -837
-3682   N
ATOM 11305 CZ ARG B 655   28.019 -9.229 110.282 1.00112.58
C
ANISOU11305 CZ ARG B 655   11887 22610   8277 -4792   -749
-4019   C
ATOM 11306 NH1 ARG B 655   27.811 -8.194 111.075 1.00133.05
N
ANISOU11306 NH1 ARG B 655   14355 25386 10811 -4755   -683
-4416   N
ATOM 11307 NH2 ARG B 655   27.359 -9.312 109.133 1.00109.95
N
ANISOU11307 NH2 ARG B 655   11725 21946   8104 -4662   -743
-3989   N
ATOM 11308 N   ILE B 656   31.738 -13.844 109.260 1.00129.24
N
ANISOU11308 N   ILE B 656   14234 24500 10371 -5101 -1319
-2312   N
ATOM 11309 CA ILE B 656   31.708 -14.581 110.525 1.00116.80
C
ANISOU11309 CA ILE B 656   12502 23349   8527 -5240 -1375
-2079   C
ATOM 11310 C   ILE B 656   33.108 -14.946 111.012 1.00111.30
C
ANISOU11310 C   ILE B 656   11737 22778   7772 -5289 -1510
-1927   C
ATOM 11311 O   ILE B 656   33.453 -14.712 112.177 1.00114.23
O
ANISOU11311 O   ILE B 656   11929 23513   7960 -5393 -1490
-1995   O
ATOM 11312 CB ILE B 656   30.816 -15.829 110.397 1.00109.45
C
ANISOU11312 CB ILE B 656   11647 22433   7507 -5284 -1453
-1690   C
ATOM 11313 CG1 ILE B 656   29.360 -15.422 110.161 1.00108.93
C
ANISOU11313 CG1 ILE B 656   11577 22369   7441 -5257 -1299
-1866   C
ATOM 11314 CG2 ILE B 656   30.934 -16.707 111.629 1.00112.55
C
ANISOU11314 CG2 ILE B 656   11912 23218   7632 -5455 -1548
-1384   C
ATOM 11315 CD1 ILE B 656   28.735 -14.673 111.317 1.00111.97
C
ANISOU11315 CD1 ILE B 656   11731 23177   7636 -5331 -1149
-2143   C
ATOM 11316 N   VAL B 657   33.939 -15.517 110.139 1.00109.43
N
ANISOU11316 N   VAL B 657   11625 22271   7683 -5203 -1653
-1734   N
ATOM 11317 CA VAL B 657   35.312 -15.837 110.535 1.00110.88
C

```
ANISOU11317  CA  VAL B 657    11722  22587   7820  -5215  -1791
-1615       C
ATOM  11318  C   VAL B 657      36.109 -14.585 110.889  1.00112.14
C
ANISOU11318  C   VAL B 657    11748  22850   8009  -5255  -1680
-1994       C
ATOM  11319  O   VAL B 657      36.692 -14.500 111.974  1.00147.60
O
ANISOU11319  O   VAL B 657    16071  27684  12328  -5347  -1699
-2011       O
ATOM  11320  CB  VAL B 657      36.011 -16.655 109.437  1.00108.80
C
ANISOU11320  CB  VAL B 657    11598  22022   7718  -5079  -1968
-1375       C
ATOM  11321  CG1 VAL B 657      37.471 -16.881 109.799  1.00110.52
C
ANISOU11321  CG1 VAL B 657    11695  22402   7895  -5057  -2108
-1304       C
ATOM  11322  CG2 VAL B 657      35.304 -17.989 109.256  1.00108.31
C
ANISOU11322  CG2 VAL B 657    11678  21870   7604  -5058  -2119
-972       C
ATOM  11323  N   PHE B 658      36.133 -13.587 110.003  1.00110.35
N
ANISOU11323  N   PHE B 658    11605  22328   7996  -5204  -1569
-2299       N
ATOM  11324  CA  PHE B 658      36.985 -12.424 110.259  1.00111.80
C
ANISOU11324  CA  PHE B 658    11692  22558   8227  -5268  -1490
-2637       C
ATOM  11325  C   PHE B 658      36.497 -11.580 111.431  1.00116.96
C
ANISOU11325  C   PHE B 658    12215  23497   8729  -5363  -1367
-2921       C
ATOM  11326  O   PHE B 658      37.308 -11.121 112.241  1.00152.78
O
ANISOU11326  O   PHE B 658    16601  28278  13169  -5456  -1362
-3057       O
ATOM  11327  CB  PHE B 658      37.133 -11.555 109.008  1.00109.62
C
ANISOU11327  CB  PHE B 658    11563  21866   8220  -5221  -1418
-2873       C
ATOM  11328  CG  PHE B 658      38.026 -12.148 107.954  1.00121.53
C
ANISOU11328  CG  PHE B 658    13131  23177   9868  -5155  -1528
-2673       C
ATOM  11329  CD1 PHE B 658      38.578 -13.408 108.114  1.00107.98
C
ANISOU11329  CD1 PHE B 658    11355  21620   8052  -5100  -1702
-2318       C
ATOM  11330  CD2 PHE B 658      38.349 -11.420 106.819  1.00151.37
C
ANISOU11330  CD2 PHE B 658    17019  26621  13873  -5148  -1469
-2851       C
```

```
ATOM  11331  CE1 PHE B 658      39.410 -13.942 107.148  1.00140.20
C
ANISOU11331  CE1 PHE B 658    15466  25549  12257  -5005  -1814
-2169       C
ATOM  11332  CE2 PHE B 658      39.181 -11.946 105.851  1.00151.38
C
ANISOU11332  CE2 PHE B 658    17038  26494  13986  -5090  -1560
-2687       C
ATOM  11333  CZ  PHE B 658      39.713 -13.209 106.015  1.00164.30
C
ANISOU11333  CZ  PHE B 658    18593  28313  15520  -5003  -1733
-2360       C
ATOM  11334  N   GLY B 659      35.189 -11.359 111.543  1.00125.08
N
ANISOU11334  N   GLY B 659    13278  24522   9725  -5333  -1269
-3028       N
ATOM  11335  CA  GLY B 659      34.693 -10.481 112.594  1.00150.16
C
ANISOU11335  CA  GLY B 659    16314  27972  12767  -5391  -1154
-3349       C
ATOM  11336  C   GLY B 659      34.879 -11.021 114.003  1.00161.02
C
ANISOU11336  C   GLY B 659    17472  29863  13848  -5513  -1186
-3198       C
ATOM  11337  O   GLY B 659      35.403 -10.329 114.880  1.00158.98
O
ANISOU11337  O   GLY B 659    17064  29850  13490  -5595  -1148
-3431       O
ATOM  11338  N   PHE B 660      34.452 -12.262 114.241  1.00140.81
N
ANISOU11338  N   PHE B 660    14898  27465  11139  -5542  -1266
-2800       N
ATOM  11339  CA  PHE B 660      34.636 -12.887 115.551  1.00123.49
C
ANISOU11339  CA  PHE B 660    12515  25750   8656  -5678  -1318
-2597       C
ATOM  11340  C   PHE B 660      36.100 -13.137 115.921  1.00124.81
C
ANISOU11340  C   PHE B 660    12608  26038   8777  -5721  -1444
-2478       C
ATOM  11341  O   PHE B 660      36.489 -12.911 117.072  1.00130.35
O
ANISOU11341  O   PHE B 660    13117  27134   9278  -5830  -1428
-2556       O
ATOM  11342  CB  PHE B 660      33.833 -14.186 115.629  1.00123.24
C
ANISOU11342  CB  PHE B 660    12526  25805   8495  -5723  -1398
-2168       C
ATOM  11343  CG  PHE B 660      32.352 -13.987 115.485  1.00122.74
C
ANISOU11343  CG  PHE B 660    12470  25749   8417  -5713  -1264
-2279       C
ATOM  11344  CD1 PHE B 660      31.569 -14.918 114.827  1.00120.83
C
```

```
ANISOU11344  CD1 PHE B 660    12370  25331   8211  -5700  -1319
-1970       C
ATOM  11345  CD2 PHE B 660      31.741 -12.867 116.023  1.00124.50
C
ANISOU11345  CD2 PHE B 660    12548  26170   8587  -5709  -1094
-2706       C
ATOM  11346  CE1 PHE B 660      30.205 -14.734 114.706  1.00121.21
C
ANISOU11346  CE1 PHE B 660    12394  25426   8233  -5695  -1192
-2079       C
ATOM  11347  CE2 PHE B 660      30.380 -12.676 115.902  1.00124.38
C
ANISOU11347  CE2 PHE B 660    12507  26202   8551  -5673   -979
-2828       C
ATOM  11348  CZ  PHE B 660      29.611 -13.611 115.243  1.00122.41
C
ANISOU11348  CZ  PHE B 660    12379  25803   8327  -5672  -1021
-2511       C
ATOM  11349  N   MET B 661      36.928 -13.623 114.994  1.00122.64
N
ANISOU11349  N   MET B 661    12459  25468   8669  -5631  -1573
-2293       N
ATOM  11350  CA  MET B 661      38.348 -13.768 115.321  1.00124.17
C
ANISOU11350  CA  MET B 661    12550  25806   8822  -5650  -1688
-2226       C
ATOM  11351  C   MET B 661      39.039 -12.426 115.562  1.00125.53
C
ANISOU11351  C   MET B 661    12616  26035   9046  -5709  -1570
-2656       C
ATOM  11352  O   MET B 661      39.897 -12.324 116.446  1.00186.26
O
ANISOU11352  O   MET B 661    20134  34051  16586  -5793  -1605
-2685       O
ATOM  11353  CB  MET B 661      39.083 -14.564 114.233  1.00121.82
C
ANISOU11353  CB  MET B 661    12385  25209   8694  -5517  -1855
-1965       C
ATOM  11354  CG  MET B 661      39.474 -13.806 112.962  1.00147.38
C
ANISOU11354  CG  MET B 661    15727  28059  12211  -5438  -1789
-2202       C
ATOM  11355  SD  MET B 661      41.052 -12.929 113.102  1.00120.95
S
ANISOU11355  SD  MET B 661    12219  24834   8904  -5501  -1774
-2460       S
ATOM  11356  CE  MET B 661      41.075 -12.002 111.570  1.00158.95
C
ANISOU11356  CE  MET B 661    17199  29155  14039  -5462  -1667
-2720       C
ATOM  11357  N   LEU B 662      38.691 -11.388 114.796  1.00123.85
N
ANISOU11357  N   LEU B 662    12514  25502   9043  -5675  -1444
-2987       N
```

```
ATOM  11358  CA  LEU B 662      39.294 -10.075 115.023  1.00125.55
C
ANISOU11358  CA  LEU B 662    12663  25721   9317  -5754  -1351
-3397       C
ATOM  11359  C   LEU B 662      38.930  -9.488 116.388  1.00155.86
C
ANISOU11359  C   LEU B 662    16325  29955  12939  -5855  -1264
-3634       C
ATOM  11360  O   LEU B 662      39.800  -8.961 117.091  1.00181.15
O
ANISOU11360  O   LEU B 662    19386  33387  16057  -5958  -1261
-3803       O
ATOM  11361  CB  LEU B 662      38.902  -9.121 113.893  1.00123.29
C
ANISOU11361  CB  LEU B 662    12573  24962   9309  -5697  -1261
-3676       C
ATOM  11362  CG  LEU B 662      39.579  -7.752 113.865  1.00126.97
C
ANISOU11362  CG  LEU B 662    13042  25305   9894  -5791  -1197
-4076       C
ATOM  11363  CD1 LEU B 662      41.080  -7.916 113.998  1.00167.95
C
ANISOU11363  CD1 LEU B 662    18105  30659  15048  -5887  -1274
-3982       C
ATOM  11364  CD2 LEU B 662      39.251  -7.038 112.569  1.00122.47
C
ANISOU11364  CD2 LEU B 662    12710  24211   9613  -5735  -1150
-4248       C
ATOM  11365  N   ILE B 663      37.654  -9.556 116.783  1.00129.46
N
ANISOU11365  N   ILE B 663    12970  26724   9495  -5831  -1190
-3665       N
ATOM  11366  CA  ILE B 663      37.304  -9.155 118.148  1.00133.23
C
ANISOU11366  CA  ILE B 663    13237  27656   9728  -5926  -1116
-3857       C
ATOM  11367  C   ILE B 663      37.975 -10.067 119.173  1.00135.65
C
ANISOU11367  C   ILE B 663    13359  28416   9767  -6035  -1212
-3545       C
ATOM  11368  O   ILE B 663      38.420  -9.607 120.231  1.00139.08
O
ANISOU11368  O   ILE B 663    13602  29216  10027  -6140  -1183
-3723       O
ATOM  11369  CB  ILE B 663      35.775  -9.086 118.339  1.00166.47
C
ANISOU11369  CB  ILE B 663    17437  31944  13871  -5875  -1014
-3950       C
ATOM  11370  CG1 ILE B 663      35.117 -10.457 118.200  1.00186.61
C
ANISOU11370  CG1 ILE B 663    20018  34565  16318  -5873  -1076
-3484       C
ATOM  11371  CG2 ILE B 663      35.164  -8.098 117.354  1.00179.60
C
```

```
ANISOU11371  CG2 ILE B 663    19284  33153  15801  -5741   -938
-4291       C
ATOM  11372  CD1 ILE B 663      33.615 -10.432 118.374  1.00181.07
C
ANISOU11372  CD1 ILE B 663    19276  33987  15536  -5846   -969
-3560       C
ATOM  11373  N   ALA B 664      38.054 -11.369 118.882  1.00134.18
N
ANISOU11373  N   ALA B 664    13235  28205   9543  -6008  -1345
-3079       N
ATOM  11374  CA  ALA B 664      38.850 -12.271 119.713  1.00153.21
C
ANISOU11374  CA  ALA B 664    15512  30968  11734  -6086  -1484
-2756       C
ATOM  11375  C   ALA B 664      40.309 -11.833 119.759  1.00157.01
C
ANISOU11375  C   ALA B 664    15913  31484  12260  -6102  -1537
-2882       C
ATOM  11376  O   ALA B 664      40.941 -11.851 120.821  1.00174.57
O
ANISOU11376  O   ALA B 664    17944  34115  14270  -6201  -1569
-2879       O
ATOM  11377  CB  ALA B 664      38.735 -13.706 119.200  1.00147.35
C
ANISOU11377  CB  ALA B 664    14907  30079  11001  -6022  -1658
-2250       C
ATOM  11378  N   LEU B 665      40.867 -11.462 118.606  1.00155.24
N
ANISOU11378  N   LEU B 665    15825  30859  12302  -6017  -1547
-2982       N
ATOM  11379  CA  LEU B 665      42.281 -11.114 118.528  1.00157.08
C
ANISOU11379  CA  LEU B 665    15971  31128  12582  -6045  -1599
-3075       C
ATOM  11380  C   LEU B 665      42.594  -9.886 119.374  1.00167.43
C
ANISOU11380  C   LEU B 665    17132  32672  13812  -6185  -1478
-3493       C
ATOM  11381  O   LEU B 665      43.609  -9.849 120.079  1.00202.68
O
ANISOU11381  O   LEU B 665    21420  37454  18137  -6267  -1528
-3505       O
ATOM  11382  CB  LEU B 665      42.689 -10.881 117.073  1.00148.75
C
ANISOU11382  CB  LEU B 665    15083  29607  11827  -5954  -1608
-3120       C
ATOM  11383  CG  LEU B 665      44.146 -10.489 116.821  1.00161.53
C
ANISOU11383  CG  LEU B 665    16606  31255  13514  -5998  -1647
-3222       C
ATOM  11384  CD1 LEU B 665      45.088 -11.554 117.369  1.00155.18
C
ANISOU11384  CD1 LEU B 665    15641  30789  12530  -5956  -1825
-2899       C
```

ATOM 11385 CD2 LEU B 665   44.388 -10.261 115.334 1.00166.80
C
ANISOU11385 CD2 LEU B 665   17435 31472 14468 -5930 -1637
-3259   C
ATOM 11386 N   ILE B 666   41.728 -8.875 119.319 1.00164.50
N
ANISOU11386 N   ILE B 666   16830 32146 13526 -6202 -1334
-3848   N
ATOM 11387 CA ILE B 666   41.986 -7.613 120.003 1.00173.23
C
ANISOU11387 CA ILE B 666   17833 33390 14596 -6318 -1238
-4289   C
ATOM 11388 C   ILE B 666   41.558 -7.677 121.467 1.00183.09
C
ANISOU11388 C   ILE B 666   18864 35163 15537 -6398 -1199
-4340   C
ATOM 11389 O   ILE B 666   42.332 -7.326 122.364 1.00197.56
O
ANISOU11389 O   ILE B 666   20515 37337 17213 -6515 -1203
-4474   O
ATOM 11390 CB ILE B 666   41.288 -6.458 119.262 1.00155.69
C
ANISOU11390 CB ILE B 666   15802 30730 12622 -6276 -1136
-4669   C
ATOM 11391 CG1 ILE B 666   41.763 -6.390 117.809 1.00137.45
C
ANISOU11391 CG1 ILE B 666   13698 27923 10603 -6226 -1173
-4602   C
ATOM 11392 CG2 ILE B 666   41.571 -5.136 119.959 1.00159.91
C
ANISOU11392 CG2 ILE B 666   16262 31359 13136 -6390 -1072
-5138   C
ATOM 11393 CD1 ILE B 666   43.230 -6.062 117.657 1.00138.65
C
ANISOU11393 CD1 ILE B 666   13784 28093 10803 -6346 -1218
-4647   C
ATOM 11394 N   TRP B 667   40.331 -8.125 121.743 1.00163.98
N
ANISOU11394 N   TRP B 667   16443 32850 13012 -6351 -1156
-4235   N
ATOM 11395 CA TRP B 667   39.767 -8.009 123.084 1.00149.10
C
ANISOU11395 CA TRP B 667   14339 31470 10843 -6438 -1088
-4350   C
ATOM 11396 C   TRP B 667   39.484 -9.349 123.756 1.00149.99
C
ANISOU11396 C   TRP B 667   14340 31958 10691 -6488 -1162
-3886   C
ATOM 11397 O   TRP B 667   38.932 -9.356 124.862 1.00153.17
O
ANISOU11397 O   TRP B 667   14547 32816 10836 -6580 -1101
-3937   O
ATOM 11398 CB TRP B 667   38.473 -7.182 123.060 1.00149.28
C

```
ANISOU11398  CB  TRP B 667    14395  31398  10924  -6371   -955
-4711       C
ATOM  11399  CG  TRP B 667      38.649  -5.770 122.576  1.00149.40
C
ANISOU11399  CG  TRP B 667    14532  31054  11181  -6331   -904
-5201       C
ATOM  11400  CD1 TRP B 667      39.053  -4.694 123.312  1.00153.02
C
ANISOU11400  CD1 TRP B 667    14881  31670  11591  -6410   -867
-5619       C
ATOM  11401  CD2 TRP B 667      38.368  -5.272 121.261  1.00168.38
C
ANISOU11401  CD2 TRP B 667    17205  32864  13908  -6212   -898
-5323       C
ATOM  11402  NE1 TRP B 667      39.073  -3.564 122.529  1.00152.30
N
ANISOU11402  NE1 TRP B 667    14997  31088  11782  -6355   -855
-5982       N
ATOM  11403  CE2 TRP B 667      38.652  -3.892 121.267  1.00155.88
C
ANISOU11403  CE2 TRP B 667    15682  31081  12465  -6234   -872
-5803       C
ATOM  11404  CE3 TRP B 667      37.917  -5.862 120.076  1.00141.93
C
ANISOU11404  CE3 TRP B 667    14059  29127  10741  -6096   -924
-5070       C
ATOM  11405  CZ2 TRP B 667      38.501  -3.093 120.135  1.00146.09
C
ANISOU11405  CZ2 TRP B 667    14709  29259  11538  -6153   -876
-6016       C
ATOM  11406  CZ3 TRP B 667      37.766  -5.068 118.954  1.00139.80
C
ANISOU11406  CZ3 TRP B 667    14030  28314  10774  -6008   -911
-5293       C
ATOM  11407  CH2 TRP B 667      38.057  -3.699 118.992  1.00141.92
C
ANISOU11407  CH2 TRP B 667    14363  28385  11175  -6040   -890
-5753       C
ATOM  11408  N   GLU B 668      39.819 -10.467 123.111  1.00147.55
N
ANISOU11408  N   GLU B 668    14156  31466  10441  -6432  -1302
-3442       N
ATOM  11409  CA  GLU B 668      39.493 -11.833 123.553  1.00148.17
C
ANISOU11409  CA  GLU B 668    14204  31779  10315  -6473  -1416
-2951       C
ATOM  11410  C   GLU B 668      38.048 -11.976 124.040  1.00149.09
C
ANISOU11410  C   GLU B 668    14261  32106  10279  -6533  -1309
-2941       C
ATOM  11411  O   GLU B 668      37.768 -12.523 125.109  1.00152.25
O
ANISOU11411  O   GLU B 668    14490  32967  10389  -6670  -1325
-2751       O
```

```
ATOM  11412  CB  GLU B 668      40.498 -12.335 124.603  1.00154.95
C
ANISOU11412  CB  GLU B 668    14875  33075  10923  -6576  -1534
-2755       C
ATOM  11413  CG  GLU B 668      40.555 -11.605 125.943  1.00173.30
C
ANISOU11413  CG  GLU B 668    16931  35920  12994  -6722  -1426
-3049       C
ATOM  11414  CD  GLU B 668      41.164 -12.453 127.045  1.00220.38
C
ANISOU11414  CD  GLU B 668    22719  42362  18653  -6836  -1558
-2722       C
ATOM  11415  OE1 GLU B 668      42.340 -12.219 127.398  1.00224.76
O
ANISOU11415  OE1 GLU B 668    23163  43074  19159  -6856  -1618
-2797       O
ATOM  11416  OE2 GLU B 668      40.470 -13.357 127.555  1.00239.14
O
ANISOU11416  OE2 GLU B 668    25067  44961  20834  -6916  -1609
-2383       O
ATOM  11417  N   PHE B 669      37.111 -11.489 123.223  1.00163.67
N
ANISOU11417  N   PHE B 669    16243  33625  12321  -6433  -1202
-3142       N
ATOM  11418  CA  PHE B 669      35.713 -11.459 123.637  1.00147.54
C
ANISOU11418  CA  PHE B 669    14110  31804  10146  -6474  -1081
-3210       C
ATOM  11419  C   PHE B 669      35.053 -12.835 123.577  1.00146.88
C
ANISOU11419  C   PHE B 669    14086  31774   9949  -6541  -1170
-2693       C
ATOM  11420  O   PHE B 669      34.017 -13.035 124.221  1.00154.55
O
ANISOU11420  O   PHE B 669    14914  33088  10720  -6648  -1087
-2658       O
ATOM  11421  CB  PHE B 669      34.945 -10.472 122.754  1.00153.51
C
ANISOU11421  CB  PHE B 669    14991  32183  11152  -6322   -957
-3604       C
ATOM  11422  CG  PHE B 669      33.582 -10.124 123.268  1.00147.05
C
ANISOU11422  CG  PHE B 669    14023  31648  10201  -6331   -814
-3818       C
ATOM  11423  CD1 PHE B 669      33.419  -9.598 124.536  1.00151.45
C
ANISOU11423  CD1 PHE B 669    14300  32734  10512  -6428   -727
-4078       C
ATOM  11424  CD2 PHE B 669      32.465 -10.306 122.477  1.00147.81
C
ANISOU11424  CD2 PHE B 669    14239  31509  10415  -6235   -767
-3779       C
ATOM  11425  CE1 PHE B 669      32.163  -9.271 125.007  1.00157.62
C
```

```
ANISOU11425  CE1 PHE B 669    14906  33819  11164  -6422   -598
-4298       C
ATOM  11426  CE2 PHE B 669      31.209  -9.979 122.943  1.00146.57
C
ANISOU11426  CE2 PHE B 669    13910  31652  10129  -6231   -638
-3992       C
ATOM  11427  CZ  PHE B 669      31.058  -9.461 124.209  1.00151.07
C
ANISOU11427  CZ  PHE B 669    14185  32764  10453  -6320   -553
-4256       C
ATOM  11428  N   ASP B 670      35.613 -13.760 122.789  1.00152.56
N
ANISOU11428  N   ASP B 670    15011  32155  10800  -6482  -1342
-2308       N
ATOM  11429  CA  ASP B 670      35.201 -15.160 122.665  1.00156.72
C
ANISOU11429  CA  ASP B 670    15647  32651  11249  -6544  -1486
-1777       C
ATOM  11430  C   ASP B 670      33.921 -15.349 121.851  1.00150.87
C
ANISOU11430  C   ASP B 670    15044  31654  10627  -6501  -1413
-1737       C
ATOM  11431  O   ASP B 670      33.917 -16.094 120.865  1.00157.80
O
ANISOU11431  O   ASP B 670    16154  32128  11676  -6417  -1535
-1456       O
ATOM  11432  CB  ASP B 670      35.058 -15.804 124.049  1.00179.16
C
ANISOU11432  CB  ASP B 670    18283  36056  13733  -6766  -1529
-1522       C
ATOM  11433  CG  ASP B 670      36.366 -15.815 124.823  1.00196.27
C
ANISOU11433  CG  ASP B 670    20329  38473  15771  -6802  -1637
-1486       C
ATOM  11434  OD1 ASP B 670      37.438 -15.829 124.181  1.00194.34
O
ANISOU11434  OD1 ASP B 670    20205  37926  15710  -6661  -1754
-1469       O
ATOM  11435  OD2 ASP B 670      36.326 -15.814 126.071  1.00226.54
O
ANISOU11435  OD2 ASP B 670    23930  42833  19312  -6974  -1605
-1478       O
ATOM  11436  N   PHE B 671      32.833 -14.701 122.281  1.00146.56
N
ANISOU11436  N   PHE B 671    14338  31367   9981  -6552  -1224
-2022       N
ATOM  11437  CA  PHE B 671      31.512 -14.735 121.651  1.00167.24
C
ANISOU11437  CA  PHE B 671    17024  33842  12677  -6514  -1126
-2052       C
ATOM  11438  C   PHE B 671      30.812 -16.074 121.889  1.00141.85
C
ANISOU11438  C   PHE B 671    13827  30780   9288  -6700  -1217
-1544       C
```

```
ATOM  11439  O   PHE B 671      31.467 -17.116 121.997  1.00142.29
O
ANISOU11439  O   PHE B 671    13989  30785   9291  -6779  -1417
-1108       O
ATOM  11440  CB  PHE B 671      31.593 -14.425 120.152  1.00183.49
C
ANISOU11440  CB  PHE B 671    19351  35280  15088  -6294  -1138
-2165       C
ATOM  11441  CG  PHE B 671      30.289 -13.965 119.564  1.00185.50
C
ANISOU11441  CG  PHE B 671    19627  35416  15438  -6206   -994
-2390       C
ATOM  11442  CD1 PHE B 671      29.457 -14.841 118.894  1.00184.02
C
ANISOU11442  CD1 PHE B 671    19575  35054  15291  -6224  -1033
-2080       C
ATOM  11443  CD2 PHE B 671      29.896 -12.642 119.691  1.00178.96
C
ANISOU11443  CD2 PHE B 671    18687  34649  14661  -6097   -835
-2924       C
ATOM  11444  CE1 PHE B 671      28.253 -14.402 118.363  1.00163.11
C
ANISOU11444  CE1 PHE B 671    16925  32330  12721  -6135   -901
-2299       C
ATOM  11445  CE2 PHE B 671      28.698 -12.201 119.161  1.00149.62
C
ANISOU11445  CE2 PHE B 671    14981  30835  11032  -5983   -721
-3147       C
ATOM  11446  CZ  PHE B 671      27.876 -13.082 118.496  1.00146.52
C
ANISOU11446  CZ  PHE B 671    14700  30303  10668  -6002   -747
-2833       C
ATOM  11447  N   SER B 672      29.481 -16.057 121.966  1.00142.52
N
ANISOU11447  N   SER B 672    13811  31054   9287  -6773  -1088
-1599       N
ATOM  11448  CA  SER B 672      28.698 -17.266 122.214  1.00143.94
C
ANISOU11448  CA  SER B 672    13991  31406   9294  -6999  -1159
-1135       C
ATOM  11449  C   SER B 672      28.580 -18.111 120.948  1.00140.16
C
ANISOU11449  C   SER B 672    13839  30370   9044  -6921  -1297
-811       C
ATOM  11450  O   SER B 672      28.026 -17.658 119.941  1.00142.06
O
ANISOU11450  O   SER B 672    14190  30289   9499  -6751  -1209
-1024       O
ATOM  11451  CB  SER B 672      27.313 -16.899 122.738  1.00150.37
C
ANISOU11451  CB  SER B 672    14539  32667   9929  -7113   -960
-1340       C
ATOM  11452  OG  SER B 672      26.505 -18.053 122.888  1.00166.31
O
```

ANISOU11452 OG SER B 672   16560 34838 11795 -7367 -1025
-890   O
ATOM 11453 N   ALA B 673   29.101 -19.340 121.004 1.00140.74
N
ANISOU11453 N   ALA B 673   14073 30330   9073 -7036 -1530
-301   N
ATOM 11454 CA ALA B 673   28.880 -20.317 119.939 1.00158.35
C
ANISOU11454 CA ALA B 673   16604 32080 11480 -7001 -1688
56   C
ATOM 11455 C   ALA B 673   27.401 -20.644 119.743 1.00164.40
C
ANISOU11455 C   ALA B 673   17338 32939 12186 -7155 -1588
142   C
ATOM 11456 O   ALA B 673   26.939 -20.818 118.609 1.00161.85
O
ANISOU11456 O   ALA B 673   17214 32208 12073 -7041 -1596
166   O
ATOM 11457 CB ALA B 673   29.671 -21.592 120.234 1.00151.70
C
ANISOU11457 CB ALA B 673   15921 31149 10569 -7105 -1984
581   C
ATOM 11458 N   PHE B 674   26.649 -20.752 120.838 1.00142.29
N
ANISOU11458 N   PHE B 674   14272 30700   9092 -7427 -1496
193   N
ATOM 11459 CA PHE B 674   25.209 -20.991 120.755 1.00143.10
C
ANISOU11459 CA PHE B 674   14275 30991   9106 -7601 -1381
240   C
ATOM 11460 C   PHE B 674   24.462 -19.879 120.022 1.00162.10
C
ANISOU11460 C   PHE B 674   16609 33308 11674 -7363 -1154
-263   C
ATOM 11461 O   PHE B 674   23.548 -20.156 119.236 1.00174.90
O
ANISOU11461 O   PHE B 674   18317 34748 13388 -7362 -1122
-204   O
ATOM 11462 CB PHE B 674   24.632 -21.182 122.158 1.00148.46
C
ANISOU11462 CB PHE B 674   14617 32371   9418 -7942 -1311
334   C
ATOM 11463 CG PHE B 674   23.236 -21.741 122.170 1.00150.14
C
ANISOU11463 CG PHE B 674   14723 32821   9504 -8210 -1249
513   C
ATOM 11464 CD1 PHE B 674   23.021 -23.109 122.126 1.00151.51
C
ANISOU11464 CD1 PHE B 674   15076 32870   9620 -8501 -1459
1082   C
ATOM 11465 CD2 PHE B 674   22.138 -20.898 122.228 1.00150.71
C
ANISOU11465 CD2 PHE B 674   14507 33243   9515 -8173   -997
105   C

```
ATOM  11466  CE1 PHE B 674      21.735 -23.626 122.138  1.00153.36           C
ANISOU11466  CE1 PHE B 674    15197  33338   9733  -8790  -1406   1248       C
ATOM  11467  CE2 PHE B 674      20.850 -21.409 122.240  1.00152.53           C
ANISOU11467  CE2 PHE B 674    14600  33739   9617  -8431   -938    258       C
ATOM  11468  CZ  PHE B 674      20.649 -22.775 122.195  1.00153.83           C
ANISOU11468  CZ  PHE B 674    14938  33791   9720  -8760  -1138    836       C
ATOM  11469  N   MET B 675      24.822 -18.618 120.264  1.00140.31           N
ANISOU11469  N   MET B 675    13698  30665   8950  -7159  -1009   -760       N
ATOM  11470  CA  MET B 675      24.260 -17.529 119.464  1.00164.82           C
ANISOU11470  CA  MET B 675    16794  33578  12252  -6887   -843  -1241       C
ATOM  11471  C   MET B 675      24.575 -17.656 117.975  1.00168.86           C
ANISOU11471  C   MET B 675    17658  33402  13098  -6661   -934  -1185       C
ATOM  11472  O   MET B 675      23.693 -17.444 117.134  1.00149.00           O
ANISOU11472  O   MET B 675    15197  30704  10713  -6548   -853  -1318       O
ATOM  11473  CB  MET B 675      24.751 -16.186 119.996  1.00138.82           C
ANISOU11473  CB  MET B 675    13325  30460   8961  -6717   -722  -1765       C
ATOM  11474  CG  MET B 675      24.147 -15.810 121.329  1.00143.67           C
ANISOU11474  CG  MET B 675    13545  31779   9264  -6880   -583  -1958       C
ATOM  11475  SD  MET B 675      22.361 -15.560 121.245  1.00145.03           S
ANISOU11475  SD  MET B 675    13472  32286   9347  -6892   -401  -2172       S
ATOM  11476  CE  MET B 675      21.755 -17.108 121.920  1.00148.17           C
ANISOU11476  CE  MET B 675    13760  33095   9442  -7350   -477  -1550       C
ATOM  11477  N   VAL B 676      25.817 -17.991 117.622  1.00143.72           N
ANISOU11477  N   VAL B 676    14697  29858  10051  -6587  -1104  -1002       N
ATOM  11478  CA  VAL B 676      26.134 -18.251 116.218  1.00126.67           C
ANISOU11478  CA  VAL B 676    12855  27084   8189  -6398  -1208   -906       C
ATOM  11479  C   VAL B 676      25.332 -19.436 115.694  1.00126.04           C
```

```
ANISOU11479  C   VAL B 676    12925  26859   8106  -6536  -1302
-490       C
ATOM  11480  O   VAL B 676      24.853 -19.429 114.554  1.00158.68
O
ANISOU11480  O   VAL B 676    17224  30628  12438  -6399  -1286
-532       O
ATOM  11481  CB  VAL B 676      27.646 -18.472 116.037  1.00125.55
C
ANISOU11481  CB  VAL B 676    12875  26663   8164  -6307  -1387
-775       C
ATOM  11482  CG1 VAL B 676      27.976 -18.596 114.572  1.00145.07
C
ANISOU11482  CG1 VAL B 676    15631  28545  10943  -6096  -1472
-744       C
ATOM  11483  CG2 VAL B 676      28.428 -17.335 116.648  1.00126.70
C
ANISOU11483  CG2 VAL B 676    12856  26994   8291  -6224  -1294
-1171       C
ATOM  11484  N   LEU B 677      25.184 -20.474 116.517  1.00129.20
N
ANISOU11484  N   LEU B 677    13276  27534   8279  -6824  -1414
-77       N
ATOM  11485  CA  LEU B 677      24.282 -21.578 116.203  1.00129.59
C
ANISOU11485  CA  LEU B 677    13435  27516   8287  -7027  -1497
313       C
ATOM  11486  C   LEU B 677      22.845 -21.108 115.975  1.00129.57
C
ANISOU11486  C   LEU B 677    13262  27721   8247  -7053  -1281
72       C
ATOM  11487  O   LEU B 677      22.180 -21.560 115.035  1.00136.51
O
ANISOU11487  O   LEU B 677    14301  28315   9252  -7042  -1306
196       O
ATOM  11488  CB  LEU B 677      24.350 -22.611 117.334  1.00133.96
C
ANISOU11488  CB  LEU B 677    13920  28406   8571  -7374  -1643
755       C
ATOM  11489  CG  LEU B 677      23.492 -23.882 117.419  1.00136.13
C
ANISOU11489  CG  LEU B 677    14273  28724   8727  -7710  -1768
1234       C
ATOM  11490  CD1 LEU B 677      22.098 -23.615 117.988  1.00138.81
C
ANISOU11490  CD1 LEU B 677    14287  29608   8845  -7951  -1550
1104       C
ATOM  11491  CD2 LEU B 677      23.400 -24.582 116.081  1.00135.91
C
ANISOU11491  CD2 LEU B 677    14596  28086   8955  -7602  -1917
1446       C
ATOM  11492  N   ILE B 678      22.343 -20.213 116.827  1.00132.05
N
ANISOU11492  N   ILE B 678    13240  28547   8386  -7078  -1078
-283       N
```

ATOM 11493 CA ILE B 678   21.043 -19.581 116.589 1.00132.14
C
ANISOU11493 CA ILE B 678   13057 28771   8379 -7023   -872
-603   C
ATOM 11494 C   ILE B 678   21.002 -18.787 115.283 1.00136.19
C
ANISOU11494 C   ILE B 678   13748 28798   9201 -6660   -813
-939   C
ATOM 11495 O   ILE B 678   20.030 -18.882 114.523 1.00196.12
O
ANISOU11495 O   ILE B 678   21368 36283 16864 -6619   -757
-963   O
ATOM 11496 CB ILE B 678   20.659 -18.701 117.793 1.00135.99
C
ANISOU11496 CB ILE B 678   13139 29900   8629 -7070   -689
-966   C
ATOM 11497 CG1 ILE B 678   20.374 -19.572 119.017 1.00140.71
C
ANISOU11497 CG1 ILE B 678   13522 31048   8893 -7485   -729
-607   C
ATOM 11498 CG2 ILE B 678   19.456 -17.831 117.466 1.00135.97
C
ANISOU11498 CG2 ILE B 678   12935 30075   8652 -6905   -491
-1398   C
ATOM 11499 CD1 ILE B 678   19.196 -20.513 118.835 1.00141.98
C
ANISOU11499 CD1 ILE B 678   13645 31352   8950 -7769   -740
-284   C
ATOM 11500 N   ILE B 679   22.039 -17.996 114.992 1.00125.72
N
ANISOU11500 N   ILE B 679   12535 27177   8055 -6408   -829
-1199   N
ATOM 11501 CA ILE B 679   22.104 -17.326 113.690 1.00121.68
C
ANISOU11501 CA ILE B 679   12227 26158   7847 -6096   -804
-1463   C
ATOM 11502 C   ILE B 679   22.112 -18.323 112.535 1.00118.59
C
ANISOU11502 C   ILE B 679   12141 25292   7625 -6098   -948
-1094   C
ATOM 11503 O   ILE B 679   21.409 -18.136 111.534 1.00116.29
O
ANISOU11503 O   ILE B 679   11942 24758   7484 -5956   -895
-1208   O
ATOM 11504 CB ILE B 679   23.329 -16.393 113.619 1.00120.45
C
ANISOU11504 CB ILE B 679   12144 25773   7847 -5892   -819
-1756   C
ATOM 11505 CG1 ILE B 679   23.243 -15.296 114.677 1.00123.58
C
ANISOU11505 CG1 ILE B 679   12253 26601   8101 -5864   -677
-2182   C
ATOM 11506 CG2 ILE B 679   23.452 -15.771 112.236 1.00116.50
C

ANISOU11506 CG2 ILE B 679   11872 24730   7661 -5613   -812
-1982   C
ATOM 11507 CD1 ILE B 679   24.382 -14.297 114.610 1.00122.74
C
ANISOU11507 CD1 ILE B 679   12216 26266   8152 -5690   -688
-2501   C
ATOM 11508 N   ALA B 680   22.902 -19.390 112.650 1.00118.71
N
ANISOU11508 N   ALA B 680   12321 25165   7620 -6243 -1147
-657   N
ATOM 11509 CA ALA B 680   22.922 -20.422 111.616 1.00116.31
C
ANISOU11509 CA ALA B 680   12311 24413   7469 -6249 -1314
-296   C
ATOM 11510 C   ALA B 680   21.560 -21.076 111.401 1.00117.08
C
ANISOU11510 C   ALA B 680   12382 24623   7480 -6429 -1277
-112   C
ATOM 11511 O   ALA B 680   21.136 -21.273 110.257 1.00161.99
O
ANISOU11511 O   ALA B 680   18249 29957 13344 -6321 -1295
-87   O
ATOM 11512 CB ALA B 680   23.970 -21.479 111.959 1.00117.29
C
ANISOU11512 CB ALA B 680   12593 24416   7558 -6370 -1562
135   C
ATOM 11513 N   ILE B 681   20.861 -21.424 112.482 1.00120.97
N
ANISOU11513 N   ILE B 681   12638 25627   7696 -6722 -1225
19   N
ATOM 11514 CA ILE B 681   19.490 -21.921 112.357 1.00122.22
C
ANISOU11514 CA ILE B 681   12705 25982   7751 -6925 -1160
144   C
ATOM 11515 C   ILE B 681   18.556 -20.889 111.727 1.00120.64
C
ANISOU11515 C   ILE B 681   12370 25830   7638 -6689   -947
-310   C
ATOM 11516 O   ILE B 681   17.743 -21.226 110.857 1.00222.30
O
ANISOU11516 O   ILE B 681   25332 38534 20597 -6685   -939
-244   O
ATOM 11517 CB ILE B 681   18.973 -22.386 113.731 1.00127.22
C
ANISOU11517 CB ILE B 681   13059 27228   8051 -7309 -1134
340   C
ATOM 11518 CG1 ILE B 681   19.745 -23.624 114.195 1.00128.98
C
ANISOU11518 CG1 ILE B 681   13475 27328   8203 -7566 -1395
872   C
ATOM 11519 CG2 ILE B 681   17.478 -22.681 113.668 1.00128.95
C
ANISOU11519 CG2 ILE B 681   13097 27758   8142 -7524 -1026
379   C

```
ATOM  11520  CD1 ILE B 681      19.363 -24.096 115.580  1.00178.32
C
ANISOU11520  CD1 ILE B 681    19462  34169  14121  -7968  -1395
1093       C
ATOM  11521  N   LEU B 682      18.649 -19.623 112.143  1.00121.11
N
ANISOU11521  N   LEU B 682    12222  26109   7685  -6483   -789
-782       N
ATOM  11522  CA  LEU B 682      17.882 -18.572 111.471  1.00146.31
C
ANISOU11522  CA  LEU B 682    15329  29263  10999  -6198   -628
-1237       C
ATOM  11523  C   LEU B 682      18.248 -18.434 109.996  1.00137.06
C
ANISOU11523  C   LEU B 682    14486  27446  10142  -5929   -696
-1280       C
ATOM  11524  O   LEU B 682      17.367 -18.255 109.147  1.00130.60
O
ANISOU11524  O   LEU B 682    13688  26515   9419  -5803   -631
-1406       O
ATOM  11525  CB  LEU B 682      18.078 -17.237 112.193  1.00146.82
C
ANISOU11525  CB  LEU B 682    15163  29602  11020  -6012   -495
-1736       C
ATOM  11526  CG  LEU B 682      17.467 -17.113 113.591  1.00165.82
C
ANISOU11526  CG  LEU B 682    17169  32724  13111  -6220   -383
-1825       C
ATOM  11527  CD1 LEU B 682      17.948 -15.846 114.288  1.00169.03
C
ANISOU11527  CD1 LEU B 682    17408  33313  13503  -6025   -297
-2299       C
ATOM  11528  CD2 LEU B 682      15.943 -17.144 113.517  1.00168.66
C
ANISOU11528  CD2 LEU B 682    17281  33449  13353  -6274   -259
-1920       C
ATOM  11529  N   ASN B 683      19.540 -18.502 109.673  1.00151.22
N
ANISOU11529  N   ASN B 683    16519  28843  12095  -5838   -827
-1187       N
ATOM  11530  CA  ASN B 683      19.968 -18.591 108.278  1.00161.47
C
ANISOU11530  CA  ASN B 683    18128  29551  13674  -5639   -916
-1149       C
ATOM  11531  C   ASN B 683      19.406 -19.827 107.578  1.00148.63
C
ANISOU11531  C   ASN B 683    16671  27734  12068  -5786  -1025
-745       C
ATOM  11532  O   ASN B 683      18.875 -19.737 106.465  1.00162.93
O
ANISOU11532  O   ASN B 683    18602  29269  14035  -5639  -1001
-822       O
ATOM  11533  CB  ASN B 683      21.498 -18.569 108.211  1.00149.54
C
```

```
ANISOU11533  CB  ASN B 683    16788  27742  12288  -5563  -1049
-1086       C
ATOM  11534  CG  ASN B 683      22.047 -19.266 106.977  1.00146.89
C
ANISOU11534  CG  ASN B 683    16766  26872  12172  -5478  -1211
-840       C
ATOM  11535  OD1 ASN B 683      22.275 -18.635 105.944  1.00152.86
O
ANISOU11535  OD1 ASN B 683    17652  27279  13150  -5248  -1184
-1064       O
ATOM  11536  ND2 ASN B 683      22.290 -20.568 107.090  1.00142.65
N
ANISOU11536  ND2 ASN B 683    16357  26266  11576  -5664  -1395
-381       N
ATOM  11537  N   ASP B 684      19.520 -20.991 108.219  1.00150.46
N
ANISOU11537  N   ASP B 684    16926  28098  12143  -6085  -1160
-310       N
ATOM  11538  CA  ASP B 684      19.072 -22.251 107.623  1.00151.15
C
ANISOU11538  CA  ASP B 684    17210  27967  12253  -6262  -1303
104       C
ATOM  11539  C   ASP B 684      17.582 -22.255 107.283  1.00142.04
C
ANISOU11539  C   ASP B 684    15923  27009  11035  -6337  -1168
30       C
ATOM  11540  O   ASP B 684      17.177 -22.810 106.255  1.00135.24
O
ANISOU11540  O   ASP B 684    15254  25829  10301  -6323  -1235
176       O
ATOM  11541  CB  ASP B 684      19.407 -23.414 108.556  1.00167.85
C
ANISOU11541  CB  ASP B 684    19357  30230  14190  -6597  -1482
566       C
ATOM  11542  CG  ASP B 684      19.215 -24.762 107.894  1.00176.66
C
ANISOU11542  CG  ASP B 684    20753  30995  15373  -6763  -1696
1013       C
ATOM  11543  OD1 ASP B 684      18.545 -25.631 108.489  1.00187.31
O
ANISOU11543  OD1 ASP B 684    22051  32579  16538  -7120  -1757
1329       O
ATOM  11544  OD2 ASP B 684      19.739 -24.953 106.777  1.00176.84
O
ANISOU11544  OD2 ASP B 684    21049  30509  15635  -6549  -1814
1044       O
ATOM  11545  N   GLY B 685      16.749 -21.657 108.134  1.00151.18
N
ANISOU11545  N   GLY B 685    16738  28712  11992  -6416   -983
-203       N
ATOM  11546  CA  GLY B 685      15.329 -21.545 107.836  1.00113.76
C
ANISOU11546  CA  GLY B 685    11818  24223   7184  -6454   -844
-328       C
```

```
ATOM  11547  C   GLY B 685      14.952 -20.588 106.721  1.00110.72
C
ANISOU11547  C   GLY B 685    11473  23589   7006  -6076   -737
-724       C
ATOM  11548  O   GLY B 685      13.829 -20.670 106.214  1.00110.86
O
ANISOU11548  O   GLY B 685    11404  23713   7006  -6085   -663
-772       O
ATOM  11549  N   THR B 686      15.846 -19.682 106.329  1.00132.20
N
ANISOU11549  N   THR B 686    14317  25996   9919  -5759   -734
-1005       N
ATOM  11550  CA  THR B 686      15.513 -18.635 105.367  1.00136.74
C
ANISOU11550  CA  THR B 686    14922  26349  10682  -5402   -638
-1411       C
ATOM  11551  C   THR B 686      16.234 -18.748 104.027  1.00150.57
C
ANISOU11551  C   THR B 686    17027  27472  12712  -5214   -751
-1341       C
ATOM  11552  O   THR B 686      16.023 -17.892 103.161  1.00144.39
O
ANISOU11552  O   THR B 686    16301  26467  12096  -4927   -687
-1655       O
ATOM  11553  CB  THR B 686      15.785 -17.252 105.973  1.00116.03
C
ANISOU11553  CB  THR B 686    12127  23902   8058  -5179   -525
-1884       C
ATOM  11554  OG1 THR B 686      17.102 -17.221 106.537  1.00129.50
O
ANISOU11554  OG1 THR B 686    13919  25505   9778  -5226   -607
-1811       O
ATOM  11555  CG2 THR B 686      14.759 -16.932 107.051  1.00111.06
C
ANISOU11555  CG2 THR B 686    11104  23920   7174  -5277   -384
-2071       C
ATOM  11556  N   ILE B 687      17.071 -19.771 103.816  1.00134.96
N
ANISOU11556  N   ILE B 687    15285  25204  10790  -5357   -929
-948       N
ATOM  11557  CA  ILE B 687      17.824 -19.872 102.566  1.00117.96
C
ANISOU11557  CA  ILE B 687    13436  22489   8893  -5173  -1040
-900       C
ATOM  11558  C   ILE B 687      17.084 -20.614 101.457  1.00123.23
C
ANISOU11558  C   ILE B 687    14258  22919   9643  -5190  -1092
-714       C
ATOM  11559  O   ILE B 687      17.648 -20.785 100.367  1.00164.12
O
ANISOU11559  O   ILE B 687    19684  27647  15027  -5048  -1190
-661       O
ATOM  11560  CB  ILE B 687      19.216 -20.506 102.803  1.00128.42
C
```

```
ANISOU11560  CB  ILE B 687    14933  23597  10263  -5249  -1225
-625       C
ATOM  11561  CG1 ILE B 687      19.169 -21.748 103.714  1.00163.52
C
ANISOU11561  CG1 ILE B 687    19362  28255  14512  -5582  -1354
-190       C
ATOM  11562  CG2 ILE B 687      20.183 -19.467 103.351  1.00113.65
C
ANISOU11562  CG2 ILE B 687    12978  21784   8421  -5116  -1170
-915       C
ATOM  11563  CD1 ILE B 687      18.273 -22.896 103.268  1.00176.21
C
ANISOU11563  CD1 ILE B 687    21076  29792  16083  -5784  -1440
144       C
ATOM  11564  N   MET B 688      15.848 -21.055 101.684  1.00 97.47
N
ANISOU11564  N   MET B 688    10847  19962   6225  -5368  -1030
-623       N
ATOM  11565  CA  MET B 688      15.076 -21.642 100.595  1.00105.95
C
ANISOU11565  CA  MET B 688    12049  20828   7379  -5375  -1064
-497       C
ATOM  11566  C   MET B 688      14.207 -20.614  99.879  1.00 99.00
C
ANISOU11566  C   MET B 688    11056  19990   6571  -5099   -900
-895       C
ATOM  11567  O   MET B 688      13.473 -20.974  98.953  1.00151.37
O
ANISOU11567  O   MET B 688    17760  26494  13261  -5078   -906
-840       O
ATOM  11568  CB  MET B 688      14.220 -22.804 101.109  1.00152.11
C
ANISOU11568  CB  MET B 688    17820  26946  13028  -5757  -1112
-141       C
ATOM  11569  CG  MET B 688      15.048 -23.931 101.705  1.00156.27
C
ANISOU11569  CG  MET B 688    18516  27359  13502  -6028  -1322
295       C
ATOM  11570  SD  MET B 688      16.259 -24.563 100.523  1.00117.26
S
ANISOU11570  SD  MET B 688    14004  21711   8840  -5859  -1567
499       S
ATOM  11571  CE  MET B 688      16.981 -25.915 101.451  1.00118.51
C
ANISOU11571  CE  MET B 688    14310  21830   8888  -6188  -1829
1007       C
ATOM  11572  N   THR B 689      14.282 -19.348 100.296  1.00 95.47
N
ANISOU11572  N   THR B 689    10444  19710   6121  -4884   -770
-1298       N
ATOM  11573  CA  THR B 689      13.816 -18.241  99.467  1.00105.25
C
ANISOU11573  CA  THR B 689    11668  20828   7493  -4544   -668
-1697       C
```

```
ATOM  11574  C   THR B 689      14.650 -18.147  98.194  1.00112.57
C
ANISOU11574  C   THR B 689    12914  21181   8676  -4356   -758
-1687       C
ATOM  11575  O   THR B 689      14.136 -17.821  97.116  1.00146.24
O
ANISOU11575  O   THR B 689    17258  25248  13059  -4159   -728
-1826       O
ATOM  11576  CB  THR B 689      13.874 -16.933 100.264  1.00 95.78
C
ANISOU11576  CB  THR B 689    10269  19873   6250  -4364   -553
-2120       C
ATOM  11577  OG1 THR B 689      12.860 -16.944 101.276  1.00 99.32
O
ANISOU11577  OG1 THR B 689    10379  20901   6459  -4496   -450
-2187       O
ATOM  11578  CG2 THR B 689      13.657 -15.727  99.361  1.00 94.35
C
ANISOU11578  CG2 THR B 689    10157  19444   6248  -3987   -495
-2528       C
ATOM  11579  N   ILE B 690      15.945 -18.451  98.314  1.00103.21
N
ANISOU11579  N   ILE B 690    11897  19751   7567  -4420   -872
-1518       N
ATOM  11580  CA  ILE B 690      16.934 -18.227  97.260  1.00 95.67
C
ANISOU11580  CA  ILE B 690    11200  18308   6843  -4252   -948
-1548       C
ATOM  11581  C   ILE B 690      16.533 -18.913  95.957  1.00133.72
C
ANISOU11581  C   ILE B 690    16205  22830  11771  -4218  -1015
-1378       C
ATOM  11582  O   ILE B 690      16.650 -18.325  94.874  1.00132.36
O
ANISOU11582  O   ILE B 690    16164  22362  11766  -4002   -991
-1560       O
ATOM  11583  CB  ILE B 690      18.317 -18.700  97.747  1.00 96.45
C
ANISOU11583  CB  ILE B 690    11397  18290   6960  -4373  -1080
-1333       C
ATOM  11584  CG1 ILE B 690      18.783 -17.843  98.927  1.00112.18
C
ANISOU11584  CG1 ILE B 690    13210  20549   8863  -4375  -1003
-1559       C
ATOM  11585  CG2 ILE B 690      19.334 -18.685  96.619  1.00125.03
C
ANISOU11585  CG2 ILE B 690    15260  21444  10801  -4239  -1173
-1312       C
ATOM  11586  CD1 ILE B 690      18.945 -16.377  98.591  1.00118.14
C
ANISOU11586  CD1 ILE B 690    13961  21180   9747  -4126   -894
-2005       C
ATOM  11587  N   SER B 691      16.066 -20.161  96.030  1.00147.92
N
```

```
ANISOU11587  N   SER B 691    18030  24695  13480  -4445  -1106
-1027       N
ATOM  11588  CA  SER B 691      15.640 -20.851  94.815  1.00130.09
C
ANISOU11588  CA  SER B 691    15948  22160  11321  -4427  -1179
-872       C
ATOM  11589  C   SER B 691      14.466 -20.143  94.148  1.00115.81
C
ANISOU11589  C   SER B 691    14034  20447   9521  -4246  -1034
-1149       C
ATOM  11590  O   SER B 691      14.411 -20.050  92.916  1.00158.56
O
ANISOU11590  O   SER B 691    19603  25560  15081  -4082  -1050
-1203       O
ATOM  11591  CB  SER B 691      15.277 -22.302  95.133  1.00133.67
C
ANISOU11591  CB  SER B 691    16451  22674  11664  -4738  -1315
-455       C
ATOM  11592  OG  SER B 691      16.351 -22.964  95.777  1.00161.09
O
ANISOU11592  OG  SER B 691    20030  26053  15123  -4879  -1475
-196       O
ATOM  11593  N   LYS B 692      13.519 -19.636  94.934  1.00118.61
N
ANISOU11593  N   LYS B 692    14115  21237   9716  -4259   -898
-1332       N
ATOM  11594  CA  LYS B 692      12.251 -19.146  94.410  1.00138.82
C
ANISOU11594  CA  LYS B 692    16536  23962  12249  -4103   -783
-1558       C
ATOM  11595  C   LYS B 692      12.269 -17.635  94.194  1.00154.06
C
ANISOU11595  C   LYS B 692    18418  25850  14267  -3751   -677
-2012       C
ATOM  11596  O   LYS B 692      11.207 -17.013  94.080  1.00186.32
O
ANISOU11596  O   LYS B 692    22330  30162  18303  -3580   -578
-2268       O
ATOM  11597  CB  LYS B 692      11.110 -19.548  95.344  1.00154.22
C
ANISOU11597  CB  LYS B 692    18191  26445  13962  -4326   -710
-1494       C
ATOM  11598  CG  LYS B 692      11.066 -21.041  95.632  1.00160.17
C
ANISOU11598  CG  LYS B 692    19013  27228  14617  -4719   -829
-1032       C
ATOM  11599  CD  LYS B 692       9.806 -21.433  96.382  1.00154.58
C
ANISOU11599  CD  LYS B 692    18005  27061  13669  -4974   -743
-970       C
ATOM  11600  CE  LYS B 692       9.748 -22.936  96.613  1.00141.77
C
ANISOU11600  CE  LYS B 692    16494  25419  11955  -5401   -883
-493       C
```

ATOM 11601 NZ LYS B 692   10.743 -23.387 97.625 1.00164.09
N
ANISOU11601 NZ LYS B 692   19394 28232 14720 -5608   -985
-258   N
ATOM 11602 N   ASP B 693   13.458 -17.040 94.142 1.00144.12
N
ANISOU11602 N   ASP B 693   17315 24303 13142 -3645   -708
-2116   N
ATOM 11603 CA ASP B 693   13.651 -15.618 93.895 1.00141.24
C
ANISOU11603 CA ASP B 693   16974 23802 12889 -3343   -637
-2527   C
ATOM 11604 C   ASP B 693   13.264 -15.218 92.466 1.00135.64
C
ANISOU11604 C   ASP B 693   16422 22789 12327 -3096   -628
-2660   C
ATOM 11605 O   ASP B 693   12.910 -16.042 91.619 1.00135.36
O
ANISOU11605 O   ASP B 693   16471 22602 12357 -3130   -661
-2415   O
ATOM 11606 CB ASP B 693   15.097 -15.239 94.194 1.00142.64
C
ANISOU11606 CB ASP B 693   17286 23744 13168 -3370   -685
-2552   C
ATOM 11607 CG ASP B 693   15.257 -13.774 94.523 1.00137.89
C
ANISOU11607 CG ASP B 693   16646 23123 12623 -3150   -612
-2981   C
ATOM 11608 OD1 ASP B 693   14.355 -12.978 94.184 1.00133.61
O
ANISOU11608 OD1 ASP B 693   16050 22618 12097 -2906   -547
-3274   O
ATOM 11609 OD2 ASP B 693   16.283 -13.419 95.129 1.00138.13
O
ANISOU11609 OD2 ASP B 693   16704 23095 12684 -3215   -633
-3030   O
ATOM 11610 N   ARG B 694   13.339 -13.902 92.221 1.00159.15
N
ANISOU11610 N   ARG B 694   19442 25572 15457 -2794   -565
-3005   N
ATOM 11611 CA ARG B 694   12.900 -13.231 90.999 1.00161.79
C
ANISOU11611 CA ARG B 694   19905 25542 16025 -2460   -526
-3135   C
ATOM 11612 C   ARG B 694   14.064 -12.578 90.251 1.00128.65
C
ANISOU11612 C   ARG B 694   15980 20728 12172 -2304   -531
-3116   C
ATOM 11613 O   ARG B 694   13.835 -11.699 89.414 1.00123.34
O
ANISOU11613 O   ARG B 694   15431 19744 11690 -2019   -506
-3280   O
ATOM 11614 CB ARG B 694   11.841 -12.161 91.295 1.00185.37
C

```
ANISOU11614  CB  ARG B 694    22722  28820  18890  -2206   -473
-3573       C
ATOM  11615  CG  ARG B 694      10.398 -12.618  91.546  1.00204.71
C
ANISOU11615  CG  ARG B 694    24892  31806  21081  -2232   -439
-3610       C
ATOM  11616  CD  ARG B 694      10.219 -13.561  92.725  1.00230.10
C
ANISOU11616  CD  ARG B 694    27873  35456  24099  -2570   -422
-3367       C
ATOM  11617  NE  ARG B 694       8.802 -13.702  93.054  1.00233.94
N
ANISOU11617  NE  ARG B 694    28050  36422  24415  -2545   -356
-3441       N
ATOM  11618  CZ  ARG B 694       8.317 -14.509  93.991  1.00232.95
C
ANISOU11618  CZ  ARG B 694    27674  36747  24089  -2842   -323
-3249       C
ATOM  11619  NH1 ARG B 694       7.026 -14.475  94.292  1.00228.35
N
ANISOU11619  NH1 ARG B 694    26777  36634  23352  -2805   -251
-3372       N
ATOM  11620  NH2 ARG B 694       9.133 -15.288  94.688  1.00226.22
N
ANISOU11620  NH2 ARG B 694    26872  35899  23184  -3169   -365
-2953       N
ATOM  11621  N   VAL B 695      15.306 -12.974  90.547  1.00122.31
N
ANISOU11621  N   VAL B 695    15265  19768  11437  -2493   -572
-2918       N
ATOM  11622  CA  VAL B 695      16.481 -12.286  90.010  1.00119.21
C
ANISOU11622  CA  VAL B 695    15084  18887  11322  -2396   -571
-2928       C
ATOM  11623  C   VAL B 695      16.516 -12.318  88.487  1.00130.21
C
ANISOU11623  C   VAL B 695    16677  19827  12970  -2218   -561
-2779       C
ATOM  11624  O   VAL B 695      16.305 -13.361  87.855  1.00160.35
O
ANISOU11624  O   VAL B 695    20517  23614  16796  -2270   -586
-2516       O
ATOM  11625  CB  VAL B 695      17.765 -12.896  90.590  1.00148.28
C
ANISOU11625  CB  VAL B 695    18780  22564  14996  -2639   -625
-2717       C
ATOM  11626  CG1 VAL B 695      18.824 -11.818  90.774  1.00169.05
C
ANISOU11626  CG1 VAL B 695    21503  24955  17772  -2604   -611
-2898       C
ATOM  11627  CG2 VAL B 695      17.479 -13.607  91.901  1.00203.83
C
ANISOU11627  CG2 VAL B 695    25604  30126  21716  -2889   -664
-2669       C
```

ATOM 11628 N   LYS B 696   16.788 -11.152 87.899 1.00137.51
N
ANISOU11628 N   LYS B 696   17757 20395 14097 -2017   -534
-2952   N
ATOM 11629 CA LYS B 696   16.833 -10.986 86.451 1.00111.39
C
ANISOU11629 CA LYS B 696   14641 16667 11014 -1843   -523
-2833   C
ATOM 11630 C   LYS B 696   18.001 -11.765 85.843 1.00114.08
C
ANISOU11630 C   LYS B 696   15087 16776 11484 -1978   -542
-2520   C
ATOM 11631 O   LYS B 696   19.068 -11.871 86.458 1.00183.36
O
ANISOU11631 O   LYS B 696   23846 25567 20255 -2148   -562
-2471   O
ATOM 11632 CB LYS B 696   16.952 -9.499 86.107 1.00118.86
C
ANISOU11632 CB LYS B 696   15750 17283 12128 -1642   -515
-3075   C
ATOM 11633 CG LYS B 696   18.313 -8.899 86.438 1.00128.22
C
ANISOU11633 CG LYS B 696   17042 18248 13426 -1781   -525
-3098   C
ATOM 11634 CD LYS B 696   18.322 -7.385 86.317 1.00138.19
C
ANISOU11634 CD LYS B 696   18476 19201 14828 -1620   -544
-3369   C
ATOM 11635 CE LYS B 696   19.742 -6.844 86.411 1.00168.20
C
ANISOU11635 CE LYS B 696   22408 22741 18758 -1804   -555
-3338   C
ATOM 11636 NZ LYS B 696   19.779 -5.357 86.461 1.00193.17
N
ANISOU11636 NZ LYS B 696   25759 25589 22047 -1696   -600
-3616   N
ATOM 11637 N   PRO B 697   17.819 -12.351 84.656 1.00114.51
N
ANISOU11637 N   PRO B 697   15226 16647 11637 -1897   -542
-2321   N
ATOM 11638 CA PRO B 697   18.951 -12.958 83.944 1.00113.02
C
ANISOU11638 CA PRO B 697   15135 16226 11582 -1968   -560
-2076   C
ATOM 11639 C   PRO B 697   20.074 -11.975 83.642 1.00105.25
C
ANISOU11639 C   PRO B 697   14278 14955 10759 -1966   -534
-2127   C
ATOM 11640 O   PRO B 697   19.851 -10.783 83.418 1.00115.89
O
ANISOU11640 O   PRO B 697   15726 16121 12187 -1849   -507
-2302   O
ATOM 11641 CB PRO B 697   18.310 -13.483 82.654 1.00114.10
C

```
ANISOU11641  CB  PRO B 697    15339  16229  11785  -1829   -556
-1939       C
ATOM  11642  CG  PRO B 697      17.180 -12.546  82.407  1.00114.58
C
ANISOU11642  CG  PRO B 697    15414  16283  11838  -1630   -520
-2143       C
ATOM  11643  CD  PRO B 697      16.636 -12.222  83.786  1.00114.68
C
ANISOU11643  CD  PRO B 697    15271  16631  11672  -1689   -523
-2362       C
ATOM  11644  N   SER B 698      21.295 -12.501  83.640  1.00 68.20
N
ANISOU11644  N   SER B 698     9580  10226   6108  -2101   -557
-1970       N
ATOM  11645  CA  SER B 698      22.435 -11.785  83.082  1.00113.40
C
ANISOU11645  CA  SER B 698    15410  15701  11977  -2131   -528
-1956       C
ATOM  11646  C   SER B 698      22.239 -11.594  81.579  1.00105.95
C
ANISOU11646  C   SER B 698    14604  14488  11163  -1981   -494
-1859       C
ATOM  11647  O   SER B 698      21.904 -12.559  80.878  1.00 93.86
O
ANISOU11647  O   SER B 698    13058  12979   9627  -1908   -507
-1706       O
ATOM  11648  CB  SER B 698      23.731 -12.547  83.354  1.00134.64
C
ANISOU11648  CB  SER B 698    18020  18484  14655  -2283   -567
-1809       C
ATOM  11649  OG  SER B 698      24.855 -11.846  82.851  1.00117.61
O
ANISOU11649  OG  SER B 698    15926  16153  12606  -2346   -533
-1804       O
ATOM  11650  N   PRO B 699      22.427 -10.381  81.045  1.00103.00
N
ANISOU11650  N   PRO B 699    14379  13857  10900  -1943   -461
-1936       N
ATOM  11651  CA  PRO B 699      22.263 -10.192  79.595  1.00100.81
C
ANISOU11651  CA  PRO B 699    14234  13347  10722  -1818   -435
-1816       C
ATOM  11652  C   PRO B 699      23.426 -10.774  78.804  1.00123.17
C
ANISOU11652  C   PRO B 699    17047  16158  13595  -1909   -417
-1619       C
ATOM  11653  O   PRO B 699      23.220 -11.527  77.847  1.00111.73
O
ANISOU11653  O   PRO B 699    15592  14708  12153  -1810   -411
-1482       O
ATOM  11654  CB  PRO B 699      22.192  -8.666  79.441  1.00 69.99
C
ANISOU11654  CB  PRO B 699    10514   9166   6913  -1787   -435
-1951       C
```

```
ATOM  11655  CG   PRO B 699      22.874  -8.124  80.653  1.00 71.39
C
ANISOU11655  CG   PRO B 699    10654   9403   7067  -1962   -451
-2108       C
ATOM  11656  CD   PRO B 699      22.608  -9.106  81.759  1.00103.93
C
ANISOU11656  CD   PRO B 699    14566  13878  11044  -2001   -465
-2150       C
ATOM  11657  N    THR B 700      24.651 -10.435  79.198  1.00 86.81
N
ANISOU11657  N    THR B 700    12414  11566   9004  -2094   -410
-1624       N
ATOM  11658  CA   THR B 700      25.823 -11.073  78.624  1.00 67.44
C
ANISOU11658  CA   THR B 700     9881   9188   6555  -2176   -398
-1472       C
ATOM  11659  C    THR B 700      25.880 -12.538  79.063  1.00 83.52
C
ANISOU11659  C    THR B 700    11762  11455   8518  -2138   -455
-1402       C
ATOM  11660  O    THR B 700      25.426 -12.880  80.159  1.00128.68
O
ANISOU11660  O    THR B 700    17415  17312  14163  -2161   -501
-1468       O
ATOM  11661  CB   THR B 700      27.096 -10.333  79.044  1.00 85.37
C
ANISOU11661  CB   THR B 700    12131  11470   8837  -2401   -382
-1513       C
ATOM  11662  OG1  THR B 700      28.214 -10.806  78.279  1.00 98.89
O
ANISOU11662  OG1  THR B 700    13753  13279  10543  -2462   -359
-1378       O
ATOM  11663  CG2  THR B 700      27.380 -10.519  80.530  1.00105.77
C
ANISOU11663  CG2  THR B 700    14589  14254  11345  -2510   -427
-1630       C
ATOM  11664  N    PRO B 701      26.374 -13.439  78.198  1.00 83.56
N
ANISOU11664  N    PRO B 701    11715  11501   8532  -2071   -466
-1269       N
ATOM  11665  CA   PRO B 701      26.794 -14.760  78.688  1.00110.90
C
ANISOU11665  CA   PRO B 701    15050  15146  11943  -2055   -554
-1205       C
ATOM  11666  C    PRO B 701      27.617 -14.678  79.966  1.00132.74
C
ANISOU11666  C    PRO B 701    17707  18077  14651  -2213   -595
-1261       C
ATOM  11667  O    PRO B 701      28.526 -13.850  80.085  1.00114.62
O
ANISOU11667  O    PRO B 701    15384  15798  12367  -2347   -550
-1314       O
ATOM  11668  CB   PRO B 701      27.602 -15.334  77.514  1.00 93.79
C
```

```
ANISOU11668  CB  PRO B 701    12838  12991   9806  -1972   -550
-1112       C
ATOM  11669  CG  PRO B 701      27.997 -14.145  76.695  1.00115.63
C
ANISOU11669  CG  PRO B 701    15667  15655  12611  -2045   -445
-1120       C
ATOM  11670  CD  PRO B 701      26.834 -13.200  76.821  1.00 86.46
C
ANISOU11670  CD  PRO B 701    12128  11775   8949  -2035   -409
-1183       C
ATOM  11671  N   ASP B 702      27.297 -15.544  80.924  1.00133.44
N
ANISOU11671  N   ASP B 702    17737  18301  14665  -2219   -689
-1237       N
ATOM  11672  CA  ASP B 702      27.883 -15.531  82.258  1.00121.34
C
ANISOU11672  CA  ASP B 702    16100  16956  13048  -2364   -743
-1283       C
ATOM  11673  C   ASP B 702      28.492 -16.899  82.535  1.00100.03
C
ANISOU11673  C   ASP B 702    13313  14386  10307  -2318   -879
-1154       C
ATOM  11674  O   ASP B 702      27.838 -17.924  82.315  1.00116.41
O
ANISOU11674  O   ASP B 702    15436  16417  12377  -2219   -962
-1050       O
ATOM  11675  CB  ASP B 702      26.822 -15.174  83.306  1.00101.88
C
ANISOU11675  CB  ASP B 702    13651  14563  10495  -2432   -742
-1380       C
ATOM  11676  CG  ASP B 702      27.409 -14.925  84.682  1.00132.93
C
ANISOU11676  CG  ASP B 702    17474  18708  14324  -2599   -780
-1460       C
ATOM  11677  OD1 ASP B 702      26.673 -14.405  85.547  1.00133.73
O
ANISOU11677  OD1 ASP B 702    17567  18902  14341  -2665   -761
-1589       O
ATOM  11678  OD2 ASP B 702      28.602 -15.225  84.901  1.00176.07
O
ANISOU11678  OD2 ASP B 702    22845  24277  19778  -2657   -829
-1412       O
ATOM  11679  N   SER B 703      29.736 -16.924  83.015  1.00135.24
N
ANISOU11679  N   SER B 703    17651  18999  14737  -2389   -921
-1163       N
ATOM  11680  CA  SER B 703      30.490 -18.170  83.050  1.00142.16
C
ANISOU11680  CA  SER B 703    18447  19969  15596  -2286  -1066
-1052       C
ATOM  11681  C   SER B 703      30.756 -18.685  84.459  1.00137.67
C
ANISOU11681  C   SER B 703    17802  19594  14912  -2380  -1200
-1009       C
```

```
ATOM  11682  O   SER B 703      31.387 -19.738  84.602  1.00141.72
O
ANISOU11682  O   SER B 703    18266  20170  15410  -2281  -1356
-908       O
ATOM  11683  CB  SER B 703      31.822 -17.998  82.310  1.00171.05
C
ANISOU11683  CB  SER B 703    21991  23702  19296  -2239  -1034
-1084       C
ATOM  11684  OG  SER B 703      31.616 -17.688  80.942  1.00175.40
O
ANISOU11684  OG  SER B 703    22607  24105  19930  -2151   -929
-1092       O
ATOM  11685  N   TRP B 704      30.293 -17.984  85.495  1.00166.40
N
ANISOU11685  N   TRP B 704    20190  20790  22245  -4847  -2566
-362       N
ATOM  11686  CA  TRP B 704      30.486 -18.398  86.889  1.00139.81
C
ANISOU11686  CA  TRP B 704    16820  17362  18941  -4789  -2601
-312       C
ATOM  11687  C   TRP B 704      31.952 -18.684  87.214  1.00 83.43
C
ANISOU11687  C   TRP B 704     9619  10183  11896  -4788  -2592
-437       C
ATOM  11688  O   TRP B 704      32.310 -19.760  87.698  1.00 98.32
O
ANISOU11688  O   TRP B 704    11405  12014  13936  -4767  -2604
-504       O
ATOM  11689  CB  TRP B 704      29.591 -19.599  87.193  1.00135.14
C
ANISOU11689  CB  TRP B 704    16148  16734  18466  -4762  -2624
-286       C
ATOM  11690  CG  TRP B 704      29.487 -20.050  88.612  1.00121.53
C
ANISOU11690  CG  TRP B 704    14434  14959  16784  -4716  -2675
-186       C
ATOM  11691  CD1 TRP B 704      29.797 -19.341  89.733  1.00102.81
C
ANISOU11691  CD1 TRP B 704    12160  12578  14324  -4693  -2709
-89       C
ATOM  11692  CD2 TRP B 704      28.913 -21.282  89.064  1.00 99.06
C
ANISOU11692  CD2 TRP B 704    11505  12068  14064  -4698  -2709
-154       C
ATOM  11693  NE1 TRP B 704      29.511 -20.082  90.855  1.00125.38
N
ANISOU11693  NE1 TRP B 704    14999  15394  17244  -4665  -2761
-1       N
ATOM  11694  CE2 TRP B 704      28.961 -21.276  90.470  1.00108.59
C
ANISOU11694  CE2 TRP B 704    12765  13246  15250  -4668  -2766
-29       C
ATOM  11695  CE3 TRP B 704      28.387 -22.402  88.412  1.00104.96
C
```

```
ANISOU11695  CE3 TRP B 704    12139  12798  14944  -4711  -2704
-218       C
ATOM  11696  CZ2 TRP B 704      28.504 -22.345  91.238  1.00130.25
C
ANISOU11696  CZ2 TRP B 704    15453  15944  18094  -4653  -2821
52       C
ATOM  11697  CZ3 TRP B 704      27.933 -23.462  89.175  1.00 98.16
C
ANISOU11697  CZ3 TRP B 704    11221  11885  14189  -4692  -2759
-142       C
ATOM  11698  CH2 TRP B 704      27.992 -23.425  90.573  1.00113.63
C
ANISOU11698  CH2 TRP B 704    13237  13817  16120  -4664  -2819
1       C
ATOM  11699  N   LYS B 705      32.812 -17.709  86.938  1.00 99.72
N
ANISOU11699  N   LYS B 705    11741  12275  13875  -4815  -2576
-465       N
ATOM  11700  CA  LYS B 705      34.194 -17.803  87.389  1.00118.39
C
ANISOU11700  CA  LYS B 705    14062  14601  16319  -4812  -2577
-560       C
ATOM  11701  C   LYS B 705      34.264 -17.577  88.896  1.00108.84
C
ANISOU11701  C   LYS B 705    12934  13348  15073  -4765  -2636
-435       C
ATOM  11702  O   LYS B 705      33.914 -16.503  89.397  1.00104.20
O
ANISOU11702  O   LYS B 705    12480  12779  14333  -4757  -2663
-304       O
ATOM  11703  CB  LYS B 705      35.074 -16.809  86.633  1.00130.26
C
ANISOU11703  CB  LYS B 705    15603  16148  17741  -4863  -2549
-616       C
ATOM  11704  CG  LYS B 705      34.539 -15.392  86.578  1.00 77.95
C
ANISOU11704  CG  LYS B 705     9139   9574  10903  -4882  -2565
-463       C
ATOM  11705  CD  LYS B 705      34.014 -15.068  85.184  1.00137.70
C
ANISOU11705  CD  LYS B 705    16715  17215  18388  -4947  -2531
-472       C
ATOM  11706  CE  LYS B 705      35.137 -15.049  84.154  1.00102.07
C
ANISOU11706  CE  LYS B 705    12133  12740  13910  -5017  -2489
-619       C
ATOM  11707  NZ  LYS B 705      36.222 -14.095  84.515  1.00 90.90
N
ANISOU11707  NZ  LYS B 705    10783  11323  12434  -5034  -2502
-605       N
ATOM  11708  N   LEU B 706      34.709 -18.613  89.610  1.00 83.94
N
ANISOU11708  N   LEU B 706     9695  10134  12063  -4742  -2662
-479       N
```

```
ATOM  11709  CA  LEU B 706      34.712 -18.605  91.070  1.00 77.80
C
ANISOU11709  CA  LEU B 706     8987   9315  11258  -4707  -2732
-349       C
ATOM  11710  C   LEU B 706      35.569 -17.477  91.624  1.00 77.50
C
ANISOU11710  C   LEU B 706     9053   9276  11119  -4711  -2756
-315       C
ATOM  11711  O   LEU B 706      35.224 -16.865  92.640  1.00 76.83
O
ANISOU11711  O   LEU B 706     9081   9185  10925  -4692  -2806
-179       O
ATOM  11712  CB  LEU B 706      35.203 -19.953  91.594  1.00 78.69
C
ANISOU11712  CB  LEU B 706     8984   9364  11553  -4698  -2763
-407       C
ATOM  11713  CG  LEU B 706      34.314 -21.151  91.260  1.00 96.59
C
ANISOU11713  CG  LEU B 706    11152  11618  13930  -4695  -2758
-420       C
ATOM  11714  CD1 LEU B 706      35.000 -22.441  91.653  1.00 93.36
C
ANISOU11714  CD1 LEU B 706    10620  11141  13712  -4699  -2790
-496       C
ATOM  11715  CD2 LEU B 706      32.962 -21.033  91.952  1.00 81.05
C
ANISOU11715  CD2 LEU B 706     9271   9662  11862  -4670  -2803
-223       C
ATOM  11716  N   LYS B 707      36.707 -17.214  90.983  1.00111.57
N
ANISOU11716  N   LYS B 707    13322  13594  15477  -4740  -2723
-445       N
ATOM  11717  CA  LYS B 707      37.641 -16.205  91.473  1.00128.85
C
ANISOU11717  CA  LYS B 707    15596  15773  17589  -4748  -2752
-422       C
ATOM  11718  C   LYS B 707      36.992 -14.827  91.583  1.00117.68
C
ANISOU11718  C   LYS B 707    14337  14397  15978  -4750  -2765
-292       C
ATOM  11719  O   LYS B 707      37.202 -14.116  92.572  1.00105.90
O
ANISOU11719  O   LYS B 707    12947  12882  14407  -4737  -2819
-205       O
ATOM  11720  CB  LYS B 707      38.871 -16.144  90.559  1.00105.65
C
ANISOU11720  CB  LYS B 707    12566  12840  14735  -4789  -2706
-590       C
ATOM  11721  CG  LYS B 707      39.790 -17.380  90.595  1.00 80.31
C
ANISOU11721  CG  LYS B 707     9192   9579  11743  -4792  -2697
-749       C
ATOM  11722  CD  LYS B 707      39.169 -18.591  89.889  1.00108.68
C
```

```
ANISOU11722  CD  LYS B 707    12651  13175  15467  -4793  -2651
-850       C
ATOM  11723  CE  LYS B 707      40.190 -19.669  89.568  1.00104.95
C
ANISOU11723  CE  LYS B 707    11992  12664  15222  -4816  -2617
-1066       C
ATOM  11724  NZ  LYS B 707      40.738 -20.293  90.801  1.00126.95
N
ANISOU11724  NZ  LYS B 707    14767  15383  18087  -4799  -2685
-1021       N
ATOM  11725  N   GLU B 708      36.210 -14.425  90.578  1.00126.39
N
ANISOU11725  N   GLU B 708    15459  15557  17007  -4772  -2721
-282       N
ATOM  11726  CA  GLU B 708      35.459 -13.174  90.681  1.00 98.28
C
ANISOU11726  CA  GLU B 708    12036  12027  13280  -4775  -2739
-158       C
ATOM  11727  C   GLU B 708      34.457 -13.167  91.835  1.00105.02
C
ANISOU11727  C   GLU B 708    12955  12861  14087  -4732  -2782
-34       C
ATOM  11728  O   GLU B 708      34.365 -12.176  92.569  1.00118.60
O
ANISOU11728  O   GLU B 708    14783  14570  15709  -4724  -2820
42       O
ATOM  11729  CB  GLU B 708      34.745 -12.873  89.361  1.00 95.30
C
ANISOU11729  CB  GLU B 708    11657  11712  12842  -4813  -2693
-162       C
ATOM  11730  CG  GLU B 708      35.659 -12.349  88.260  1.00128.18
C
ANISOU11730  CG  GLU B 708    15807  15914  16982  -4877  -2660
-245       C
ATOM  11731  CD  GLU B 708      34.903 -11.974  86.995  1.00 87.09
C
ANISOU11731  CD  GLU B 708    10622  10780  11690  -4930  -2628
-222       C
ATOM  11732  OE1 GLU B 708      33.842 -11.324  87.103  1.00 76.32
O
ANISOU11732  OE1 GLU B 708     9345   9427  10225  -4922  -2652
-102       O
ATOM  11733  OE2 GLU B 708      35.381 -12.307  85.890  1.00 98.18
O
ANISOU11733  OE2 GLU B 708    11951  12226  13127  -4987  -2583
-326       O
ATOM  11734  N   ILE B 709      33.693 -14.245  92.017  1.00 75.26
N
ANISOU11734  N   ILE B 709     9118   9086  10392  -4709  -2779
-17       N
ATOM  11735  CA  ILE B 709      32.700 -14.231  93.092  1.00109.12
C
ANISOU11735  CA  ILE B 709    13461  13366  14633  -4680  -2818
104       C
```

```
ATOM  11736  C   ILE B 709      33.312 -14.454  94.473  1.00 74.80
C
ANISOU11736  C   ILE B 709     9141   8975  10305  -4665  -2877
140       C
ATOM  11737  O   ILE B 709      32.806 -13.919  95.467  1.00 74.39
O
ANISOU11737  O   ILE B 709     9172   8923  10169  -4658  -2913
229       O
ATOM  11738  CB  ILE B 709      31.582 -15.252  92.817  1.00 88.66
C
ANISOU11738  CB  ILE B 709    10791  10786  12107  -4668  -2803
131       C
ATOM  11739  CG1 ILE B 709      32.156 -16.644  92.578  1.00 92.95
C
ANISOU11739  CG1 ILE B 709    11206  11297  12814  -4667  -2797
40       C
ATOM  11740  CG2 ILE B 709      30.734 -14.804  91.639  1.00109.78
C
ANISOU11740  CG2 ILE B 709    13472  13508  14730  -4685  -2761
132       C
ATOM  11741  CD1 ILE B 709      31.106 -17.701  92.409  1.00 75.52
C
ANISOU11741  CD1 ILE B 709     8919   9089  10686  -4657  -2796
72       C
ATOM  11742  N   PHE B 710      34.387 -15.235  94.574  1.00 96.68
N
ANISOU11742  N   PHE B 710    11839  11707  13189  -4668  -2892
68       N
ATOM  11743  CA  PHE B 710      35.107 -15.327  95.841  1.00 75.80
C
ANISOU11743  CA  PHE B 710     9230   9017  10552  -4665  -2962
109       C
ATOM  11744  C   PHE B 710      35.669 -13.974  96.261  1.00 80.24
C
ANISOU11744  C   PHE B 710     9911   9576  10999  -4675  -2987
125       C
ATOM  11745  O   PHE B 710      35.545 -13.571  97.423  1.00 95.46
O
ANISOU11745  O   PHE B 710    11924  11491  12855  -4674  -3042
204       O
ATOM  11746  CB  PHE B 710      36.225 -16.366  95.738  1.00 76.76
C
ANISOU11746  CB  PHE B 710     9240   9090  10835  -4669  -2977
16       C
ATOM  11747  CG  PHE B 710      35.735 -17.785  95.607  1.00 77.18
C
ANISOU11747  CG  PHE B 710     9178   9126  11022  -4661  -2977
8       C
ATOM  11748  CD1 PHE B 710      36.631 -18.820  95.398  1.00 98.06
C
ANISOU11748  CD1 PHE B 710    11698  11721  13839  -4667  -2985
-96       C
ATOM  11749  CD2 PHE B 710      34.387 -18.089  95.709  1.00 76.69
C
```

```
ANISOU11749  CD2 PHE B 710     9123   9091  10926  -4650  -2974
100       C
ATOM  11750  CE1 PHE B 710      36.194 -20.129  95.277  1.00 78.77
C
ANISOU11750  CE1 PHE B 710     9146   9252  11532  -4663  -2993
-107       C
ATOM  11751  CE2 PHE B 710      33.946 -19.396  95.590  1.00109.84
C
ANISOU11751  CE2 PHE B 710    13214  13266  15256  -4645  -2984
101       C
ATOM  11752  CZ  PHE B 710      34.853 -20.417  95.377  1.00 78.27
C
ANISOU11752  CZ  PHE B 710     9097   9213  11429  -4651  -2996
-2       C
ATOM  11753  N   ALA B 711      36.290 -13.257  95.322  1.00107.49
N
ANISOU11753  N   ALA B 711    13367  13041  14434  -4691  -2950
49       N
ATOM  11754  CA  ALA B 711      36.738 -11.889  95.577  1.00110.10
C
ANISOU11754  CA  ALA B 711    13809  13365  14659  -4704  -2976
69       C
ATOM  11755  C   ALA B 711      35.583 -10.956  95.929  1.00105.44
C
ANISOU11755  C   ALA B 711    13320  12798  13945  -4697  -2982
153       C
ATOM  11756  O   ALA B 711      35.696 -10.139  96.849  1.00103.76
O
ANISOU11756  O   ALA B 711    13202  12562  13661  -4698  -3030
192       O
ATOM  11757  CB  ALA B 711      37.501 -11.355  94.366  1.00114.22
C
ANISOU11757  CB  ALA B 711    14305  13904  15187  -4735  -2935
-17       C
ATOM  11758  N   THR B 712      34.472 -11.046  95.196  1.00 74.46
N
ANISOU11758  N   THR B 712     9371   8916  10004  -4693  -2934
172       N
ATOM  11759  CA  THR B 712      33.296 -10.238  95.513  1.00 81.15
C
ANISOU11759  CA  THR B 712    10292   9781  10762  -4685  -2938
241       C
ATOM  11760  C   THR B 712      32.765 -10.511  96.917  1.00140.12
C
ANISOU11760  C   THR B 712    17789  17239  18213  -4672  -2975
300       C
ATOM  11761  O   THR B 712      32.417  -9.576  97.648  1.00141.42
O
ANISOU11761  O   THR B 712    18035  17395  18302  -4674  -3000
323       O
ATOM  11762  CB  THR B 712      32.199 -10.484  94.477  1.00106.48
C
ANISOU11762  CB  THR B 712    13451  13032  13976  -4685  -2888
254       C
```

ATOM 11763 OG1 THR B 712   32.697 -10.172 93.170 1.00106.27
O
ANISOU11763 OG1 THR B 712   13406 13025 13945 -4714 -2857
202   O
ATOM 11764 CG2 THR B 712   30.978 -9.624 94.772 1.00119.92
C
ANISOU11764 CG2 THR B 712   15213 14745 15606 -4677 -2894
315   C
ATOM 11765 N   GLY B 713   32.689 -11.781 97.309 1.00132.77
N
ANISOU11765 N   GLY B 713   16787 16307 17354 -4665 -2982
321   N
ATOM 11766 CA GLY B 713   32.185 -12.113 98.634 1.00 94.58
C
ANISOU11766 CA GLY B 713   11972 11470 12492 -4669 -3022
391   C
ATOM 11767 C   GLY B 713   33.100 -11.720 99.781 1.00 89.12
C
ANISOU11767 C   GLY B 713   11357 10746 11760 -4686 -3086
396   C
ATOM 11768 O   GLY B 713   32.637 -11.194 100.797 1.00108.18
O
ANISOU11768 O   GLY B 713   13840 13169 14094 -4702 -3112
429   O
ATOM 11769 N   VAL B 714   34.403 -11.980 99.646 1.00 89.39
N
ANISOU11769 N   VAL B 714   11374 10739 11851 -4689 -3115
353   N
ATOM 11770 CA VAL B 714   35.349 -11.659 100.716 1.00 75.41
C
ANISOU11770 CA VAL B 714   9674   8928 10050 -4708 -3190
363   C
ATOM 11771 C   VAL B 714   35.384 -10.162 101.021 1.00 75.31
C
ANISOU11771 C   VAL B 714   9774   8908   9932 -4716 -3203
342   C
ATOM 11772 O   VAL B 714   35.385 -9.760 102.191 1.00107.20
O
ANISOU11772 O   VAL B 714   13892 12937 13903 -4737 -3256
367   O
ATOM 11773 CB VAL B 714   36.751 -12.211 100.384 1.00128.79
C
ANISOU11773 CB VAL B 714   16377 15641 16915 -4708 -3217
311   C
ATOM 11774 CG1 VAL B 714   37.326 -11.572 99.134 1.00128.96
C
ANISOU11774 CG1 VAL B 714   16379 15663 16956 -4703 -3167
224   C
ATOM 11775 CG2 VAL B 714   37.700 -11.995 101.554 1.00104.43
C
ANISOU11775 CG2 VAL B 714   13363 12509 13808 -4731 -3310
338   C
ATOM 11776 N   VAL B 715   35.421 -9.312 99.994 1.00 75.05
N

```
ANISOU11776  N   VAL B 715     9751   8877   9886  -4706  -3163
295       N
ATOM  11777  CA  VAL B 715      35.408  -7.873 100.250  1.00107.67
C
ANISOU11777  CA  VAL B 715    13984  12989  13938  -4712  -3187
275       C
ATOM  11778  C   VAL B 715      34.062  -7.416 100.808  1.00123.07
C
ANISOU11778  C   VAL B 715    15969  14967  15826  -4711  -3171
294       C
ATOM  11779  O   VAL B 715      34.009  -6.560 101.700  1.00156.34
O
ANISOU11779  O   VAL B 715    20262  19158  19980  -4724  -3211
272       O
ATOM  11780  CB  VAL B 715      35.803  -7.093  98.979  1.00 92.39
C
ANISOU11780  CB  VAL B 715    12047  11047  12010  -4713  -3160
235       C
ATOM  11781  CG1 VAL B 715      37.154  -7.577  98.461  1.00125.64
C
ANISOU11781  CG1 VAL B 715    16207  15240  16293  -4723  -3167
197       C
ATOM  11782  CG2 VAL B 715      34.741  -7.218  97.904  1.00 74.51
C
ANISOU11782  CG2 VAL B 715     9730   8830   9750  -4703  -3092
246       C
ATOM  11783  N   LEU B 716      32.956  -7.963 100.296  1.00 74.20
N
ANISOU11783  N   LEU B 716     9713   8823   9657  -4699  -3114
321       N
ATOM  11784  CA  LEU B 716      31.642  -7.624 100.837  1.00 99.34
C
ANISOU11784  CA  LEU B 716    12909  12037  12800  -4701  -3096
332       C
ATOM  11785  C   LEU B 716      31.490  -8.049 102.297  1.00138.45
C
ANISOU11785  C   LEU B 716    17885  17006  17715  -4729  -3130
356       C
ATOM  11786  O   LEU B 716      30.948  -7.297 103.115  1.00148.20
O
ANISOU11786  O   LEU B 716    19170  18247  18893  -4747  -3140
322       O
ATOM  11787  CB  LEU B 716      30.547  -8.259  99.979  1.00 84.81
C
ANISOU11787  CB  LEU B 716    10982  10239  11001  -4685  -3035
366       C
ATOM  11788  CG  LEU B 716      29.111  -7.859 100.322  1.00111.48
C
ANISOU11788  CG  LEU B 716    14351  13648  14359  -4687  -3009
371       C
ATOM  11789  CD1 LEU B 716      28.906  -6.367 100.096  1.00128.02
C
ANISOU11789  CD1 LEU B 716    16504  15709  16428  -4681  -3018
312       C
```

ATOM 11790 CD2 LEU B 716   28.118 -8.668 99.504 1.00134.85
C
ANISOU11790 CD2 LEU B 716   17219 16646 17371 -4674 -2961
418   C
ATOM 11791 N   GLY B 717   31.965 -9.247 102.643 1.00 99.14
N
ANISOU11791 N   GLY B 717   12867 12033 12769 -4741 -3153
410   N
ATOM 11792 CA GLY B 717   31.958 -9.676 104.034 1.00 85.13
C
ANISOU11792 CA GLY B 717   11122 10276 10949 -4784 -3202
452   C
ATOM 11793 C   GLY B 717   33.005 -9.012 104.907 1.00 94.37
C
ANISOU11793 C   GLY B 717   12390 11402 12063 -4810 -3274
419   C
ATOM 11794 O   GLY B 717   32.767 -8.777 106.095 1.00 83.22
O
ANISOU11794 O   GLY B 717   11034 10011 10573 -4854 -3308
418   O
ATOM 11795 N   GLY B 718   34.174 -8.712 104.344 1.00 75.92
N
ANISOU11795 N   GLY B 718   10071   9009   9765 -4789 -3300
387   N
ATOM 11796 CA GLY B 718   35.210 -8.039 105.112 1.00 76.68
C
ANISOU11796 CA GLY B 718   10260   9054   9820 -4812 -3378
357   C
ATOM 11797 C   GLY B 718   34.825 -6.647 105.578 1.00 90.05
C
ANISOU11797 C   GLY B 718   12042 10738 11437 -4819 -3386
282   C
ATOM 11798 O   GLY B 718   35.174 -6.237 106.687 1.00141.92
O
ANISOU11798 O   GLY B 718   18692 17290 17942 -4855 -3451
258   O
ATOM 11799 N   TYR B 719   34.108 -5.900 104.738 1.00 76.35
N
ANISOU11799 N   TYR B 719   10290   9007   9713 -4788 -3330
238   N
ATOM 11800 CA TYR B 719   33.509 -4.642 105.178 1.00 99.28
C
ANISOU11800 CA TYR B 719   13255 11898 12570 -4791 -3336
152   C
ATOM 11801 C   TYR B 719   32.587 -4.826 106.379 1.00129.96
C
ANISOU11801 C   TYR B 719   17149 15839 16393 -4830 -3331
132   C
ATOM 11802 O   TYR B 719   32.640 -4.048 107.338 1.00147.50
O
ANISOU11802 O   TYR B 719   19443 18045 18555 -4855 -3377
49   O
ATOM 11803 CB TYR B 719   32.735 -4.011 104.018 1.00 90.03
C

```
ANISOU11803  CB  TYR B 719    12042  10725  11440  -4755  -3280
128       C
ATOM  11804  CG  TYR B 719      31.772  -2.923 104.441  1.00 76.33
C
ANISOU11804  CG  TYR B 719    10333   8982   9688  -4754  -3277
34       C
ATOM  11805  CD1 TYR B 719      32.213  -1.632 104.701  1.00 77.72
C
ANISOU11805  CD1 TYR B 719    10586   9089   9857  -4750  -3338
-56       C
ATOM  11806  CD2 TYR B 719      30.420  -3.198 104.602  1.00 76.00
C
ANISOU11806  CD2 TYR B 719    10229   8998   9650  -4757  -3220
26       C
ATOM  11807  CE1 TYR B 719      31.324  -0.634 105.057  1.00 84.39
C
ANISOU11807  CE1 TYR B 719    11440   9917  10709  -4744  -3341
-168       C
ATOM  11808  CE2 TYR B 719      29.532  -2.217 104.990  1.00 76.41
C
ANISOU11808  CE2 TYR B 719    10285   9039   9707  -4755  -3218
-83       C
ATOM  11809  CZ  TYR B 719      29.985  -0.936 105.207  1.00 91.48
C
ANISOU11809  CZ  TYR B 719    12267  10875  11619  -4746  -3279
-188       C
ATOM  11810  OH  TYR B 719      29.100   0.027 105.631  1.00115.14
O
ANISOU11810  OH  TYR B 719    15256  13854  14638  -4740  -3283
-324       O
ATOM  11811  N   GLN B 720      31.731  -5.846 106.346  1.00100.62
N
ANISOU11811  N   GLN B 720    13353  12190  12686  -4839  -3279
200       N
ATOM  11812  CA  GLN B 720      30.922  -6.172 107.519  1.00132.98
C
ANISOU11812  CA  GLN B 720    17449  16358  16719  -4893  -3276
198       C
ATOM  11813  C   GLN B 720      31.751  -6.556 108.739  1.00105.88
C
ANISOU11813  C   GLN B 720    14086  12929  13213  -4953  -3355
227       C
ATOM  11814  O   GLN B 720      31.405  -6.185 109.867  1.00 78.63
O
ANISOU11814  O   GLN B 720    10683   9518   9675  -5006  -3378
167       O
ATOM  11815  CB  GLN B 720      29.918  -7.271 107.185  1.00170.55
C
ANISOU11815  CB  GLN B 720    22103  21185  21513  -4896  -3214
287       C
ATOM  11816  CG  GLN B 720      28.691  -6.725 106.514  1.00174.18
C
ANISOU11816  CG  GLN B 720    22504  21662  22014  -4865  -3143
236       C
```

```
ATOM  11817  CD  GLN B 720      28.084  -5.619 107.351  1.00207.84
C
ANISOU11817  CD  GLN B 720    26804  25936  26231  -4888  -3145
105       C
ATOM  11818  OE1 GLN B 720      27.548  -5.860 108.433  1.00229.33
O
ANISOU11818  OE1 GLN B 720    29518  28726  28889  -4946  -3144
89       O
ATOM  11819  NE2 GLN B 720      28.161  -4.395 106.848  1.00199.97
N
ANISOU11819  NE2 GLN B 720    25840  24873  25266  -4846  -3153
4       N
ATOM  11820  N   ALA B 721      32.837  -7.305 108.545  1.00114.64
N
ANISOU11820  N   ALA B 721    15197  14002  14359  -4950  -3402
312       N
ATOM  11821  CA  ALA B 721      33.752  -7.572 109.652  1.00110.79
C
ANISOU11821  CA  ALA B 721    14782  13500  13812  -5007  -3497
345       C
ATOM  11822  C   ALA B 721      34.317  -6.290 110.255  1.00 79.75
C
ANISOU11822  C   ALA B 721    10962   9520   9819  -5017  -3554
231       C
ATOM  11823  O   ALA B 721      34.370  -6.145 111.481  1.00 96.61
O
ANISOU11823  O   ALA B 721    13169  11682  11856  -5081  -3611
205       O
ATOM  11824  CB  ALA B 721      34.882  -8.487 109.180  1.00 78.90
C
ANISOU11824  CB  ALA B 721    10709   9408   9859  -4989  -3543
434       C
ATOM  11825  N   ILE B 722      34.748  -5.350 109.412  1.00 79.47
N
ANISOU11825  N   ILE B 722    10944   9414   9837  -4959  -3548
162       N
ATOM  11826  CA  ILE B 722      35.195  -4.046 109.901  1.00 80.35
C
ANISOU11826  CA  ILE B 722    11154   9469   9906  -4960  -3608
45       C
ATOM  11827  C   ILE B 722      34.076  -3.301 110.627  1.00 80.81
C
ANISOU11827  C   ILE B 722    11235   9574   9896  -4980  -3584
-81       C
ATOM  11828  O   ILE B 722      34.272  -2.783 111.733  1.00 87.08
O
ANISOU11828  O   ILE B 722    12113  10369  10606  -5021  -3650
-169       O
ATOM  11829  CB  ILE B 722      35.758  -3.209 108.739  1.00 87.11
C
ANISOU11829  CB  ILE B 722    12009  10246  10843  -4900  -3604
13       C
ATOM  11830  CG1 ILE B 722      36.954  -3.919 108.102  1.00 86.02
C
```

```
ANISOU11830  CG1 ILE B 722    11842  10070  10772  -4888  -3631
108       C
ATOM  11831  CG2 ILE B 722      36.139  -1.810 109.216  1.00105.46
C
ANISOU11831  CG2 ILE B 722    14430  12503  13139  -4898  -3675
-110       C
ATOM  11832  CD1 ILE B 722      37.392  -3.316 106.788  1.00115.10
C
ANISOU11832  CD1 ILE B 722    15498  13706  14530  -4841  -3608
94       C
ATOM  11833  N   MET B 723      32.887  -3.238 110.021  1.00 80.01
N
ANISOU11833  N   MET B 723    11053   9514   9833  -4952  -3496
-104       N
ATOM  11834  CA  MET B 723      31.745  -2.598 110.676  1.00 88.84
C
ANISOU11834  CA  MET B 723    12167  10682  10907  -4968  -3470
-240       C
ATOM  11835  C   MET B 723      31.334  -3.288 111.976  1.00116.28
C
ANISOU11835  C   MET B 723    15652  14258  14270  -5050  -3481
-231       C
ATOM  11836  O   MET B 723      30.962  -2.618 112.947  1.00155.43
O
ANISOU11836  O   MET B 723    20657  19251  19149  -5081  -3509
-379       O
ATOM  11837  CB  MET B 723      30.560  -2.543 109.714  1.00 79.50
C
ANISOU11837  CB  MET B 723    10881   9521   9805  -4925  -3377
-244       C
ATOM  11838  CG  MET B 723      30.808  -1.672 108.498  1.00104.22
C
ANISOU11838  CG  MET B 723    14008  12564  13028  -4858  -3374
-268       C
ATOM  11839  SD  MET B 723      31.009   0.077 108.877  1.00 80.27
S
ANISOU11839  SD  MET B 723    11055   9442  10002  -4835  -3447
-468       S
ATOM  11840  CE  MET B 723      32.782   0.250 108.666  1.00 80.61
C
ANISOU11840  CE  MET B 723    11190   9397  10041  -4831  -3535
-398       C
ATOM  11841  N   THR B 724      31.379  -4.619 112.013  1.00 80.81
N
ANISOU11841  N   THR B 724    11116   9820   9770  -5089  -3467
-66       N
ATOM  11842  CA  THR B 724      31.130  -5.349 113.256  1.00 81.69
C
ANISOU11842  CA  THR B 724    11242  10030   9765  -5185  -3495
-21       C
ATOM  11843  C   THR B 724      32.151  -5.019 114.343  1.00 83.11
C
ANISOU11843  C   THR B 724    11547  10189   9843  -5239  -3605
-63       C
```

```
ATOM  11844  O   THR B 724      31.789  -4.793 115.504  1.00 84.34
O
ANISOU11844  O   THR B 724    11751  10425   9871  -5309  -3636
-150       O
ATOM  11845  CB  THR B 724      31.110  -6.856 112.984  1.00 81.05
C
ANISOU11845  CB  THR B 724    11086   9989   9721  -5211  -3478
180       C
ATOM  11846  OG1 THR B 724      30.217  -7.139 111.901  1.00151.15
O
ANISOU11846  OG1 THR B 724    19853  18878  18697  -5154  -3384
212       O
ATOM  11847  CG2 THR B 724      30.650  -7.615 114.219  1.00 82.00
C
ANISOU11847  CG2 THR B 724    11206  10228   9723  -5324  -3503
246       C
ATOM  11848  N   VAL B 725      33.434  -4.998 113.986  1.00 98.00
N
ANISOU11848  N   VAL B 725    13484  11973  11780  -5209  -3670
-8       N
ATOM  11849  CA  VAL B 725      34.486  -4.637 114.937  1.00 84.52
C
ANISOU11849  CA  VAL B 725    11897  10226   9992  -5254  -3786
-42       C
ATOM  11850  C   VAL B 725      34.321  -3.213 115.465  1.00 85.59
C
ANISOU11850  C   VAL B 725    12111  10344  10065  -5239  -3816
-262       C
ATOM  11851  O   VAL B 725      34.433  -2.973 116.674  1.00145.27
O
ANISOU11851  O   VAL B 725    19756  17950  17492  -5304  -3888
-344       O
ATOM  11852  CB  VAL B 725      35.870  -4.845 114.298  1.00 84.25
C
ANISOU11852  CB  VAL B 725    11881  10078  10054  -5215  -3846
55       C
ATOM  11853  CG1 VAL B 725      36.948  -4.154 115.114  1.00 85.73
C
ANISOU11853  CG1 VAL B 725    12193  10200  10181  -5243  -3968
-8       C
ATOM  11854  CG2 VAL B 725      36.163  -6.329 114.185  1.00 83.86
C
ANISOU11854  CG2 VAL B 725    11769  10050  10046  -5245  -3858
245       C
ATOM  11855  N   ILE B 726      34.044  -2.246 114.589  1.00 84.99
N
ANISOU11855  N   ILE B 726    12007  10204  10080  -5154  -3772
-369       N
ATOM  11856  CA  ILE B 726      33.876  -0.886 115.100  1.00 86.21
C
ANISOU11856  CA  ILE B 726    12230  10332  10194  -5131  -3815
-594       C
ATOM  11857  C   ILE B 726      32.559  -0.708 115.848  1.00 86.85
C
```

```
ANISOU11857  C   ILE B 726    12280  10531  10188  -5159  -3773
-745       C
ATOM  11858  O   ILE B 726      32.453   0.172 116.710  1.00131.41
O
ANISOU11858  O   ILE B 726    17993  16191  15745  -5160  -3832
-952       O
ATOM  11859  CB  ILE B 726      34.002   0.158 113.976  1.00 85.63
C
ANISOU11859  CB  ILE B 726    12137  10148  10252  -5040  -3800
-661       C
ATOM  11860  CG1 ILE B 726      32.773   0.128 113.067  1.00108.20
C
ANISOU11860  CG1 ILE B 726    14878  13038  13195  -4997  -3687
-665       C
ATOM  11861  CG2 ILE B 726      35.284  -0.066 113.183  1.00 85.10
C
ANISOU11861  CG2 ILE B 726    12086   9984  10262  -5017  -3837
-512       C
ATOM  11862  CD1 ILE B 726      32.777   1.212 112.020  1.00136.30
C
ANISOU11862  CD1 ILE B 726    18419  16497  16871  -4921  -3682
-731       C
ATOM  11863  N   PHE B 727      31.537  -1.508 115.541  1.00 85.81
N
ANISOU11863  N   PHE B 727    12041  10487  10076  -5176  -3676
-661       N
ATOM  11864  CA  PHE B 727      30.365  -1.559 116.413  1.00100.78
C
ANISOU11864  CA  PHE B 727    13903  12521  11868  -5225  -3646
-777       C
ATOM  11865  C   PHE B 727      30.732  -2.017 117.821  1.00112.26
C
ANISOU11865  C   PHE B 727    15446  14077  13131  -5329  -3725
-772       C
ATOM  11866  O   PHE B 727      30.322  -1.404 118.814  1.00 96.57
O
ANISOU11866  O   PHE B 727    13510  12173  11009  -5348  -3766
-977       O
ATOM  11867  CB  PHE B 727      29.301  -2.480 115.813  1.00119.48
C
ANISOU11867  CB  PHE B 727    16135  14963  14300  -5238  -3535
-645       C
ATOM  11868  CG  PHE B 727      28.120  -2.710 116.718  1.00146.72
C
ANISOU11868  CG  PHE B 727    19534  18573  17640  -5306  -3502
-727       C
ATOM  11869  CD1 PHE B 727      27.097  -1.781 116.796  1.00140.81
C
ANISOU11869  CD1 PHE B 727    18739  17852  16912  -5260  -3474
-956       C
ATOM  11870  CD2 PHE B 727      28.038  -3.858 117.493  1.00135.06
C
ANISOU11870  CD2 PHE B 727    18051  17223  16043  -5418  -3509
-575       C
```

```
ATOM  11871  CE1 PHE B 727      26.013  -1.992 117.630  1.00120.27
C
ANISOU11871  CE1 PHE B 727    16083  15412  14203  -5321  -3449
-1044       C
ATOM  11872  CE2 PHE B 727      26.958  -4.076 118.329  1.00 87.29
C
ANISOU11872  CE2 PHE B 727    11948  11343   9875  -5493  -3481
-640       C
ATOM  11873  CZ  PHE B 727      25.944  -3.143 118.398  1.00 94.48
C
ANISOU11873  CZ  PHE B 727    12818  12292  10789  -5452  -3451
-874       C
ATOM  11874  N   PHE B 728      31.497  -3.104 117.923  1.00142.86
N
ANISOU11874  N   PHE B 728    19341  17952  16988  -5393  -3756
-546       N
ATOM  11875  CA  PHE B 728      31.940  -3.600 119.223  1.00123.37
C
ANISOU11875  CA  PHE B 728    16961  15573  14343  -5505  -3846
-505       C
ATOM  11876  C   PHE B 728      32.805  -2.582 119.961  1.00 91.21
C
ANISOU11876  C   PHE B 728    13030  11446  10180  -5494  -3962
-677       C
ATOM  11877  O   PHE B 728      32.604  -2.330 121.153  1.00 93.07
O
ANISOU11877  O   PHE B 728    13342  11796  10226  -5552  -4023
-811       O
ATOM  11878  CB  PHE B 728      32.700  -4.913 119.034  1.00112.24
C
ANISOU11878  CB  PHE B 728    15535  14134  12976  -5560  -3874
-225       C
ATOM  11879  CG  PHE B 728      33.172  -5.533 120.315  1.00 90.48
C
ANISOU11879  CG  PHE B 728    12862  11463  10053  -5689  -3977
-141       C
ATOM  11880  CD1 PHE B 728      34.516  -5.789 120.518  1.00 91.06
C
ANISOU11880  CD1 PHE B 728    13018  11437  10142  -5712  -4089
-32       C
ATOM  11881  CD2 PHE B 728      32.272  -5.875 121.310  1.00 98.05
C
ANISOU11881  CD2 PHE B 728    13810  12607  10838  -5793  -3968
-163       C
ATOM  11882  CE1 PHE B 728      34.956  -6.364 121.695  1.00147.31
C
ANISOU11882  CE1 PHE B 728    20221  18633  17116  -5839  -4197
59       C
ATOM  11883  CE2 PHE B 728      32.706  -6.454 122.489  1.00109.12
C
ANISOU11883  CE2 PHE B 728    15292  14102  12069  -5925  -4072
-71       C
ATOM  11884  CZ  PHE B 728      34.050  -6.697 122.682  1.00134.50
C
```

ANISOU11884 CZ PHE B 728   18594 17202 15307 -5949 -4189
44   C
ATOM 11885 N   TRP B 729   33.765 -1.975 119.260 1.00 90.79
N
ANISOU11885 N   TRP B 729   13015 11230 10251 -5417 -3999
-681   N
ATOM 11886 CA TRP B 729   34.556 -0.886 119.833 1.00 92.45
C
ANISOU11886 CA TRP B 729   13350 11371 10406 -5392 -4110
-854   C
ATOM 11887 C   TRP B 729   33.690   0.291 120.277 1.00123.74
C
ANISOU11887 C   TRP B 729   17331 15387 14298 -5339 -4107
-1162   C
ATOM 11888 O   TRP B 729   33.900   0.850 121.359 1.00158.15
O
ANISOU11888 O   TRP B 729   21798 19796 18497 -5357 -4203
-1339   O
ATOM 11889 CB TRP B 729   35.606 -0.435 118.815 1.00 91.60
C
ANISOU11889 CB TRP B 729   13252 11084 10467 -5317 -4135
-789   C
ATOM 11890 CG TRP B 729   36.339   0.819 119.178 1.00121.73
C
ANISOU11890 CG TRP B 729   17178 14808 14267 -5278 -4241
-968   C
ATOM 11891 CD1 TRP B 729   37.526   0.906 119.841 1.00113.00
C
ANISOU11891 CD1 TRP B 729   16186 13645 13104 -5319 -4370
-940   C
ATOM 11892 CD2 TRP B 729   35.943   2.165 118.886 1.00 95.87
C
ANISOU11892 CD2 TRP B 729   13904 11478 11044 -5190 -4238
-1198   C
ATOM 11893 NE1 TRP B 729   37.893   2.221 119.984 1.00145.20
N
ANISOU11893 NE1 TRP B 729   20339 17639 17191 -5262 -4445
-1139   N
ATOM 11894 CE2 TRP B 729   36.937   3.015 119.407 1.00124.65
C
ANISOU11894 CE2 TRP B 729   17668 15036 14657 -5182 -4367
-1302   C
ATOM 11895 CE3 TRP B 729   34.843   2.734 118.236 1.00138.59
C
ANISOU11895 CE3 TRP B 729   19223 16898 16537 -5120 -4147
-1325   C
ATOM 11896 CZ2 TRP B 729   36.865   4.403 119.301 1.00124.62
C
ANISOU11896 CZ2 TRP B 729   17694 14957 14699 -5105 -4407
-1530   C
ATOM 11897 CZ3 TRP B 729   34.772   4.112 118.134 1.00130.24
C
ANISOU11897 CZ3 TRP B 729   18193 15763 15530 -5045 -4190
-1551   C

```
ATOM  11898  CH2 TRP B 729      35.776   4.931 118.662  1.00121.20
C
ANISOU11898  CH2 TRP B 729    17167  14534  14350  -5038  -4318
-1653       C
ATOM  11899  N   ALA B 730      32.722   0.692 119.451  1.00 92.78
N
ANISOU11899  N   ALA B 730    13305  11456  10493  -5264  -4009
-1242       N
ATOM  11900  CA  ALA B 730      31.820   1.783 119.818  1.00 93.75
C
ANISOU11900  CA  ALA B 730    13424  11623  10573  -5200  -4011
-1550       C
ATOM  11901  C   ALA B 730      30.993   1.467 121.061  1.00 95.29
C
ANISOU11901  C   ALA B 730    13640  12021  10544  -5260  -4023
-1675       C
ATOM  11902  O   ALA B 730      30.750   2.351 121.892  1.00 97.36
O
ANISOU11902  O   ALA B 730    13975  12344  10674  -5216  -4091
-1962       O
ATOM  11903  CB  ALA B 730      30.903   2.124 118.644  1.00124.16
C
ANISOU11903  CB  ALA B 730    17143  15421  14610  -5122  -3907
-1575       C
ATOM  11904  N   ALA B 731      30.546   0.221 121.208  1.00 94.48
N
ANISOU11904  N   ALA B 731    13476  12039  10384  -5356  -3962
-1471       N
ATOM  11905  CA  ALA B 731      29.801  -0.169 122.402  1.00 96.66
C
ANISOU11905  CA  ALA B 731    13825  12534  10367  -5501  -3996
-1511       C
ATOM  11906  C   ALA B 731      30.691  -0.465 123.606  1.00 98.87
C
ANISOU11906  C   ALA B 731    14272  12890  10404  -5620  -4123
-1462       C
ATOM  11907  O   ALA B 731      30.240  -0.308 124.746  1.00168.08
O
ANISOU11907  O   ALA B 731    23215  21843  18806  -5800  -4205
-1547       O
ATOM  11908  CB  ALA B 731      28.925  -1.384 122.094  1.00 95.62
C
ANISOU11908  CB  ALA B 731    13591  12514  10227  -5633  -3901
-1260       C
ATOM  11909  N   HIS B 732      31.937  -0.884 123.393  1.00 97.52
N
ANISOU11909  N   HIS B 732    14080  12593  10379  -5565  -4151
-1314       N
ATOM  11910  CA  HIS B 732      32.769  -1.358 124.496  1.00141.57
C
ANISOU11910  CA  HIS B 732    19782  18239  15767  -5674  -4268
-1231       C
ATOM  11911  C   HIS B 732      33.765  -0.306 124.974  1.00120.07
C
```

ANISOU11911 C   HIS B 732   17206 15422 12993 -5622 -4396
-1413   C
ATOM 11912 O   HIS B 732   33.621   0.243 126.072 1.00113.32
O
ANISOU11912 O   HIS B 732   16494 14691 11872 -5664 -4489
-1610   O
ATOM 11913 CB HIS B 732   33.520 -2.627 124.081 1.00117.46
C
ANISOU11913 CB HIS B 732   16691 15110 12828 -5764 -4269
-874   C
ATOM 11914 CG HIS B 732   34.494 -3.120 125.106 1.00113.69
C
ANISOU11914 CG HIS B 732   16334 14662 12200 -5879 -4405
-760   C
ATOM 11915 ND1 HIS B 732   34.126 -3.440 126.394 1.00117.24
N
ANISOU11915 ND1 HIS B 732   16879 15321 12346 -6028 -4477
-767   N
ATOM 11916 CD2 HIS B 732   35.829 -3.336 125.033 1.00 99.74
C
ANISOU11916 CD2 HIS B 732   14634 12746 10518 -5901 -4504
-610   C
ATOM 11917 CE1 HIS B 732   35.189 -3.841 127.067 1.00103.15
C
ANISOU11917 CE1 HIS B 732   15174 13509 10510 -6089 -4597
-655   C
ATOM 11918 NE2 HIS B 732   36.237 -3.786 126.265 1.00101.87
N
ANISOU11918 NE2 HIS B 732   15006 13120 10579 -6029 -4623
-550   N
ATOM 11919 N   LYS B 733   34.785 -0.033 124.159 1.00100.72
N
ANISOU11919 N   LYS B 733   14762 12759 10747 -5575 -4421
-1318   N
ATOM 11920 CA LYS B 733   35.850   0.884 124.558 1.00111.00
C
ANISOU11920 CA LYS B 733   16195 13954 12025 -5540 -4551
-1447   C
ATOM 11921 C   LYS B 733   35.327   2.288 124.845 1.00146.67
C
ANISOU11921 C   LYS B 733   20754 18493 16481 -5428 -4573
-1815   C
ATOM 11922 O   LYS B 733   35.761   2.932 125.808 1.00145.12
O
ANISOU11922 O   LYS B 733   20688 18332 16118 -5422 -4691
-2003   O
ATOM 11923 CB LYS B 733   36.924   0.927 123.470 1.00103.87
C
ANISOU11923 CB LYS B 733   15268 12830 11366 -5501 -4559
-1270   C
ATOM 11924 CG LYS B 733   38.107   1.837 123.769 1.00107.25
C
ANISOU11924 CG LYS B 733   15821 13129 11802 -5474 -4696
-1364   C

```
ATOM  11925  CD  LYS B 733      38.866   1.386 125.005  1.00115.55
C
ANISOU11925  CD  LYS B 733    17000  14237  12667  -5584  -4834
-1307       C
ATOM  11926  CE  LYS B 733      40.116   2.227 125.216  1.00130.53
C
ANISOU11926  CE  LYS B 733    19013  15985  14598  -5560  -4976
-1369       C
ATOM  11927  NZ  LYS B 733      40.898   1.793 126.407  1.00151.92
N
ANISOU11927  NZ  LYS B 733    21852  18738  17134  -5672  -5124
-1303       N
ATOM  11928  N   THR B 734      34.398   2.780 124.032  1.00147.32
N
ANISOU11928  N   THR B 734    20727  18554  16695  -5334  -4469
-1930       N
ATOM  11929  CA  THR B 734      33.836   4.116 124.197  1.00162.11
C
ANISOU11929  CA  THR B 734    22618  20430  18546  -5214  -4491
-2288       C
ATOM  11930  C   THR B 734      32.339   4.050 124.497  1.00134.43
C
ANISOU11930  C   THR B 734    19056  17104  14919  -5195  -4419
-2452       C
ATOM  11931  O   THR B 734      31.722   2.982 124.511  1.00103.40
O
ANISOU11931  O   THR B 734    15106  13287  10894  -5335  -4359
-2238       O
ATOM  11932  CB  THR B 734      34.096   4.973 122.954  1.00186.87
C
ANISOU11932  CB  THR B 734    25690  23357  21955  -5123  -4462
-2302       C
ATOM  11933  OG1 THR B 734      33.732   6.333 123.223  1.00181.38
O
ANISOU11933  OG1 THR B 734    25023  22646  21245  -5016  -4511
-2658       O
ATOM  11934  CG2 THR B 734      33.285   4.464 121.776  1.00188.69
C
ANISOU11934  CG2 THR B 734    25763  23566  22366  -5107  -4318
-2147       C
ATOM  11935  N   ASP B 735      31.766   5.230 124.743  1.00154.53
N
ANISOU11935  N   ASP B 735    21681  19654  17382  -5141  -4471
-2768       N
ATOM  11936  CA  ASP B 735      30.424   5.394 125.288  1.00151.94
C
ANISOU11936  CA  ASP B 735    21471  19491  16768  -5252  -4476
-2924       C
ATOM  11937  C   ASP B 735      29.452   5.924 124.240  1.00151.41
C
ANISOU11937  C   ASP B 735    21260  19332  16938  -5162  -4388
-3020       C
ATOM  11938  O   ASP B 735      28.361   6.386 124.589  1.00139.16
O
```

```
ANISOU11938  O   ASP B 735    19798  17880  15198  -5215  -4395
-3228       O
ATOM  11939  CB  ASP B 735      30.463   6.345 126.489  1.00136.48
C
ANISOU11939  CB  ASP B 735    19742  17623  14491  -5250  -4609
-3260       C
ATOM  11940  CG  ASP B 735      31.326   5.823 127.622  1.00132.01
C
ANISOU11940  CG  ASP B 735    19337  17174  13646  -5358  -4707
-3170       C
ATOM  11941  OD1 ASP B 735      31.489   6.549 128.627  1.00118.82
O
ANISOU11941  OD1 ASP B 735    17858  15584  11705  -5343  -4817
-3436       O
ATOM  11942  OD2 ASP B 735      31.841   4.691 127.509  1.00121.51
O
ANISOU11942  OD2 ASP B 735    17943  15853  12370  -5453  -4677
-2840       O
ATOM  11943  N   PHE B 736      29.847   5.873 122.965  1.00149.09
N
ANISOU11943  N   PHE B 736    20748  18862  17037  -5029  -4297
-2879       N
ATOM  11944  CA  PHE B 736      29.238   6.679 121.908  1.00159.10
C
ANISOU11944  CA  PHE B 736    21871  20000  18580  -4904  -4235
-3004       C
ATOM  11945  C   PHE B 736      27.731   6.465 121.787  1.00128.71
C
ANISOU11945  C   PHE B 736    18017  16245  14643  -5006  -4185
-3023       C
ATOM  11946  O   PHE B 736      26.962   7.428 121.692  1.00146.68
O
ANISOU11946  O   PHE B 736    20308  18479  16943  -4964  -4215
-3280       O
ATOM  11947  CB  PHE B 736      29.926   6.368 120.578  1.00188.11
C
ANISOU11947  CB  PHE B 736    25404  23515  22556  -4858  -4146
-2735       C
ATOM  11948  CG  PHE B 736      29.272   7.010 119.391  1.00184.46
C
ANISOU11948  CG  PHE B 736    24814  22934  22339  -4787  -4079
-2777       C
ATOM  11949  CD1 PHE B 736      29.438   8.361 119.135  1.00146.78
C
ANISOU11949  CD1 PHE B 736    20055  18037  17679  -4706  -4135
-3000       C
ATOM  11950  CD2 PHE B 736      28.487   6.258 118.531  1.00146.95
C
ANISOU11950  CD2 PHE B 736    19930  18198  17704  -4809  -3961
-2585       C
ATOM  11951  CE1 PHE B 736      28.834   8.949 118.044  1.00124.71
C
ANISOU11951  CE1 PHE B 736    17145  15137  15105  -4658  -4077
-3022       C
```

```
ATOM  11952  CE2 PHE B 736      27.880   6.838 117.441  1.00136.85
C
ANISOU11952  CE2 PHE B 736    18540  16816  16642  -4750  -3906
-2613       C
ATOM  11953  CZ  PHE B 736      28.053   8.186 117.195  1.00125.95
C
ANISOU11953  CZ  PHE B 736    17175  15313  15369  -4678  -3965
-2827       C
ATOM  11954  N   PHE B 737      27.290   5.206 121.783  1.00105.97
N
ANISOU11954  N   PHE B 737    15106  13492  11667  -5151  -4107
-2755       N
ATOM  11955  CA  PHE B 737      25.862   4.913 121.671  1.00109.44
C
ANISOU11955  CA  PHE B 737    15521  14050  12011  -5282  -4042
-2744       C
ATOM  11956  C   PHE B 737      25.048   5.478 122.832  1.00128.91
C
ANISOU11956  C   PHE B 737    18199  16702  14080  -5414  -4106
-3030       C
ATOM  11957  O   PHE B 737      23.975   6.054 122.618  1.00118.52
O
ANISOU11957  O   PHE B 737    16869  15395  12768  -5427  -4081
-3215       O
ATOM  11958  CB  PHE B 737      25.648   3.406 121.551  1.00112.65
C
ANISOU11958  CB  PHE B 737    15848  14585  12369  -5434  -3945
-2392       C
ATOM  11959  CG  PHE B 737      26.234   2.811 120.303  1.00147.16
C
ANISOU11959  CG  PHE B 737    20010  18792  17114  -5285  -3849
-2150       C
ATOM  11960  CD1 PHE B 737      27.057   1.699 120.368  1.00154.66
C
ANISOU11960  CD1 PHE B 737    20933  19762  18067  -5311  -3823
-1891       C
ATOM  11961  CD2 PHE B 737      25.979   3.379 119.065  1.00135.28
C
ANISOU11961  CD2 PHE B 737    18339  17121  15939  -5114  -3782
-2200       C
ATOM  11962  CE1 PHE B 737      27.598   1.150 119.220  1.00129.33
C
ANISOU11962  CE1 PHE B 737    17560  16423  15157  -5175  -3724
-1702       C
ATOM  11963  CE2 PHE B 737      26.522   2.838 117.914  1.00121.91
C
ANISOU11963  CE2 PHE B 737    16493  15316  14513  -5000  -3680
-1991       C
ATOM  11964  CZ  PHE B 737      27.333   1.722 117.992  1.00 90.56
C
ANISOU11964  CZ  PHE B 737    12565  11374  10469  -5088  -3671
-1713       C
ATOM  11965  N   SER B 738      25.529   5.327 124.065  1.00152.61
N
```

```
ANISOU11965  N   SER B 738    21401  19865  16720  -5509  -4177
-3084       N
ATOM  11966  CA  SER B 738      24.762   5.829 125.200  1.00120.44
C
ANISOU11966  CA  SER B 738    17543  16019  12199  -5622  -4198
-3382       C
ATOM  11967  C   SER B 738      24.890   7.335 125.381  1.00128.51
C
ANISOU11967  C   SER B 738    18669  16892  13266  -5428  -4305
-3797       C
ATOM  11968  O   SER B 738      23.947   7.981 125.853  1.00119.79
O
ANISOU11968  O   SER B 738    17687  15904  11925  -5457  -4286
-4108       O
ATOM  11969  CB  SER B 738      25.210   5.128 126.479  1.00121.03
C
ANISOU11969  CB  SER B 738    17799  16352  11834  -5792  -4225
-3295       C
ATOM  11970  OG  SER B 738      26.577   5.384 126.758  1.00133.87
O
ANISOU11970  OG  SER B 738    19484  17828  13551  -5663  -4351
-3295       O
ATOM  11971  N   ASP B 739      26.036   7.906 125.016  1.00153.97
N
ANISOU11971  N   ASP B 739    21836  19881  16785  -5236  -4398
-3820       N
ATOM  11972  CA  ASP B 739      26.170   9.357 124.961  1.00158.44
C
ANISOU11972  CA  ASP B 739    22433  20276  17491  -5048  -4494
-4187       C
ATOM  11973  C   ASP B 739      25.198   9.969 123.956  1.00177.99
C
ANISOU11973  C   ASP B 739    24763  22609  20256  -4980  -4447
-4298       C
ATOM  11974  O   ASP B 739      24.534  10.971 124.245  1.00191.68
O
ANISOU11974  O   ASP B 739    26591  24316  21922  -4923  -4498
-4660       O
ATOM  11975  CB  ASP B 739      27.615   9.732 124.626  1.00132.00
C
ANISOU11975  CB  ASP B 739    18991  16744  14420  -4902  -4555
-4131       C
ATOM  11976  CG  ASP B 739      27.861  11.227 124.696  1.00163.63
C
ANISOU11976  CG  ASP B 739    23019  20612  18541  -4745  -4646
-4507       C
ATOM  11977  OD1 ASP B 739      27.076  11.930 125.367  1.00188.12
O
ANISOU11977  OD1 ASP B 739    26278  23776  21422  -4733  -4702
-4853       O
ATOM  11978  OD2 ASP B 739      28.844  11.698 124.085  1.00179.09
O
ANISOU11978  OD2 ASP B 739    24839  22414  20795  -4646  -4643
-4462       O
```

ATOM 11979 N   THR B 740   25.100   9.370 122.767 1.00169.90
N
ANISOU11979 N   THR B 740   23509 21488 19558 -4979 -4349
-3998   N
ATOM 11980 CA THR B 740   24.173   9.859 121.748 1.00146.54
C
ANISOU11980 CA THR B 740   20394 18398 16889 -4929 -4302
-4059   C
ATOM 11981 C   THR B 740   22.713   9.582 122.101 1.00142.65
C
ANISOU11981 C   THR B 740   19977 18073 16150 -5096 -4249
-4141   C
ATOM 11982 O   THR B 740   21.878 10.493 122.055 1.00145.02
O
ANISOU11982 O   THR B 740   20307 18307 16486 -5060 -4280
-4440   O
ATOM 11983 CB THR B 740   24.518   9.247 120.390 1.00125.85
C
ANISOU11983 CB THR B 740   17515 15658 14643 -4873 -4191
-3714   C
ATOM 11984 OG1 THR B 740   25.919   9.408 120.136 1.00134.74
O
ANISOU11984 OG1 THR B 740   18586 16685 15923 -4754 -4195
-3634   O
ATOM 11985 CG2 THR B 740   23.729   9.935 119.287 1.00114.05
C
ANISOU11985 CG2 THR B 740   15851 14010 13473 -4800 -4150
-3791   C
ATOM 11986 N   PHE B 741   22.374   8.340 122.453 1.00136.27
N
ANISOU11986 N   PHE B 741   19186 17501 15088 -5298 -4150
-3890   N
ATOM 11987 CA PHE B 741   20.973   7.939 122.474 1.00141.10
C
ANISOU11987 CA PHE B 741   19771 18305 15537 -5491 -4032
-3897   C
ATOM 11988 C   PHE B 741   20.427   7.613 123.859 1.00170.53
C
ANISOU11988 C   PHE B 741   23705 22419 18668 -5709 -3953
-4054   C
ATOM 11989 O   PHE B 741   19.210   7.446 123.995 1.00182.83
O
ANISOU11989 O   PHE B 741   25229 24208 20031 -5882 -3810
-4150   O
ATOM 11990 CB PHE B 741   20.762   6.722 121.560 1.00130.59
C
ANISOU11990 CB PHE B 741   18211 16988 14420 -5579 -3921
-3471   C
ATOM 11991 CG PHE B 741   21.186   6.950 120.137 1.00144.00
C
ANISOU11991 CG PHE B 741   19691 18378 16645 -5367 -3930
-3322   C
ATOM 11992 CD1 PHE B 741   22.212   6.203 119.578 1.00139.77
C

```
ANISOU11992  CD1 PHE B 741    19046  17763  16295  -5279  -3894
-3023       C
ATOM  11993  CD2 PHE B 741      20.559   7.906 119.355  1.00161.11
C
ANISOU11993  CD2 PHE B 741    21756  20359  19099  -5260  -3953
-3498       C
ATOM  11994  CE1 PHE B 741      22.605   6.407 118.268  1.00132.41
C
ANISOU11994  CE1 PHE B 741    17919  16618  15775  -5091  -3847
-2916       C
ATOM  11995  CE2 PHE B 741      20.946   8.114 118.043  1.00154.83
C
ANISOU11995  CE2 PHE B 741    20754  19340  18735  -5083  -3924
-3363       C
ATOM  11996  CZ  PHE B 741      21.971   7.364 117.499  1.00138.39
C
ANISOU11996  CZ  PHE B 741    18575  17225  16781  -4999  -3855
-3079       C
ATOM  11997  N   GLY B 742      21.273   7.529 124.882  1.00154.47
N
ANISOU11997  N   GLY B 742    21862  20500  16328  -5709  -4014
-4096       N
ATOM  11998  CA  GLY B 742      20.847   7.127 126.213  1.00123.39
C
ANISOU11998  CA  GLY B 742    18113  16987  11783  -5911  -3911
-4212       C
ATOM  11999  C   GLY B 742      20.098   5.810 126.257  1.00122.81
C
ANISOU11999  C   GLY B 742    17912  17247  11504  -6198  -3741
-3914       C
ATOM  12000  O   GLY B 742      20.309   4.904 125.444  1.00146.15
O
ANISOU12000  O   GLY B 742    20674  20090  14765  -6242  -3742
-3519       O
ATOM  12001  N   VAL B 743      19.229   5.700 127.271  1.00126.85
N
ANISOU12001  N   VAL B 743    18524  18210  11463  -6382  -3574
-4122       N
ATOM  12002  CA  VAL B 743      18.313   4.586 127.534  1.00151.30
C
ANISOU12002  CA  VAL B 743    21491  21749  14248  -6681  -3382
-3923       C
ATOM  12003  C   VAL B 743      19.068   3.445 128.206  1.00172.65
C
ANISOU12003  C   VAL B 743    24250  24636  16713  -6833  -3428
-3556       C
ATOM  12004  O   VAL B 743      18.651   2.946 129.257  1.00193.37
O
ANISOU12004  O   VAL B 743    26950  27742  18780  -7028  -3299
-3568       O
ATOM  12005  CB  VAL B 743      17.584   4.104 126.263  1.00144.24
C
ANISOU12005  CB  VAL B 743    20293  20723  13789  -6754  -3330
-3682       C
```

ATOM 12006 CG1 VAL B 743   16.614   2.983 126.605 1.00125.81
C
ANISOU12006 CG1 VAL B 743   17772 18837 11193 -6974 -3099
-3437   C
ATOM 12007 CG2 VAL B 743   16.837   5.261 125.605 1.00134.46
C
ANISOU12007 CG2 VAL B 743   18993 19284 12811 -6614 -3300
-4040   C
ATOM 12008 N   ARG B 744   20.181   3.027 127.610 1.00168.11
N
ANISOU12008 N   ARG B 744   23625 23701 16549 -6731 -3594
-3237   N
ATOM 12009 CA ARG B 744   21.088   2.069 128.223 1.00150.70
C
ANISOU12009 CA ARG B 744   21482 21584 14193 -6835 -3669
-2924   C
ATOM 12010 C   ARG B 744   22.509   2.573 128.019 1.00129.92
C
ANISOU12010 C   ARG B 744   18936 18547 11880 -6583 -3858
-2933   C
ATOM 12011 O   ARG B 744   22.756   3.446 127.185 1.00119.72
O
ANISOU12011 O   ARG B 744   17592 16908 10988 -6345 -3921
-3085   O
ATOM 12012 CB ARG B 744   20.915   0.661 127.635 1.00178.78
C
ANISOU12012 CB ARG B 744   24806 25182 17941 -7016 -3625
-2451   C
ATOM 12013 CG ARG B 744   19.621 -0.032 128.049 1.00183.73
C
ANISOU12013 CG ARG B 744   25326 26298 18186 -7316 -3452
-2377   C
ATOM 12014 CD ARG B 744   19.506 -0.182 129.561 1.00168.69
C
ANISOU12014 CD ARG B 744   23617 24887 15590 -7491 -3393
-2472   C
ATOM 12015 NE ARG B 744   19.170 -1.548 129.958 1.00190.23
N
ANISOU12015 NE ARG B 744   26221 27979 18080 -7789 -3341
-2075   N
ATOM 12016 CZ ARG B 744   20.062 -2.514 130.150 1.00166.90
C
ANISOU12016 CZ ARG B 744   23265 24937 15211 -7869 -3469
-1696   C
ATOM 12017 NH1 ARG B 744   19.670 -3.711 130.566 1.00144.03
N
ANISOU12017 NH1 ARG B 744   20250 22391 12083 -8148 -3431
-1340   N
ATOM 12018 NH2 ARG B 744   21.354 -2.268 129.974 1.00124.71
N
ANISOU12018 NH2 ARG B 744   18033 19180 10173 -7670 -3635
-1678   N
ATOM 12019 N   SER B 745   23.452   2.014 128.781 1.00122.33
N

```
ANISOU12019  N   SER B 745    18085  17650  10744  -6643  -3945
-2763       N
ATOM  12020  CA  SER B 745      24.797   2.582 128.832  1.00147.43
C
ANISOU12020  CA  SER B 745    21358  20527  14131  -6426  -4112
-2832       C
ATOM  12021  C   SER B 745      25.817   1.506 129.186  1.00120.57
C
ANISOU12021  C   SER B 745    17955  17127  10729  -6528  -4188
-2478       C
ATOM  12022  O   SER B 745      26.758   1.747 129.949  1.00122.21
O
ANISOU12022  O   SER B 745    18320  17313  10802  -6482  -4309
-2541       O
ATOM  12023  CB  SER B 745      24.877   3.745 129.827  1.00186.31
C
ANISOU12023  CB  SER B 745    26534  25541  18716  -6323  -4171
-3259       C
ATOM  12024  OG  SER B 745      25.991   4.579 129.552  1.00186.25
O
ANISOU12024  OG  SER B 745    26551  25186  19030  -6070  -4327
-3386       O
ATOM  12025  N   ILE B 746      25.620   0.300 128.652  1.00118.05
N
ANISOU12025  N   ILE B 746    17455  16834  10565  -6669  -4123
-2106       N
ATOM  12026  CA  ILE B 746      26.570  -0.789 128.851  1.00116.82
C
ANISOU12026  CA  ILE B 746    17272  16638  10475  -6755  -4194
-1757       C
ATOM  12027  C   ILE B 746      27.938  -0.455 128.263  1.00128.12
C
ANISOU12027  C   ILE B 746    18667  17689  12325  -6500  -4291
-1751       C
ATOM  12028  O   ILE B 746      28.052   0.219 127.231  1.00131.82
O
ANISOU12028  O   ILE B 746    19020  17896  13171  -6268  -4264
-1871       O
ATOM  12029  CB  ILE B 746      26.027  -2.092 128.242  1.00114.51
C
ANISOU12029  CB  ILE B 746    16769  16410  10328  -6913  -4099
-1385       C
ATOM  12030  CG1 ILE B 746      25.735  -1.903 126.753  1.00119.64
C
ANISOU12030  CG1 ILE B 746    17206  16781  11472  -6710  -4014
-1381       C
ATOM  12031  CG2 ILE B 746      24.777  -2.534 128.982  1.00117.66
C
ANISOU12031  CG2 ILE B 746    17185  17258  10263  -7211  -4004
-1349       C
ATOM  12032  CD1 ILE B 746      25.590  -3.195 125.986  1.00124.93
C
ANISOU12032  CD1 ILE B 746    17664  17405  12400  -6765  -3943
-1012       C
```

```
ATOM  12033  N   ARG B 747      28.988  -0.924 128.939  1.00168.72
N
ANISOU12033  N   ARG B 747    23897  22824  17384  -6552  -4401
-1608       N
ATOM  12034  CA  ARG B 747      30.348  -0.892 128.409  1.00158.40
C
ANISOU12034  CA  ARG B 747    22523  21208  16454  -6357  -4470
-1536       C
ATOM  12035  C   ARG B 747      30.951  -2.280 128.579  1.00154.02
C
ANISOU12035  C   ARG B 747    21916  20670  15934  -6502  -4500
-1159       C
ATOM  12036  O   ARG B 747      31.303  -2.942 127.597  1.00151.57
O
ANISOU12036  O   ARG B 747    21421  20178  15989  -6403  -4437
-959       O
ATOM  12037  CB  ARG B 747      31.269   0.125 129.099  1.00114.21
C
ANISOU12037  CB  ARG B 747    17094  15536  10763  -6238  -4606
-1792       C
ATOM  12038  CG  ARG B 747      31.083   1.613 128.808  1.00114.73
C
ANISOU12038  CG  ARG B 747    17190  15486  10915  -6022  -4613
-2177       C
ATOM  12039  CD  ARG B 747      29.799   2.216 129.333  1.00159.62
C
ANISOU12039  CD  ARG B 747    23000  21400  16248  -6095  -4571
-2440       C
ATOM  12040  NE  ARG B 747      29.984   3.651 129.541  1.00204.81
N
ANISOU12040  NE  ARG B 747    28830  27026  21964  -5900  -4648
-2835       N
ATOM  12041  CZ  ARG B 747      29.339   4.380 130.446  1.00190.17
C
ANISOU12041  CZ  ARG B 747    27174  25370  19712  -5920  -4666
-3155       C
ATOM  12042  NH1 ARG B 747      28.332   3.859 131.131  1.00177.11
N
ANISOU12042  NH1 ARG B 747    25617  24055  17622  -6131  -4573
-3136       N
ATOM  12043  NH2 ARG B 747      29.609   5.674 130.565  1.00163.44
N
ANISOU12043  NH2 ARG B 747    23866  21855  16378  -5719  -4750
-3512       N
ATOM  12044  N   ASP B 748      31.068  -2.723 129.831  1.00160.02
N
ANISOU12044  N   ASP B 748    22839  21658  16303  -6728  -4594
-1069       N
ATOM  12045  CA  ASP B 748      31.718  -3.979 130.180  1.00134.25
C
ANISOU12045  CA  ASP B 748    19553  18410  13045  -6886  -4663
-717       C
ATOM  12046  C   ASP B 748      30.729  -5.055 130.621  1.00115.83
C
```

ANISOU12046 C   ASP B 748   17190 16381 10439 -7187 -4616
-457   C
ATOM 12047 O   ASP B 748   31.136 -6.058 131.216 1.00116.87
O
ANISOU12047 O   ASP B 748   17341 16597 10468 -7377 -4700
-170   O
ATOM 12048 CB ASP B 748   32.752 -3.728 131.276 1.00117.09
C
ANISOU12048 CB ASP B 748   17573 16253 10662 -6927 -4831
-765   C
ATOM 12049 CG ASP B 748   32.152 -3.044 132.484 1.00124.74
C
ANISOU12049 CG ASP B 748   18767 17526 11104 -7051 -4869
-1000   C
ATOM 12050 OD1 ASP B 748   32.030 -3.695 133.541 1.00202.47
O
ANISOU12050 OD1 ASP B 748   28723 27639 20565 -7303 -4928
-839   O
ATOM 12051 OD2 ASP B 748   31.799 -1.849 132.372 1.00122.45
O
ANISOU12051 OD2 ASP B 748   18539 17215 10773 -6886 -4835
-1353   O
ATOM 12052 N   ASN B 749   29.437 -4.869 130.345 1.00118.65
N
ANISOU12052 N   ASN B 749   17487 16912 10680 -7246 -4487
-543   N
ATOM 12053 CA ASN B 749   28.419 -5.853 130.721 1.00158.27
C
ANISOU12053 CA ASN B 749   22442 22259 15435 -7543 -4426
-296   C
ATOM 12054 C   ASN B 749   28.343 -6.895 129.606 1.00143.68
C
ANISOU12054 C   ASN B 749   20350 20229 14011 -7501 -4360
3   C
ATOM 12055 O   ASN B 749   27.482 -6.860 128.724 1.00143.39
O
ANISOU12055 O   ASN B 749   20161 20170 14150 -7437 -4232
-24   O
ATOM 12056 CB ASN B 749   27.083 -5.168 130.976 1.00184.56
C
ANISOU12056 CB ASN B 749   25810 25893 18420 -7624 -4306
-542   C
ATOM 12057 CG ASN B 749   26.084 -6.073 131.666 1.00180.68
C
ANISOU12057 CG ASN B 749   25275 25849 17527 -7964 -4244
-311   C
ATOM 12058 OD1 ASN B 749   26.372 -7.236 131.949 1.00197.92
O
ANISOU12058 OD1 ASN B 749   27395 28096 19711 -8149 -4310
56   O
ATOM 12059 ND2 ASN B 749   24.913 -5.533 131.972 1.00158.70
N
ANISOU12059 ND2 ASN B 749   22517 23398 14384 -8045 -4115
-529   N

ATOM 12060 N   ASN B 750   29.282 -7.844 129.666 1.00122.40
N
ANISOU12060 N   ASN B 750   17625 17401 11481 -7522 -4454
281   N
ATOM 12061 CA ASN B 750   29.573 -8.723 128.534 1.00121.76
C
ANISOU12061 CA ASN B 750   17338 17069 11855 -7368 -4402
496   C
ATOM 12062 C   ASN B 750   28.360 -9.532 128.079 1.00115.93
C
ANISOU12062 C   ASN B 750   16424 16485 11139 -7493 -4291
694   C
ATOM 12063 O   ASN B 750   28.095 -9.641 126.877 1.00122.38
O
ANISOU12063 O   ASN B 750   17073 17117 12307 -7281 -4178
680   O
ATOM 12064 CB ASN B 750   30.725 -9.663 128.893 1.00128.86
C
ANISOU12064 CB ASN B 750   18256 17856 12851 -7405 -4534
754   C
ATOM 12065 CG ASN B 750   32.046 -8.938 129.036 1.00153.45
C
ANISOU12065 CG ASN B 750   21485 20752 16068 -7222 -4627
574   C
ATOM 12066 OD1 ASN B 750   32.122 -7.722 128.863 1.00160.14
O
ANISOU12066 OD1 ASN B 750   22397 21527 16923 -7059 -4598
260   O
ATOM 12067 ND2 ASN B 750   33.099 -9.683 129.352 1.00162.33
N
ANISOU12067 ND2 ASN B 750   22626 21770 17281 -7248 -4747
776   N
ATOM 12068 N   HIS B 751   27.613 -10.115 129.022 1.00111.39
N
ANISOU12068 N   HIS B 751   15872 16267 10184 -7833 -4318
885   N
ATOM 12069 CA HIS B 751   26.430 -10.887 128.641 1.00128.11
C
ANISOU12069 CA HIS B 751   17800 18560 12314 -7970 -4217
1091   C
ATOM 12070 C   HIS B 751   25.380 -10.037 127.931 1.00127.09
C
ANISOU12070 C   HIS B 751   17592 18464 12231 -7870 -4063
834   C
ATOM 12071 O   HIS B 751   24.791 -10.472 126.935 1.00107.53
O
ANISOU12071 O   HIS B 751   14922 15889 10047 -7766 -3962
926   O
ATOM 12072 CB HIS B 751   25.816 -11.534 129.884 1.00169.82
C
ANISOU12072 CB HIS B 751   23112 24288 17123 -8377 -4272
1330   C
ATOM 12073 CG HIS B 751   26.681 -12.578 130.520 1.00194.23
C

```
ANISOU12073  CG  HIS B 751    26239  27355  20204  -8512  -4431
1661       C
ATOM  12074  ND1 HIS B 751      26.264 -13.333 131.596  1.00197.25
N
ANISOU12074  ND1 HIS B 751    26623  28116  20207  -8878  -4503
1952       N
ATOM  12075  CD2 HIS B 751      27.938 -12.991 130.237  1.00198.09
C
ANISOU12075  CD2 HIS B 751    26753  27501  21012  -8331  -4535
1750       C
ATOM  12076  CE1 HIS B 751      27.227 -14.167 131.946  1.00199.96
C
ANISOU12076  CE1 HIS B 751    27000  28318  20658  -8922  -4659
2218       C
ATOM  12077  NE2 HIS B 751      28.254 -13.980 131.137  1.00205.98
N
ANISOU12077  NE2 HIS B 751    27779  28644  21841  -8589  -4679
2090       N
ATOM  12078  N   GLU B 752      25.122  -8.830 128.435  1.00144.76
N
ANISOU12078  N   GLU B 752    19982  20838  14184  -7885  -4048
503       N
ATOM  12079  CA  GLU B 752      24.319  -7.854 127.700  1.00164.01
C
ANISOU12079  CA  GLU B 752    22365  23230  16722  -7739  -3925
211       C
ATOM  12080  C   GLU B 752      24.995  -7.378 126.414  1.00155.09
C
ANISOU12080  C   GLU B 752    21169  21647  16109  -7357  -3901
82       C
ATOM  12081  O   GLU B 752      24.340  -7.260 125.372  1.00170.66
O
ANISOU12081  O   GLU B 752    22985  23508  18351  -7222  -3791
43       O
ATOM  12082  CB  GLU B 752      23.964  -6.675 128.609  1.00185.72
C
ANISOU12082  CB  GLU B 752    25313  26227  19026  -7810  -3924
-144       C
ATOM  12083  CG  GLU B 752      22.943  -7.047 129.683  1.00198.31
C
ANISOU12083  CG  GLU B 752    26922  28368  20060  -8160  -3872
-71       C
ATOM  12084  CD  GLU B 752      22.352  -5.844 130.393  1.00185.07
C
ANISOU12084  CD  GLU B 752    25420  26970  17927  -8163  -3802
-489       C
ATOM  12085  OE1 GLU B 752      21.392  -6.026 131.171  1.00173.91
O
ANISOU12085  OE1 GLU B 752    24004  26059  16015  -8400  -3703
-489       O
ATOM  12086  OE2 GLU B 752      22.848  -4.720 130.176  1.00178.54
O
ANISOU12086  OE2 GLU B 752    24728  25880  17230  -7909  -3834
-822       O
```

```
ATOM  12087  N   LEU B 753      26.298  -7.093 126.472  1.00123.28
N
ANISOU12087  N   LEU B 753    17248  17381  12213  -7183  -3998
17       N
ATOM  12088  CA  LEU B 753      27.017  -6.559 125.315  1.00111.98
C
ANISOU12088  CA  LEU B 753    15746  15581  11222  -6827  -3965
-122       C
ATOM  12089  C   LEU B 753      27.004  -7.488 124.101  1.00129.55
C
ANISOU12089  C   LEU B 753    17752  17628  13842  -6667  -3872
82       C
ATOM  12090  O   LEU B 753      26.792  -7.026 122.974  1.00132.65
O
ANISOU12090  O   LEU B 753    18029  17846  14526  -6434  -3774
-45       O
ATOM  12091  CB  LEU B 753      28.456  -6.237 125.717  1.00127.97
C
ANISOU12091  CB  LEU B 753    17899  17440  13282  -6712  -4085
-189       C
ATOM  12092  CG  LEU B 753      29.347  -5.572 124.669  1.00 99.45
C
ANISOU12092  CG  LEU B 753    14216  13506  10066  -6367  -4053
-349       C
ATOM  12093  CD1 LEU B 753      28.737  -4.259 124.231  1.00 99.43
C
ANISOU12093  CD1 LEU B 753    14219  13459  10102  -6239  -4001
-656       C
ATOM  12094  CD2 LEU B 753      30.740  -5.349 125.225  1.00100.08
C
ANISOU12094  CD2 LEU B 753    14412  13476  10138  -6307  -4176
-386       C
ATOM  12095  N   MET B 754      27.227  -8.792 124.290  1.00 98.67
N
ANISOU12095  N   MET B 754    13783  13762   9946  -6773  -3904
387       N
ATOM  12096  CA  MET B 754      27.250  -9.679 123.125  1.00 95.31
C
ANISOU12096  CA  MET B 754    13161  13165   9889  -6576  -3814
533       C
ATOM  12097  C   MET B 754      25.866  -9.953 122.545  1.00126.98
C
ANISOU12097  C   MET B 754    17020  17281  13945  -6621  -3697
591       C
ATOM  12098  O   MET B 754      25.768 -10.358 121.382  1.00173.50
O
ANISOU12098  O   MET B 754    22753  23007  20162  -6394  -3599
613       O
ATOM  12099  CB  MET B 754      27.933 -11.010 123.450  1.00 95.33
C
ANISOU12099  CB  MET B 754    13139  13154   9929  -6639  -3895
820       C
ATOM  12100  CG  MET B 754      27.158 -11.930 124.360  1.00111.96
C
```

ANISOU12100 CG MET B 754   15244 15541 11755 -6977 -3952
1103   C
ATOM 12101 SD MET B 754   27.955 -13.545 124.455 1.00158.25
S
ANISOU12101 SD MET B 754   21039 21317 17773 -6977 -4050
1439   S
ATOM 12102 CE MET B 754   29.394 -13.162 125.448 1.00198.95
C
ANISOU12102 CE MET B 754   26400 26412 22780 -7035 -4217
1398   C
ATOM 12103 N   GLY B 755   24.800 -9.742 123.317 1.00 97.73
N
ANISOU12103 N   GLY B 755   13352 13866   9913 -6906 -3696
603   N
ATOM 12104 CA GLY B 755   23.463 -9.772 122.743 1.00 99.99
C
ANISOU12104 CA GLY B 755   13485 14253 10254 -6941 -3577
612   C
ATOM 12105 C   GLY B 755   23.231 -8.697 121.696 1.00120.84
C
ANISOU12105 C   GLY B 755   16079 16692 13141 -6683 -3489
337   C
ATOM 12106 O   GLY B 755   22.635 -8.958 120.648 1.00132.52
O
ANISOU12106 O   GLY B 755   17393 18072 14888 -6536 -3388
370   O
ATOM 12107 N   ALA B 756   23.695 -7.475 121.967 1.00155.83
N
ANISOU12107 N   ALA B 756   20658 21060 17490 -6619 -3537
62   N
ATOM 12108 CA ALA B 756   23.668 -6.415 120.961 1.00142.71
C
ANISOU12108 CA ALA B 756   18954 19176 16091 -6354 -3479
-187   C
ATOM 12109 C   ALA B 756   24.498 -6.761 119.728 1.00133.28
C
ANISOU12109 C   ALA B 756   17643 17697 15300 -6033 -3424
-146   C
ATOM 12110 O   ALA B 756   24.052 -6.544 118.595 1.00207.19
O
ANISOU12110 O   ALA B 756   26869 26933 24920 -5849 -3326
-206   O
ATOM 12111 CB ALA B 756   24.158 -5.103 121.575 1.00136.87
C
ANISOU12111 CB ALA B 756   18400 18413 15190 -6332 -3564
-478   C
ATOM 12112 N   VAL B 757   25.706 -7.292 119.925 1.00101.77
N
ANISOU12112 N   VAL B 757   13696 13619 11353 -5966 -3480
-57   N
ATOM 12113 CA VAL B 757   26.525 -7.740 118.798 1.00 86.60
C
ANISOU12113 CA VAL B 757   11694 11493   9718 -5750 -3435
16   C

```
ATOM  12114  C   VAL B 757      25.832  -8.841 117.997  1.00 96.97
C
ANISOU12114  C   VAL B 757    12862  12820  11163  -5748  -3356
222       C
ATOM  12115  O   VAL B 757      25.846  -8.824 116.760  1.00134.70
O
ANISOU12115  O   VAL B 757    17592  17477  16111  -5636  -3308
248       O
ATOM  12116  CB  VAL B 757      27.910  -8.190 119.297  1.00 87.30
C
ANISOU12116  CB  VAL B 757    11904  11528   9740  -5800  -3551
139       C
ATOM  12117  CG1 VAL B 757      28.746  -8.729 118.144  1.00 87.35
C
ANISOU12117  CG1 VAL B 757    11895  11368   9927  -5712  -3557
293       C
ATOM  12118  CG2 VAL B 757      28.625  -7.032 119.974  1.00 88.60
C
ANISOU12118  CG2 VAL B 757    12213  11658   9791  -5784  -3634
-73       C
ATOM  12119  N   TYR B 758      25.222  -9.814 118.678  1.00118.97
N
ANISOU12119  N   TYR B 758    15576  15761  13866  -5870  -3350
371       N
ATOM  12120  CA  TYR B 758      24.435 -10.833 117.983  1.00105.04
C
ANISOU12120  CA  TYR B 758    13664  14017  12228  -5864  -3280
554       C
ATOM  12121  C   TYR B 758      23.341 -10.228 117.108  1.00117.32
C
ANISOU12121  C   TYR B 758    15110  15550  13914  -5774  -3168
431       C
ATOM  12122  O   TYR B 758      23.193 -10.593 115.935  1.00129.92
O
ANISOU12122  O   TYR B 758    16641  17048  15675  -5680  -3125
510       O
ATOM  12123  CB  TYR B 758      23.818 -11.795 119.002  1.00 93.87
C
ANISOU12123  CB  TYR B 758    12228  12823  10615  -6119  -3329
787       C
ATOM  12124  CG  TYR B 758      22.548 -12.474 118.521  1.00 87.04
C
ANISOU12124  CG  TYR B 758    11195  12042   9832  -6154  -3246
923       C
ATOM  12125  CD1 TYR B 758      22.599 -13.634 117.763  1.00 85.14
C
ANISOU12125  CD1 TYR B 758    10822  11693   9833  -5992  -3203
1049       C
ATOM  12126  CD2 TYR B 758      21.294 -11.954 118.832  1.00 88.94
C
ANISOU12126  CD2 TYR B 758    11404  12479   9909  -6351  -3209
911       C
ATOM  12127  CE1 TYR B 758      21.439 -14.257 117.326  1.00103.71
C
```

ANISOU12127 CE1 TYR B 758   13019 14109 12277 -6004 -3131
1163   C
ATOM 12128 CE2 TYR B 758   20.131 -12.566 118.393 1.00127.67
C
ANISOU12128 CE2 TYR B 758   16137 17461 14910 -6378 -3130
1043   C
ATOM 12129 CZ TYR B 758   20.209 -13.719 117.642 1.00130.85
C
ANISOU12129 CZ TYR B 758   16417 17733 15566 -6197 -3096
1175   C
ATOM 12130 OH TYR B 758   19.058 -14.338 117.203 1.00110.23
O
ANISOU12130 OH TYR B 758   13637 15182 13064 -6206 -3022
1300   O
ATOM 12131 N   LEU B 759   22.561 -9.301 117.664 1.00107.66
N
ANISOU12131 N   LEU B 759   13927 14451 12528 -5911 -3172
305   N
ATOM 12132 CA LEU B 759   21.485 -8.667 116.905 1.00101.17
C
ANISOU12132 CA LEU B 759   13004 13609 11828 -5853 -3084
193   C
ATOM 12133 C   LEU B 759   22.017 -7.864 115.718 1.00112.88
C
ANISOU12133 C   LEU B 759   14468 14854 13569 -5567 -3036
3   C
ATOM 12134 O   LEU B 759   21.457 -7.929 114.617 1.00192.07
O
ANISOU12134 O   LEU B 759   24406 24814 23759 -5488 -2971
40   O
ATOM 12135 CB LEU B 759   20.644 -7.803 117.849 1.00101.41
C
ANISOU12135 CB LEU B 759   13112 13849 11568 -6121 -3124
94   C
ATOM 12136 CG LEU B 759   19.231 -7.365 117.441 1.00105.11
C
ANISOU12136 CG LEU B 759   13457 14395 12086 -6184 -3046
37   C
ATOM 12137 CD1 LEU B 759   19.206 -6.048 116.707 1.00 96.17
C
ANISOU12137 CD1 LEU B 759   12337 13076 11127 -5996 -3034
-233   C
ATOM 12138 CD2 LEU B 759   18.562 -8.454 116.609 1.00 87.52
C
ANISOU12138 CD2 LEU B 759   11033 12139 10080 -6104 -2953
244   C
ATOM 12139 N   GLN B 760   23.094 -7.102 115.920 1.00123.06
N
ANISOU12139 N   GLN B 760   15902 16056 14798 -5529 -3112
-113   N
ATOM 12140 CA GLN B 760   23.724 -6.367 114.822 1.00111.12
C
ANISOU12140 CA GLN B 760   14441 14369 13412 -5401 -3119
-177   C

```
ATOM  12141  C   GLN B 760      24.239  -7.289 113.719  1.00 88.49
C
ANISOU12141  C   GLN B 760    11552  11419  10653  -5350  -3107
33       C
ATOM  12142  O   GLN B 760      24.013  -7.037 112.530  1.00121.28
O
ANISOU12142  O   GLN B 760    15658  15485  14939  -5257  -3063
29       O
ATOM  12143  CB  GLN B 760      24.867  -5.512 115.382  1.00 83.04
C
ANISOU12143  CB  GLN B 760    11040  10748   9764  -5388  -3213
-310       C
ATOM  12144  CG  GLN B 760      25.443  -4.452 114.442  1.00 82.43
C
ANISOU12144  CG  GLN B 760    11012  10508   9800  -5269  -3228
-419       C
ATOM  12145  CD  GLN B 760      26.357  -5.036 113.377  1.00118.05
C
ANISOU12145  CD  GLN B 760    15538  14915  14400  -5217  -3232
-238       C
ATOM  12146  OE1 GLN B 760      27.224  -5.861 113.670  1.00 81.30
O
ANISOU12146  OE1 GLN B 760    10933  10264   9693  -5259  -3281
-96       O
ATOM  12147  NE2 GLN B 760      26.174  -4.599 112.137  1.00131.41
N
ANISOU12147  NE2 GLN B 760    17185  16518  16225  -5126  -3189
-251       N
ATOM  12148  N   VAL B 761      24.936  -8.362 114.093  1.00 81.03
N
ANISOU12148  N   VAL B 761    10639  10499   9649  -5408  -3158
209       N
ATOM  12149  CA  VAL B 761      25.389  -9.352 113.116  1.00120.05
C
ANISOU12149  CA  VAL B 761    15547  15371  14696  -5356  -3161
387       C
ATOM  12150  C   VAL B 761      24.218 -10.015 112.396  1.00129.70
C
ANISOU12150  C   VAL B 761    16626  16633  16020  -5335  -3082
473       C
ATOM  12151  O   VAL B 761      24.245 -10.191 111.171  1.00174.75
O
ANISOU12151  O   VAL B 761    22294  22258  21846  -5245  -3055
509       O
ATOM  12152  CB  VAL B 761      26.298 -10.390 113.798  1.00120.84
C
ANISOU12152  CB  VAL B 761    15696  15490  14728  -5429  -3249
548       C
ATOM  12153  CG1 VAL B 761      26.590 -11.549 112.853  1.00116.03
C
ANISOU12153  CG1 VAL B 761    15023  14820  14243  -5375  -3258
714       C
ATOM  12154  CG2 VAL B 761      27.597  -9.727 114.238  1.00 81.27
C
```

```
ANISOU12154  CG2 VAL B 761    10824  10404   9650  -5427  -3333
472       C
ATOM  12155  N   SER B 762      23.174 -10.385 113.137  1.00 89.86
N
ANISOU12155  N   SER B 762    11498  11719  10928  -5423  -3045
507       N
ATOM  12156  CA  SER B 762      22.014 -11.045 112.540  1.00106.39
C
ANISOU12156  CA  SER B 762    13446  13854  13125  -5411  -2973
600       C
ATOM  12157  C   SER B 762      21.323 -10.196 111.473  1.00 97.65
C
ANISOU12157  C   SER B 762    12285  12676  12141  -5313  -2903
479       C
ATOM  12158  O   SER B 762      20.977 -10.706 110.401  1.00 94.87
O
ANISOU12158  O   SER B 762    11864  12277  11904  -5247  -2874
561       O
ATOM  12159  CB  SER B 762      21.017 -11.433 113.633  1.00119.80
C
ANISOU12159  CB  SER B 762    15055  15721  14744  -5536  -2942
645       C
ATOM  12160  OG  SER B 762      19.891 -12.097 113.084  1.00 84.84
O
ANISOU12160  OG  SER B 762    10476  11331  10429  -5526  -2874
749       O
ATOM  12161  N   ILE B 763      21.095  -8.909 111.745  1.00 78.92
N
ANISOU12161  N   ILE B 763     9940  10293   9751  -5301  -2887
279       N
ATOM  12162  CA  ILE B 763      20.375  -8.071 110.781  1.00 78.28
C
ANISOU12162  CA  ILE B 763     9800  10142   9802  -5217  -2833
169       C
ATOM  12163  C   ILE B 763      21.112  -7.977 109.449  1.00 95.65
C
ANISOU12163  C   ILE B 763    12052  12211  12080  -5113  -2852
206       C
ATOM  12164  O   ILE B 763      20.521  -8.193 108.383  1.00109.47
O
ANISOU12164  O   ILE B 763    13726  13929  13940  -5059  -2812
259       O
ATOM  12165  CB  ILE B 763      20.116  -6.669 111.360  1.00 79.05
C
ANISOU12165  CB  ILE B 763     9921  10232   9881  -5217  -2837
-72       C
ATOM  12166  CG1 ILE B 763      19.162  -6.732 112.547  1.00 80.23
C
ANISOU12166  CG1 ILE B 763     9986  10530   9968  -5318  -2807
-133       C
ATOM  12167  CG2 ILE B 763      19.571  -5.739 110.288  1.00121.06
C
ANISOU12167  CG2 ILE B 763    15193  15450  15353  -5124  -2806
-178       C
```

```
ATOM  12168  CD1 ILE B 763      19.051  -5.411 113.268  1.00112.36
C
ANISOU12168  CD1 ILE B 763    14104  14604  13983  -5333  -2846
-392       C
ATOM  12169  N   ILE B 764      22.407  -7.662 109.476  1.00 86.93
N
ANISOU12169  N   ILE B 764    11073  11039  10918  -5091  -2915
182       N
ATOM  12170  CA  ILE B 764      23.107  -7.491 108.207  1.00 79.58
C
ANISOU12170  CA  ILE B 764    10180  10001  10056  -5003  -2929
207       C
ATOM  12171  C   ILE B 764      23.472  -8.824 107.561  1.00 75.69
C
ANISOU12171  C   ILE B 764     9655   9506   9596  -4988  -2936
379       C
ATOM  12172  O   ILE B 764      23.697  -8.876 106.346  1.00 95.41
O
ANISOU12172  O   ILE B 764    12144  11942  12167  -4920  -2928
406       O
ATOM  12173  CB  ILE B 764      24.347  -6.604 108.381  1.00 85.10
C
ANISOU12173  CB  ILE B 764    11007  10623  10703  -4982  -2991
115       C
ATOM  12174  CG1 ILE B 764      23.957  -5.308 109.087  1.00 89.76
C
ANISOU12174  CG1 ILE B 764    11625  11210  11271  -4993  -2998
-80       C
ATOM  12175  CG2 ILE B 764      24.879  -6.210 107.022  1.00 81.35
C
ANISOU12175  CG2 ILE B 764    10552  10053  10304  -4901  -2994
123       C
ATOM  12176  CD1 ILE B 764      22.989  -4.459 108.299  1.00 85.21
C
ANISOU12176  CD1 ILE B 764    10975  10592  10808  -4940  -2955
-174       C
ATOM  12177  N   SER B 765      23.494  -9.916 108.325  1.00 76.14
N
ANISOU12177  N   SER B 765     9690   9632   9608  -5052  -2957
493       N
ATOM  12178  CA  SER B 765      23.680 -11.230 107.722  1.00121.25
C
ANISOU12178  CA  SER B 765    15350  15336  15382  -5034  -2973
643       C
ATOM  12179  C   SER B 765      22.431 -11.695 106.987  1.00110.87
C
ANISOU12179  C   SER B 765    13914  14048  14162  -5010  -2914
696       C
ATOM  12180  O   SER B 765      22.492 -12.685 106.249  1.00 75.11
O
ANISOU12180  O   SER B 765     9335   9496   9707  -4977  -2925
794       O
ATOM  12181  CB  SER B 765      24.085 -12.256 108.782  1.00116.63
C
```

```
ANISOU12181  CB  SER B 765    14773  14805  14734  -5114  -3032
762       C
ATOM  12182  OG  SER B 765      25.290 -11.873 109.423  1.00112.95
O
ANISOU12182  OG  SER B 765    14422  14306  14188  -5137  -3097
723       O
ATOM  12183  N   GLN B 766      21.303 -11.019 107.193  1.00102.70
N
ANISOU12183  N   GLN B 766    12823  13058  13139  -5027  -2856
625       N
ATOM  12184  CA  GLN B 766      20.089 -11.249 106.424  1.00 75.23
C
ANISOU12184  CA  GLN B 766     9229   9592   9764  -4999  -2801
659       C
ATOM  12185  C   GLN B 766      19.877 -10.203 105.338  1.00 74.62
C
ANISOU12185  C   GLN B 766     9161   9439   9753  -4925  -2775
557       C
ATOM  12186  O   GLN B 766      19.258 -10.506 104.314  1.00 74.21
O
ANISOU12186  O   GLN B 766     9042   9366   9789  -4883  -2752
605       O
ATOM  12187  CB  GLN B 766      18.876 -11.236 107.363  1.00100.33
C
ANISOU12187  CB  GLN B 766    12309  12873  12937  -5074  -2751
657       C
ATOM  12188  CG  GLN B 766      17.644 -11.964 106.859  1.00 75.98
C
ANISOU12188  CG  GLN B 766     9085   9825   9961  -5070  -2706
757       C
ATOM  12189  CD  GLN B 766      17.783 -13.470 106.954  1.00 76.25
C
ANISOU12189  CD  GLN B 766     9079   9891  10001  -5096  -2745
938       C
ATOM  12190  OE1 GLN B 766      17.842 -14.029 108.049  1.00118.46
O
ANISOU12190  OE1 GLN B 766    14414  15321  15276  -5182  -2765
1016       O
ATOM  12191  NE2 GLN B 766      17.828 -14.135 105.807  1.00 75.72
N
ANISOU12191  NE2 GLN B 766     8988   9758  10023  -5029  -2764
1006       N
ATOM  12192  N   ALA B 767      20.379  -8.982 105.541  1.00 74.70
N
ANISOU12192  N   ALA B 767     9254   9404   9722  -4913  -2788
422       N
ATOM  12193  CA  ALA B 767      20.156  -7.880 104.609  1.00 77.91
C
ANISOU12193  CA  ALA B 767     9670   9739  10195  -4854  -2777
328       C
ATOM  12194  C   ALA B 767      20.999  -7.962 103.341  1.00 99.73
C
ANISOU12194  C   ALA B 767    12487  12432  12973  -4795  -2804
373       C
```

```
ATOM  12195  O   ALA B 767      20.647  -7.322 102.345  1.00139.51
O
ANISOU12195  O   ALA B 767    17513  17425  18071  -4754  -2796
345       O
ATOM  12196  CB  ALA B 767      20.427  -6.543 105.303  1.00 93.92
C
ANISOU12196  CB  ALA B 767    11763  11736  12186  -4863  -2796
163       C
ATOM  12197  N   LEU B 768      22.099  -8.716 103.349  1.00 94.69
N
ANISOU12197  N   LEU B 768    11901  11787  12288  -4795  -2839
439       N
ATOM  12198  CA  LEU B 768      22.977  -8.774 102.184  1.00112.95
C
ANISOU12198  CA  LEU B 768    14254  14044  14618  -4748  -2859
459       C
ATOM  12199  C   LEU B 768      22.455  -9.677 101.071  1.00118.35
C
ANISOU12199  C   LEU B 768    14859  14736  15375  -4721  -2838
542       C
ATOM  12200  O   LEU B 768      23.040  -9.687  99.983  1.00147.86
O
ANISOU12200  O   LEU B 768    18615  18437  19128  -4690  -2846
544       O
ATOM  12201  CB  LEU B 768      24.390  -9.207 102.609  1.00100.51
C
ANISOU12201  CB  LEU B 768    12749  12450  12989  -4758  -2906
477       C
ATOM  12202  CG  LEU B 768      24.799 -10.660 102.902  1.00106.32
C
ANISOU12202  CG  LEU B 768    13449  13210  13737  -4776  -2929
577       C
ATOM  12203  CD1 LEU B 768      23.715 -11.431 103.647  1.00 83.78
C
ANISOU12203  CD1 LEU B 768    10516  10425  10890  -4819  -2913
650       C
ATOM  12204  CD2 LEU B 768      25.234 -11.408 101.643  1.00 79.76
C
ANISOU12204  CD2 LEU B 768    10046   9814  10445  -4735  -2928
612       C
ATOM  12205  N   ILE B 769      21.378 -10.427 101.310  1.00104.57
N
ANISOU12205  N   ILE B 769    13021  13039  13673  -4740  -2813
606       N
ATOM  12206  CA  ILE B 769      20.685 -11.108 100.219  1.00101.78
C
ANISOU12206  CA  ILE B 769    12588  12685  13397  -4714  -2797
671       C
ATOM  12207  C   ILE B 769      20.191 -10.099  99.190  1.00 86.83
C
ANISOU12207  C   ILE B 769    10699  10758  11534  -4685  -2783
623       C
ATOM  12208  O   ILE B 769      20.313 -10.311  97.977  1.00 95.37
O
```

```
ANISOU12208  O   ILE B 769    11775  11818  12643  -4661  -2789
646       O
ATOM  12209  CB  ILE B 769      19.531 -11.967 100.767  1.00114.13
C
ANISOU12209  CB  ILE B 769    14047  14307  15010  -4744  -2777
752       C
ATOM  12210  CG1 ILE B 769      20.050 -12.943 101.822  1.00 95.70
C
ANISOU12210  CG1 ILE B 769    11713  12007  12640  -4785  -2806
818       C
ATOM  12211  CG2 ILE B 769      18.845 -12.717  99.636  1.00 72.83
C
ANISOU12211  CG2 ILE B 769     8738   9067   9868  -4717  -2772
819       C
ATOM  12212  CD1 ILE B 769      18.957 -13.669 102.571  1.00132.34
C
ANISOU12212  CD1 ILE B 769    16253  16717  17312  -4833  -2790
907       C
ATOM  12213  N   PHE B 770      19.612  -8.995  99.661  1.00105.20
N
ANISOU12213  N   PHE B 770    13030  13079  13864  -4694  -2770
552       N
ATOM  12214  CA  PHE B 770      19.175  -7.924  98.770  1.00 87.92
C
ANISOU12214  CA  PHE B 770    10846  10845  11714  -4671  -2773
507       C
ATOM  12215  C   PHE B 770      20.334  -7.353  97.957  1.00 89.14
C
ANISOU12215  C   PHE B 770    11097  10951  11821  -4652  -2808
483       C
ATOM  12216  O   PHE B 770      20.197  -7.114  96.752  1.00133.64
O
ANISOU12216  O   PHE B 770    16733  16566  17478  -4639  -2819
510       O
ATOM  12217  CB  PHE B 770      18.509  -6.812  99.586  1.00 92.98
C
ANISOU12217  CB  PHE B 770    11469  11475  12384  -4686  -2760
406       C
ATOM  12218  CG  PHE B 770      17.269  -7.248 100.327  1.00113.16
C
ANISOU12218  CG  PHE B 770    13909  14085  15000  -4716  -2715
418       C
ATOM  12219  CD1 PHE B 770      16.012  -6.916  99.854  1.00108.03
C
ANISOU12219  CD1 PHE B 770    13163  13426  14459  -4710  -2693
416       C
ATOM  12220  CD2 PHE B 770      17.363  -7.973 101.506  1.00128.10
C
ANISOU12220  CD2 PHE B 770    15783  16042  16845  -4757  -2698
438       C
ATOM  12221  CE1 PHE B 770      14.873  -7.310 100.535  1.00125.99
C
ANISOU12221  CE1 PHE B 770    15315  15754  16802  -4743  -2644
427       C
```

```
ATOM  12222  CE2 PHE B 770      16.227  -8.370 102.190  1.00110.00
C
ANISOU12222  CE2 PHE B 770    13375  13813  14606  -4797  -2653
458       C
ATOM  12223  CZ  PHE B 770      14.981  -8.036 101.705  1.00110.34
C
ANISOU12223  CZ  PHE B 770    13312  13846  14766  -4789  -2620
448       C
ATOM  12224  N   VAL B 771      21.478  -7.114  98.603  1.00 72.38
N
ANISOU12224  N   VAL B 771     9055   8815   9631  -4658  -2828
439       N
ATOM  12225  CA  VAL B 771      22.659  -6.608  97.901  1.00 90.58
C
ANISOU12225  CA  VAL B 771    11443  11079  11895  -4647  -2860
421       C
ATOM  12226  C   VAL B 771      23.160  -7.589  96.844  1.00 97.03
C
ANISOU12226  C   VAL B 771    12240  11911  12715  -4641  -2854
483       C
ATOM  12227  O   VAL B 771      23.384  -7.212  95.688  1.00103.88
O
ANISOU12227  O   VAL B 771    13126  12763  13579  -4639  -2863
490       O
ATOM  12228  CB  VAL B 771      23.769  -6.257  98.907  1.00 90.16
C
ANISOU12228  CB  VAL B 771    11470  11006  11780  -4658  -2887
366       C
ATOM  12229  CG1 VAL B 771      25.017  -5.798  98.168  1.00 80.03
C
ANISOU12229  CG1 VAL B 771    10259   9683  10465  -4651  -2919
355       C
ATOM  12230  CG2 VAL B 771      23.285  -5.178  99.866  1.00105.55
C
ANISOU12230  CG2 VAL B 771    13438  12935  13731  -4668  -2897
273       C
ATOM  12231  N   THR B 772      23.358  -8.855  97.220  1.00106.59
N
ANISOU12231  N   THR B 772    13409  13152  13936  -4644  -2843
523       N
ATOM  12232  CA  THR B 772      23.908  -9.827  96.274  1.00 88.65
C
ANISOU12232  CA  THR B 772    11107  10886  11689  -4638  -2838
550       C
ATOM  12233  C   THR B 772      22.987 -10.073  95.081  1.00 91.64
C
ANISOU12233  C   THR B 772    11427  11279  12114  -4634  -2822
586       C
ATOM  12234  O   THR B 772      23.456 -10.174  93.941  1.00118.25
O
ANISOU12234  O   THR B 772    14800  14647  15482  -4639  -2819
574       O
ATOM  12235  CB  THR B 772      24.201 -11.148  96.988  1.00 82.61
C
```

```
ANISOU12235  CB  THR B 772    10296  10137  10953  -4642  -2843
585       C
ATOM  12236  OG1 THR B 772      23.066 -11.535  97.771  1.00142.06
O
ANISOU12236  OG1 THR B 772    17772  17698  18506  -4652  -2834
636       O
ATOM  12237  CG2 THR B 772      25.415 -11.007  97.893  1.00125.78
C
ANISOU12237  CG2 THR B 772    15829  15585  16376  -4651  -2872
553       C
ATOM  12238  N   ARG B 773      21.679 -10.182  95.318  1.00101.53
N
ANISOU12238  N   ARG B 773    12621  12549  13409  -4633  -2811
626       N
ATOM  12239  CA  ARG B 773      20.724 -10.275  94.213  1.00 95.75
C
ANISOU12239  CA  ARG B 773    11837  11822  12721  -4632  -2807
666       C
ATOM  12240  C   ARG B 773      20.774  -9.059  93.296  1.00103.96
C
ANISOU12240  C   ARG B 773    12932  12838  13729  -4639  -2825
645       C
ATOM  12241  O   ARG B 773      20.803  -9.193  92.068  1.00 92.03
O
ANISOU12241  O   ARG B 773    11418  11334  12214  -4653  -2832
665       O
ATOM  12242  CB  ARG B 773      19.305 -10.459  94.752  1.00107.52
C
ANISOU12242  CB  ARG B 773    13249  13330  14274  -4631  -2795
711       C
ATOM  12243  CG  ARG B 773      18.868 -11.893  94.935  1.00 89.88
C
ANISOU12243  CG  ARG B 773    10930  11123  12099  -4633  -2787
775       C
ATOM  12244  CD  ARG B 773      19.886 -12.770  95.630  1.00115.21
C
ANISOU12244  CD  ARG B 773    14150  14335  15290  -4636  -2794
772       C
ATOM  12245  NE  ARG B 773      19.395 -14.102  95.716  1.00157.67
N
ANISOU12245  NE  ARG B 773    19439  19726  20742  -4639  -2801
842       N
ATOM  12246  CZ  ARG B 773      19.709 -15.278  95.209  1.00151.21
C
ANISOU12246  CZ  ARG B 773    18573  18895  19984  -4634  -2816
860       C
ATOM  12247  NH1 ARG B 773      18.715 -16.074  94.863  1.00153.85
N
ANISOU12247  NH1 ARG B 773    18820  19234  20400  -4635  -2823
926       N
ATOM  12248  NH2 ARG B 773      20.963 -15.705  95.174  1.00140.97
N
ANISOU12248  NH2 ARG B 773    17303  17576  18683  -4631  -2828
812       N
```

```
ATOM  12249  N   SER B 774      20.793  -7.865  93.874  1.00 95.26
N
ANISOU12249  N   SER B 774    11882  11708  12606  -4638  -2839
605       N
ATOM  12250  CA  SER B 774      20.809  -6.647  93.077  1.00 97.83
C
ANISOU12250  CA  SER B 774    12258  12000  12914  -4649  -2873
596       C
ATOM  12251  C   SER B 774      22.116  -6.447  92.318  1.00 95.08
C
ANISOU12251  C   SER B 774    11980  11649  12496  -4668  -2888
583       C
ATOM  12252  O   SER B 774      23.207  -6.713  92.828  1.00 98.52
O
ANISOU12252  O   SER B 774    12450  12085  12897  -4665  -2879
546       O
ATOM  12253  CB  SER B 774      20.545  -5.437  93.974  1.00 89.05
C
ANISOU12253  CB  SER B 774    11173  10844  11818  -4642  -2891
537       C
ATOM  12254  OG  SER B 774      20.677  -4.229  93.249  1.00100.15
O
ANISOU12254  OG  SER B 774    12631  12204  13218  -4654  -2939
532       O
ATOM  12255  N   ARG B 775      21.984  -5.974  91.077  1.00101.01
N
ANISOU12255  N   ARG B 775    12749  12399  13231  -4696  -2913
619       N
ATOM  12256  CA  ARG B 775      23.109  -5.537  90.261  1.00 86.33
C
ANISOU12256  CA  ARG B 775    10955  10543  11303  -4731  -2931
611       C
ATOM  12257  C   ARG B 775      23.249  -4.024  90.281  1.00 73.41
C
ANISOU12257  C   ARG B 775     9390   8854   9650  -4744  -2987
609       C
ATOM  12258  O   ARG B 775      24.352  -3.501  90.089  1.00116.00
O
ANISOU12258  O   ARG B 775    14847  14237  14990  -4768  -3006
591       O
ATOM  12259  CB  ARG B 775      22.919  -5.987  88.806  1.00101.36
C
ANISOU12259  CB  ARG B 775    12837  12490  13185  -4775  -2928
656       C
ATOM  12260  CG  ARG B 775      23.026  -7.483  88.536  1.00114.30
C
ANISOU12260  CG  ARG B 775    14406  14174  14848  -4772  -2879
638       C
ATOM  12261  CD  ARG B 775      24.407  -8.023  88.833  1.00123.57
C
ANISOU12261  CD  ARG B 775    15588  15358  16006  -4771  -2848
568       C
ATOM  12262  NE  ARG B 775      24.547  -9.410  88.399  1.00149.84
N
```

```
ANISOU12262  NE  ARG B 775    18838  18717  19378  -4774  -2809
535       N
ATOM  12263  CZ  ARG B 775      25.176  -9.796  87.294  1.00147.65
C
ANISOU12263  CZ  ARG B 775    18542  18476  19083  -4821  -2787
491       C
ATOM  12264  NH1 ARG B 775      25.501  -8.906  86.367  1.00144.59
N
ANISOU12264  NH1 ARG B 775    18212  18112  18616  -4879  -2803
504       N
ATOM  12265  NH2 ARG B 775      25.275 -11.088  87.013  1.00144.38
N
ANISOU12265  NH2 ARG B 775    18043  18077  18738  -4819  -2755
439       N
ATOM  12266  N   SER B 776      22.135  -3.329  90.507  1.00 73.60
N
ANISOU12266  N   SER B 776     9392   8838   9733  -4730  -3018
623       N
ATOM  12267  CA  SER B 776      22.066  -1.893  90.734  1.00108.78
C
ANISOU12267  CA  SER B 776    13894  13222  14213  -4732  -3080
602       C
ATOM  12268  C   SER B 776      22.005  -1.658  92.238  1.00113.85
C
ANISOU12268  C   SER B 776    14530  13832  14898  -4692  -3063
511       C
ATOM  12269  O   SER B 776      22.714  -2.323  93.001  1.00131.46
O
ANISOU12269  O   SER B 776    16769  16088  17091  -4678  -3024
473       O
ATOM  12270  CB  SER B 776      20.854  -1.281  90.029  1.00112.54
C
ANISOU12270  CB  SER B 776    14338  13669  14755  -4745  -3129
659       C
ATOM  12271  OG  SER B 776      20.913   0.134  90.060  1.00151.58
O
ANISOU12271  OG  SER B 776    19327  18533  19732  -4754  -3205
644       O
ATOM  12272  N   TRP B 777      21.174  -0.716  92.677  1.00110.44
N
ANISOU12272  N   TRP B 777    14075  13339  14547  -4679  -3096
468       N
ATOM  12273  CA  TRP B 777      20.957  -0.525  94.103  1.00106.00
C
ANISOU12273  CA  TRP B 777    13491  12756  14027  -4653  -3072
360       C
ATOM  12274  C   TRP B 777      19.885  -1.486  94.610  1.00128.13
C
ANISOU12274  C   TRP B 777    16195  15611  16879  -4640  -3008
362       C
ATOM  12275  O   TRP B 777      18.895  -1.757  93.925  1.00117.48
O
ANISOU12275  O   TRP B 777    14780  14274  15585  -4643  -3004
424       O
```

```
ATOM  12276  CB  TRP B 777      20.544   0.916  94.385  1.00 91.41
C
ANISOU12276  CB  TRP B 777    11649  10819  12264  -4649  -3132
281       C
ATOM  12277  CG  TRP B 777      21.547   1.937  93.939  1.00 75.74
C
ANISOU12277  CG  TRP B 777     9760   8773  10245  -4665  -3210
287       C
ATOM  12278  CD1 TRP B 777      21.580   2.584  92.739  1.00 97.68
C
ANISOU12278  CD1 TRP B 777    12569  11518  13027  -4694  -3280
375       C
ATOM  12279  CD2 TRP B 777      22.648   2.450  94.702  1.00 97.65
C
ANISOU12279  CD2 TRP B 777    12610  11513  12980  -4661  -3234
210       C
ATOM  12280  NE1 TRP B 777      22.641   3.458  92.700  1.00137.23
N
ANISOU12280  NE1 TRP B 777    17664  16474  18001  -4710  -3345
362       N
ATOM  12281  CE2 TRP B 777      23.311   3.396  93.894  1.00113.29
C
ANISOU12281  CE2 TRP B 777    14660  13437  14947  -4687  -3318
258       C
ATOM  12282  CE3 TRP B 777      23.140   2.198  95.986  1.00103.13
C
ANISOU12282  CE3 TRP B 777    13322  12218  13644  -4645  -3202
114       C
ATOM  12283  CZ2 TRP B 777      24.440   4.090  94.328  1.00 96.82
C
ANISOU12283  CZ2 TRP B 777    12656  11300  12831  -4691  -3368
210       C
ATOM  12284  CZ3 TRP B 777      24.262   2.888  96.416  1.00104.84
C
ANISOU12284  CZ3 TRP B 777    13624  12385  13826  -4649  -3254
62       C
ATOM  12285  CH2 TRP B 777      24.899   3.823  95.589  1.00 99.19
C
ANISOU12285  CH2 TRP B 777    12971  11608  13109  -4669  -3335
109       C
ATOM  12286  N   SER B 778      20.106  -2.005  95.820  1.00133.52
N
ANISOU12286  N   SER B 778    16868  16323  17539  -4632  -2965
303       N
ATOM  12287  CA  SER B 778      19.210  -2.994  96.419  1.00101.36
C
ANISOU12287  CA  SER B 778    12703  12309  13502  -4632  -2906
316       C
ATOM  12288  C   SER B 778      17.780  -2.480  96.572  1.00116.61
C
ANISOU12288  C   SER B 778    14535  14218  15552  -4632  -2896
274       C
ATOM  12289  O   SER B 778      16.817  -3.187  96.255  1.00124.93
O
```

```
ANISOU12289  O   SER B 778    15501  15307  16659  -4634  -2866
340       O
ATOM  12290  CB  SER B 778      19.763  -3.425  97.775  1.00 81.50
C
ANISOU12290  CB  SER B 778    10207   9826  10933  -4639  -2879
259       C
ATOM  12291  OG  SER B 778      21.074  -3.938  97.643  1.00 73.04
O
ANISOU12291  OG  SER B 778     9213   8767   9772  -4639  -2891
298       O
ATOM  12292  N   PHE B 779      17.626  -1.249  97.067  1.00 99.19
N
ANISOU12292  N   PHE B 779    12336  11950  13403  -4631  -2923
156       N
ATOM  12293  CA  PHE B 779      16.297  -0.668  97.257  1.00103.36
C
ANISOU12293  CA  PHE B 779    12755  12447  14070  -4634  -2912
86       C
ATOM  12294  C   PHE B 779      15.536  -0.513  95.946  1.00151.44
C
ANISOU12294  C   PHE B 779    18803  18505  20230  -4632  -2947
188       C
ATOM  12295  O   PHE B 779      14.318  -0.722  95.901  1.00184.75
O
ANISOU12295  O   PHE B 779    22908  22734  24555  -4636  -2918
199       O
ATOM  12296  CB  PHE B 779      16.404   0.673  97.991  1.00101.19
C
ANISOU12296  CB  PHE B 779    12496  12099  13852  -4633  -2945
-88       C
ATOM  12297  CG  PHE B 779      17.500   1.572  97.486  1.00113.02
C
ANISOU12297  CG  PHE B 779    14117  13527  15299  -4624  -3026
-87       C
ATOM  12298  CD1 PHE B 779      18.776   1.496  98.021  1.00 76.08
C
ANISOU12298  CD1 PHE B 779     9541   8858  10506  -4623  -3037
-111       C
ATOM  12299  CD2 PHE B 779      17.248   2.511  96.498  1.00111.23
C
ANISOU12299  CD2 PHE B 779    13899  13220  15142  -4623  -3098
-54       C
ATOM  12300  CE1 PHE B 779      19.783   2.326  97.569  1.00101.66
C
ANISOU12300  CE1 PHE B 779    12885  12034  13709  -4620  -3111
-103       C
ATOM  12301  CE2 PHE B 779      18.254   3.346  96.041  1.00105.06
C
ANISOU12301  CE2 PHE B 779    13226  12376  14316  -4624  -3179
-38       C
ATOM  12302  CZ  PHE B 779      19.520   3.256  96.579  1.00 93.17
C
ANISOU12302  CZ  PHE B 779    11816  10884  12701  -4623  -3182
-66       C
```

```
ATOM  12303  N   VAL B 780      16.230  -0.139  94.873  1.00175.36
N
ANISOU12303  N   VAL B 780    21922  21501  23204  -4632  -3013
267       N
ATOM  12304  CA  VAL B 780      15.597  -0.056  93.558  1.00154.92
C
ANISOU12304  CA  VAL B 780    19311  18894  20658  -4642  -3060
383       C
ATOM  12305  C   VAL B 780      15.062  -1.414  93.107  1.00129.63
C
ANISOU12305  C   VAL B 780    16050  15767  17437  -4644  -3015
491       C
ATOM  12306  O   VAL B 780      13.938  -1.514  92.599  1.00142.70
O
ANISOU12306  O   VAL B 780    17621  17414  19183  -4648  -3025
547       O
ATOM  12307  CB  VAL B 780      16.589   0.527  92.533  1.00128.25
C
ANISOU12307  CB  VAL B 780    16049  15485  17197  -4659  -3138
452       C
ATOM  12308  CG1 VAL B 780      16.011   0.449  91.130  1.00125.18
C
ANISOU12308  CG1 VAL B 780    15646  15095  16821  -4683  -3191
588       C
ATOM  12309  CG2 VAL B 780      16.939   1.963  92.896  1.00131.43
C
ANISOU12309  CG2 VAL B 780    16497  15794  17648  -4657  -3203
358       C
ATOM  12310  N   GLU B 781      15.840  -2.477  93.298  1.00103.38
N
ANISOU12310  N   GLU B 781    12762  12510  14008  -4641  -2972
519       N
ATOM  12311  CA  GLU B 781      15.735  -3.671  92.471  1.00 99.83
C
ANISOU12311  CA  GLU B 781    12294  12116  13519  -4648  -2959
629       C
ATOM  12312  C   GLU B 781      15.216  -4.911  93.203  1.00 84.03
C
ANISOU12312  C   GLU B 781    10209  10175  11544  -4639  -2893
642       C
ATOM  12313  O   GLU B 781      15.077  -5.965  92.573  1.00 81.56
O
ANISOU12313  O   GLU B 781     9871   9902  11217  -4642  -2887
724       O
ATOM  12314  CB  GLU B 781      17.119  -3.941  91.855  1.00109.01
C
ANISOU12314  CB  GLU B 781    13559  13302  14556  -4659  -2972
655       C
ATOM  12315  CG  GLU B 781      17.234  -5.052  90.826  1.00119.80
C
ANISOU12315  CG  GLU B 781    14919  14722  15880  -4675  -2965
738       C
ATOM  12316  CD  GLU B 781      18.494  -4.927  89.989  1.00119.60
C
```

```
ANISOU12316  CD  GLU B 781    14983  14708  15750  -4703  -2985
746       C
ATOM  12317  OE1 GLU B 781      19.602  -4.946  90.566  1.00 75.39
O
ANISOU12317  OE1 GLU B 781     9436   9112  10096  -4696  -2965
691       O
ATOM  12318  OE2 GLU B 781      18.378  -4.810  88.752  1.00152.38
O
ANISOU12318  OE2 GLU B 781    19152  18869  19877  -4740  -3021
807       O
ATOM  12319  N   ARG B 782      14.889  -4.826  94.497  1.00116.92
N
ANISOU12319  N   ARG B 782    14325  14349  15751  -4636  -2849
564       N
ATOM  12320  CA  ARG B 782      14.534  -6.005  95.293  1.00158.40
C
ANISOU12320  CA  ARG B 782    19506  19667  21013  -4641  -2793
590       C
ATOM  12321  C   ARG B 782      13.125  -5.931  95.886  1.00119.55
C
ANISOU12321  C   ARG B 782    14451  14755  16217  -4652  -2754
571       C
ATOM  12322  O   ARG B 782      12.960  -5.874  97.113  1.00108.33
O
ANISOU12322  O   ARG B 782    12987  13362  14812  -4670  -2707
496       O
ATOM  12323  CB  ARG B 782      15.563  -6.224  96.400  1.00178.15
C
ANISOU12323  CB  ARG B 782    22068  22195  23427  -4646  -2772
532       C
ATOM  12324  CG  ARG B 782      16.967  -6.480  95.879  1.00129.76
C
ANISOU12324  CG  ARG B 782    16049  16062  17192  -4637  -2800
554       C
ATOM  12325  CD  ARG B 782      16.955  -7.643  94.904  1.00118.43
C
ANISOU12325  CD  ARG B 782    14590  14656  15753  -4634  -2803
653       C
ATOM  12326  NE  ARG B 782      16.397  -8.847  95.511  1.00108.00
N
ANISOU12326  NE  ARG B 782    13184  13381  14468  -4640  -2771
705       N
ATOM  12327  CZ  ARG B 782      15.712  -9.768  94.844  1.00 72.70
C
ANISOU12327  CZ  ARG B 782     8643   8928  10052  -4638  -2772
786       C
ATOM  12328  NH1 ARG B 782      15.219 -10.822  95.479  1.00 88.13
N
ANISOU12328  NH1 ARG B 782    10520  10921  12046  -4647  -2751
840       N
ATOM  12329  NH2 ARG B 782      15.500  -9.625  93.544  1.00 74.59
N
ANISOU12329  NH2 ARG B 782     8890   9147  10305  -4635  -2801
820       N
```

```
ATOM  12330  N   PRO B 783      12.080  -5.943  95.045  1.00100.14
N
ANISOU12330  N   PRO B 783    11918  12278  13853  -4651  -2770
638       N
ATOM  12331  CA  PRO B 783      10.708  -5.948  95.575  1.00169.88
C
ANISOU12331  CA  PRO B 783    20602  21121  22822  -4666  -2725
627       C
ATOM  12332  C   PRO B 783      10.234  -7.300  96.101  1.00170.81
C
ANISOU12332  C   PRO B 783    20633  21314  22952  -4681  -2675
707       C
ATOM  12333  O   PRO B 783       9.023  -7.526  96.187  1.00174.49
O
ANISOU12333  O   PRO B 783    20967  21794  23537  -4694  -2644
746       O
ATOM  12334  CB  PRO B 783       9.884  -5.507  94.363  1.00180.01
C
ANISOU12334  CB  PRO B 783    21850  22351  24195  -4659  -2779
692       C
ATOM  12335  CG  PRO B 783      10.600  -6.156  93.227  1.00165.64
C
ANISOU12335  CG  PRO B 783    20126  20540  22270  -4650  -2832
795       C
ATOM  12336  CD  PRO B 783      12.078  -6.023  93.572  1.00123.92
C
ANISOU12336  CD  PRO B 783    14968  15265  16851  -4644  -2831
733       C
ATOM  12337  N   GLY B 784      11.148  -8.206  96.451  1.00107.64
N
ANISOU12337  N   GLY B 784    12697  13359  14843  -4681  -2671
741       N
ATOM  12338  CA  GLY B 784      10.745  -9.539  96.870  1.00101.60
C
ANISOU12338  CA  GLY B 784    11853  12656  14093  -4698  -2644
837       C
ATOM  12339  C   GLY B 784       9.967  -9.627  98.170  1.00139.81
C
ANISOU12339  C   GLY B 784    16575  17553  18993  -4739  -2573
812       C
ATOM  12340  O   GLY B 784      10.538  -9.531  99.260  1.00187.93
O
ANISOU12340  O   GLY B 784    22701  23685  25019  -4766  -2547
748       O
ATOM  12341  N   ALA B 785       8.648  -9.807  98.052  1.00113.44
N
ANISOU12341  N   ALA B 785    13093  14224  15783  -4752  -2543
865       N
ATOM  12342  CA  ALA B 785       7.782  -9.936  99.224  1.00 91.35
C
ANISOU12342  CA  ALA B 785    10153  11495  13060  -4803  -2460
852       C
ATOM  12343  C   ALA B 785       8.135 -11.151 100.081  1.00 97.57
C
```

```
ANISOU12343  C   ALA B 785    10933  12366  13773  -4838  -2446
943       C
ATOM  12344  O   ALA B 785       8.142 -11.067 101.315  1.00 87.58
O
ANISOU12344  O   ALA B 785     9625  11169  12481  -4891  -2390
896       O
ATOM  12345  CB  ALA B 785       6.321 -10.000  98.779  1.00127.42
C
ANISOU12345  CB  ALA B 785    14564  16058  17794  -4808  -2432
917       C
ATOM  12346  N   LEU B 786       8.417 -12.293  99.446  1.00 84.97
N
ANISOU12346  N   LEU B 786     9370  10766  12147  -4816  -2502
1073       N
ATOM  12347  CA  LEU B 786       8.845 -13.486 100.177  1.00101.26
C
ANISOU12347  CA  LEU B 786    11434  12893  14149  -4847  -2511
1170       C
ATOM  12348  C   LEU B 786      10.144 -13.261 100.947  1.00 94.37
C
ANISOU12348  C   LEU B 786    10684  12035  13135  -4862  -2523
1095       C
ATOM  12349  O   LEU B 786      10.258 -13.655 102.113  1.00104.30
O
ANISOU12349  O   LEU B 786    11915  13369  14344  -4920  -2498
1123       O
ATOM  12350  CB  LEU B 786       8.993 -14.657  99.202  1.00118.80
C
ANISOU12350  CB  LEU B 786    13671  15079  16389  -4812  -2582
1293       C
ATOM  12351  CG  LEU B 786       9.439 -16.005  99.773  1.00 99.24
C
ANISOU12351  CG  LEU B 786    11186  12642  13878  -4837  -2615
1406       C
ATOM  12352  CD1 LEU B 786       8.458 -16.503 100.822  1.00 77.80
C
ANISOU12352  CD1 LEU B 786     8326  10014  11218  -4900  -2565
1505       C
ATOM  12353  CD2 LEU B 786       9.600 -17.028  98.660  1.00 76.65
C
ANISOU12353  CD2 LEU B 786     8337   9723  11064  -4796  -2689
1488       C
ATOM  12354  N   LEU B 787      11.134 -12.634 100.312  1.00121.36
N
ANISOU12354  N   LEU B 787    14237  15387  16486  -4817  -2563
1010       N
ATOM  12355  CA  LEU B 787      12.366 -12.277 101.010  1.00 89.88
C
ANISOU12355  CA  LEU B 787    10369  11405  12376  -4829  -2577
932       C
ATOM  12356  C   LEU B 787      12.122 -11.282 102.144  1.00117.65
C
ANISOU12356  C   LEU B 787    13864  14960  15878  -4874  -2525
813       C
```

```
ATOM  12357  O   LEU B 787      12.691 -11.423 103.234  1.00128.50           O
ANISOU12357  O   LEU B 787    15275  16388  17160  -4922  -2523    797       O
ATOM  12358  CB  LEU B 787      13.378 -11.713 100.013  1.00 96.84           C
ANISOU12358  CB  LEU B 787    11381  12208  13206  -4773  -2624    870       C
ATOM  12359  CG  LEU B 787      14.755 -11.358 100.577  1.00107.74           C
ANISOU12359  CG  LEU B 787    12890  13580  14468  -4777  -2647    799       C
ATOM  12360  CD1 LEU B 787      15.401 -12.573 101.224  1.00116.78           C
ANISOU12360  CD1 LEU B 787    14046  14766  15560  -4804  -2671    887       C
ATOM  12361  CD2 LEU B 787      15.653 -10.781  99.494  1.00 89.69           C
ANISOU12361  CD2 LEU B 787    10708  11222  12147  -4728  -2683    751       C
ATOM  12362  N   MET B 788      11.283 -10.272 101.908  1.00 93.50           N
ANISOU12362  N   MET B 788    10739  11871  12914  -4865  -2488    722       N
ATOM  12363  CA  MET B 788      10.867  -9.357 102.972  1.00 94.35           C
ANISOU12363  CA  MET B 788    10788  12013  13049  -4912  -2432    585       C
ATOM  12364  C   MET B 788      10.203 -10.072 104.147  1.00110.69           C
ANISOU12364  C   MET B 788    12732  14200  15126  -4993  -2368    647       C
ATOM  12365  O   MET B 788      10.512  -9.784 105.309  1.00113.28           O
ANISOU12365  O   MET B 788    13072  14589  15380  -5051  -2348    570       O
ATOM  12366  CB  MET B 788       9.923  -8.296 102.404  1.00 76.62           C
ANISOU12366  CB  MET B 788     8458   9704  10951  -4888  -2405    488       C
ATOM  12367  CG  MET B 788      10.623  -7.157 101.694  1.00 91.64           C
ANISOU12367  CG  MET B 788    10481  11504  11504  -4835  -2462    378       C
ATOM  12368  SD  MET B 788      11.480  -6.084 102.865  1.00133.89           S
ANISOU12368  SD  MET B 788    15916  16848  18108  -4858  -2471    178       S
ATOM  12369  CE  MET B 788      10.101  -5.176 103.564  1.00 77.64           C
ANISOU12369  CE  MET B 788     8601   9731  11168  -4897  -2397     11       C
ATOM  12370  N   ILE B 789       9.286 -11.000 103.874  1.00 85.82           N
```

```
ANISOU12370  N   ILE B 789     9460  11087  12059  -5004  -2341
793       N
ATOM  12371  CA  ILE B 789       8.704 -11.800 104.952  1.00 78.56
C
ANISOU12371  CA  ILE B 789     8419  10292  11140  -5087  -2282
891       C
ATOM  12372  C   ILE B 789       9.764 -12.621 105.682  1.00106.08
C
ANISOU12372  C   ILE B 789    12007  13834  14464  -5127  -2335
971       C
ATOM  12373  O   ILE B 789       9.791 -12.664 106.917  1.00130.84
O
ANISOU12373  O   ILE B 789    15111  17075  17528  -5213  -2299
966       O
ATOM  12374  CB  ILE B 789       7.586 -12.703 104.399  1.00100.79
C
ANISOU12374  CB  ILE B 789    11092  13124  14080  -5081  -2259
1053       C
ATOM  12375  CG1 ILE B 789       6.387 -11.863 103.949  1.00145.23
C
ANISOU12375  CG1 ILE B 789    16585  18716  19880  -5065  -2190
976       C
ATOM  12376  CG2 ILE B 789       7.174 -13.741 105.435  1.00 80.16
C
ANISOU12376  CG2 ILE B 789     8371  10641  11446  -5166  -2217
1202       C
ATOM  12377  CD1 ILE B 789       5.690 -11.132 105.078  1.00182.35
C
ANISOU12377  CD1 ILE B 789    21139  23495  24651  -5138  -2073
863       C
ATOM  12378  N   ALA B 790      10.659 -13.274 104.939  1.00 87.58
N
ANISOU12378  N   ALA B 790     9783  11427  12068  -5072  -2422
1045       N
ATOM  12379  CA  ALA B 790      11.755 -14.010 105.569  1.00 77.63
C
ANISOU12379  CA  ALA B 790     8621  10199  10677  -5104  -2483
1113       C
ATOM  12380  C   ALA B 790      12.662 -13.110 106.406  1.00 87.21
C
ANISOU12380  C   ALA B 790     9947  11425  11764  -5135  -2489
973       C
ATOM  12381  O   ALA B 790      13.029 -13.459 107.533  1.00 78.16
O
ANISOU12381  O   ALA B 790     8818  10365  10515  -5215  -2498
1012       O
ATOM  12382  CB  ALA B 790      12.568 -14.744 104.501  1.00 76.86
C
ANISOU12382  CB  ALA B 790     8612  10011  10580  -5031  -2566
1182       C
ATOM  12383  N   PHE B 791      13.034 -11.947 105.869  1.00 99.29
N
ANISOU12383  N   PHE B 791    11557  12868  13299  -5077  -2494
817       N
```

```
ATOM  12384  CA  PHE B 791      13.794 -10.962 106.638  1.00 88.09
C
ANISOU12384  CA  PHE B 791    10239  11449  11780  -5100  -2505
664       C
ATOM  12385  C   PHE B 791      13.035 -10.455 107.862  1.00 78.02
C
ANISOU12385  C   PHE B 791     8867  10275  10502  -5187  -2441
576       C
ATOM  12386  O   PHE B 791      13.583 -10.412 108.970  1.00 87.34
O
ANISOU12386  O   PHE B 791    10104  11528  11555  -5258  -2459
543       O
ATOM  12387  CB  PHE B 791      14.189  -9.798 105.727  1.00 99.98
C
ANISOU12387  CB  PHE B 791    11829  12838  13322  -5017  -2527
526       C
ATOM  12388  CG  PHE B 791      14.934  -8.696 106.427  1.00 76.43
C
ANISOU12388  CG  PHE B 791     8948   9834  10257  -5029  -2551
358       C
ATOM  12389  CD1 PHE B 791      16.319  -8.713 106.496  1.00 76.04
C
ANISOU12389  CD1 PHE B 791     9052   9748  10093  -5013  -2615
358       C
ATOM  12390  CD2 PHE B 791      14.254  -7.622 106.982  1.00 77.12
C
ANISOU12390  CD2 PHE B 791     8971   9929  10402  -5051  -2515
187       C
ATOM  12391  CE1 PHE B 791      17.009  -7.696 107.130  1.00 76.36
C
ANISOU12391  CE1 PHE B 791     9188   9763  10061  -5020  -2646
205       C
ATOM  12392  CE2 PHE B 791      14.937  -6.602 107.616  1.00 77.47
C
ANISOU12392  CE2 PHE B 791     9112   9945  10377  -5055  -2554
14       C
ATOM  12393  CZ  PHE B 791      16.317  -6.639 107.689  1.00 77.08
C
ANISOU12393  CZ  PHE B 791     9226   9862  10199  -5039  -2621
29       C
ATOM  12394  N   LEU B 792      11.776 -10.056 107.680  1.00 95.50
N
ANISOU12394  N   LEU B 792    10928  12498  12860  -5189  -2366
532       N
ATOM  12395  CA  LEU B 792      10.968  -9.568 108.797  1.00104.45
C
ANISOU12395  CA  LEU B 792    11932  13733  14021  -5274  -2291
436       C
ATOM  12396  C   LEU B 792      10.788 -10.610 109.900  1.00104.86
C
ANISOU12396  C   LEU B 792    11938  13973  13930  -5442  -2290
634       C
ATOM  12397  O   LEU B 792      10.983 -10.310 111.082  1.00 94.04
O
```

ANISOU12397 O   LEU B 792   10628 12773 12330 -5663 -2346
663   O
ATOM 12398 CB LEU B 792   9.606 -9.092 108.286 1.00109.42
C
ANISOU12398 CB LEU B 792   12388 14342 14843 -5272 -2221
405   C
ATOM 12399 CG LEU B 792   9.449 -7.581 108.076 1.00111.80
C
ANISOU12399 CG LEU B 792   12709 14566 15205 -5278 -2255
207   C
ATOM 12400 CD1 LEU B 792   9.337 -6.858 109.410 1.00118.61
C
ANISOU12400 CD1 LEU B 792   13600 15600 15867 -5543 -2319
171   C
ATOM 12401 CD2 LEU B 792   10.600 -7.011 107.259 1.00 79.02
C
ANISOU12401 CD2 LEU B 792   8726 10252 11046 -5117 -2312
80   C
ATOM 12402 N   ILE B 793   10.423 -11.839 109.536 1.00 80.65
N
ANISOU12402 N   ILE B 793   8785 10912 10946 -5391 -2253
803   N
ATOM 12403 CA ILE B 793   10.241 -12.897 110.533 1.00 82.29
C
ANISOU12403 CA ILE B 793   8941 11300 11026 -5553 -2265
1027   C
ATOM 12404 C   ILE B 793   11.546 -13.249 111.244 1.00 82.07
C
ANISOU12404 C   ILE B 793   9074 11297 10813 -5585 -2349
1045   C
ATOM 12405 O   ILE B 793   11.591 -13.330 112.478 1.00 84.48
O
ANISOU12405 O   ILE B 793   9405 11814 10881 -5831 -2400
1170   O
ATOM 12406 CB ILE B 793   9.606 -14.139 109.884 1.00 81.87
C
ANISOU12406 CB ILE B 793   8773 11219 11117 -5477 -2233
1200   C
ATOM 12407 CG1 ILE B 793   8.197 -13.815 109.383 1.00 82.34
C
ANISOU12407 CG1 ILE B 793   8647 11272 11367 -5456 -2130
1186   C
ATOM 12408 CG2 ILE B 793   9.585 -15.302 110.865 1.00 83.22
C
ANISOU12408 CG2 ILE B 793   8893 11541 11186 -5598 -2249
1419   C
ATOM 12409 CD1 ILE B 793   7.250 -13.355 110.474 1.00 85.77
C
ANISOU12409 CD1 ILE B 793   8950 11958 11681 -5736 -2106
1315   C
ATOM 12410 N   ALA B 794   12.625 -13.468 110.489 1.00107.84
N
ANISOU12410 N   ALA B 794   12481 14417 14076 -5464 -2426
1025   N

ATOM 12411 CA ALA B 794   13.894 -13.832 111.120 1.00 80.59
C
ANISOU12411 CA ALA B 794   9177 10980 10465 -5504 -2516
1065   C
ATOM 12412 C   ALA B 794   14.444 -12.734 112.026 1.00 80.92
C
ANISOU12412 C   ALA B 794   9314 11057 10377 -5553 -2522
878   C
ATOM 12413 O   ALA B 794   14.915 -13.018 113.133 1.00 82.25
O
ANISOU12413 O   ALA B 794   9547 11351 10355 -5701 -2581
959   O
ATOM 12414 CB ALA B 794   14.922 -14.195 110.050 1.00128.50
C
ANISOU12414 CB ALA B 794   15369 16900 16556 -5396 -2603
1098   C
ATOM 12415 N   GLN B 795   14.402 -11.479 111.583 1.00 80.38
N
ANISOU12415 N   GLN B 795   9278 10901 10363 -5483 -2501
669   N
ATOM 12416 CA GLN B 795   14.942 -10.388 112.391 1.00 81.48
C
ANISOU12416 CA GLN B 795   9544 11082 10332 -5580 -2560
519   C
ATOM 12417 C   GLN B 795   13.996 -9.874 113.470 1.00 87.30
C
ANISOU12417 C   GLN B 795   10251 12070 10851 -5898 -2579
560   C
ATOM 12418 O   GLN B 795   14.465 -9.266 114.437 1.00 86.70
O
ANISOU12418 O   GLN B 795   10308 12107 10526 -6067 -2649
481   O
ATOM 12419 CB GLN B 795   15.386 -9.239 111.486 1.00 79.89
C
ANISOU12419 CB GLN B 795   9404 10689 10262 -5393 -2555
290   C
ATOM 12420 CG GLN B 795   16.503 -9.639 110.540 1.00118.19
C
ANISOU12420 CG GLN B 795   14383 15419 15104 -5307 -2621
382   C
ATOM 12421 CD GLN B 795   17.563 -10.476 111.239 1.00 87.70
C
ANISOU12421 CD GLN B 795   10622 11602 11097 -5366 -2691
503   C
ATOM 12422 OE1 GLN B 795   18.180 -10.028 112.206 1.00 79.72
O
ANISOU12422 OE1 GLN B 795   9704 10632   9956 -5423 -2732
416   O
ATOM 12423 NE2 GLN B 795   17.764 -11.701 110.766 1.00 78.68
N
ANISOU12423 NE2 GLN B 795   9461 10449   9985 -5354 -2716
697   N
ATOM 12424 N   LEU B 796   12.688 -10.092 113.338 1.00116.05
N

```
ANISOU12424  N   LEU B 796    13713  15816  14565  -5989  -2507
664       N
ATOM  12425  CA  LEU B 796      11.804  -9.964 114.494  1.00105.09
C
ANISOU12425  CA  LEU B 796    12246  14752  12932  -6333  -2489
758       C
ATOM  12426  C   LEU B 796      12.151 -10.966 115.590  1.00 94.30
C
ANISOU12426  C   LEU B 796    10911  13614  11306  -6537  -2528
983       C
ATOM  12427  O   LEU B 796      12.238 -10.606 116.769  1.00140.22
O
ANISOU12427  O   LEU B 796    16793  19695  16788  -6821  -2558
969       O
ATOM  12428  CB  LEU B 796      10.348 -10.140 114.060  1.00119.26
C
ANISOU12428  CB  LEU B 796    13797  16613  14902  -6362  -2380
859       C
ATOM  12429  CG  LEU B 796       9.285 -10.117 115.159  1.00118.83
C
ANISOU12429  CG  LEU B 796    13578  16946  14625  -6713  -2301
982       C
ATOM  12430  CD1 LEU B 796       9.308  -8.787 115.894  1.00129.12
C
ANISOU12430  CD1 LEU B 796    14956  18413  15689  -6927  -2313
728       C
ATOM  12431  CD2 LEU B 796       7.907 -10.395 114.579  1.00100.26
C
ANISOU12431  CD2 LEU B 796    10961  14614  12521  -6678  -2179
1107       C
ATOM  12432  N   ILE B 797      12.357 -12.230 115.218  1.00 89.77
N
ANISOU12432  N   ILE B 797    10292  12950  10867  -6402  -2533
1176       N
ATOM  12433  CA  ILE B 797      12.781 -13.249 116.181  1.00101.97
C
ANISOU12433  CA  ILE B 797    11870  14669  12205  -6567  -2596
1407       C
ATOM  12434  C   ILE B 797      14.125 -12.903 116.818  1.00 91.21
C
ANISOU12434  C   ILE B 797    10735  13283  10636  -6607  -2707
1309       C
ATOM  12435  O   ILE B 797      14.292 -12.998 118.040  1.00 93.89
O
ANISOU12435  O   ILE B 797    11137  13887  10651  -6891  -2762
1413       O
ATOM  12436  CB  ILE B 797      12.817 -14.633 115.507  1.00106.37
C
ANISOU12436  CB  ILE B 797    12343  15074  13000  -6368  -2594
1581       C
ATOM  12437  CG1 ILE B 797      11.398 -15.093 115.164  1.00101.06
C
ANISOU12437  CG1 ILE B 797    11441  14481  12476  -6385  -2494
1725       C
```

```
ATOM  12438  CG2 ILE B 797      13.517 -15.651 116.397  1.00 90.44
C
ANISOU12438  CG2 ILE B 797    10387  13164  10811  -6491  -2694
1795       C
ATOM  12439  CD1 ILE B 797      11.348 -16.248 114.190  1.00136.20
C
ANISOU12439  CD1 ILE B 797    15820  18720  17210  -6132  -2480
1798       C
ATOM  12440  N   ALA B 798      15.102 -12.490 116.010  1.00 88.37
N
ANISOU12440  N   ALA B 798    10494  12630  10454  -6329  -2732
1110       N
ATOM  12441  CA  ALA B 798      16.369 -12.021 116.570  1.00 88.42
C
ANISOU12441  CA  ALA B 798    10704  12593  10298  -6341  -2826
997       C
ATOM  12442  C   ALA B 798      16.201 -10.813 117.490  1.00 97.95
C
ANISOU12442  C   ALA B 798    12017  13989  11210  -6582  -2857
837       C
ATOM  12443  O   ALA B 798      16.818 -10.755 118.560  1.00125.36
O
ANISOU12443  O   ALA B 798    15631  17610  14390  -6772  -2942
853       O
ATOM  12444  CB  ALA B 798      17.344 -11.694 115.439  1.00117.37
C
ANISOU12444  CB  ALA B 798    14428  15935  14231  -5989  -2812
804       C
ATOM  12445  N   THR B 799      15.387  -9.835 117.090  1.00 99.92
N
ANISOU12445  N   THR B 799    12204  14233  11526  -6576  -2794
661       N
ATOM  12446  CA  THR B 799      15.115  -8.682 117.950  1.00104.75
C
ANISOU12446  CA  THR B 799    12903  15042  11853  -6807  -2815
457       C
ATOM  12447  C   THR B 799      14.445  -9.073 119.268  1.00100.28
C
ANISOU12447  C   THR B 799    12284  14919  10897  -7212  -2795
598       C
ATOM  12448  O   THR B 799      14.840  -8.601 120.340  1.00119.20
O
ANISOU12448  O   THR B 799    14837  17526  12929  -7423  -2856
477       O
ATOM  12449  CB  THR B 799      14.263  -7.660 117.197  1.00128.55
C
ANISOU12449  CB  THR B 799    15824  17960  15058  -6719  -2751
252       C
ATOM  12450  OG1 THR B 799      14.957  -7.239 116.015  1.00149.20
O
ANISOU12450  OG1 THR B 799    18489  20206  17995  -6363  -2770
123       O
ATOM  12451  CG2 THR B 799      13.996  -6.443 118.073  1.00114.67
C
```

```
ANISOU12451  CG2 THR B 799    14163  16399  13006  -6940  -2774
-23       C
ATOM  12452  N   LEU B 800      13.430  -9.938 119.207  1.00 98.81
N
ANISOU12452  N   LEU B 800    11869  14902  10772  -7317  -2701
848       N
ATOM  12453  CA  LEU B 800      12.786 -10.441 120.422  1.00102.53
C
ANISOU12453  CA  LEU B 800    12233  15842  10884  -7704  -2653
1036       C
ATOM  12454  C   LEU B 800      13.743 -11.201 121.338  1.00106.11
C
ANISOU12454  C   LEU B 800    12834  16408  11077  -7837  -2773
1216       C
ATOM  12455  O   LEU B 800      13.691 -11.043 122.563  1.00163.16
O
ANISOU12455  O   LEU B 800    20104  24025  17866  -8174  -2787
1210       O
ATOM  12456  CB  LEU B 800      11.593 -11.325 120.054  1.00105.74
C
ANISOU12456  CB  LEU B 800    12342  16351  11484  -7719  -2523
1316       C
ATOM  12457  CG  LEU B 800      10.391 -10.616 119.423  1.00111.92
C
ANISOU12457  CG  LEU B 800    12921  17137  12465  -7684  -2379
1187       C
ATOM  12458  CD1 LEU B 800       9.387 -11.627 118.888  1.00118.01
C
ANISOU12458  CD1 LEU B 800    13429  17905  13505  -7596  -2270
1485       C
ATOM  12459  CD2 LEU B 800       9.734  -9.668 120.416  1.00106.94
C
ANISOU12459  CD2 LEU B 800    12212  16944  11478  -8038  -2272
991       C
ATOM  12460  N   ILE B 801      14.602 -12.050 120.778  1.00100.67
N
ANISOU12460  N   ILE B 801    12209  15409  10634  -7585  -2857
1364       N
ATOM  12461  CA  ILE B 801      15.658 -12.672 121.577  1.00101.37
C
ANISOU12461  CA  ILE B 801    12453  15539  10524  -7676  -2989
1508       C
ATOM  12462  C   ILE B 801      16.597 -11.633 122.190  1.00102.23
C
ANISOU12462  C   ILE B 801    12818  15640  10384  -7725  -3079
1238       C
ATOM  12463  O   ILE B 801      16.947 -11.718 123.373  1.00142.73
O
ANISOU12463  O   ILE B 801    18061  21039  15131  -7994  -3157
1296       O
ATOM  12464  CB  ILE B 801      16.428 -13.707 120.730  1.00110.23
C
ANISOU12464  CB  ILE B 801    13574  16305  12003  -7358  -3045
1650       C
```

```
ATOM  12465  CG1 ILE B 801      15.778 -15.094 120.828  1.00 98.82
C
ANISOU12465  CG1 ILE B 801    11942  14985  10622  -7429  -3034
2001       C
ATOM  12466  CG2 ILE B 801      17.892 -13.780 121.153  1.00 97.59
C
ANISOU12466  CG2 ILE B 801    12185  14571  10323  -7309  -3180
1624       C
ATOM  12467  CD1 ILE B 801      14.325 -15.161 120.409  1.00107.89
C
ANISOU12467  CD1 ILE B 801    12851  16253  11888  -7454  -2892
2066       C
ATOM  12468  N   ALA B 802      17.007 -10.635 121.411  1.00100.02
N
ANISOU12468  N   ALA B 802    12632  15059  10312  -7458  -3072
941       N
ATOM  12469  CA  ALA B 802      17.885  -9.588 121.933  1.00100.82
C
ANISOU12469  CA  ALA B 802    12972  15113  10220  -7450  -3157
666       C
ATOM  12470  C   ALA B 802      17.281  -8.795 123.094  1.00138.36
C
ANISOU12470  C   ALA B 802    17802  20273  14496  -7778  -3148
478       C
ATOM  12471  O   ALA B 802      17.969  -8.528 124.086  1.00142.33
O
ANISOU12471  O   ALA B 802    18509  20908  14663  -7898  -3242
391       O
ATOM  12472  CB  ALA B 802      18.275  -8.638 120.801  1.00 97.84
C
ANISOU12472  CB  ALA B 802    12632  14357  10184  -7096  -3138
402       C
ATOM  12473  N   VAL B 803      16.012  -8.398 122.995  1.00142.95
N
ANISOU12473  N   VAL B 803    18220  21070  15024  -7909  -3030
383       N
ATOM  12474  CA  VAL B 803      15.351  -7.732 124.120  1.00115.93
C
ANISOU12474  CA  VAL B 803    14837  18121  11089  -8226  -2993
159       C
ATOM  12475  C   VAL B 803      15.149  -8.645 125.333  1.00114.00
C
ANISOU12475  C   VAL B 803    14547  18368  10401  -8591  -3005
416       C
ATOM  12476  O   VAL B 803      15.378  -8.218 126.471  1.00123.26
O
ANISOU12476  O   VAL B 803    15926  19809  11096  -8616  -2972
261       O
ATOM  12477  CB  VAL B 803      14.031  -7.073 123.671  1.00125.29
C
ANISOU12477  CB  VAL B 803    15814  19433  12357  -8274  -2844
-31       C
ATOM  12478  CG1 VAL B 803      14.317  -5.956 122.675  1.00140.40
C
```

ANISOU12478 CG1 VAL B 803   17829 20900 14618 -7938 -2871
-331   C
ATOM 12479 CG2 VAL B 803   13.082 -8.083 123.069 1.00164.68
C
ANISOU12479 CG2 VAL B 803   20464 24465 17641 -8322 -2719
330   C
ATOM 12480 N   TYR B 804   14.722 -9.898 125.140 1.00113.48
N
ANISOU12480 N   TYR B 804   14241 18365 10510 -8685 -2959
839   N
ATOM 12481 CA TYR B 804   14.154 -10.645 126.262 1.00120.24
C
ANISOU12481 CA TYR B 804   14999 19701 10986 -8887 -2831
1112   C
ATOM 12482 C   TYR B 804   14.869 -11.926 126.683 1.00137.03
C
ANISOU12482 C   TYR B 804   17115 21882 13069 -9152 -3052
1513   C
ATOM 12483 O   TYR B 804   14.493 -12.497 127.714 1.00123.27
O
ANISOU12483 O   TYR B 804   15336 20522 10979 -9256 -2928
1755   O
ATOM 12484 CB TYR B 804   12.691 -11.011 125.957 1.00125.63
C
ANISOU12484 CB TYR B 804   15354 20544 11836 -8807 -2528
1300   C
ATOM 12485 CG TYR B 804   11.774 -9.823 125.757 1.00136.97
C
ANISOU12485 CG TYR B 804   16766 22001 13277 -8524 -2250
947   C
ATOM 12486 CD1 TYR B 804   12.018 -8.612 126.391 1.00148.55
C
ANISOU12486 CD1 TYR B 804   18494 23536 14414 -8353 -2174
528   C
ATOM 12487 CD2 TYR B 804   10.658 -9.919 124.937 1.00135.67
C
ANISOU12487 CD2 TYR B 804   16308 21775 13466 -8426 -2075
1031   C
ATOM 12488 CE1 TYR B 804   11.193 -7.520 126.188 1.00148.58
C
ANISOU12488 CE1 TYR B 804   18461 23527 14464 -8089 -1937
195   C
ATOM 12489 CE2 TYR B 804   9.818 -8.840 124.745 1.00131.35
C
ANISOU12489 CE2 TYR B 804   15721 21227 12958 -8168 -1836
716   C
ATOM 12490 CZ TYR B 804   10.092 -7.643 125.369 1.00134.05
C
ANISOU12490 CZ TYR B 804   16320 21623 12991 -7997 -1769
296   C
ATOM 12491 OH TYR B 804   9.246 -6.572 125.194 1.00137.39
O
ANISOU12491 OH TYR B 804   16690 22031 13481 -7733 -1539
-24   O

```
ATOM  12492  N   ALA B 805      15.867 -12.405 125.947  1.00149.70
N
ANISOU12492  N   ALA B 805    18833  22976  15070  -8839  -3156
1623       N
ATOM  12493  CA  ALA B 805      16.497 -13.676 126.296  1.00143.53
C
ANISOU12493  CA  ALA B 805    18062  22156  14318  -8884  -3276
2009       C
ATOM  12494  C   ALA B 805      17.561 -13.504 127.378  1.00150.75
C
ANISOU12494  C   ALA B 805    19243  23180  14857  -9034  -3438
1968       C
ATOM  12495  O   ALA B 805      18.488 -12.700 127.230  1.00120.66
O
ANISOU12495  O   ALA B 805    15661  19090  11092  -8830  -3501
1683       O
ATOM  12496  CB  ALA B 805      17.108 -14.326 125.056  1.00131.62
C
ANISOU12496  CB  ALA B 805    16541  20103  13364  -8468  -3311
2097       C
ATOM  12497  N   ASN B 806      17.414 -14.258 128.471  1.00176.23
N
ANISOU12497  N   ASN B 806    22427  26815  17718  -9378  -3503
2269       N
ATOM  12498  CA  ASN B 806      18.285 -14.178 129.641  1.00173.28
C
ANISOU12498  CA  ASN B 806    22299  26622  16919  -9553  -3659
2271       C
ATOM  12499  C   ASN B 806      18.873 -15.559 129.910  1.00179.39
C
ANISOU12499  C   ASN B 806    23032  27305  17822  -9628  -3800
2736       C
ATOM  12500  O   ASN B 806      18.820 -16.060 131.039  1.00186.47
O
ANISOU12500  O   ASN B 806    23935  28608  18306  -9960  -3887
2984       O
ATOM  12501  CB  ASN B 806      17.531 -13.671 130.873  1.00176.09
C
ANISOU12501  CB  ASN B 806    22694  27644  16568  -9850  -3591
2156       C
ATOM  12502  CG  ASN B 806      17.094 -12.226 130.743  1.00172.88
C
ANISOU12502  CG  ASN B 806    22419  27233  16035  -9560  -3356
1651       C
ATOM  12503  OD1 ASN B 806      17.783 -11.408 130.131  1.00135.46
O
ANISOU12503  OD1 ASN B 806    17833  22154  11480  -9455  -3511
1314       O
ATOM  12504  ND2 ASN B 806      15.950 -11.900 131.332  1.00190.25
N
ANISOU12504  ND2 ASN B 806    24551  29807  17928  -9424  -2978
1597       N
ATOM  12505  N   TRP B 807      19.432 -16.179 128.874  1.00176.23
N
```

ANISOU12505 N   TRP B 807   22597 26395 17968 -9293 -3832
2836   N
ATOM 12506 CA TRP B 807   19.981 -17.534 128.937 1.00181.17
C
ANISOU12506 CA TRP B 807   23180 26870 18786 -9269 -3971
3228   C
ATOM 12507 C   TRP B 807   21.419 -17.507 129.445 1.00174.47
C
ANISOU12507 C   TRP B 807   22577 25822 17893 -9238 -4144
3207   C
ATOM 12508 O   TRP B 807   22.370 -17.342 128.680 1.00183.48
O
ANISOU12508 O   TRP B 807   23809 26514 19391 -8892 -4171
3032   O
ATOM 12509 CB TRP B 807   19.890 -18.200 127.571 1.00176.85
C
ANISOU12509 CB TRP B 807   22485 25906 18804 -8872 -3920
3263   C
ATOM 12510 CG TRP B 807   18.498 -18.238 127.030 1.00165.34
C
ANISOU12510 CG TRP B 807   20786 24594 17440 -8864 -3749
3284   C
ATOM 12511 CD1 TRP B 807   17.346 -18.415 127.741 1.00167.70
C
ANISOU12511 CD1 TRP B 807   20905 25376 17437 -9202 -3669
3488   C
ATOM 12512 CD2 TRP B 807   18.105 -18.074 125.664 1.00164.73
C
ANISOU12512 CD2 TRP B 807   20603 24193 17793 -8498 -3623
3094   C
ATOM 12513 NE1 TRP B 807   16.262 -18.384 126.899 1.00165.07
N
ANISOU12513 NE1 TRP B 807   20357 25007 17356 -9050 -3496
3442   N
ATOM 12514 CE2 TRP B 807   16.701 -18.175 125.618 1.00176.09
C
ANISOU12514 CE2 TRP B 807   21809 25904 19194 -8624 -3479
3199   C
ATOM 12515 CE3 TRP B 807   18.806 -17.858 124.473 1.00153.27
C
ANISOU12515 CE3 TRP B 807   19217 22272 16748 -8081 -3606
2849   C
ATOM 12516 CZ2 TRP B 807   15.983 -18.066 124.429 1.00177.58
C
ANISOU12516 CZ2 TRP B 807   21851 25874 19747 -8341 -3340
3069   C
ATOM 12517 CZ3 TRP B 807   18.092 -17.750 123.294 1.00152.46
C
ANISOU12517 CZ3 TRP B 807   18969 21986 16971 -7820 -3468
2718   C
ATOM 12518 CH2 TRP B 807   16.696 -17.854 123.280 1.00168.18
C
ANISOU12518 CH2 TRP B 807   20752 24223 18927 -7949 -3347
2828   C

```
ATOM  12519  N   GLU B 808      21.572 -17.656 130.764  1.00179.85
N
ANISOU12519  N   GLU B 808    23348  26865  18121  -9604  -4256
3385       N
ATOM  12520  CA  GLU B 808      22.902 -17.661 131.369  1.00189.59
C
ANISOU12520  CA  GLU B 808    24807  27929  19300  -9602  -4427
3392       C
ATOM  12521  C   GLU B 808      23.770 -18.782 130.807  1.00179.32
C
ANISOU12521  C   GLU B 808    23467  26213  18454  -9373  -4551
3635       C
ATOM  12522  O   GLU B 808      24.977 -18.602 130.609  1.00168.44
O
ANISOU12522  O   GLU B 808    22232  24487  17279  -9156  -4633
3500       O
ATOM  12523  CB  GLU B 808      22.789 -17.792 132.889  1.00202.45
C
ANISOU12523  CB  GLU B 808    26513  30055  20353 -10036  -4524
3587       C
ATOM  12524  CG  GLU B 808      22.263 -16.553 133.587  1.00197.07
C
ANISOU12524  CG  GLU B 808    25961  29781  19137 -10159  -4421
3228       C
ATOM  12525  CD  GLU B 808      23.278 -15.428 133.594  1.00170.59
C
ANISOU12525  CD  GLU B 808    22883  26133  15801  -9909  -4448
2805       C
ATOM  12526  OE1 GLU B 808      22.928 -14.306 133.171  1.00171.46
O
ANISOU12526  OE1 GLU B 808    23052  26217  15880  -9729  -4317
2396       O
ATOM  12527  OE2 GLU B 808      24.428 -15.667 134.021  1.00129.50
O
ANISOU12527  OE2 GLU B 808    17827  20717  10660  -9889  -4608
2892       O
ATOM  12528  N   PHE B 809      23.175 -19.951 130.556  1.00184.09
N
ANISOU12528  N   PHE B 809    23868  26860  19219  -9394  -4570
3974       N
ATOM  12529  CA  PHE B 809      23.909 -21.043 129.920  1.00194.30
C
ANISOU12529  CA  PHE B 809    25107  27761  20956  -9113  -4682
4138       C
ATOM  12530  C   PHE B 809      24.449 -20.657 128.546  1.00162.83
C
ANISOU12530  C   PHE B 809    21121  23314  17434  -8631  -4578
3783       C
ATOM  12531  O   PHE B 809      25.496 -21.167 128.130  1.00110.50
O
ANISOU12531  O   PHE B 809    14520  16350  11113  -8386  -4665
3774       O
ATOM  12532  CB  PHE B 809      23.028 -22.294 129.819  1.00202.64
C
```

```
ANISOU12532  CB  PHE B 809    25933  28945  22115  -9166  -4711
4504       C
ATOM  12533  CG  PHE B 809      21.720 -22.076 129.104  1.00214.49
C
ANISOU12533  CG  PHE B 809    27243  30557  23698  -9083  -4513
4402       C
ATOM  12534  CD1 PHE B 809      20.570 -21.765 129.811  1.00212.51
C
ANISOU12534  CD1 PHE B 809    26892  30803  23049  -9437  -4424
4515       C
ATOM  12535  CD2 PHE B 809      21.637 -22.213 127.726  1.00171.70
C
ANISOU12535  CD2 PHE B 809    21724  24764  18751  -8659  -4406
4196       C
ATOM  12536  CE1 PHE B 809      19.366 -21.576 129.156  1.00201.64
C
ANISOU12536  CE1 PHE B 809    25317  29516  21781  -9353  -4234
4429       C
ATOM  12537  CE2 PHE B 809      20.437 -22.025 127.066  1.00165.93
C
ANISOU12537  CE2 PHE B 809    20818  24111  18117  -8580  -4230
4113       C
ATOM  12538  CZ  PHE B 809      19.301 -21.707 127.781  1.00163.94
C
ANISOU12538  CZ  PHE B 809    20465  24323  17503  -8919  -4146
4235       C
ATOM  12539  N   ALA B 810      23.766 -19.767 127.830  1.00131.22
N
ANISOU12539  N   ALA B 810    17072  19304  13480  -8498  -4392
3489       N
ATOM  12540  CA  ALA B 810      24.225 -19.321 126.522  1.00138.82
C
ANISOU12540  CA  ALA B 810    18024  19873  14848  -8065  -4283
3158       C
ATOM  12541  C   ALA B 810      25.001 -18.007 126.566  1.00113.29
C
ANISOU12541  C   ALA B 810    14980  16526  11538  -7982  -4258
2815       C
ATOM  12542  O   ALA B 810      25.318 -17.460 125.505  1.00139.44
O
ANISOU12542  O   ALA B 810    18275  19560  15145  -7647  -4154
2531       O
ATOM  12543  CB  ALA B 810      23.037 -19.190 125.567  1.00178.53
C
ANISOU12543  CB  ALA B 810    22876  24911  20046  -7924  -4104
3055       C
ATOM  12544  N   LYS B 811      25.275 -17.475 127.760  1.00125.50
N
ANISOU12544  N   LYS B 811    16698  18297  12689  -8275  -4349
2825       N
ATOM  12545  CA  LYS B 811      26.084 -16.274 127.974  1.00140.25
C
ANISOU12545  CA  LYS B 811    18762  20060  14466  -8198  -4360
2507       C
```

ATOM 12546 C   LYS B 811   25.345 -14.990 127.612 1.00134.52
C
ANISOU12546 C   LYS B 811   18052 19407 13650 -8142 -4208
2164   C
ATOM 12547 O   LYS B 811   25.980 -13.961 127.357 1.00131.88
O
ANISOU12547 O   LYS B 811   17838 18886 13387 -7947 -4191
1853   O
ATOM 12548 CB LYS B 811   27.416 -16.331 127.210 1.00150.48
C
ANISOU12548 CB LYS B 811   20084 20934 16157 -7834 -4393
2381   C
ATOM 12549 CG LYS B 811   28.323 -17.488 127.596 1.00153.70
C
ANISOU12549 CG LYS B 811   20494 21240 16665 -7865 -4558
2665   C
ATOM 12550 CD LYS B 811   29.644 -17.413 126.845 1.00143.34
C
ANISOU12550 CD LYS B 811   19191 19557 15714 -7511 -4565
2487   C
ATOM 12551 CE LYS B 811   30.701 -18.310 127.472 1.00145.50
C
ANISOU12551 CE LYS B 811   19513 19748 16025 -7581 -4756
2723   C
ATOM 12552 NZ LYS B 811   30.412 -19.752 127.236 1.00164.73
N
ANISOU12552 NZ LYS B 811   21788 22165 18638 -7576 -4806
3016   N
ATOM 12553 N   ILE B 812   24.016 -15.019 127.585 1.00109.35
N
ANISOU12553 N   ILE B 812   14736 16491 10322 -8304 -4104
2216   N
ATOM 12554 CA ILE B 812   23.212 -13.985 126.947 1.00108.29
C
ANISOU12554 CA ILE B 812   14559 16367 10220 -8195 -3949
1911   C
ATOM 12555 C   ILE B 812   22.288 -13.352 127.978 1.00127.51
C
ANISOU12555 C   ILE B 812   17040 19273 12135 -8544 -3915
1826   C
ATOM 12556 O   ILE B 812   21.705 -14.052 128.813 1.00161.49
O
ANISOU12556 O   ILE B 812   21272 23956 16130 -8875 -3944
2099   O
ATOM 12557 CB ILE B 812   22.410 -14.560 125.761 1.00154.03
C
ANISOU12557 CB ILE B 812   20118 22032 16375 -8006 -3827
1996   C
ATOM 12558 CG1 ILE B 812   23.285 -14.584 124.510 1.00181.11
C
ANISOU12558 CG1 ILE B 812   23529 25004 20282 -7570 -3801
1855   C
ATOM 12559 CG2 ILE B 812   21.133 -13.766 125.509 1.00182.91
C

ANISOU12559 CG2 ILE B 812   23682 25882 19933 -8077 -3680
1816   C
ATOM 12560 CD1 ILE B 812   23.448 -13.229 123.849 1.00144.05
C
ANISOU12560 CD1 ILE B 812   18901 20131 15701 -7345 -3718
1476   C
ATOM 12561 N   ARG B 813   22.154 -12.027 127.917 1.00112.52
N
ANISOU12561 N   ARG B 813   15252 17373 10125 -8455 -3852
1435   N
ATOM 12562 CA ARG B 813   21.165 -11.324 128.720 1.00132.11
C
ANISOU12562 CA ARG B 813   17767 20314 12115 -8710 -3783
1256   C
ATOM 12563 C   ARG B 813   20.508 -10.239 127.878 1.00151.10
C
ANISOU12563 C   ARG B 813   20129 22606 14677 -8517 -3651
903   C
ATOM 12564 O   ARG B 813   21.185 -9.507 127.149 1.00111.98
O
ANISOU12564 O   ARG B 813   15260 17256 10034 -8203 -3661
673   O
ATOM 12565 CB ARG B 813   21.787 -10.732 129.991 1.00168.75
C
ANISOU12565 CB ARG B 813   22680 25160 16277 -8825 -3879
1090   C
ATOM 12566 CG ARG B 813   20.780 -10.055 130.909 1.00171.73
C
ANISOU12566 CG ARG B 813   23125 26084 16041 -9030 -3782
866   C
ATOM 12567 CD ARG B 813   21.310 -9.938 132.331 1.00128.08
C
ANISOU12567 CD ARG B 813   17846 20852   9968 -9166 -3863
843   C
ATOM 12568 NE ARG B 813   21.494 -11.256 132.931 1.00136.85
N
ANISOU12568 NE ARG B 813   18874 22145 10978 -9430 -3967
1319   N
ATOM 12569 CZ ARG B 813   20.508 -12.020 133.396 1.00155.18
C
ANISOU12569 CZ ARG B 813   21028 24954 12980 -9715 -3907
1603   C
ATOM 12570 NH1 ARG B 813   20.743 -13.276 133.740 1.00163.13
N
ANISOU12570 NH1 ARG B 813   21924 26024 14034 -9929 -4026
2075   N
ATOM 12571 NH2 ARG B 813   19.261 -11.571 133.386 1.00146.71
N
ANISOU12571 NH2 ARG B 813   19864 24281 11599 -9766 -3723
1435   N
ATOM 12572 N   GLY B 814   19.183 -10.151 127.994 1.00155.06
N
ANISOU12572 N   GLY B 814   20475 23477 14963 -8714 -3529
876   N

```
ATOM  12573  CA  GLY B 814      18.420  -9.221 127.180  1.00154.29
C
ANISOU12573  CA  GLY B 814    20297  23287  15040  -8564  -3403
575       C
ATOM  12574  C   GLY B 814      18.801  -7.772 127.420  1.00180.30
C
ANISOU12574  C   GLY B 814    23840  26501  18164  -8405  -3419
107       C
ATOM  12575  O   GLY B 814      19.290  -7.397 128.488  1.00190.94
O
ANISOU12575  O   GLY B 814    25419  28040  19087  -8477  -3483
-31       O
ATOM  12576  N   ILE B 815      18.571  -6.949 126.395  1.00154.12
N
ANISOU12576  N   ILE B 815    20477  22882  15200  -8159  -3358
-130       N
ATOM  12577  CA  ILE B 815      18.830  -5.514 126.455  1.00146.62
C
ANISOU12577  CA  ILE B 815    19728  21803  14177  -7973  -3372
-576       C
ATOM  12578  C   ILE B 815      17.728  -4.772 125.703  1.00160.99
C
ANISOU12578  C   ILE B 815    21405  23609  16156  -7914  -3251
-808       C
ATOM  12579  O   ILE B 815      17.313  -5.187 124.616  1.00149.42
O
ANISOU12579  O   ILE B 815    19714  21936  15122  -7826  -3196
-629       O
ATOM  12580  CB  ILE B 815      20.226  -5.155 125.905  1.00111.54
C
ANISOU12580  CB  ILE B 815    15412  16857  10110  -7647  -3489
-618       C
ATOM  12581  CG1 ILE B 815      20.543  -3.690 126.195  1.00113.00
C
ANISOU12581  CG1 ILE B 815    15812  16947  10174  -7479  -3529
-1056       C
ATOM  12582  CG2 ILE B 815      20.339  -5.472 124.420  1.00106.97
C
ANISOU12582  CG2 ILE B 815    14640  15881  10123  -7394  -3452
-469       C
ATOM  12583  CD1 ILE B 815      22.018  -3.390 126.240  1.00160.54
C
ANISOU12583  CD1 ILE B 815    21990  22647  16361  -7259  -3665
-1087       C
ATOM  12584  N   GLY B 816      17.228  -3.699 126.314  1.00176.55
N
ANISOU12584  N   GLY B 816    23509  25812  17759  -7946  -3199
-1216       N
ATOM  12585  CA  GLY B 816      15.977  -3.047 125.967  1.00190.16
C
ANISOU12585  CA  GLY B 816    25081  27631  19541  -7857  -2992
-1432       C
ATOM  12586  C   GLY B 816      15.666  -2.682 124.526  1.00160.76
C
```

```
ANISOU12586  C   GLY B 816    21187  23538  16359  -7776  -3045
-1477       C
ATOM  12587  O   GLY B 816      16.551  -2.606 123.669  1.00162.52
O
ANISOU12587  O   GLY B 816    21442  23287  17023  -7502  -3139
-1378       O
ATOM  12588  N   TRP B 817      14.372  -2.452 124.275  1.00137.26
N
ANISOU12588  N   TRP B 817    17990  20653  13509  -7609  -2762
-1507       N
ATOM  12589  CA  TRP B 817      13.878  -2.092 122.946  1.00135.98
C
ANISOU12589  CA  TRP B 817    17642  20178  13845  -7525  -2784
-1537       C
ATOM  12590  C   TRP B 817      14.542  -0.835 122.394  1.00164.46
C
ANISOU12590  C   TRP B 817    21439  23414  17634  -7400  -2983
-1894       C
ATOM  12591  O   TRP B 817      14.846  -0.762 121.197  1.00204.58
O
ANISOU12591  O   TRP B 817    26440  28073  23221  -7149  -3032
-1759       O
ATOM  12592  CB  TRP B 817      12.362  -1.898 122.993  1.00127.28
C
ANISOU12592  CB  TRP B 817    16299  19265  12798  -7330  -2428
-1556       C
ATOM  12593  CG  TRP B 817      11.584  -3.168 123.062  1.00134.72
C
ANISOU12593  CG  TRP B 817    16970  20463  13755  -7443  -2253
-1140       C
ATOM  12594  CD1 TRP B 817      11.090  -3.773 124.180  1.00131.79
C
ANISOU12594  CD1 TRP B 817    16552  20520  13003  -7469  -2016
-983       C
ATOM  12595  CD2 TRP B 817      11.175  -3.975 121.953  1.00148.06
C
ANISOU12595  CD2 TRP B 817    18389  21997  15868  -7550  -2303
-824       C
ATOM  12596  NE1 TRP B 817      10.417  -4.922 123.836  1.00131.89
N
ANISOU12596  NE1 TRP B 817    16277  20641  13195  -7589  -1926
-574       N
ATOM  12597  CE2 TRP B 817      10.453  -5.066 122.474  1.00136.79
C
ANISOU12597  CE2 TRP B 817    16758  20902  14312  -7635  -2102
-479       C
ATOM  12598  CE3 TRP B 817      11.357  -3.885 120.570  1.00129.11
C
ANISOU12598  CE3 TRP B 817    15903  19217  13935  -7590  -2505
-798       C
ATOM  12599  CZ2 TRP B 817       9.912  -6.060 121.660  1.00125.78
C
ANISOU12599  CZ2 TRP B 817    15078  19451  13260  -7751  -2107
-121       C
```

```
ATOM  12600  CZ3 TRP B 817      10.820  -4.872 119.764  1.00115.47
C
ANISOU12600  CZ3 TRP B 817    13900  17449  12523  -7706  -2501
-454       C
ATOM  12601  CH2 TRP B 817      10.107  -5.946 120.311  1.00118.09
C
ANISOU12601  CH2 TRP B 817    14033  18097  12737  -7781  -2308
-125       C
ATOM  12602  N   GLY B 818      14.770   0.166 123.245  1.00163.28
N
ANISOU12602  N   GLY B 818    21525  23335  17178  -7275  -2962
-2264       N
ATOM  12603  CA  GLY B 818      15.482   1.355 122.801  1.00159.66
C
ANISOU12603  CA  GLY B 818    21246  22488  16931  -7069  -3133
-2555       C
ATOM  12604  C   GLY B 818      16.903   1.083 122.347  1.00163.17
C
ANISOU12604  C   GLY B 818    21772  22558  17668  -6836  -3279
-2312       C
ATOM  12605  O   GLY B 818      17.348   1.611 121.325  1.00183.39
O
ANISOU12605  O   GLY B 818    24289  24721  20671  -6568  -3351
-2313       O
ATOM  12606  N   TRP B 819      17.639   0.266 123.100  1.00140.30
N
ANISOU12606  N   TRP B 819    18974  19811  14522  -6934  -3313
-2115       N
ATOM  12607  CA  TRP B 819      18.972  -0.135 122.659  1.00140.31
C
ANISOU12607  CA  TRP B 819    19004  19497  14809  -6731  -3417
-1885       C
ATOM  12608  C   TRP B 819      18.925  -0.955 121.371  1.00107.81
C
ANISOU12608  C   TRP B 819    14648  15163  11152  -6616  -3365
-1559       C
ATOM  12609  O   TRP B 819      19.739  -0.743 120.464  1.00100.48
O
ANISOU12609  O   TRP B 819    13685  13898  10594  -6337  -3405
-1523       O
ATOM  12610  CB  TRP B 819      19.664  -0.922 123.774  1.00138.60
C
ANISOU12610  CB  TRP B 819    18925  19505  14232  -6894  -3461
-1729       C
ATOM  12611  CG  TRP B 819      21.137  -1.079 123.581  1.00146.24
C
ANISOU12611  CG  TRP B 819    19964  20180  15422  -6690  -3572
-1605       C
ATOM  12612  CD1 TRP B 819      21.795  -2.193 123.148  1.00156.73
C
ANISOU12612  CD1 TRP B 819    21187  21394  16969  -6655  -3574
-1262       C
ATOM  12613  CD2 TRP B 819      22.139  -0.086 123.815  1.00114.24
C
```

```
ANISOU12613  CD2 TRP B 819    16082  15925  11400  -6487  -3686
-1843       C
ATOM  12614  NE1 TRP B 819      23.147  -1.956 123.102  1.00127.28
N
ANISOU12614  NE1 TRP B 819    17546  17426  13387  -6456  -3668
-1282       N
ATOM  12615  CE2 TRP B 819      23.384  -0.668 123.504  1.00109.61
C
ANISOU12615  CE2 TRP B 819    15468  15133  11047  -6356  -3742
-1621       C
ATOM  12616  CE3 TRP B 819      22.105   1.240 124.254  1.00109.76
C
ANISOU12616  CE3 TRP B 819    15678  15332  10693  -6393  -3744
-2234       C
ATOM  12617  CZ2 TRP B 819      24.581   0.030 123.619  1.00104.40
C
ANISOU12617  CZ2 TRP B 819    14916  14269  10483  -6161  -3847
-1760       C
ATOM  12618  CZ3 TRP B 819      23.294   1.930 124.367  1.00115.94
C
ANISOU12618  CZ3 TRP B 819    16575  15889  11587  -6184  -3867
-2362       C
ATOM  12619  CH2 TRP B 819      24.515   1.324 124.053  1.00116.14
C
ANISOU12619  CH2 TRP B 819    16548  15737  11843  -6081  -3914
-2118       C
ATOM  12620  N   ALA B 820      17.982  -1.898 121.275  1.00126.50
N
ANISOU12620  N   ALA B 820    16836  17744  13484  -6814  -3261
-1333       N
ATOM  12621  CA  ALA B 820      17.767  -2.639 120.032  1.00124.18
C
ANISOU12621  CA  ALA B 820    16317  17252  13611  -6675  -3195
-1064       C
ATOM  12622  C   ALA B 820      17.395  -1.731 118.861  1.00105.38
C
ANISOU12622  C   ALA B 820    13842  14594  11606  -6441  -3178
-1223       C
ATOM  12623  O   ALA B 820      17.865  -1.938 117.736  1.00107.02
O
ANISOU12623  O   ALA B 820    13956  14527  12180  -6183  -3164
-1107       O
ATOM  12624  CB  ALA B 820      16.685  -3.700 120.243  1.00110.97
C
ANISOU12624  CB  ALA B 820    14458  15883  11822  -6938  -3089
-817       C
ATOM  12625  N   GLY B 821      16.549  -0.729 119.103  1.00115.88
N
ANISOU12625  N   GLY B 821    15188  16010  12832  -6531  -3173
-1505       N
ATOM  12626  CA  GLY B 821      16.280   0.284 118.089  1.00114.10
C
ANISOU12626  CA  GLY B 821    14897  15500  12958  -6314  -3193
-1677       C
```

```
ATOM  12627  C   GLY B 821      17.517   0.990 117.561  1.00126.23
C
ANISOU12627  C   GLY B 821    16533  16709  14721  -6008  -3293
-1774       C
ATOM  12628  O   GLY B 821      17.626   1.255 116.362  1.00163.23
O
ANISOU12628  O   GLY B 821    21097  21143  19779  -5780  -3279
-1738       O
ATOM  12629  N   VAL B 822      18.459   1.307 118.448  1.00109.39
N
ANISOU12629  N   VAL B 822    14605  14603  12354  -6005  -3381
-1900       N
ATOM  12630  CA  VAL B 822      19.758   1.830 118.025  1.00107.36
C
ANISOU12630  CA  VAL B 822    14407  14081  12303  -5737  -3458
-1953       C
ATOM  12631  C   VAL B 822      20.508   0.838 117.139  1.00 93.02
C
ANISOU12631  C   VAL B 822    12461  12147  10736  -5585  -3388
-1660       C
ATOM  12632  O   VAL B 822      21.109   1.222 116.128  1.00 90.49
O
ANISOU12632  O   VAL B 822    12058  11610  10715  -5343  -3369
-1676       O
ATOM  12633  CB  VAL B 822      20.592   2.226 119.258  1.00 99.28
C
ANISOU12633  CB  VAL B 822    13620  13141  10962  -5784  -3563
-2121       C
ATOM  12634  CG1 VAL B 822      21.998   2.641 118.842  1.00122.56
C
ANISOU12634  CG1 VAL B 822    16588  15847  14132  -5531  -3625
-2139       C
ATOM  12635  CG2 VAL B 822      19.901   3.346 120.023  1.00103.10
C
ANISOU12635  CG2 VAL B 822    14249  13721  11204  -5869  -3618
-2489       C
ATOM  12636  N   ILE B 823      20.489  -0.446 117.499  1.00 92.74
N
ANISOU12636  N   ILE B 823    12401  12272  10563  -5728  -3337
-1406       N
ATOM  12637  CA  ILE B 823      21.105  -1.470 116.654  1.00120.98
C
ANISOU12637  CA  ILE B 823    15856  15745  14367  -5577  -3262
-1158       C
ATOM  12638  C   ILE B 823      20.453  -1.537 115.274  1.00142.68
C
ANISOU12638  C   ILE B 823    18408  18365  17438  -5421  -3159
-1104       C
ATOM  12639  O   ILE B 823      21.146  -1.632 114.254  1.00152.11
O
ANISOU12639  O   ILE B 823    19523  19395  18876  -5187  -3102
-1071       O
ATOM  12640  CB  ILE B 823      21.049  -2.838 117.356  1.00131.59
C
```

```
ANISOU12640  CB  ILE B 823    17205  17288  15506  -5776  -3243
-901       C
ATOM  12641  CG1 ILE B 823      21.635  -2.745 118.763  1.00135.63
C
ANISOU12641  CG1 ILE B 823    17918  17953  15661  -5955  -3347
-955       C
ATOM  12642  CG2 ILE B 823      21.849  -3.850 116.564  1.00 89.91
C
ANISOU12642  CG2 ILE B 823    11830  11881  10453  -5593  -3183
-700       C
ATOM  12643  CD1 ILE B 823      21.334  -3.957 119.613  1.00126.25
C
ANISOU12643  CD1 ILE B 823    16736  17025  14210  -6222  -3339
-711       C
ATOM  12644  N   TRP B 824      19.121  -1.491 115.214  1.00129.27
N
ANISOU12644  N   TRP B 824    16624  16762  15729  -5556  -3124
-1102       N
ATOM  12645  CA  TRP B 824      18.436  -1.366 113.926  1.00 86.23
C
ANISOU12645  CA  TRP B 824    10998  11180  10586  -5407  -3043
-1082       C
ATOM  12646  C   TRP B 824      18.847  -0.103 113.174  1.00 85.33
C
ANISOU12646  C   TRP B 824    10886  10854  10680  -5197  -3074
-1294       C
ATOM  12647  O   TRP B 824      19.115  -0.148 111.967  1.00134.33
O
ANISOU12647  O   TRP B 824    16983  16922  17132  -4990  -2995
-1254       O
ATOM  12648  CB  TRP B 824      16.920  -1.384 114.137  1.00 90.91
C
ANISOU12648  CB  TRP B 824    11493  11920  11127  -5614  -3014
-1067       C
ATOM  12649  CG  TRP B 824      16.324  -2.754 114.267  1.00100.42
C
ANISOU12649  CG  TRP B 824    12587  13298  12272  -5751  -2931
-796       C
ATOM  12650  CD1 TRP B 824      15.946  -3.380 115.420  1.00138.28
C
ANISOU12650  CD1 TRP B 824    17410  18373  16758  -6039  -2931
-692       C
ATOM  12651  CD2 TRP B 824      16.011  -3.658 113.200  1.00 89.97
C
ANISOU12651  CD2 TRP B 824    11098  11895  11193  -5607  -2831
-603       C
ATOM  12652  NE1 TRP B 824      15.433  -4.624 115.137  1.00120.47
N
ANISOU12652  NE1 TRP B 824    15002  16200  14571  -6077  -2846
-422       N
ATOM  12653  CE2 TRP B 824      15.461  -4.817 113.781  1.00103.47
C
ANISOU12653  CE2 TRP B 824    12738  13814  12759  -5801  -2789
-378       C
```

ATOM 12654 CE3 TRP B 824   16.150 -3.601 111.810 1.00108.15
C
ANISOU12654 CE3 TRP B 824   13306 13988 13798 -5338 -2768
-613   C
ATOM 12655 CZ2 TRP B 824   15.050 -5.910 113.021 1.00 84.78
C
ANISOU12655 CZ2 TRP B 824   10221 11420 10573 -5706 -2701
-174   C
ATOM 12656 CZ3 TRP B 824   15.742 -4.686 111.059 1.00117.25
C
ANISOU12656 CZ3 TRP B 824   14322 15134 15092 -5257 -2671
-431   C
ATOM 12657 CH2 TRP B 824   15.198 -5.824 111.664 1.00 81.95
C
ANISOU12657 CH2 TRP B 824   9794 10840 10503 -5427 -2645
-221   C
ATOM 12658 N   LEU B 825   18.890   1.035 113.868 1.00 87.73
N
ANISOU12658 N   LEU B 825   11316 11148 10872 -5254 -3182
-1534   N
ATOM 12659 CA LEU B 825   19.327   2.285 113.248 1.00 87.21
C
ANISOU12659 CA LEU B 825   11250 10887 10999 -5067 -3224
-1738   C
ATOM 12660 C   LEU B 825   20.764   2.208 112.729 1.00121.50
C
ANISOU12660 C   LEU B 825   15608 15126 15430 -4877 -3194
-1687   C
ATOM 12661 O   LEU B 825   21.052   2.662 111.616 1.00161.38
O
ANISOU12661 O   LEU B 825   20658 20054 20605 -4825 -3188
-1625   O
ATOM 12662 CB LEU B 825   19.165   3.430 114.253 1.00 90.64
C
ANISOU12662 CB LEU B 825   11840 11338 11263 -5164 -3360
-2025   C
ATOM 12663 CG LEU B 825   19.405   4.891 113.848 1.00 91.15
C
ANISOU12663 CG LEU B 825   11913 11214 11505 -5014 -3433
-2284   C
ATOM 12664 CD1 LEU B 825   20.885   5.284 113.883 1.00 90.13
C
ANISOU12664 CD1 LEU B 825   11857 11000 11388 -4861 -3462
-2337   C
ATOM 12665 CD2 LEU B 825   18.799   5.177 112.481 1.00 89.34
C
ANISOU12665 CD2 LEU B 825   11493 10857 11594 -4903 -3354
-2240   C
ATOM 12666 N   TYR B 826   21.677   1.642 113.521 1.00112.72
N
ANISOU12666 N   TYR B 826   14614 14085 14128 -4930 -3230
-1612   N
ATOM 12667 CA TYR B 826   23.049   1.415 113.064 1.00112.03
C

```
ANISOU12667  CA  TYR B 826    14642  13924  13999  -4926  -3257
-1438       C
ATOM  12668  C   TYR B 826      23.119   0.508 111.837  1.00 85.89
C
ANISOU12668  C   TYR B 826    11278  10587  10771  -4926  -3181
-1156       C
ATOM  12669  O   TYR B 826      23.889   0.771 110.906  1.00132.65
O
ANISOU12669  O   TYR B 826    17247  16404  16749  -4880  -3198
-1069       O
ATOM  12670  CB  TYR B 826      23.875   0.830 114.211  1.00120.50
C
ANISOU12670  CB  TYR B 826    15830  15088  14866  -4997  -3308
-1399       C
ATOM  12671  CG  TYR B 826      25.316   0.535 113.863  1.00108.43
C
ANISOU12671  CG  TYR B 826    14411  13483  13305  -4999  -3346
-1225       C
ATOM  12672  CD1 TYR B 826      26.265   1.550 113.833  1.00 85.23
C
ANISOU12672  CD1 TYR B 826    11576  10432  10376  -4948  -3429
-1337       C
ATOM  12673  CD2 TYR B 826      25.733  -0.764 113.596  1.00107.41
C
ANISOU12673  CD2 TYR B 826    14275  13394  13143  -5050  -3309
-959       C
ATOM  12674  CE1 TYR B 826      27.582   1.285 113.513  1.00 84.77
C
ANISOU12674  CE1 TYR B 826    11606  10305  10299  -4953  -3468
-1178       C
ATOM  12675  CE2 TYR B 826      27.051  -1.042 113.286  1.00 84.67
C
ANISOU12675  CE2 TYR B 826    11483  10441  10246  -5046  -3353
-820       C
ATOM  12676  CZ  TYR B 826      27.970  -0.013 113.244  1.00 83.65
C
ANISOU12676  CZ  TYR B 826    11450  10203  10129  -4999  -3430
-927       C
ATOM  12677  OH  TYR B 826      29.285  -0.288 112.946  1.00109.48
O
ANISOU12677  OH  TYR B 826    14799  13406  13395  -4999  -3478
-789       O
ATOM  12678  N   SER B 827      22.340  -0.570 111.826  1.00 81.80
N
ANISOU12678  N   SER B 827    10661  10170  10250  -4977  -3106
-1015       N
ATOM  12679  CA  SER B 827      22.303  -1.472 110.677  1.00 80.26
C
ANISOU12679  CA  SER B 827    10410   9957  10126  -4964  -3043
-776       C
ATOM  12680  C   SER B 827      21.856  -0.781 109.390  1.00 96.45
C
ANISOU12680  C   SER B 827    12402  11905  12340  -4889  -3020
-795       C
```

```
ATOM  12681  O   SER B 827      22.446  -1.001 108.325  1.00 78.65
O
ANISOU12681  O   SER B 827    10178   9590  10116  -4852  -3019
-652       O
ATOM  12682  CB  SER B 827      21.382  -2.656 110.979  1.00118.07
C
ANISOU12682  CB  SER B 827    15090  14874  14898  -5032  -2972
-649       C
ATOM  12683  OG  SER B 827      21.809  -3.367 112.126  1.00 91.30
O
ANISOU12683  OG  SER B 827    11755  11589  11348  -5118  -3000
-597       O
ATOM  12684  N   ILE B 828      20.821   0.057 109.469  1.00123.00
N
ANISOU12684  N   ILE B 828    15678  15250  15808  -4869  -3010
-975       N
ATOM  12685  CA  ILE B 828      20.348   0.803 108.301  1.00 86.21
C
ANISOU12685  CA  ILE B 828    10960  10488  11308  -4808  -3000
-1000       C
ATOM  12686  C   ILE B 828      21.432   1.692 107.692  1.00 79.98
C
ANISOU12686  C   ILE B 828    10285   9581  10523  -4759  -3070
-1017       C
ATOM  12687  O   ILE B 828      21.625   1.693 106.471  1.00121.31
O
ANISOU12687  O   ILE B 828    15521  14758  15812  -4728  -3064
-883       O
ATOM  12688  CB  ILE B 828      19.100   1.623 108.674  1.00 80.98
C
ANISOU12688  CB  ILE B 828    10179   9818  10773  -4799  -2991
-1227       C
ATOM  12689  CG1 ILE B 828      17.915   0.691 108.931  1.00 80.77
C
ANISOU12689  CG1 ILE B 828    10007   9901  10781  -4849  -2909
-1157       C
ATOM  12690  CG2 ILE B 828      18.773   2.640 107.595  1.00 81.01
C
ANISOU12690  CG2 ILE B 828    10143   9697  10940  -4742  -3006
-1284       C
ATOM  12691  CD1 ILE B 828      17.461  -0.068 107.703  1.00 79.49
C
ANISOU12691  CD1 ILE B 828     9771   9733  10700  -4839  -2842
-930       C
ATOM  12692  N   VAL B 829      22.172   2.443 108.509  1.00 81.06
N
ANISOU12692  N   VAL B 829    10519   9685  10595  -4755  -3144
-1174       N
ATOM  12693  CA  VAL B 829      23.202   3.277 107.887  1.00 81.17
C
ANISOU12693  CA  VAL B 829    10631   9586  10625  -4716  -3211
-1167       C
ATOM  12694  C   VAL B 829      24.410   2.460 107.431  1.00 89.82
C
```

```
ANISOU12694  C   VAL B 829    11812  10686  11629  -4727  -3217
-943       C
ATOM  12695  O   VAL B 829      25.101   2.844 106.480  1.00126.18
O
ANISOU12695  O   VAL B 829    16463  15213  16266  -4699  -3249
-858       O
ATOM  12696  CB  VAL B 829      23.614   4.412 108.842  1.00 82.83
C
ANISOU12696  CB  VAL B 829    10915   9747  10809  -4702  -3298
-1416       C
ATOM  12697  CG1 VAL B 829      22.421   5.310 109.134  1.00121.32
C
ANISOU12697  CG1 VAL B 829    15692  14603  15803  -4677  -3299
-1671       C
ATOM  12698  CG2 VAL B 829      24.180   3.848 110.131  1.00 93.36
C
ANISOU12698  CG2 VAL B 829    12326  11167  11978  -4745  -3323
-1448       C
ATOM  12699  N   THR B 830      24.687   1.336 108.091  1.00 94.53
N
ANISOU12699  N   THR B 830    12426  11374  12117  -4772  -3192
-847       N
ATOM  12700  CA  THR B 830      25.701   0.397 107.614  1.00117.30
C
ANISOU12700  CA  THR B 830    15360  14267  14941  -4782  -3190
-640       C
ATOM  12701  C   THR B 830      25.321  -0.232 106.274  1.00 94.39
C
ANISOU12701  C   THR B 830    12387  11369  12109  -4757  -3132
-477       C
ATOM  12702  O   THR B 830      26.168  -0.404 105.389  1.00 77.28
O
ANISOU12702  O   THR B 830    10258   9163   9941  -4736  -3148
-364       O
ATOM  12703  CB  THR B 830      25.937  -0.687 108.668  1.00135.61
C
ANISOU12703  CB  THR B 830    17699  16682  17143  -4843  -3183
-578       C
ATOM  12704  OG1 THR B 830      26.398  -0.083 109.883  1.00168.75
O
ANISOU12704  OG1 THR B 830    21982  20880  21255  -4869  -3251
-729       O
ATOM  12705  CG2 THR B 830      26.975  -1.681 108.190  1.00131.28
C
ANISOU12705  CG2 THR B 830    17188  16133  16560  -4848  -3189
-385       C
ATOM  12706  N   TYR B 831      24.043  -0.578 106.119  1.00 95.19
N
ANISOU12706  N   TYR B 831    12379  11522  12268  -4762  -3068
-473       N
ATOM  12707  CA  TYR B 831      23.546  -1.307 104.953  1.00 95.32
C
ANISOU12707  CA  TYR B 831    12321  11556  12340  -4743  -3015
-323       C
```

```
ATOM  12708  C   TYR B 831      23.767  -0.585 103.621  1.00 78.84
C
ANISOU12708  C   TYR B 831    10250   9386  10319  -4699  -3040
-293       C
ATOM  12709  O   TYR B 831      24.197  -1.208 102.643  1.00 75.33
O
ANISOU12709  O   TYR B 831     9811   8947   9864  -4686  -3030
-156       O
ATOM  12710  CB  TYR B 831      22.060  -1.589 105.179  1.00 79.44
C
ANISOU12710  CB  TYR B 831    10185   9604  10393  -4760  -2954
-352       C
ATOM  12711  CG  TYR B 831      21.261  -2.027 103.980  1.00 75.65
C
ANISOU12711  CG  TYR B 831     9619   9128   9999  -4736  -2909
-239       C
ATOM  12712  CD1 TYR B 831      20.391  -1.147 103.349  1.00 75.90
C
ANISOU12712  CD1 TYR B 831     9588   9104  10146  -4711  -2907
-308       C
ATOM  12713  CD2 TYR B 831      21.316  -3.338 103.525  1.00 74.89
C
ANISOU12713  CD2 TYR B 831     9494   9086   9874  -4742  -2877
-73       C
ATOM  12714  CE1 TYR B 831      19.660  -1.535 102.247  1.00150.71
C
ANISOU12714  CE1 TYR B 831    18990  18581  19691  -4694  -2877
-199       C
ATOM  12715  CE2 TYR B 831      20.562  -3.747 102.441  1.00142.22
C
ANISOU12715  CE2 TYR B 831    17946  17618  18474  -4720  -2845
18       C
ATOM  12716  CZ  TYR B 831      19.742  -2.839 101.801  1.00179.89
C
ANISOU12716  CZ  TYR B 831    22668  22337  23345  -4698  -2845
-39       C
ATOM  12717  OH  TYR B 831      18.963  -3.249 100.745  1.00179.07
O
ANISOU12717  OH  TYR B 831    22492  22239  23309  -4683  -2823
57       O
ATOM  12718  N   PHE B 832      23.466   0.711 103.546  1.00 76.78
N
ANISOU12718  N   PHE B 832     9994   9051  10129  -4680  -3077
-422       N
ATOM  12719  CA  PHE B 832      23.533   1.397 102.253  1.00102.59
C
ANISOU12719  CA  PHE B 832    13271  12248  13462  -4652  -3108
-373       C
ATOM  12720  C   PHE B 832      24.876   1.367 101.519  1.00104.96
C
ANISOU12720  C   PHE B 832    13661  12516  13702  -4647  -3150
-267       C
ATOM  12721  O   PHE B 832      24.858   1.148 100.295  1.00119.22
O
```

```
ANISOU12721  O   PHE B 832    15451  14324  15524  -4640  -3143
-152       O
ATOM  12722  CB  PHE B 832      23.050   2.847 102.399  1.00124.70
C
ANISOU12722  CB  PHE B 832    16062  14961  16358  -4638  -3158
-538       C
ATOM  12723  CG  PHE B 832      21.595   2.967 102.739  1.00126.85
C
ANISOU12723  CG  PHE B 832    16215  15253  16730  -4641  -3113
-643       C
ATOM  12724  CD1 PHE B 832      20.630   2.691 101.784  1.00111.63
C
ANISOU12724  CD1 PHE B 832    14197  13334  14883  -4635  -3077
-550       C
ATOM  12725  CD2 PHE B 832      21.190   3.382 103.993  1.00135.67
C
ANISOU12725  CD2 PHE B 832    17304  16380  17863  -4651  -3113
-845       C
ATOM  12726  CE1 PHE B 832      19.286   2.801 102.081  1.00 84.56
C
ANISOU12726  CE1 PHE B 832    10644   9920  11565  -4642  -3034
-644       C
ATOM  12727  CE2 PHE B 832      19.846   3.497 104.296  1.00115.58
C
ANISOU12727  CE2 PHE B 832    14631  13858  15426  -4658  -3068
-958       C
ATOM  12728  CZ  PHE B 832      18.894   3.207 103.338  1.00 88.23
C
ANISOU12728  CZ  PHE B 832    11069  10397  12058  -4654  -3025
-852       C
ATOM  12729  N   PRO B 833      26.040   1.566 102.145  1.00 76.75
N
ANISOU12729  N   PRO B 833    10177   8919  10064  -4655  -3196
-299       N
ATOM  12730  CA  PRO B 833      27.295   1.448 101.383  1.00105.09
C
ANISOU12730  CA  PRO B 833    13830  12487  13611  -4656  -3228
-195       C
ATOM  12731  C   PRO B 833      27.755   0.027 101.081  1.00126.87
C
ANISOU12731  C   PRO B 833    16569  15319  16317  -4665  -3179
-70       C
ATOM  12732  O   PRO B 833      28.843  -0.138 100.517  1.00103.18
O
ANISOU12732  O   PRO B 833    13608  12306  13290  -4669  -3200
-7       O
ATOM  12733  CB  PRO B 833      28.310   2.166 102.290  1.00 85.67
C
ANISOU12733  CB  PRO B 833    11464   9971  11117  -4662  -3301
-286       C
ATOM  12734  CG  PRO B 833      27.747   2.011 103.652  1.00 91.77
C
ANISOU12734  CG  PRO B 833    12223  10779  11865  -4674  -3284
-402       C
```

```
ATOM  12735  CD  PRO B 833      26.272   2.180 103.462  1.00 78.04
C
ANISOU12735  CD  PRO B 833    10389   9059  10203  -4664  -3238
-454       C
ATOM  12736  N   LEU B 834      26.982  -1.002 101.432  1.00102.38
N
ANISOU12736  N   LEU B 834    13400  12290  13210  -4671  -3118
-39       N
ATOM  12737  CA  LEU B 834      27.249  -2.348 100.927  1.00109.19
C
ANISOU12737  CA  LEU B 834    14225  13211  14052  -4673  -3077
77       C
ATOM  12738  C   LEU B 834      27.139  -2.421  99.406  1.00110.44
C
ANISOU12738  C   LEU B 834    14352  13369  14242  -4661  -3061
151       C
ATOM  12739  O   LEU B 834      27.967  -3.053  98.739  1.00 86.27
O
ANISOU12739  O   LEU B 834    11293  10323  11163  -4663  -3056
210       O
ATOM  12740  CB  LEU B 834      26.287  -3.350 101.568  1.00 95.38
C
ANISOU12740  CB  LEU B 834    12402  11533  12304  -4686  -3026
102       C
ATOM  12741  CG  LEU B 834      26.451  -3.687 103.047  1.00 74.56
C
ANISOU12741  CG  LEU B 834     9790   8928   9610  -4719  -3037
64       C
ATOM  12742  CD1 LEU B 834      25.189  -4.339 103.580  1.00121.78
C
ANISOU12742  CD1 LEU B 834    15685  14981  15606  -4742  -2987
77       C
ATOM  12743  CD2 LEU B 834      27.627  -4.629 103.213  1.00 74.46
C
ANISOU12743  CD2 LEU B 834     9810   8926   9554  -4731  -3060
141       C
ATOM  12744  N   ASP B 835      26.113  -1.776  98.848  1.00 89.33
N
ANISOU12744  N   ASP B 835    11643  10679  11621  -4652  -3057
139       N
ATOM  12745  CA  ASP B 835      25.960  -1.643  97.401  1.00 73.87
C
ANISOU12745  CA  ASP B 835     9667   8717   9682  -4652  -3058
207       C
ATOM  12746  C   ASP B 835      27.123  -0.912  96.733  1.00 82.32
C
ANISOU12746  C   ASP B 835    10811   9743  10724  -4666  -3110
219       C
ATOM  12747  O   ASP B 835      27.579  -1.320  95.658  1.00102.30
O
ANISOU12747  O   ASP B 835    13336  12302  13232  -4682  -3098
285       O
ATOM  12748  CB  ASP B 835      24.649  -0.915  97.109  1.00 78.99
C
```

```
ANISOU12748  CB  ASP B 835    10270   9340  10403  -4645  -3064
186       C
ATOM  12749  CG  ASP B 835      23.436  -1.801  97.303  1.00 86.08
C
ANISOU12749  CG  ASP B 835    11072  10293  11339  -4639  -3006
209       C
ATOM  12750  OD1 ASP B 835      23.486  -2.986  96.907  1.00 76.04
O
ANISOU12750  OD1 ASP B 835     9767   9079  10045  -4641  -2968
287       O
ATOM  12751  OD2 ASP B 835      22.434  -1.308  97.864  1.00 74.04
O
ANISOU12751  OD2 ASP B 835     9500   8753   9879  -4635  -3000
141       O
ATOM  12752  N   VAL B 836      27.611   0.169  97.343  1.00 84.81
N
ANISOU12752  N   VAL B 836    11191   9992  11042  -4666  -3168
149       N
ATOM  12753  CA  VAL B 836      28.789   0.862  96.818  1.00 75.36
C
ANISOU12753  CA  VAL B 836    10063   8750   9821  -4685  -3225
166       C
ATOM  12754  C   VAL B 836      30.014  -0.047  96.791  1.00 78.98
C
ANISOU12754  C   VAL B 836    10534   9247  10230  -4697  -3204
200       C
ATOM  12755  O   VAL B 836      30.770  -0.062  95.811  1.00106.60
O
ANISOU12755  O   VAL B 836    14039  12755  13708  -4722  -3210
247       O
ATOM  12756  CB  VAL B 836      29.054   2.141  97.634  1.00168.49
C
ANISOU12756  CB  VAL B 836    21921  20457  21639  -4680  -3301
74       C
ATOM  12757  CG1 VAL B 836      30.283   2.863  97.105  1.00154.13
C
ANISOU12757  CG1 VAL B 836    20171  18588  19802  -4704  -3369
103       C
ATOM  12758  CG2 VAL B 836      27.834   3.050  97.596  1.00130.31
C
ANISOU12758  CG2 VAL B 836    17056  15574  16880  -4667  -3326
21       C
ATOM  12759  N   PHE B 837      30.232  -0.814  97.858  1.00 86.05
N
ANISOU12759  N   PHE B 837    11423  10164  11108  -4685  -3183
172       N
ATOM  12760  CA  PHE B 837      31.255  -1.860  97.843  1.00128.97
C
ANISOU12760  CA  PHE B 837    16847  15633  16521  -4693  -3163
203       C
ATOM  12761  C   PHE B 837      31.015  -2.907  96.756  1.00131.96
C
ANISOU12761  C   PHE B 837    17150  16075  16912  -4697  -3102
260       C
```

```
ATOM  12762  O   PHE B 837      31.962  -3.340  96.088  1.00140.79
O
ANISOU12762  O   PHE B 837    18255  17209  18031  -4713  -3093
272       O
ATOM  12763  CB  PHE B 837      31.352  -2.515  99.220  1.00 97.12
C
ANISOU12763  CB  PHE B 837    12819  11612  12472  -4688  -3164
177       C
ATOM  12764  CG  PHE B 837      32.426  -1.922 100.085  1.00 89.03
C
ANISOU12764  CG  PHE B 837    11873  10531  11422  -4698  -3231
132       C
ATOM  12765  CD1 PHE B 837      33.729  -2.386 100.006  1.00 75.53
C
ANISOU12765  CD1 PHE B 837    10174   8813   9712  -4710  -3253
153       C
ATOM  12766  CD2 PHE B 837      32.142  -0.880 100.952  1.00 90.59
C
ANISOU12766  CD2 PHE B 837    12129  10682  11610  -4698  -3278
55       C
ATOM  12767  CE1 PHE B 837      34.725  -1.834 100.791  1.00115.20
C
ANISOU12767  CE1 PHE B 837    15271  13781  14719  -4722  -3325
118       C
ATOM  12768  CE2 PHE B 837      33.133  -0.321 101.738  1.00 96.01
C
ANISOU12768  CE2 PHE B 837    12894  11313  12275  -4709  -3350
9       C
ATOM  12769  CZ  PHE B 837      34.426  -0.800 101.659  1.00 86.82
C
ANISOU12769  CZ  PHE B 837    11744  10139  11103  -4722  -3375
50       C
ATOM  12770  N   LYS B 838      29.766  -3.334  96.567  1.00117.31
N
ANISOU12770  N   LYS B 838    15239  14257  15078  -4684  -3061
284       N
ATOM  12771  CA  LYS B 838      29.450  -4.240  95.462  1.00 83.76
C
ANISOU12771  CA  LYS B 838    10921  10061  10844  -4690  -3012
330       C
ATOM  12772  C   LYS B 838      29.820  -3.654  94.102  1.00101.03
C
ANISOU12772  C   LYS B 838    13124  12250  13014  -4721  -3022
351       C
ATOM  12773  O   LYS B 838      30.381  -4.356  93.253  1.00130.62
O
ANISOU12773  O   LYS B 838    16834  16035  16760  -4741  -2991
356       O
ATOM  12774  CB  LYS B 838      27.964  -4.598  95.491  1.00 94.68
C
ANISOU12774  CB  LYS B 838    12245  11471  12256  -4674  -2981
356       C
ATOM  12775  CG  LYS B 838      27.511  -5.428  94.299  1.00120.71
C
```

```
ANISOU12775  CG  LYS B 838    15476  14816  15573  -4681  -2941
402       C
ATOM  12776  CD  LYS B 838      26.006  -5.641  94.306  1.00129.27
C
ANISOU12776  CD  LYS B 838    16503  15918  16695  -4668  -2922
434       C
ATOM  12777  CE  LYS B 838      25.273  -4.394  93.842  1.00135.44
C
ANISOU12777  CE  LYS B 838    17310  16668  17485  -4675  -2954
441       C
ATOM  12778  NZ  LYS B 838      23.814  -4.465  94.129  1.00126.69
N
ANISOU12778  NZ  LYS B 838    16139  15565  16433  -4660  -2940
455       N
ATOM  12779  N   PHE B 839      29.513  -2.377  93.871  1.00100.77
N
ANISOU12779  N   PHE B 839    13140  12174  12972  -4732  -3069
357       N
ATOM  12780  CA  PHE B 839      29.991  -1.706  92.662  1.00109.86
C
ANISOU12780  CA  PHE B 839    14321  13325  14096  -4777  -3095
391       C
ATOM  12781  C   PHE B 839      31.514  -1.673  92.596  1.00102.32
C
ANISOU12781  C   PHE B 839    13396  12364  13116  -4804  -3108
368       C
ATOM  12782  O   PHE B 839      32.103  -1.924  91.538  1.00100.61
O
ANISOU12782  O   PHE B 839    13160  12191  12875  -4849  -3087
382       O
ATOM  12783  CB  PHE B 839      29.424  -0.286  92.583  1.00103.05
C
ANISOU12783  CB  PHE B 839    13507  12399  13247  -4784  -3164
405       C
ATOM  12784  CG  PHE B 839      27.918  -0.225  92.568  1.00117.28
C
ANISOU12784  CG  PHE B 839    15266  14200  15093  -4762  -3157
421       C
ATOM  12785  CD1 PHE B 839      27.262   0.964  92.847  1.00 94.85
C
ANISOU12785  CD1 PHE B 839    12449  11288  12303  -4753  -3219
402       C
ATOM  12786  CD2 PHE B 839      27.160  -1.344  92.251  1.00 84.34
C
ANISOU12786  CD2 PHE B 839    11023  10091  10930  -4752  -3096
449       C
ATOM  12787  CE1 PHE B 839      25.878   1.033  92.830  1.00 84.65
C
ANISOU12787  CE1 PHE B 839    11103   9989  11072  -4734  -3213
406       C
ATOM  12788  CE2 PHE B 839      25.777  -1.284  92.232  1.00 97.32
C
ANISOU12788  CE2 PHE B 839    12621  11732  12626  -4735  -3093
466       C
```

```
ATOM  12789  CZ  PHE B 839      25.135  -0.093  92.521  1.00 94.08
C
ANISOU12789  CZ  PHE B 839    12226  11251  12267  -4727  -3149
444       C
ATOM  12790  N   ALA B 840      32.165  -1.356  93.715  1.00 89.20
N
ANISOU12790  N   ALA B 840    11777  10652  11462  -4783  -3144
325       N
ATOM  12791  CA  ALA B 840      33.623  -1.272  93.755  1.00 75.52
C
ANISOU12791  CA  ALA B 840    10071   8904   9721  -4806  -3167
303       C
ATOM  12792  C   ALA B 840      34.301  -2.586  93.377  1.00 75.33
C
ANISOU12792  C   ALA B 840     9974   8936   9712  -4815  -3105
283       C
ATOM  12793  O   ALA B 840      35.290  -2.584  92.635  1.00132.32
O
ANISOU12793  O   ALA B 840    17178  16171  16926  -4857  -3100
270       O
ATOM  12794  CB  ALA B 840      34.079  -0.822  95.143  1.00108.86
C
ANISOU12794  CB  ALA B 840    14350  13060  13952  -4780  -3221
260       C
ATOM  12795  N   ILE B 841      33.794  -3.719  93.868  1.00 96.42
N
ANISOU12795  N   ILE B 841    12589  11635  12413  -4780  -3062
273       N
ATOM  12796  CA  ILE B 841      34.428  -4.986  93.507  1.00101.69
C
ANISOU12796  CA  ILE B 841    13175  12341  13123  -4785  -3013
240       C
ATOM  12797  C   ILE B 841      34.027  -5.468  92.116  1.00127.50
C
ANISOU12797  C   ILE B 841    16379  15671  16392  -4814  -2957
242       C
ATOM  12798  O   ILE B 841      34.813  -6.154  91.450  1.00116.49
O
ANISOU12798  O   ILE B 841    14921  14307  15034  -4840  -2921
190       O
ATOM  12799  CB  ILE B 841      34.121  -6.054  94.571  1.00 76.71
C
ANISOU12799  CB  ILE B 841     9972   9173   9999  -4744  -3004
236       C
ATOM  12800  CG1 ILE B 841      34.986  -7.299  94.347  1.00 74.59
C
ANISOU12800  CG1 ILE B 841     9617   8920   9805  -4747  -2975
188       C
ATOM  12801  CG2 ILE B 841      32.639  -6.411  94.575  1.00 73.80
C
ANISOU12801  CG2 ILE B 841     9576   8834   9629  -4722  -2974
276       C
ATOM  12802  CD1 ILE B 841      36.466  -7.017  94.309  1.00 75.25
C
```

```
ANISOU12802  CD1 ILE B 841     9709   8975   9910  -4771  -3002
140       C
ATOM  12803  N   ARG B 842      32.835  -5.132  91.637  1.00139.25
N
ANISOU12803  N   ARG B 842    17878  17179  17851  -4816  -2951
292       N
ATOM  12804  CA  ARG B 842      32.412  -5.643  90.340  1.00127.55
C
ANISOU12804  CA  ARG B 842    16340  15758  16364  -4850  -2905
297       C
ATOM  12805  C   ARG B 842      32.836  -4.725  89.203  1.00128.22
C
ANISOU12805  C   ARG B 842    16465  15867  16387  -4921  -2920
318       C
ATOM  12806  O   ARG B 842      32.613  -5.058  88.034  1.00105.83
O
ANISOU12806  O   ARG B 842    13592  13091  13529  -4970  -2886
320       O
ATOM  12807  CB  ARG B 842      30.896  -5.865  90.315  1.00 89.58
C
ANISOU12807  CB  ARG B 842    11513  10962  11562  -4825  -2896
349       C
ATOM  12808  CG  ARG B 842      30.056  -4.631  90.033  1.00106.66
C
ANISOU12808  CG  ARG B 842    13738  13106  13681  -4839  -2941
413       C
ATOM  12809  CD  ARG B 842      28.572  -4.907  90.276  1.00104.01
C
ANISOU12809  CD  ARG B 842    13370  12773  13378  -4804  -2934
451       C
ATOM  12810  NE  ARG B 842      28.078  -6.033  89.487  1.00126.70
N
ANISOU12810  NE  ARG B 842    16167  15700  16273  -4813  -2887
457       N
ATOM  12811  CZ  ARG B 842      27.893  -7.265  89.955  1.00108.41
C
ANISOU12811  CZ  ARG B 842    13781  13396  14014  -4779  -2851
432       C
ATOM  12812  NH1 ARG B 842      27.623  -8.261  89.127  1.00102.75
N
ANISOU12812  NH1 ARG B 842    12994  12720  13328  -4794  -2815
421       N
ATOM  12813  NH2 ARG B 842      28.100  -7.524  91.238  1.00 81.69
N
ANISOU12813  NH2 ARG B 842    10399   9981  10658  -4738  -2856
414       N
ATOM  12814  N   TYR B 843      33.436  -3.577  89.528  1.00 75.62
N
ANISOU12814  N   TYR B 843     9879   9158   9694  -4936  -2978
336       N
ATOM  12815  CA  TYR B 843      34.063  -2.735  88.515  1.00 87.00
C
ANISOU12815  CA  TYR B 843    11357  10621  11079  -5016  -3002
360       C
```

```
ATOM  12816  C   TYR B 843      35.179  -3.479  87.791  1.00123.24
C
ANISOU12816  C   TYR B 843    15880  15268  15678  -5066  -2946
288       C
ATOM  12817  O   TYR B 843      35.326  -3.358  86.569  1.00157.46
O
ANISOU12817  O   TYR B 843    20203  19667  19959  -5147  -2926
298       O
ATOM  12818  CB  TYR B 843      34.599  -1.465  89.173  1.00 76.81
C
ANISOU12818  CB  TYR B 843    10152   9255   9779  -5015  -3084
384       C
ATOM  12819  CG  TYR B 843      35.020  -0.378  88.213  1.00 93.76
C
ANISOU12819  CG  TYR B 843    12349  11408  11867  -5099  -3134
440       C
ATOM  12820  CD1 TYR B 843      36.320  -0.317  87.727  1.00127.48
C
ANISOU12820  CD1 TYR B 843    16610  15707  16121  -5164  -3127
411       C
ATOM  12821  CD2 TYR B 843      34.122   0.600  87.807  1.00 78.02
C
ANISOU12821  CD2 TYR B 843    10409   9391   9846  -5121  -3195
523       C
ATOM  12822  CE1 TYR B 843      36.712   0.682  86.853  1.00133.04
C
ANISOU12822  CE1 TYR B 843    17362  16423  16765  -5255  -3179
474       C
ATOM  12823  CE2 TYR B 843      34.503   1.604  86.935  1.00 86.36
C
ANISOU12823  CE2 TYR B 843    11515  10449  10849  -5208  -3257
592       C
ATOM  12824  CZ  TYR B 843      35.799   1.641  86.461  1.00115.03
C
ANISOU12824  CZ  TYR B 843    15142  14118  14448  -5278  -3247
572       C
ATOM  12825  OH  TYR B 843      36.183   2.640  85.593  1.00140.23
O
ANISOU12825  OH  TYR B 843    18382  17318  17580  -5378  -3313
652       O
ATOM  12826  N   ILE B 844      35.969  -4.255  88.527  1.00 77.90
N
ANISOU12826  N   ILE B 844    10087   9504  10007  -5027  -2924
211       N
ATOM  12827  CA  ILE B 844      37.032  -5.060  87.936  1.00130.49
C
ANISOU12827  CA  ILE B 844    16659  16207  16715  -5065  -2870
114       C
ATOM  12828  C   ILE B 844      36.550  -6.483  87.640  1.00120.06
C
ANISOU12828  C   ILE B 844    15233  14922  15462  -5041  -2802
50       C
ATOM  12829  O   ILE B 844      35.988  -7.164  88.500  1.00 76.46
O
```

```
ANISOU12829  O   ILE B 844     9689   9366   9995  -4970  -2803
53       O
ATOM  12830  CB  ILE B 844      38.287  -5.070  88.842  1.00 82.10
C
ANISOU12830  CB  ILE B 844    10524  10021  10651  -5043  -2898
60       C
ATOM  12831  CG1 ILE B 844      39.381  -5.960  88.242  1.00 78.38
C
ANISOU12831  CG1 ILE B 844     9936   9584  10261  -5080  -2842
-61       C
ATOM  12832  CG2 ILE B 844      37.933  -5.491  90.263  1.00 76.93
C
ANISOU12832  CG2 ILE B 844     9882   9304  10042  -4957  -2927
71       C
ATOM  12833  CD1 ILE B 844      39.837  -5.533  86.861  1.00 79.31
C
ANISOU12833  CD1 ILE B 844    10038   9776  10321  -5182  -2811
-80       C
TER   12834      ILE B 844
HETATM12835 MG    MG A 901      -9.845 -12.773  50.105  1.00140.10
MG
ANISOU12835 MG    MG A 901    16566  21341  15322    732   3777
-1158      MG
HETATM12836 MG    MG A 902     -11.312 -19.990  52.491  1.00105.22
MG
ANISOU12836 MG    MG A 902    11721  17409  10851   -684   2606
-391      MG
HETATM12837  K     K A 903     -13.245  -4.235  34.199  1.00108.46
K
ANISOU12837  K     K A 903     9751  19607  11850    922    585
-1608       K
HETATM12838  PG  ACP A 904      -7.621 -10.585  52.278  1.00143.75
P
ANISOU12838  PG  ACP A 904    17582  21544  15492    743   4326
-1625       P
HETATM12839  O1G ACP A 904      -7.959 -11.359  50.998  1.00 85.89
O
ANISOU12839  O1G ACP A 904    10113  14197   8327    790   4064
-1510       O
HETATM12840  O2G ACP A 904      -8.449  -9.303  52.335  1.00123.49
O
ANISOU12840  O2G ACP A 904    15004  19020  12898    988   4688
-1650       O
HETATM12841  O3G ACP A 904      -6.155 -10.265  52.310  1.00102.37
O
ANISOU12841  O3G ACP A 904    12589  16103  10203    679   4302
-1805       O
HETATM12842  PB  ACP A 904      -9.860 -11.913  53.806  1.00112.45
P
ANISOU12842  PB  ACP A 904    13186  18087  11453    513   4310
-1260       P
HETATM12843  O1B ACP A 904     -10.384 -12.311  52.461  1.00136.15
O
ANISOU12843  O1B ACP A 904    16035  21086  14612    625   4124
```

```
-1145       O
HETATM12844  O2B ACP A 904     -10.530 -10.692  54.322  1.00 94.17
O
ANISOU12844  O2B ACP A 904    10877  15841   9063    696   4705
-1301       O
HETATM12845  C3B ACP A 904      -8.032 -11.665  53.702  1.00123.38
C
ANISOU12845  C3B ACP A 904    14881  19192  12806    442   4232
-1484       C
HETATM12846  PA  ACP A 904     -11.427 -14.098  54.927  1.00106.25
P
ANISOU12846  PA  ACP A 904    12005  17735  10630    117   4009
-878       P
HETATM12847  O1A ACP A 904     -11.243 -15.329  54.132  1.00 91.52
O
ANISOU12847  O1A ACP A 904    10090  15810   8873    -15   3650
-783       O
HETATM12848  O2A ACP A 904     -12.602 -13.331  54.451  1.00161.68
O
ANISOU12848  O2A ACP A 904    18848  24883  17699    365   4220
-791       O
HETATM12849  O3A ACP A 904     -10.076 -13.187  54.846  1.00 91.12
O
ANISOU12849  O3A ACP A 904    10379  15563   8680    201   4123
-1121       O
HETATM12850  O5' ACP A 904     -11.603 -14.515  56.531  1.00114.23
O
ANISOU12850  O5' ACP A 904    12997  18928  11476   -133   4070
-827       O
HETATM12851  C5' ACP A 904     -11.733 -13.478  57.486  1.00133.60
C
ANISOU12851  C5' ACP A 904    15538  21437  13788    -80   4422
-928       C
HETATM12852  C4' ACP A 904     -11.843 -14.124  58.883  1.00120.97
C
ANISOU12852  C4' ACP A 904    13917  20013  12031   -374   4403
-860       C
HETATM12853  O4' ACP A 904     -12.484 -15.247  58.805  1.00108.86
O
ANISOU12853  O4' ACP A 904    12182  18625  10556   -503   4168
-666       O
HETATM12854  C3' ACP A 904     -10.396 -14.459  59.354  1.00142.17
C
ANISOU12854  C3' ACP A 904    16822  22555  14639   -596   4238
-963       C
HETATM12855  O3' ACP A 904     -10.259 -14.225  60.687  1.00 96.93
O
ANISOU12855  O3' ACP A 904    11187  16936   8706   -791   4397
-1004       O
HETATM12856  C2' ACP A 904     -10.240 -15.939  59.068  1.00145.62
C
ANISOU12856  C2' ACP A 904    17150  22999  15182   -775   3848
-800       C
HETATM12857  O2' ACP A 904      -9.315 -16.580  60.083  1.00133.51
```

```
O
ANISOU12857  O2' ACP A 904    15718  21471  13537  -1070   3702
-779       O
HETATM12858  C1' ACP A 904     -11.459 -16.434  59.173  1.00102.30
C
ANISOU12858  C1' ACP A 904    11443  17715   9710   -800   3835
-635       C
HETATM12859  N9  ACP A 904     -11.645 -17.537  58.243  1.00 97.69
N
ANISOU12859  N9  ACP A 904    10744  17091   9282   -838   3521
-500       N
HETATM12860  C8  ACP A 904     -11.360 -17.535  56.922  1.00 97.60
C
ANISOU12860  C8  ACP A 904    10764  16900   9421   -683   3402
-541       C
HETATM12861  N7  ACP A 904     -11.675 -18.745  56.418  1.00114.55
N
ANISOU12861  N7  ACP A 904    12811  19057  11656   -817   3126
-392       N
HETATM12862  C5  ACP A 904     -12.154 -19.494  57.418  1.00 95.98
C
ANISOU12862  C5  ACP A 904    10359  16890   9218  -1045   3066
-253       C
HETATM12863  C6  ACP A 904     -12.620 -20.792  57.459  1.00 95.62
C
ANISOU12863  C6  ACP A 904    10209  16923   9200  -1264   2825
-73       C
HETATM12864  N6  ACP A 904     -12.789 -21.910  56.442  1.00 95.92
N
ANISOU12864  N6  ACP A 904    10222  16863   9359  -1358   2546
31       N
HETATM12865  N1  ACP A 904     -13.052 -21.310  58.613  1.00 97.14
N
ANISOU12865  N1  ACP A 904    10315  17312   9280  -1477   2818
47       N
HETATM12866  C2  ACP A 904     -13.037 -20.558  59.753  1.00134.31
C
ANISOU12866  C2  ACP A 904    15045  22149  13840  -1487   3054
-14       C
HETATM12867  N3  ACP A 904     -12.575 -19.268  59.711  1.00121.44
N
ANISOU12867  N3  ACP A 904    13538  20431  12175  -1279   3306
-203       N
HETATM12868  C4  ACP A 904     -12.138 -18.743  58.552  1.00 96.06
C
ANISOU12868  C4  ACP A 904    10405  17014   9080  -1056   3308
-318       C
HETATM12869  C1B LMT A 905      16.012   4.823  24.283  1.00181.33
C
ANISOU12869  C1B LMT A 905    31731  18180  18988  -1002  -1445
2337       C
HETATM12870  C2B LMT A 905      15.238   3.540  24.575  1.00189.61
C
ANISOU12870  C2B LMT A 905    32758  19268  20019   -907  -1215
```

```
2393       C
HETATM12871  C3B LMT A 905      14.019   3.430  23.743  1.00189.96
C
ANISOU12871  C3B LMT A 905    32463  19461  20252   -906   -777
2416       C
HETATM12872  C4B LMT A 905      13.161   4.624  23.852  1.00193.48
C
ANISOU12872  C4B LMT A 905    32983  19910  20622   -928   -520
2345       C
HETATM12873  C5B LMT A 905      13.937   5.929  23.602  1.00178.89
C
ANISOU12873  C5B LMT A 905    31175  18016  18777  -1014   -748
2296       C
HETATM12874  C6B LMT A 905      13.047   7.087  23.936  1.00175.05
C
ANISOU12874  C6B LMT A 905    30853  17480  18177  -1016   -509
2221       C
HETATM12875  O1B LMT A 905      16.494   4.769  23.009  1.00166.61
O
ANISOU12875  O1B LMT A 905    29472  16417  17416  -1078  -1513
2381       O
HETATM12876  O2B LMT A 905      16.091   2.404  24.309  1.00177.63
O
ANISOU12876  O2B LMT A 905    31128  17736  18629   -879  -1495
2459       O
HETATM12877  O3B LMT A 905      13.248   2.249  24.177  1.00193.77
O
ANISOU12877  O3B LMT A 905    32985  19942  20697   -832   -560
2466       O
HETATM12878  O4' LMT A 905      12.103   4.521  22.890  1.00193.89
O
ANISOU12878  O4' LMT A 905    32669  20100  20898   -922   -168
2364       O
HETATM12879  O5B LMT A 905      15.153   6.023  24.413  1.00179.45
O
ANISOU12879  O5B LMT A 905    31579  17939  18664  -1029  -1174
2272       O
HETATM12880  O6B LMT A 905      12.191   7.347  22.849  1.00160.55
O
ANISOU12880  O6B LMT A 905    28644  15776  16583  -1025   -224
2239       O
HETATM12881  C1' LMT A 905      20.207   5.597  21.227  1.00159.32
C
ANISOU12881  C1' LMT A 905    28240  15338  16957  -1430  -2599
2406       C
HETATM12882  C2' LMT A 905      20.172   4.511  22.303  1.00165.14
C
ANISOU12882  C2' LMT A 905    29232  16000  17512  -1250  -2730
2397       C
HETATM12883  C3' LMT A 905      18.781   4.104  22.639  1.00159.42
C
ANISOU12883  C3' LMT A 905    28521  15380  16671  -1142  -2269
2412       C
HETATM12884  C4' LMT A 905      17.881   5.258  22.959  1.00160.11
```

```
C
ANISOU12884  C4' LMT A 905    28735  15486  16613  -1164  -1965
2360       C
HETATM12885  C5' LMT A 905      17.967   6.382  21.922  1.00146.01
C
ANISOU12885  C5' LMT A 905    26702  13765  15010  -1310  -1889
2359       C
HETATM12886  C6' LMT A 905      17.245   7.607  22.446  1.00121.35
C
ANISOU12886  C6' LMT A 905    23791  10590  11726  -1314  -1694
2292       C
HETATM12887  O1' LMT A 905      21.508   6.042  21.041  1.00158.22
O
ANISOU12887  O1' LMT A 905    27904  15215  16996  -1534  -2968
2263       O
HETATM12888  O2' LMT A 905      20.886   3.358  21.817  1.00177.61
O
ANISOU12888  O2' LMT A 905    30627  17565  19292  -1222  -2987
2443       O
HETATM12889  O3' LMT A 905      18.825   3.227  23.782  1.00158.76
O
ANISOU12889  O3' LMT A 905    28746  15192  16382  -1009  -2407
2410       O
HETATM12890  O5' LMT A 905      19.362   6.765  21.607  1.00164.04
O
ANISOU12890  O5' LMT A 905    28986  15943  17398  -1441  -2319
2358       O
HETATM12891  O6' LMT A 905      16.920   8.449  21.386  1.00 98.45
O
ANISOU12891  O6' LMT A 905    20602   7784   9021  -1413  -1504
2311       O
HETATM12892  C1  LMT A 905      21.719   6.604  19.748  1.00153.97
C
ANISOU12892  C1  LMT A 905    26880  14874  16749  -1706  -2821
2221       C
HETATM12893  C2  LMT A 905      23.207   7.198  19.592  1.00144.26
C
ANISOU12893  C2  LMT A 905    25381  13686  15747  -1850  -3196
2014       C
HETATM12894  C3  LMT A 905      24.302   6.134  19.857  1.00145.24
C
ANISOU12894  C3  LMT A 905    25338  13826  16019  -1765  -3603
1872       C
HETATM12895  C4  LMT A 905      24.667   6.095  21.357  1.00158.06
C
ANISOU12895  C4  LMT A 905    27464  15209  17383  -1623  -3981
1822       C
HETATM12896  C5  LMT A 905      25.652   7.263  21.685  1.00143.81
C
ANISOU12896  C5  LMT A 905    25650  13340  15650  -1750  -4278
1627       C
HETATM12897  C6  LMT A 905      25.794   7.602  23.210  1.00111.80
C
ANISOU12897  C6  LMT A 905    22178   9039  11261  -1626  -4595
```

```
1580       C
HETATM12898  C7  LMT A 905      26.259   6.421  24.099  1.00132.99
C
ANISOU12898  C7  LMT A 905    25087  11617  13825  -1414  -4990
1555       C
HETATM12899  C8  LMT A 905      25.144   5.517  24.607  1.00128.67
C
ANISOU12899  C8  LMT A 905    24948  10985  12957  -1244  -4763
1792       C
HETATM12900  C9  LMT A 905      24.101   6.340  25.475  1.00146.20
C
ANISOU12900  C9  LMT A 905    27748  13058  14744  -1224  -4508
1906       C
HETATM12901  C10 LMT A 905      23.015   5.364  26.011  1.00133.68
C
ANISOU12901  C10 LMT A 905    26563  11387  12843  -1074  -4260
2133       C
HETATM12902  C11 LMT A 905      21.966   6.149  26.841  1.00143.36
C
ANISOU12902  C11 LMT A 905    28139  12596  13737  -1060  -3905
2108       C
HETATM12903  C12 LMT A 905      20.880   5.195  27.314  1.00139.30
C
ANISOU12903  C12 LMT A 905    27680  12174  13074   -941  -3517
2156       C
HETATM12904 MG    MG B 901      18.450 -32.667  59.159  1.00104.53
MG
ANISOU12904 MG    MG B 901    11692  18309   9714    133  -1036
-2269      MG
HETATM12905 MG    MG B 902      14.921 -34.893  56.689  1.00111.49
MG
ANISOU12905 MG    MG B 902    13054  18465  10841   -325  -1149
-2249      MG
HETATM12906  K      K B 903      27.063 -32.782  74.921  1.00117.94
K
ANISOU12906  K      K B 903    16941  14494  13377   -220  -3112
-618       K
HETATM12907  PG  ACP B 904      18.989 -29.567  56.853  1.00156.77
P
HETATM12908  O1G ACP B 904      20.198 -29.196  56.000  1.00158.85
O
HETATM12909  O2G ACP B 904      19.429 -30.356  58.094  1.00193.45
O
HETATM12910  O3G ACP B 904      18.273 -28.314  57.263  1.00 97.76
O
HETATM12911  PB  ACP B 904      18.633 -32.282  55.630  1.00125.08
P
HETATM12912  O1B ACP B 904      20.049 -32.127  55.209  1.00 74.84
O
HETATM12913  O2B ACP B 904      18.548 -33.061  56.906  1.00140.55
O
HETATM12914  C3B ACP B 904      17.856 -30.616  55.863  1.00 94.96
C
HETATM12915  PA  ACP B 904      17.653 -34.690  54.297  1.00145.01
```

P
HETATM12916 O1A ACP B 904   18.896 -35.337 54.778 1.00145.41
O
HETATM12917 O2A ACP B 904   16.495 -35.170 55.076 1.00161.72
O
HETATM12918 O3A ACP B 904   17.768 -33.066 54.448 1.00114.63
O
HETATM12919 O5' ACP B 904   17.404 -35.029 52.679 1.00149.34
O
HETATM12920 C5' ACP B 904   18.461 -34.795 51.765 1.00160.22
C
HETATM12921 C4' ACP B 904   17.926 -34.968 50.324 1.00134.67
C
HETATM12922 O4' ACP B 904   17.008 -35.884 50.284 1.00121.05
O
HETATM12923 C3' ACP B 904   17.261 -33.629 49.880 1.00112.24
C
HETATM12924 O3' ACP B 904   17.556 -33.346 48.582 1.00112.56
O
HETATM12925 C2' ACP B 904   15.777 -33.891 50.021 1.00107.44
C
HETATM12926 O2' ACP B 904   15.000 -33.102 48.988 1.00118.12
O
HETATM12927 C1' ACP B 904   15.641 -35.182 49.800 1.00119.67
C
HETATM12928 N9 ACP B 904   14.523 -35.703 50.569 1.00130.68
N
HETATM12929 C8 ACP B 904   14.192 -35.379 51.839 1.00153.83
C
HETATM12930 N7 ACP B 904   13.091 -36.079 52.184 1.00150.08
N
HETATM12931 C5 ACP B 904   12.742 -36.833 51.136 1.00132.53
C
HETATM12932 C6 ACP B 904   11.709 -37.727 50.945 1.00143.37
C
HETATM12933 N6 ACP B 904   10.554 -38.241 51.789 1.00158.84
N
HETATM12934 N1 ACP B 904   11.583 -38.362 49.777 1.00138.21
N
HETATM12935 C2 ACP B 904   12.474 -38.131 48.769 1.00138.65
C
HETATM12936 N3 ACP B 904   13.501 -37.243 48.959 1.00131.29
N
HETATM12937 C4 ACP B 904   13.630 -36.599 50.132 1.00128.18
C
HETATM12938 C1B LMT B 905   25.939 -2.783 85.595 1.00130.11
C
ANISOU12938 C1B LMT B 905   16732 16161 16545 -5018 -3061
731   C
HETATM12939 C2B LMT B 905   25.069 -4.032 85.451 1.00134.48
C
ANISOU12939 C2B LMT B 905   17203 16752 17140 -4992 -3010
723   C
HETATM12940 C3B LMT B 905   25.738 -5.241 85.980 1.00128.37

C
ANISOU12940 C3B LMT B 905   16377 16003 16396 -4955 -2937
636   C
HETATM12941 C4B LMT B 905   27.065 -5.439 85.372 1.00104.09
C
ANISOU12941 C4B LMT B 905   13311 12972 13267 -5010 -2906
581   C
HETATM12942 C5B LMT B 905   27.960 -4.198 85.534 1.00106.05
C
ANISOU12942 C5B LMT B 905   13641 13189 13464 -5038 -2951
594   C
HETATM12943 C6B LMT B 905   29.218 -4.406 84.748 1.00112.98
C
ANISOU12943 C6B LMT B 905   14515 14124 14288 -5110 -2917
544   C
HETATM12944 O1B LMT B 905   26.010 -2.432 86.911 1.00101.28
O
ANISOU12944 O1B LMT B 905   13092 12442 12948 -4948 -3070
689   O
HETATM12945 O2B LMT B 905   23.829 -3.831 86.167 1.00100.84
O
ANISOU12945 O2B LMT B 905   12917 12440 12957 -4932 -3035
752   O
HETATM12946 O3B LMT B 905   24.897 -6.422 85.700 1.00116.82
O
ANISOU12946 O3B LMT B 905   14833 14571 14981 -4940 -2902
635   O
HETATM12947 O4' LMT B 905   27.700 -6.563 85.995 1.00 93.62
O
ANISOU12947 O4' LMT B 905   11923 11649 12000 -4967 -2849
492   O
HETATM12948 O5B LMT B 905   27.313 -2.970 85.062 1.00 98.07
O
ANISOU12948 O5B LMT B 905   12691 12152 12418 -5076 -3029
689   O
HETATM12949 O6B LMT B 905   30.032 -5.344 85.410 1.00150.22
O
ANISOU12949 O6B LMT B 905   19176 18837 19065 -5066 -2862
445   O
HETATM12950 C1' LMT B 905   25.677   1.203 88.938 1.00108.45
C
ANISOU12950 C1' LMT B 905   14132 13093 13983 -4882 -3273
664   C
HETATM12951 C2' LMT B 905   24.562   0.882 87.940 1.00104.97
C
ANISOU12951 C2' LMT B 905   13646 12689 13550 -4908 -3276
743   C
HETATM12952 C3' LMT B 905   24.616 -0.547 87.523 1.00107.26
C
ANISOU12952 C3' LMT B 905   13886 13072 13795 -4914 -3190
743   C
HETATM12953 C4' LMT B 905   25.975 -0.968 87.044 1.00 96.45
C
ANISOU12953 C4' LMT B 905   12546 11760 12341 -4960 -3155

```
725       C
HETATM12954  C5' LMT B 905      27.098  -0.540  87.997  1.00100.08
C
ANISOU12954  C5' LMT B 905    13050  12176  12801  -4935  -3162
660       C
HETATM12955  C6' LMT B 905      28.443  -0.808  87.355  1.00116.88
C
ANISOU12955  C6' LMT B 905    15197  14355  14856  -4994  -3136
647       C
HETATM12956  O1' LMT B 905      25.607   2.523  89.374  1.00111.09
O
ANISOU12956  O1' LMT B 905    14507  13331  14373  -4874  -3358
646       O
HETATM12957  O2' LMT B 905      23.284   1.140  88.556  1.00 79.48
O
ANISOU12957  O2' LMT B 905    10366   9408  10425  -4858  -3291
723       O
HETATM12958  O3' LMT B 905      23.653  -0.755  86.472  1.00119.71
O
ANISOU12958  O3' LMT B 905    15432  14681  15370  -4951  -3207
822       O
HETATM12959  O5' LMT B 905      27.016   0.895  88.358  1.00112.39
O
ANISOU12959  O5' LMT B 905    14666  13645  14393  -4933  -3251
673       O
HETATM12960  O6' LMT B 905      29.445  -0.732  88.320  1.00153.51
O
ANISOU12960  O6' LMT B 905    19859  18960  19510  -4962  -3132
582       O
HETATM12961  C1  LMT B 905      26.125   3.519  88.494  1.00 94.91
C
ANISOU12961  C1  LMT B 905    12519  11250  12291  -4939  -3445
719       C
HETATM12962  C2  LMT B 905      25.602   4.939  89.036  1.00 97.42
C
ANISOU12962  C2  LMT B 905    12855  11440  12718  -4914  -3550
692       C
HETATM12963  C3  LMT B 905      26.066   6.118  88.144  1.00114.36
C
ANISOU12963  C3  LMT B 905    15067  13533  14851  -4985  -3671
786       C
HETATM12964  C4  LMT B 905      27.602   6.268  88.227  1.00121.40
C
ANISOU12964  C4  LMT B 905    16024  14436  15667  -5018  -3676
776       C
HETATM12965  C5  LMT B 905      28.030   7.677  87.705  1.00 91.18
C
ANISOU12965  C5  LMT B 905    12262  10522  11860  -5078  -3818
852       C
HETATM12966  C6  LMT B 905      27.573   7.895  86.228  1.00103.98
C
ANISOU12966  C6  LMT B 905    13891  12182  13433  -5169  -3877
1006       C
HETATM12967  C7  LMT B 905      28.177   6.887  85.224  1.00117.36
```

```
C
ANISOU12967  C7  LMT B 905    15586  14026  14979  -5247  -3790
1062       C
HETATM12968  C8  LMT B 905      29.678   7.055  85.031  1.00127.93
C
ANISOU12968  C8  LMT B 905    16974  15396  16236  -5310  -3790
1066       C
HETATM12969  C9  LMT B 905      30.514   6.059  85.949  1.00112.54
C
ANISOU12969  C9  LMT B 905    14999  13490  14273  -5248  -3669
937       C
HETATM12970  C10 LMT B 905      32.035   6.279  85.707  1.00 92.72
C
ANISOU12970  C10 LMT B 905    12527  11004  11697  -5316  -3678
941       C
HETATM12971  C11 LMT B 905      32.848   5.239  86.522  1.00 83.55
C
ANISOU12971  C11 LMT B 905    11330   9883  10533  -5260  -3569
823       C
HETATM12972  C12 LMT B 905      32.590   5.441  88.007  1.00102.61
C
ANISOU12972  C12 LMT B 905    13748  12197  13042  -5152  -3586
734       C
CONECT 273912836
CONECT 274012836
CONECT 440112835
CONECT 444912837
CONECT 445712837
CONECT 447512837
CONECT 459212837
CONECT 459312837
CONECT1081812904
CONECT1086612906
CONECT1087412906
CONECT1089212906
CONECT1100912906
CONECT1101012906
CONECT12835 44011283912843
CONECT12836 2739 2740
CONECT12837 4449 4457 4475 4592
CONECT12837 4593
CONECT1283812839128401284112845
CONECT128391283512838
CONECT1284012838
CONECT1284112838
CONECT1284212843128441284512849
CONECT128431283512842
CONECT1284412842
CONECT128451283812842
CONECT1284612847128481284912850
CONECT1284712846
CONECT1284812846
CONECT128491284212846
CONECT128501284612851
```

CONECT128511285012852
CONECT12852128511285312854
CONECT128531285212858
CONECT12854128521285512856
CONECT1285512854
CONECT12856128541285712858
CONECT1285712856
CONECT12858128531285612859
CONECT12859128581286012868
CONECT128601285912861
CONECT128611286012862
CONECT12862128611286312868
CONECT12863128621286412865
CONECT1286412863
CONECT128651286312866
CONECT128661286512867
CONECT128671286612868
CONECT12868128591286212867
CONECT12869128701287512879
CONECT12870128691287112876
CONECT12871128701287212877
CONECT12872128711287312878
CONECT12873128721287412879
CONECT128741287312880
CONECT128751286912884
CONECT1287612870
CONECT1287712871
CONECT1287812872
CONECT128791286912873
CONECT1288012874
CONECT12881128821288712890
CONECT12882128811288312888
CONECT12883128821288412889
CONECT12884128751288312885
CONECT12885128841288612890
CONECT128861288512891
CONECT128871288112892
CONECT1288812882
CONECT1288912883
CONECT128901288112885
CONECT1289112886
CONECT128921288712893
CONECT128931289212894
CONECT128941289312895
CONECT128951289412896
CONECT128961289512897
CONECT128971289612898
CONECT128981289712899
CONECT128991289812900
CONECT129001289912901
CONECT129011290012902
CONECT129021290112903
CONECT1290312902
CONECT12904108181290912913

```
CONECT1290512917
CONECT12906108661087410892110009
CONECT1290611010
CONECT12907129081290912910129914
CONECT1290812907
CONECT129091290412907
CONECT1291012907
CONECT12911129121291312914129918
CONECT1291212911
CONECT129131290412911
CONECT129141290712911
CONECT12915129161291712918129919
CONECT1291612915
CONECT129171290512915
CONECT129181291112915
CONECT129191291512920
CONECT129201291912921
CONECT12921129201292212923
CONECT129221292112927
CONECT12923129211292412925
CONECT1292412923
CONECT12925129231292612927
CONECT1292612925
CONECT12927129221292512928
CONECT12928129271292912937
CONECT129291292812930
CONECT129301292912931
CONECT12931129301293212937
CONECT12932129311293312934
CONECT1293312932
CONECT129341293212935
CONECT129351293412936
CONECT129361293512937
CONECT12937129281293112936
CONECT12938129391294412948
CONECT12939129381294012945
CONECT12940129391294112946
CONECT12941129401294212947
CONECT12942129411294312948
CONECT129431294212949
CONECT129441293812953
CONECT1294512939
CONECT1294612940
CONECT1294712941
CONECT129481293812942
CONECT1294912943
CONECT12950129511295612959
CONECT12951129501295212957
CONECT12952129511295312958
CONECT12953129441295212954
CONECT12954129531295512959
CONECT129551295412960
CONECT129561295012961
CONECT1295712951
```

```
CONECT1295812952
CONECT129591295012954
CONECT1296012955
CONECT129611295612962
CONECT129621296112963
CONECT129631296212964
CONECT129641296312965
CONECT129651296412966
CONECT129661296512967
CONECT129671296612968
CONECT129681296712969
CONECT129691296812970
CONECT129701296912971
CONECT129711297012972
CONECT1297212971
MASTER      536    0   10   70   46    0   15    612970    2  154
130
END
```
